# Loss of Gαq reshapes fibroblast traits and drives tumor-stroma remodeling in oral cancer progression

**DOI:** 10.1038/s44319-026-00751-2

**Published:** 2026-04-10

**Authors:** Inmaculada Navarro-Lérida, Raquel Huertas-Lárez, Gemma Paredes-García, Dafne García-Mateos, María Isabel Jiménez-López, Llara Prieto-Fernández, Ania Pascual, Virginia Ávila-Oca, Juan Antonio López, Miguel A del Pozo, Saúl Álvarez-Teijeiro, Juana María García-Pedrero, Ramón García-Escudero, Federico Mayor, Catalina Ribas

**Affiliations:** 1 Departamento de Biología Molecular, Instituto Universitario de Biología Molecular IUBM-Universidad Autónoma de Madrid and Centro de Biología Molecular Severo Ochoa (Universidad Autónoma de Madrid-CSIC), 28049 Madrid, Spain; 2 Instituto de Investigación Sanitaria Hospital Universitario La Princesa, 28006 Madrid, Spain; 3 Centro de Investigación Biomédica en Red de Enfermedades Cardiovasculares (CIBERCV), Madrid, Spain; 4 Conexión Cáncer-CSIC (Consejo Superior de Investigaciones Científicas), Madrid, Spain; 5 Centro Andaluz de Biología Molecular y Medicina Regenerativa, Sevilla, Spain; 6 Instituto de Investigación Sanitaria del Principado de Asturias (ISPA), 33011 Oviedo, Spain; 7 Instituto Universitario de Oncología del Principado de Asturias, Universidad de Oviedo, 33006 Oviedo, Asturias Spain; 8 Spanish Biomedical Research Network in Cancer (CIBERONC), Instituto de Salud Carlos III, 28029 Madrid, Spain; 9 Molecular and Translational Oncology Division, Centro de Investigaciones Energéticas, Medioambientales y Tecnológicas (CIEMAT), Madrid, Spain; 10 Proteomics Unit; Centro Nacional de Investigaciones Cardiovasculares (CNIC), Madrid, Spain; 11 Mechanoadaptation and Caveolae Biology Laboratory, Cell and Developmental Biology Area, Centro Nacional de Investigaciones Cardiovasculares (CNIC), Madrid, Spain; 12 Biomedical Research Institute imas12, 12 de Octubre University Hospital, Madrid, Spain

**Keywords:** Tumor Stroma, CAFs, Head and Neck Squamous Cell Carcinoma (HNSCC), Gαq Protein, Exosomes, Cancer, Membranes & Trafficking, Signal Transduction

## Abstract

Head and neck squamous cell carcinoma is an aggressive malignancy with limited therapeutic options and poor outcomes. A key driver of tumor progression is the tumor microenvironment, particularly cancer-associated fibroblasts, which remodel the extracellular matrix and secrete pro-tumorigenic signals. Emerging evidence suggests that vesicle trafficking pathways regulate these secretory functions, though their role in HNSCC remains unclear. Building on our discovery of Gαq as an autophagy regulator, we investigated how its absence reshapes fibroblast behavior and modulates crosstalk with HNSCC cells. We demonstrate that loss of Gαq reprograms murine fibroblasts into a CAF-like phenotype through deregulated intracellular trafficking and increased ceramide accumulation. Gαq-deficient fibroblasts show increased collagen I deposition, ECM remodeling, and secretion. Their exosomes, enriched in tumor-promoting growth factor receptors, suppress Caveolin-1 in tumor cells and induce an EMT-like phenotype that fuels HNSCC growth. In co-culture and in vivo, Gαq-silenced fibroblasts form “railroad-track” structures guiding cancer cell migration and invasion. Reduced Gαq expression in human fibroblasts recapitulates these features, identifying Gαq as a key regulator of fibroblast plasticity and tumor–stroma interactions in HNSCC progression.

## Synopsis

Loss of Gαq reprograms fibroblasts towards a CAF phenotype by altering ceramide homeostasis. This disrupts endolysosomal degradative and secretory pathways, as well as extracellular matrix remodeling and exosome release, fueling oral cancer progression.

- CAF-like Gαq-deficient fibroblasts increased collagen I deposition, ECM remodeling and enhanced secretory activity.
- Gαq-deficient fibroblasts secrete exosomes that are enriched in pro-tumorigenic growth factor receptors.
- These secreted exosomes promote EMT-like traits, aberrant tumor growth and invasion in oral cancer cells.
- The Gαq pathway in fibroblasts is a key modulator of tumour-stroma-interactions in oral cancer and a potential therapeutic target.

## Abstract (web summary)

Loss of Gαq reprograms fibroblasts towards a CAF phenotype by altering ceramide homeostasis. This disrupts endolysosomal degradative and secretory pathways, as well as extracellular matrix remodeling and exosome release, fueling oral cancer progression.

## Graphical abstract

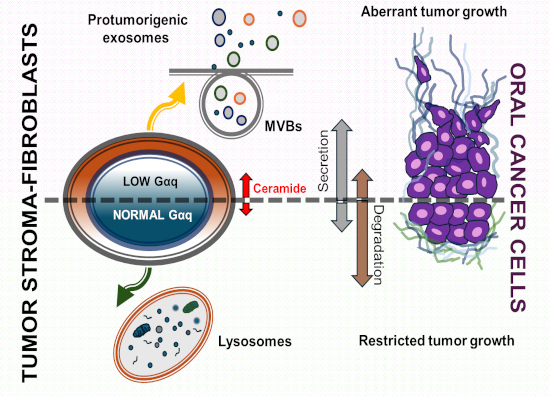

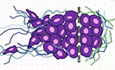

## Introduction

Tumors are constantly seeking alternative adaptive mechanisms to ensure progression, and it is now recognized that the changing tumor microenvironment (TME) and the mechanisms of bidirectional communication between cancer and stromal cells are critical in this quest. Consisting of a complex meshwork of extracellular matrix (ECM) proteins, secreted factors and different types of cells, the TME is a dynamic entity that provides both biophysical and biochemical cues that influence multiple parameters of cancer growth and aggressiveness (de Visser and Joyce, 2023). Key regulators of the TME are cancer-associated fibroblasts (CAFs), a heterogeneous and plastic population of fibroblasts that are activated in pathological contexts and drive tumor progression through various mechanisms (Cao et al, 2025; LeBleu and Kalluri, 2018).

Although most solid tumors are highly dependent on CAFs, this cell population is particularly relevant in head and neck squamous cell carcinoma (HNSCC), a severe and complex malignancy (Johnson et al, 2020). High density of biologically heterogenous CAFs has been associated with poor prognostic features (proliferation, migration, and invasion) and higher rates of local recurrence in HNSCC (Knops et al, 2020). Remarkably elevated amounts of CAFs, up to 80-90% of tumor volume, occur in later stage HNSCC associated with a higher metastatic phenotype (Ansems and Span, 2020; Kumar et al, 2018).

From a molecular perspective, CAFs can express high levels of several specific markers, such as the platelet-derived growth factor receptor α/β (PDGFR α/β) (Gamradt et al, 2023; Hu et al, 2023; Nurmik et al, 2020; Saint and Van Obberghen-Schilling, 2021). Interestingly, dysregulation of spatio-temporally controlled PDGFR-induced signaling has emerged as a critical mechanism governing tumor growth and survival. Within the context of HNSCC, the overexpression of PDGF and its receptor has been associated with oral tumorigenesis and poor prognosis (Lin et al, 2020; Watts et al, 2016). However, the underlying mechanisms regulating PDGFR modulation and dynamics remain poorly understood.

CAFs play a critical role in shaping the mechanical tumor microenvironment by depositing and remodeling ECM components (Chen et al, 2021; Sahai et al, 2020). Besides, CAFs secretome is emerging as a key regulator of tumor progression, by enabling communication with cancer cells and other cells in the TME via both soluble factors and exosomes. As a membranous nanovesicle system, exosomes can mediate the intercellular transport of multiple molecules such as proteins, nucleic acids, and lipids, which can influence a variety of cell functions both locally and remotely (Colombo et al, 2014; Kalluri and LeBleu, 2020). Membrane proteins reported to be present on specific exosomes include the epidermal growth factor receptor (EGFR), glycosylphosphatidylinositol (GPI), HER2, and PDGFR, among many others (Kalluri and LeBleu, 2020). These receptors may directly activate pro-survival signaling pathways in the recipient cells, thus promoting tumor progression and resistance to chemotherapy (Feng et al, 2022; Wandrey et al, 2023).

Of note, emerging evidence shows a direct correlation between exosomes and the aggressiveness of HNSCC (Li et al, 2022; Teng et al, 2021) and highlights the potential role of the interplay between autophagy, a self-catabolic process, and exosome biogenesis and secretion as a pivotal mechanism in regulating this type of tumor. Both cellular processes depend on the regulation of the endolysosomal system and rely on the convergence of degradative and secretory regulators (Salimi et al, 2020; Zubkova et al, 2024). However, the mechanisms underlying the specific sorting of certain growth factor receptors to exosomes and the crosstalk between secretory and autophagy pathways in pathological contexts remain unclear.

G-protein-coupled receptors (GPCR) play a fundamental role in the control of cellular homeostasis. However, aberrant expression, function, or mutations of GPCRs and/or their intracellular signaling networks also significantly contribute to many facets of tumorigenesis, often acting as driving oncogenes themselves (Dorsam and Gutkind, 2007; O’Hayre et al, 2014). Interestingly, recent studies suggest the potential involvement of GPCRs and their downstream signaling pathways in the biogenesis, secretion, homing, and uptake of extracellular vesicles (Bebelman et al, 2020). Within this system, mutated versions of the Gαq protein are present in different tumor types, particularly in uveal and cutaneous melanomas (Onken et al, 2022; Yu et al, 2014). Gαq signaling drives tumorigenesis by mechanisms related to the activation of its canonical effectors, such as phospholipase C-beta, as well as via an emerging complex interactome involving a novel effector region capable of interacting with proteins with PB1 domains (Sánchez-Fernández et al, 2016). Remarkably, we have reported that Gαq is a crucial regulator of the autophagy process in mouse fibroblasts, by mechanisms involving the modulation of active mTORC1 complexes via this novel non-canonical effector region (Cabezudo et al, 2021). Therefore, we set to explore whether Gαq may play a more general role in the crosstalk between endolysosomal and secretory pathways and its potential impact on the tumor stroma in HNSCC pathological contexts.

Here, we demonstrate that the absence of Gαq in fibroblasts leads to their activation, resulting in matrix remodeling and contractile features comparable to malignant CAFs, and to marked alterations in protein trafficking and degradation, which result in aberrant cargo sorting and the release of exosomes enriched in several tumor growth factor receptors, especially PDGFR, a key driver of HNSCC progression. Mechanistically, these defects arise from disrupted lipid homeostasis, which underlies the imbalance between degradative and secretory pathways. Consistently, Gαq knockout (GαqKO) fibroblasts or their derived exosomes foster oral cancer cell growth and invasiveness. Our data put forward stromal Gαq as a new player in the modulation of the HNSCC tumor microenvironment by controlling fibroblast plasticity and functionality, integrating ECM remodeling with lipid-mediated control of exosome/autophagic flux in the HNSCC microenvironment.

## Results

### Gαq deficiency in fibroblasts promotes cancer-associated fibroblasts (CAFs) traits

**Figure 1:**
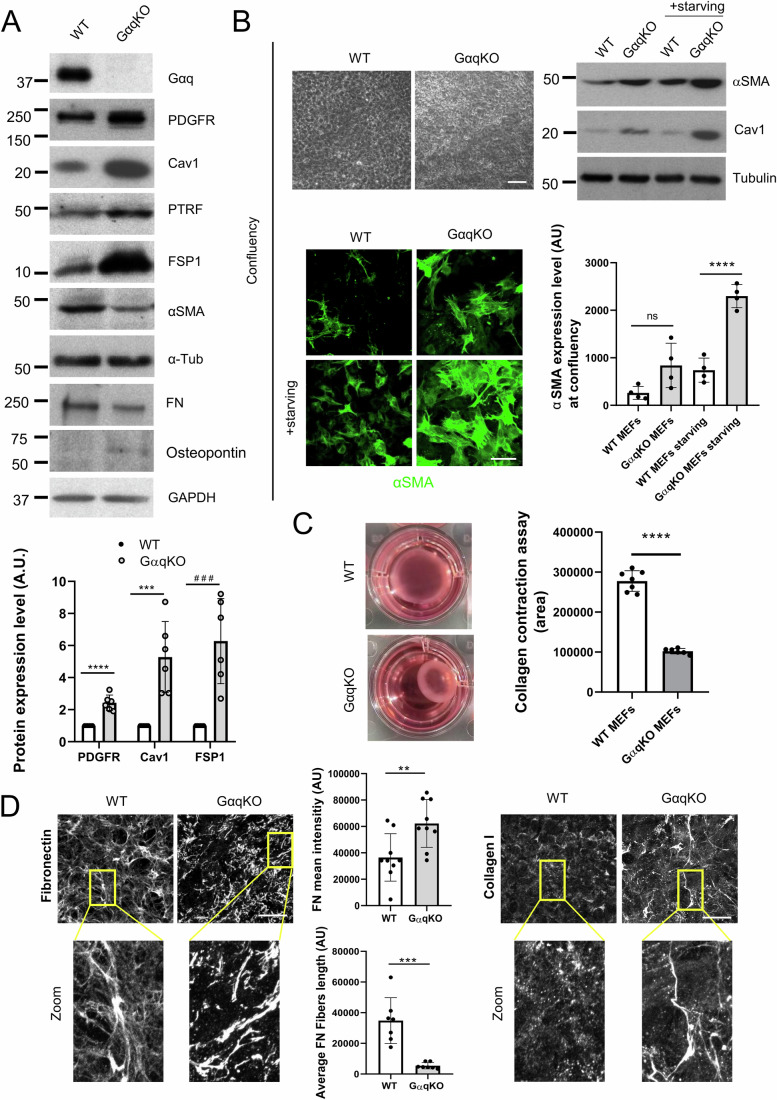
Lack of Gαq in fibroblasts induces in Cancer-Associated Fibroblasts (CAFs)-like features. (**A**) Western blot assessing fibroblast activation markers in wild-type and GαqKO MEFs after 24 h in culture. Tubulin and GAPDH were used as loading controls. Quantification of the markers PDGFR, Caveolin-1, αSMA, and FSP1 protein levels is shown in the graph below (*n* = 6). (**B**) Phase-contrast images (Scale bar, 100 µm) and αSMA protein expression in wild-type and GαqKO MEFs under basal and starvation conditions at high confluency. Confocal images show αSMA staining under the same conditions (Scale bar, 20 µm). The chart shows the amounts of αSMA at confluent conditions (*n* = 4). (**C**) Collagen gel contraction assay. Representative photomicrographs show collagen gel contraction by wild-type or lacking Gαq expression MEFs. Plot represents the contracted gel surface area (*n* ≥ 6). (**D**) Z-stack projections of confocal microscopy images showing Fibronectin (left panels) and Collagen I (right panels) in the matrix deposited by wild-type or GαqKO MEFs (Scale bar, 25 µm). The charts represent average intensity (upper graph) and fiber length (lower graph) of FN in each case (*n* = 9 and *n* = 7, respectively). Data information: In (**A**–**D**), data were presented as mean ± SD. The number of biological replicates is specified above in the description of each panel. Statistical significance in (**A**) was determined using an unpaired *t*-test (^###^*p* = 0.0007; ***p = 0.0008; ****p < 0.0001); in (**B**) was determined using an one-way ANOVA test (ns *p* = 0.1065; ****p < 0.0001); in (**C**) was determined using a unpaired *t*-test with Welch’s correction (****p < 0.0001); and in (**D**) for measuring mean intensity it was determined using an unpaired *t*-test (**p = 0.008) and for fiber length using a Mann–Whitney test (***p = 0.0006). Source data are available online for this figure.

**Figure EV1:**
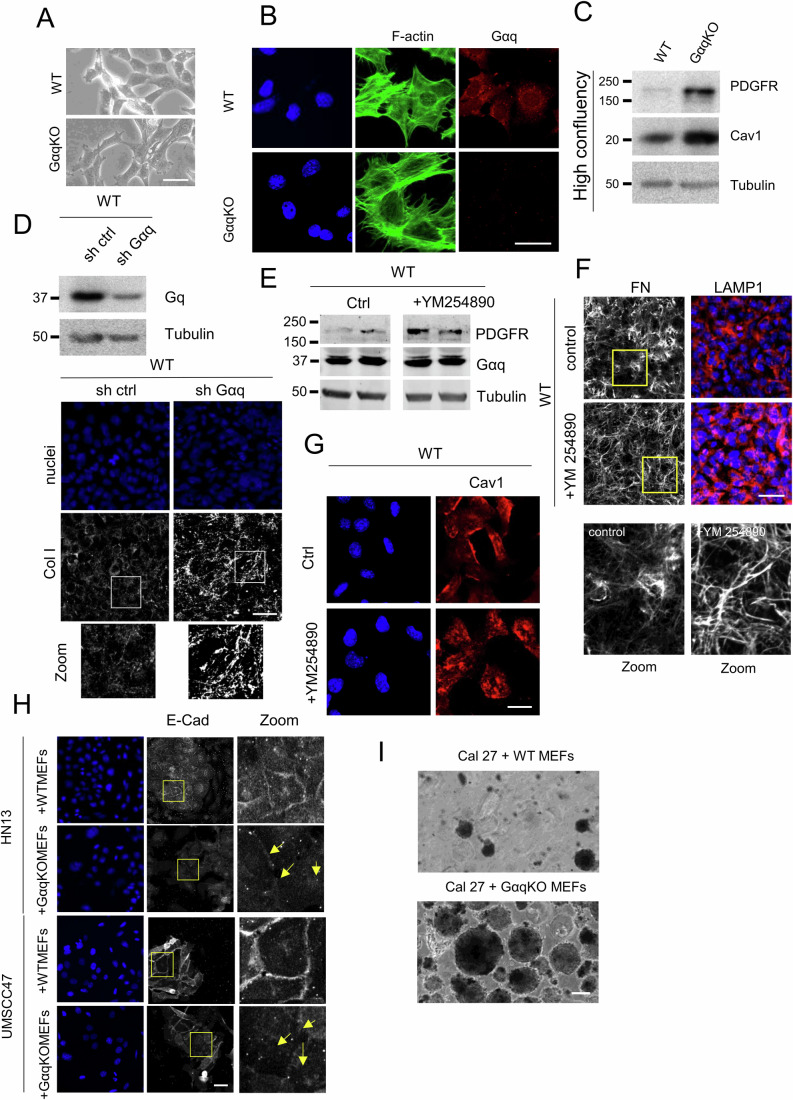
Lack or inhibition of Gαq activity modifies fibroblast-specific features. (**A**) Phase-contrast microscope images of WT and GαqKO MEFs 24 h after seeding (Scale bar, 25 µm). (**B**) Confocal analysis of F-actin and Gαq subcellular distribution in WT and GαqKO MEFs (Scale bar 25 µm). (**C**) Western blot analysis of Cav1 and PDGFR expression in fibroblasts after 96 h in culture. (**D**) Knock-down efficiency of Gαq in WT MEFs upon lentiviral infection of short-hairpin RNA constructs was assessed by Western blot analysis and PDGFR distribution upon Gαq depletion by confocal microscopy. Z-stack projections of confocal microscopy images showing increased collagen I matrix deposition in Gαq-depleted MEFs (Scale bar, 50 µm). Zoomed images are also shown. (**E**) Western blot analysis of PDGFR expression levels in WT MEFs treated for 24 h with the Gαq inhibitor YM254890 (5 µM). (**F**, **G**) Fibronectin matrix deposition (in gray) and lysosomal distribution (in red) (Scale bar 50 µm) (**F**) or Cav1 staining (**G**) in WT MEFs treated with the Gαq inhibitor YM254890 (5 µM). A LAMP1 antibody was used as a lysosome marker (Scale bar 25 µm). (**H**) Microscopy images of HN13 and UMSCC47 cells co-cultured with WT or GαqKO fibroblasts. Cells were stained for the epithelial-mesenchymal transition marker E-cadherin (in gray), and nuclei were stained with Hoechst. Zoomed images show E-cadherin redistribution from cell-cell contact sites to intracellular compartments in the presence of GαqKO fibroblasts (arrows) (Scale bar, 25 µm). (**I**) Representative phase-contrast images (Scale bar,) of spheroids formed in Matrigel after 72 h of co-culture of Cal27 tumor cells and either WT or GαqKO MEFs (Scale bar, 150 µm). Source data are available online for this figure.

To address the potential role of Gαq at the stromal level, we assessed the impact of Gαq absence on the activation status of Mouse Embryonic Fibroblasts (MEFs). After 24 h of seeding, we compared the expression of key CAF-related biomarkers between wild-type (WT) and Gαq knockout (GαqKO) MEFs. The lack of Gαq expression, as confirmed by both Western blot (Fig. 1A) and immunofluorescence (Fig. EV1B), was associated with a significant upregulation of most of the CAFs markers assessed (PDGFR, Caveolin-1, PTRF (also referred to as Cavin-1), FSP1 and osteopontin), except for α-smooth muscle actin (α-SMA) (Fig. 1A).

Since molecular variations between CAFs with different levels of α-SMA have been reported (Chen et al, 2017; Muchlińska et al, 2022; Patel et al, 2018) and differences in the expression of α-SMA seem to be critically influenced by different factors, including cell morphology and adhesion, we evaluated whether those features could underlie the observed slightly reduced levels of α-SMA in GαqKO MEFs after 24 h in culture. Of note, assessment of cell morphology by phase-contrast microscopy at this time point revealed that GαqKO fibroblasts displayed a slightly delayed cell attachment, a more apparent spindle-like shape (Fig. EV1A), and a higher F-actin stress fiber pattern (Fig. EV1B) compared to WT MEFs. To address the potential impact of differences in initial cell adhesion, we determined the expression levels of α-SMA at confluency, after a period of 3 to 5 days of culture (Fig. 1B). In the absence of apparent differences in cell morphology at this stage (Fig. 1B upper left panels), there was a significant shift to higher expression of the α-SMA marker in the GαqKO MEFs compared to WT cells under such confluent conditions, which was further enhanced upon starvation (Fig. 1B). Other key CAF markers like Cav1 and PDGFR showed an even higher overexpression compared to the 24 h culture period (Fig. EV1C). Interestingly, under these confluent conditions, GαqKO MEFs displayed a much higher contractile capacity compared with WT cells, as indicated by collagen contraction assays (Fig. 1C).

In addition to increased contractile properties, one of the main features of CAFs is their ability to produce large amounts of ECM proteins, such as collagens, proteoglycans, and glycoproteins (Walker et al, 2018). The ECM matrices deposited by WT versus GαqKO MEFs were thus analyzed to elucidate the functional implications of Gαq deficiency on this key characteristic. Fibroblasts lacking Gαq expression deposited a fibronectin (FN) fiber matrix different from that of WT cells, characterized by shorter and significantly thicker FN fibers as evidenced by fluorescent immunolabelling and confocal imaging (Fig. 1D, left images and graphs). Moreover, under the same experimental conditions, wild-type MEFs deposited almost no collagen I fibers, in contrast to the prominent collagen I matrix observed in GαqKO MEFs (Fig. 1D, right images). This phenotype was also mimicked by specific Gαq knockdown in wild-type MEFs using a lentiviral system (Fig. EV1D). Interestingly, treatment of WT MEFs with YM254890, a specific Gαq inhibitor, reproduced several key features of the GαqKO phenotype, including increased PDGFR expression, altered ECM architecture and the internal redistribution of Cav1 (Fig. EV1E–G).

Overall, these data indicated that Gαq plays a relevant role in modulating fibroblast activation, since its absence triggers most features of CAFs.

### GαqKO fibroblasts drastically shape the architecture, proliferation, and invasion potential of oral tumor cells in both 2D and 3D co-culture models

**Figure 2:**
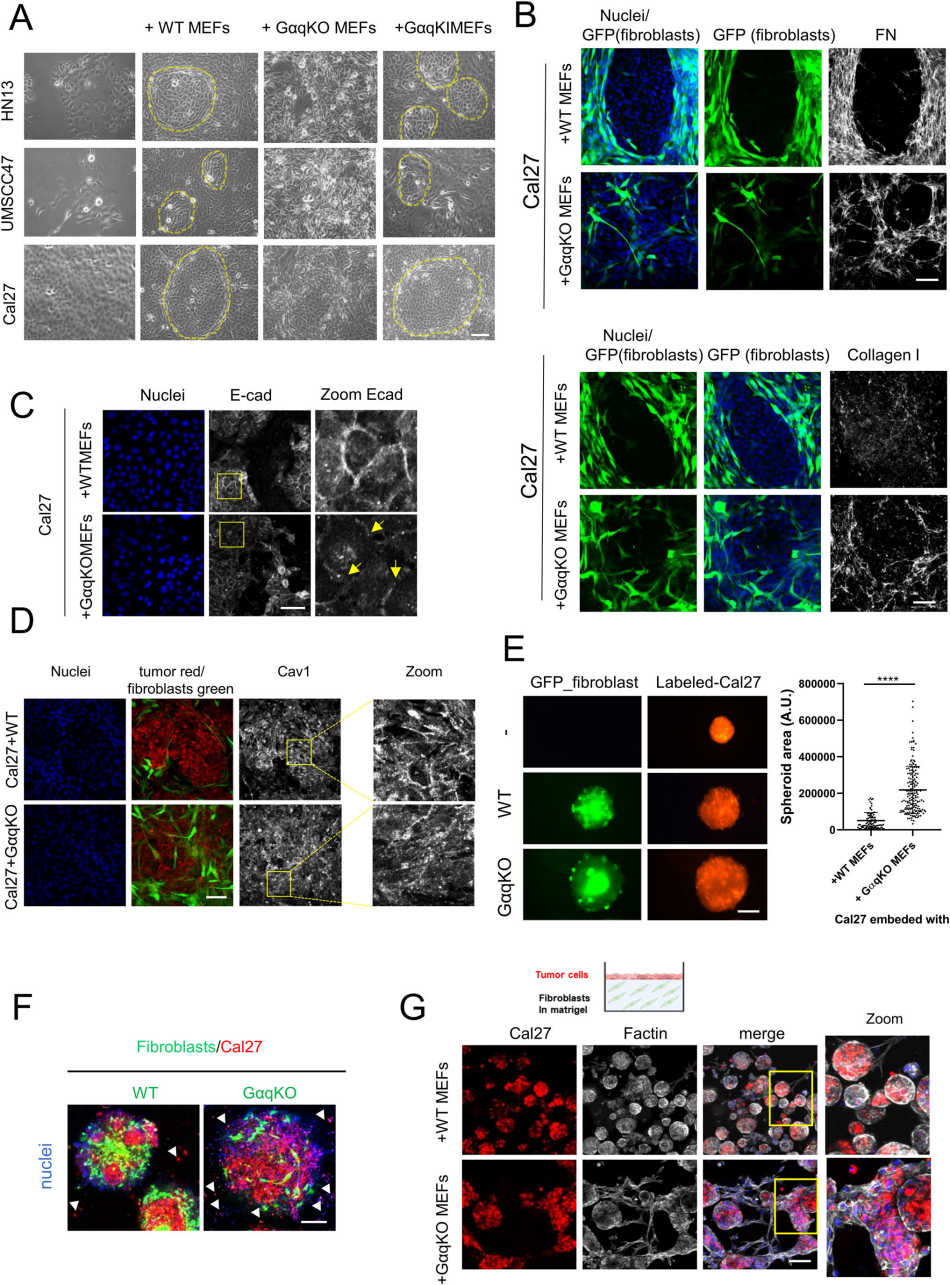
Impact of GαqKO Fibroblasts on oral tumor cell organization, growth, and invasion in 2D and 3D co-culture models. (**A**) Phase-contrast images (Scale bar, 100 µm) of 2D co-cultures of different human squamous cell carcinoma cell lines (Cal27, HN13, and UMSCC47) with either WT, GαqKO, or GαqKI (lentiviral-reconstituted of wild-type Gαq) MEFs. (**B**) Confocal microscopy analysis of the matrix deposited by GFP-labeled WT or GαqKO MEFs co-cultured with Cal27 cells. Fibroblasts are shown in green, fibronectin (FN) or collagen I in gray and DAPI-labeled nuclei in blue (Scale bar, 50 µm). (**C**) Microscopy images of Cal27 cells co-cultured with WT or GαqKO fibroblasts. Cells were stained for the epithelial-mesenchymal transition marker E-cadherin (in gray); nuclei were labeled with Hoechst. Zoomed images show the redistribution of E-cadherin from cell-cell contact sites to intracellular compartments in the presence of GαqKO fibroblasts (arrows) (Scale bar, 50 µm). (**D**) Distribution of Caveolin-1 (in gray) under the same conditions as in panels (B) and (C) (Scale bar, 50 µm) (**E**) Epifluorescence microscopy of tumor spheroid formation and growth formed by cell-tracker prelabeled Cal27 cells (red) co-cultured with GFP-expressing WT or GαqKO MEFs (green). The plot represents the area of spheroid formation in each condition (Scale bar, 150 µm). (**F**) Invasion assay of Cal27 cells spheroids (red) generated in the presence of WT or GαqKO MEFs (green) and embedded into Matrigel. Arrows indicate invading tumor cells (Scale bar 150 µm). (**G**) The top scheme shows the assay protocol. Representative Z-stack confocal images show the invasion of Cal27 cells (red) into Matrigel embedded with either WT or GαqKO MEFs. F-actin is shown in gray and nuclei in blue (Scale bar, 100 µm). Data information: In (**E**), data were presented as mean ± SD, *n* ≥ 82 spheroids per condition, from three biological replicates. Representative images of *n* = 3 biological replicates are shown for all panels. Statistical significance in (**E**) was determined using a Mann–Whitney test (****p < 0.0001). Source data are available online for this figure.

To determine the potential influence of such pro-tumoral stromal features triggered upon Gαq loss, we established a contact co-culture system using fibroblasts (with or without Gαq) and human head and neck squamous carcinoma (HNSCC) cells. Thus, three different oral HNSCC cell lines (HN13, UMSCC47 and Cal27) were co-seeded with wild-type (WT), Gαq knockout (GαqKO) or Gαq knock-in (GαqKI, a lentiviral-reconstituted version of GαqKO MEFs expressing wild-type Gαq) MEFs. After 72 h of co-culture, 2D phase-contrast images revealed a completely different pattern of tumor-fibroblast distribution depending on stromal Gαq expression. While the three different oral tumor cells remained “encapsulated” in the presence of either WT or GαqKI MEFs, the distribution detected in the presence of GαqKO MEFs was far more chaotic, with tumor cells and fibroblasts exhibiting an intertwined pattern with less clearly defined encapsulated structures (Fig. 2A). To better visualize such differential distribution and further explore ECM deposition and matrix organization under these co-culture conditions, FN and collagen I staining was performed in co-cultures of Cal27 oral cancer cells with WT or GαqKO fibroblasts stably expressing GFP. Again, in the presence of WT MEFs, we observed encapsulated oral tumor cells, which were evenly distributed and formed a fibronectin-rich capsule largely devoid of collagen fibers (Fig. 2B, upper images of each panel). In contrast, GαqKO MEFs fostered an entirely distinct pattern in which tumor cells and fibroblasts intermingled, leading to a partial reduction of tumor cell-cell contacts and higher cell scattering (Fig. 2B, lower images of each panel). The most striking feature of the matrix developed under these conditions was a robust distribution of collagen I fibers, which created well-defined “railroad-tracks” moving away from the tumor cells (Fig. 2B, lower panel). This distinctive matrix architecture was accompanied by altered E-Cadherin and Caveolin-1 localization within tumor cells, both exhibiting partial depletion from cell junctions and intracellular accumulation (Figs. 2C,D and EV1H) consistent with the initiation of epithelial-mesenchymal transition (EMT)-like processes and the acquisition of a more migratory phenotype.

To mimic the natural cell environment to a greater extent, we used a three-dimensional (3D)-in vitro culture model to evaluate the impact of GFP-expressing WT and GαqKO fibroblasts on red-labeled oral Cal27 tumor cells spheroid formation. Interestingly, we observed that spheroids generated in the presence of GαqKO MEFs drastically and significantly increased in number and size compared to their wild-type counterparts (Figs. 2E and EV1I). In addition, the GαqKO-generated spheroids displayed much higher tumor cell spreading and invasion capacity when embedded into Matrigel compared to their WT counterparts (Fig. 2F).

Tumor invasion was further analyzed by confocal microscopy using a 3D organotypic invasion assay. For this purpose, MEFs (either WT or lacking Gαq expression) were embedded into Matrigel, while red fluorescent-labeled tumor cells were seeded on top, mimicking the natural environment of epithelial tissue (scheme in Fig. 2G). Again, the resulting spheroids were significantly larger in the presence of GαqKO compared to WT MEFs. Moreover, only under these conditions did the spheroids gain the ability to enter the Matrigel by following the “railroad” structures generated in the presence of the GαqKO MEFs (Fig. 2G).

Overall, these data strongly suggested a dual role of GαqKO MEFs in promoting tumor growth and invasion, via their higher ECM secretory capacity, the formation of collagen-rich fiber patterns, and additional factors released by these fibroblasts.

### Proteomic analysis reveals an altered endosomal/lysosomal system in GαqKO MEFs

To gain further insight into the molecular mechanisms underlying the tumor-promoting phenotype exerted by GαqKO MEFs, we performed a high-throughput proteomic analysis of these cells compared to the WT control fibroblasts (Dataset EV1).

**Figure 3:**
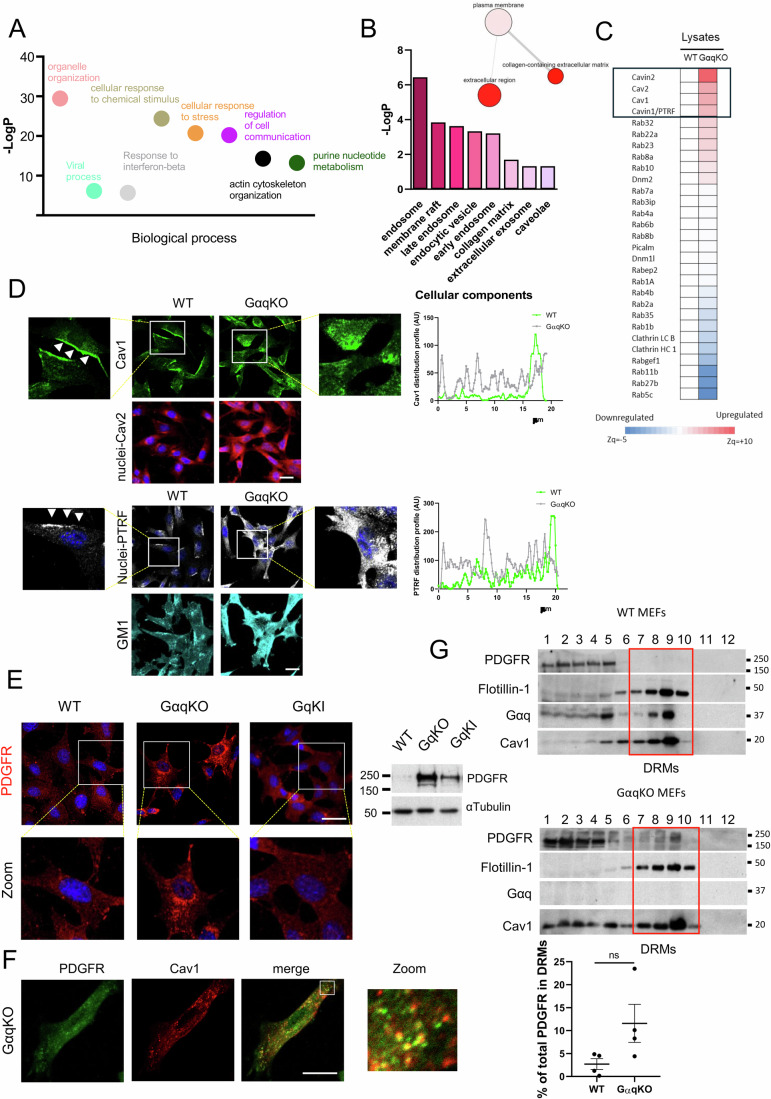
Gαq deficiency in fibroblasts alters intracellular trafficking networks and the caveolae system. (**A**) Chart showing the most significant enriched GO biological processes terms that are significantly altered in GαqKO MEFs compared to WT MEFs. The y-axis represents the statistical significance of the enrichment as −log10(*p* value). (**B**) Enrichment diagram of the most significant “cellular component” categories upregulated in GαqKO MEFs. The network on the right shows STRING interactions corresponding to upregulated extracellular categories in the proteomic dataset. (**C**) Clustered heatmap of intracellular trafficking-related proteins differentially expressed in GαqKO MEFs compared to WT. Caveolar components represent the most prominently upregulated family. (**D**) Confocal microscopy analysis of the subcellular distribution of caveolar components Caveolin-1 and Caveolin-2 (upper panels) and PTRF/Cavin and the lipid raft marker GM1 (stained with cholera toxin) (lower panels) (Scale bar, 25 µm). Zoom images represent the polarized distribution of Caveolin-1 in WT MEFs (arrowheads) versus its internalized pattern in GαqKO MEFs. (**E**) Subcellular distribution of PDGF receptors (PDGFR) in WT, GαqKO and GαqKI MEFs, as indicated. (Scale bar, 25 µm). (**F**) Colocalization analysis of PDGFR (green) and Caveolin-1 (red) in GαqKO MEFs, as determined by confocal microscopy. Merged yellow signals are highlighted in the zoomed area (Scale bar, 25 µm). (**G**) Western blot analysis of sucrose density gradient fractions from WT and GαqKO MEFs. Fractions were analyzed for the distribution of Cav1, Gαq, Flotillin and PDGFR. Red box denotes detergent-resistant membranes (DRMs)-enriched fractions (7–10). The chart shows the amounts of PDGFR present in DRMs fractions relative to the total amount in all fractions. Data information: In (**D**) histograms quantify pixel intensities of Caveolin-1 (upper chart) and PTRF (lower chart) in WT and GαqKO MEFs and in (**G**) data are presented as mean ± SD, *n* = 4 biological replicates. Statistical significance in (**G**) was determined using an unpaired *t*-test (ns, *p* = 0.0849). In (**A**–**C**), differences in protein abundance or functional behavior were estimated by comparing the groups’ Zq or Zc medians, respectively, as determined by the WSPP statistical model. Proteins or functional changes were considered statistically significant with a two-sided *t*-test comparison *p* value <0.05 of the *Z* values. Functional enrichment analysis was assessed by GSEA using the IPA™ (Qiagen) and DAVID resources. See M&M sections for further details. Source data are available online for this figure.

Notably, interaction networks and functional annotation enrichment analysis revealed a plethora of biological processes significantly altered in GαqKO MEFs, particularly those related to organelle organization and endocytic trafficking (Fig. 3A,B) as also evidenced by a completely altered expression profile of most of Rab proteins family members, which are pivotal regulators of both secretory and endocytic pathways as well as of the integrity of intracellular organelles (Fig. 3C). In addition to these changes in intracellular trafficking components, the endocytic machinery itself appeared significantly altered in GαqKO fibroblasts. In the absence of Gαq expression, the clathrin system seems to be downregulated, while a markedly significant upregulation of most of caveolar components (Caveolin-1, Caveolin-2, Cavin-1, and Cavin-2) was observed compared to wild-type fibroblasts (Fig. 3C). This proteomic analysis therefore further confirmed our initial observations (Fig. 1A) that Cavin-1/PRTF and Cav1 were among the most upregulated markers in GαqKO fibroblasts.

**Figure EV2:**
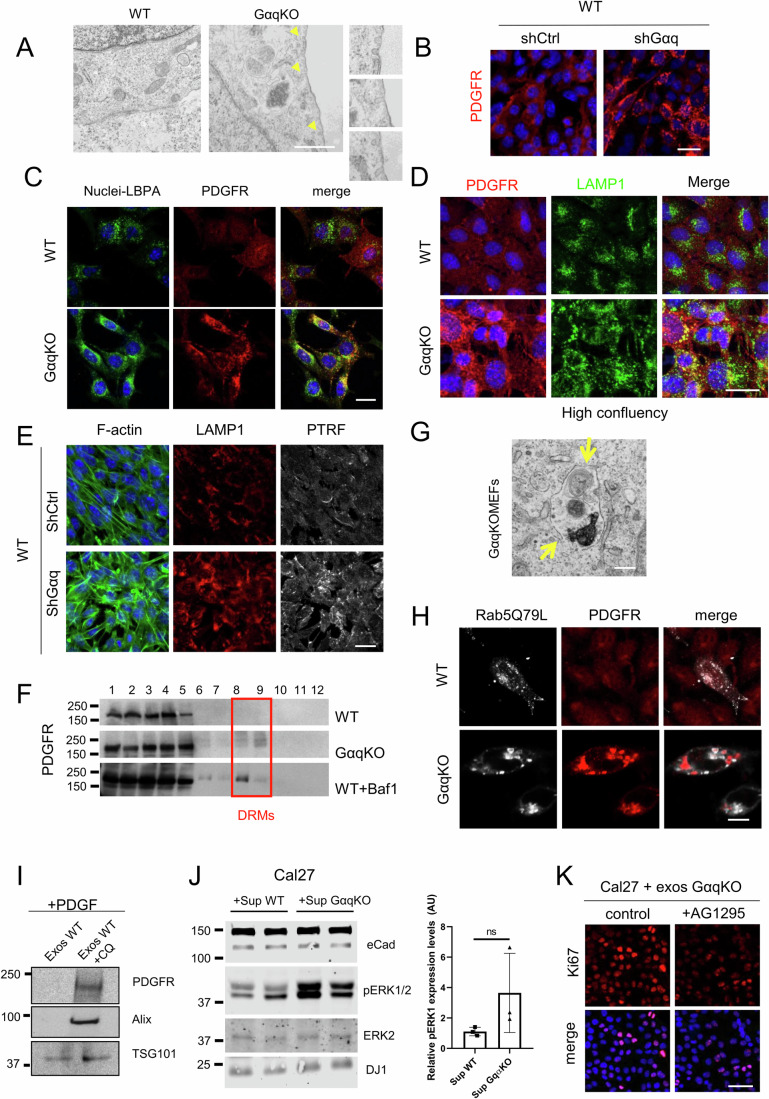
Absence of Gαq alters Cav1 dynamics, lysosomal localization, and turnover. (**A**) WT and GαqKO MEFs were analyzed by electron microscopy. A higher number of caveolae (indicated by arrows) are present in GαqKO MEFs compared to WT (Scale bar 1 µm). (**B**) Distribution pattern of PDGFR in WT MEFs upon Gαq silencing by lentiviral infection with a short-hairpin RNA targeting Gαq (Scale bar, 25 µm). (**C**) Confocal microscopy analysis of the subcellular distribution of the MBV marker LBPA (green) and PDGFR (red) in WT and GαqKO MEFs. Colocalization is shown in yellow (Scale bar, 25 µm). (**D**) Confocal microscopy analysis of LAMP1 (green) and PDGFR (red) subcellular distribution in WT and GαqKO MEFs under confluent conditions (Scale bar, 25 µm). (**E**) Distribution pattern of F-actin, LAMP1 and PTRF upon Gαq silencing in WT MEFs by lentiviral infection with a short-hairpin RNA targeting Gαq (Scale bar, 25 µm). (**F**) PDGFR expression distribution by Western blot analysis of sucrose density gradient fractions from WT and GαqKO MEFs and WT MEFs treated with Bafilomycin 1 (1 nM). The red box denotes DRMs-enriched fractions. (**G**) Electron microscopy image showing an aberrantly loaded lysosome in GαqKO MEFs, indicated by the yellow arrows (Scale bar 200 nm). (**H**) Distribution of PDGFR (red) in MEFs expressing Rab5(Q79L) (gray). Aberrant accumulation of PDFGR in MVBs is preferentially observed in GαqKO MEFs (Scale bar, 10 µm). (**I**) Western blot analysis of the indicated proteins in exosomes derived from PDGF-treated WT cells in the absence or presence of chloroquine (CQ, 1 µM). TSG101 is used as an exosomal marker. (**J**) Western blot analysis showing ERK activation in Cal27 cells treated with supernatants from WT and GαqKO MEFs; quantification of p-ERK levels is shown. (**K**) Effect of GαqKO MEF-derived exosomes and PDGFR inhibitor AG1295 on Cal27 cell proliferation assessed by Ki67 staining (Scale bar, 50 µm). Data information: In (J) data are presented as mean ± SD, *n* = 3 biological replicates. Statistical significance in (**J**) was determined using an unpaired *t*-test with Welch’s correction (ns *p* = 0.2321). Source data are available online for this figure.

To investigate how Gαq deficiency affects the caveolar system, we analyzed the subcellular distribution of key caveolar components using confocal microscopy. Our results revealed a clear exclusion of all three analyzed components (Cav1, Cav2, and Cavin-1/PTRF) from the plasma membrane, along with their accumulation in intracellular membrane structures in GαqKO MEFs, thus presenting a phenotype drastically opposite to the preferential membrane localization observed in wild-type fibroblasts (Fig. 3D). This redistribution was associated with a marked upregulation of the lipid raft marker GM1, suggesting a compensatory remodeling of membrane lipids microdomains in GαqKO MEFs (Fig. 3D). Electron microscopy further revealed an enrichment of caveolar structures (Fig. EV2A) supporting the altered membrane architecture.

Building on our previous observation of markedly elevated PDGFR expression in GαqKO MEFs (Fig. 1A) and considering the marked alterations in caveolar organization together with the established functional interplay between Gαq and Cav1 (Jastrzębski et al, 2017; Liu et al, 1996; Rogers and Fantauzzo, 2020; Smart et al, 1999), we sought to examine in detail how the absence of Gαq affects the subcellular localization and trafficking of this receptor. Confocal microscopy studies revealed that, unlike in WT or GαqKI cells, PDGFR in GαqKO MEFs was predominantly distributed in intracellular dots under basal conditions (Fig. 3E), showing strong colocalization with Caveolin-1 (Fig. 3F). Notably, a similar intracellular accumulation of PDGFR was reproduced upon Gαq knockdown in a wild-type MEF background (Fig. EV2B), confirming that this phenotype directly results from Gαq depletion.

Subcellular fractionation assays further confirmed that in GαqKO MEFs, Cav1, regardless of its intracellular compartmentalization, was associated with detergent-resistant membranes (DRMs). Notably, GαqKO fibroblasts showed a clear accumulation of PDGFR within these DRM domains, a feature that was not detected in WT fibroblasts (Fig. 3G).

Taken together, our data suggest an important involvement of the caveolar system and endocytic trafficking in defining the GαqKO fibroblasts phenotype, with potential implications for the homeostatic regulation of key growth factor receptors.

### Ceramide-dependent lipid imbalance drives aberrant PDGFR trafficking and intracellular accumulation in GαqKO MEFs

**Figure 4:**
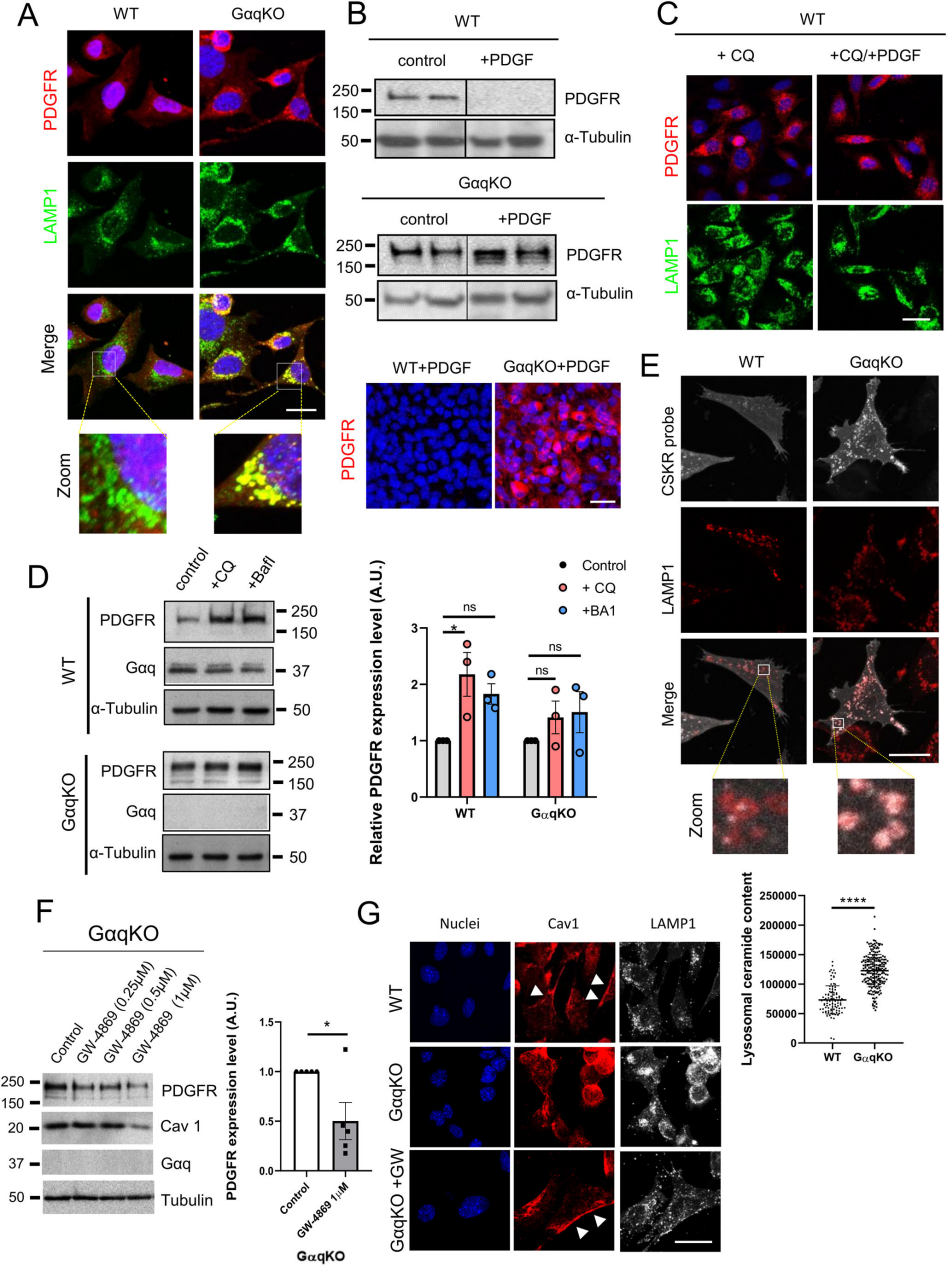
Intracellular accumulation of PDGFR in GαqKO MEFs results from altered trafficking and impaired degradation through a ceramide-dependent mechanism. (**A**) Subcellular localization pattern of PDGFR (in red) and of the lysosomal marker LAMP1 (in green) in WT and GαqKO MEFs. (Scale bar, 25 µm). (**B**) Representative Western blot (upper) and confocal microscopy analysis (lower) of PDGFR expression in WT and GαqKO MEFs under basal conditions or upon stimulation with PDGF (20 ng/ml). Tubulin was used as a loading control (Scale bar, 50 µm). (**C**) Confocal microscopy analysis of WT MEFs treated with Chloroquine (CQ, 1 µM) in the presence or absence of PDGF (20 ng/ml) and analyzed by using PDGFR (in red) and LAMP1 (in green) antibodies (Scale bar, 25 µm). (**D**) Western blot analysis of WT and GαqKO MEFs under control conditions or after treatment with CQ (1 µM) or Bafilomycin 1 (BAf1, 1 nM), using PDGFR and Gαq antibodies. Tubulin was used as a loading control. Graph represents relative PDGFR levels (*n* = 3). (**E**) Colocalization analysis of a ceramide probe corresponding to eGFP-tagged KSR1 CA3 domain (KSR, aa 317–400) (ceramide-binding domain) (in gray) and of the lysosomal marker LAMP1 (in red) in WT and GαqKO MEFs. Low panel: Zoomed regions highlighting ceramide probe-lysosomal colocalization. The chart quantifies ceramide content in lysosome compartments (*n* > 90 lysosomes) (Scale bar, 25 µm). (**F**) Western blot analysis showing the effect of exosome inhibitor GW4689 on PDGFR and Cav1 expression in GαqKO MEFs. The chart represents the effect of GW4689 (1 µM) after 24 h of treatment (*n* = 5). (**G**) Representative confocal microscopy images displaying the impact of exosome inhibitor treatment on the subcellular distribution of Cav1 (Arrowheads indicate plasma membrane polarization) and LAMP1 in GαqKO MEFs (Scale bar, 25 µm). Data information: In (**D**–**F**) data were presented as mean ± SD. The number of biological replicates is specified above in the description of each panel. Statistical significance in (**D**) was determined using a one-way ANOVA (WT Ctrl vs. WT + CQ, *p = 0.0427; WT Ctrl vs. WT + BA1, ns *p* = 0.2274*;* GαqKO Ctrl vs. GαqKO + CQ, ns *p* = 0.8594*;* GαqKO Ctrl vs. GαqKO + BA1, ns *p* = 0.7164), in (**E**, **F**) was determined using an unpaired *t*-test (*p = 0.0286*;* ****p < 0.0001). Source data are available online for this figure.

To further elucidate the Gαq- and Cav1-dependent mechanisms underlying the altered PDGFR trafficking observed in GαqKO MEFs and precisely identify the subcellular compartments where the receptor accumulates, we performed confocal colocalization analysis using markers of multivesicular bodies (LBPA) (Fig. EV2C) and the lysosomal marker LAMP1 at both low- (Fig. 4A) and high-confluence conditions (Fig. EV2D). This analysis revealed that Gαq deficiency induced a pronounced perinuclear redistribution of lysosomes, a phenotype recapitulated by Gαq knockdown in WT MEFs (Fig. EV2E), pointing to a disruption of lysosomal organization. Concomitantly, a significant accumulation of PDGFR within lysosomal compartments was observed, indicating that, although the receptor was efficiently targeted to the degradative pathway, its lysosomal turnover was partially compromised. To validate this observation, WT or GαqKO MEFs were stimulated with the ligand PDGFβ, a treatment known to induce PDGFR degradation through lysosomal mechanisms (Pandey et al, 2023). Following stimulation, PDGFR was efficiently degraded in WT MEFs, whereas GαqKO MEFs exhibited persistent receptor accumulation, as shown by Western blot and confocal microscopy analysis (Fig. 4B). These results confirmed that loss of Gαq impairs PDGFR degradation.

In this regard, we observed that lysosomal inhibition using either bafilomycin A1 (Baf A1), a drug that impairs lysosomal acidification, or chloroquine (CQ), a classical autophagy inhibitor that prevents autophagosomes from fusing with lysosomes, thereby blocking the degradation pathway, led in WT MEFs to increased PDGFR levels, a marked accumulation in intracellular compartments and an enriched distribution in DRMs, even without ligand stimulation (Figs. 4C,D and EV2F), thus effectively mimicking the phenotype observed in GαqKO MEFs. Notably, GαqKO MEFs PDGFR remained largely unaffected by these treatments, highlighting their preexisting defects in lysosomal degradation. In this line, electron microscopy revealed an increased presence of large and fully loaded lysosomal structures in GαqKO MEFs, likely because of altered degradation mechanisms (Fig. EV2G).

To investigate the mechanisms underlying the impaired lysosomal degradative capacity observed in Gαq-deficient cells, we focused on alterations in lipid metabolism associated with the loss of Gαq signaling. Given that Gαq depletion would disrupt diacylglycerol (DAG) production by its effector PLCβ (Harden et al, 2011; Sánchez-Fernández et al, 2014) and that ceramide shares structural and functional similarities with DAG, we hypothesized that the absence of Gαq may induce compensatory ceramide accumulation. Ceramide is a lipid mediator known to destabilize lysosomal membranes, alter intra-lysosomal pH, and impair fusion with autophagosomes (Chitkara and Atilla-Gokcumen, 2025; Ermini et al, 2021; Paciotti et al, 2020). Furthermore, ceramide regulates receptor trafficking and signaling by modulating Caveolin-1 interactions within lipid rafts, thereby influencing the localization and internalization of receptor tyrosine kinases such as PDGFR (Zundel et al, 2000). To test this hypothesis, we employed a ceramide-binding fluorescent probe based on the CA3 domain of KSR1 (Girik et al, 2024), which enables visualization of intracellular ceramide distribution. Confocal microscopy revealed a marked increase in ceramide levels in GαqKO fibroblasts, with prominent lysosomal accumulation confirmed by colocalization with the LAMP1 marker (Fig. 4E). Of note, this phenotype was rescued by treatment of GαqKO fibroblasts with GW4869, a neutral sphingomyelinase (nSMase1/2) inhibitor that prevents the conversion of sphingomyelin to ceramide (Fig. 4F,G). GW4869 treatment significantly decreased the expression of CAF-associated markers (Cav1 and PDGFR) and restored lysosomal organization as well as the plasma membrane distribution of Caveolin-1. Together, these results demonstrate that reducing ceramide levels restores lysosomal integrity and partially rescues the GαqKO phenotype, supporting the conclusion that ceramide accumulation is a major driver of lysosomal mislocalization and impaired degradative capacity in Gαq-deficient fibroblasts.

### Exosomes represent the preferred route of PDGFR release in the absence of Gαq expression

To explore how GαqKO MEFs can maintain homeostasis despite their impaired lysosomal features, we evaluated the contribution of exosome release as a potential adaptive strategy to reduce cellular stress and to partially maintain the levels of proteins that cannot be efficiently degraded at the lysosomal level. Increasing evidence demonstrates that autophagic and exosomal pathways are interconnected (Xing et al, 2021; Zubkova et al, 2024), and our previous studies have shown that Gαq plays a crucial role in controlling autophagy (Cabezudo et al, 2021), which may influence exosome release through autophagy-dependent secretion mechanisms. Thus, we chose to investigate whether PDGFR could be sorted and released through the exosomal pathway in GαqKO MEFs as a compensatory mechanism.

In general, the process of protein release through exosome generation requires its prior sorting into multivesicular bodies (MVB). As previously shown (Fig. EV2C), in GαqKO MEFs, the internal pool of PDGFR strongly colocalizes with the MVB marker LBPA. Notably, strong clusters of PDGFR localized to intraluminal vesicles (ILVs) within MVBs were also clearly observed when endosomes were artificially enlarged by expressing the constitutively active form of Rab5 (Rab5Q67L) (Fig. EV2H). These observations suggested that MVBs are one of the destinations of endocytosed PDGFR in GαqKO MEFs.

**Figure 5:**
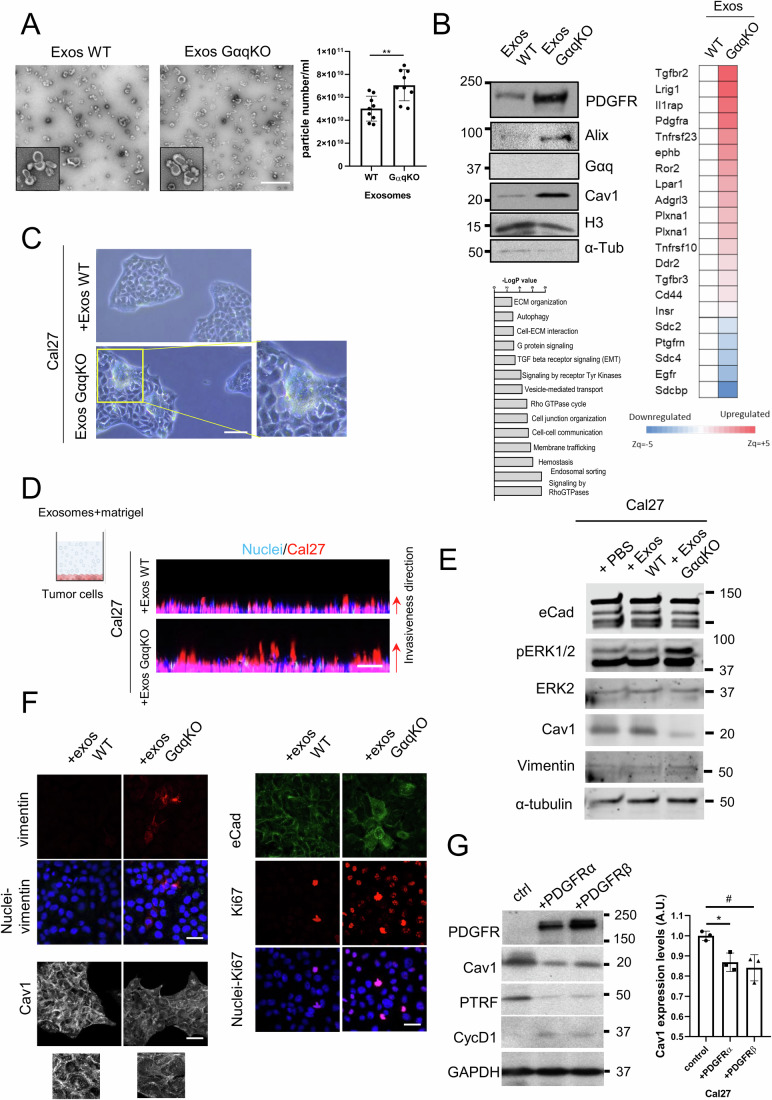
GαqKO MEF-derived exosomes loaded with PDGFR trigger tumor-promoting behaviors in oral cancer cells through Cav1 downregulation and EMT-like mechanisms. (**A**) Exosomes isolated from culture supernatants of WT and GαqKO fibroblasts. Representative electron microscopy images (Scale bar, 200 nm) and quantification of exosome particles relative to cell number are shown (*n* = 9). (**B**) List of the most significantly up- or down-regulated growth factor receptors present in GαqKO fibroblast-derived exosomes. Western blot analysis shows the expression of selected proteins in exosomes derived from equal numbers of WT and GαqKO MEFs. The lower panel shows an enrichment diagram of the “cellular component” category for proteins upregulated in GαqKO MEF-derived exosomes compared with WT. (**C**) Phase-contrast microscopy showing morphological changes in Cal27 human oral cancer cells 48 h after exposure to exosomes derived from either WT or GαqKO MEFs (Scale bar, 50 µm). Zoomed images point to aberrant tumor growth features. (**D**) Representative Matrigel invasion assay of Cal27 tumor cells in the presence of exosomes from WT or GαqKO MEFs. A scheme of the protocol is shown. Arrows indicate direction and extent of invasive migration (Scale bar, 100 µm). (**E**) Western blot analysis of Cal27 cells in the presence of exosomes derived from WT and GαqKO MEFs. The expression of E-cadherin, phosphorylated ERK1/2, total ERK, Caveolin-1, Vimentin, and Tubulin (loading control) was evaluated. (**F**) Confocal microscopy analysis of Cal27 cells treated as in panel (D). Representative staining patterns for Vimentin, E-cadherin, Ki67 (proliferation marker), and Cav1 are shown. (Scale bar, 50 µm). (**G**) Western blot analysis of Ca27 cell lysates overexpressing different PDGFR isoforms, evaluating the expression of selected markers (ctrl, control). The graph shows the effect of PDGFR overexpression on Cav1 protein levels (*n* = 3). Data information: In (**A**, **G**) data were presented as mean ± SD. The number of biological replicates is specified above in the description of each panel. Statistical significance in (**A**) was determined using an unpaired *t*-test (** p = 0.0028); in (**G**) was determined using a one-way ANOVA (*p = 0.0264; ^#^*p* = 0.0116). In (**B**), differences in protein abundance or functional behavior were estimated by comparing the groups’ Zq or Zc medians, respectively, as determined by the WSPP statistical model. Proteins or functional changes were considered statistically significant with a two-sided *t*-test comparison *p* value <0.05 of the *Z* values. Functional enrichment analysis was assessed by GSEA using the IPA™ (Qiagen) and DAVID resources. See M&M sections for further details. Source data are available online for this figure.

To evaluate whether this accumulation of PDGFR may lead to its specific release via exosomes, we purified and analyzed exosomes produced by either WT or GαqKO MEFs. Interestingly, in the absence of Gαq, fibroblasts exhibited a slight but consistent increase in the number of exosomes released (Fig. 5A). More strikingly, exosomes derived from GαqKO fibroblasts exhibited a drastic and substantial enrichment of PDGFR compared to those released by their wild-type counterparts, along with other markers such as Cav1 (Fig. 5B). This phenotype was mimicked when WT MEFs were treated with the lysosomal inhibitor CQ (Fig. EV2I), consistent with the notion that a disrupted degradative mechanism in GαqKO MEFs redirects PDGFR towards exosome-mediated secretion.

To further explore the differential exosome sorting modulated by Gαq expression, we performed a comparative proteomic analysis of the exosomes released by WT and GαqKO MEFs (Dataset EV2). This analysis not only confirmed the strong and significant upregulation of PDGFR within the exosomes produced by GαqKO MEFs, as previously observed by Western blot analysis, but also intriguingly uncovered that a large number of other specific tumor growth factor receptors were markedly present in these exosomes along with other changes in components of secretory, trafficking and signaling networks (Fig. 5B; Dataset EV2). Therefore, exosome release appears to represent an alternative pathway in GαqKO MEFs, to facilitate the secretion of many different tumor growth factors otherwise unable to undergo proper lysosomal degradation.

Next, we investigated the functional effects of exosomes derived from wild-type and GαqKO fibroblasts on oral cancer Cal27 cells. After 48 h, cells treated with GαqKO-derived exosomes exhibited markedly increased proliferation and invasiveness compared to controls, as shown by 2D and 3D invasion assays (Fig. 5C,D). Confocal microscopy and Western blot analysis revealed that these PDGFR-containing GαqKO exosomes initiated a cascade of tumor-promoting signals in Cal27 cells characterized by the loss of Cav1 and elevated Ki67 expression (Fig. 5E,F). Cav1 acts as a negative regulator of ERK/MAPK signaling by sequestering key components; therefore, its loss has been linked to sustained ERK activation, cell proliferation and tumor growth. In addition, Cav1 deficiency has been linked to epithelial-mesenchymal transition (EMT) and metastatic progression (Cerezo et al, 2009; Fiucci et al, 2002). Consistently, Cal27 cells treated with GαqKO-derived exosomes (Fig. 5E,F) or their conditioned medium (Fig. EV2J) exhibited persistent ERK activation, E-cadherin relocalization and increased vimentin expression, consistent with enhanced proliferative activity and the induction of an EMT-like phenotype. Supporting a role for exosome-mediated PDGFR transfer in these effects, PDGFR overexpression in Cal27 cells reduced Cav1 and PTRF levels (Fig. 5G). In contrast, pharmacological inhibition of PDGFR with AG1295 partially attenuated the proliferative response induced by GαqKO-derived exosomes, further validating PDGFR as a critical mediator of this signaling axis (Fig. EV2K).

Collectively, these findings indicate that GαqKO fibroblasts enhance tumor progression through exosome-mediated PDGFR transfer, leading to lipid remodeling, caveolae destabilization, Cav1 loss, and sustained ERK activation, events that together promote tumor cell proliferation, invasion, and metastatic potential.

### Tumors bearing aberrant desmoplastic stroma induced by GαqKO MEFs promote an aggressive metastatic phenotype in vivo

**Figure 6:**
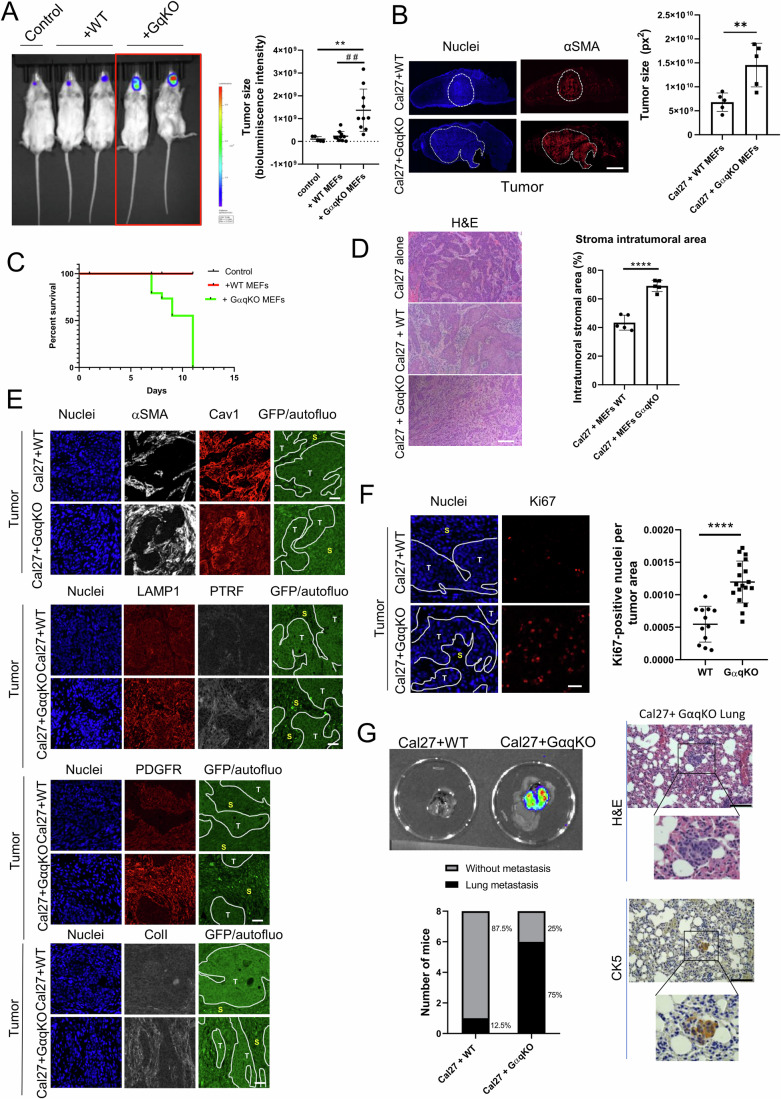
GαqKO MEFs trigger desmoplastic and aggressive tumors in vivo. (**A**) Bioluminescence imaging of orthotopic tongue allografts in mice transplanted with a luciferase-expressing tumor tongue cancer cell line (Cal27-luc) either alone (control) or in combination with WT or GαqKO MEFs. The color Scale correlates with luminescence intensity. The graph displays the quantification of tumor cell-derived bioluminescence (n ≥ 5 mice per condition). (**B**) Representative tissue sections of whole tongue tumors generated as in panel (**A**), stained for nuclei (blue) and αSMA (red). The tumor area is delineated by dotted lines. (Scale bar, 1000 µm). The chart represents tumor size under the indicated conditions (*n* = 5 mice/group). (**C**) Survival curves for mice injected with tumor cells alone (control) or co-injected with WT or GαqKO MEFs. (**D**) Hematoxylin-Eosin staining of tumor samples corresponding to the different conditions, obtained 11 days after cancer cells injection into the mice (Scale bar, 100 µm). The graph illustrates the amount of stroma in tumors generated under each condition (*n* = 5 mice/group). (**E**) Immunohistochemical staining patterns of the indicated markers in the tumor samples. S stroma, T tumor areas (delineated by dotted lines) (Scale bar, 50 µm). (**F**) Evaluation of tumor proliferative capacity determined by Ki67 immunostaining. (Scale bar, 50 µm). The chart represents proliferation rate per tumor area (*n* ≥ 12 images). (**G**) Analysis of tumor metastasis. Left panel: Representative bioluminescence images of lungs from mice co-injected with WT or Gαq-KO MEFs. Graphs represent the percentage of animals displaying lung metastasis (*n* = 8 animals per condition). Right panel: metastatic lesions detected in lungs co-injected with Gαq-KO MEFs, visualized by hematoxylin/eosin staining and by cytokeratin K5 immunostaining (Scale bar, 100 µm). Data information: In (**A**, **B**, **D**, **F**), data were presented as mean ± SD. The number of biological replicates is specified above in the description of each panel. Statistical significance in (**A**) was determined using a Kruskal–Wallis test (**p = 0.0019*;* ##*p* = 0.0038), in (**B**, **D**, **F**) was determined using an unpaired *t*-test (**p = 0.0081; ****p < 0.0001). Source data are available online for this figure.

**Figure EV3:**
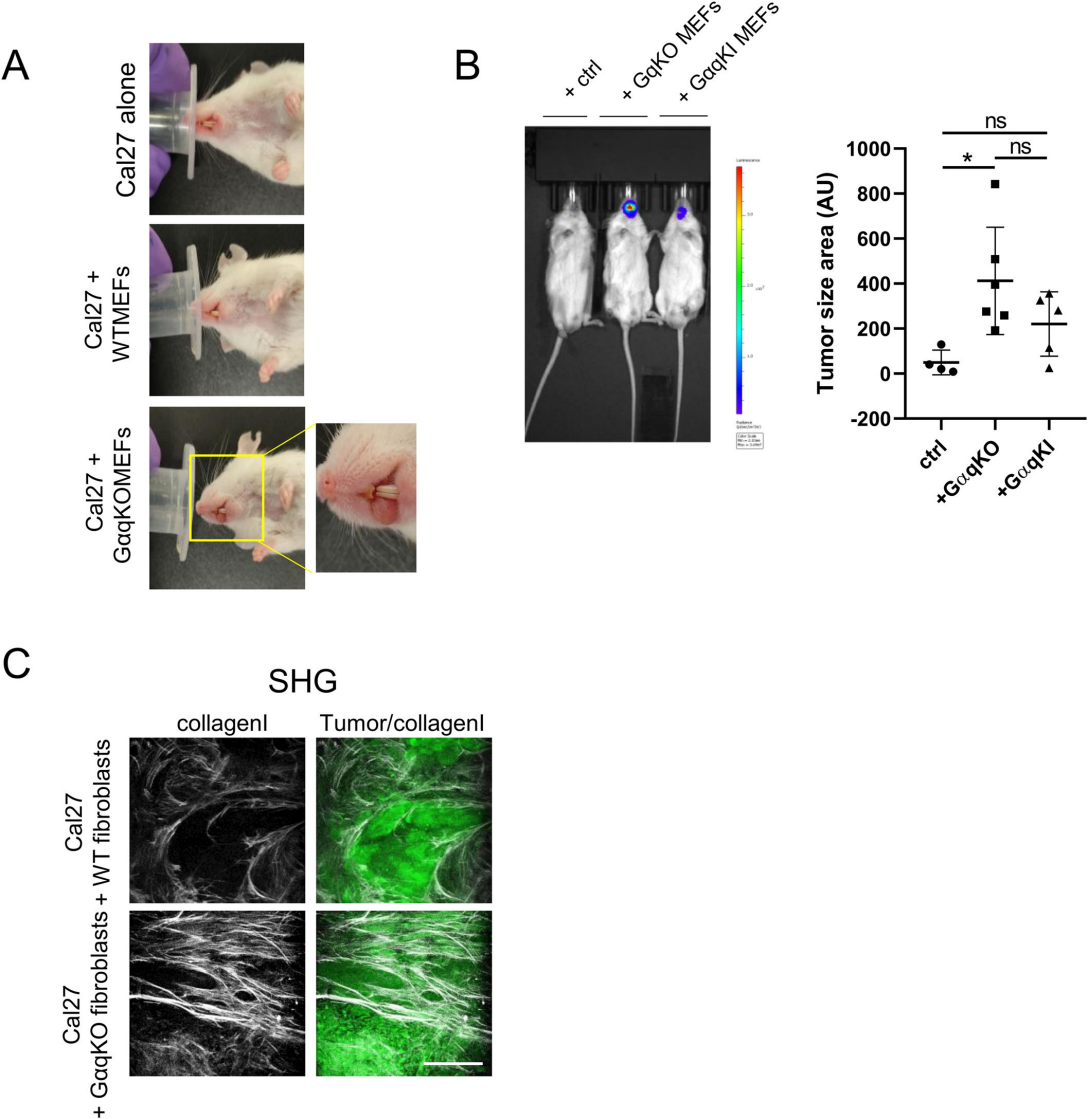
Large desmoplastic stroma-bearing tumors are induced by GαqKO MEFs in vivo. (**A**) Representative macroscopic images of tongue tumor generated by orthotopic injection of Cal27 oral cancer cells alone or in combination with WT or GαqKO MEFs. (**B**) Bioluminescence detection of tumor cells from orthotopic injection of Cal27 cells alone or in combination with either GαqKO MEFs or a Gαq-reconstituted version (GαqKI MEFs). Graphs depict tumor size determined by analysis of H&E-stained tissue images (*n* ≥ 4 animals per condition) (**C**) Representative images of self-assembled collagen matrix organization in the indicated tumors, as measured by second harmonic generation (SHG) microscopy (Scale bar, 50 µm). Data information: In (**B**), data were presented as mean ± SD, *n* ≥ 4 animals per condition. Statistical significance in (**B**) was determined using a one-way ANOVA (Ctrl vs. GαqKI, ns *p* = 0.5292*;* GαqKO vs. GαqKI, ns *p* = 0.2977*;* Ctrl vs. GαqKO, *p = 0.0243). Source data are available online for this figure.

To validate the role of GαqKO MEFs in orchestrating a more favorable tumor-promoting environment compared to their wild-type counterparts in vivo, orthotopic tongue allografts were generated by injecting luminescent and GFP-expressing Cal27 cells, either alone or in combination with WT or GαqKO MEFs, in immunodeficient NSG mice, followed by tumor tracking by bioluminescence-based imaging (Fig. 6). Strikingly, 7 days after injection, animals co-injected with GαqKO MEFs exhibited rapid and pronounced tumor growth at the tongue, as detected by bioluminescence imaging (Fig. 6A), macroscopically (Fig. EV3A) and by confocal microscopy analysis of tumor tissues (Fig. 6B), leading to a severe mice condition, which forced us to euthanize the animals ~10–12 days after cell injection following the established humane endpoint protocol (Fig. 6C). Interestingly, when we used Gαq-reconstituted GαqKO fibroblasts (GαqKI), survival and tumor progression data were like those observed in mice co-injected with WT MEFs (Fig. EV3B), underscoring the critical role of stromal Gαq in mitigating tumor-promoting effects.

Remarkably, histological analysis by hematoxylin/eosin staining of the generated tumors revealed that those formed in the presence of GαqKO fibroblasts exhibited a pronounced stromal desmoplastic reaction, a phenotype that was markedly different from that observed in tumors generated by Cal27 cells alone or co-injected with WT MEFs (Fig. 6D), stressing the relevance of GαqKO fibroblasts in determining tumor microenvironment features.

A detailed immunofluorescence analysis of the tumors generated in vivo confirmed a robust stromal upregulation of key CAF markers also detected in in vitro settings, such as αSMA, Cav1, PDGFR, LAMP1, or PTRF, specifically in the tumors generated by co-injection with GαqKO MEFs (Fig. 6E). Regarding matrix organization, collagen I analysis revealed a stronger and much stiffer collagen matrix surrounding tumors in conditions involving GαqKO MEFs, as determined through immunostaining (Fig. 6E) and multiphoton excitation microscopy coupled with second harmonic generation (MPE-SHG) imaging (Fig. EV3C).

Consistent with this altered tumor microenvironment, tumors formed in the presence of GαqKO fibroblasts exhibited markedly enhanced proliferative activity, as determined by Ki67 staining (Fig. 6F). Interestingly, such a combination of remodeled extracellular milieu and elevated proliferative potential upon GαqKO fibroblasts co-injection resulted in tumors with higher metastatic potency, with 75% of cases disseminating to the lungs, as demonstrated by bioluminescence imaging and hematoxylin-eosin and specific Keratin5 staining (Fig. 6G).

Taken together, our results strongly indicated a critical role of GαqKO MEFs in driving aberrant tumor growth, stromal remodeling, and increased tumor metastatic aggressiveness in vivo.

### Downregulation of Gαq in human fibroblasts and HNSCC-derived CAFs recapitulates the phenotype of GαqKO murine fibroblasts

**Figure 7:**
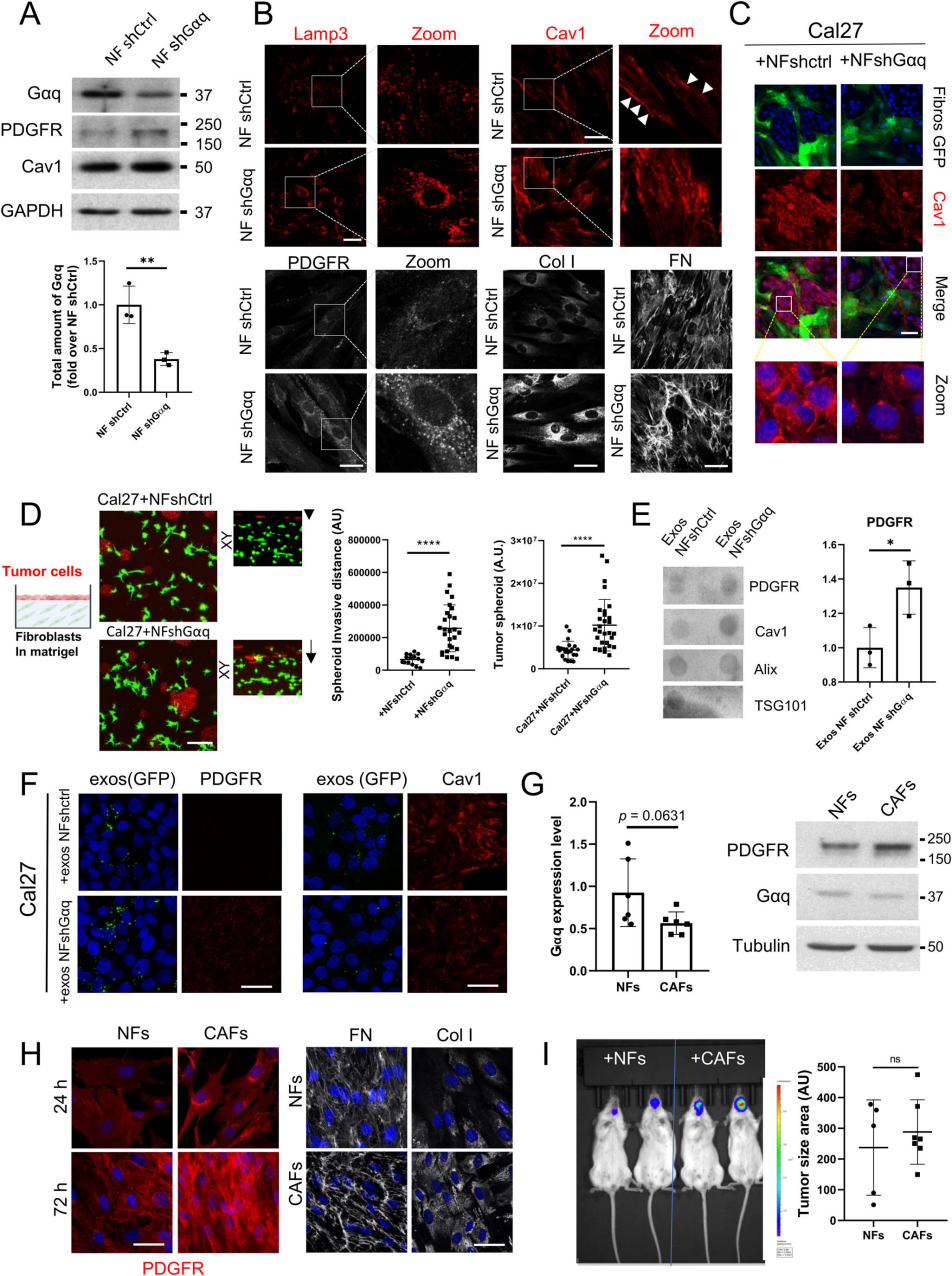
Patient-derived HNSCC CAFs display reduced Gαq expression and foster enhanced tumor progression of oral cancer cells. (**A**) Knockdown efficiency of Gαq in human normal fibroblasts obtained from healthy tongue tissue upon lentiviral infection of short-hairpin RNA constructs was assessed by Western blot analysis, along with levels of PDGFR, Cav1 (membrane polarization indicated by arrowheads) and GAPDH as loading control. Quantification of Gαq levels is shown in the graph (*n* = 3). (**B**) Confocal images showing the effect of Gαq silencing on LAMP3, Cav1, PDGFR distribution and on FN and collagen I matrix deposition. (Scale bar, 50 µm). (**C**) Microscopy images of Cal27 cells co-cultured with NF shctrl or NF shGαq fibroblasts. Cells were stained for Cav1 (in red) (Scale bar, 50 µm). (**D**) The left scheme shows the assay protocol. Images show the Z-stack confocal analysis of Cal27 cells (red) invading Matrigel embedded with either NF shctrl or NF shGαq fibroblasts (green) (Scale bar, 50 µm). Graphs represent tumor spheroid size (*n* ≥ 23 spheroids) and invasion capacity (mean ± SD, *n* ≥ 15 spheroids) (Scale bar, 100 µm). (**E**) Exosomes isolated from culture supernatants of NF shctrl or NF shGαq fibroblasts. Representative dot blot and PDGFR quantification of exosome particles relative to cell number are shown (*n* = 3). (**F**) Staining patterns of PDGFR and Cav1 in Cal27 cells treated with exosomes derived from NF shctrl or NFs shGαq (Scale bar, 50 µm). (**G**) Western blot analysis and quantification of Gαq expression in normal fibroblasts (NFs) and cancer-associated-fibroblasts (CAFs) derived from HNSCC tumor patients (*n* = 6 patients). PDGFR expression levels were also measured in one selected pair of NF-CAFs. (**H**) PDGFR subcellular distribution in the selected NF-CAF samples at either low or high confluency (Scale bar 50 µm) and Z-stack projection of confocal microscopy images showing fibronectin (left panels) and collagen I (right panels) in matrix deposited by NFs or CAFs (Scale bar, 25 µm). (**I**) Bioluminescence imaging of orthotopic tongue allografts in mice transplanted with a luciferase-expressing tumor tongue cancer cell line (Cal27-luc) in combination with NFs or CAFs. Color scale correlates with luminescence intensity. The graph represents tumor size calculated from H&E-stained sections (*n* ≥ 5). Data information: In (**A**, **D**, E, **G**, **I**) data were presented as mean ± SD. The number of biological replicates is specified above in the description of each panel. Statistical significance in (**A**, **E**, **G**, **I**) was determined using an unpaired *t*-test (ns *p* = 0.5117; *p = 0.0355*;* **p = 0.0090), in (**D**) was determined using a Mann–Whitney test (****p < 0.0001). Source data are available online for this figure.

**Figure EV4:**
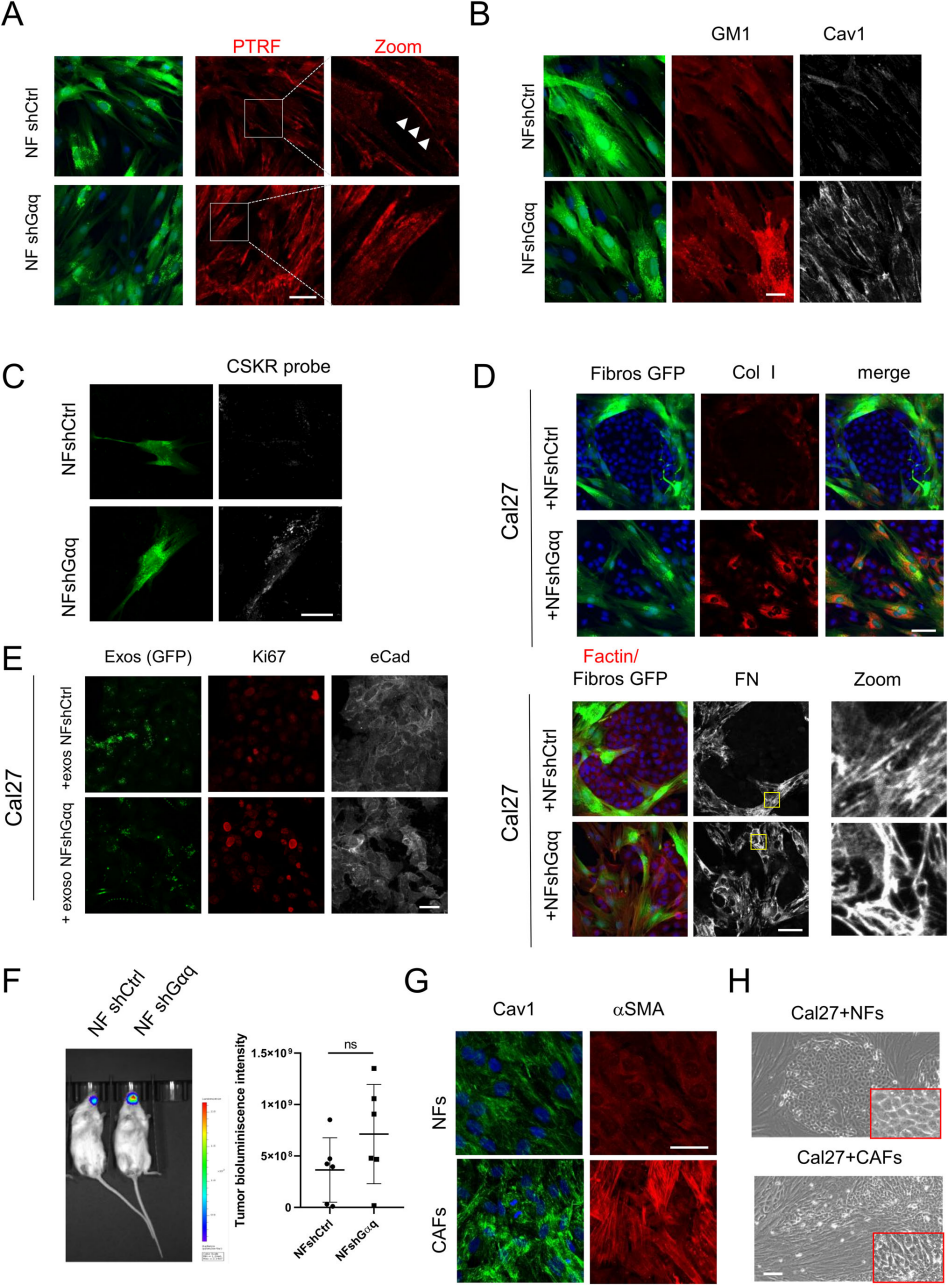
Effects of Gαq knockdown on normal human fibroblasts. Analysis by confocal microscopy of PTRF (**A**) or GM1 (**B**) staining in control (NF shctrl) and Gαq-silenced (NF shGαq) human fibroblasts (Scale bar, 25 µm). (**C**) Distribution of ceramide probe corresponding to eGFP-tagged KSR1 CA3 domain (KSR, aa 317–400) (ceramide-binding domain) (in gray) in NF shctrl and NF shGαq human fibroblasts (Scale bar 25 µm). (**D**) Analysis of matrix deposited in co-cultures of GFP-labeled NF shctrl or NF shGαq human fibroblasts with Cal27 cells. Fibroblasts are shown in green, FN in gray, collagen I in red, and DAPI-labeled nuclei in blue (Scale bar, 50 µm). (**E**) Staining pattern of Ki67 in Cal27 cells treated with exosomes derived from NF shctrl or NFs shGαq (Scale bar, 50 µm). (**F**) Representative bioluminescence images of orthotopic tongue tumors generated by co-injection of a luciferase-expressing tumor tongue cancer cell line (Cal27-luc) with either NF shctrl or NF shGαq human fibroblasts. (**G**) Confocal microscopy images displaying F-actin (green) and α-SMA (red) in NFs or CAFs (Scale bar, 50 µm). (**H**) Phase-contrast microscope images of one selected pair of NF-CAFs co-culture with Cal27 cells (Scale bar, 100 µm), showing differential morphological features. Data information: In (**F**) data were presented as mean ± SD, *n* = 6 animals per condition. Statistical significance in (**F**) was determined using an unpaired *t*-test (ns *p* = 0.1676). Source data are available online for this figure.

Although MEFs represent a robust and widely used experimental model, they do not fully reproduce the complexity of cancer-associated fibroblasts (CAFs). To enhance the translational value of our findings, we extended our study to normal human fibroblasts (NF), in which Gαq expression was downregulated using a lentiviral short-hairpin RNA approach (Fig. 7A). Human NFs with decreased Gαq levels displayed the same signature previously observed in GαqKO MEFs, consisting of an increased matrix deposition, upregulation of caveolar components, Cav1 and PTRF, and lysosomal marker LAMP3 expression levels, as well as increased intracellular accumulation of PDGFR, GM1 and the ceramide-binding probe CSKR (Figs. 7A,B and EV4A–C). In addition, in both 2D and 3D co-culture systems, Gαq-deficient human fibroblasts markedly altered the architecture, proliferation and invasion potential of Cal27 cells (Figs. 7C,D and EV4D), consistent with the effects previously observed in GαqKO MEFs. Furthermore, exosomes secreted by these fibroblasts were enriched in PDGFR (Fig. 7E), and able to promote in Cal27 tumor cells downregulation of Cav1 expression concomitant with an enhanced PDGFR presence (Fig. 7F) and an increased proliferation (Fig. EV4E), again similar to features of GαqKO MEFs-derived exosomes. Moreover, animals co-injected with Gαq-downregulated NFs also displayed increased tumor growth in vivo (Fig. EV4F). Overall, Gαq loss in both MEFs and human fibroblasts triggers similar CAF-like features and promotes a very close phenotype in Cal27 cells' tumoral progression.

**Figure EV5:**
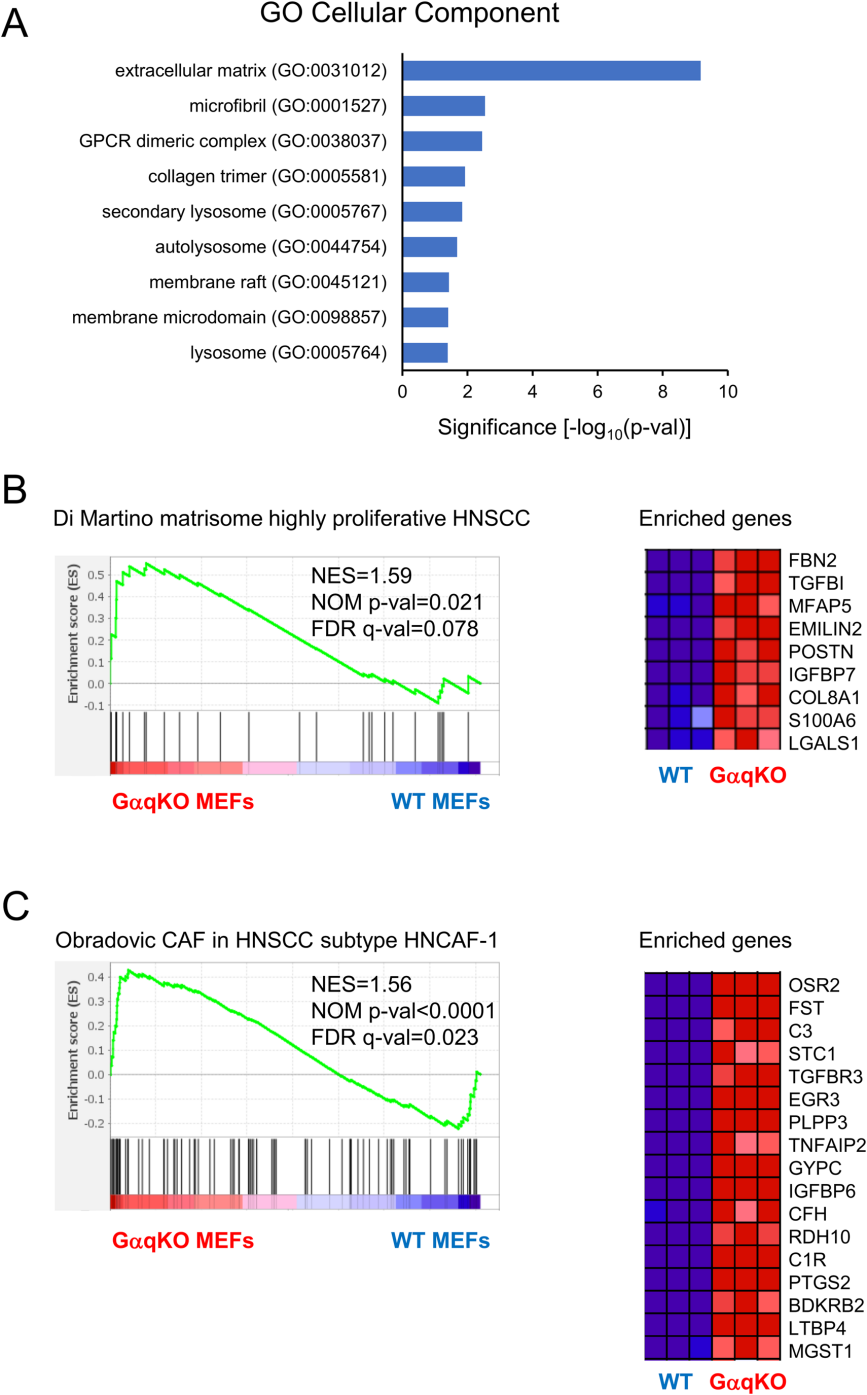
RNA-Seq profiling of WT and GαqKO MEFs. (**A**) Overexpressed genes in GαqKO versus WT MEFs showed overrepresentation of Gene Ontology cellular component terms related to extracellular matrix (ECM) organization and membrane microdomain remodeling, which validates our proteomic observations. Barplots represent the significance of the overrepresentation as -log_10_(*p* val) upon the hypergeometric distribution test. (**B**) GSEA analysis from GαqKO MEFs showed enrichment with a matrisome signature of highly proliferative HNSCC (Data ref: Di Martino et al, 2021a; Di Martino et al, 2021b). (**C**) GSEA analysis from GαqKO MEFs showed enrichment with a signature of the HNCAF-1 population of CAFs obtained from HNSCC, which is defined by an immunostimulatory phenotype and enriched in extracellular matrix organization and immune-related pathways (Obradovic et al, 2022). Enriched genes in (**B**, **C**) are shown on the right of each panel. See M&M sections for further details. Source data are available online for this figure.

To get insight into the clinical relevance of our findings, we performed an RNA-seq analysis comparing WT and GαqKO MEFs (Dataset EV3). Gene Ontology analysis revealed significant enrichment in pathways related to extracellular matrix (ECM) organization and membrane microdomain remodeling in GαqKO MEFs compared to WT, validating our proteomic observations (Fig. EV5A). Comparative analysis revealed that GαqKO MEFs share a strong and consistent signature associated to collagen matrix remodeling and broader ECM dynamics in HNSCC, as evidenced by their similarity to the matrisome profiles of highly proliferative HNSCC tumors (Fig. EV5B) as reported (Data ref: Di Martino et al, 2021a; Di Martino et al, 2021b). This correlation underscores the relevance of GαqKO MEFs as a model for studying tumor-associated fibroblast behavior in the context of hyperproliferative carcinoma. Furthermore, GαqKO MEFs show significant transcriptional similarity with the HNCAF-1 subtype (Fig. EV5C), a CAF population associated with poor overall survival in HNSCC, as detailed by Obradovic et al, (2022) in their Supplementary Table 2, who defined the signatures of the five CAF subtypes analyzed (Obradovic et al, 2022). The overlap with the HNCAF-1 signature further suggests that GαqKO MEFs adopt a pro-inflammatory phenotype and may contribute to immune modulation within the tumor microenvironment, both of which are key functional hallmarks of activated CAFs.

Finally, we explored whether Gαq expression is altered in human HNSCC-derived CAFs, by Western blot analysis of CAF subpopulations obtained from minced human tumor tissue of surgically resected HNSCC and of normal fibroblasts (NFs) obtained simultaneously from healthy tissue of the same patients. Although variability was observed between sample pairs and the differences did not reach statistical significance, we found a moderate but consistent downregulation of Gαq expression in the CAFs compared to NFs, which correlated with an upregulated PDGFR expression in such tumor fibroblasts (n of 6, Fig. 7G). In addition, CAFs showed a more spindle-like morphology compared to NFs, which was associated with a high intracellular accumulation of PDGFR and a marked deposition of ECM components such as FN and Col I (Fig. 7H), as well as enhanced Cav1 and αSMA labeling (Fig. EV4G). All these features were like those displayed by GαqKO MEFs or upon Gαq downregulation in human normal fibroblasts (NFs) described above.

Interestingly, the 2D co-culture of Cal27 oral cancer cells with patient-derived CAFs resulted in a phenotype characterized by a disorganized and more chaotic organization and reduced tumor cell-cell contact, again features observed upon co-culture with GαqKO MEFs or NF shGαq fibroblasts. In contrast, the presence of control NFs induced the formation of a highly confined and encapsulated tumor cell distribution (Fig. EV4H). In vivo, all these traits manifested as a notably aggressive and invasive tumor phenotype upon orthotopic tongue co-injection of bioluminescent Cal27 cells with CAFs, again resembling the features observed in GαqKO MEFs and NF shGαq cells, whereas tumors formed with NFs exhibited decreased size (Fig. 7I). Overall, our data provide compelling evidence that stromal Gαq expression plays a pivotal role in shaping the tumor microenvironment and controlling HNSCC tumor progression.

## Discussion

HNSCC is one of the most severe and challenging types of cancer due to its high level of heterogeneity and the limited availability of efficient therapeutic strategies (Hu et al, 2023). Continuous co-evolution and communication of tumor cells with the TME seems to be determinant in HNSCC progression (Jumaniyazova et al, 2023). Cancer-Associated Fibroblasts (CAFs) are key components of TME, and are essential in shaping tumor dynamics and driving HNSCC progression. Therefore, a better comprehension of the mechanisms that govern CAFs' features and their interactions with other TME components is crucial for developing targeted therapies that could improve treatment outcomes for HNSCC patients (Bhat et al, 2021; Hu et al, 2023; Mazilu et al, 2021).

**Figure 8:**
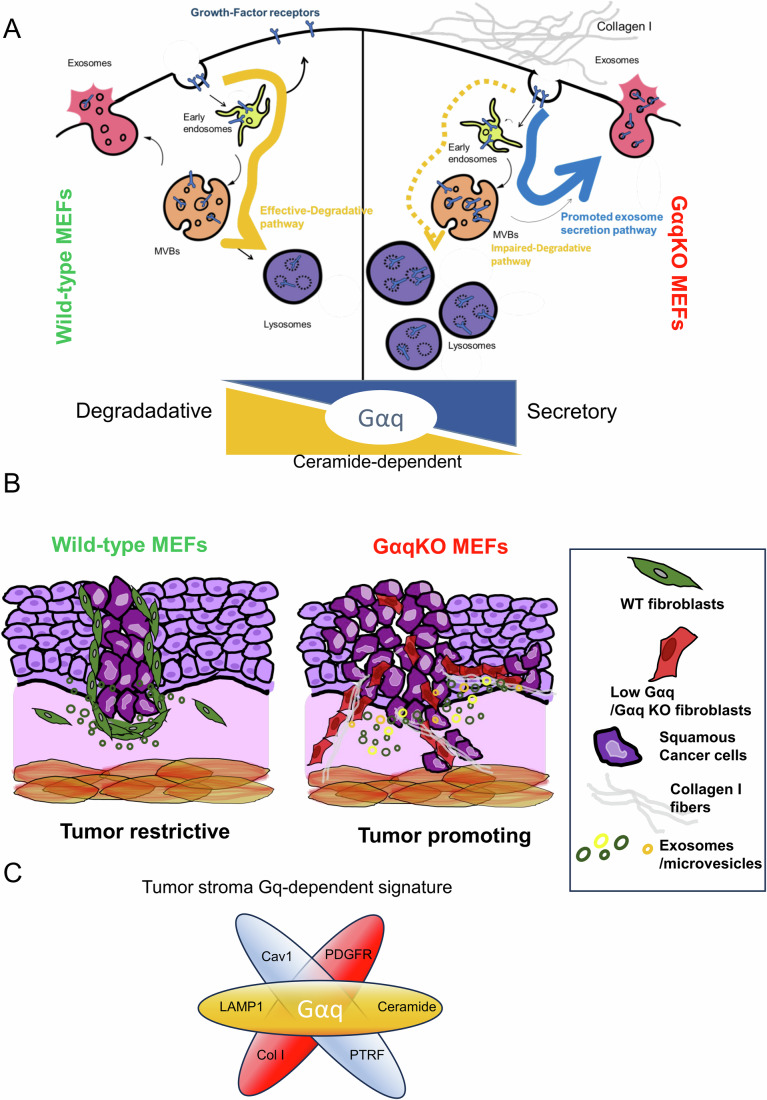
Proposed model for the role of stromal Gαq in HNSCC tumor progression. Gαq expression in the tumor stroma emerges as a key regulator of fibroblast traits and a key determinant of HNSCC progression. (**A**) The absence of Gαq expression in fibroblasts switches trafficking and degradative pathways towards a preferentially secretory phenotype through a ceramide-dependent mechanism. This shift favors the specific sorting of tumor growth factor receptors and their release via the exosomal pathway, rather than their lysosomal degradation, as observed in wild-type mouse embryonic fibroblasts (WT MEFs). Such an altered secretory profile is also reflected by significant collagen I matrix deposition in GαqKO MEFs. (**B**) At the functional level, a collagen I-rich matrix and the secretion of exosomes highly enriched in tumor growth factor receptors driven by GαqKO downregulation, fosters aberrant growth and dissemination of oral cancer cells. (**C**) A distinctive Gαq-deficiency molecular signature, driven by Cav1, PTRF, Col1, FN, and LAMP1, along with elevated ceramide, emerges as a novel HNSCC stromal biomarker.

We unveil here an unforeseen pivotal role of fibroblast Gαq in fostering HNSCC cancer progression by remodeling the tumor microenvironment (TME). MEFs lacking Gαq exhibit elevated expression of specific fibroblast activation markers and enhanced matrix remodeling and contractile features, resembling those of tumor-promoting CAFs. This phenotype is accompanied by profound alterations in endocytic and degradative pathways linked to disrupted lipid metabolism and ceramide accumulation, which impair PDGFR degradation and redirect its trafficking. Consequently, PDGFR and other growth factor receptors are selectively sorted into exosomes (Fig. 8A). Functionally, GαqKO MEFs and their exosomes support oral HNSCC cell growth and invasiveness in vitro and markedly enhance desmoplastic stroma formation in vivo (Fig. 8B). Similar features are observed upon Gαq downmodulation in normal human fibroblasts and in patient-derived HNSCC CAFs, underscoring the relevance of these mechanisms and warranting further investigation.

In general, ECM organization is a key determinant in tumor aggressiveness (Di Martino et al, 2021b; Pickup et al, 2014). In HNSCC, a strong desmoplastic reaction driven by CAFs enhances invasiveness (Knops et al, 2020). These cells exhibit diverse subpopulations with both tumor-promoting and tumor-suppressing roles, depending on their interaction with the ECM (Federspiel et al, 2025; Kang et al, 2021; Li et al, 2021). In this context, Gαq-deficient fibroblasts display a pro-tumorigenic phenotype, characterized by a collagen-rich, linearized matrix with a chaotic distribution that significantly promotes oral HNSCC cell invasion. Further supporting this phenotype, RNA-seq analysis of GαqKO fibroblasts uncovered a robust enrichment of a matrisome gene signature found in proliferative HNSCC (Di Martino et al, 2021b).

Regarding CAFs markers, α-SMA is particularly important in HNSCC, where its expression levels have also been shown to correlate with patient outcomes (Elbarrawy & Elbarrawy, 2021; Hu et al, 2023). Prolonged culture and confluence drive GαqKO MEFs toward a myofibroblast-like phenotype, characterized by a marked increase in α-SMA expression and enhanced contractility. Of note, our proteomic analysis also reveals that GαqKO MEFs exhibit traits of inflammatory fibroblasts, such as high expression of PLA2, CD34, and JAK/STAT pathways, and a strong transcriptional alignment with the pro-inflammatory HNCAF-1 subtype (Obradovic et al, 2022). These data strongly suggest that Gαq deficiency drives a pro-inflammatory, ECM-remodeling phenotype characteristic of activated CAFs. Further studies will be required to confirm this phenotypic switch.

PDGFR also serves as a key biomarker for cancer-associated fibroblasts (CAFs) in oral squamous cell carcinoma (Kartha et al, 2016). Its signaling depends on proper membrane organization, involving ligand binding, dimerization, phosphorylation, and endocytosis processes that typically occur via clathrin-coated pits, although caveolae can also initiate PDGFR signaling (Jastrzębski et al, 2017; Liu et al, 1996; Pandey et al, 2023). We uncover that Gαq deficiency profoundly disrupts PDGFR trafficking and degradation, resulting in its intracellular accumulation and increased exosomal release, along with other growth factor receptors, ultimately influencing tumor cell proliferation and growth. Although our data support that PDGFR is involved in the proliferative response induced by GαqKO-derived exosomes, additional cargo components, including other growth factor receptors, DNA, RNA, and microRNAs, may contribute to the observed phenotype. Isoform-specific functions of PDGFRα and PDGFRβ, as well as their stromal-specific roles in HNSCC progression, are also likely biologically significant. Future studies focused on PDGFR isoform-specific trafficking, processing, and signaling dynamics at the stromal level will be important for deepening our understanding of their contribution to tumor progression.

Under normal conditions, Gαq activates PLCβ to generate diacylglycerol (DAG) in response to stimulation of a variety of GPCRs. Upon Gαq depletion, we detect an unbalanced lipid metabolism leading to compensatory ceramide accumulation in GαqKO MEFs. Ceramide, produced through sphingomyelin hydrolysis, influences lysosomal membrane curvature and enzyme activity, and its accumulation is linked to lysosomal stress, defective autophagy, and impaired degradative capacity (Ermini et al, 2021; Paciotti et al, 2020; Tang et al, 2022). In parallel, ceramide promotes exosome biogenesis via nSMase2-dependent budding of multivesicular bodies, and pharmacological inhibition of nSMase2 markedly decreases exosome secretion (Skotland et al, 2017; Trajkovic et al, 2008). Therefore, ceramide accumulation could act as a molecular bridge between lysosomal dysfunction and enhanced exosomal signaling in Gαq-deficient cells.

At the plasma membrane, ceramide also displaces cholesterol from lipid rafts, reducing Caveolin-1 (Cav1) association (Yu et al, 2005), and modulates glycosphingolipid nanodomains as a precursor for gangliosides such as GM1 (Arumugam et al, 2021). Both effects are evident in Gαq-deficient MEFS, which display high Cav1 internalization and aberrant GM1 expression, features also observed upon Gαq silencing in normal human fibroblasts and in HNSCC-derived CAFs.

Consistent with these findings, proteomic analysis of GαqKO MEFs revealed downregulation of clathrin-mediated internalization and upregulation of caveolar proteins, particularly Cav1. Given that Cav1 regulates exosome biogenesis and cargo sorting via canonical and autophagy-dependent pathways (Albacete-Albacete et al, 2020; Ariotti et al, 2021), it is tempting to suggest that Cav1-dependent mechanisms may be involved in redirecting PDGFR from lysosomal degradation toward exosomal secretion upon Gαq loss in fibroblasts. The altered Rab GTPase expression pattern identified in our proteomic analysis further suggests a reprogramming of vesicular trafficking, shifting receptor turnover away from degradation and toward secretion (Blanc and Vidal, 2018; Brunel et al, 2021). Collectively, these observations indicate that loss of Gαq disrupts caveolae-dependent trafficking and endolysosomal homeostasis, thereby promoting pro-tumoral exosome release and signaling remodeling. Nevertheless, additional studies will be required to further define the precise mechanistic relationship underlying the rerouting of PDGR toward exosomal secretion.

From a functional perspective, we find that the presence of Gαq-depleted fibroblasts markedly affects Cal27 oral cancer cells' features by paracrine regulatory mechanisms, involving secreted factors. Exosomes enriched in PDGFR and other tumor-promoting growth factors secreted by fibroblasts upon Gαq downregulation drive aberrant Cal27 cells tumor proliferation and enhanced invasion and aggressiveness, both in vitro and in vivo. Mechanistically, we find that these exosomes induce a marked downregulation of Cav1 expression in recipient tumor cells. Cav1 is a well-established negative regulator of the ERK/MAPK pathway, by sequestering ERK signaling components within caveolar microdomains, and its loss is commonly associated with sustained ERK activation and enhanced cell proliferation (Cerezo et al, 2009; Fiucci et al, 2002). Thus, in our model, reduced Cav1 expression would enable persistent ERK-driven proliferation of Cal27 cells and foster tumor proliferation. Moreover, decreased Cav1 expression has been linked to epithelial-mesenchymal transition (EMT) and fibrotic remodeling, both processes known to facilitate tumor invasion and metastasis (Li et al, 2013; Navratil et al, 2025; Strippoli et al, 2015). Although treatment of Cal27 cells with GαqKO-derived exosomes did not elicit a fully developed EMT phenotype, since E-cadherin levels were not drastically reduced, we observed an altered E-cadherin membrane distribution and increased vimentin expression, indicating a partial EMT-like reprogramming. Further studies will be required to dissect the molecular interplay among ceramide metabolism, Cav1, and the endocytic machinery, aiming to better understand how Gαq modulates intracellular trafficking and to identify potential strategies to restore normal fibroblast phenotypes and counteract the CAF-like state.

Finally, and from a translational perspective, cancer-associated fibroblasts (CAFs) isolated from HNSCC patients exhibited a slight but consistent reduction in Gαq expression compared with normal fibroblasts; however, this difference did not reach statistical significance. Nevertheless, this downward trend may be biologically relevant given the pronounced heterogeneity of tumor fibroblast populations. Patient-derived CAFs also exhibited phenotypic features reminiscent of Gαq-deficient MEFs, including increased PDGFR expression with intracellular accumulation and enhanced collagen I deposition. Ongoing patient-matched and population-resolved quantitative analyses are expected to refine these observations and clarify their clinical relevance. Supporting this, silencing Gαq in normal human fibroblasts similarly induced CAF-like features, confirming a causal relationship. Functionally, both Gαq-deficient fibroblasts and patient-derived CAFs with low Gαq expression enhanced ECM remodeling and secretion, promoting the proliferation and invasiveness of oral HNSCC cells in 2D and 3D in vitro co-culture assays and fostering desmoplastic tumor formation in vivo (Fig. 8B). Collectively, these findings suggest that Gαq plays a pivotal role in maintaining stromal homeostasis and that its loss contributes to the establishment of a tumor-promoting microenvironment in HNSCC.

Although the mechanisms underlying reduced Gαq levels in CAFs remain to be elucidated, several regulatory layers may contribute to this phenotype. The GNAQ gene promoter activity can be modulated transcriptionally by the early growth response factor EGR-1 and by a functional polymorphism reported to alter transcription factor binding (Frey et al, 2008; Jalagadugula et al, 2006; Klenke et al, 2010). Epigenetic modifications (Wang et al, 2023) or microRNAs predicted to target GNAQ mRNA (Yang et al, 2021) have also been reported to modulate GNAQ expression. Furthermore, Gαq protein abundance is controlled post-translationally through ubiquitination and proteasome-dependent degradation (Johansson et al, 2005), which could also contribute to the modulation of Gαq levels in the tumor microenvironment.

In sum, our data strongly suggest that altered Gαq expression in the tumor stroma acts as a novel and critical regulator of HNSCC progression through two interconnected mechanism: (1) promotion of a collagen I-rich matrix and ECM remodeling that facilitate tumor dissemination, and (2) secretion of exosomes highly enriched in tumor growth factor receptors as a consequence of an imbalanced degradative capacity, thereby driving aberrant tumor growth. We further identify a distinctive molecular signature associated with Gαq deficiency, characterized by markedly elevated stromal expression of Caveolin-1, PTRF, Collagen I, Fibronectin, and LAMP1, alongside with increased ceramide accumulation (Fig. 8C). This profile reflects a profound rewiring of ECM architecture, vesicular trafficking, and metabolic pathways, including autophagy, degradation, and exosome biogenesis, and underscores the potential of Gαq-regulated processes as diagnostic or prognostic biomarkers in HNSCC.

A deeper understanding of how CAFs integrate these ECM-modifying and metabolic functions in a Gαq-dependent manner may guide the development of stromal-targeted therapeutic strategies to halt HNSCC progression.

## Methods

**Table:** Reagents and tools table

Reagent/resource	Reference or source	Identifier or catalog number
**Experimental models**		
NOD.Cg-Prkdc^scid^ Il2rg^tm1Wjl^/SzJ (*M. musculus*)		Dr. Miguel R. Campanero García (CBM, Spain)
Wild-type MEFs (*M. musculus)*	(Vogt et al, 2003)	Dr. S. Offermanns (Max-Planck-Institute for Heart and Lung Research, Germany)
Gαq/11-Knock-Out MEFs (*M. musculus)*	(Vogt et al, 2003)	Dr. S. Offermanns (Max-Planck-Institute for Heart and Lung Research, Germany)
UMSCC47 (*H. sapiens*)	(Madera et al, 2015)	Dr J.S Gutkind (UCSD, USA), originally from laboratory of Dr. Thomas Carey (University of Michigan)
HN13 (*H. sapiens*)	(Sobral et al, 2014)	Dr J.S Gutkind (UCSD, USA)
Cal27 (*H. sapiens*)	(Sobral et al, 2014)	Dr J.S Gutkind (UCSD, USA)
Normal fibroblast, NFs (*H. sapiens*)	(Prieto-Fernandez et al, 2023)	Dr. Juana García Pedrero and Dr. Saúl Álvarez Teijeiro (University of Oviedo, Spain)
Cancer-associated fibroblasts, CAFs (*H. sapiens*)	(Prieto-Fernandez et al, 2023)	Dr. Juana García Pedrero and Dr. Saúl Álvarez Teijeiro (University of Oviedo, Spain)
**Recombinant DNA**		
pSD44-Gαq	Addgene	N/A
shRNA h/m Gαq	Clontech	N/A
pIRSIN-Luc-IRES-GFPfp	(Stamatakis et al, 2022)	Patricia Fuentes (CBM, Spain)
CSKR probe	(Girik et al, 2024)	Paula Nunes (UNIGE, Switzerland)
Rab5Q79L	Addgene	#28046
pHAGE-PDGFRa	Addgene	#116769
pHAGE-PDGFRb	Addgene	#116770
**Antibodies**		
Rabbit anti-fibronectin	Merck	F3648
Rabbit anti-collagen I	Novus Biologicals	NB 600-408
Mouse anti-α-SMA	Invitrogen	14976082
Mouse anti-Ki67	Thermo Fisher	MS-1068
Rabbit anti-Caveolin-1	Cell Signaling	D46G3
Rabbit anti-PDGFR	Abcam	32570
Rabbit anti-PTRF	Proteintech	18892-1-AP
Rabbit anti-E-Cadherin	Cell Signaling	3195
Rabbit anti-Gαq	Abcam	75825
Mouse anti-αtubulin	Sigma	T5168
Mouse anti-GAPDH	Santa Cruz	sc-47724
Rabbit anti-Flotillin-1	Abcam	133497
Mouse anti-Alix	Cell Signaling	2171S
Mouse anti-Tsg101	Abcam	ab83
Rabbit anti-pERK1/2	Cell Signaling	9101
Mouse anti-ERK1/2	Cell Signaling	9107S
Mouse anti-Vimentin	Santa Cruz	Sc6260
Rabbit anti-Cyclin D1	Cell Signaling	2922
Rabbit anti-Keratin5	BioLegend	905504
Mouse anti-Lamp3	DSHB	H5C6
Rat anti-Lamp1	DSHB	1D4B
Rabbit anti-Histone H3	Abcam	Ab1791
Rabbit anti-FSP1	Sigma-Aldrich	ABF32
Rabbit anti-Osteopontin	Proteintech	22952-1-AP
Mouse anti-LBPA	Echelon	6C4
Goat anti-Mouse HRP	NordicMubio	GAM/IgG(H + L)/PO
Goat anti-Rabbit HRP	NordicMubio	GAR/IgG(H + L)/PO
DAPI	Merck	268298
Alexa 488 Donkey anti-Mouse IgG	Thermo Fisher	A-21202
Alexa 488 Donkey anti-Rabbit IgG	Thermo Fisher	A-21206
Alexa 488 Donkey anti-Rat IgG	Thermo Fisher	A-21208
Alexa 555 Donkey anti-Mouse IgG	Thermo Fisher	A-31570
Alexa 555 Donkey anti-Rabbit IgG	Thermo Fisher	A-31572
Alexa 555 Goat anti-Rat IgG	Thermo Fisher	A-21434
Alexa 647 Donkey anti-Mouse IgG	Thermo Fisher	A-31571
Alexa 647 Donkey anti-Rabbit IgG	Thermo Fisher	A-31573
Phalloidin Alexa Fluor 555	Thermo Fisher	A-34055
Cell Tracker Red CMTPX	Thermo Fisher	C34552
Cholera Toxin Subunit B (Recombinant), Alexa Fluor™ 594 Conjugate	Invitrogen	C34777
**Oligonucleotides and other sequence-based reagents**		
**Chemicals, enzymes and other reagents**		
Isoflurane	Covetrus	029405
D-luciferin	Promega	E1601
10% neutral-buffered formalin	Sigma-Aldrich	HT501128
Horse-Serum	Life technologies	26050-088
Sub-X mounting medium	Leica	3801740
4% paraformaldehyde	Santa Cruz	sc-281692
Triton X-100	Merck-Sigma	X100
Fluoromount-G	Fisher Scientific	#00-4958-02
Bovine Serum Albumin (BSA)	Roche	3117332001
Lipofectamine LTX and PLUS reagent	Invitrogen	15338-100
OptiMEM	Gibco Life Technologies	31985062
DMEM	Gibco Life Technologies	11965092
RNeasy Mini Kit	Qiagen	79306
Tween 20	Sigma-Aldrich	8221840050
Matrigel	Corning	354230
Western Lightning ECL plus	Perkin Elmer	NEL103E001EA
Tween 20	Sigma-Aldrich	8221840050
Nitrocellulose Membrane, 0.45 μm	Bio-Rad	1620115
Corning® Collagen I, High Concentration, Rat Tail	Sigma	354249
Sub-X Clearing Medium	Leica	3803670E
TMT10plex™	Thermo Fisher	90406
RNase-Free DNase Set	Qiagen	79254
Recombinant mouse PDGF-BB	PeproTech	315-18-50
AG1295	Millipore	658550
Bafilomycin A1	Sigma-Aldrich	B1793
Chloroquine	Sigma-Aldrich	C6628
YM254890	FUJIFILM Wako Pure Chemical Corporation	257-00631
**Software**		
GraphPad Prism 8.0.1	https://www.graphpad.com/	
ImageJ 1.50e	https://imagej.net/software/fiji/	
Proteome Discoverer 2.1.0.81	https://www.thermofisher.com/es/es/home/industrial/mass-spectrometry/liquid-chromatography-mass-spectrometry-lc-ms/lc-ms-software/multi-omics-data-analysis/proteome-discoverer-software.html	
iSanXoT	https://cnic-proteomics.github.io/iSanXoT/	
Image Lab 6.1	https://www.bio-rad.com/es-es/product/image-lab-software?ID=KRE6P5E8Z	
RStudio 2025.09.2	https://posit.co/download/rstudio-desktop/	
**Other**		
BD FACSAria Fusion	BD Biosciences	
AH 627 rotor	Sorvall	
SW40 Ti and SW28.1 Swinging-Bucket Rotor rotors	Beckman coulter	
Ultra-Clear centrifuge Tubes	Beckman coulter	344058
Ultra-Clear centrifuge Tubes	Beckman coulter	344060
Nanosight NS300	Malvern Panalytical	
Ultracentrifuge Beckman XP-100	Beckman coulter	
Direct Detect Spectrometer	Millipore	
Orbitrap Fusion mass spectrometer	Thermo Fisher	
Illumina NovaSeq 6000	Illumina	

### Animal model

NOD.Cg-Prkdcscid Il2rgtm1Wjl/SzJ (NOD-SCID IL2rγnull 4; NSG) immunodeficient mice were kindly provided by Dr. Miguel R. Campanero García from Centro de Biología Molecular Severo Ochoa (CBM). All mice were bred under specific pathogen-free conditions in accordance with CBM institutional guidelines. Experiments were performed in accordance with Spanish legislation for animal protection and were approved by the local governmental animal care committee (Proex 118.8-21). Orthotopic tongue injections of HNSCC were conducted on young mice ranging from 9 to 14 weeks old. To account for potential gender-associated variability, both males and females were included as part of our studies.

### Cell culture

Wild-type and Gαq/11-Knock-Out (referred to in the manuscript as GαqKO) mouse embryonic fibroblasts (MEFs) were kindly provided by Dr. S. Offermanns (Max-Planck-Institute for Heart and Lung Research, Germany). MEFs were cultured in Dulbecco´s modified Eagle medium (DMEM) supplemented with 10% fetal bovine serum (FBS), 100 U/ml penicillin and 100 µg/ml streptomycin at 37 °C in a humidified 5% CO_2_-95% air incubator.

Human oral squamous carcinoma cell lines (UMSCC47, HN13, and Cal27 (RRID:CVCL_1107), kindly provided by Dr J.S Gutkind, UCSD, USA) were cultured in DMEM/F-12 medium with 10% FBS, 100 U/ml penicillin and 100 µg/ml streptomycin. Cell lines were authenticated by short tandem repeat (STR) and tested for mycoplasma contamination.

Normal fibroblasts (NFs) and cancer-associated fibroblasts (CAFs) were kindly provided by Dr. Juana García Pedrero and Dr. Saúl Alvarez Teijeiro (Department of Otolaryngology and Instituto Universitario de Oncología del Principado de Asturias, University of Oviedo, Spain). Primary cancer-associated fibroblasts (CAFs) were isolated from freshly minced tumor tissue of surgically resected HNSCC patients at the Hospital Universitario Central de Asturias (HUCA). Normal fibroblasts (NF) were obtained from the non-tumoral counterpart tissue of these patients. All fibroblasts were cultured in DMEM supplemented with 10% fetal bovine serum (FBS) (Gibco, Waltham, MA, USA), 100 U/mL penicillin, 200 mg/mL streptomycin, 2 mM L-glutamine, 20 mM HEPES (pH 7.3) and 100 mM MEM non-essential amino acids. All cell lines were tested periodically for mycoplasma contamination by PCR. All procedures were conducted in accordance with institutional review board guidelines and were approved by the Regional Ethics Committee of the Principality of Asturias (CEImPA; approval date: March 9, 2023; approval number: 2023.018, corresponding to project PI22/00167). Written informed consent was obtained from all patients. HNSCC tissue samples were provided by the Biobank of the Principality of Asturias (PT23/00077), which is part of the Instituto de Salud Carlos III (ISCIII) Platform of Biobanks and Biomodels.

### Generation of lentiviral vectors and reconstituted GαqKO MEFs cell lines

A pSD44-Gαq wild-type vector (Addgene) was used for lentiviral reconstitution of GαqKO fibroblasts, generating GαqKI MEFs cell line. For Gαq knockdown, a shRNA targeting human/mouse Gαq (sequence: CACCAAGCTGGTGTATCAGAA) was cloned into the short-hairpin lentiviral vector pLVX-shRNA2, which contains a ZsGreen 1 reporter (Clontech), and the vector was used to transfect HEK293T cells to produce viral particles. Fibroblasts were exposed to viral supernatants, and infection efficiency was monitored by Zsgreen 1 expression. GFP-positive cells were isolated by cell sorting using a FACSAria Fusion system in the Flow Cytometry Service of the Centro de Biología Molecular Severo Ochoa.

### Generation of luciferase-expressing oral cancer cell lines

Cal27 cells were transduced with lentiviral particles generated with the pIRSIN-Luc-IRES-GFPfp vector, a generous gift from Patricia Fuentes (Centro de Biología Molecular, Spain), in the presence of 8 μg/ml Polybrene. GFP (and Luciferase)-positive cells were isolated by cell sorting using a FACSAria Fusion system in the Flow cytometry service of the Centro de Biología Molecular Severo Ochoa.

### Orthotopic injection of cancer cells and fibroblasts

A total volume of 50 μl was injected into the tongue of 9–14-week-old NSG mice, containing either 1 × 10^6^ Cal27 cancer cells or 1 × 10^6^ Cal27 cancer cells + 1 × 10^6^ of the indicated MEFs variants to induce orthotopic tumors. Cal27 cell line stably expresses GFP and luciferase. In the experiments performed using NFs and CAFs, the injected volumes contained either 750,000 Cal27 cancer cells or 750,000 Cal27 cancer cells + 750,000 NFs or CAFs. Tumor growth, in each condition, was monitored by bioluminescence imaging (IVIS Spectrum) after isoflurane anesthesia (1.5%, Abbott, Madrid, Spain) and intraperitoneal injection (i.p) of D-luciferin (150 mg/kg; Promega), according to the manufacturer’s instructions. Tumors were processed for histology and immunohistochemistry.

### Histological analysis and immunohistochemistry

Tissue samples were fixed in 10% neutral-buffered formalin (NBF) overnight and paraffin-embedded. Sections (5 μm) were stained with H&E or processed for immunohistochemistry. Briefly, the first step was deparaffinization of tissue sections by 15-min incubation at 55 °C and later submersion (three times in Sub-X Clearing Medium and three times in 100% ethanol, followed by 95, 70, and 50% ethanol and distilled water washes). Antigen retrieval was carried out in citrate buffer pH 6 for 5 min in the microwave and then cooled down 15 min at room temperature. After washing with PBS twice (5 min, each wash), sections were then incubated with blocking solution (3% Horse-Serum-PBS) for 1 h. Primary antibody dilution was made in blocking solution, and the incubation was carried out in the humidity chamber overnight at 4 °C. Sections were stained with antibodies against FN, Collagen I, α-SMA, Ki67, Caveolin-1, PDGFR, PTRF, and E-Cadherin. The following day, sections were washed extensively twice with PBS and once with 0.1% Tween-PBS. Next, fluorophore-conjugated secondary antibodies were diluted in blocking solution and incubated for 2 h (1:200), and afterwards, sections were washed twice with PBS and once with 0.1% Tween-PBS. Finally, the tissue sections were mounted and analyzed by confocal microscopy.

### Immunofluorescence microscopy of cultured cells

Cells grown on glass coverslips were washed twice with PBS and fixed for 15 min at room temperature in 4% paraformaldehyde in PBS. Fixed cells were washed extensively with PBS and permeabilized for 5 min in PBS containing 0.1% Triton X-100 and 2% BSA to reduce non-specific binding. Next, cells were incubated for 1 h at room temperature in PBS containing 0.2% BSA with primary antibodies (typically diluted 1:200). After washing, Alexa fluor-647 or 546 phalloidin, fluorescently labeled cholera toxin B subunit (CTB, por GM1 detection) or fluorescent secondary antibodies were applied in PBS for 1 h, at room temperature. Coverslips were mounted with Fluoromount-G^TM^ aqueous mounting medium and examined with a Laser Scanning Confocal Microscope LSM800 coupled to an inverted Axio Observer Microscope (Zeiss) typically through 40X (ACS APO 40.0×1.15 OIL) or 63X (ACS APO 63.0×1.30 OIL) objectives.

### Fibronectin fiber quantification

The procedure for image processing and quantification of fibronectin (FN) length, fiber size, and extracellular thickness was done using a macro in Fiji (ImageJ 1.50e x64) as described in (Albacete-Albacete et al, 2020).

### Western blot, dot blot, and cell fractionation

Cell proteins were resolved by SDS-PAGE and transferred to nitrocellulose membranes. Membranes were blocked for 2 h in TBS containing 5% bovine serum albumin (BSA) and incubated overnight at 4 °C with primary antibodies (typically diluted 1:1000). After washing, membranes were incubated for 1 h at room temperature with horseradish peroxidase-conjugated goat anti-rabbit or goat anti-mouse secondary antibody. Detection was performed by Western Lightning ECL plus (Perkin Elmer). Band intensities were quantified with Image Lab software (Bio-Rad).

For dot blot assays, samples (5 µL) were directly applied onto a nitrocellulose membrane and allowed to air dry. Subsequent steps, including blocking, antibody incubations and detection, were performed as described for Western Blot analysis.

### Preparation of detergent-resistant membrane fractions

Detergent-resistant membranes (DRMs) derived from WT or GαqKO MEFs lysates were purified as described in Navarro-Lerida et al. JCS (2002) (Navarro-Lérida et al, 2002). Briefly, two p150 plates of each fibroblast cell line were scraped and resuspended in 2 ml (final volume) of MES buffered saline (MBS: 25 mM MES, pH 6.5, 0.15 M NaCl, 1 mM PMSF plus 1% Triton X-100) at 4 °C. Cells were homogenized by a minimum of ten strokes through a syringe (0.5 × 16 mm) on ice. The homogenate was brought up to 4 ml by adding 2 ml of 80% sucrose in MBS and placed at the bottom of a 13 ml Ultraclear tube (Beckman SW40). The discontinuous sucrose gradient (40–30–5%) was formed by sequentially loading 4 ml 30% sucrose and 4 ml 5% sucrose in MBS. Cell fractions were separated by centrifugation at 200,000×*g* for 18 h (SW40 rotor, Beckman) at 4 °C. A light, scattered band confined to the 5–30% sucrose interface was observed that contained most Caveolin-1 protein and excluded most other proteins. Twelve (1 ml) fractions were collected, starting from the bottom of the tube. An equal volume of cold acetone was added to each fraction to precipitate proteins overnight at 4 °C. Protein pellets were collected by centrifugation at 16,000×*g*, air dried for 2 h to eliminate acetone traces, and finally, they were analyzed by SDS-PAGE and Western blot.

### Spheroid formation assay

After trypsinization, Cal27 cells were resuspended in FBS-free DMEMF12 medium, counted, and adjusted to the desired concentration (usually 1 × 10^4^ cells/ml). A final 20 µL drop containing the cell suspension and 5X methyl cellulose in FBS-free medium was placed on the inner surface of a 150 mm culture dish lid. The lid was then placed on a plate containing 10 ml PBS to humidify the culture chamber. Under gravity, cells aggregated at the bottom of the hanging drop. After 24 h, the resulting cell aggregates were lifted with a pipette. For 3D spheroid invasion assays, the aggregates were embedded into a 25-μl 1:1 mix of Matrigel and medium or, alternatively, spheroids were deposited onto a monolayer generated by WT/GαqKO fibroblasts, or alternatively by NF shctrl/NF shGαq. After 4-6 days, spheroids were fixed with PFA 4% in PBS and processed for immunofluorescence and confocal microscopy to monitor cell migration/invasion in each condition.

### Collagen gel contraction assay

The assay was performed as previously described (Goetz et al, 2011). Briefly, 2 × 10^5^ MEF cells of the different genotypes were mixed with NaOH-titrated collagen I (Corning) to obtain a final collagen I concentration of 1 mg/ml. The mixture was immediately transferred to a 24-well plate, and lattices were allowed to solidify. Serum-containing medium was then added to each well, and the gels were manually detached by circular movements using a sterile pipette tip. Gels were placed at 37 °C, and contraction was documented.

### Isolation and characterization of exosomes released by fibroblasts

Exosomes were isolated from fibroblasts cultured in exosome-free culture medium. Conditioned medium was first collected and centrifuged at 300×*g* for 10 min at 4 °C to remove detached cells. The supernatant was collected and centrifuged at 2000×*g* for 20 min at 4 °C. Next, the supernatant was then centrifuged at 10,000×*g* for 30 min at 4 °C to completely remove contaminating apoptotic bodies, microvesicles, and cell debris. The resulting clarified supernatant was then ultracentrifuged at 110,000×*g* for 70 min at 4 °C to pellet the exosomes. The supernatant was carefully removed, and crude exosome-containing pellets were washed in ice-cold PBS. After a second round of ultracentrifugation under the same conditions, the resulting exosome pellets were resuspended in the desired volume of PBS. Protein precipitates were monitored by Western blot for the expression of the exosomal markers Alix or Tsg101. In all experiments, the exosomes used corresponded to the total exosome pellets resulting from the serial centrifugation steps; usually, 10^6^ exosome particles were added per cell. Exosome uptake was performed in exosome-free medium. Exosome concentrations and size distributions were determined by Nanoparticle Tracking Analysis (Nanosight).

### Electron microscopy

Exosomes were visualized by transmission electron microscopy (TEM) according to the method of Thery et al, (2006) (Théry et al, 2006). Briefly, exosome suspensions were fixed in 2% PFA and transferred to formvar/carbon–coated EM grids. After 20 min, grids were placed sample-side down on a 100-μl drop of PBS for 2 min on a sheet of parafilm. Grids were then transferred to 1% glutaraldehyde for 5 min and then washed with distilled water. Samples were contrasted in 2% uranyl acetate and examined by TEM.

For cellular ultrastructure analysis, WT and GαqKO MEFs were processed for EM following standard procedures. Briefly, cells were fixed in 0.1 M cacodylate buffer, pH 7.4, containing 2.5% glutaraldehyde (+1 mg/ml ruthenium red), and then post-fixed in 1% osmium tetroxide (+1 mg/ml ruthenium red), followed by treatment with 2% uranyl acetate. The samples were dehydrated, embedded in LX112 Epon resin, ultrathin-sectioned, stained and imaged by TEM.

### Second harmonic generation microscopy

SHG imaging of collagen architecture in orthotopic tumors generated by injection of oral cancer cells and fibroblasts in the mice tongue was done as described (Provenzano et al, 2006).

### Proteomic analysis

Lysates derived from WT or GαqKO MEFs, as well as exosomes derived from these cell lines, were digested using the filter-aided sample preparation (FASP) protocol (Wiśniewski et al, 2009). Briefly, samples were dissolved in 50 mM Tris-HCl, pH 8.5, 4% SDS, and 5 mM Tris(2-carboxyethyl) phosphine (TCEP), boiled for 10 min, and centrifuged. Protein concentration in the supernatant was measured with the Direct Detect® Spectrometer (Millipore). About 100 μg of protein were diluted in 8 M urea in 0.1 M Tris-HCl, pH 8.5 (UA buffer) containing 5 mM TCEP and loaded onto 30 kDa centrifugal filter devices (Millipore). After the reduction buffer was removed by washing with UA, proteins were alkylated by incubation in 20 mM iodoacetamide in UA for 20 min in the dark. Alkylation reagents were removed by washing three times with UA, followed by three additional washes with 50 mM ammonium bicarbonate. Proteins were digested overnight at 37 °C with modified trypsin (Promega) in 50 mM ammonium bicarbonate at a 40:1 protein:trypsin (w/w) ratio. The resulting peptides were eluted by centrifugation with 50 mM ammonium bicarbonate (twice) and 0.5 M sodium chloride. Trifluoroacetic acid (TFA) was added to a final concentration of 1%, and the peptides were finally desalted onto Oasis-HLB cartridges and dried-down for further analysis.

For the quantitative analysis, tryptic peptides were dissolved in 150 mM triethylammonium bicarbonate (TEAB) buffer, and the peptide concentration was determined by measuring amide bonds with the Direct Detect system. Equal amounts of each peptide sample were labeled using the 10plex TMT Multiplex reagents (Thermo Fisher). Briefly, each peptide solution was independently labeled at room temperature for 1 h with one TMT reagent vial previously reconstituted with acetonitrile (ACN). After incubation at room temperature for 1 h, the reaction was stopped with diluted TFA and peptides were combined. Samples were concentrated in a Speed Vac, desalted onto Oasis-HLB cartridges, and dried-down for mass spectrometry (LC-MS/MS) analysis.

Digested peptides were loaded into the LC-MS/MS system for online desalting onto C18 cartridges and analyzed by LC-MS/MS using a C18 reversed phase nano-column (75 µm I.D. × 50 cm, 2 µm particle size, Acclaim PepMap RSLC, 100 C18; Thermo Fisher Scientific) in a continuous acetonitrile gradient consisting of 0–30% B for 360 min and 50–90% B for 3 min (A = 0.1% formic acid; B = 100% acetonitrile, 0.1% formic acid). A flow rate of 200 nl/min was used to elute peptides from the RP nano-column to an emitter nanospray needle for real-time ionization and peptide fragmentation in an Orbitrap Fusion mass spectrometer (Thermo Fisher). During the chromatography run, we examined an enhanced FT-resolution spectrum (resolution = 70,000) followed by the HCD MS/MS spectra from the nth-most intense parent ions. Dynamic exclusion was set at 40 s. For increased proteome coverage, labeled samples were also fractioned by high-pH reverse phase chromatography using C18 microcartridges (Thermo Fisher). Fractions were analyzed using the same system and conditions described before.

Protein identification and quantification. All spectra were analysed with Proteome Discoverer (version 2.1.0.81, Thermo Fisher Scientific) using SEQUEST-HT (Thermo Fisher Scientific). The Uniprot database, containing all mouse sequences (March 03, 2013) concatenated with decoy sequences generated using DecoyPyrat (Wright and Choudhary, 2016), was searched with the following parameters: trypsin digestion with two maximum missed cleavage sites; precursor and fragment mass tolerances of 2 and 0.03 Da, respectively; methionine oxidation as a dynamic modification; and carbamidomethyl cysteine and N-terminal and Lys TMT6plex modifications as fixed modifications.

For quantitative analysis, the iSanXoT program (Rodríguez et al, 2024) quantifies the intensity of TMT reporter ions derived from the isobaric labeling of fragmentation spectra. First, the false discovery rate (FDR) was calculated using the corrected Xcorr score (cXcorr) (Choi and Nesvizhskii, 2008; Keller et al, 2002), with an additional filter for precursor mass tolerance of 12 ppm (Bonzon-Kulichenko et al, 2015). Identified peptides had an FDR of 1% or lower.

Next, peptide-to-protein assignment was performed using an in-house developed method, where each peptide is assigned to the most likely protein in the UniProtKB/Swiss-Prot database. Proteins are ranked according to the number of peptides with which they are identified. The algorithm assigns each peptide to the protein with the highest number of peptides. In cases of tied proteins, it prioritizes assignments based on the number of peptide-spectrum matches.

Subsequently, quantitative information from TMT reporter intensities was integrated from the spectrum level to the peptide level, and then to the protein level, to quantify the relative abundance of each protein. This was done according to the WSPP (weighted spectrum, peptide, and protein) statistical model (Navarro et al, 2014) and the generic integration algorithm (GIA) (García-Marqués et al, 2016). This model standardizes peptide and protein abundance quantification as log_2_-ratios, with values expressed in units of standard deviation for peptides (Zp) and proteins (Zq) according to their estimated variances.

Differences in protein abundance or functional behavior were estimated by comparing the groups’ Zq or Zc medians, respectively, as determined by the WSPP statistical model. Proteins or functional changes were considered statistically significant with a two-sided *t*-test comparison *p* value <0.05 of the *Z* values. Functional enrichment analysis was assessed by GSEA using the IPA™ (Qiagen) and DAVID resources; gene subsets related to ECM remodeling were further manually curated from the MSigDB hallmark gene set collection 75. Visualization of enriched functional annotation terms was performed using the open source REVIGO suite and or GOrilla software.

The mass spectrometry proteomics data have been deposited to the ProteomeXchange Consortium via the PRIDE (Perez-Riverol et al, 2025) partner repository with the dataset identifier PXD061641.

### Plasmid transfection

Cells were transfected with the desired plasmids using Lipofectamine™ 2000 (Thermo Fisher Scientific) combined with LTPXplus according to the manufacturer’s protocol. Briefly, plasmid DNA and transfection reagents were mixed in Opti-MEM® reduced-serum medium and incubated for complex formation before being added to cultured cells. After 48 h, transfection efficiency was assessed by confocal microscopy or Western blot.

### RNA sequencing and analysis

Total RNA was isolated from GαqKO and WT MEFs (three biological replicates each genotype) using the miRNeasy Mini Kit (Qiagen), and genomic DNA was removed using the RNase-Free DNase Set (Qiagen) according to the manufacturer’s instructions. RNA sequencing was performed after poly-A selection and using Illumina NovaSeq 2 × 150 bp, reads were aligned to the GRCm39 mouse genome with “Rbowtie2”, counts extracted with the feature Counts function from “Rsubread” and gencode annotation release 32 (gencode.vM32.annotation.gft). Differential expression analysis was performed with DESeq2 in R. Significantly deregulated genes were considered if log2 ratio >1 (UP) or <−1 (DOWN), *p* value <0.05, and *p*_adjust_ < 0.05. Gene Ontology overrepresentation of terms was performed using the ToppFun utility within the ToppGene Suite (https://toppgene.cchmc.org/) using the UP genes. Raw data has been deposited on the GEO database under the accession number GSE312614.

Enrichment analysis was performed using gene set enrichment analysis (GSEA). Normalized counts were used to run comparisons between “GαqKO versus WT MEFs”, and using the gene sets of the MSigDB database from the GSEA webpage corresponding to Di Martino et al (2021a) and Obradovic et al (2022) reports (Data ref: Di Martino et al, 2021a; Di Martino et al, 2021b; Obradovic et al, 2022). Significant enrichment of individual gene sets was considered when both NOM *p* val <0.05 and FDR *q* val <0.25 threshold was found. The normalized enrichment score (NES) is the primary statistic for examining gene set enrichment results. NES can be used to compare analysis results across gene sets.

### Reagents and plasmids

The following primary antibodies were used: anti-PTRF (18892-1-AP) and anti-Osteopontin (22952-1-AP) were from Proteintech. TSG101 (AB83), PDGFR (ab32570), flotillin-1 (ab133497) and GNAQ (ab75825), and anti-Histone3 (Ab1791) were from Abcam. Anti-tubulin (T5168), anti-Fibronectin (F3648), and FSP1 (ABF32) were from Sigma. Anti-Smooth muscle actin (14976082) was from Invitrogen. Rabbit monoclonal Caveolin-1 (D46G3), Alix (3A9), anti-pERK1/2 (9101), anti-ERK1/2 (9107S), anti-Cyclin D1 (2922) and E-Cadherin (3195) were from Cell Signaling; Rat LAMP1 (1D4B) and mouse LAMP3 (H5C6) were from Hybridoma Bank. Anti-LBPA (6C4) was from Echelon (Z-LBPA). Anti-collagen I (NB 600-408) was from Novus Biologicals. Anti-keratin5 (905504) was from BioLegend. Anti-GAPDH (sc-47724) was from Santa Cruz. Cholera Toxin Subunit B-alexa Fluor 594 conjugated (C34777) was from Invitrogen. Alexa fluor 488, 546 and 647-conjugated phalloidin and fluorescent secondary antibodies were from Thermo Fisher. Matrigel (354230) and rat tail Collagen I (354249) were from Corning. Bafilomycin (1 nM) and Chloroquine (1 µM) were purchased from Sigma-Aldrich. AG1295 (PDGFR inhibitor, 10 µM Millipore) or the specific Gαq/11/14 inhibitor YM254890 (5 µM, Wako) were used as indicated in the figure legends. Recombinant mouse PDGF-BB protein was obtained from PeproTech (315-18-50). For the generation of enlarged endosomes/MVBs, cells were transfected with a plasmid expressing the constitutive active form of Rab5 (Rab5 (Q79L)).

For ceramide detection, we employed the plasmid encoding to eGFP-tagged C-KSR1 CA3 domain (KSR, aa 317–400) (ceramide-binding domain) described in (Girik et al, 2024). The plasmids encoding the PDGFRα and PDGFRβ isoforms (pHAGE-PDGFRa and pHAGE-PDGFRb) were obtained from Addgene, originally deposited by Dr. Gordon Mills and Kenneth Scott. We acknowledge them for providing this resource (Ng et al, 2018).

### Statistical analysis

Sample size was chosen based on previous experience, on the type of experiment and on the anticipated variation according to previous experience from studies using related methods (Cabezudo et al, 2021; Palacios-García et al, 2020). The number of animals used was estimated considering the minimum number to obtain statistical analysis to determine if there were differences between groups with a confidence level of 95% (*p* < 0.05). We used at least five to six animals per group condition, per experiment.

Sample organism participants were randomly allocated into experimental groups. In the process of analyzing experimental results, there was no blind assignment of researchers.

Error bars depict SEM or SD as indicated in figure legends. Statistical significance was determined with GraphPad Prism 8.0.1 Software (Graphpad, San Diego, CA). Normality was assessed using the Shapiro–Wilk test, and homogeneity of variances was evaluated with the *F* test. Statistical significance was determined using an unpaired Student’s *t*-test for pairwise comparisons or one-way ANOVA for multiple group comparisons. When significant differences in variances were detected, Welch’s correction was applied. For data that did not follow a normal distribution, the Mann–Whitney *U*-test and the Kruskal–Wallis test were used for pairwise and multiple comparisons, respectively; *p < 0.05, **p < 0.01, ***p < 0.001.

## Supplementary information

Peer Review File

Dataset EV1

Dataset EV2

Dataset EV3

Source data Fig. 1

Source data Fig. 2

Source data Fig. 3

Source data Fig. 4

Source data Fig. 5

Source data Fig. 6

Source data Fig. 7

Figure EV1 Source Data

Figure EV2 Source Data

Figure EV3 Source Data

Figure EV4 Source Data

Figure EV5 Source Data

Expanded View Figures

## Data Availability

The datasets produced in this study are available in the following databases: Proteomics data: The mass‑spectrometry proteomics datasets derived from lysates of WT and GαqKO MEFs, as well as their corresponding exosomes, have been deposited in the ProteomeXchange Consortium via the PRIDE partner repository (Perez-Riverol et al, 2025), under the accession number PXD061641. : https://proteomecentral.proteomexchange.org/ui?pxid=PXD061641. RNA‑seq data: RNA‑sequencing datasets generated from lysates of wild‑type (WT) and GαqKO MEFs are available in the Gene Expression Omnibus (GEO) under accession GSE312614, https://www.ncbi.nlm.nih.gov/geo/query/acc.cgi?acc=GSE312614. The source data of this paper are collected in the following database record: biostudies:S-SCDT-10_1038-S44319-026-00751-2.
