# Supplementary figures and images for: Loss of Gαq reshapes fibroblast traits and drives tumor-stroma remodeling in oral cancer progression (part 1 of 5)
Source: EMBO Rep. 2026 Apr 10;27(10):2639–74. doi: 10.1038/s44319-026-00751-2 (PMC13219523; doi:10.1038/s44319-026-00751-2)

## Slide 1
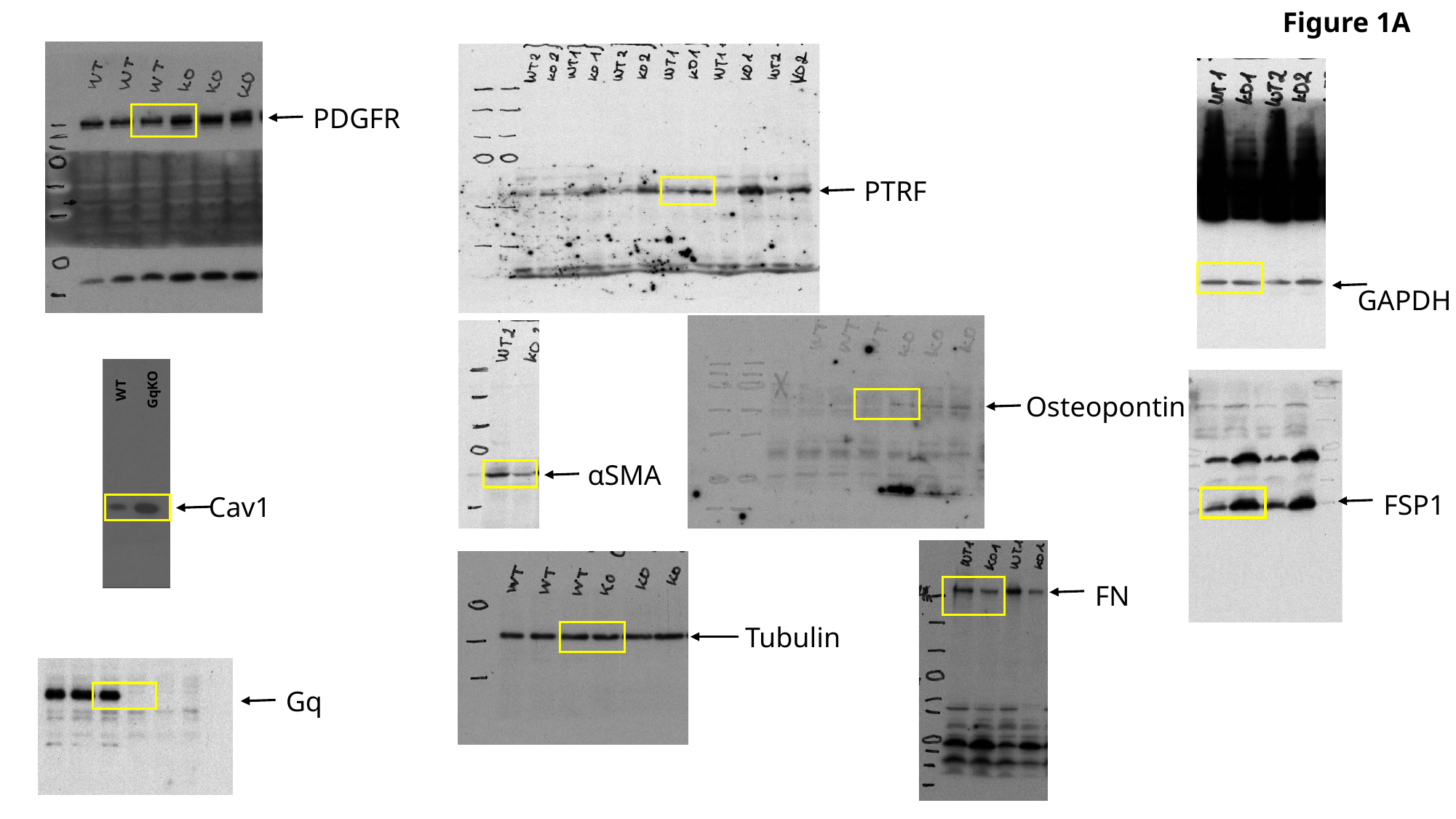

Figure 1A
PDGFR
PTRF
GAPDH
GqKO
WT
Osteopontin
αSMA
FSP1
Cav1
FN
Tubulin
Gq

Supplement: Supplementary file 5 — Source data Fig. 1 [file 44319_2026_751_MOESM5_ESM.zip › Raw_data_Figure 1/Figure 1A/raw_blots_1A.pptx]

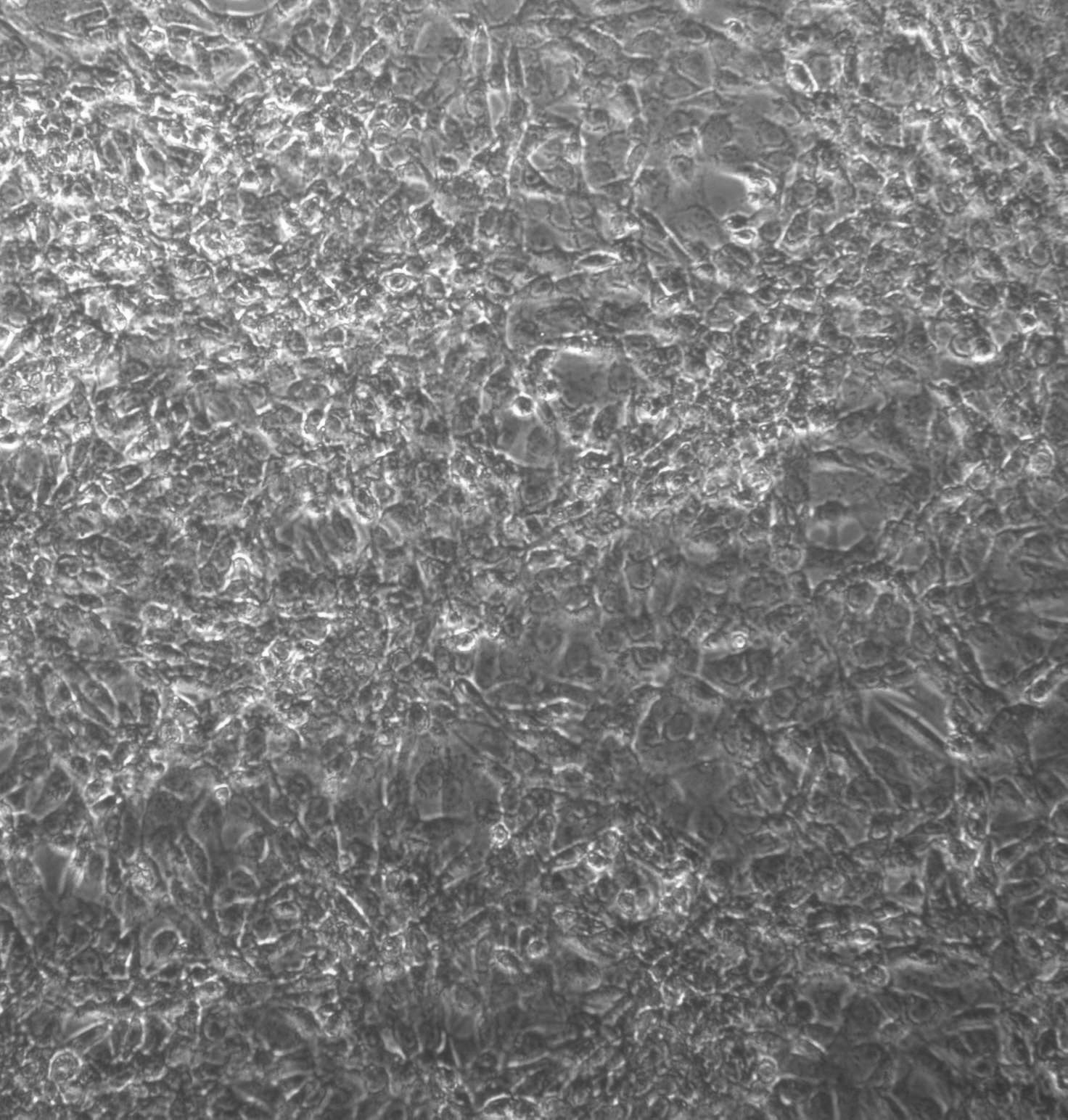

Supplement: Supplementary file 5 — Source data Fig. 1 [file 44319_2026_751_MOESM5_ESM.zip › Raw_data_Figure 1/Figure 1B/Bright Field microscopy/Fibros KO confluent b.jpg]

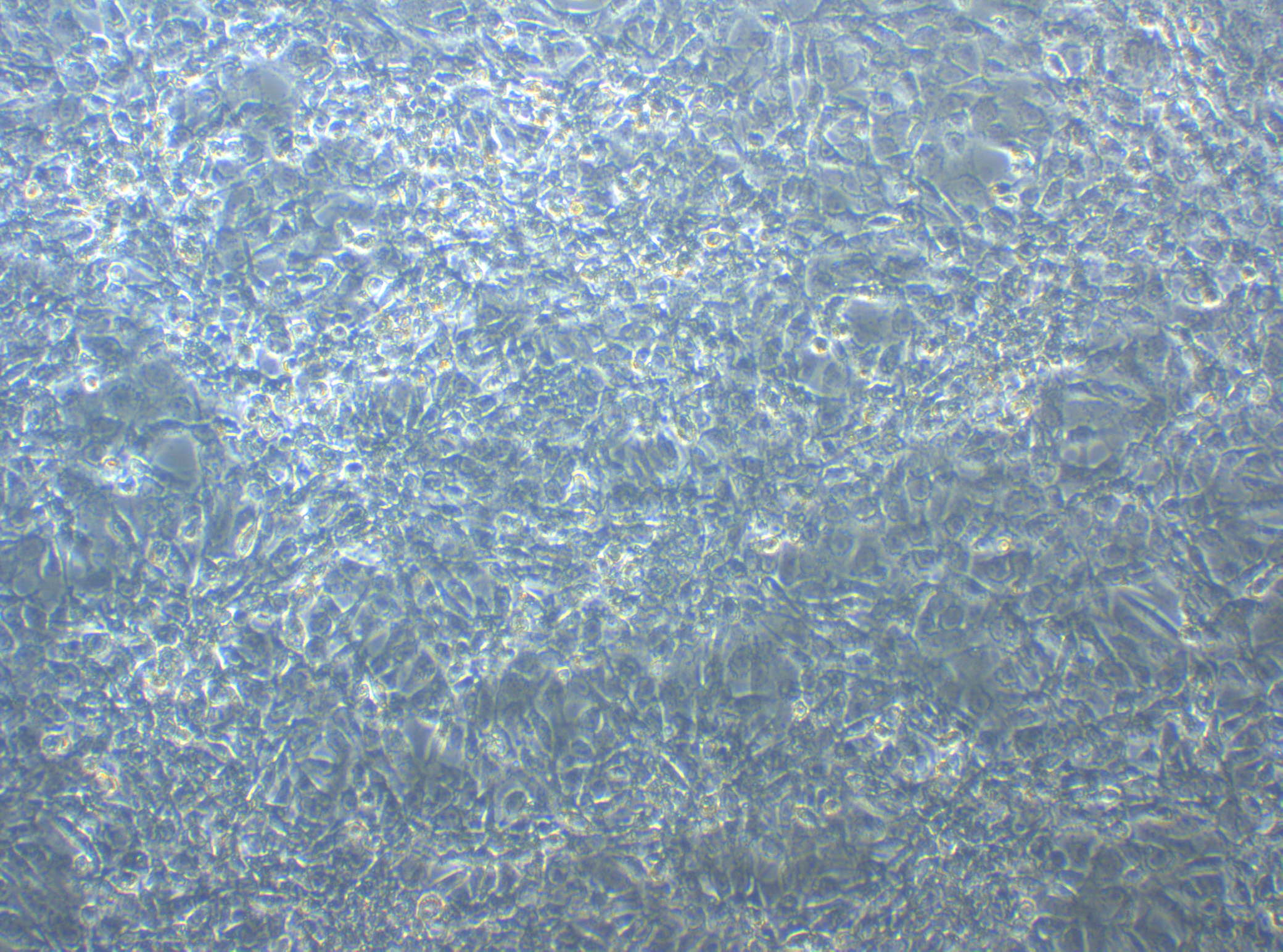

Supplement: Supplementary file 5 — Source data Fig. 1 [file 44319_2026_751_MOESM5_ESM.zip › Raw_data_Figure 1/Figure 1B/Bright Field microscopy/Fibros KO confluent.jpg]

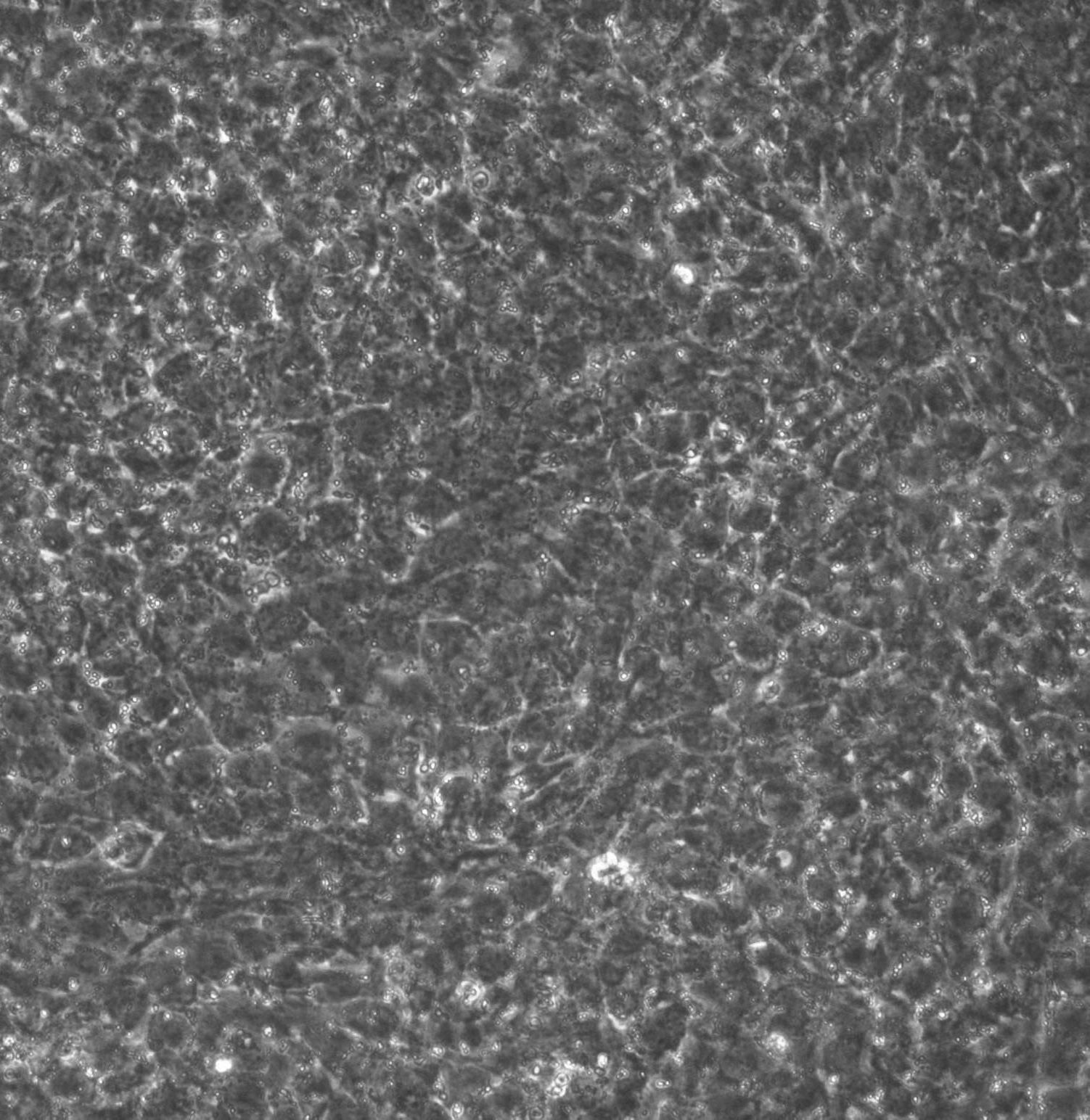

Supplement: Supplementary file 5 — Source data Fig. 1 [file 44319_2026_751_MOESM5_ESM.zip › Raw_data_Figure 1/Figure 1B/Bright Field microscopy/Fibros WT confluent b.jpg]

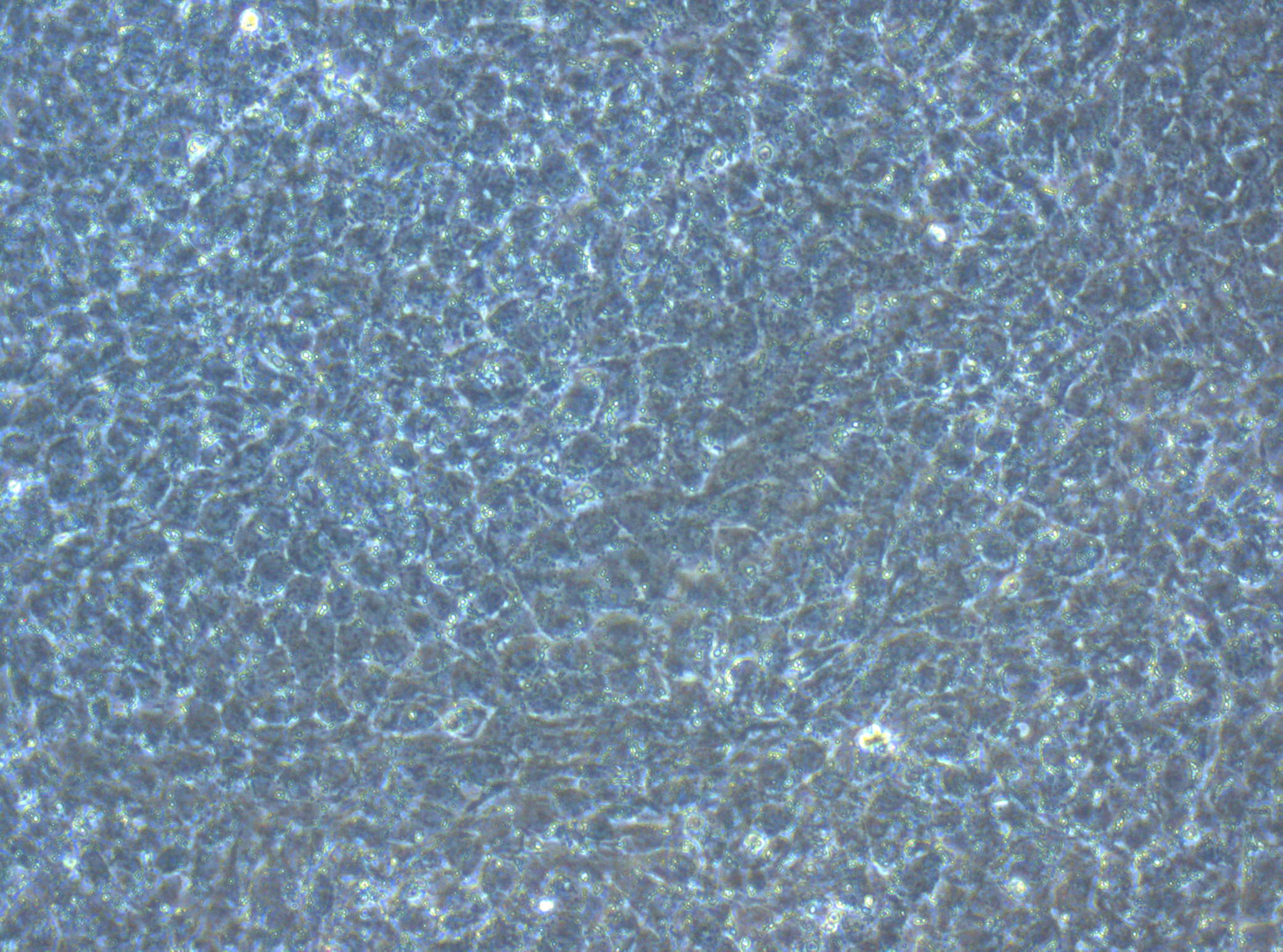

Supplement: Supplementary file 5 — Source data Fig. 1 [file 44319_2026_751_MOESM5_ESM.zip › Raw_data_Figure 1/Figure 1B/Bright Field microscopy/Fibros WT confluent.jpg]

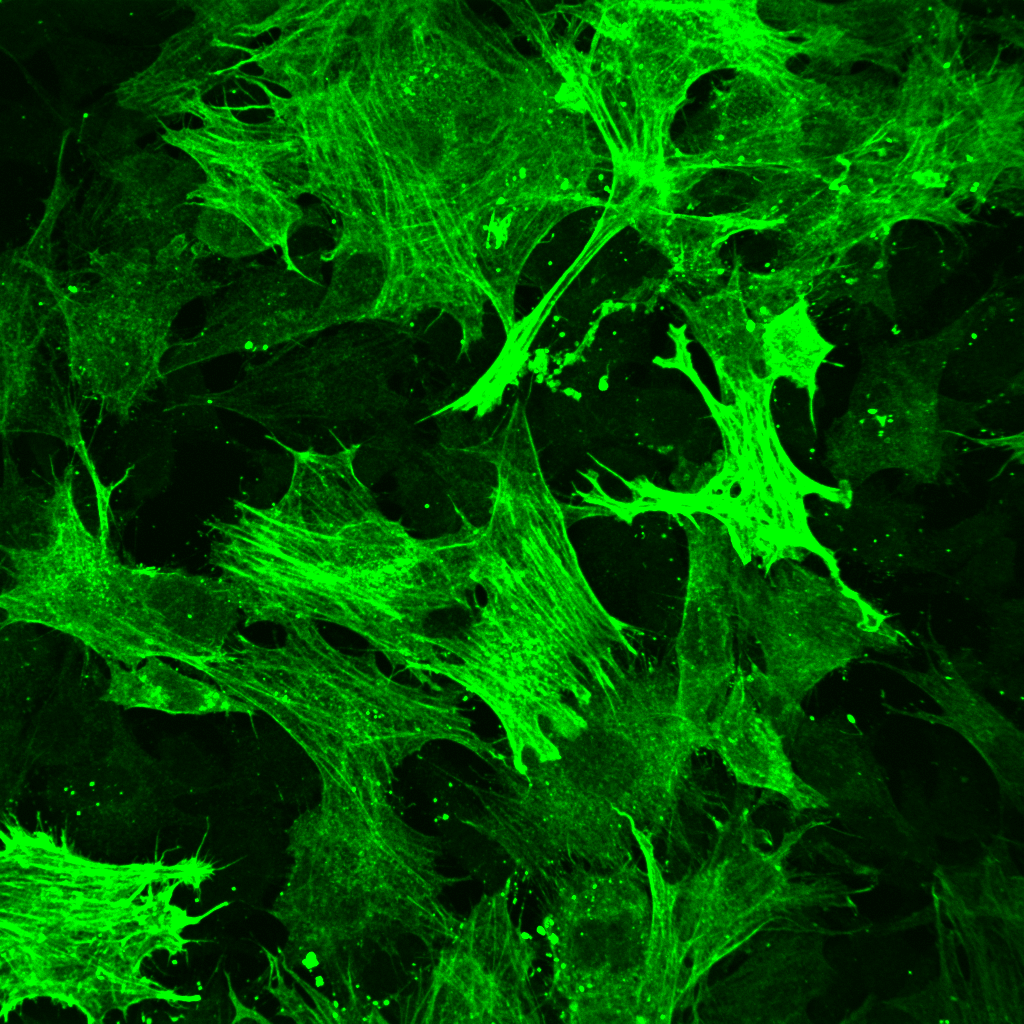

Supplement: Supplementary file 5 — Source data Fig. 1 [file 44319_2026_751_MOESM5_ESM.zip › Raw_data_Figure 1/Figure 1B/Confocal microscopy/KO 0.1%SMA.tif]

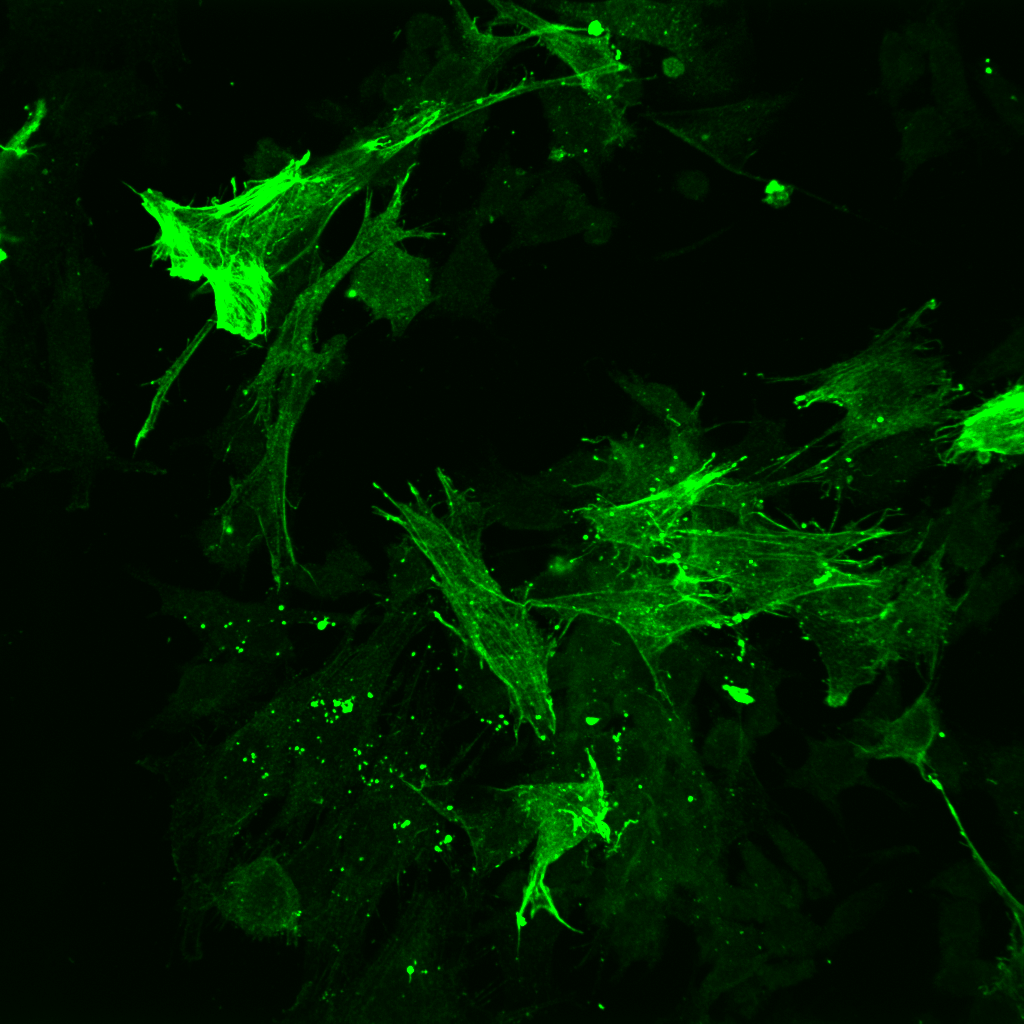

Supplement: Supplementary file 5 — Source data Fig. 1 [file 44319_2026_751_MOESM5_ESM.zip › Raw_data_Figure 1/Figure 1B/Confocal microscopy/KO SMA.tif]

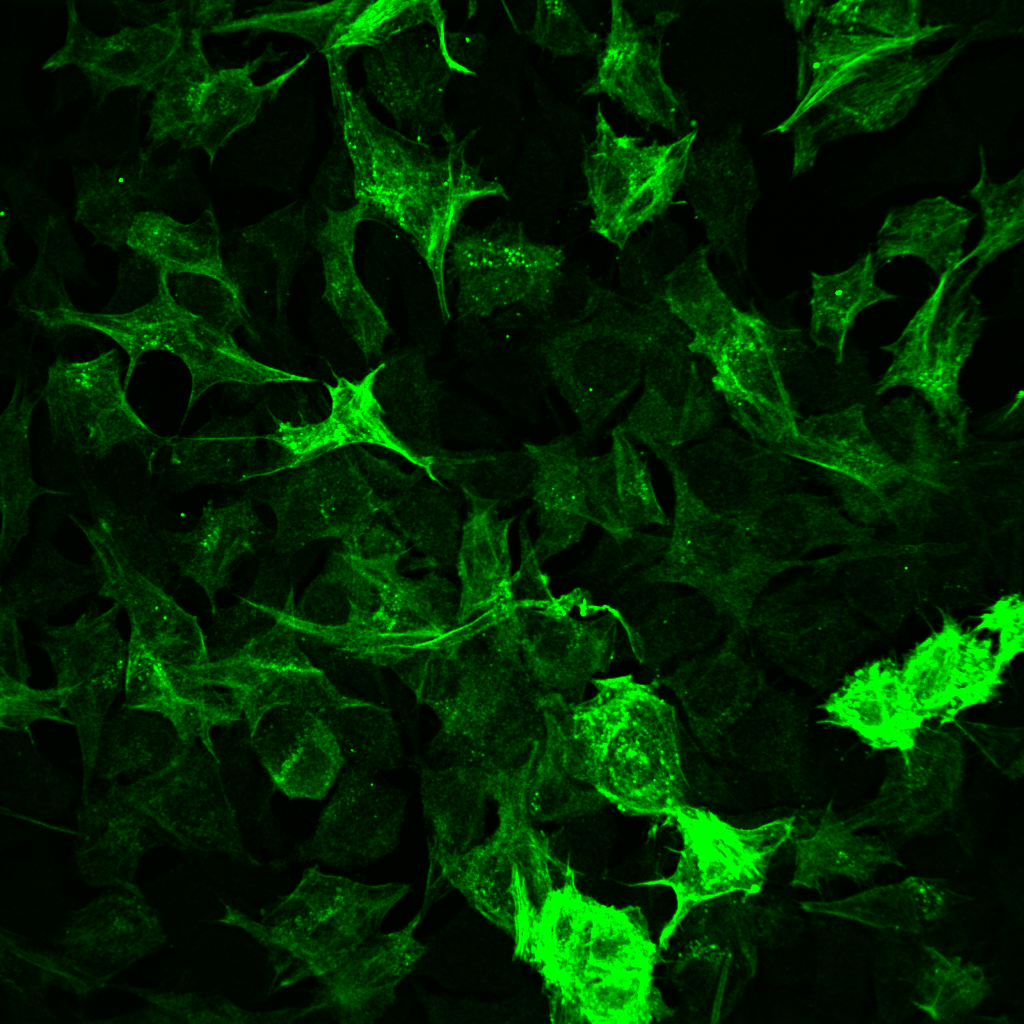

Supplement: Supplementary file 5 — Source data Fig. 1 [file 44319_2026_751_MOESM5_ESM.zip › Raw_data_Figure 1/Figure 1B/Confocal microscopy/WT 0.1%SMA.tif]

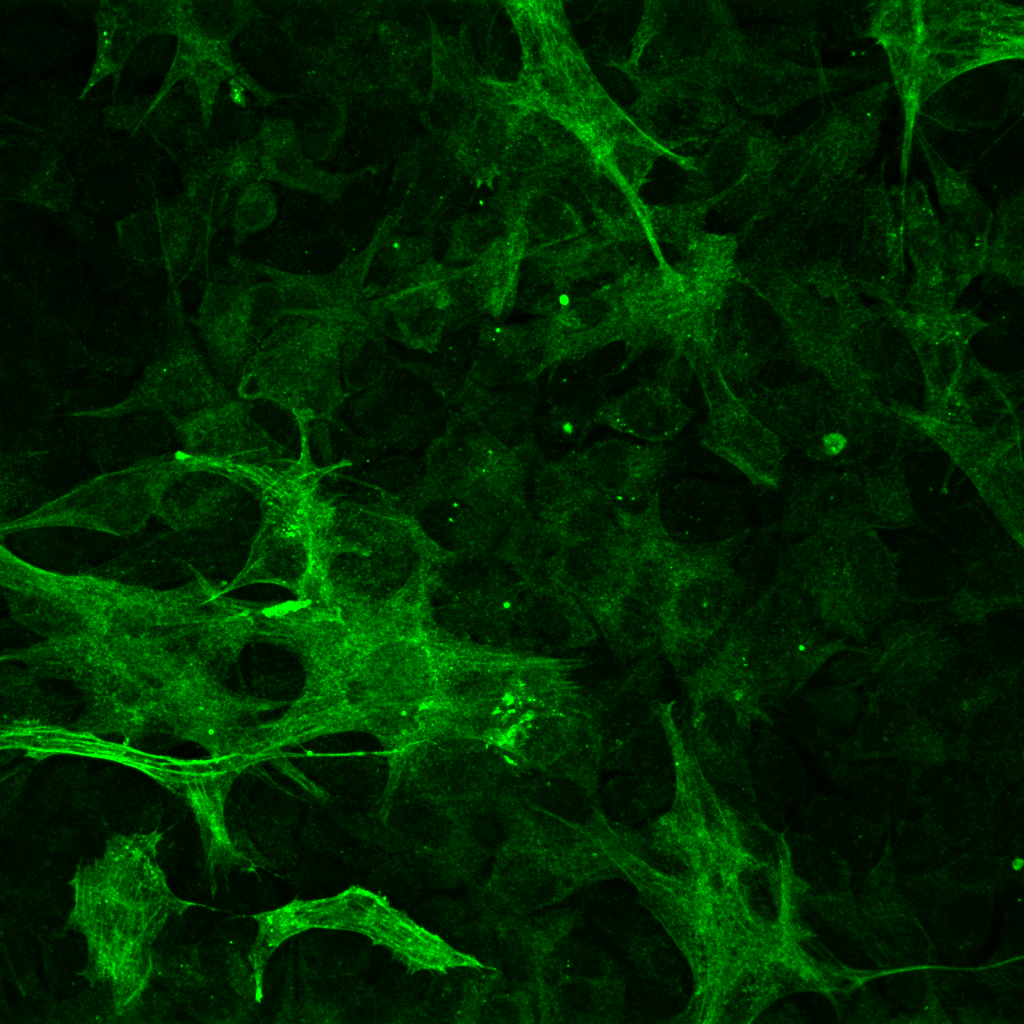

Supplement: Supplementary file 5 — Source data Fig. 1 [file 44319_2026_751_MOESM5_ESM.zip › Raw_data_Figure 1/Figure 1B/Confocal microscopy/WT SMA.tif]

## Slide 1
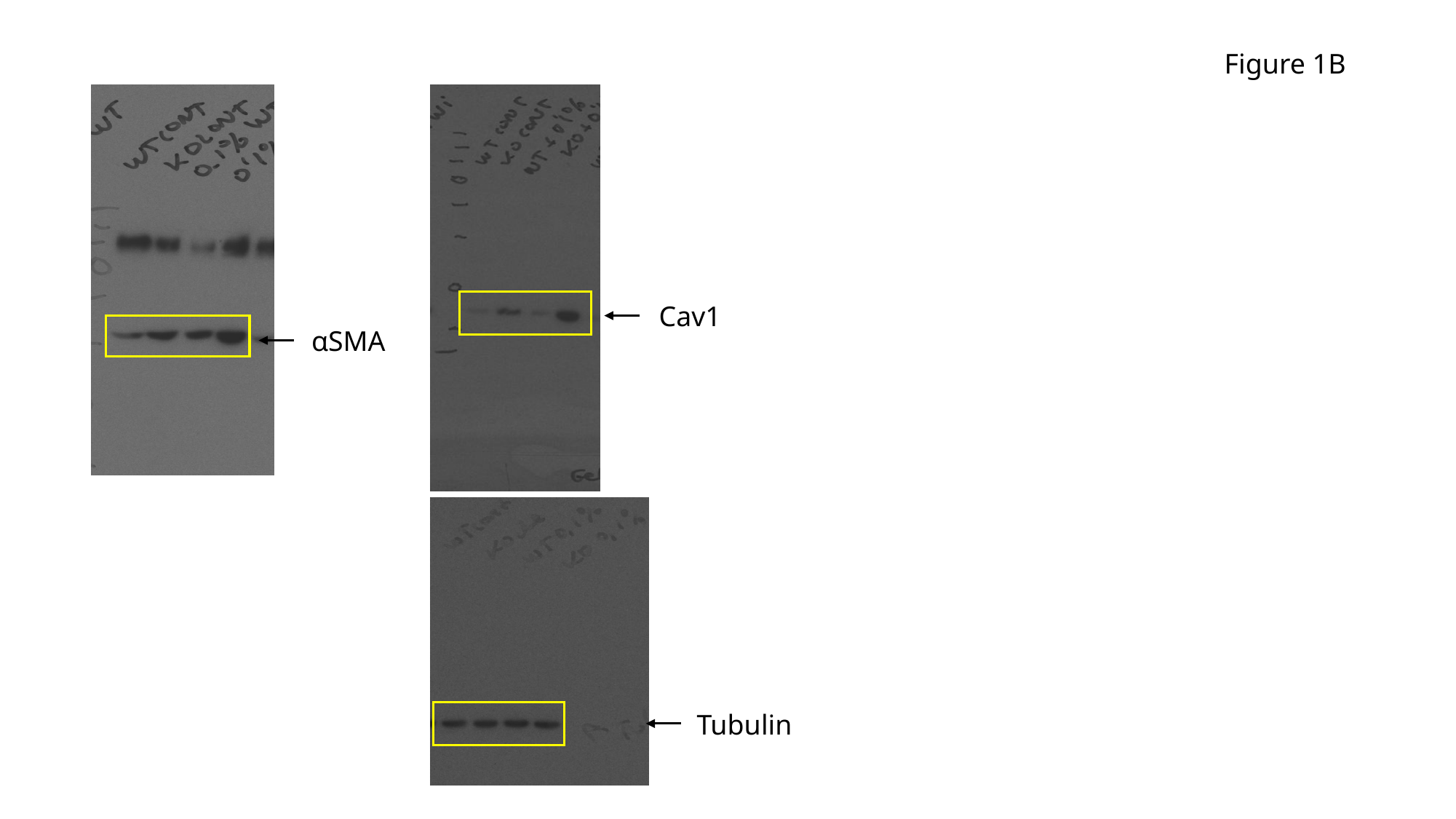

Figure 1B
Cav1
αSMA
Tubulin

Supplement: Supplementary file 5 — Source data Fig. 1 [file 44319_2026_751_MOESM5_ESM.zip › Raw_data_Figure 1/Figure 1B/raw_blots_1B.pptx]

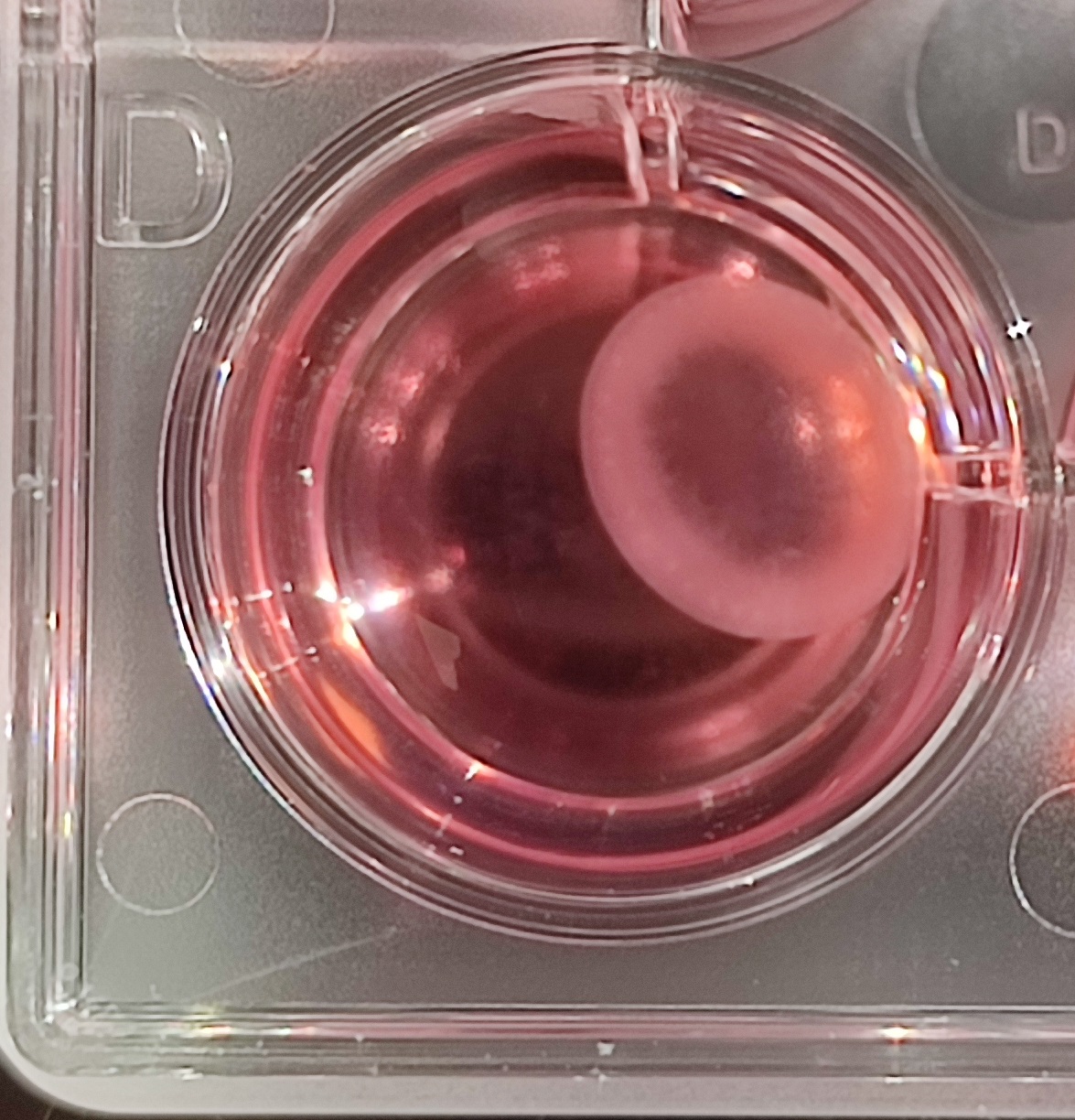

Supplement: Supplementary file 5 — Source data Fig. 1 [file 44319_2026_751_MOESM5_ESM.zip › Raw_data_Figure 1/Figure 1C/KO 1.jpg]

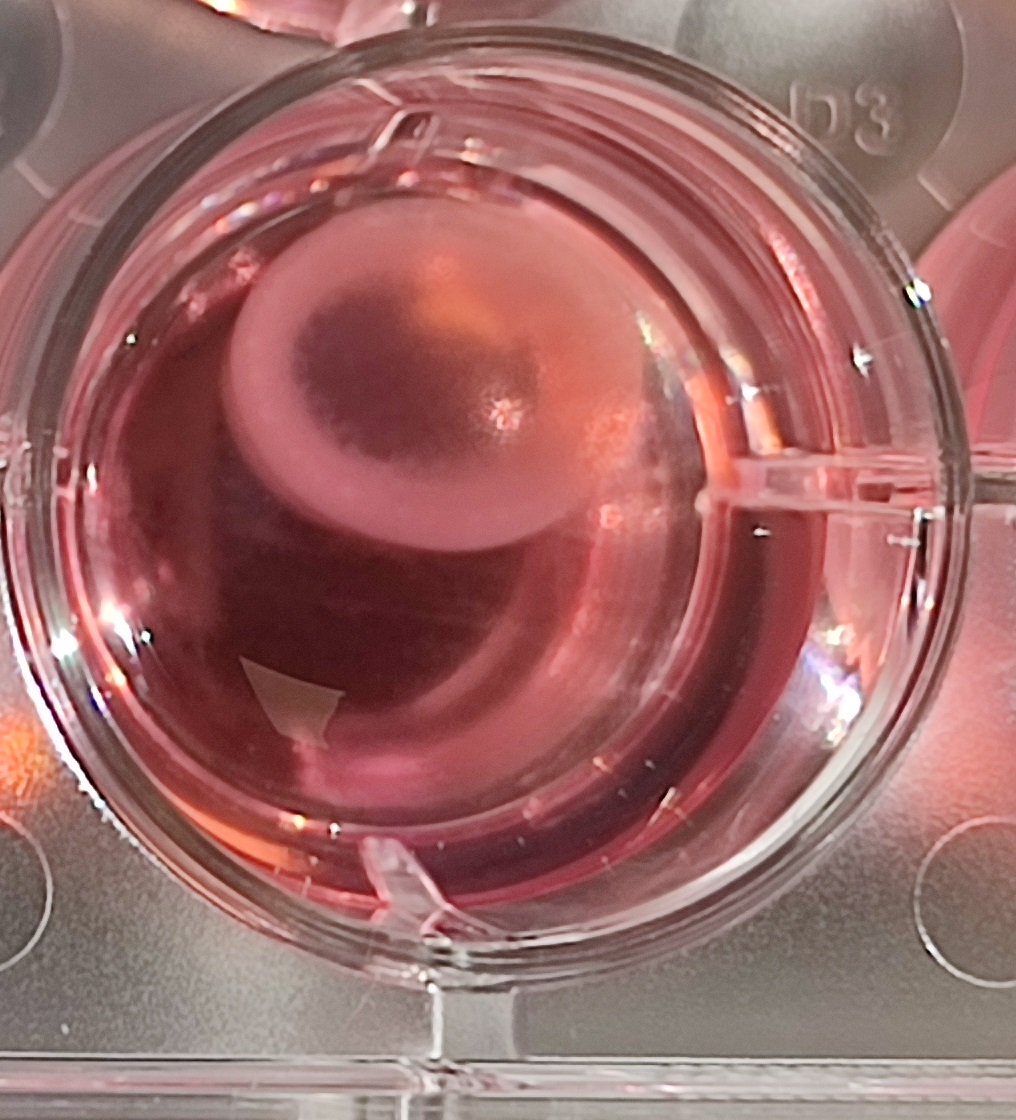

Supplement: Supplementary file 5 — Source data Fig. 1 [file 44319_2026_751_MOESM5_ESM.zip › Raw_data_Figure 1/Figure 1C/KO2.jpg]

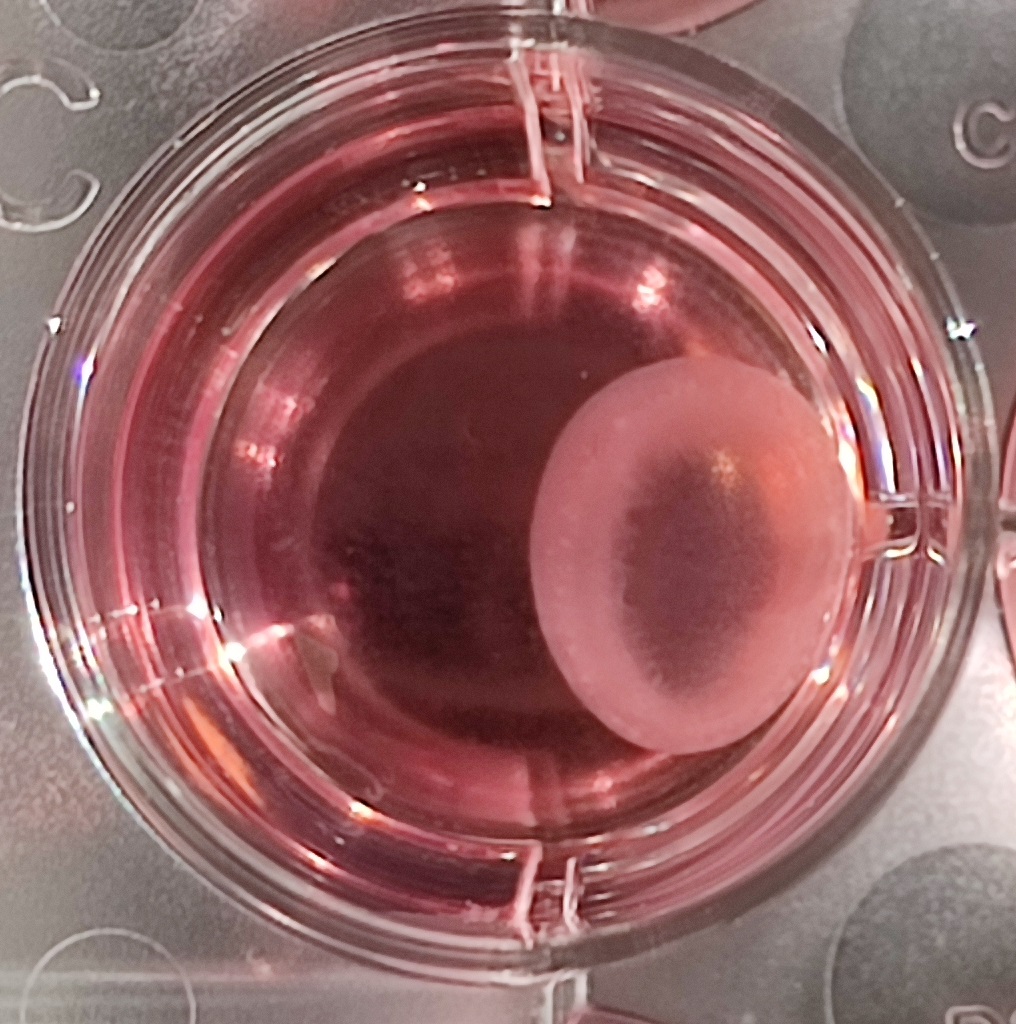

Supplement: Supplementary file 5 — Source data Fig. 1 [file 44319_2026_751_MOESM5_ESM.zip › Raw_data_Figure 1/Figure 1C/KO3.jpg]

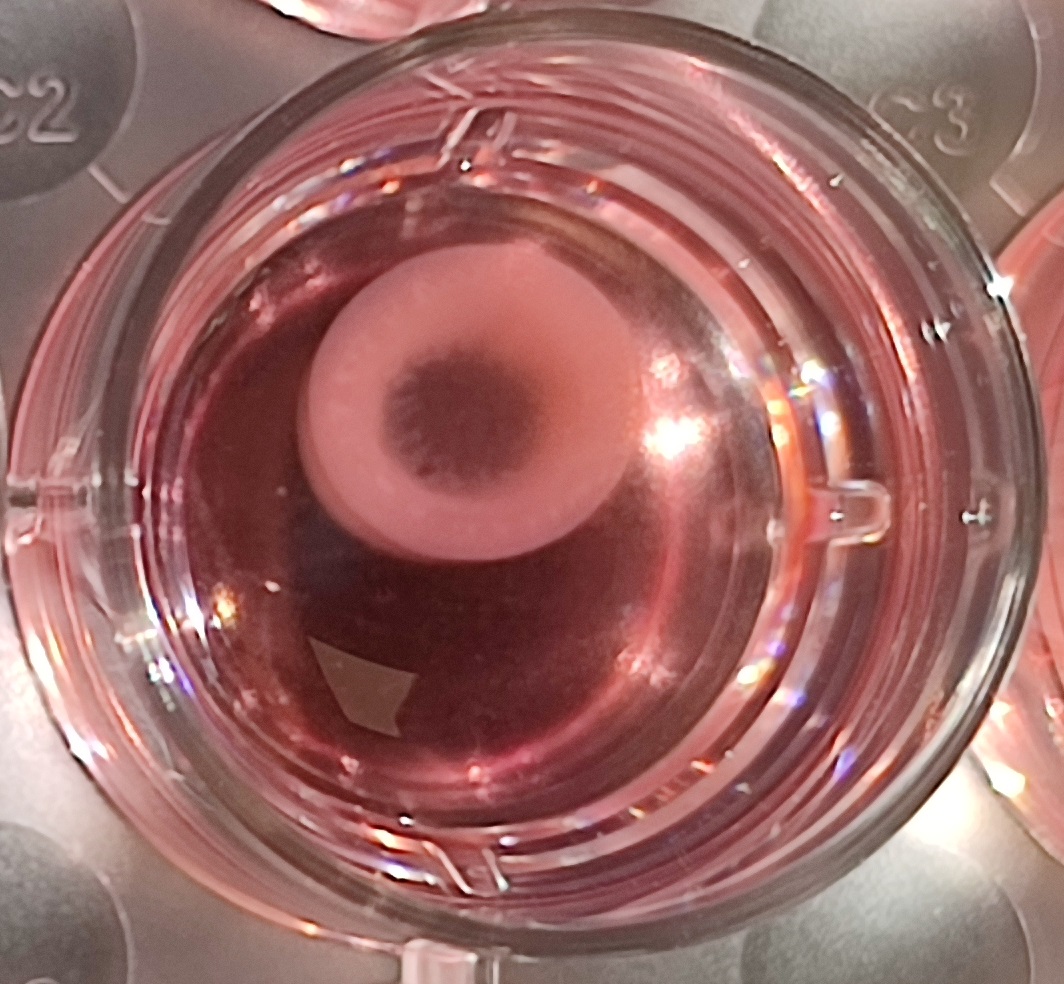

Supplement: Supplementary file 5 — Source data Fig. 1 [file 44319_2026_751_MOESM5_ESM.zip › Raw_data_Figure 1/Figure 1C/KO4.jpg]

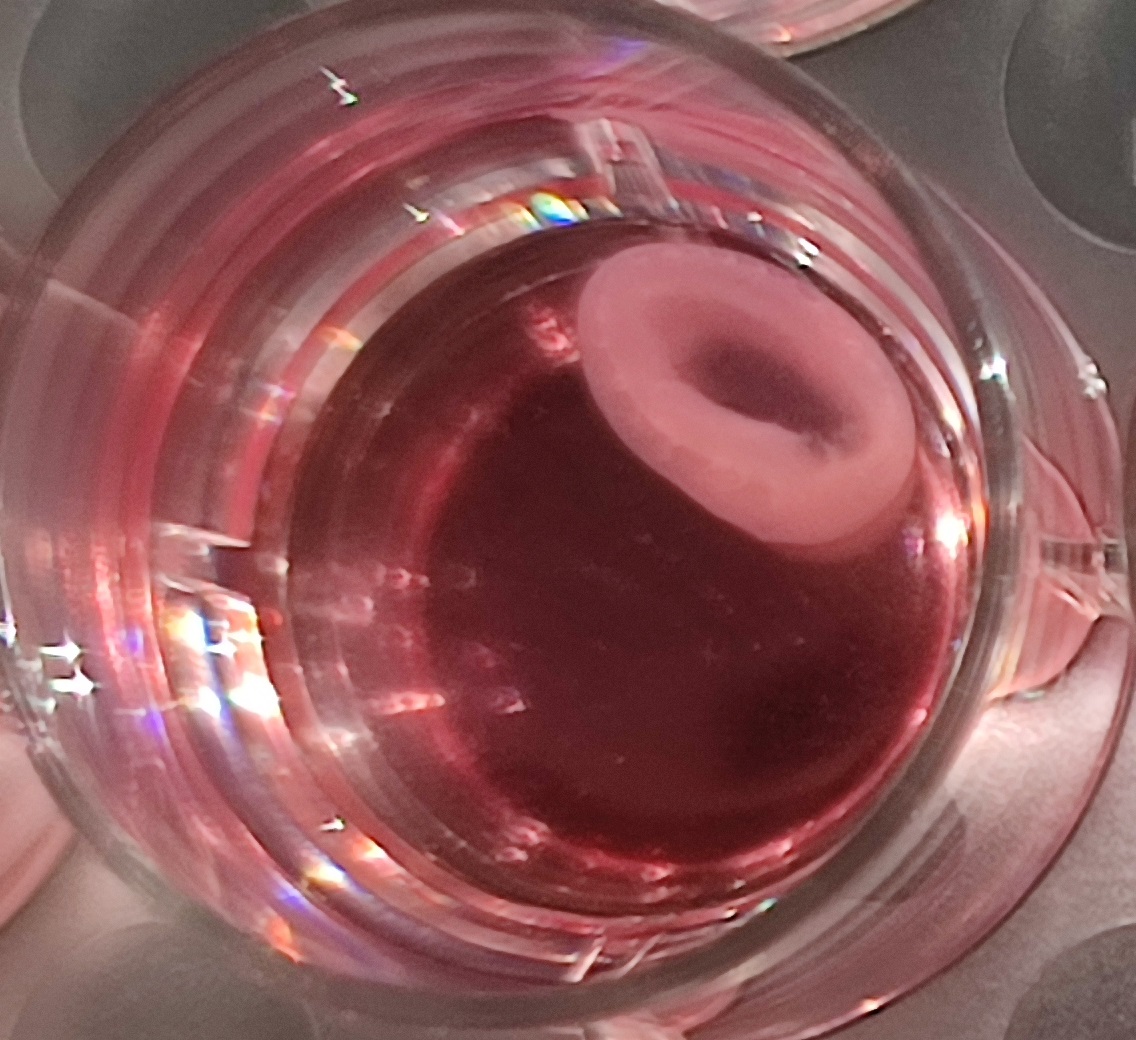

Supplement: Supplementary file 5 — Source data Fig. 1 [file 44319_2026_751_MOESM5_ESM.zip › Raw_data_Figure 1/Figure 1C/KO6.jpg]

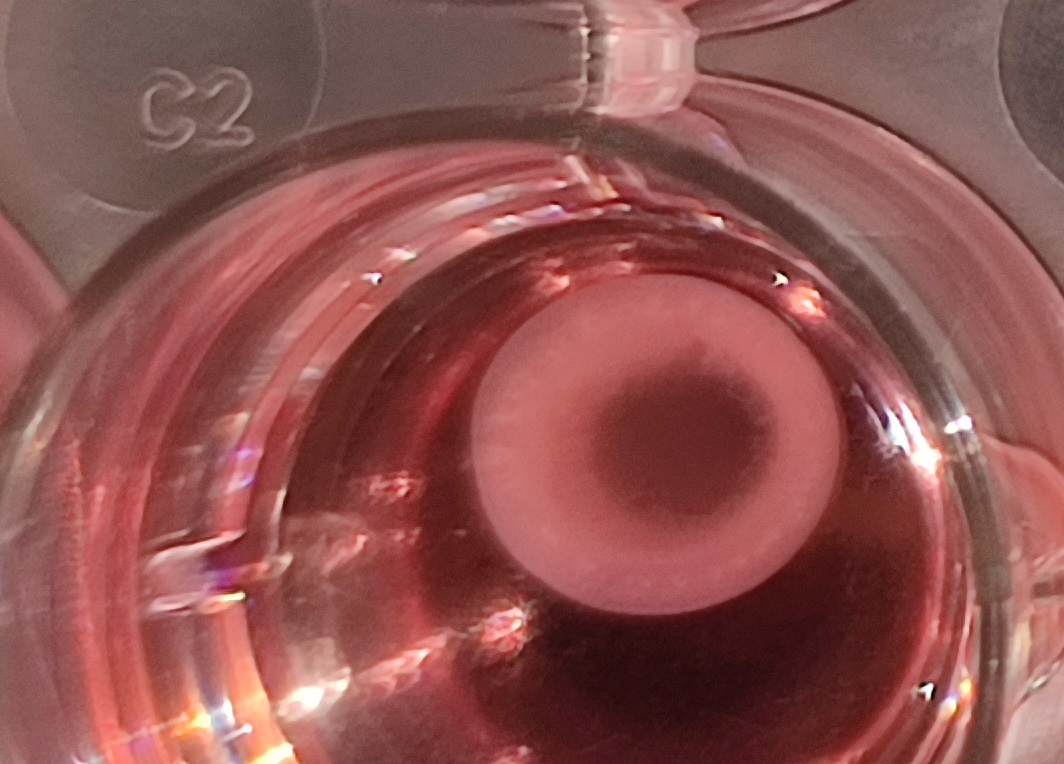

Supplement: Supplementary file 5 — Source data Fig. 1 [file 44319_2026_751_MOESM5_ESM.zip › Raw_data_Figure 1/Figure 1C/KO7.jpg]

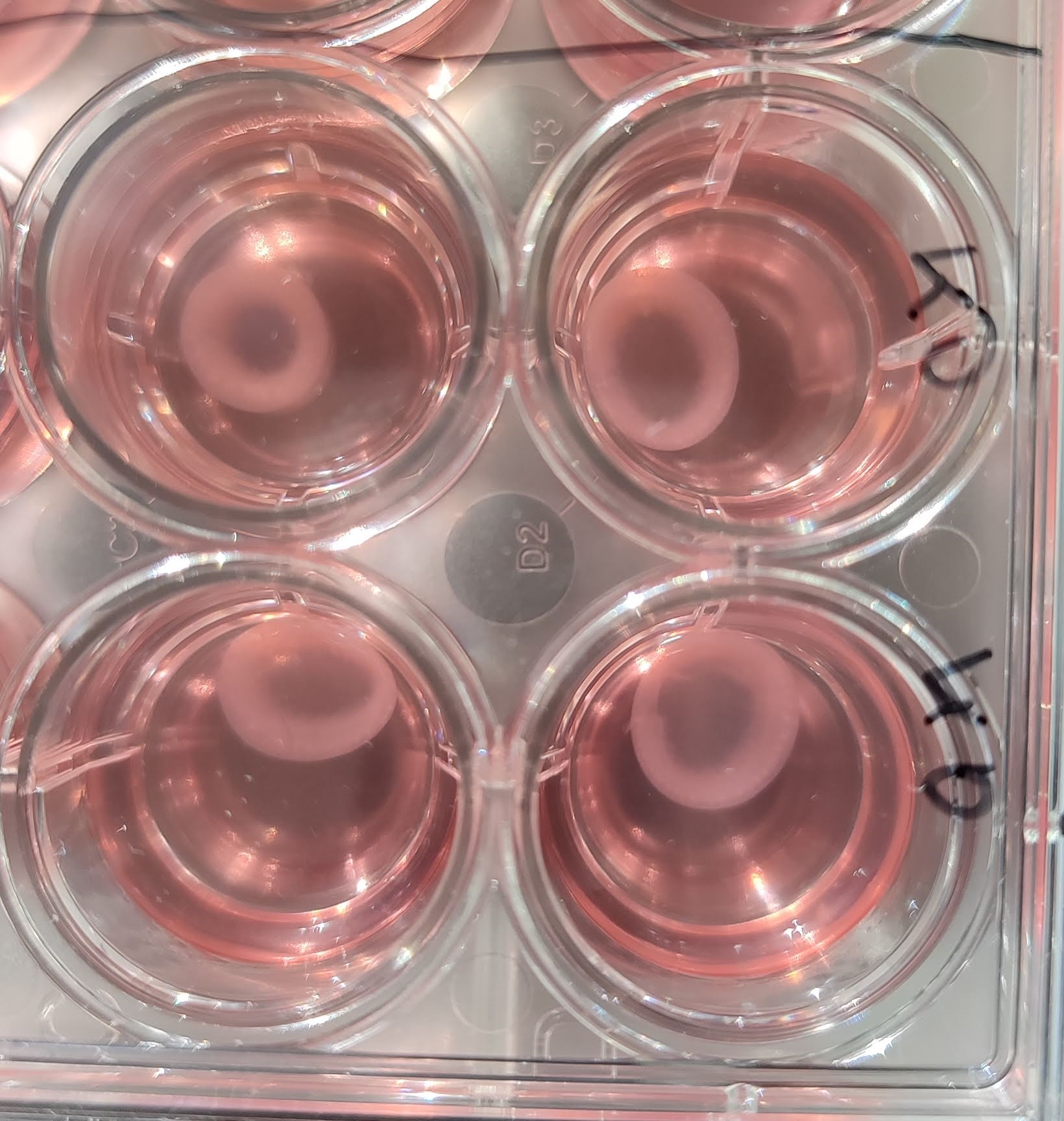

Supplement: Supplementary file 5 — Source data Fig. 1 [file 44319_2026_751_MOESM5_ESM.zip › Raw_data_Figure 1/Figure 1C/KOX4.jpg]

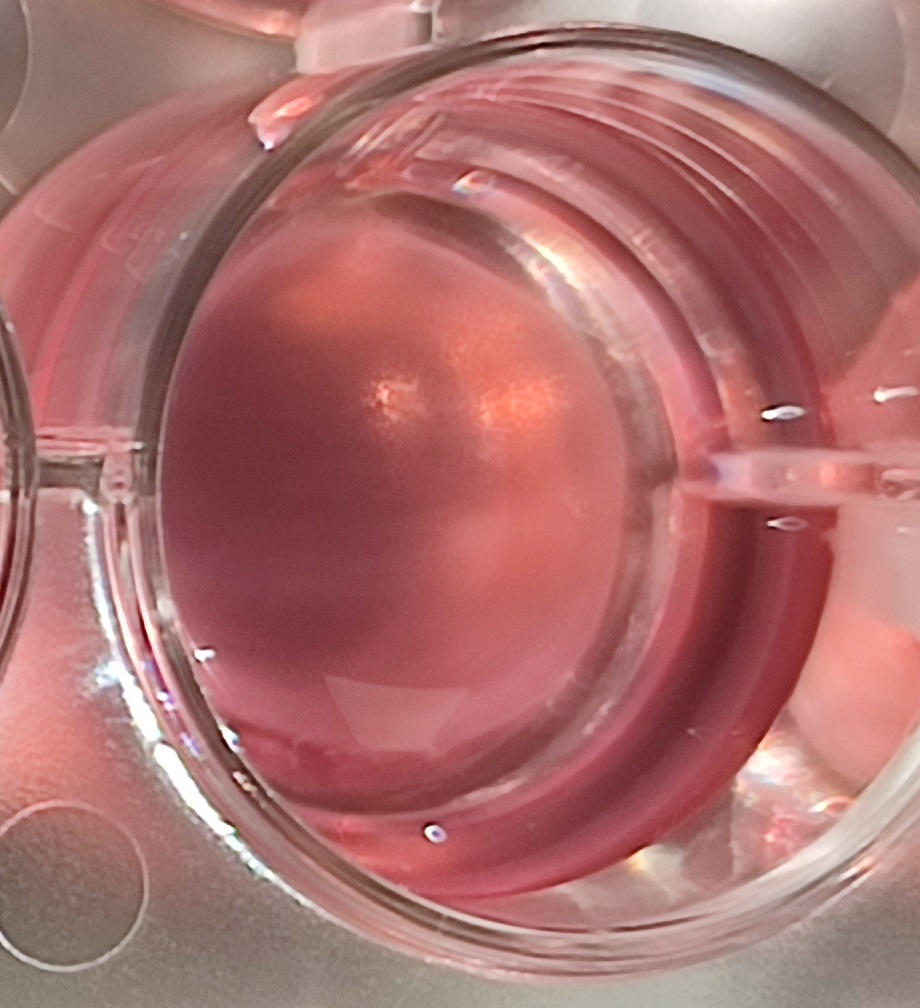

Supplement: Supplementary file 5 — Source data Fig. 1 [file 44319_2026_751_MOESM5_ESM.zip › Raw_data_Figure 1/Figure 1C/WT1.jpg]

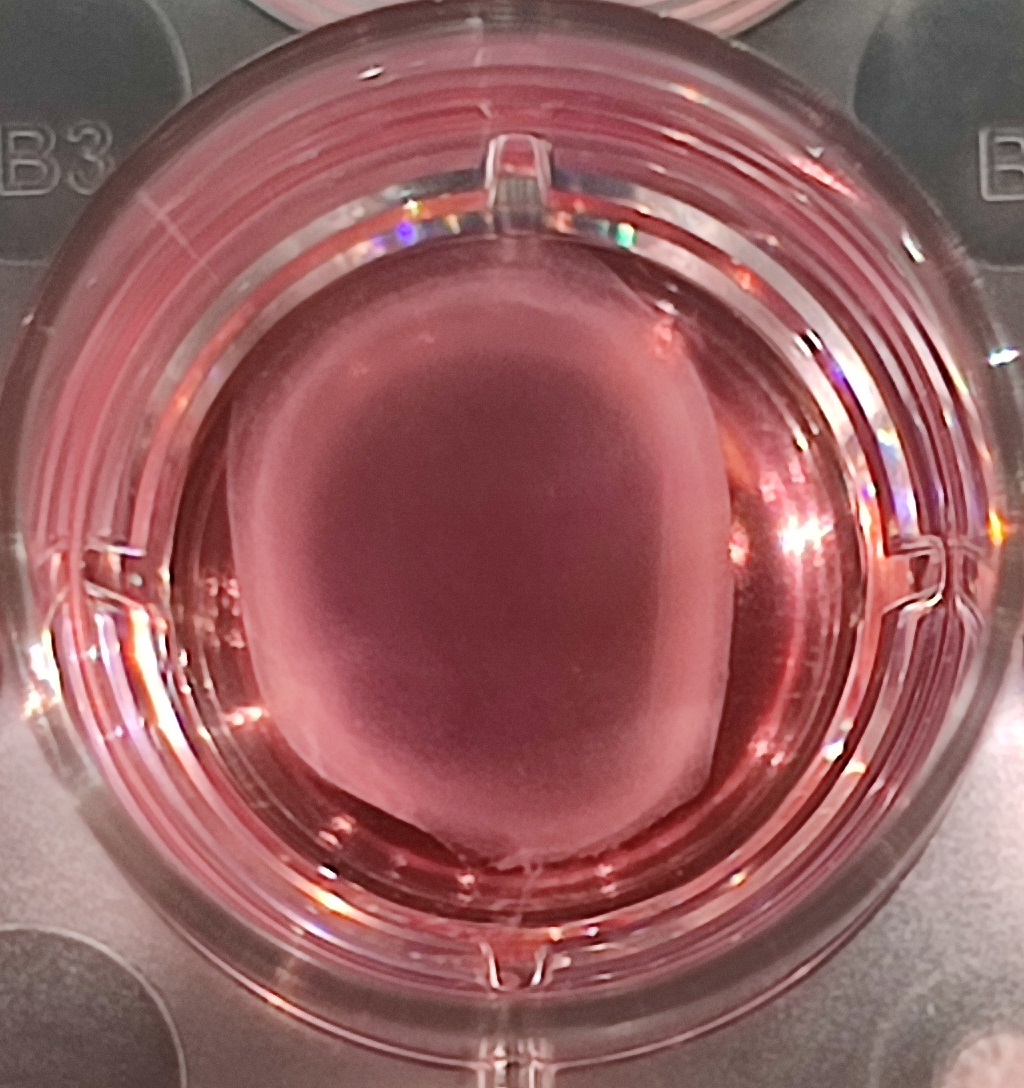

Supplement: Supplementary file 5 — Source data Fig. 1 [file 44319_2026_751_MOESM5_ESM.zip › Raw_data_Figure 1/Figure 1C/WT2.jpg]

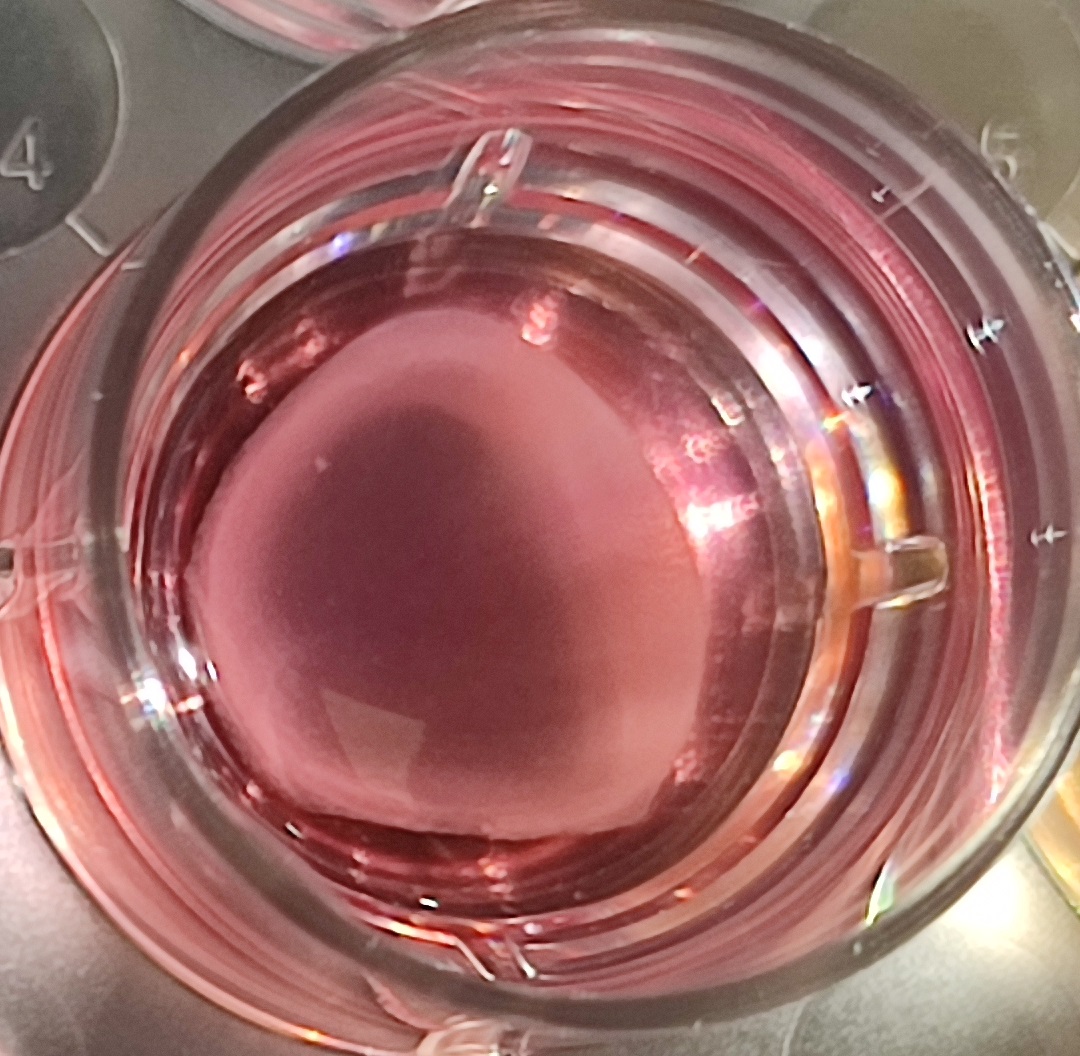

Supplement: Supplementary file 5 — Source data Fig. 1 [file 44319_2026_751_MOESM5_ESM.zip › Raw_data_Figure 1/Figure 1C/WT3.jpg]

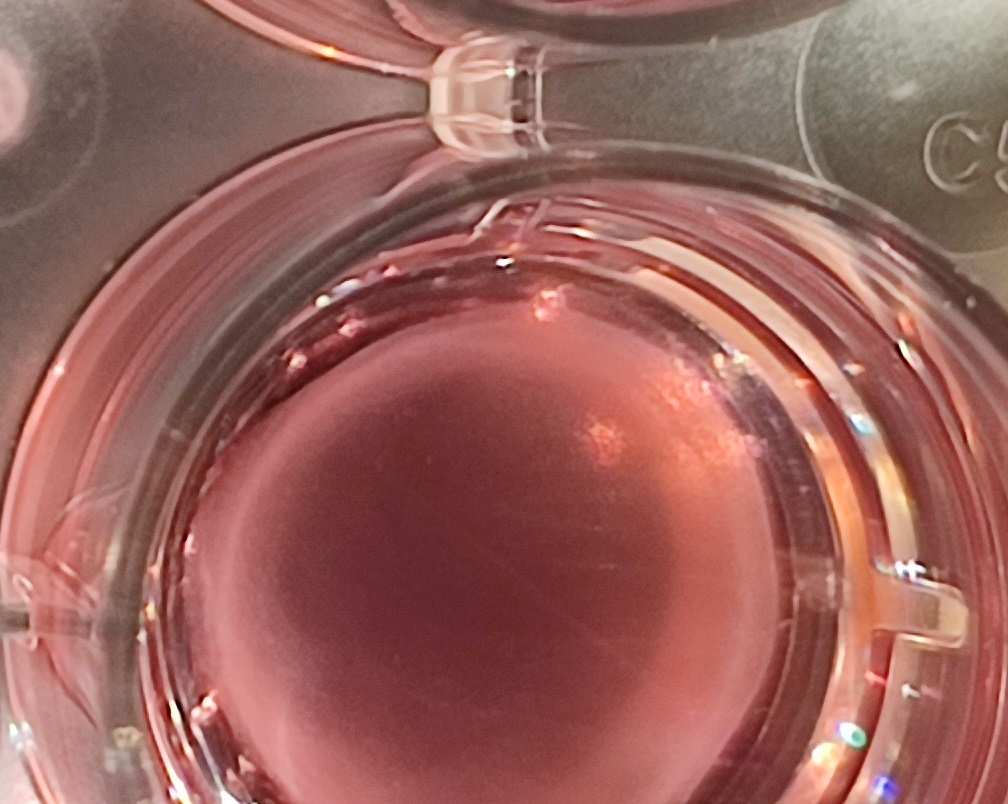

Supplement: Supplementary file 5 — Source data Fig. 1 [file 44319_2026_751_MOESM5_ESM.zip › Raw_data_Figure 1/Figure 1C/WT4.jpg]

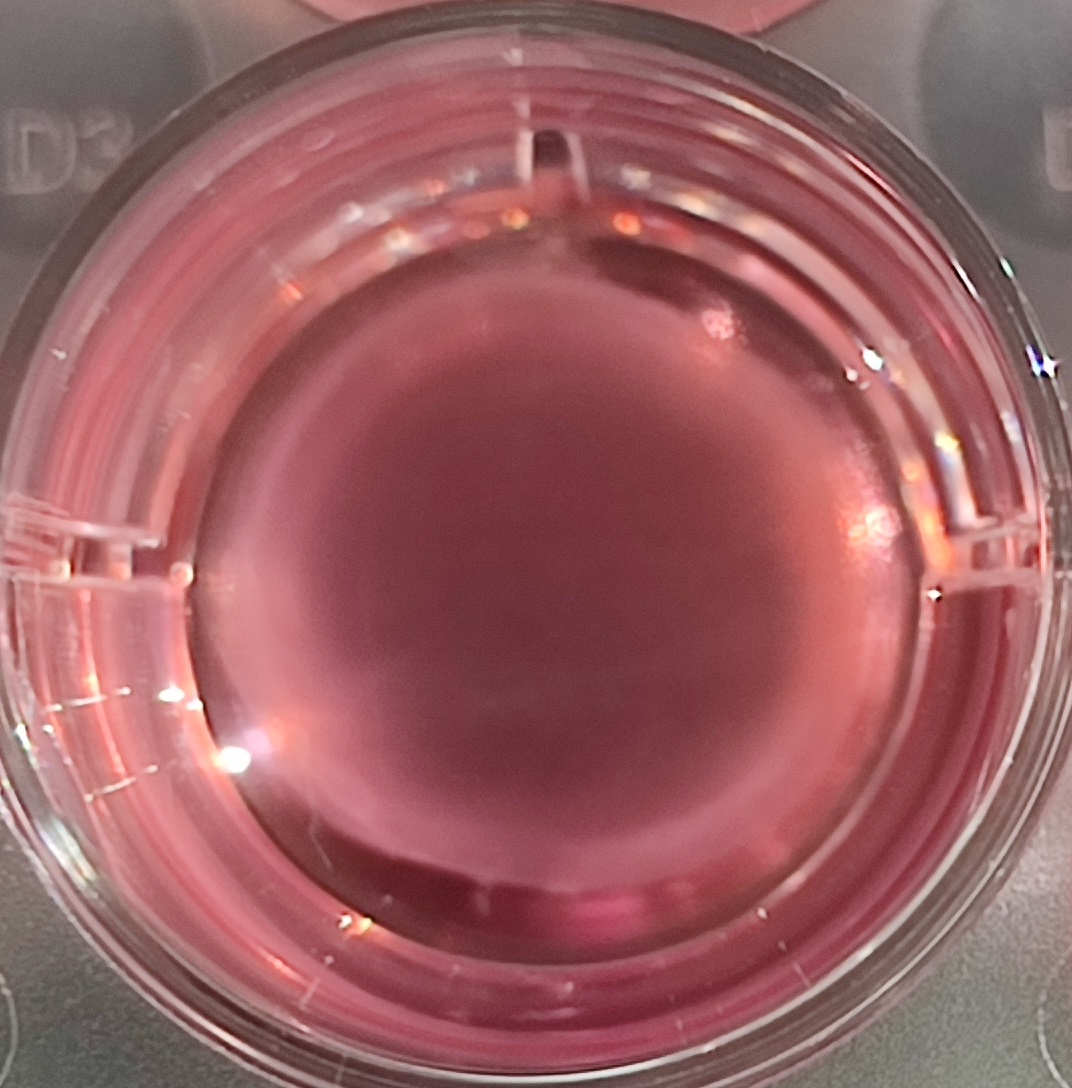

Supplement: Supplementary file 5 — Source data Fig. 1 [file 44319_2026_751_MOESM5_ESM.zip › Raw_data_Figure 1/Figure 1C/WT5.jpg]

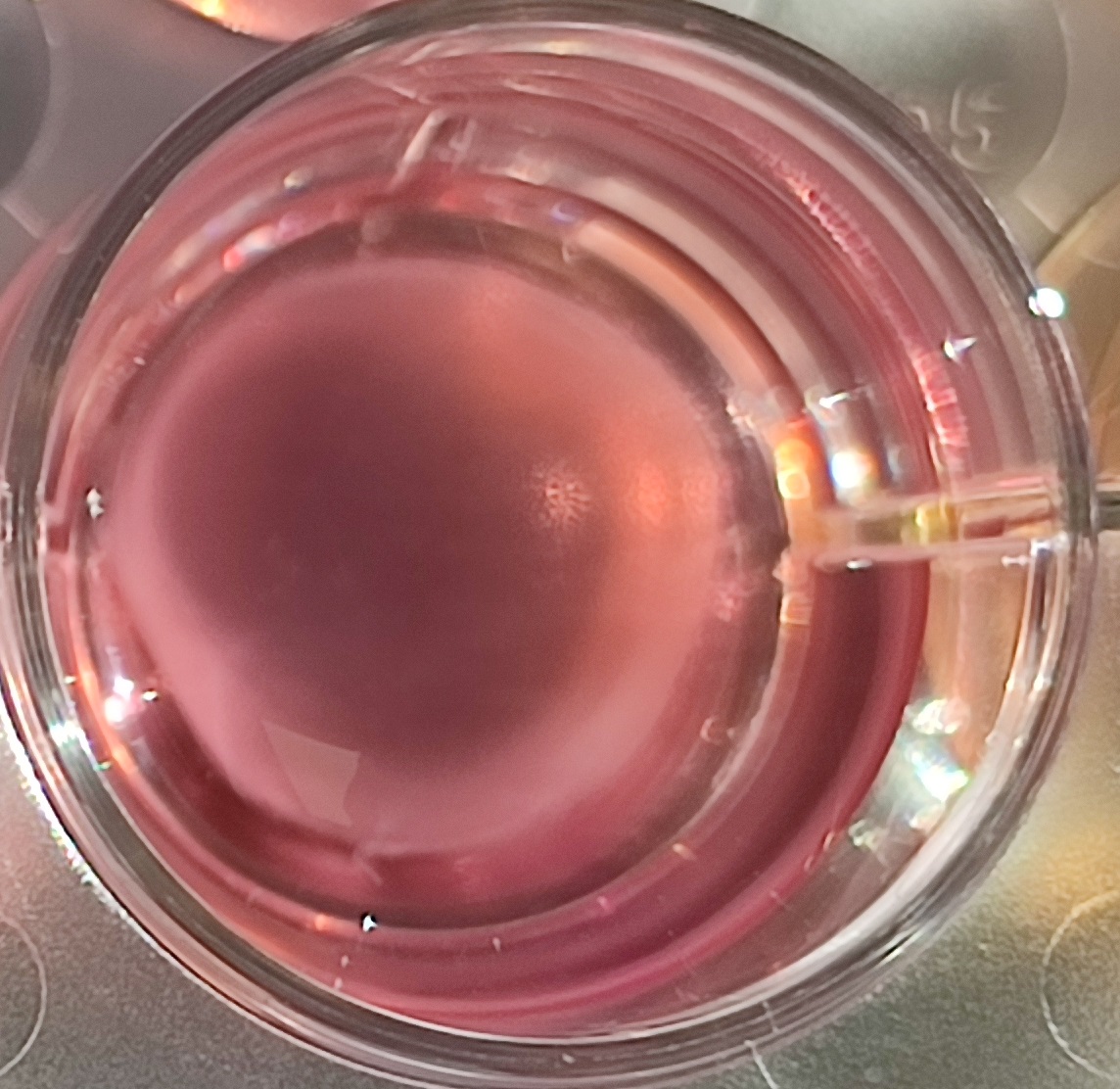

Supplement: Supplementary file 5 — Source data Fig. 1 [file 44319_2026_751_MOESM5_ESM.zip › Raw_data_Figure 1/Figure 1C/WT6.jpg]

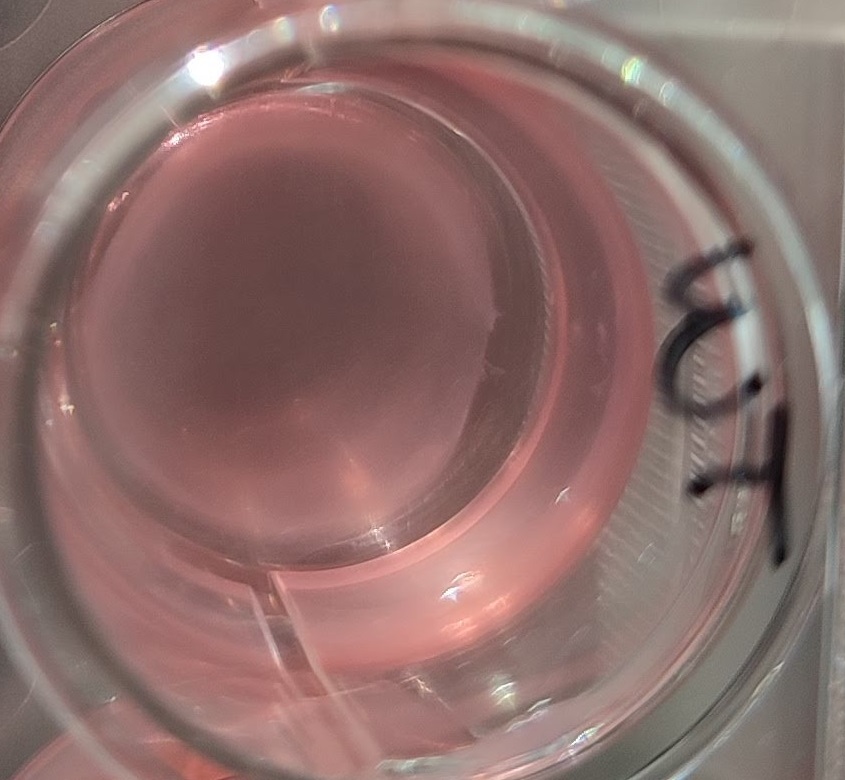

Supplement: Supplementary file 5 — Source data Fig. 1 [file 44319_2026_751_MOESM5_ESM.zip › Raw_data_Figure 1/Figure 1C/WT7.jpg]

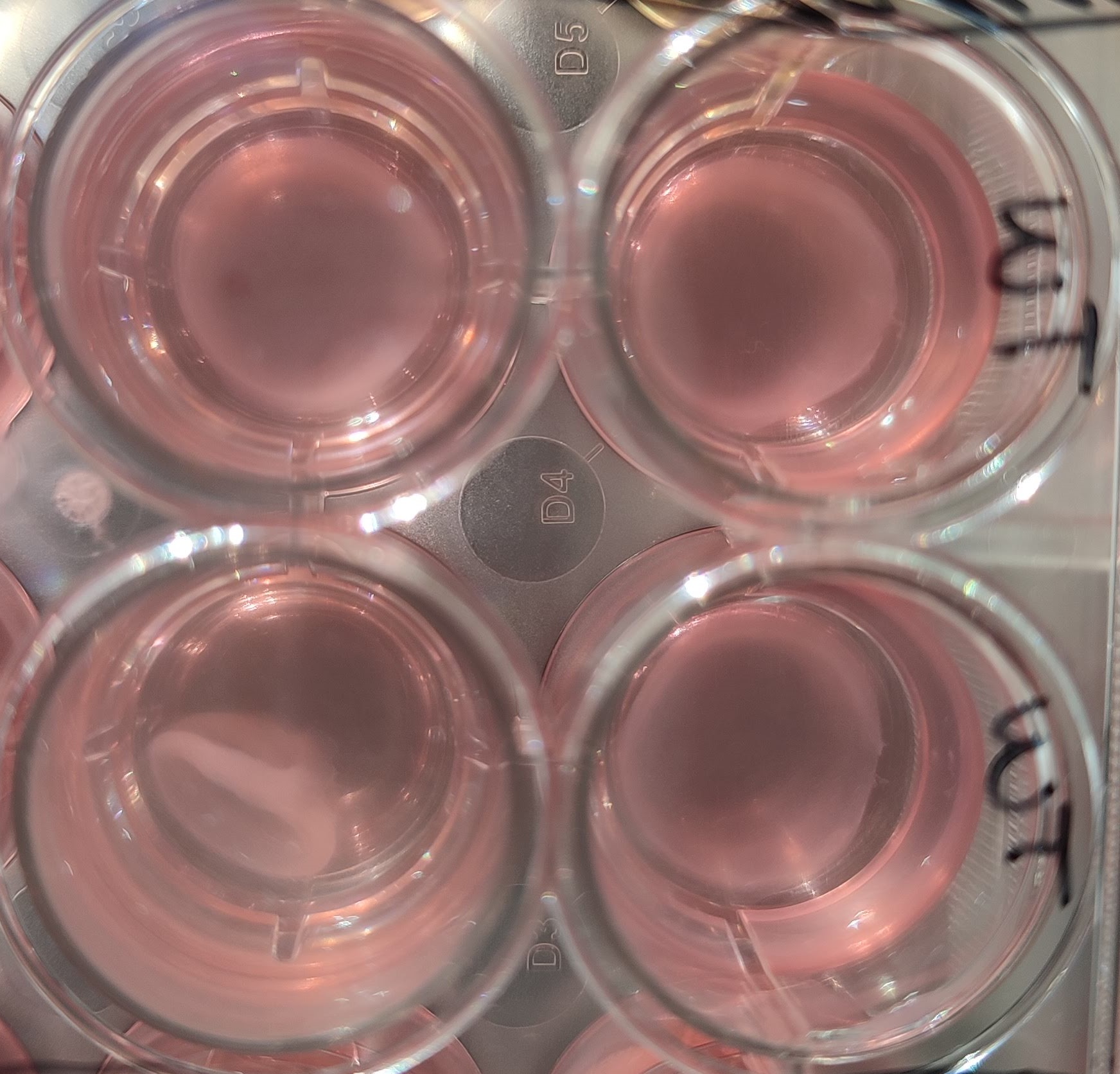

Supplement: Supplementary file 5 — Source data Fig. 1 [file 44319_2026_751_MOESM5_ESM.zip › Raw_data_Figure 1/Figure 1C/WTX4.jpg]

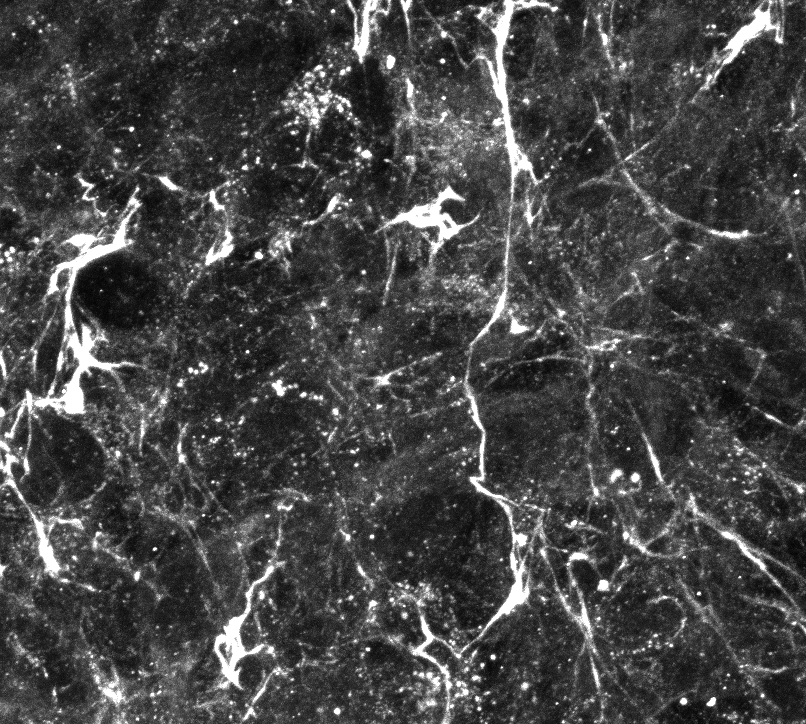

Supplement: Supplementary file 5 — Source data Fig. 1 [file 44319_2026_751_MOESM5_ESM.zip › Raw_data_Figure 1/Figure 1D/Confocal microscopy Col I/GqKO Col I selected.tif]

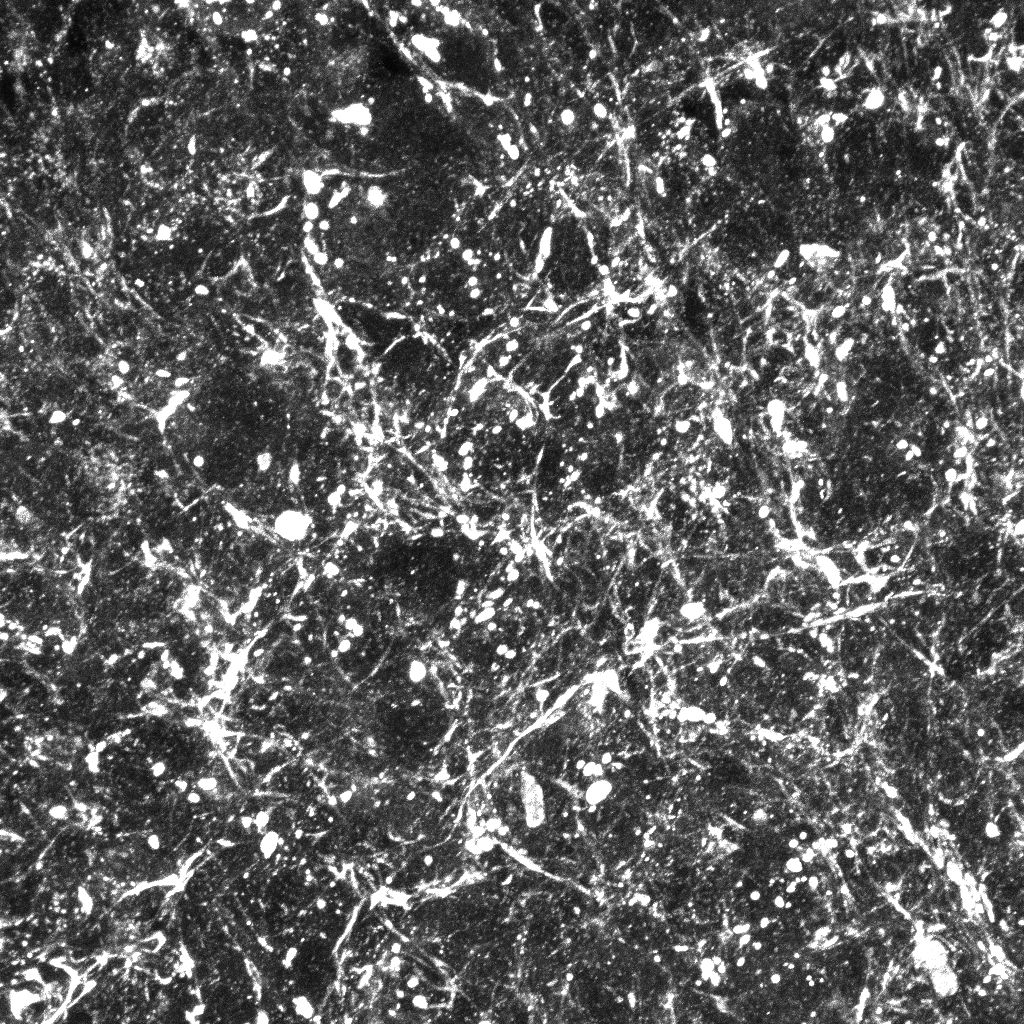

Supplement: Supplementary file 5 — Source data Fig. 1 [file 44319_2026_751_MOESM5_ESM.zip › Raw_data_Figure 1/Figure 1D/Confocal microscopy Col I/GqKO Col I _2.tif]

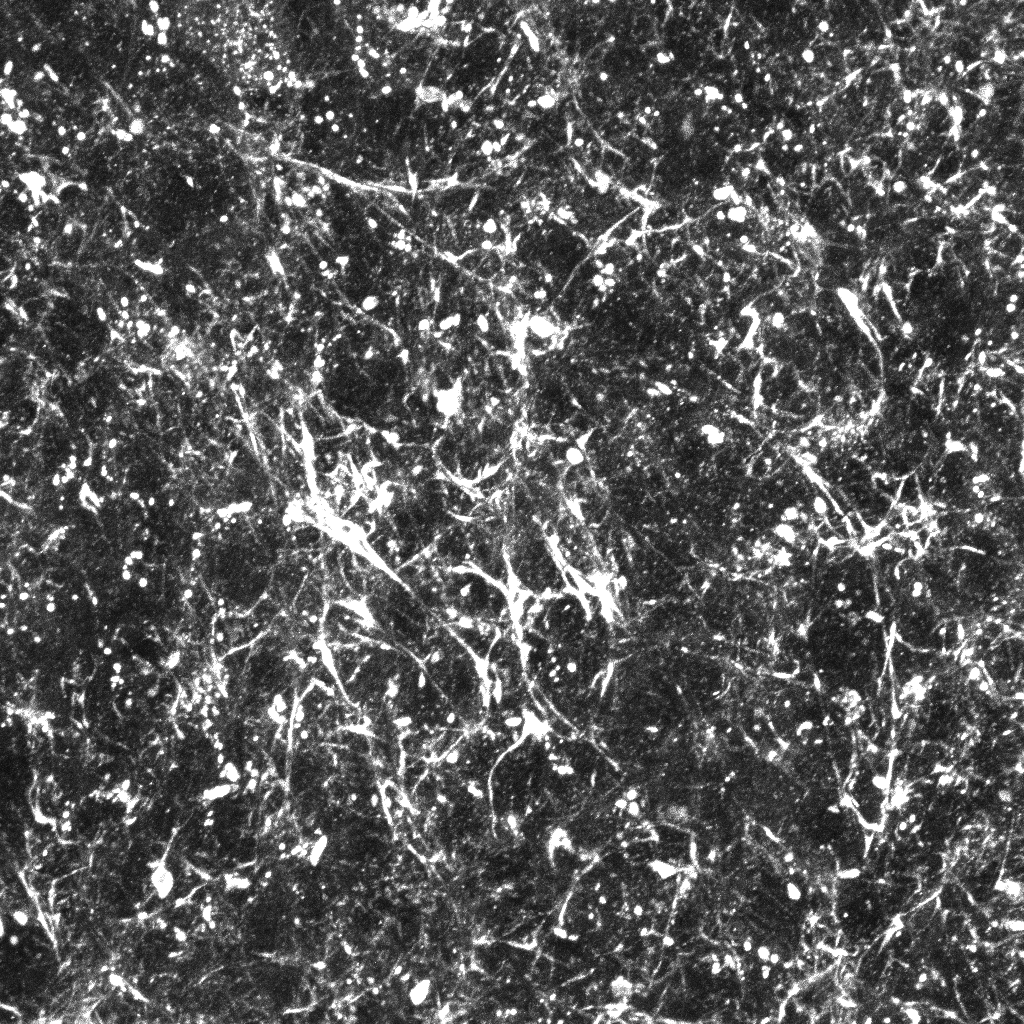

Supplement: Supplementary file 5 — Source data Fig. 1 [file 44319_2026_751_MOESM5_ESM.zip › Raw_data_Figure 1/Figure 1D/Confocal microscopy Col I/GqKO Col I.tif]

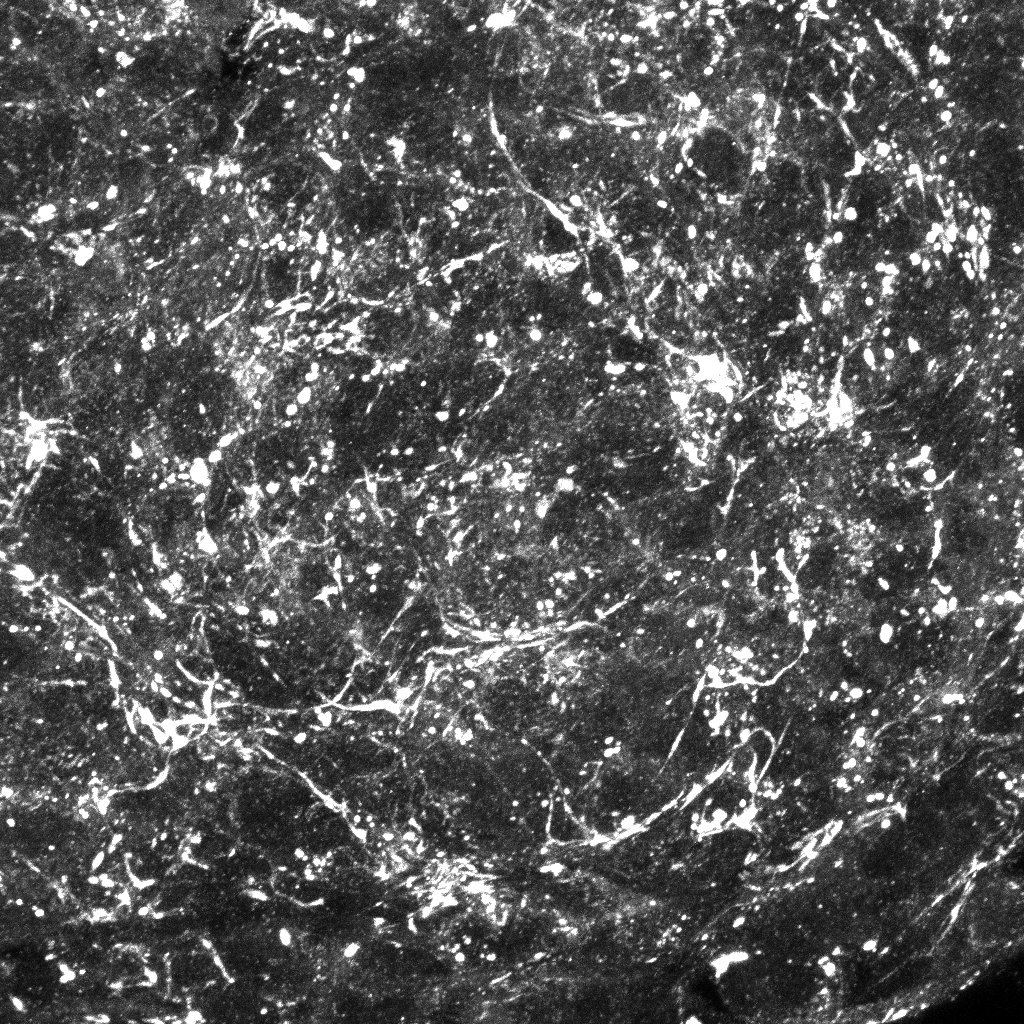

Supplement: Supplementary file 5 — Source data Fig. 1 [file 44319_2026_751_MOESM5_ESM.zip › Raw_data_Figure 1/Figure 1D/Confocal microscopy Col I/GqKO Col I_ 3.tif]

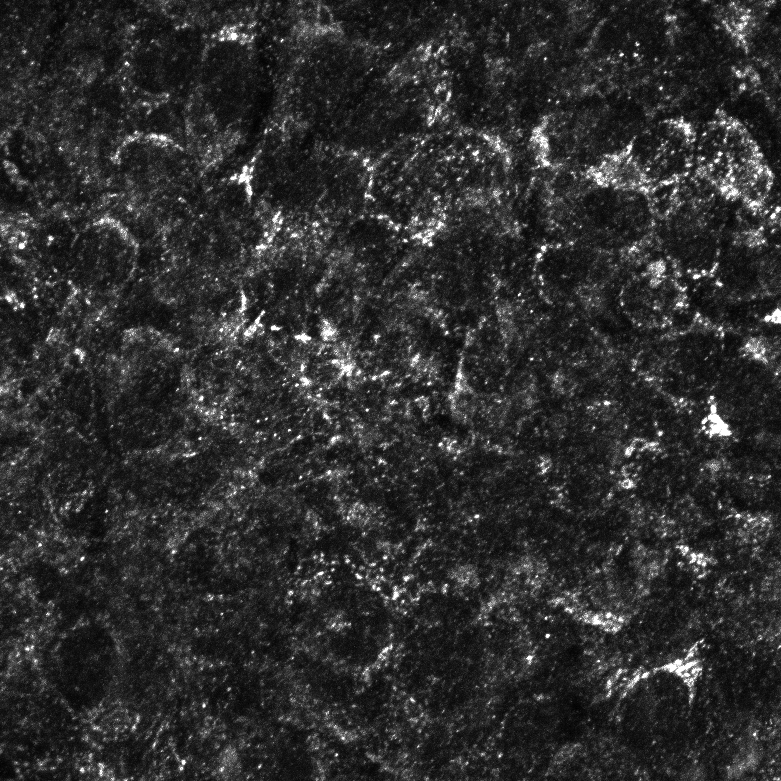

Supplement: Supplementary file 5 — Source data Fig. 1 [file 44319_2026_751_MOESM5_ESM.zip › Raw_data_Figure 1/Figure 1D/Confocal microscopy Col I/WT Col I selected.tif]

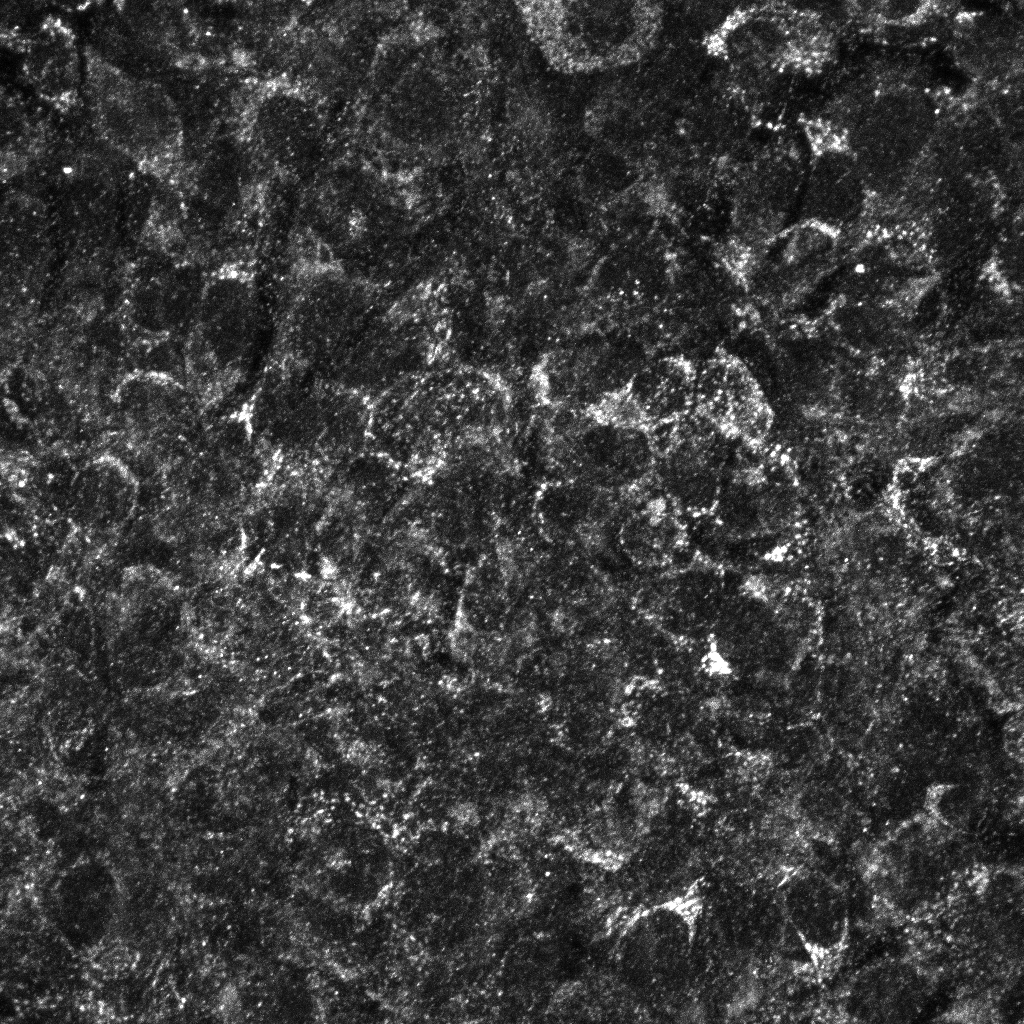

Supplement: Supplementary file 5 — Source data Fig. 1 [file 44319_2026_751_MOESM5_ESM.zip › Raw_data_Figure 1/Figure 1D/Confocal microscopy Col I/WT Col I.tif]

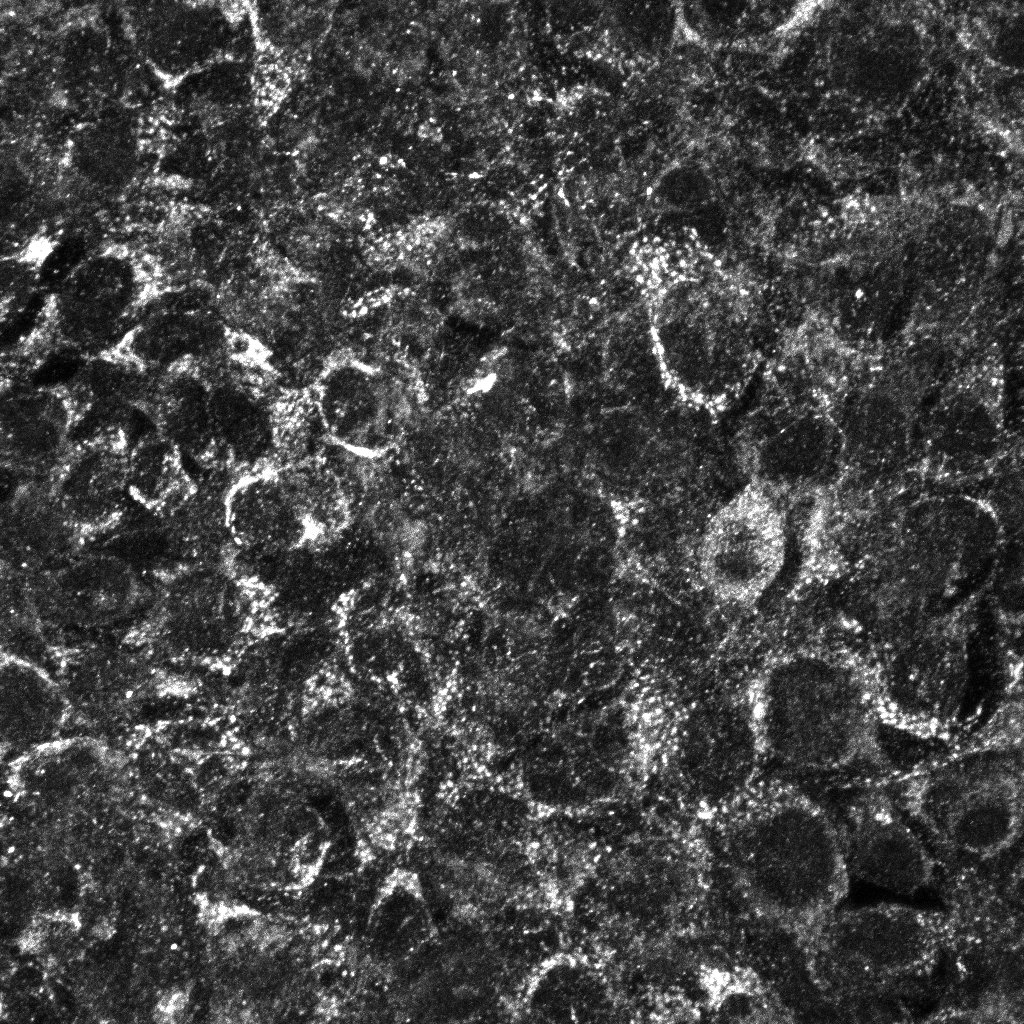

Supplement: Supplementary file 5 — Source data Fig. 1 [file 44319_2026_751_MOESM5_ESM.zip › Raw_data_Figure 1/Figure 1D/Confocal microscopy Col I/WT Col I_ 2.tif]

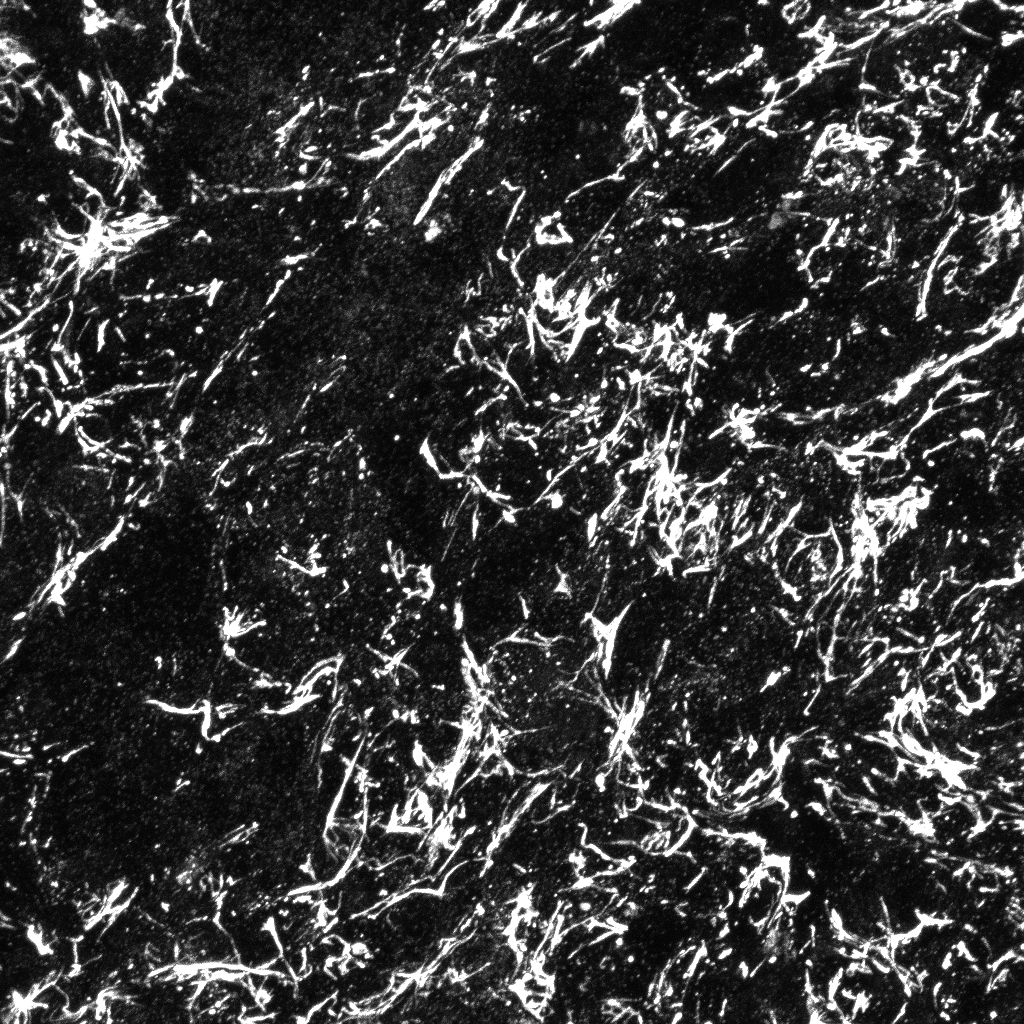

Supplement: Supplementary file 5 — Source data Fig. 1 [file 44319_2026_751_MOESM5_ESM.zip › Raw_data_Figure 1/Figure 1D/Confocal microscopy FN/GqKO selected.tif]

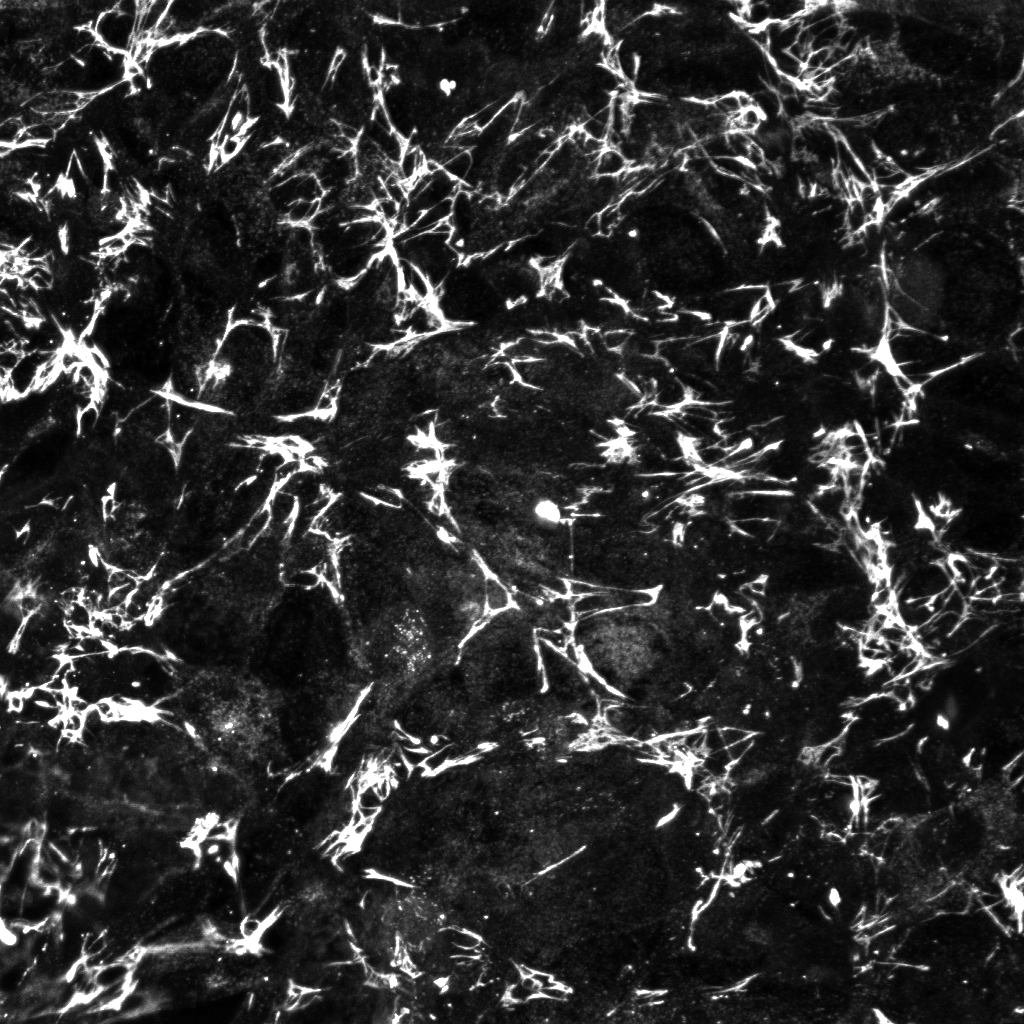

Supplement: Supplementary file 5 — Source data Fig. 1 [file 44319_2026_751_MOESM5_ESM.zip › Raw_data_Figure 1/Figure 1D/Confocal microscopy FN/GqKOMEFsFN_10Maximumintensityprojection.tif]

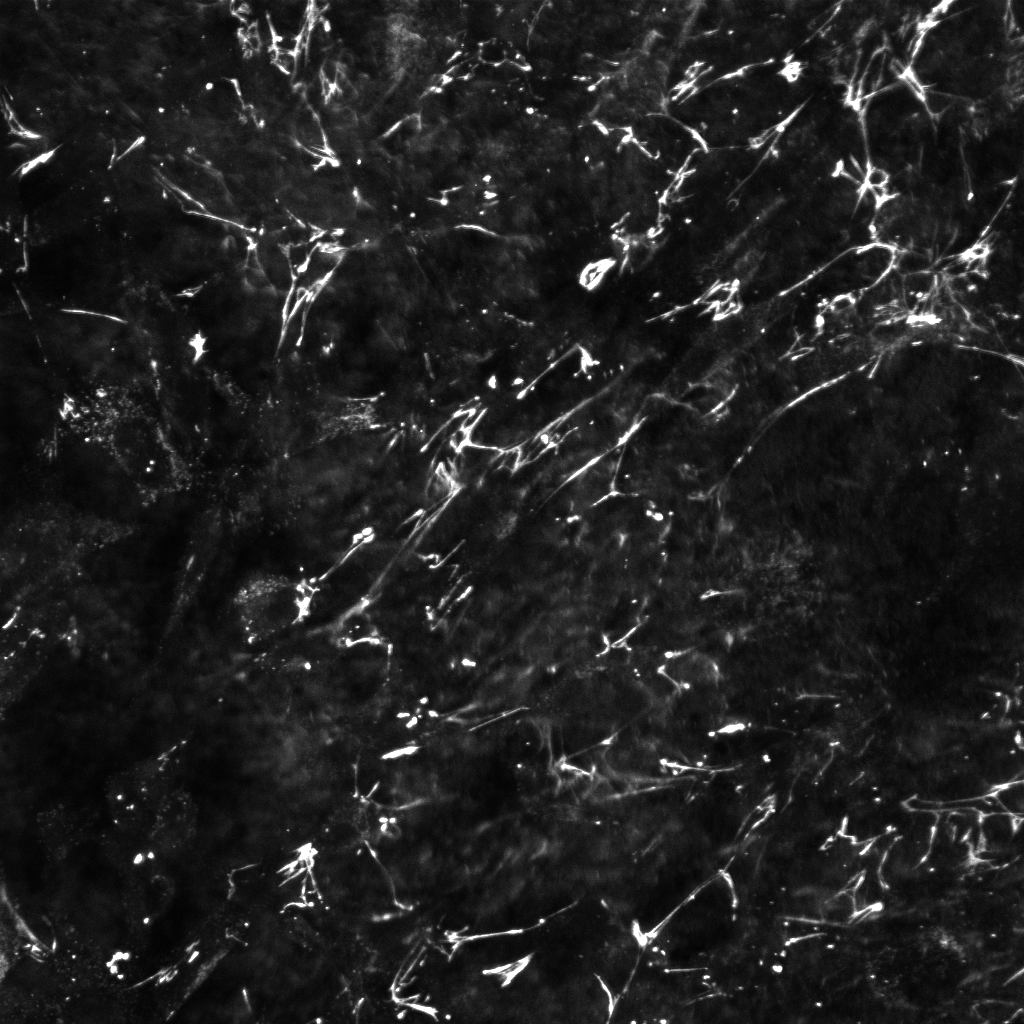

Supplement: Supplementary file 5 — Source data Fig. 1 [file 44319_2026_751_MOESM5_ESM.zip › Raw_data_Figure 1/Figure 1D/Confocal microscopy FN/GqKOMEFsFN_11Maximumintensityprojection.tif]

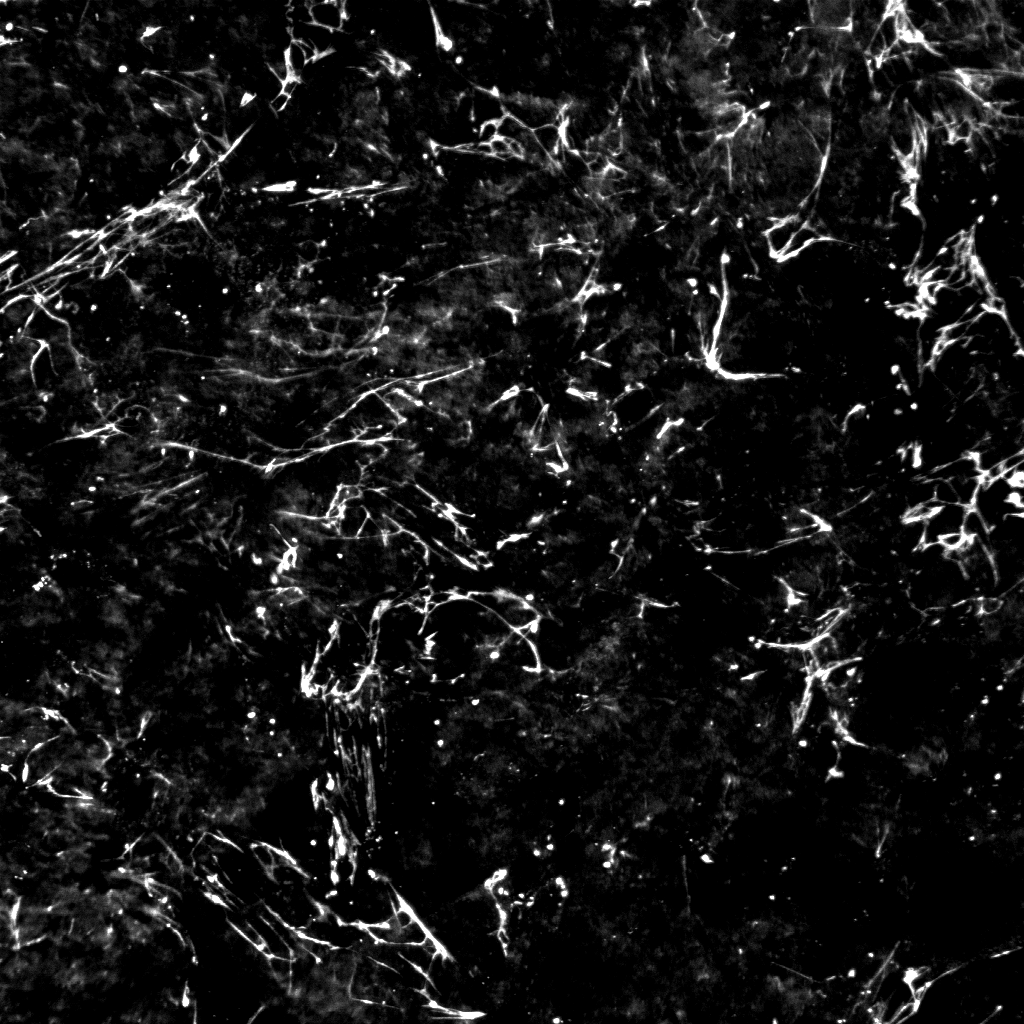

Supplement: Supplementary file 5 — Source data Fig. 1 [file 44319_2026_751_MOESM5_ESM.zip › Raw_data_Figure 1/Figure 1D/Confocal microscopy FN/GqKOMEFsFN_12Maximumintensityprojection.tif]

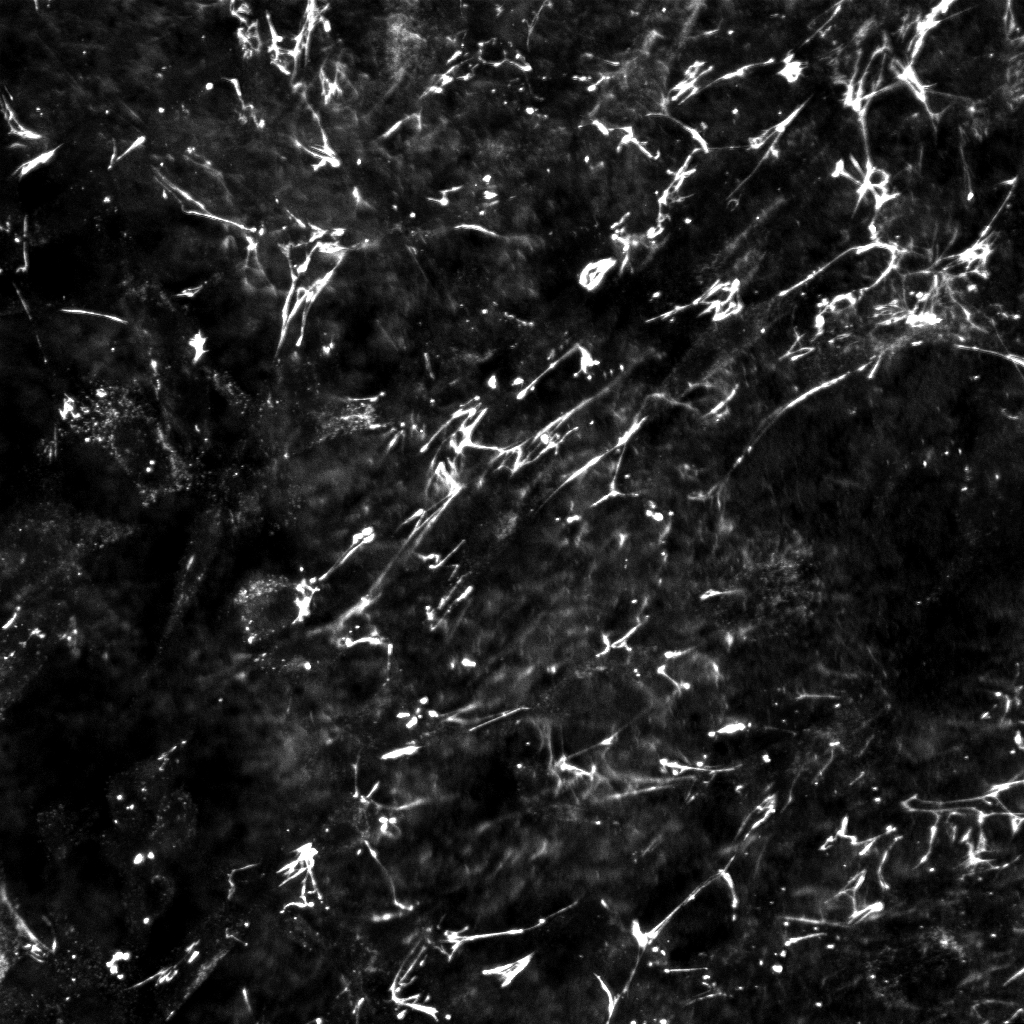

Supplement: Supplementary file 5 — Source data Fig. 1 [file 44319_2026_751_MOESM5_ESM.zip › Raw_data_Figure 1/Figure 1D/Confocal microscopy FN/GqKOMEFsFN_13Maximumintensityprojection.tif]

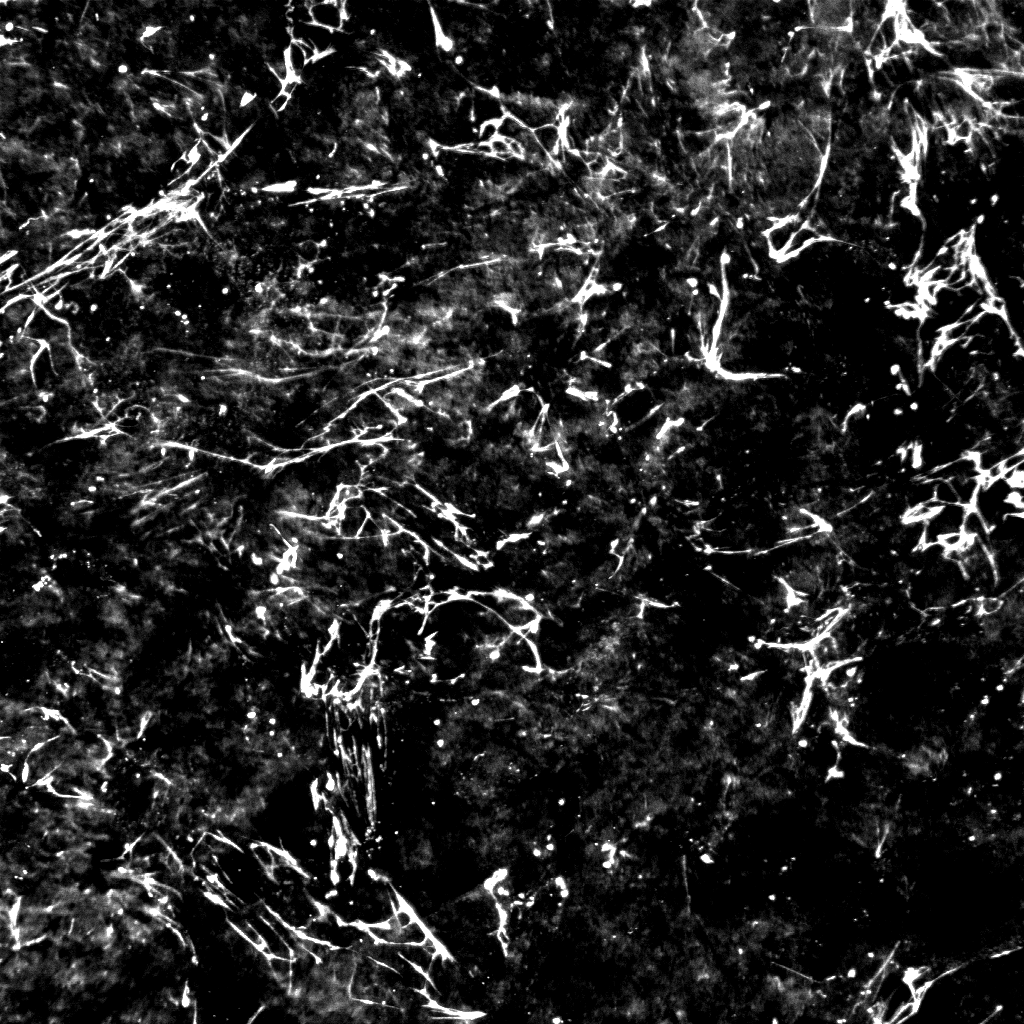

Supplement: Supplementary file 5 — Source data Fig. 1 [file 44319_2026_751_MOESM5_ESM.zip › Raw_data_Figure 1/Figure 1D/Confocal microscopy FN/GqKOMEFsFN_14Maximumintensityprojection.tif]

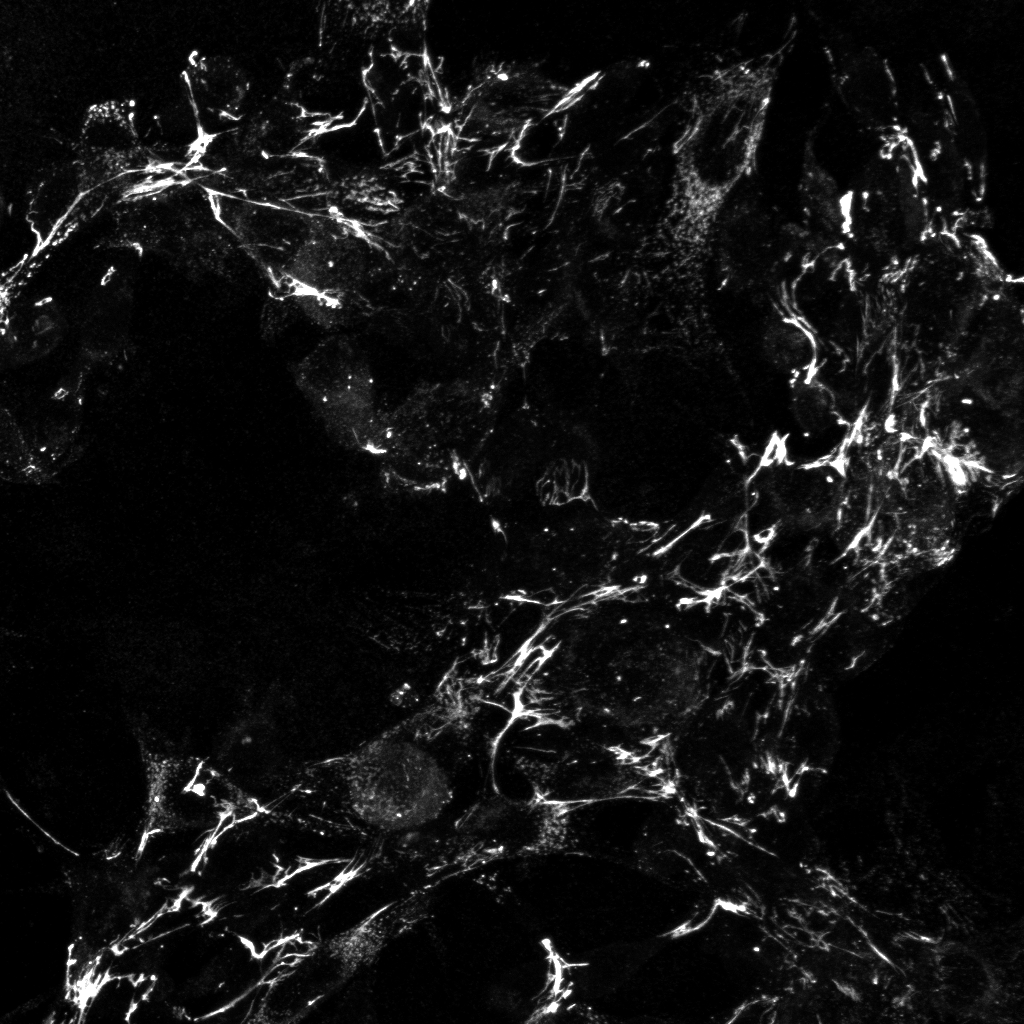

Supplement: Supplementary file 5 — Source data Fig. 1 [file 44319_2026_751_MOESM5_ESM.zip › Raw_data_Figure 1/Figure 1D/Confocal microscopy FN/GqKOMEFsFN_2Maximumintensityprojection.tif]

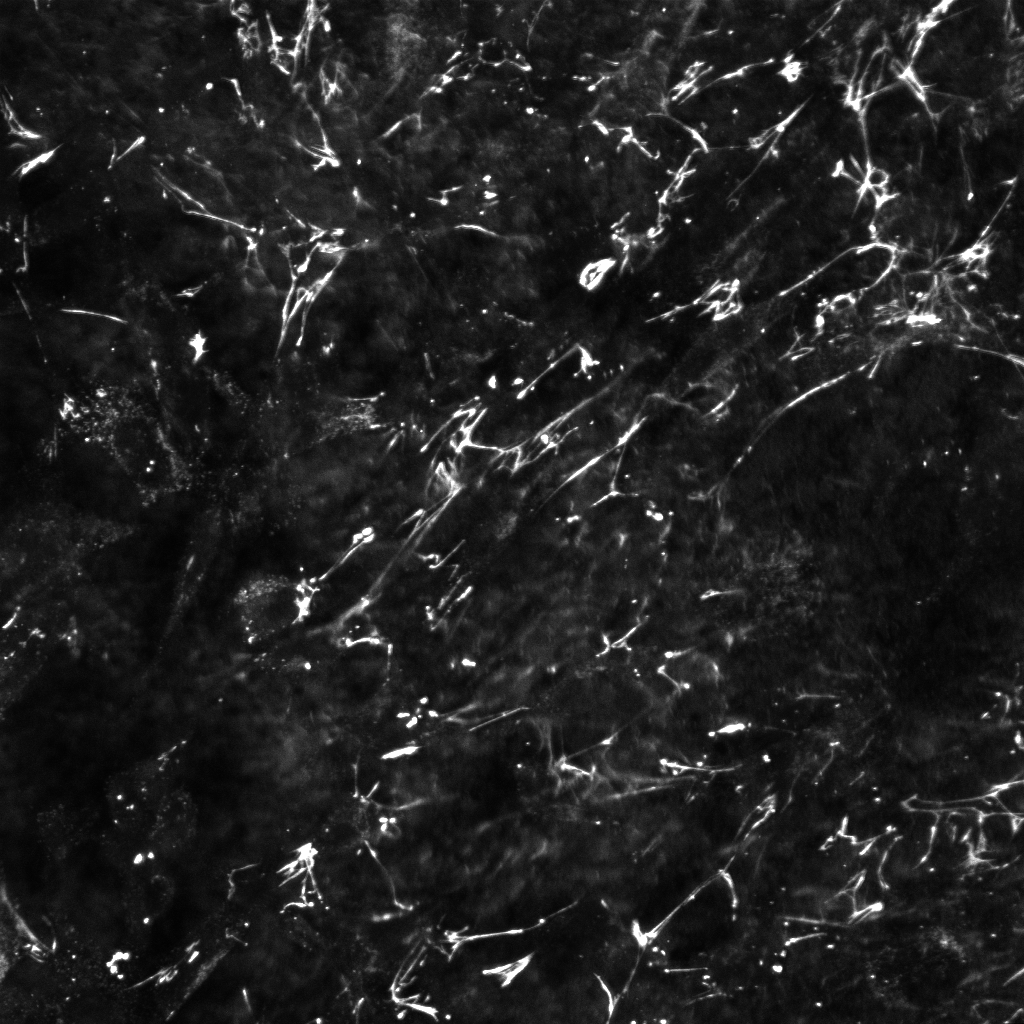

Supplement: Supplementary file 5 — Source data Fig. 1 [file 44319_2026_751_MOESM5_ESM.zip › Raw_data_Figure 1/Figure 1D/Confocal microscopy FN/GqKOMEFsFN_5Maximumintensityprojection.tif]

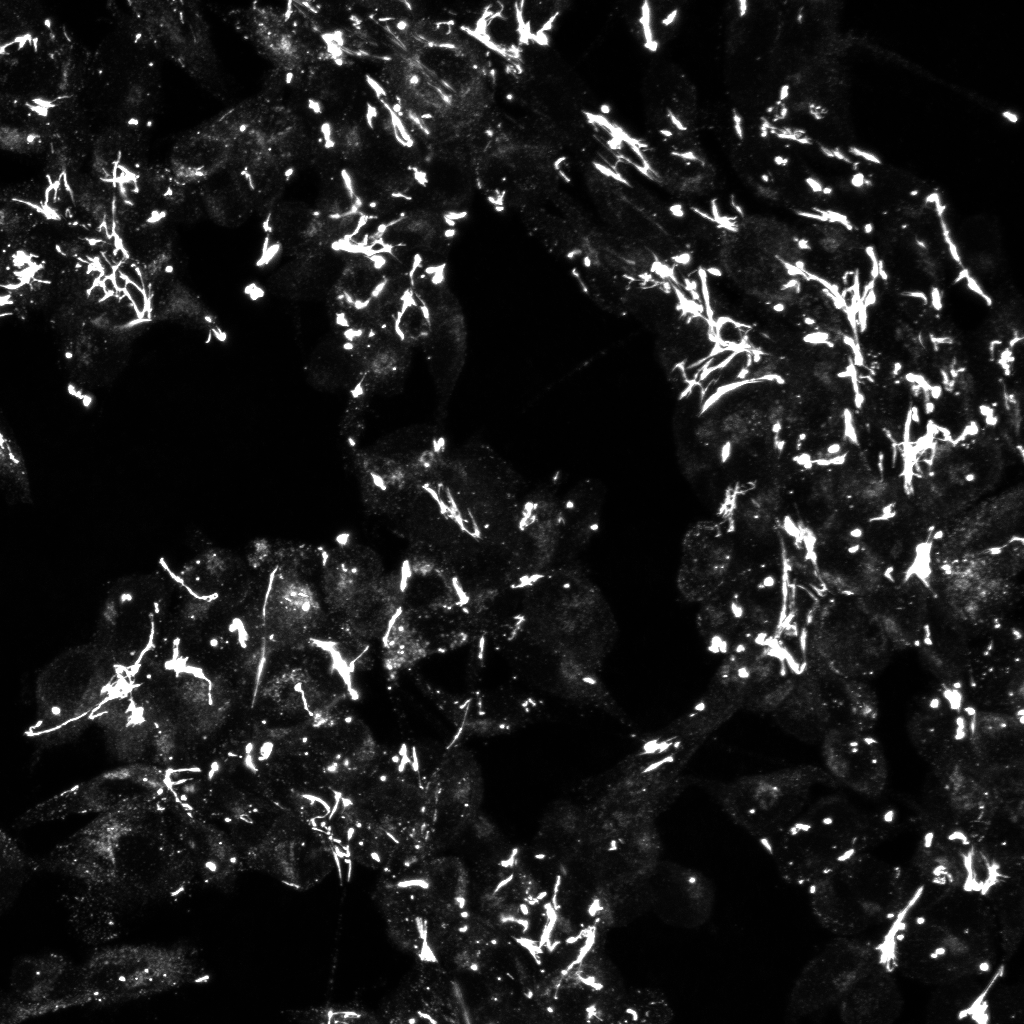

Supplement: Supplementary file 5 — Source data Fig. 1 [file 44319_2026_751_MOESM5_ESM.zip › Raw_data_Figure 1/Figure 1D/Confocal microscopy FN/GqKOMEFsFN_6Maximumintensityprojection.tif]

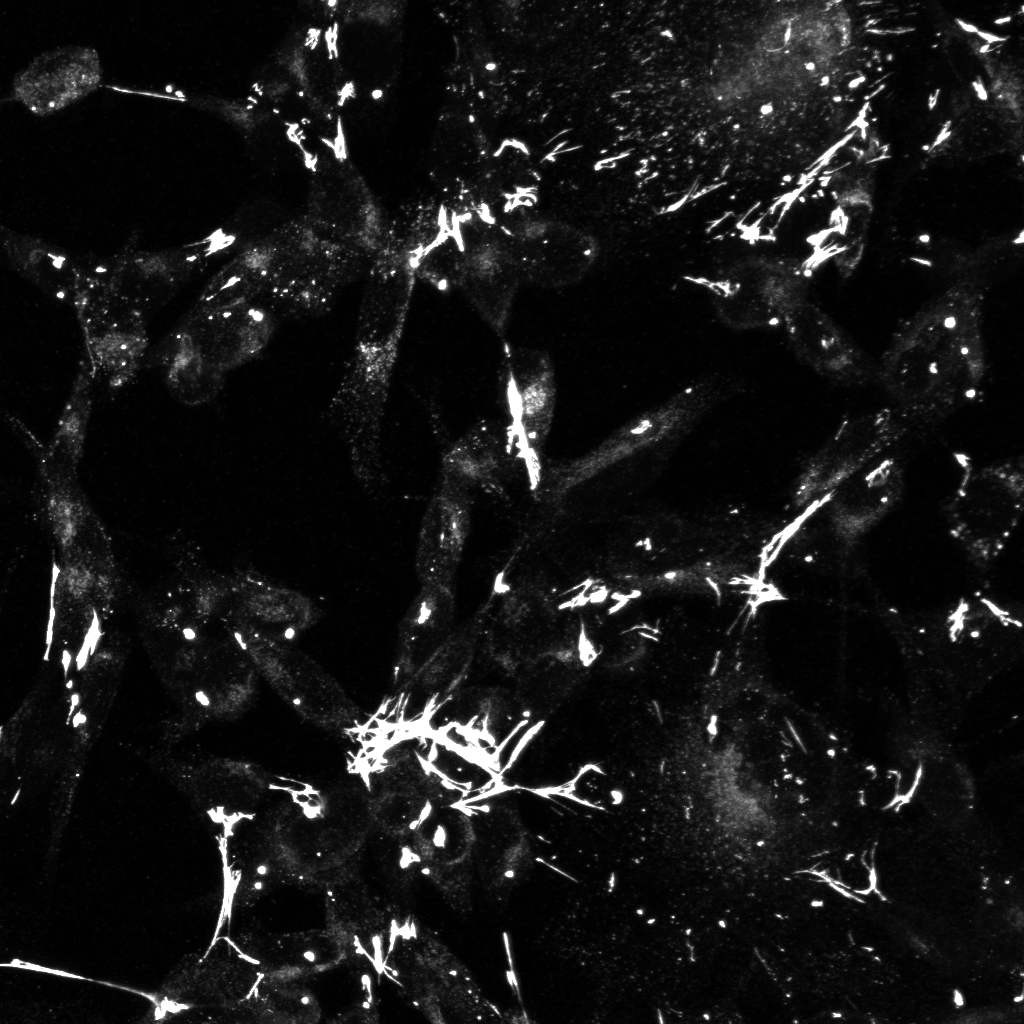

Supplement: Supplementary file 5 — Source data Fig. 1 [file 44319_2026_751_MOESM5_ESM.zip › Raw_data_Figure 1/Figure 1D/Confocal microscopy FN/GqKOMEFsFN_7Maximumintensityprojection.tif]

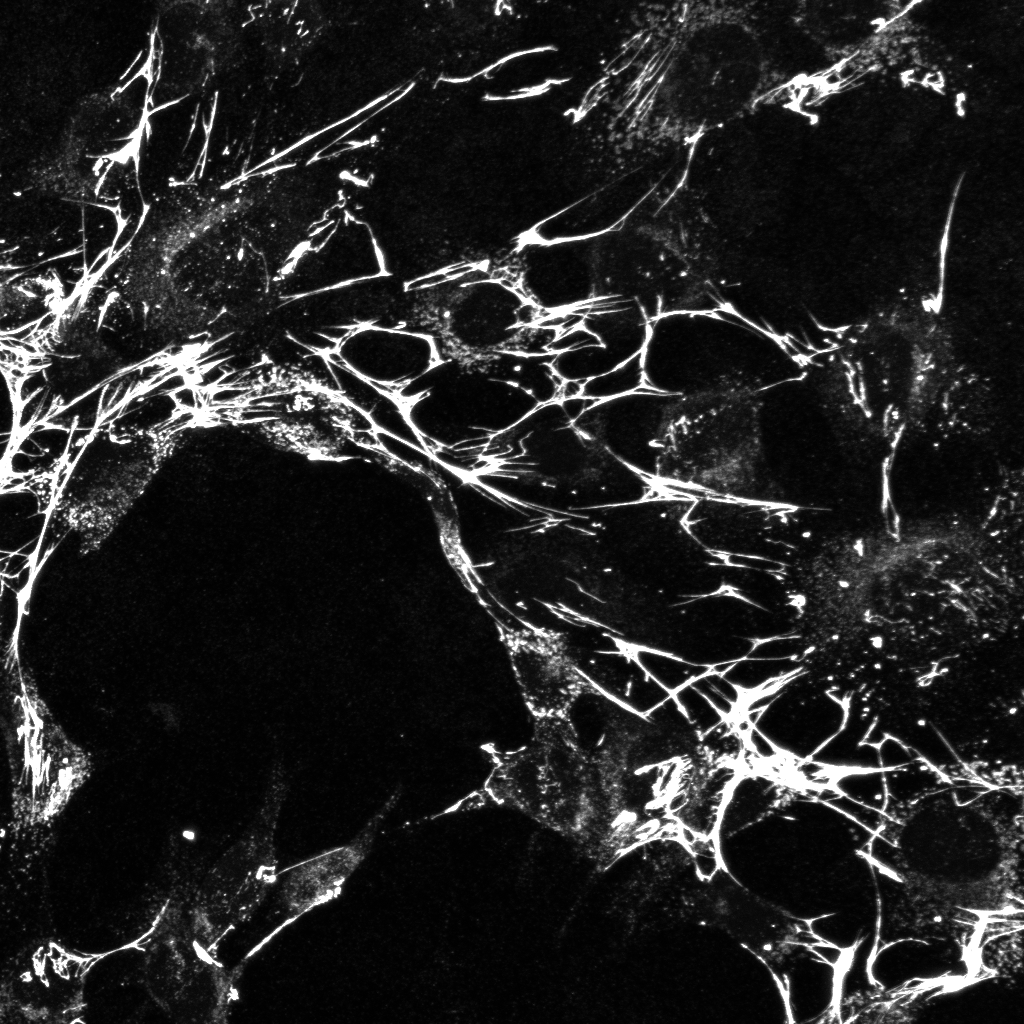

Supplement: Supplementary file 5 — Source data Fig. 1 [file 44319_2026_751_MOESM5_ESM.zip › Raw_data_Figure 1/Figure 1D/Confocal microscopy FN/GqKOMEFsFN_8Maximumintensityprojection.tif]

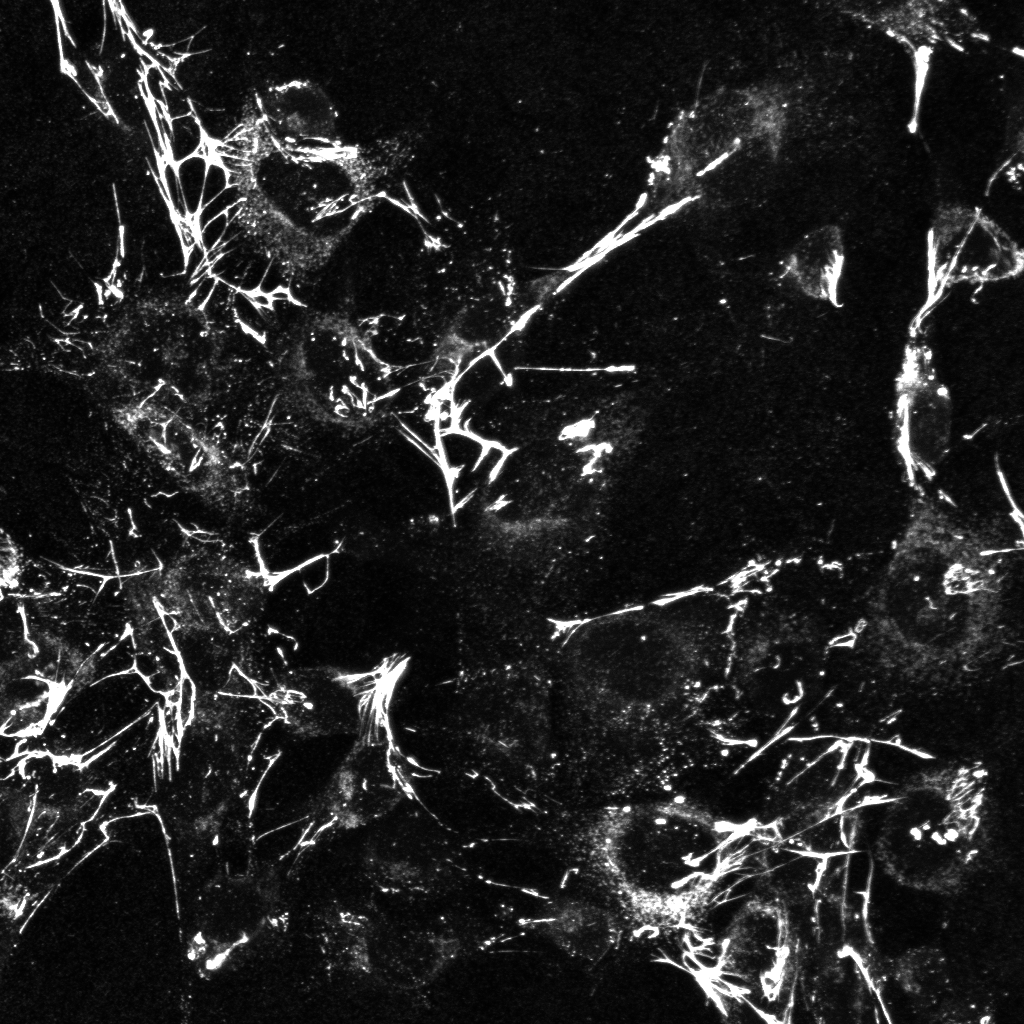

Supplement: Supplementary file 5 — Source data Fig. 1 [file 44319_2026_751_MOESM5_ESM.zip › Raw_data_Figure 1/Figure 1D/Confocal microscopy FN/GqKOMEFsFN_9Maximumintensityprojection.tif]

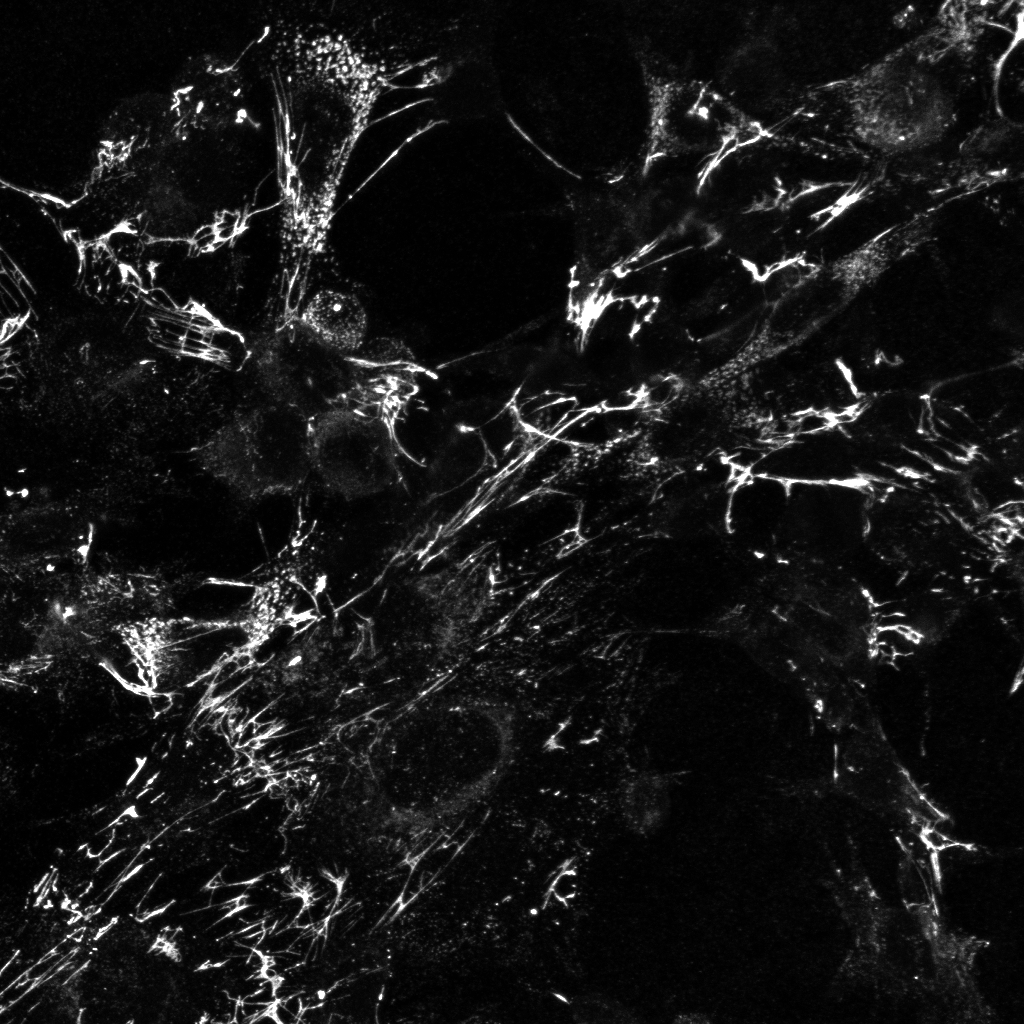

Supplement: Supplementary file 5 — Source data Fig. 1 [file 44319_2026_751_MOESM5_ESM.zip › Raw_data_Figure 1/Figure 1D/Confocal microscopy FN/GqKOMEFsFN_Maximumintensityprojection.tif]

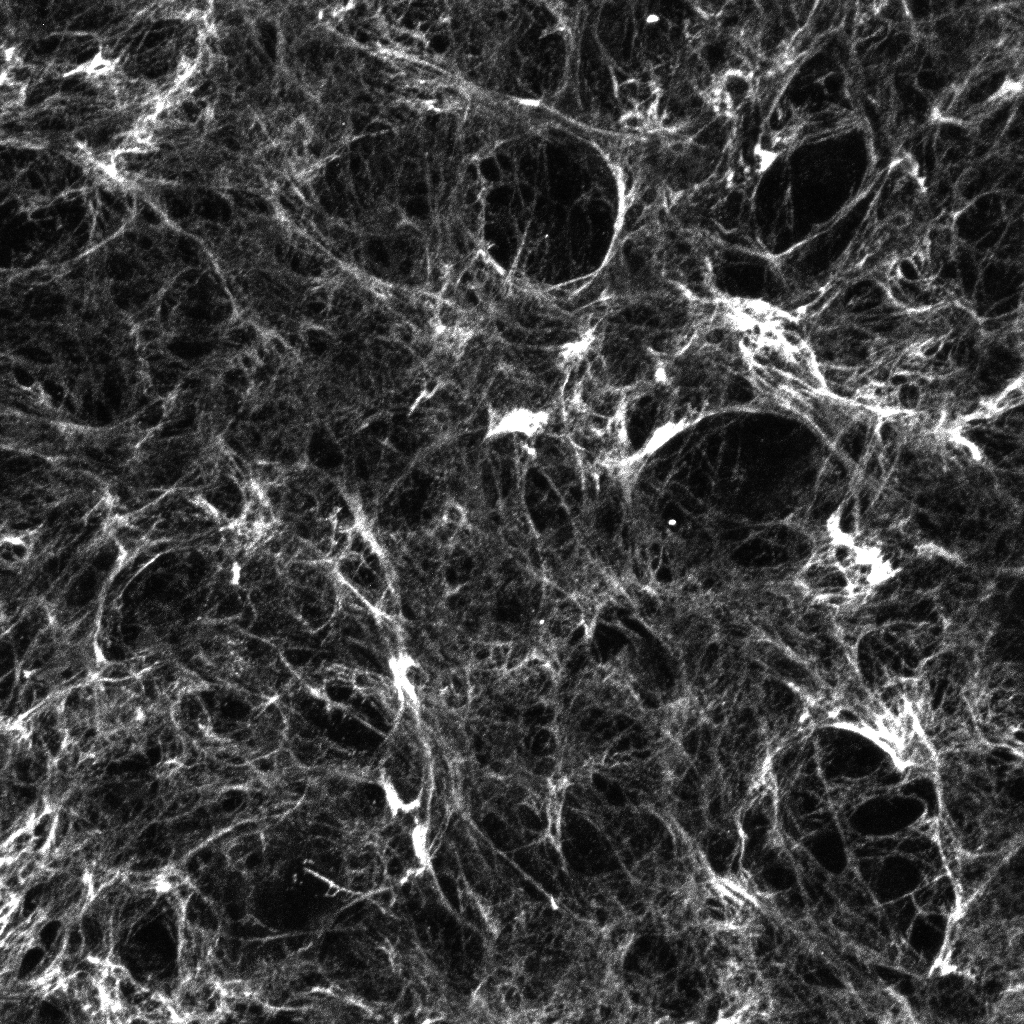

Supplement: Supplementary file 5 — Source data Fig. 1 [file 44319_2026_751_MOESM5_ESM.zip › Raw_data_Figure 1/Figure 1D/Confocal microscopy FN/WT selected.tif]

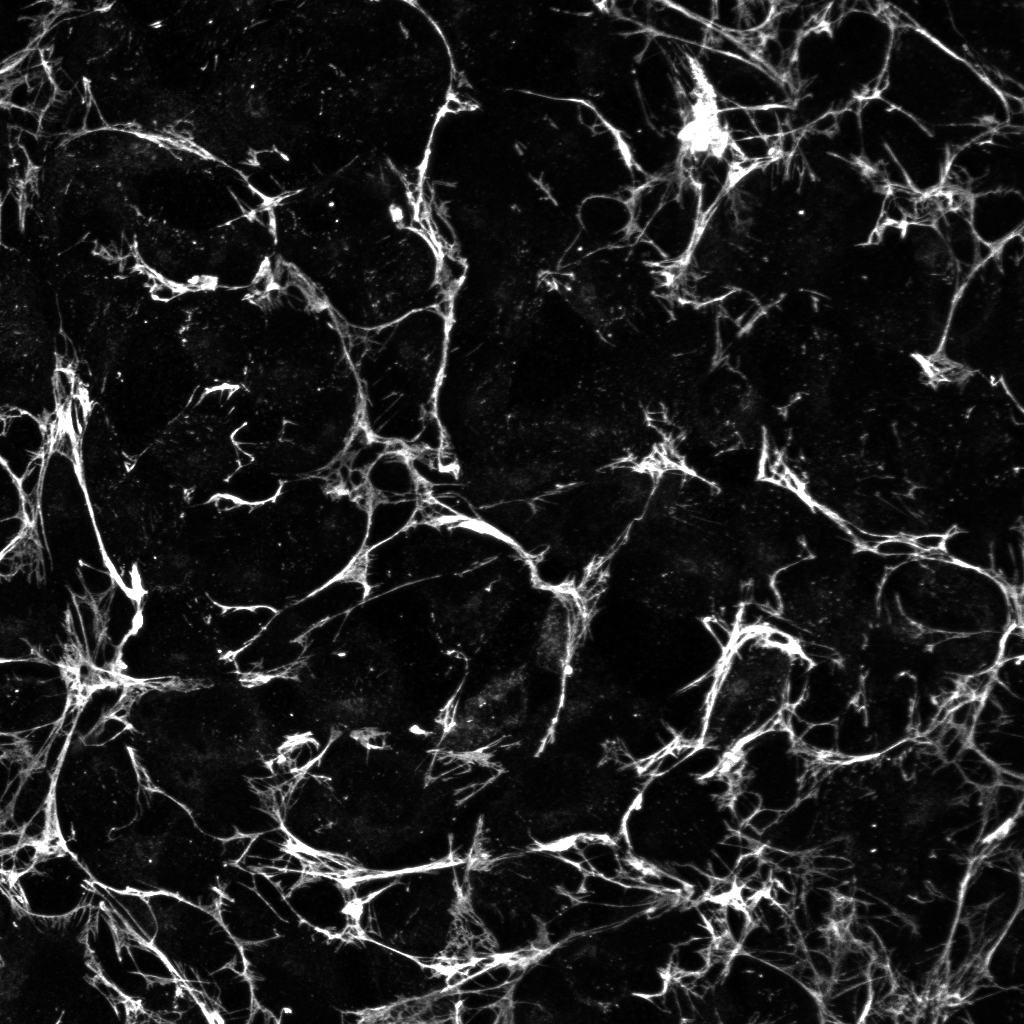

Supplement: Supplementary file 5 — Source data Fig. 1 [file 44319_2026_751_MOESM5_ESM.zip › Raw_data_Figure 1/Figure 1D/Confocal microscopy FN/WTMEFsFN_ Maximumintensityprojection.tif]

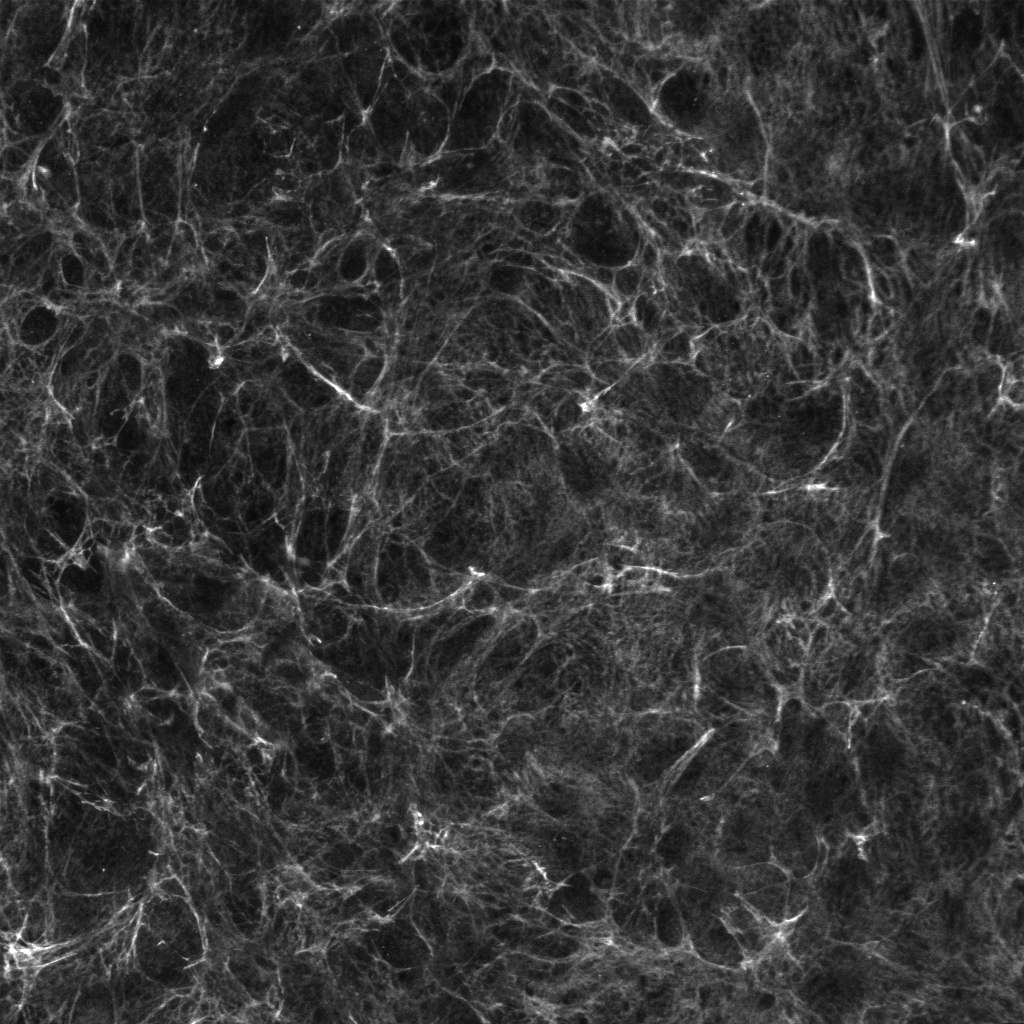

Supplement: Supplementary file 5 — Source data Fig. 1 [file 44319_2026_751_MOESM5_ESM.zip › Raw_data_Figure 1/Figure 1D/Confocal microscopy FN/WTMEFsFN_10Maximumintensityprojection.tif]

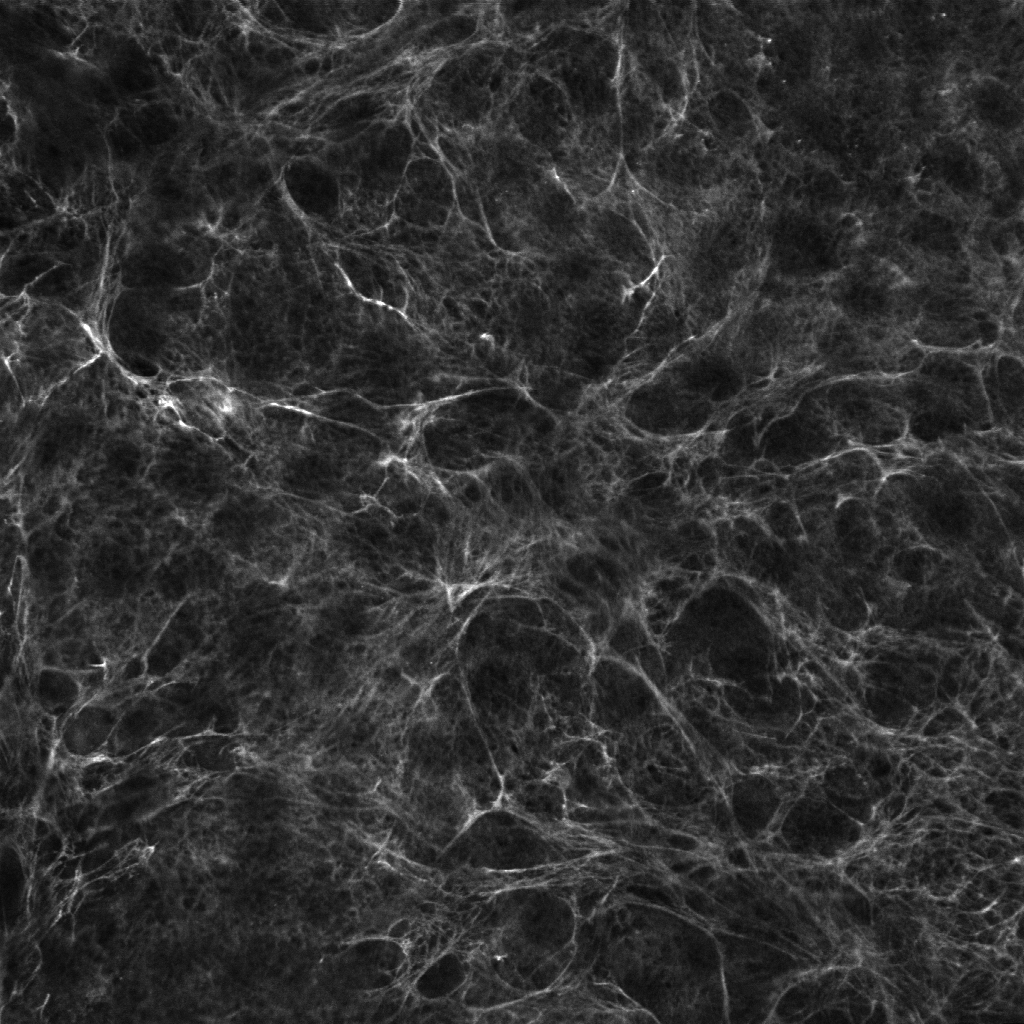

Supplement: Supplementary file 5 — Source data Fig. 1 [file 44319_2026_751_MOESM5_ESM.zip › Raw_data_Figure 1/Figure 1D/Confocal microscopy FN/WTMEFsFN_11Maximumintensityprojection.tif]

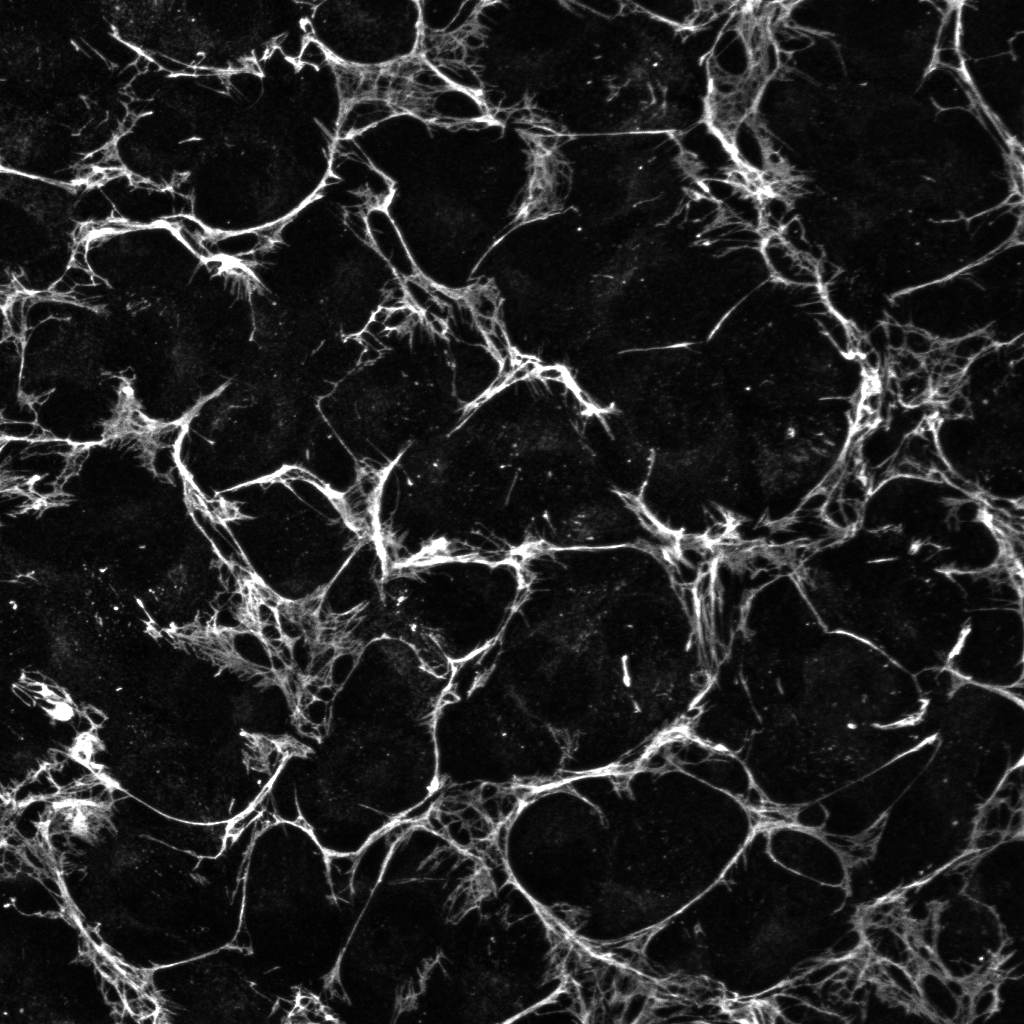

Supplement: Supplementary file 5 — Source data Fig. 1 [file 44319_2026_751_MOESM5_ESM.zip › Raw_data_Figure 1/Figure 1D/Confocal microscopy FN/WTMEFsFN_2Maximumintensityprojection.tif]

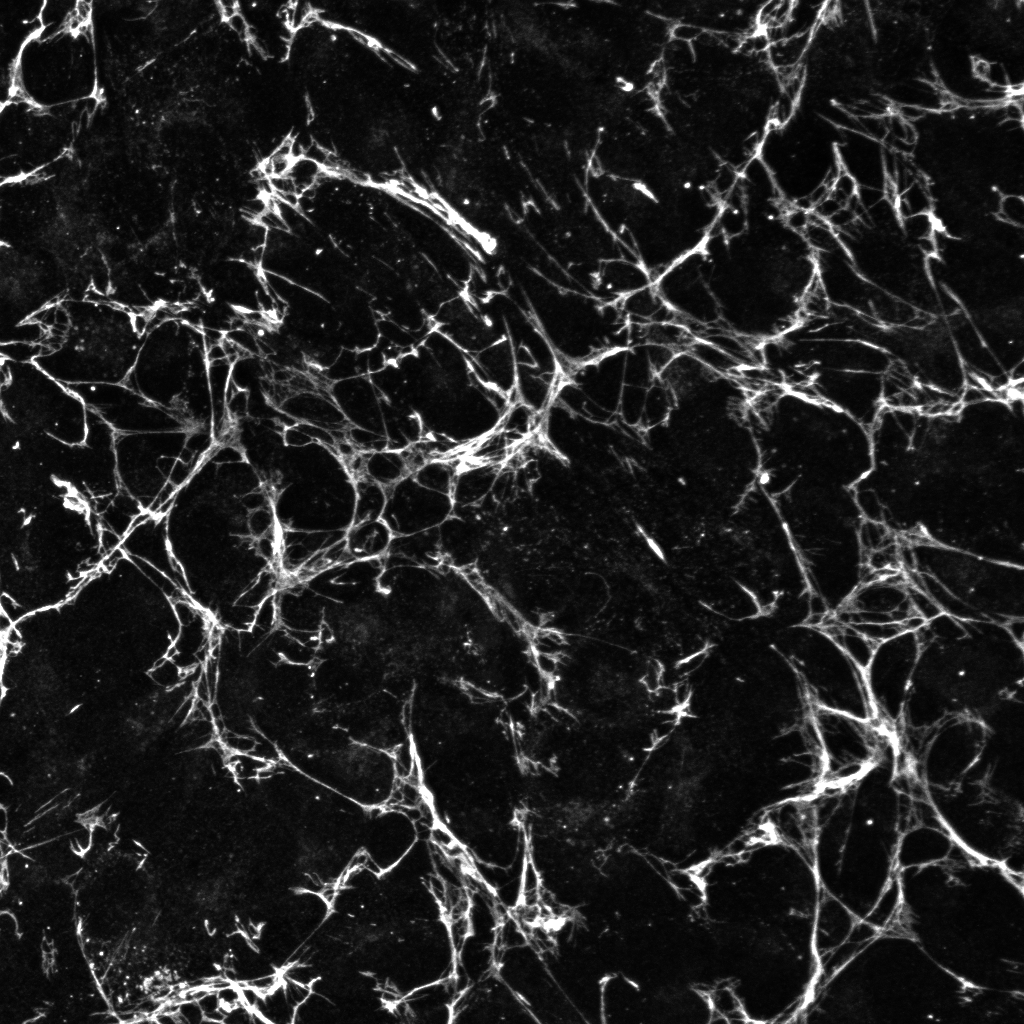

Supplement: Supplementary file 5 — Source data Fig. 1 [file 44319_2026_751_MOESM5_ESM.zip › Raw_data_Figure 1/Figure 1D/Confocal microscopy FN/WTMEFsFN_3Maximumintensityprojection.tif]

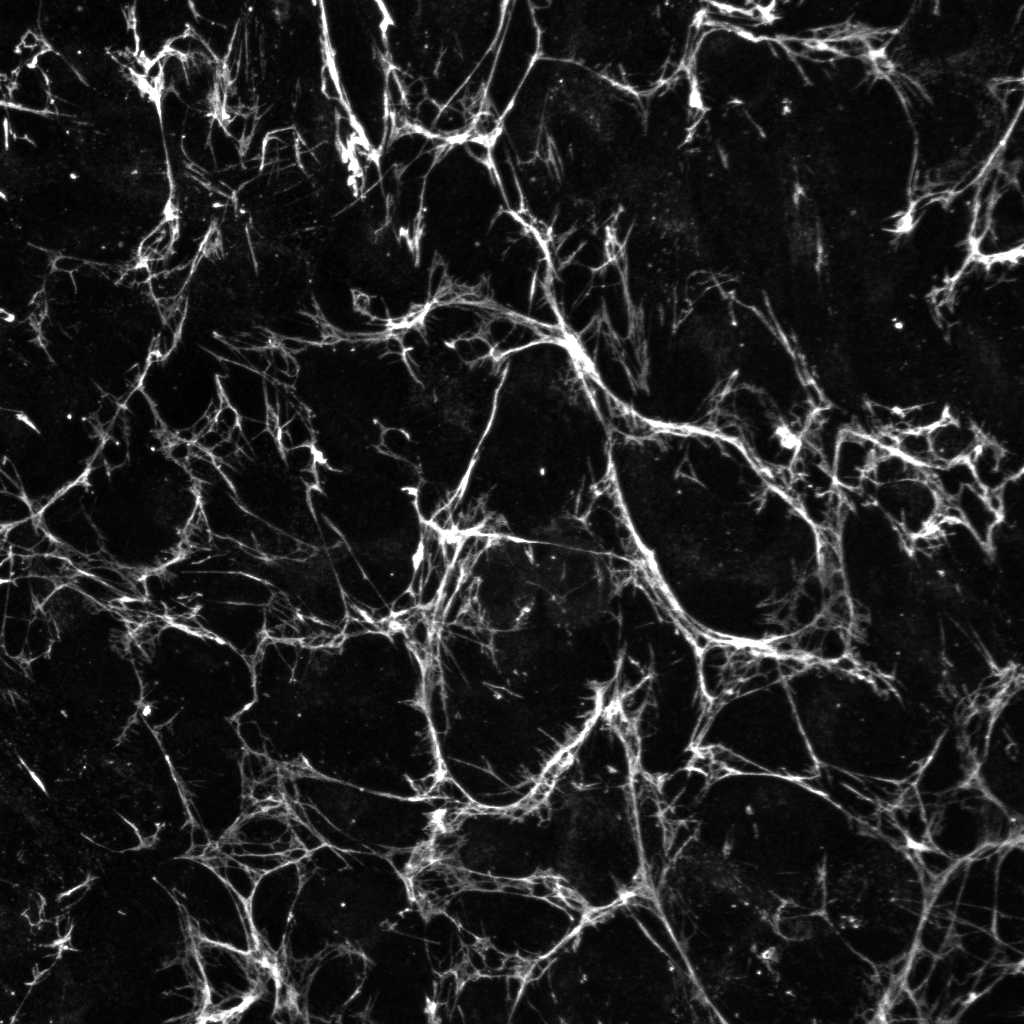

Supplement: Supplementary file 5 — Source data Fig. 1 [file 44319_2026_751_MOESM5_ESM.zip › Raw_data_Figure 1/Figure 1D/Confocal microscopy FN/WTMEFsFN_4Maximumintensityprojection.tif]

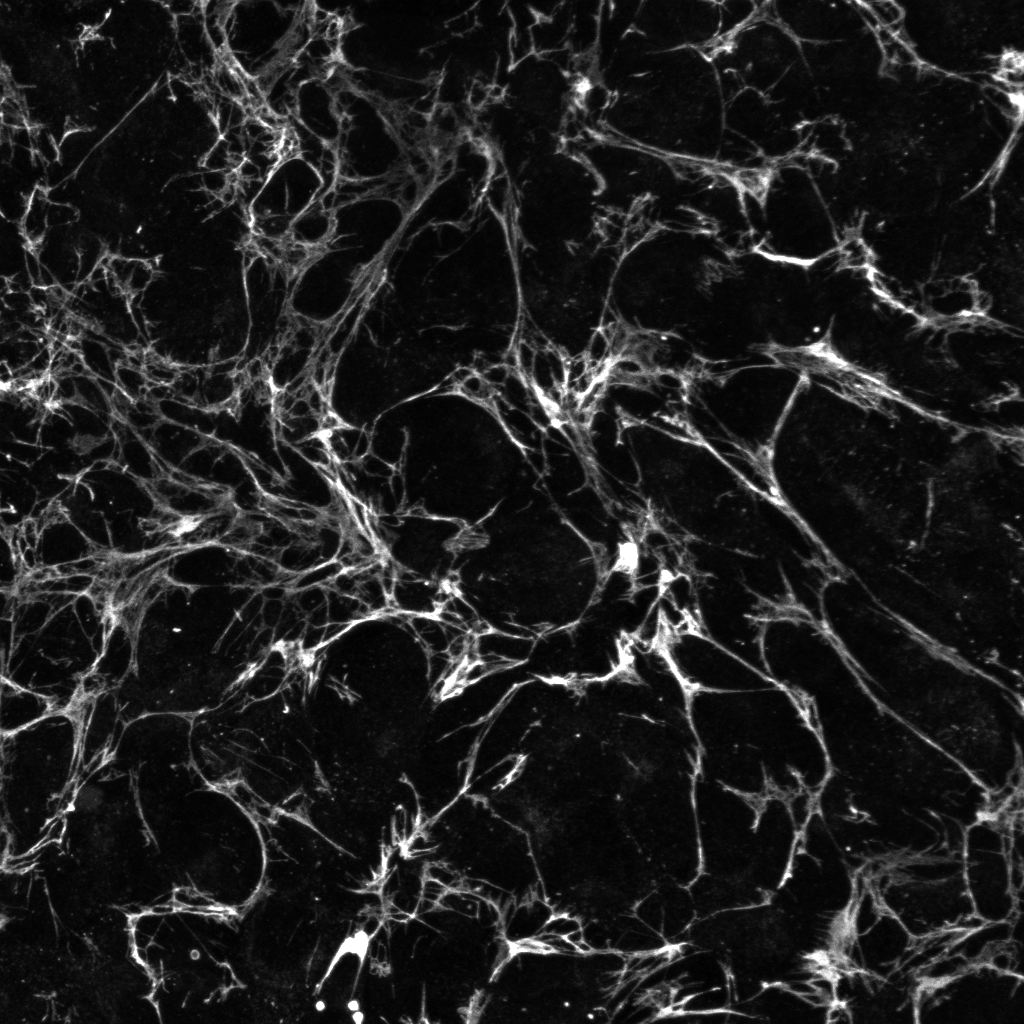

Supplement: Supplementary file 5 — Source data Fig. 1 [file 44319_2026_751_MOESM5_ESM.zip › Raw_data_Figure 1/Figure 1D/Confocal microscopy FN/WTMEFsFN_5Maximumintensityprojection.tif]

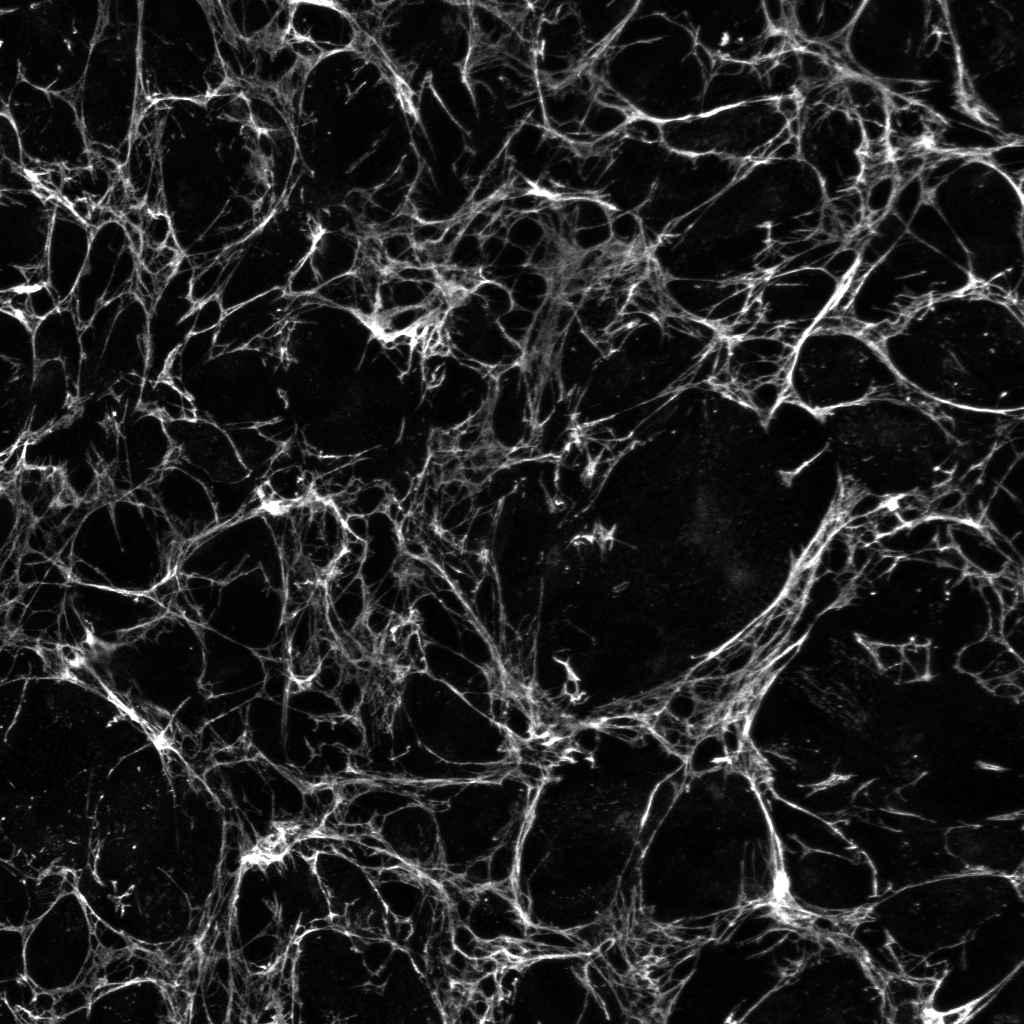

Supplement: Supplementary file 5 — Source data Fig. 1 [file 44319_2026_751_MOESM5_ESM.zip › Raw_data_Figure 1/Figure 1D/Confocal microscopy FN/WTMEFsFN_7Maximumintensityprojection.tif]

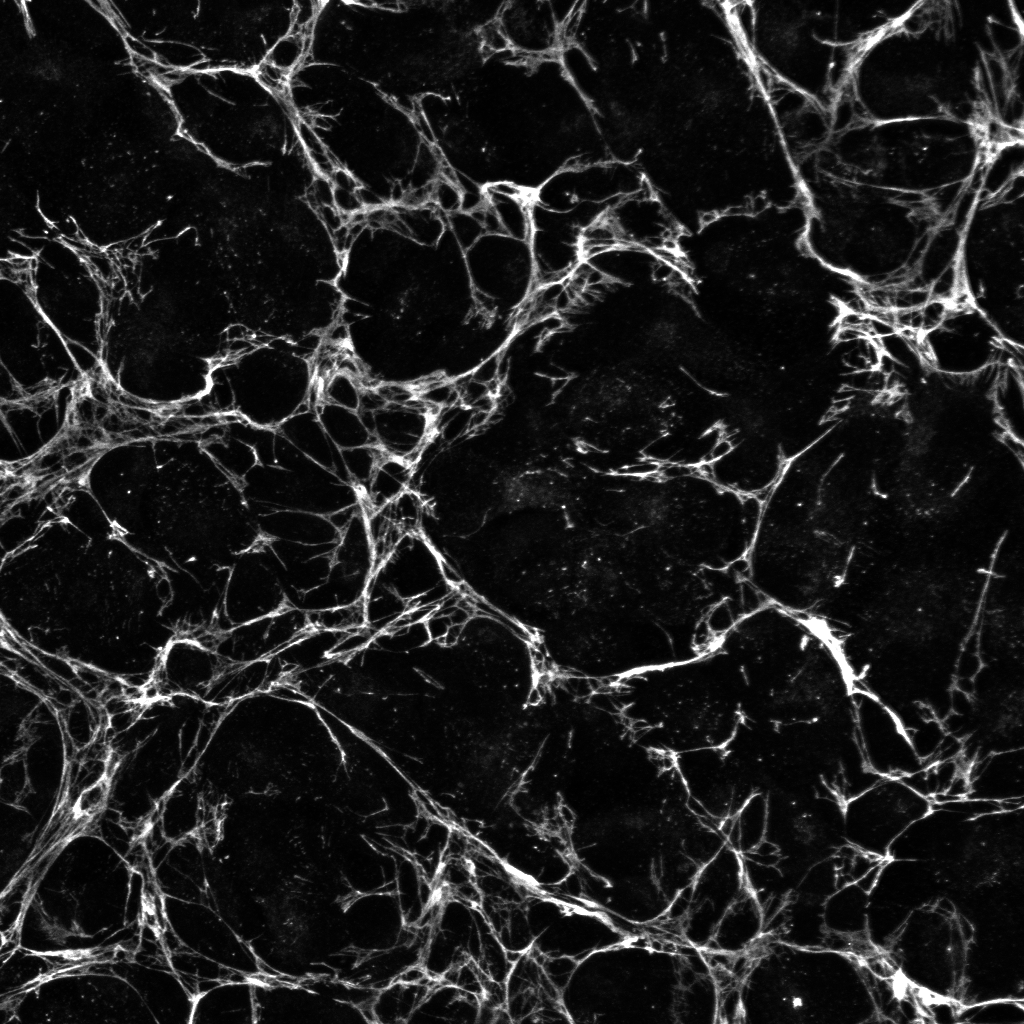

Supplement: Supplementary file 5 — Source data Fig. 1 [file 44319_2026_751_MOESM5_ESM.zip › Raw_data_Figure 1/Figure 1D/Confocal microscopy FN/WTMEFsFN_8Maximumintensityprojection.tif]

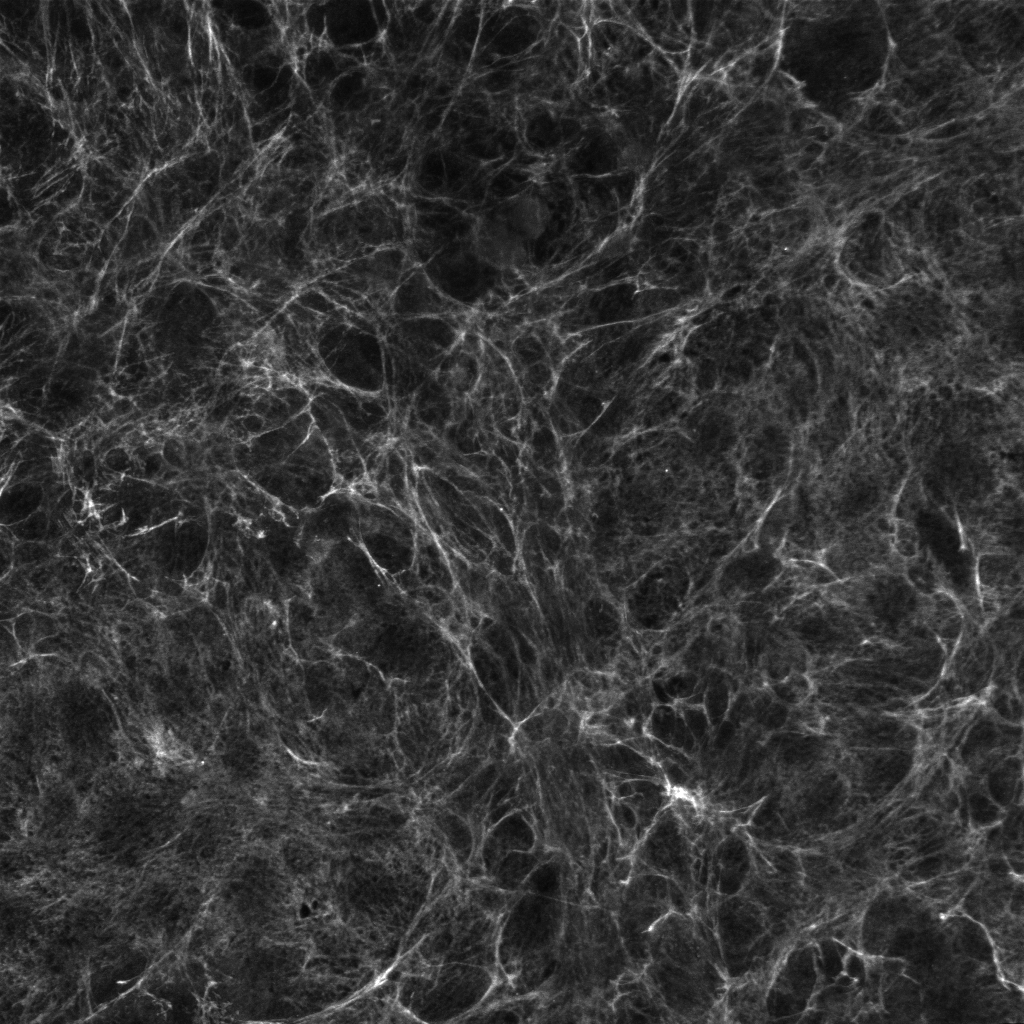

Supplement: Supplementary file 5 — Source data Fig. 1 [file 44319_2026_751_MOESM5_ESM.zip › Raw_data_Figure 1/Figure 1D/Confocal microscopy FN/WTMEFsFN_9Maximumintensityprojection.tif]

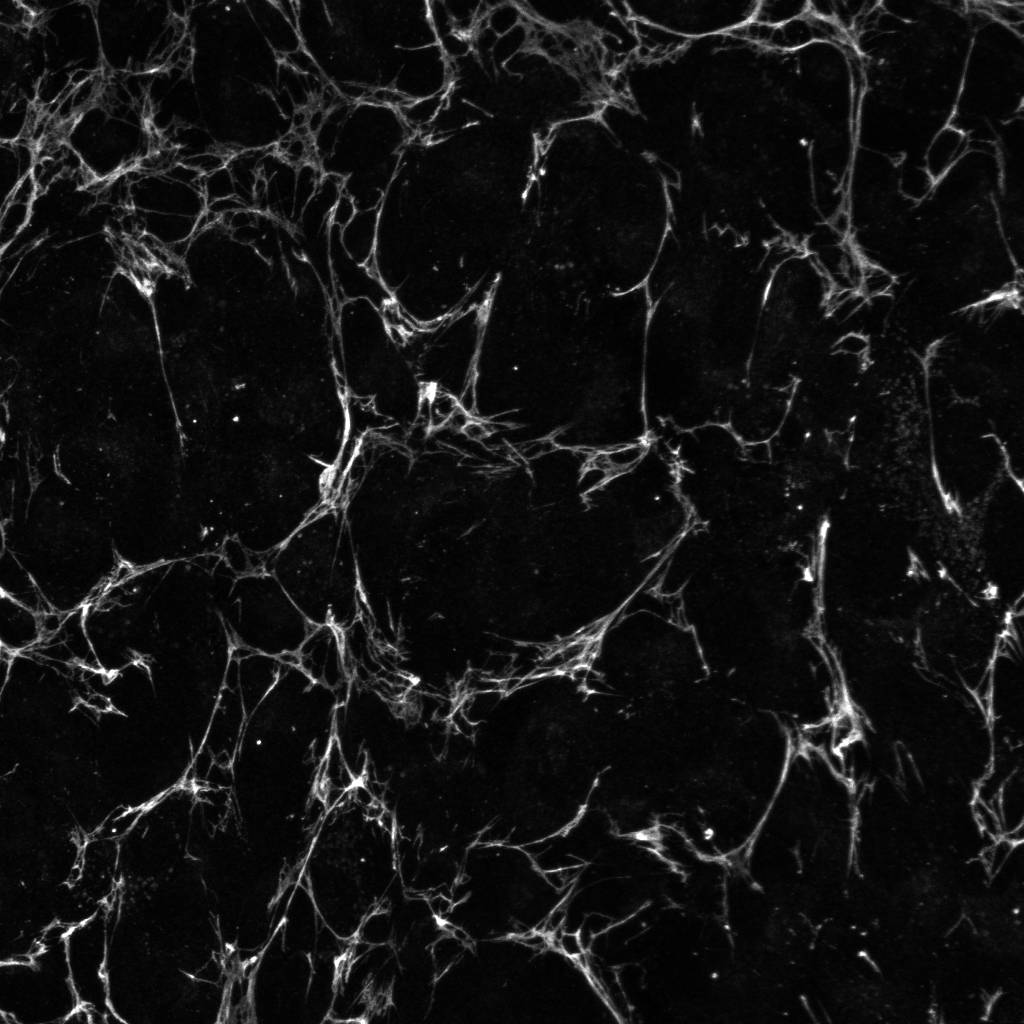

Supplement: Supplementary file 5 — Source data Fig. 1 [file 44319_2026_751_MOESM5_ESM.zip › Raw_data_Figure 1/Figure 1D/Confocal microscopy FN/WTMEFsFN_Maximumintensityprojection.tif]

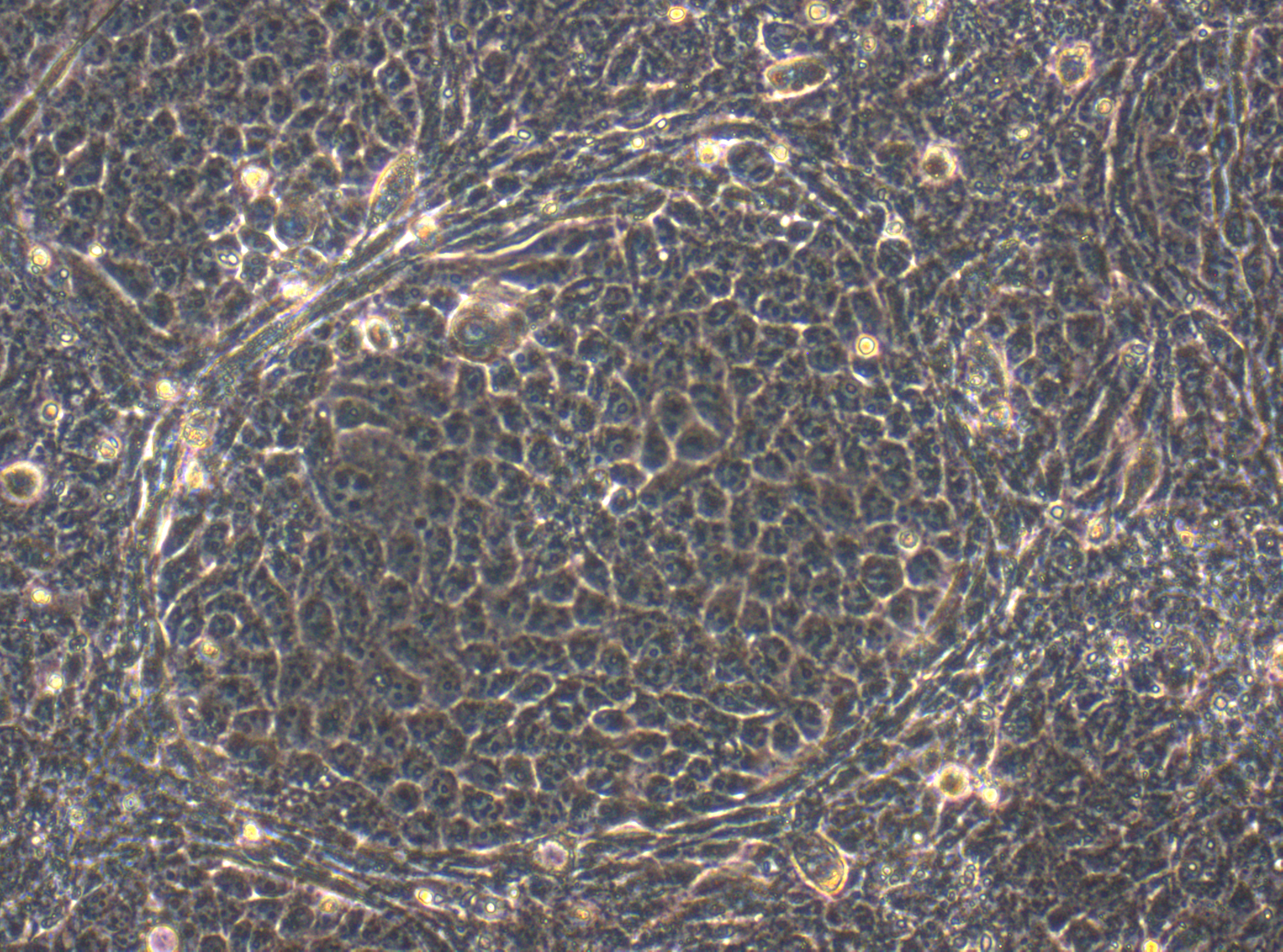

Supplement: Supplementary file 6 — Source data Fig. 2 [file 44319_2026_751_MOESM6_ESM.zip › Raw_data_Figure 2/Figure 2A/coculture Cal27/Cal27 + KI zoom.tif]

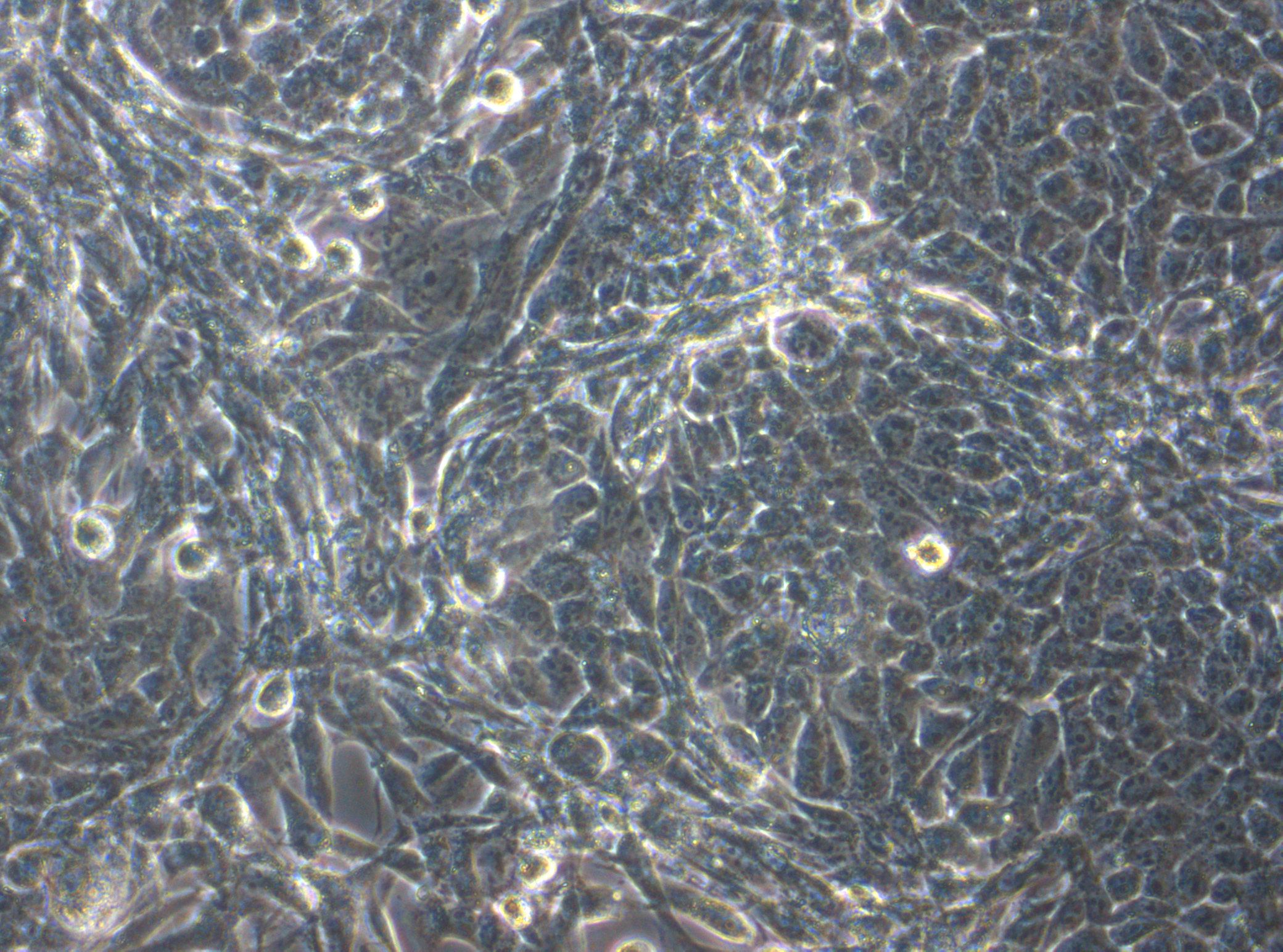

Supplement: Supplementary file 6 — Source data Fig. 2 [file 44319_2026_751_MOESM6_ESM.zip › Raw_data_Figure 2/Figure 2A/coculture Cal27/Cal27 + KO 4 zoom.tif]

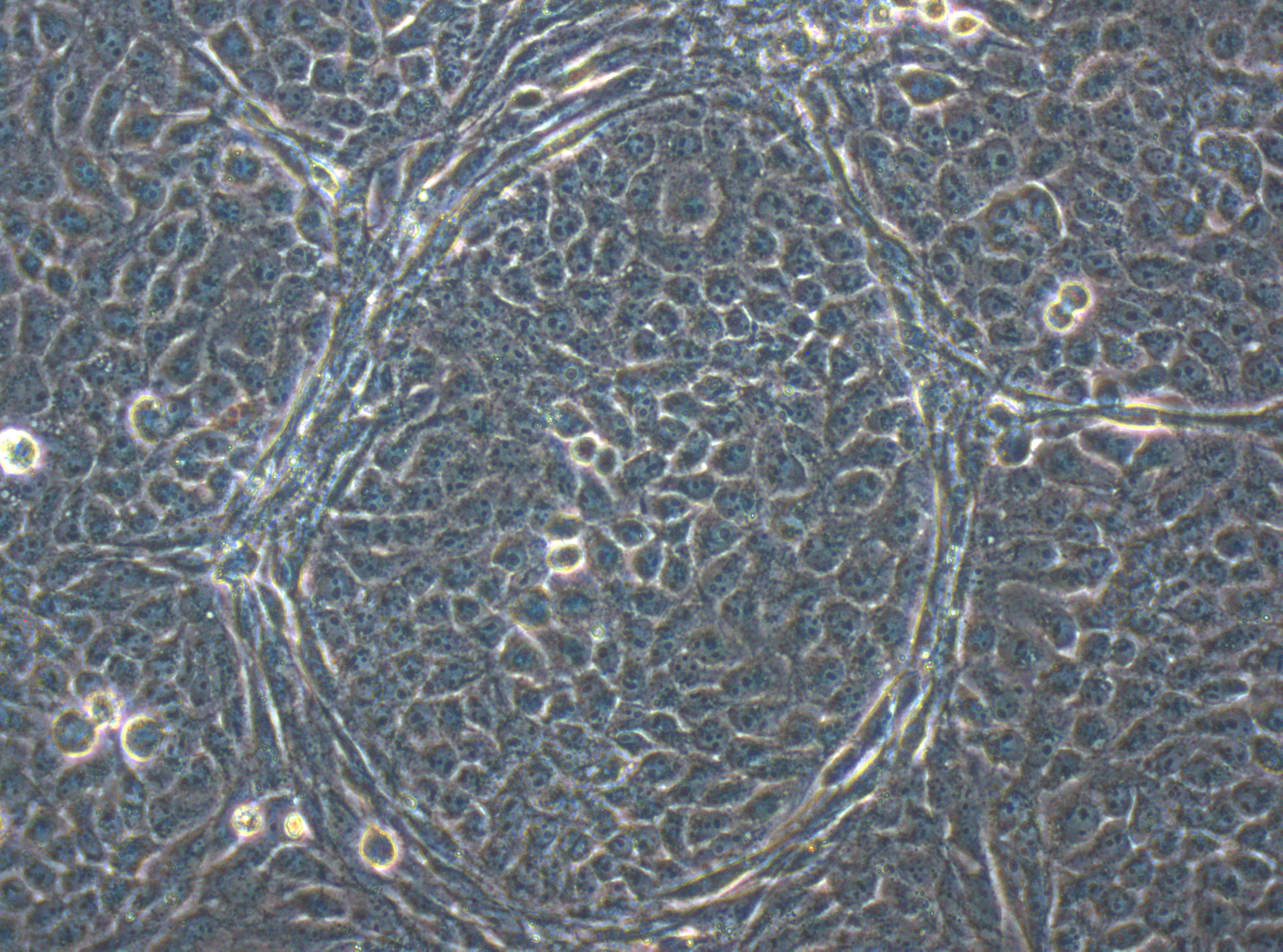

Supplement: Supplementary file 6 — Source data Fig. 2 [file 44319_2026_751_MOESM6_ESM.zip › Raw_data_Figure 2/Figure 2A/coculture Cal27/Cal27 + WT zoom.tif]

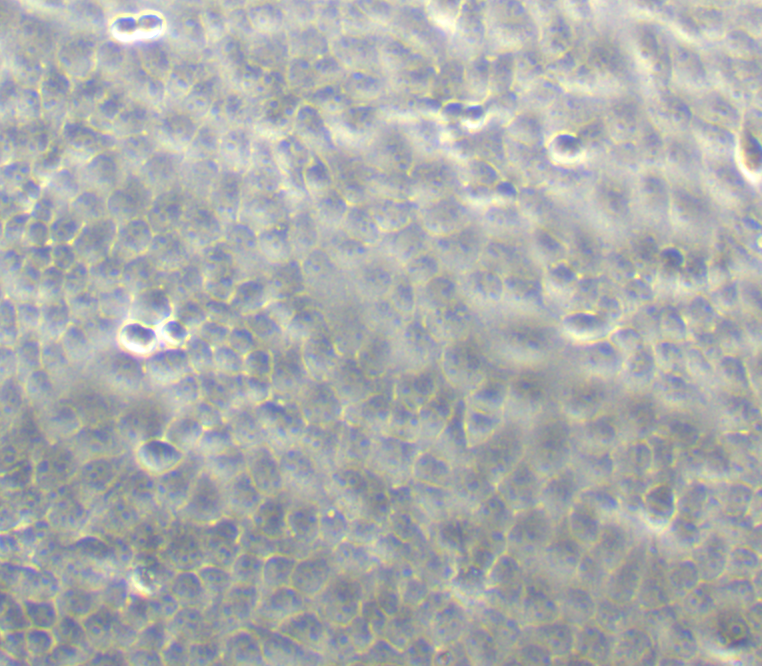

Supplement: Supplementary file 6 — Source data Fig. 2 [file 44319_2026_751_MOESM6_ESM.zip › Raw_data_Figure 2/Figure 2A/coculture Cal27/Cal27 - zoom.tif]

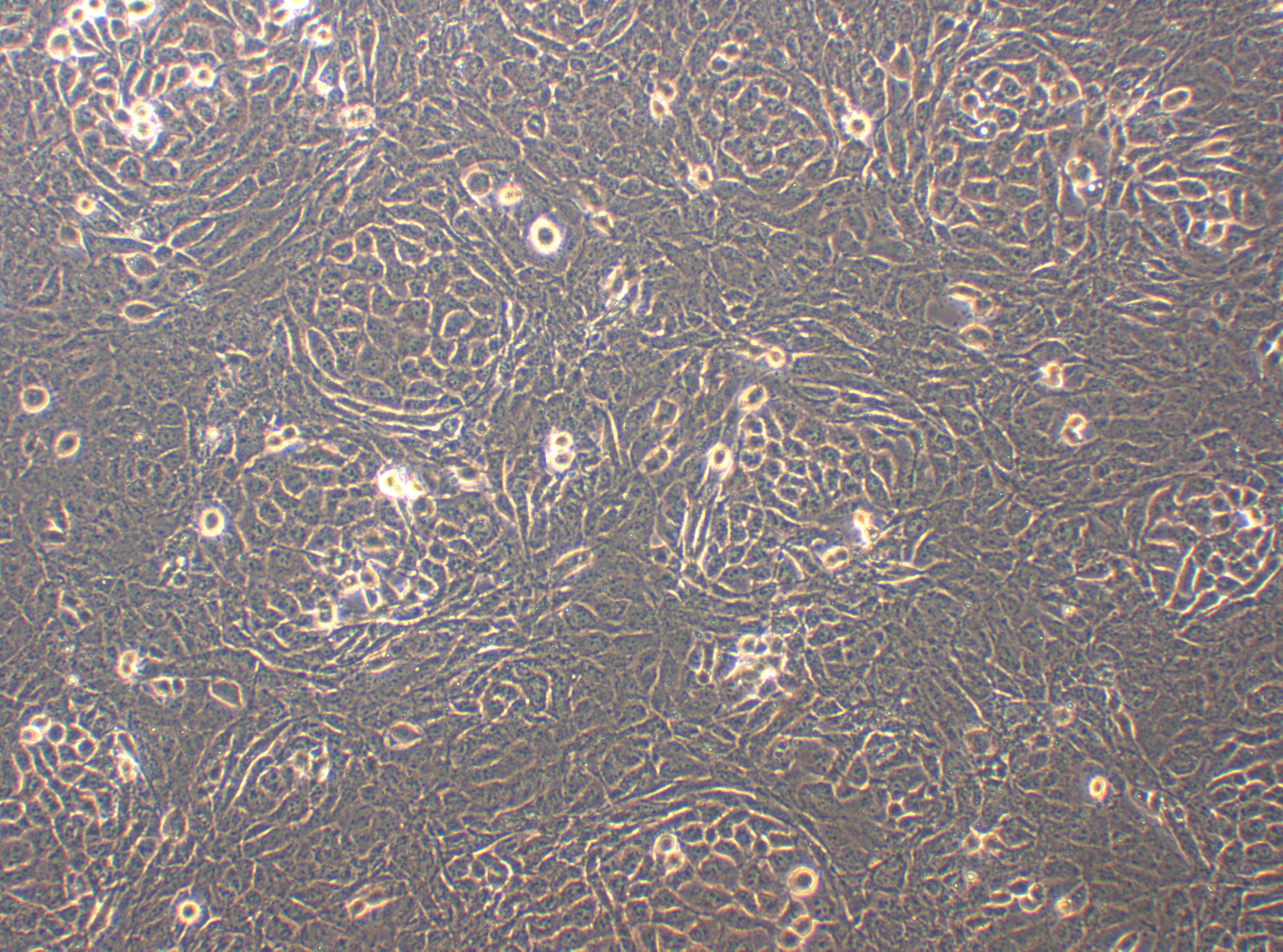

Supplement: Supplementary file 6 — Source data Fig. 2 [file 44319_2026_751_MOESM6_ESM.zip › Raw_data_Figure 2/Figure 2A/coculture HN13/hn13 +ki 2.tif]

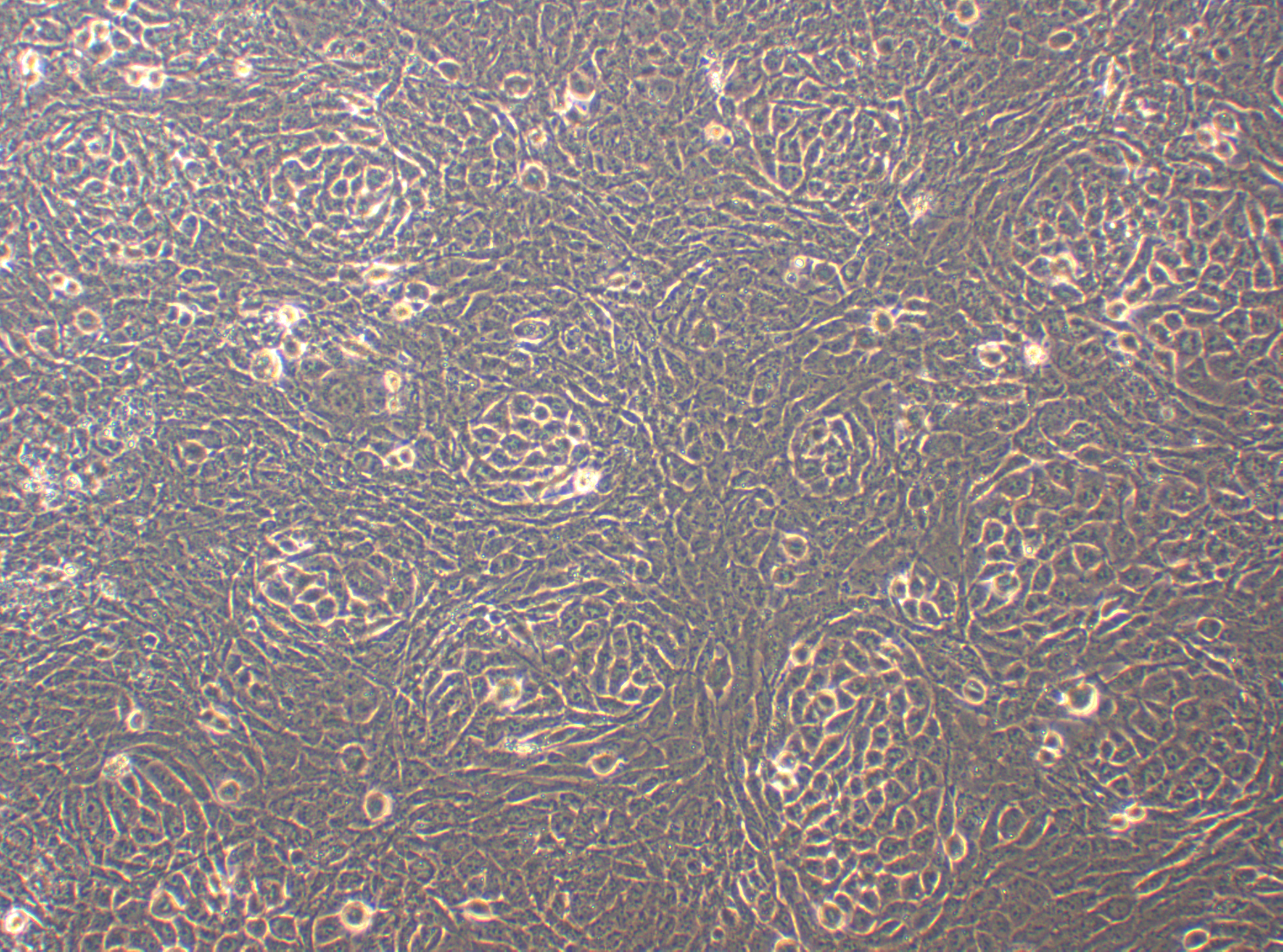

Supplement: Supplementary file 6 — Source data Fig. 2 [file 44319_2026_751_MOESM6_ESM.zip › Raw_data_Figure 2/Figure 2A/coculture HN13/hn13 +ki.tif]

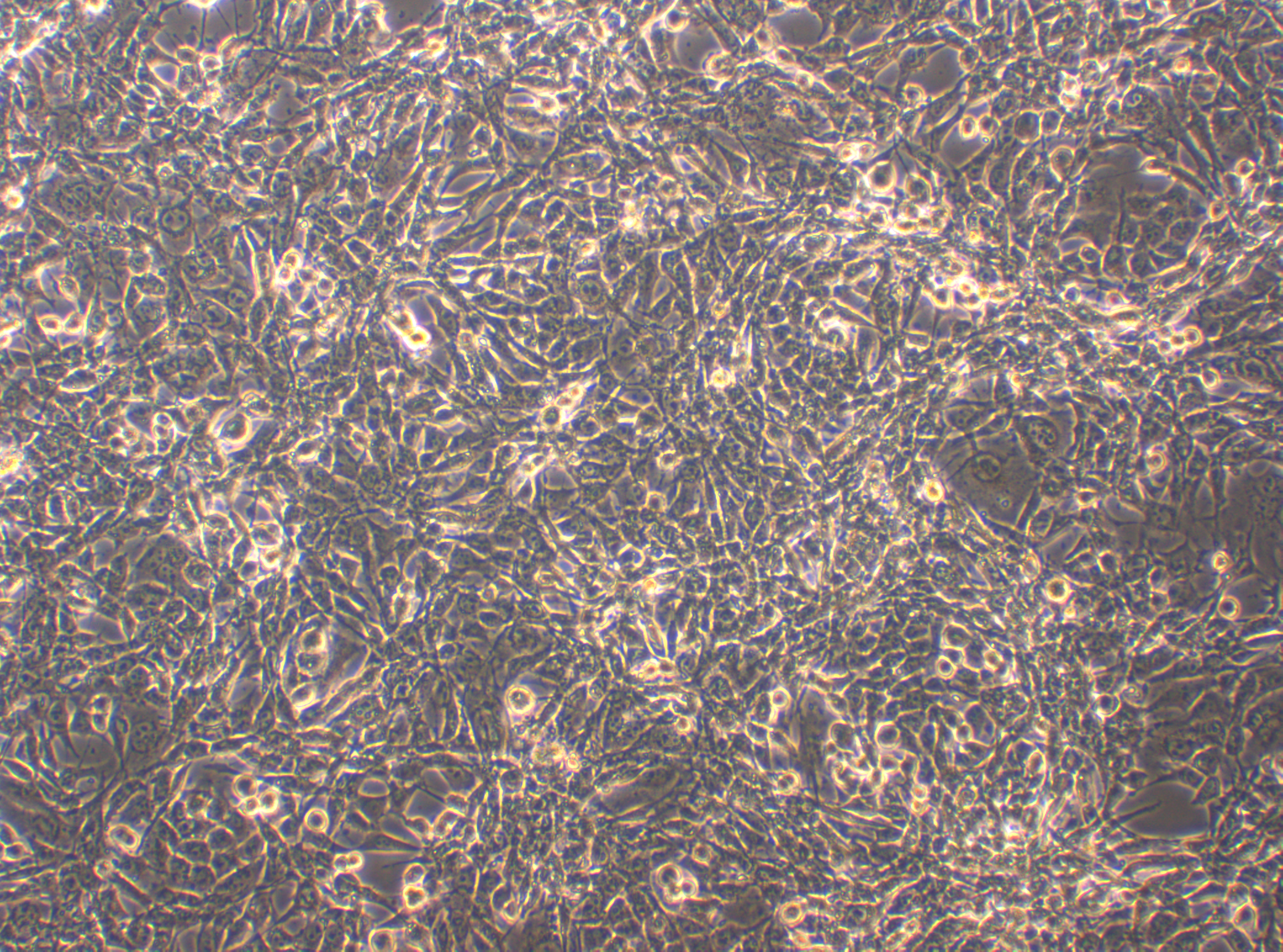

Supplement: Supplementary file 6 — Source data Fig. 2 [file 44319_2026_751_MOESM6_ESM.zip › Raw_data_Figure 2/Figure 2A/coculture HN13/hn13 +ko.tif]

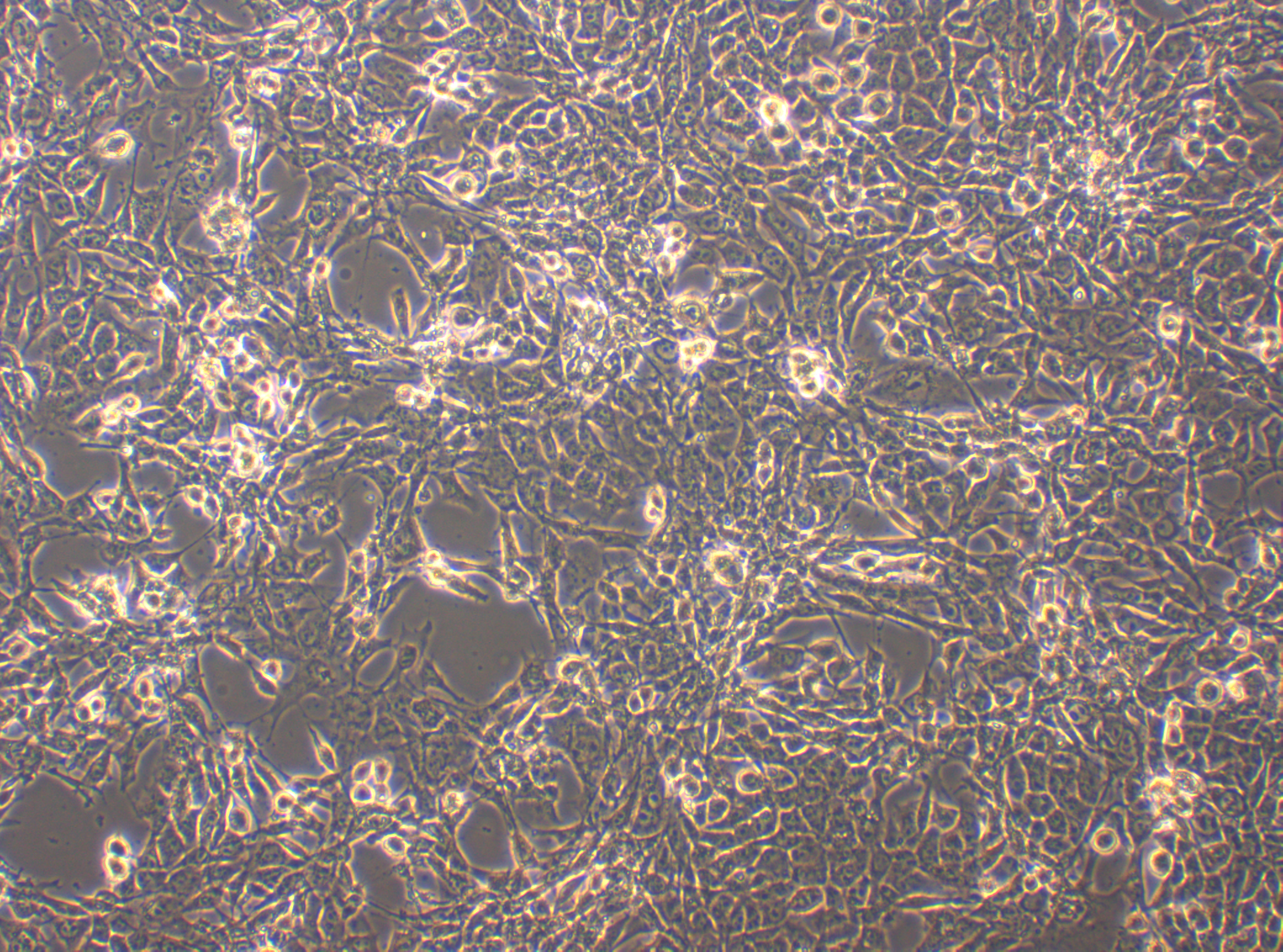

Supplement: Supplementary file 6 — Source data Fig. 2 [file 44319_2026_751_MOESM6_ESM.zip › Raw_data_Figure 2/Figure 2A/coculture HN13/hn13 +ko2.tif]

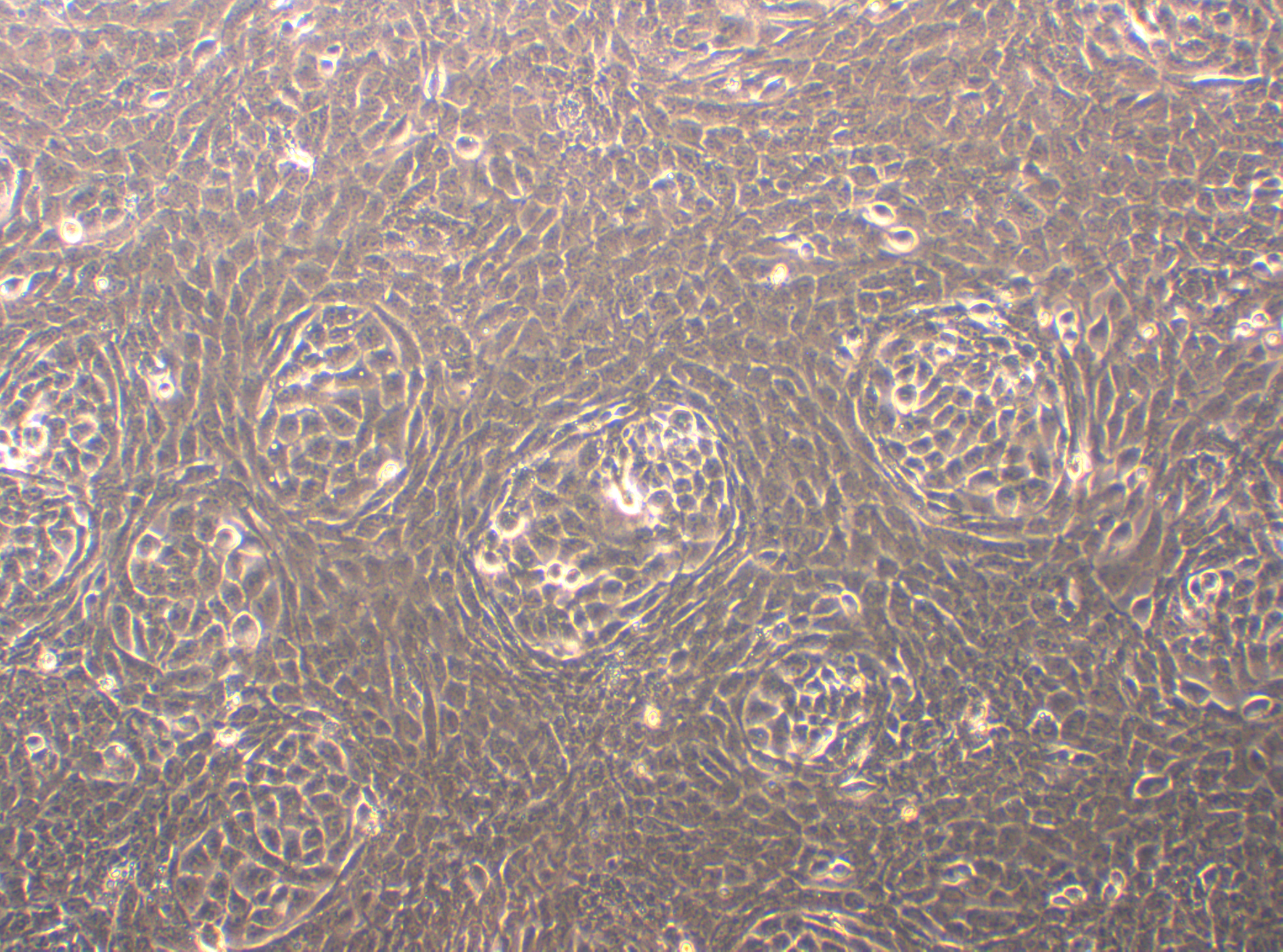

Supplement: Supplementary file 6 — Source data Fig. 2 [file 44319_2026_751_MOESM6_ESM.zip › Raw_data_Figure 2/Figure 2A/coculture HN13/hn13 +wt 2.tif]

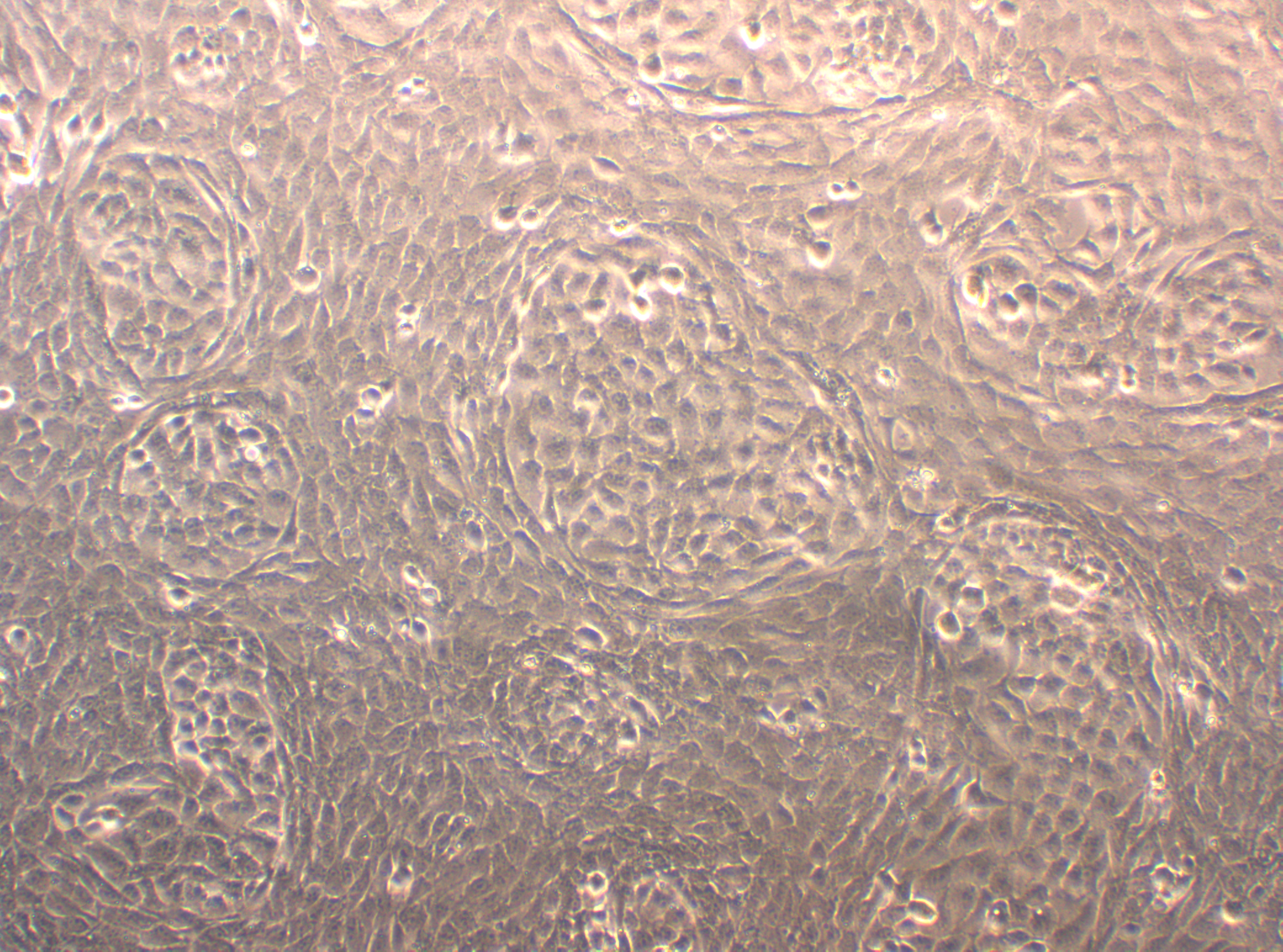

Supplement: Supplementary file 6 — Source data Fig. 2 [file 44319_2026_751_MOESM6_ESM.zip › Raw_data_Figure 2/Figure 2A/coculture HN13/hn13 +wt.tif]

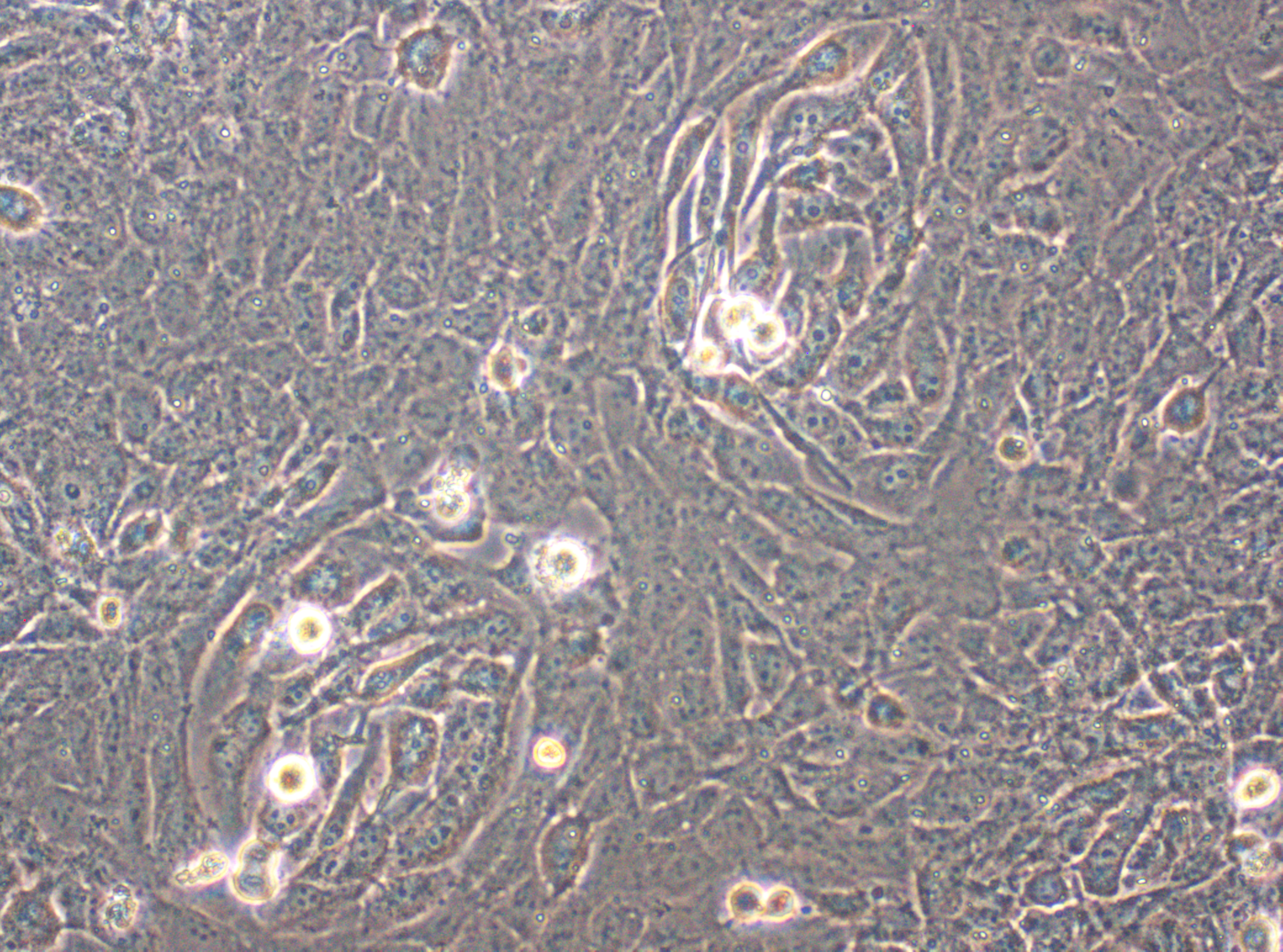

Supplement: Supplementary file 6 — Source data Fig. 2 [file 44319_2026_751_MOESM6_ESM.zip › Raw_data_Figure 2/Figure 2A/colculture UMSCC/USMcc - + Ki 2 ZOOM.tif]

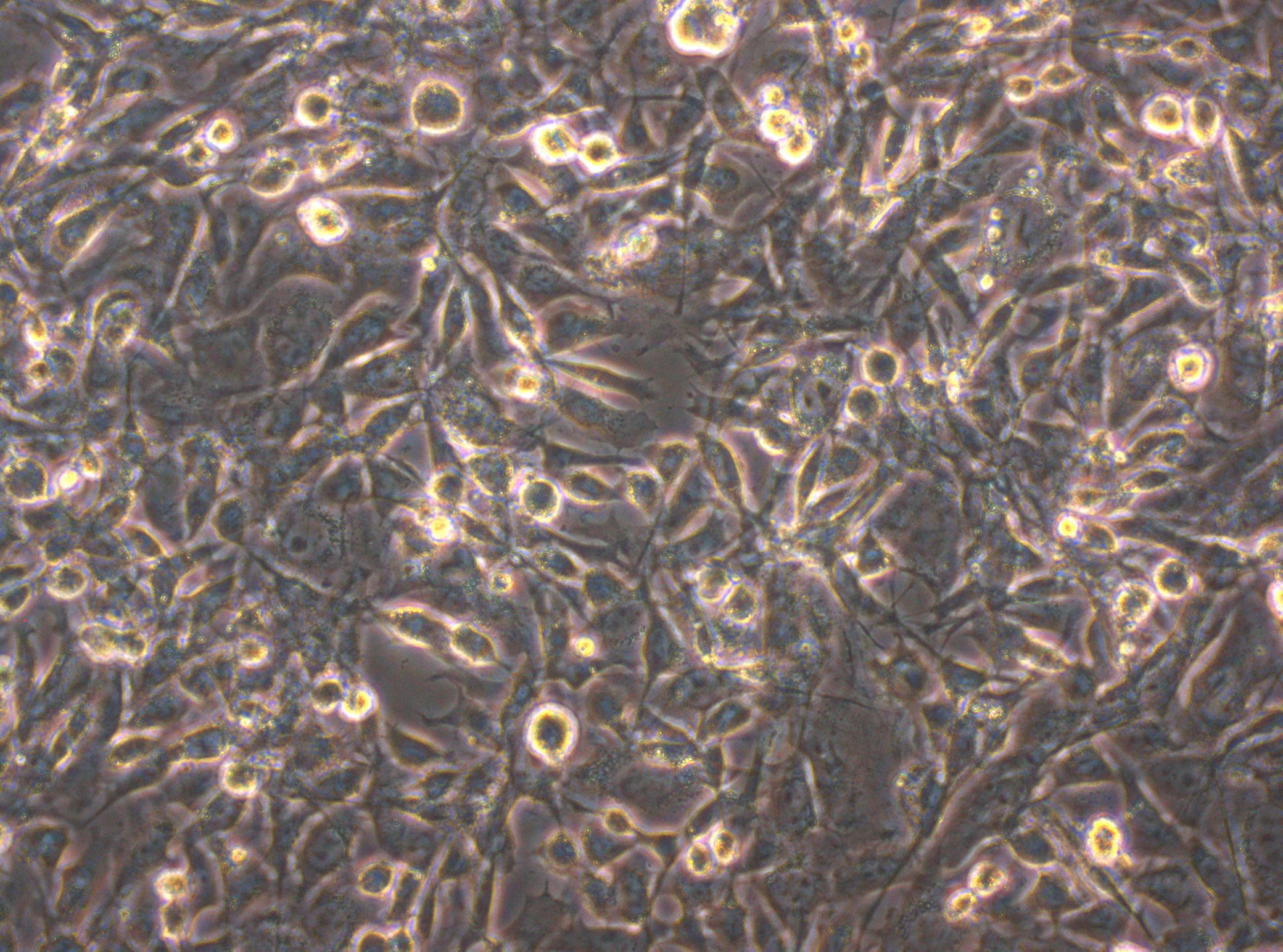

Supplement: Supplementary file 6 — Source data Fig. 2 [file 44319_2026_751_MOESM6_ESM.zip › Raw_data_Figure 2/Figure 2A/colculture UMSCC/USMcc - + KO ZOOM.tif]

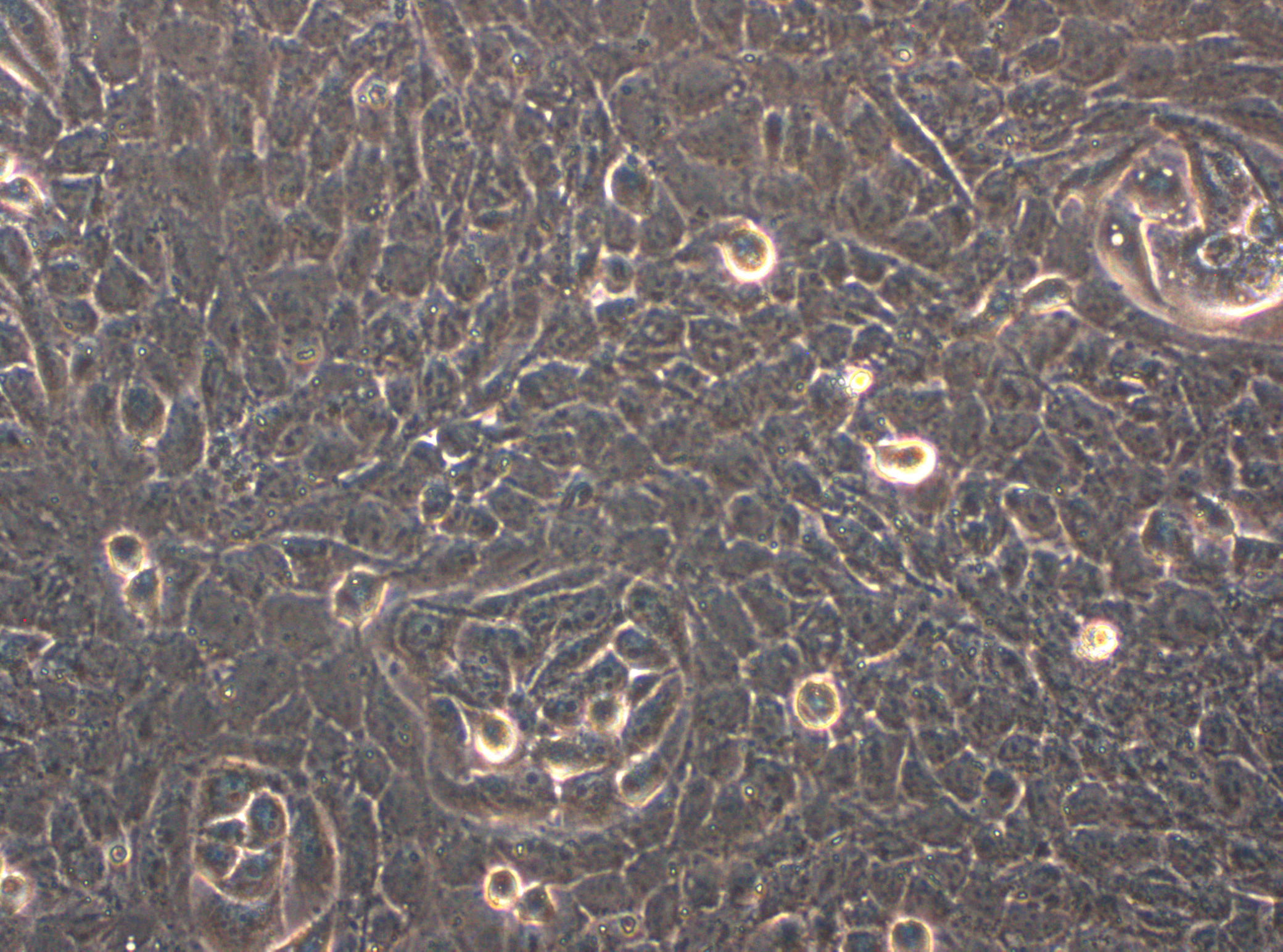

Supplement: Supplementary file 6 — Source data Fig. 2 [file 44319_2026_751_MOESM6_ESM.zip › Raw_data_Figure 2/Figure 2A/colculture UMSCC/USMcc - + wt ZOOM.tif]

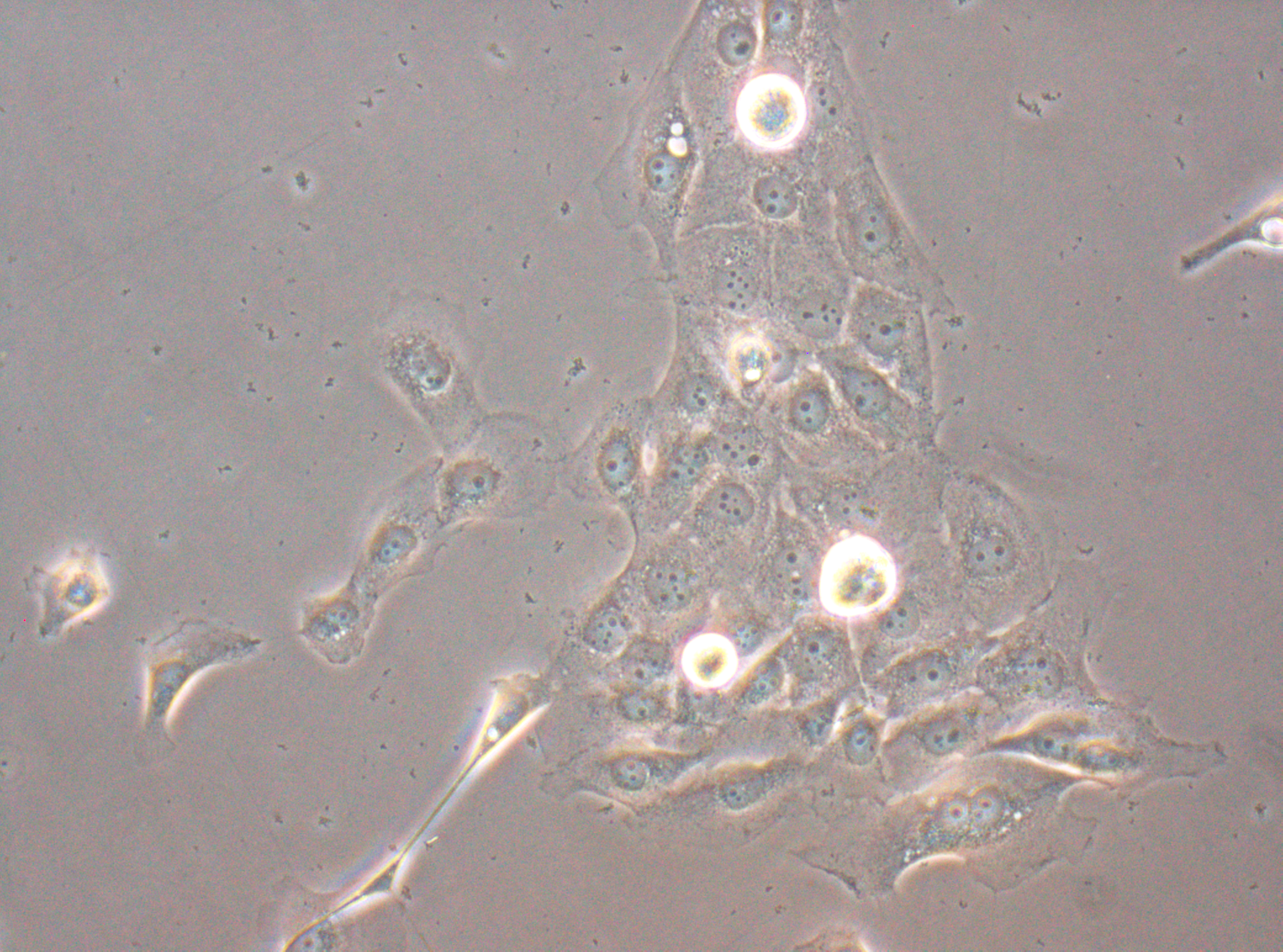

Supplement: Supplementary file 6 — Source data Fig. 2 [file 44319_2026_751_MOESM6_ESM.zip › Raw_data_Figure 2/Figure 2A/colculture UMSCC/USMCC - ZOOM.tif]

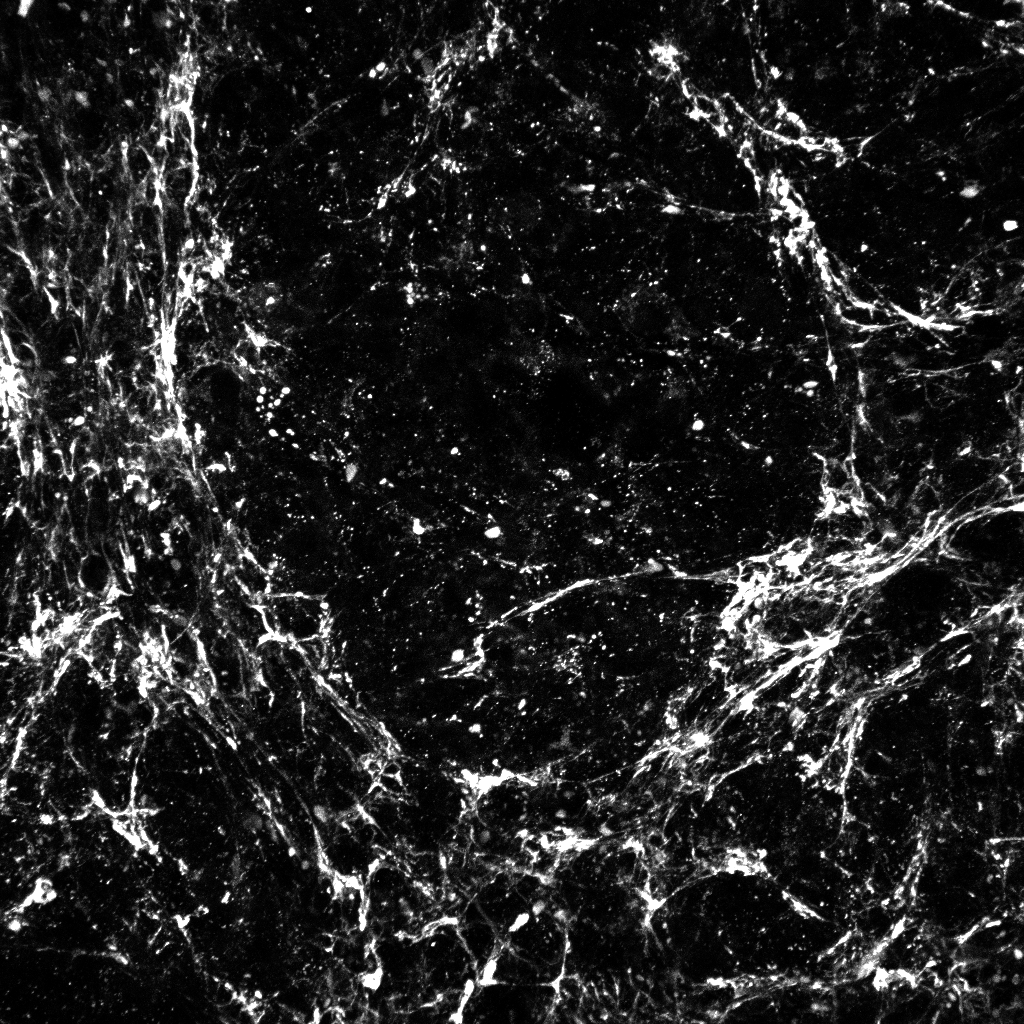

Supplement: Supplementary file 6 — Source data Fig. 2 [file 44319_2026_751_MOESM6_ESM.zip › Raw_data_Figure 2/Figure 2B/Confocal microscopy Col I/Cal27 + fibros KO col I.tif]

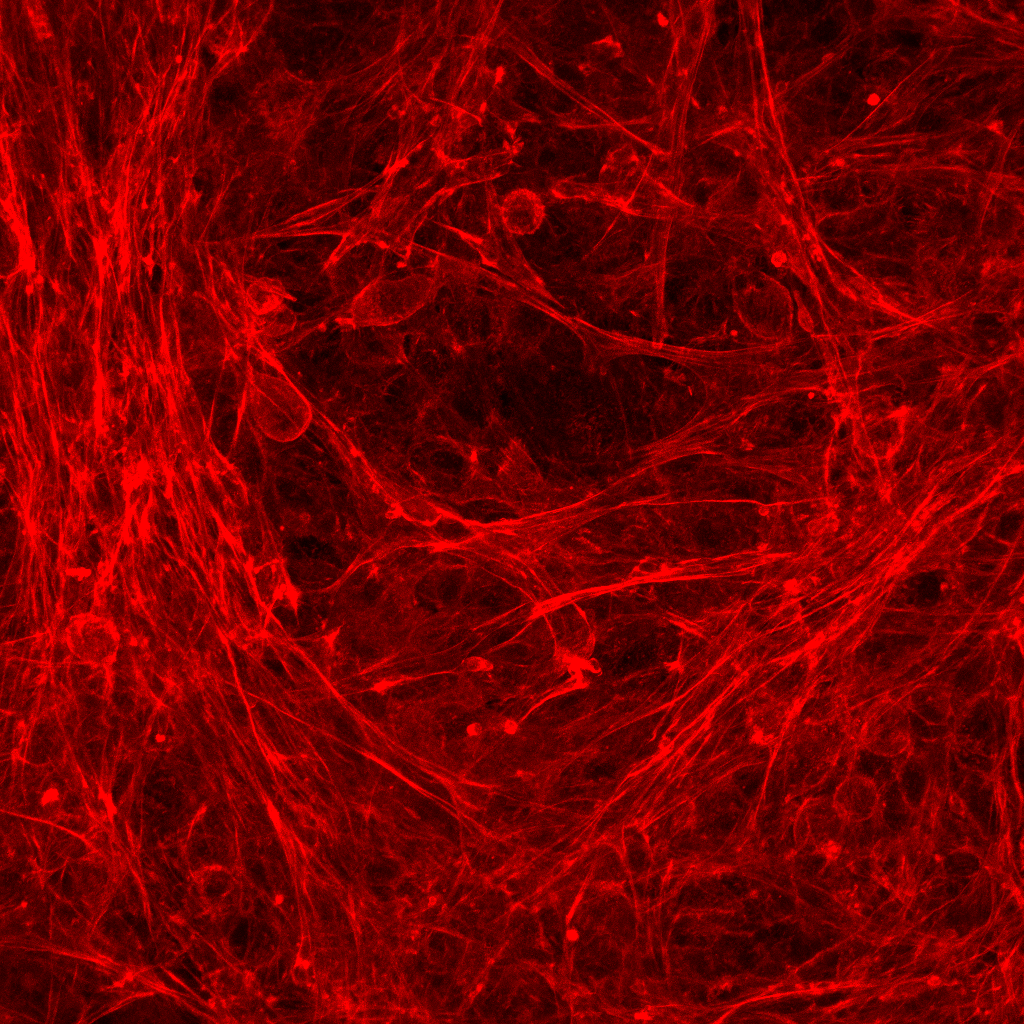

Supplement: Supplementary file 6 — Source data Fig. 2 [file 44319_2026_751_MOESM6_ESM.zip › Raw_data_Figure 2/Figure 2B/Confocal microscopy Col I/Cal27 + fibros KO Factin.tif]

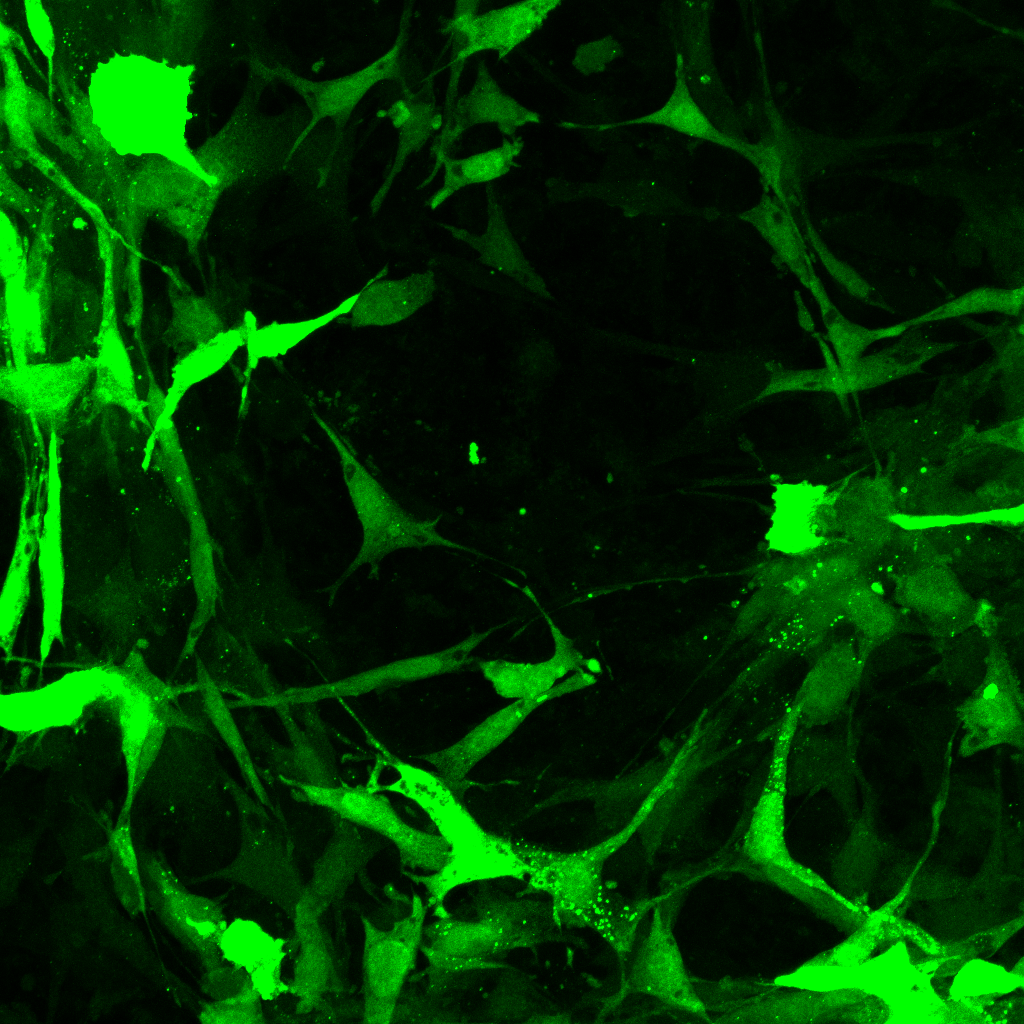

Supplement: Supplementary file 6 — Source data Fig. 2 [file 44319_2026_751_MOESM6_ESM.zip › Raw_data_Figure 2/Figure 2B/Confocal microscopy Col I/Cal27 + fibros KO green.tif]

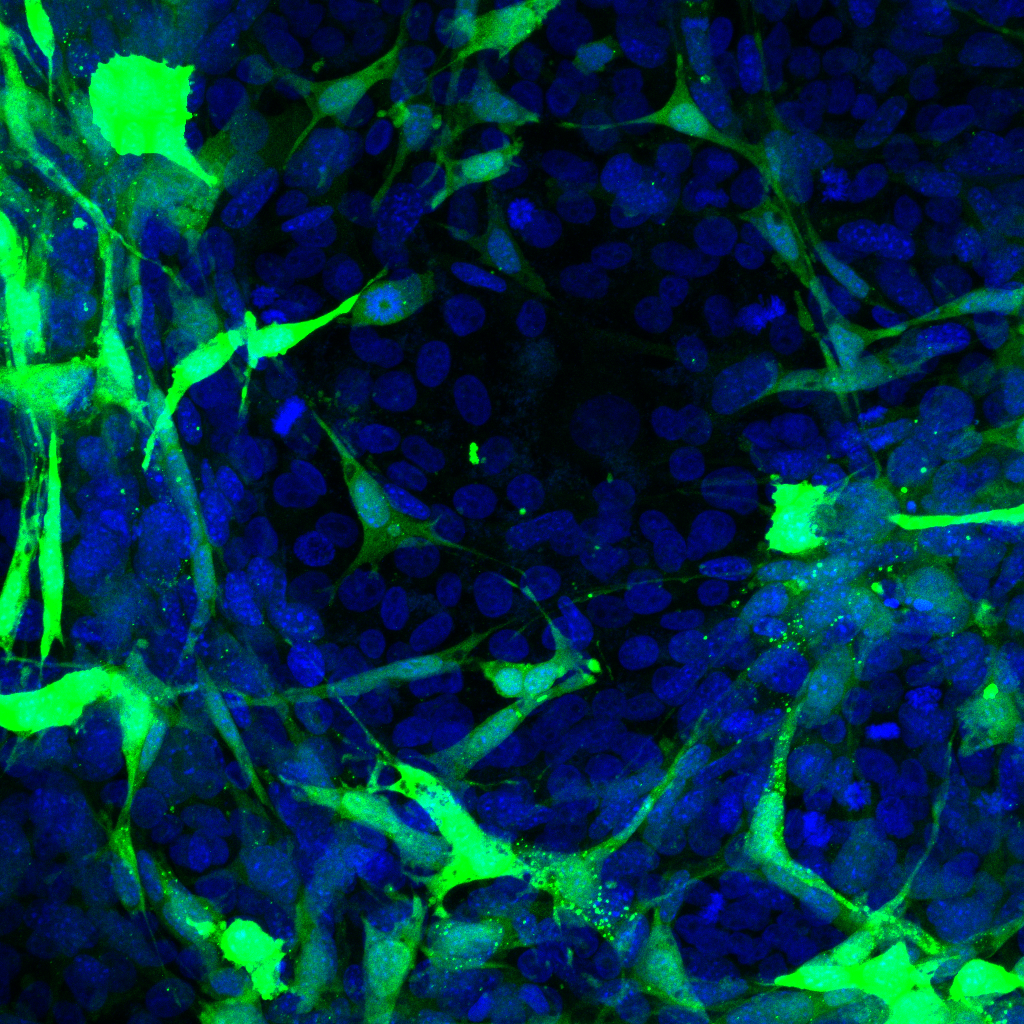

Supplement: Supplementary file 6 — Source data Fig. 2 [file 44319_2026_751_MOESM6_ESM.zip › Raw_data_Figure 2/Figure 2B/Confocal microscopy Col I/Cal27 + fibros KO merge.tif]

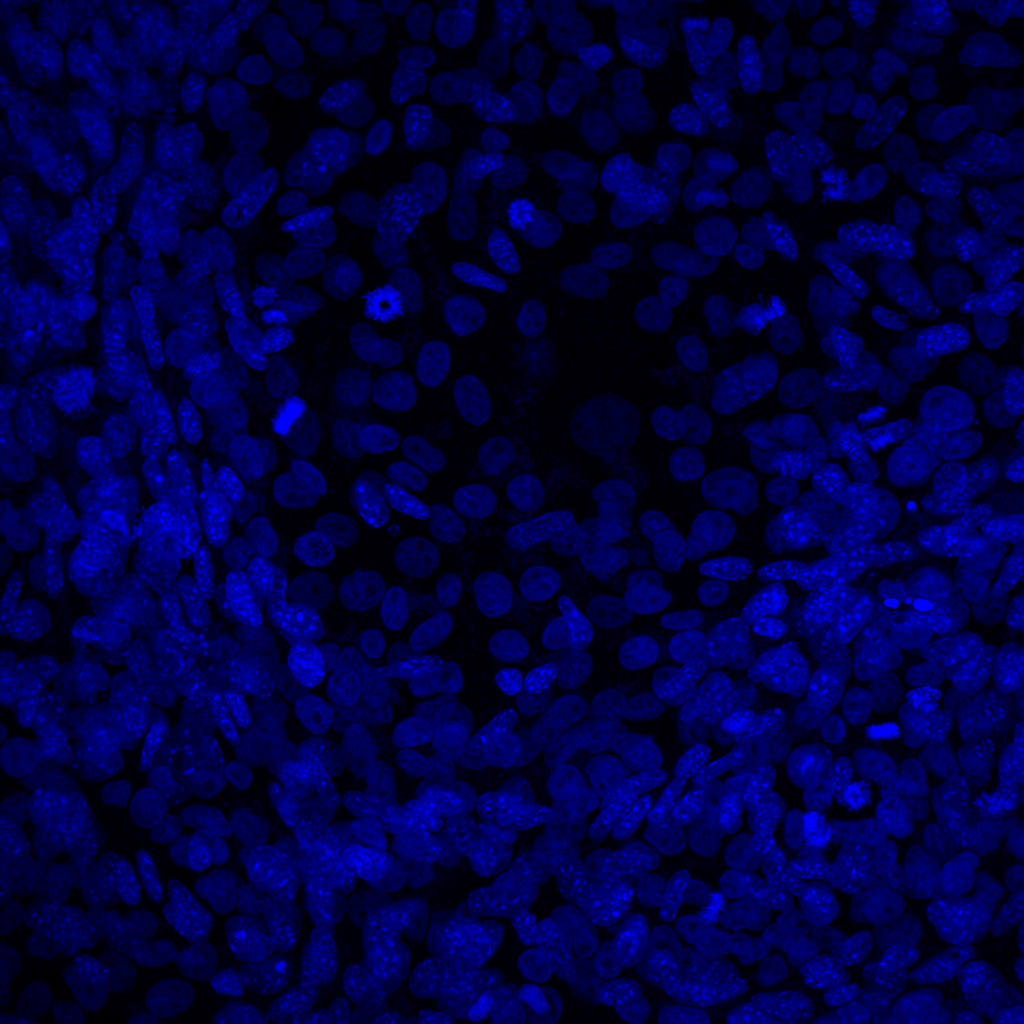

Supplement: Supplementary file 6 — Source data Fig. 2 [file 44319_2026_751_MOESM6_ESM.zip › Raw_data_Figure 2/Figure 2B/Confocal microscopy Col I/Cal27 + fibros KO nuclei.tif]

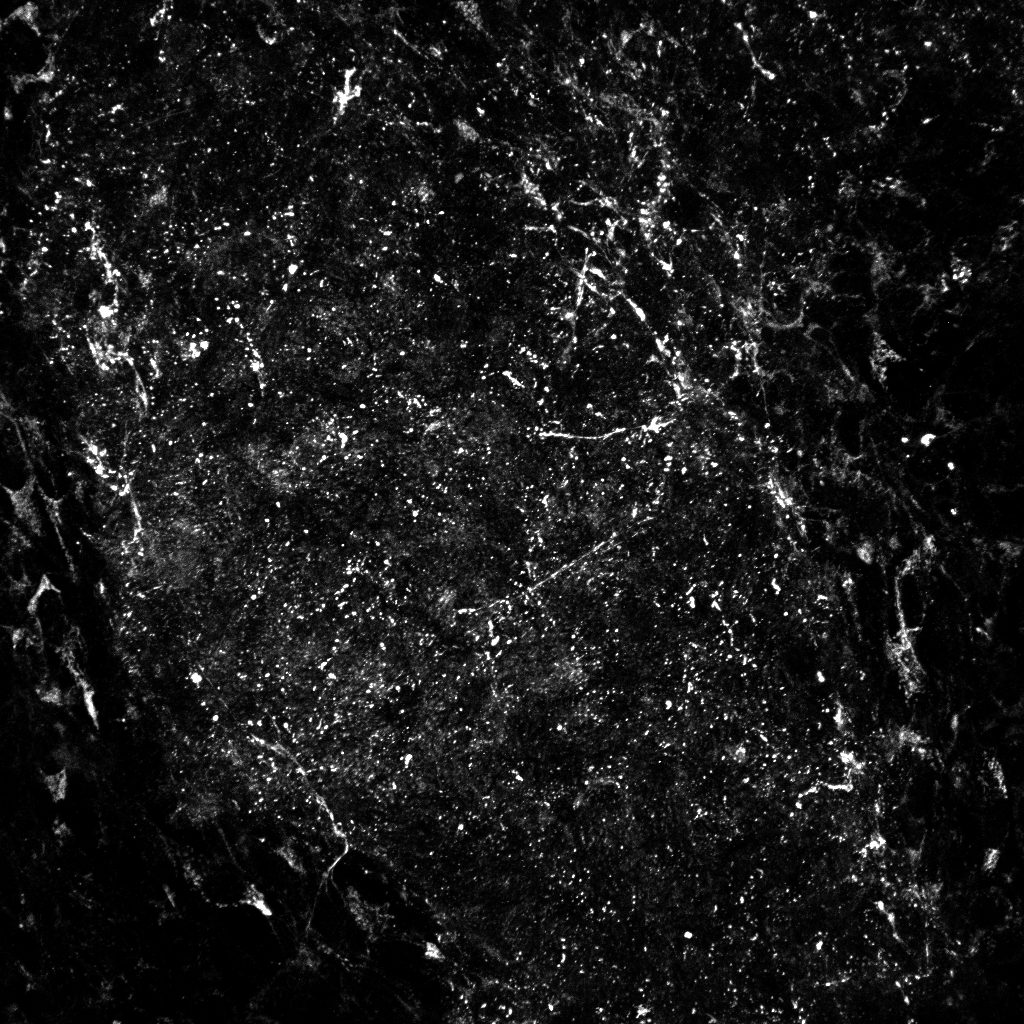

Supplement: Supplementary file 6 — Source data Fig. 2 [file 44319_2026_751_MOESM6_ESM.zip › Raw_data_Figure 2/Figure 2B/Confocal microscopy Col I/Cal27 + fibros WT col I.tif]

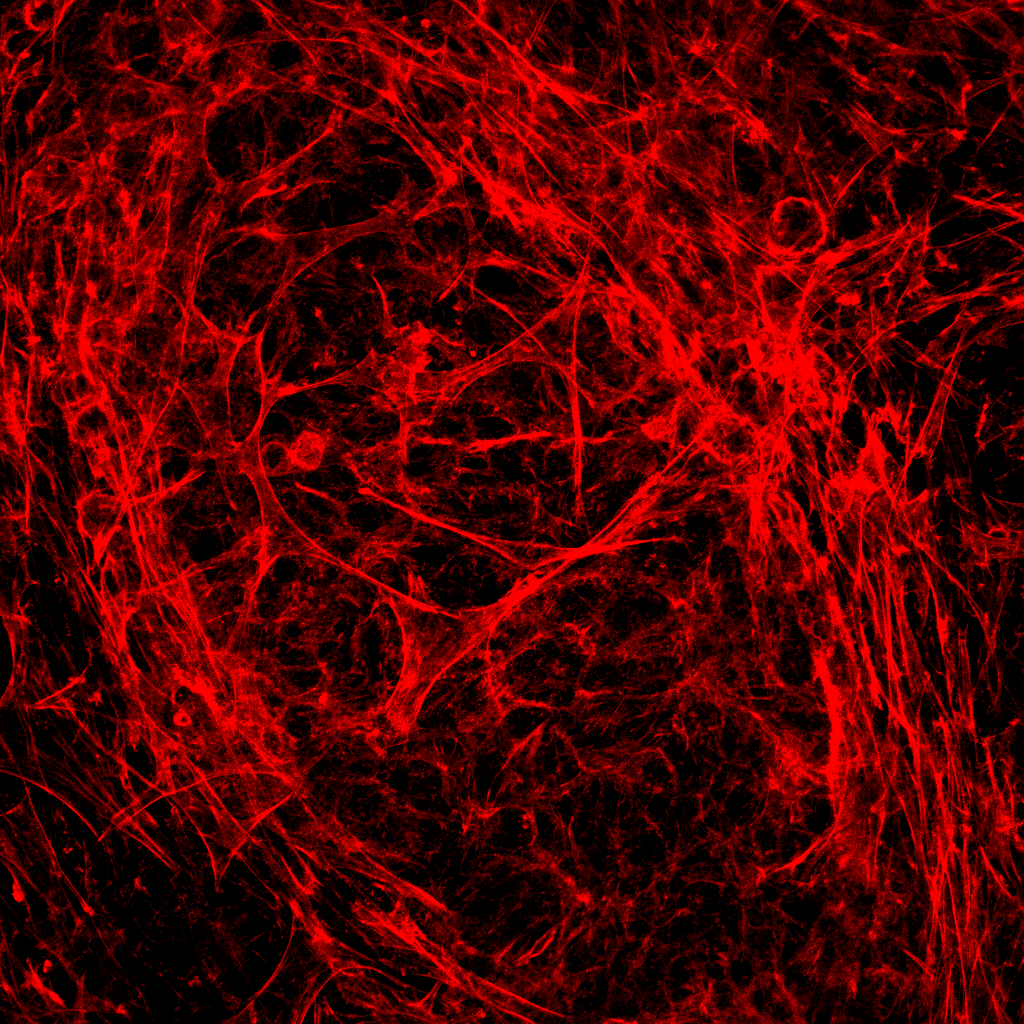

Supplement: Supplementary file 6 — Source data Fig. 2 [file 44319_2026_751_MOESM6_ESM.zip › Raw_data_Figure 2/Figure 2B/Confocal microscopy Col I/Cal27 + fibros WT Factin.tif]

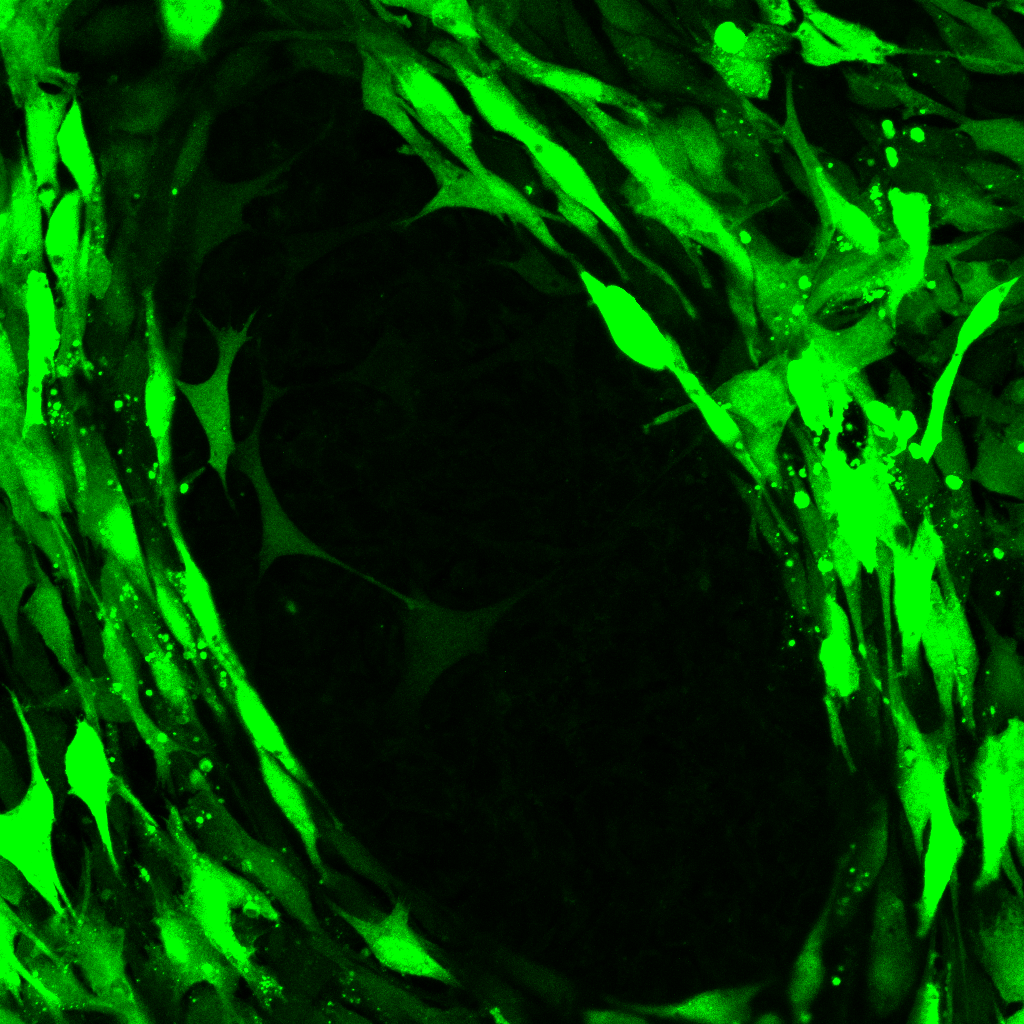

Supplement: Supplementary file 6 — Source data Fig. 2 [file 44319_2026_751_MOESM6_ESM.zip › Raw_data_Figure 2/Figure 2B/Confocal microscopy Col I/Cal27 + fibros WT green.tif]

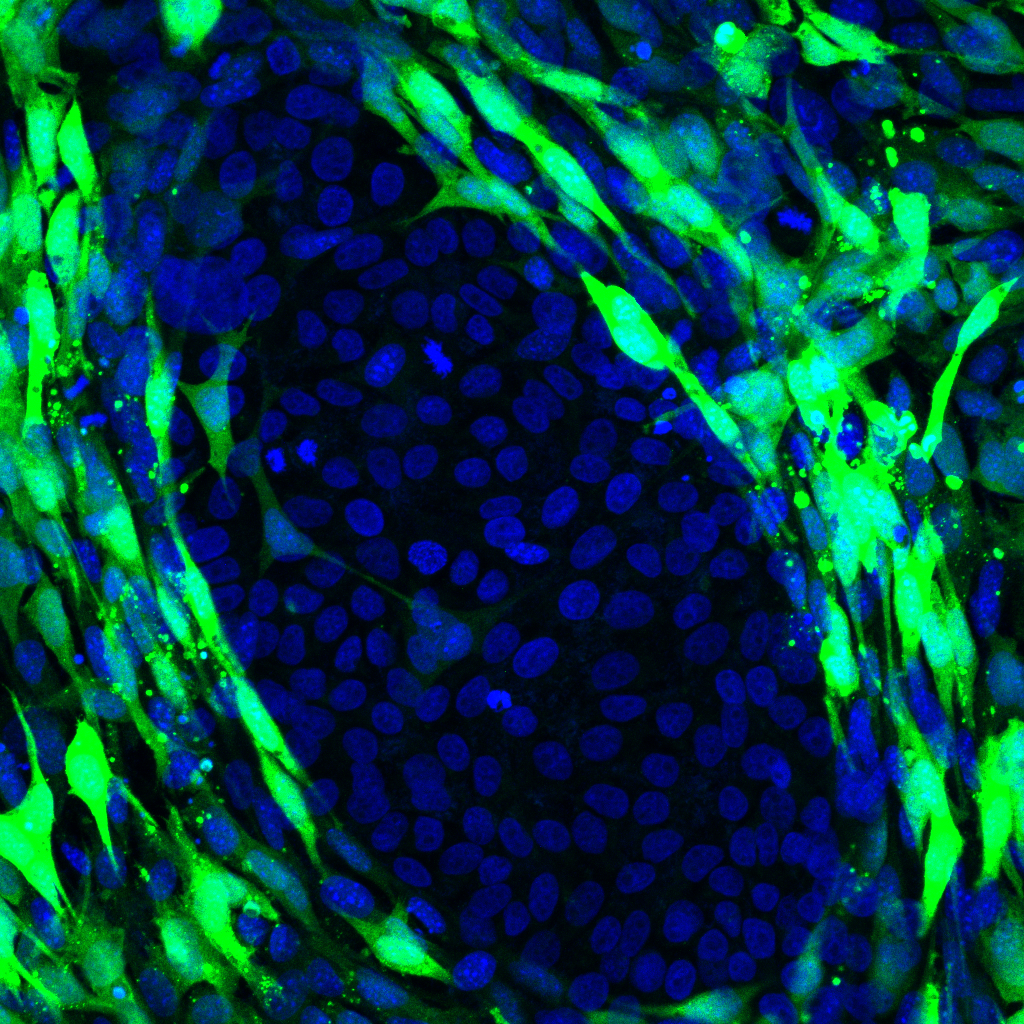

Supplement: Supplementary file 6 — Source data Fig. 2 [file 44319_2026_751_MOESM6_ESM.zip › Raw_data_Figure 2/Figure 2B/Confocal microscopy Col I/Cal27 + fibros WT merge.tif]

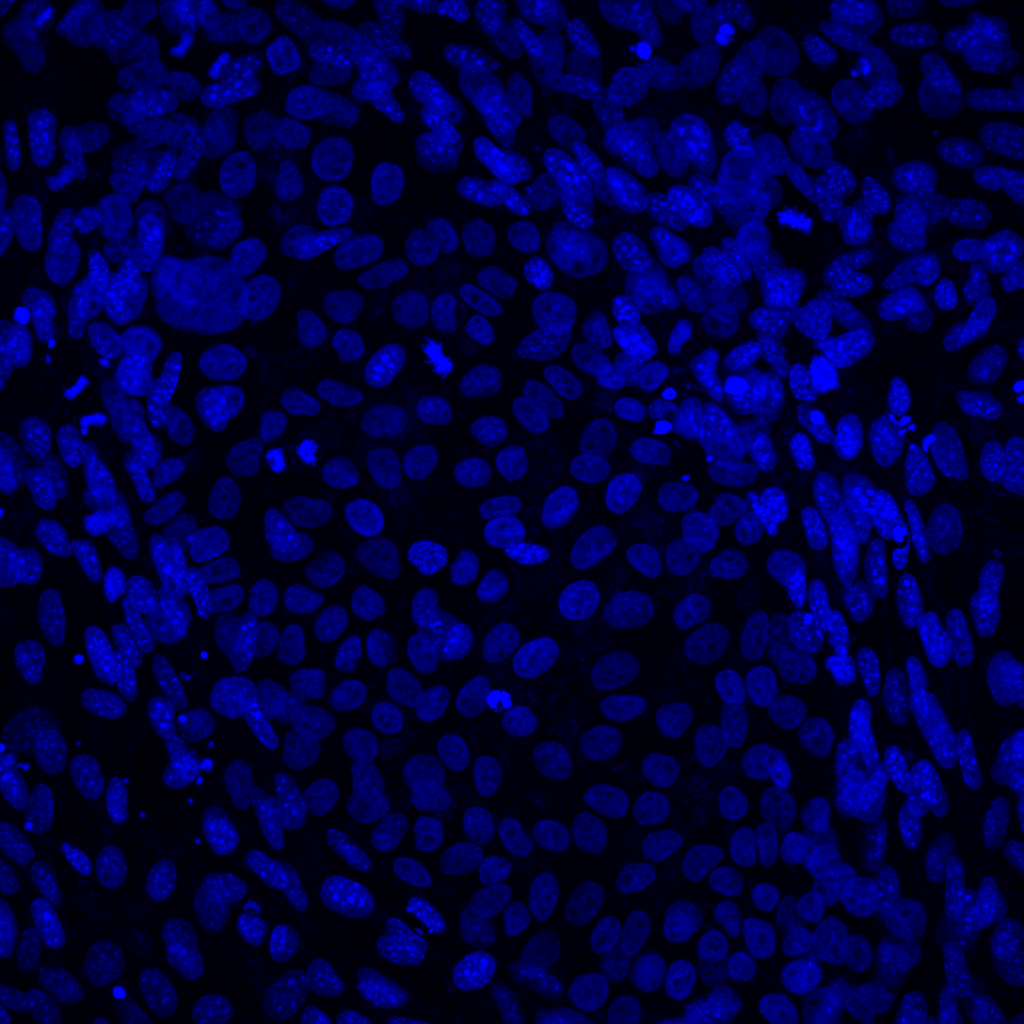

Supplement: Supplementary file 6 — Source data Fig. 2 [file 44319_2026_751_MOESM6_ESM.zip › Raw_data_Figure 2/Figure 2B/Confocal microscopy Col I/Cal27 + fibros WT nuclei.tif]

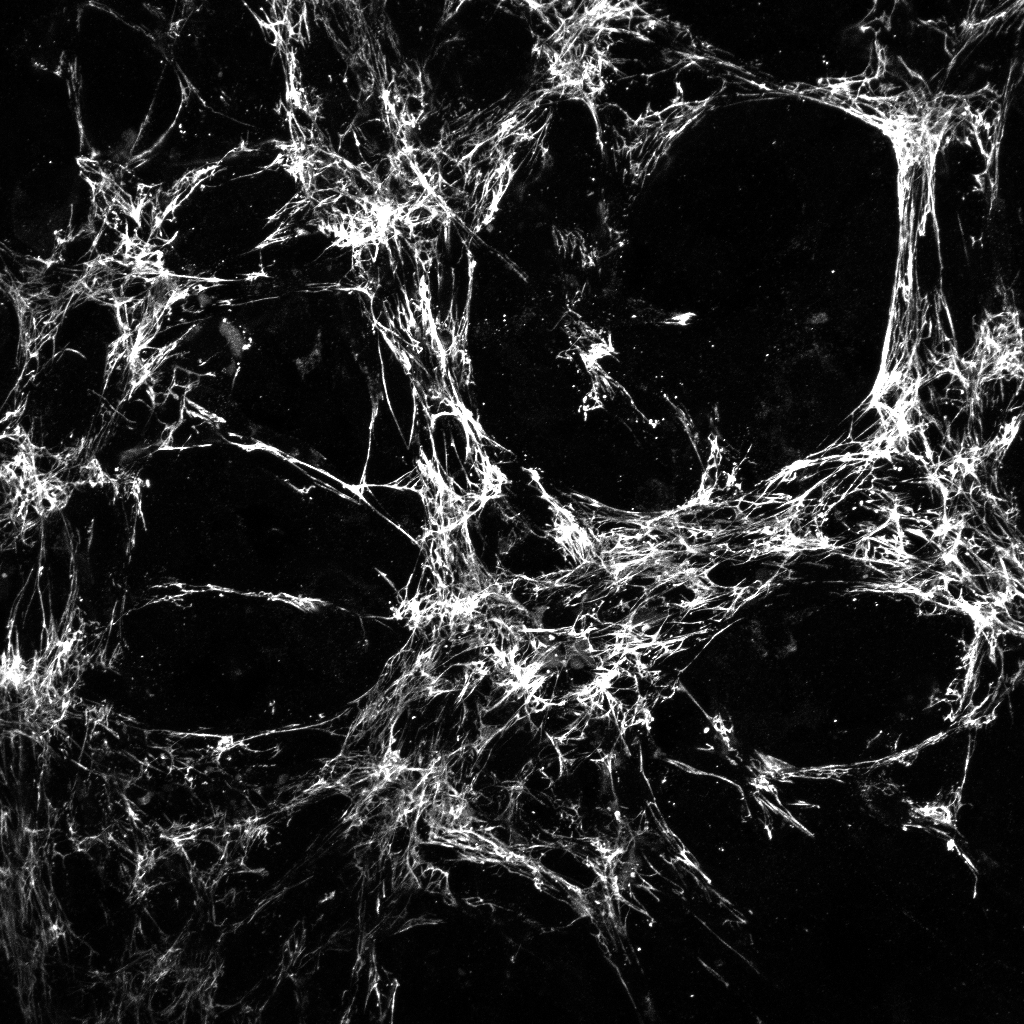

Supplement: Supplementary file 6 — Source data Fig. 2 [file 44319_2026_751_MOESM6_ESM.zip › Raw_data_Figure 2/Figure 2B/Confocal microscopy FN/FN cal 27 +GqKO.tif]

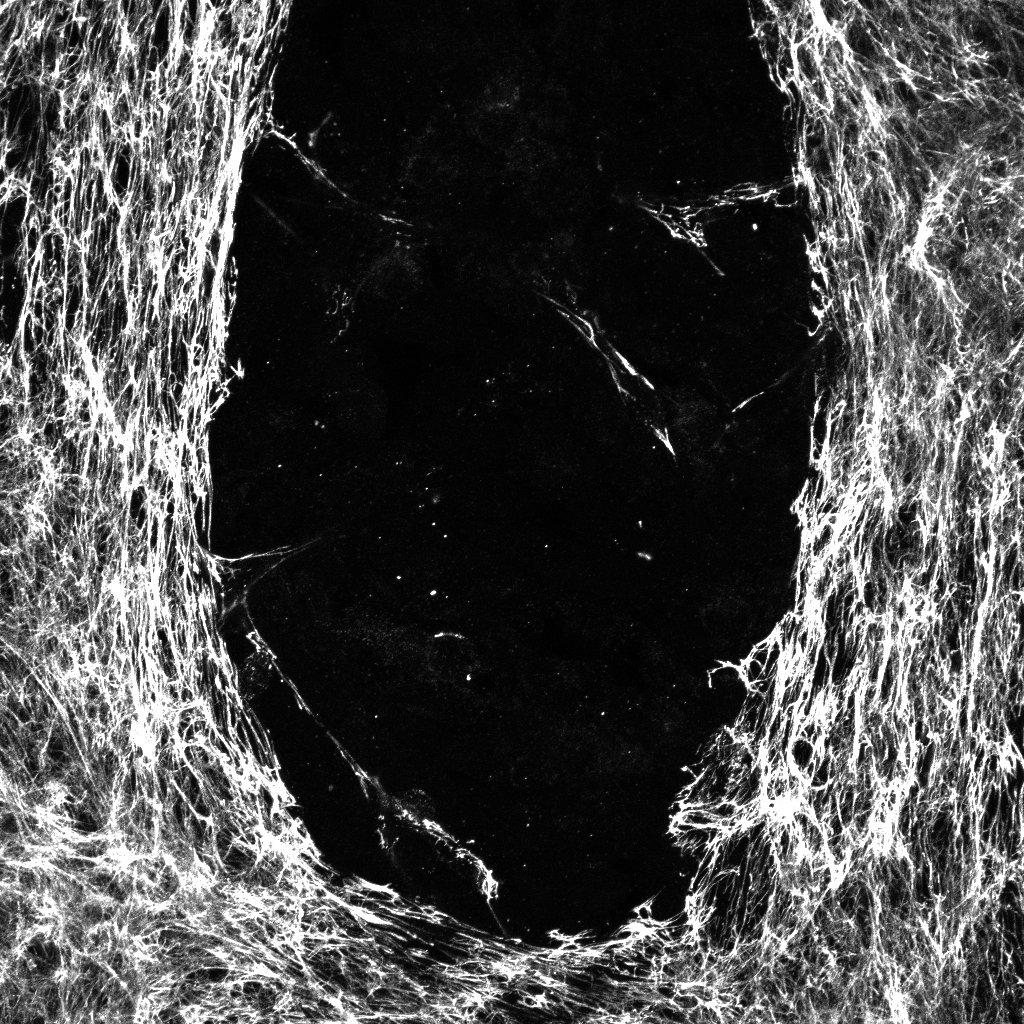

Supplement: Supplementary file 6 — Source data Fig. 2 [file 44319_2026_751_MOESM6_ESM.zip › Raw_data_Figure 2/Figure 2B/Confocal microscopy FN/FN cal27 +WT.jpg]

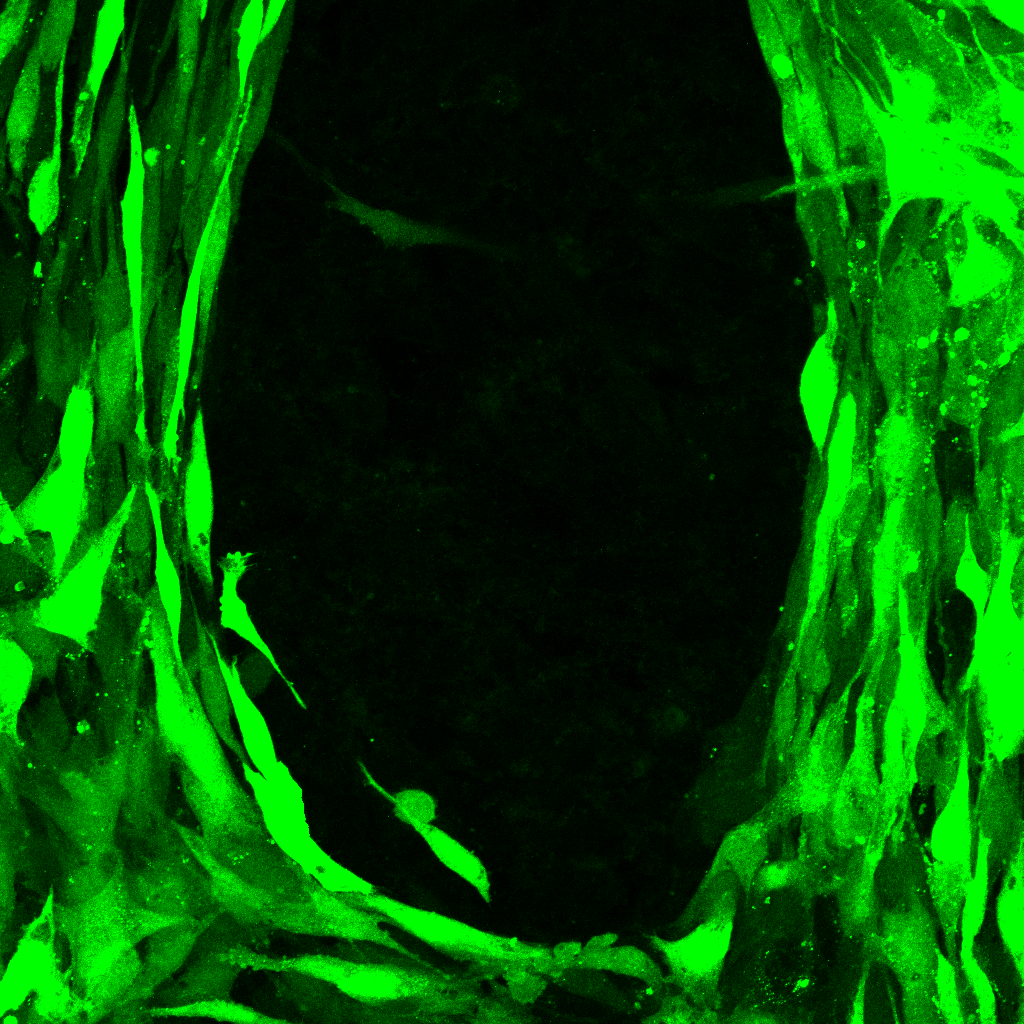

Supplement: Supplementary file 6 — Source data Fig. 2 [file 44319_2026_751_MOESM6_ESM.zip › Raw_data_Figure 2/Figure 2B/Confocal microscopy FN/GFP cal27 +WT.tif]

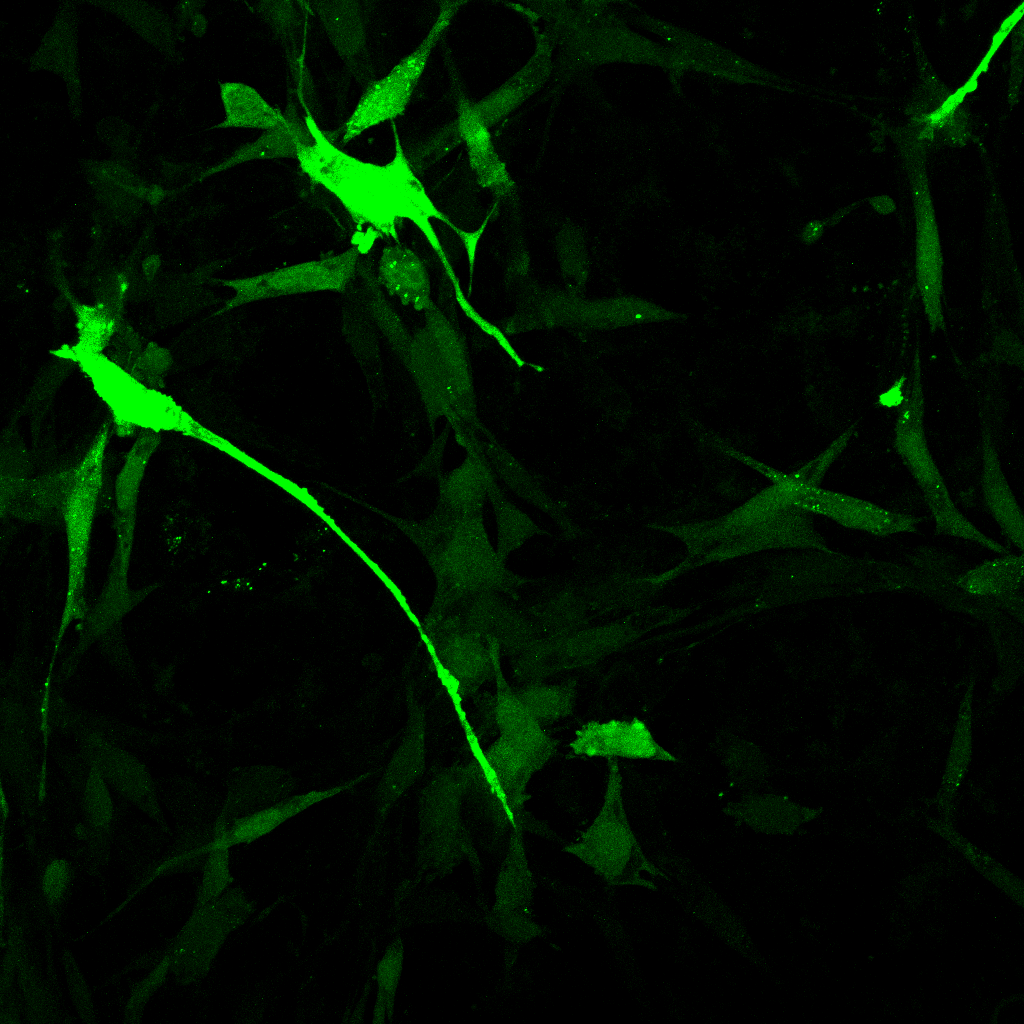

Supplement: Supplementary file 6 — Source data Fig. 2 [file 44319_2026_751_MOESM6_ESM.zip › Raw_data_Figure 2/Figure 2B/Confocal microscopy FN/GFP cal27+GqKO.tif]

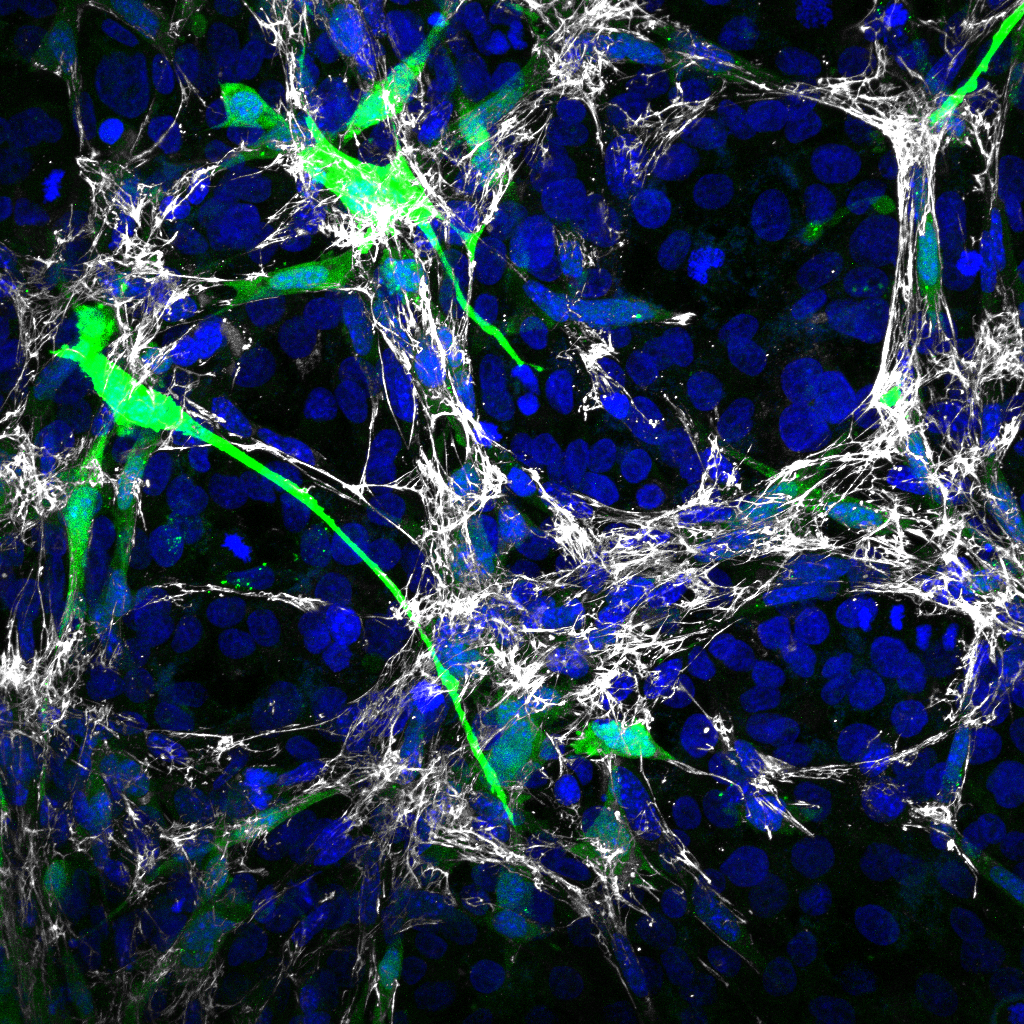

Supplement: Supplementary file 6 — Source data Fig. 2 [file 44319_2026_751_MOESM6_ESM.zip › Raw_data_Figure 2/Figure 2B/Confocal microscopy FN/merge GqKO all chanelstif.tif]

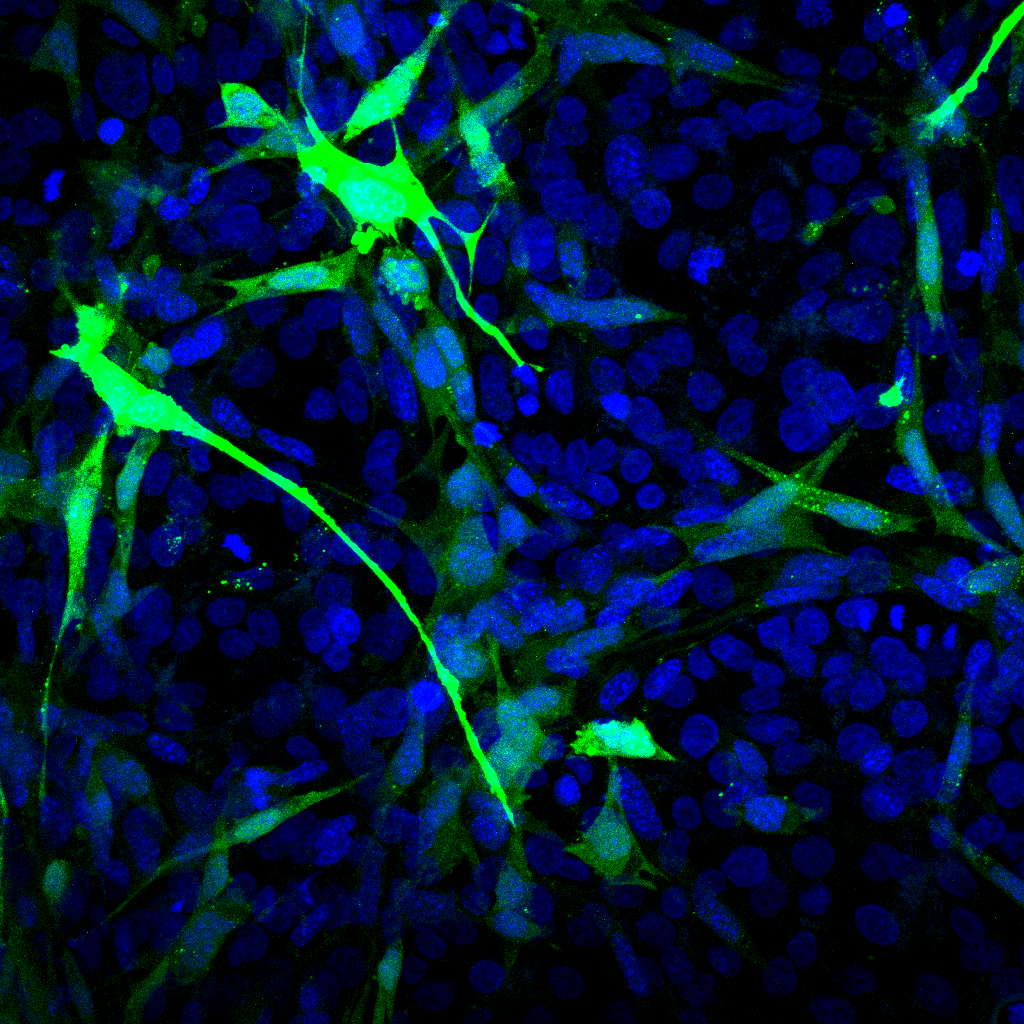

Supplement: Supplementary file 6 — Source data Fig. 2 [file 44319_2026_751_MOESM6_ESM.zip › Raw_data_Figure 2/Figure 2B/Confocal microscopy FN/merge GqKO.tif]

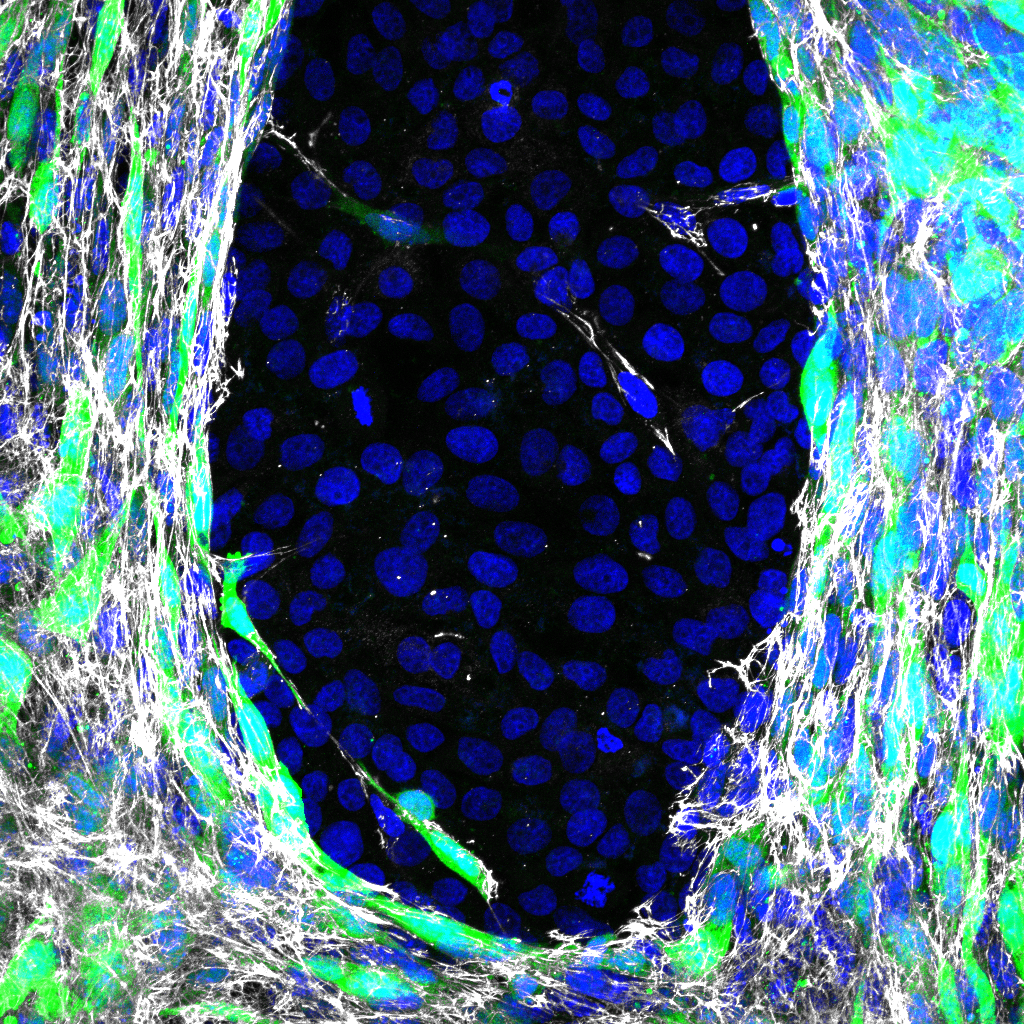

Supplement: Supplementary file 6 — Source data Fig. 2 [file 44319_2026_751_MOESM6_ESM.zip › Raw_data_Figure 2/Figure 2B/Confocal microscopy FN/merge WT all chanelstif.tif]

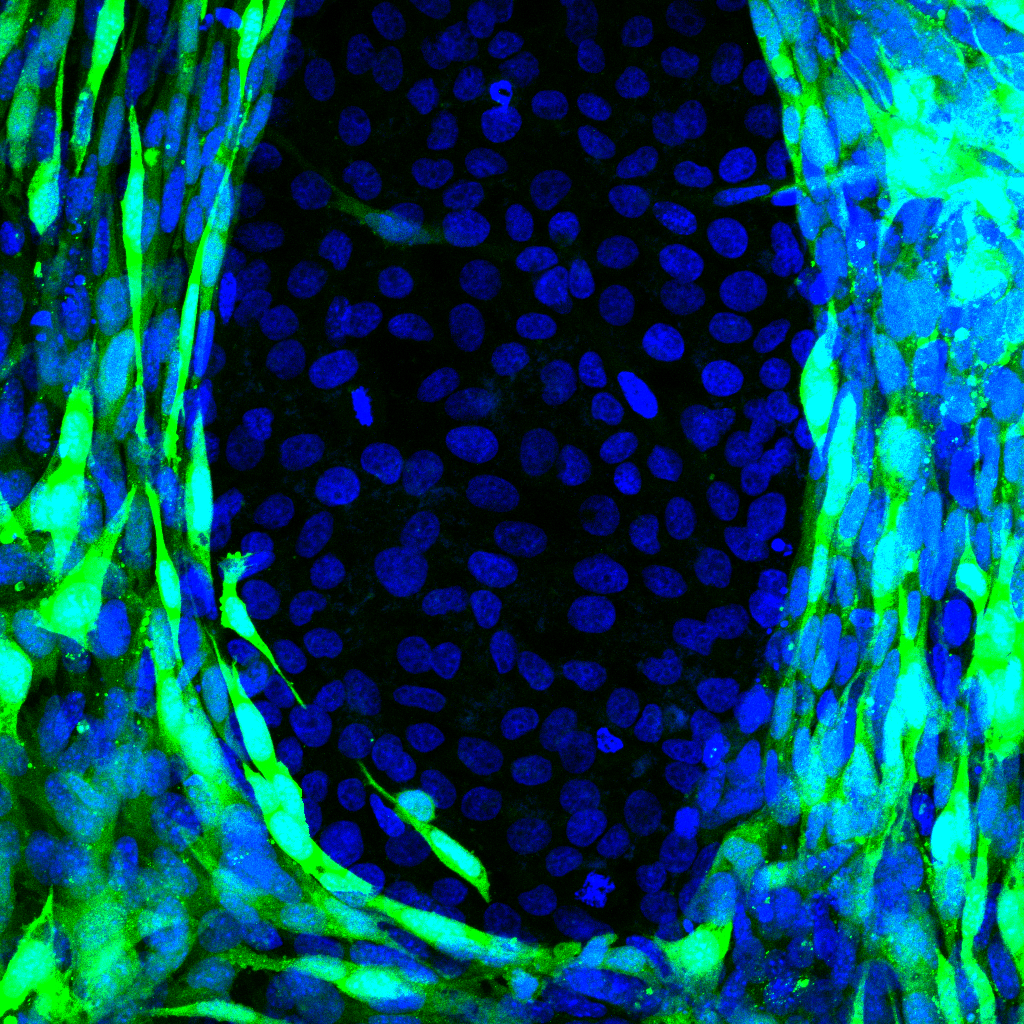

Supplement: Supplementary file 6 — Source data Fig. 2 [file 44319_2026_751_MOESM6_ESM.zip › Raw_data_Figure 2/Figure 2B/Confocal microscopy FN/merge WT.tif]

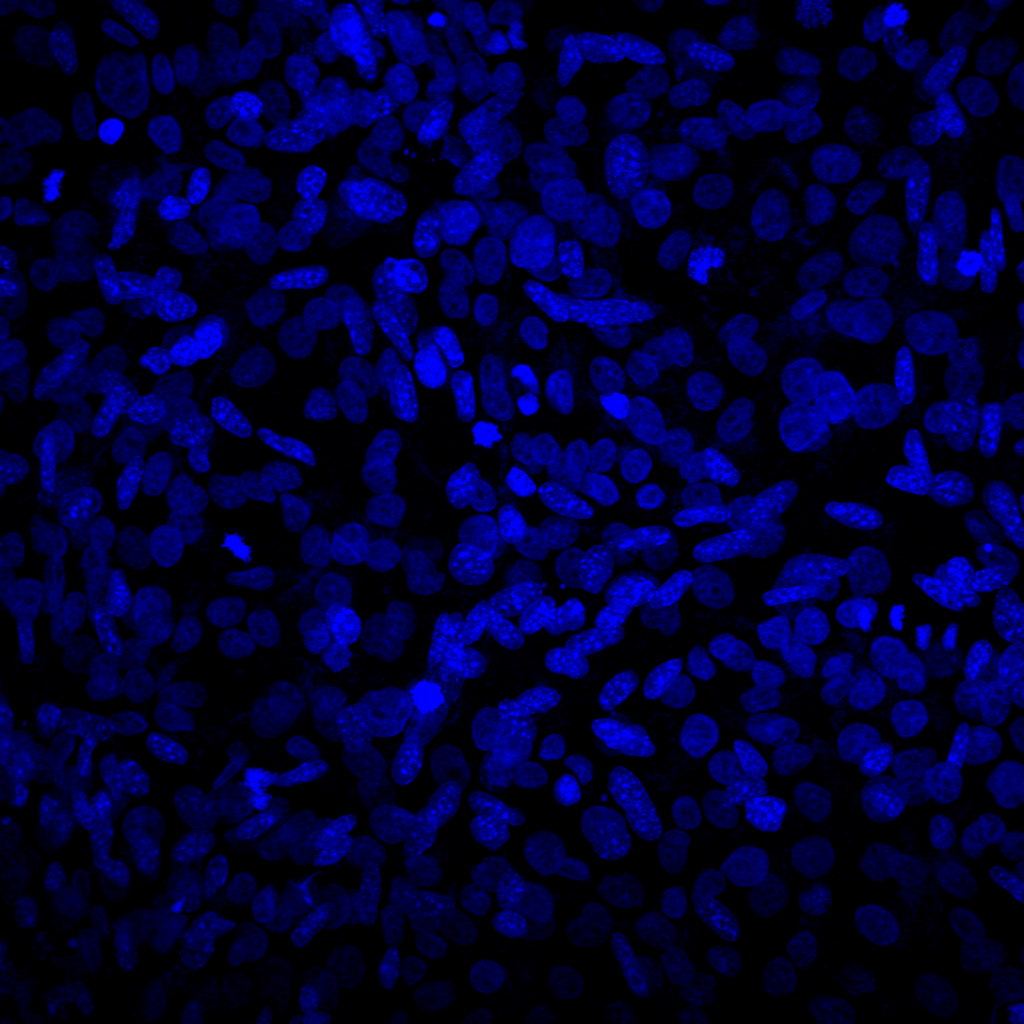

Supplement: Supplementary file 6 — Source data Fig. 2 [file 44319_2026_751_MOESM6_ESM.zip › Raw_data_Figure 2/Figure 2B/Confocal microscopy FN/nuclei cal27 +GqKO.tif]

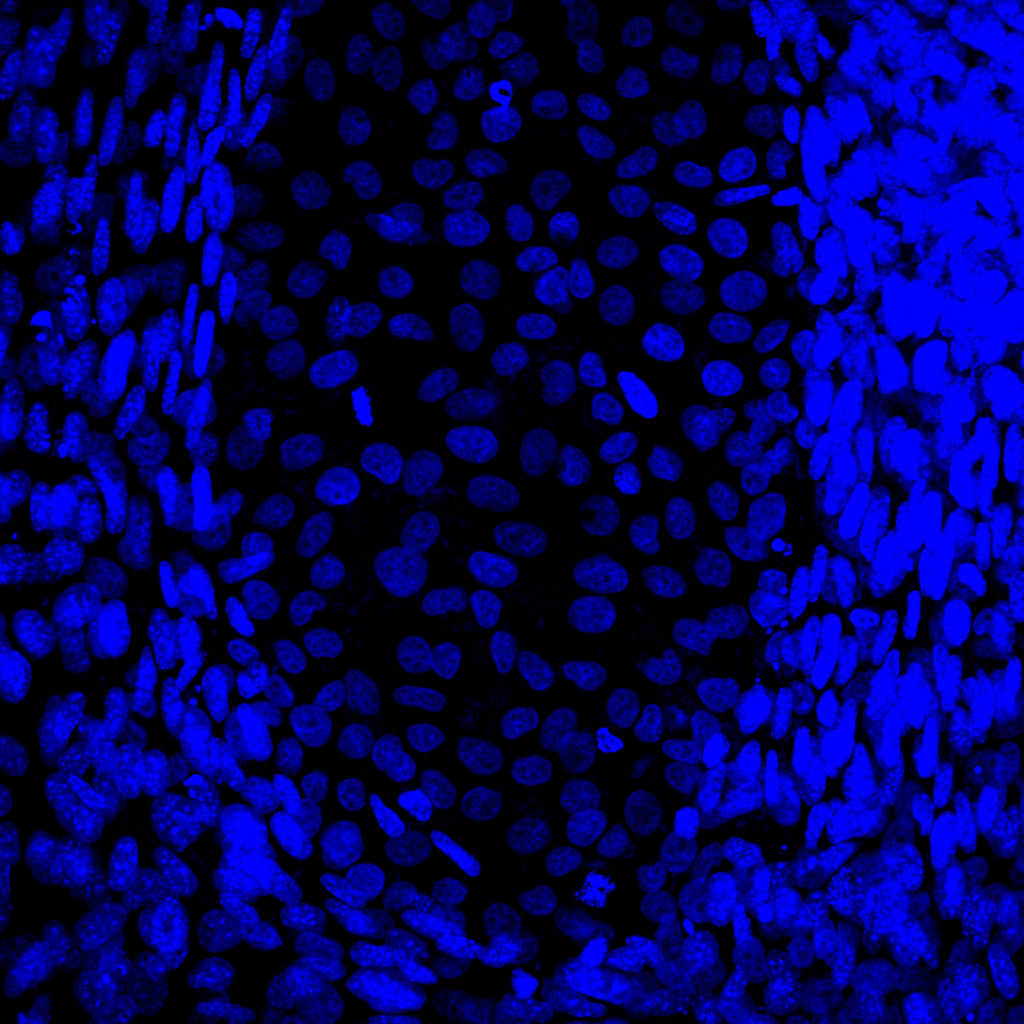

Supplement: Supplementary file 6 — Source data Fig. 2 [file 44319_2026_751_MOESM6_ESM.zip › Raw_data_Figure 2/Figure 2B/Confocal microscopy FN/nuclei cal27 +WT.tif]

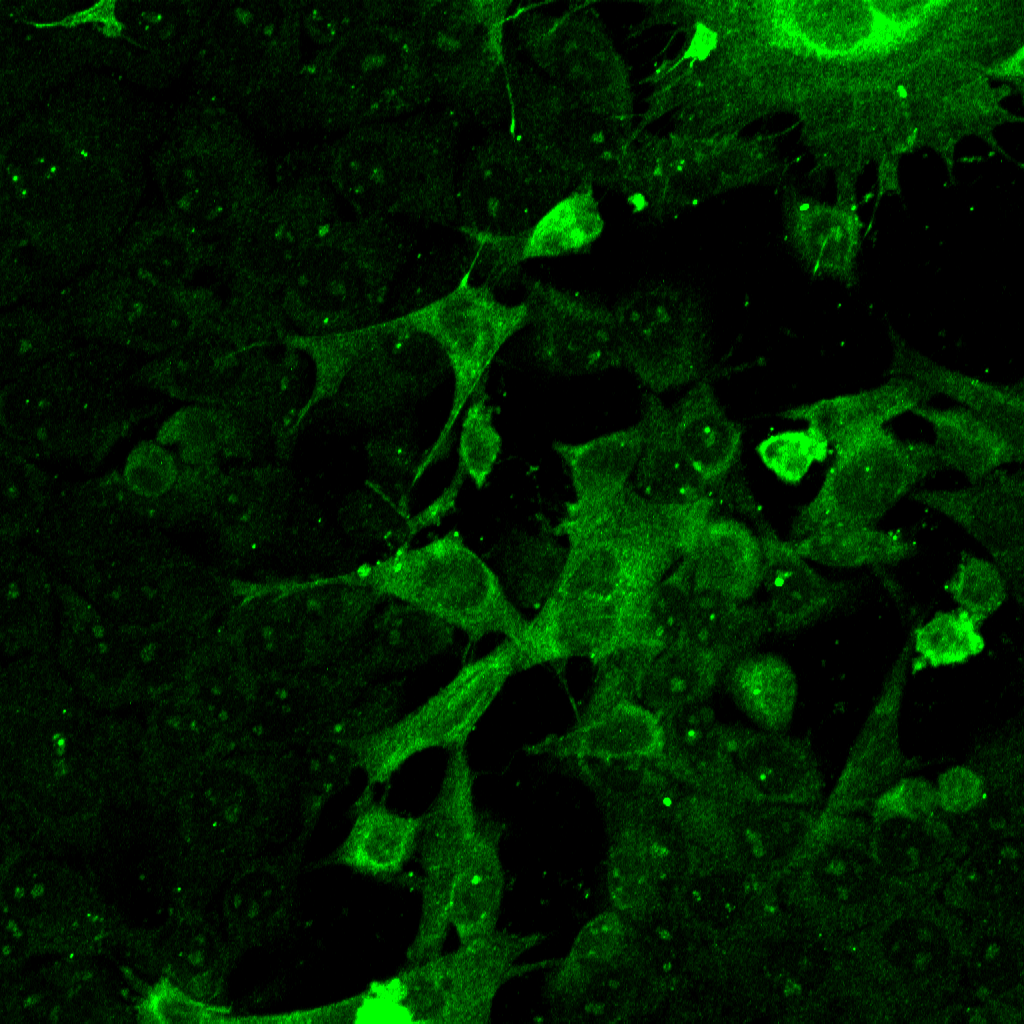

Supplement: Supplementary file 6 — Source data Fig. 2 [file 44319_2026_751_MOESM6_ESM.zip › Raw_data_Figure 2/Figure 2C/Cal 27 + fibros KO SMA.tif]

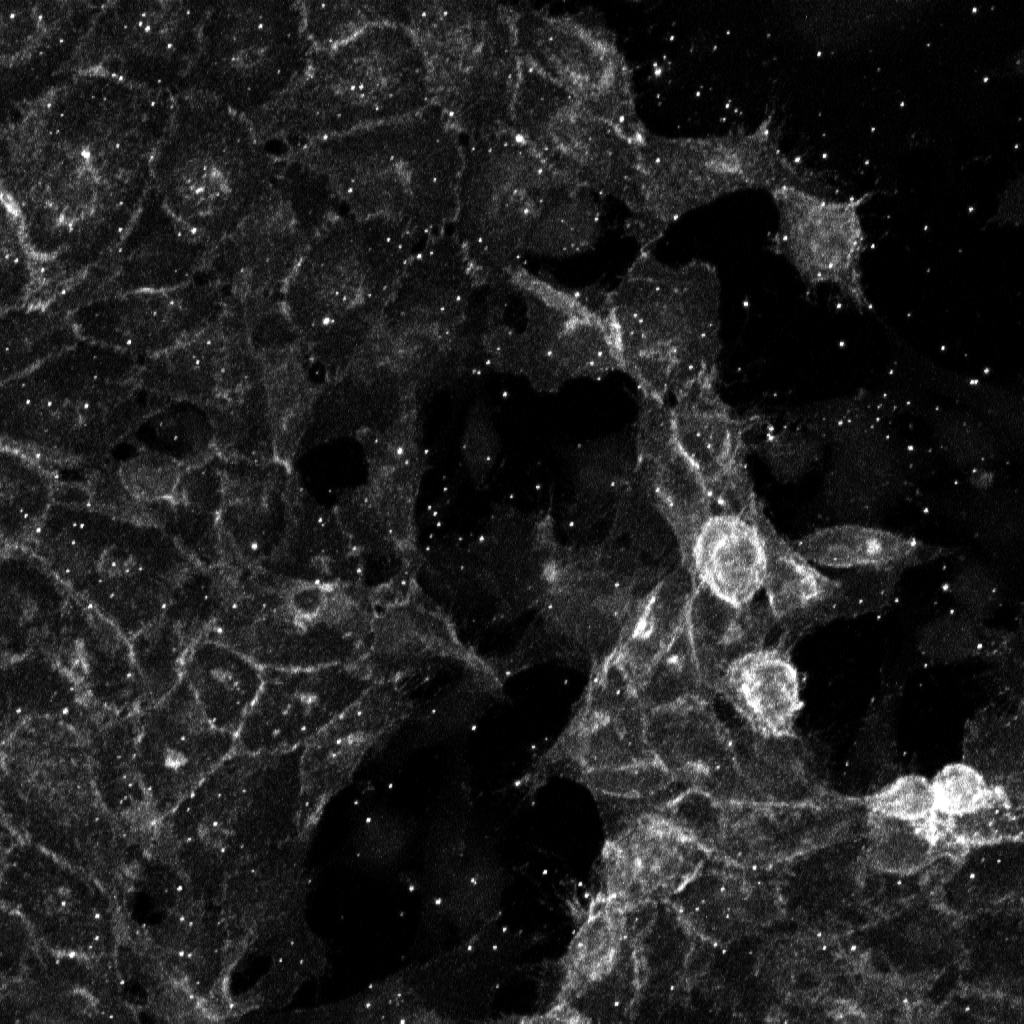

Supplement: Supplementary file 6 — Source data Fig. 2 [file 44319_2026_751_MOESM6_ESM.zip › Raw_data_Figure 2/Figure 2C/Cal 27+ fibros KO Ecad.tif]

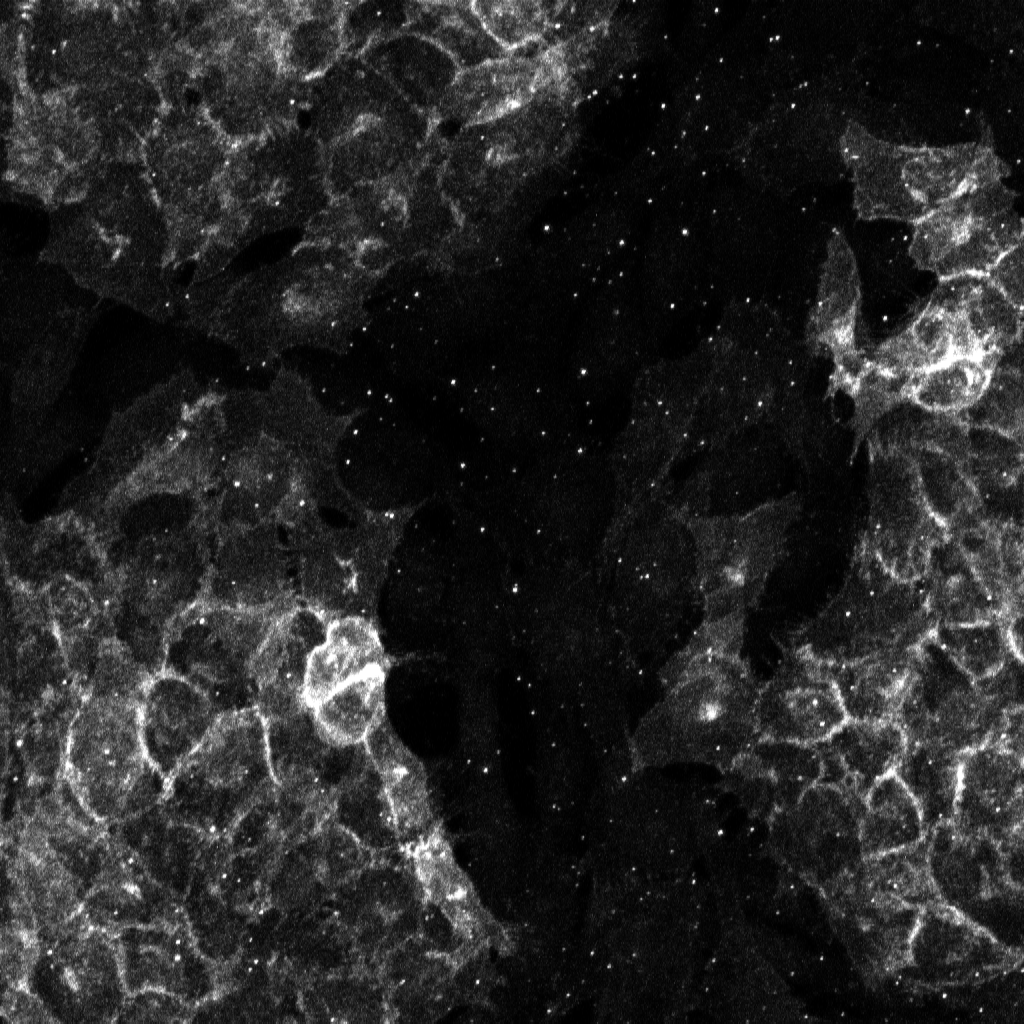

Supplement: Supplementary file 6 — Source data Fig. 2 [file 44319_2026_751_MOESM6_ESM.zip › Raw_data_Figure 2/Figure 2C/Cal 27+ fibros WT Ecad.tif]

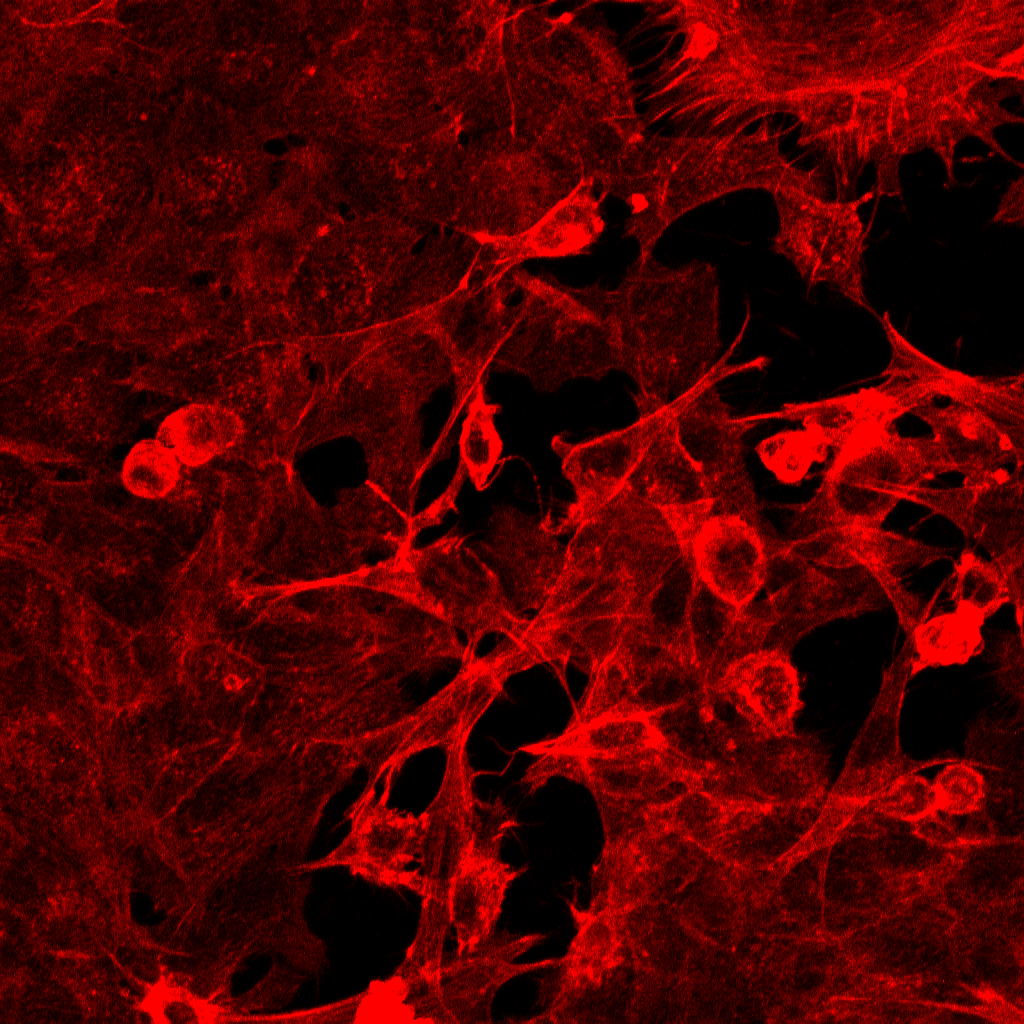

Supplement: Supplementary file 6 — Source data Fig. 2 [file 44319_2026_751_MOESM6_ESM.zip › Raw_data_Figure 2/Figure 2C/Cal27 + fibros KO Factin.tif]

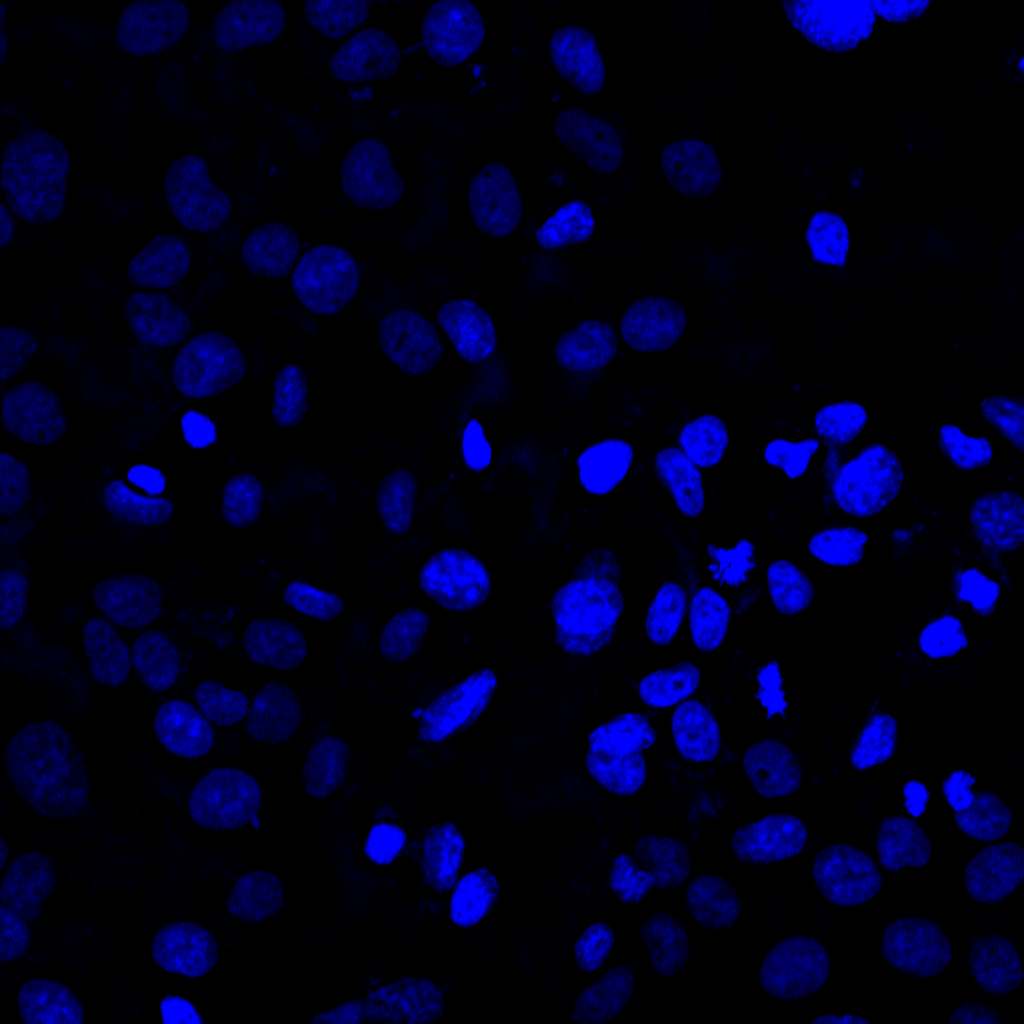

Supplement: Supplementary file 6 — Source data Fig. 2 [file 44319_2026_751_MOESM6_ESM.zip › Raw_data_Figure 2/Figure 2C/Cal27 + fibros KO nucleus.tif]

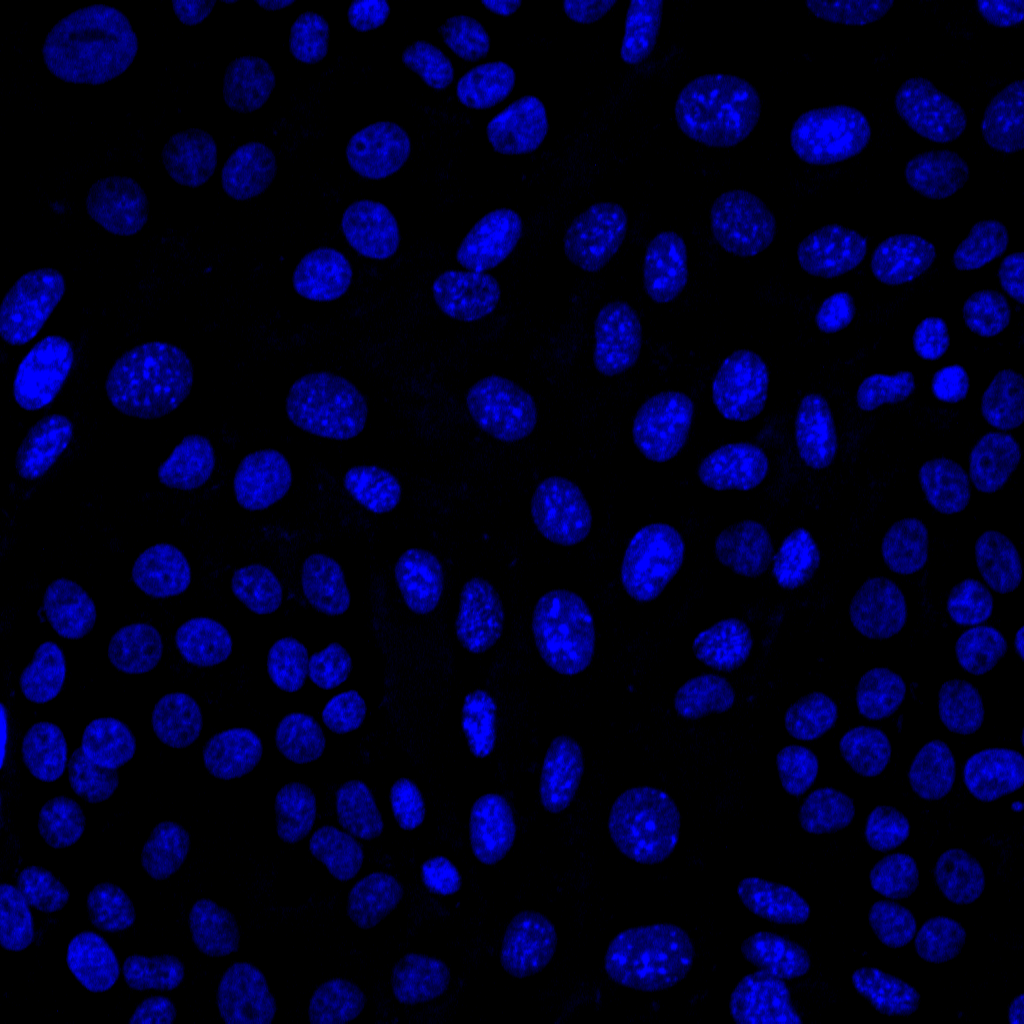

Supplement: Supplementary file 6 — Source data Fig. 2 [file 44319_2026_751_MOESM6_ESM.zip › Raw_data_Figure 2/Figure 2C/cal27 + fibros WT nucleus.tif]

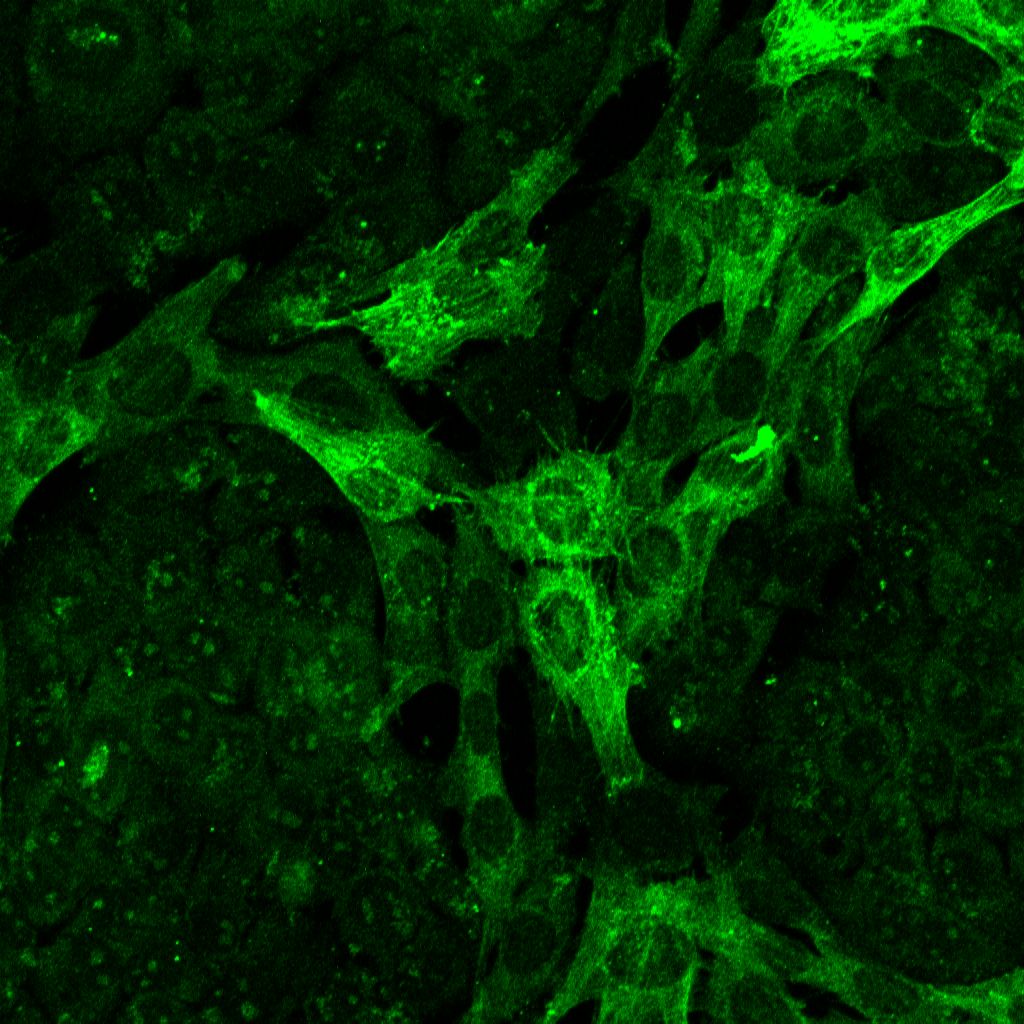

Supplement: Supplementary file 6 — Source data Fig. 2 [file 44319_2026_751_MOESM6_ESM.zip › Raw_data_Figure 2/Figure 2C/Cal27 + fibros wt SMA.tif]

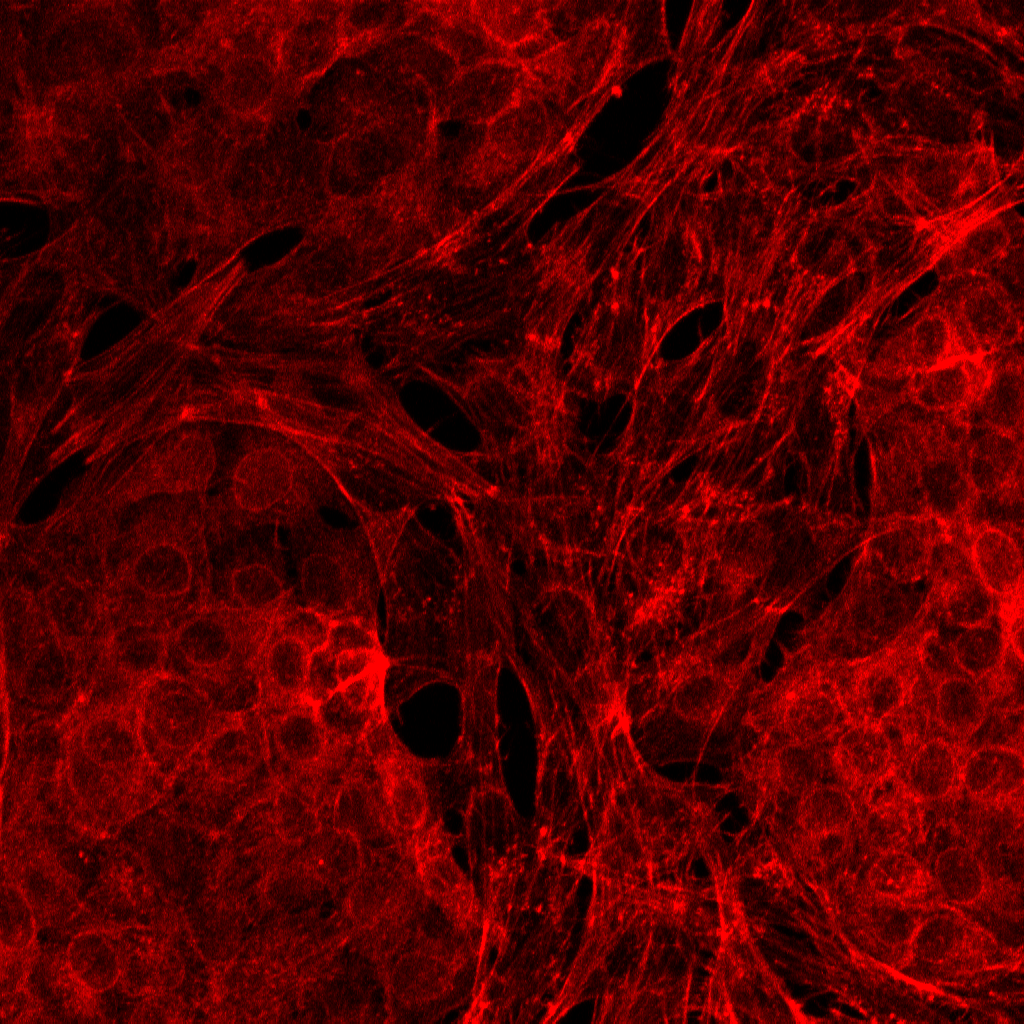

Supplement: Supplementary file 6 — Source data Fig. 2 [file 44319_2026_751_MOESM6_ESM.zip › Raw_data_Figure 2/Figure 2C/Cal27+ fibros WT Factin.tif]

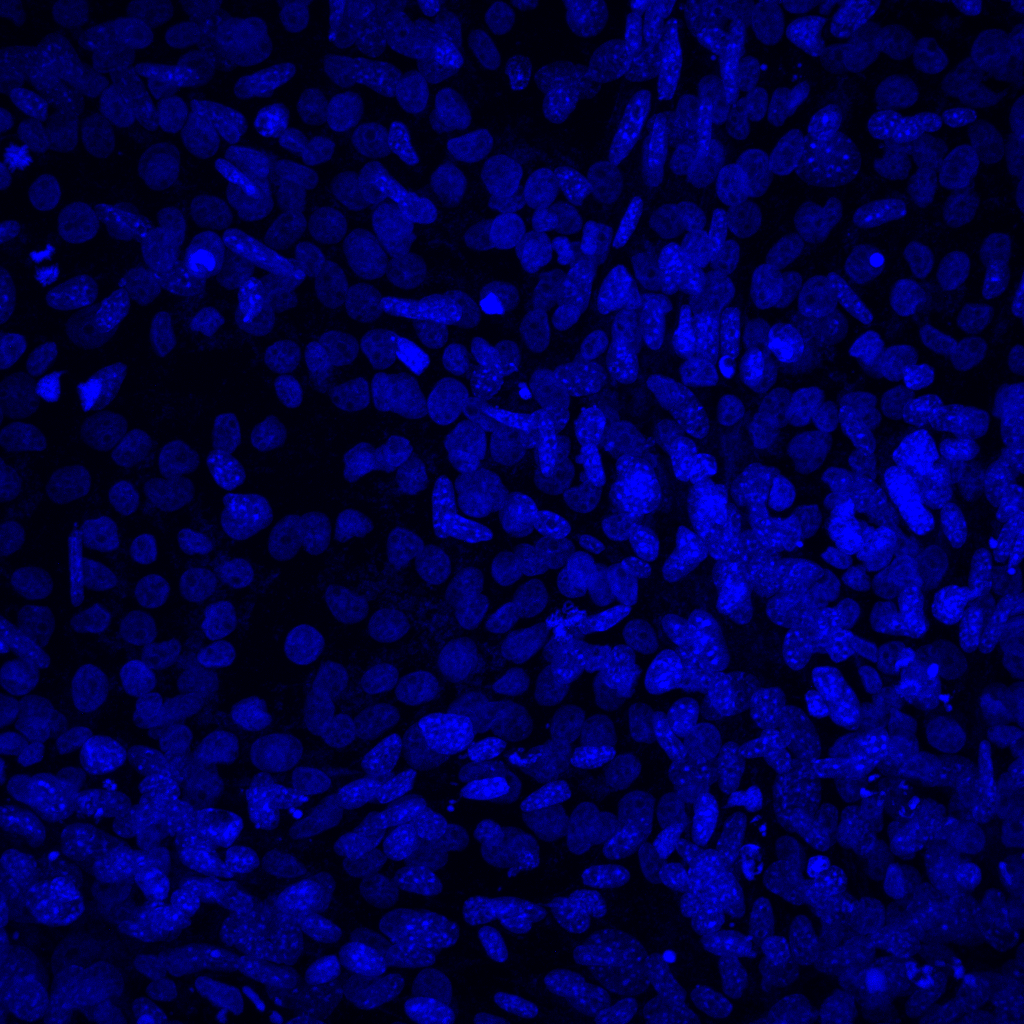

Supplement: Supplementary file 6 — Source data Fig. 2 [file 44319_2026_751_MOESM6_ESM.zip › Raw_data_Figure 2/Figure 2D/Cal27 +GqKO nuclei.tif]
