# Supplementary figures and images for: Loss of Gαq reshapes fibroblast traits and drives tumor-stroma remodeling in oral cancer progression (part 2 of 5)
Source: EMBO Rep. 2026 Apr 10;27(10):2639–74. doi: 10.1038/s44319-026-00751-2 (PMC13219523; doi:10.1038/s44319-026-00751-2)

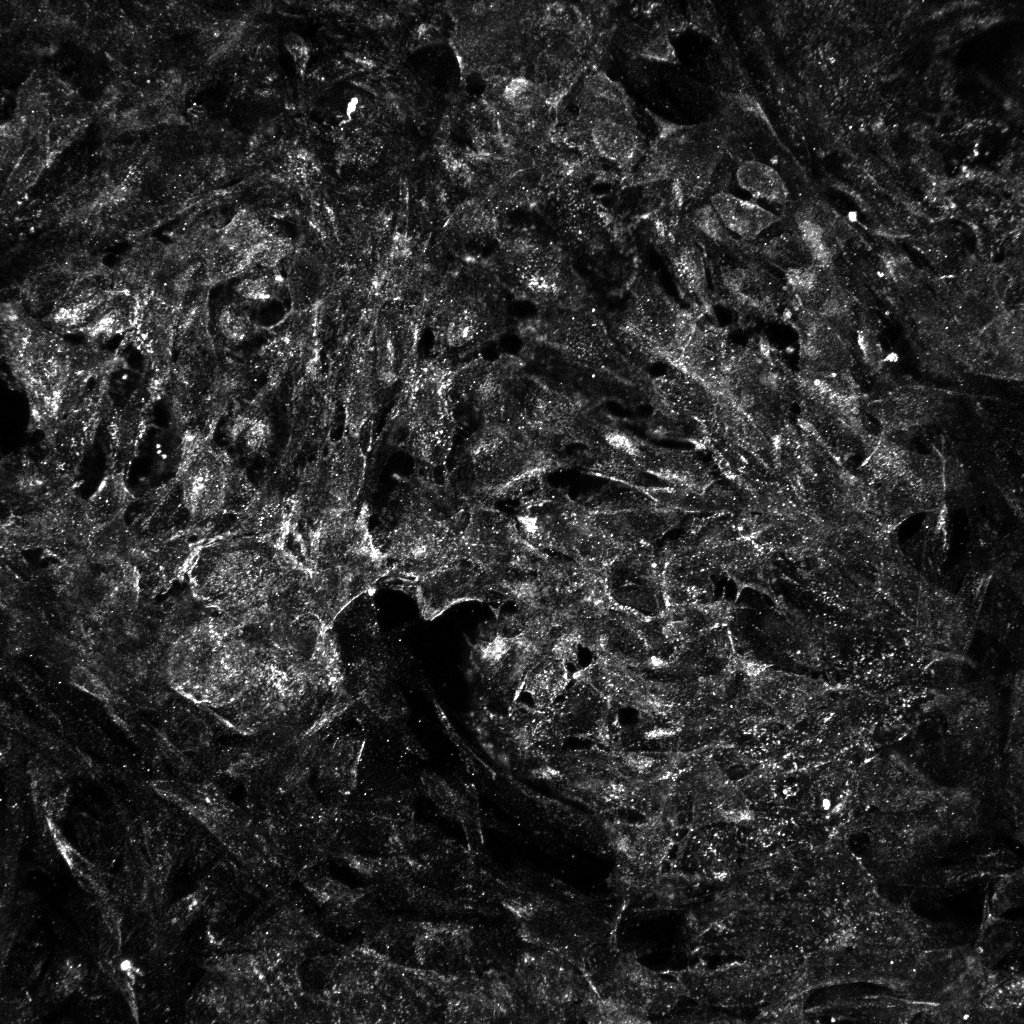

Supplement: Supplementary file 6 — Source data Fig. 2 [file 44319_2026_751_MOESM6_ESM.zip › Raw_data_Figure 2/Figure 2D/Cal27 +WT cav1 only.jpg]

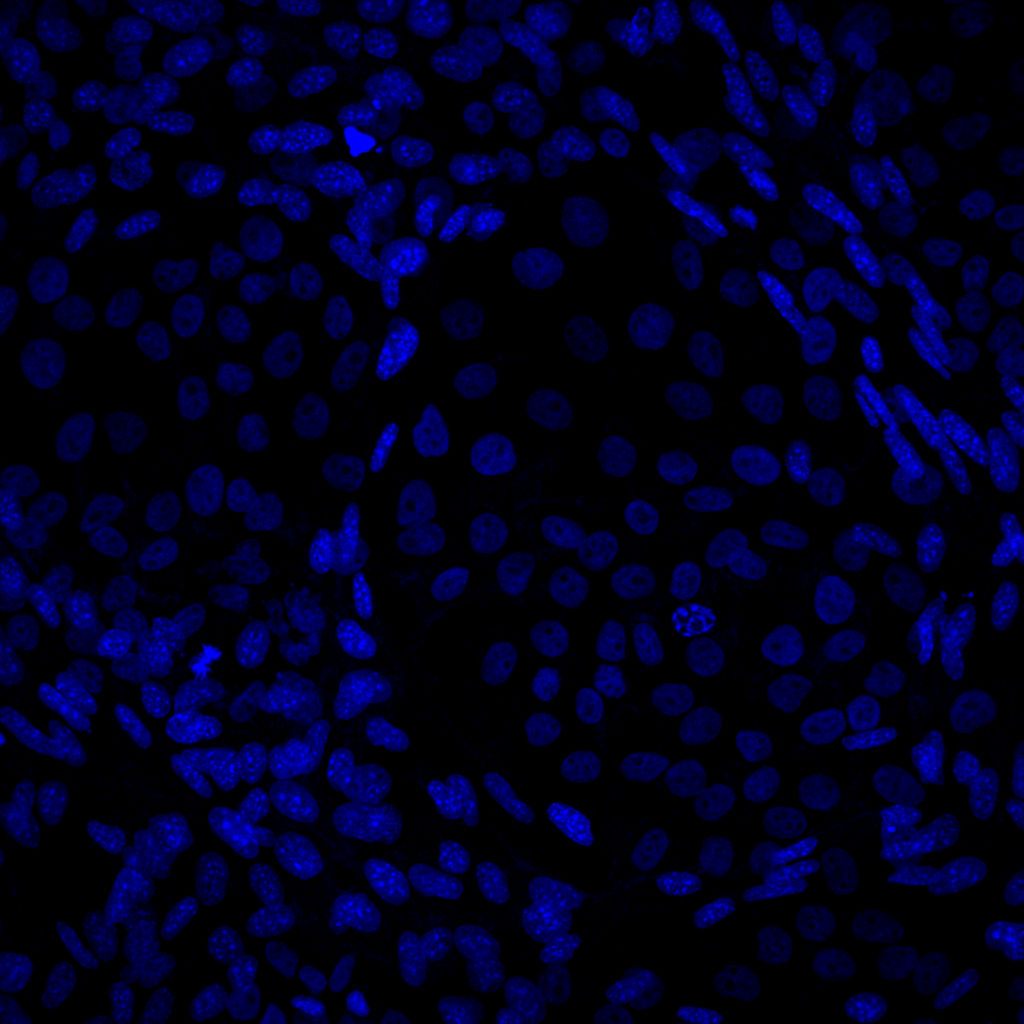

Supplement: Supplementary file 6 — Source data Fig. 2 [file 44319_2026_751_MOESM6_ESM.zip › Raw_data_Figure 2/Figure 2D/Cal27 +WT nuclei.tif]

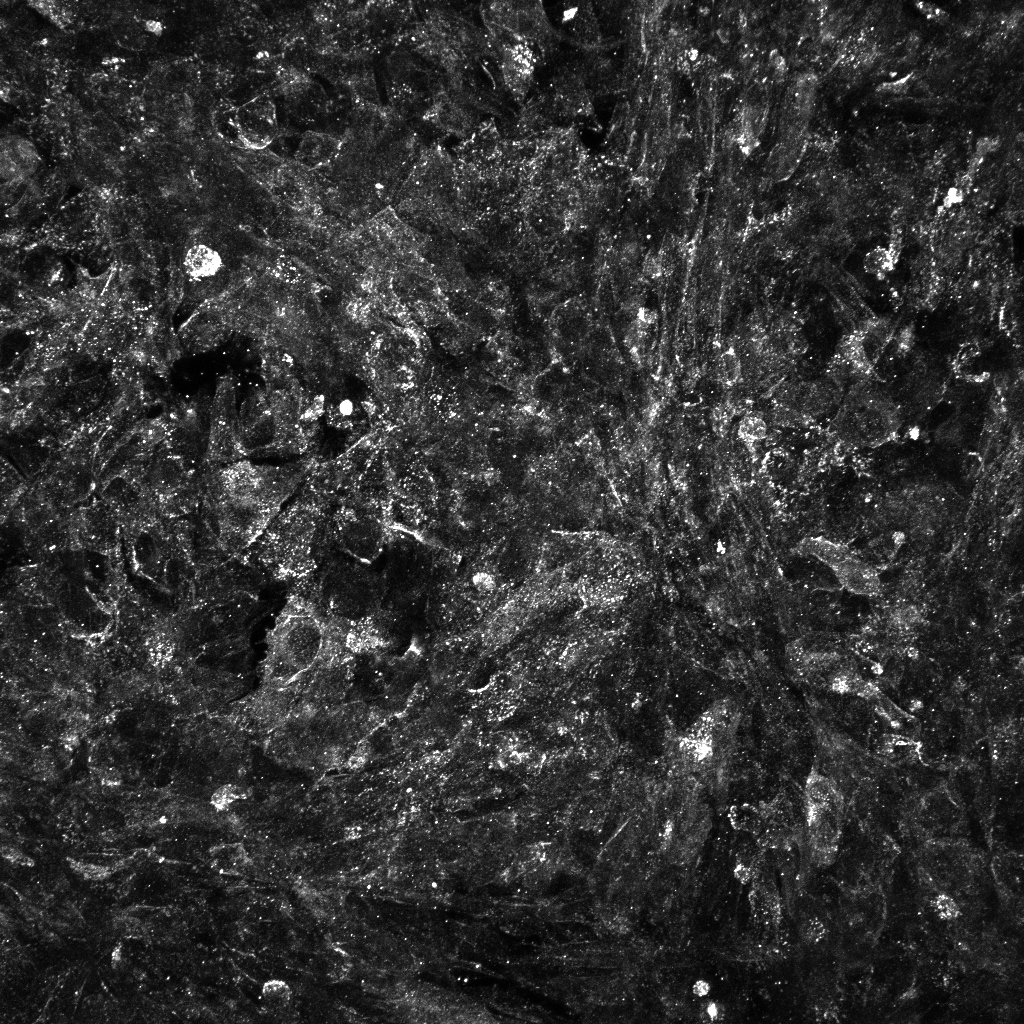

Supplement: Supplementary file 6 — Source data Fig. 2 [file 44319_2026_751_MOESM6_ESM.zip › Raw_data_Figure 2/Figure 2D/Cal27+ GqKO cav1 only.jpg]

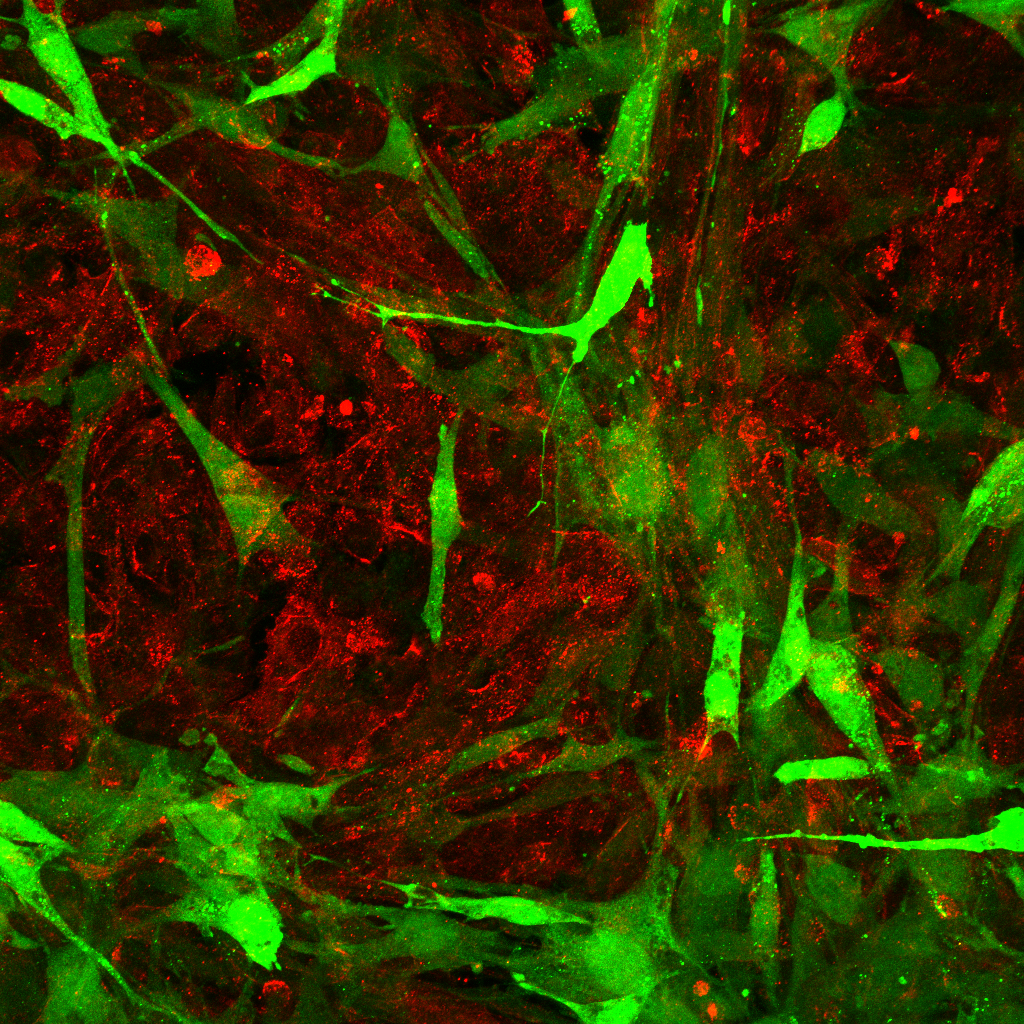

Supplement: Supplementary file 6 — Source data Fig. 2 [file 44319_2026_751_MOESM6_ESM.zip › Raw_data_Figure 2/Figure 2D/Cal27+GqKO GFP.tif]

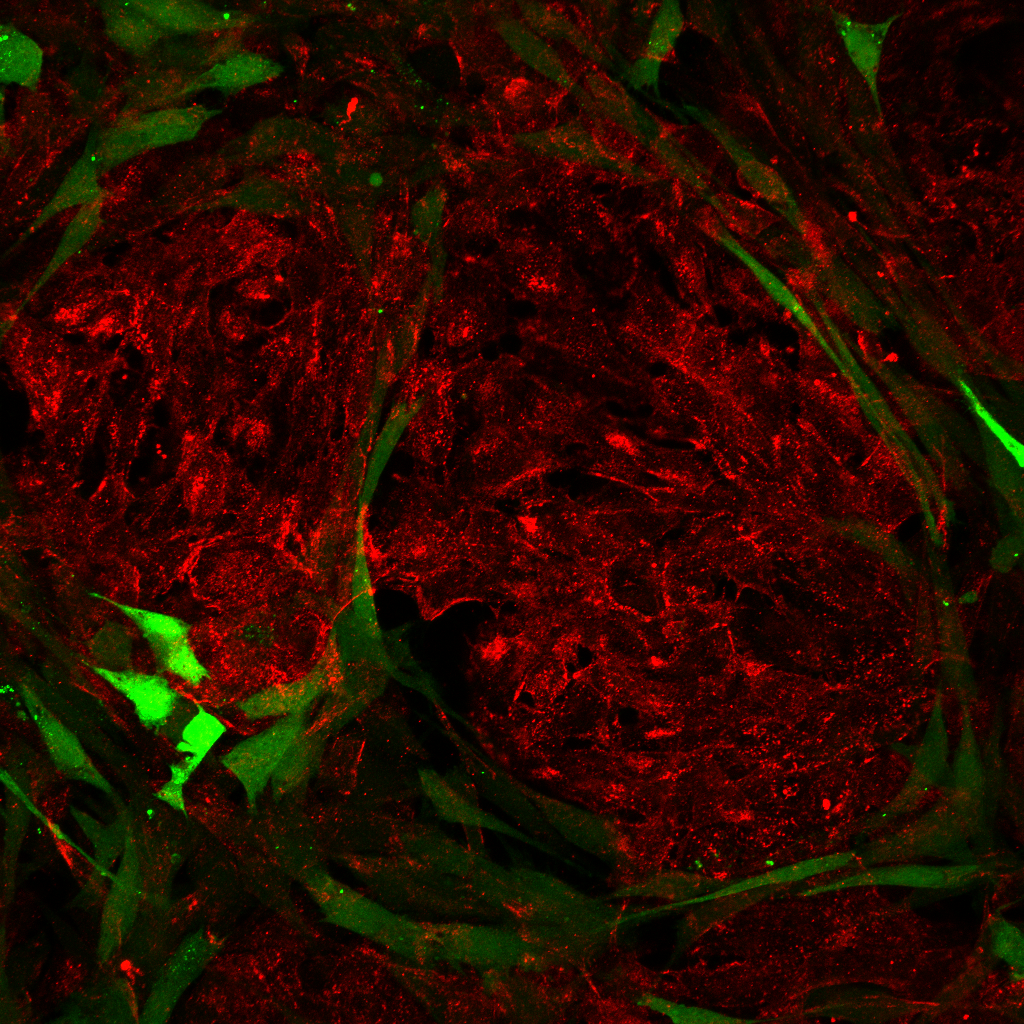

Supplement: Supplementary file 6 — Source data Fig. 2 [file 44319_2026_751_MOESM6_ESM.zip › Raw_data_Figure 2/Figure 2D/Cal27+WT GFP.tif]

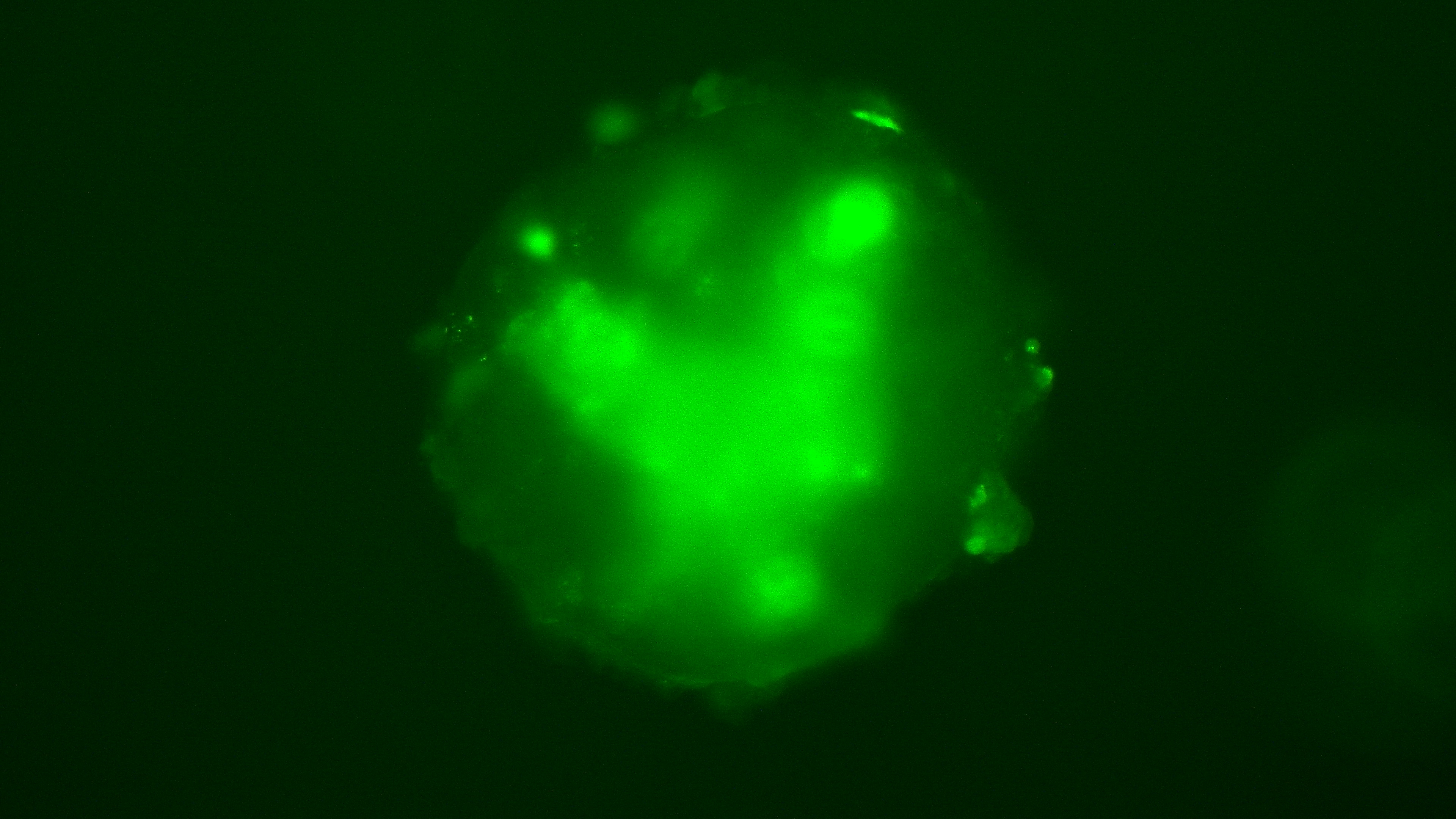

Supplement: Supplementary file 6 — Source data Fig. 2 [file 44319_2026_751_MOESM6_ESM.zip › Raw_data_Figure 2/Figure 2E/selected/ko221004111249o.jpg]

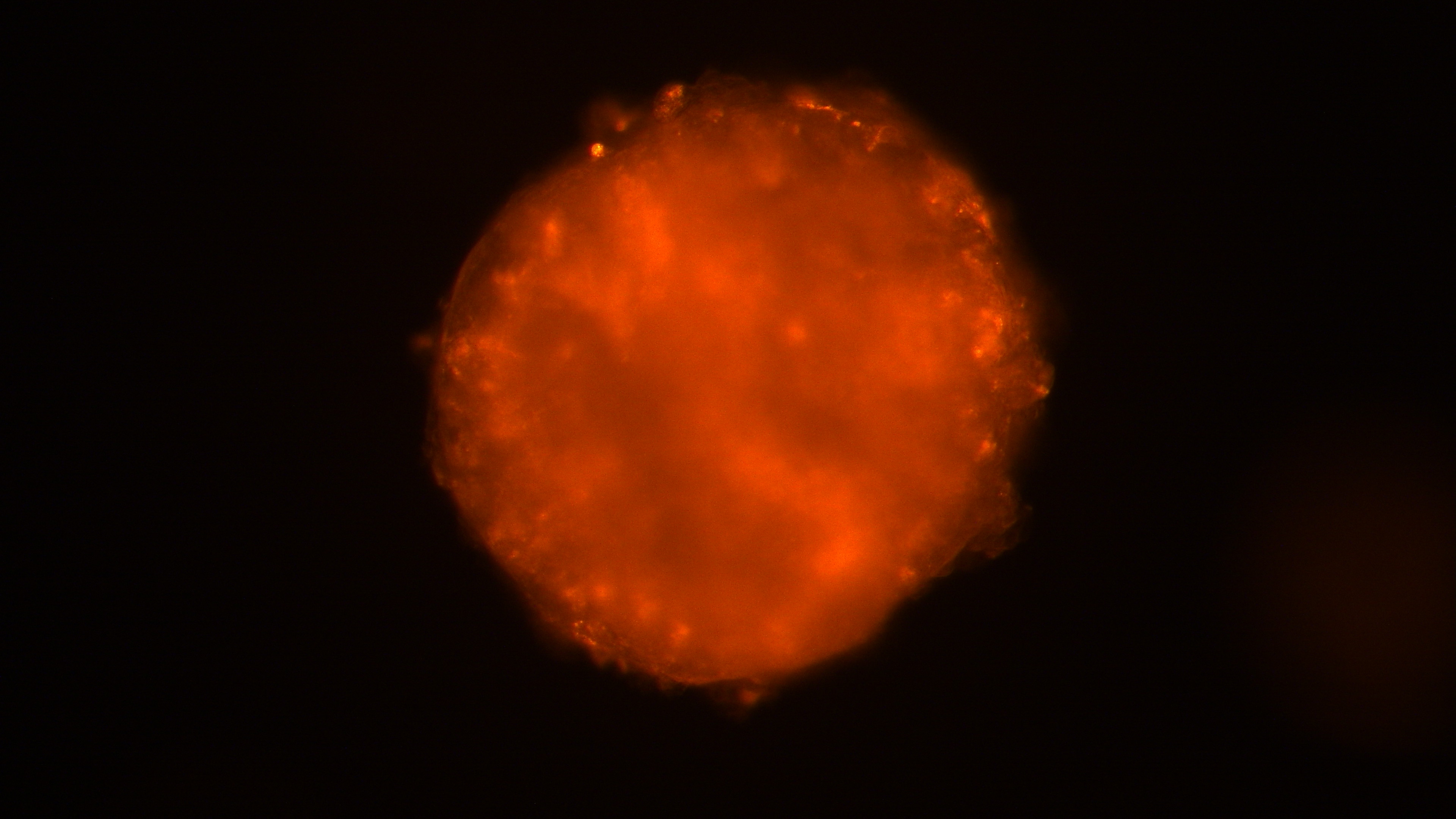

Supplement: Supplementary file 6 — Source data Fig. 2 [file 44319_2026_751_MOESM6_ESM.zip › Raw_data_Figure 2/Figure 2E/selected/ko221004111255o.jpg]

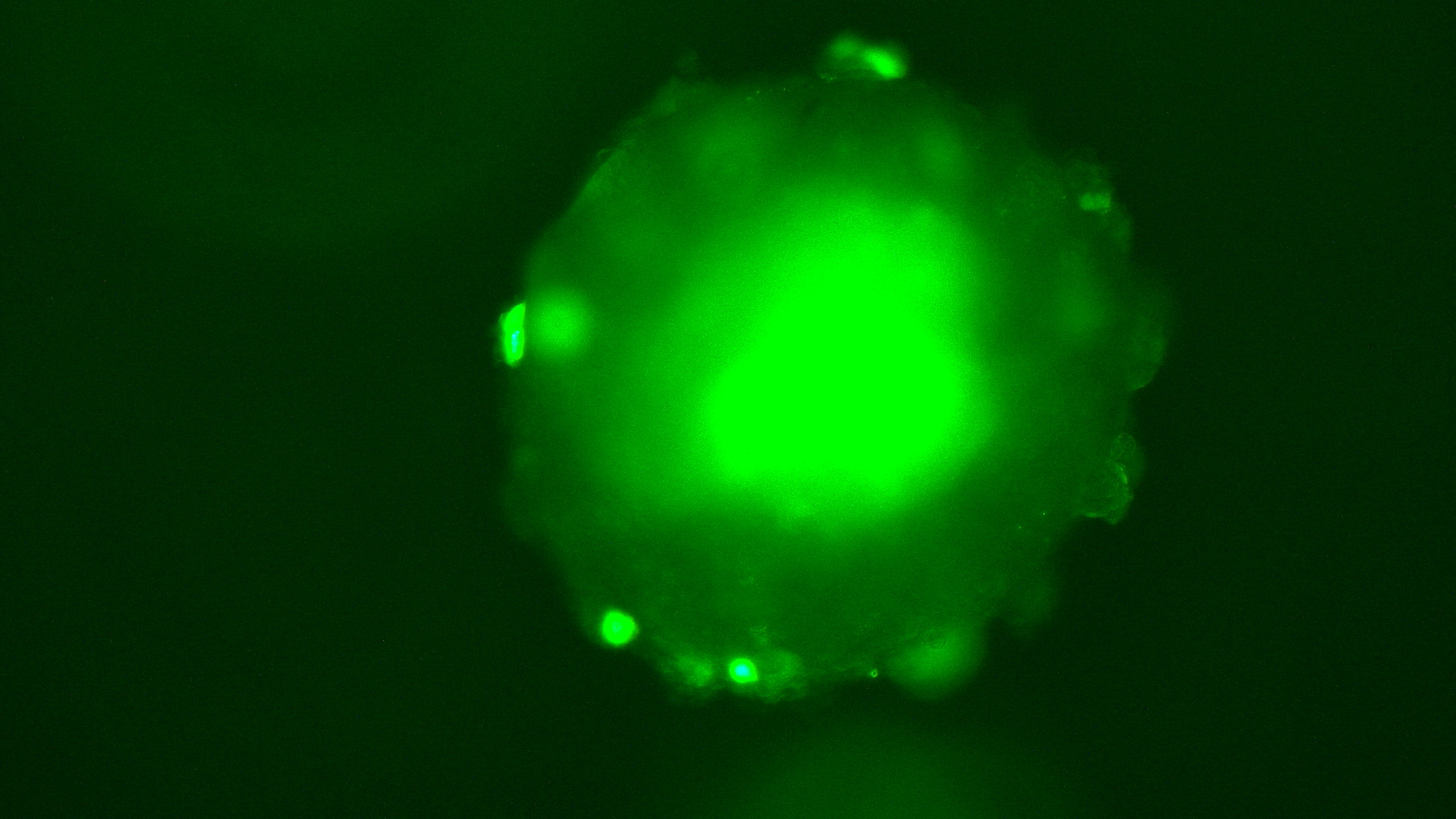

Supplement: Supplementary file 6 — Source data Fig. 2 [file 44319_2026_751_MOESM6_ESM.zip › Raw_data_Figure 2/Figure 2E/selected/kogreencalred221004145036o.jpg]

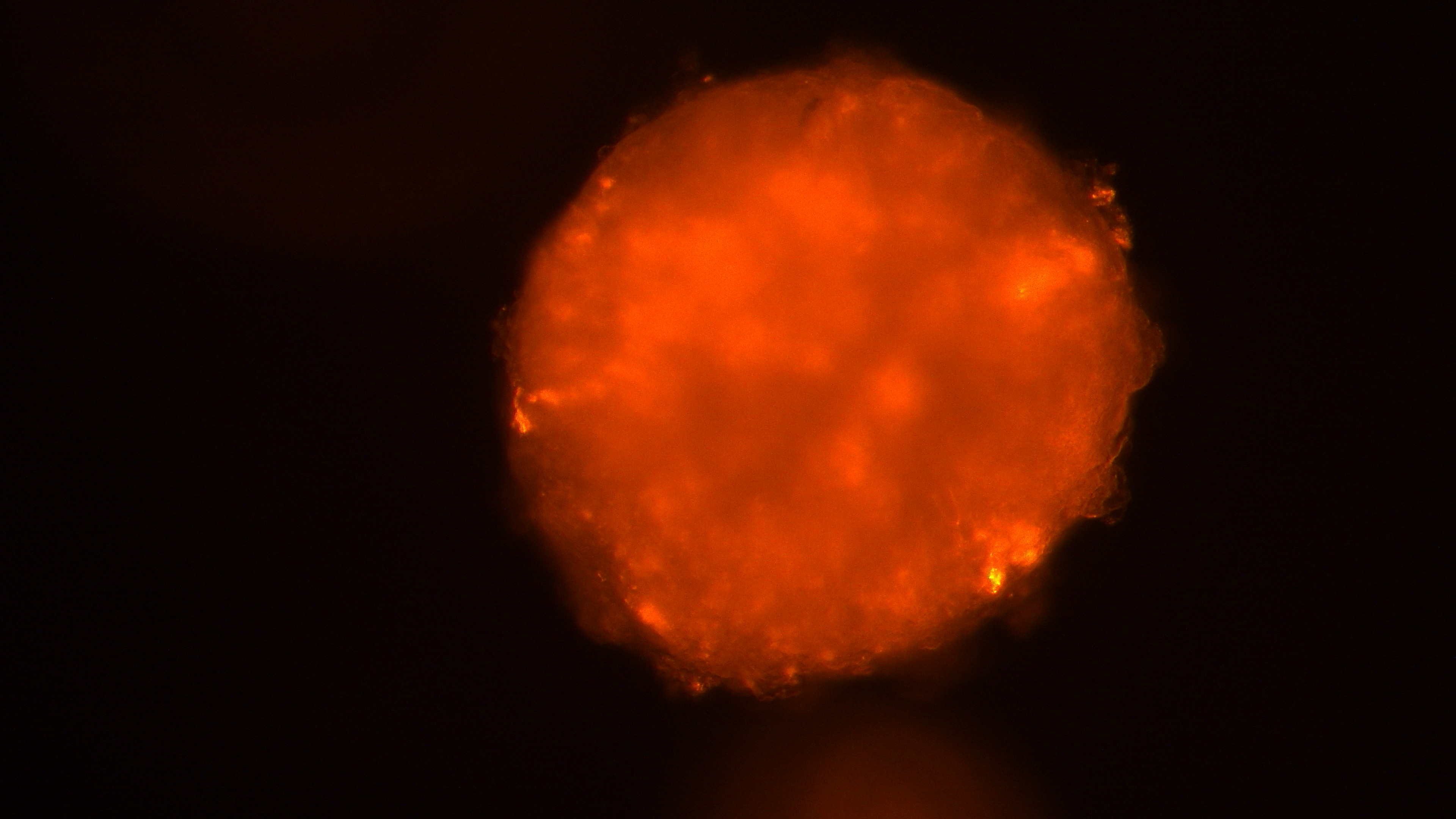

Supplement: Supplementary file 6 — Source data Fig. 2 [file 44319_2026_751_MOESM6_ESM.zip › Raw_data_Figure 2/Figure 2E/selected/kogreencalred221004145042o.jpg]

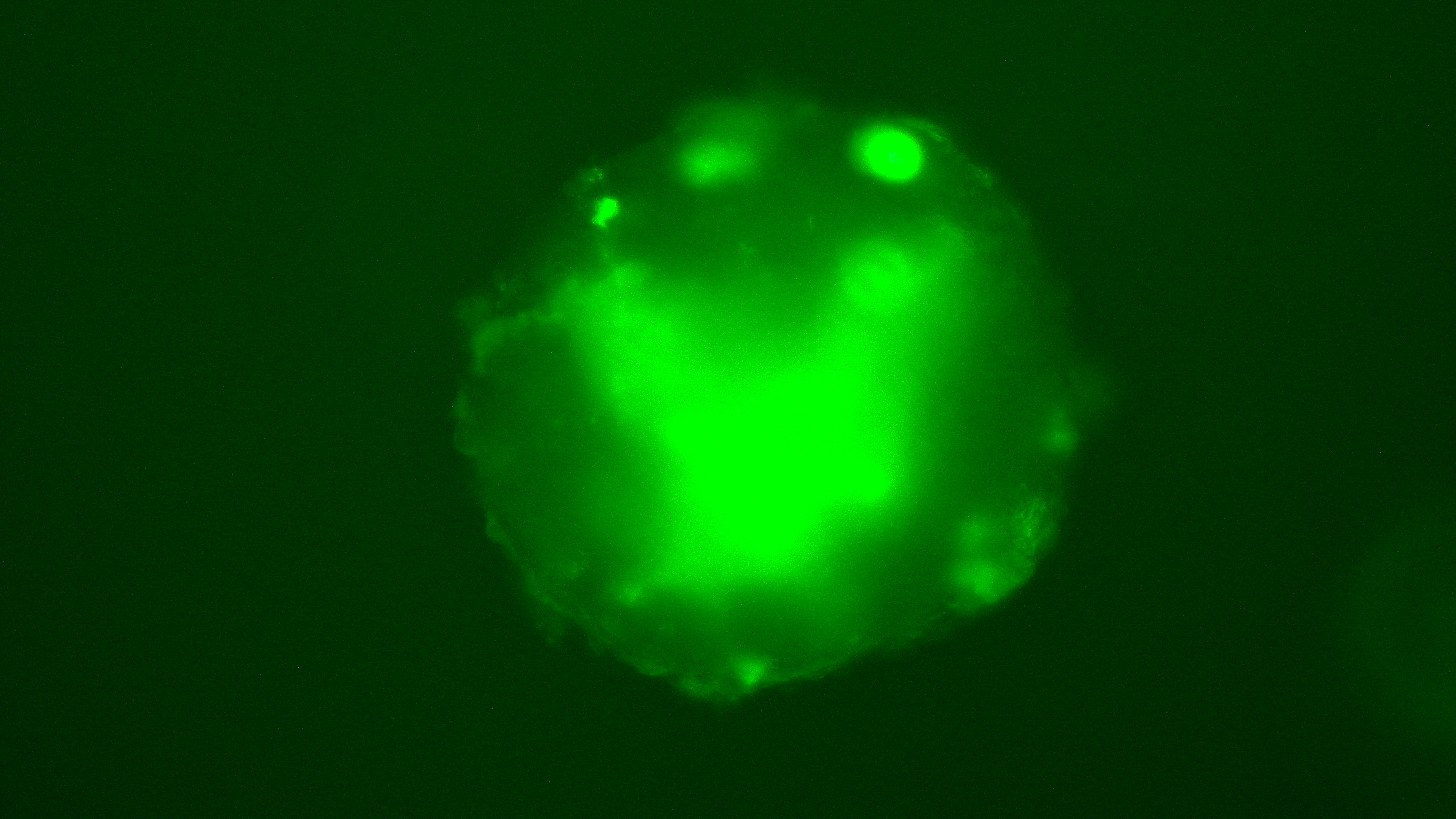

Supplement: Supplementary file 6 — Source data Fig. 2 [file 44319_2026_751_MOESM6_ESM.zip › Raw_data_Figure 2/Figure 2E/selected/kogreencalred221004145144o.jpg]

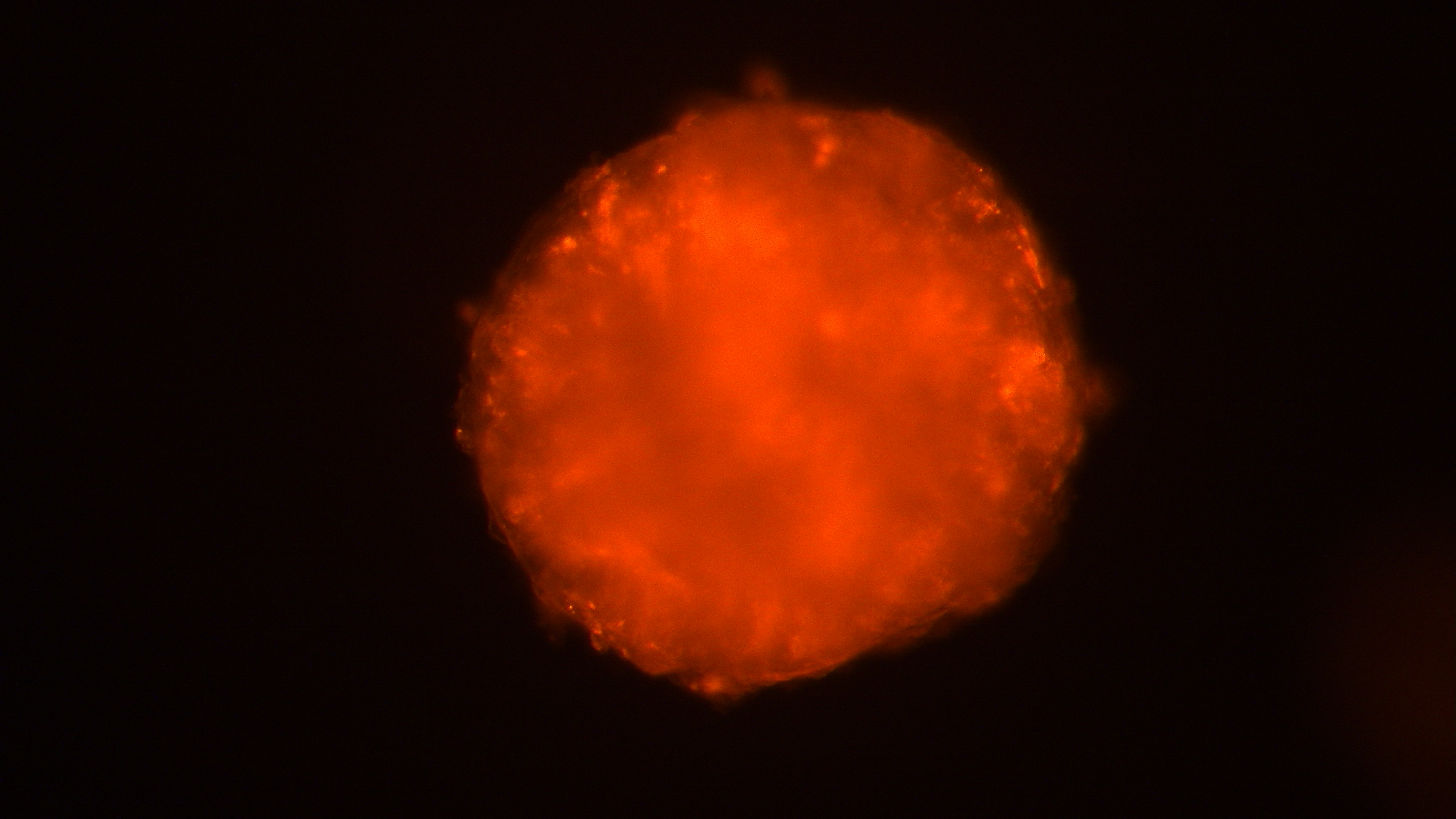

Supplement: Supplementary file 6 — Source data Fig. 2 [file 44319_2026_751_MOESM6_ESM.zip › Raw_data_Figure 2/Figure 2E/selected/kogreencalred221004145148o.jpg]

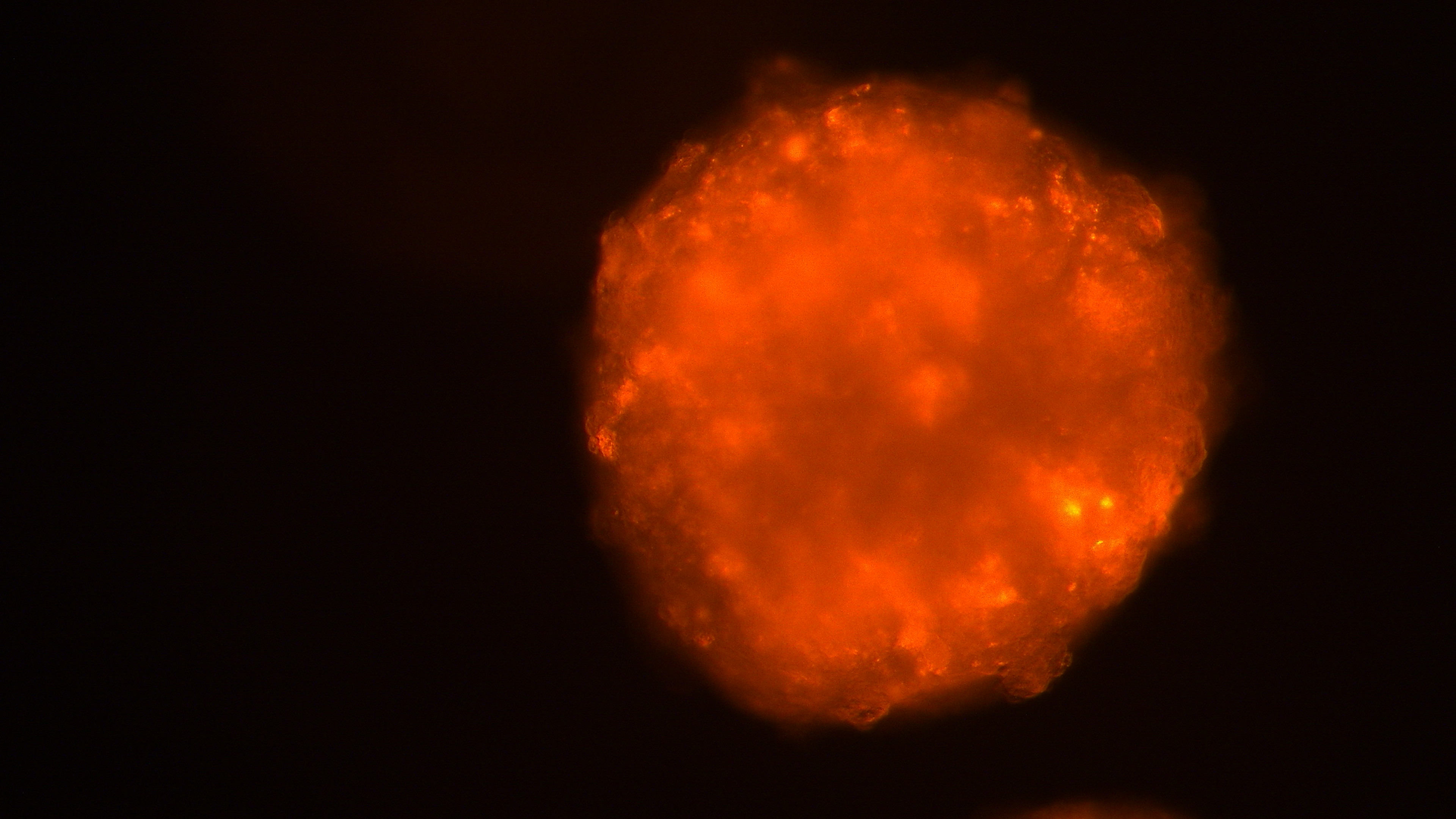

Supplement: Supplementary file 6 — Source data Fig. 2 [file 44319_2026_751_MOESM6_ESM.zip › Raw_data_Figure 2/Figure 2E/selected/kogreencalred221004145454o selected.jpg]

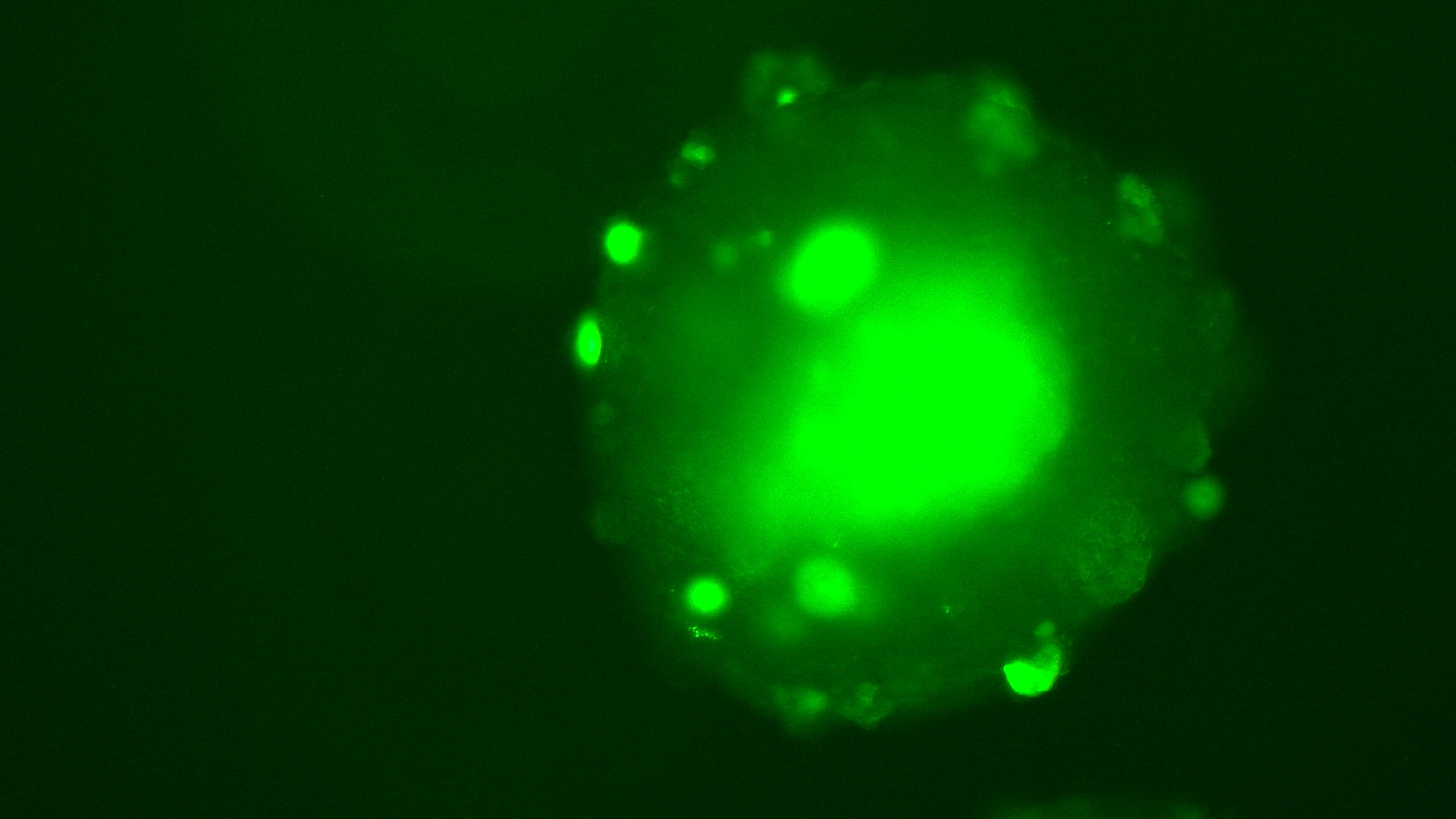

Supplement: Supplementary file 6 — Source data Fig. 2 [file 44319_2026_751_MOESM6_ESM.zip › Raw_data_Figure 2/Figure 2E/selected/kogreencalred221004145505o selected.jpg]

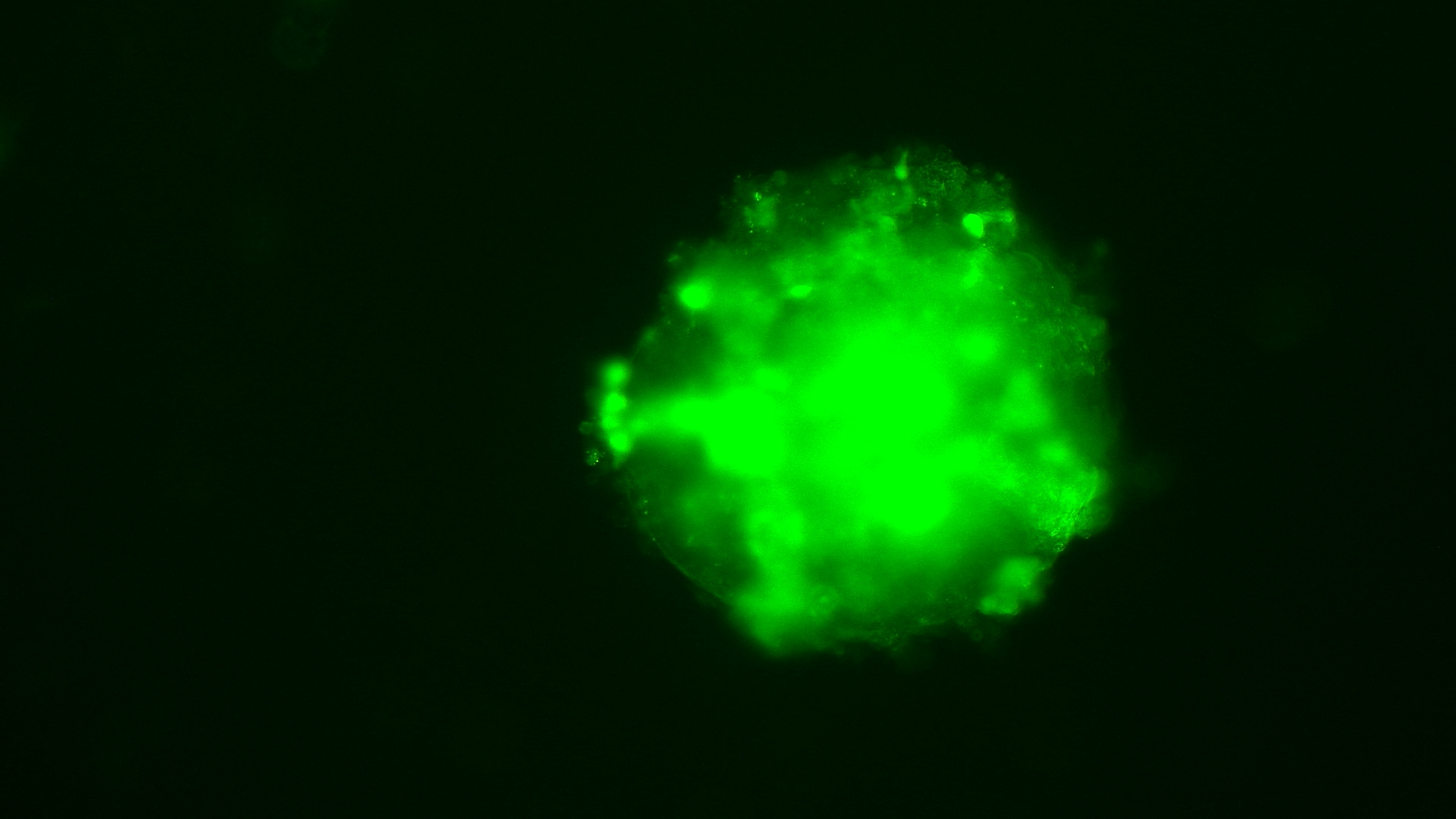

Supplement: Supplementary file 6 — Source data Fig. 2 [file 44319_2026_751_MOESM6_ESM.zip › Raw_data_Figure 2/Figure 2E/selected/wtgreencalred221004144144o selected.jpg]

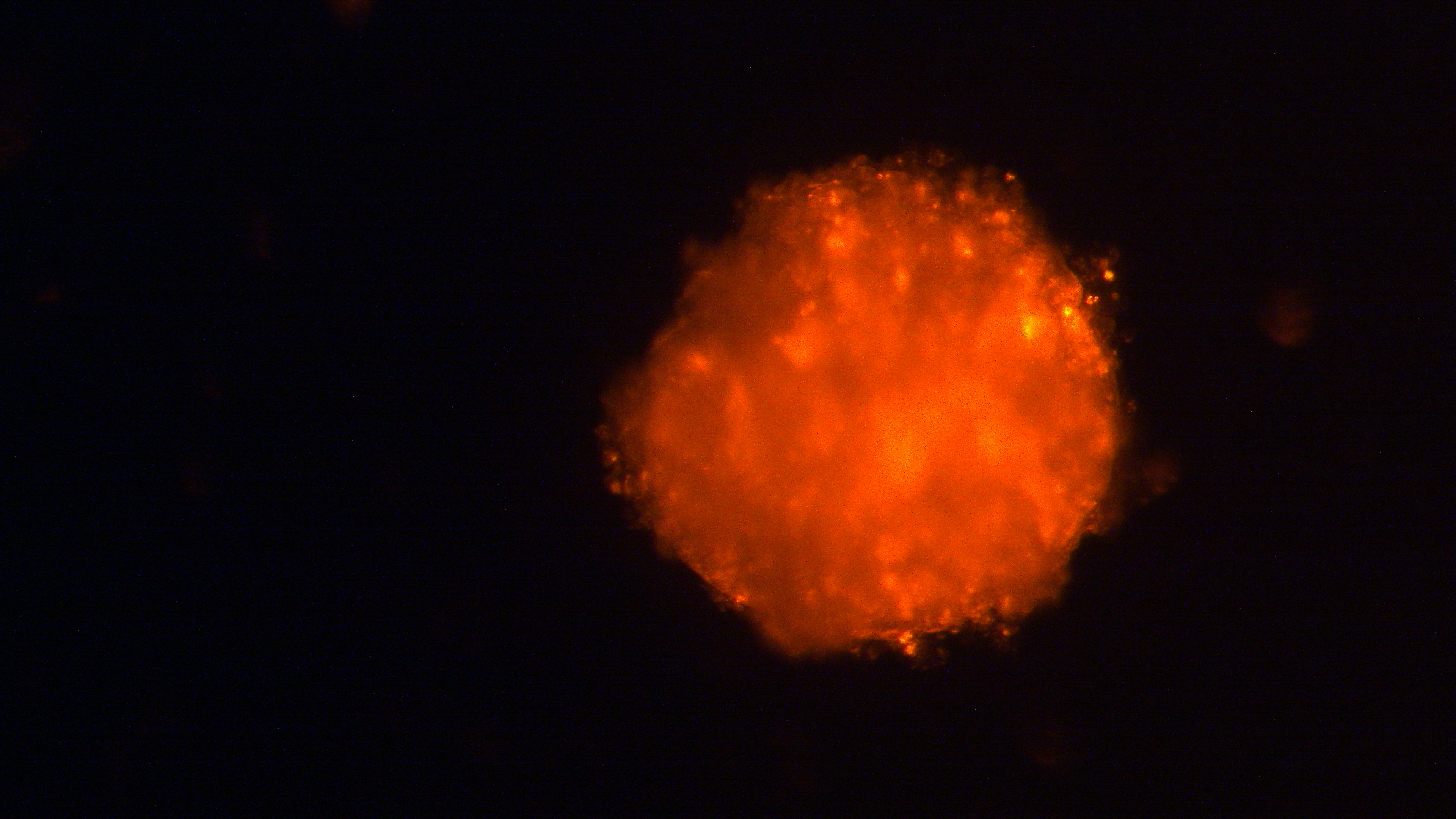

Supplement: Supplementary file 6 — Source data Fig. 2 [file 44319_2026_751_MOESM6_ESM.zip › Raw_data_Figure 2/Figure 2E/selected/wtgreencalred221004144224oselected.jpg]

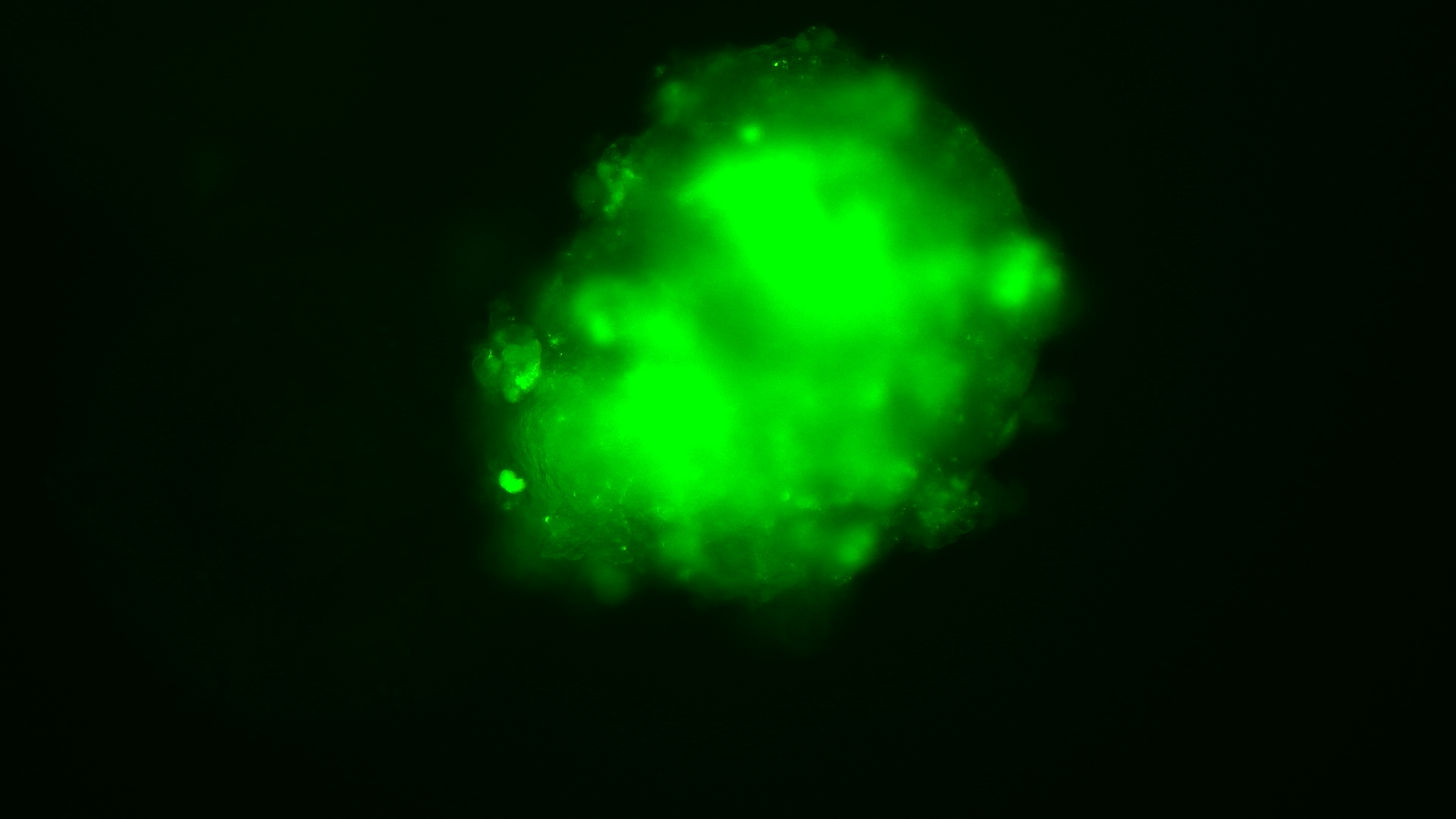

Supplement: Supplementary file 6 — Source data Fig. 2 [file 44319_2026_751_MOESM6_ESM.zip › Raw_data_Figure 2/Figure 2E/selected/wtgreencalred221004144335o.jpg]

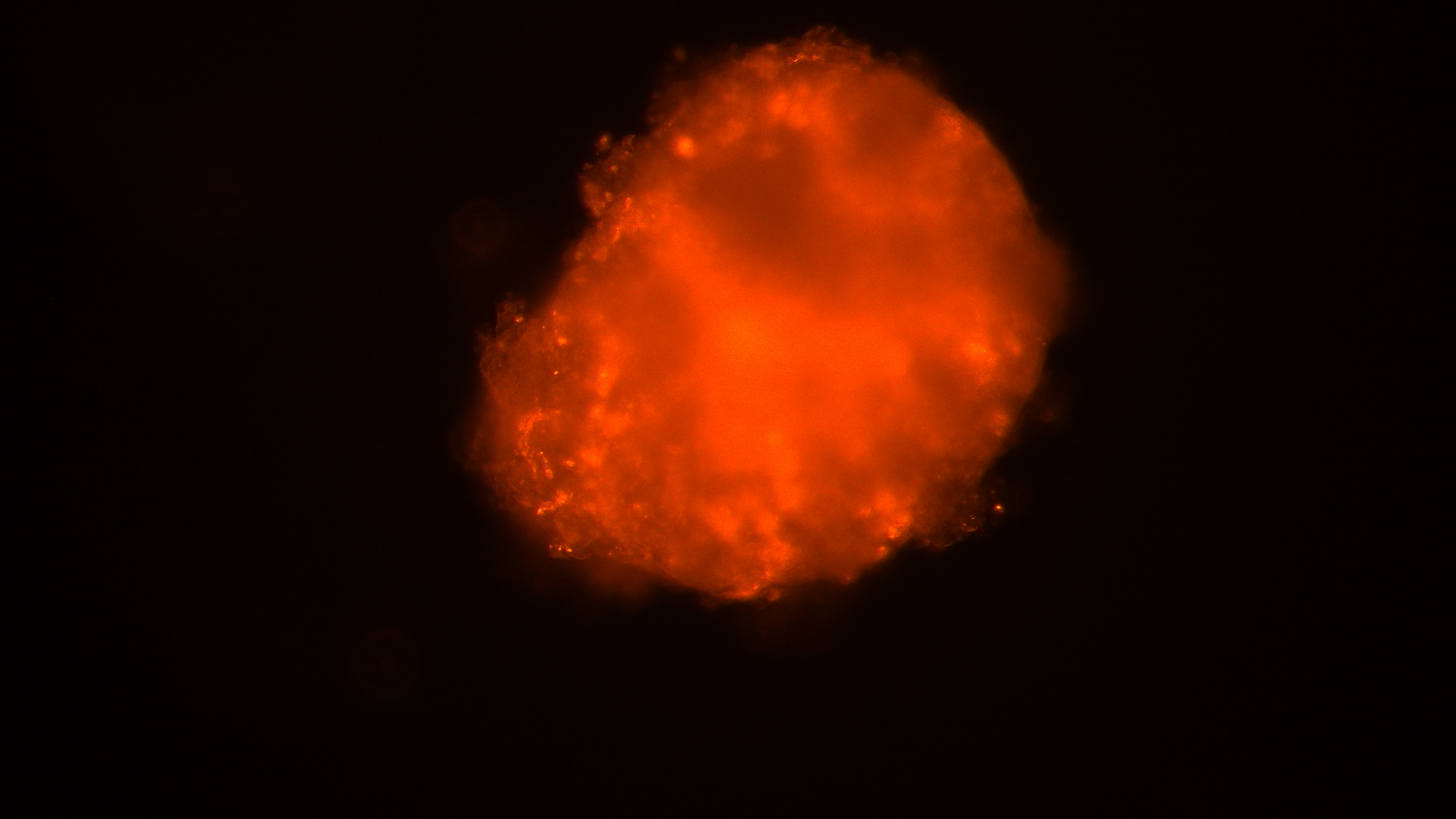

Supplement: Supplementary file 6 — Source data Fig. 2 [file 44319_2026_751_MOESM6_ESM.zip › Raw_data_Figure 2/Figure 2E/selected/wtgreencalred221004144339o.jpg]

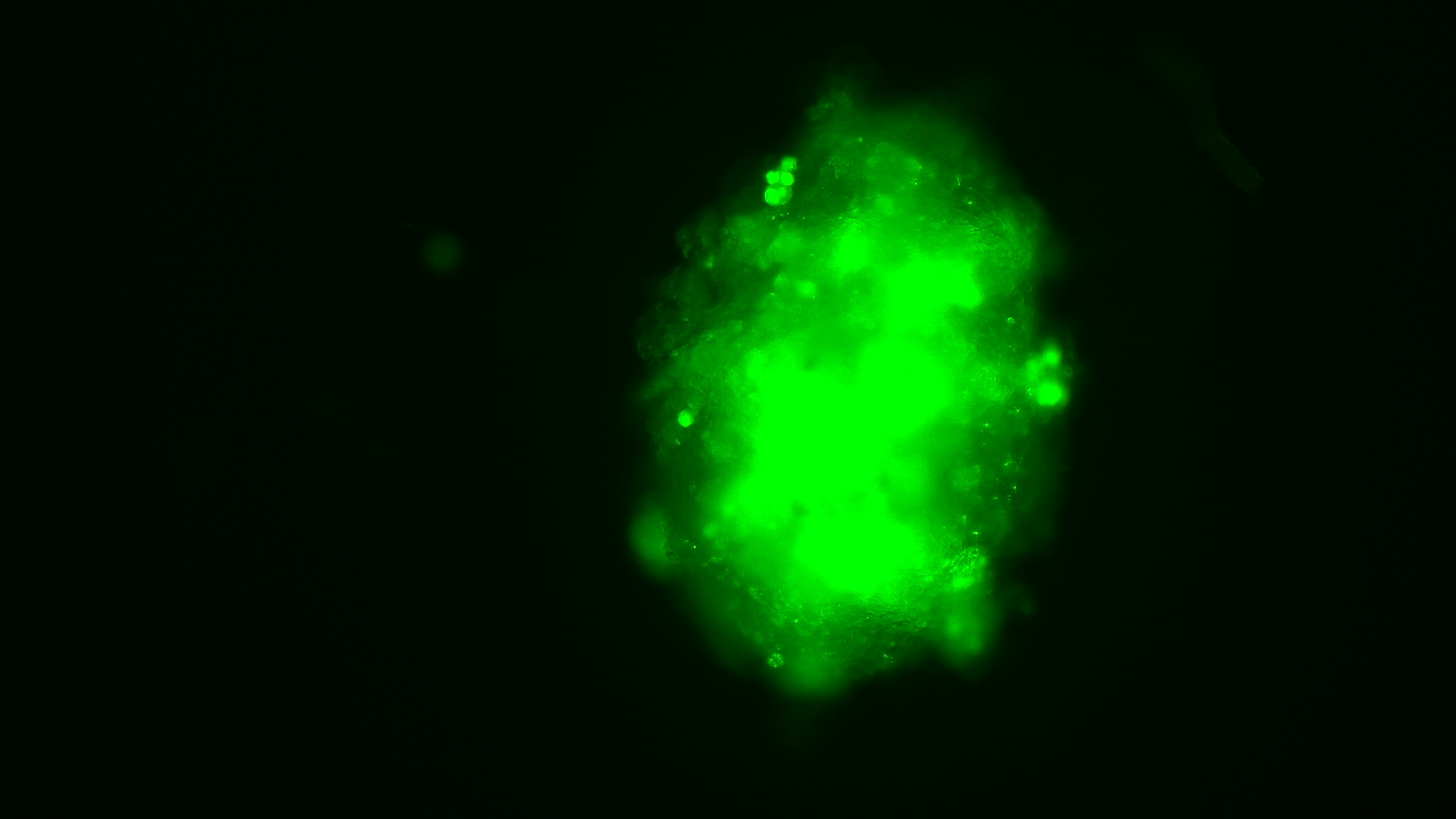

Supplement: Supplementary file 6 — Source data Fig. 2 [file 44319_2026_751_MOESM6_ESM.zip › Raw_data_Figure 2/Figure 2E/selected/wtgreencalred221004144511o.jpg]

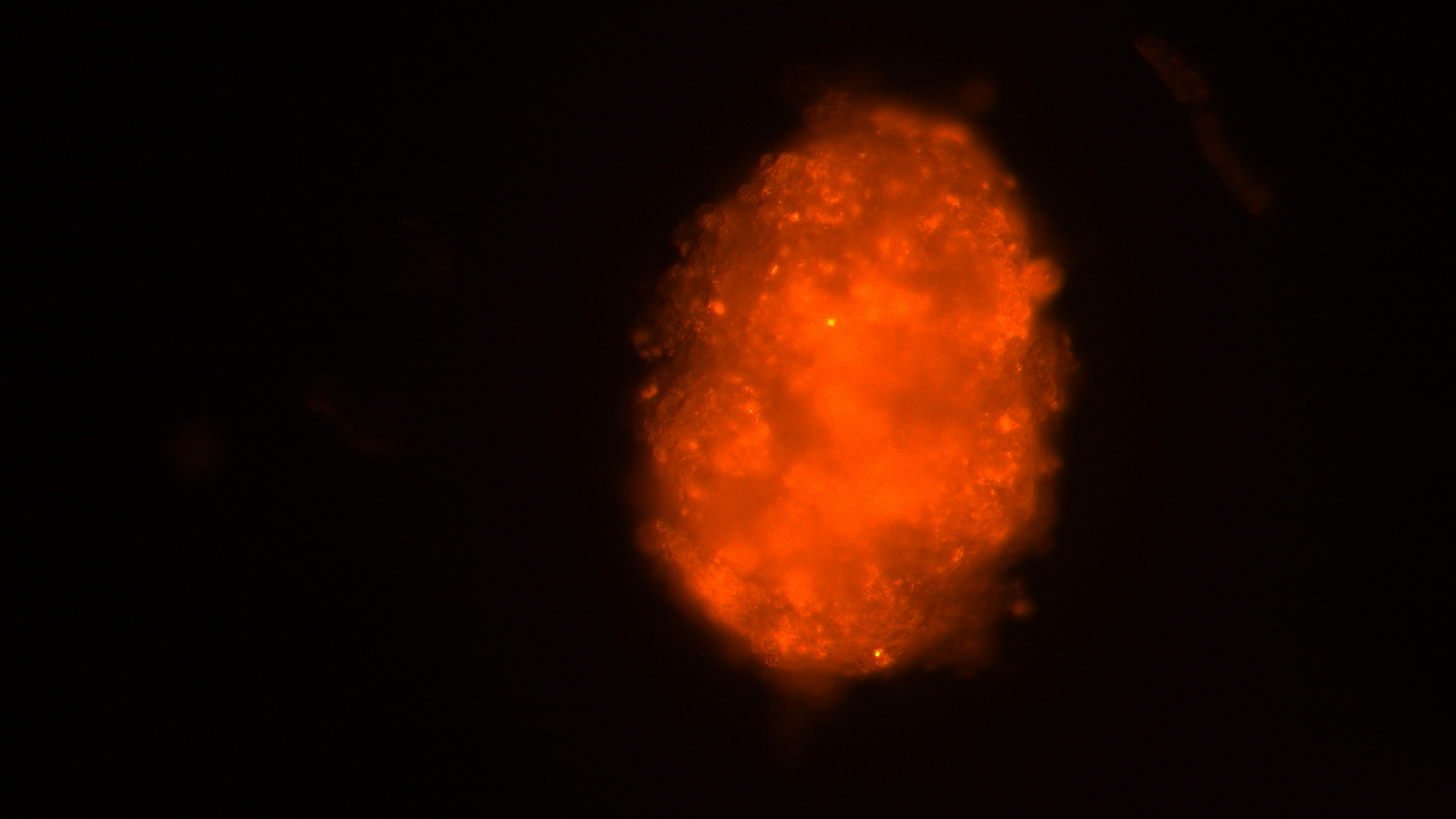

Supplement: Supplementary file 6 — Source data Fig. 2 [file 44319_2026_751_MOESM6_ESM.zip › Raw_data_Figure 2/Figure 2E/selected/wtgreencalred221004144522o.jpg]

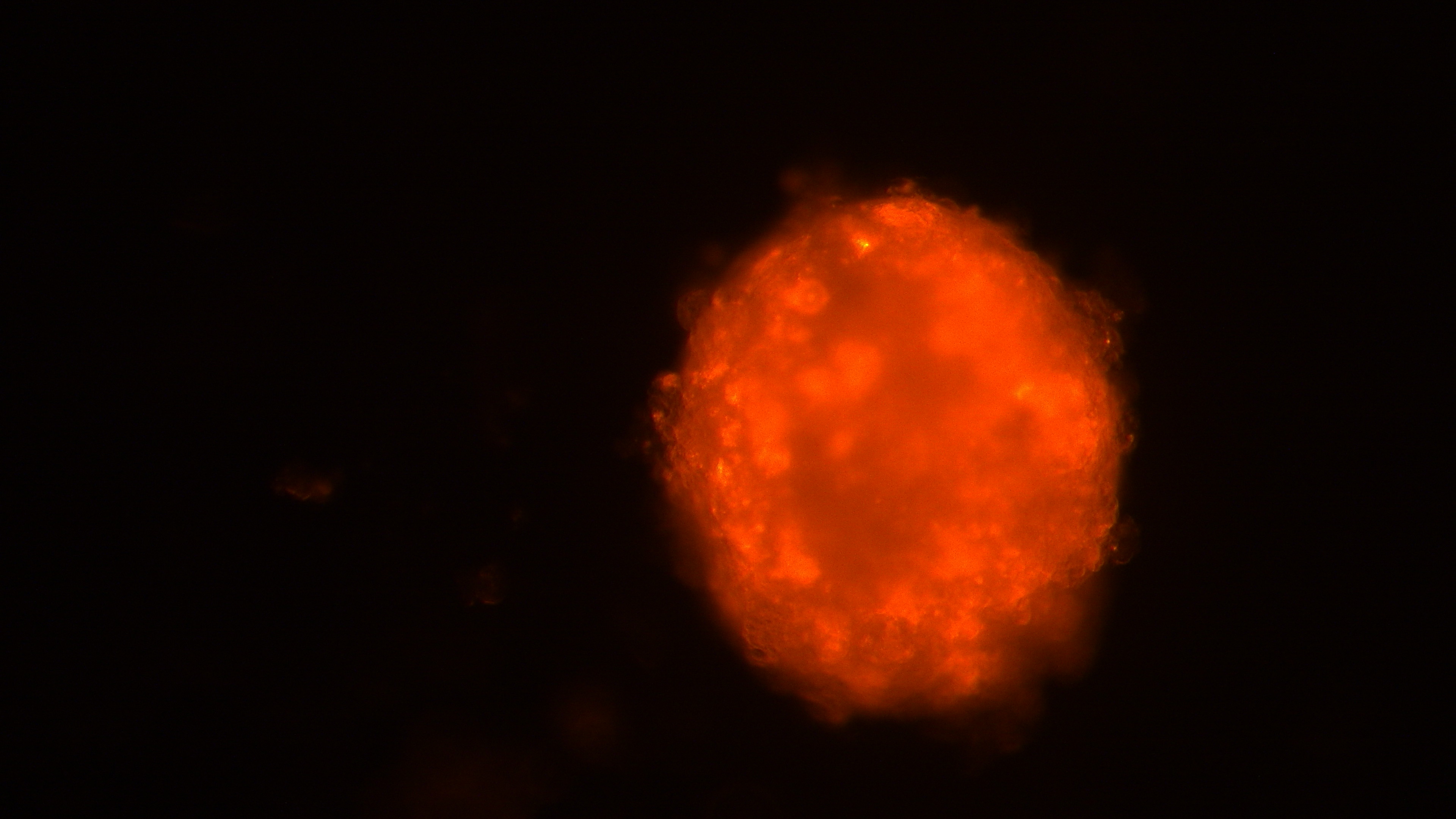

Supplement: Supplementary file 6 — Source data Fig. 2 [file 44319_2026_751_MOESM6_ESM.zip › Raw_data_Figure 2/Figure 2E/selected/wtgreencalred221004144608or.jpg]

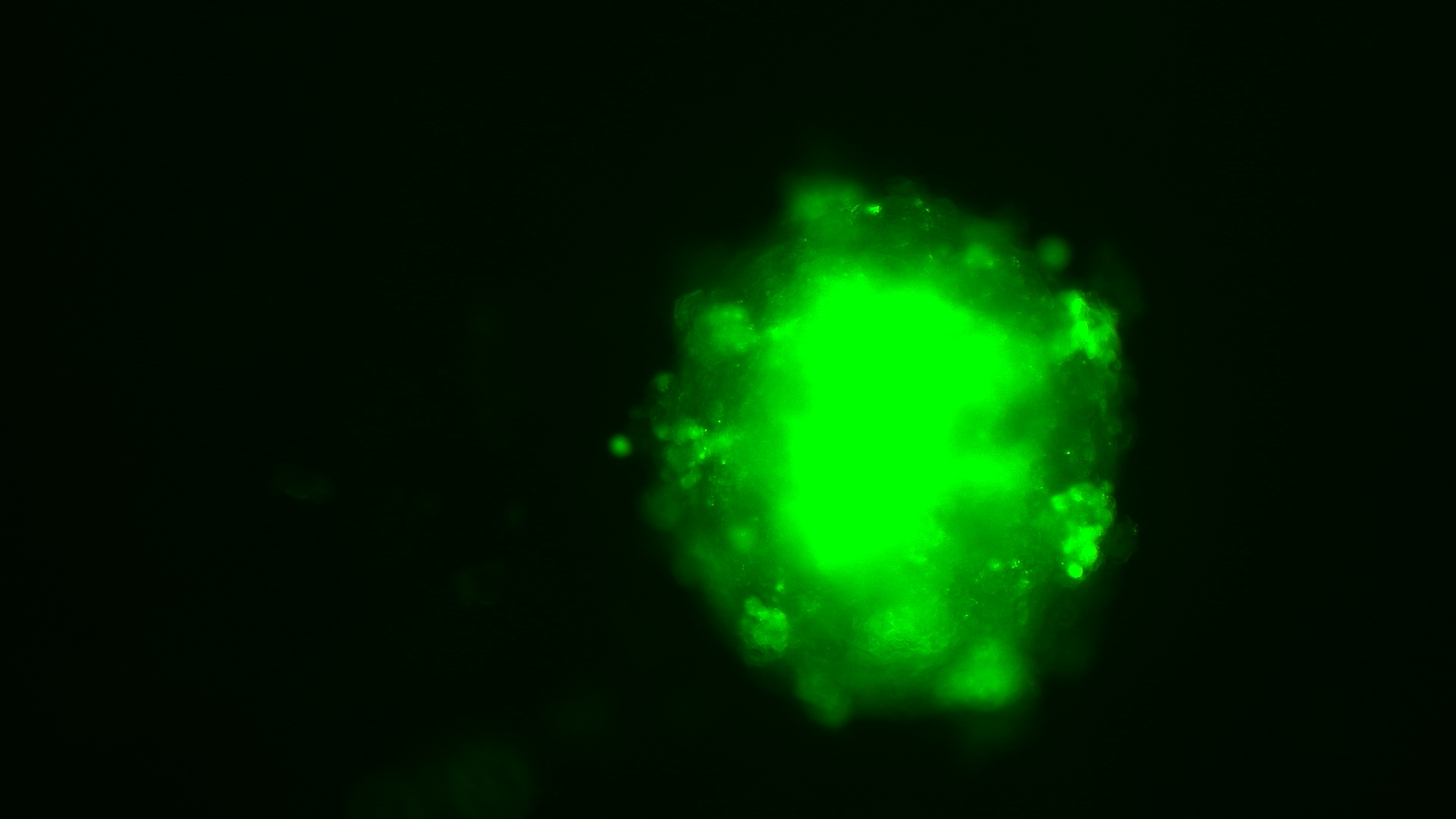

Supplement: Supplementary file 6 — Source data Fig. 2 [file 44319_2026_751_MOESM6_ESM.zip › Raw_data_Figure 2/Figure 2E/selected/wtgreencalred221004144608ov.jpg]

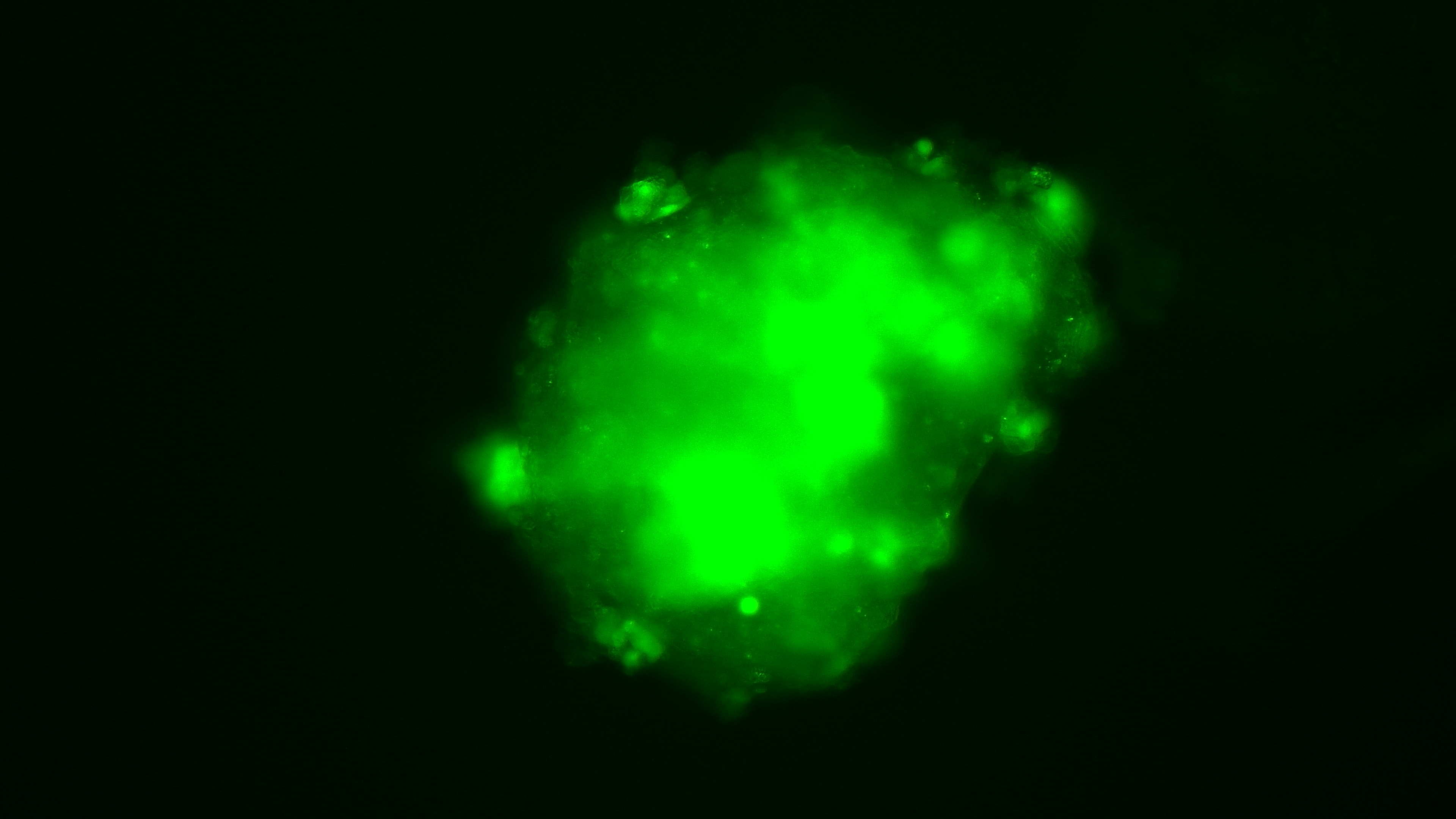

Supplement: Supplementary file 6 — Source data Fig. 2 [file 44319_2026_751_MOESM6_ESM.zip › Raw_data_Figure 2/Figure 2E/selected/wtgreencalred221004144747o.jpg]

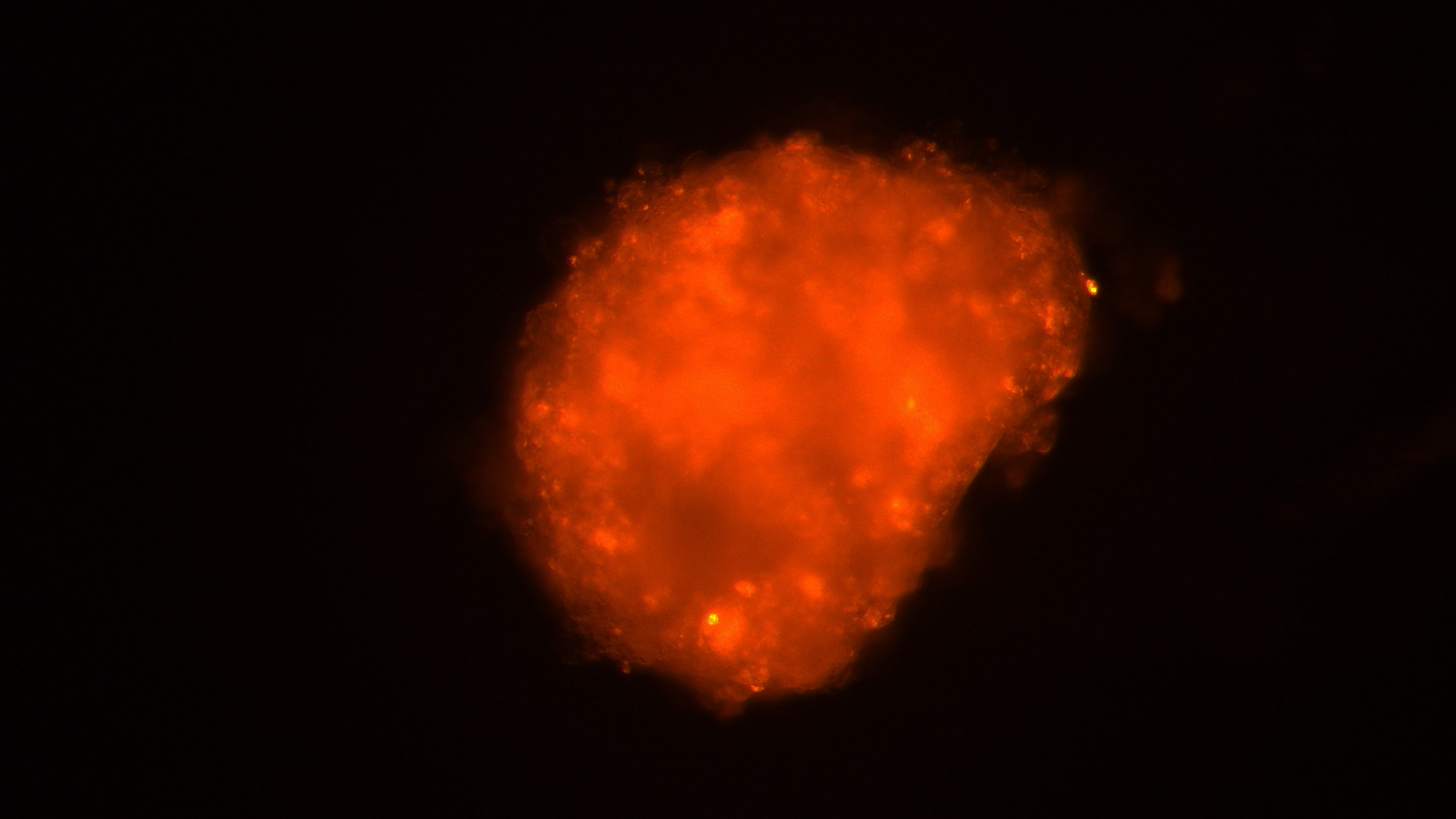

Supplement: Supplementary file 6 — Source data Fig. 2 [file 44319_2026_751_MOESM6_ESM.zip › Raw_data_Figure 2/Figure 2E/selected/wtgreencalred221004144753o.jpg]

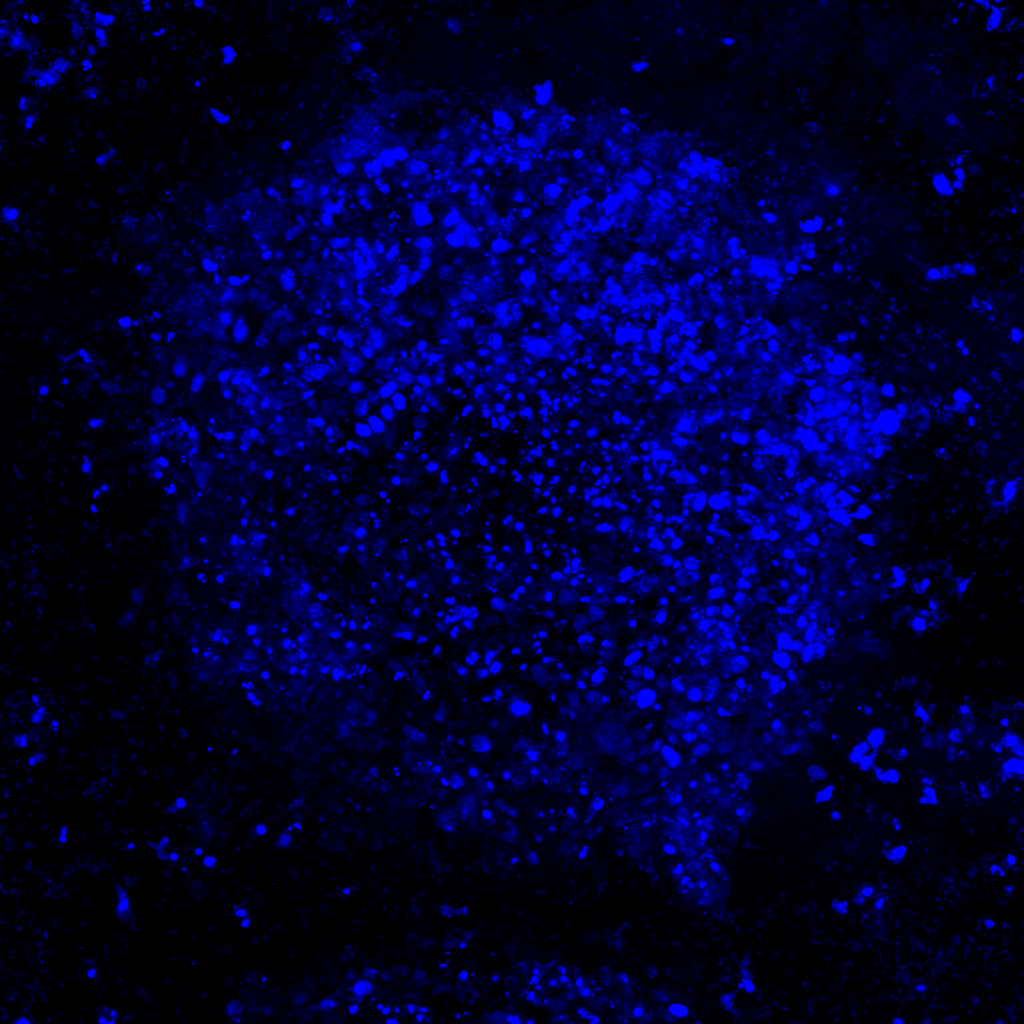

Supplement: Supplementary file 6 — Source data Fig. 2 [file 44319_2026_751_MOESM6_ESM.zip › Raw_data_Figure 2/Figure 2F/MAX_Esferoide rojo GqKO verde in matrig blue.tif]

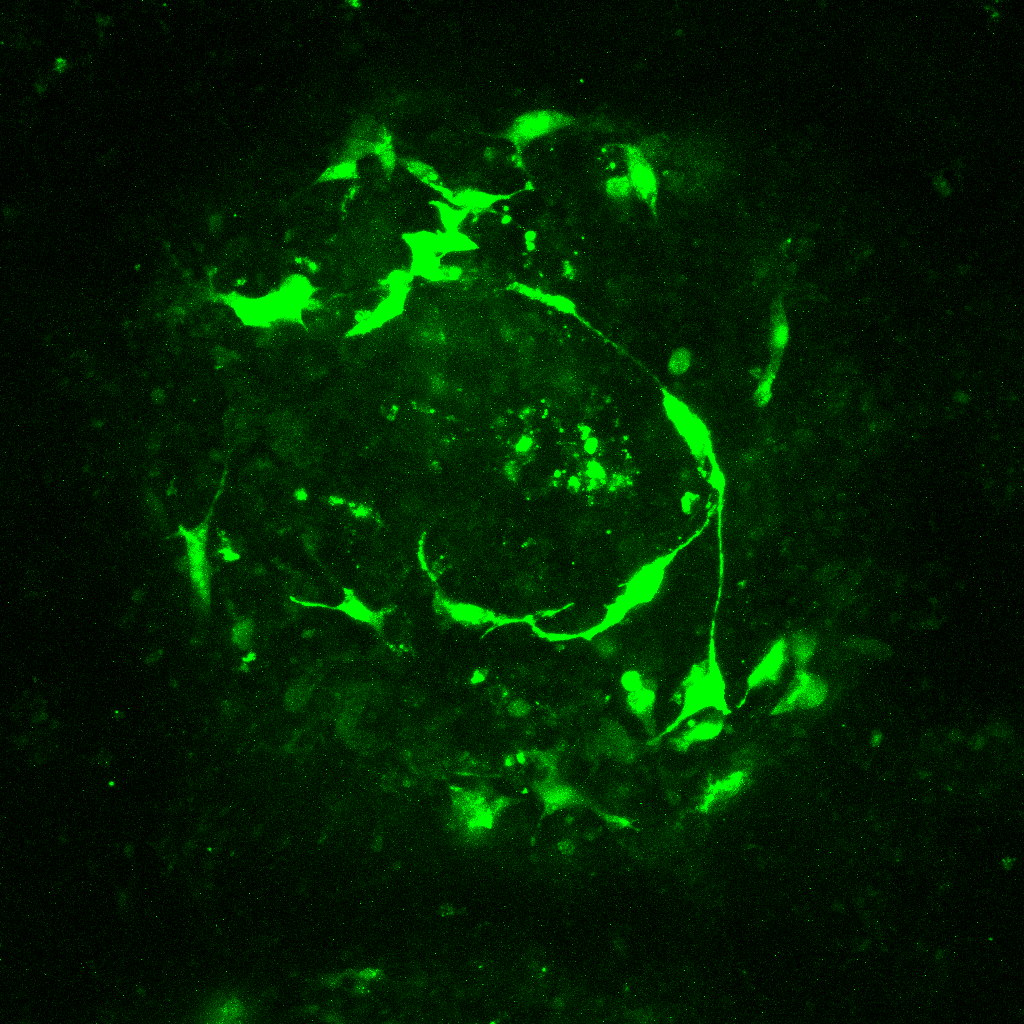

Supplement: Supplementary file 6 — Source data Fig. 2 [file 44319_2026_751_MOESM6_ESM.zip › Raw_data_Figure 2/Figure 2F/MAX_Esferoide rojo GqKO verde in matrig green.tif]

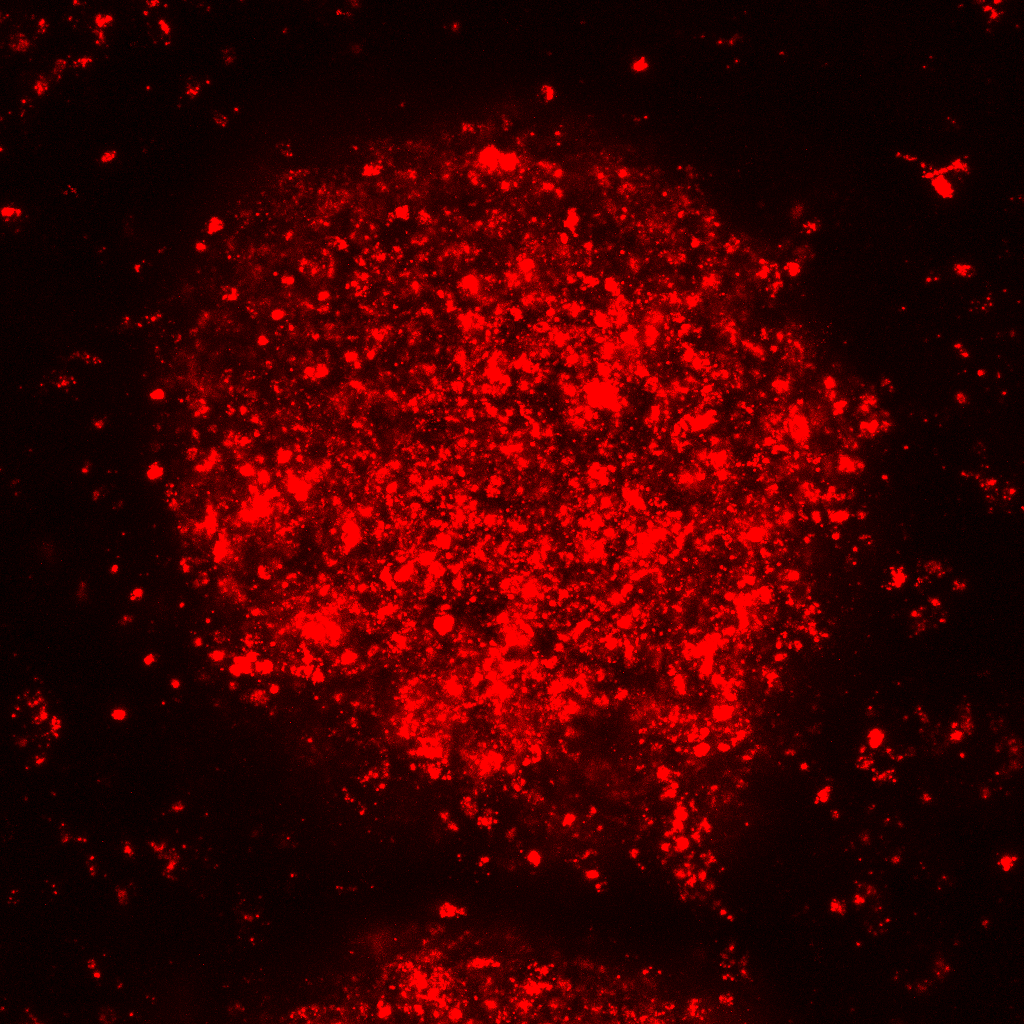

Supplement: Supplementary file 6 — Source data Fig. 2 [file 44319_2026_751_MOESM6_ESM.zip › Raw_data_Figure 2/Figure 2F/MAX_Esferoide rojo GqKO verde in matrig red.tif]

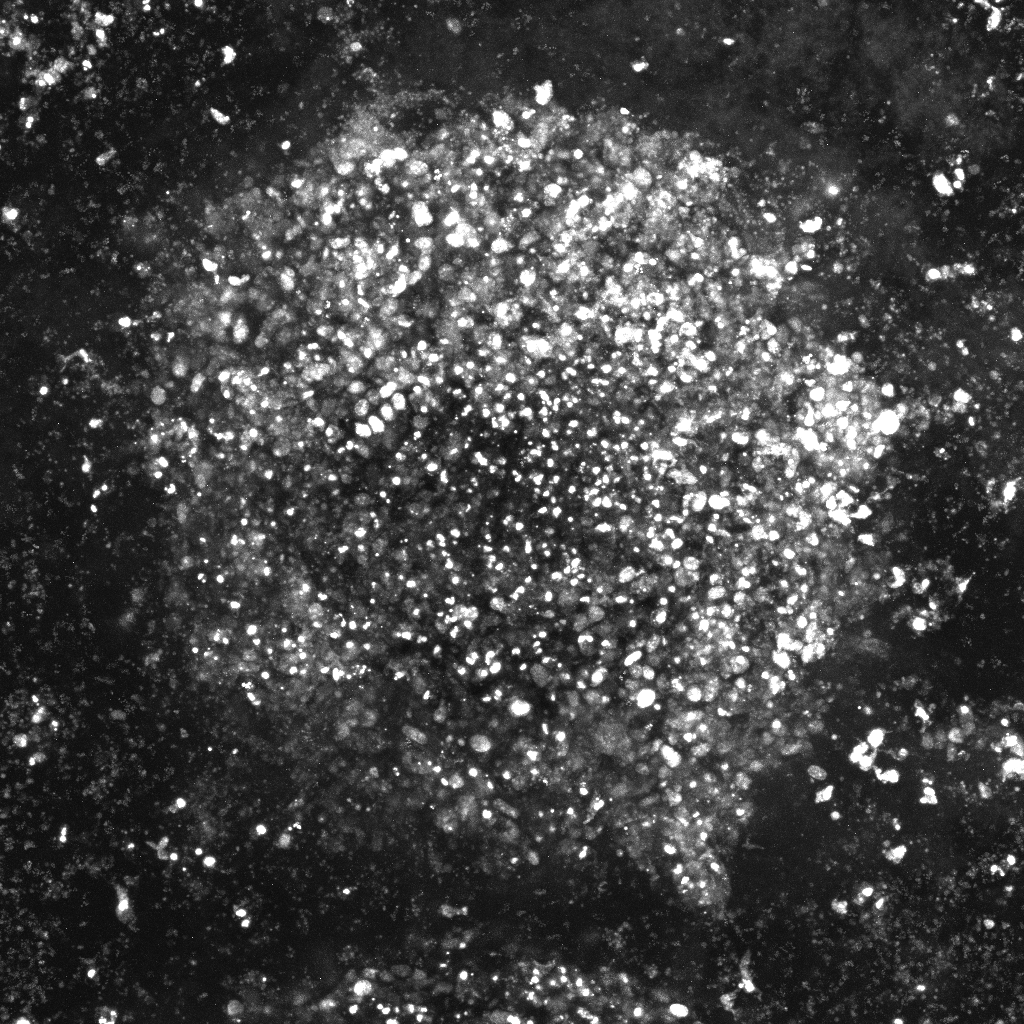

Supplement: Supplementary file 6 — Source data Fig. 2 [file 44319_2026_751_MOESM6_ESM.zip › Raw_data_Figure 2/Figure 2F/MAX_Esferoide rojo GqKO verde in matrig.tif]

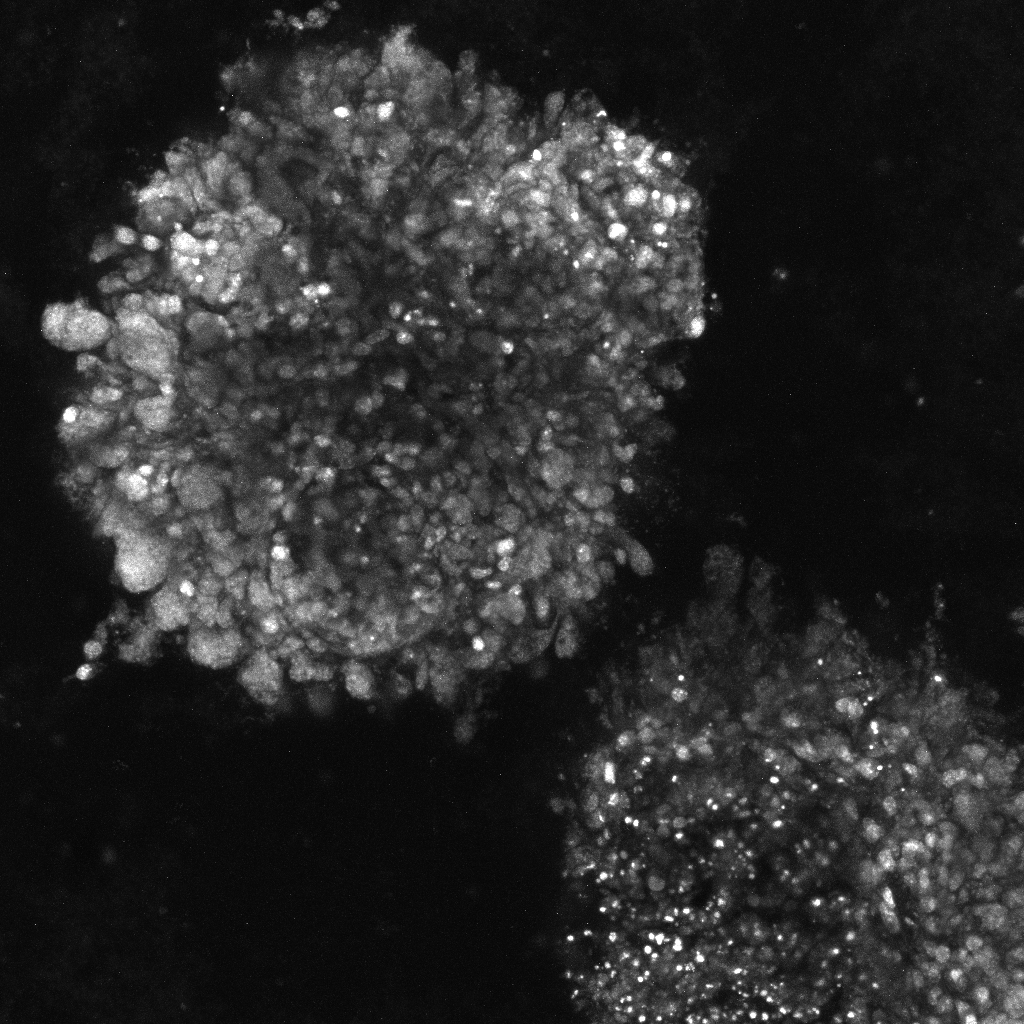

Supplement: Supplementary file 6 — Source data Fig. 2 [file 44319_2026_751_MOESM6_ESM.zip › Raw_data_Figure 2/Figure 2F/MAX_Esferoide rojo WT verde in matrig 2-1.tif]

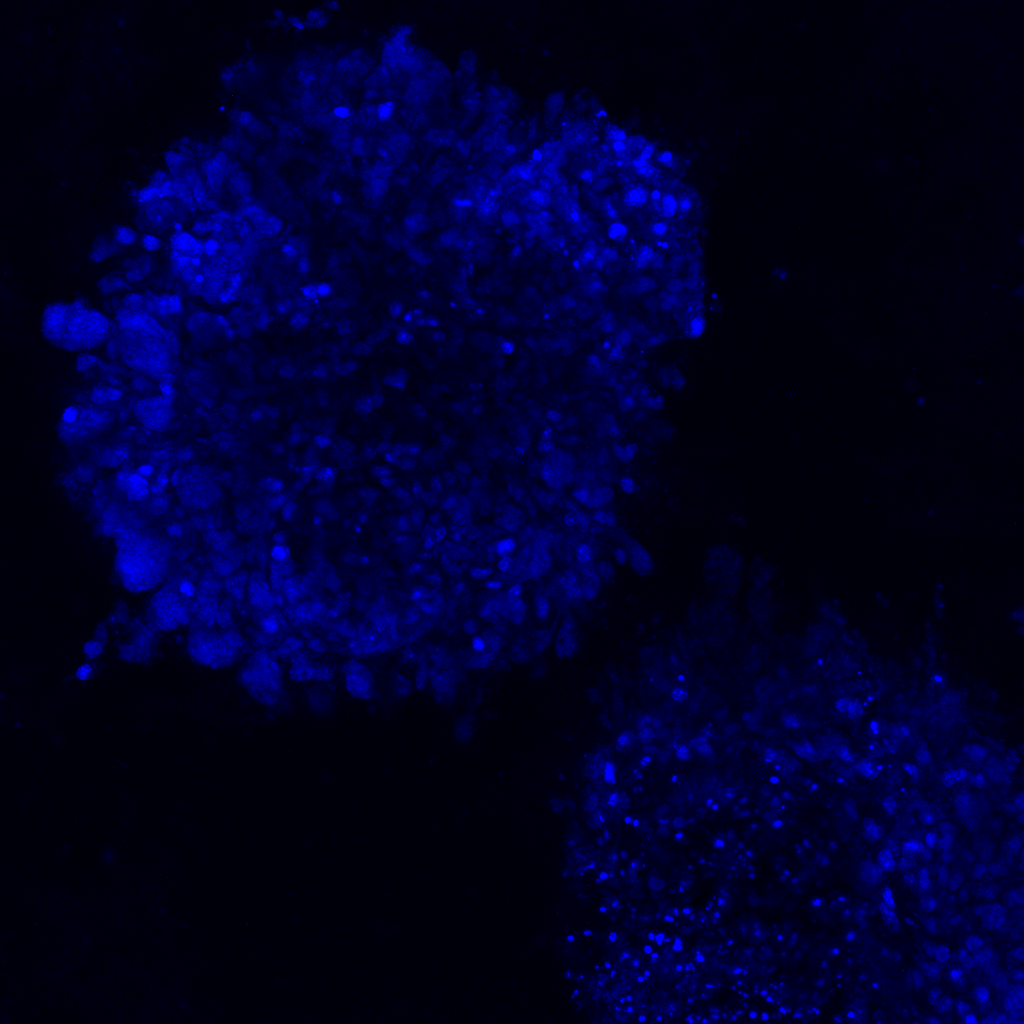

Supplement: Supplementary file 6 — Source data Fig. 2 [file 44319_2026_751_MOESM6_ESM.zip › Raw_data_Figure 2/Figure 2F/MAX_Esferoide rojo WT verde in matrig blue.tif]

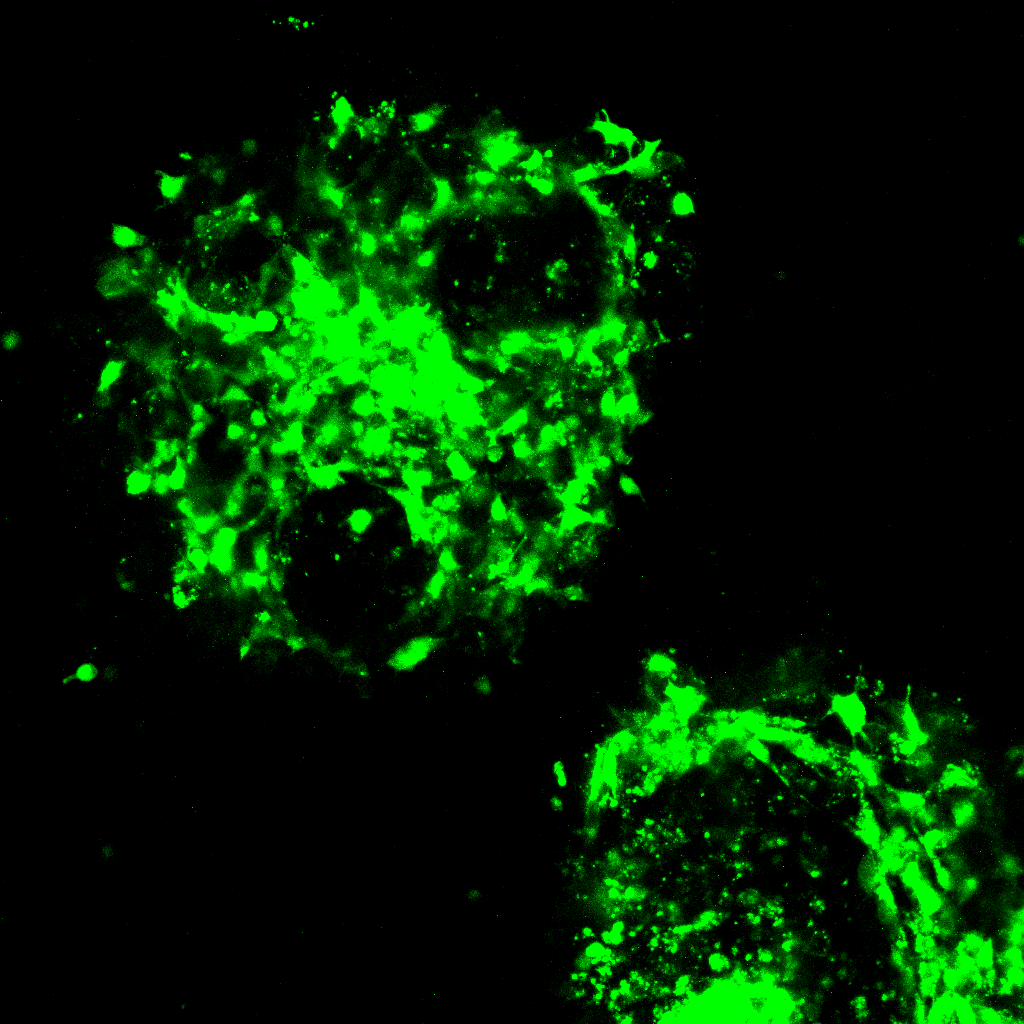

Supplement: Supplementary file 6 — Source data Fig. 2 [file 44319_2026_751_MOESM6_ESM.zip › Raw_data_Figure 2/Figure 2F/MAX_Esferoide rojo WT verde in matrig green.tif]

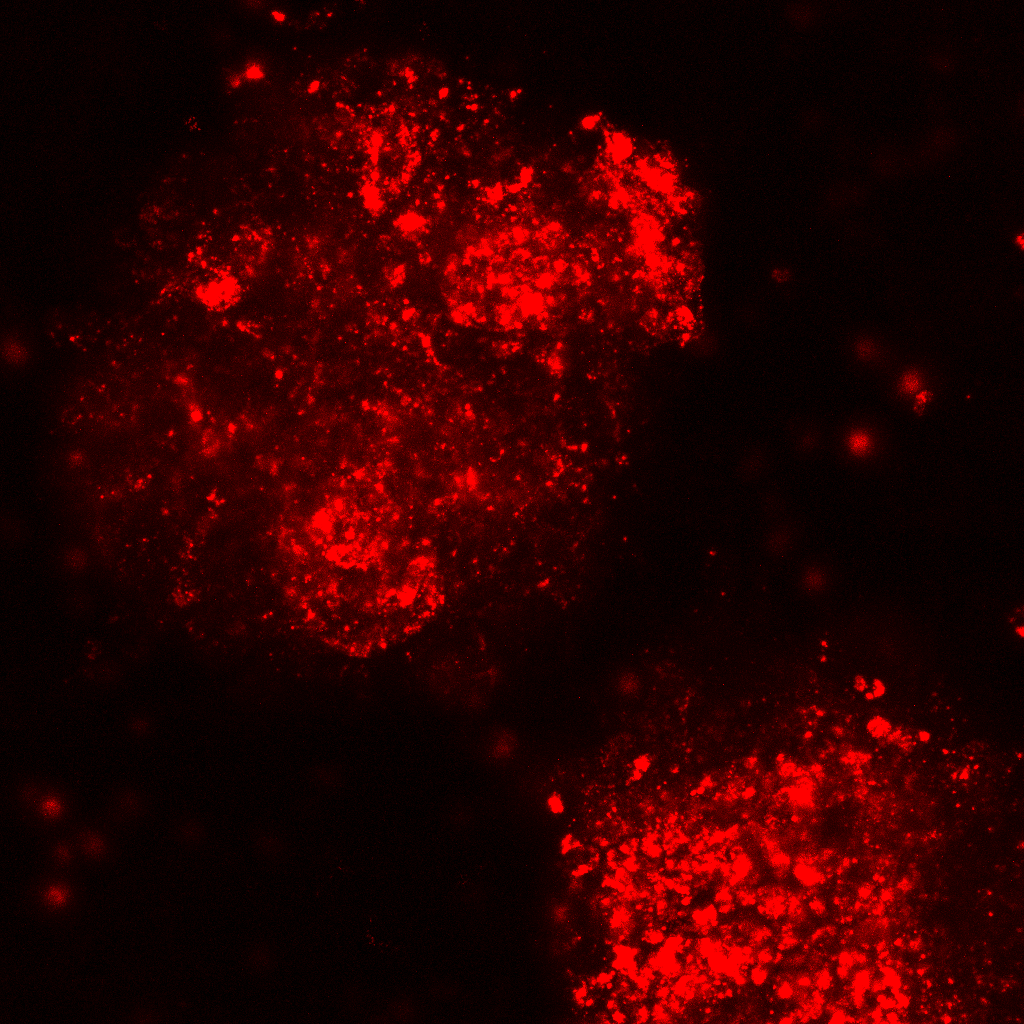

Supplement: Supplementary file 6 — Source data Fig. 2 [file 44319_2026_751_MOESM6_ESM.zip › Raw_data_Figure 2/Figure 2F/MAX_Esferoide rojo WT verde in matrig red.tif]

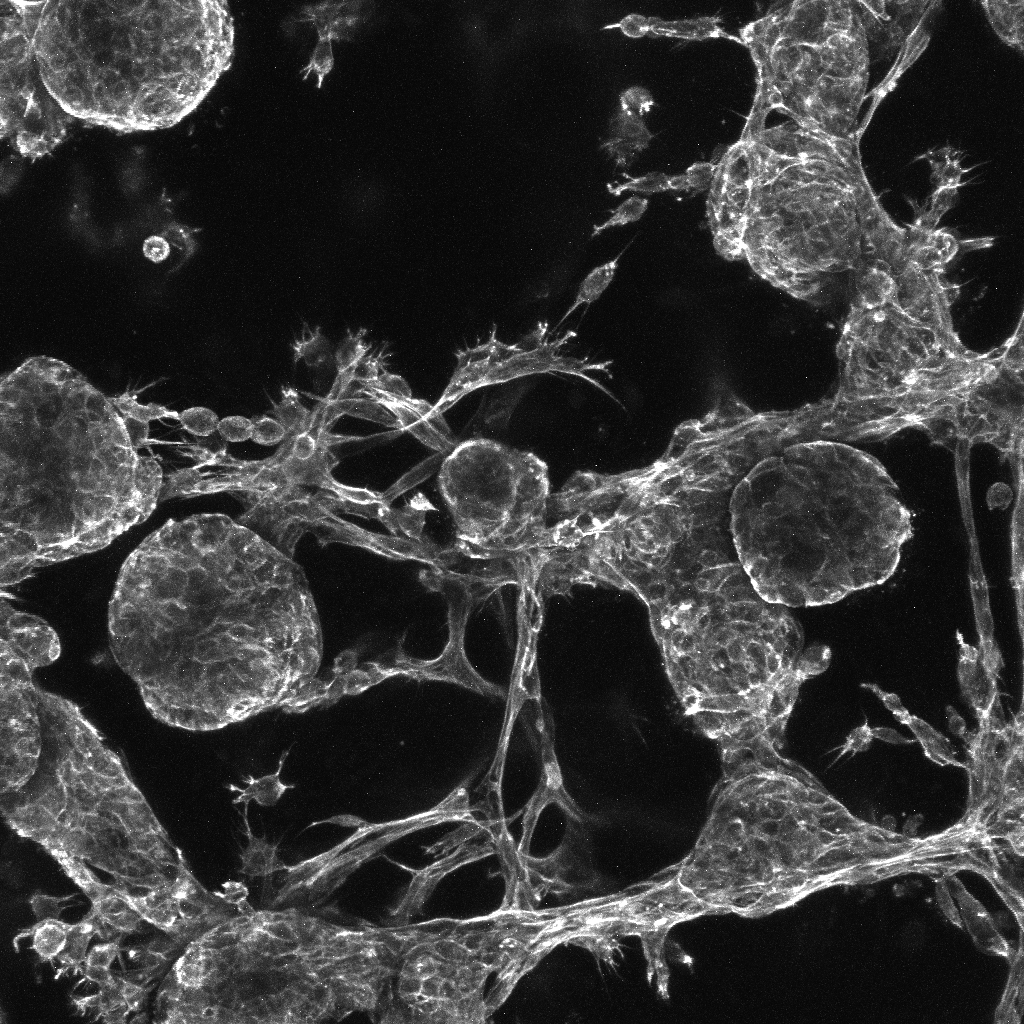

Supplement: Supplementary file 6 — Source data Fig. 2 [file 44319_2026_751_MOESM6_ESM.zip › Raw_data_Figure 2/Figure 2G/Cal27 GqKO invasion gray Factin.tif]

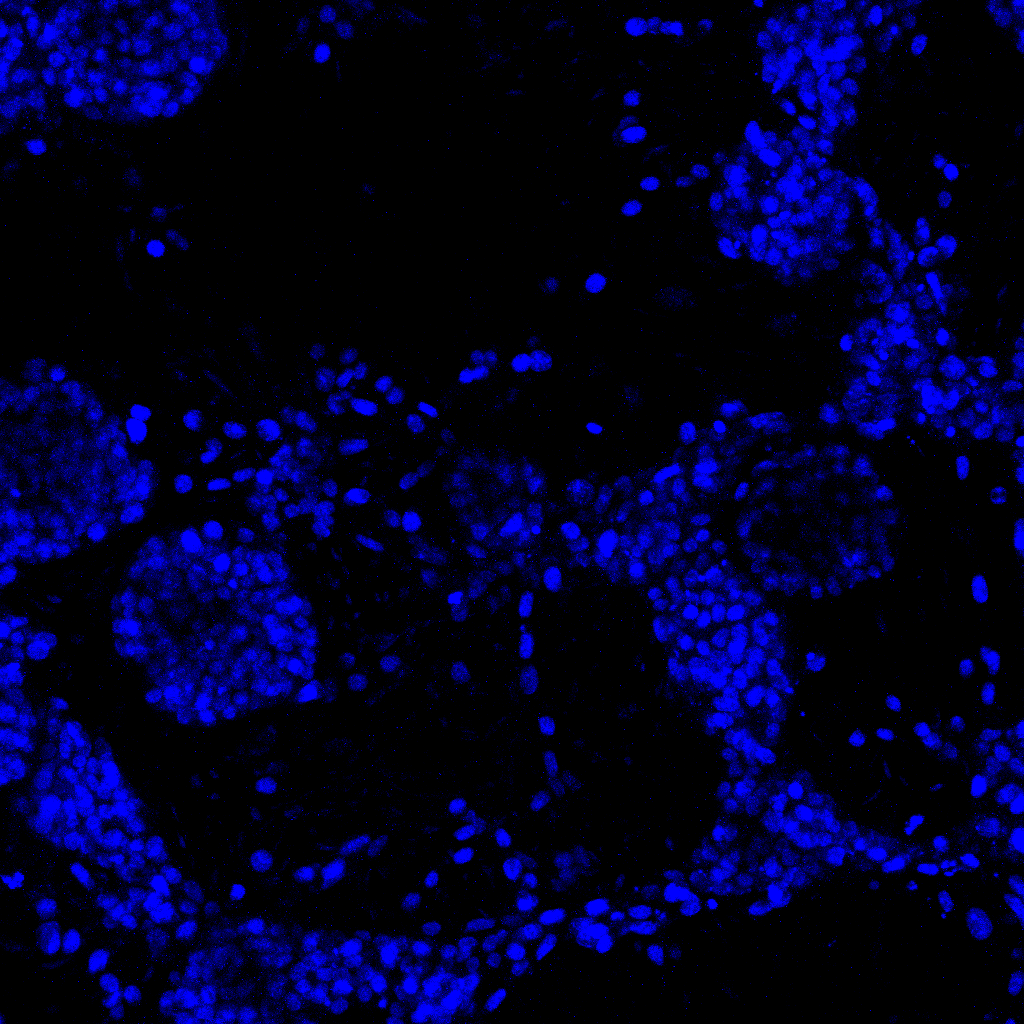

Supplement: Supplementary file 6 — Source data Fig. 2 [file 44319_2026_751_MOESM6_ESM.zip › Raw_data_Figure 2/Figure 2G/Cal27 GqKO invasion nuclei.tif]

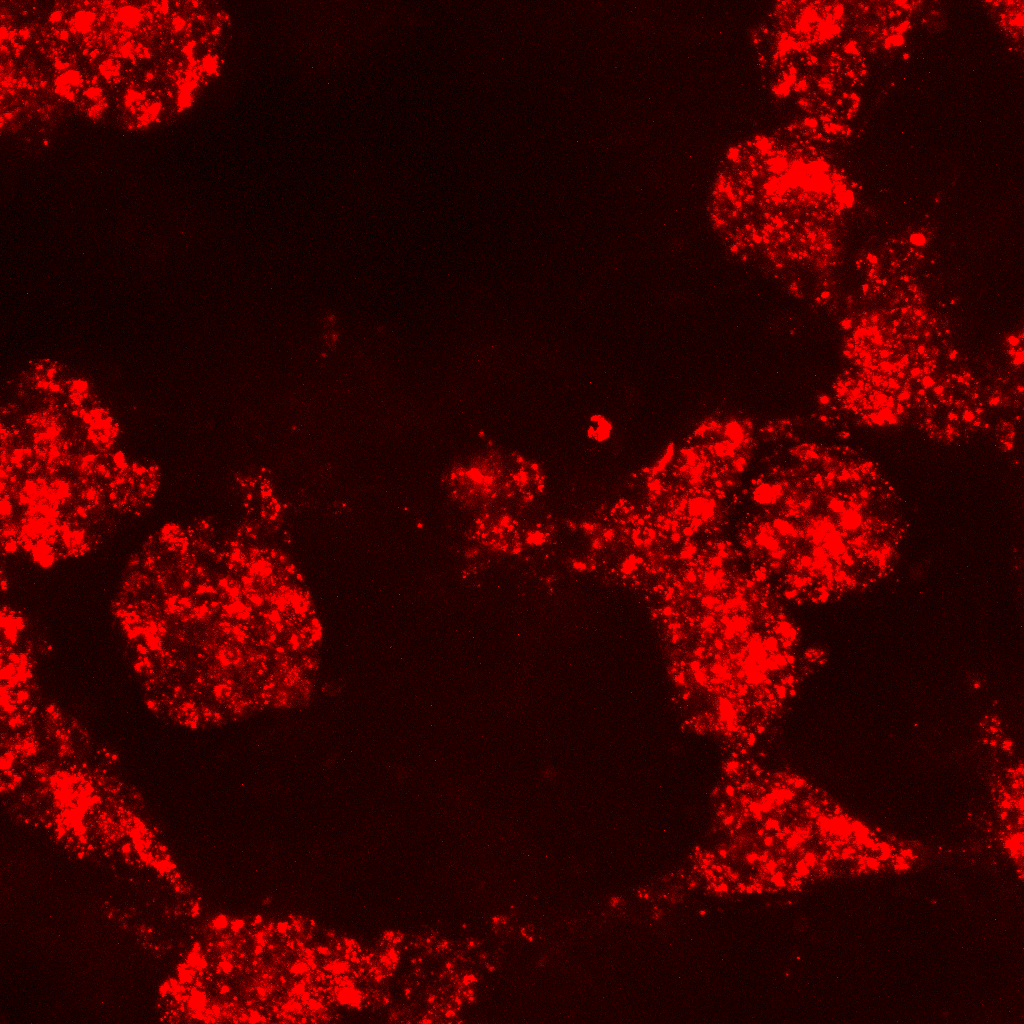

Supplement: Supplementary file 6 — Source data Fig. 2 [file 44319_2026_751_MOESM6_ESM.zip › Raw_data_Figure 2/Figure 2G/Cal27 GqKO invasion red.tif]

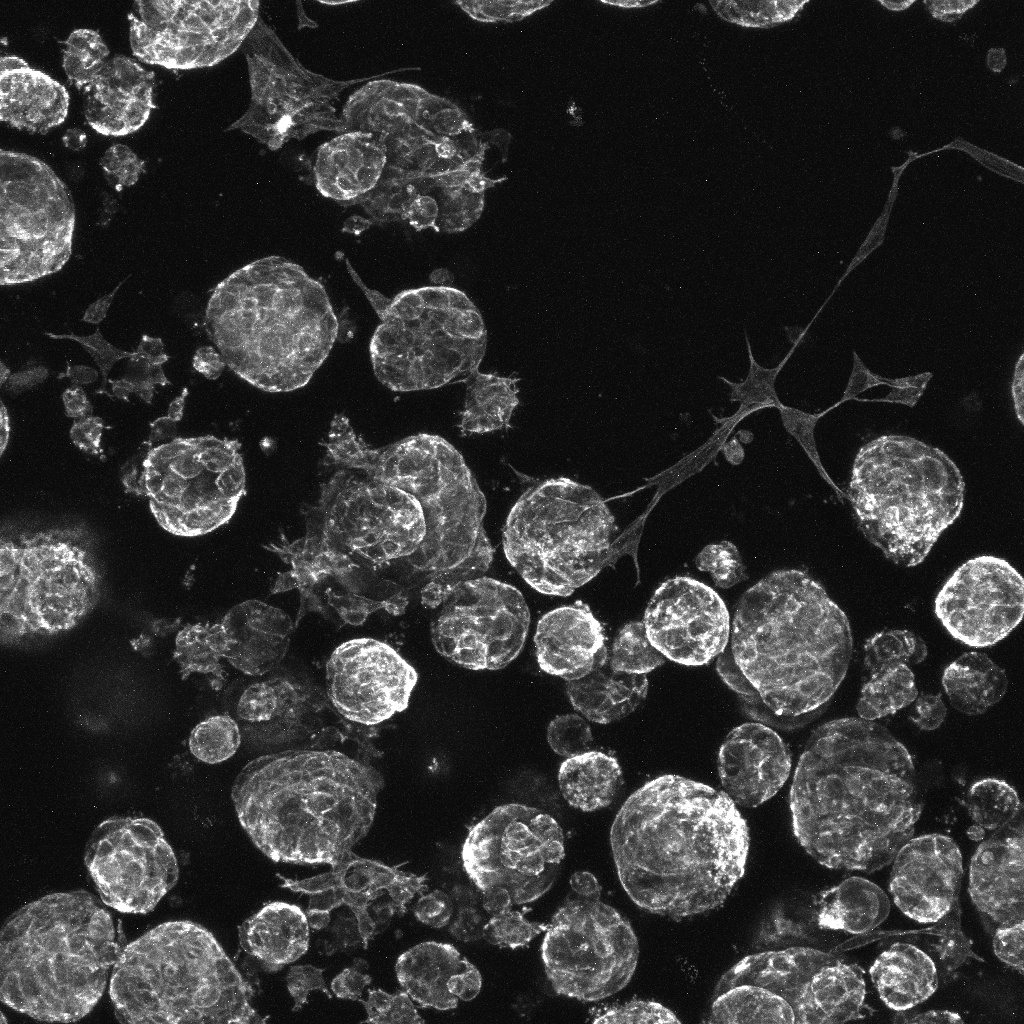

Supplement: Supplementary file 6 — Source data Fig. 2 [file 44319_2026_751_MOESM6_ESM.zip › Raw_data_Figure 2/Figure 2G/Cal27 WT invasion gray Factin.tif]

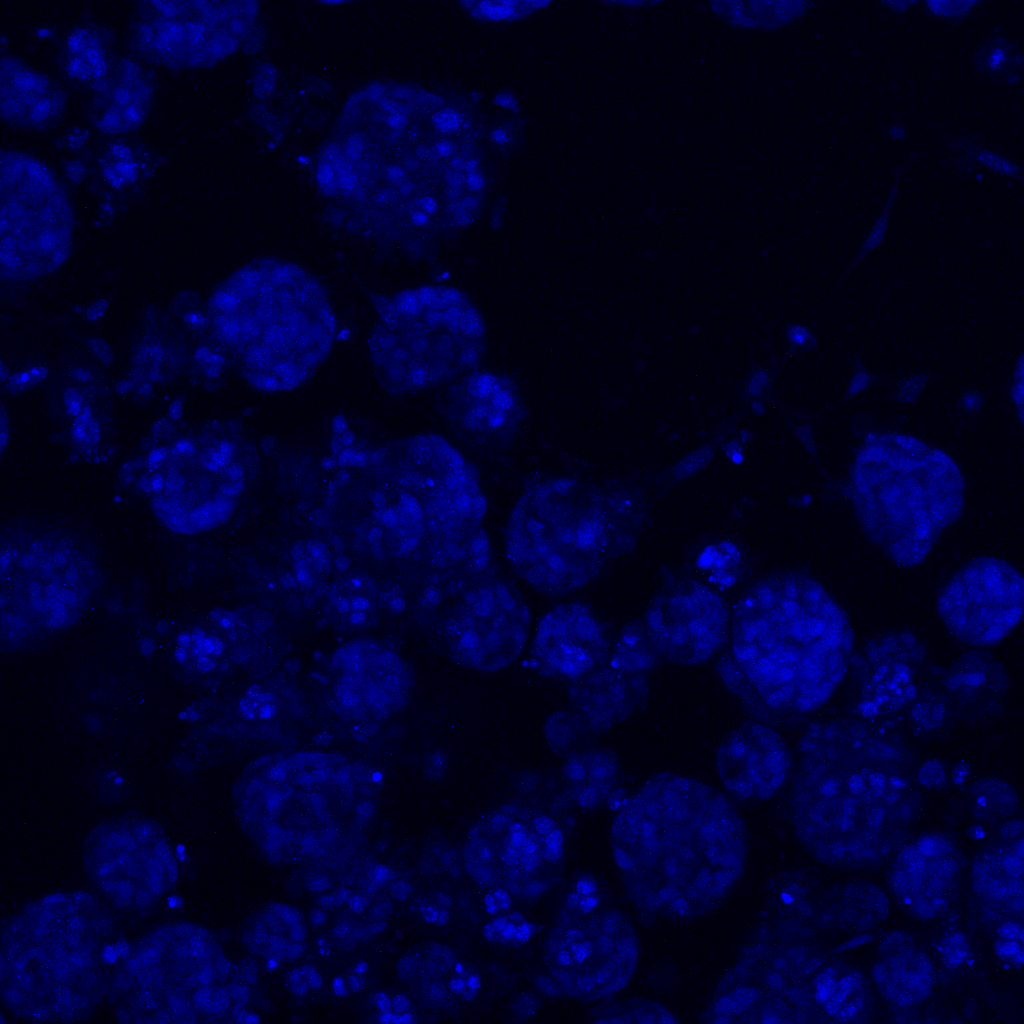

Supplement: Supplementary file 6 — Source data Fig. 2 [file 44319_2026_751_MOESM6_ESM.zip › Raw_data_Figure 2/Figure 2G/Cal27 WT invasion nuclei.tif]

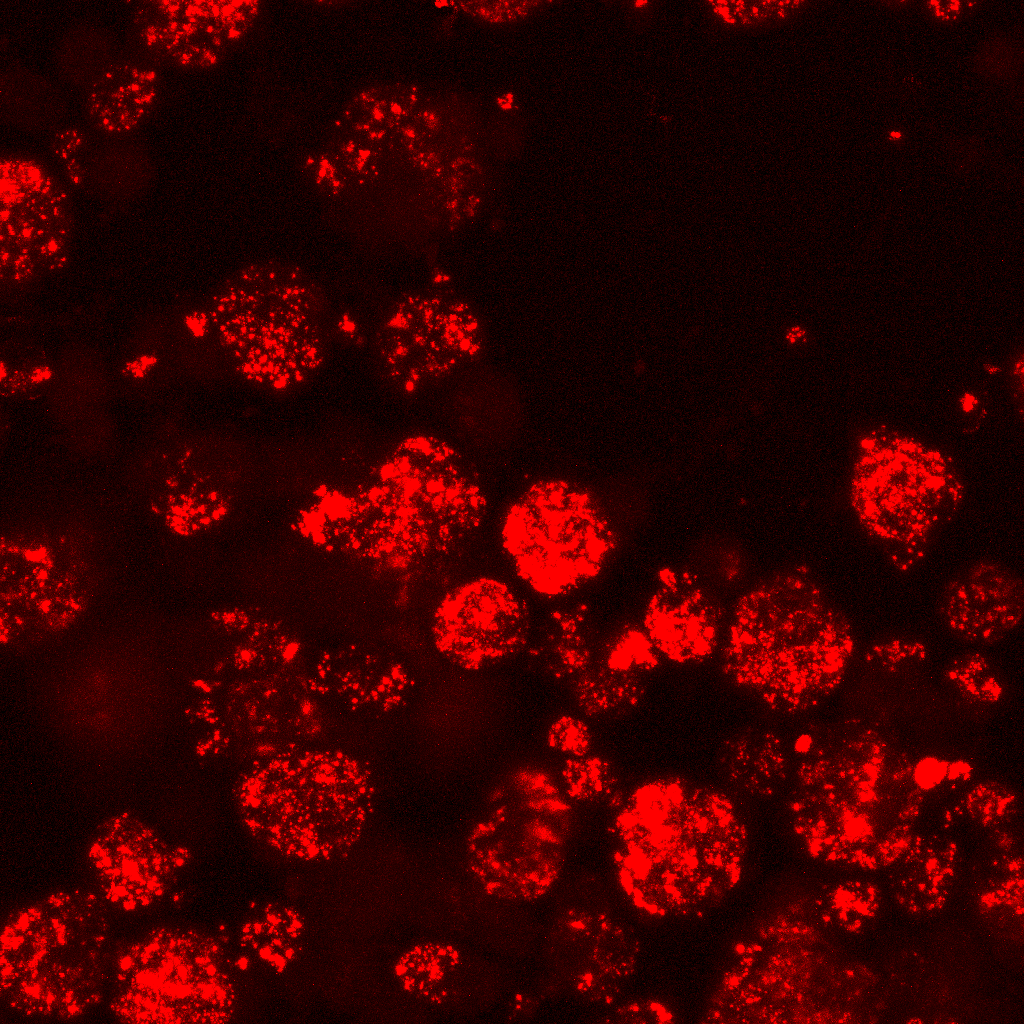

Supplement: Supplementary file 6 — Source data Fig. 2 [file 44319_2026_751_MOESM6_ESM.zip › Raw_data_Figure 2/Figure 2G/Cal27 WT invasion red.tif]

## Slide 1
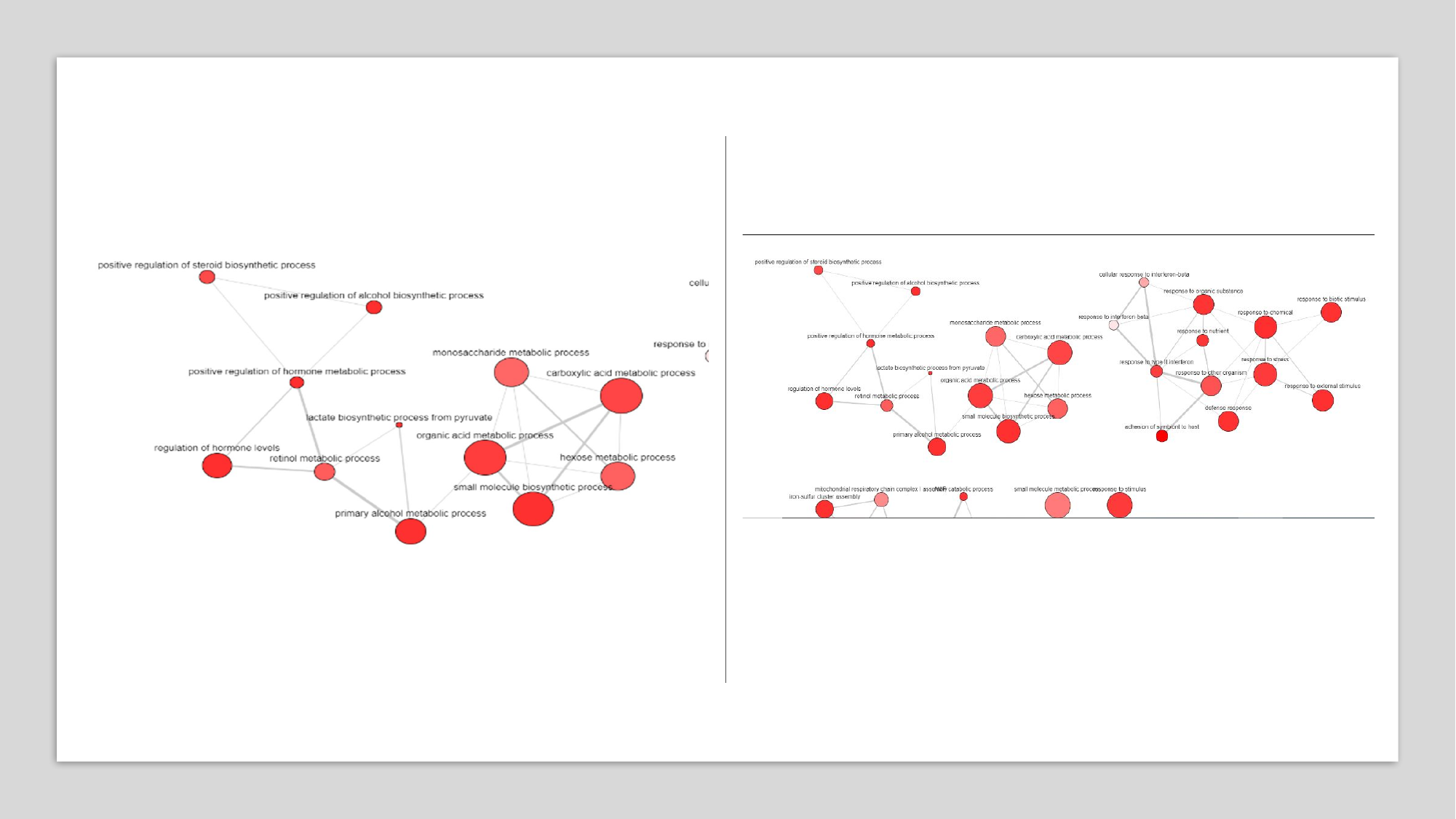

Supplement: Supplementary file 7 — Source data Fig. 3 [file 44319_2026_751_MOESM7_ESM.zip › Raw_data_Figure 3/Figure 3B/Diagrama revigo.pptx]

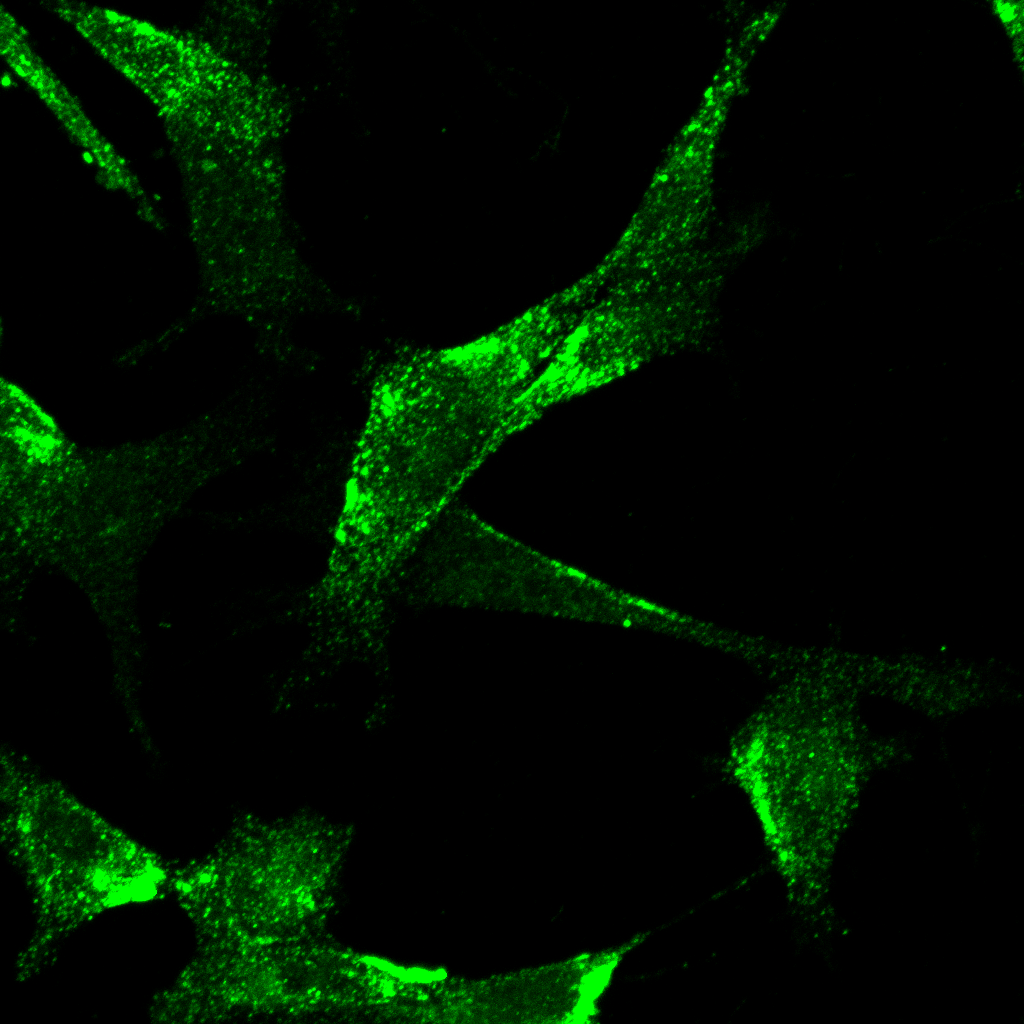

Supplement: Supplementary file 7 — Source data Fig. 3 [file 44319_2026_751_MOESM7_ESM.zip › Raw_data_Figure 3/Figure 3D/GqKO cav1 .tif]

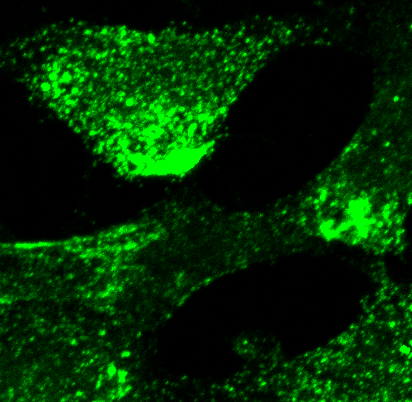

Supplement: Supplementary file 7 — Source data Fig. 3 [file 44319_2026_751_MOESM7_ESM.zip › Raw_data_Figure 3/Figure 3D/GqKO cav1 2 cropped.tif]

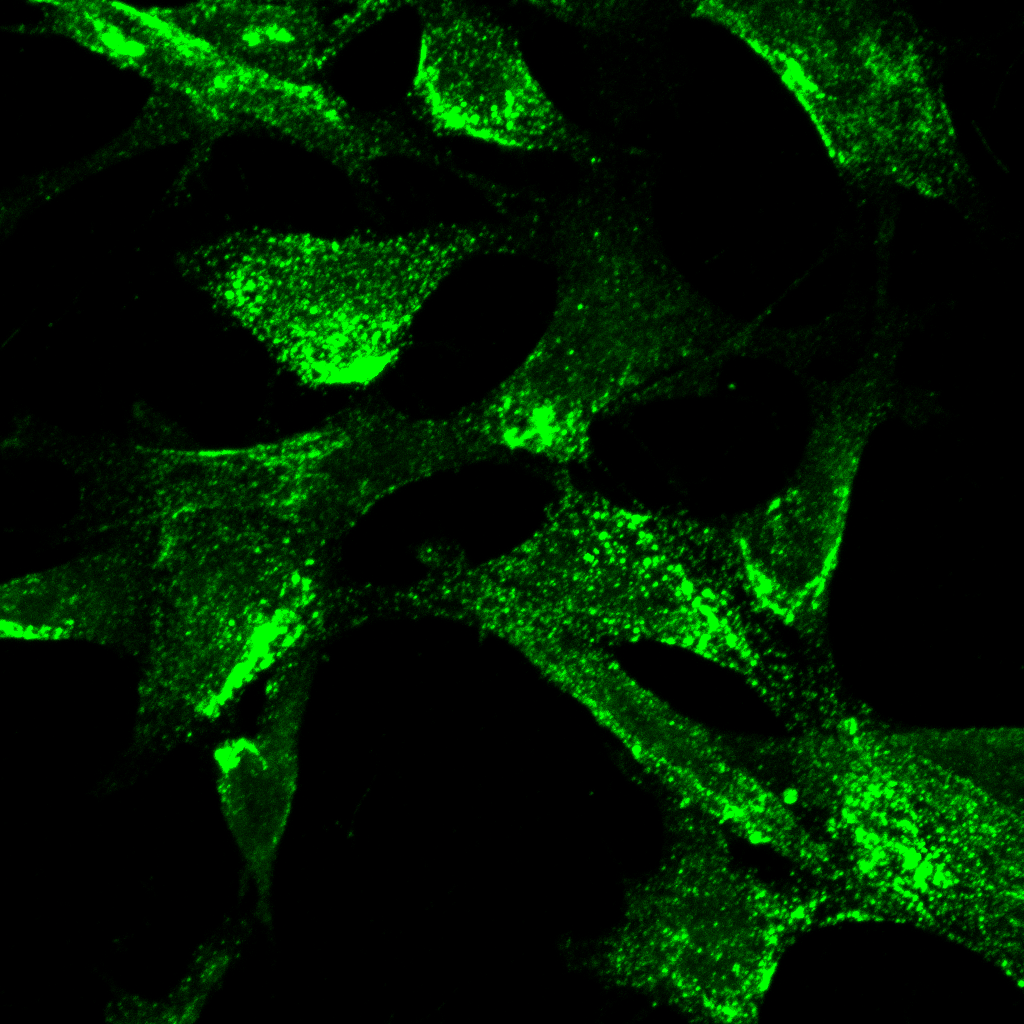

Supplement: Supplementary file 7 — Source data Fig. 3 [file 44319_2026_751_MOESM7_ESM.zip › Raw_data_Figure 3/Figure 3D/GqKO cav1 2.tif]

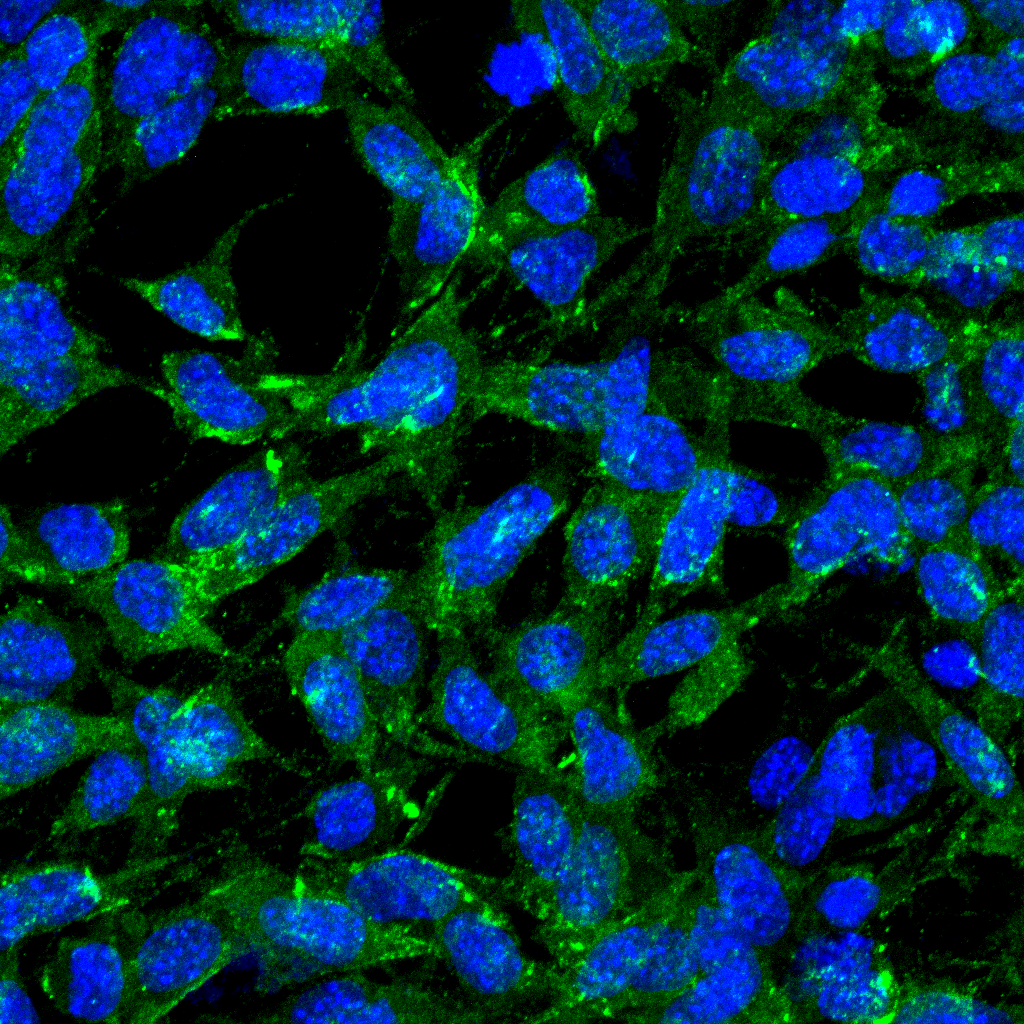

Supplement: Supplementary file 7 — Source data Fig. 3 [file 44319_2026_751_MOESM7_ESM.zip › Raw_data_Figure 3/Figure 3D/GqKO cav1 en confluencia A merge.tif]

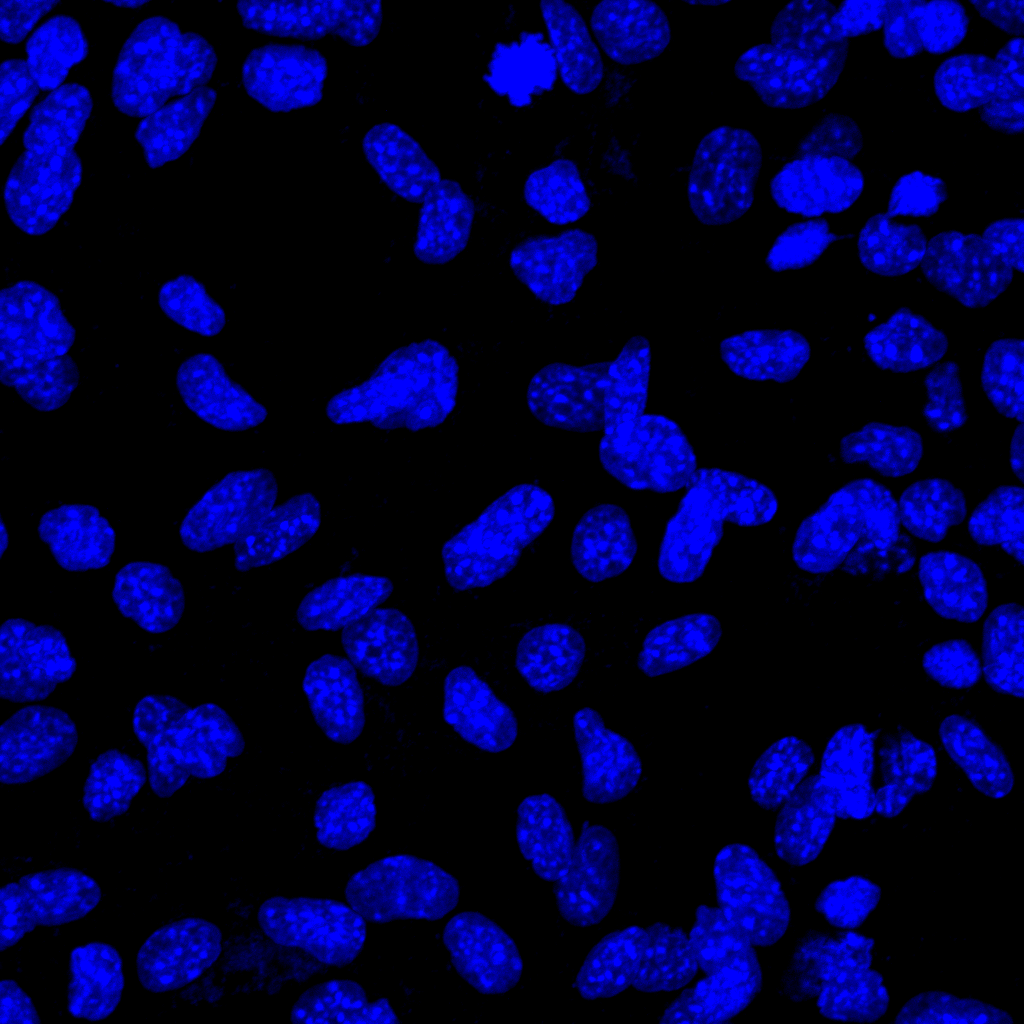

Supplement: Supplementary file 7 — Source data Fig. 3 [file 44319_2026_751_MOESM7_ESM.zip › Raw_data_Figure 3/Figure 3D/GqKO cav1 en confluencia A nuclei.tif]

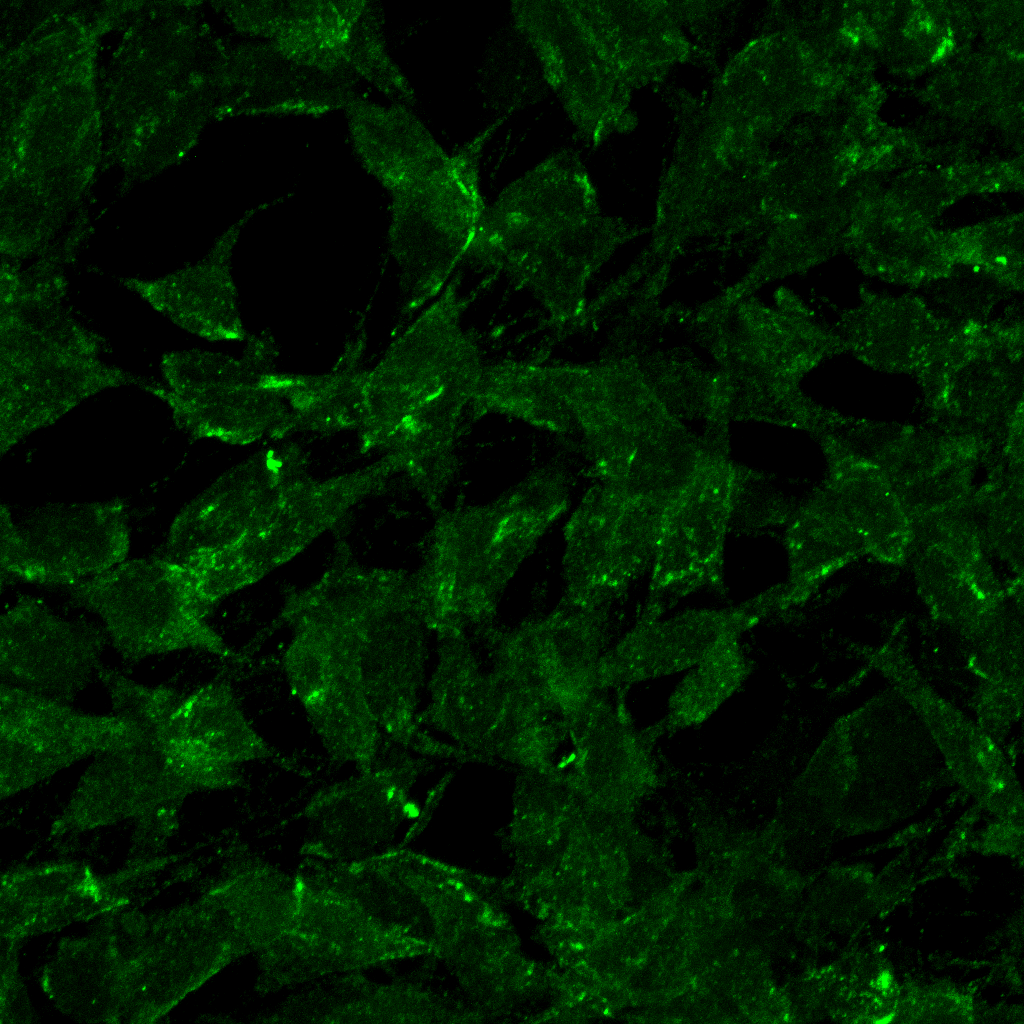

Supplement: Supplementary file 7 — Source data Fig. 3 [file 44319_2026_751_MOESM7_ESM.zip › Raw_data_Figure 3/Figure 3D/GqKO cav1 en confluencia A.tif]

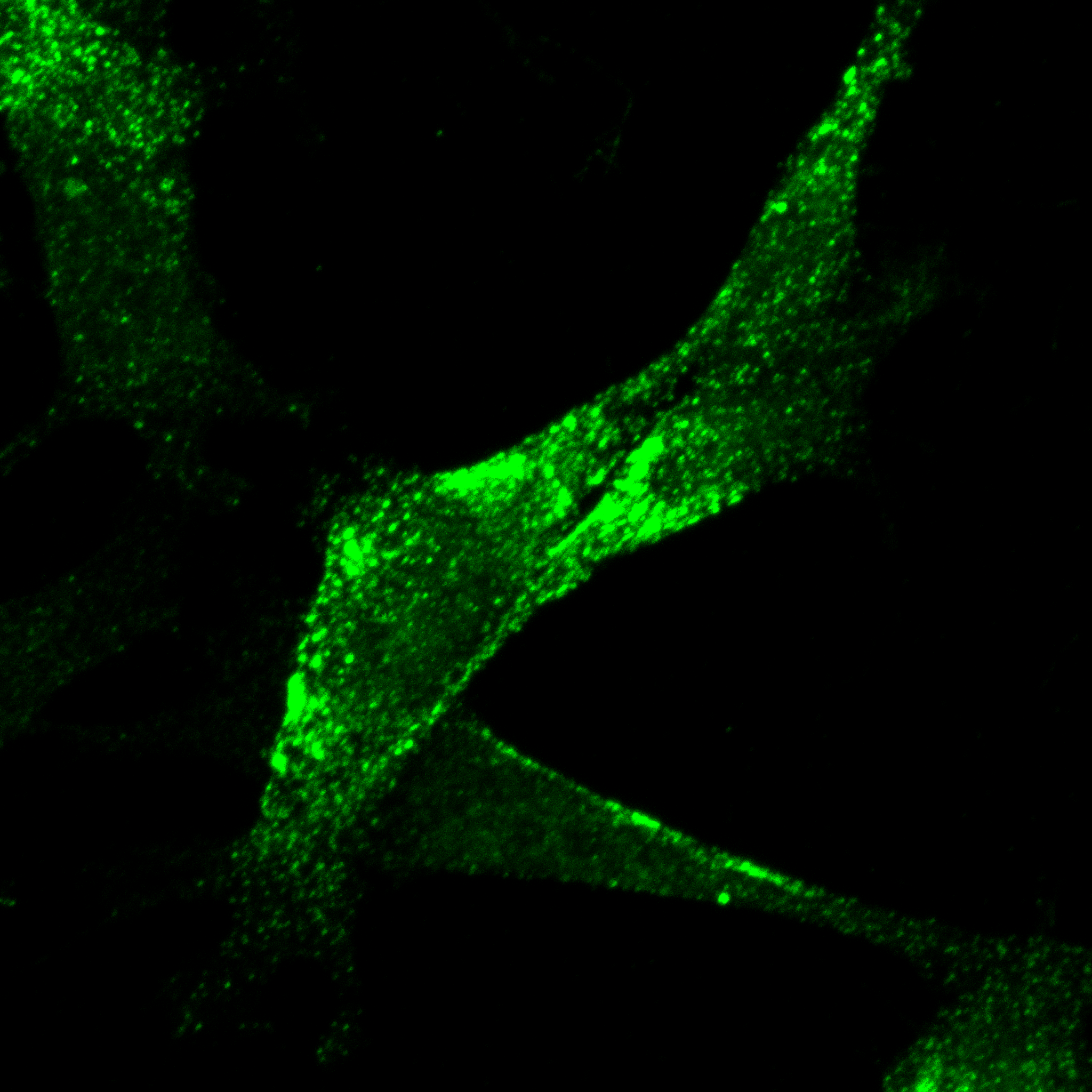

Supplement: Supplementary file 7 — Source data Fig. 3 [file 44319_2026_751_MOESM7_ESM.zip › Raw_data_Figure 3/Figure 3D/GqKO cav1 zoom .tif]

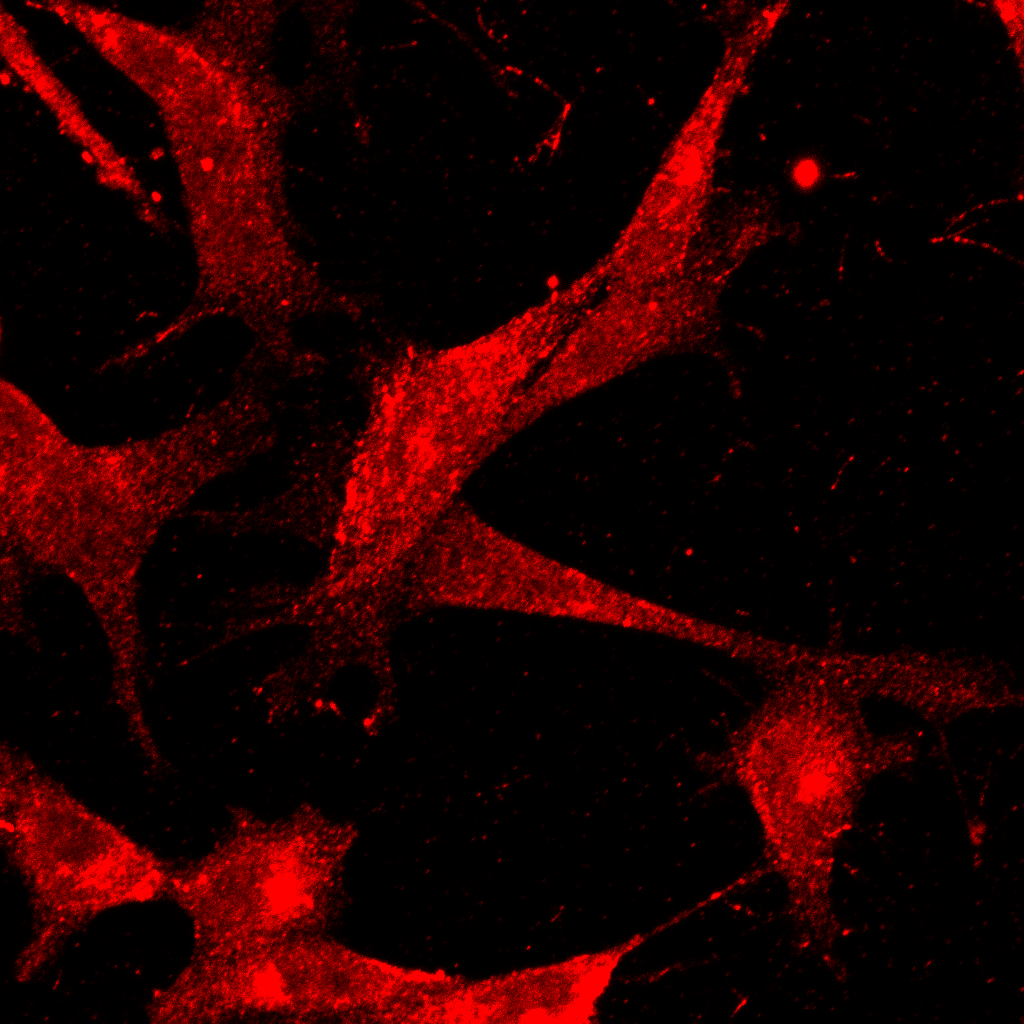

Supplement: Supplementary file 7 — Source data Fig. 3 [file 44319_2026_751_MOESM7_ESM.zip › Raw_data_Figure 3/Figure 3D/GqKO cav2 .tif]

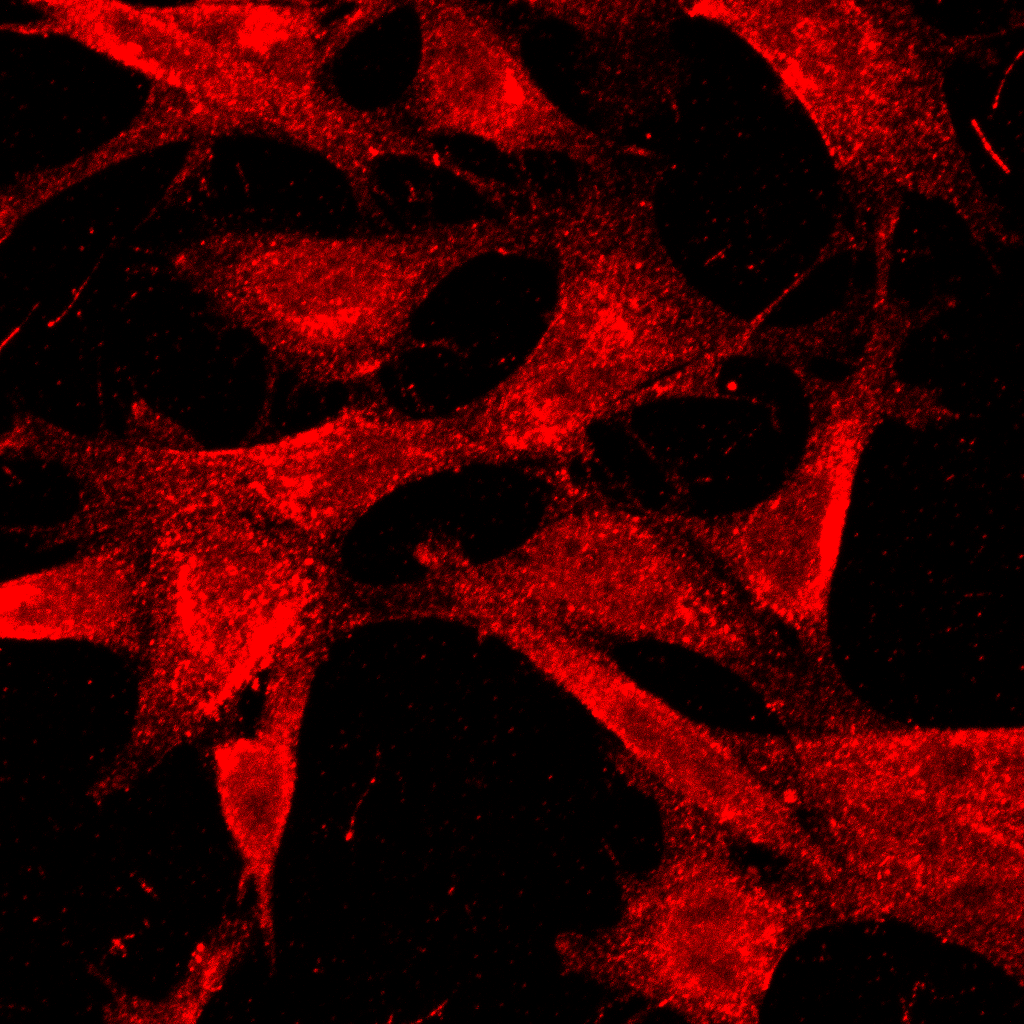

Supplement: Supplementary file 7 — Source data Fig. 3 [file 44319_2026_751_MOESM7_ESM.zip › Raw_data_Figure 3/Figure 3D/GqKO cav2 2.tif]

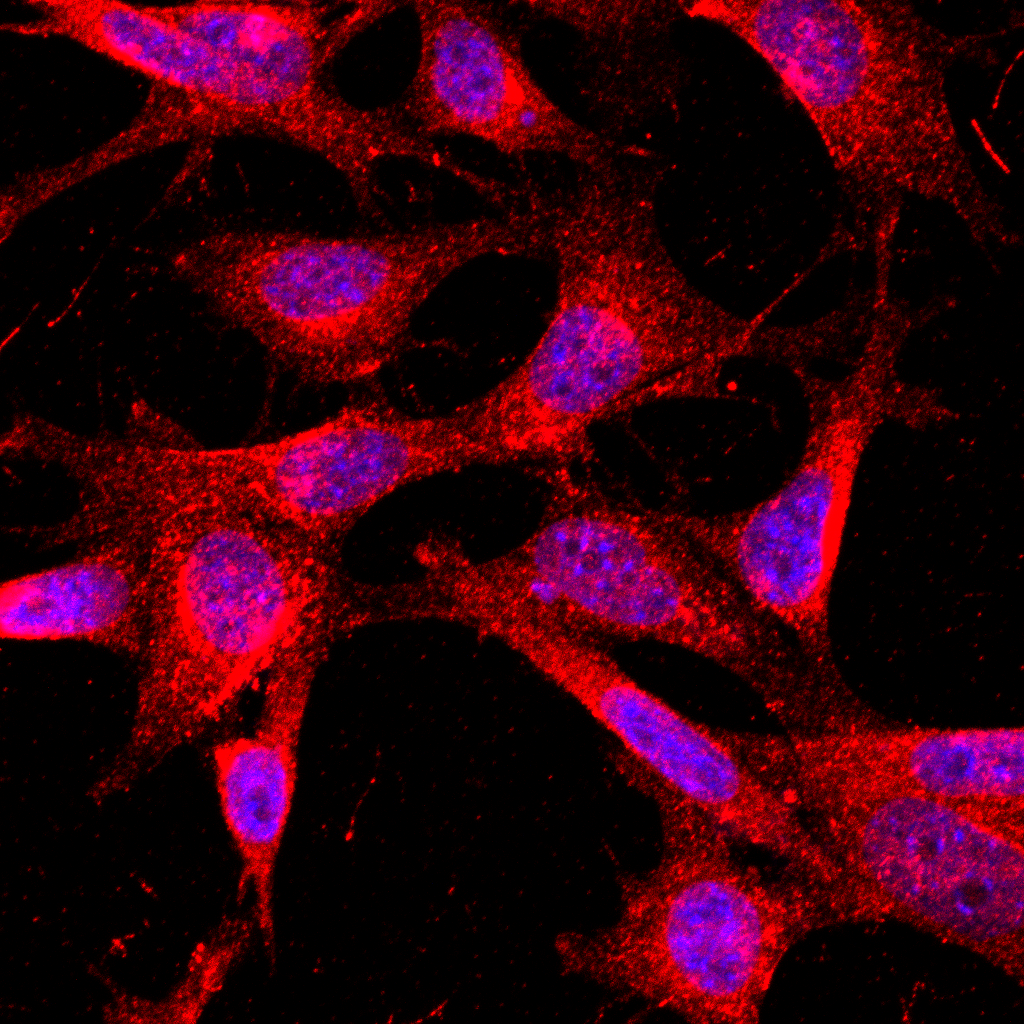

Supplement: Supplementary file 7 — Source data Fig. 3 [file 44319_2026_751_MOESM7_ESM.zip › Raw_data_Figure 3/Figure 3D/GqKO Cav2 nuclei 2.tif]

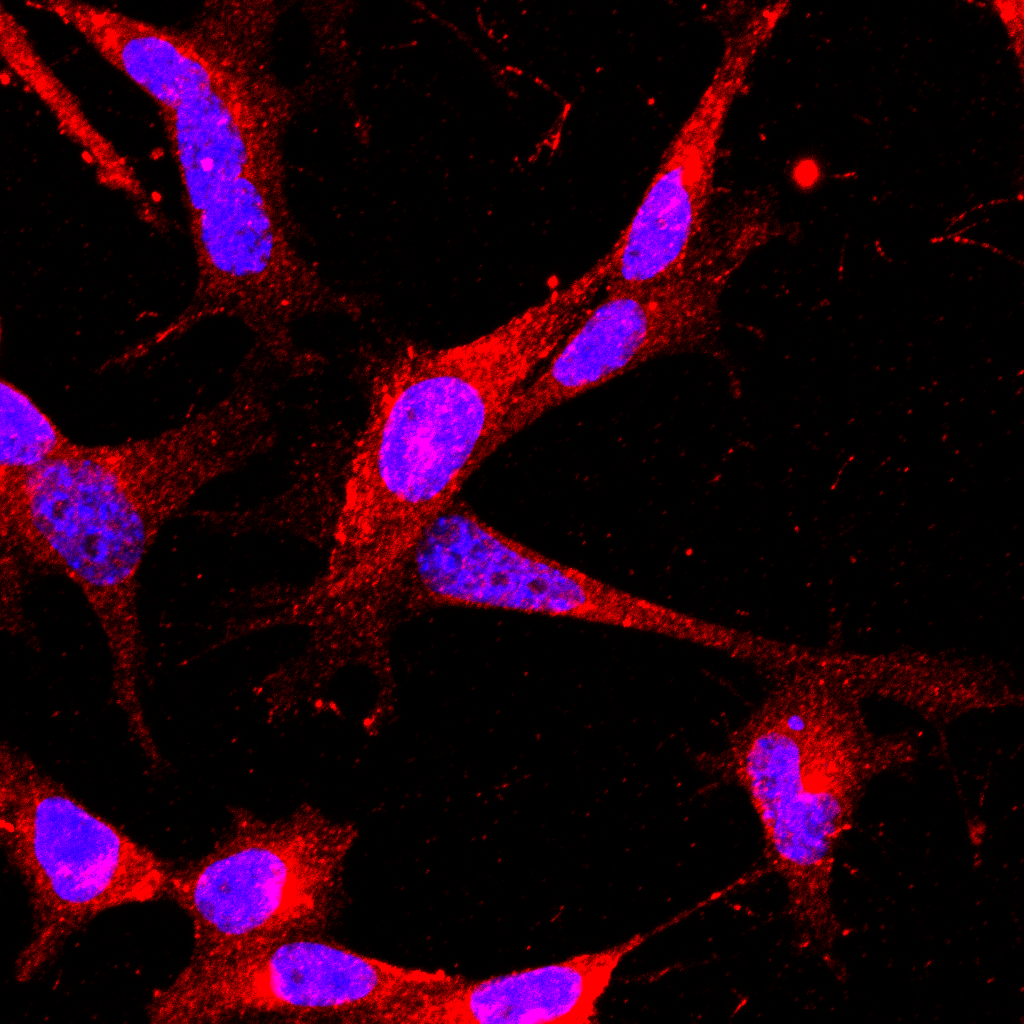

Supplement: Supplementary file 7 — Source data Fig. 3 [file 44319_2026_751_MOESM7_ESM.zip › Raw_data_Figure 3/Figure 3D/GqKO cav2 nuclei.tif]

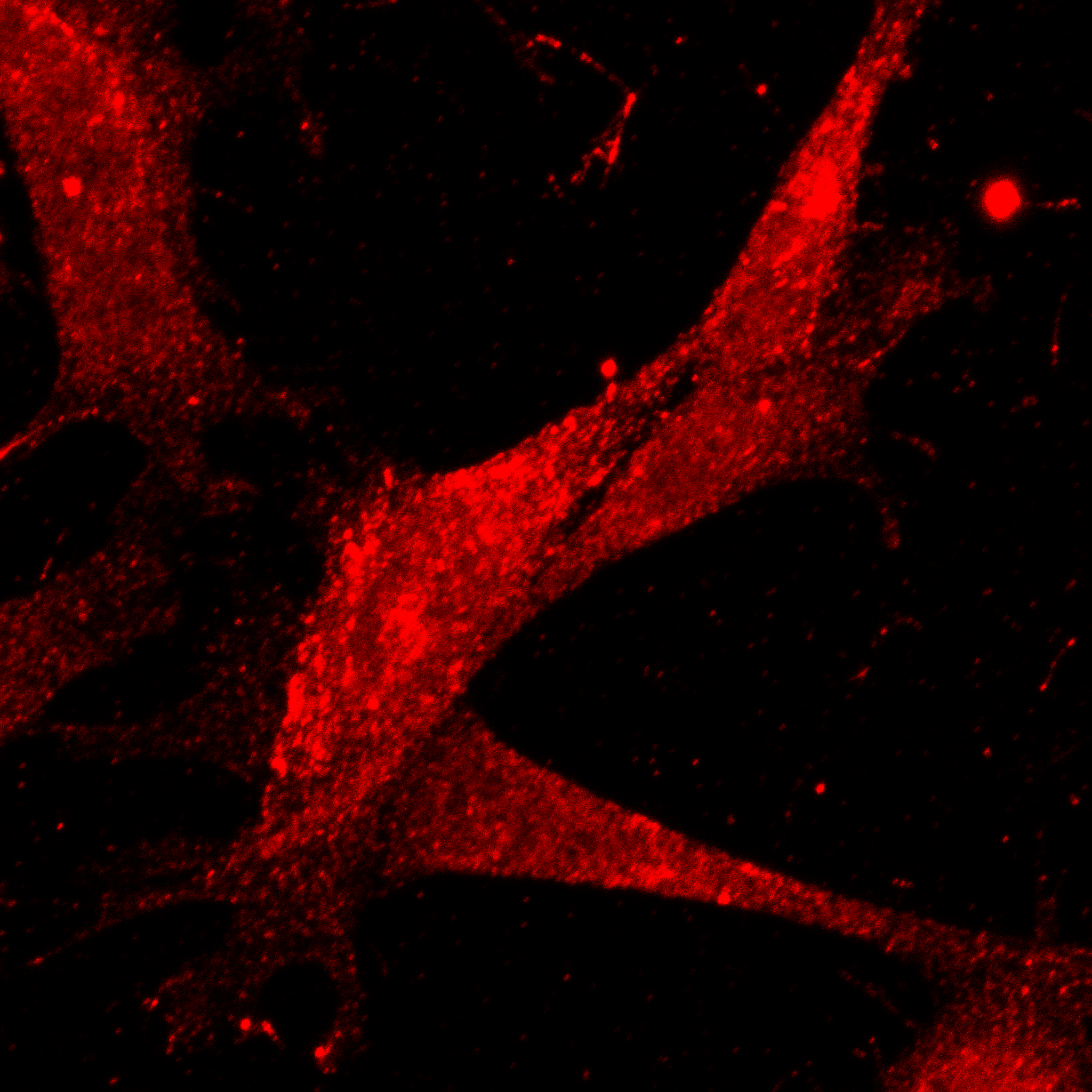

Supplement: Supplementary file 7 — Source data Fig. 3 [file 44319_2026_751_MOESM7_ESM.zip › Raw_data_Figure 3/Figure 3D/GqKO cav2 zoom .tif]

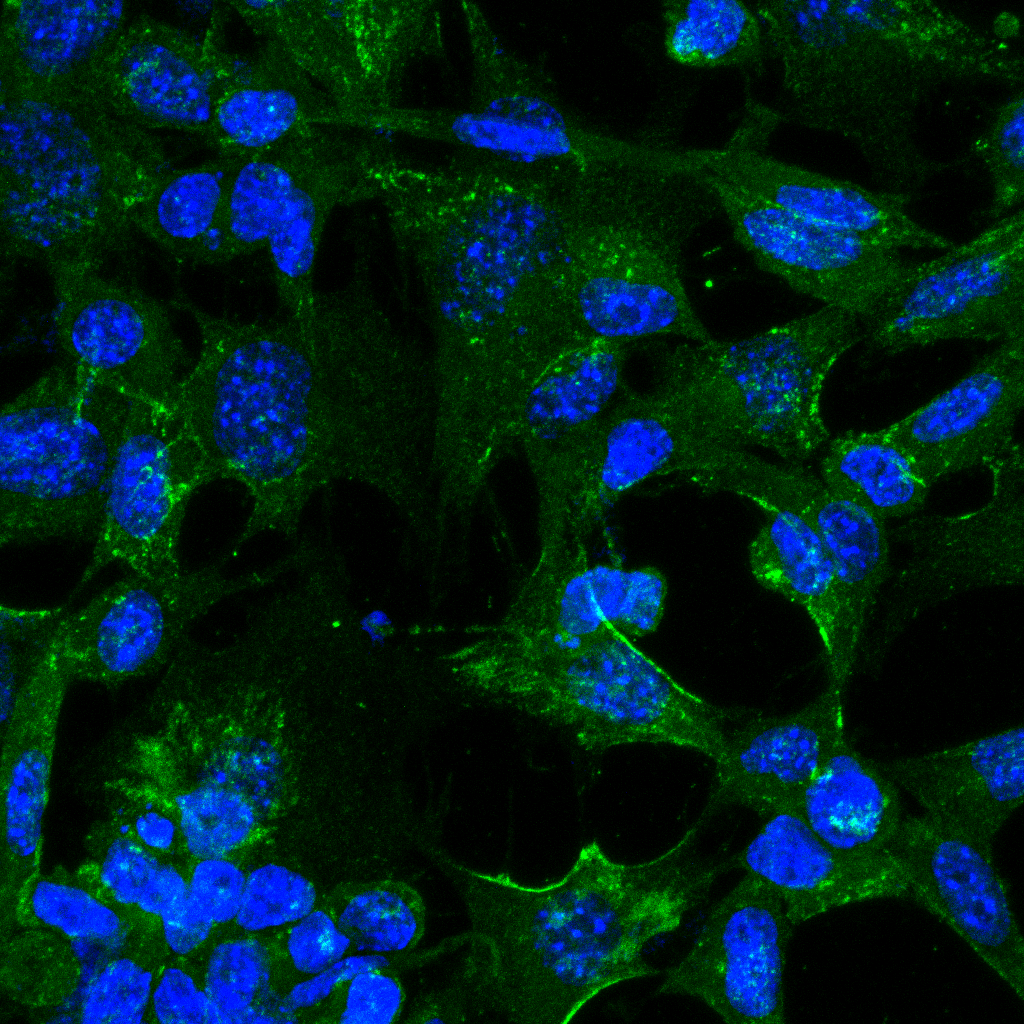

Supplement: Supplementary file 7 — Source data Fig. 3 [file 44319_2026_751_MOESM7_ESM.zip › Raw_data_Figure 3/Figure 3D/GqKO caveolin in confluencia.tif]

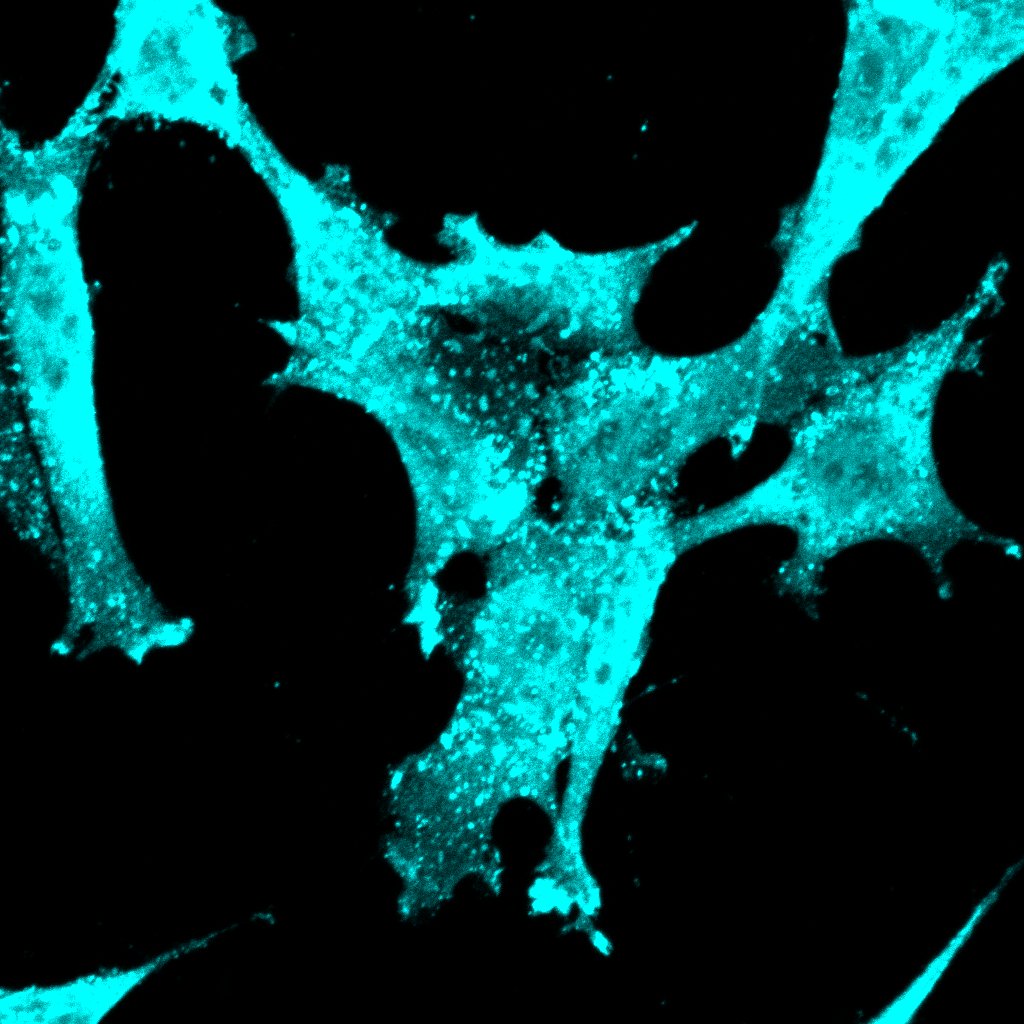

Supplement: Supplementary file 7 — Source data Fig. 3 [file 44319_2026_751_MOESM7_ESM.zip › Raw_data_Figure 3/Figure 3D/GqKO choleta toxin.jpg]

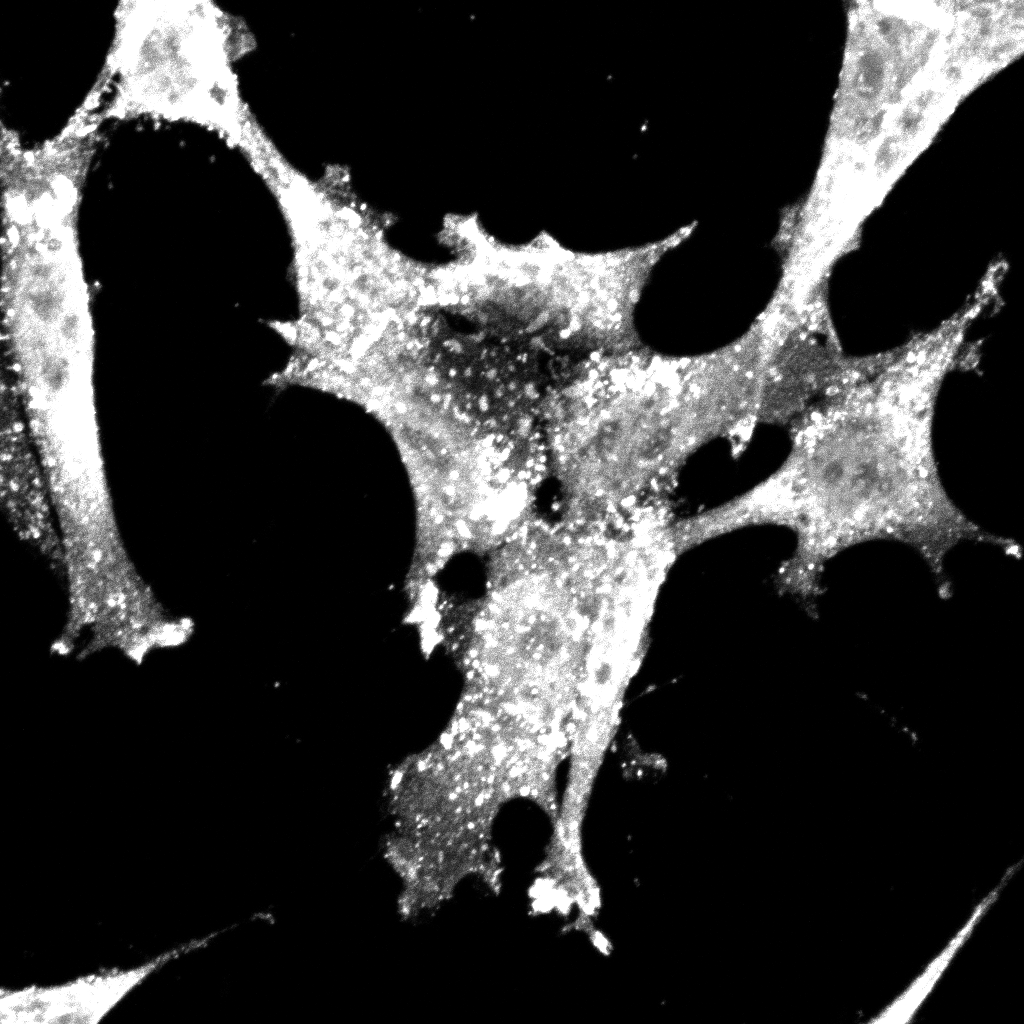

Supplement: Supplementary file 7 — Source data Fig. 3 [file 44319_2026_751_MOESM7_ESM.zip › Raw_data_Figure 3/Figure 3D/GqKO choleta toxin.tif]

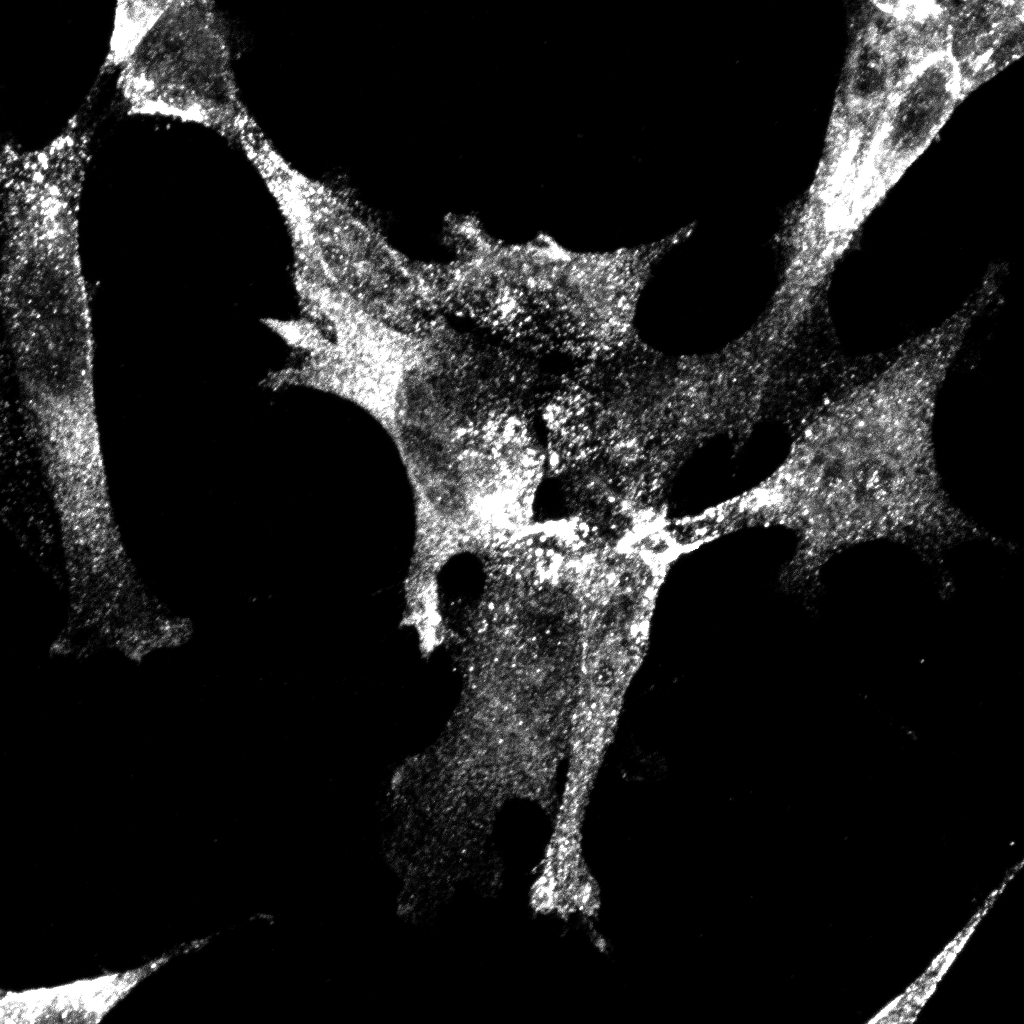

Supplement: Supplementary file 7 — Source data Fig. 3 [file 44319_2026_751_MOESM7_ESM.zip › Raw_data_Figure 3/Figure 3D/GqKO de PRTF PTRF.tif]

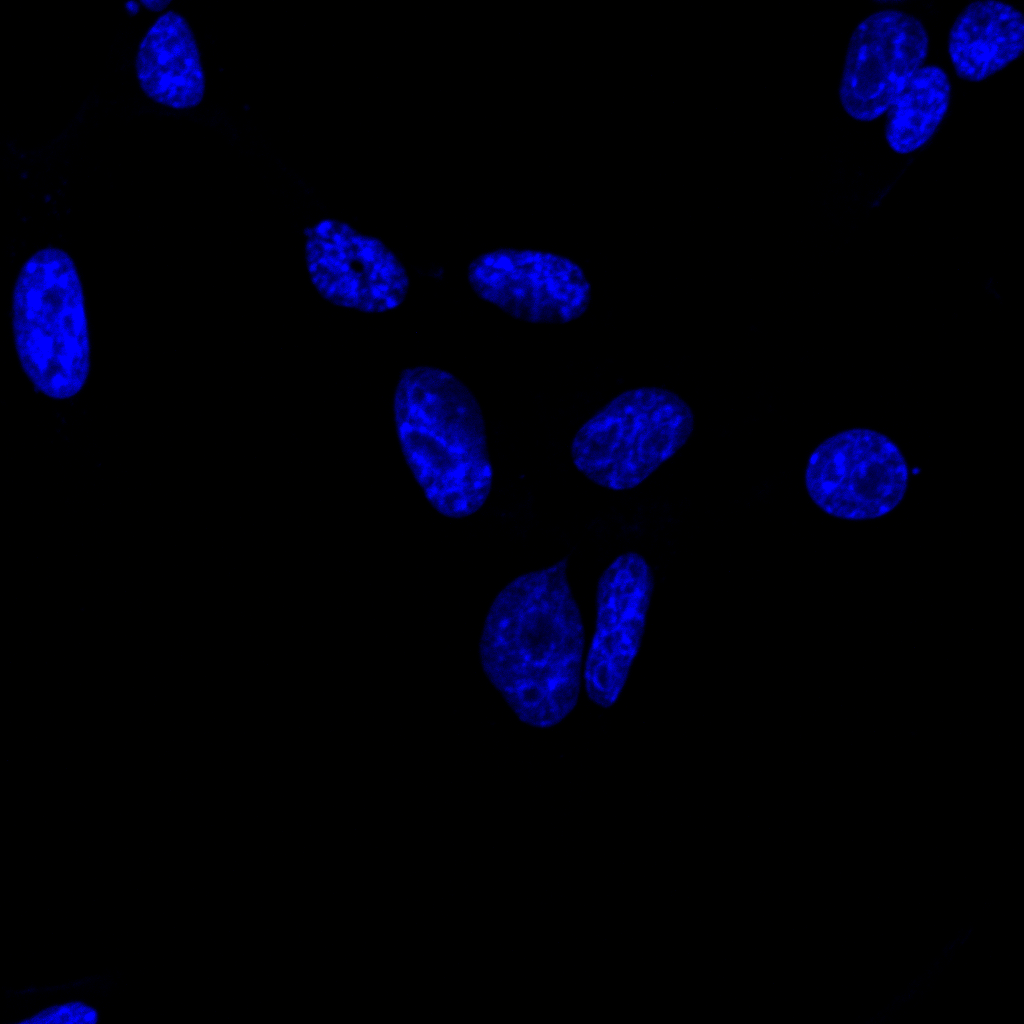

Supplement: Supplementary file 7 — Source data Fig. 3 [file 44319_2026_751_MOESM7_ESM.zip › Raw_data_Figure 3/Figure 3D/GqKO de PTRF nuclei.tif]

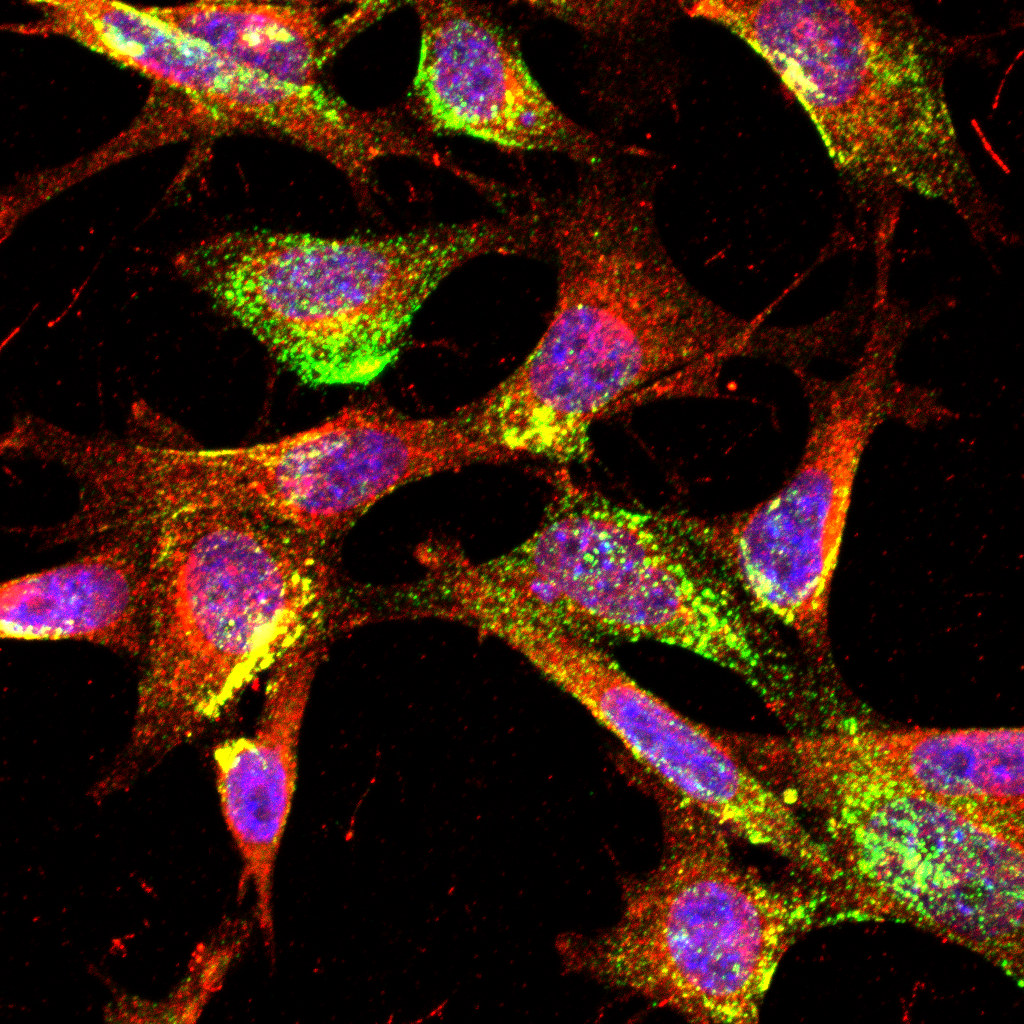

Supplement: Supplementary file 7 — Source data Fig. 3 [file 44319_2026_751_MOESM7_ESM.zip › Raw_data_Figure 3/Figure 3D/GqKO merge 2.tif]

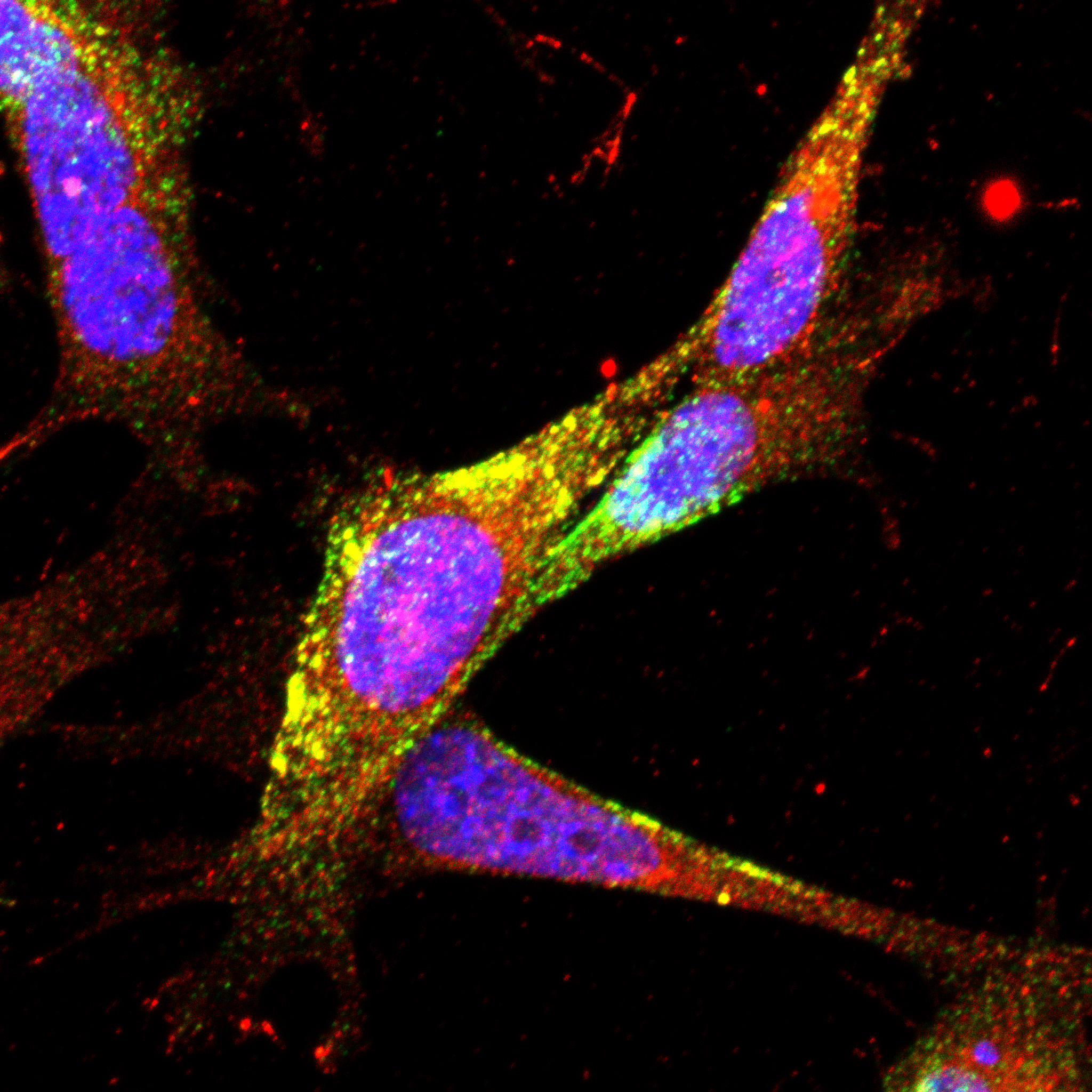

Supplement: Supplementary file 7 — Source data Fig. 3 [file 44319_2026_751_MOESM7_ESM.zip › Raw_data_Figure 3/Figure 3D/GqKO merge zoom.tif]

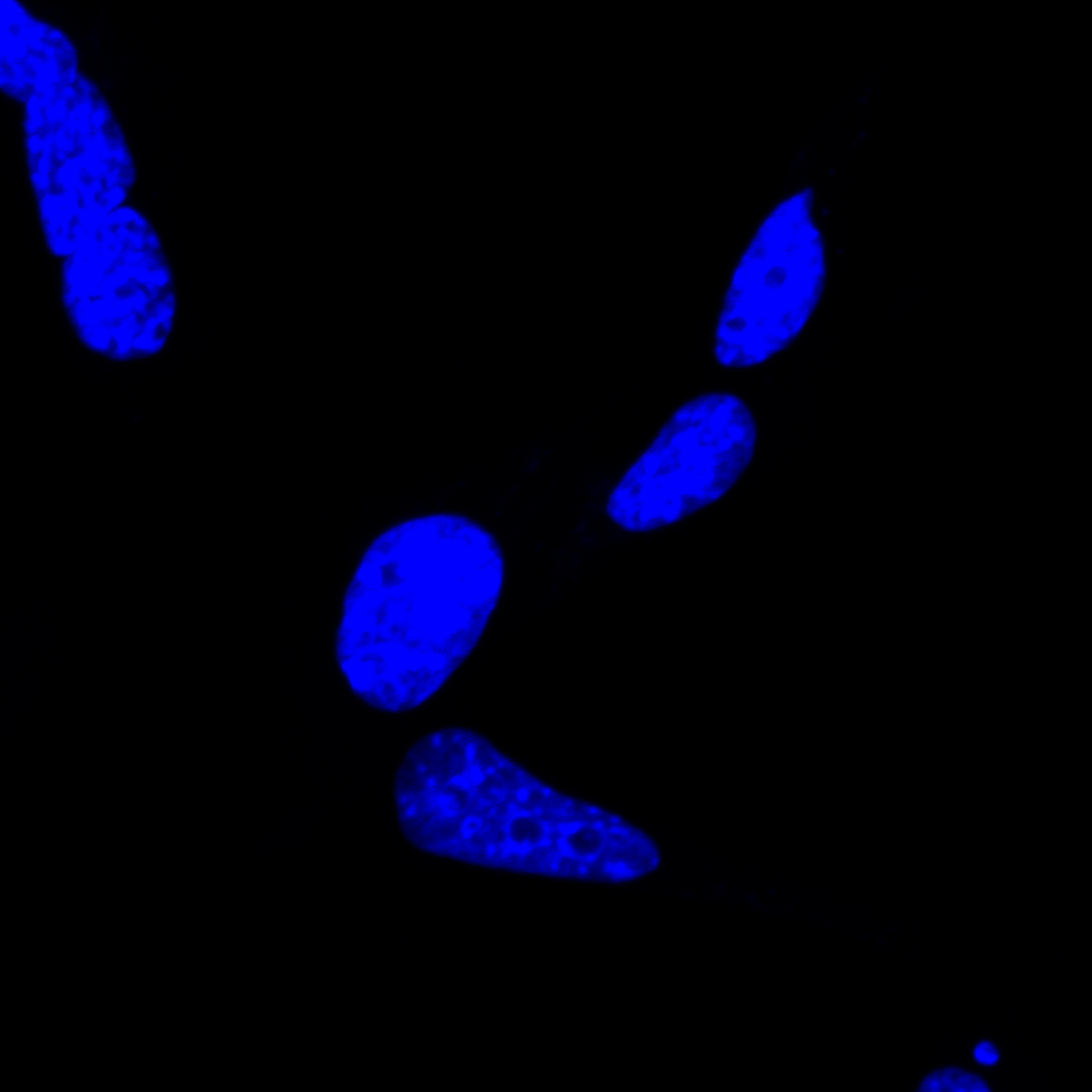

Supplement: Supplementary file 7 — Source data Fig. 3 [file 44319_2026_751_MOESM7_ESM.zip › Raw_data_Figure 3/Figure 3D/GqKO nucle zoom.tif]

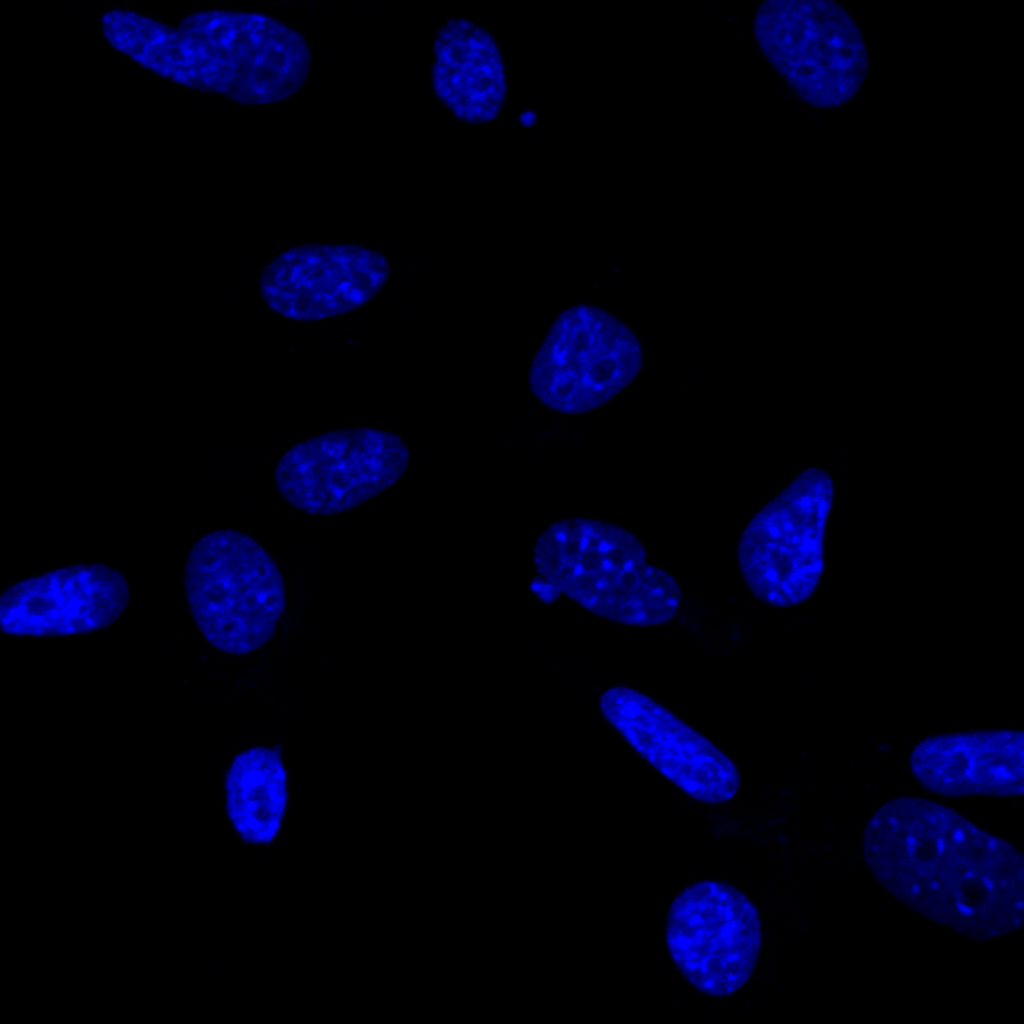

Supplement: Supplementary file 7 — Source data Fig. 3 [file 44319_2026_751_MOESM7_ESM.zip › Raw_data_Figure 3/Figure 3D/GqKO nuclei 2.tif]

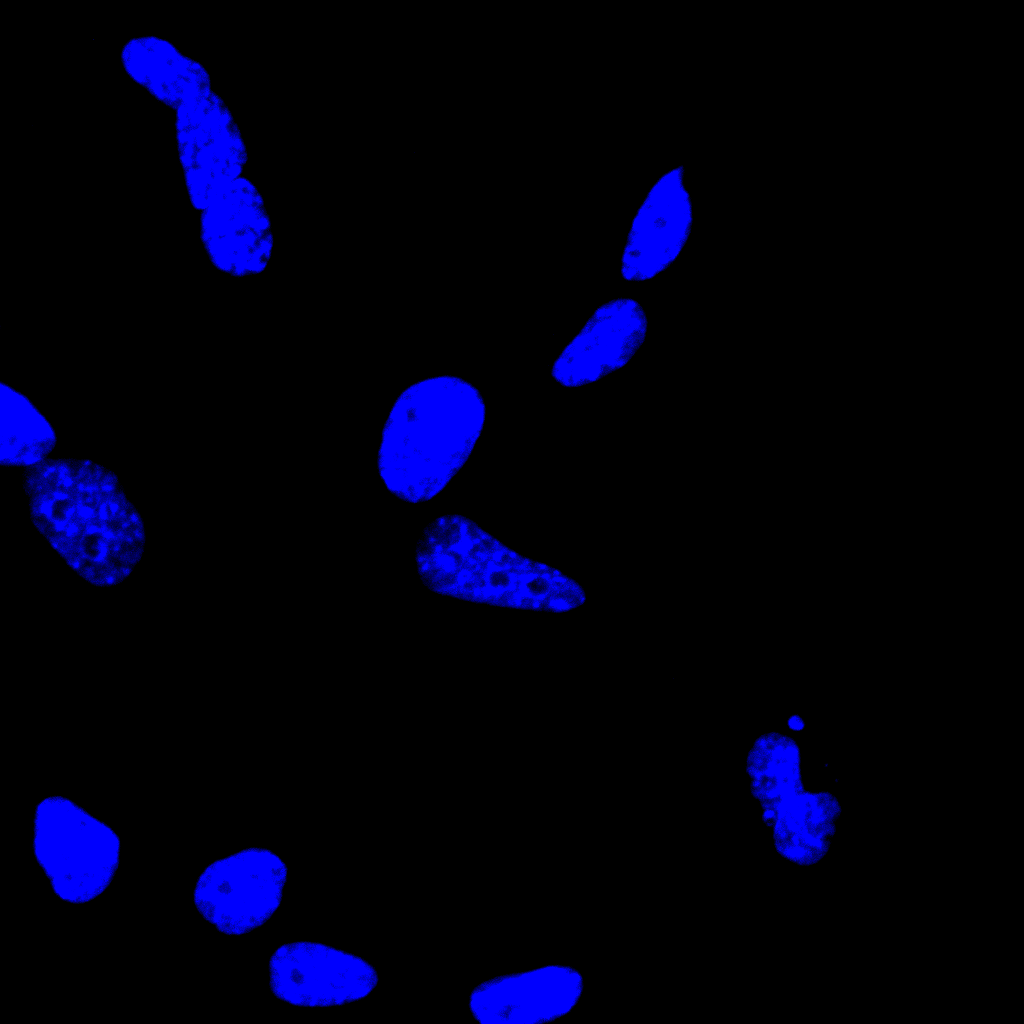

Supplement: Supplementary file 7 — Source data Fig. 3 [file 44319_2026_751_MOESM7_ESM.zip › Raw_data_Figure 3/Figure 3D/GqKO nuclei.tif]

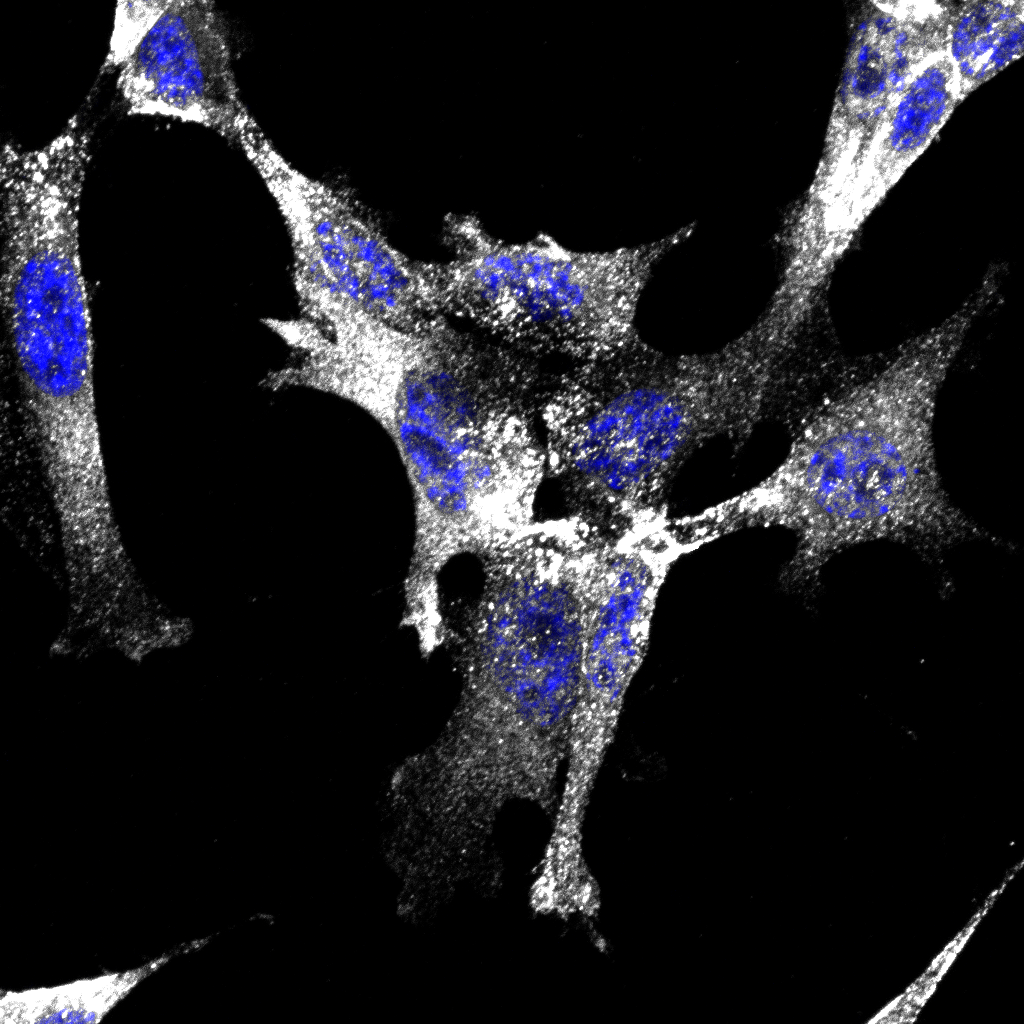

Supplement: Supplementary file 7 — Source data Fig. 3 [file 44319_2026_751_MOESM7_ESM.zip › Raw_data_Figure 3/Figure 3D/GqKO PTRF nuclei.tif]

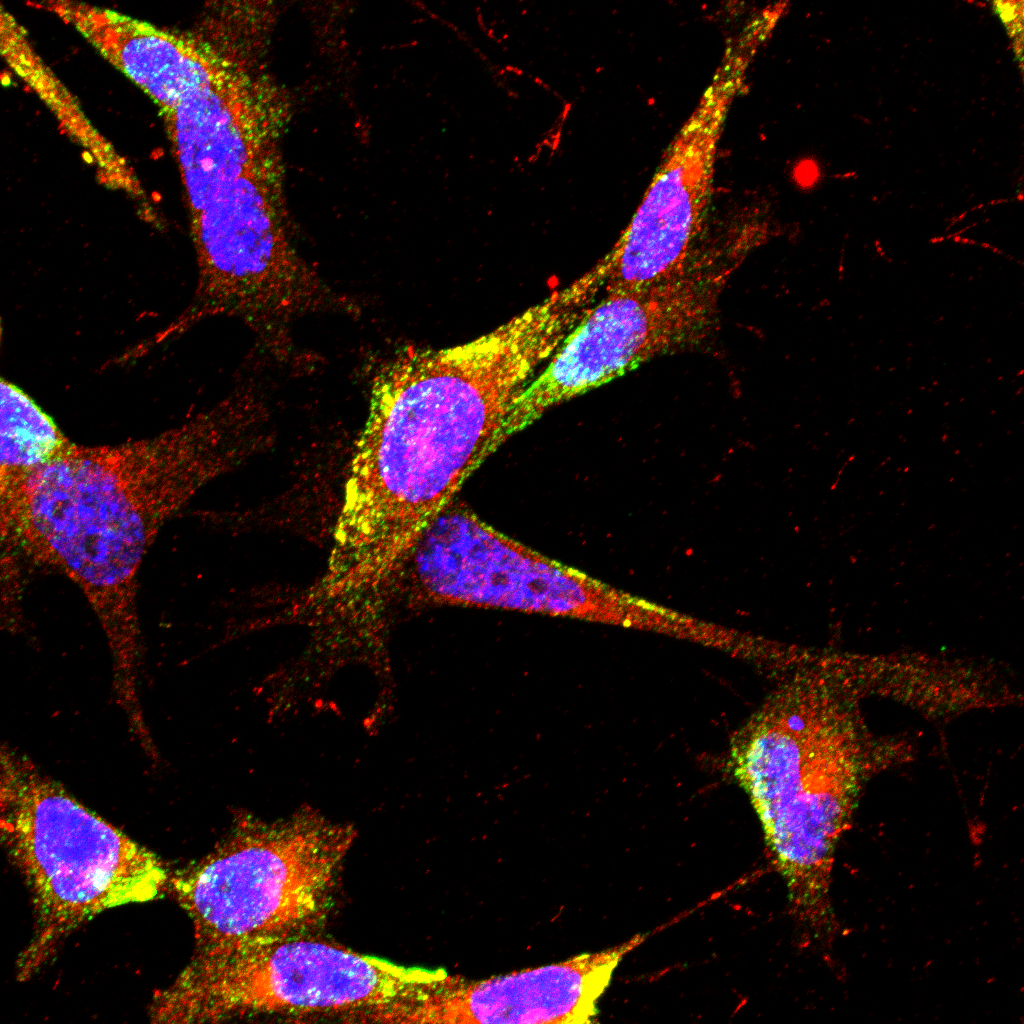

Supplement: Supplementary file 7 — Source data Fig. 3 [file 44319_2026_751_MOESM7_ESM.zip › Raw_data_Figure 3/Figure 3D/GqKOmerge.tif]

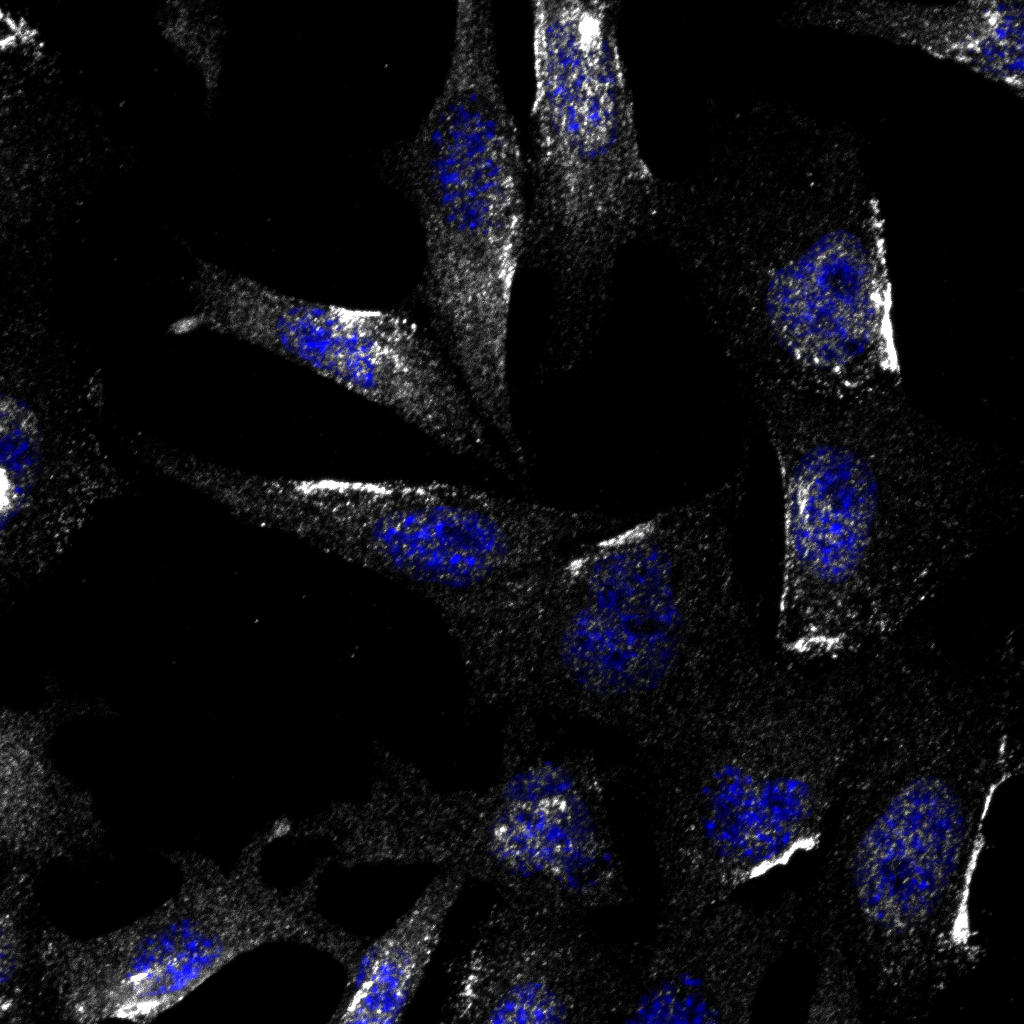

Supplement: Supplementary file 7 — Source data Fig. 3 [file 44319_2026_751_MOESM7_ESM.zip › Raw_data_Figure 3/Figure 3D/RGB.tif]

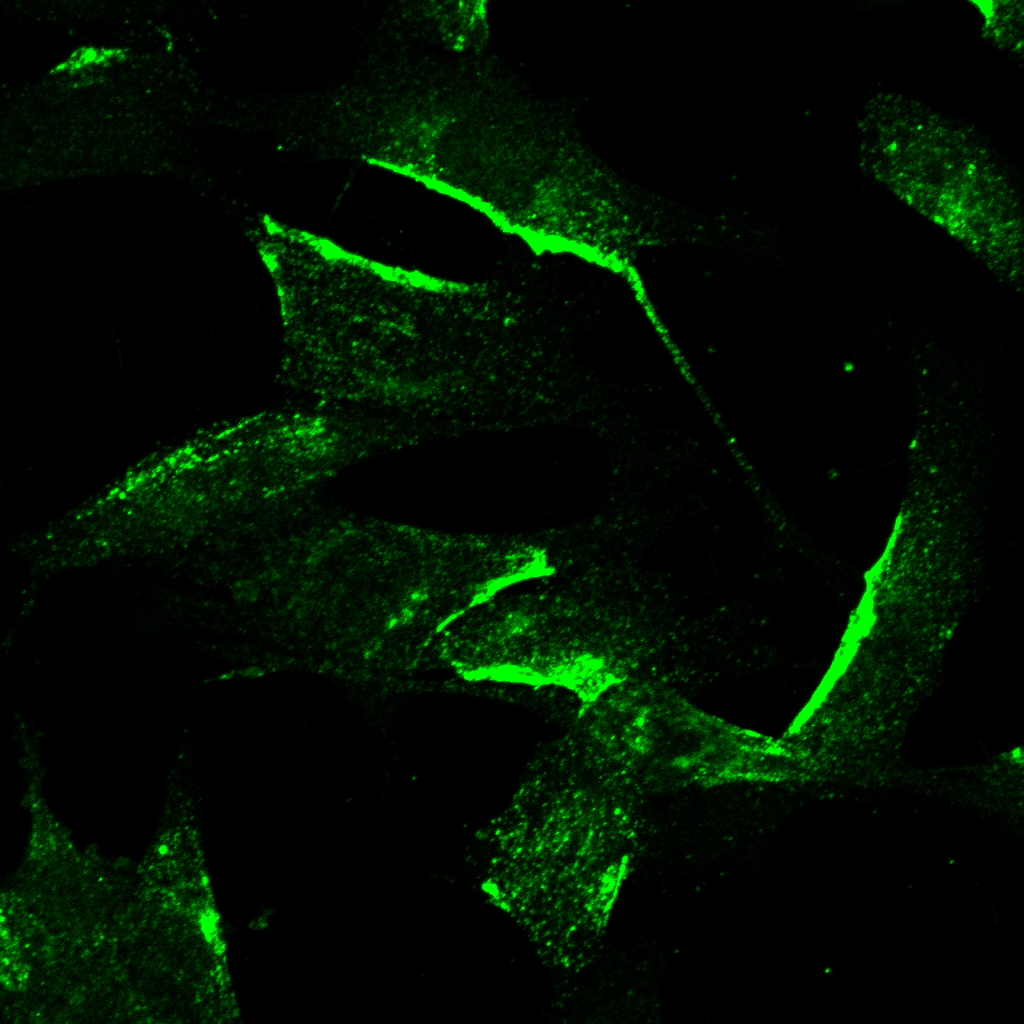

Supplement: Supplementary file 7 — Source data Fig. 3 [file 44319_2026_751_MOESM7_ESM.zip › Raw_data_Figure 3/Figure 3D/WT Cav1 2.tif]

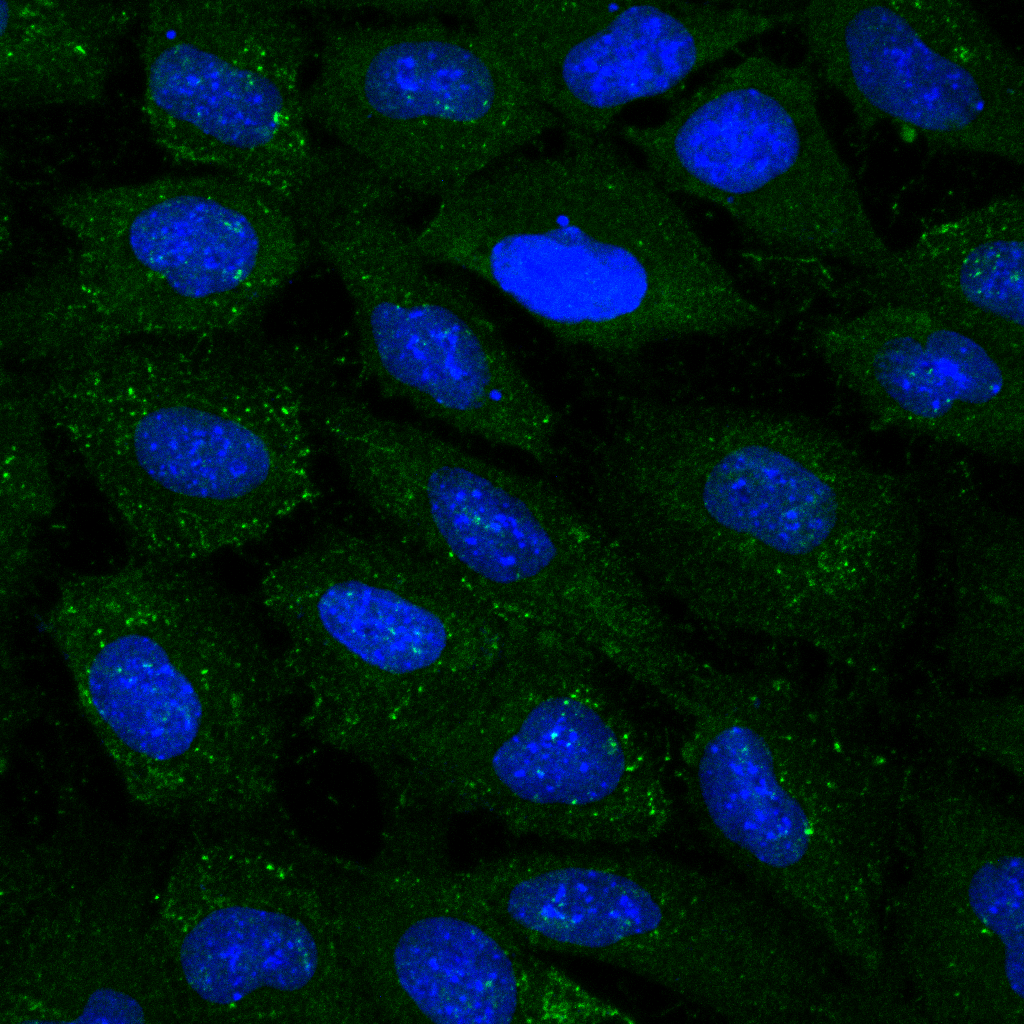

Supplement: Supplementary file 7 — Source data Fig. 3 [file 44319_2026_751_MOESM7_ESM.zip › Raw_data_Figure 3/Figure 3D/WT Cav1 en confluencia 2.tif]

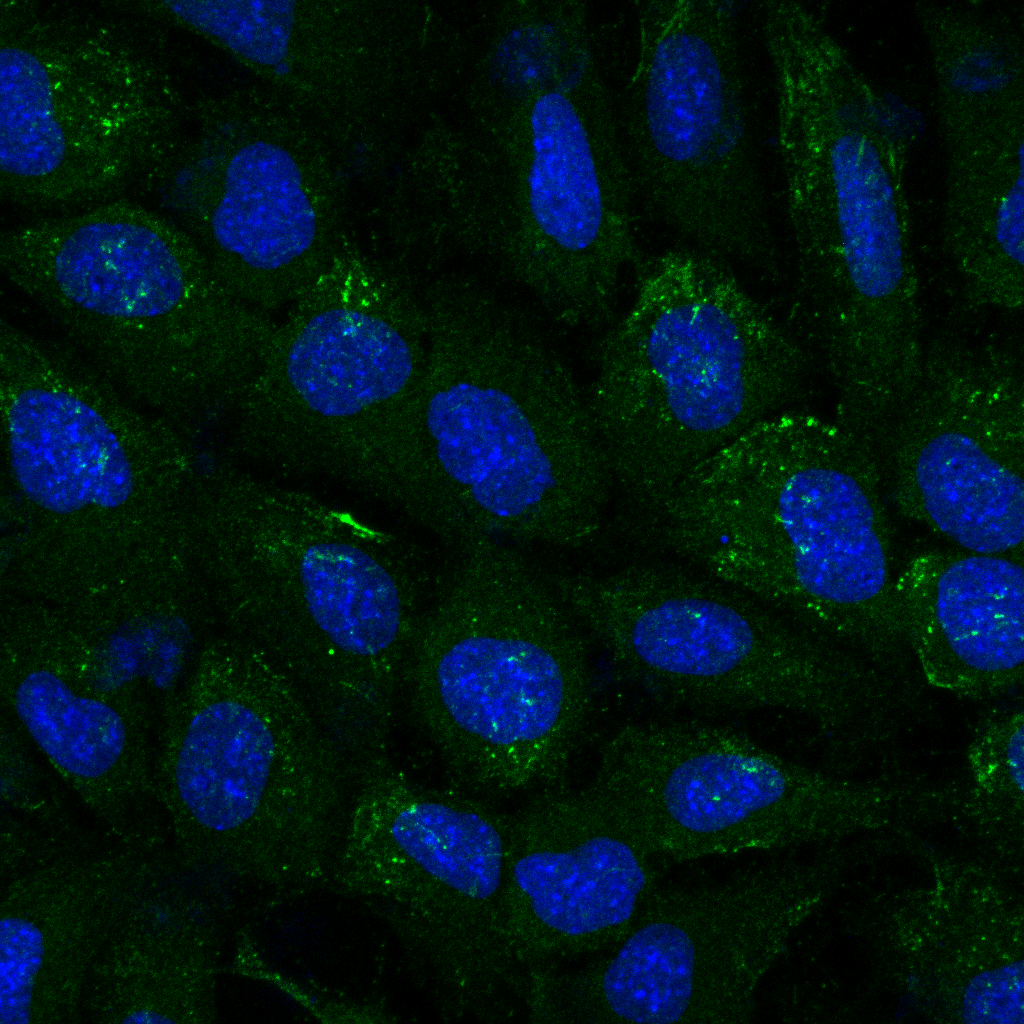

Supplement: Supplementary file 7 — Source data Fig. 3 [file 44319_2026_751_MOESM7_ESM.zip › Raw_data_Figure 3/Figure 3D/WT Cav1 en confluencia.tif]

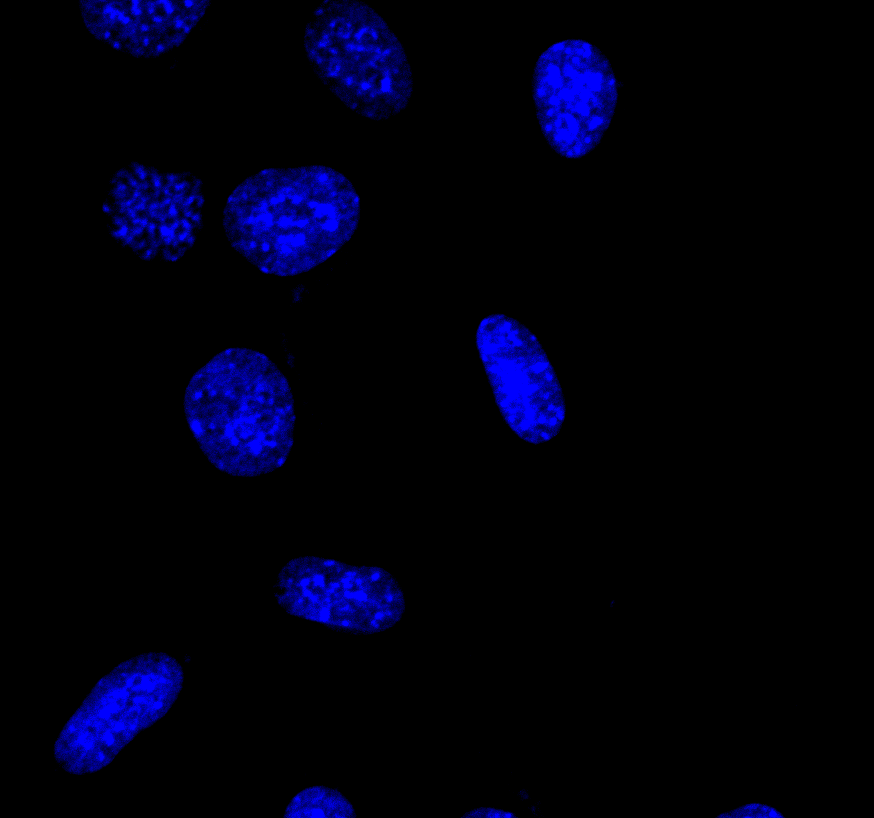

Supplement: Supplementary file 7 — Source data Fig. 3 [file 44319_2026_751_MOESM7_ESM.zip › Raw_data_Figure 3/Figure 3D/WT Cav1 en rojo nucleos blue para suplementaria.tif]

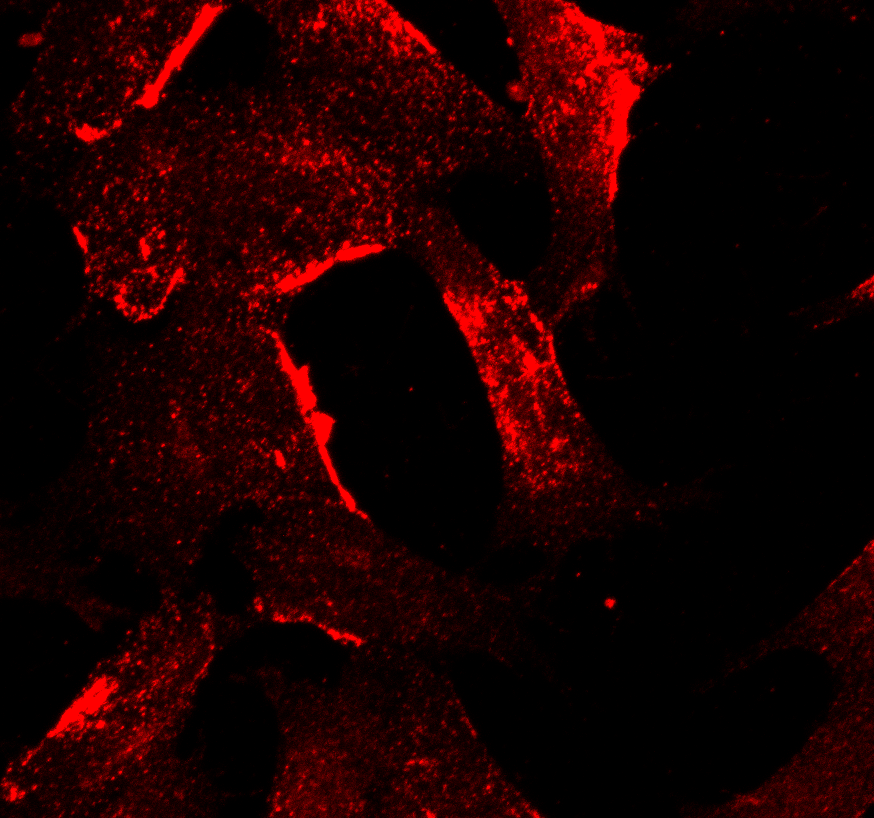

Supplement: Supplementary file 7 — Source data Fig. 3 [file 44319_2026_751_MOESM7_ESM.zip › Raw_data_Figure 3/Figure 3D/WT Cav1 en rojo para suplementaria.tif]

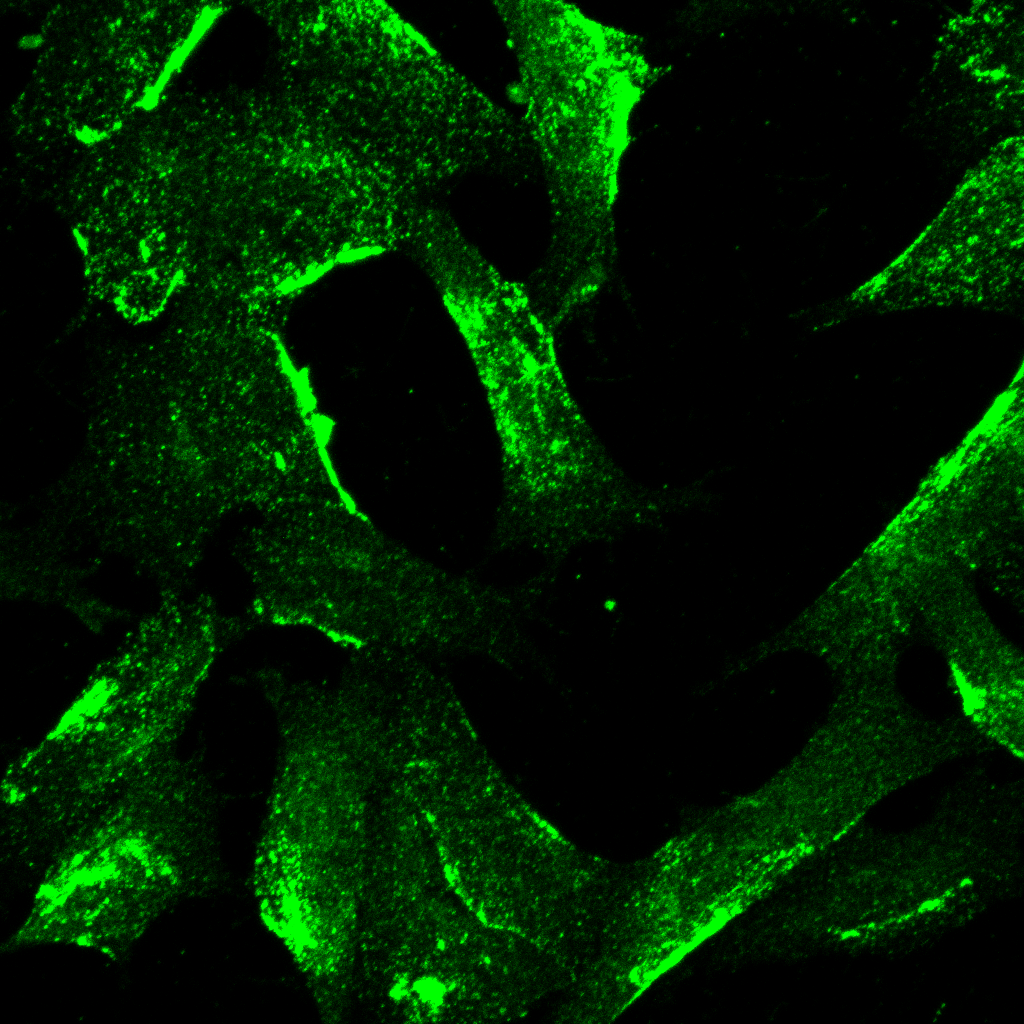

Supplement: Supplementary file 7 — Source data Fig. 3 [file 44319_2026_751_MOESM7_ESM.zip › Raw_data_Figure 3/Figure 3D/WT Cav1.tif]

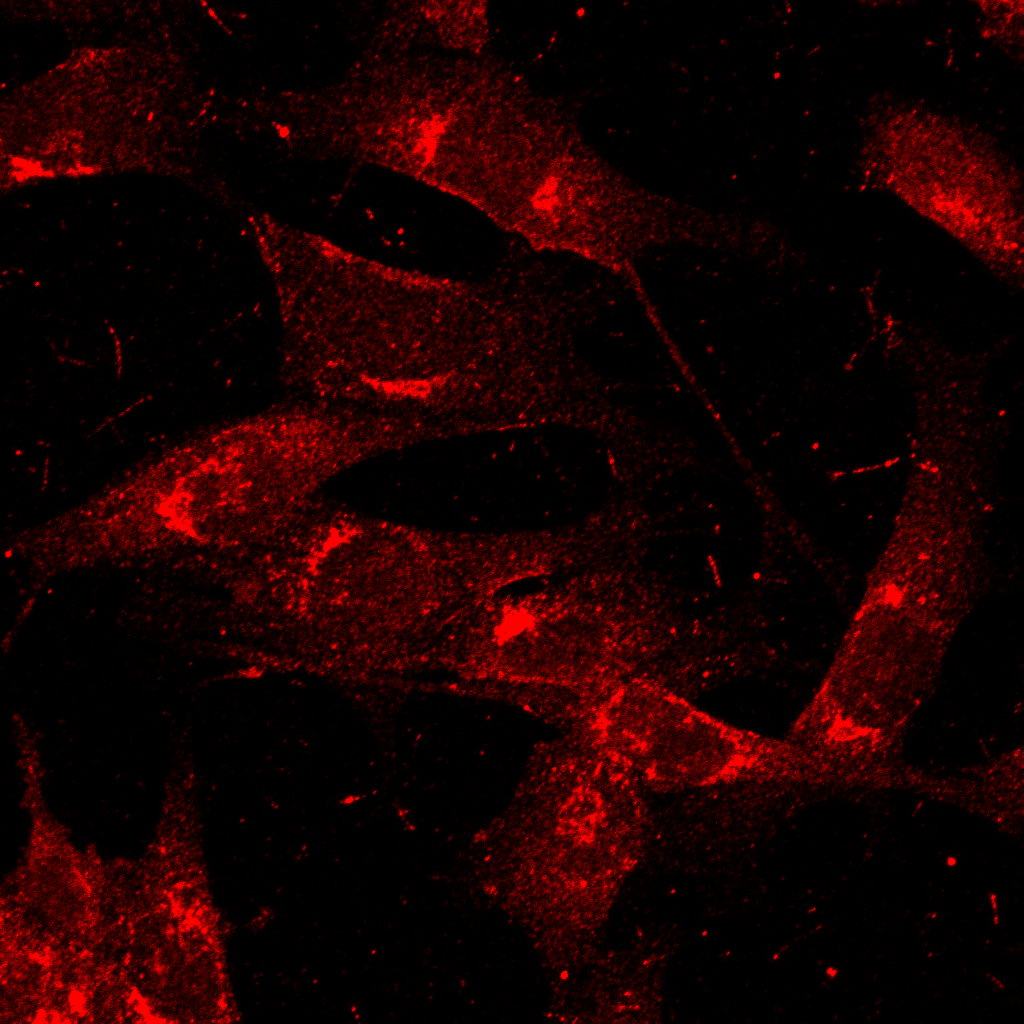

Supplement: Supplementary file 7 — Source data Fig. 3 [file 44319_2026_751_MOESM7_ESM.zip › Raw_data_Figure 3/Figure 3D/WT Cav2 2.tif]

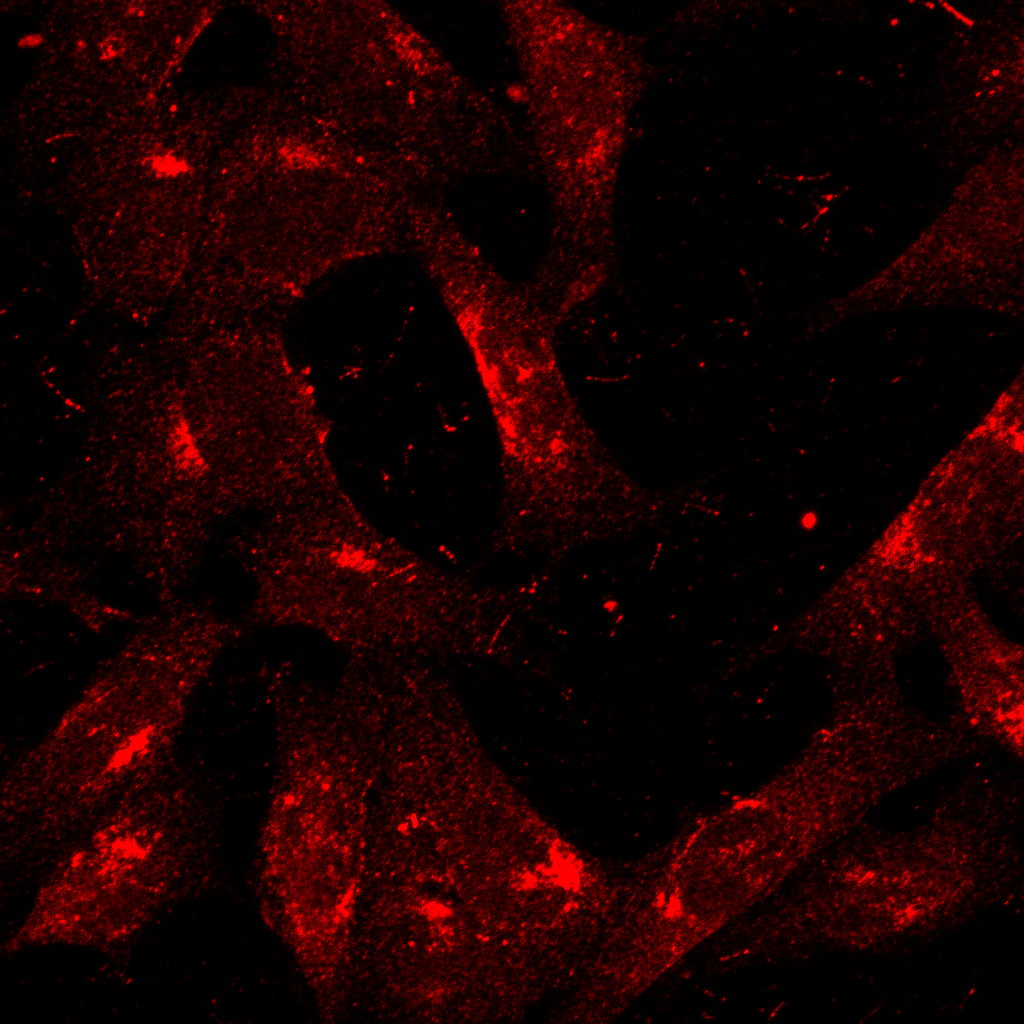

Supplement: Supplementary file 7 — Source data Fig. 3 [file 44319_2026_751_MOESM7_ESM.zip › Raw_data_Figure 3/Figure 3D/WT Cav2.tif]

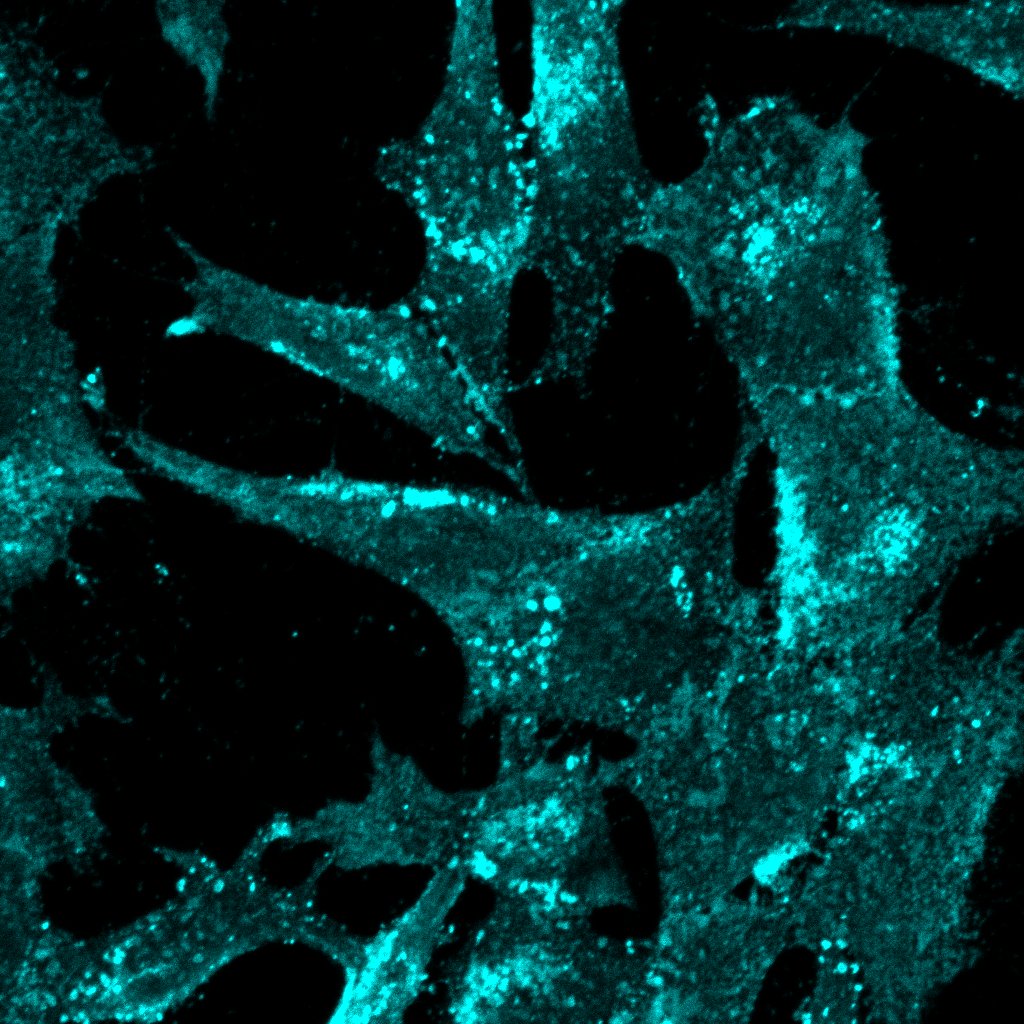

Supplement: Supplementary file 7 — Source data Fig. 3 [file 44319_2026_751_MOESM7_ESM.zip › Raw_data_Figure 3/Figure 3D/WT Cholera toxin.jpg]

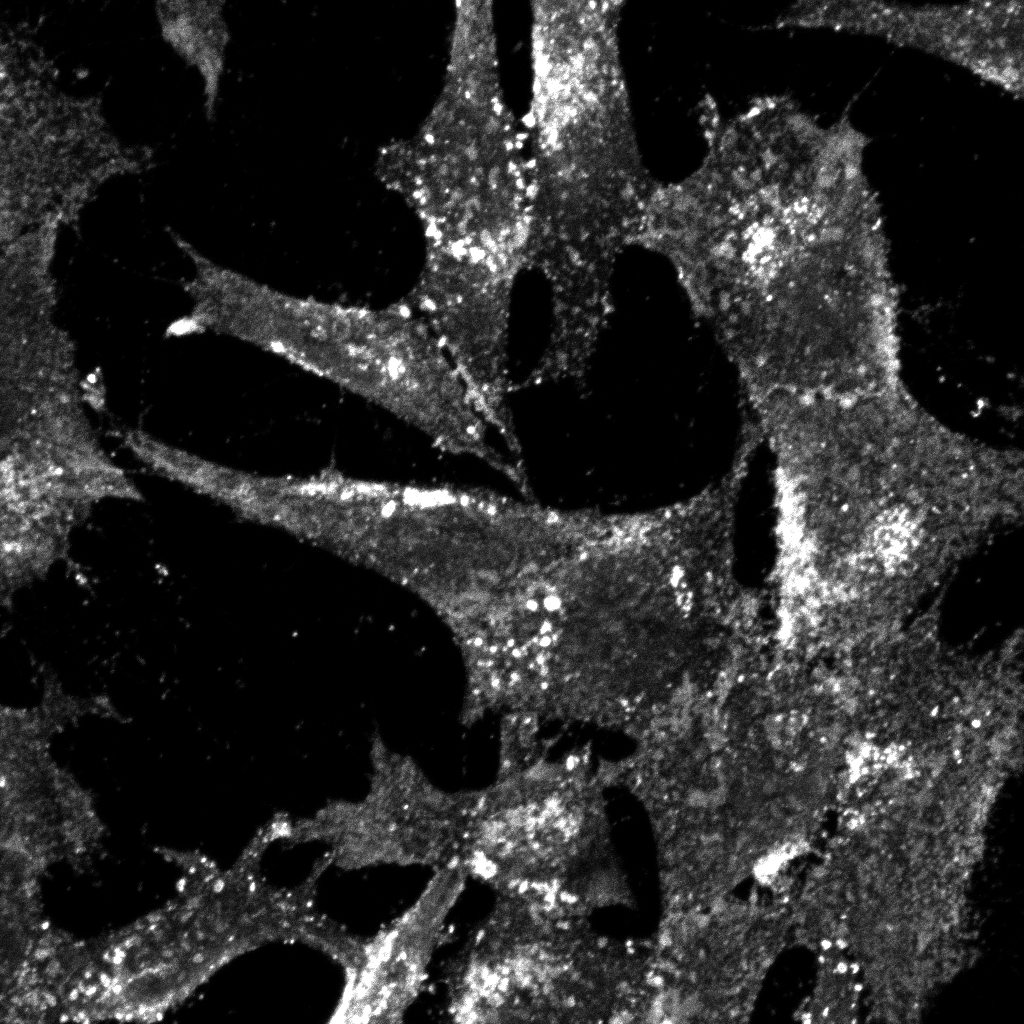

Supplement: Supplementary file 7 — Source data Fig. 3 [file 44319_2026_751_MOESM7_ESM.zip › Raw_data_Figure 3/Figure 3D/WT Cholera toxin.tif]

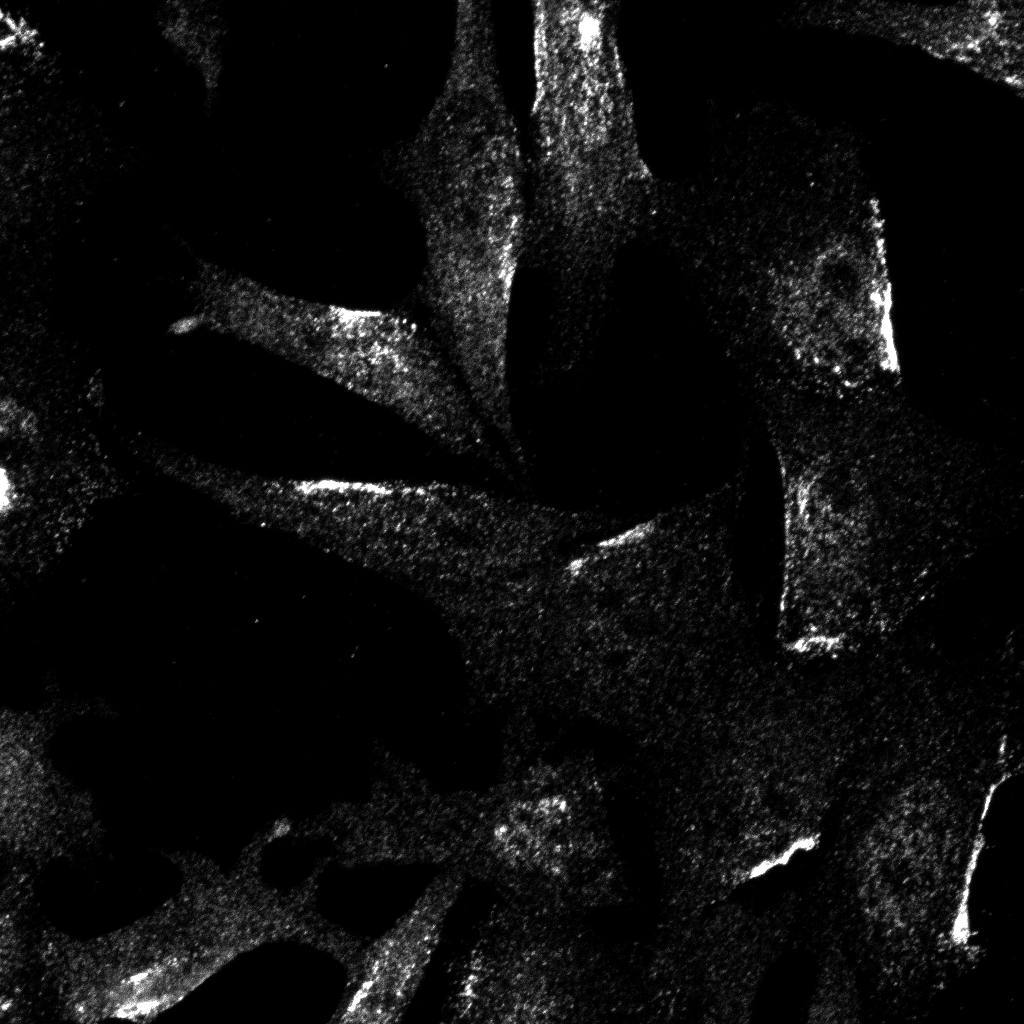

Supplement: Supplementary file 7 — Source data Fig. 3 [file 44319_2026_751_MOESM7_ESM.zip › Raw_data_Figure 3/Figure 3D/WT de PTRF PTRF.tif]

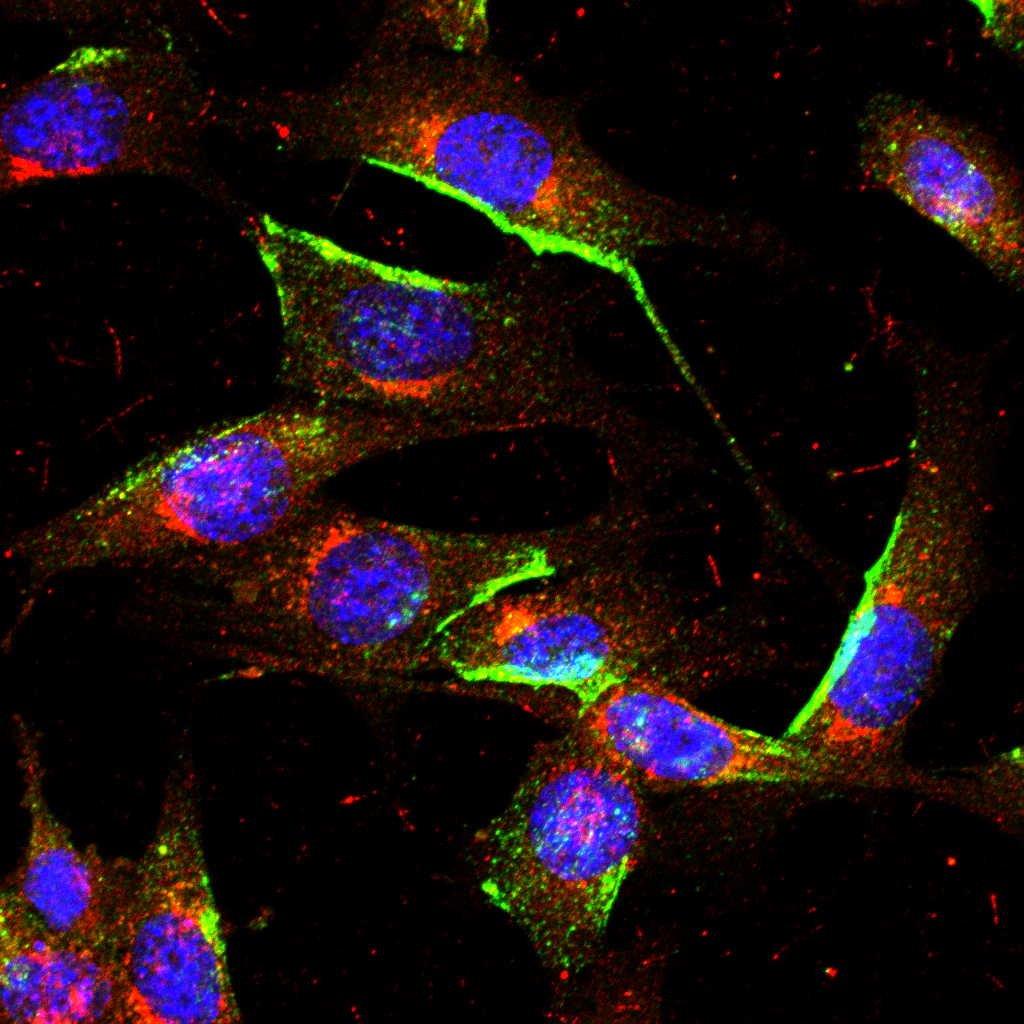

Supplement: Supplementary file 7 — Source data Fig. 3 [file 44319_2026_751_MOESM7_ESM.zip › Raw_data_Figure 3/Figure 3D/WT merge 2.tif]

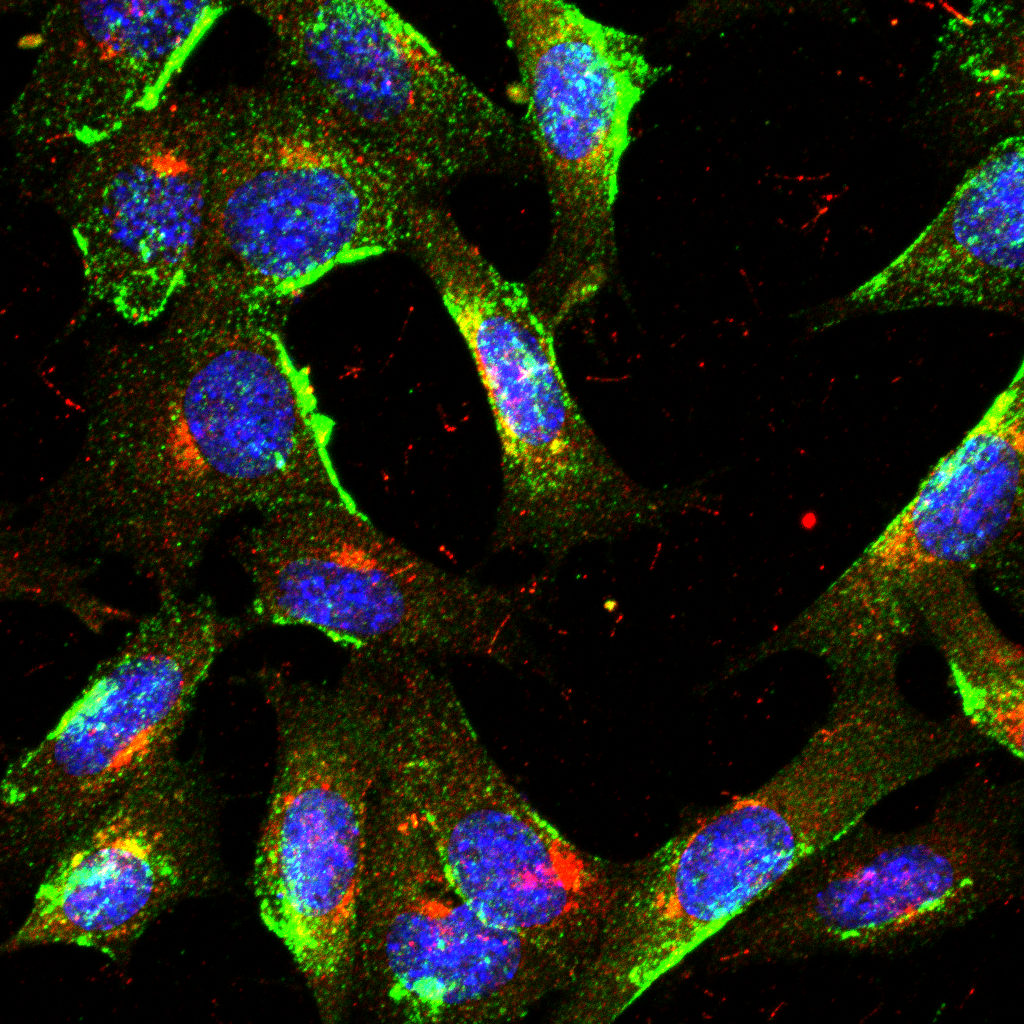

Supplement: Supplementary file 7 — Source data Fig. 3 [file 44319_2026_751_MOESM7_ESM.zip › Raw_data_Figure 3/Figure 3D/WT merge.tif]

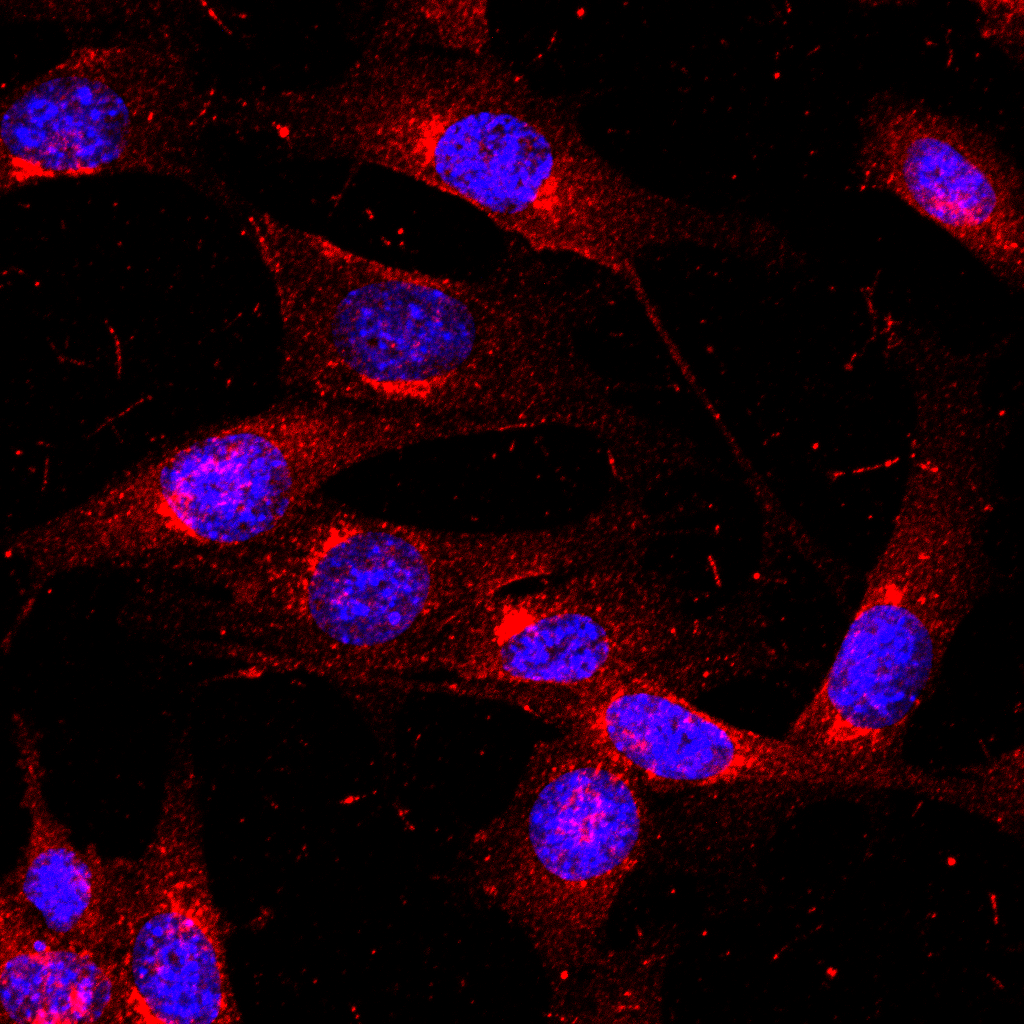

Supplement: Supplementary file 7 — Source data Fig. 3 [file 44319_2026_751_MOESM7_ESM.zip › Raw_data_Figure 3/Figure 3D/WT nuceli cav2 2.tif]

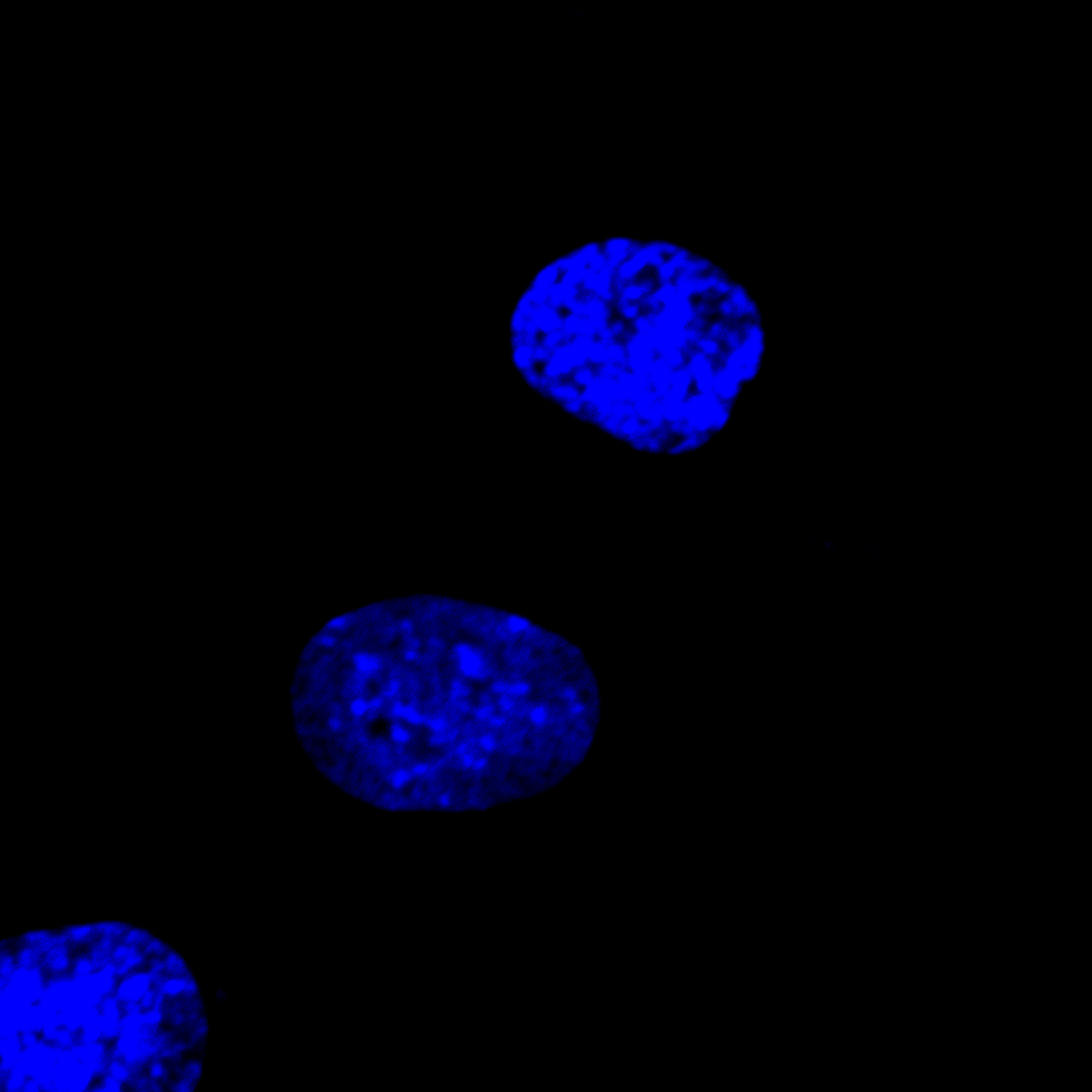

Supplement: Supplementary file 7 — Source data Fig. 3 [file 44319_2026_751_MOESM7_ESM.zip › Raw_data_Figure 3/Figure 3D/WT nuceli zoom.tif]

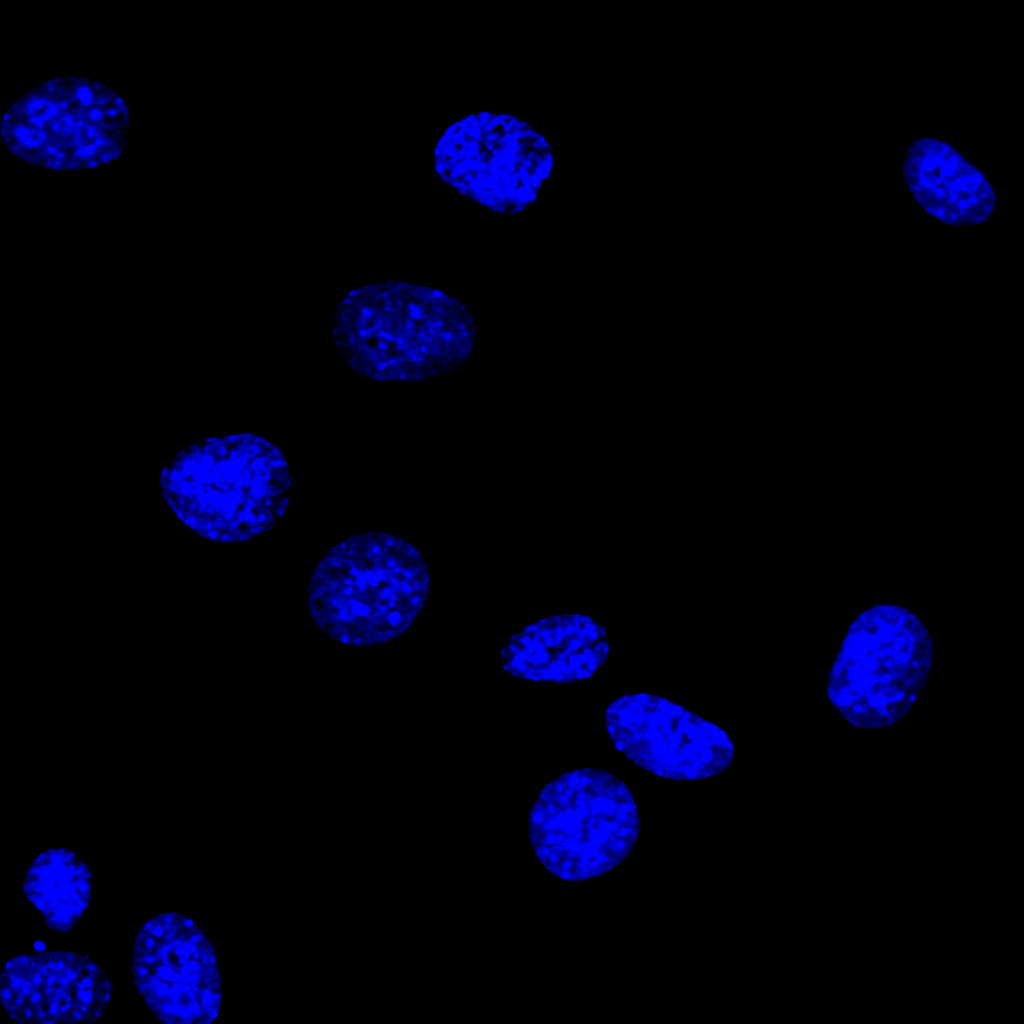

Supplement: Supplementary file 7 — Source data Fig. 3 [file 44319_2026_751_MOESM7_ESM.zip › Raw_data_Figure 3/Figure 3D/WT nuclei 2.tif]

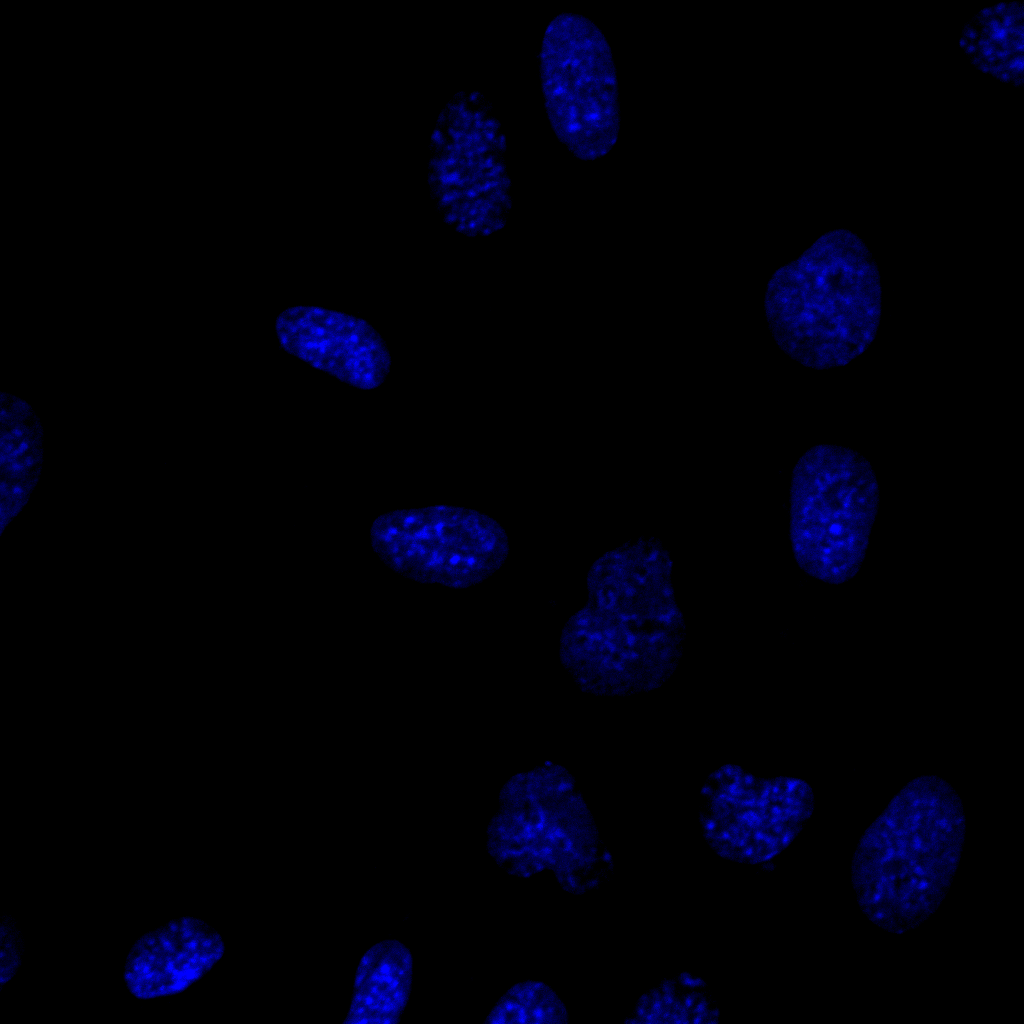

Supplement: Supplementary file 7 — Source data Fig. 3 [file 44319_2026_751_MOESM7_ESM.zip › Raw_data_Figure 3/Figure 3D/WT nuclei de PTRF.tif]

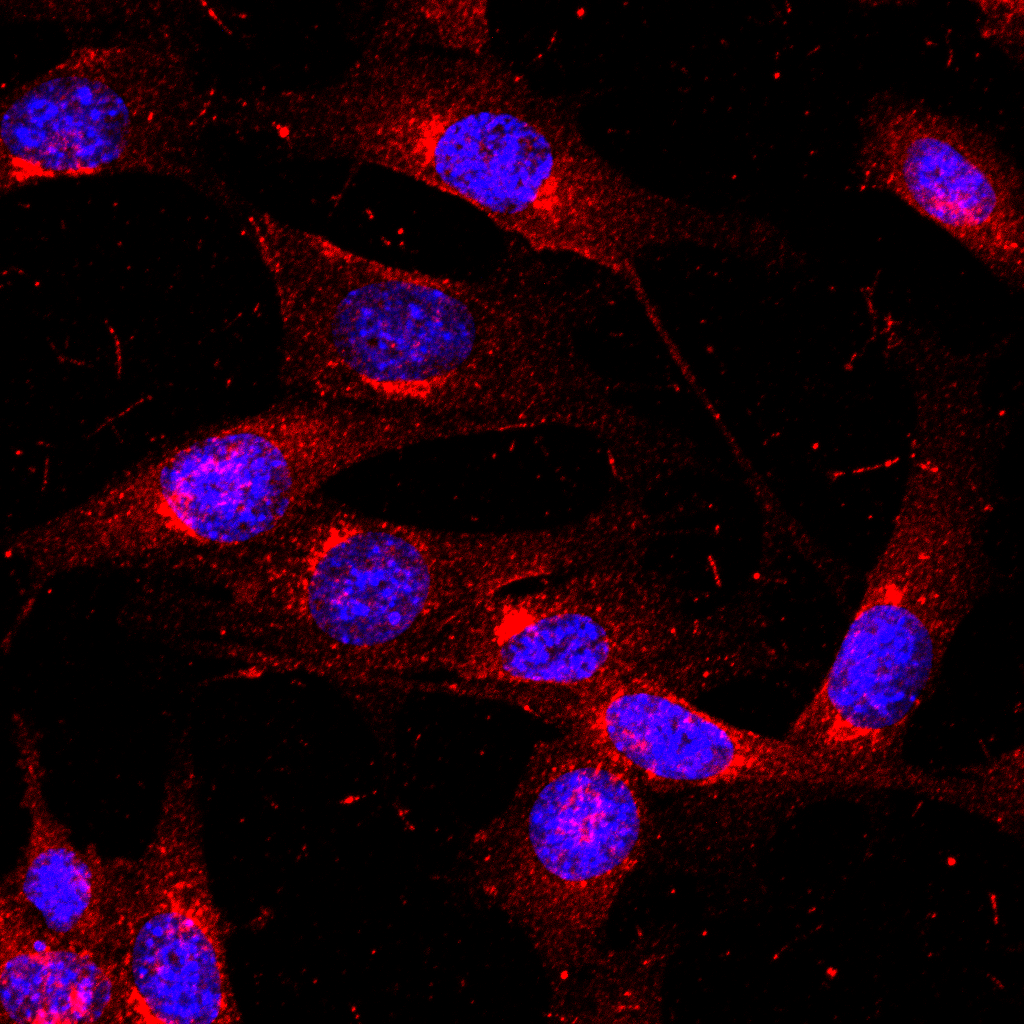

Supplement: Supplementary file 7 — Source data Fig. 3 [file 44319_2026_751_MOESM7_ESM.zip › Raw_data_Figure 3/Figure 3D/WT nuclei-cav2 2.tif]

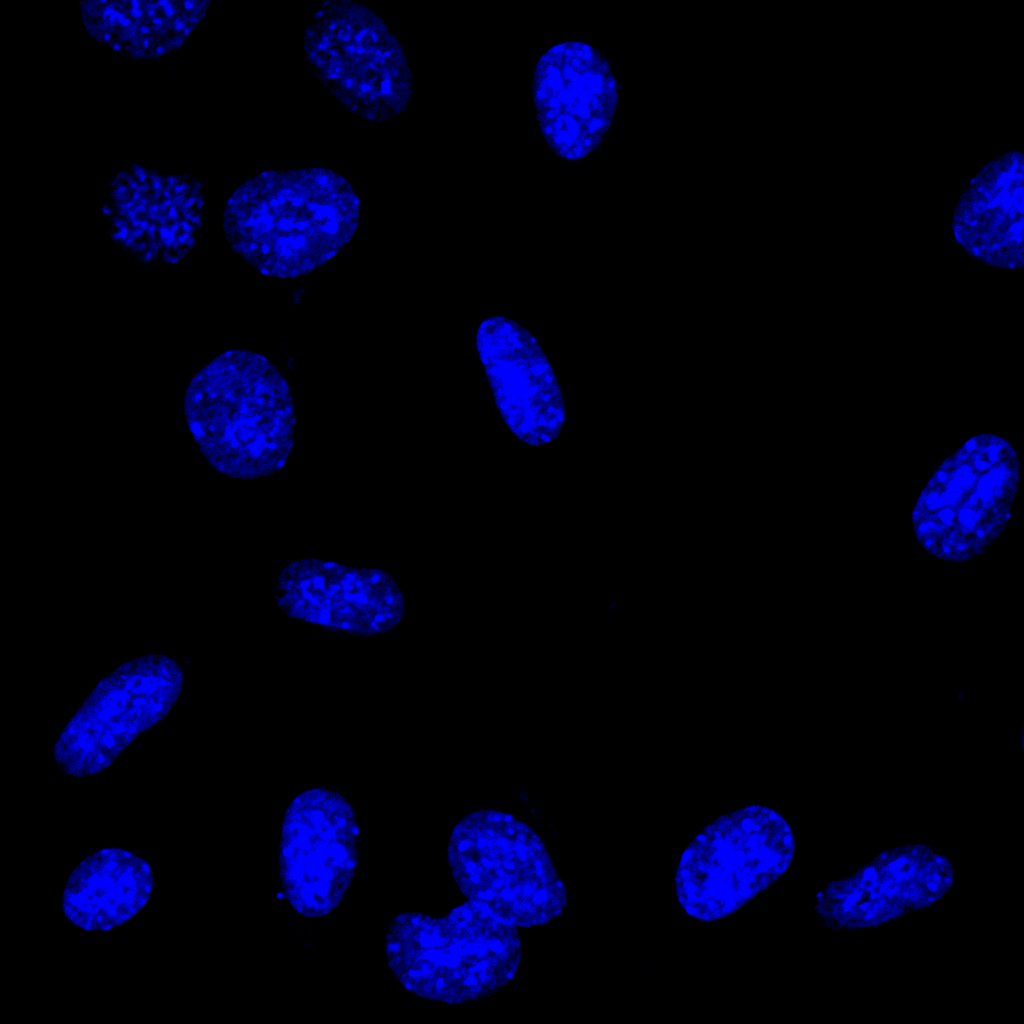

Supplement: Supplementary file 7 — Source data Fig. 3 [file 44319_2026_751_MOESM7_ESM.zip › Raw_data_Figure 3/Figure 3D/WT nuclei.tif]

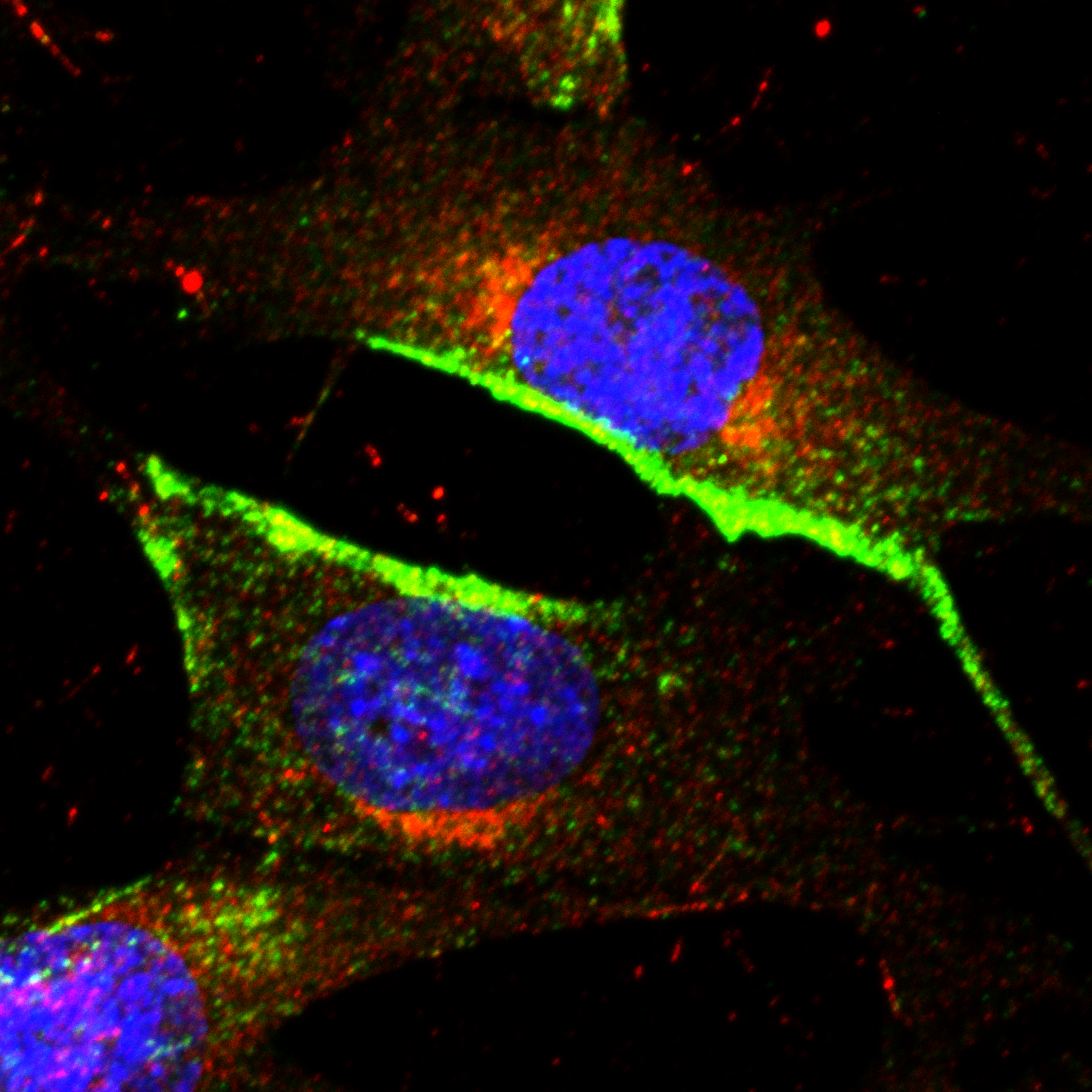

Supplement: Supplementary file 7 — Source data Fig. 3 [file 44319_2026_751_MOESM7_ESM.zip › Raw_data_Figure 3/Figure 3D/WT zoom merge.tif]

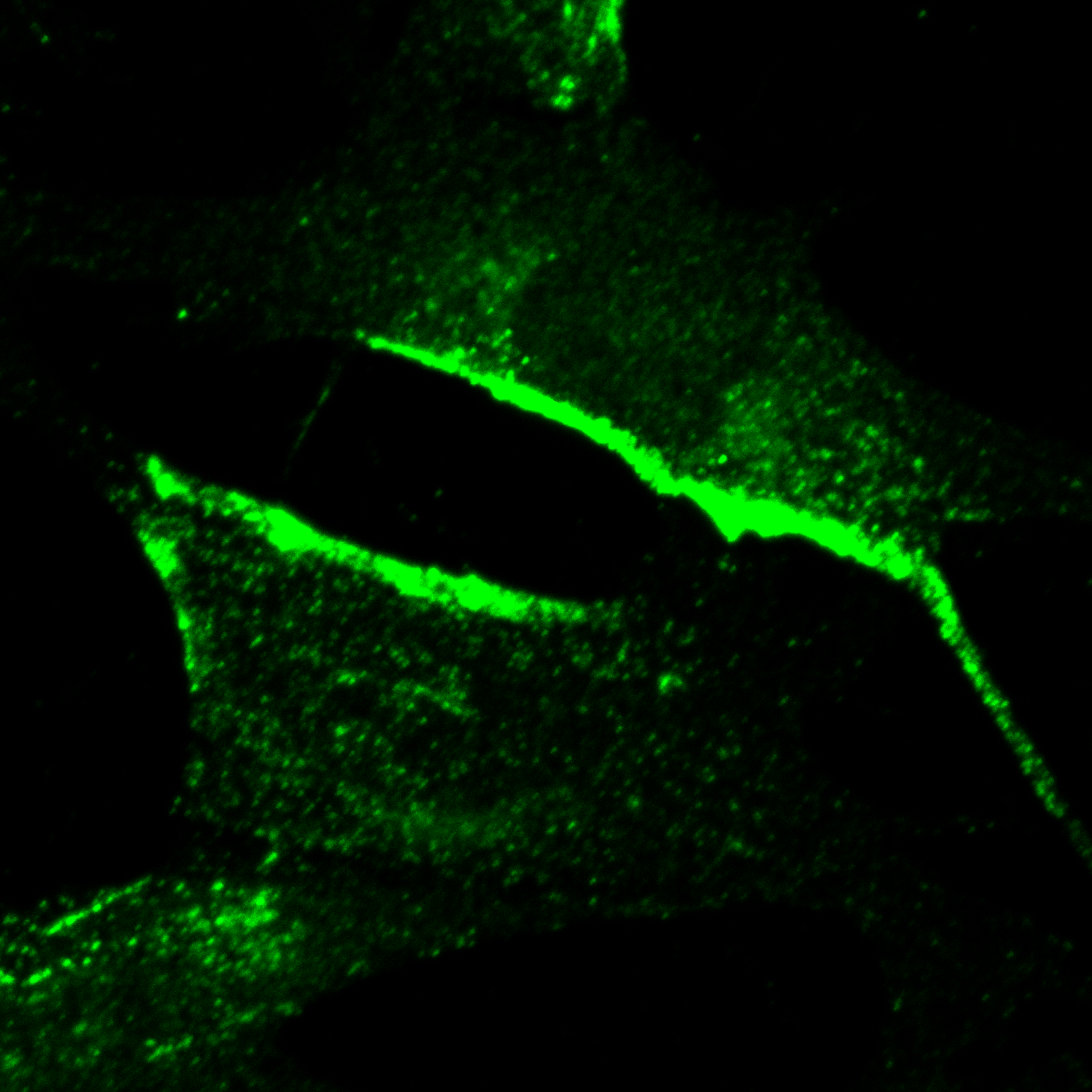

Supplement: Supplementary file 7 — Source data Fig. 3 [file 44319_2026_751_MOESM7_ESM.zip › Raw_data_Figure 3/Figure 3D/WTcav1 zoom.tif]

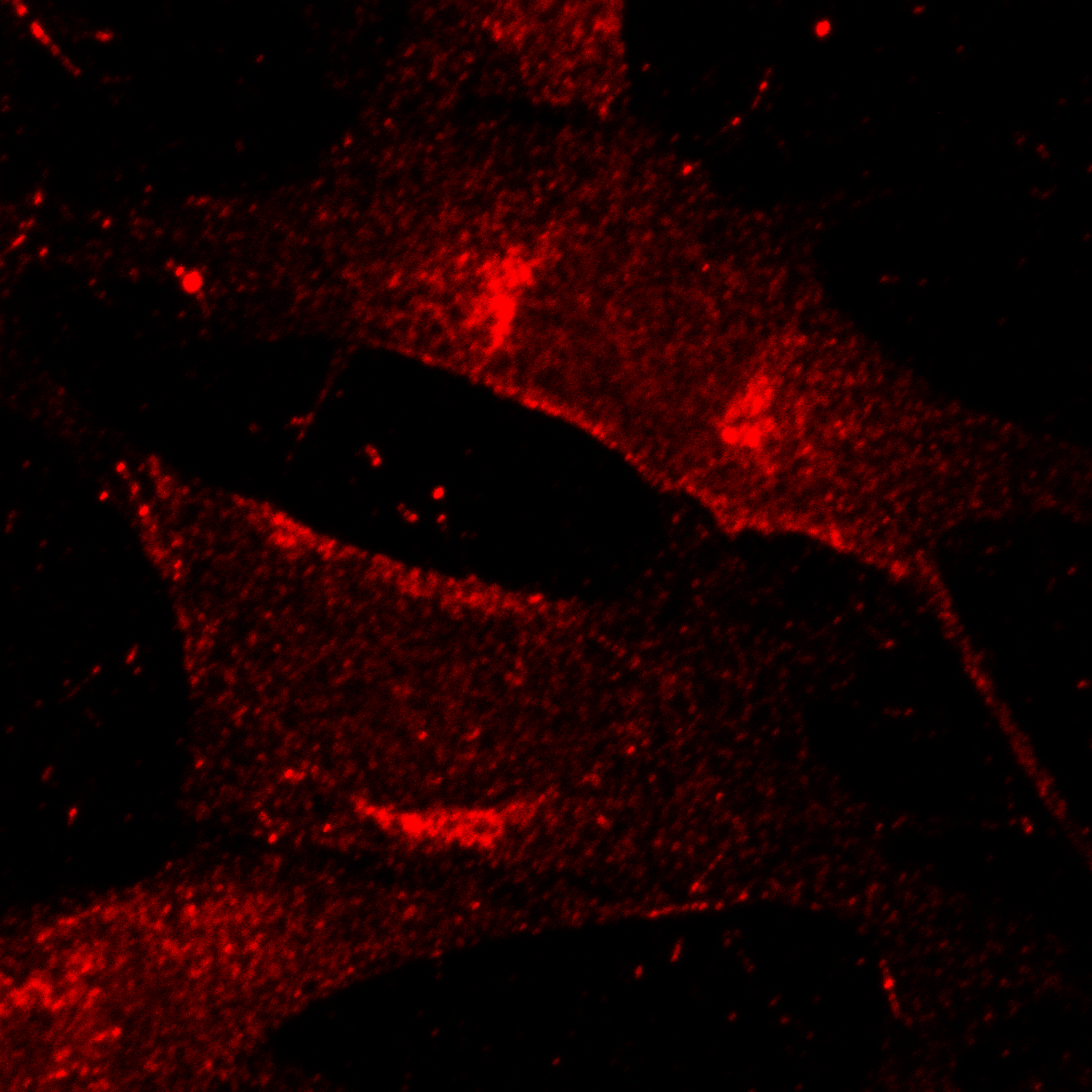

Supplement: Supplementary file 7 — Source data Fig. 3 [file 44319_2026_751_MOESM7_ESM.zip › Raw_data_Figure 3/Figure 3D/WTcav2 zoom.tif]

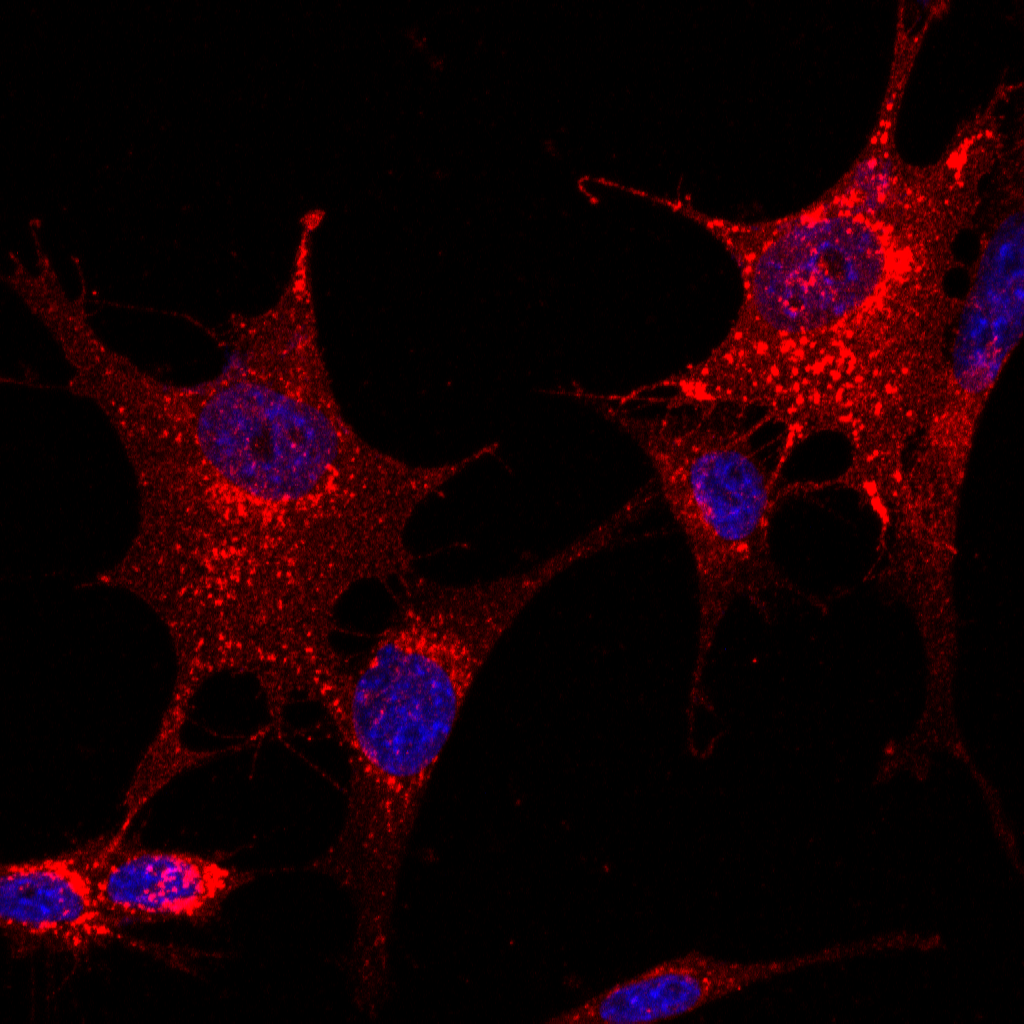

Supplement: Supplementary file 7 — Source data Fig. 3 [file 44319_2026_751_MOESM7_ESM.zip › Raw_data_Figure 3/Figure 3E/GqKO pdgfr_nuclei_merge.tif]

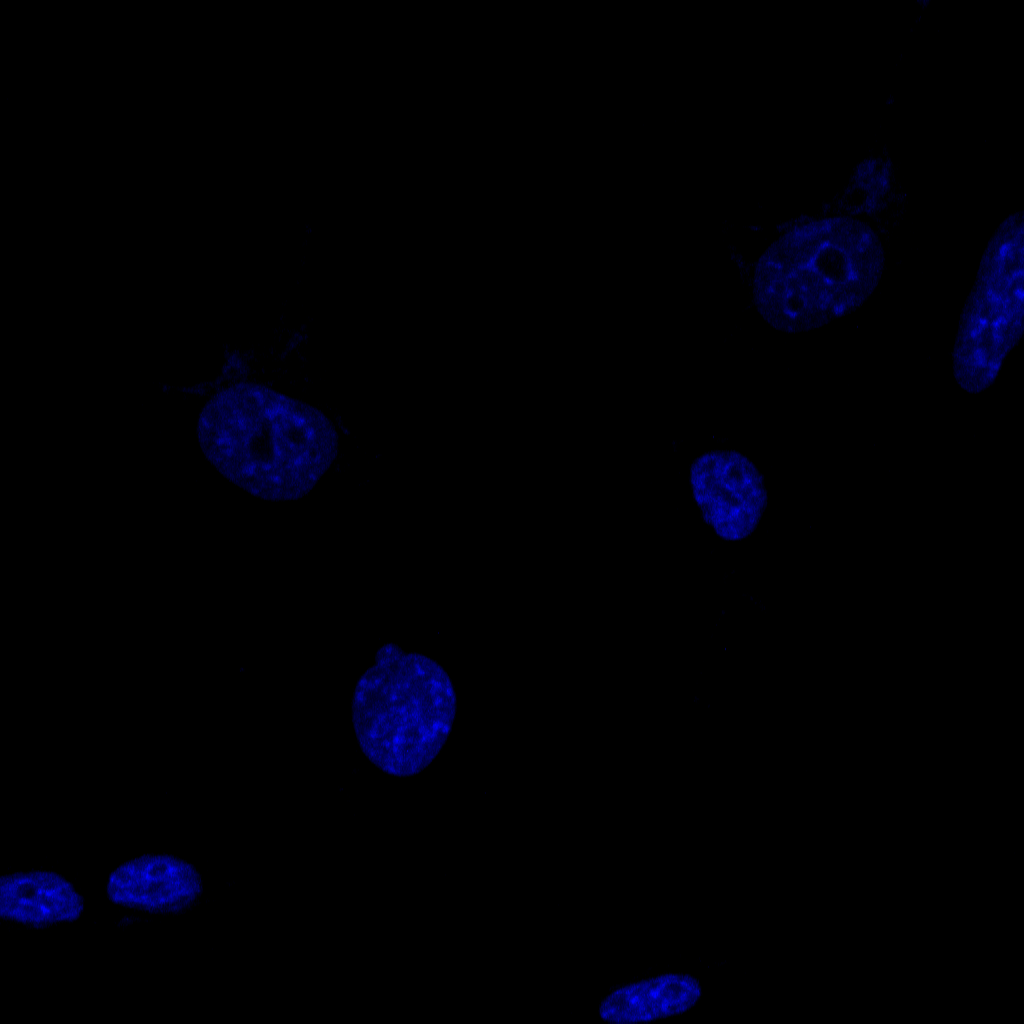

Supplement: Supplementary file 7 — Source data Fig. 3 [file 44319_2026_751_MOESM7_ESM.zip › Raw_data_Figure 3/Figure 3E/GqKOnuclei alone.tif]

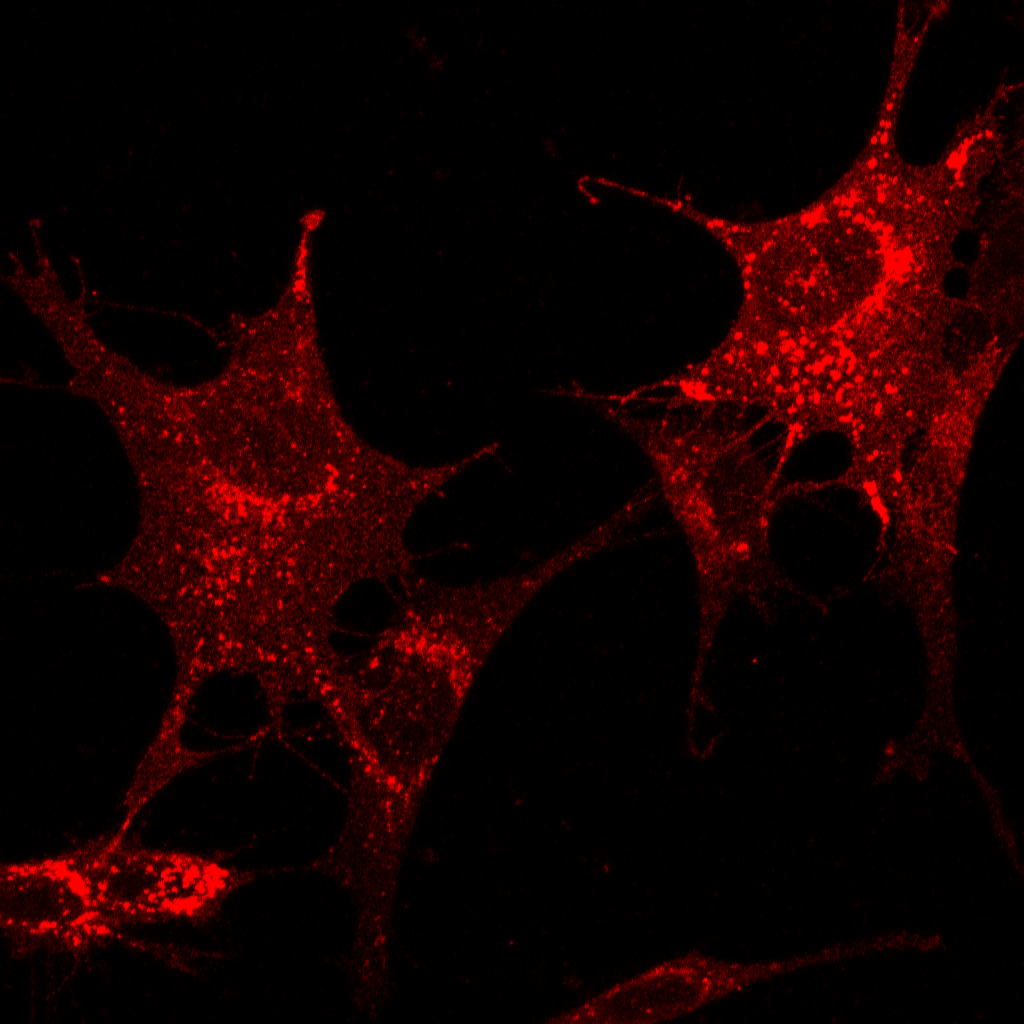

Supplement: Supplementary file 7 — Source data Fig. 3 [file 44319_2026_751_MOESM7_ESM.zip › Raw_data_Figure 3/Figure 3E/GqKOpdgfr alone.tif]

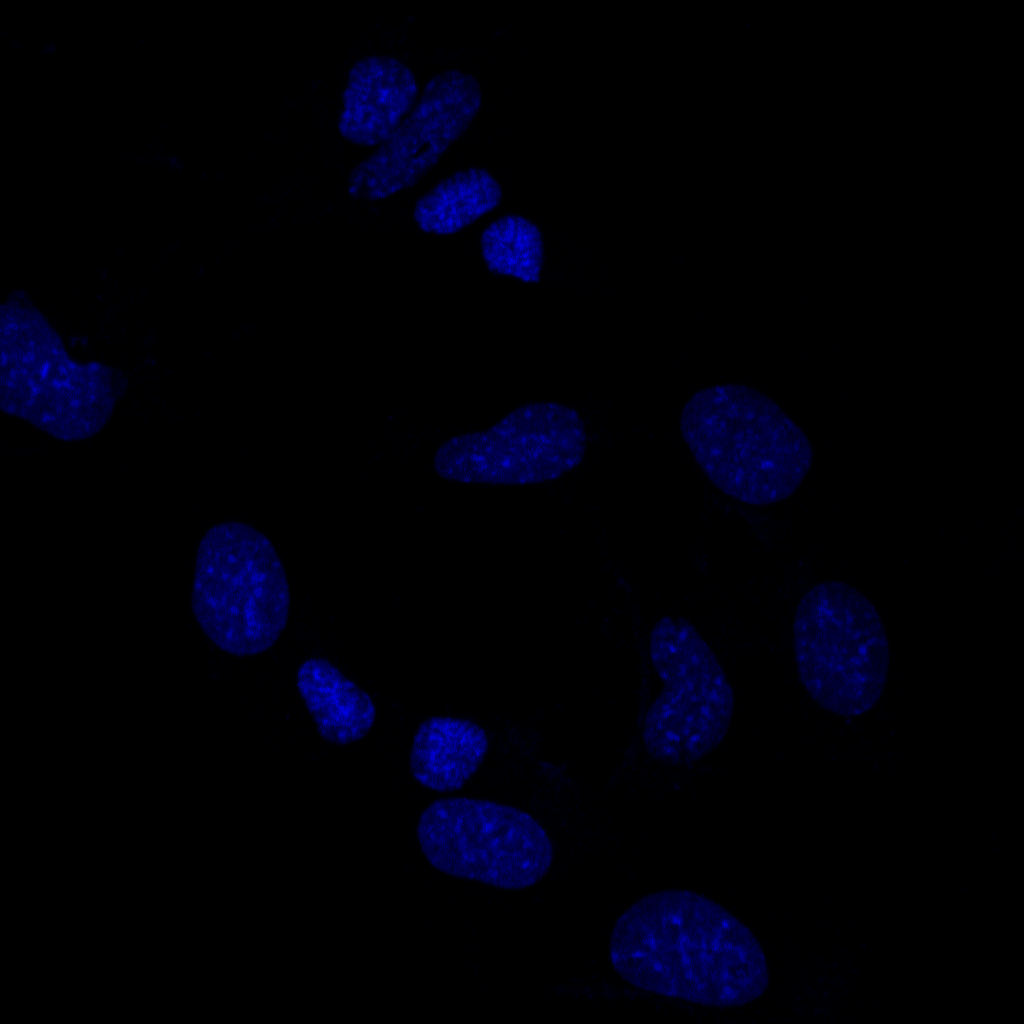

Supplement: Supplementary file 7 — Source data Fig. 3 [file 44319_2026_751_MOESM7_ESM.zip › Raw_data_Figure 3/Figure 3E/KInuclei alone.tif]

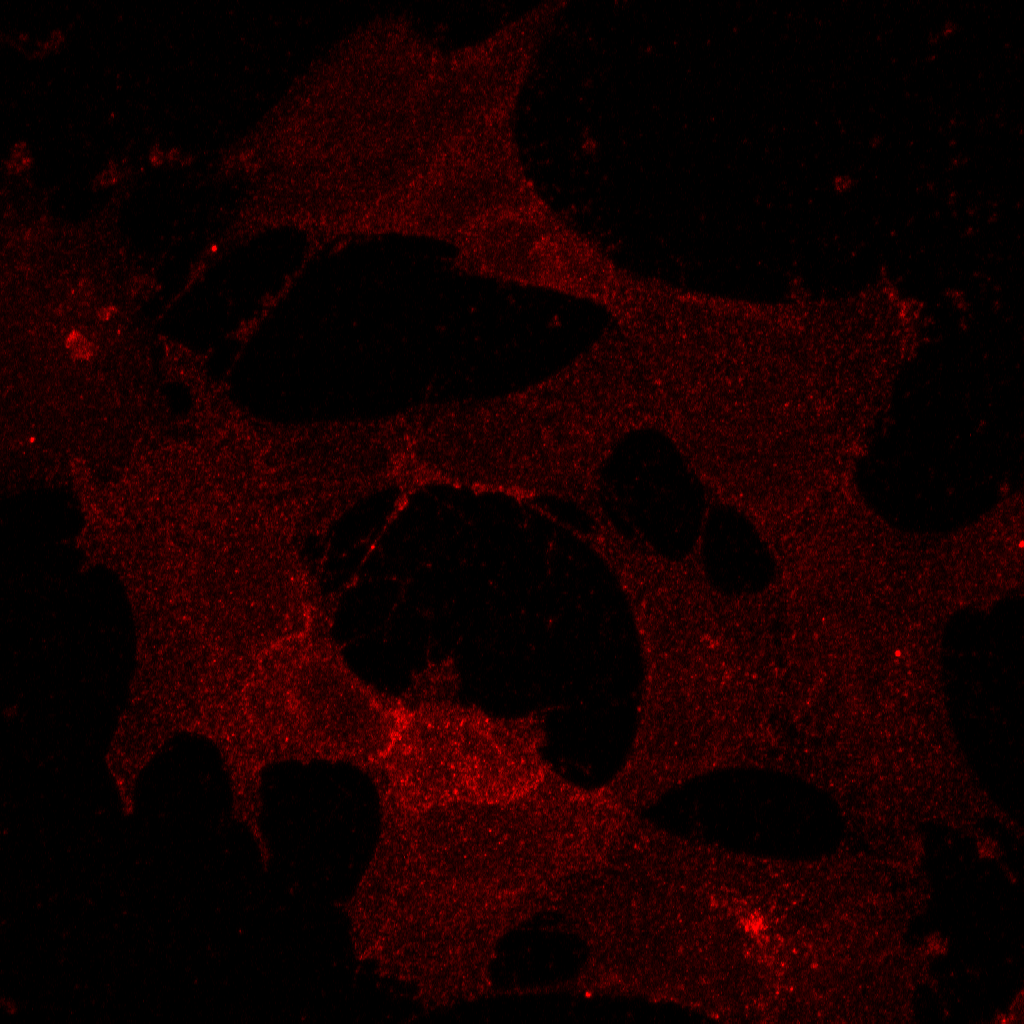

Supplement: Supplementary file 7 — Source data Fig. 3 [file 44319_2026_751_MOESM7_ESM.zip › Raw_data_Figure 3/Figure 3E/KIpdgfr alone.tif]

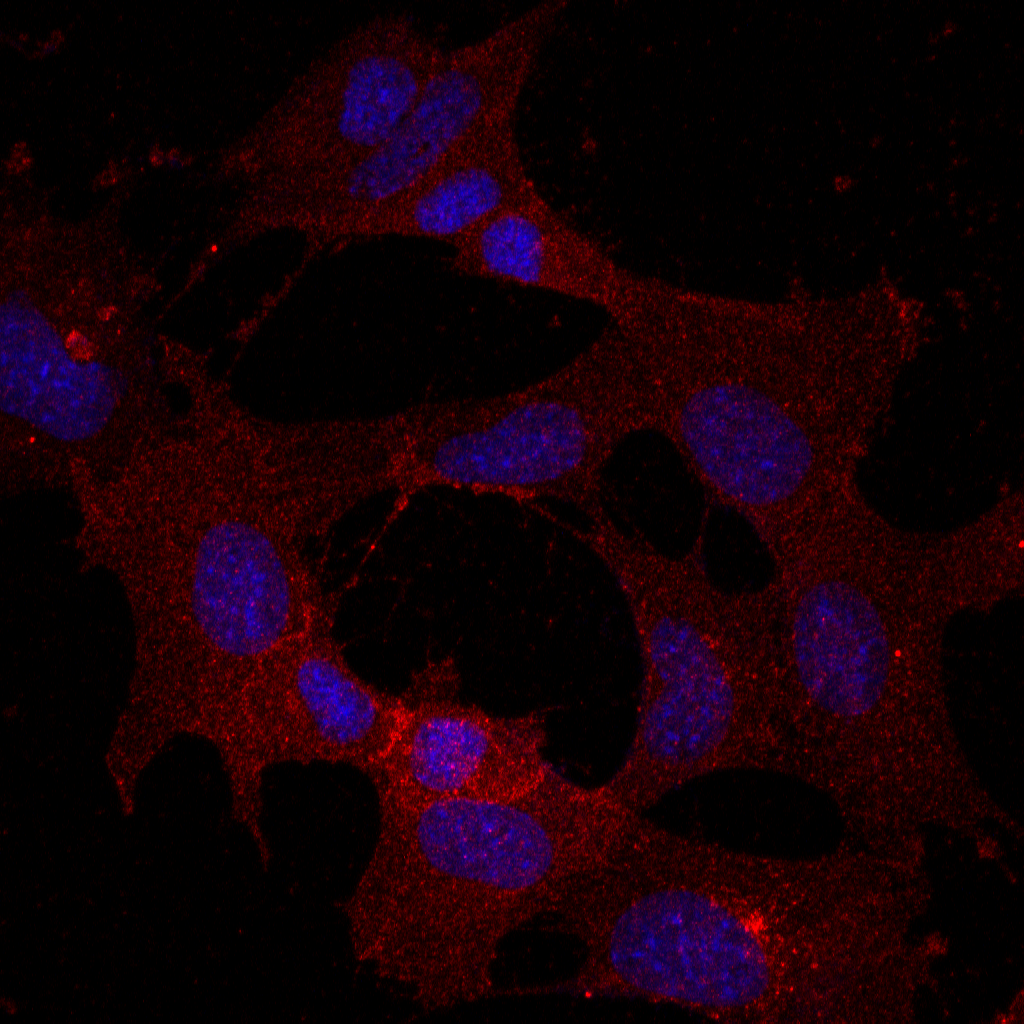

Supplement: Supplementary file 7 — Source data Fig. 3 [file 44319_2026_751_MOESM7_ESM.zip › Raw_data_Figure 3/Figure 3E/KIpdgfr_nuclei_alone.tif]

## Slide 1
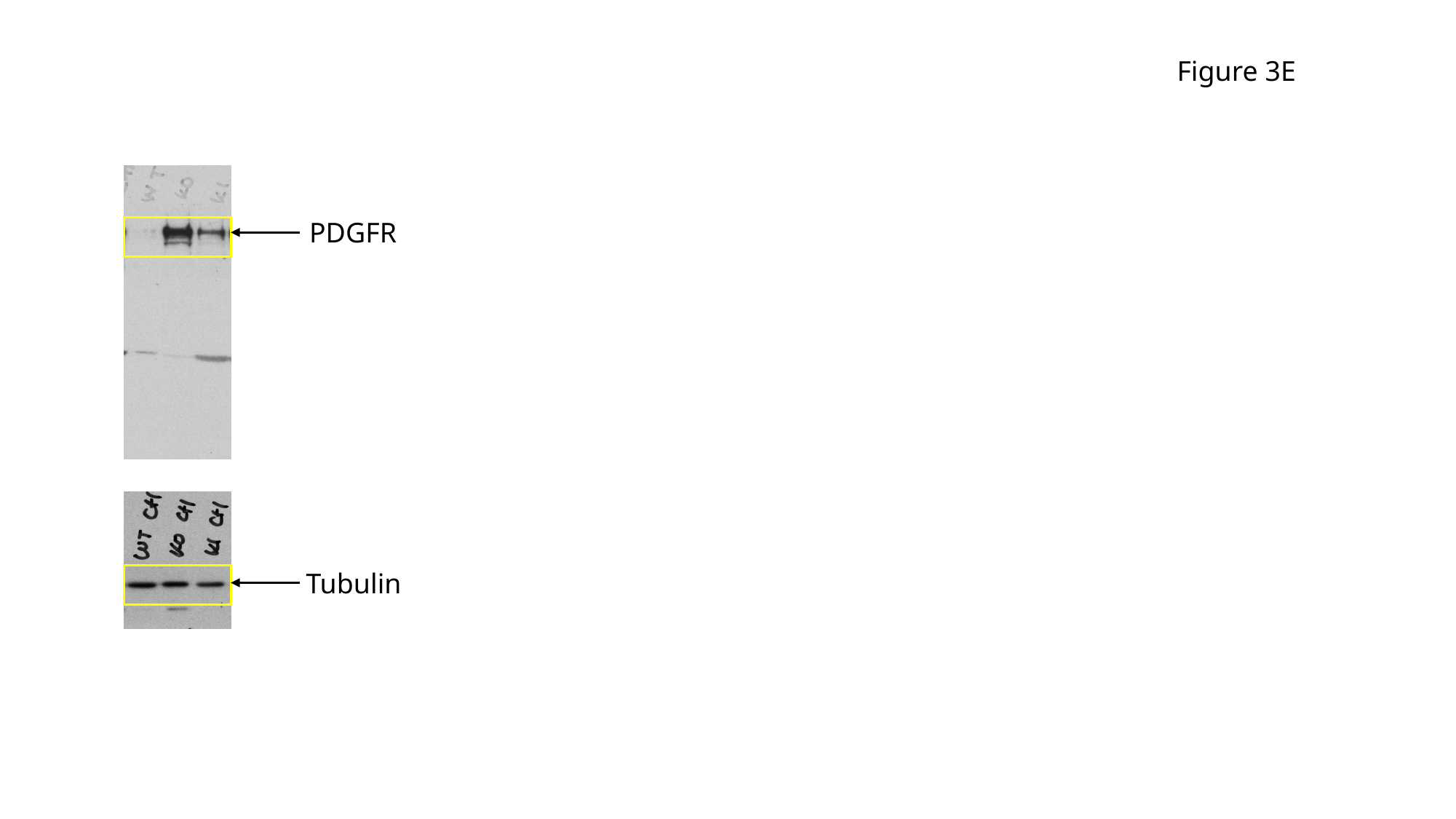

Figure 3E
PDGFR
Tubulin

Supplement: Supplementary file 7 — Source data Fig. 3 [file 44319_2026_751_MOESM7_ESM.zip › Raw_data_Figure 3/Figure 3E/raw_blots_3E.pptx]

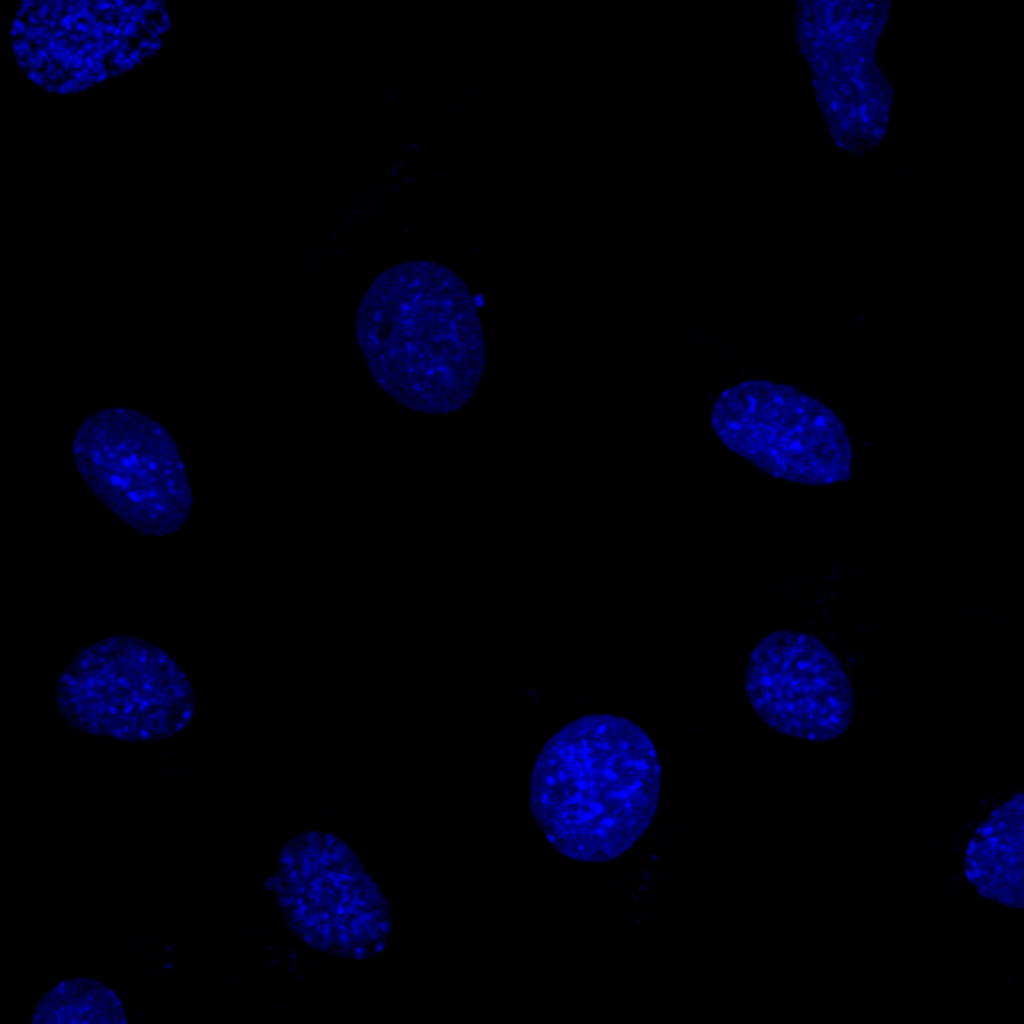

Supplement: Supplementary file 7 — Source data Fig. 3 [file 44319_2026_751_MOESM7_ESM.zip › Raw_data_Figure 3/Figure 3E/WTnuclei alone.tif]

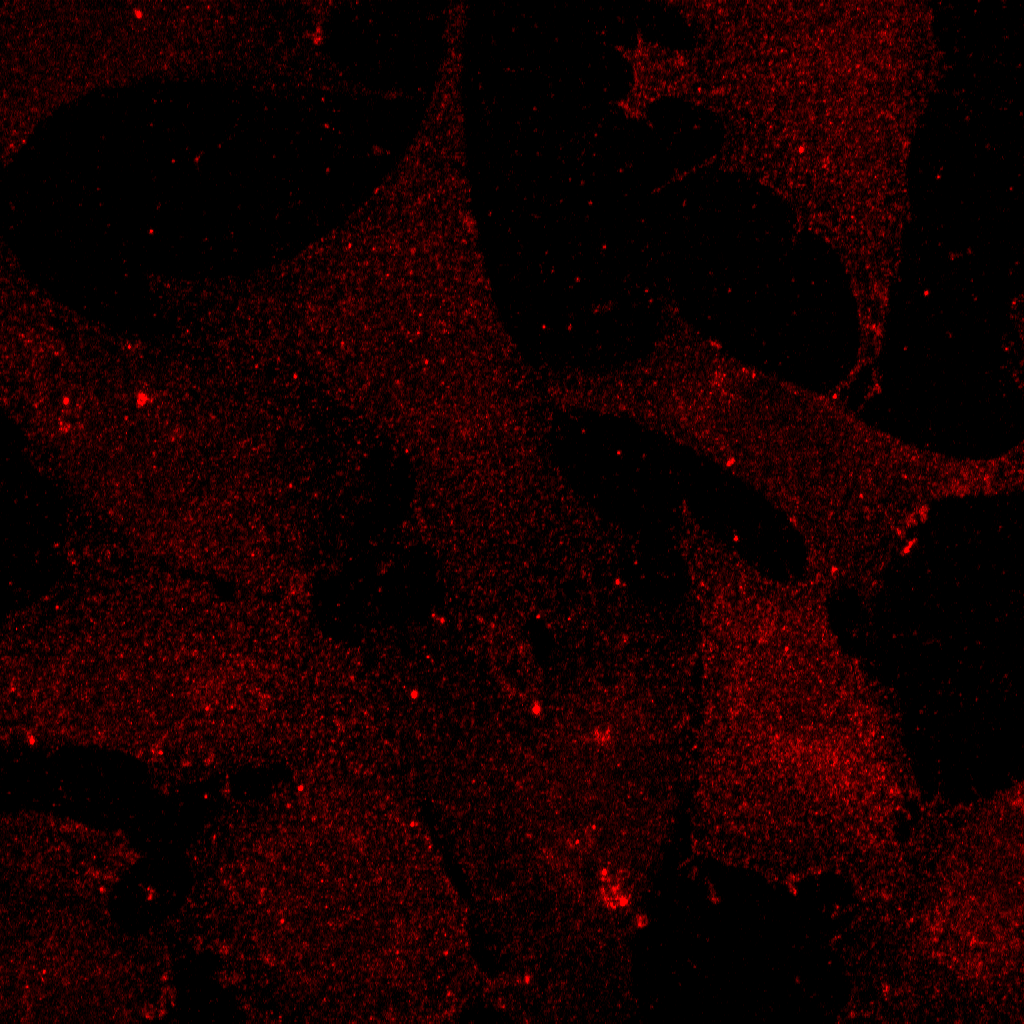

Supplement: Supplementary file 7 — Source data Fig. 3 [file 44319_2026_751_MOESM7_ESM.zip › Raw_data_Figure 3/Figure 3E/WTpdgfr alone.tif]

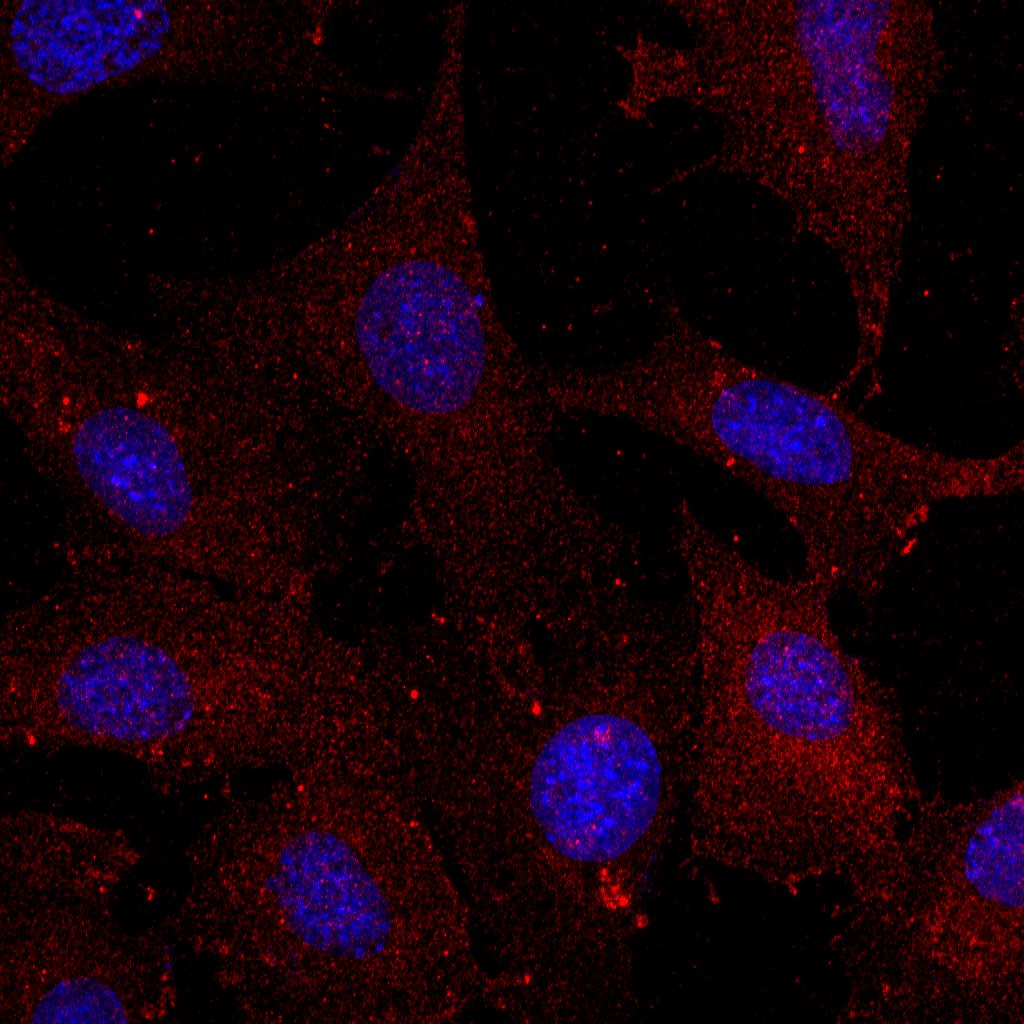

Supplement: Supplementary file 7 — Source data Fig. 3 [file 44319_2026_751_MOESM7_ESM.zip › Raw_data_Figure 3/Figure 3E/WTpdgfr_nuclei_merge.tif]

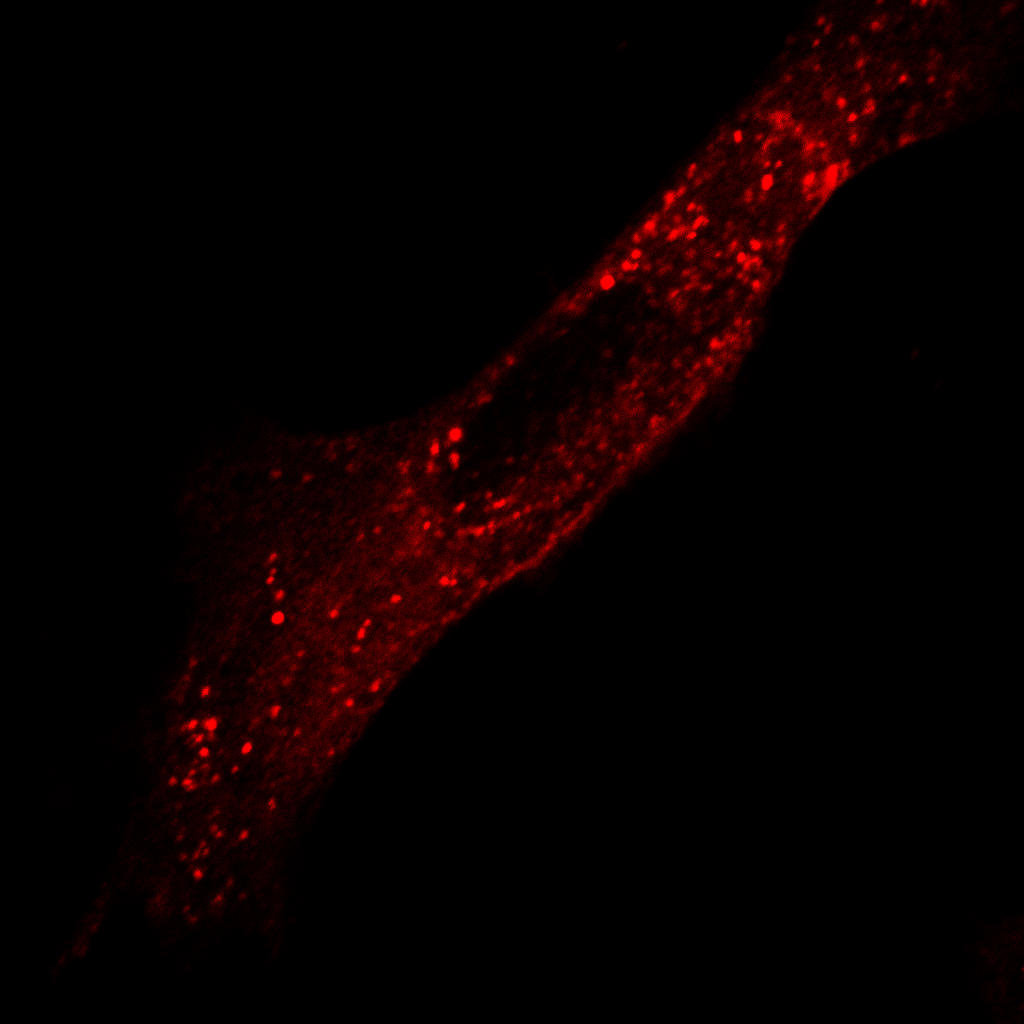

Supplement: Supplementary file 7 — Source data Fig. 3 [file 44319_2026_751_MOESM7_ESM.zip › Raw_data_Figure 3/Figure 3F/GqKO Cav1.tif]

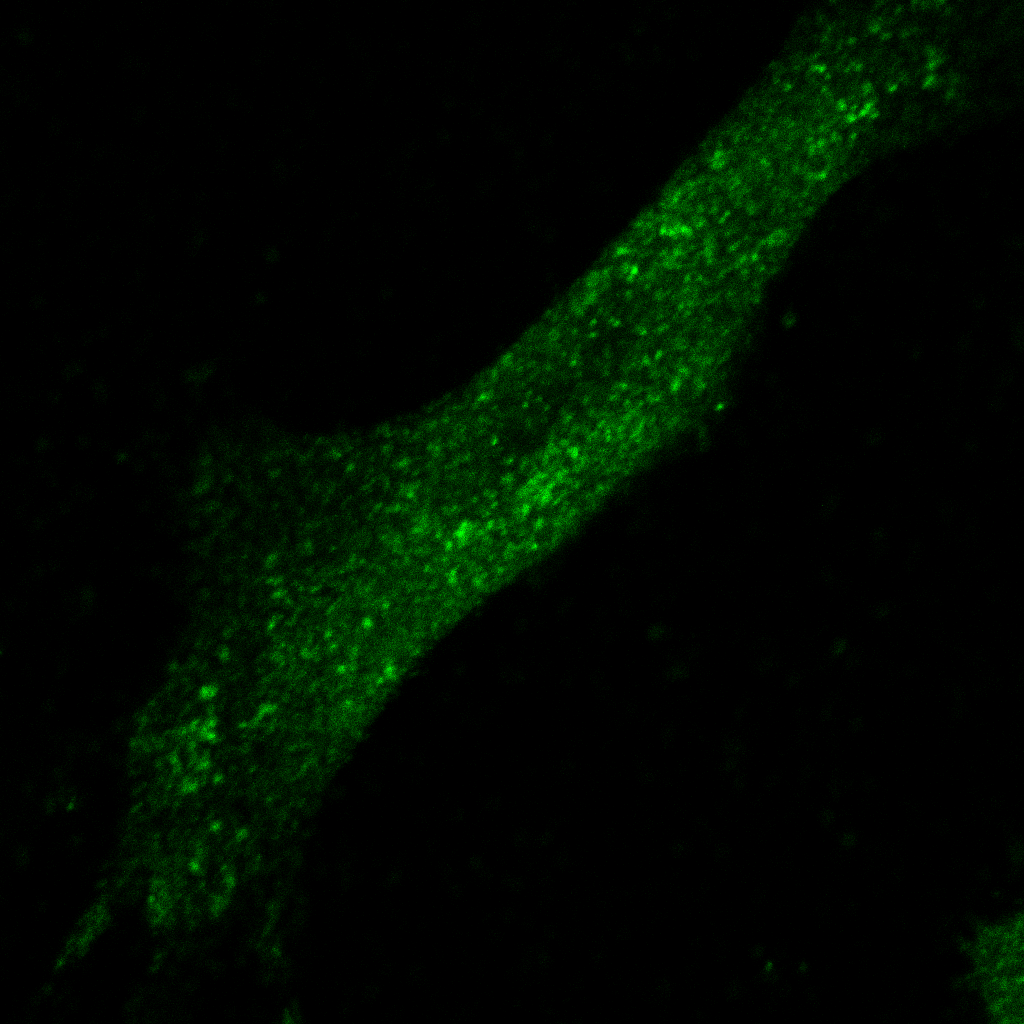

Supplement: Supplementary file 7 — Source data Fig. 3 [file 44319_2026_751_MOESM7_ESM.zip › Raw_data_Figure 3/Figure 3F/GqKO PDGFR.tif]

## Slide 1
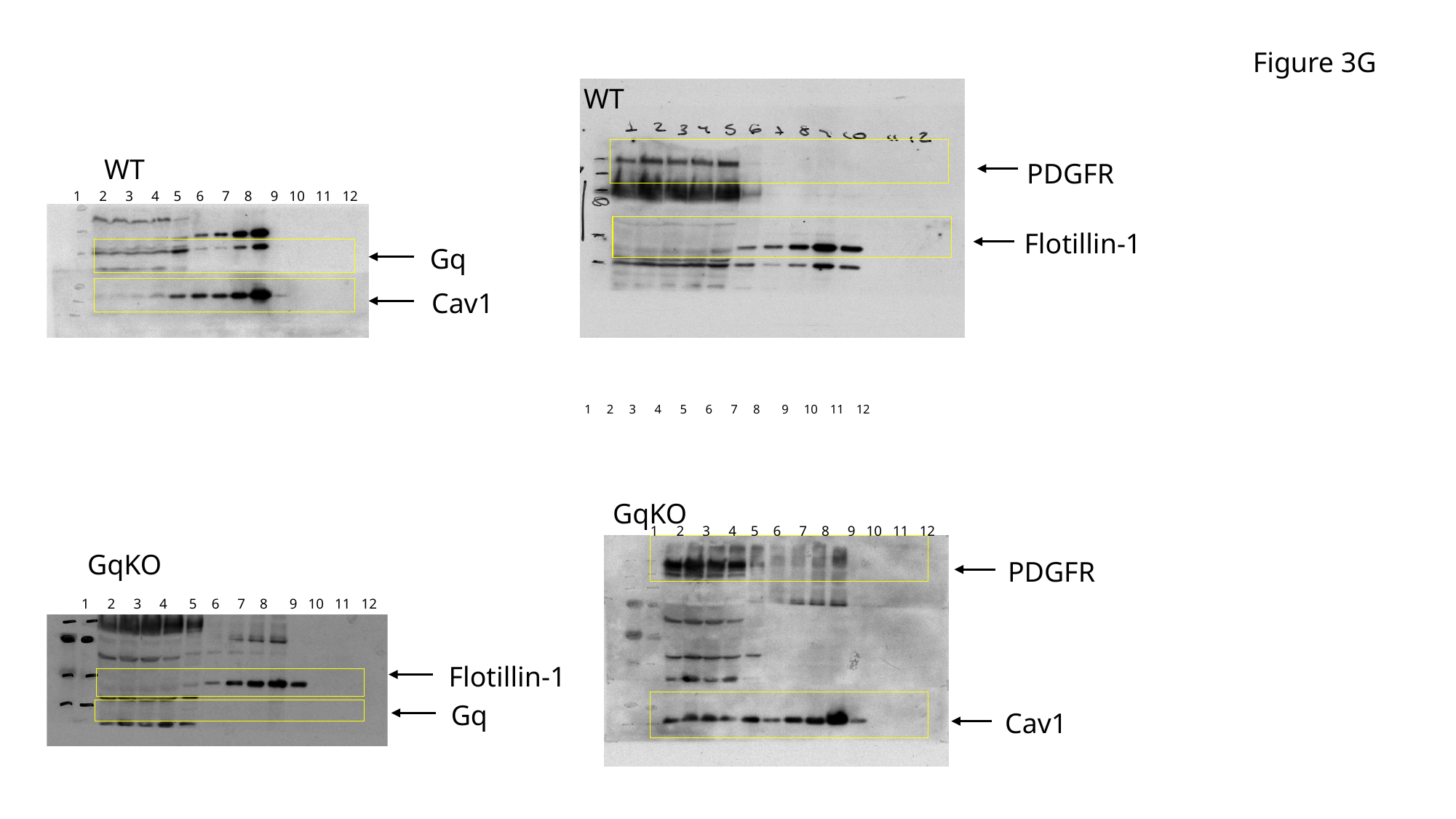

Figure 3G
WT
WT
PDGFR
1 2 3 4 5 6 7 8 9 10 11 12
Gq
Cav1
Flotillin-1
1 2 3 4 5 6 7 8 9 10 11 12
GqKO
1 2 3 4 5 6 7 8 9 10 11 12
GqKO
PDGFR
1 2 3 4 5 6 7 8 9 10 11 12
Flotillin-1
Gq
Cav1

Supplement: Supplementary file 7 — Source data Fig. 3 [file 44319_2026_751_MOESM7_ESM.zip › Raw_data_Figure 3/Figure 3G/raw_blots_3G.pptx]

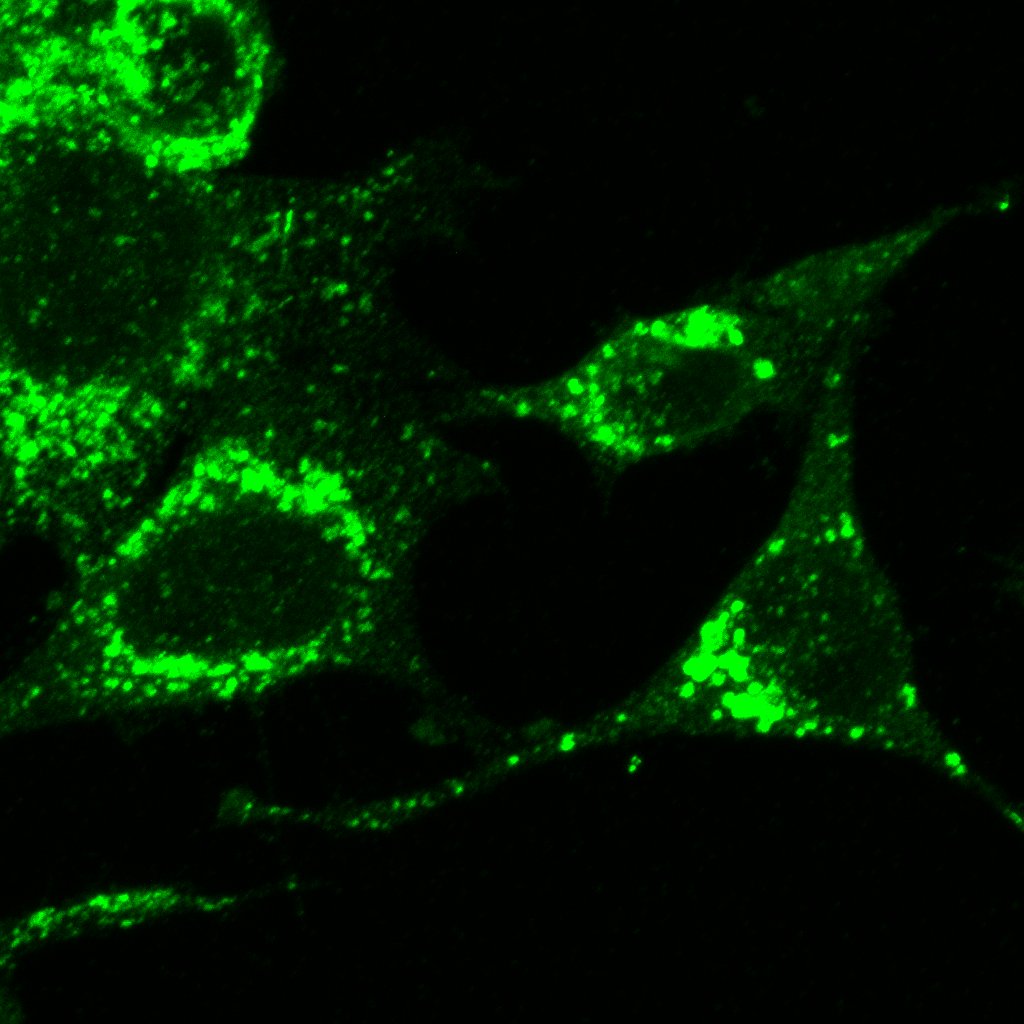

Supplement: Supplementary file 8 — Source data Fig. 4 [file 44319_2026_751_MOESM8_ESM.zip › Raw_data_Figure 4/Figure 4A/GqKO LAMP1 24 h.tif]

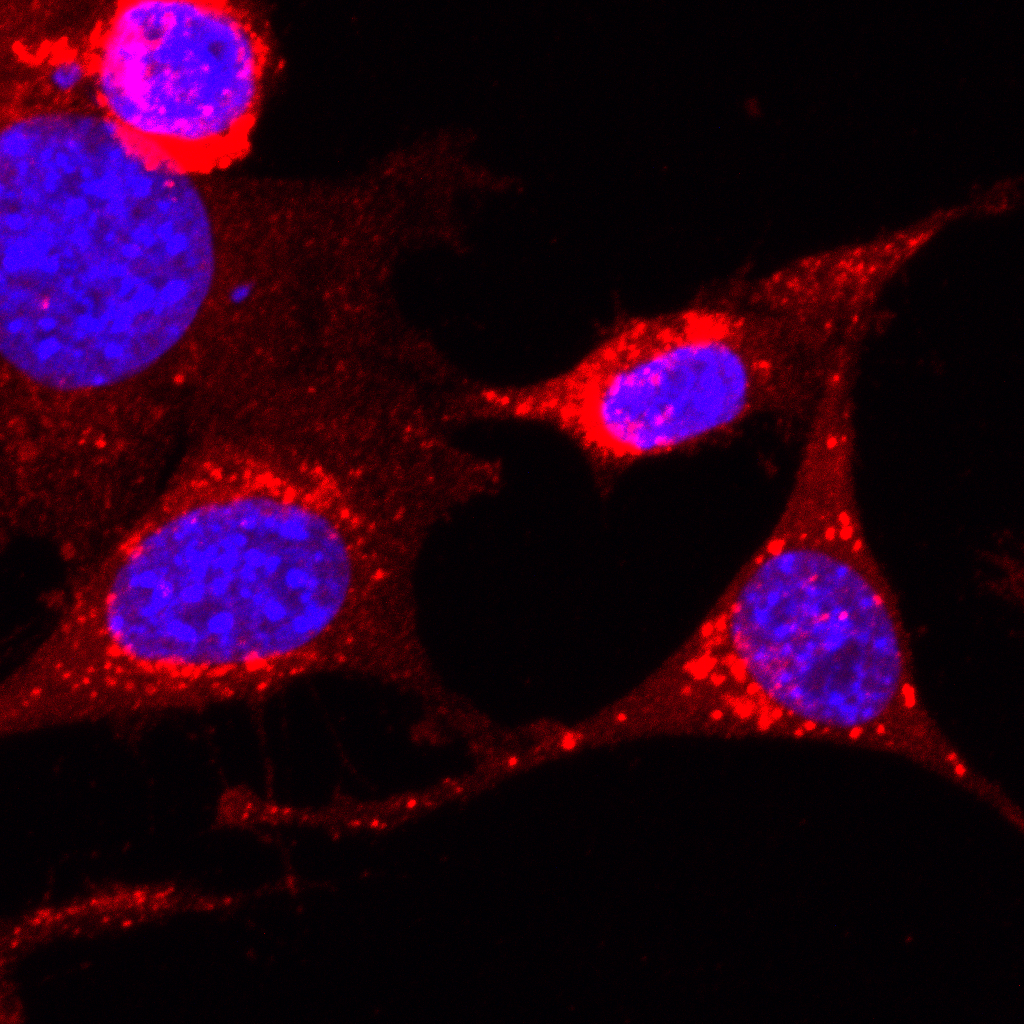

Supplement: Supplementary file 8 — Source data Fig. 4 [file 44319_2026_751_MOESM8_ESM.zip › Raw_data_Figure 4/Figure 4A/GqKO PDGFR 24 h.tif]
