# Supplementary material for: Loss of Gαq reshapes fibroblast traits and drives tumor-stroma remodeling in oral cancer progression
Source: EMBO Rep. 2026 Apr 10;27(10):2639–74. doi: 10.1038/s44319-026-00751-2 (PMC13219523; doi:10.1038/s44319-026-00751-2)
Supplement: Supplementary file 7 — Source data Fig. 3 [file 44319_2026_751_MOESM7_ESM.zip › Raw_data_Figure 3/Figure 3C/Presentaciónheatmap.pptx]

## Slide 1
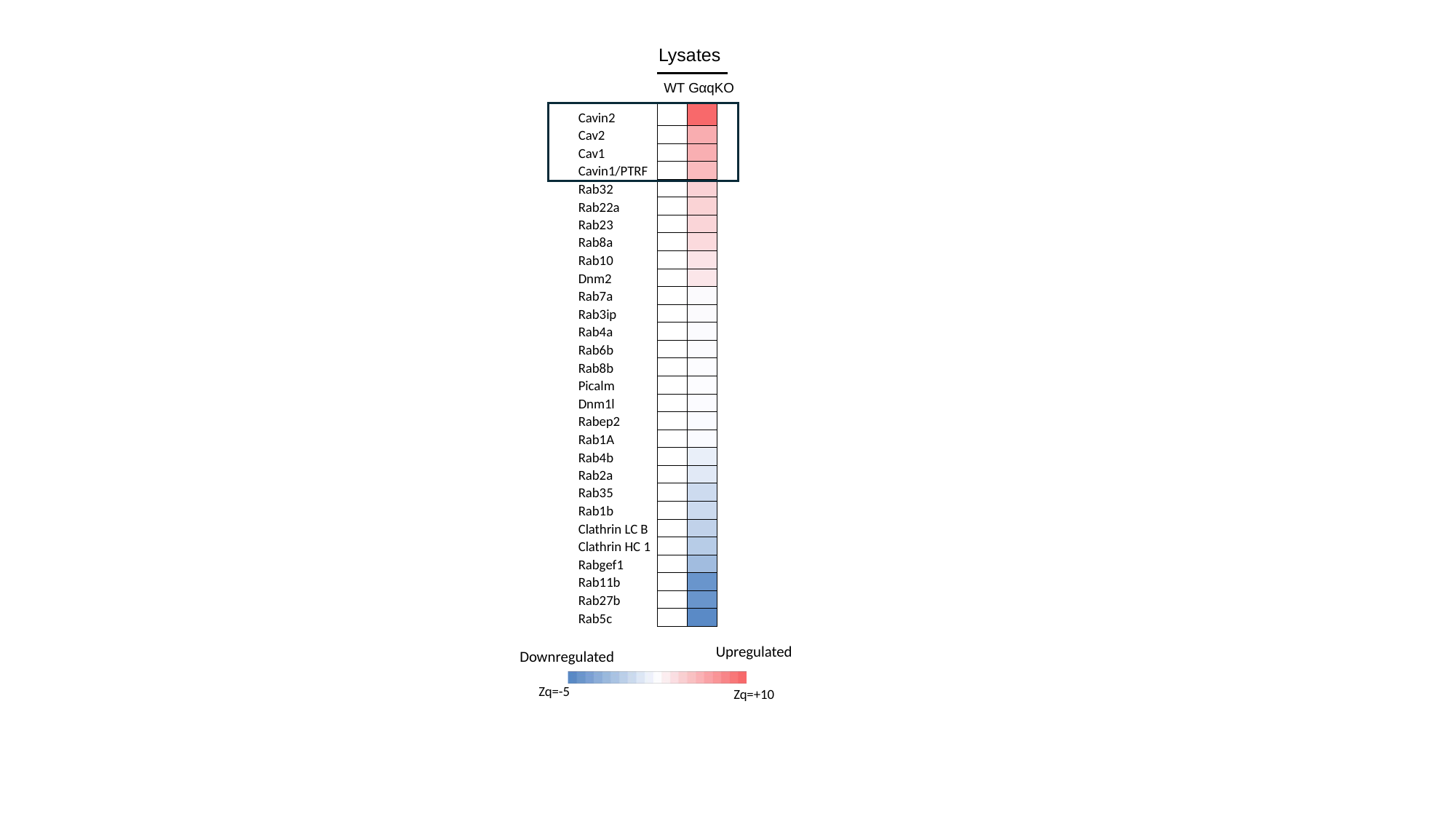

Lysates
WT GαqKO
| | |
| --- | --- |
| | |
| | |
| | |
| | |
| | |
| | |
| | |
| | |
| | |
| | |
| | |
| | |
| | |
| | |
| | |
| | |
| | |
| | |
| | |
| | |
| | |
| | |
| | |
| | |
| | |
| | |
| | |
| | |
| Cavin2 |
| --- |
| Cav2 |
| Cav1 |
| Cavin1/PTRF |
| Rab32 |
| Rab22a |
| Rab23 |
| Rab8a |
| Rab10 |
| Dnm2 |
| Rab7a |
| Rab3ip |
| Rab4a |
| Rab6b |
| Rab8b |
| Picalm |
| Dnm1l |
| Rabep2 |
| Rab1A |
| Rab4b |
| Rab2a |
| Rab35 |
| Rab1b |
| Clathrin LC B |
| Clathrin HC 1 |
| Rabgef1 |
| Rab11b |
| Rab27b |
| Rab5c |
Upregulated
Downregulated
Zq=-5
Zq=+10
