# Supplementary figures and images for: Loss of Gαq reshapes fibroblast traits and drives tumor-stroma remodeling in oral cancer progression (part 3 of 5)
Source: EMBO Rep. 2026 Apr 10;27(10):2639–74. doi: 10.1038/s44319-026-00751-2 (PMC13219523; doi:10.1038/s44319-026-00751-2)

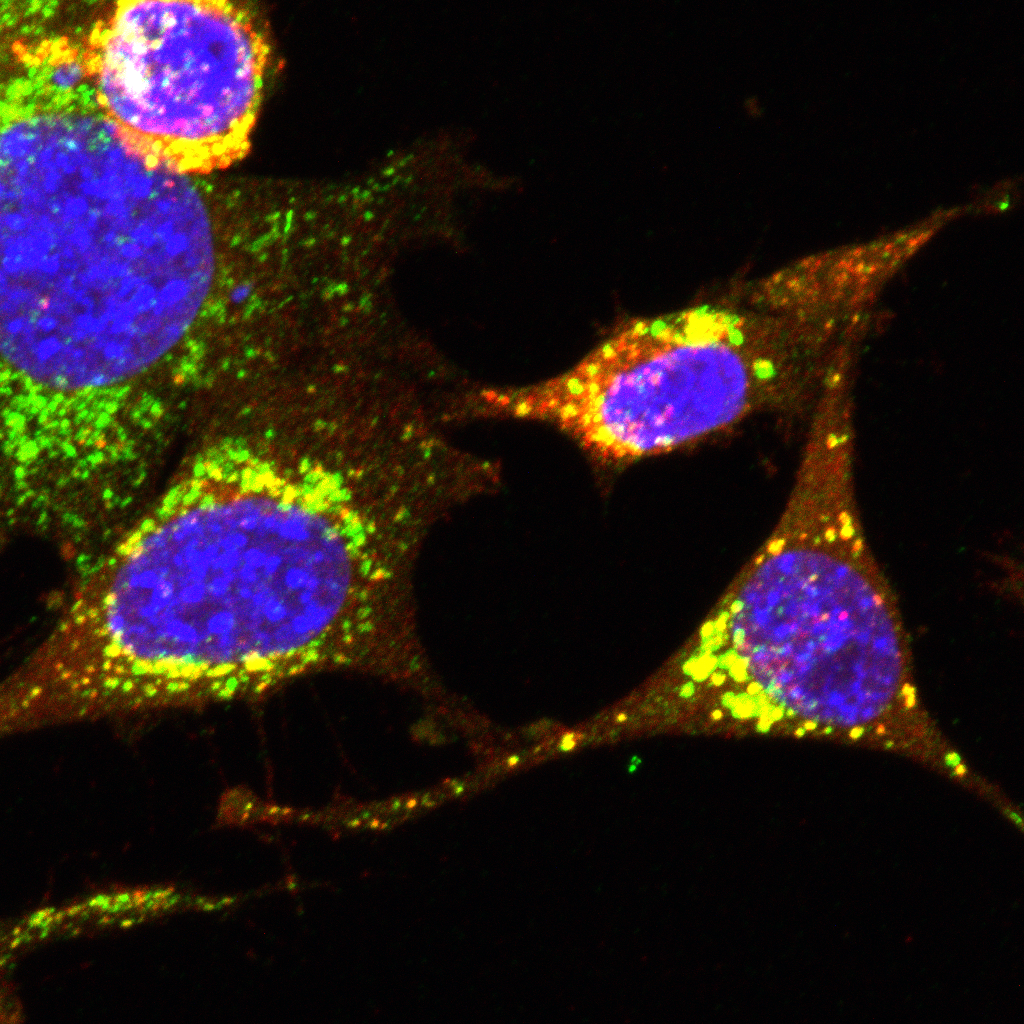

Supplement: Supplementary file 8 — Source data Fig. 4 [file 44319_2026_751_MOESM8_ESM.zip › Raw_data_Figure 4/Figure 4A/GqKO PDGFR_LAMP1 24 h merge.tif]

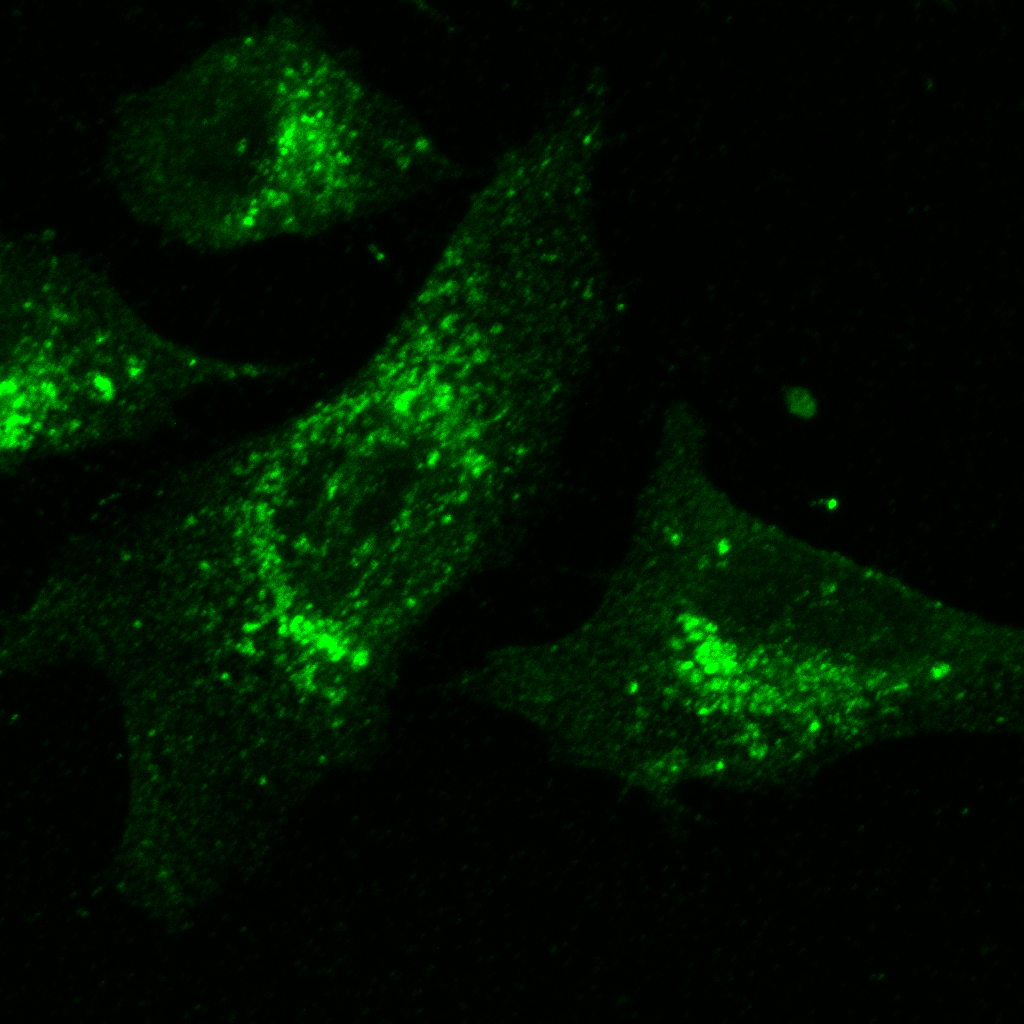

Supplement: Supplementary file 8 — Source data Fig. 4 [file 44319_2026_751_MOESM8_ESM.zip › Raw_data_Figure 4/Figure 4A/WT LAMP1 24 h.tif]

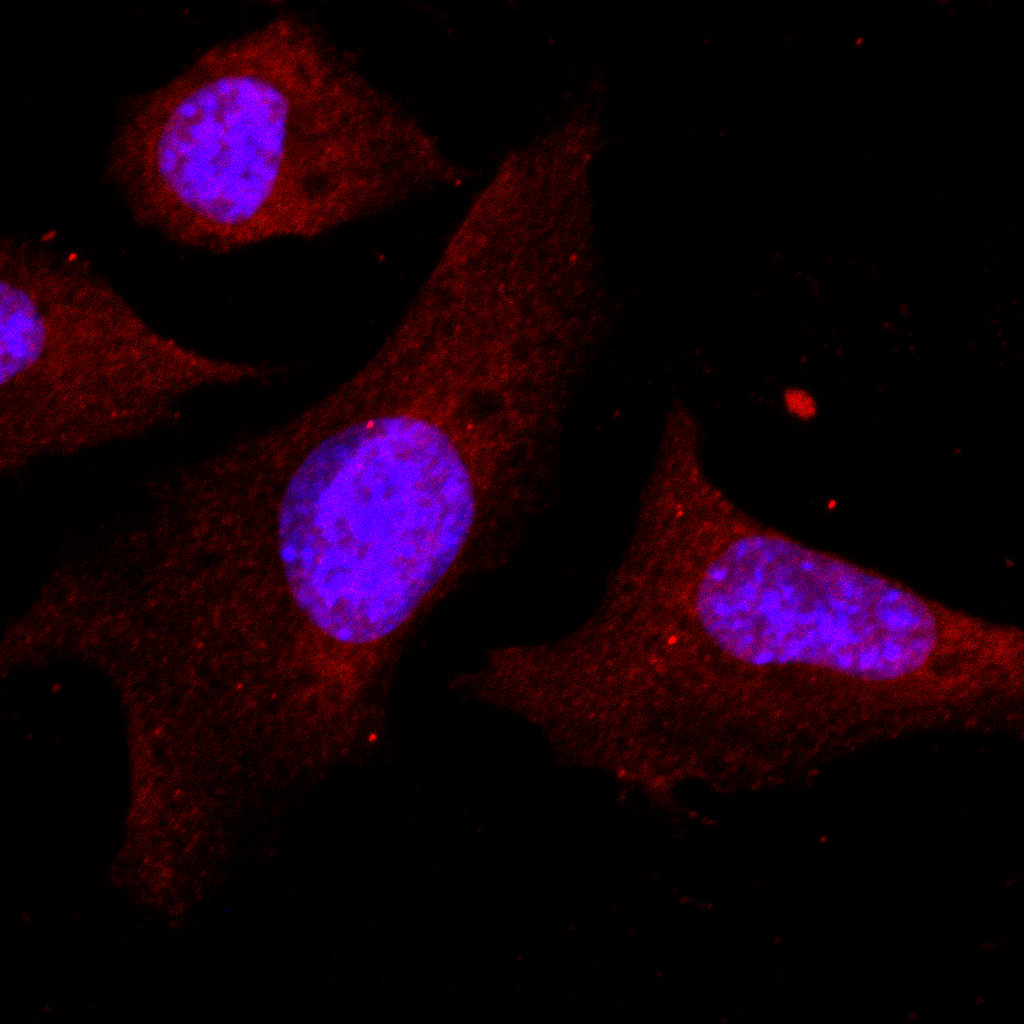

Supplement: Supplementary file 8 — Source data Fig. 4 [file 44319_2026_751_MOESM8_ESM.zip › Raw_data_Figure 4/Figure 4A/WT PDGFR 24 h.tif]

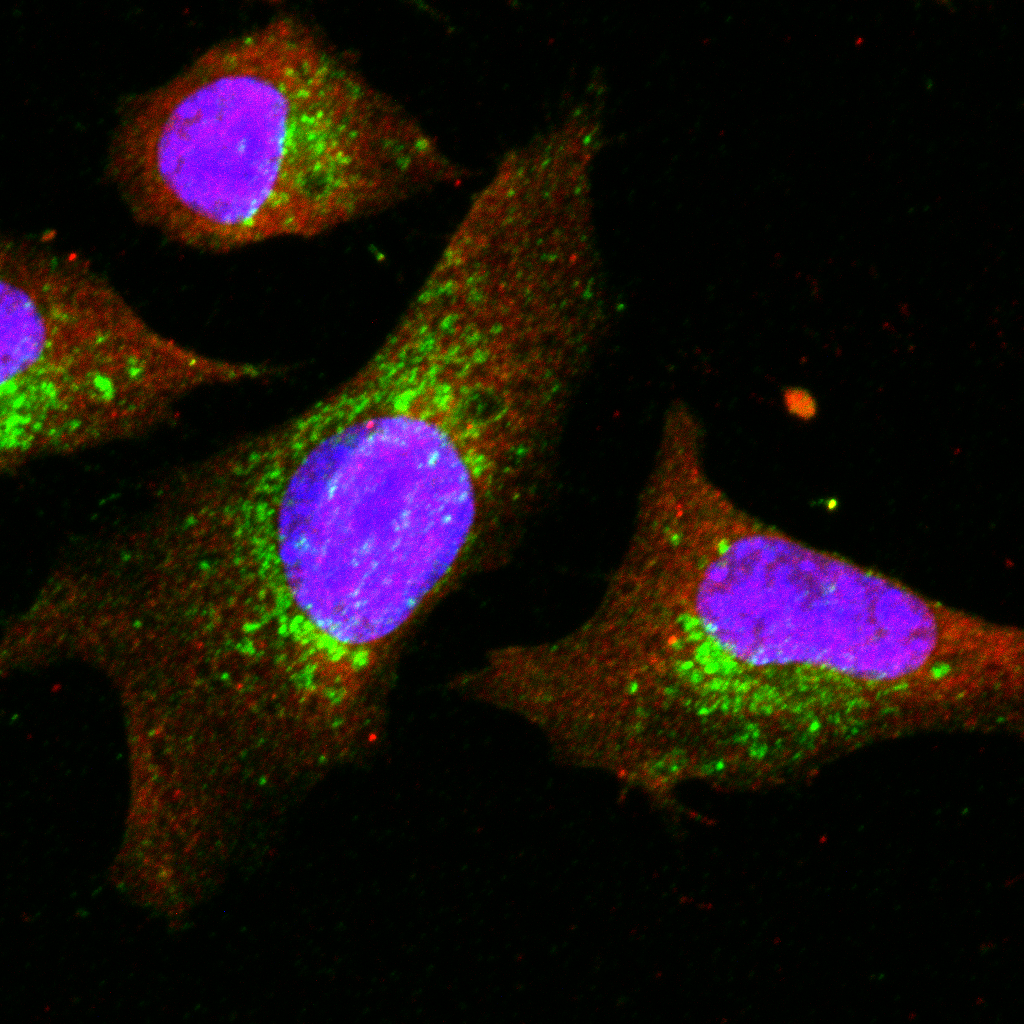

Supplement: Supplementary file 8 — Source data Fig. 4 [file 44319_2026_751_MOESM8_ESM.zip › Raw_data_Figure 4/Figure 4A/WT PDGFR_LMAP 24 h merge.tif]

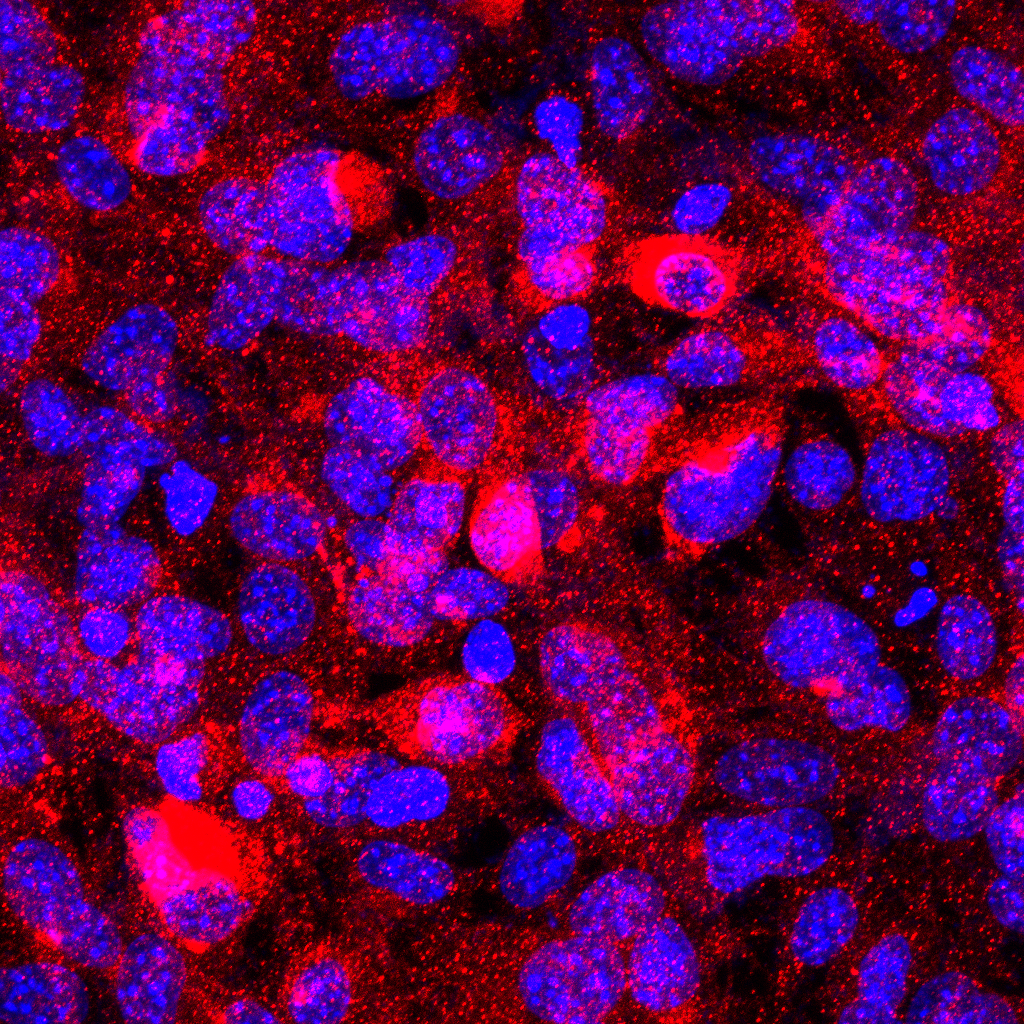

Supplement: Supplementary file 8 — Source data Fig. 4 [file 44319_2026_751_MOESM8_ESM.zip › Raw_data_Figure 4/Figure 4B/KO + PDGF PDGFR.tif]

## Slide 1
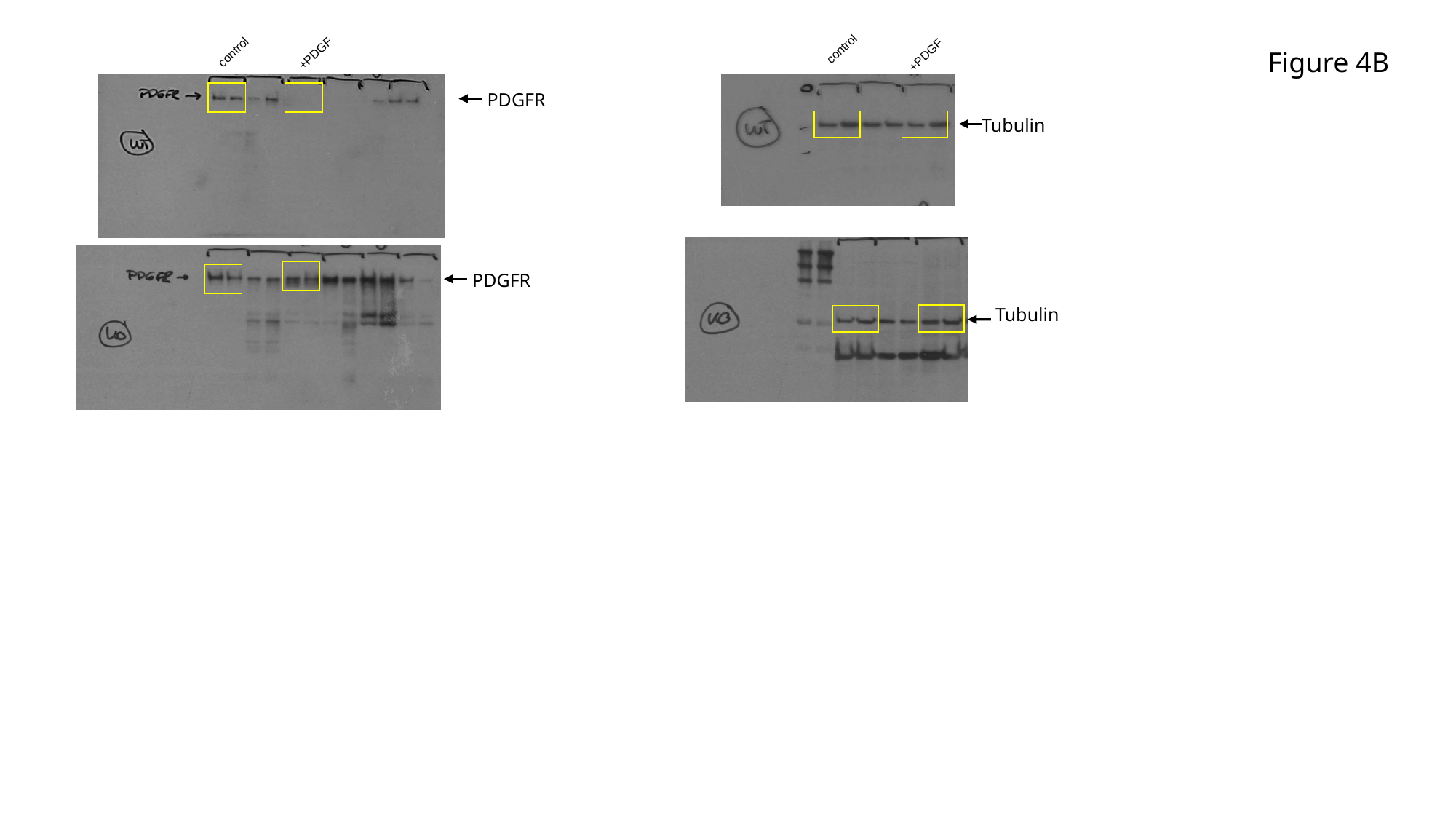

control
control
+PDGF
Figure 4B
+PDGF
PDGFR
Tubulin
Tubulin
PDGFR

Supplement: Supplementary file 8 — Source data Fig. 4 [file 44319_2026_751_MOESM8_ESM.zip › Raw_data_Figure 4/Figure 4B/raw_blots_4B.pptx]

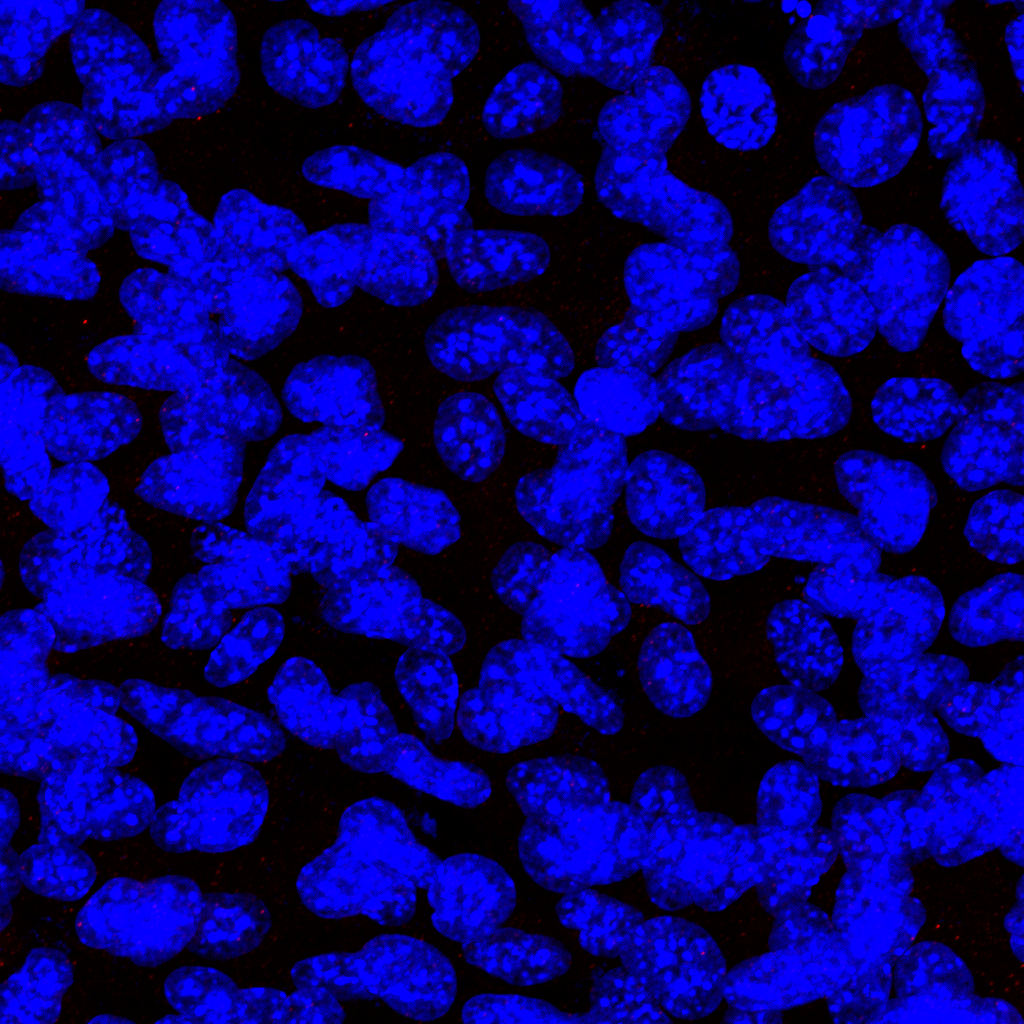

Supplement: Supplementary file 8 — Source data Fig. 4 [file 44319_2026_751_MOESM8_ESM.zip › Raw_data_Figure 4/Figure 4B/WT + PDGF PDGFR.tif]

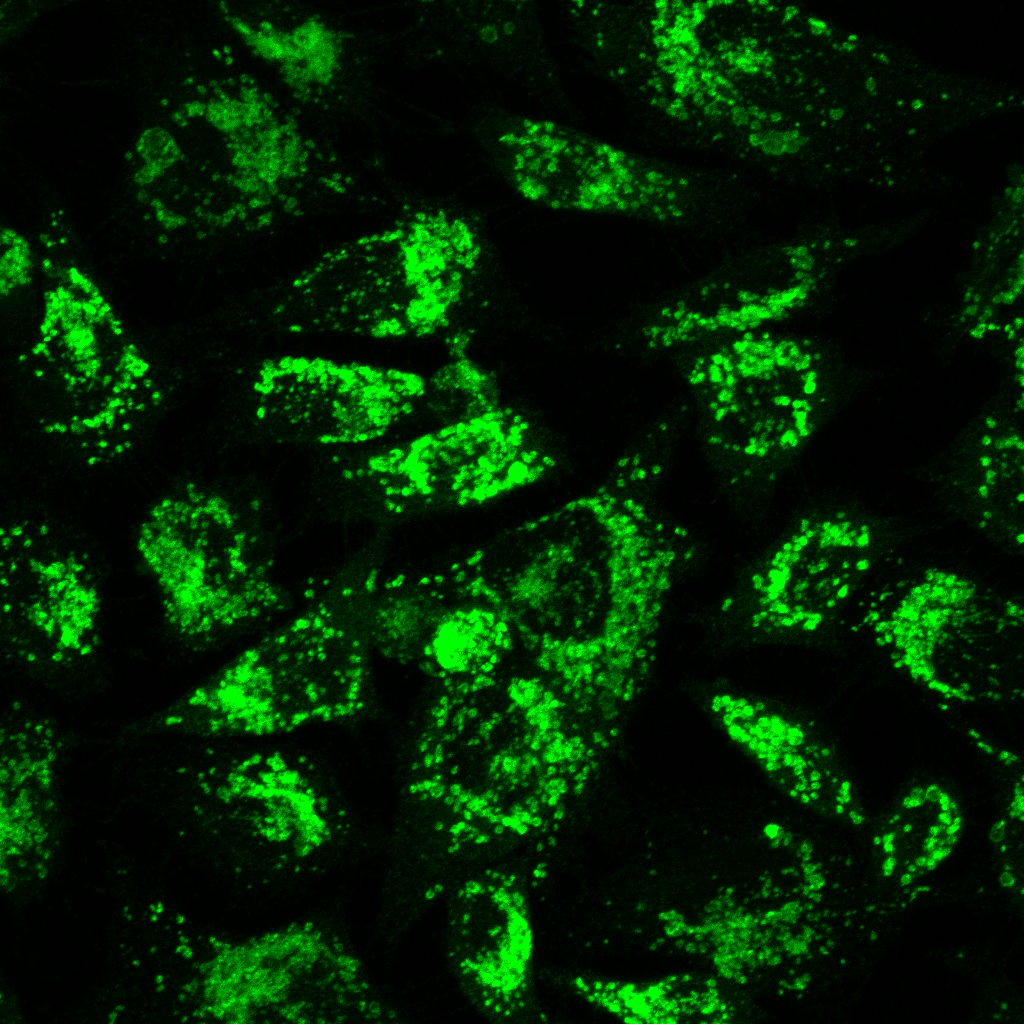

Supplement: Supplementary file 8 — Source data Fig. 4 [file 44319_2026_751_MOESM8_ESM.zip › Raw_data_Figure 4/Figure 4C/WT CQ LAMP1.tif]

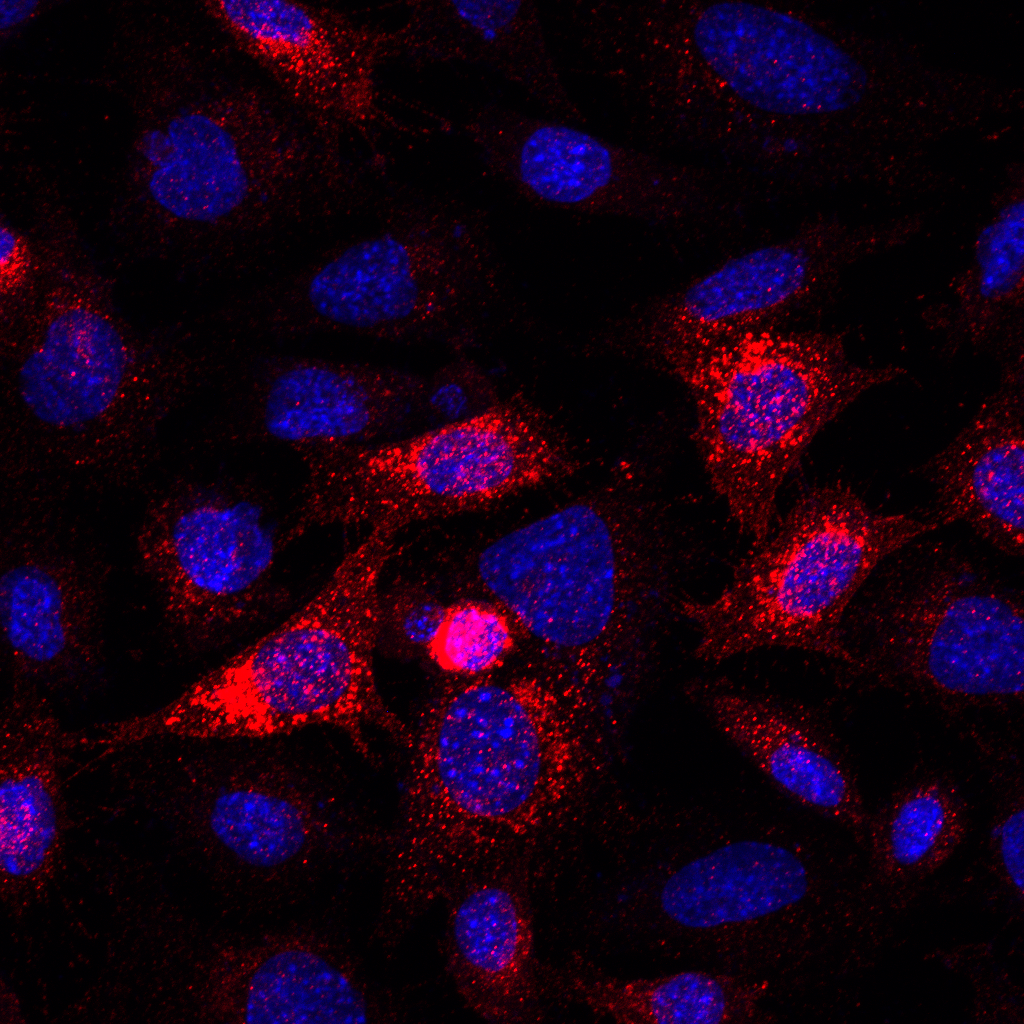

Supplement: Supplementary file 8 — Source data Fig. 4 [file 44319_2026_751_MOESM8_ESM.zip › Raw_data_Figure 4/Figure 4C/WT CQ PDGFR.tif]

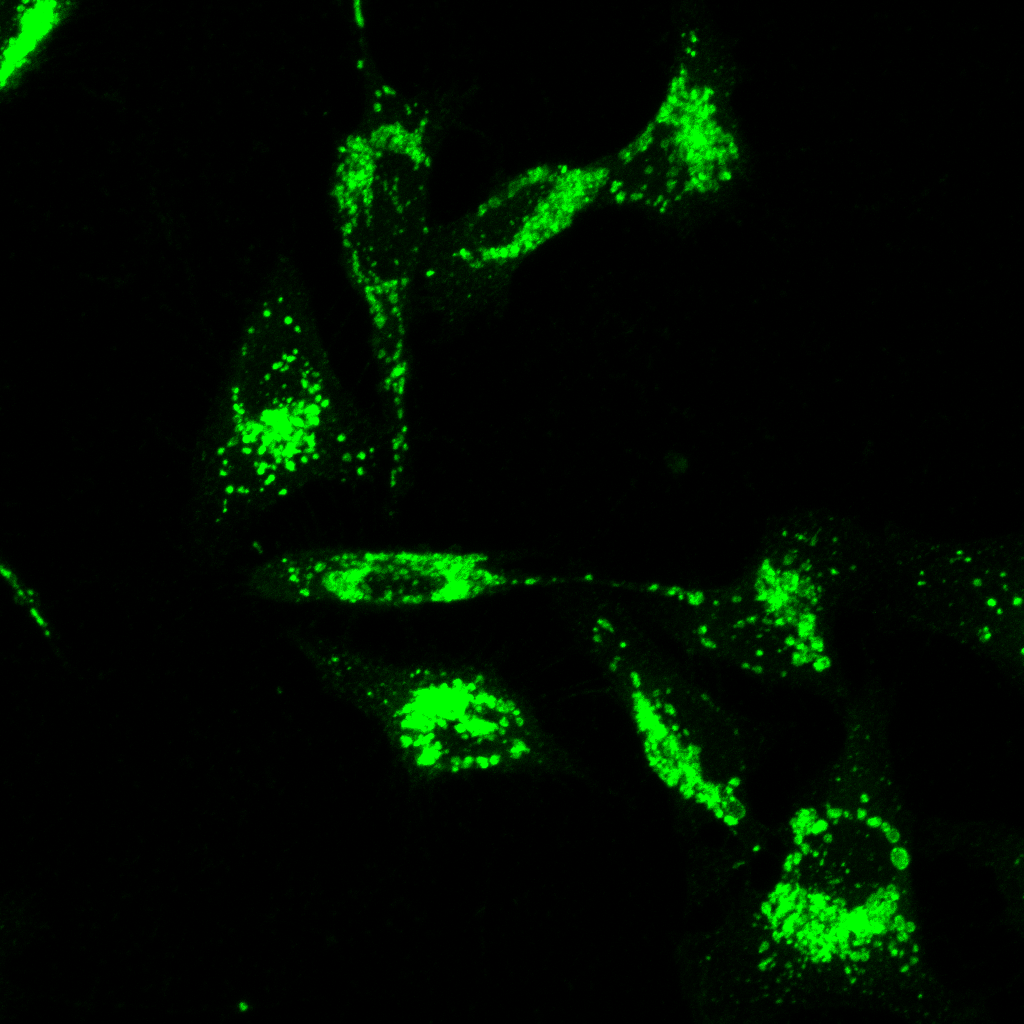

Supplement: Supplementary file 8 — Source data Fig. 4 [file 44319_2026_751_MOESM8_ESM.zip › Raw_data_Figure 4/Figure 4C/WT PDGF-CQ LAMP1.tif]

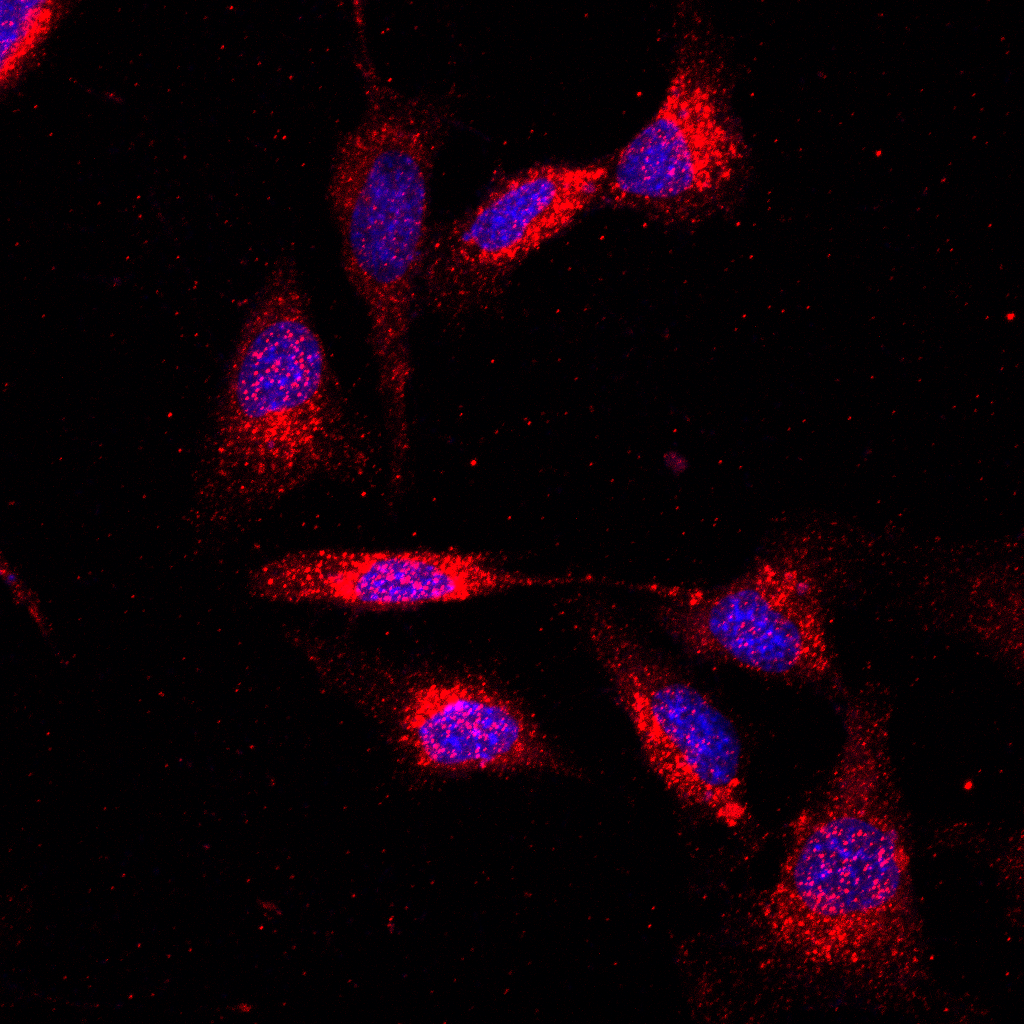

Supplement: Supplementary file 8 — Source data Fig. 4 [file 44319_2026_751_MOESM8_ESM.zip › Raw_data_Figure 4/Figure 4C/WT PDGF-CQ PDGFR .tif]

## Slide 1
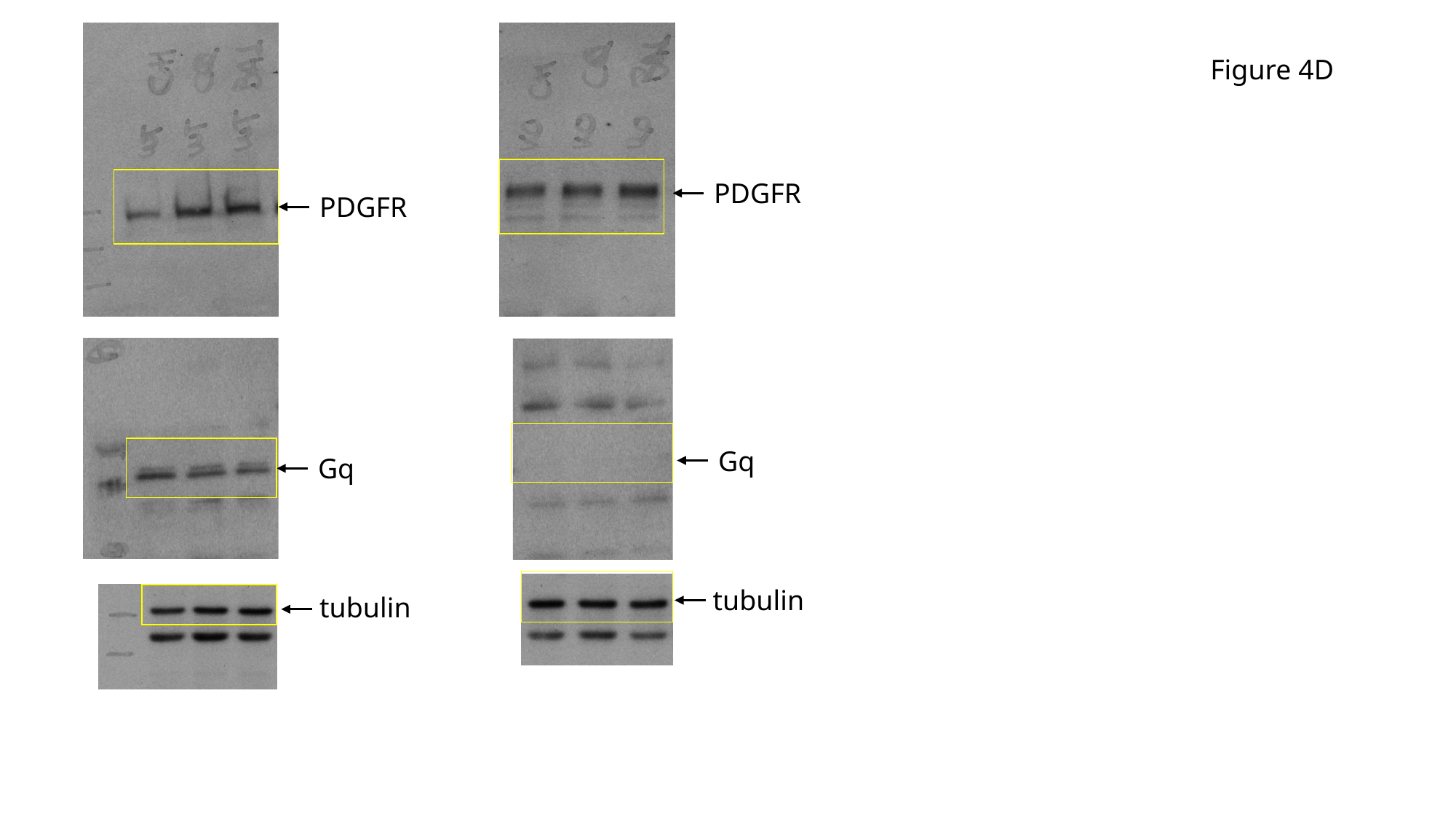

Figure 4D
PDGFR
PDGFR
Gq
Gq
tubulin
tubulin

Supplement: Supplementary file 8 — Source data Fig. 4 [file 44319_2026_751_MOESM8_ESM.zip › Raw_data_Figure 4/Figure 4D/raw_blots_4D.pptx]

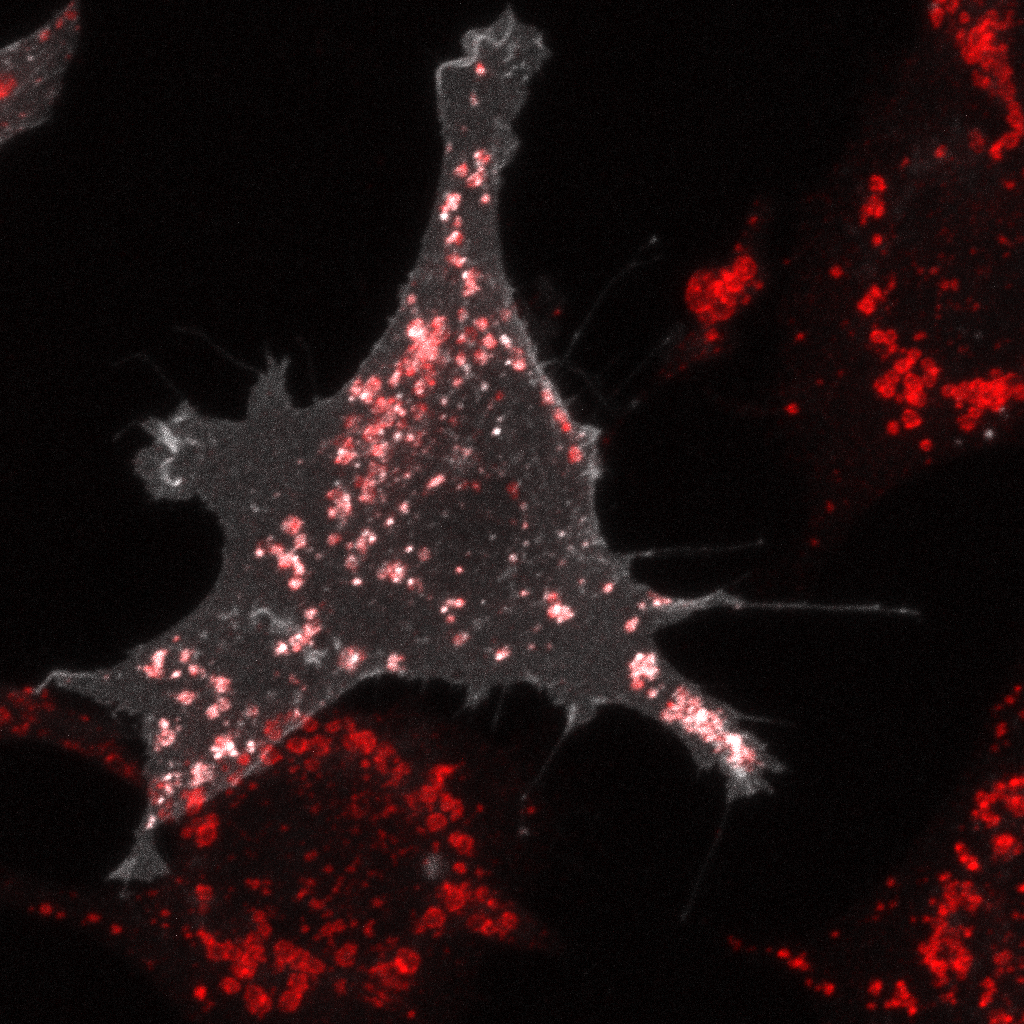

Supplement: Supplementary file 8 — Source data Fig. 4 [file 44319_2026_751_MOESM8_ESM.zip › Raw_data_Figure 4/Figure 4E/Images Figure 4E/GqKO CKSR mRFP LAMP1 red-gray.tif]

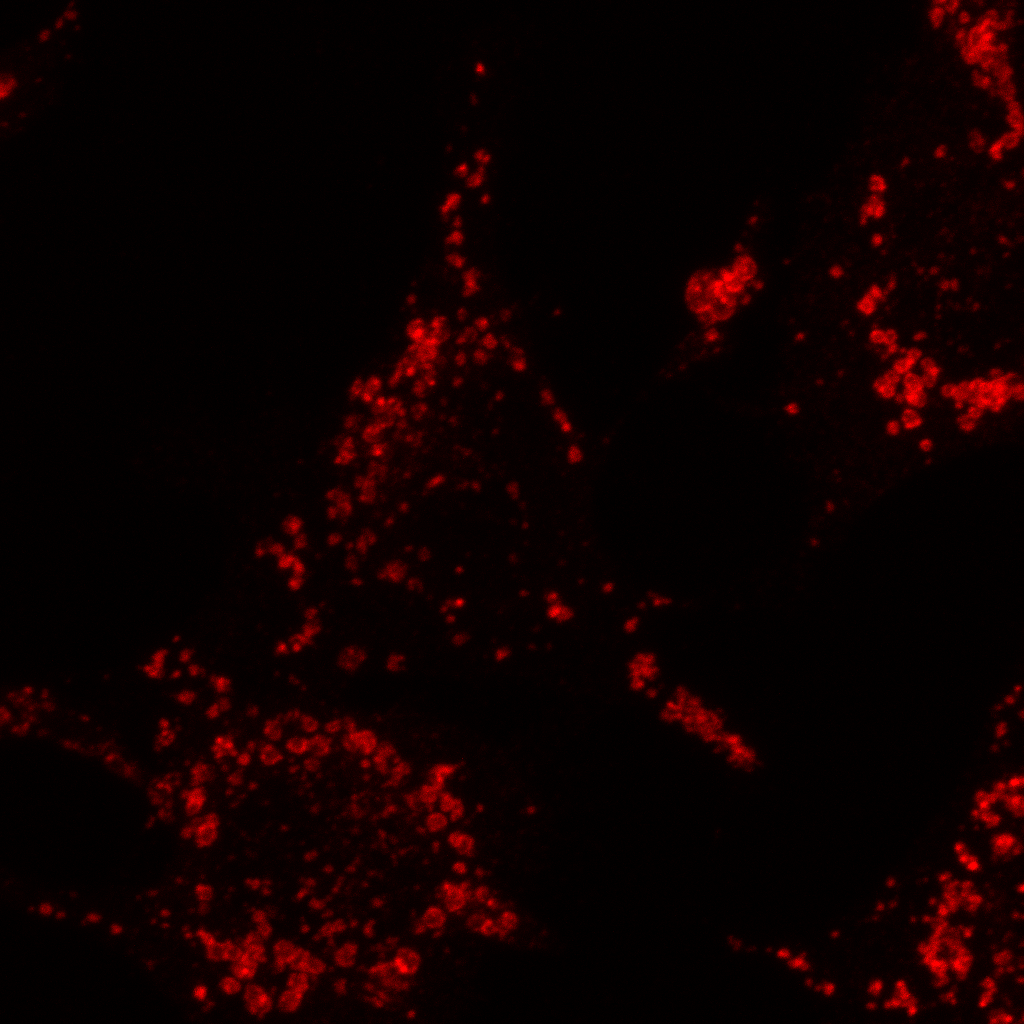

Supplement: Supplementary file 8 — Source data Fig. 4 [file 44319_2026_751_MOESM8_ESM.zip › Raw_data_Figure 4/Figure 4E/Images Figure 4E/GqKO CKSR mRFP LAMP1.tif]

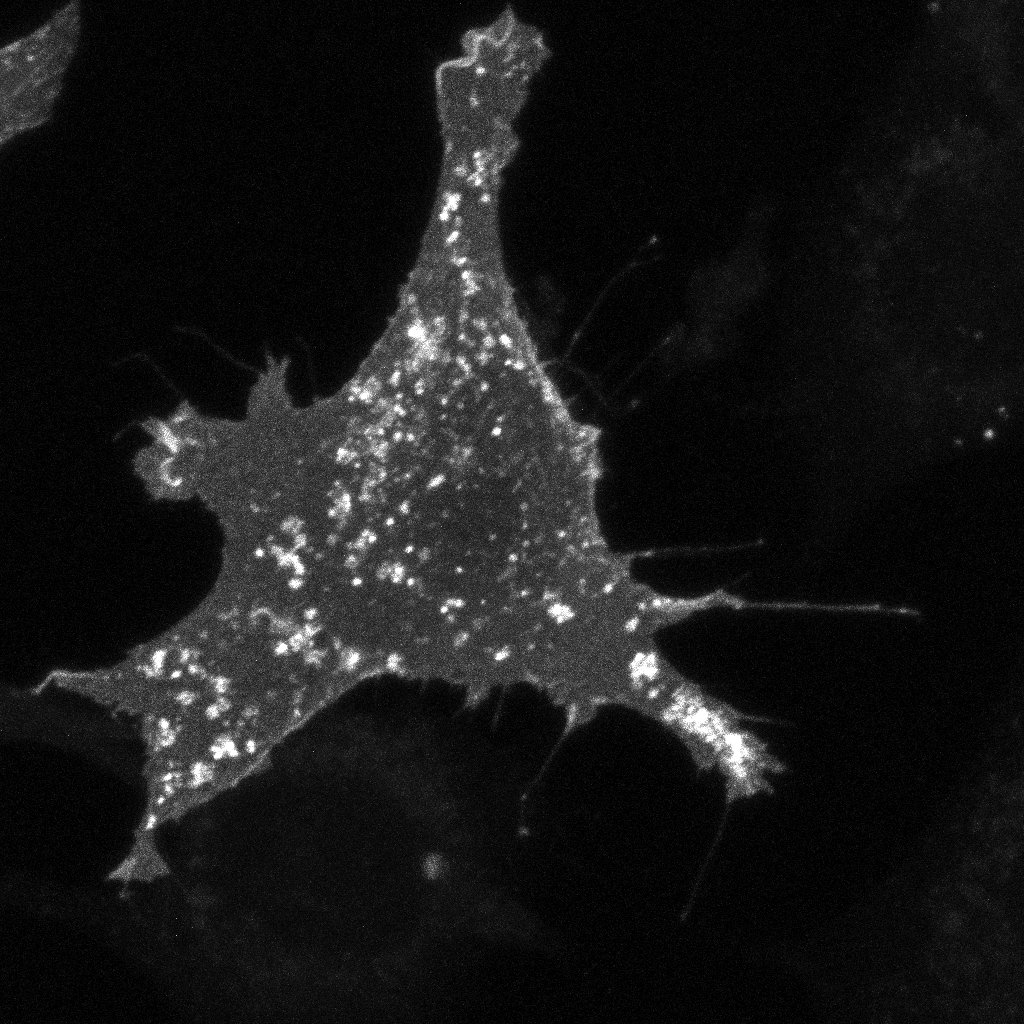

Supplement: Supplementary file 8 — Source data Fig. 4 [file 44319_2026_751_MOESM8_ESM.zip › Raw_data_Figure 4/Figure 4E/Images Figure 4E/GqKO CKSR mRFP.jpg]

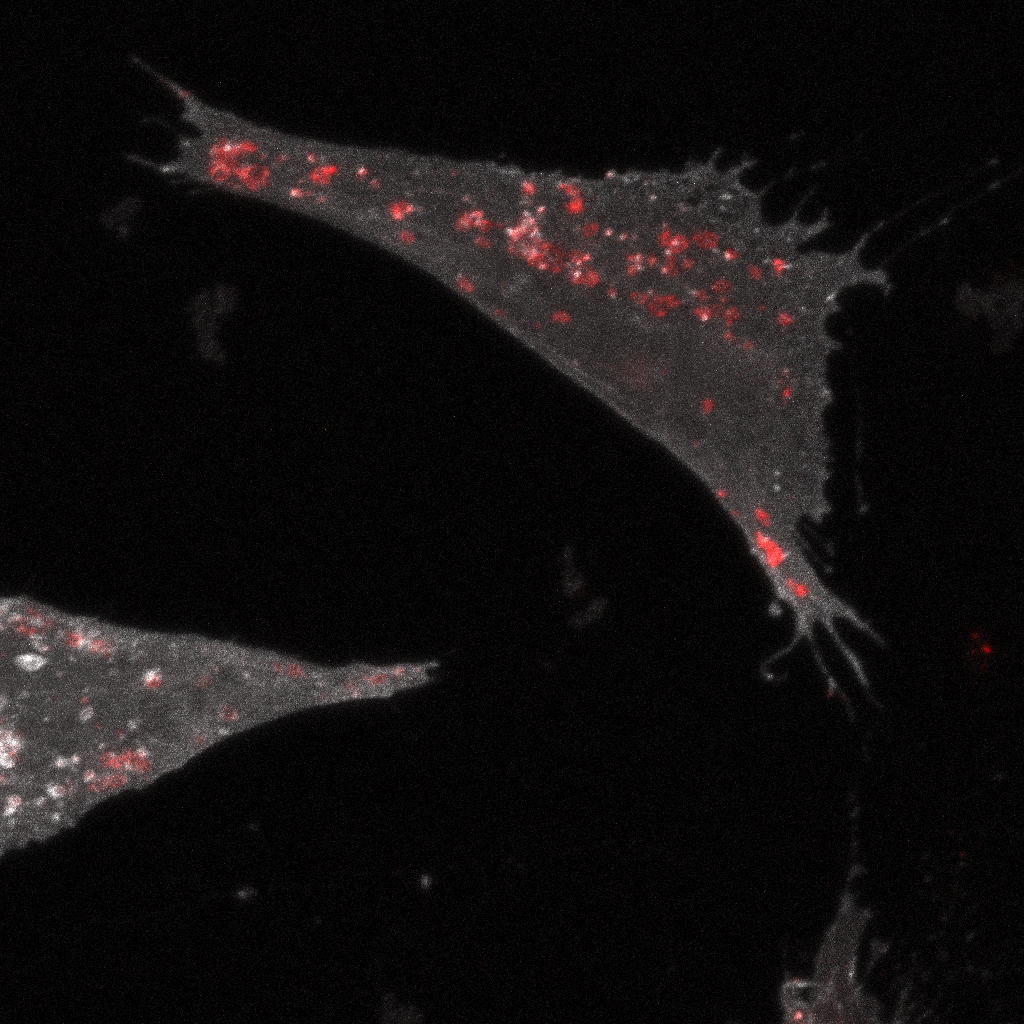

Supplement: Supplementary file 8 — Source data Fig. 4 [file 44319_2026_751_MOESM8_ESM.zip › Raw_data_Figure 4/Figure 4E/Images Figure 4E/WT CKSR mRFP LAMP1 red-gray.tif]

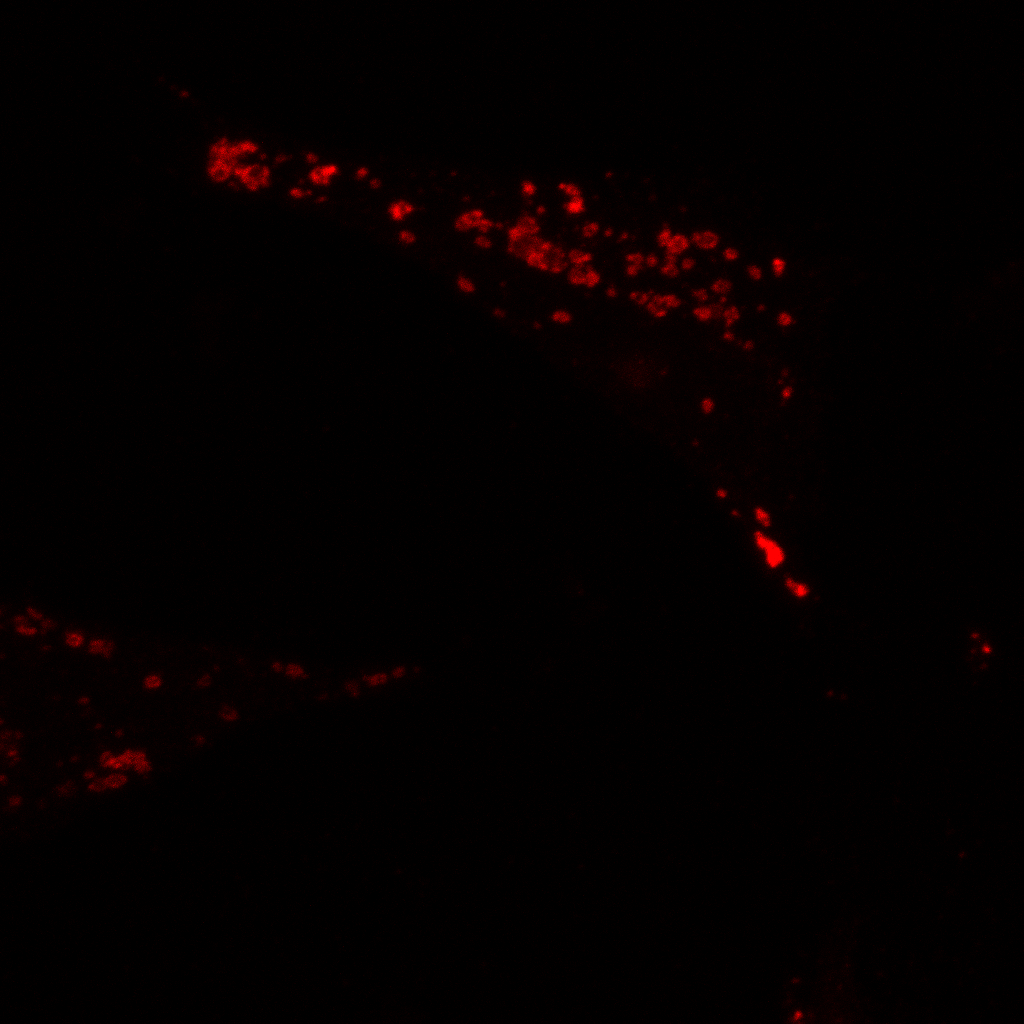

Supplement: Supplementary file 8 — Source data Fig. 4 [file 44319_2026_751_MOESM8_ESM.zip › Raw_data_Figure 4/Figure 4E/Images Figure 4E/WT CKSR mRFP LAMP1.tif]

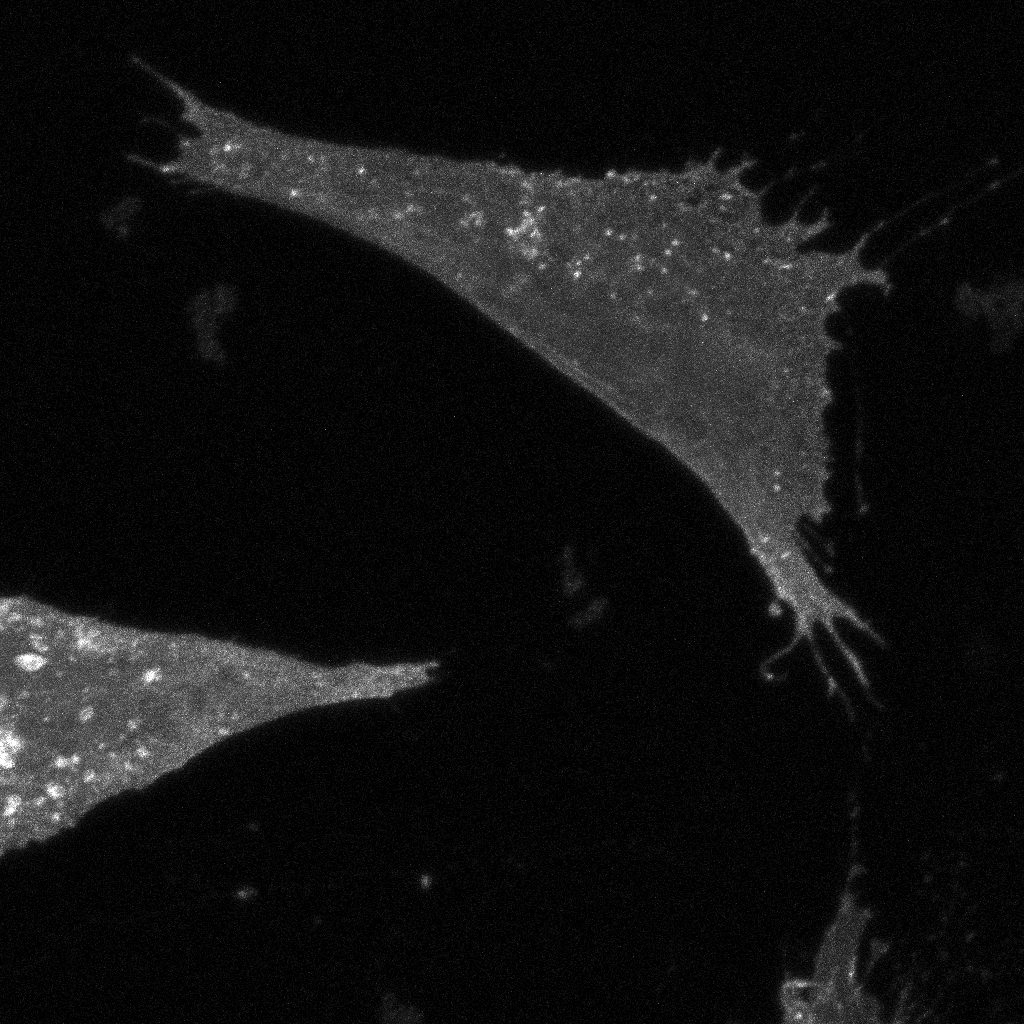

Supplement: Supplementary file 8 — Source data Fig. 4 [file 44319_2026_751_MOESM8_ESM.zip › Raw_data_Figure 4/Figure 4E/Images Figure 4E/WT CKSR mRFP.tif]

## Slide 1
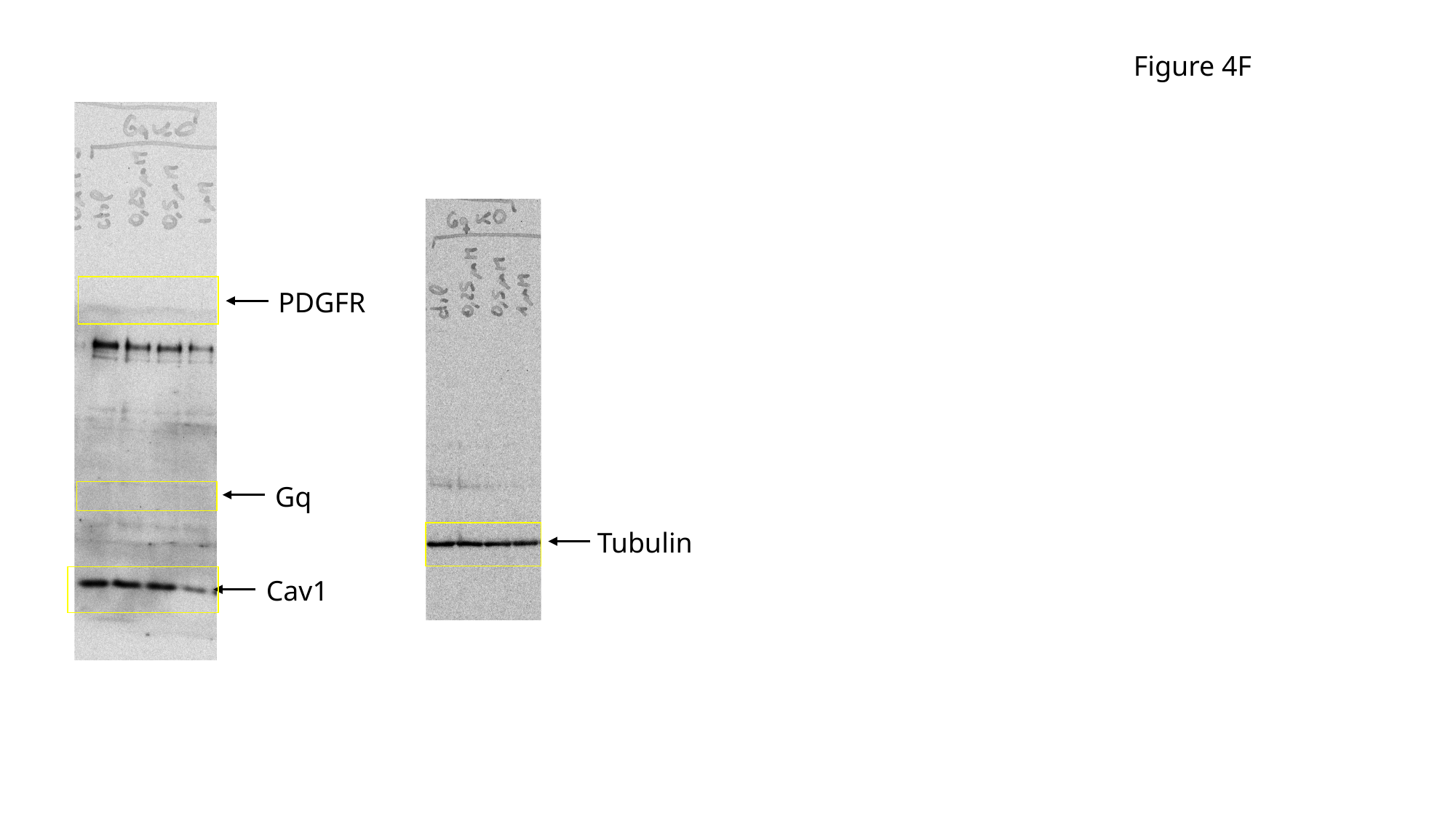

Figure 4F
PDGFR
Gq
Tubulin
Cav1

Supplement: Supplementary file 8 — Source data Fig. 4 [file 44319_2026_751_MOESM8_ESM.zip › Raw_data_Figure 4/Figure 4F/raw_blots_4F.pptx]

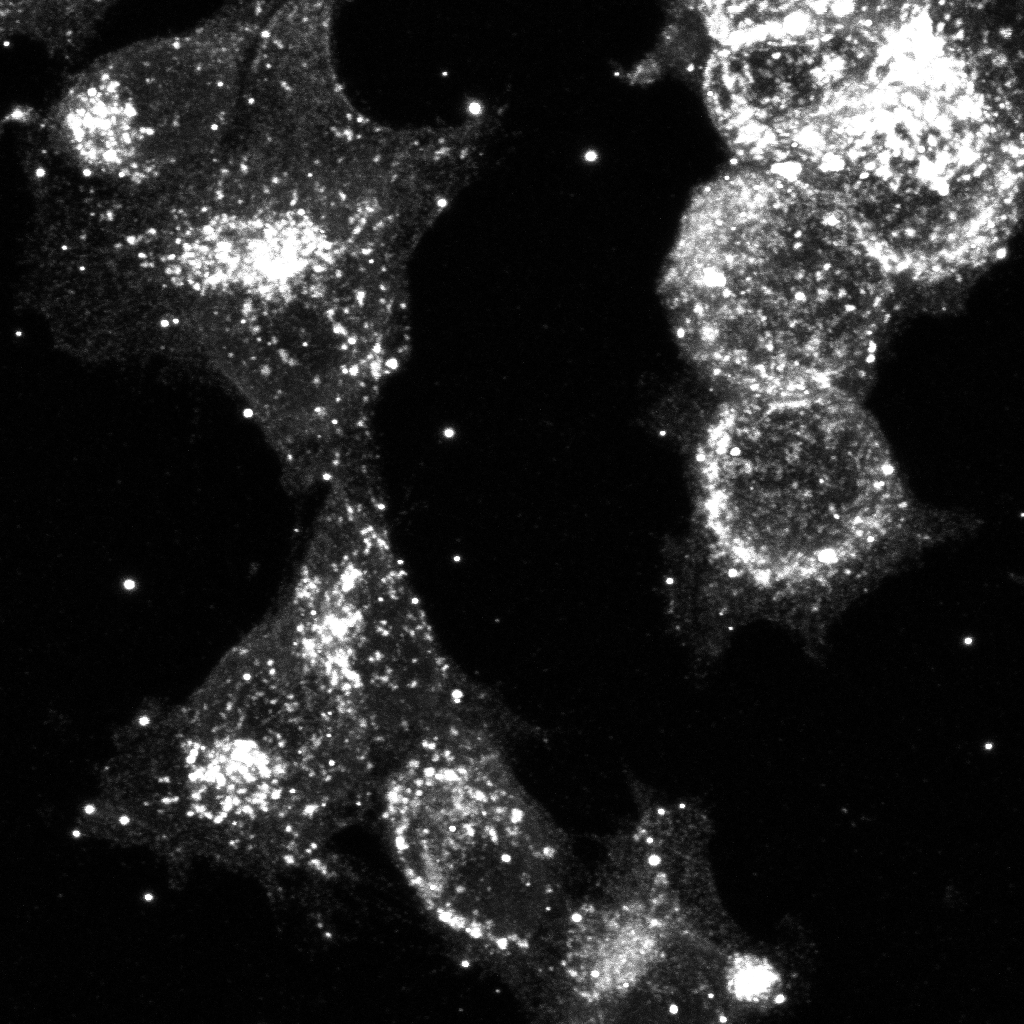

Supplement: Supplementary file 8 — Source data Fig. 4 [file 44319_2026_751_MOESM8_ESM.zip › Raw_data_Figure 4/Figure 4G/C4-GqKO control LAMP1.tif]

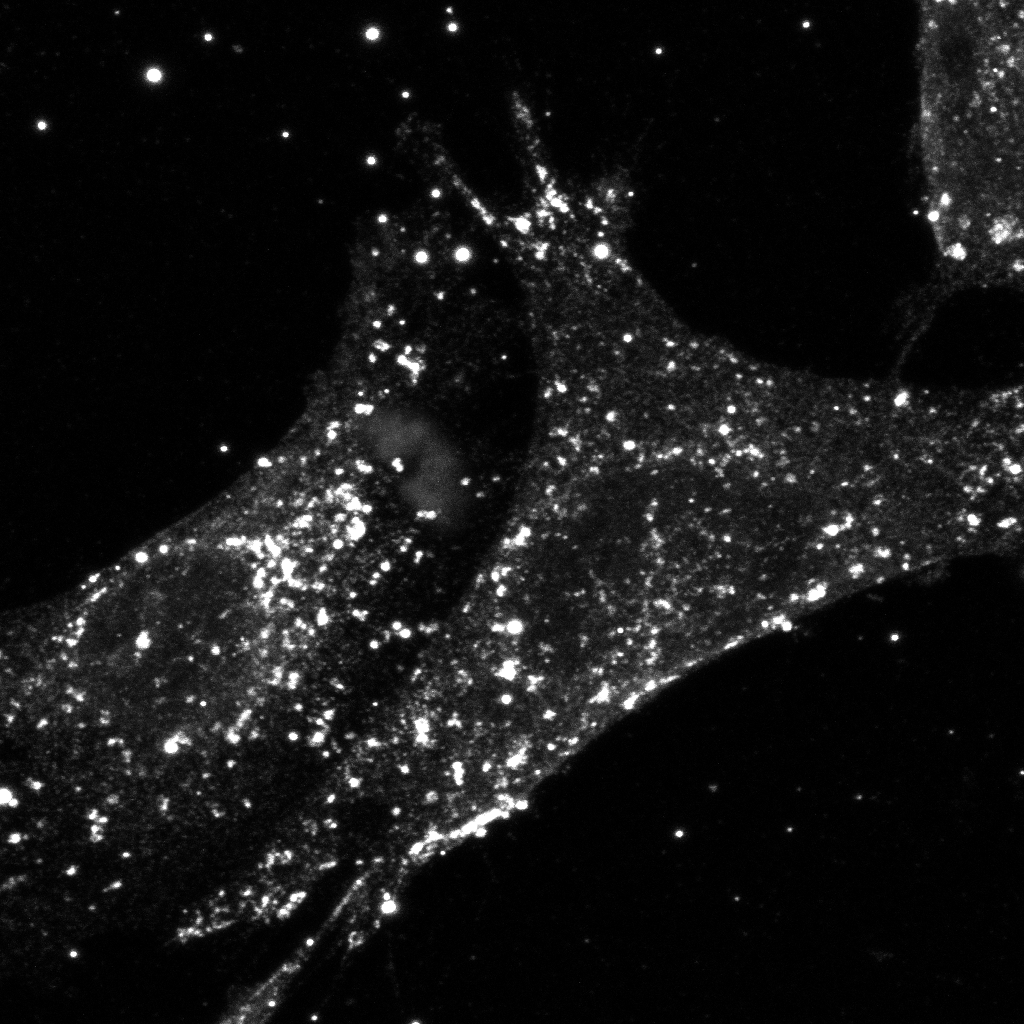

Supplement: Supplementary file 8 — Source data Fig. 4 [file 44319_2026_751_MOESM8_ESM.zip › Raw_data_Figure 4/Figure 4G/C4-GqKO GW LAMP1.tif]

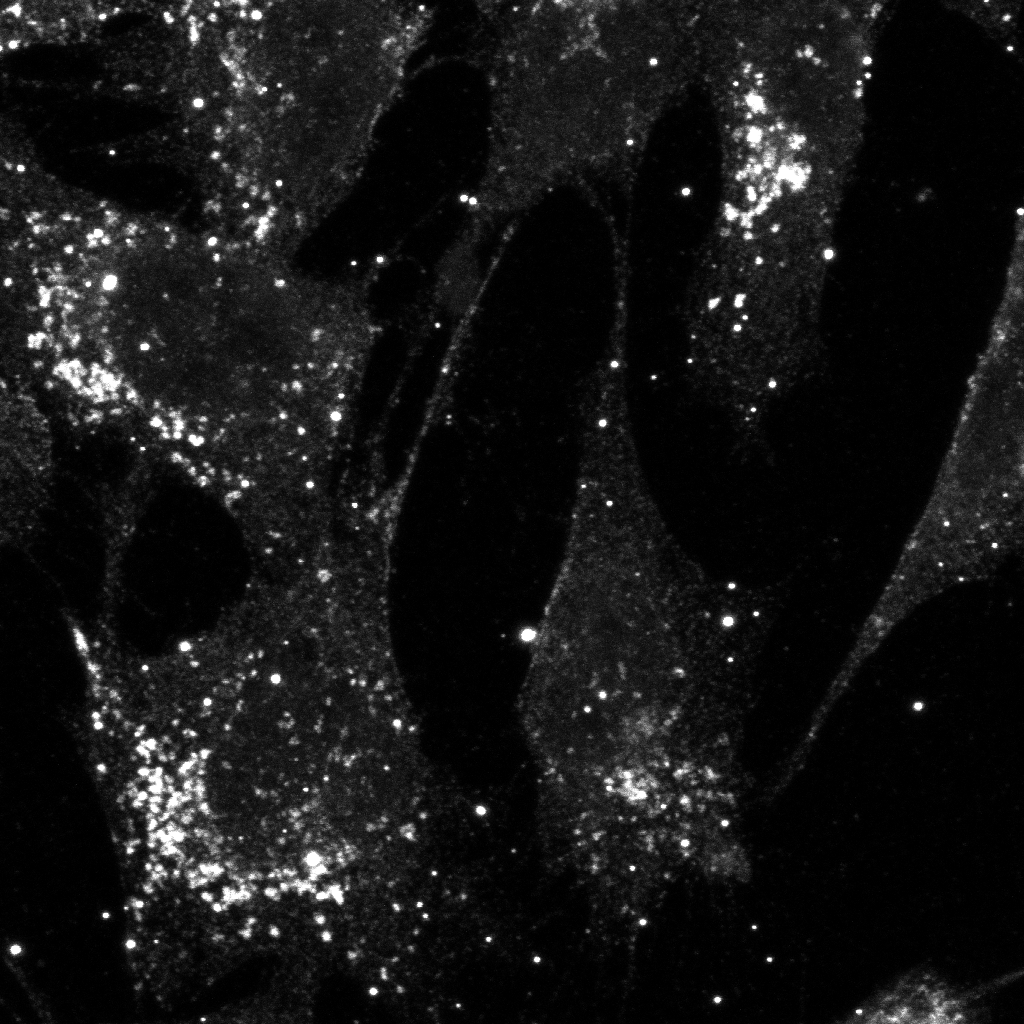

Supplement: Supplementary file 8 — Source data Fig. 4 [file 44319_2026_751_MOESM8_ESM.zip › Raw_data_Figure 4/Figure 4G/C4-WT control LAMP1.tif]

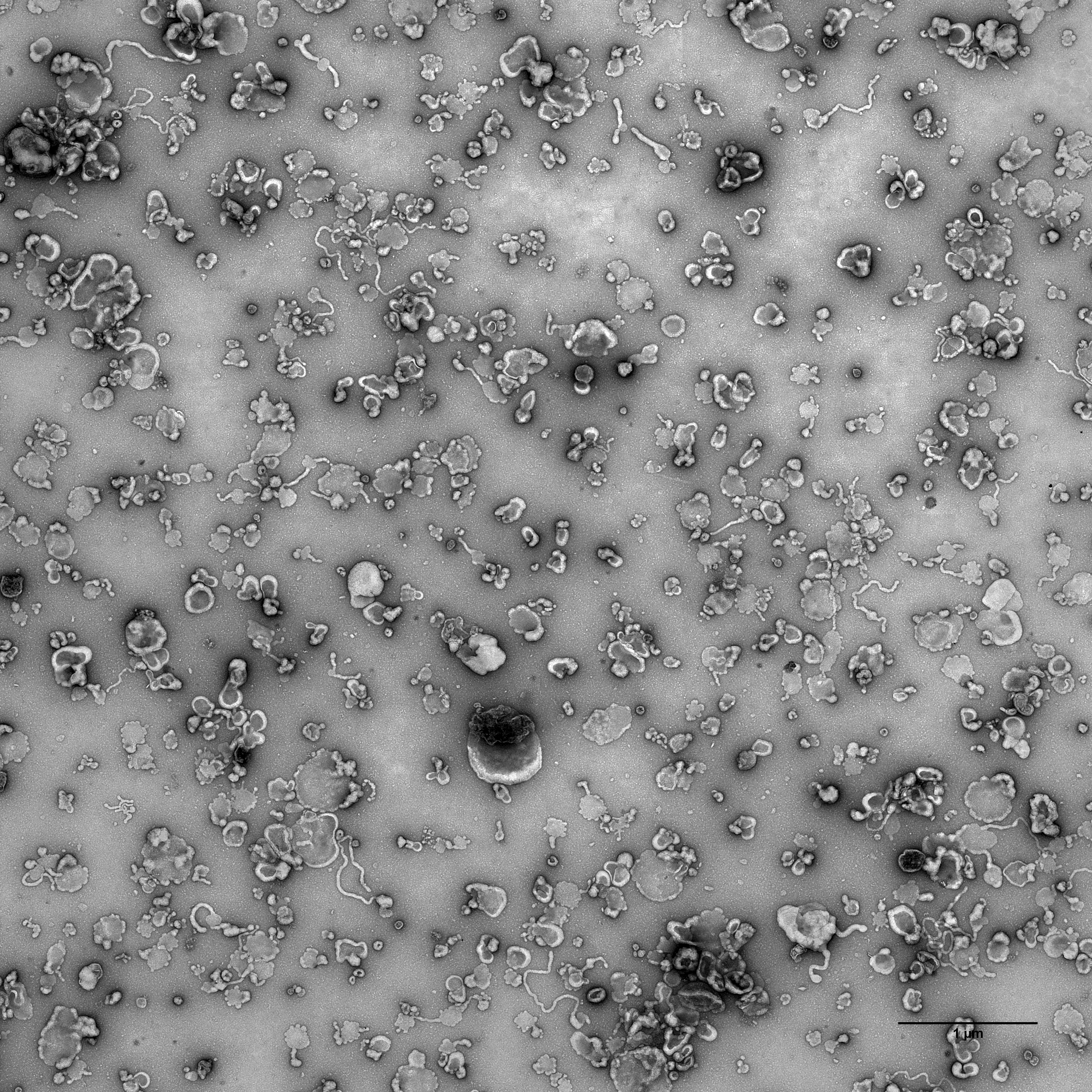

Supplement: Supplementary file 9 — Source data Fig. 5 [file 44319_2026_751_MOESM9_ESM.zip › Raw_data_Figure 5/Figure 5A/Exos EM/GqKO/6000X_0003.tif]

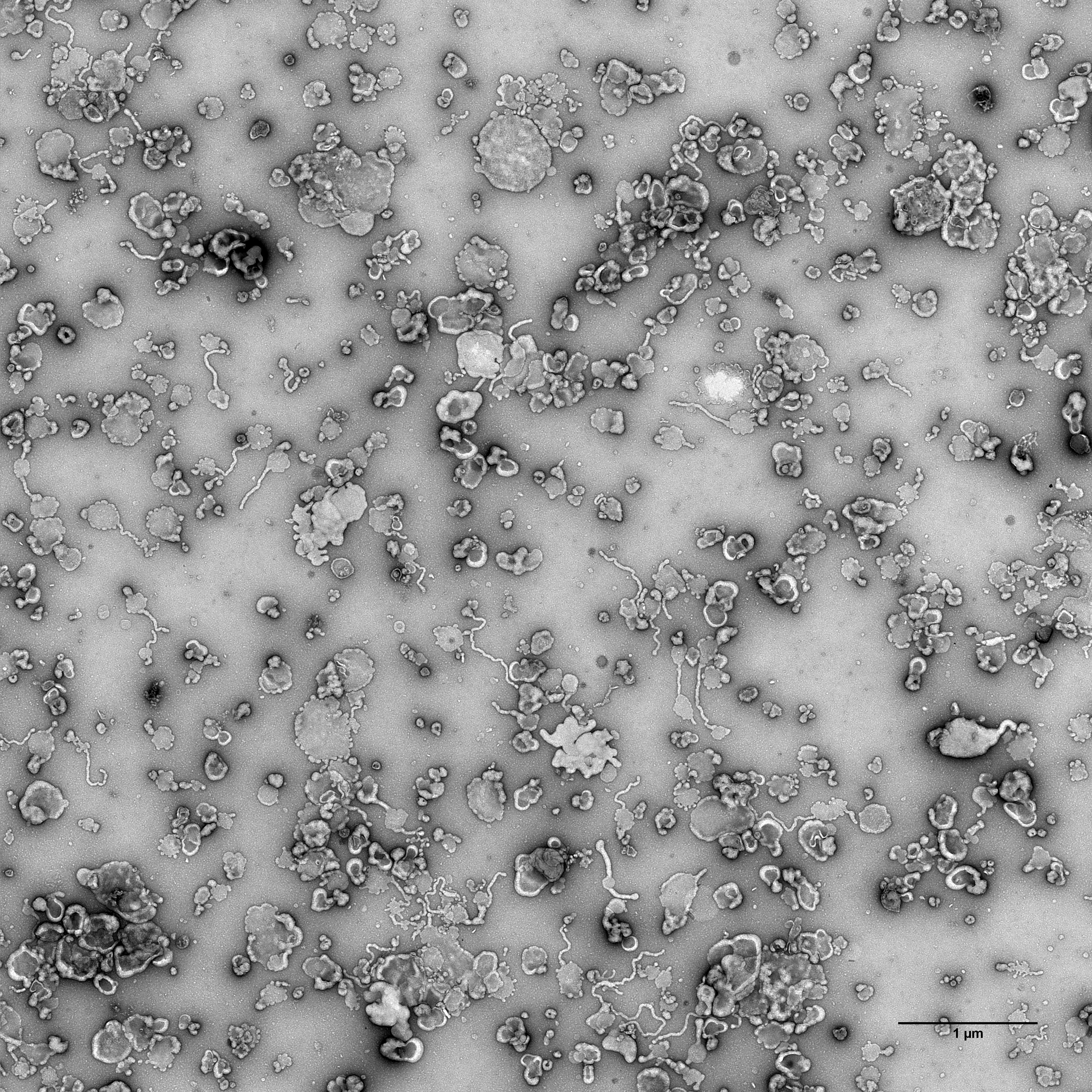

Supplement: Supplementary file 9 — Source data Fig. 5 [file 44319_2026_751_MOESM9_ESM.zip › Raw_data_Figure 5/Figure 5A/Exos EM/GqKO/6000X_0012.tif]

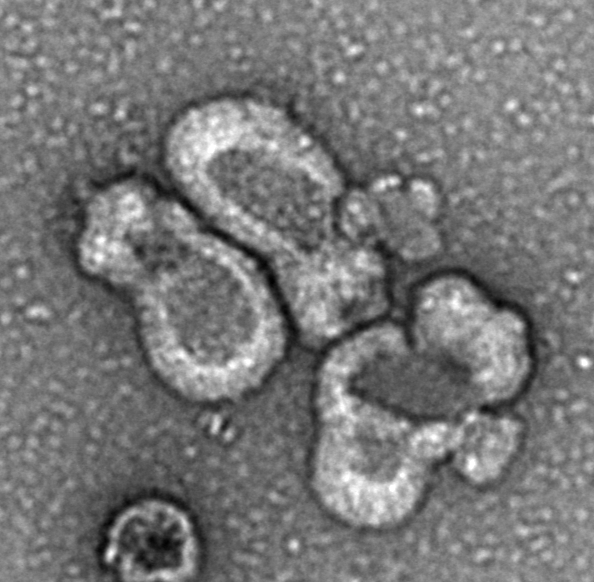

Supplement: Supplementary file 9 — Source data Fig. 5 [file 44319_2026_751_MOESM9_ESM.zip › Raw_data_Figure 5/Figure 5A/Exos EM/GqKO/GqKO cropped.tif]

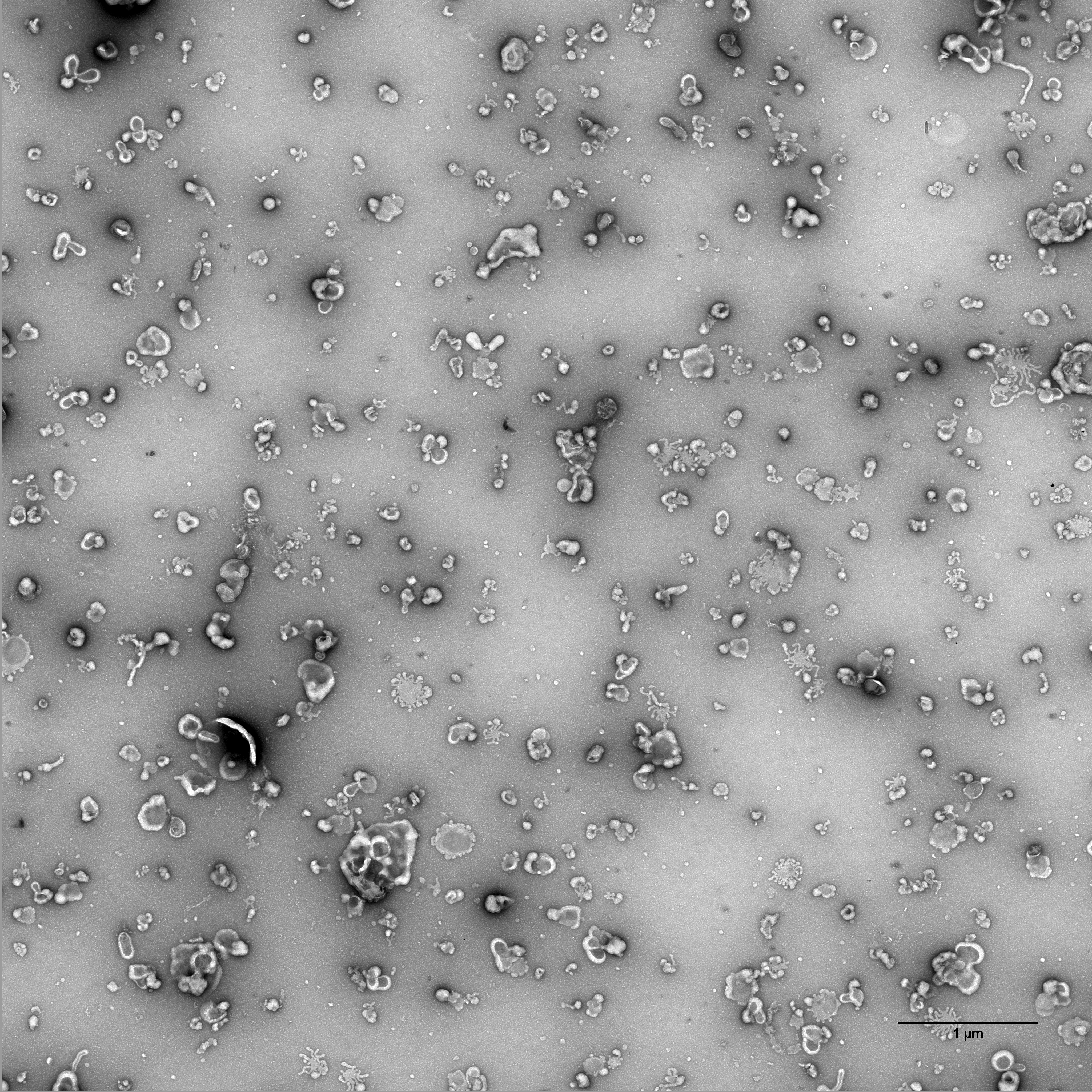

Supplement: Supplementary file 9 — Source data Fig. 5 [file 44319_2026_751_MOESM9_ESM.zip › Raw_data_Figure 5/Figure 5A/Exos EM/WT/6000X_0005.tif]

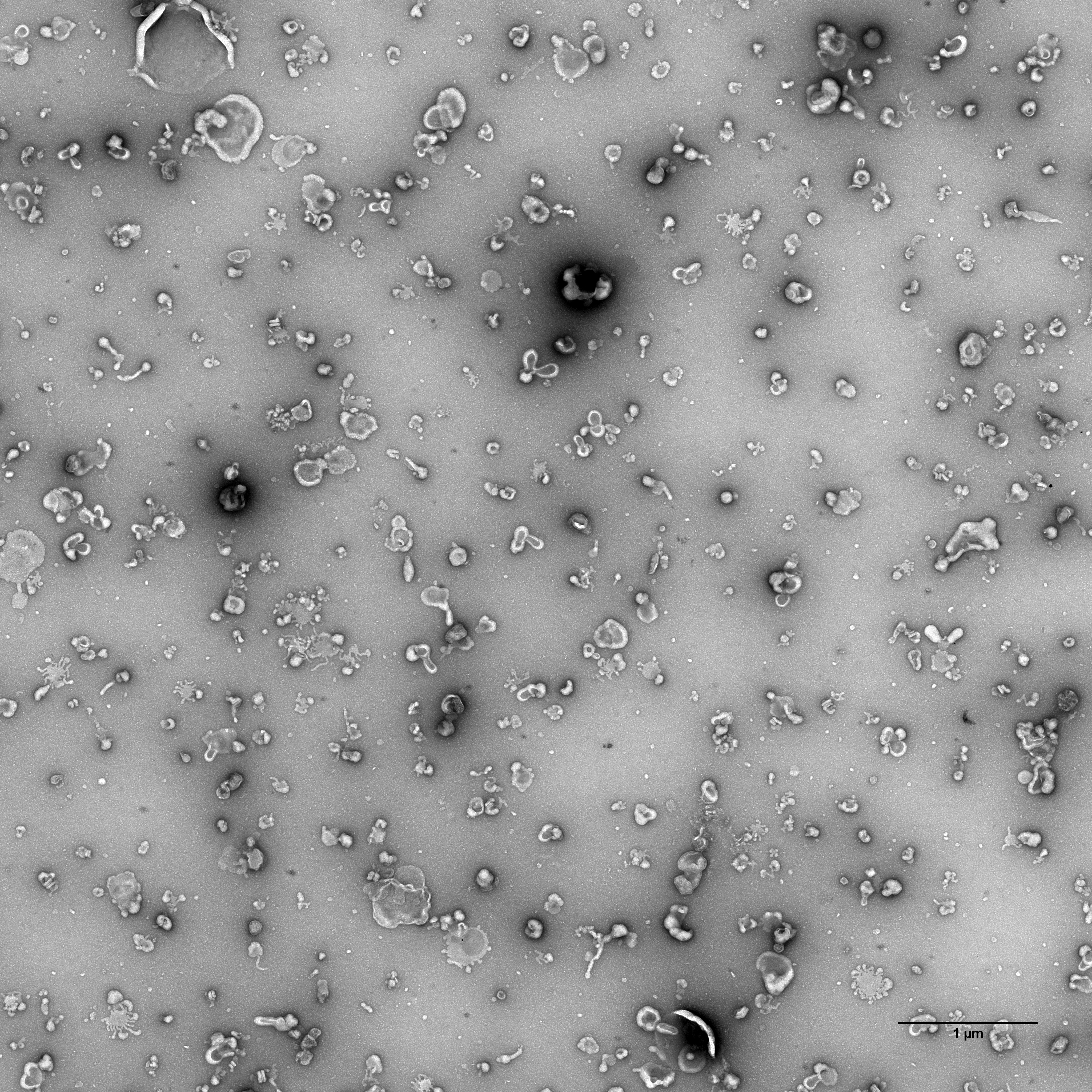

Supplement: Supplementary file 9 — Source data Fig. 5 [file 44319_2026_751_MOESM9_ESM.zip › Raw_data_Figure 5/Figure 5A/Exos EM/WT/6000X_0007.tif]

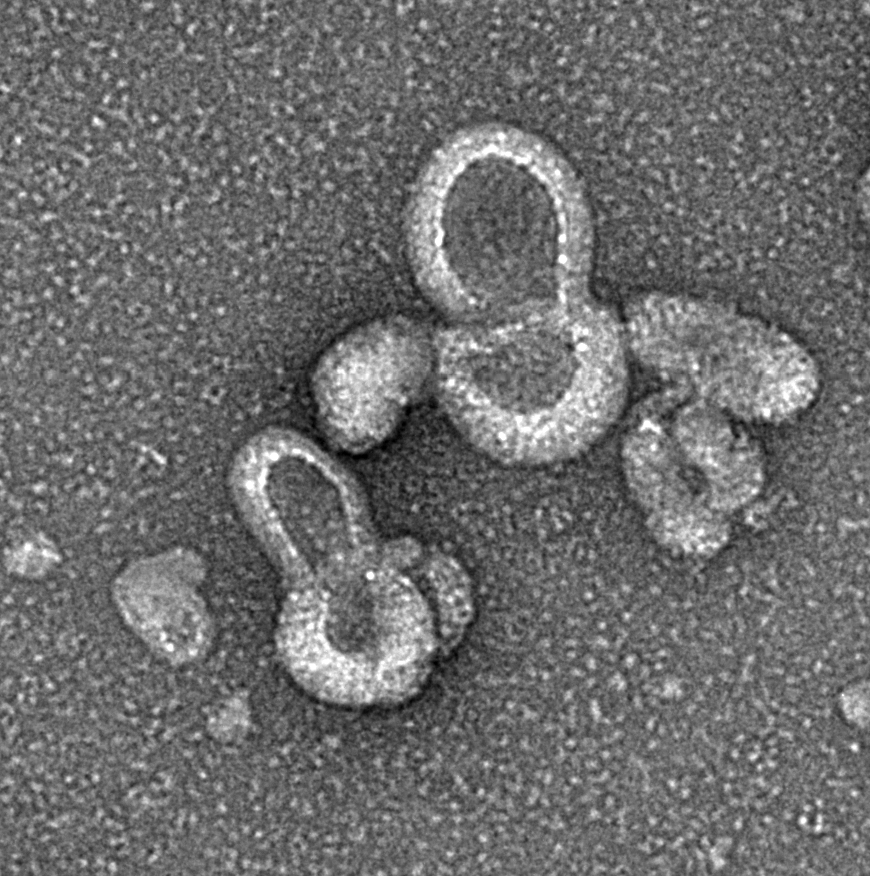

Supplement: Supplementary file 9 — Source data Fig. 5 [file 44319_2026_751_MOESM9_ESM.zip › Raw_data_Figure 5/Figure 5A/Exos EM/WT/WTcropped.tif]

## Slide 1
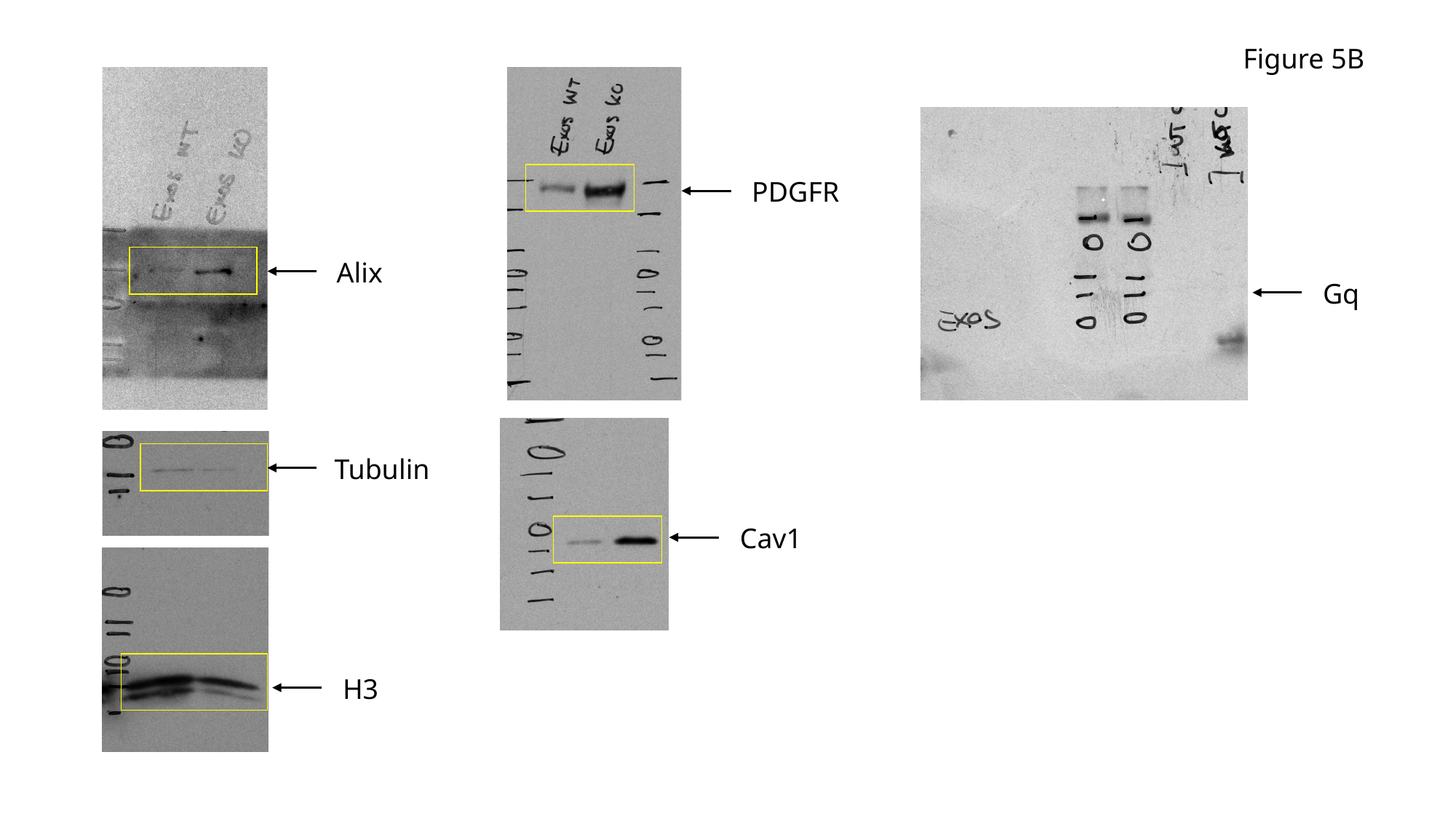

Figure 5B
PDGFR
Alix
Gq
Tubulin
Cav1
H3

Supplement: Supplementary file 9 — Source data Fig. 5 [file 44319_2026_751_MOESM9_ESM.zip › Raw_data_Figure 5/Figure 5B/raw_blots_5B.pptx]

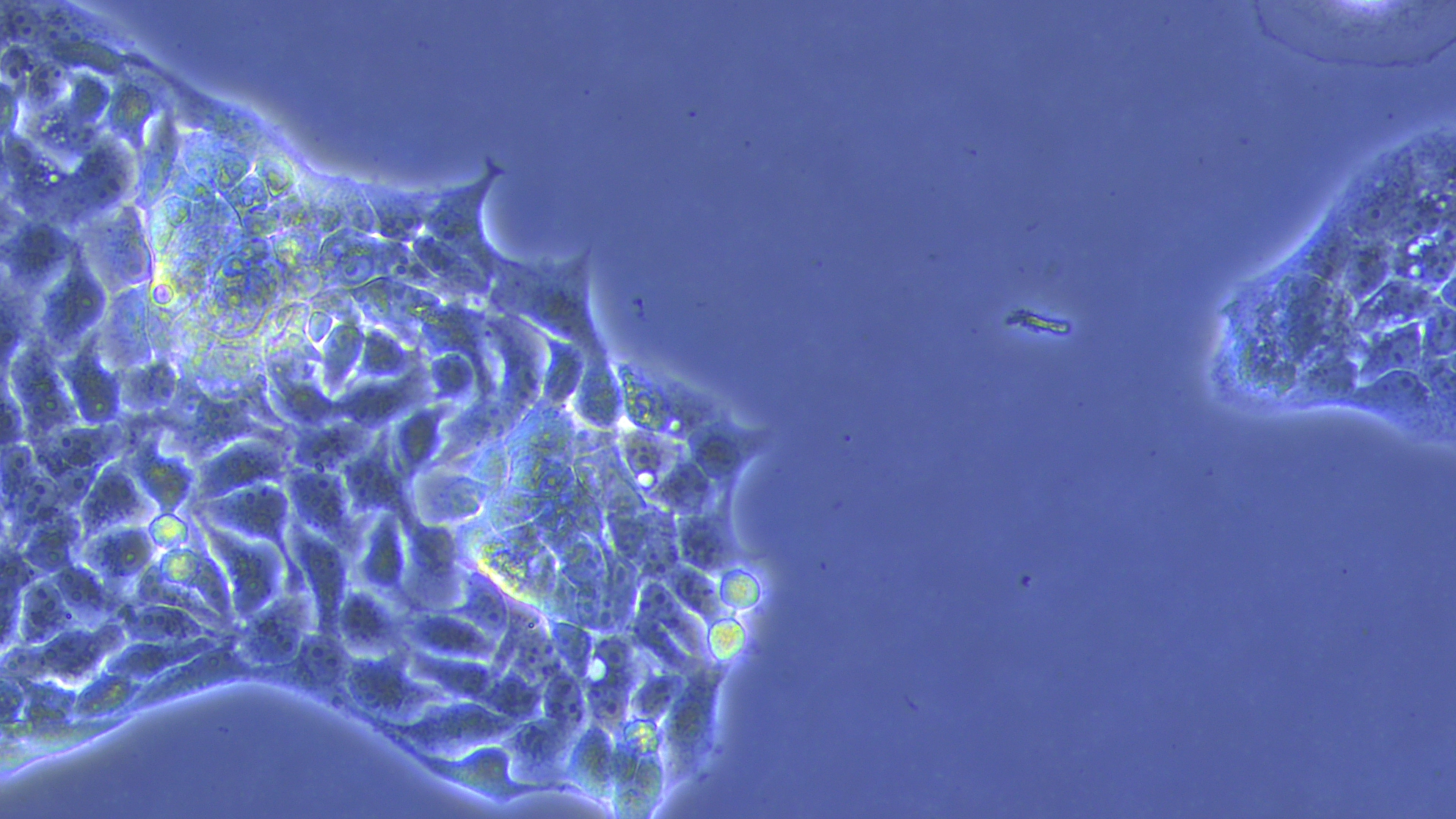

Supplement: Supplementary file 9 — Source data Fig. 5 [file 44319_2026_751_MOESM9_ESM.zip › Raw_data_Figure 5/Figure 5C/cal27+ exosko.jpg]

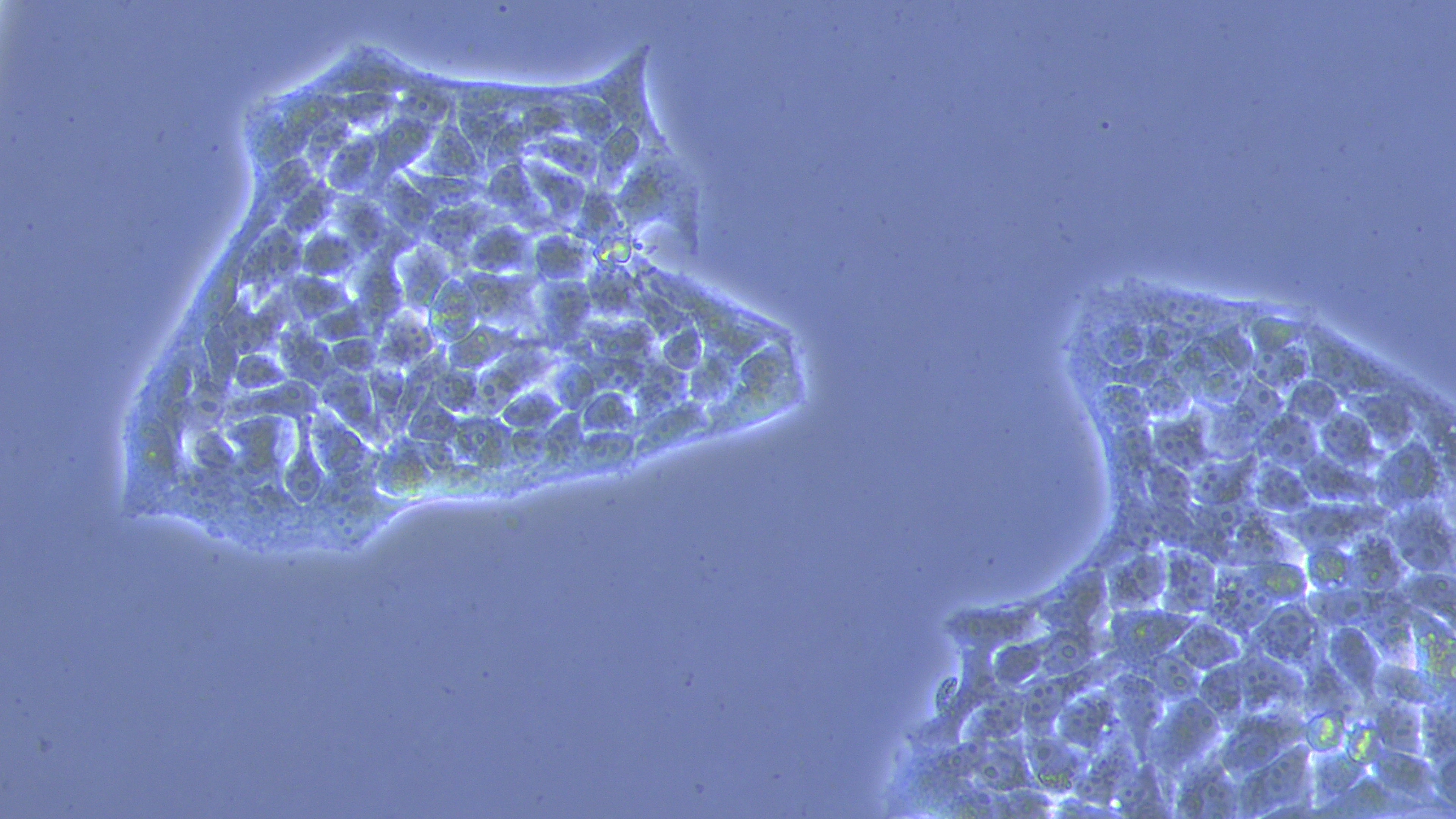

Supplement: Supplementary file 9 — Source data Fig. 5 [file 44319_2026_751_MOESM9_ESM.zip › Raw_data_Figure 5/Figure 5C/cal27+exoswt.jpg]

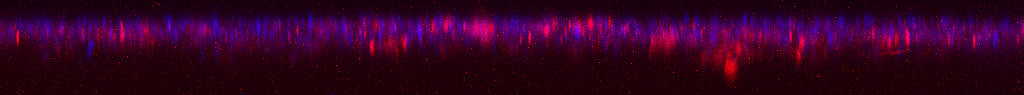

Supplement: Supplementary file 9 — Source data Fig. 5 [file 44319_2026_751_MOESM9_ESM.zip › Raw_data_Figure 5/Figure 5D/Cal27 dil bottom GqKO fibros in matrigel Z max proyect XY.tif]

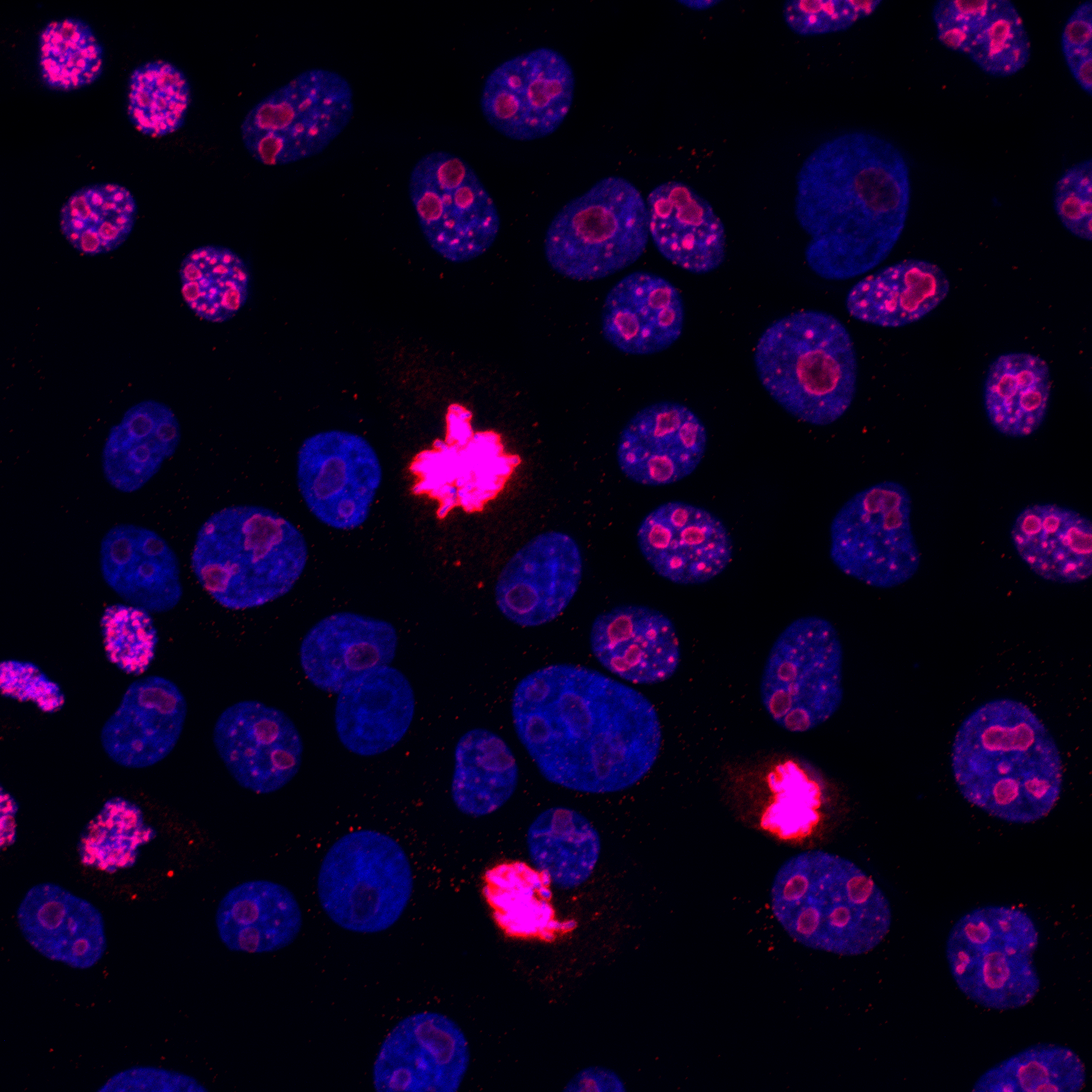

Supplement: Supplementary file 9 — Source data Fig. 5 [file 44319_2026_751_MOESM9_ESM.zip › Raw_data_Figure 5/Figure 5F/C1+C3-MAX_Cal27 + Exosomas MEFs Gq KO DAPI Ki67 555.tif]

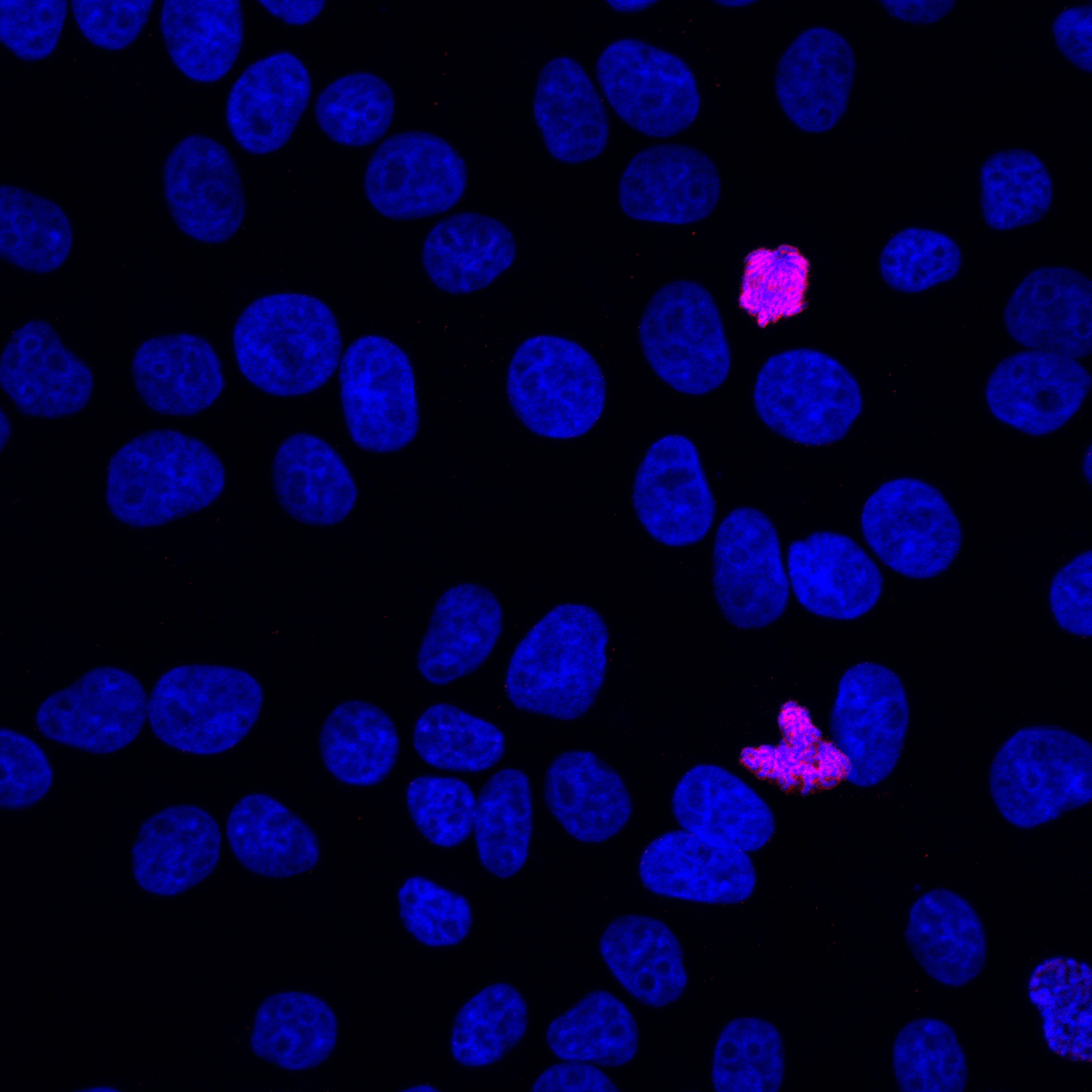

Supplement: Supplementary file 9 — Source data Fig. 5 [file 44319_2026_751_MOESM9_ESM.zip › Raw_data_Figure 5/Figure 5F/C1+C3-MAX_Cal27 + Exosomas MEFs WT DAPI Ki67 555.tif]

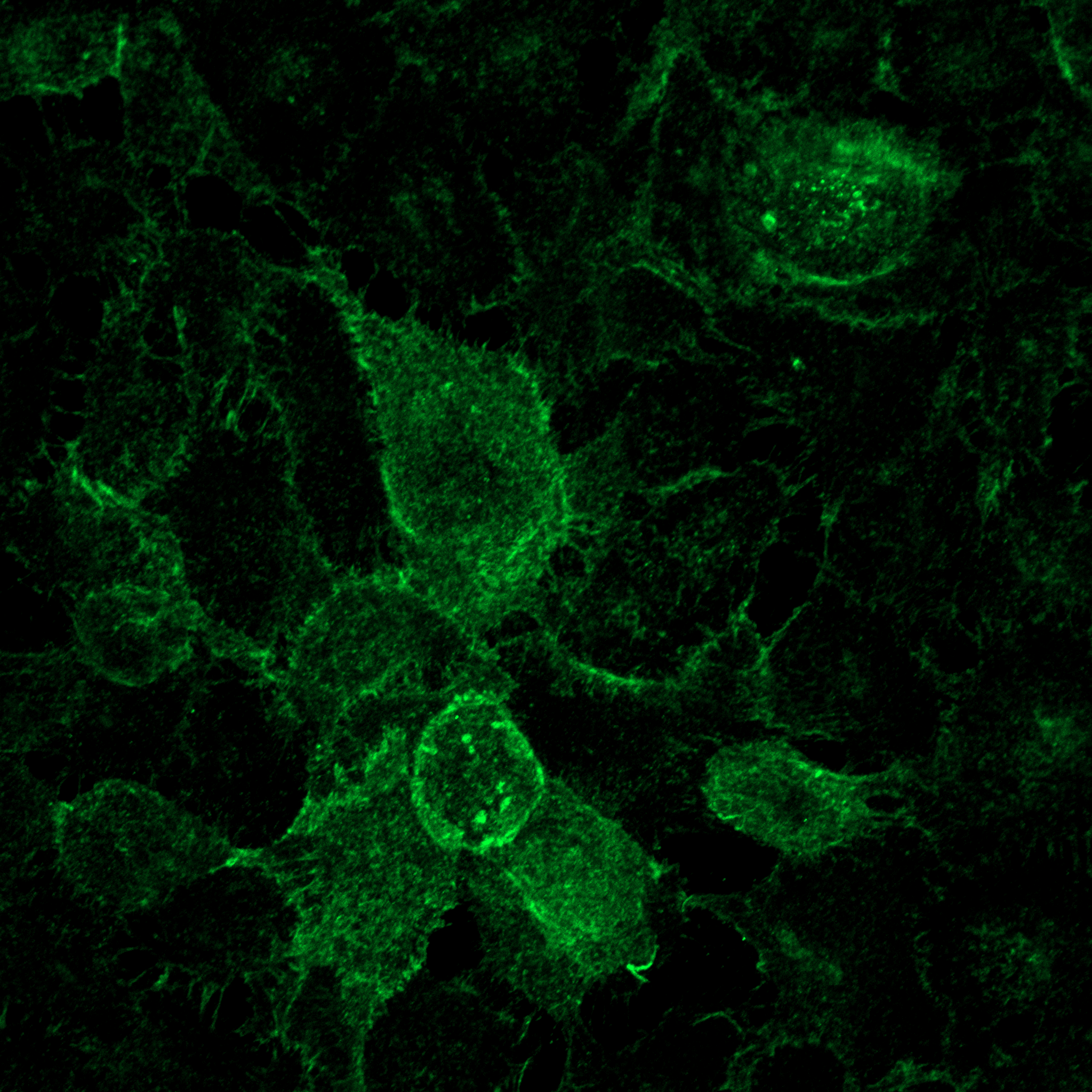

Supplement: Supplementary file 9 — Source data Fig. 5 [file 44319_2026_751_MOESM9_ESM.zip › Raw_data_Figure 5/Figure 5F/C2-MAX_Cal27 + Exosomas MEFs Gq KO ECadh 488.tif]

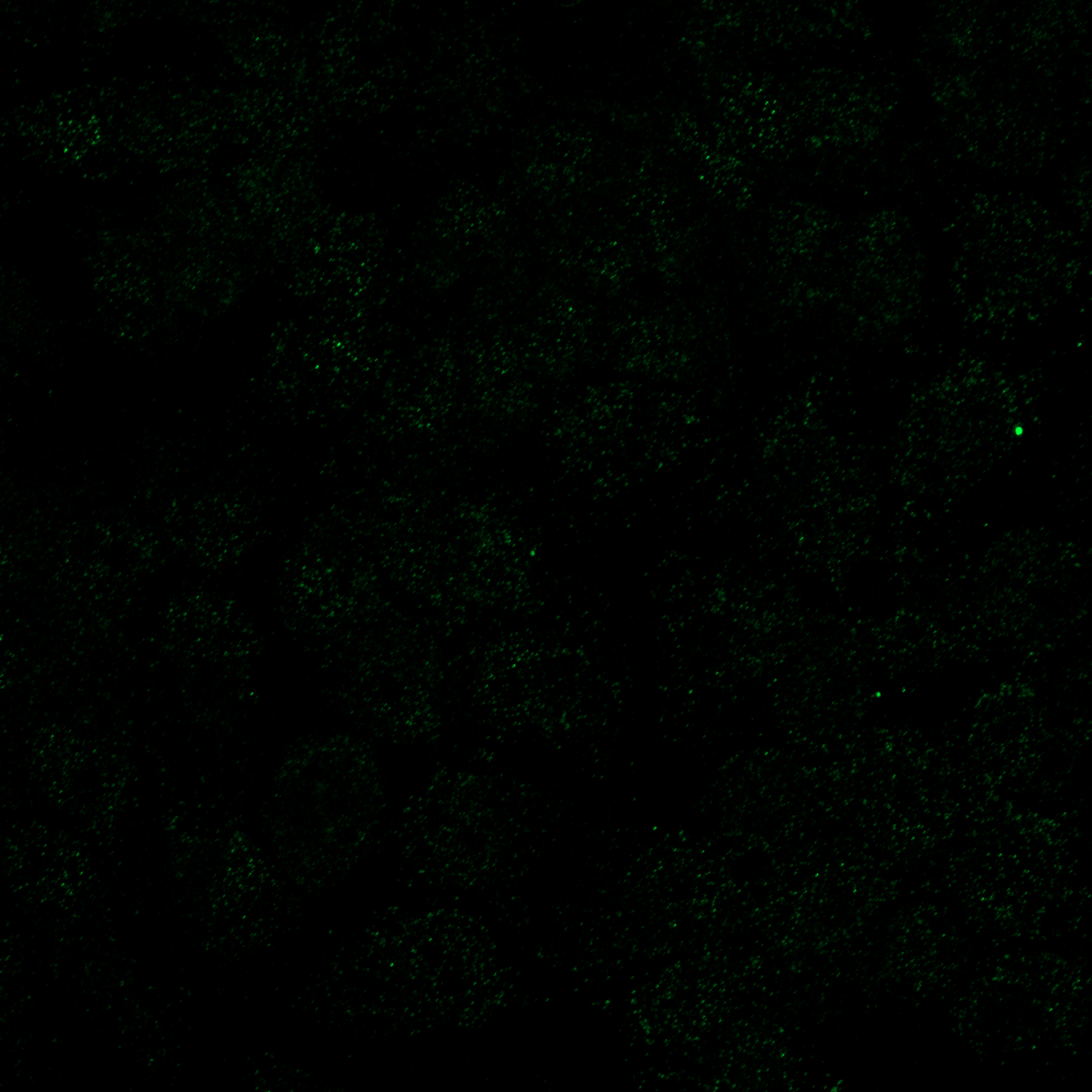

Supplement: Supplementary file 9 — Source data Fig. 5 [file 44319_2026_751_MOESM9_ESM.zip › Raw_data_Figure 5/Figure 5F/C2-MAX_Cal27 + Exosomas MEFs Gq KO PDGFR 488.tif]

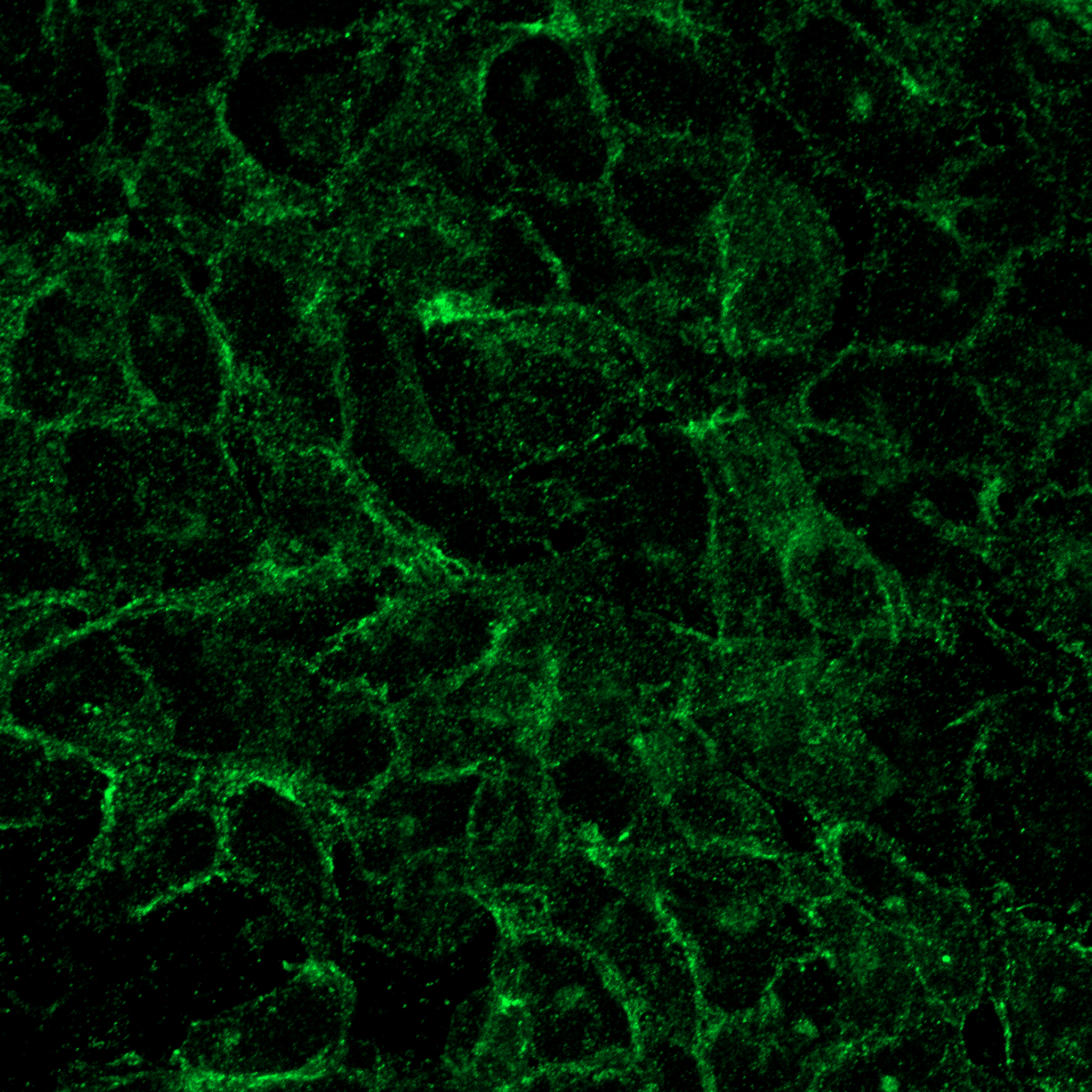

Supplement: Supplementary file 9 — Source data Fig. 5 [file 44319_2026_751_MOESM9_ESM.zip › Raw_data_Figure 5/Figure 5F/C2-MAX_Cal27 + Exosomas MEFs WT ECadh 488.tif]

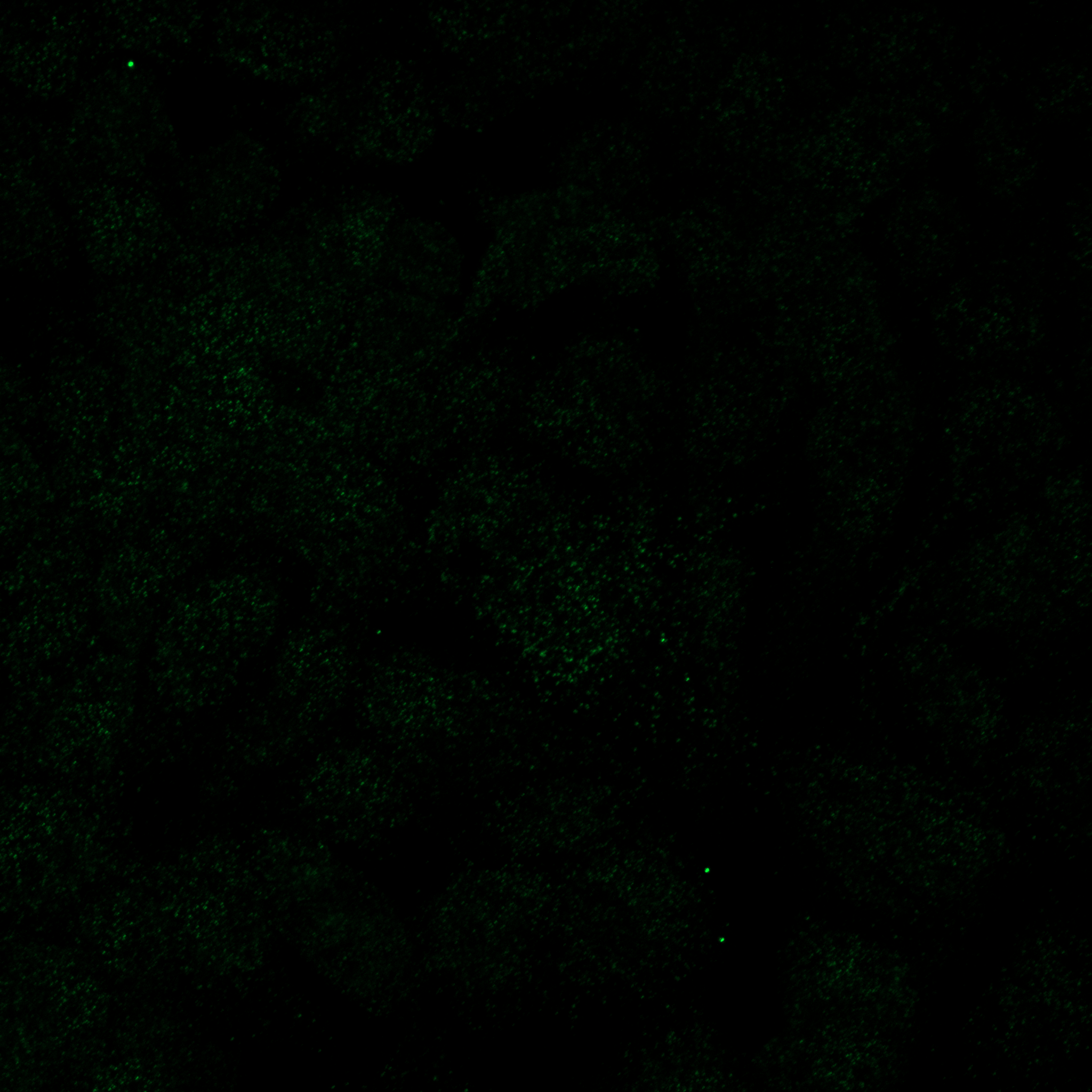

Supplement: Supplementary file 9 — Source data Fig. 5 [file 44319_2026_751_MOESM9_ESM.zip › Raw_data_Figure 5/Figure 5F/C2-MAX_Cal27 + Exosomas MEFs WT PDGFR 488.tif]

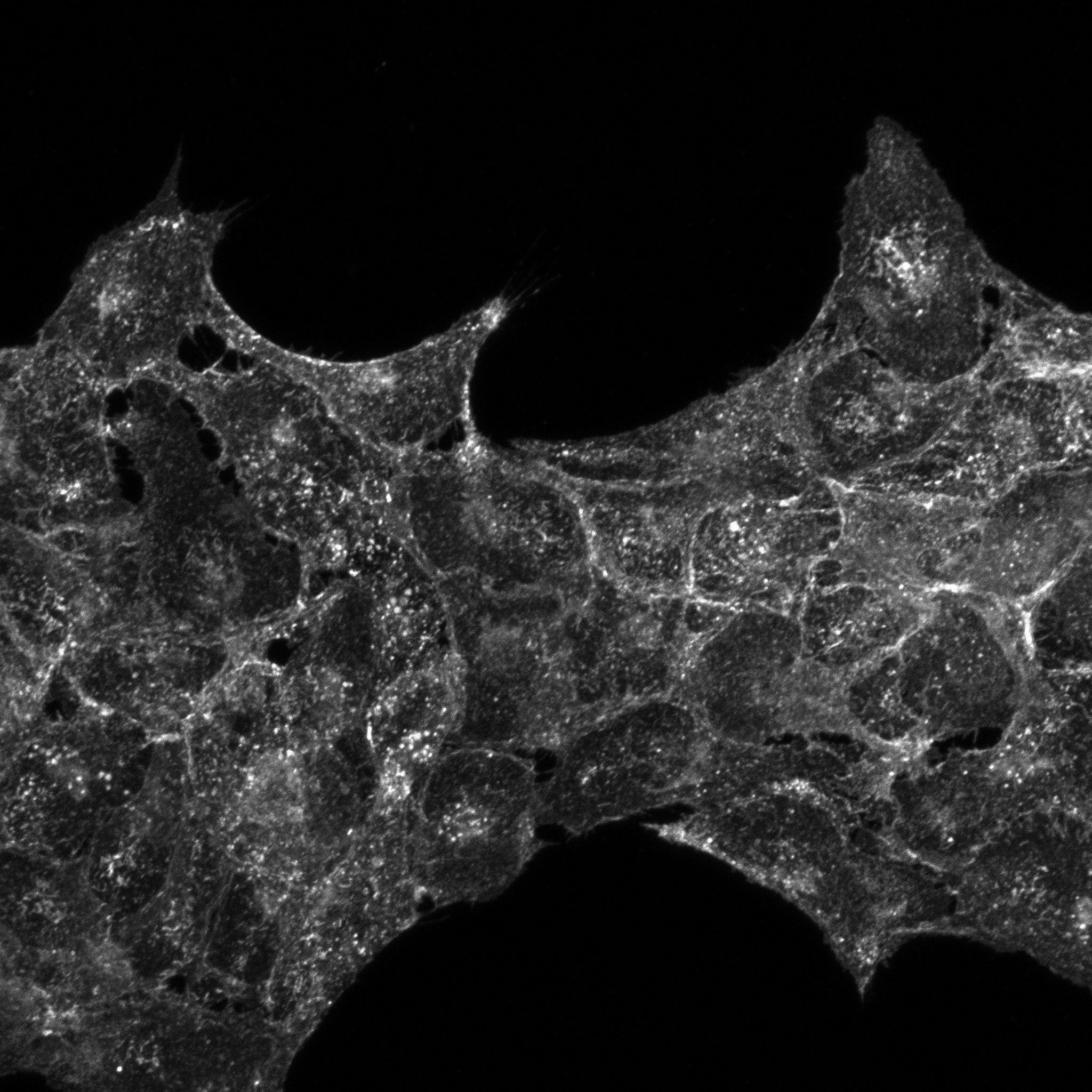

Supplement: Supplementary file 9 — Source data Fig. 5 [file 44319_2026_751_MOESM9_ESM.zip › Raw_data_Figure 5/Figure 5F/C3-MAX_Cal27 + Exosomas GqKO DAPI Vimentin 555 Cav1 647 (3) only cav1.jpg]

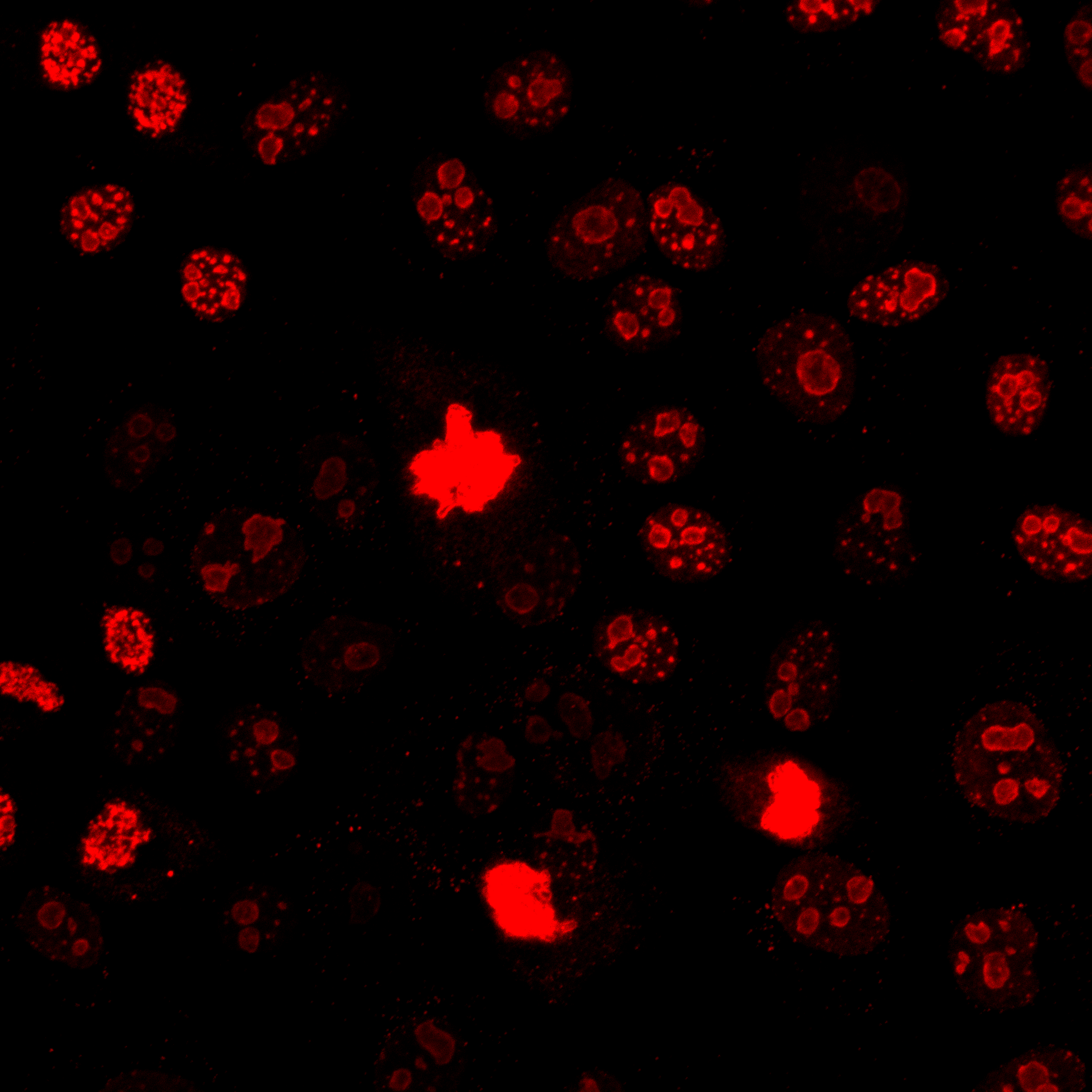

Supplement: Supplementary file 9 — Source data Fig. 5 [file 44319_2026_751_MOESM9_ESM.zip › Raw_data_Figure 5/Figure 5F/C3-MAX_Cal27 + Exosomas MEFs Gq KO Ki67 555 (1).tif]

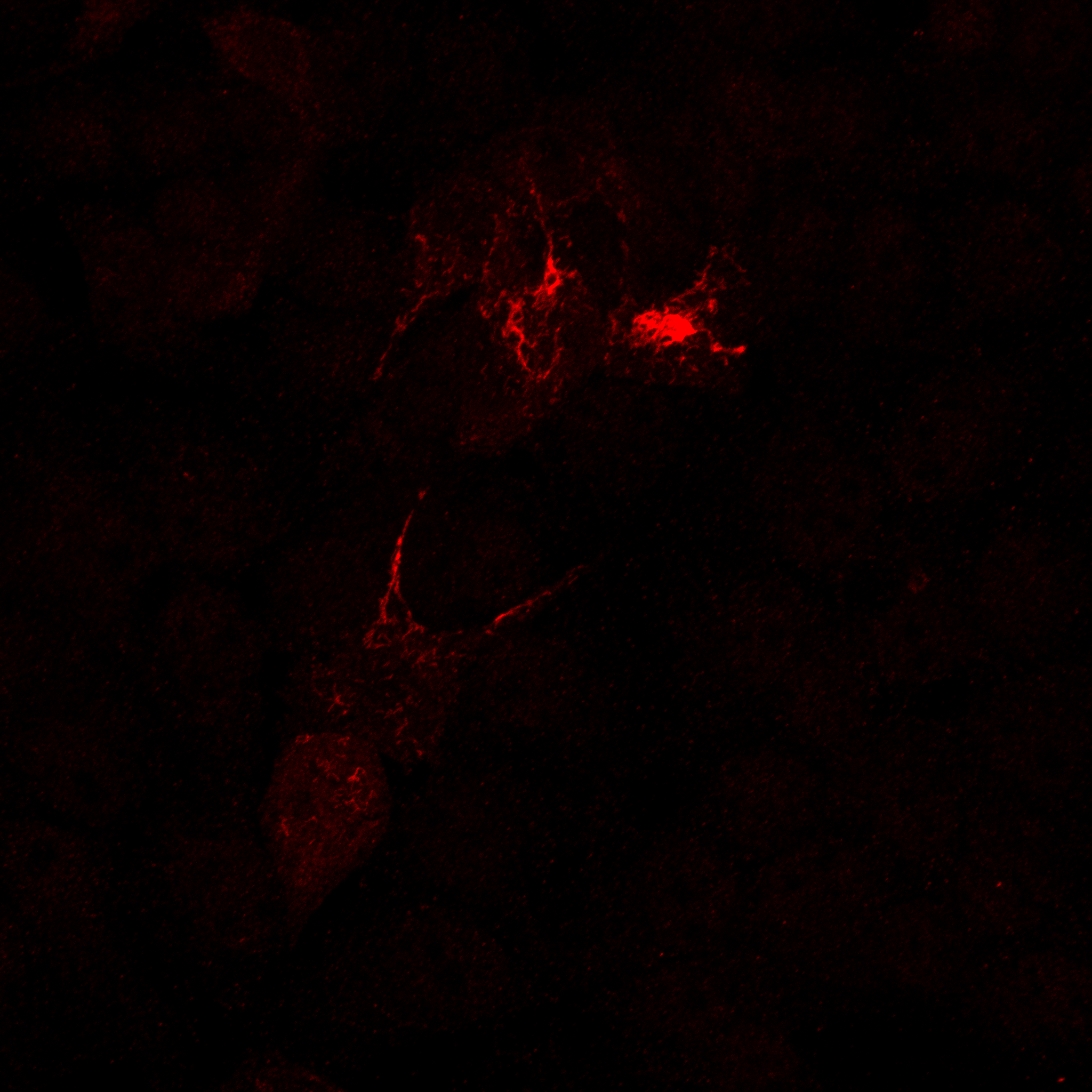

Supplement: Supplementary file 9 — Source data Fig. 5 [file 44319_2026_751_MOESM9_ESM.zip › Raw_data_Figure 5/Figure 5F/C3-MAX_Cal27 + Exosomas MEFs Gq KO Vimentin 555.tif]

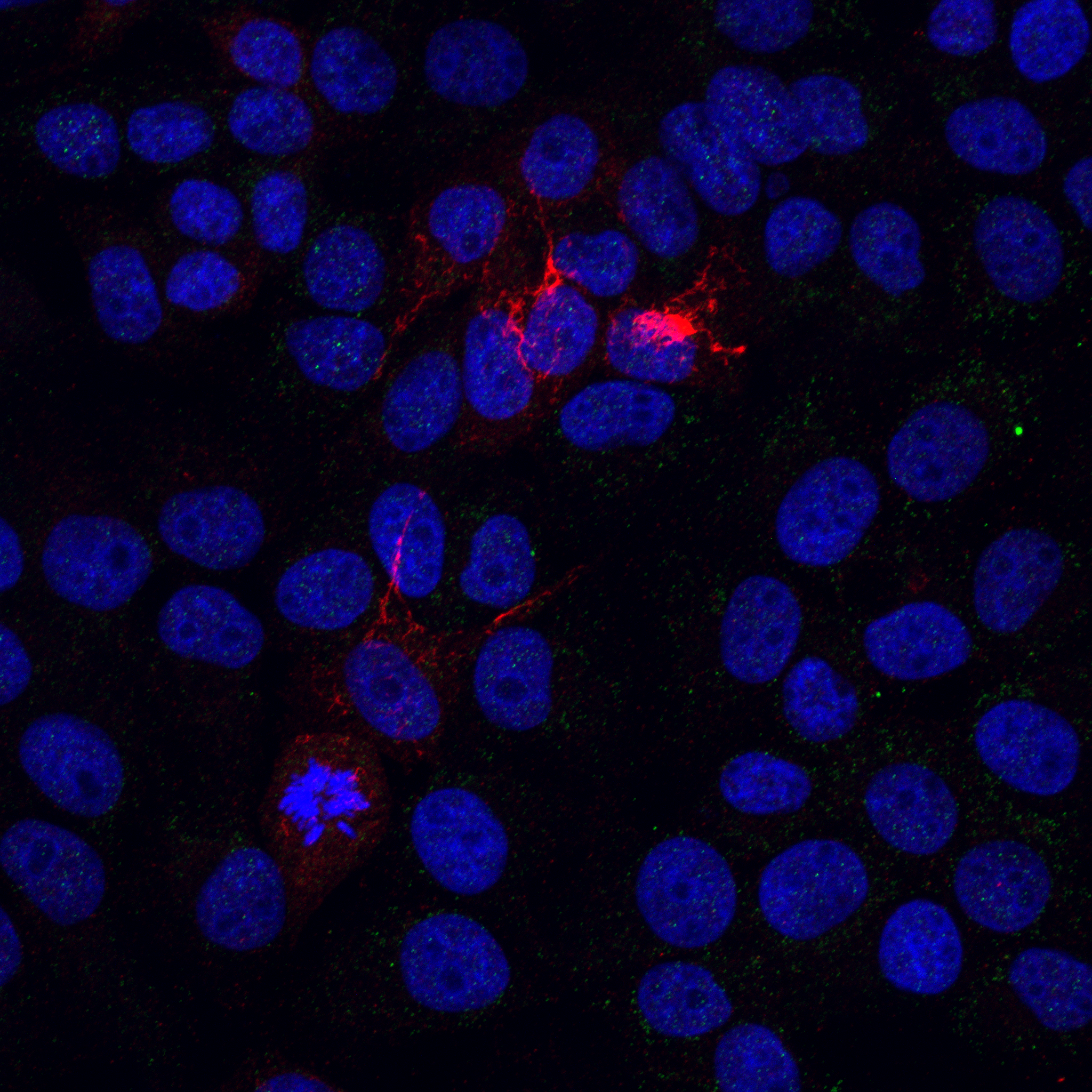

Supplement: Supplementary file 9 — Source data Fig. 5 [file 44319_2026_751_MOESM9_ESM.zip › Raw_data_Figure 5/Figure 5F/C3-MAX_Cal27 + Exosomas MEFs GqKO DAPI PDGFR 488 Vimentin 555.tif]

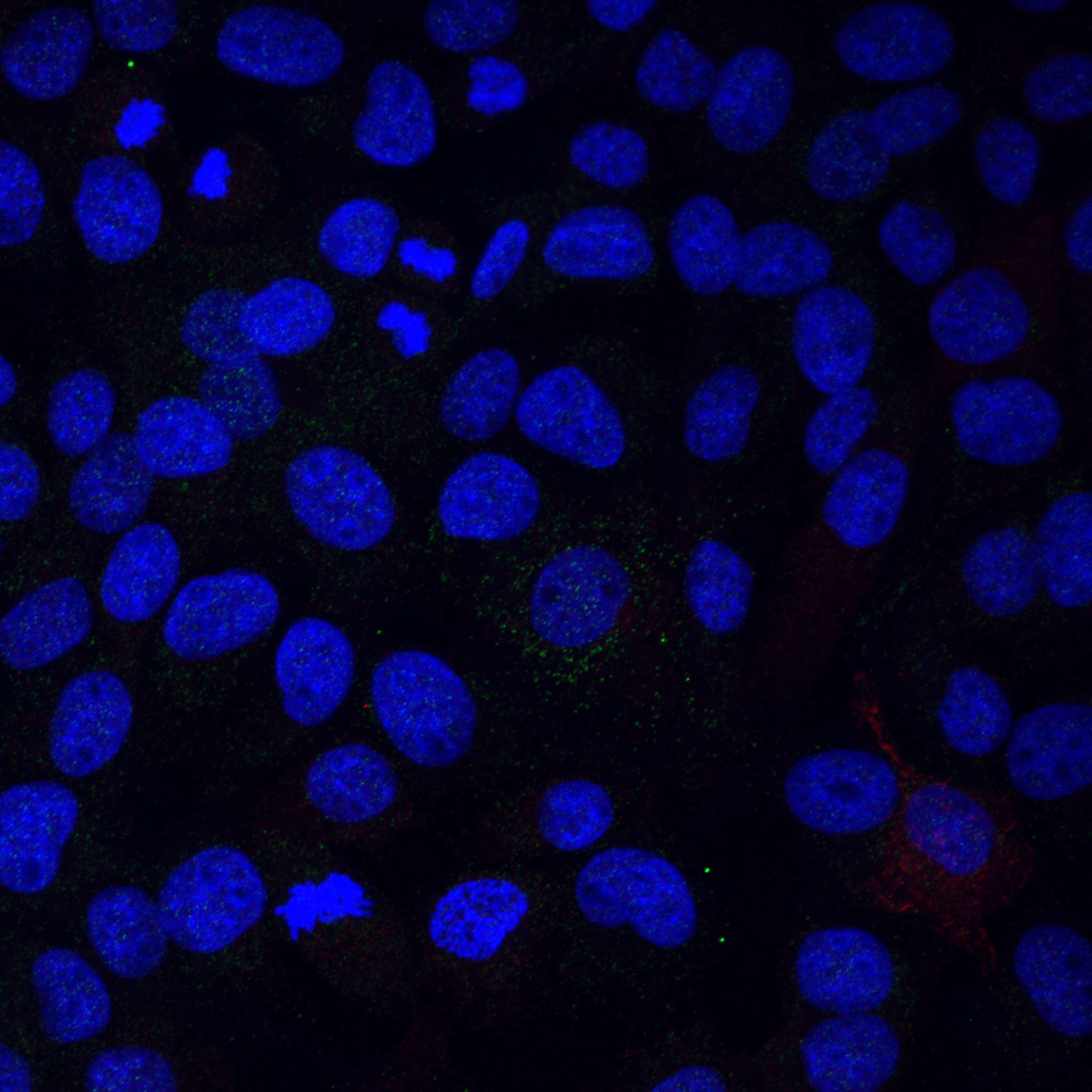

Supplement: Supplementary file 9 — Source data Fig. 5 [file 44319_2026_751_MOESM9_ESM.zip › Raw_data_Figure 5/Figure 5F/C3-MAX_Cal27 + Exosomas MEFs WT DAPI PDGFR 488 Vimentin 555.tif]

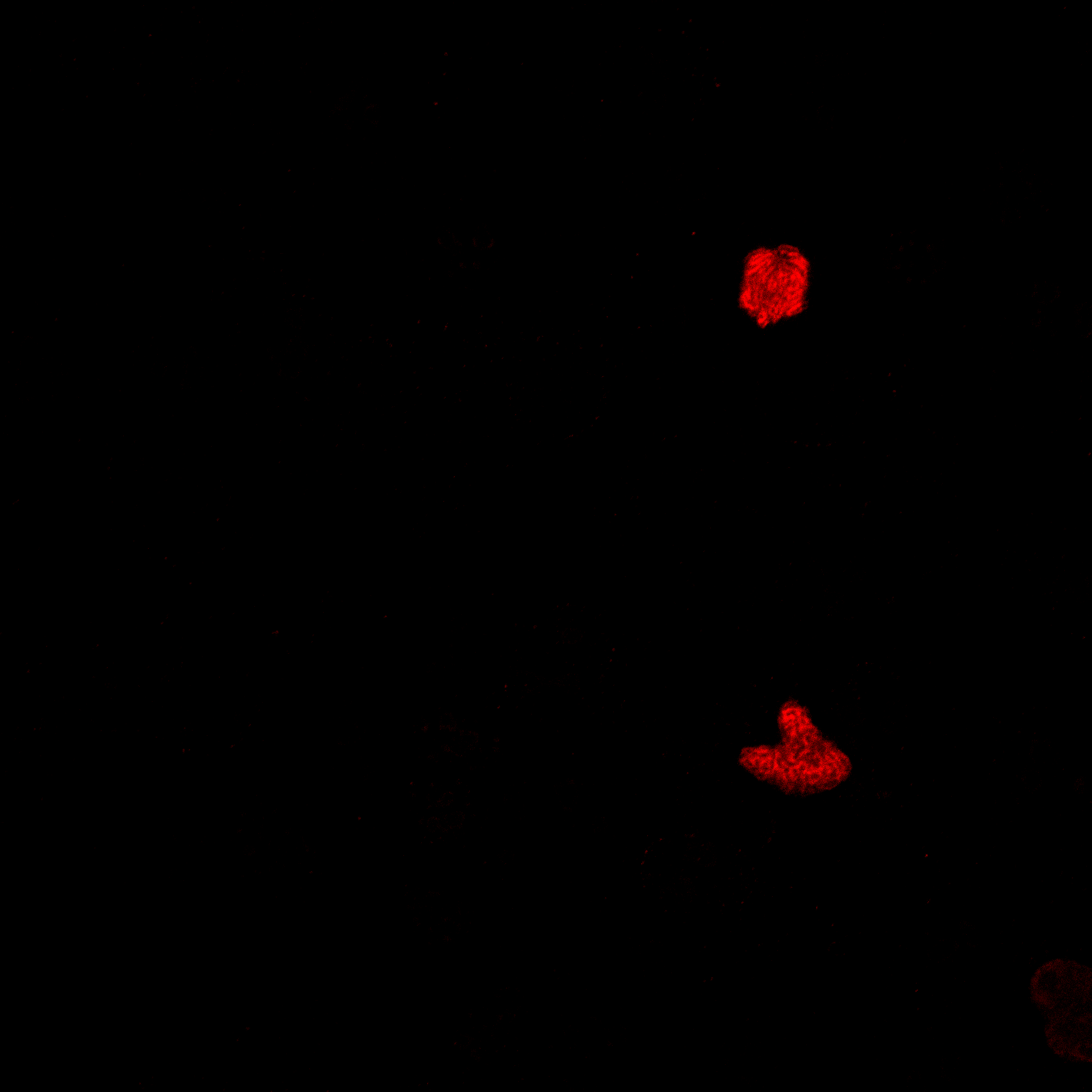

Supplement: Supplementary file 9 — Source data Fig. 5 [file 44319_2026_751_MOESM9_ESM.zip › Raw_data_Figure 5/Figure 5F/C3-MAX_Cal27 + Exosomas MEFs WT Ki67 555.tif]

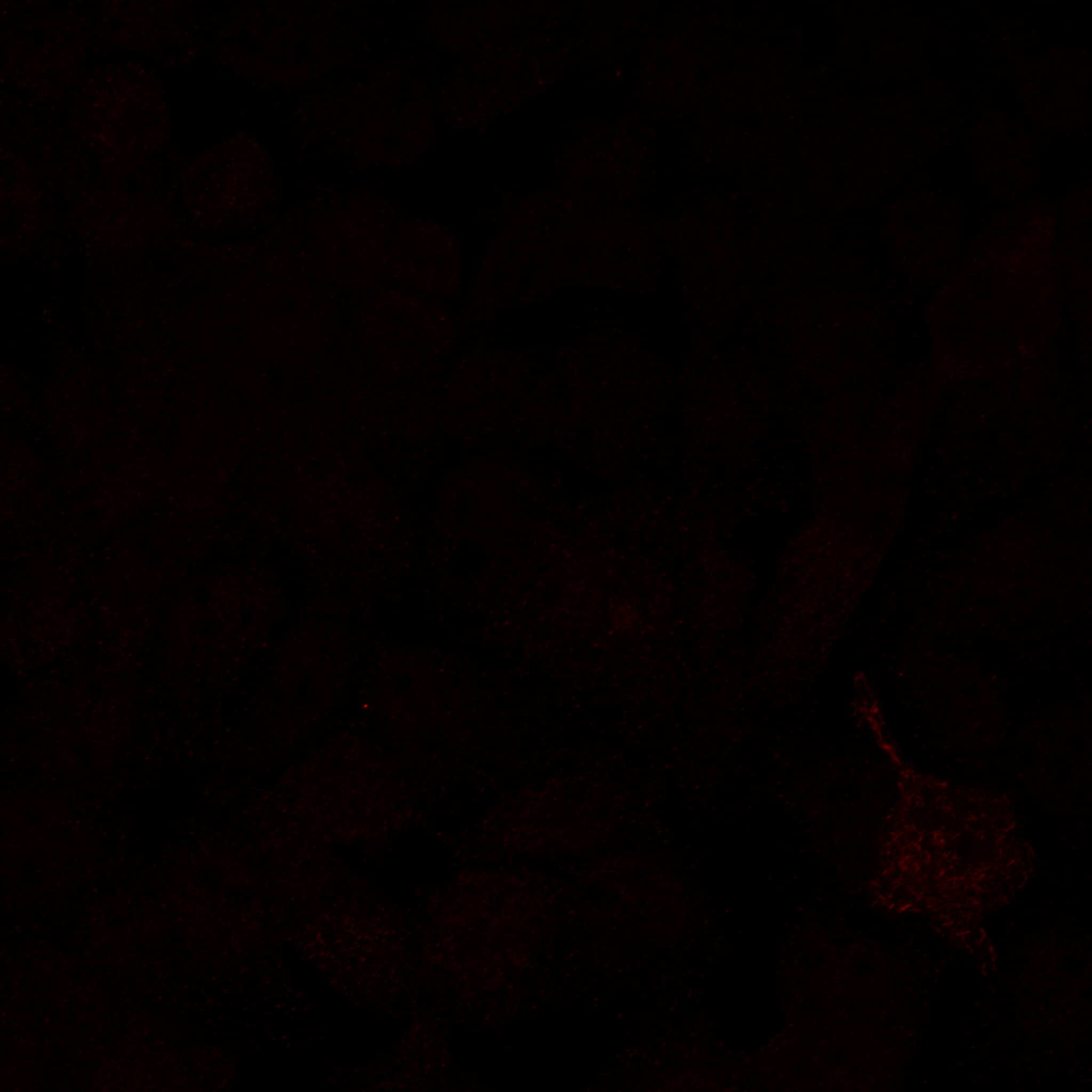

Supplement: Supplementary file 9 — Source data Fig. 5 [file 44319_2026_751_MOESM9_ESM.zip › Raw_data_Figure 5/Figure 5F/C3-MAX_Cal27 + Exosomas MEFs WT Vimentin 555.tif]

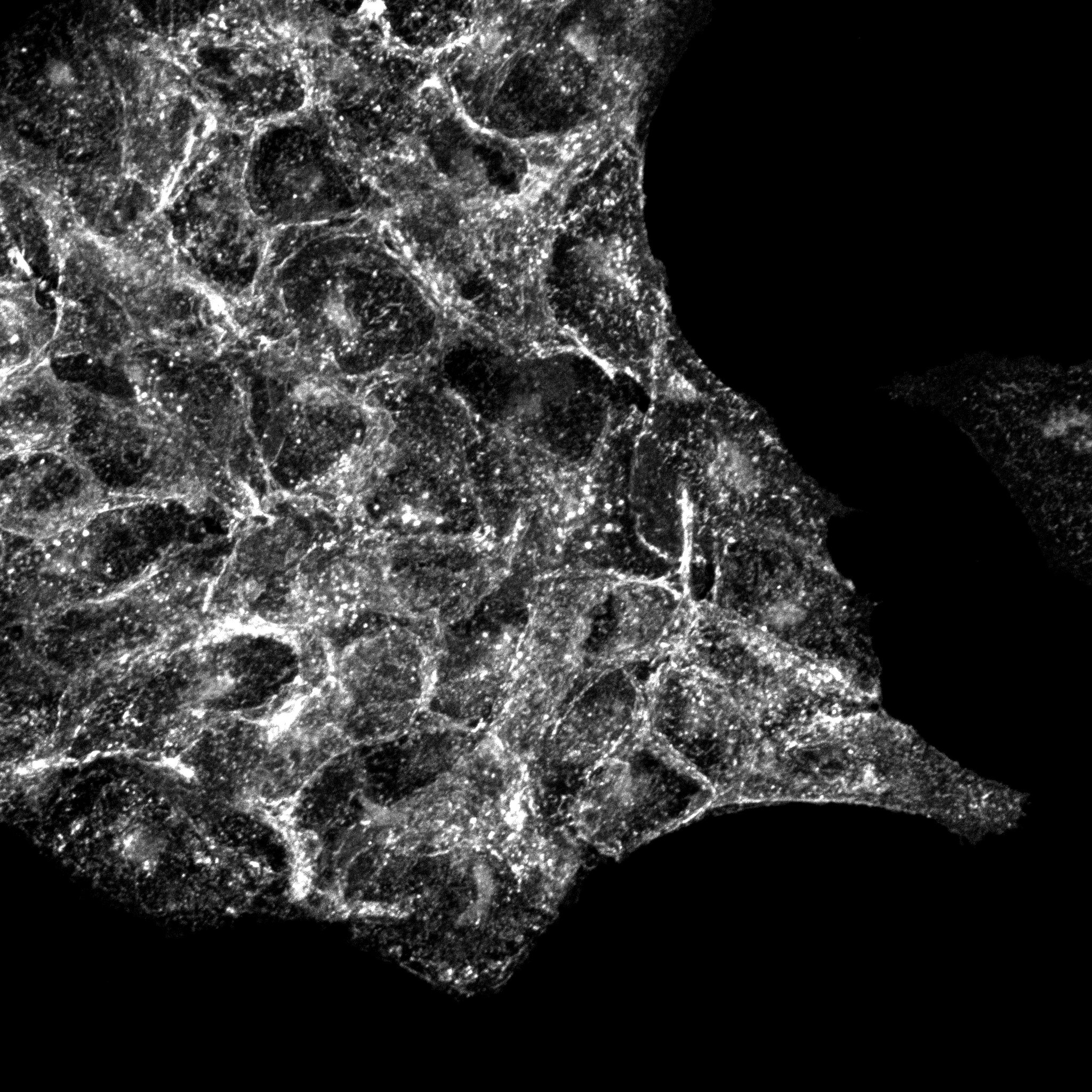

Supplement: Supplementary file 9 — Source data Fig. 5 [file 44319_2026_751_MOESM9_ESM.zip › Raw_data_Figure 5/Figure 5F/MAX_Cal27 + Exosomas WT DAPI Vimentin 555 Cav1 647 only cav1.jpg]

## Slide 1
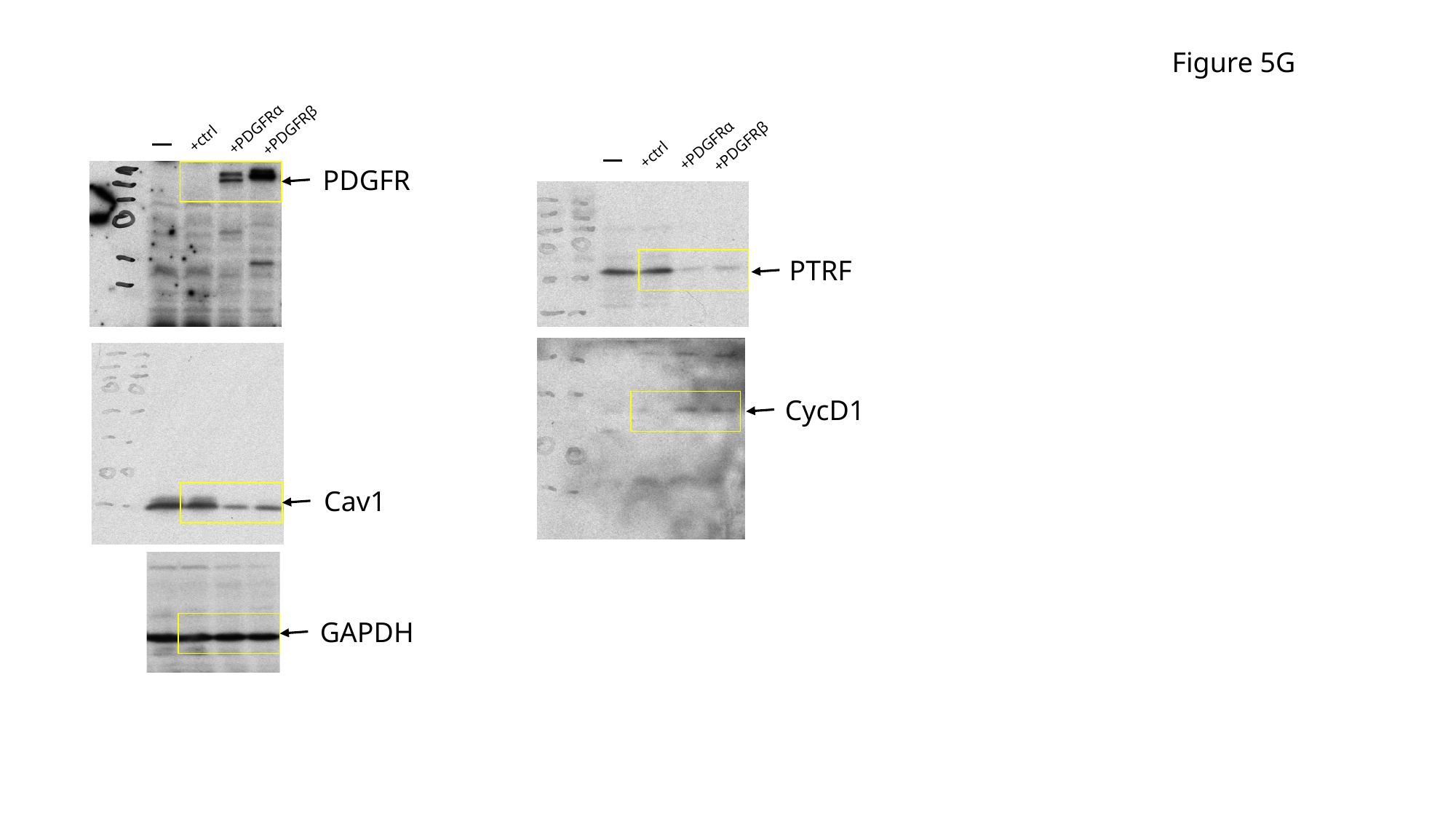

Figure 5G
+PDGFRα
+PDGFRβ
+ctrl
+PDGFRα
+PDGFRβ
+ctrl
PDGFR
PTRF
CycD1
Cav1
GAPDH

Supplement: Supplementary file 9 — Source data Fig. 5 [file 44319_2026_751_MOESM9_ESM.zip › Raw_data_Figure 5/Figure 5G/raw_blots_5G.pptx]

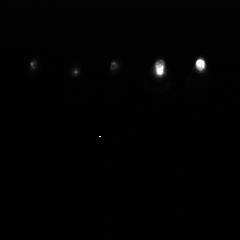

Supplement: Supplementary file 10 — Source data Fig. 6 [file 44319_2026_751_MOESM10_ESM.zip › Raw_data_Figure 6/Figure 6A/INL20230123100948_SEQ/INL20230123100948_001/luminescent (2).TIF]

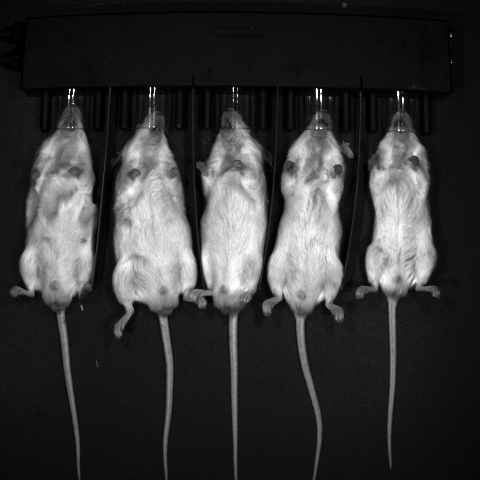

Supplement: Supplementary file 10 — Source data Fig. 6 [file 44319_2026_751_MOESM10_ESM.zip › Raw_data_Figure 6/Figure 6A/INL20230123100948_SEQ/INL20230123100948_001/photograph (2).TIF]

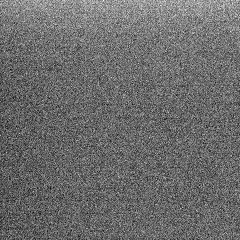

Supplement: Supplementary file 10 — Source data Fig. 6 [file 44319_2026_751_MOESM10_ESM.zip › Raw_data_Figure 6/Figure 6A/INL20230123100948_SEQ/INL20230123100948_001/readbiasonly (2).TIF]

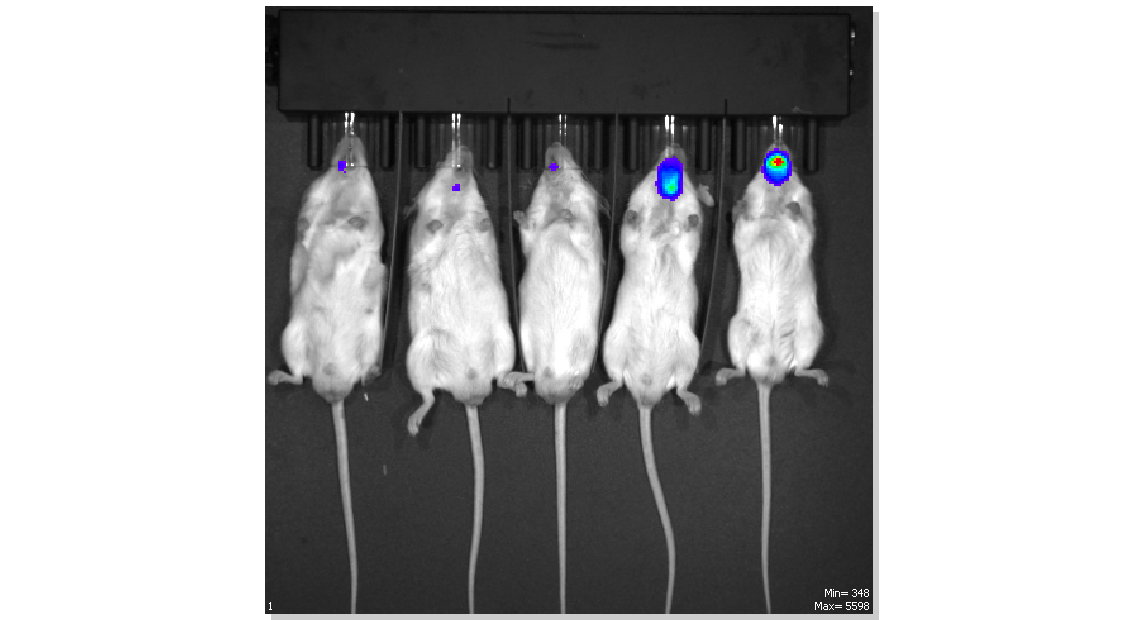

Supplement: Supplementary file 10 — Source data Fig. 6 [file 44319_2026_751_MOESM10_ESM.zip › Raw_data_Figure 6/Figure 6A/INL20230123100948_SEQ/INL20230123100948_SEQ (2).PNG]

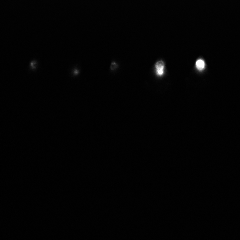

Supplement: Supplementary file 10 — Source data Fig. 6 [file 44319_2026_751_MOESM10_ESM.zip › Raw_data_Figure 6/Figure 6A/INL20230123101246_SEQ/INL20230123101246_001/luminescent (2).TIF]

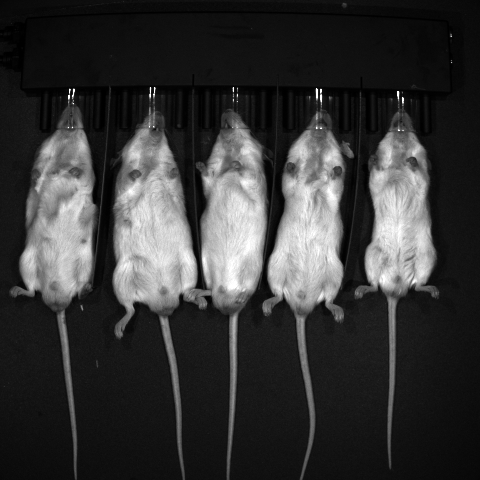

Supplement: Supplementary file 10 — Source data Fig. 6 [file 44319_2026_751_MOESM10_ESM.zip › Raw_data_Figure 6/Figure 6A/INL20230123101246_SEQ/INL20230123101246_001/photograph (2).TIF]

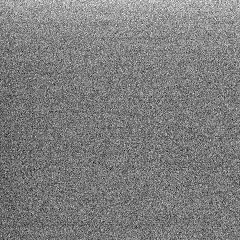

Supplement: Supplementary file 10 — Source data Fig. 6 [file 44319_2026_751_MOESM10_ESM.zip › Raw_data_Figure 6/Figure 6A/INL20230123101246_SEQ/INL20230123101246_001/readbiasonly (2).TIF]

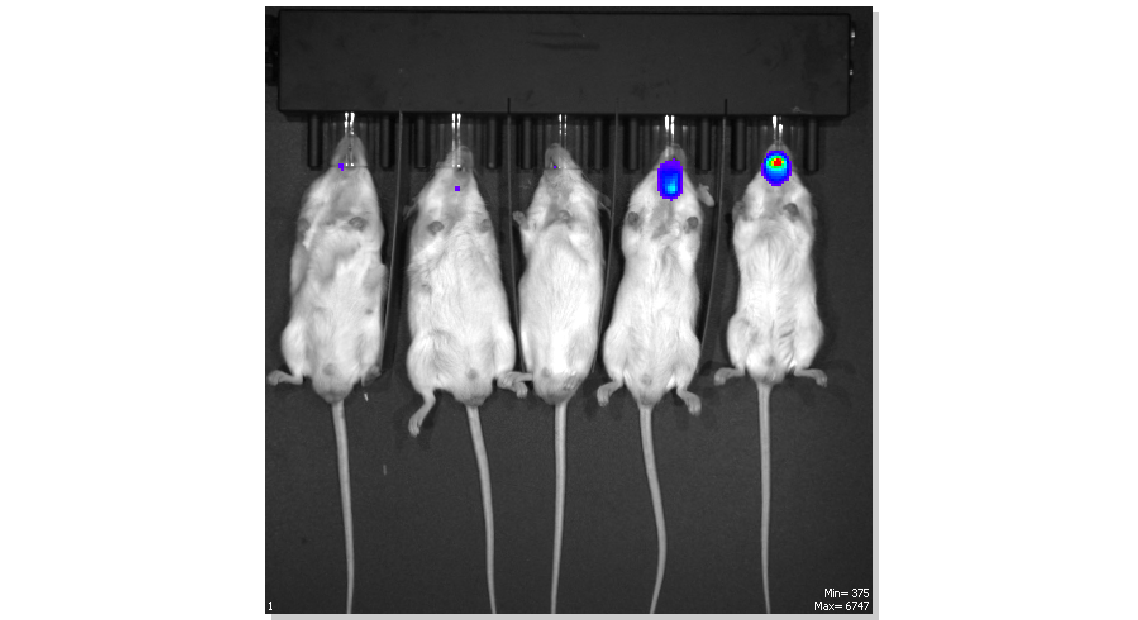

Supplement: Supplementary file 10 — Source data Fig. 6 [file 44319_2026_751_MOESM10_ESM.zip › Raw_data_Figure 6/Figure 6A/INL20230123101246_SEQ/INL20230123101246_SEQ (2).PNG]

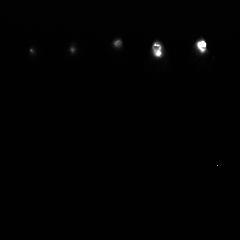

Supplement: Supplementary file 10 — Source data Fig. 6 [file 44319_2026_751_MOESM10_ESM.zip › Raw_data_Figure 6/Figure 6A/INL20230124140611_SEQ/INL20230124140611_001/luminescent (2).TIF]

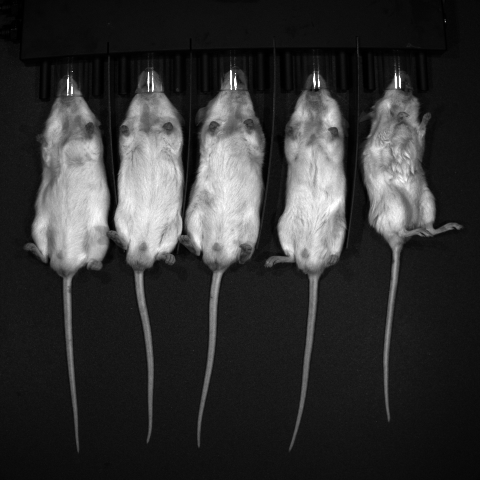

Supplement: Supplementary file 10 — Source data Fig. 6 [file 44319_2026_751_MOESM10_ESM.zip › Raw_data_Figure 6/Figure 6A/INL20230124140611_SEQ/INL20230124140611_001/photograph (2).TIF]

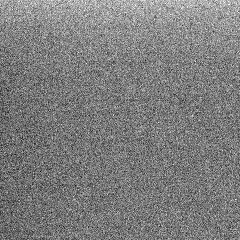

Supplement: Supplementary file 10 — Source data Fig. 6 [file 44319_2026_751_MOESM10_ESM.zip › Raw_data_Figure 6/Figure 6A/INL20230124140611_SEQ/INL20230124140611_001/readbiasonly (2).TIF]

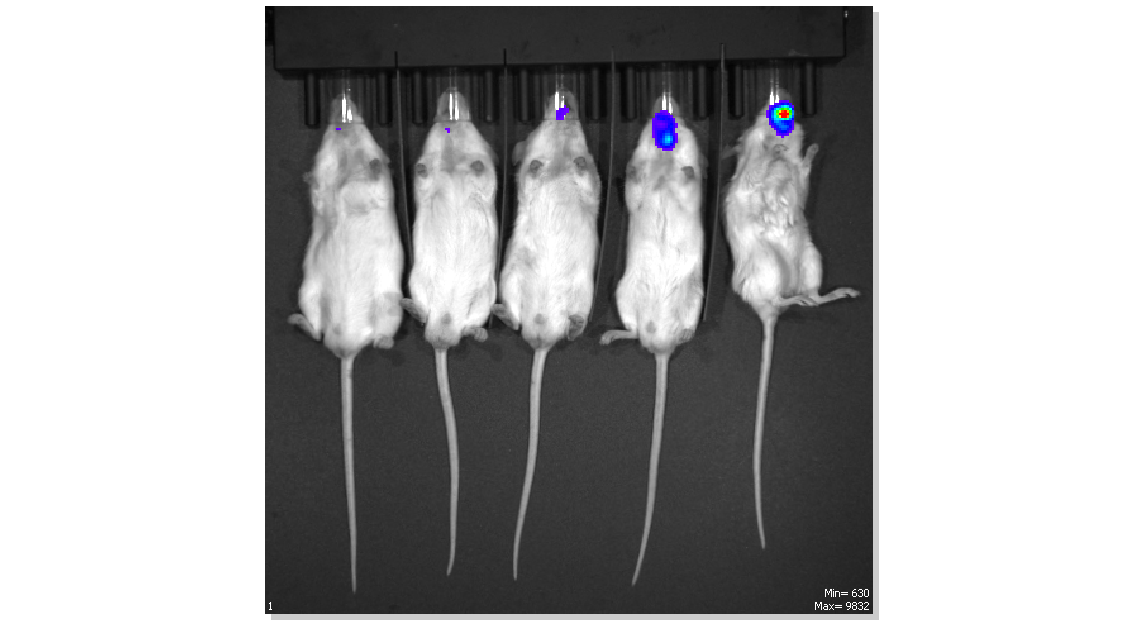

Supplement: Supplementary file 10 — Source data Fig. 6 [file 44319_2026_751_MOESM10_ESM.zip › Raw_data_Figure 6/Figure 6A/INL20230124140611_SEQ/INL20230124140611_SEQ (2).PNG]

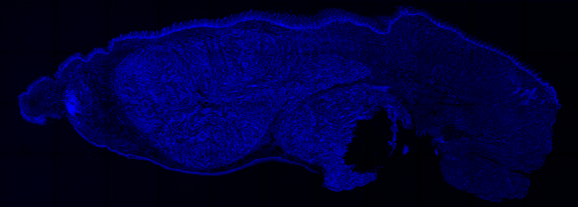

Supplement: Supplementary file 10 — Source data Fig. 6 [file 44319_2026_751_MOESM10_ESM.zip › Raw_data_Figure 6/Figure 6B/Lengua GqKO DAPI.png]

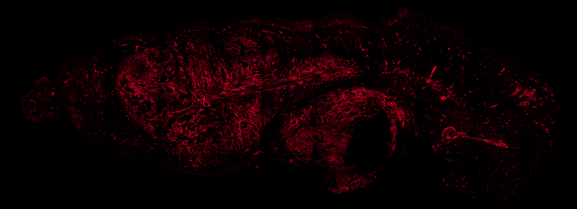

Supplement: Supplementary file 10 — Source data Fig. 6 [file 44319_2026_751_MOESM10_ESM.zip › Raw_data_Figure 6/Figure 6B/Lengua GqKO SMA.png]

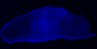

Supplement: Supplementary file 10 — Source data Fig. 6 [file 44319_2026_751_MOESM10_ESM.zip › Raw_data_Figure 6/Figure 6B/Lengua WT DAPI.png]

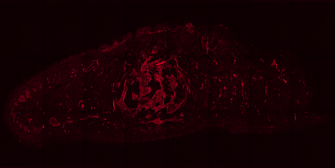

Supplement: Supplementary file 10 — Source data Fig. 6 [file 44319_2026_751_MOESM10_ESM.zip › Raw_data_Figure 6/Figure 6B/Lengua WT SMA.png]

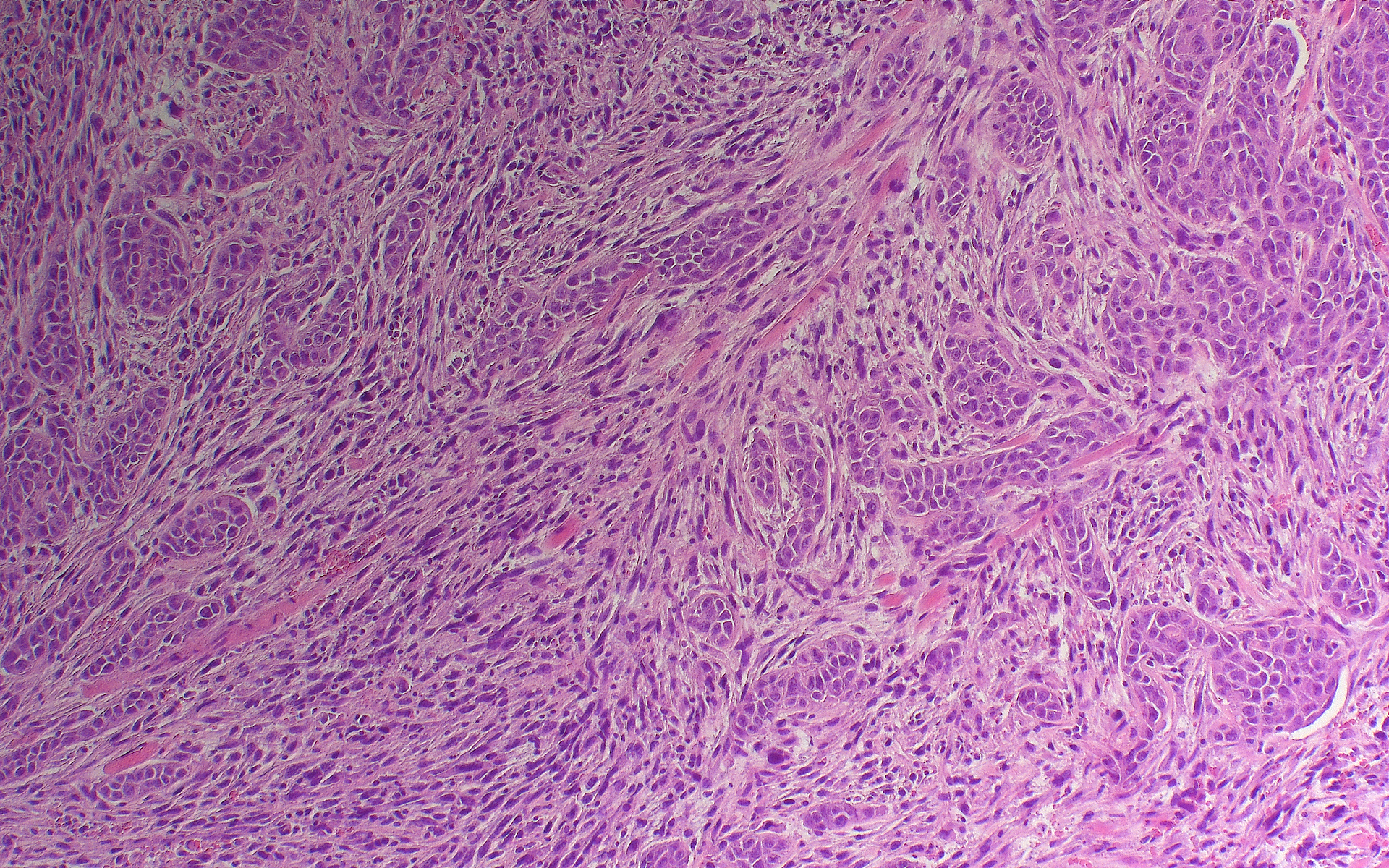

Supplement: Supplementary file 10 — Source data Fig. 6 [file 44319_2026_751_MOESM10_ESM.zip › Raw_data_Figure 6/Figure 6D/Cal27 + GqKO.tif]

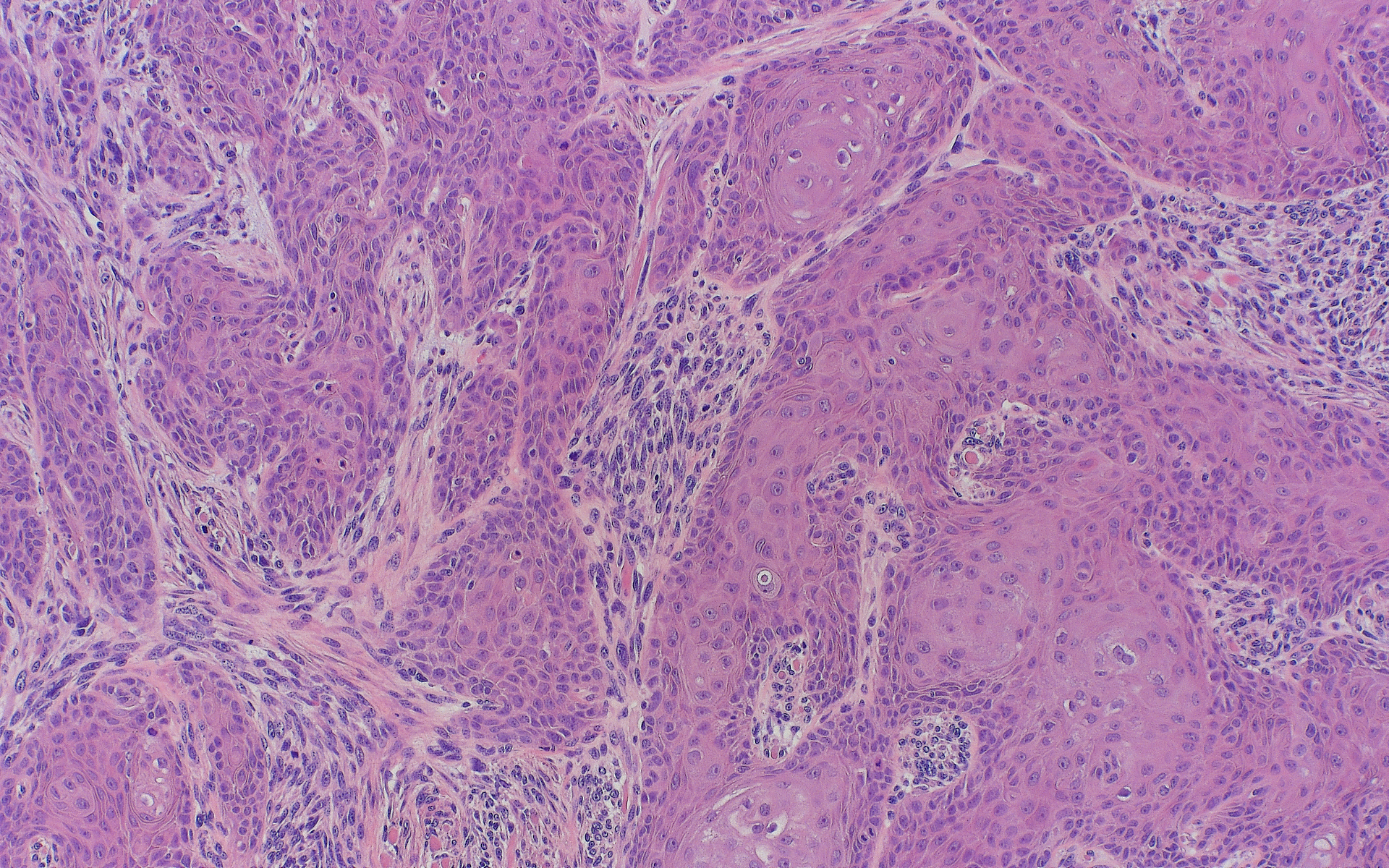

Supplement: Supplementary file 10 — Source data Fig. 6 [file 44319_2026_751_MOESM10_ESM.zip › Raw_data_Figure 6/Figure 6D/Cal27 + WT.tif]

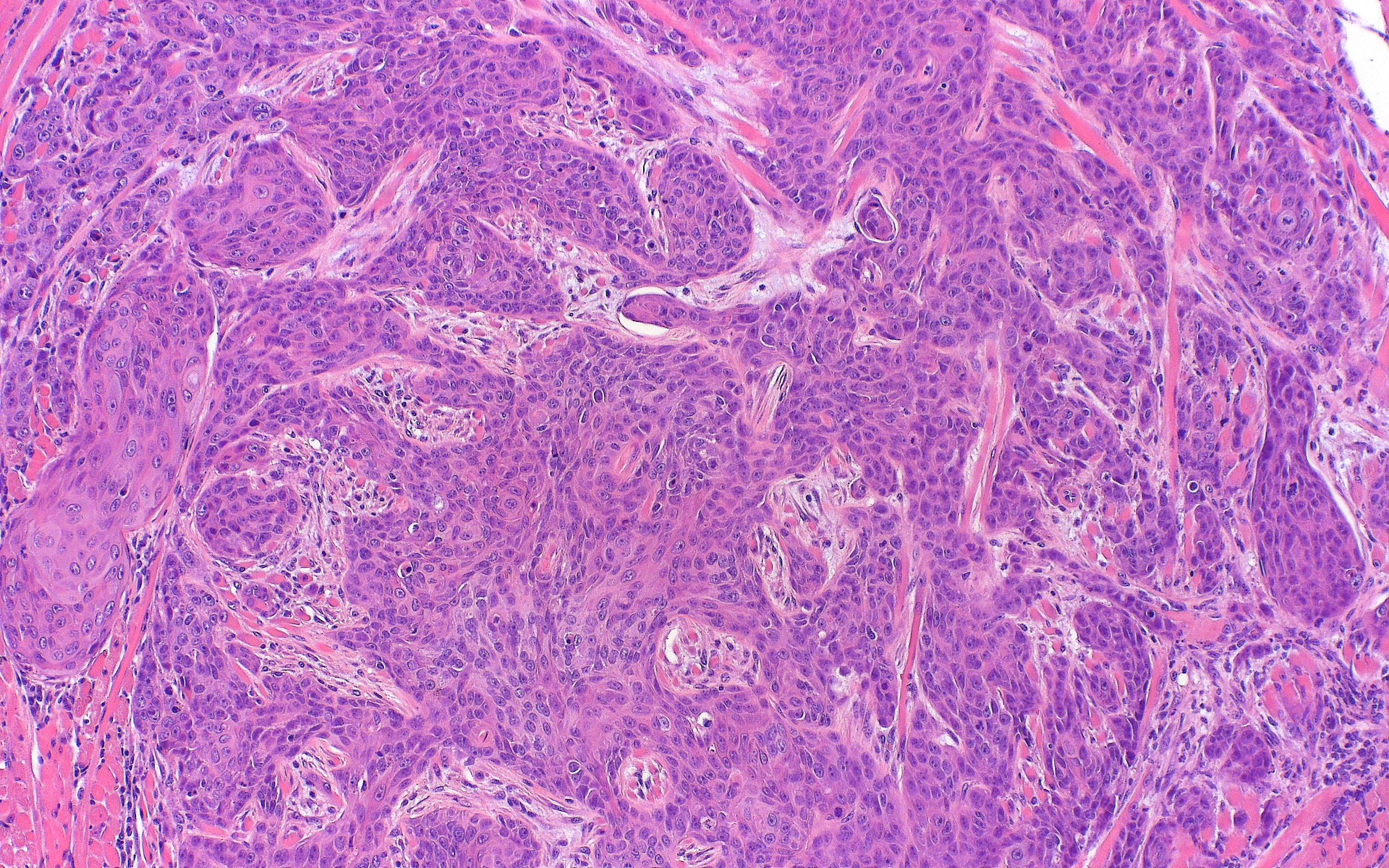

Supplement: Supplementary file 10 — Source data Fig. 6 [file 44319_2026_751_MOESM10_ESM.zip › Raw_data_Figure 6/Figure 6D/Cal27alone.tif]

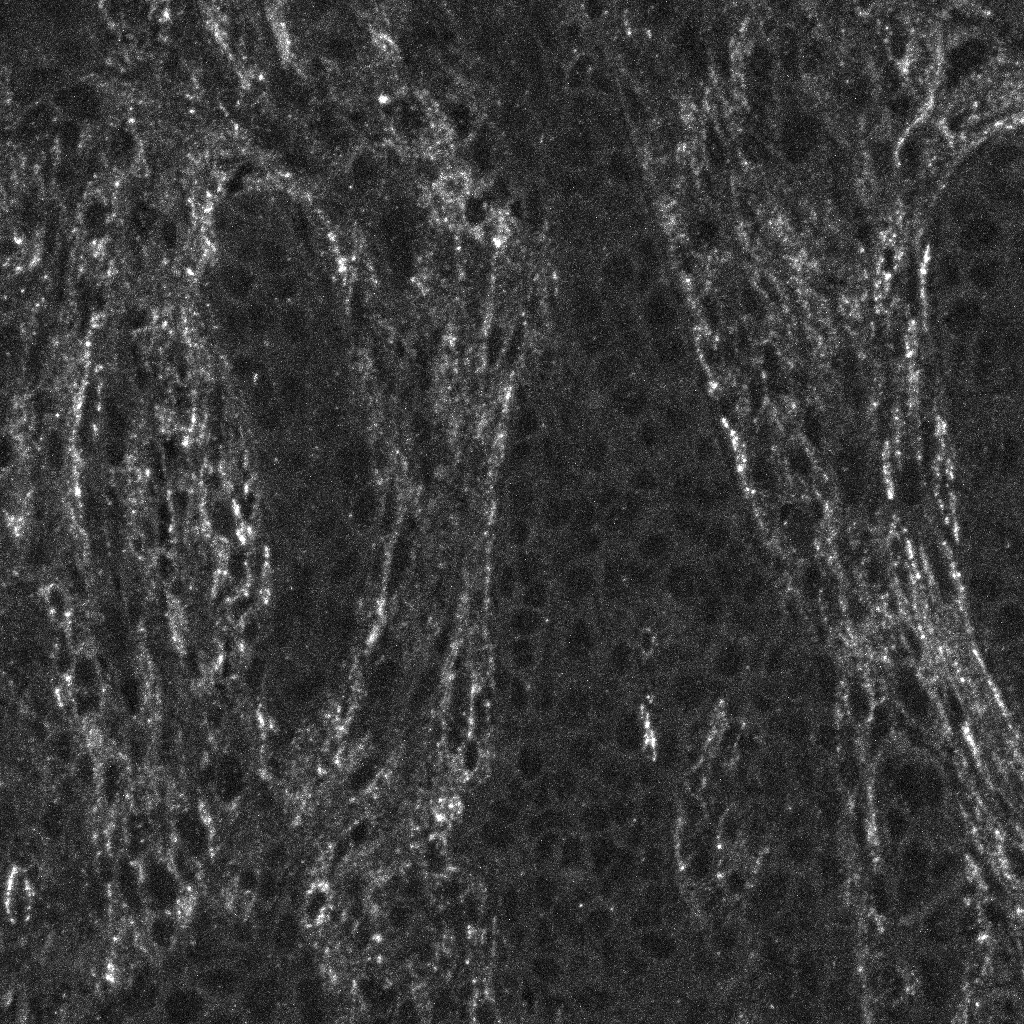

Supplement: Supplementary file 10 — Source data Fig. 6 [file 44319_2026_751_MOESM10_ESM.zip › Raw_data_Figure 6/Figure 6E/Tumor GqKO col I..tif]

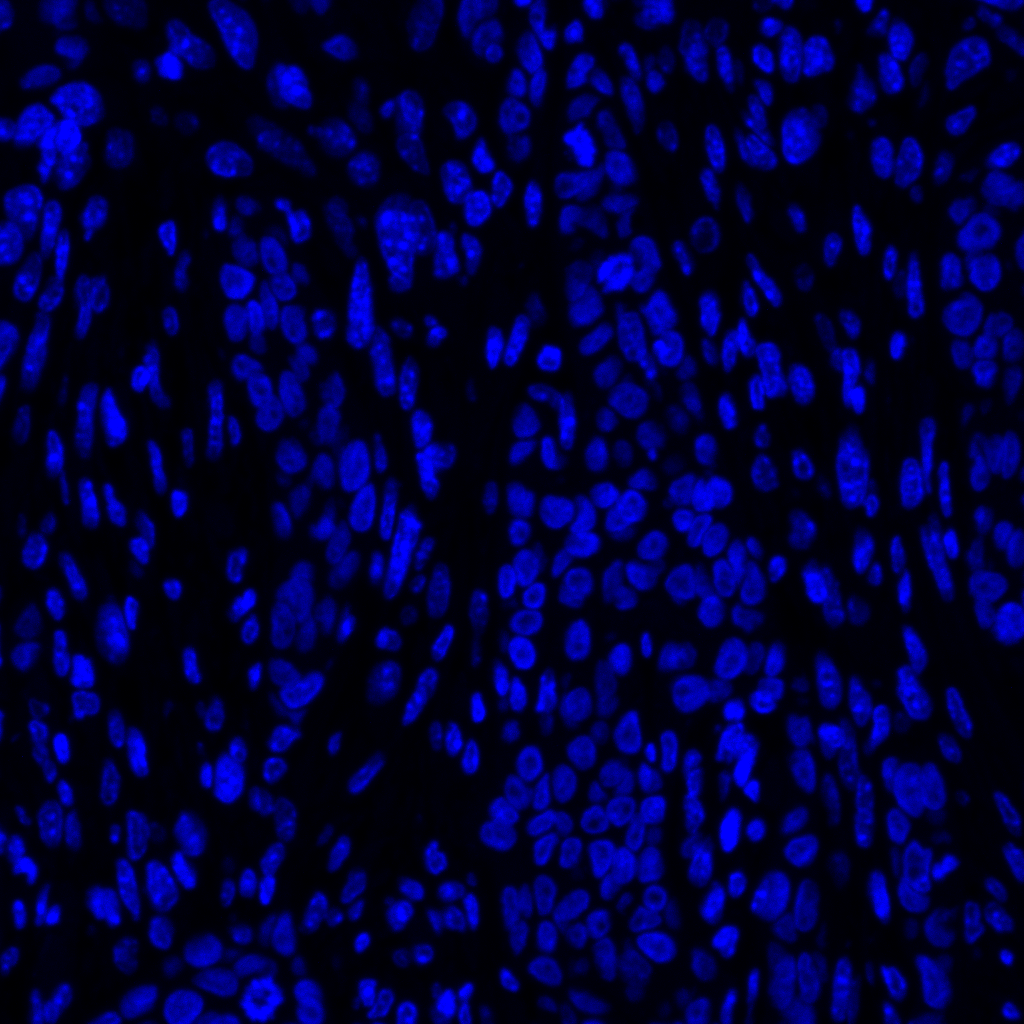

Supplement: Supplementary file 10 — Source data Fig. 6 [file 44319_2026_751_MOESM10_ESM.zip › Raw_data_Figure 6/Figure 6E/Tumor GqKO col l nuclei..tif]

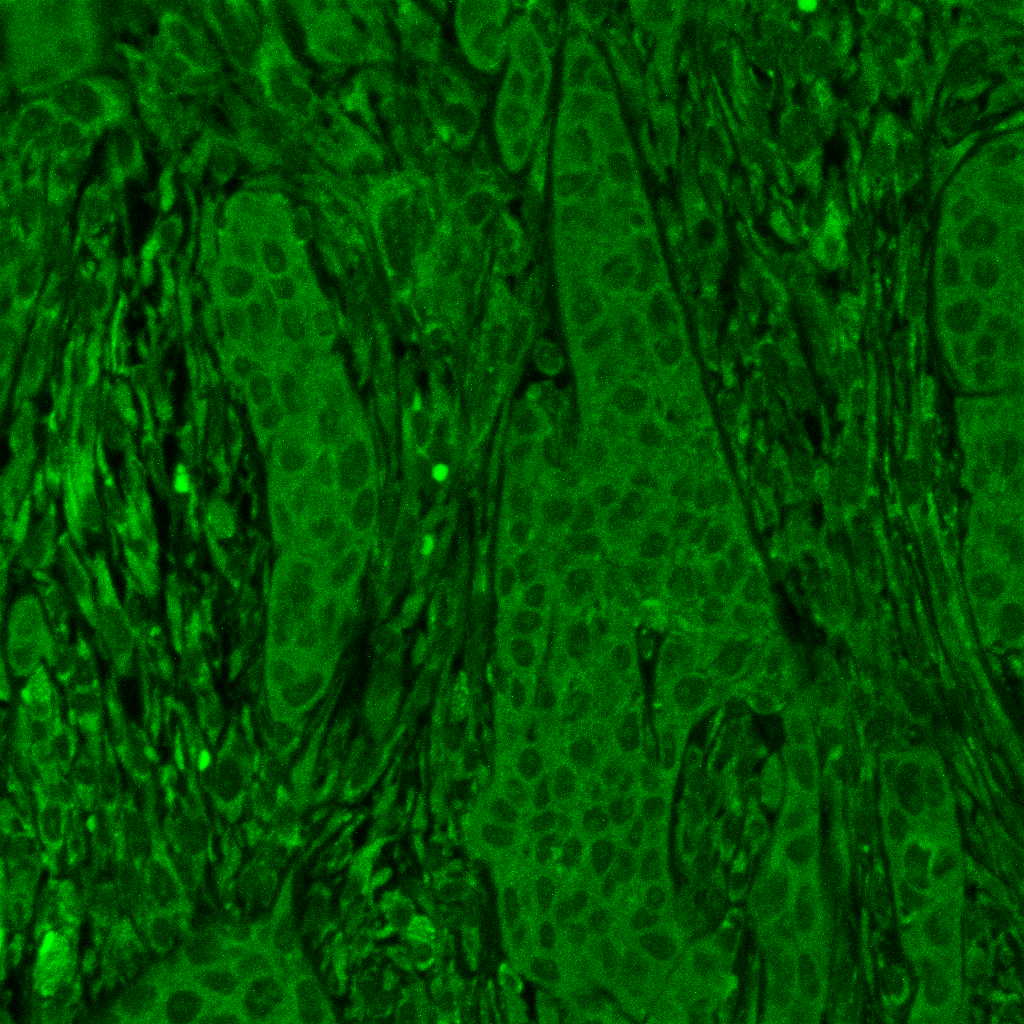

Supplement: Supplementary file 10 — Source data Fig. 6 [file 44319_2026_751_MOESM10_ESM.zip › Raw_data_Figure 6/Figure 6E/Tumor GqKO GFP de col I.tif]

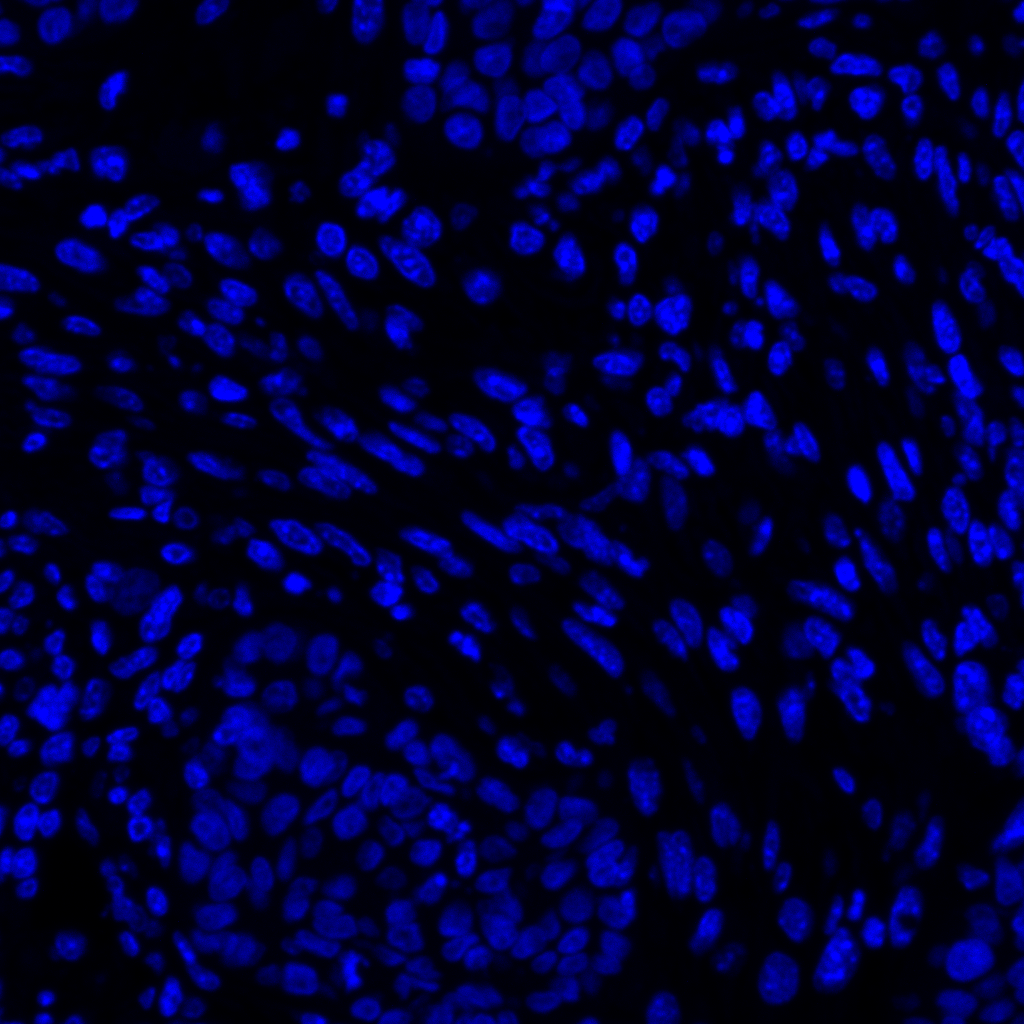

Supplement: Supplementary file 10 — Source data Fig. 6 [file 44319_2026_751_MOESM10_ESM.zip › Raw_data_Figure 6/Figure 6E/Tumor GqKO de pdgfr nuclei..tif]

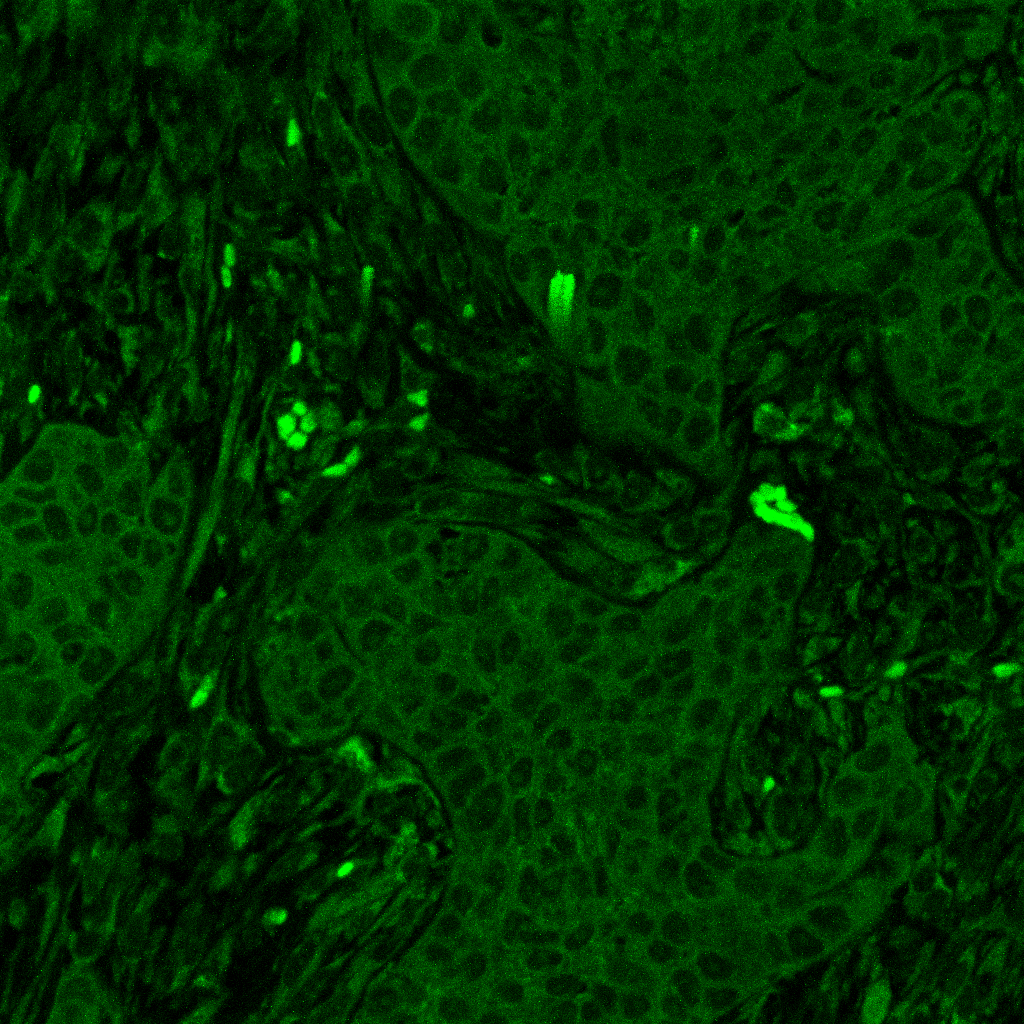

Supplement: Supplementary file 10 — Source data Fig. 6 [file 44319_2026_751_MOESM10_ESM.zip › Raw_data_Figure 6/Figure 6E/Tumor GqKO GFP de LAMP1.tif]

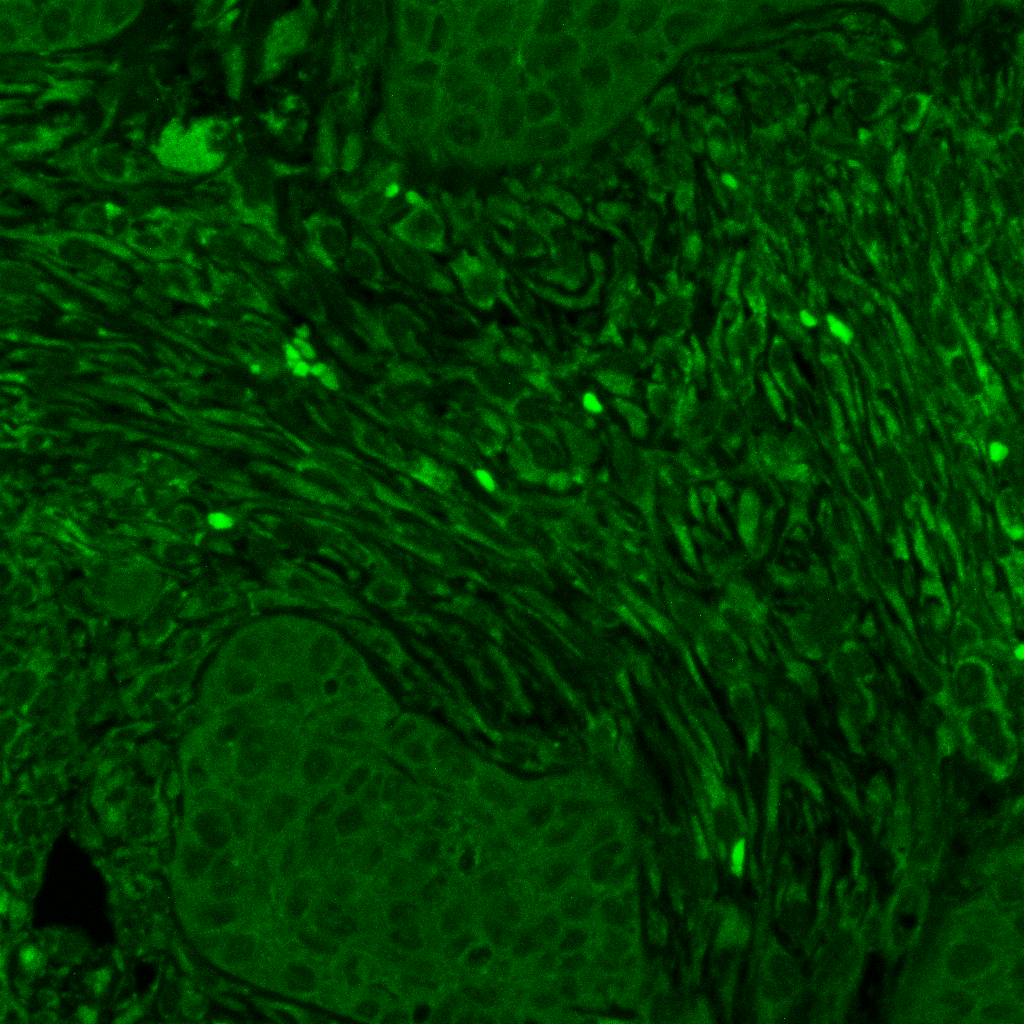

Supplement: Supplementary file 10 — Source data Fig. 6 [file 44319_2026_751_MOESM10_ESM.zip › Raw_data_Figure 6/Figure 6E/Tumor GqKO GFP de pdgfr.tif]

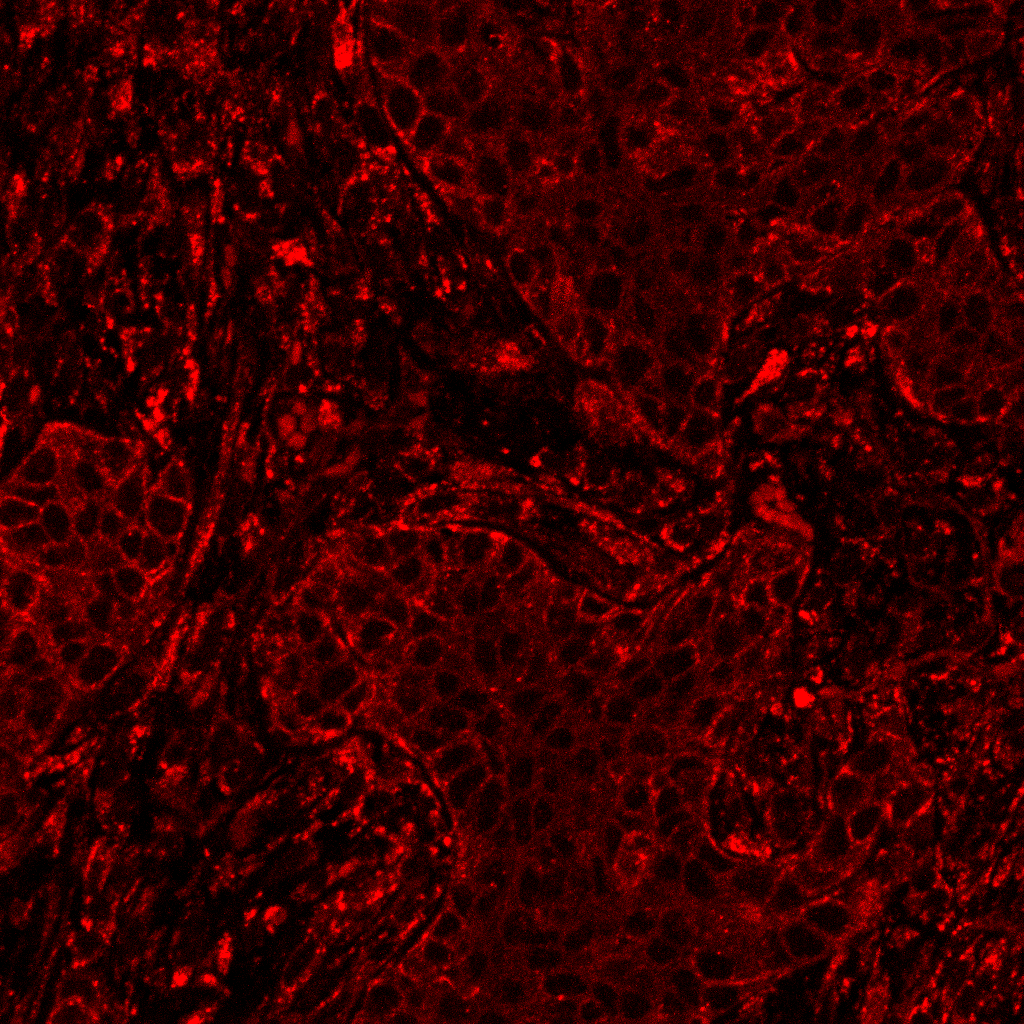

Supplement: Supplementary file 10 — Source data Fig. 6 [file 44319_2026_751_MOESM10_ESM.zip › Raw_data_Figure 6/Figure 6E/Tumor GqKO LAMP1.tif]

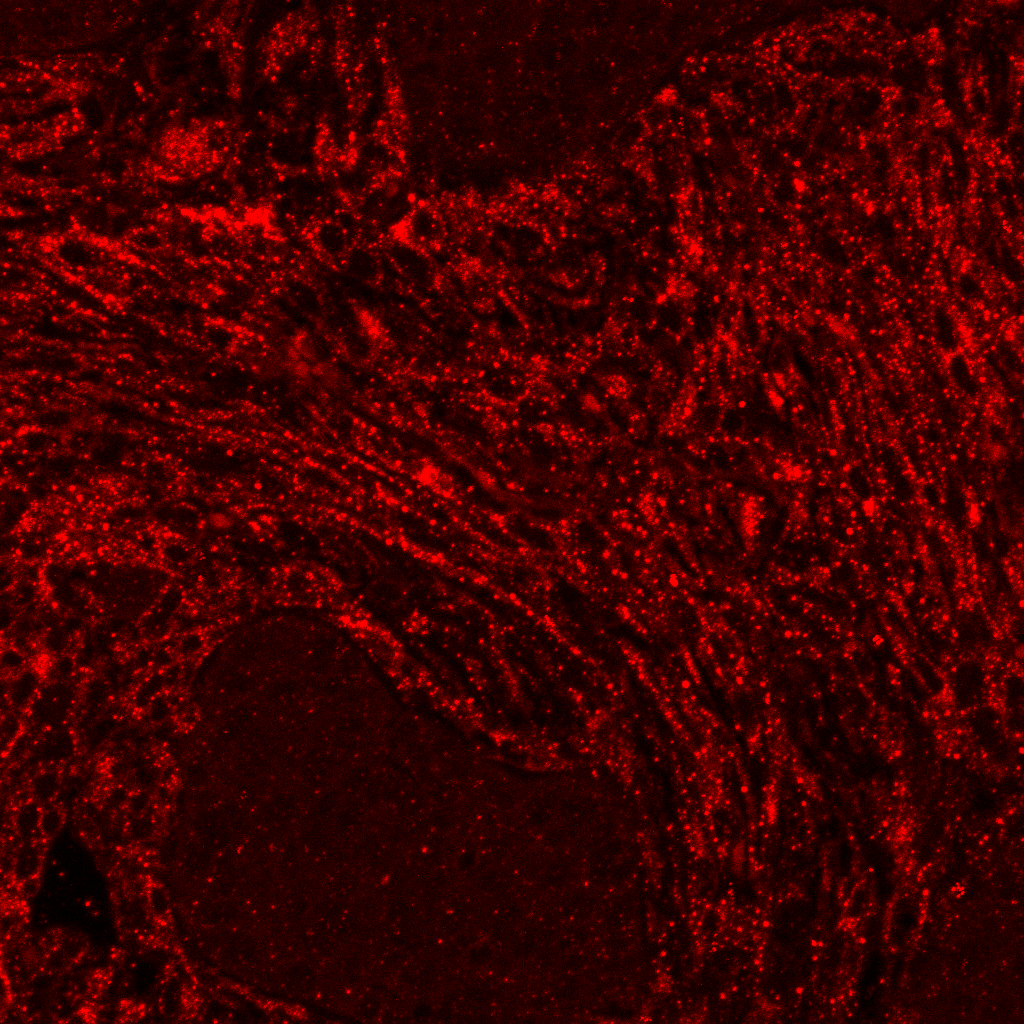

Supplement: Supplementary file 10 — Source data Fig. 6 [file 44319_2026_751_MOESM10_ESM.zip › Raw_data_Figure 6/Figure 6E/Tumor GqKO pdgfr.tif.tif]

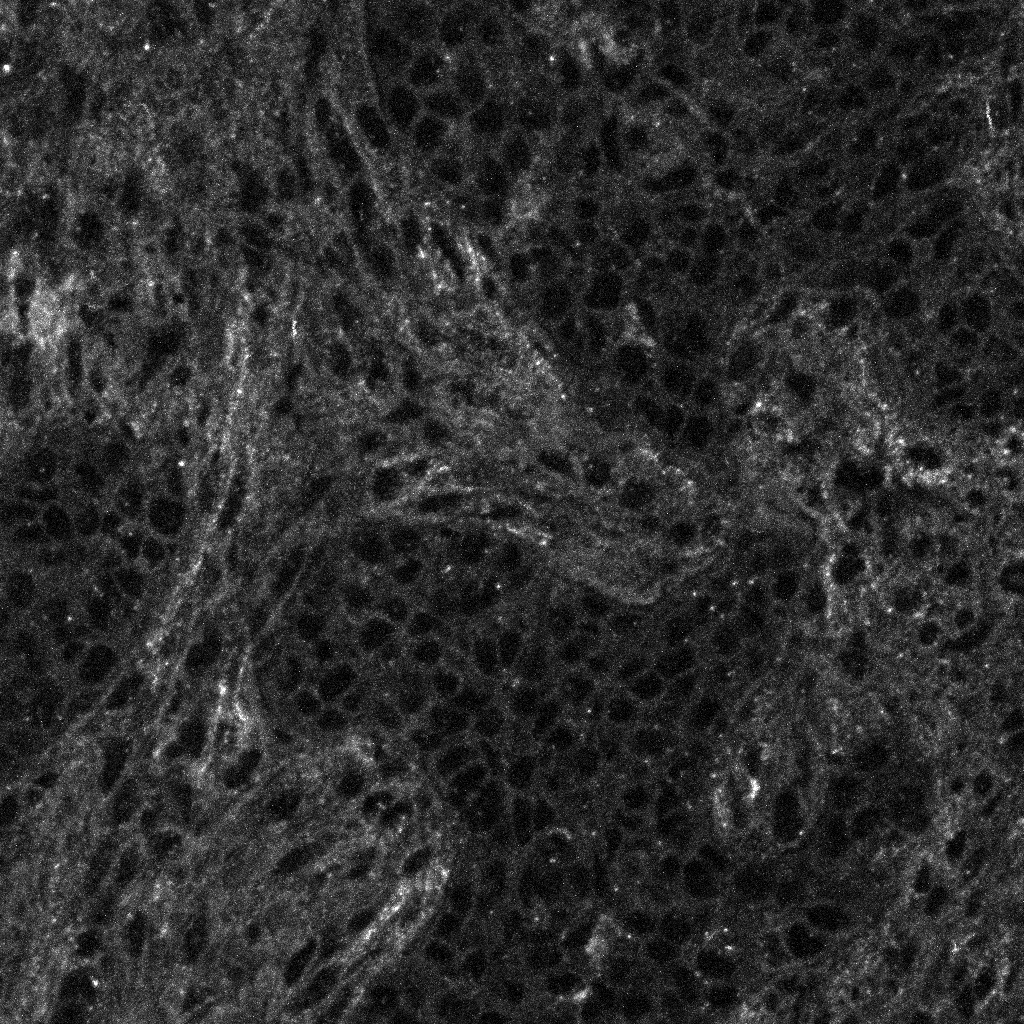

Supplement: Supplementary file 10 — Source data Fig. 6 [file 44319_2026_751_MOESM10_ESM.zip › Raw_data_Figure 6/Figure 6E/Tumor GqKO PTRF.tif]

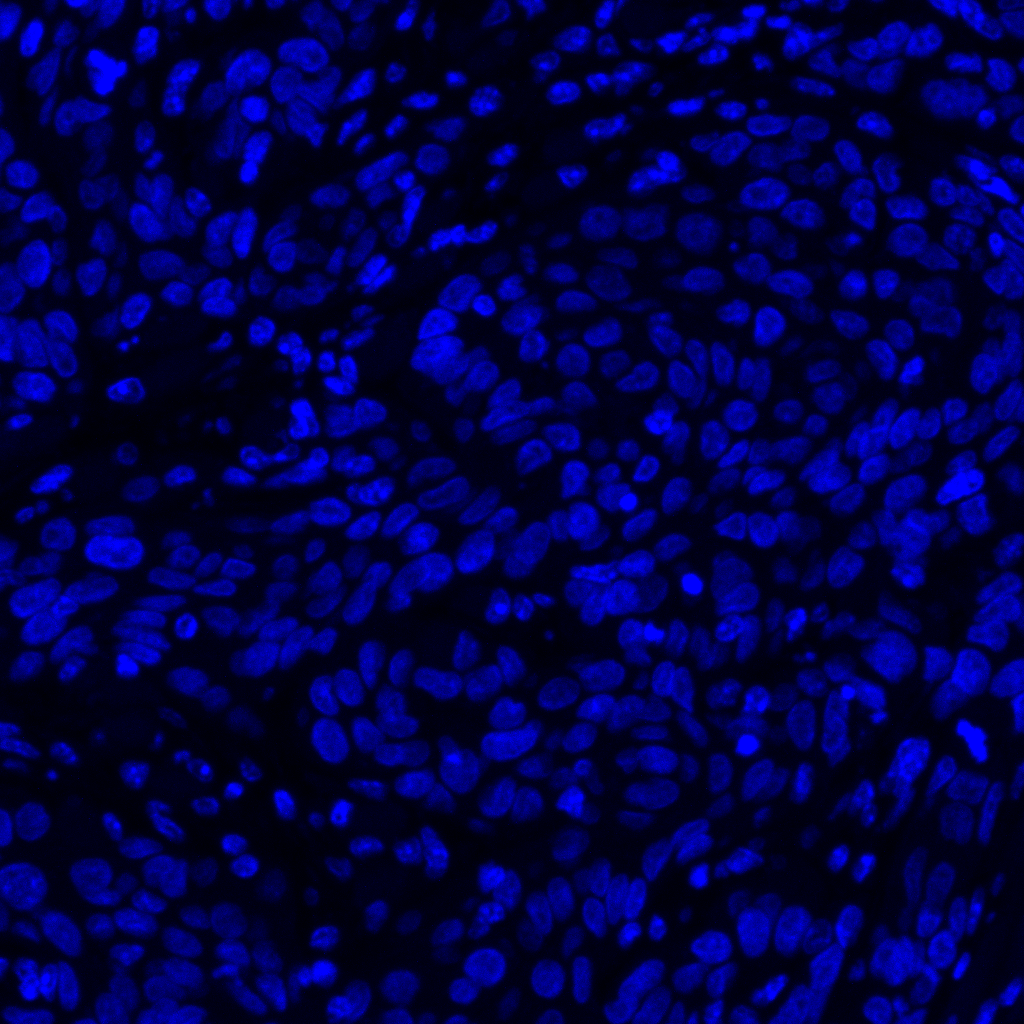

Supplement: Supplementary file 10 — Source data Fig. 6 [file 44319_2026_751_MOESM10_ESM.zip › Raw_data_Figure 6/Figure 6E/Tumor WT nuclei.tif]

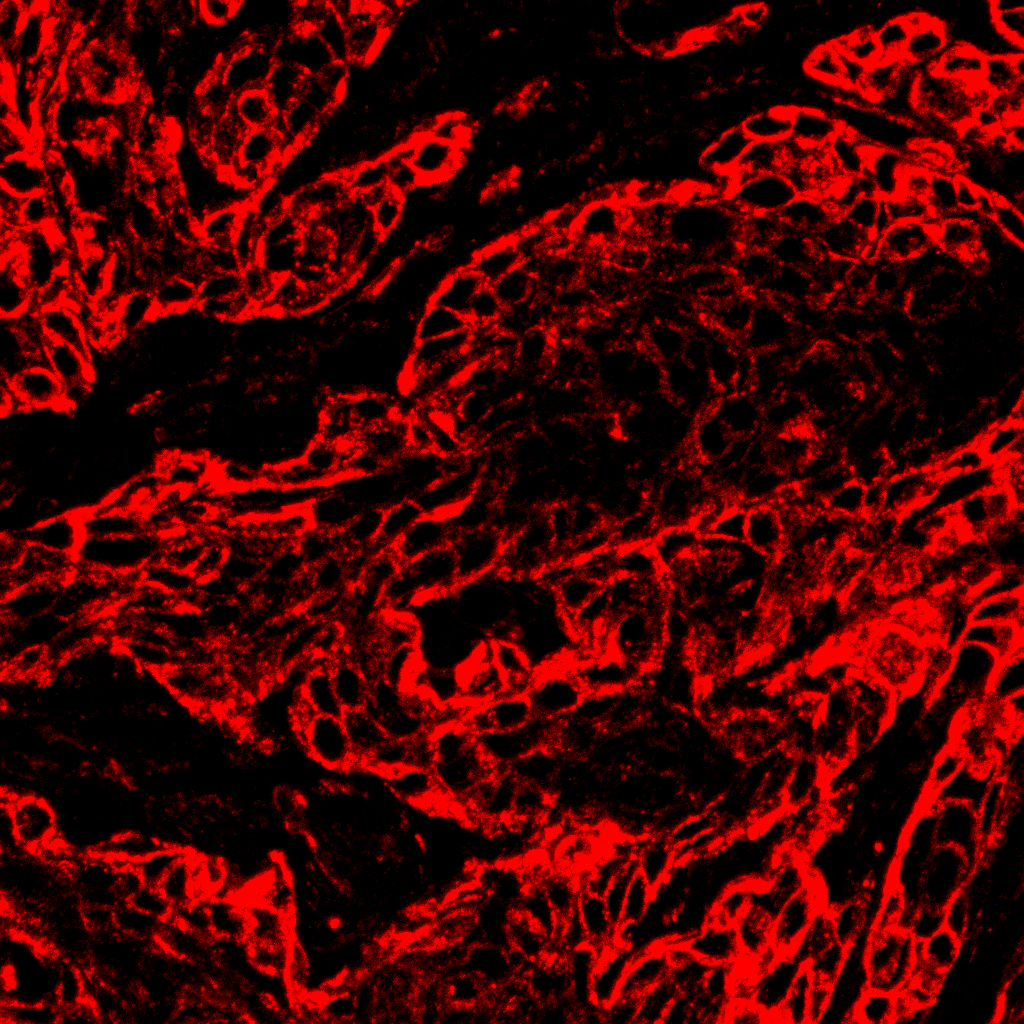

Supplement: Supplementary file 10 — Source data Fig. 6 [file 44319_2026_751_MOESM10_ESM.zip › Raw_data_Figure 6/Figure 6E/Tumor WT cav1.tif]

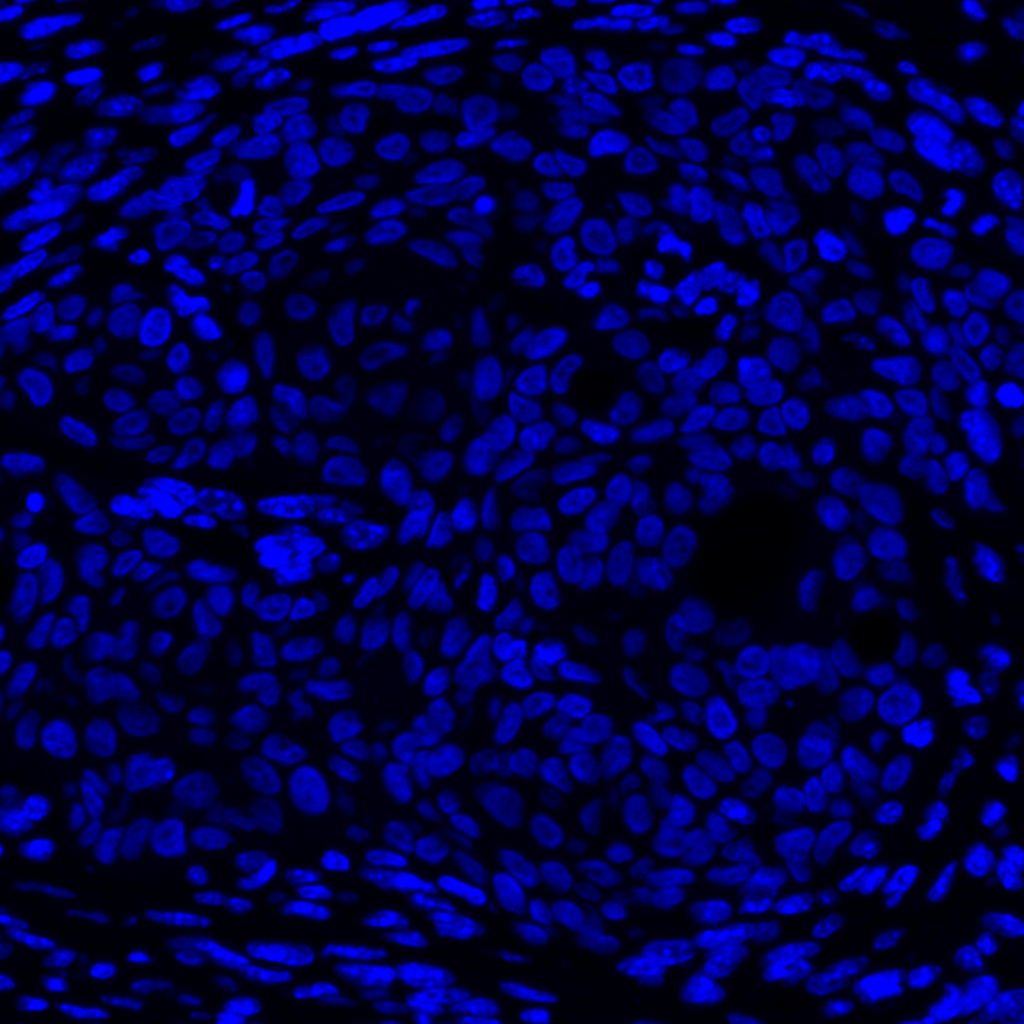

Supplement: Supplementary file 10 — Source data Fig. 6 [file 44319_2026_751_MOESM10_ESM.zip › Raw_data_Figure 6/Figure 6E/Tumor WT col l nuclei..tif]

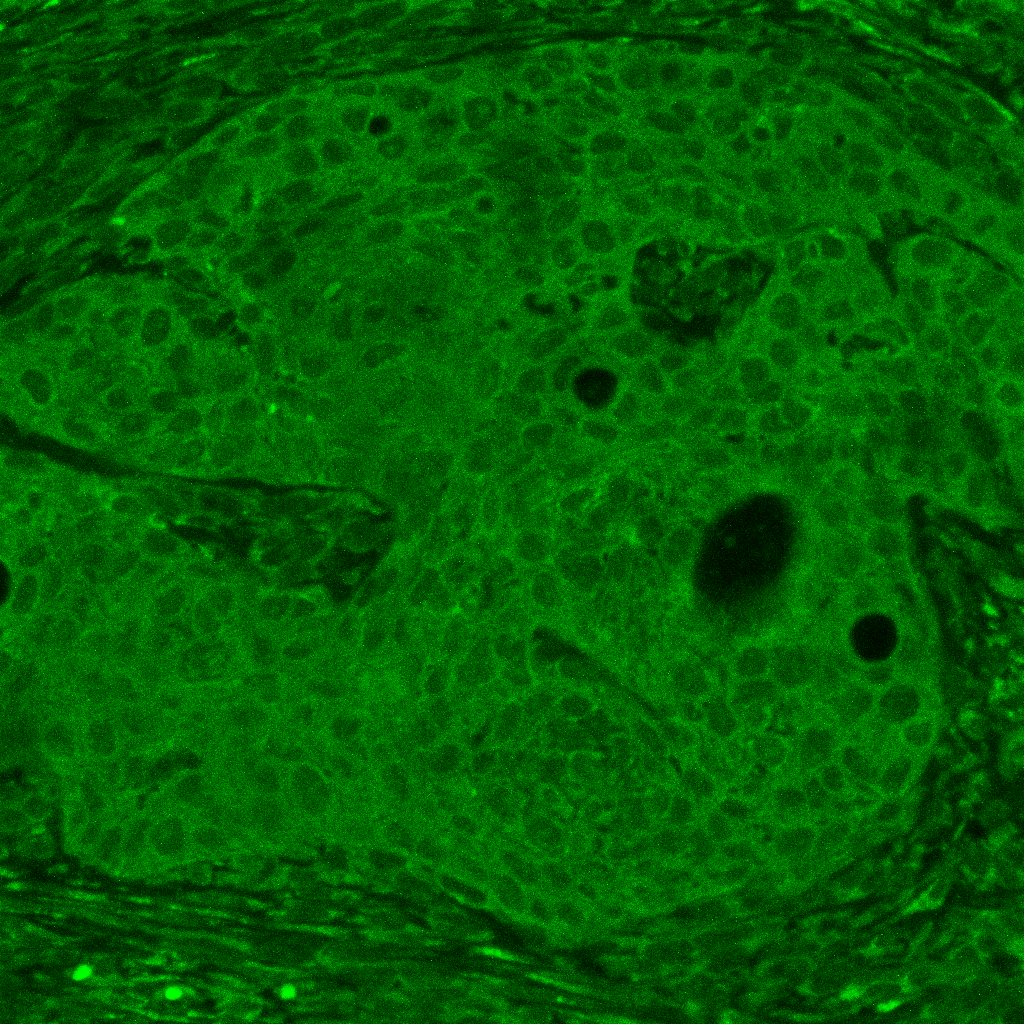

Supplement: Supplementary file 10 — Source data Fig. 6 [file 44319_2026_751_MOESM10_ESM.zip › Raw_data_Figure 6/Figure 6E/Tumor WT GFP de col I..tif]

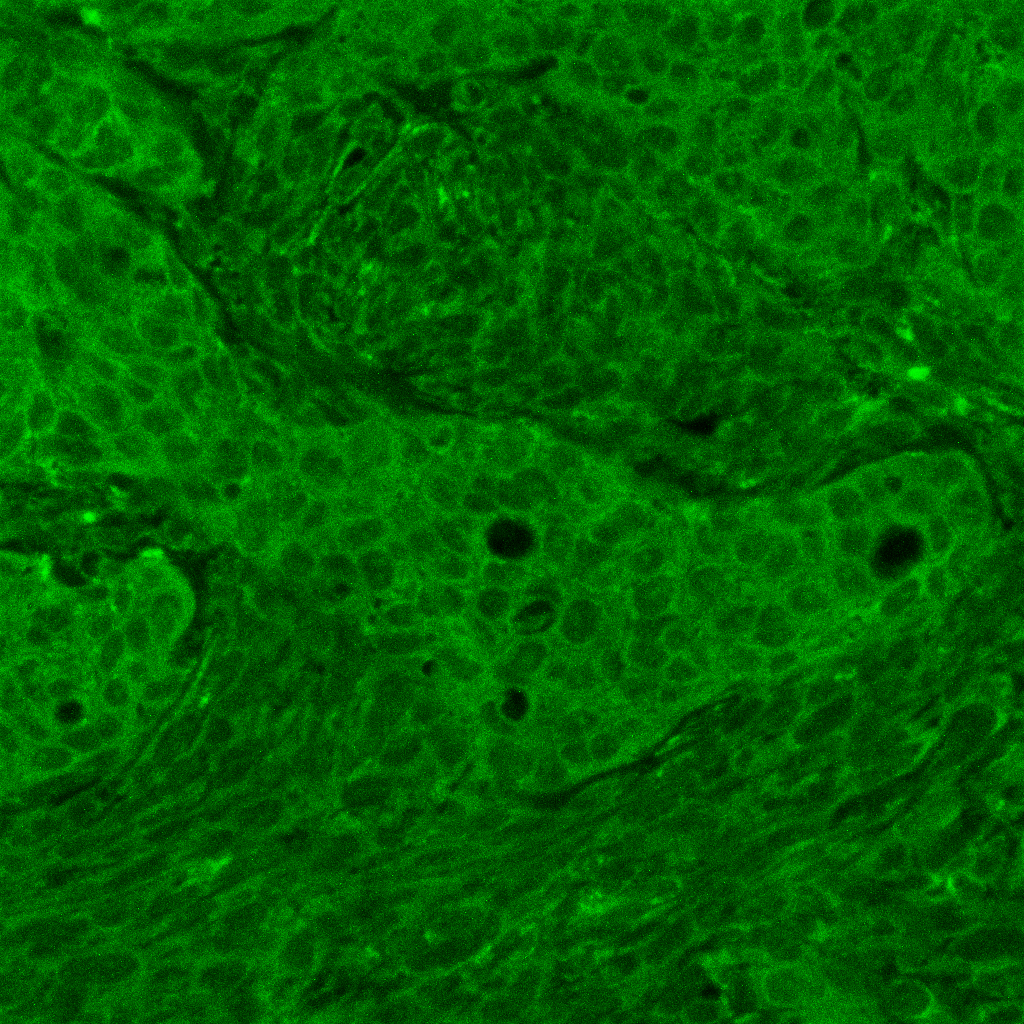

Supplement: Supplementary file 10 — Source data Fig. 6 [file 44319_2026_751_MOESM10_ESM.zip › Raw_data_Figure 6/Figure 6E/Tumor WT GFP de PDGF.tif]

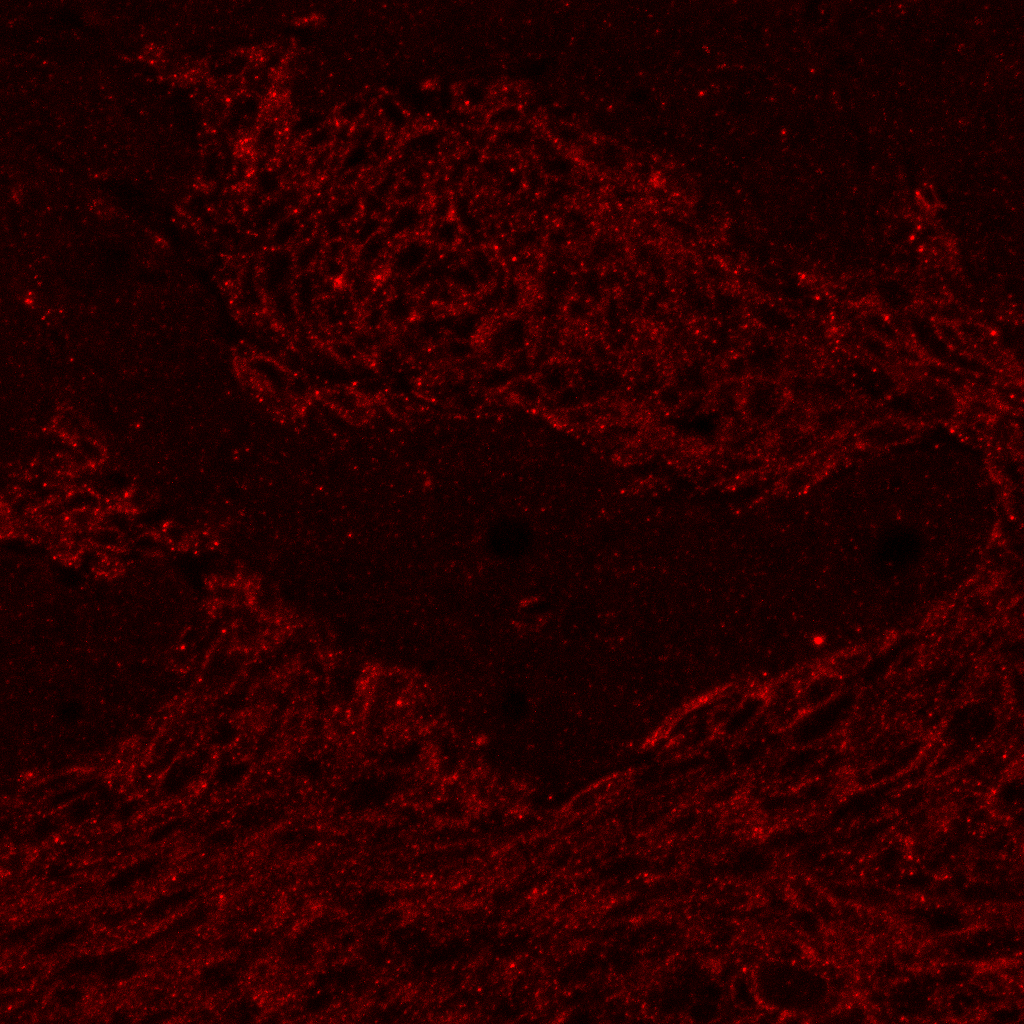

Supplement: Supplementary file 10 — Source data Fig. 6 [file 44319_2026_751_MOESM10_ESM.zip › Raw_data_Figure 6/Figure 6E/Tumor WT pdgfr.tif]

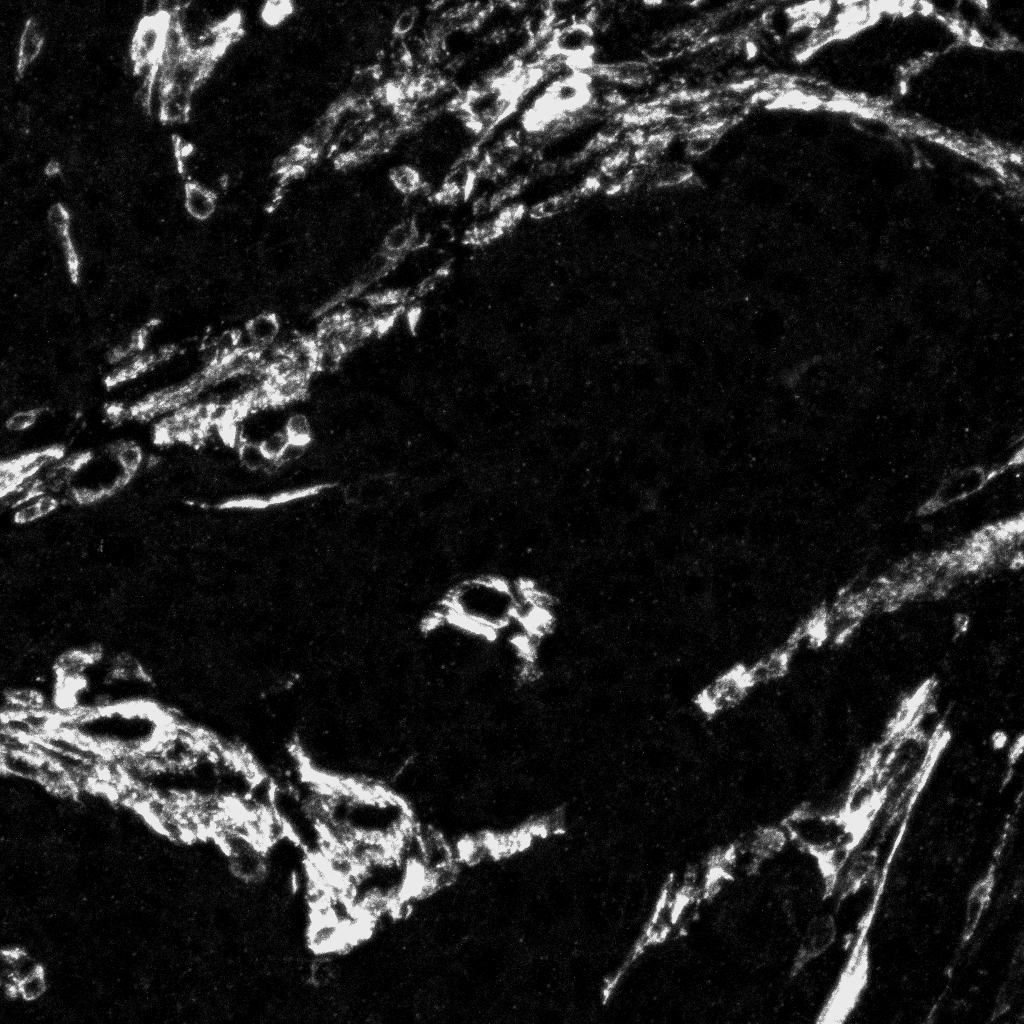

Supplement: Supplementary file 10 — Source data Fig. 6 [file 44319_2026_751_MOESM10_ESM.zip › Raw_data_Figure 6/Figure 6E/Tumor WT SMA.tif]

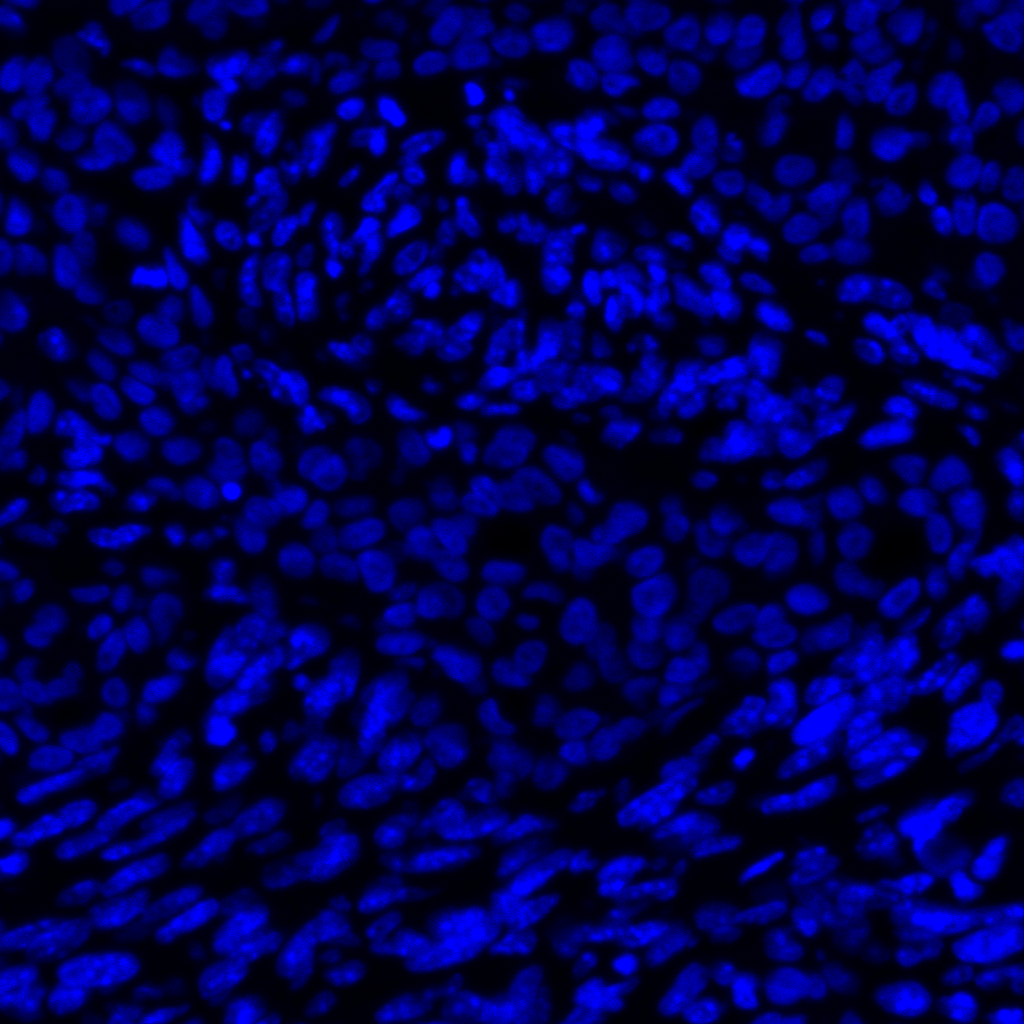

Supplement: Supplementary file 10 — Source data Fig. 6 [file 44319_2026_751_MOESM10_ESM.zip › Raw_data_Figure 6/Figure 6E/Tumor WT de pdgfr nuclei.tif]

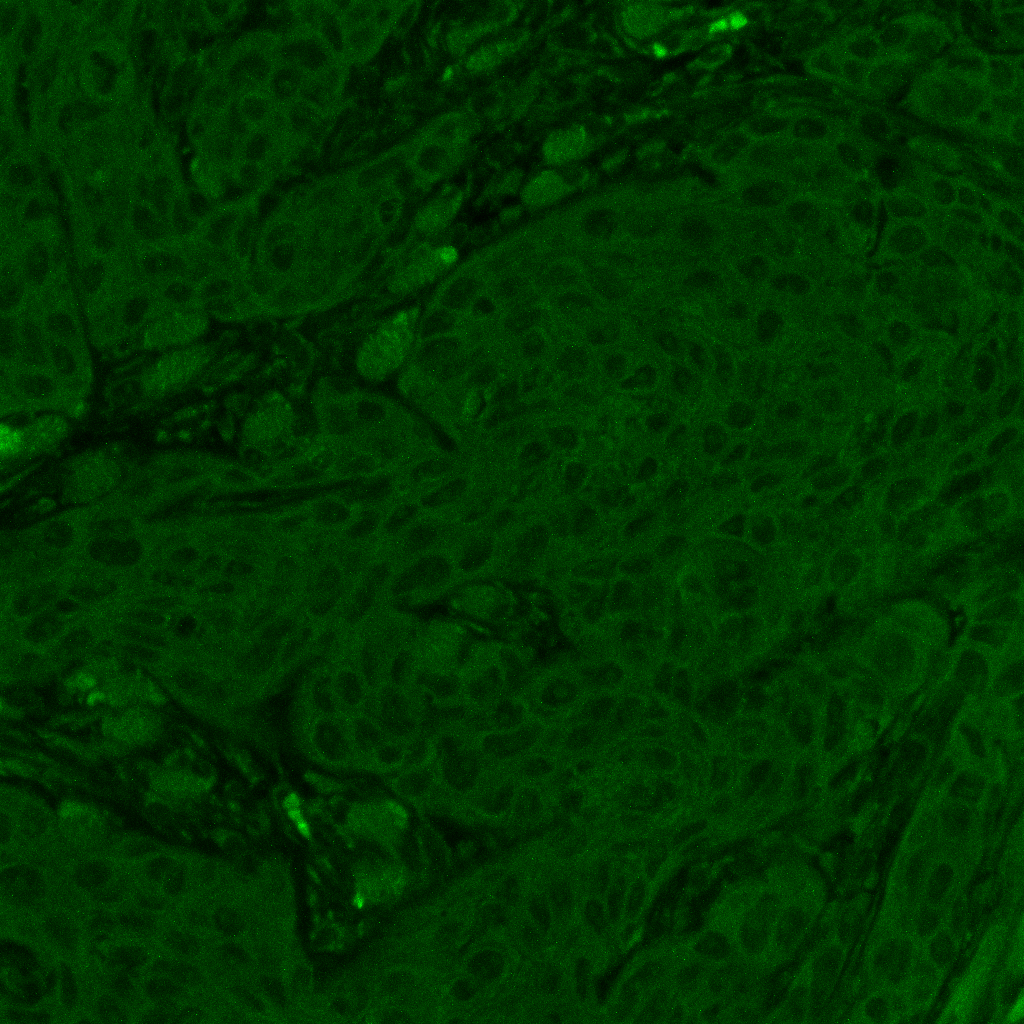

Supplement: Supplementary file 10 — Source data Fig. 6 [file 44319_2026_751_MOESM10_ESM.zip › Raw_data_Figure 6/Figure 6E/Tumor WT GFP de cav1.tif]

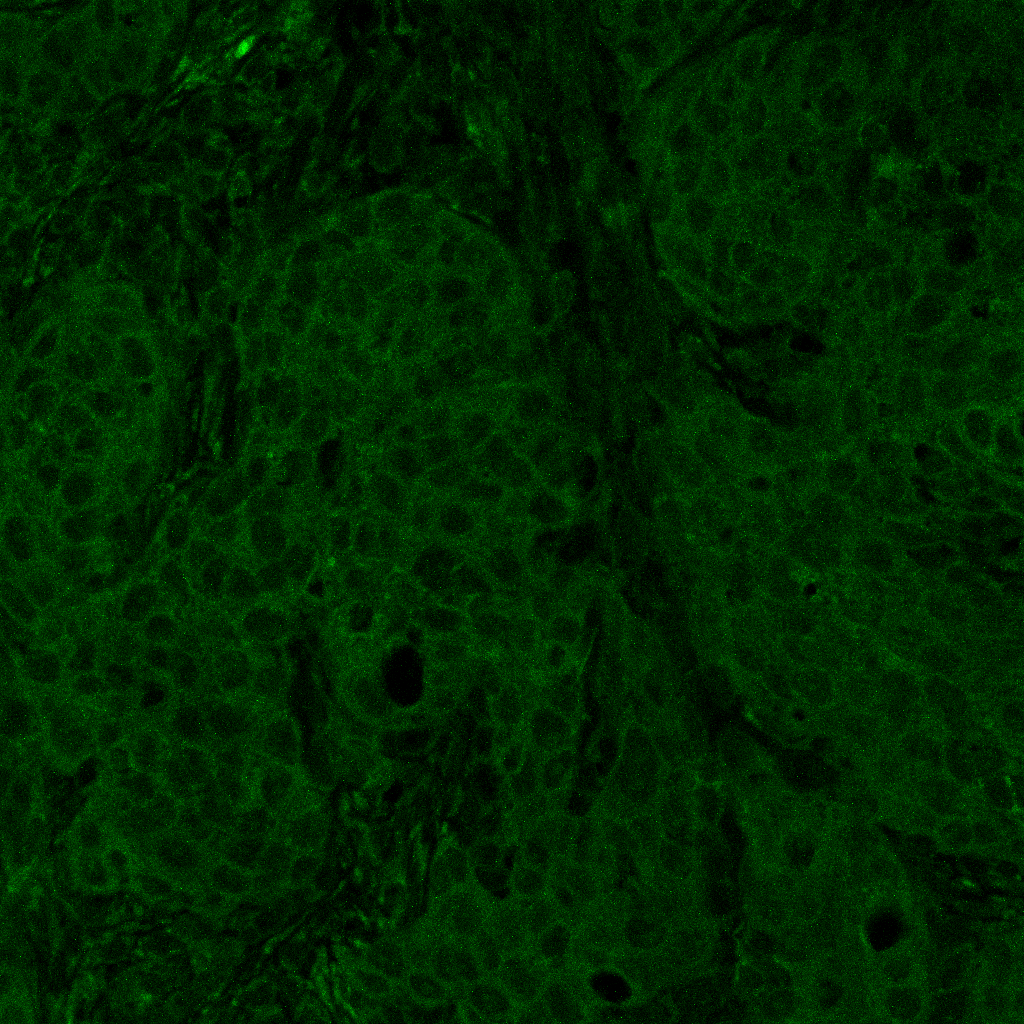

Supplement: Supplementary file 10 — Source data Fig. 6 [file 44319_2026_751_MOESM10_ESM.zip › Raw_data_Figure 6/Figure 6E/Tumor WT GFP de LAMP1.tif]

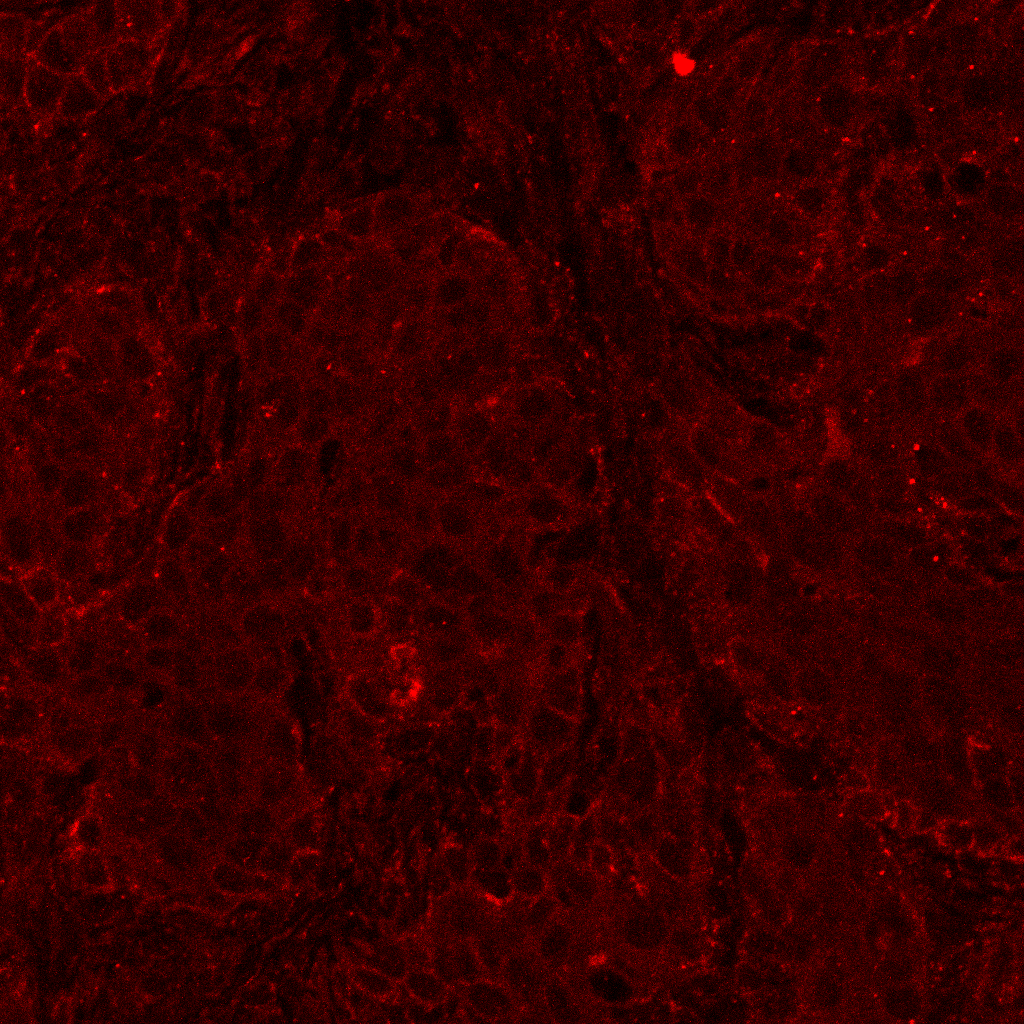

Supplement: Supplementary file 10 — Source data Fig. 6 [file 44319_2026_751_MOESM10_ESM.zip › Raw_data_Figure 6/Figure 6E/Tumor WT LAMP1.tif]

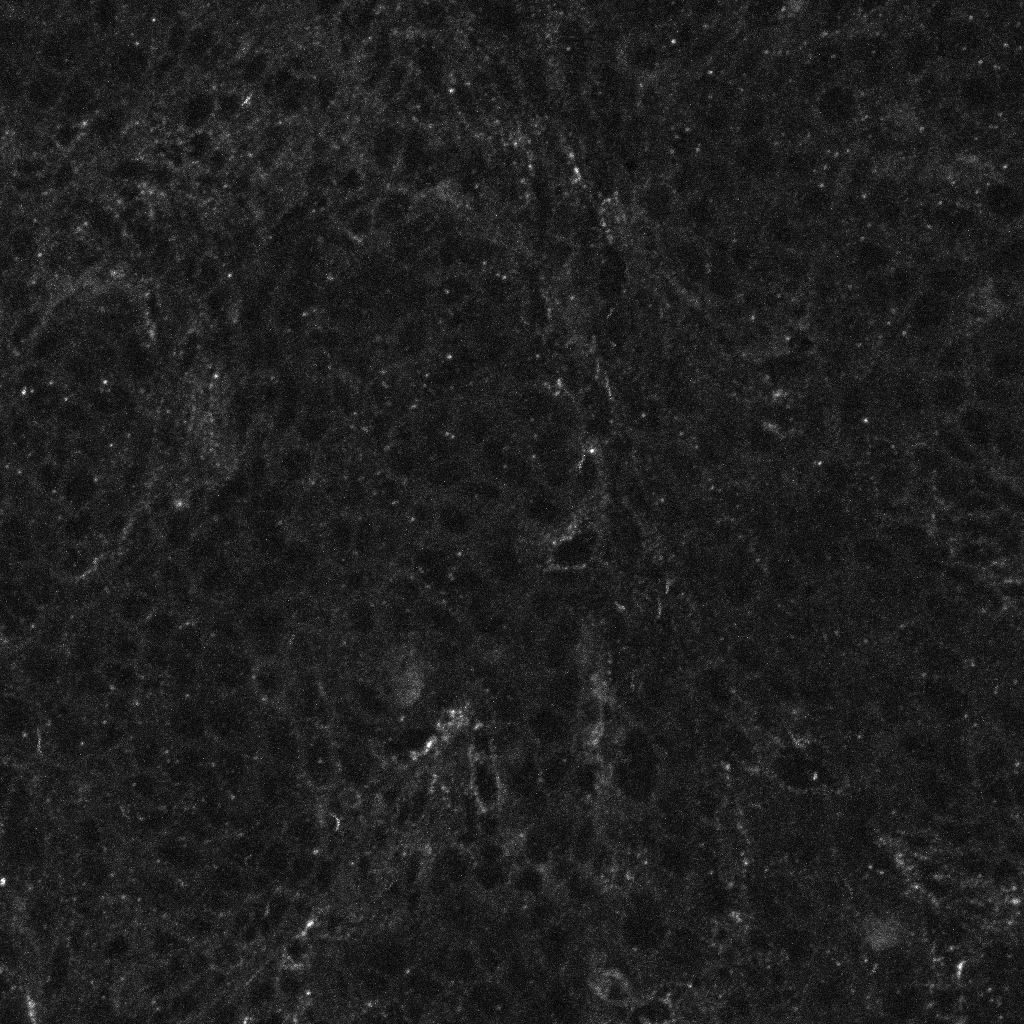

Supplement: Supplementary file 10 — Source data Fig. 6 [file 44319_2026_751_MOESM10_ESM.zip › Raw_data_Figure 6/Figure 6E/Tumor WT PTRF.tif]

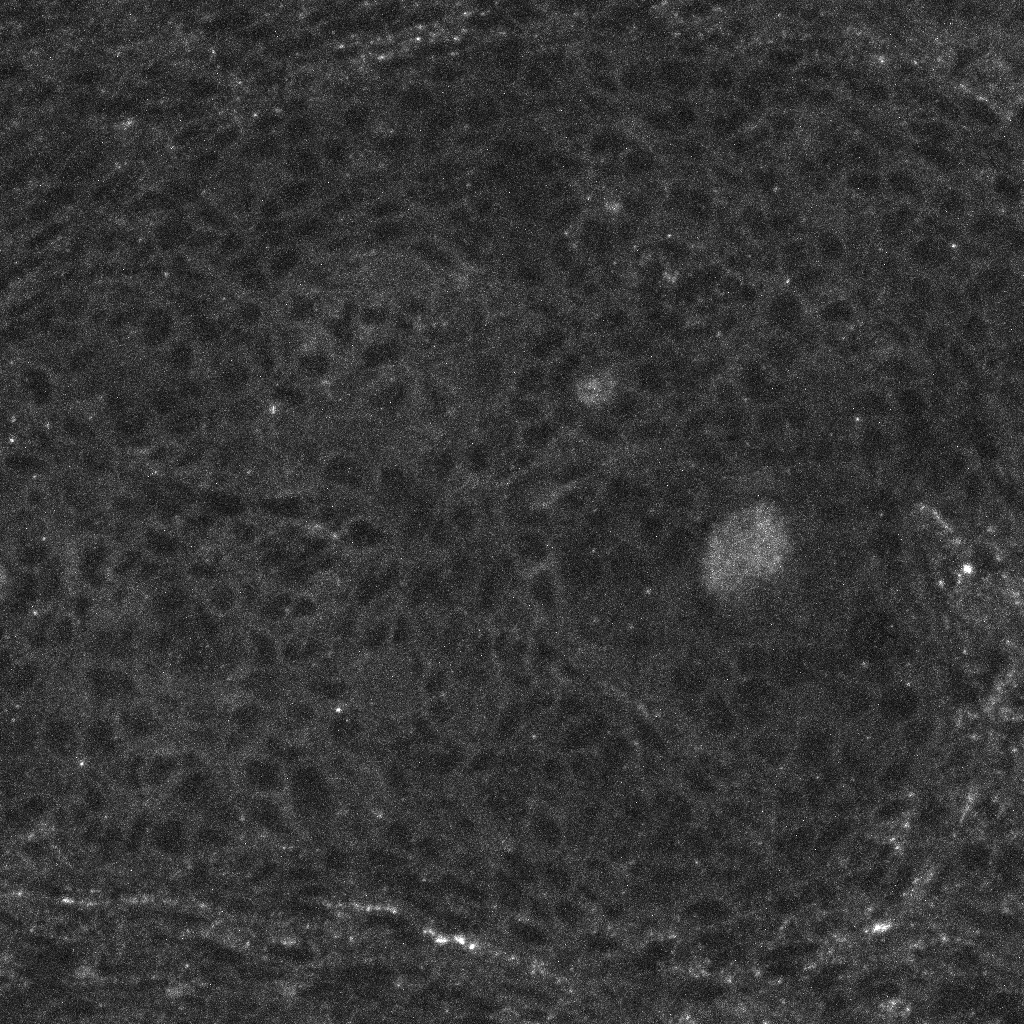

Supplement: Supplementary file 10 — Source data Fig. 6 [file 44319_2026_751_MOESM10_ESM.zip › Raw_data_Figure 6/Figure 6E/Tumor WTcol I..tif]

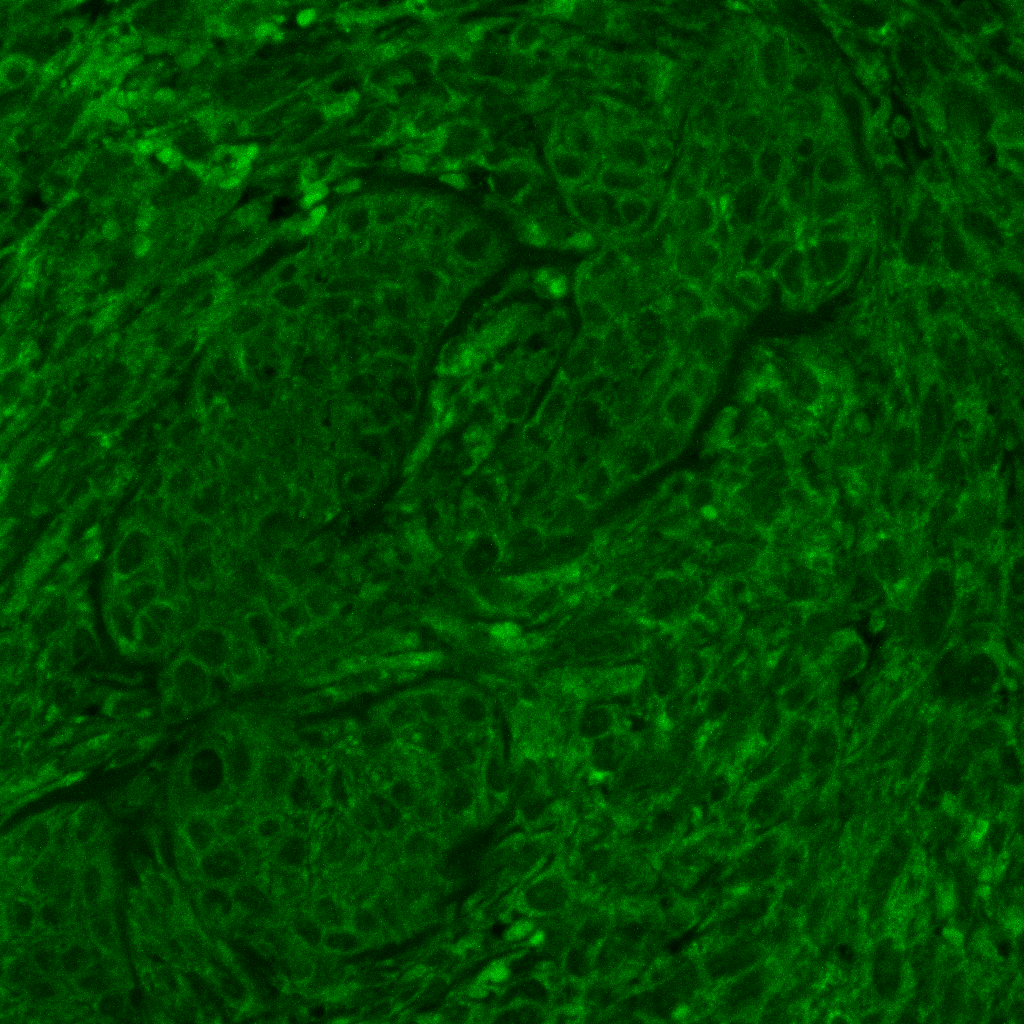

Supplement: Supplementary file 10 — Source data Fig. 6 [file 44319_2026_751_MOESM10_ESM.zip › Raw_data_Figure 6/Figure 6E/TumorKO GFP.tif]

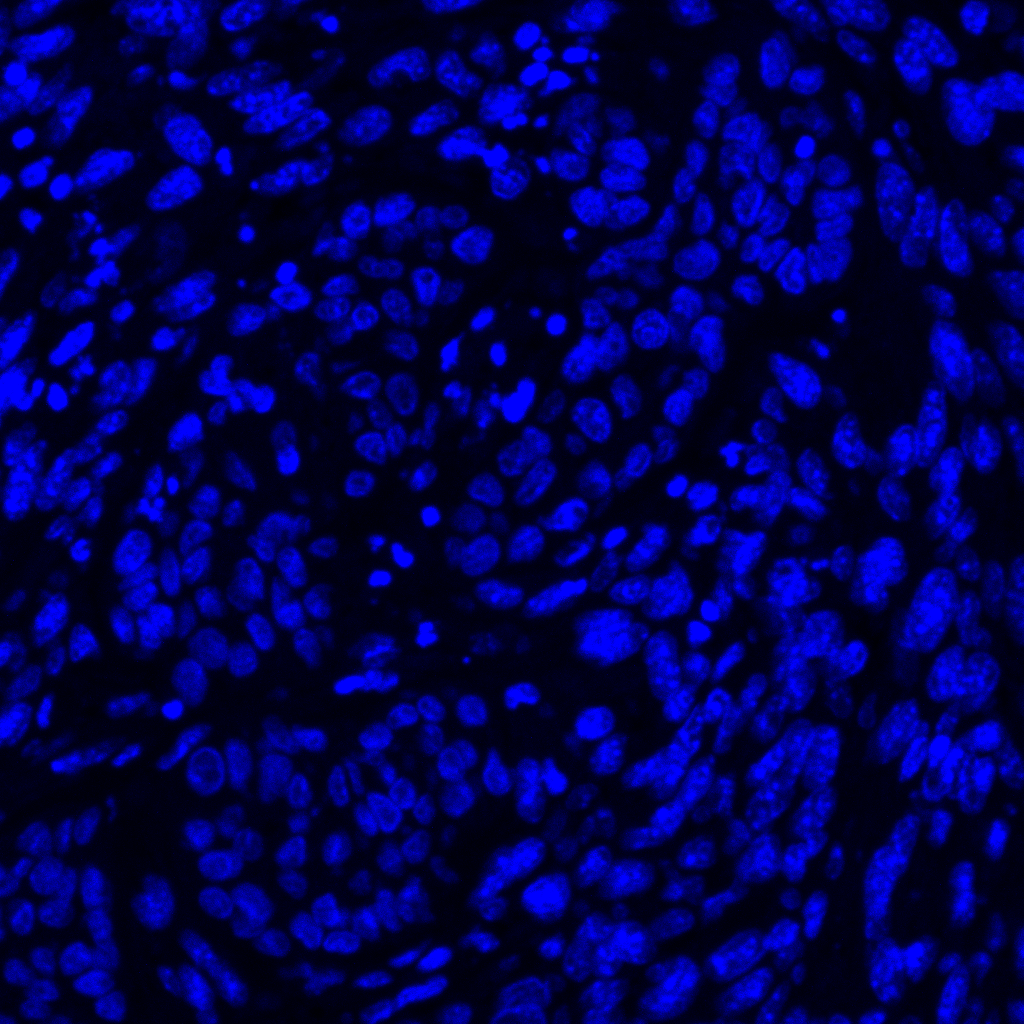

Supplement: Supplementary file 10 — Source data Fig. 6 [file 44319_2026_751_MOESM10_ESM.zip › Raw_data_Figure 6/Figure 6E/TumorKO nuclei.tif]

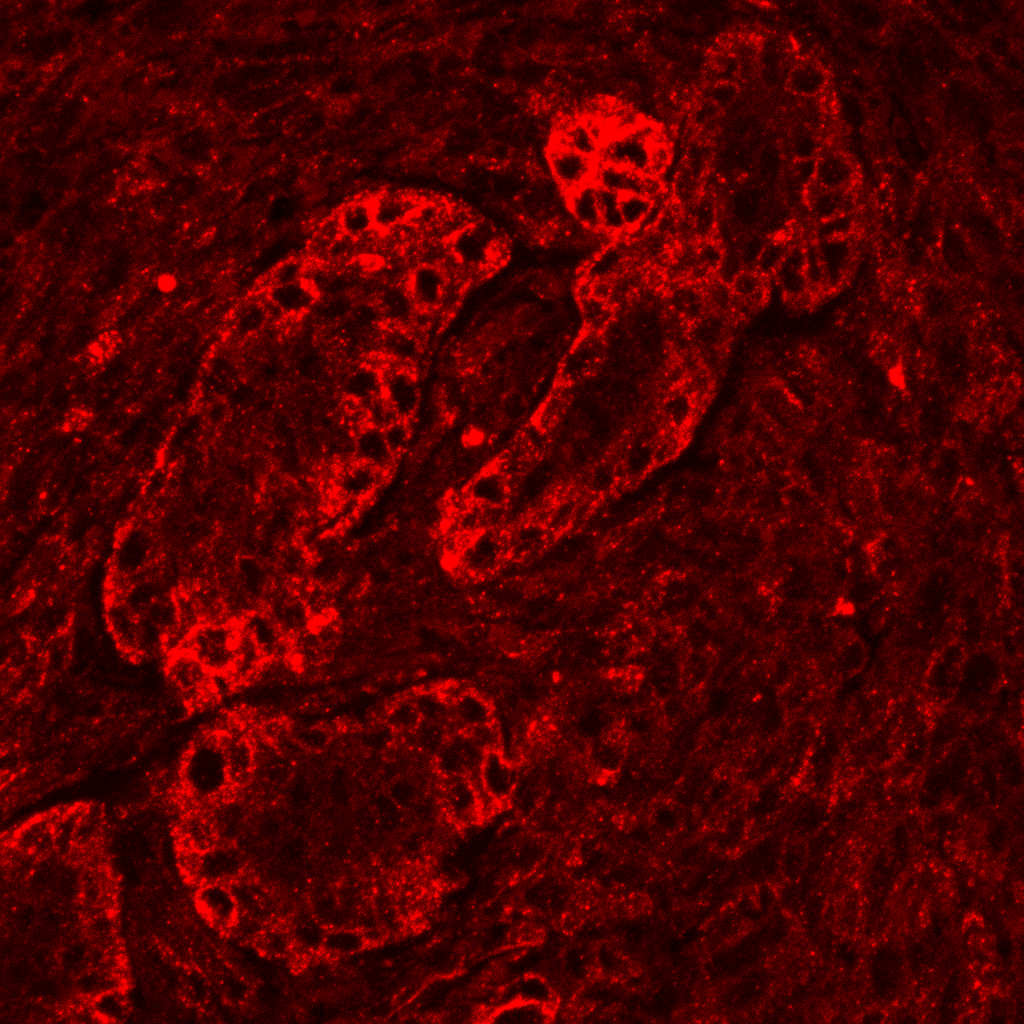

Supplement: Supplementary file 10 — Source data Fig. 6 [file 44319_2026_751_MOESM10_ESM.zip › Raw_data_Figure 6/Figure 6E/TumorKO cav1.tif]

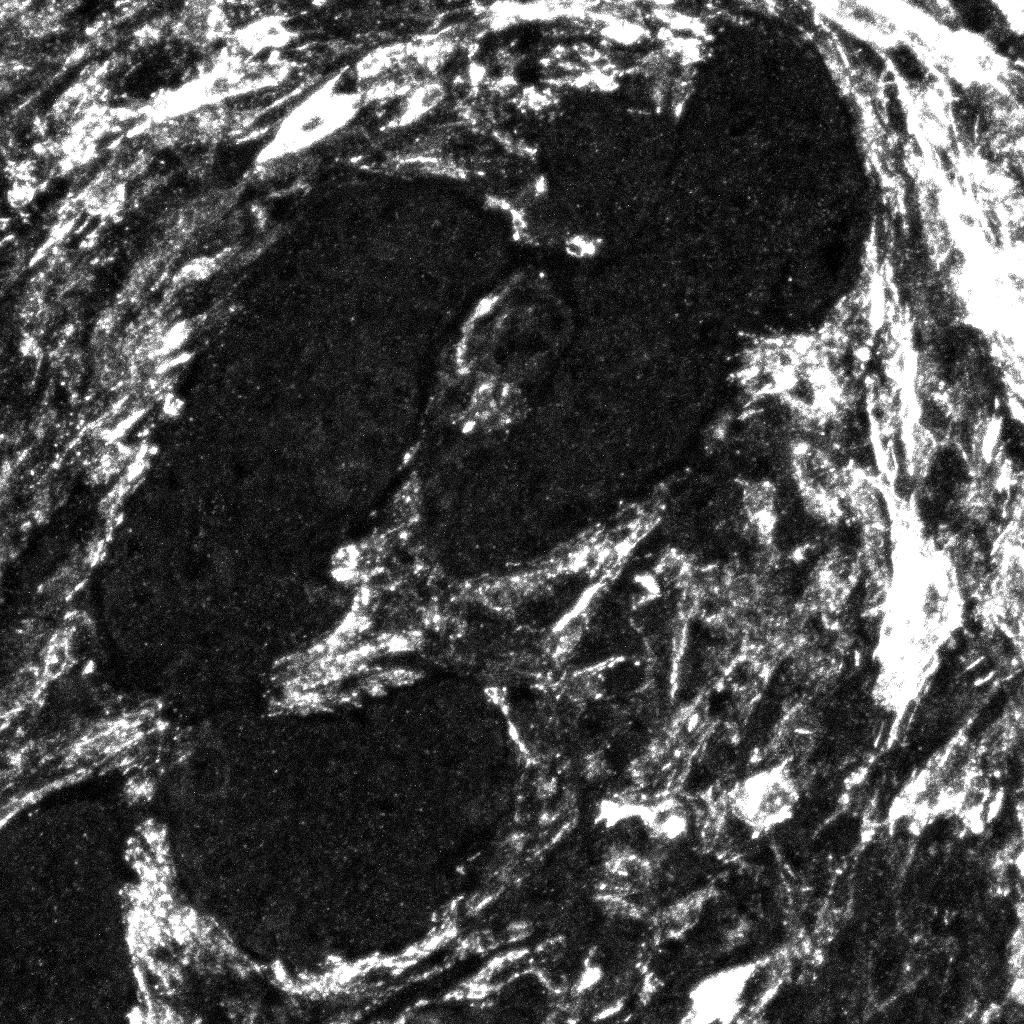

Supplement: Supplementary file 10 — Source data Fig. 6 [file 44319_2026_751_MOESM10_ESM.zip › Raw_data_Figure 6/Figure 6E/TumorKO SMA.tif]

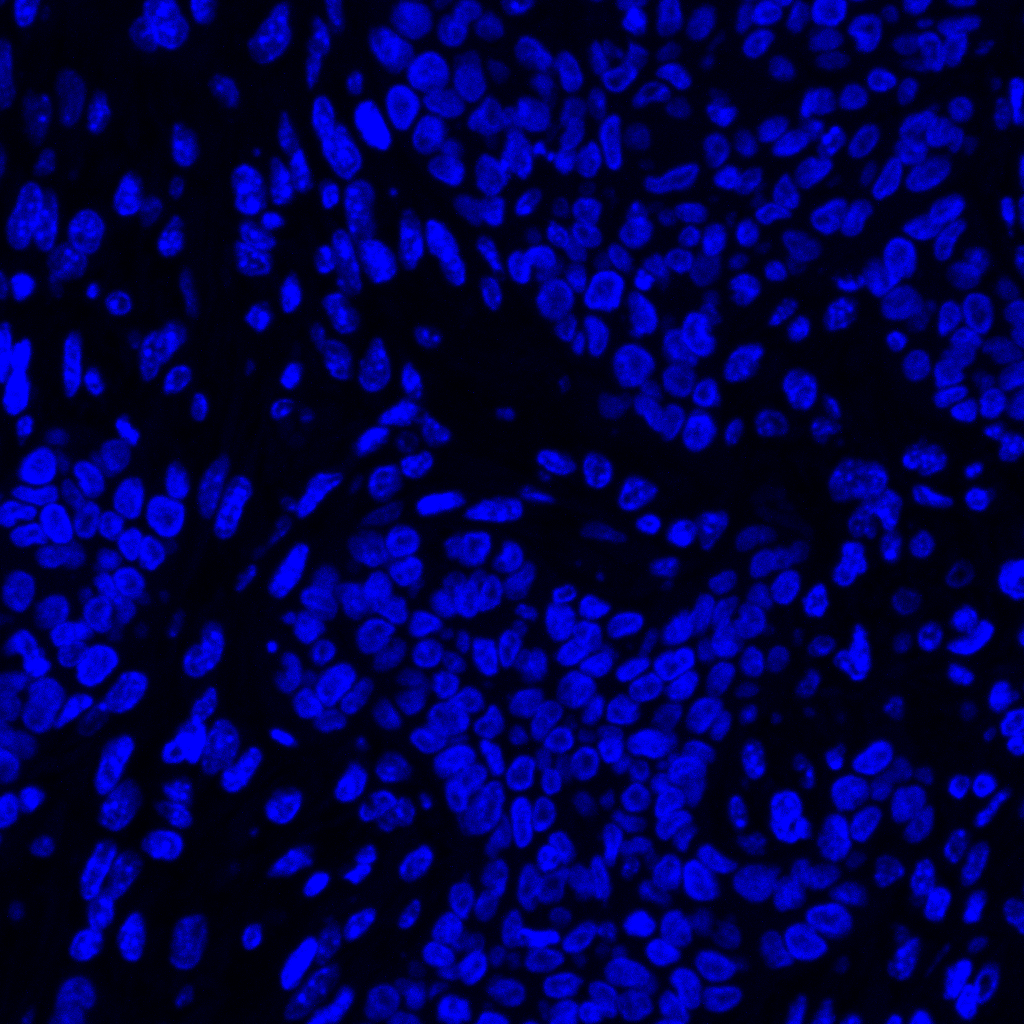

Supplement: Supplementary file 10 — Source data Fig. 6 [file 44319_2026_751_MOESM10_ESM.zip › Raw_data_Figure 6/Figure 6E/ ́Tumor GqKO de LAMP1 nuclei.tif]

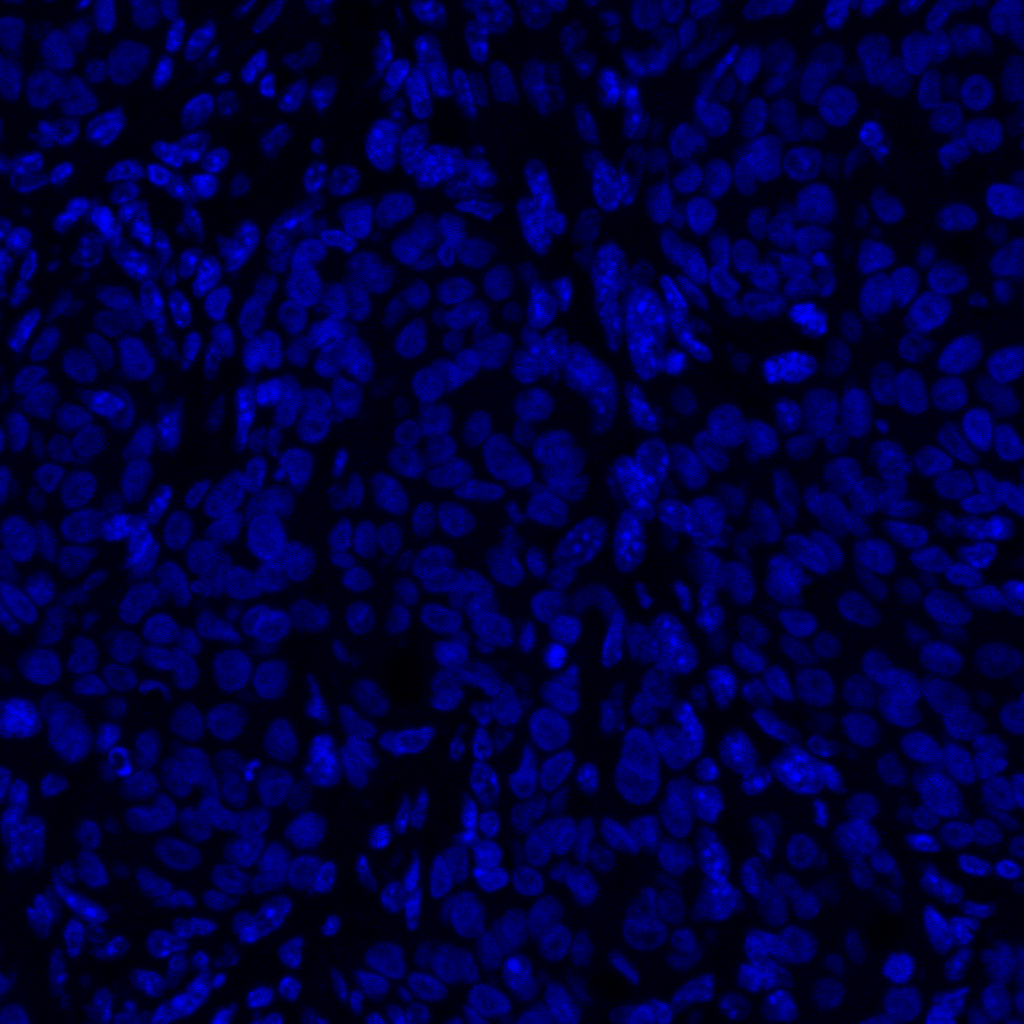

Supplement: Supplementary file 10 — Source data Fig. 6 [file 44319_2026_751_MOESM10_ESM.zip › Raw_data_Figure 6/Figure 6E/ ́Tumor WT de LAMP1 nuclei.tif]

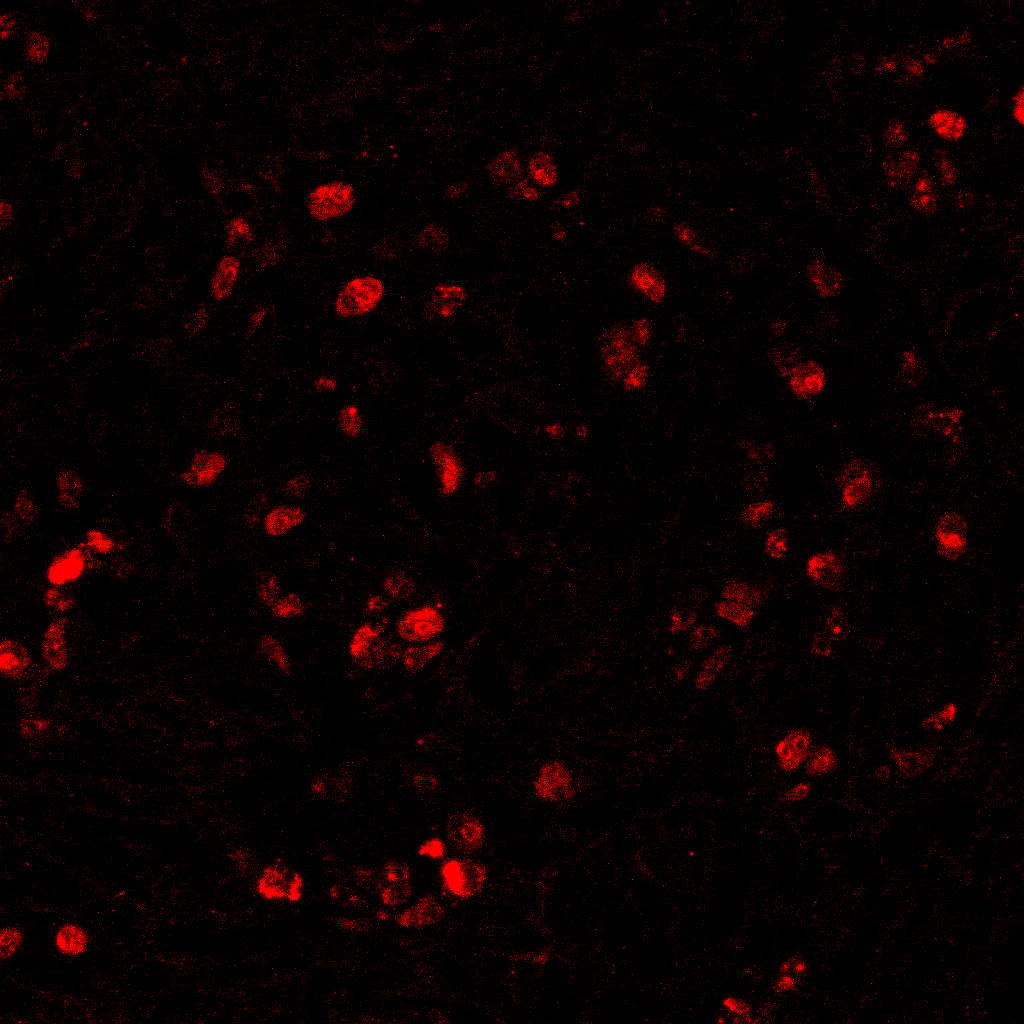

Supplement: Supplementary file 10 — Source data Fig. 6 [file 44319_2026_751_MOESM10_ESM.zip › Raw_data_Figure 6/Figure 6F/GqKO tumor Ki67.tif]

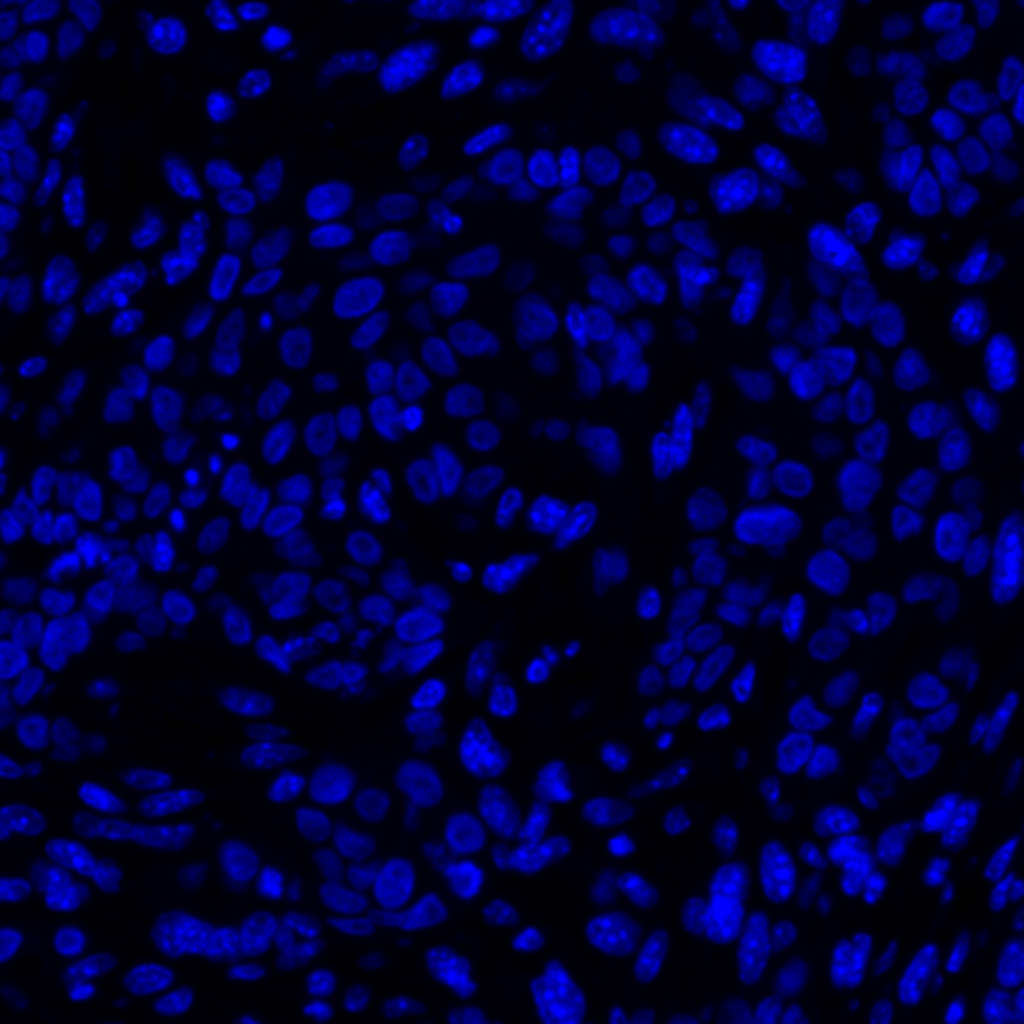

Supplement: Supplementary file 10 — Source data Fig. 6 [file 44319_2026_751_MOESM10_ESM.zip › Raw_data_Figure 6/Figure 6F/GqKO tumor nuclei de ki67.tif]

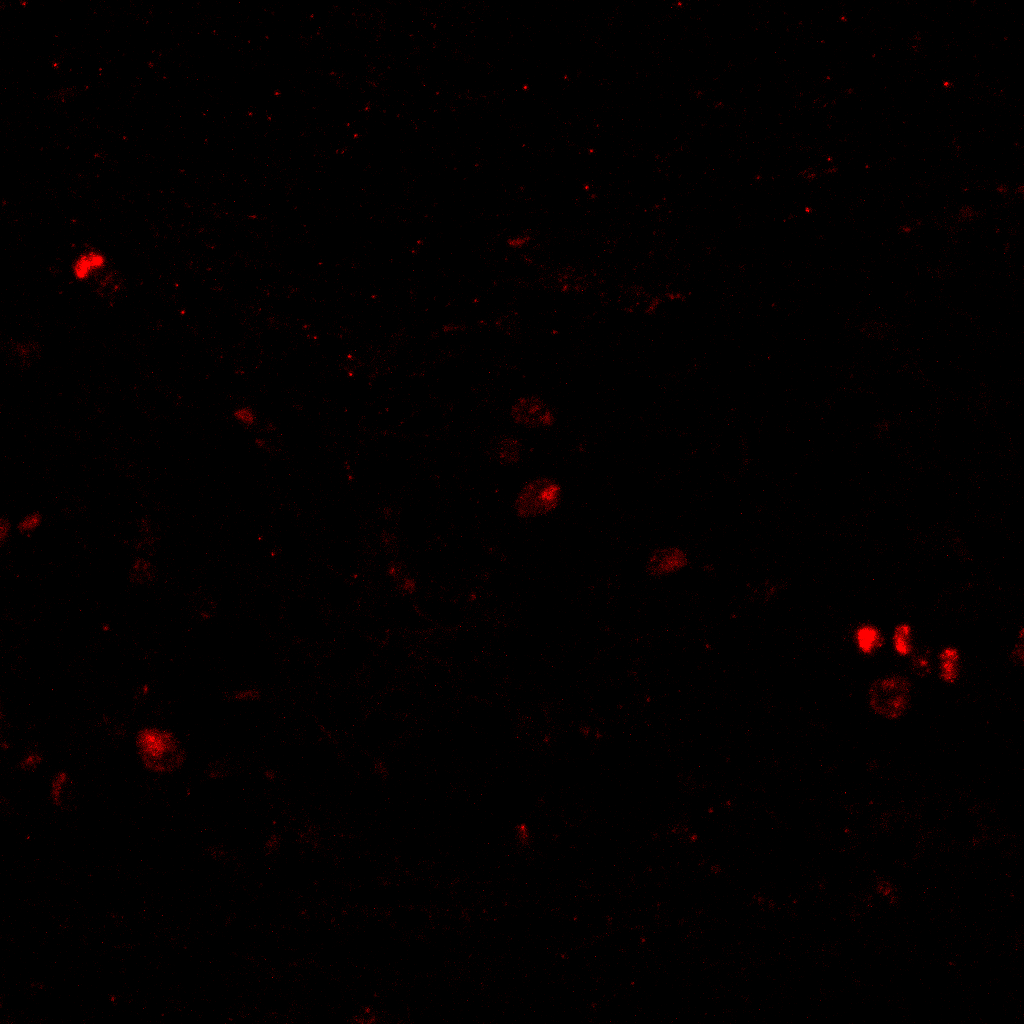

Supplement: Supplementary file 10 — Source data Fig. 6 [file 44319_2026_751_MOESM10_ESM.zip › Raw_data_Figure 6/Figure 6F/WT tumor Ki67.tif]

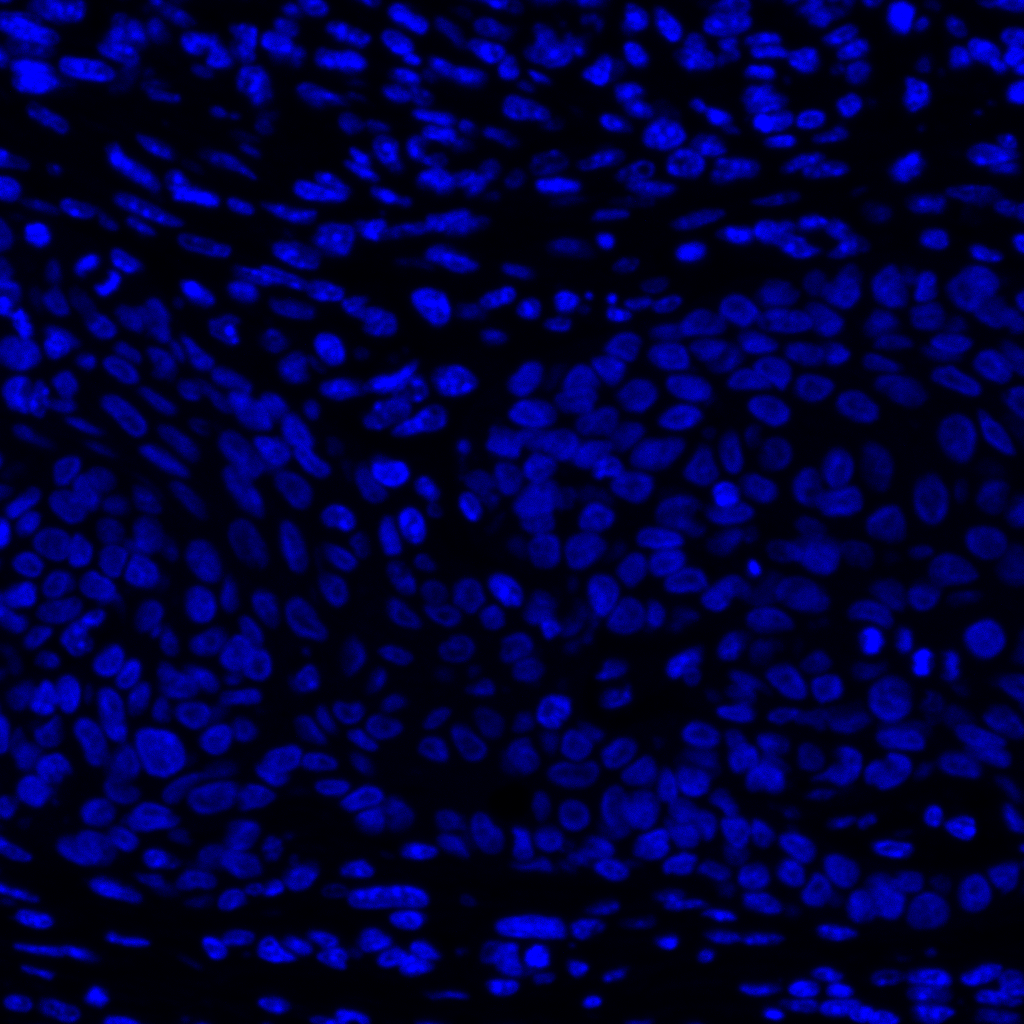

Supplement: Supplementary file 10 — Source data Fig. 6 [file 44319_2026_751_MOESM10_ESM.zip › Raw_data_Figure 6/Figure 6F/WT tumor nuclei de ki67.tif]

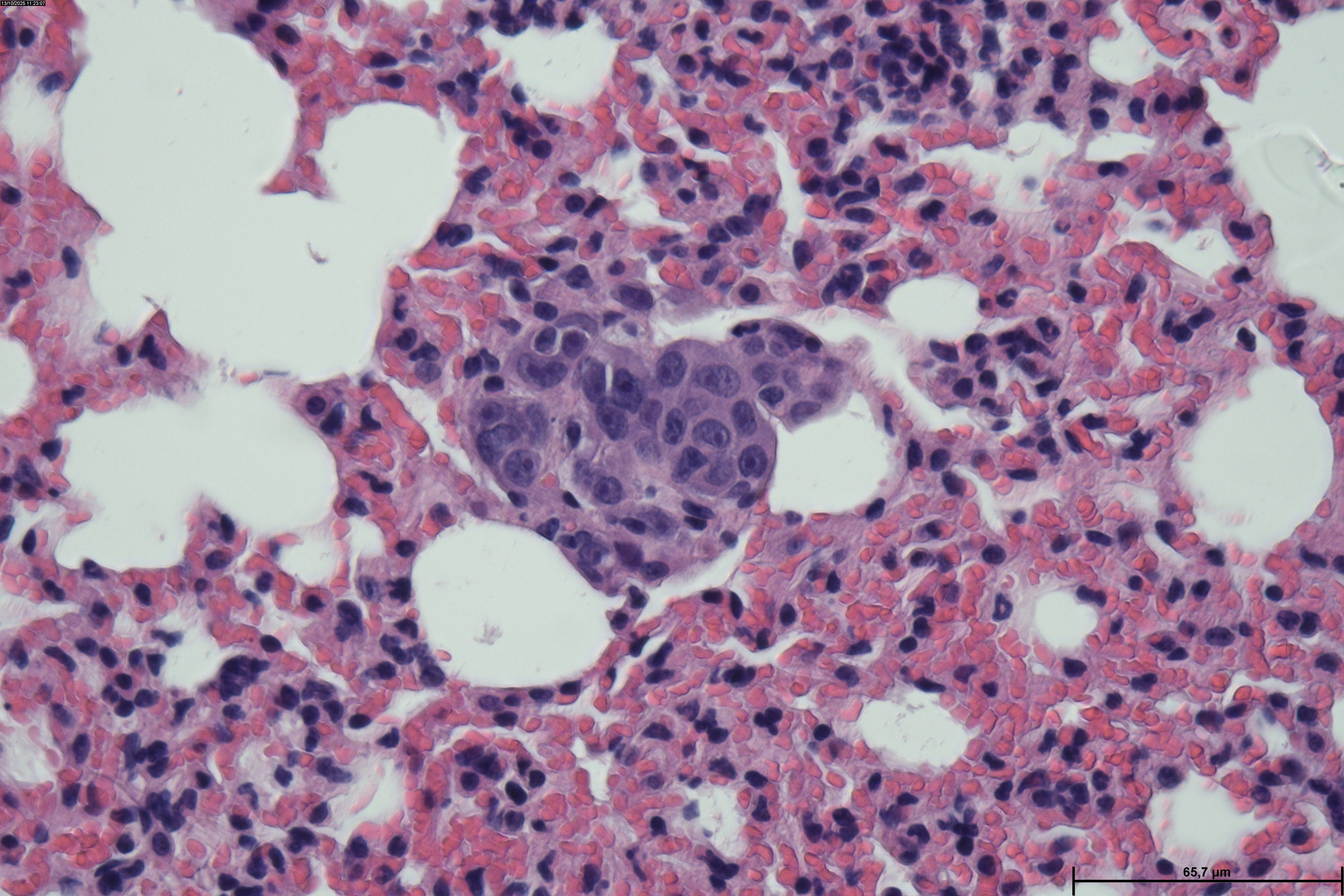

Supplement: Supplementary file 10 — Source data Fig. 6 [file 44319_2026_751_MOESM10_ESM.zip › Raw_data_Figure 6/Figure 6G/105_6_40 (2).tif]

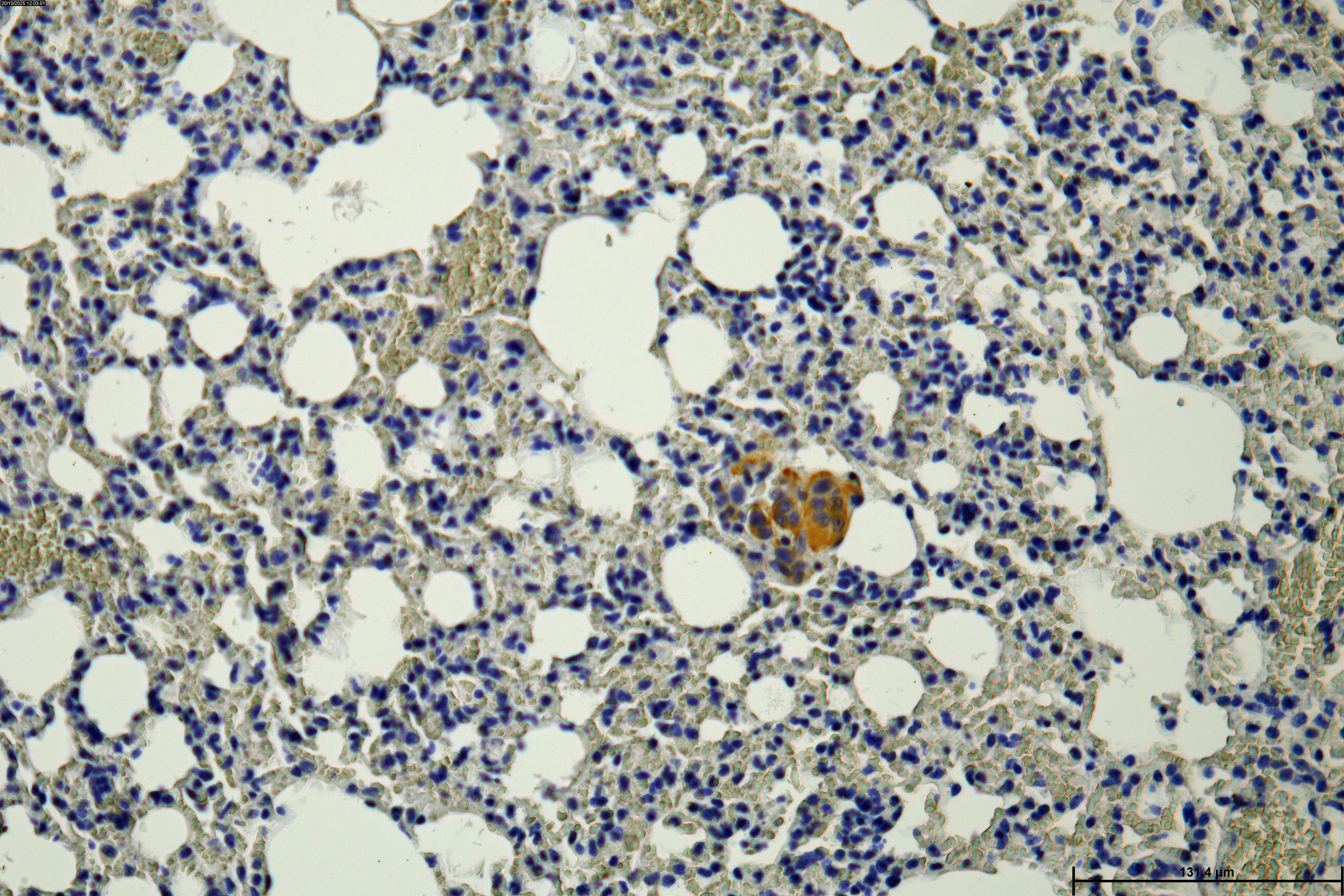

Supplement: Supplementary file 10 — Source data Fig. 6 [file 44319_2026_751_MOESM10_ESM.zip › Raw_data_Figure 6/Figure 6G/105_7_CK5_20 (2).tif]
