# Supplementary material for: Loss of Gαq reshapes fibroblast traits and drives tumor-stroma remodeling in oral cancer progression
Source: EMBO Rep. 2026 Apr 10;27(10):2639–74. doi: 10.1038/s44319-026-00751-2 (PMC13219523; doi:10.1038/s44319-026-00751-2)
Supplement: Supplementary file 9 — Source data Fig. 5 [file 44319_2026_751_MOESM9_ESM.zip › Raw_data_Figure 5/Figure 5E/raw_blots_5E.pptx]

## Slide 1
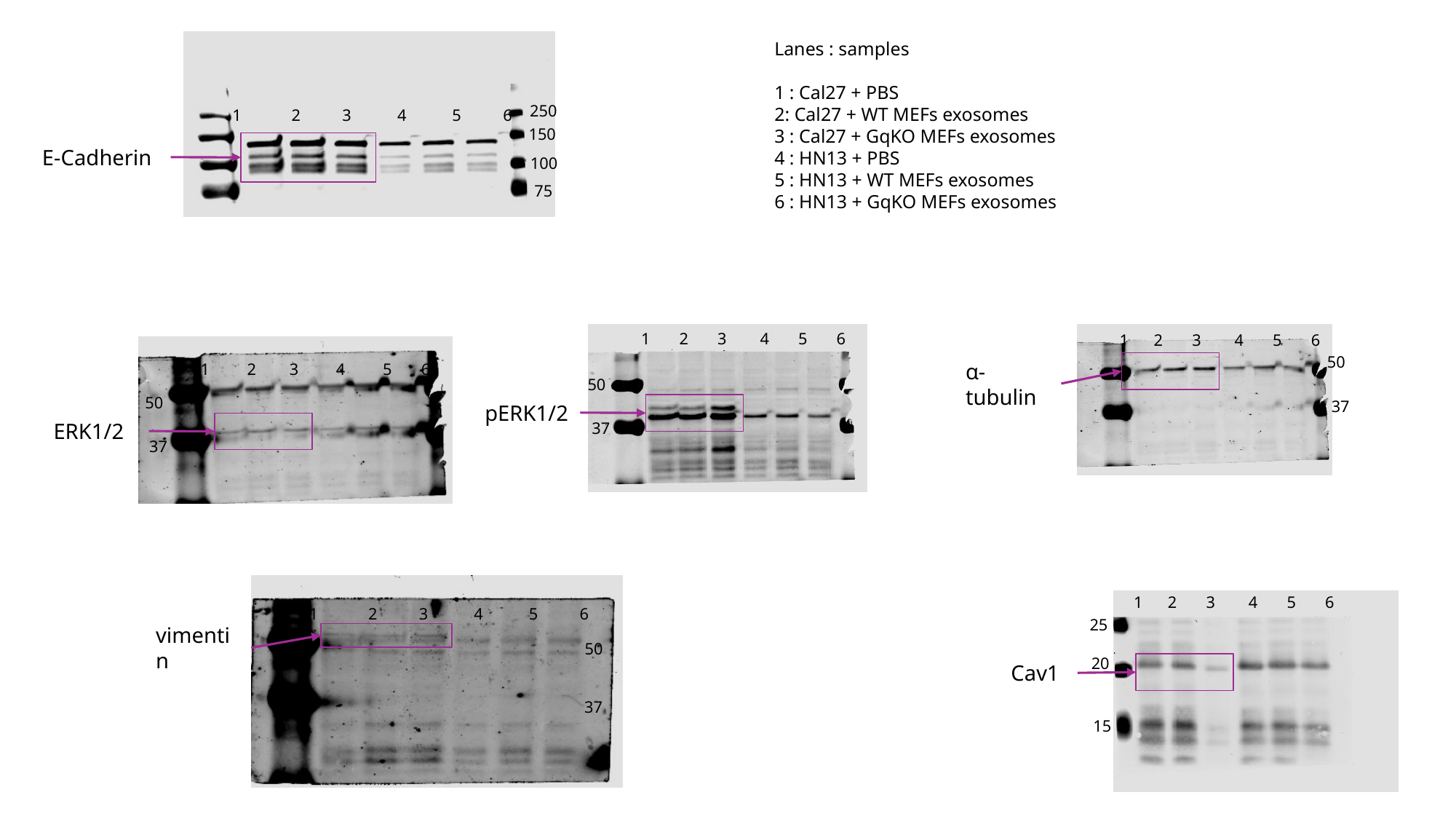

Lanes : samples
1 : Cal27 + PBS
2: Cal27 + WT MEFs exosomes
3 : Cal27 + GqKO MEFs exosomes
4 : HN13 + PBS
5 : HN13 + WT MEFs exosomes
6 : HN13 + GqKO MEFs exosomes
1 2 3 4 5 6
250
150
E-Cadherin
100
75
1 2 3 4 5 6
 1 2 3 4 5 6
1 2 3 4 5 6
50
α-tubulin
50
50
37
pERK1/2
ERK1/2
37
37
 1 2 3 4 5 6
1 2 3 4 5 6
25
vimentin
50
20
Cav1
37
15
