# Supplementary figures and images for: Loss of Gαq reshapes fibroblast traits and drives tumor-stroma remodeling in oral cancer progression (part 4 of 5)
Source: EMBO Rep. 2026 Apr 10;27(10):2639–74. doi: 10.1038/s44319-026-00751-2 (PMC13219523; doi:10.1038/s44319-026-00751-2)

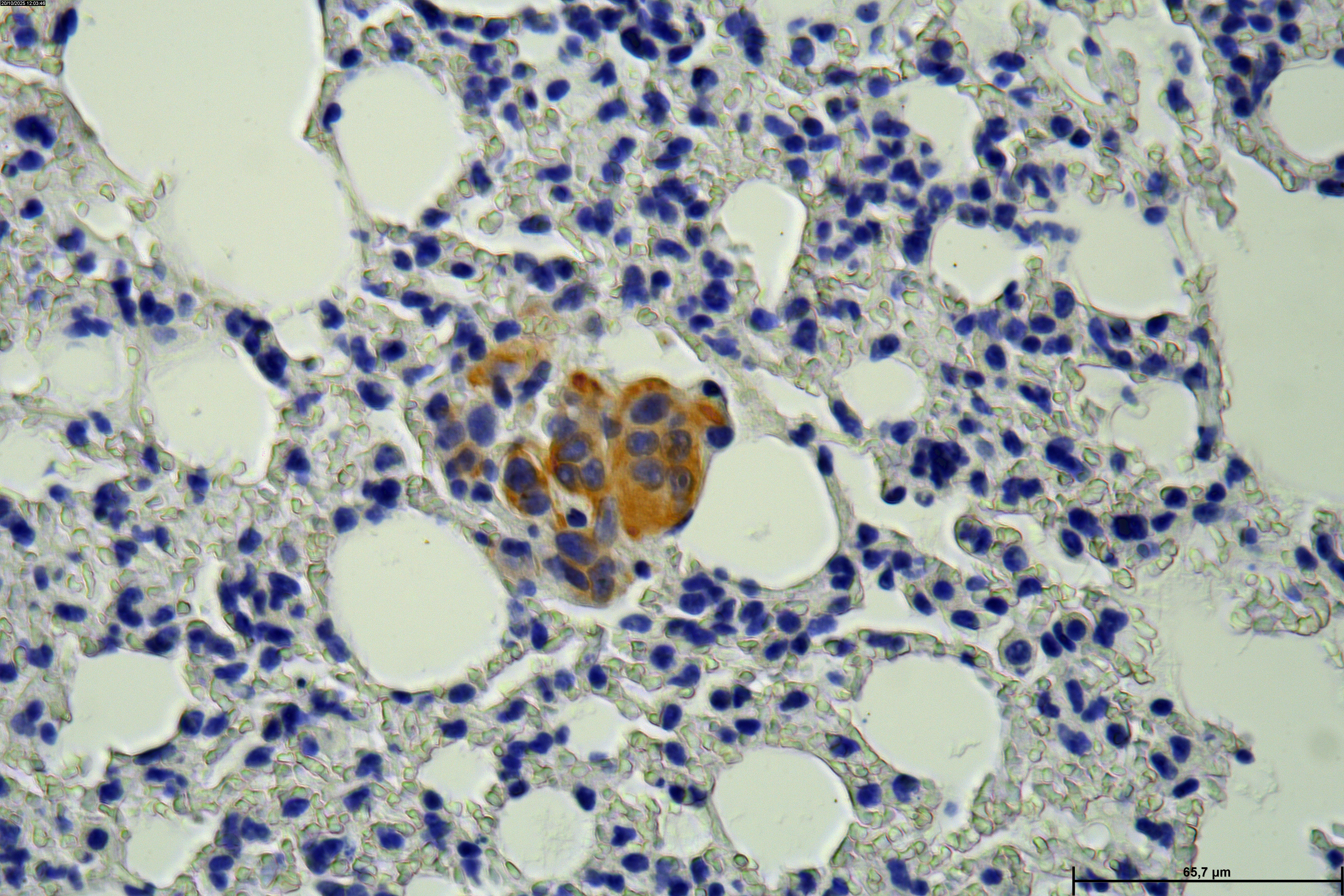

Supplement: Supplementary file 10 — Source data Fig. 6 [file 44319_2026_751_MOESM10_ESM.zip › Raw_data_Figure 6/Figure 6G/105_7_CK5_40 (1).tif]

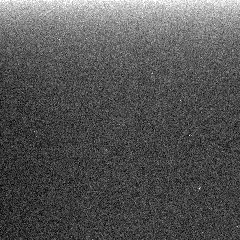

Supplement: Supplementary file 10 — Source data Fig. 6 [file 44319_2026_751_MOESM10_ESM.zip › Raw_data_Figure 6/Figure 6G/INL20251001124046_SEQ/INL20251001124046_001/background.TIF]

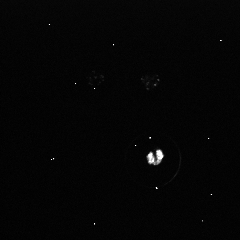

Supplement: Supplementary file 10 — Source data Fig. 6 [file 44319_2026_751_MOESM10_ESM.zip › Raw_data_Figure 6/Figure 6G/INL20251001124046_SEQ/INL20251001124046_001/luminescent.TIF]

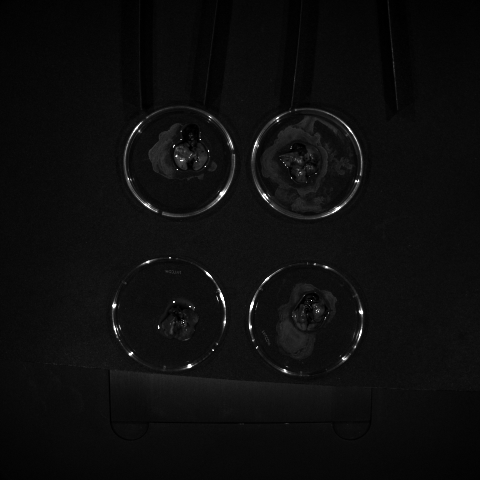

Supplement: Supplementary file 10 — Source data Fig. 6 [file 44319_2026_751_MOESM10_ESM.zip › Raw_data_Figure 6/Figure 6G/INL20251001124046_SEQ/INL20251001124046_001/photograph.TIF]

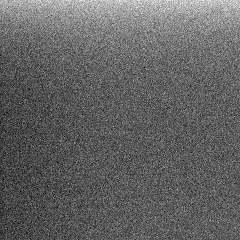

Supplement: Supplementary file 10 — Source data Fig. 6 [file 44319_2026_751_MOESM10_ESM.zip › Raw_data_Figure 6/Figure 6G/INL20251001124046_SEQ/INL20251001124046_001/readbias.TIF]

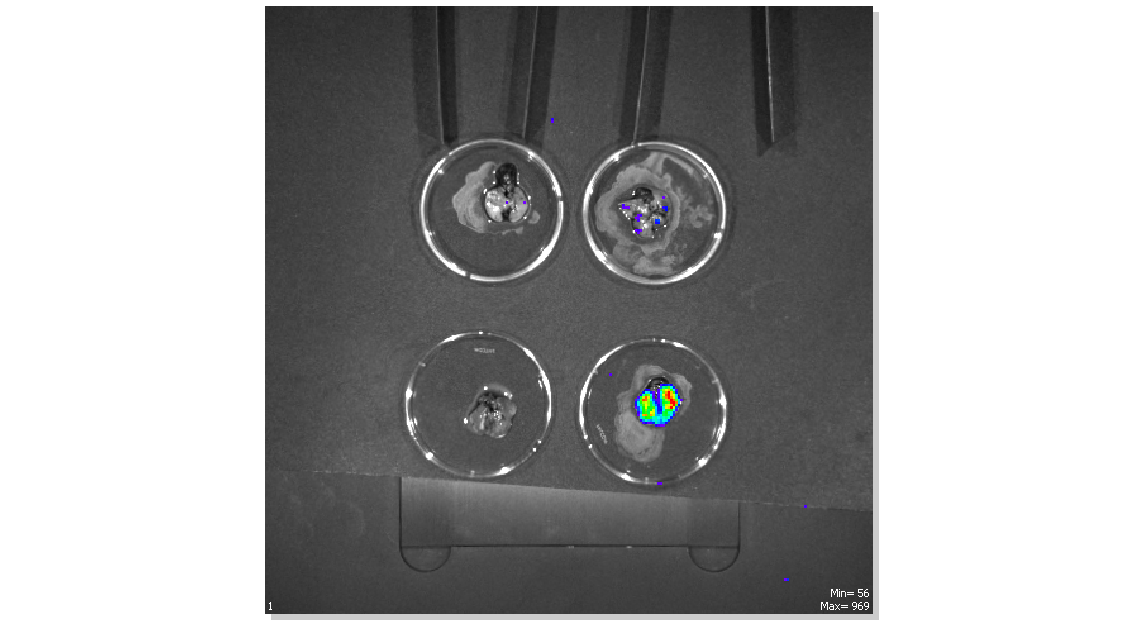

Supplement: Supplementary file 10 — Source data Fig. 6 [file 44319_2026_751_MOESM10_ESM.zip › Raw_data_Figure 6/Figure 6G/INL20251001124046_SEQ/INL20251001124046_SEQ.PNG]

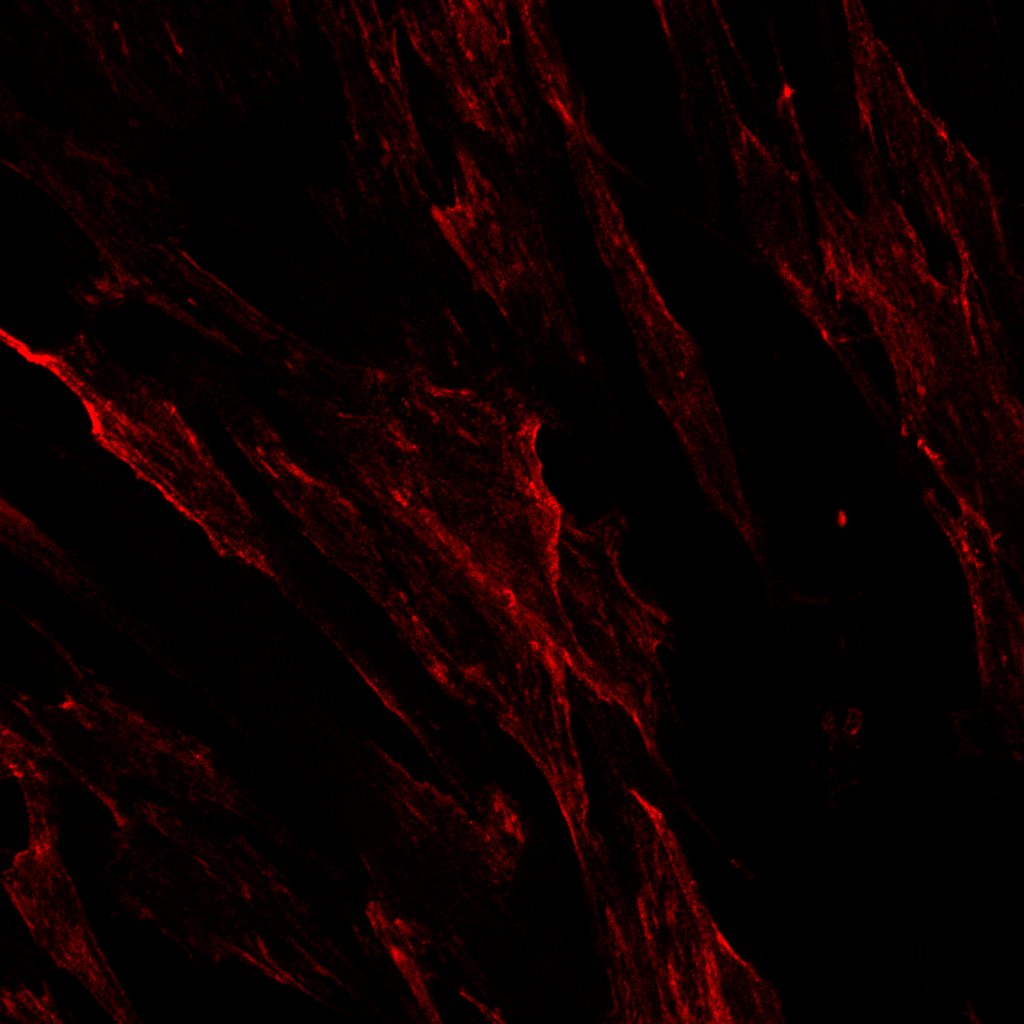

Supplement: Supplementary file 11 — Source data Fig. 7 [file 44319_2026_751_MOESM11_ESM.zip › Raw_data_Figure 7/Figure 7B/C3-NF shCtrl cav1.tif]

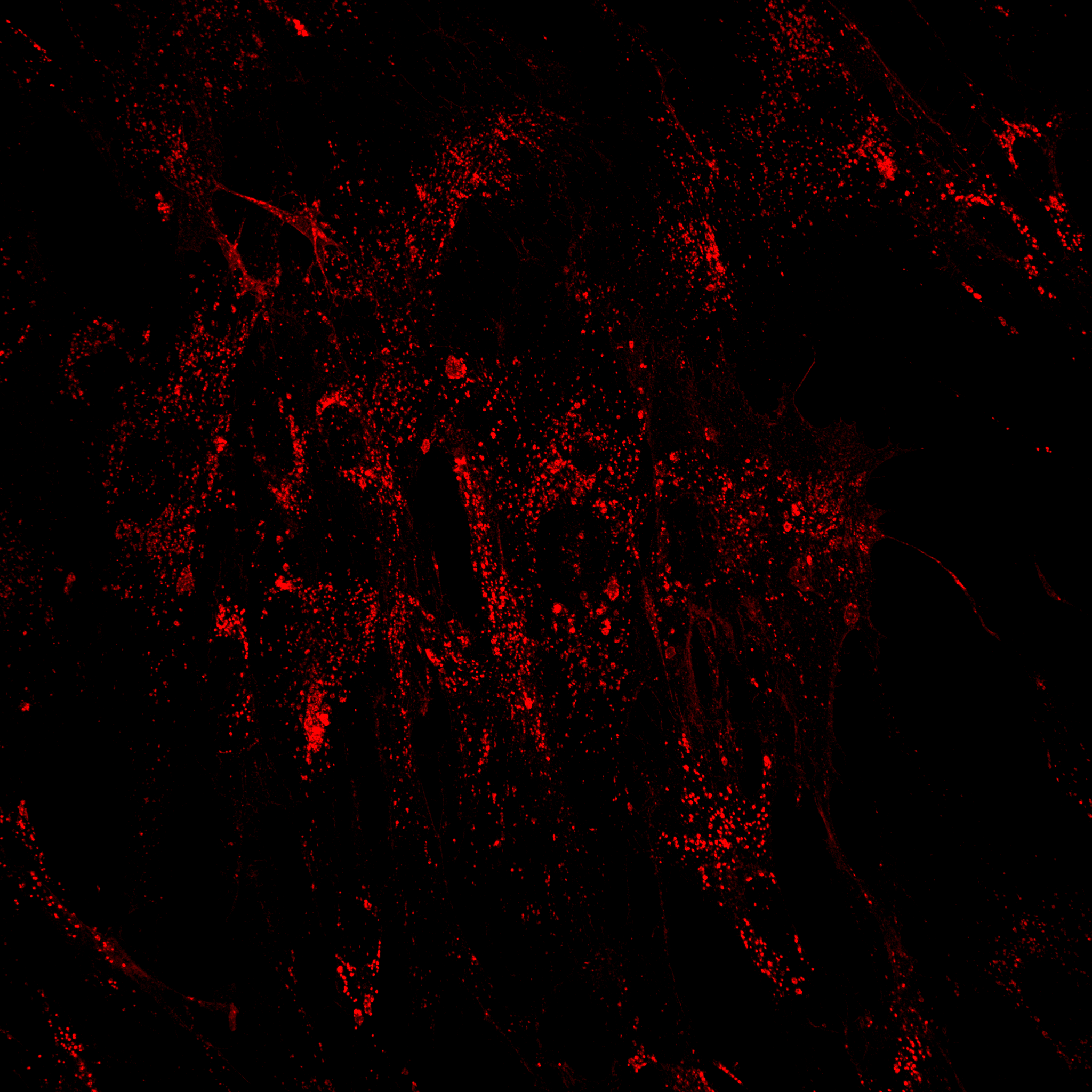

Supplement: Supplementary file 11 — Source data Fig. 7 [file 44319_2026_751_MOESM11_ESM.zip › Raw_data_Figure 7/Figure 7B/C3-NF shCtrl Lamp3.tif]

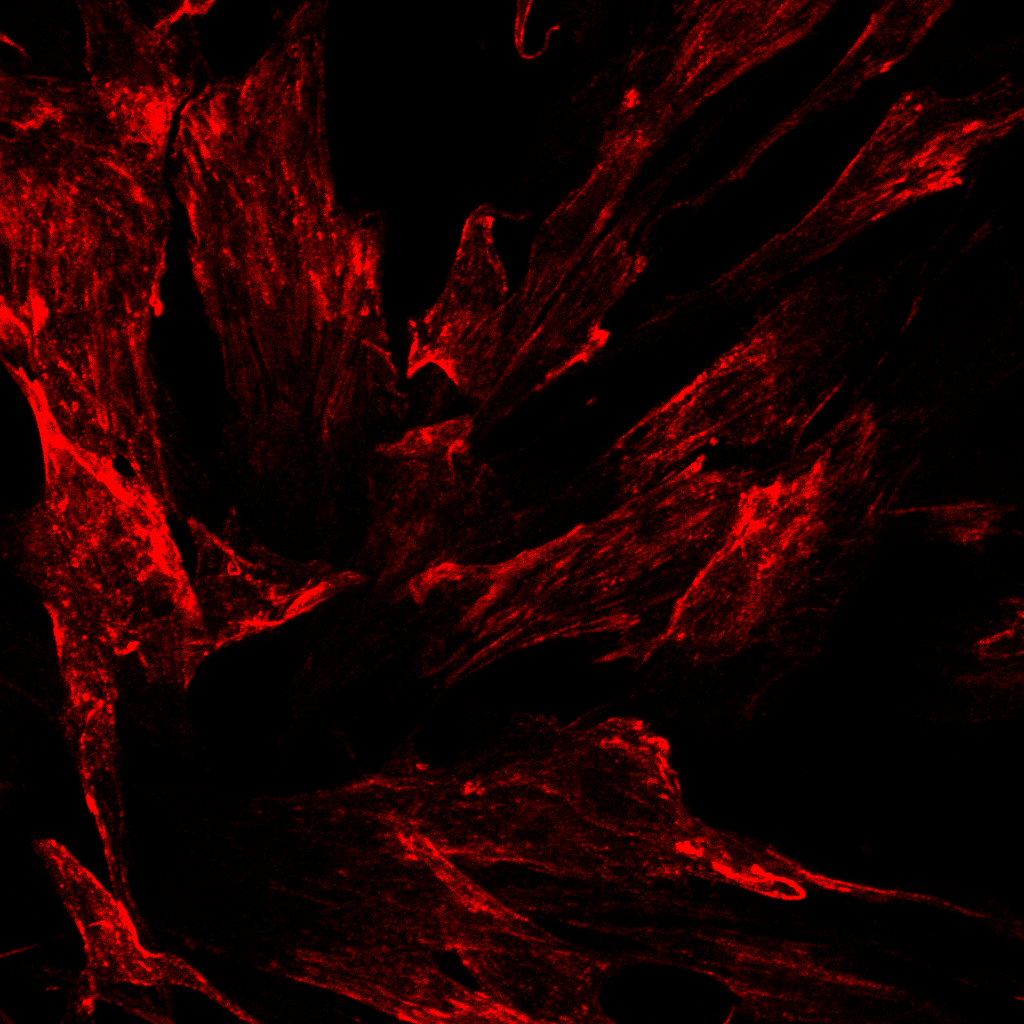

Supplement: Supplementary file 11 — Source data Fig. 7 [file 44319_2026_751_MOESM11_ESM.zip › Raw_data_Figure 7/Figure 7B/C3-NF shGq cav1.tif]

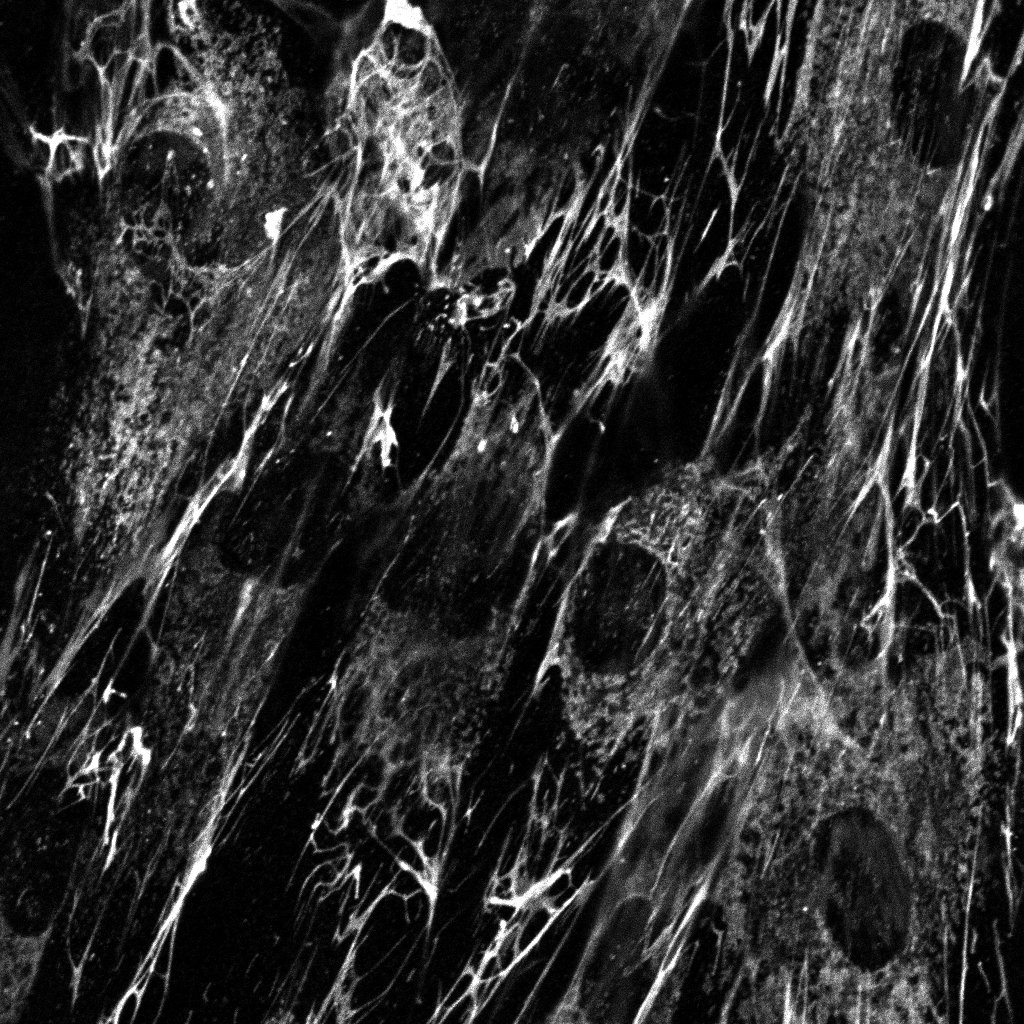

Supplement: Supplementary file 11 — Source data Fig. 7 [file 44319_2026_751_MOESM11_ESM.zip › Raw_data_Figure 7/Figure 7B/C4-NF shCtrl FN.tif]

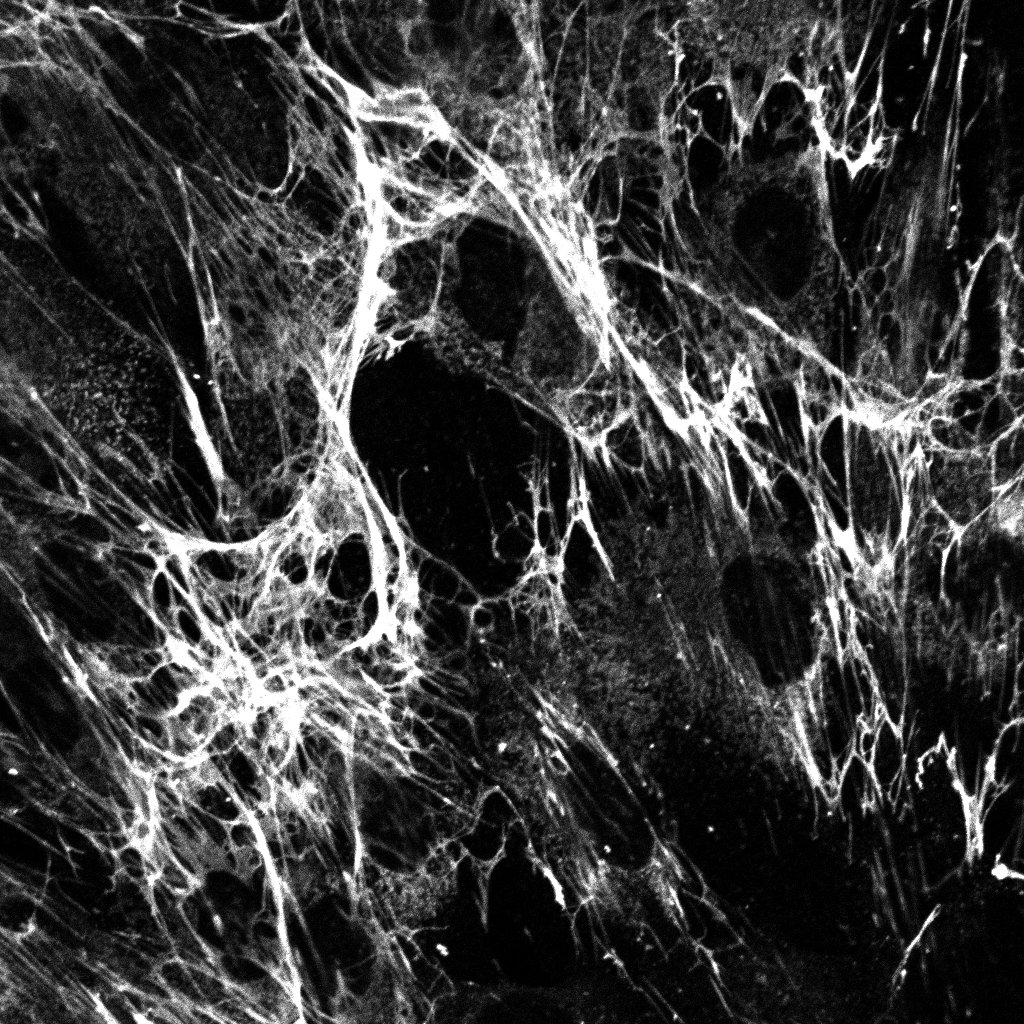

Supplement: Supplementary file 11 — Source data Fig. 7 [file 44319_2026_751_MOESM11_ESM.zip › Raw_data_Figure 7/Figure 7B/C4-NF shGq FN.tif]

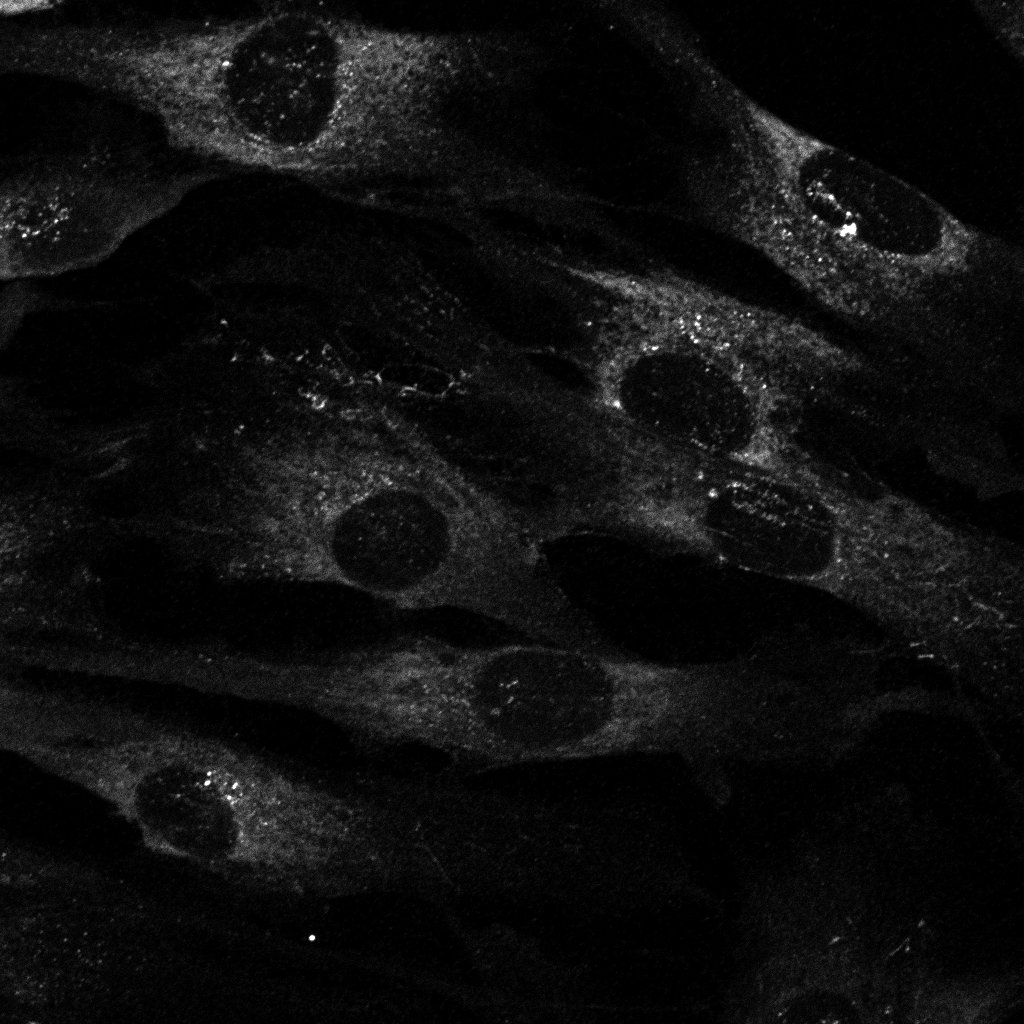

Supplement: Supplementary file 11 — Source data Fig. 7 [file 44319_2026_751_MOESM11_ESM.zip › Raw_data_Figure 7/Figure 7B/NF shCtrl col I.tif]

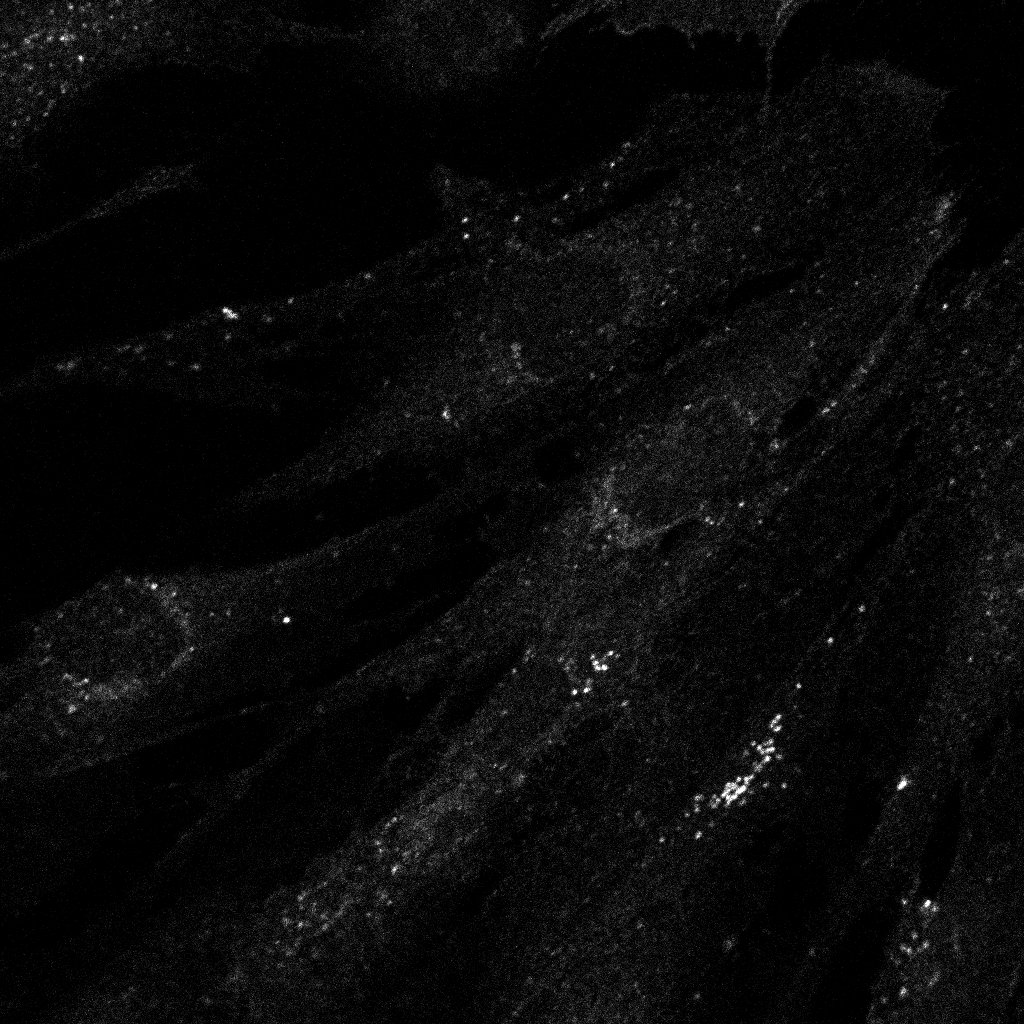

Supplement: Supplementary file 11 — Source data Fig. 7 [file 44319_2026_751_MOESM11_ESM.zip › Raw_data_Figure 7/Figure 7B/NF shCtrl PDGFR.tif]

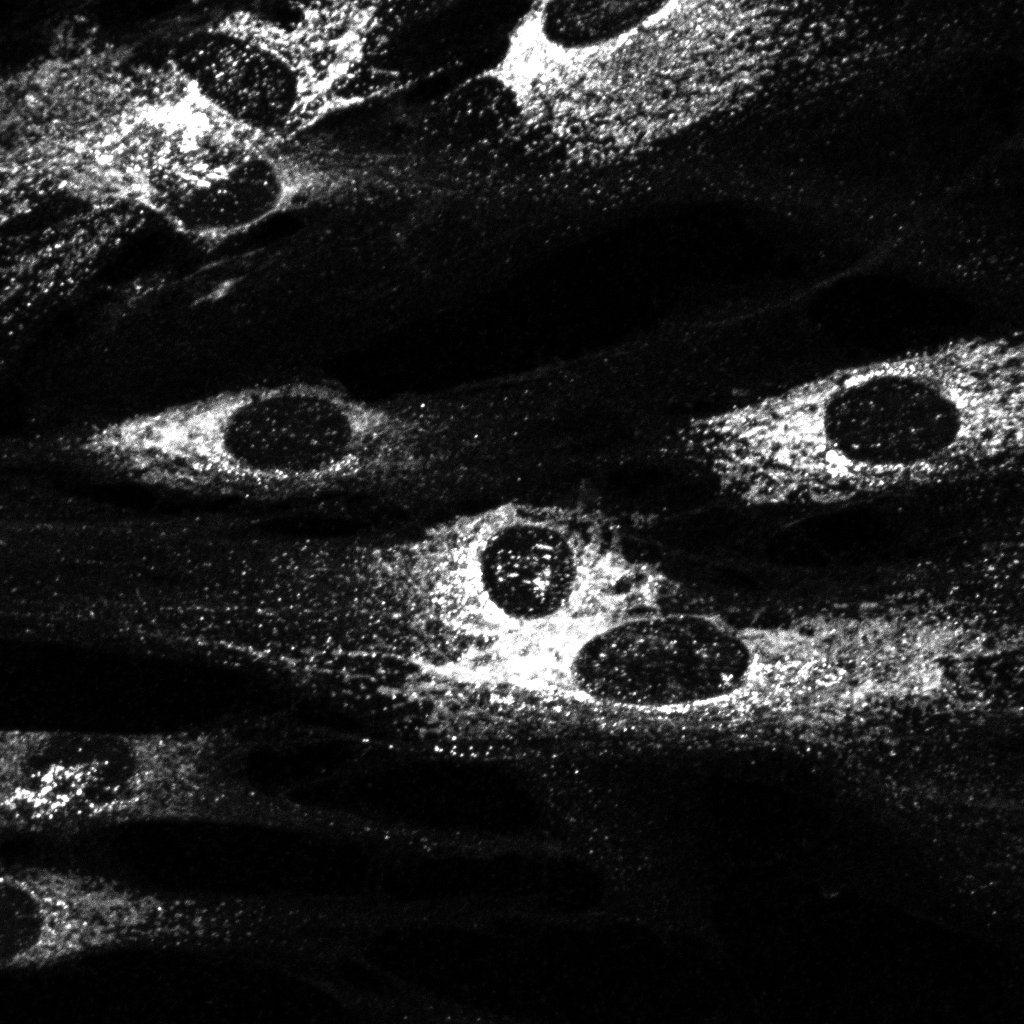

Supplement: Supplementary file 11 — Source data Fig. 7 [file 44319_2026_751_MOESM11_ESM.zip › Raw_data_Figure 7/Figure 7B/NF shGq col I.tif]

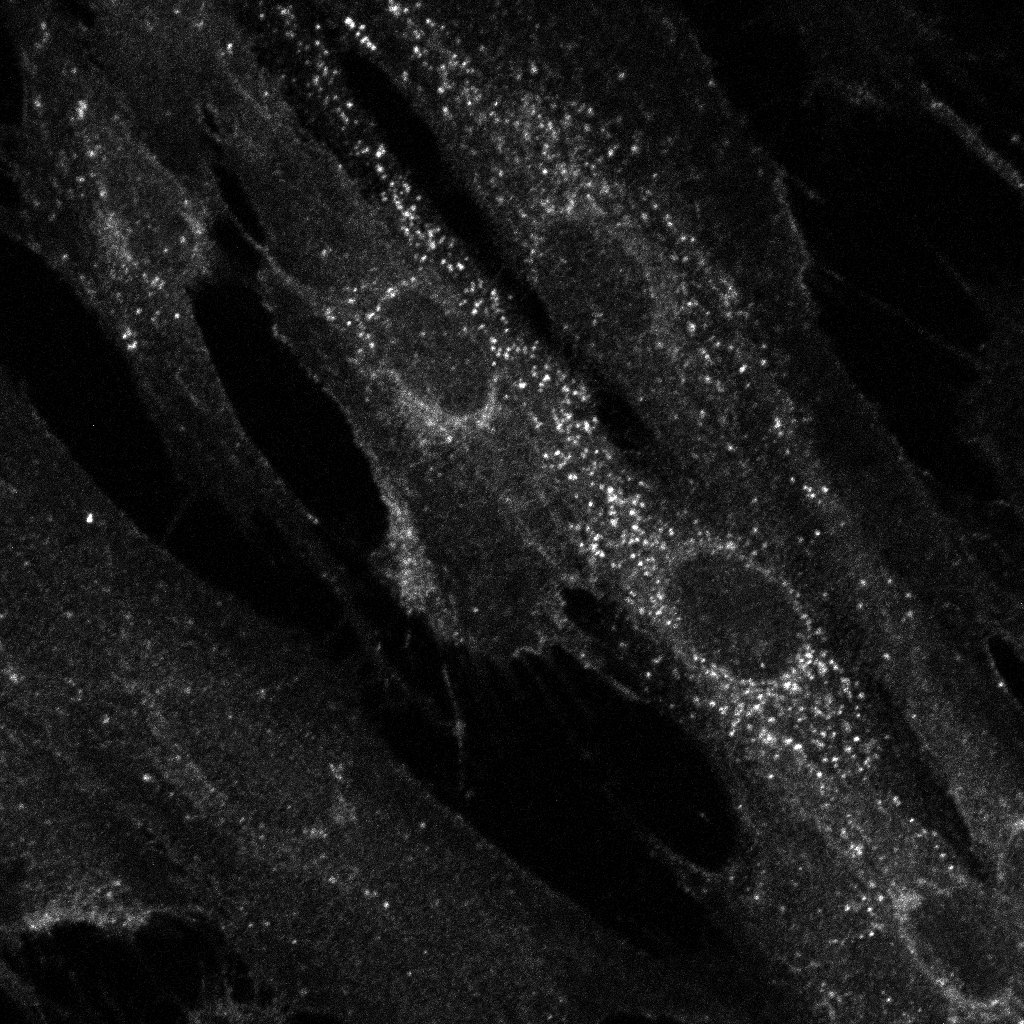

Supplement: Supplementary file 11 — Source data Fig. 7 [file 44319_2026_751_MOESM11_ESM.zip › Raw_data_Figure 7/Figure 7B/NF shGq PDGFR.tif]

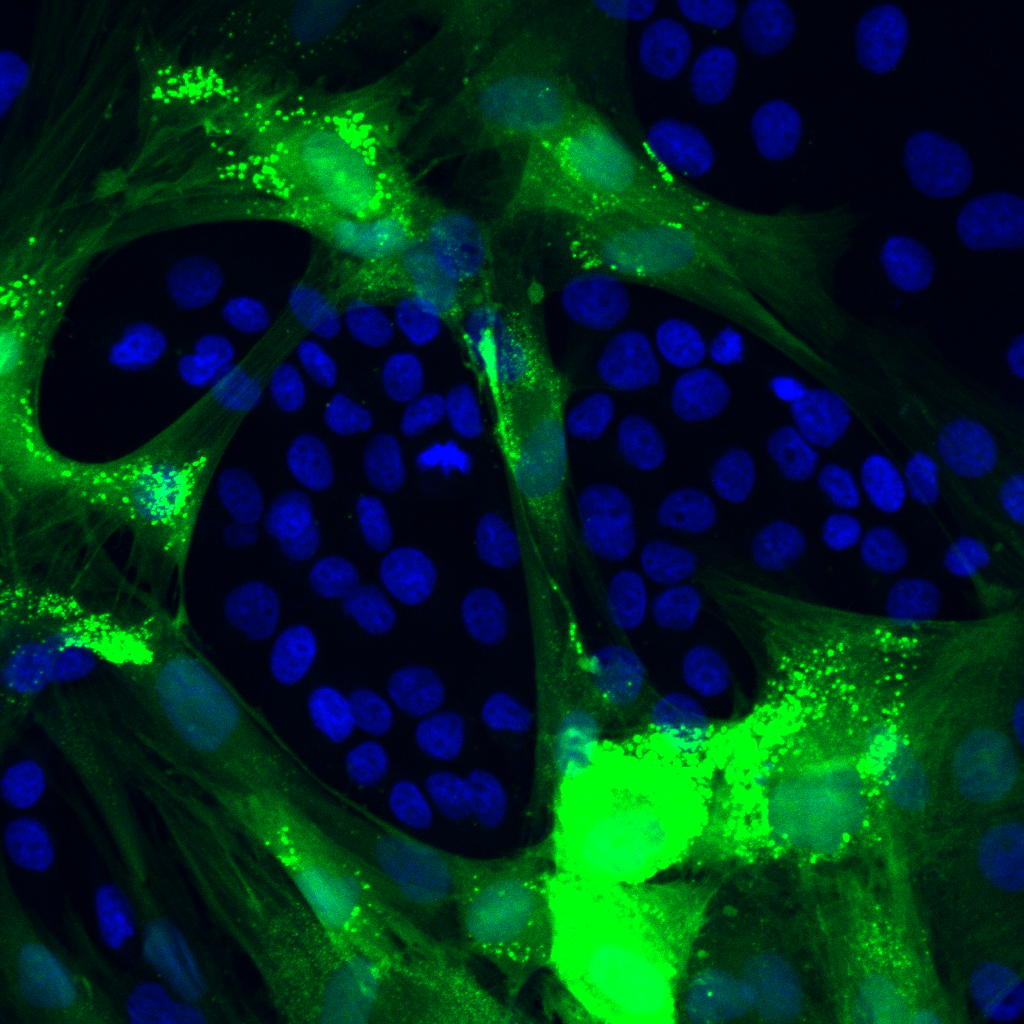

Supplement: Supplementary file 11 — Source data Fig. 7 [file 44319_2026_751_MOESM11_ESM.zip › Raw_data_Figure 7/Figure 7C/NFshctrl +cal27 de tincion cav1 blue-green.tif]

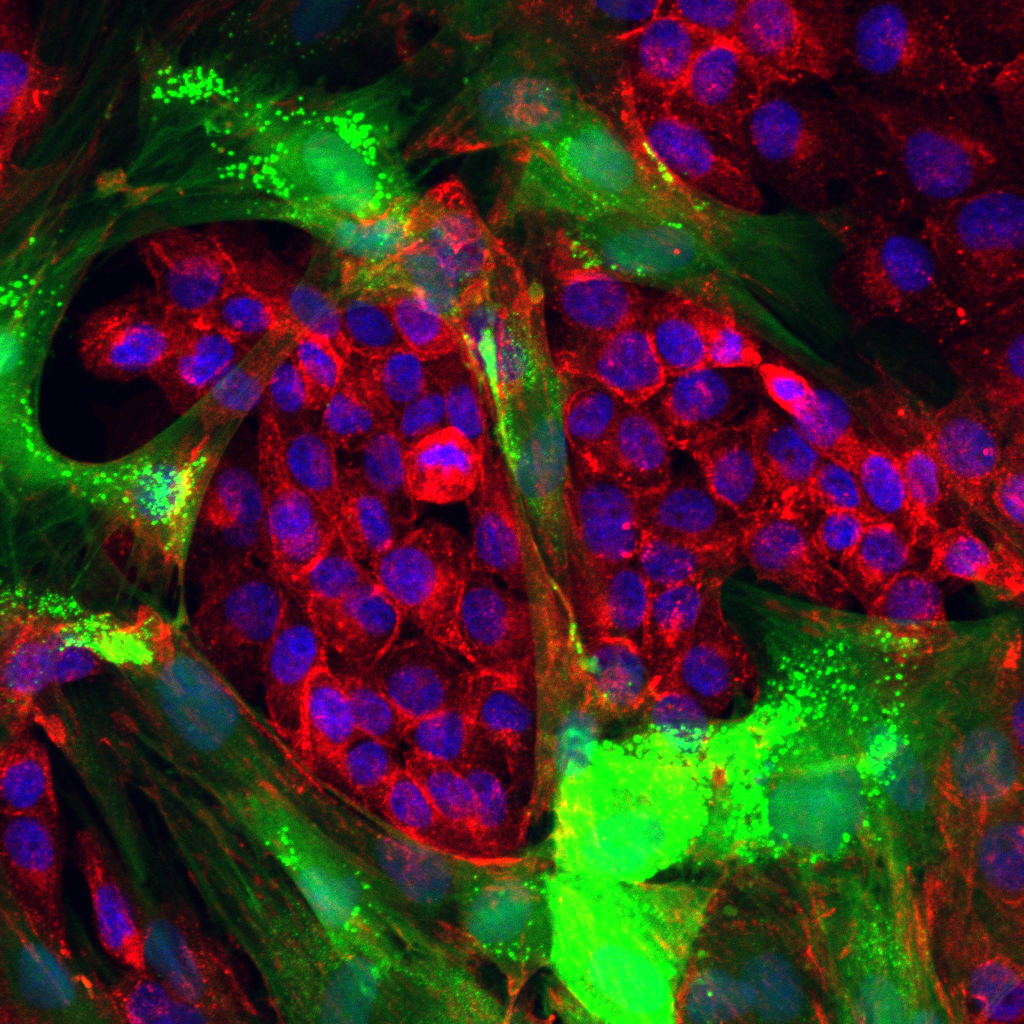

Supplement: Supplementary file 11 — Source data Fig. 7 [file 44319_2026_751_MOESM11_ESM.zip › Raw_data_Figure 7/Figure 7C/NFshctrl +cal27 de tincion cav1 merge.tif]

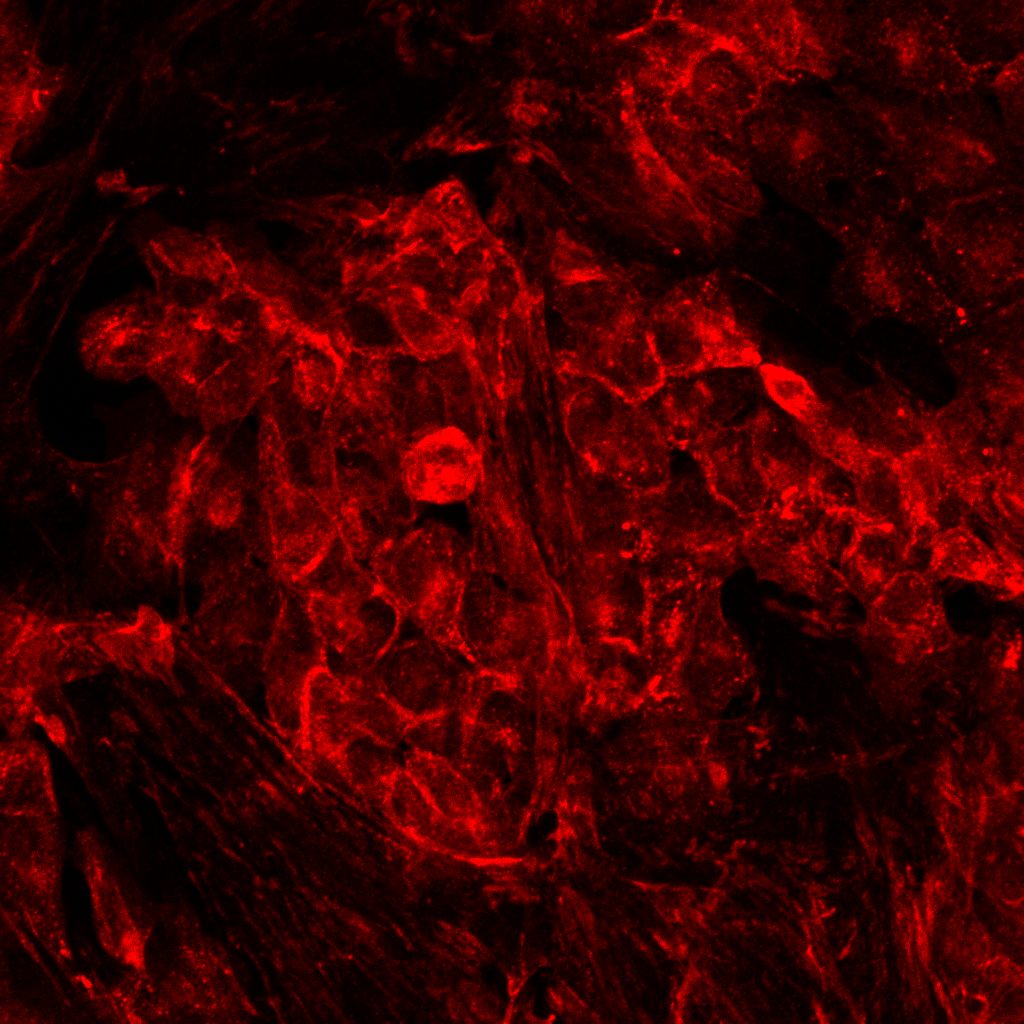

Supplement: Supplementary file 11 — Source data Fig. 7 [file 44319_2026_751_MOESM11_ESM.zip › Raw_data_Figure 7/Figure 7C/NFshctrl +cal27 de tincion cav1 red.tif]

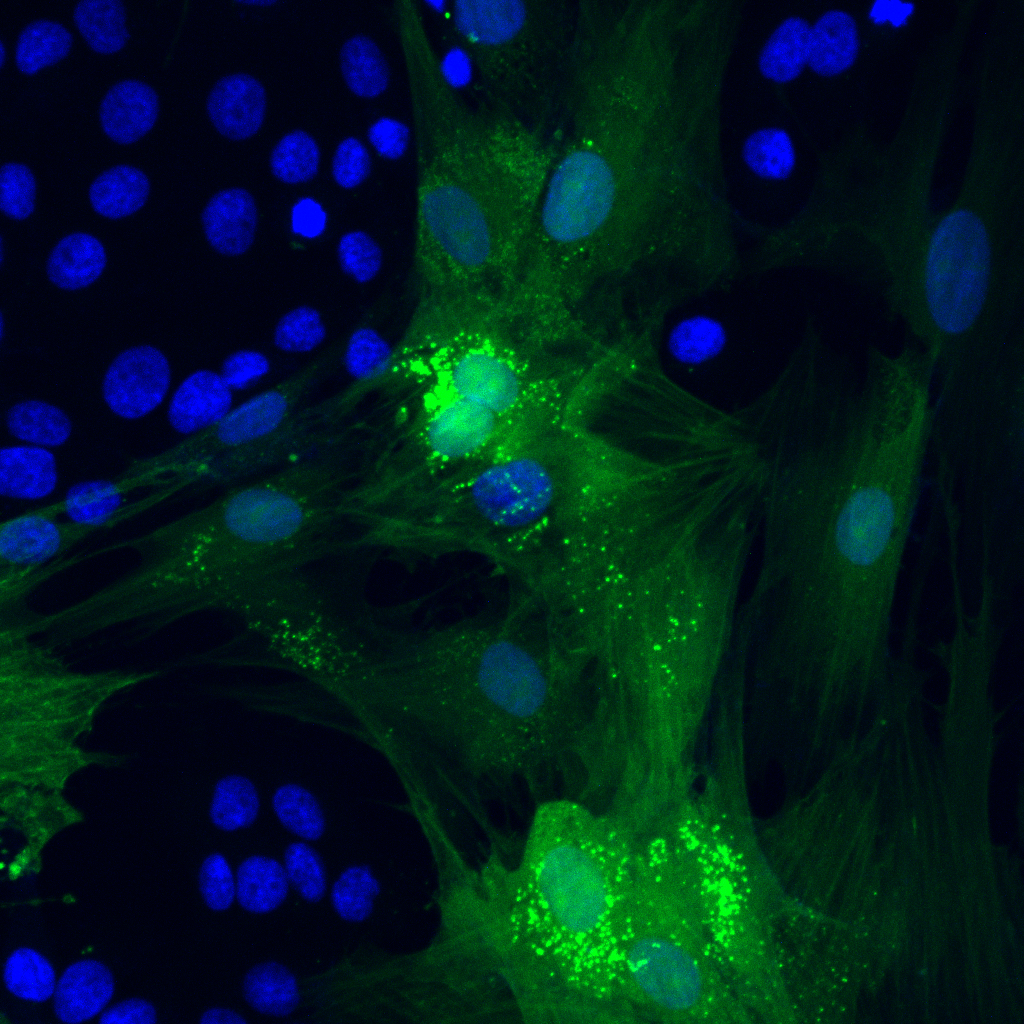

Supplement: Supplementary file 11 — Source data Fig. 7 [file 44319_2026_751_MOESM11_ESM.zip › Raw_data_Figure 7/Figure 7C/NFshGq +cal27 de tincion cav1 blue-green.tif]

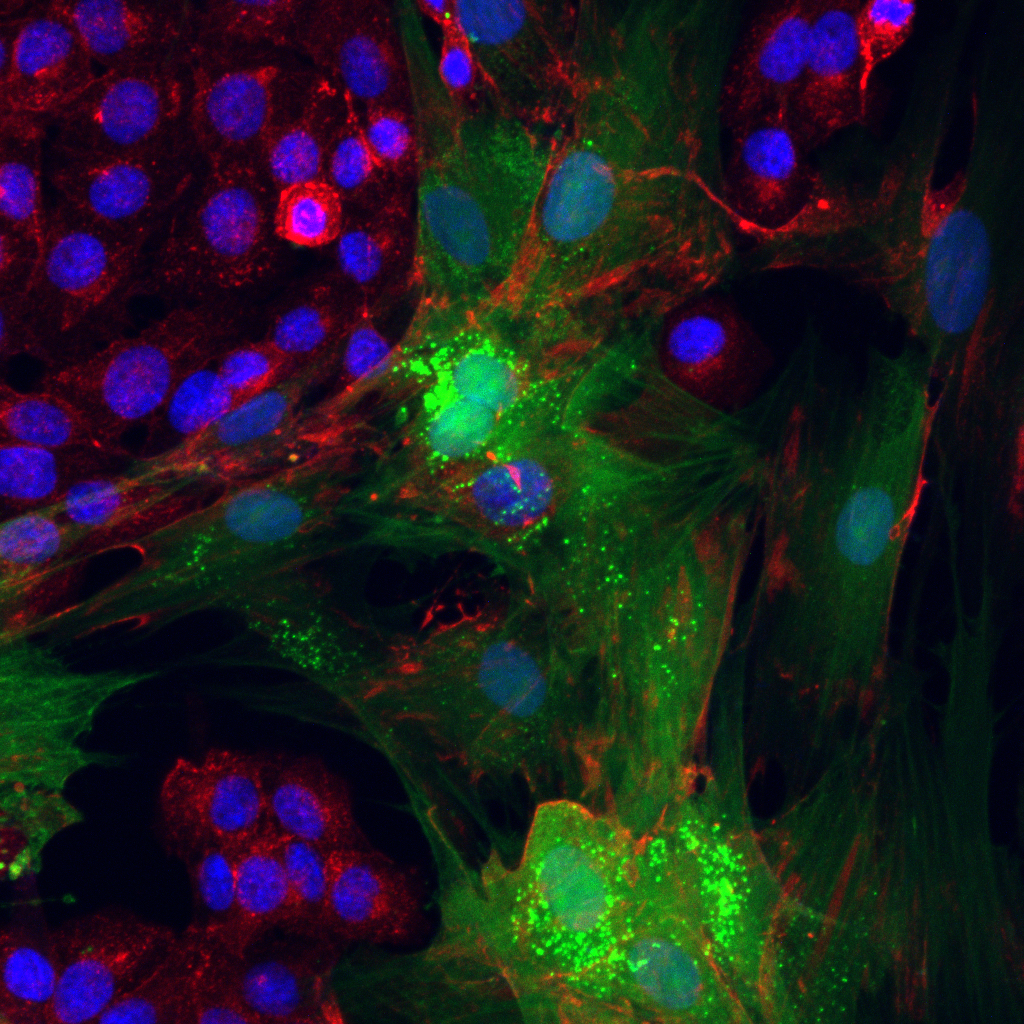

Supplement: Supplementary file 11 — Source data Fig. 7 [file 44319_2026_751_MOESM11_ESM.zip › Raw_data_Figure 7/Figure 7C/NFshGq+cal27 de tincion cav1 merge.tif]

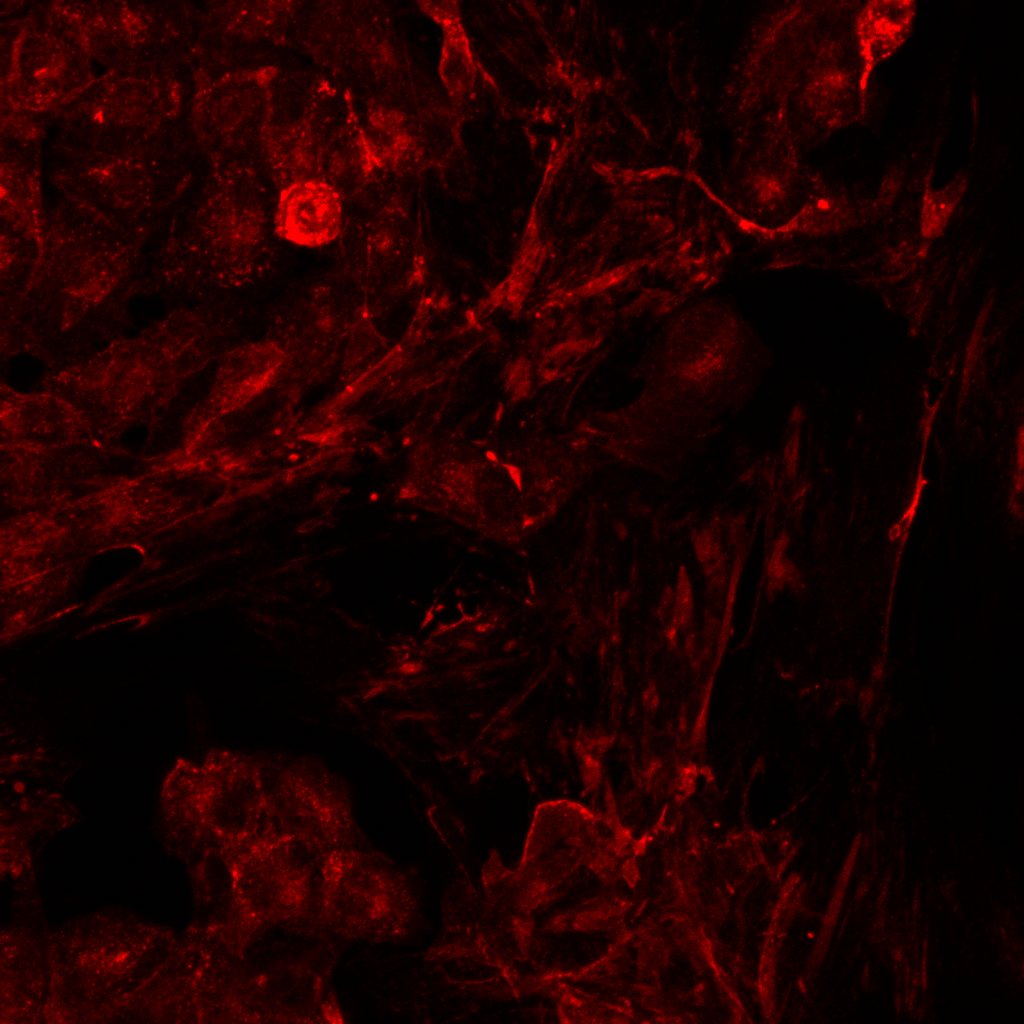

Supplement: Supplementary file 11 — Source data Fig. 7 [file 44319_2026_751_MOESM11_ESM.zip › Raw_data_Figure 7/Figure 7C/NFshGq+cal27 de tincion cav1 red.tif]

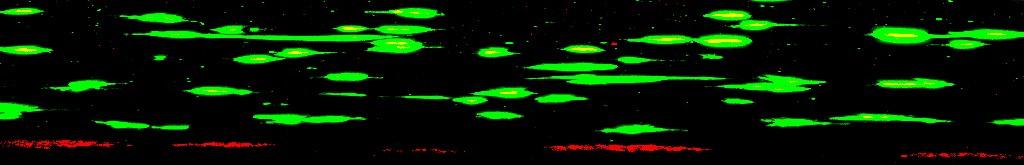

Supplement: Supplementary file 11 — Source data Fig. 7 [file 44319_2026_751_MOESM11_ESM.zip › Raw_data_Figure 7/Figure 7D/MAX_Reslice of NFshctrl.tif]

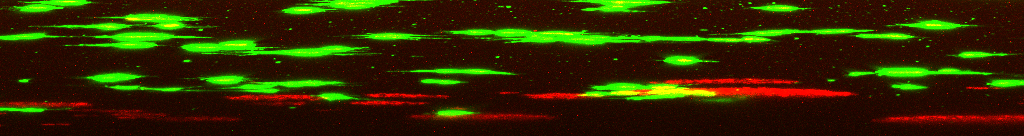

Supplement: Supplementary file 11 — Source data Fig. 7 [file 44319_2026_751_MOESM11_ESM.zip › Raw_data_Figure 7/Figure 7D/MAX_Reslice of NFshGq.tif]

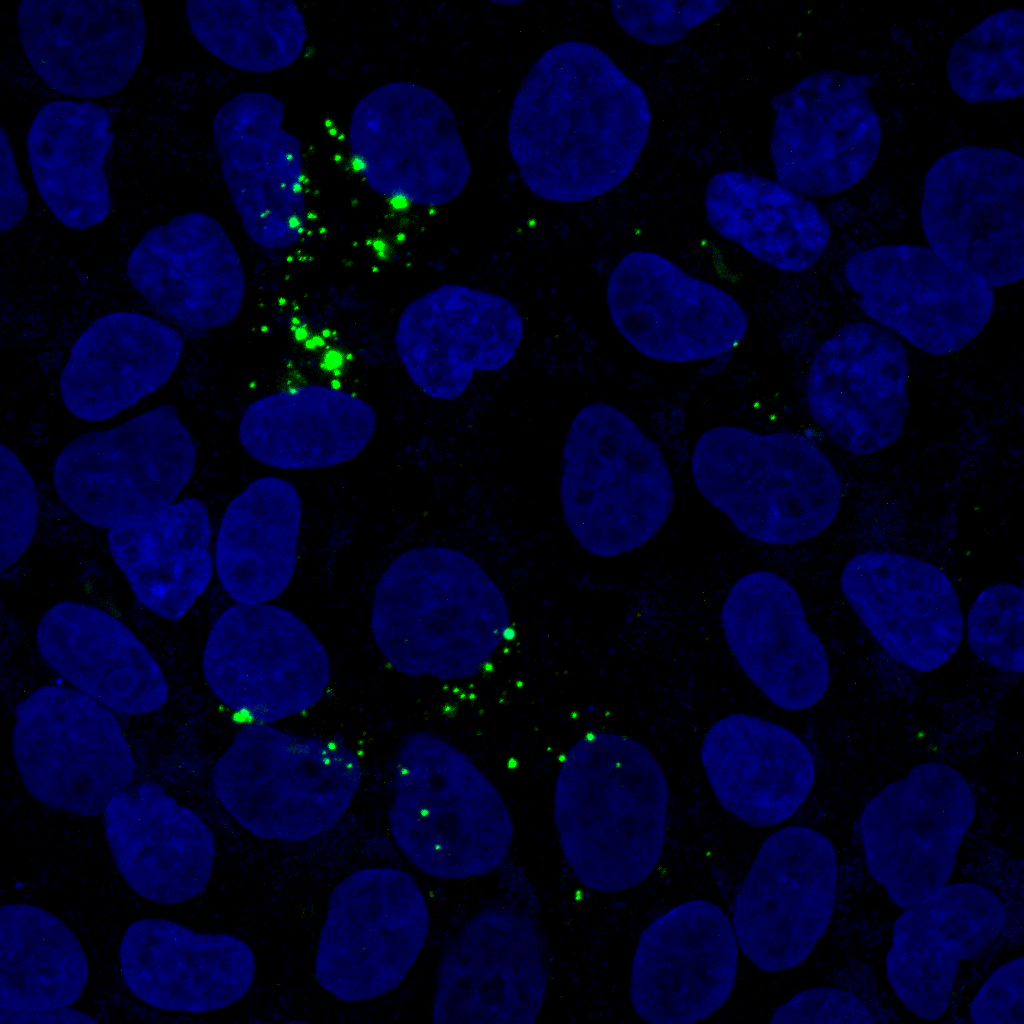

Supplement: Supplementary file 11 — Source data Fig. 7 [file 44319_2026_751_MOESM11_ESM.zip › Raw_data_Figure 7/Figure 7F/cal27 +exos NFshctrl blue green.tif]

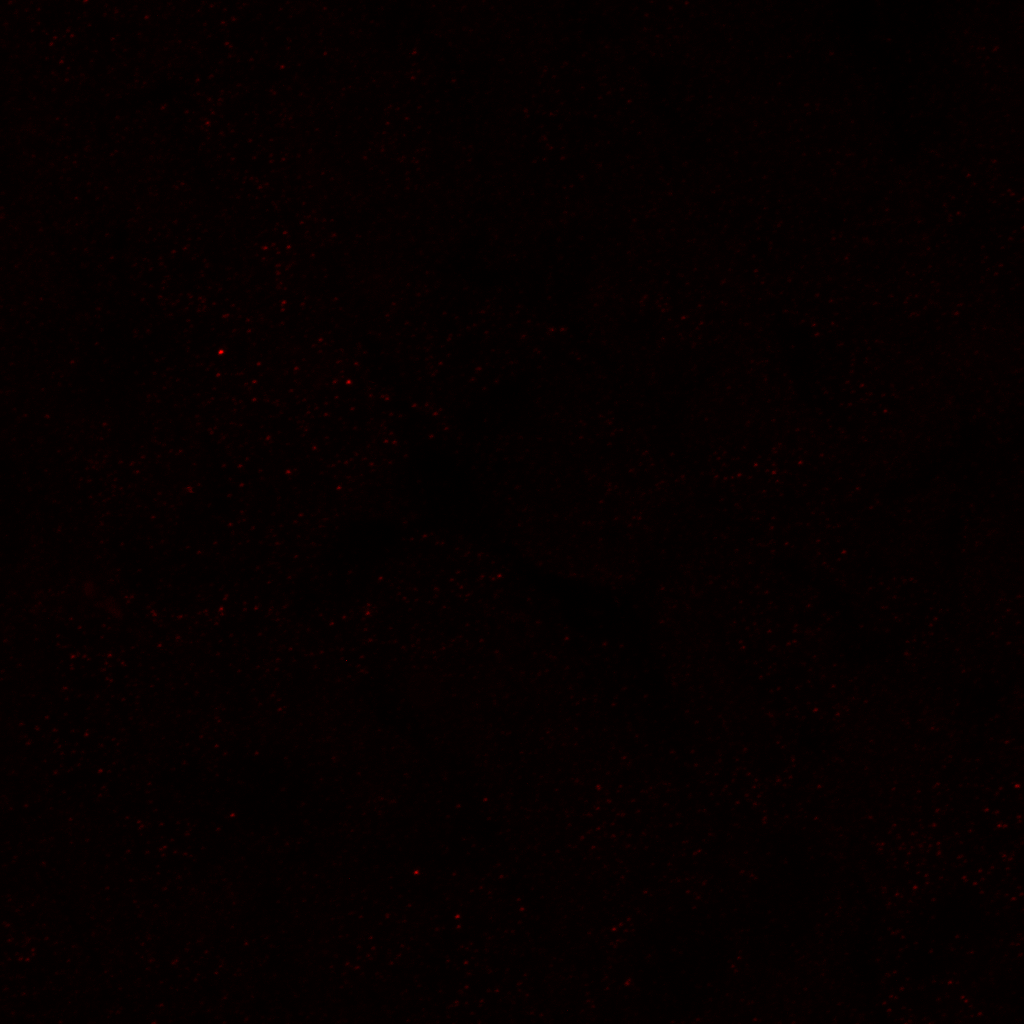

Supplement: Supplementary file 11 — Source data Fig. 7 [file 44319_2026_751_MOESM11_ESM.zip › Raw_data_Figure 7/Figure 7F/cal27 +exos NFshctrl PDGFR red.tif]

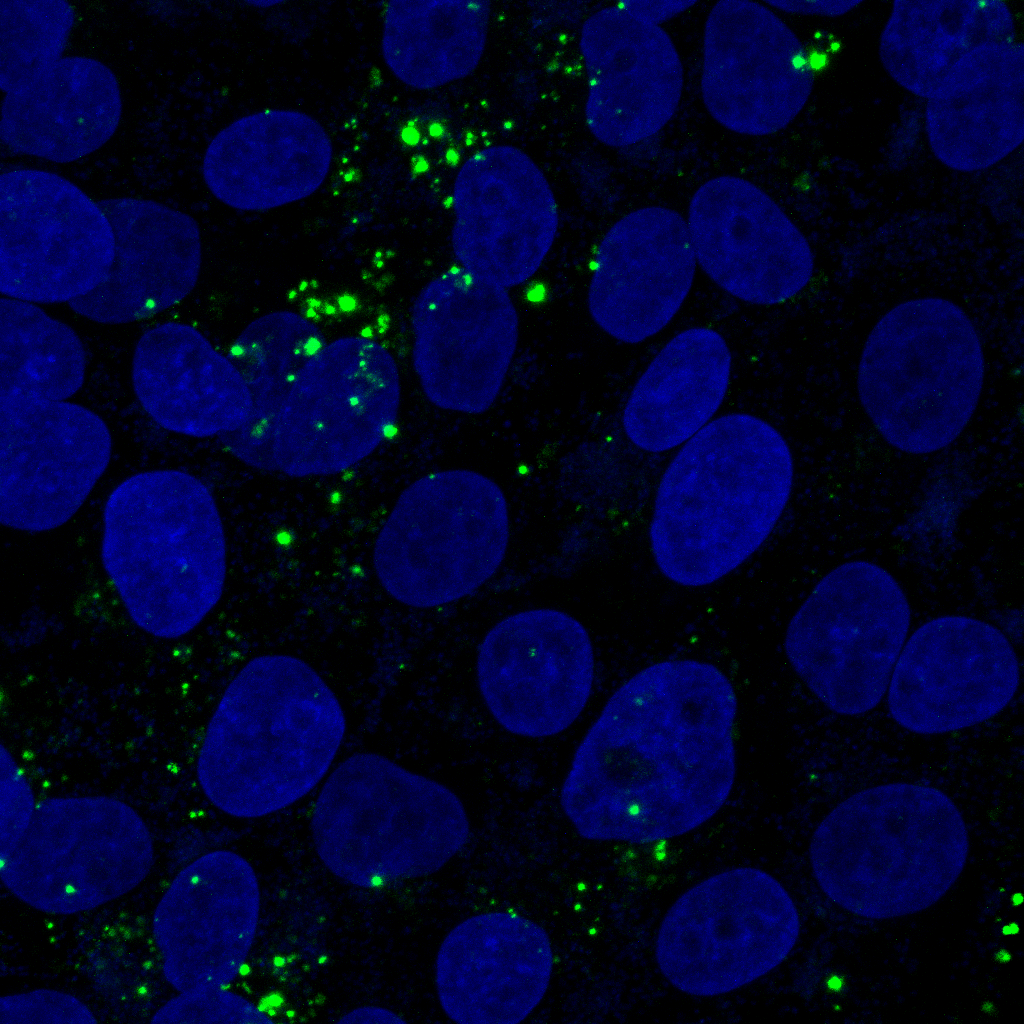

Supplement: Supplementary file 11 — Source data Fig. 7 [file 44319_2026_751_MOESM11_ESM.zip › Raw_data_Figure 7/Figure 7F/cal27 +exos NFshGq blue green.tif]

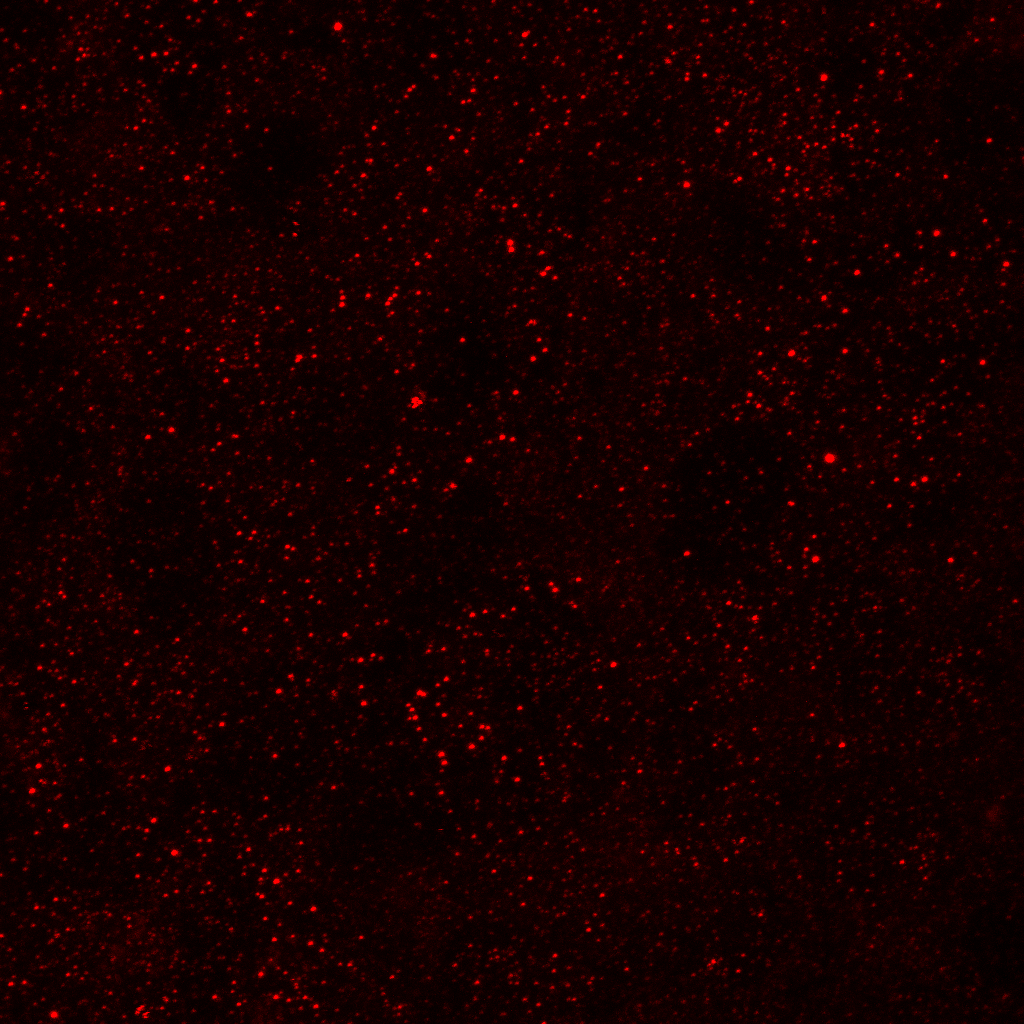

Supplement: Supplementary file 11 — Source data Fig. 7 [file 44319_2026_751_MOESM11_ESM.zip › Raw_data_Figure 7/Figure 7F/cal27 +exos NFshGq PDGFR red.tif]

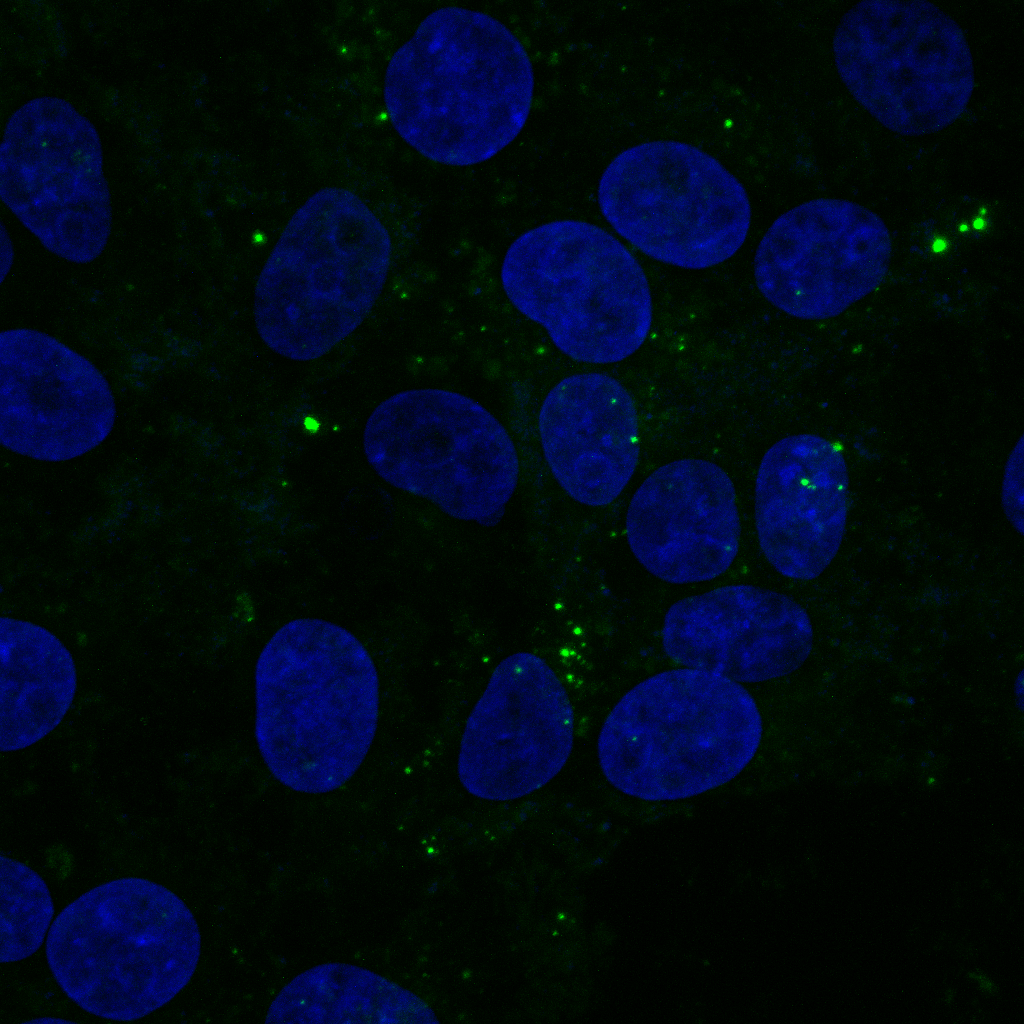

Supplement: Supplementary file 11 — Source data Fig. 7 [file 44319_2026_751_MOESM11_ESM.zip › Raw_data_Figure 7/Figure 7F/NFshctrol de cav1 blue green.tif]

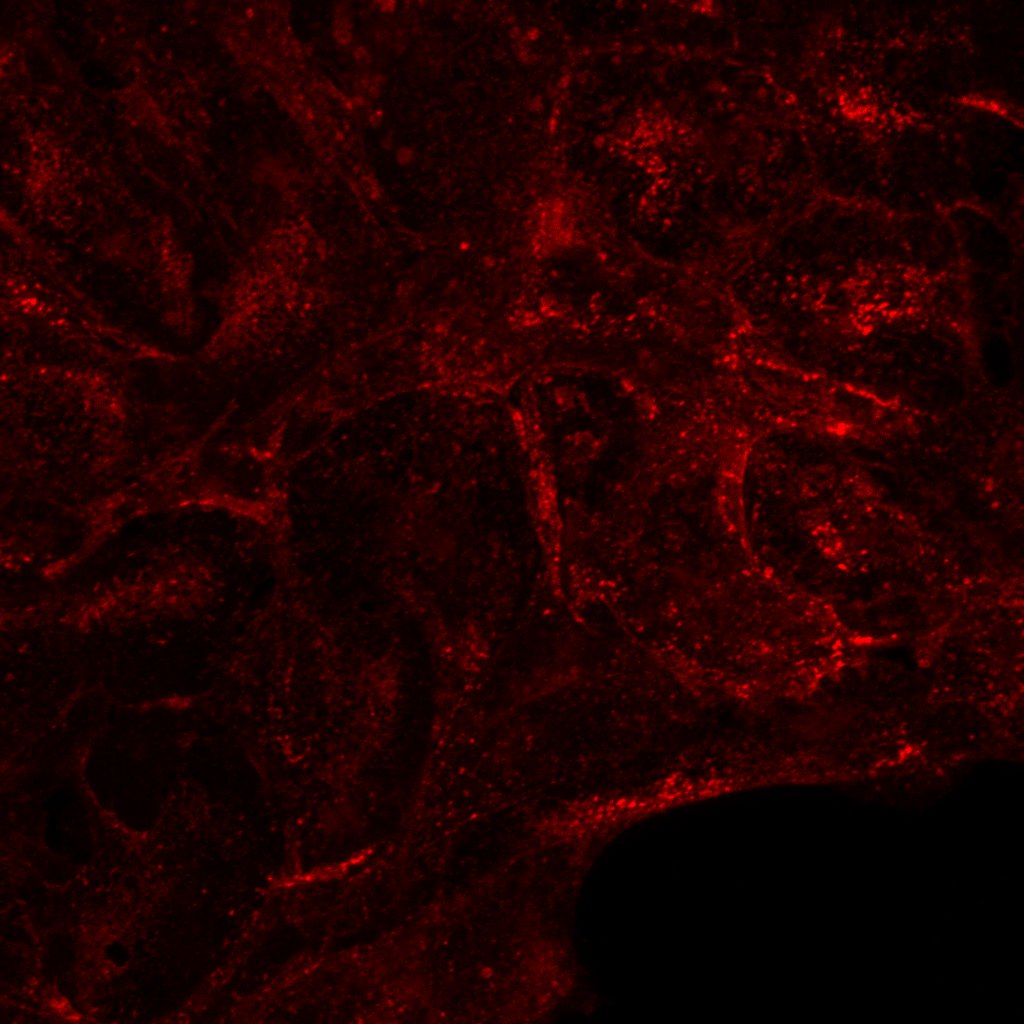

Supplement: Supplementary file 11 — Source data Fig. 7 [file 44319_2026_751_MOESM11_ESM.zip › Raw_data_Figure 7/Figure 7F/NFshctrol de cav1 red.tif]

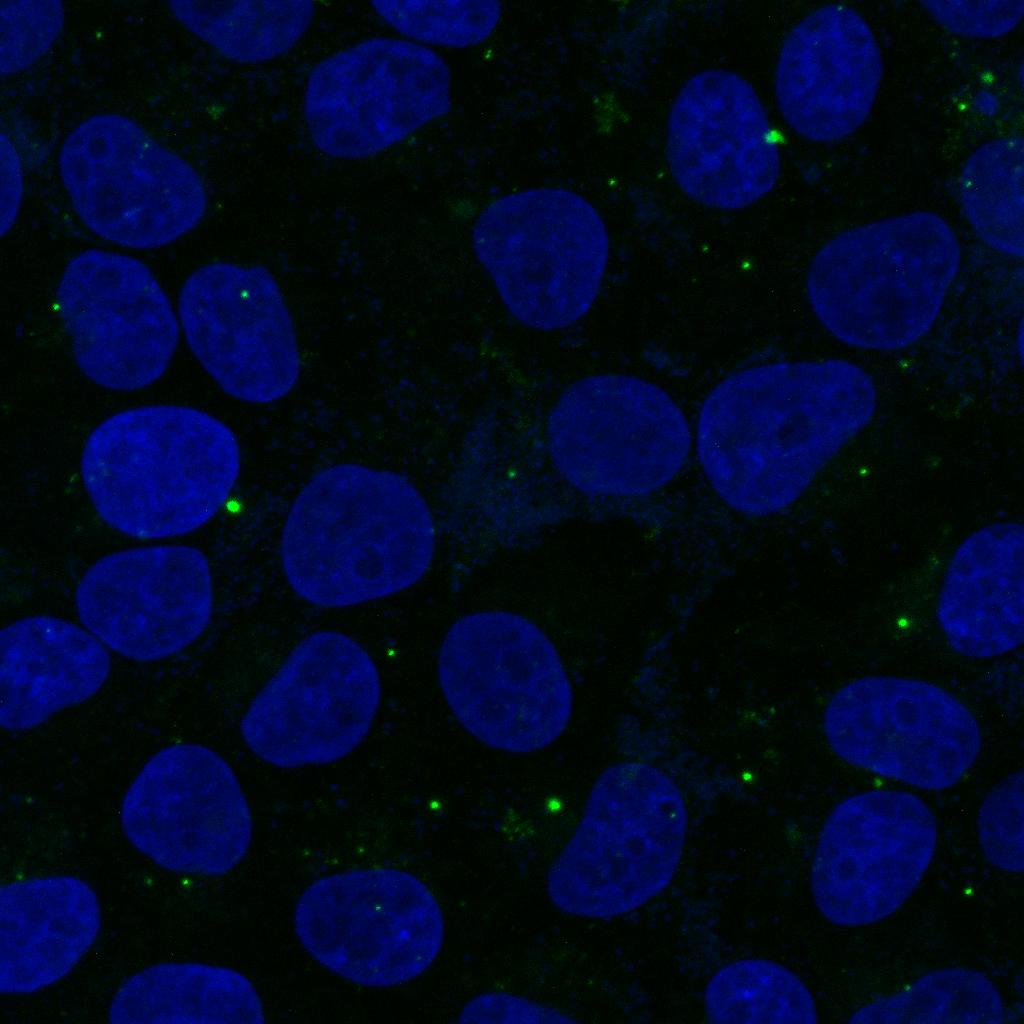

Supplement: Supplementary file 11 — Source data Fig. 7 [file 44319_2026_751_MOESM11_ESM.zip › Raw_data_Figure 7/Figure 7F/NFshGq de cav1 blue green 2.tif]

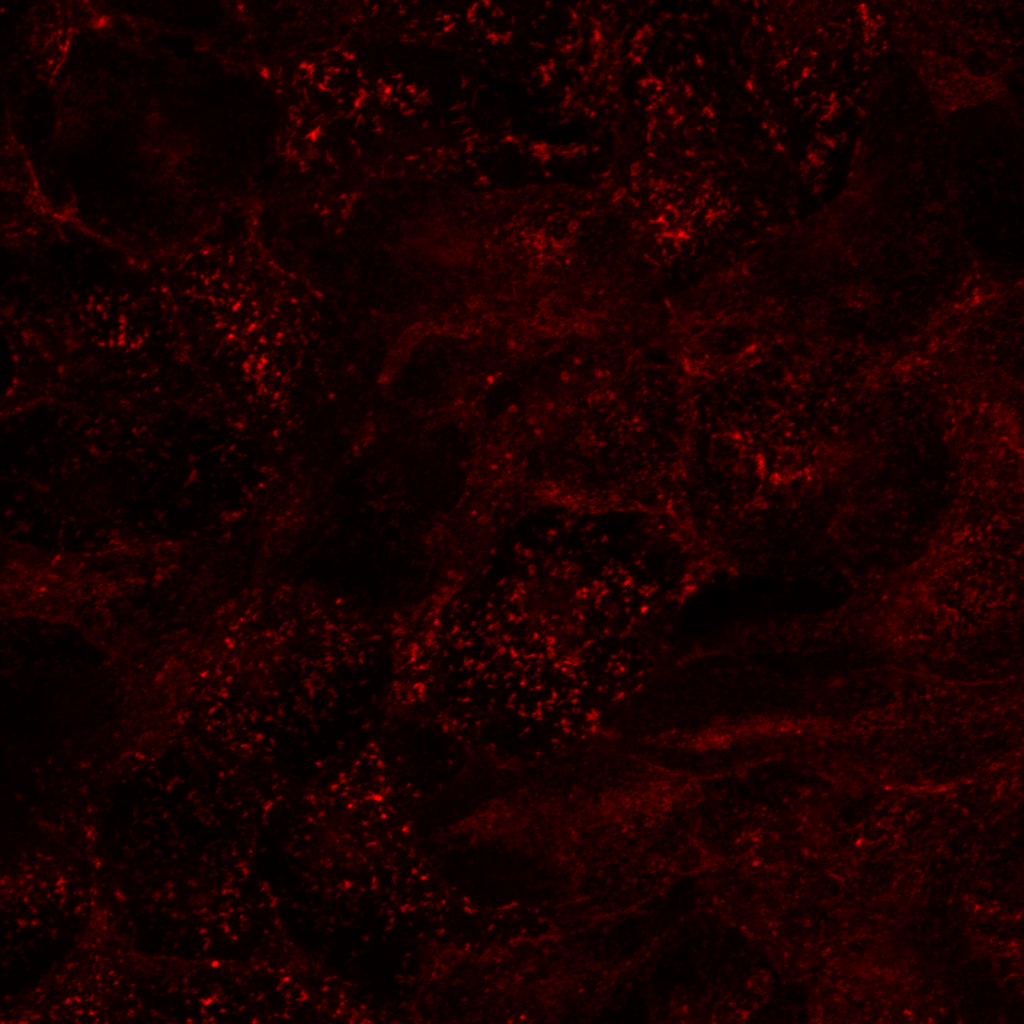

Supplement: Supplementary file 11 — Source data Fig. 7 [file 44319_2026_751_MOESM11_ESM.zip › Raw_data_Figure 7/Figure 7F/NFshGq de cav1 red 2.tif]

## Slide 1
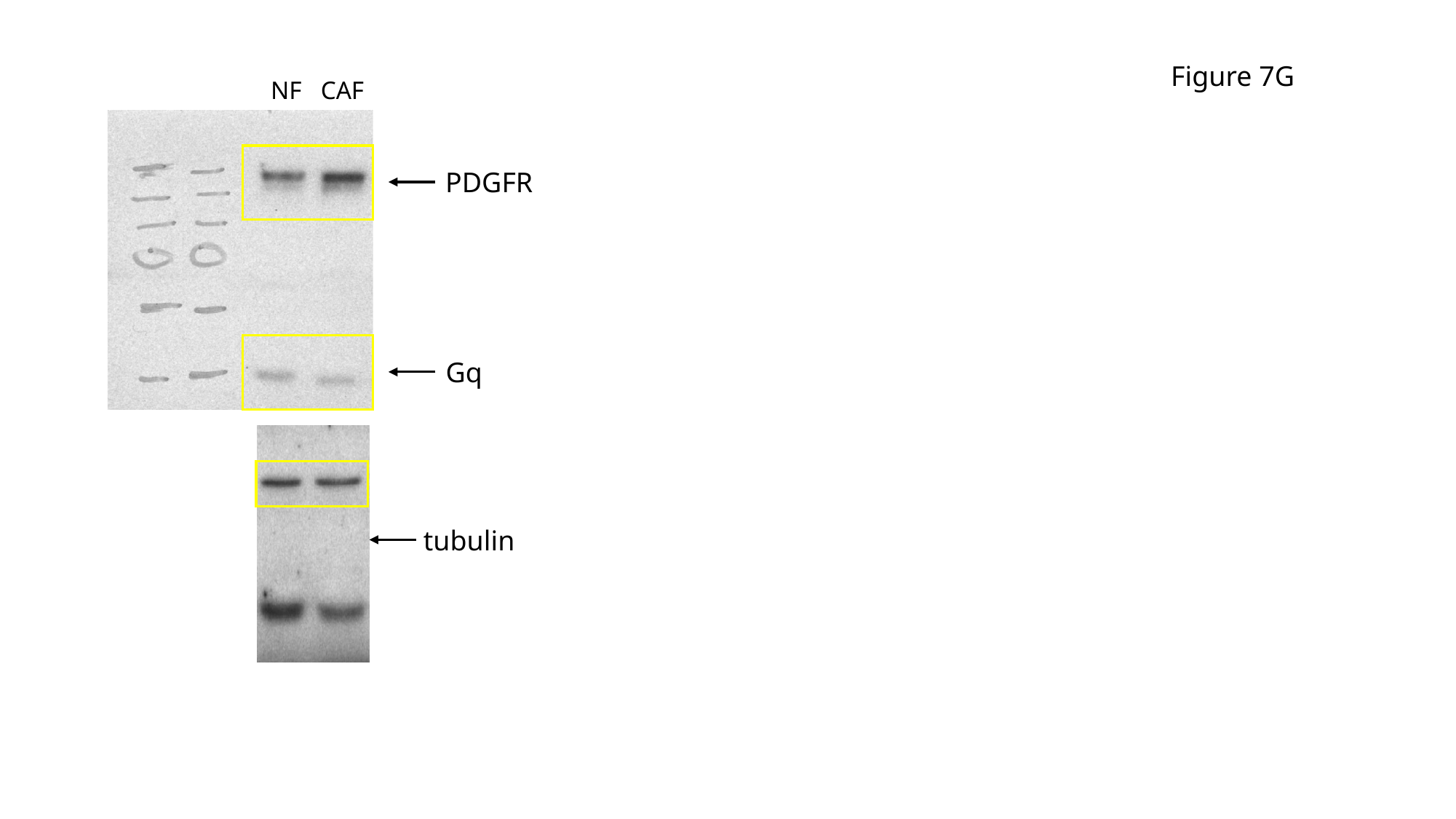

Figure 7G
NF CAF
PDGFR
Gq
tubulin

Supplement: Supplementary file 11 — Source data Fig. 7 [file 44319_2026_751_MOESM11_ESM.zip › Raw_data_Figure 7/Figure 7G/raw_blots_7G.pptx]

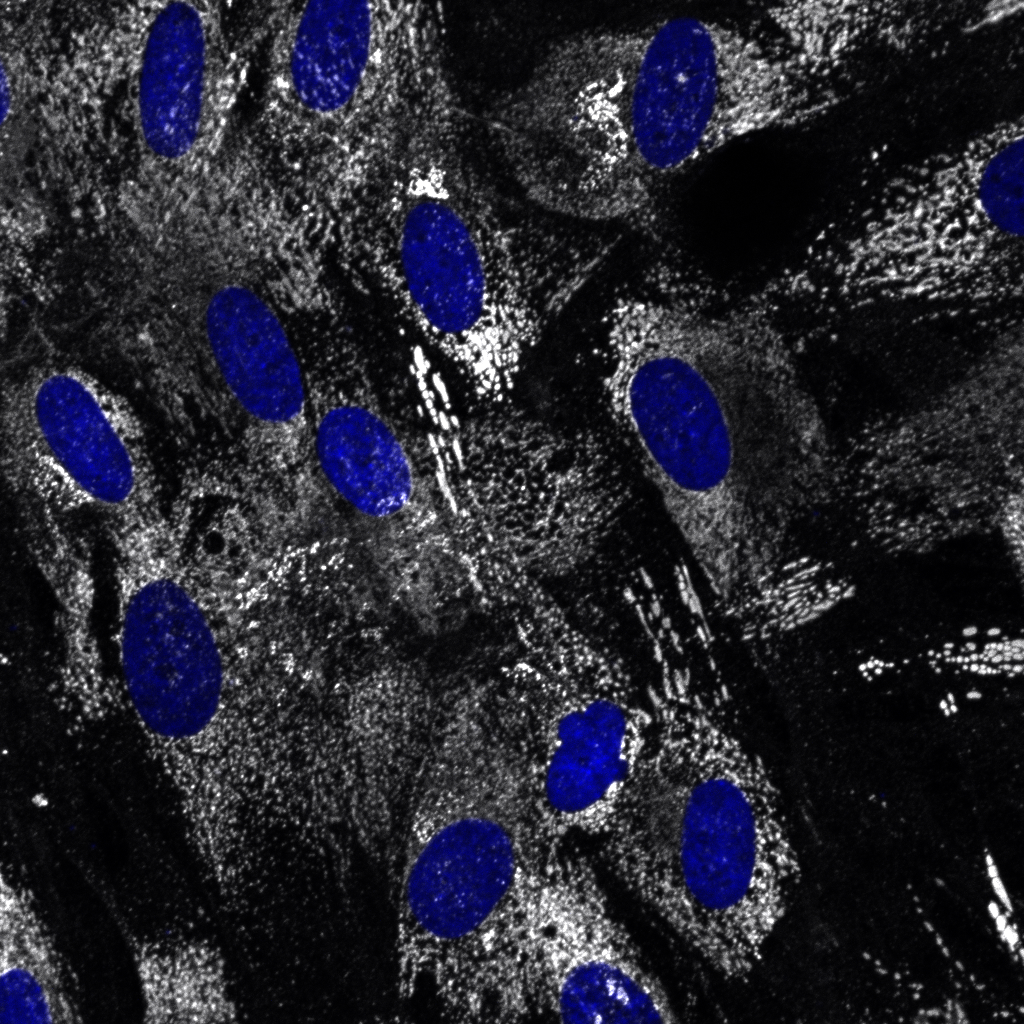

Supplement: Supplementary file 11 — Source data Fig. 7 [file 44319_2026_751_MOESM11_ESM.zip › Raw_data_Figure 7/Figure 7H/CAFs col I nuclei.tif]

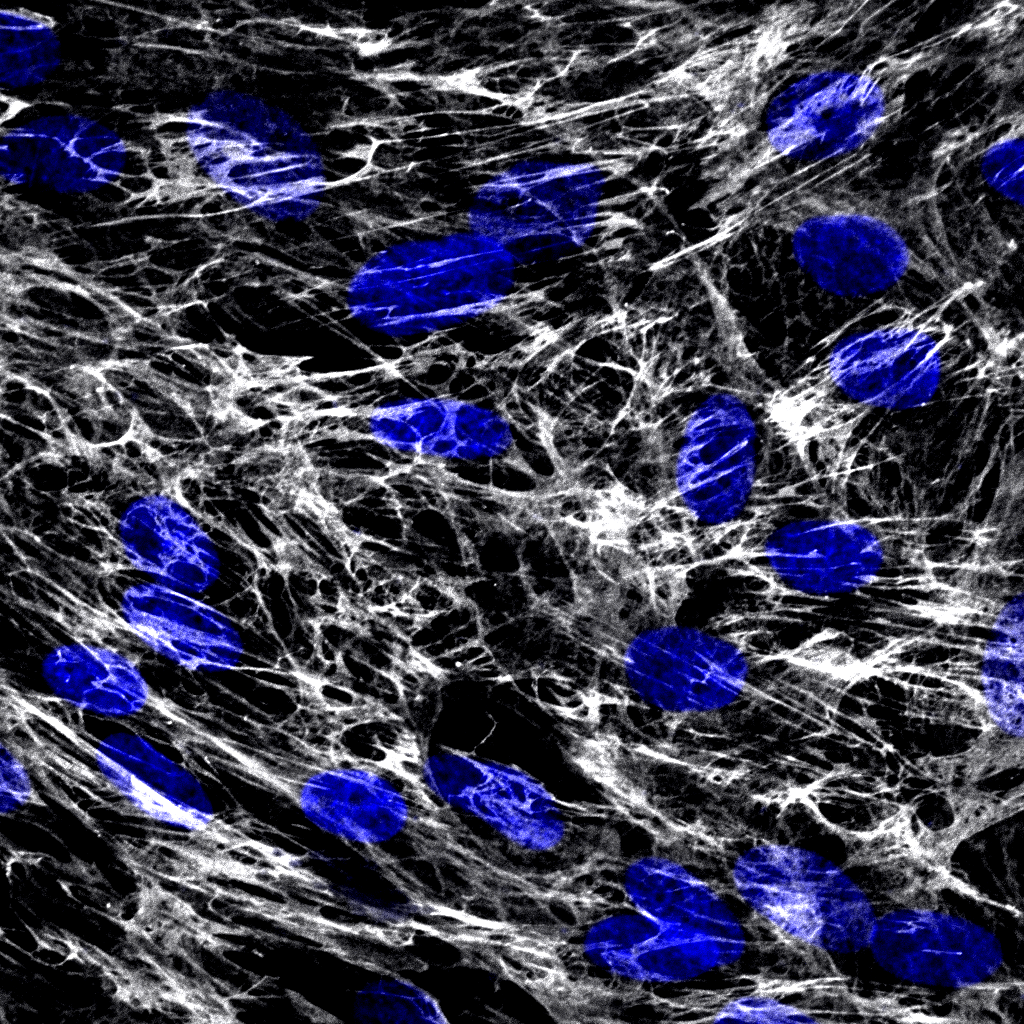

Supplement: Supplementary file 11 — Source data Fig. 7 [file 44319_2026_751_MOESM11_ESM.zip › Raw_data_Figure 7/Figure 7H/CAFs FN nuclei.tif]

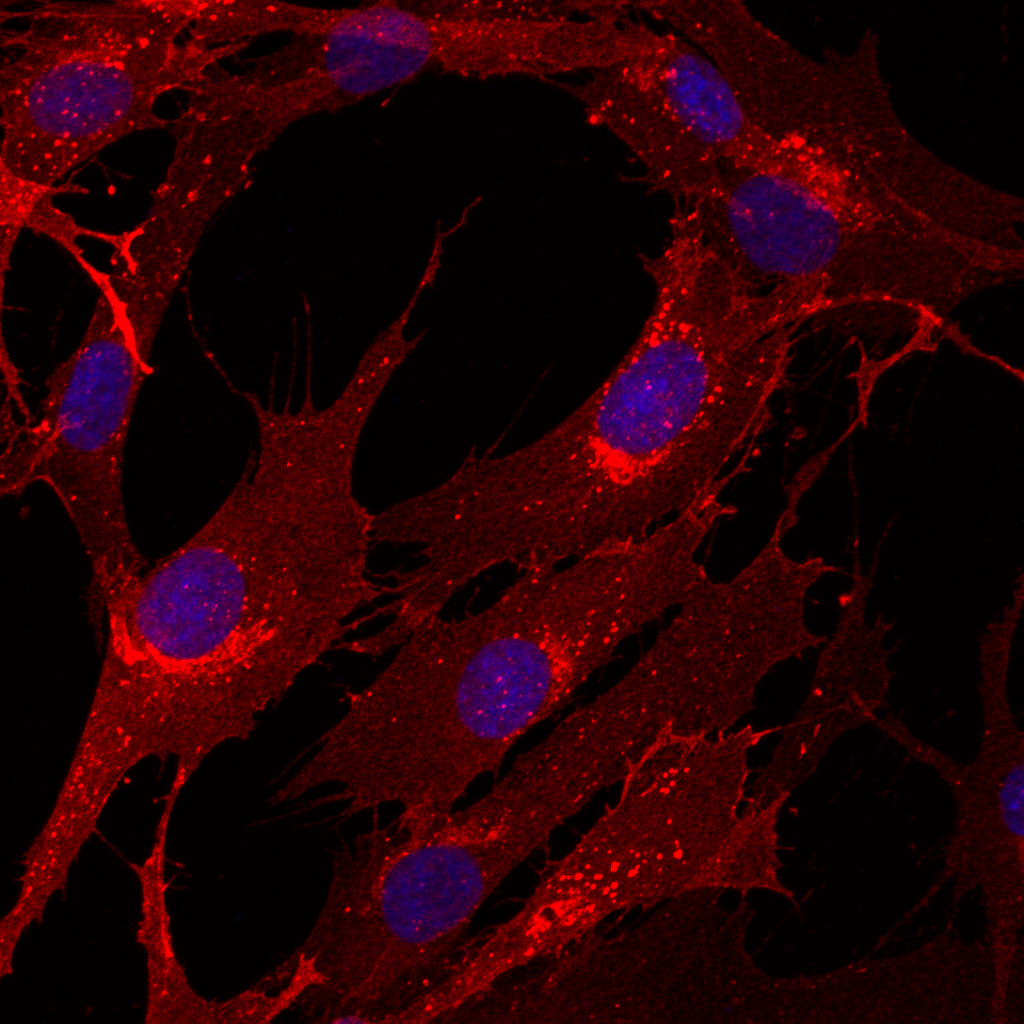

Supplement: Supplementary file 11 — Source data Fig. 7 [file 44319_2026_751_MOESM11_ESM.zip › Raw_data_Figure 7/Figure 7H/CAFs PDGFR nuclei 24 h.tif]

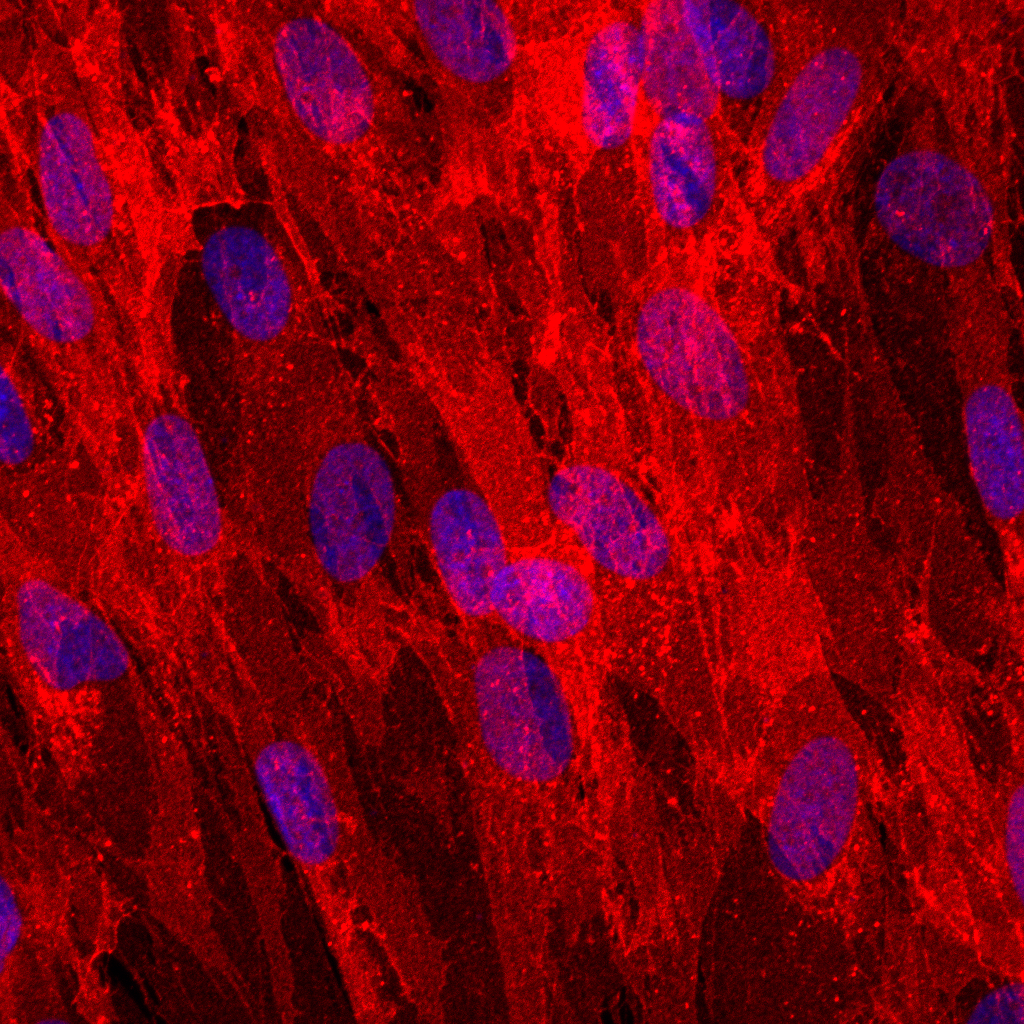

Supplement: Supplementary file 11 — Source data Fig. 7 [file 44319_2026_751_MOESM11_ESM.zip › Raw_data_Figure 7/Figure 7H/CAFs PDGFR nuclei 72h.tif]

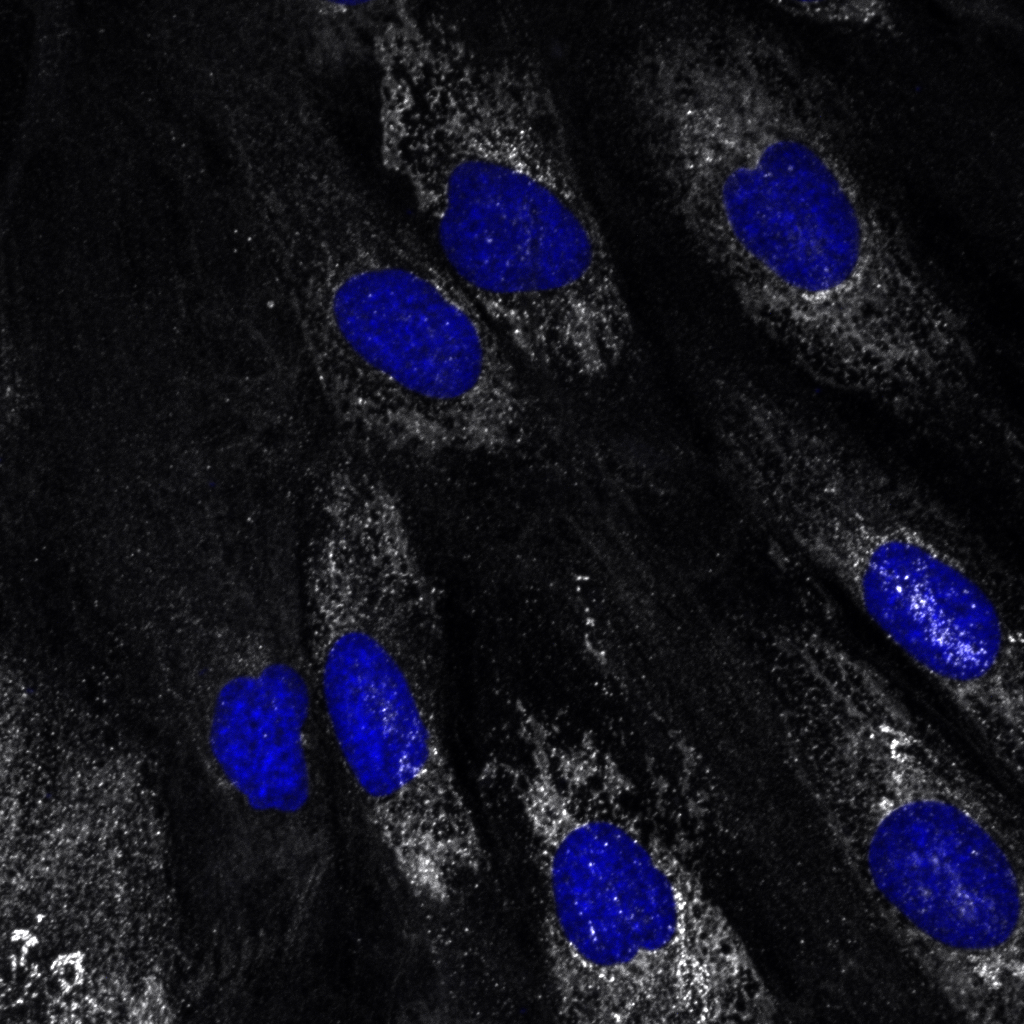

Supplement: Supplementary file 11 — Source data Fig. 7 [file 44319_2026_751_MOESM11_ESM.zip › Raw_data_Figure 7/Figure 7H/NFs col I nuclei.tif]

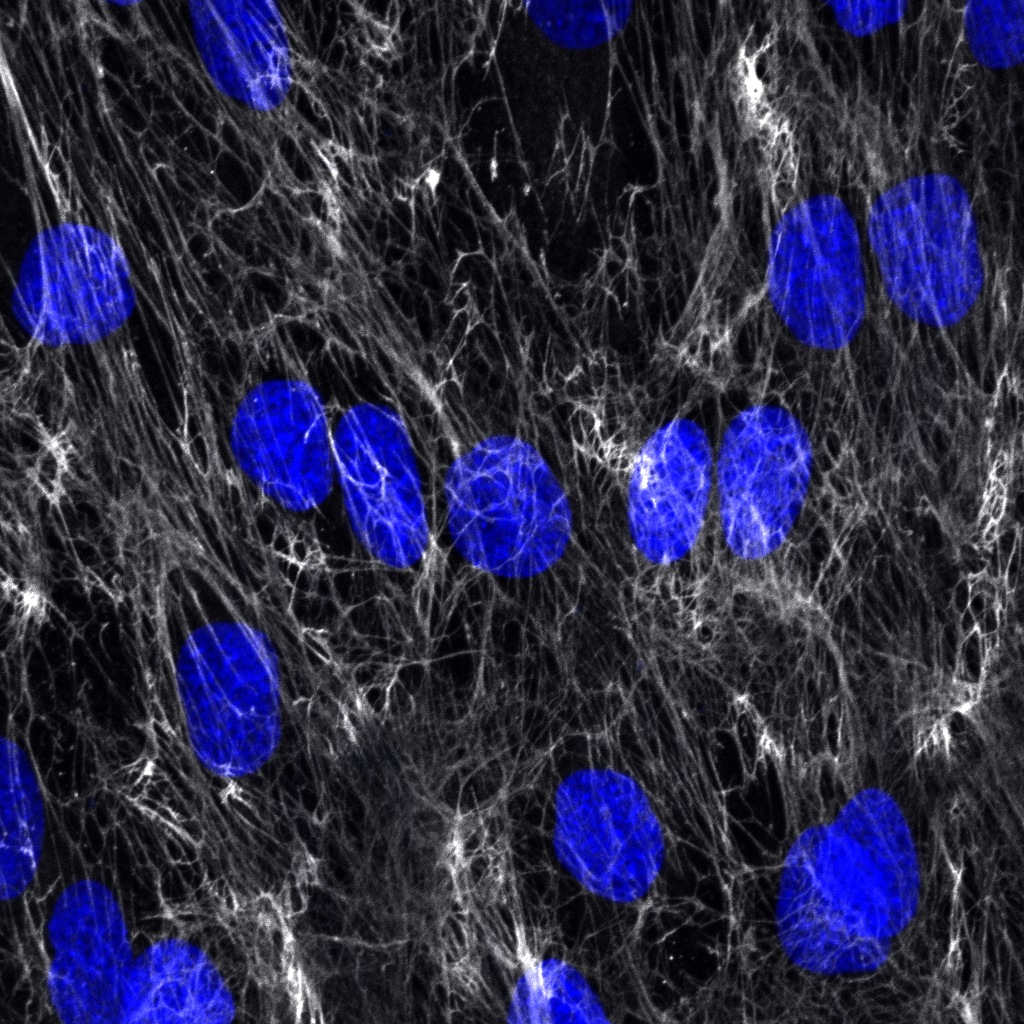

Supplement: Supplementary file 11 — Source data Fig. 7 [file 44319_2026_751_MOESM11_ESM.zip › Raw_data_Figure 7/Figure 7H/NFs FN nuclei.tif]

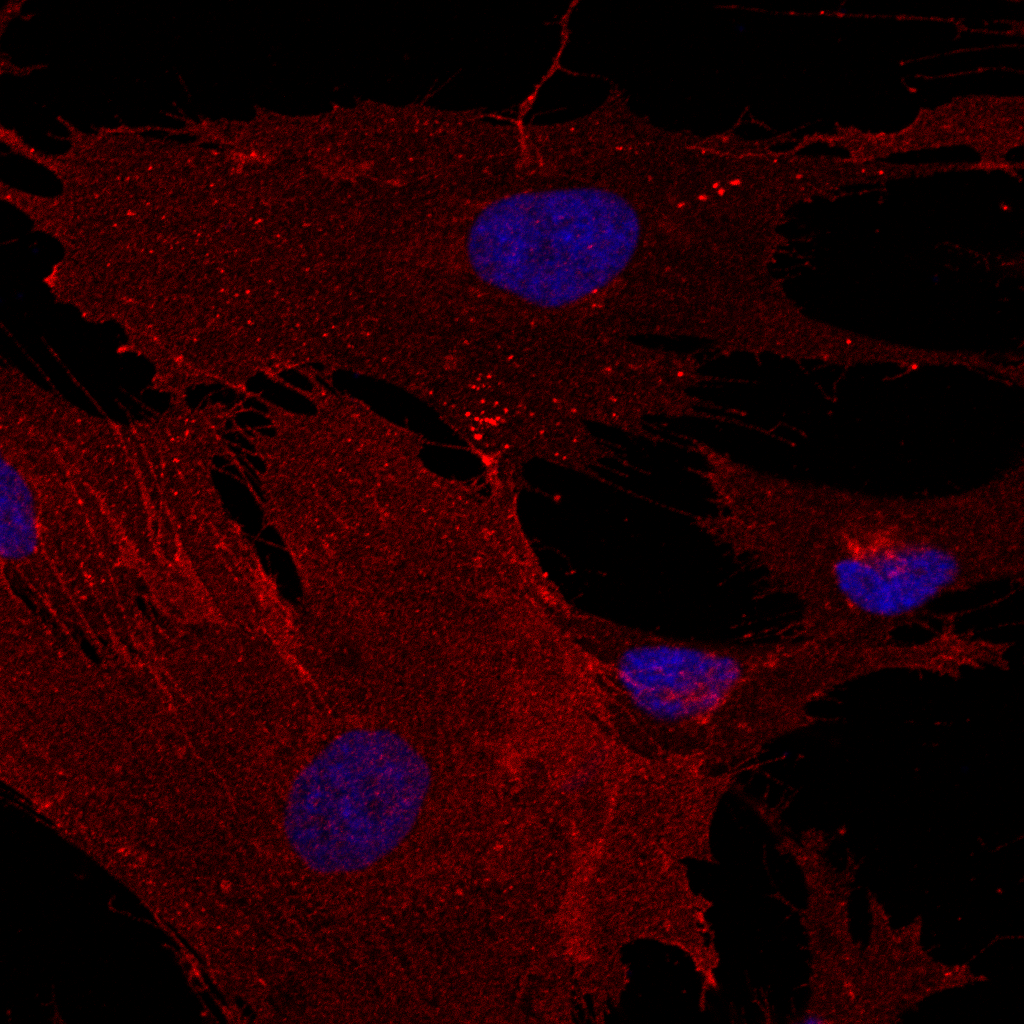

Supplement: Supplementary file 11 — Source data Fig. 7 [file 44319_2026_751_MOESM11_ESM.zip › Raw_data_Figure 7/Figure 7H/NFs PDGFR nuclei 24 h.tif]

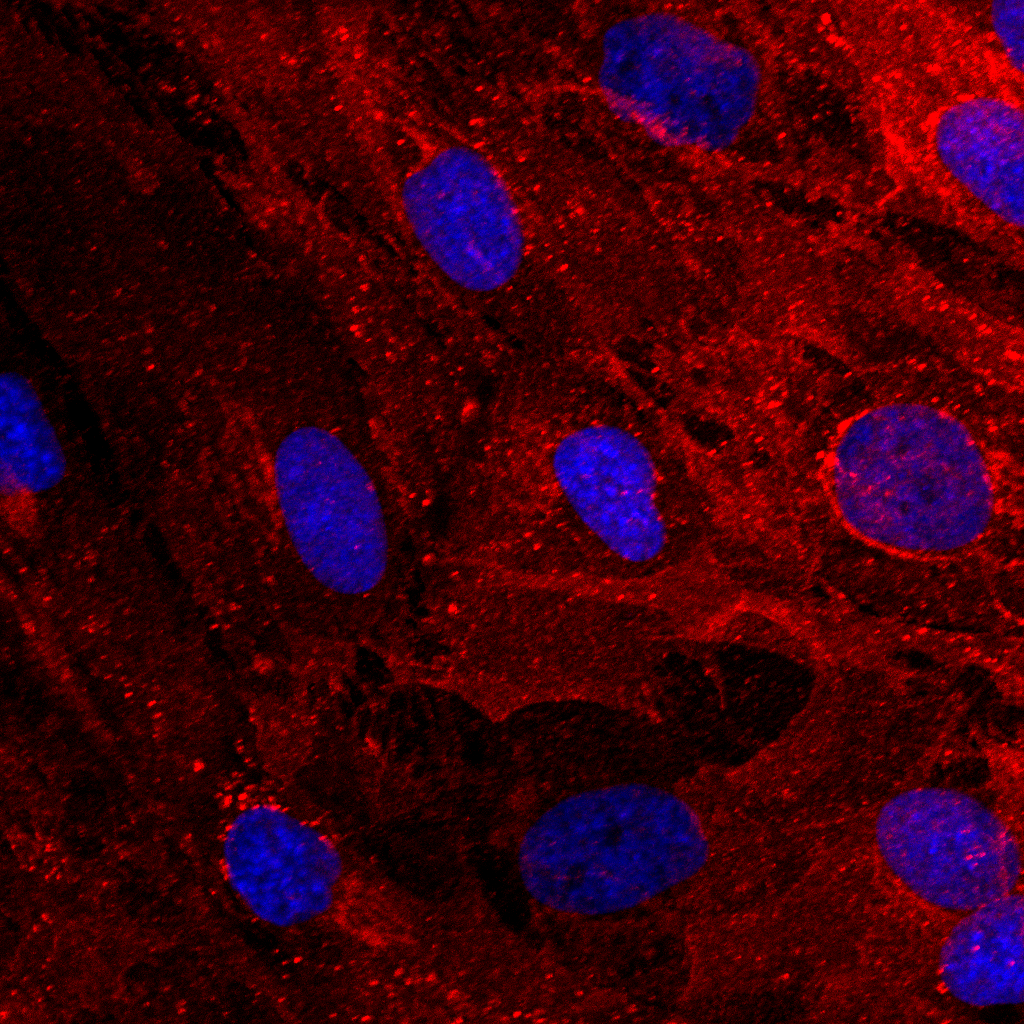

Supplement: Supplementary file 11 — Source data Fig. 7 [file 44319_2026_751_MOESM11_ESM.zip › Raw_data_Figure 7/Figure 7H/NFs PDGFR nuclei 72h.tif]

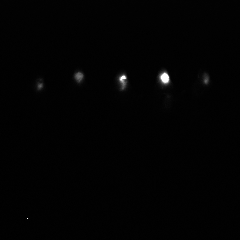

Supplement: Supplementary file 11 — Source data Fig. 7 [file 44319_2026_751_MOESM11_ESM.zip › Raw_data_Figure 7/Figure 7I/INL20231204083459_SEQ/INL20231204083459_001/luminescent.TIF]

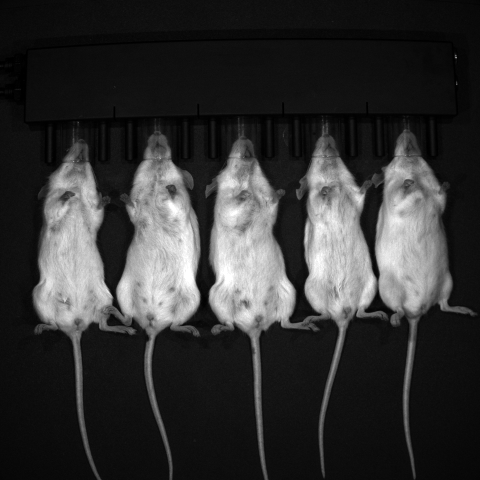

Supplement: Supplementary file 11 — Source data Fig. 7 [file 44319_2026_751_MOESM11_ESM.zip › Raw_data_Figure 7/Figure 7I/INL20231204083459_SEQ/INL20231204083459_001/photograph.TIF]

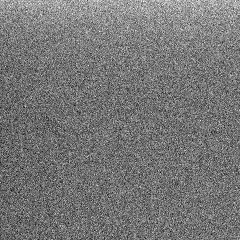

Supplement: Supplementary file 11 — Source data Fig. 7 [file 44319_2026_751_MOESM11_ESM.zip › Raw_data_Figure 7/Figure 7I/INL20231204083459_SEQ/INL20231204083459_001/readbiasonly.TIF]

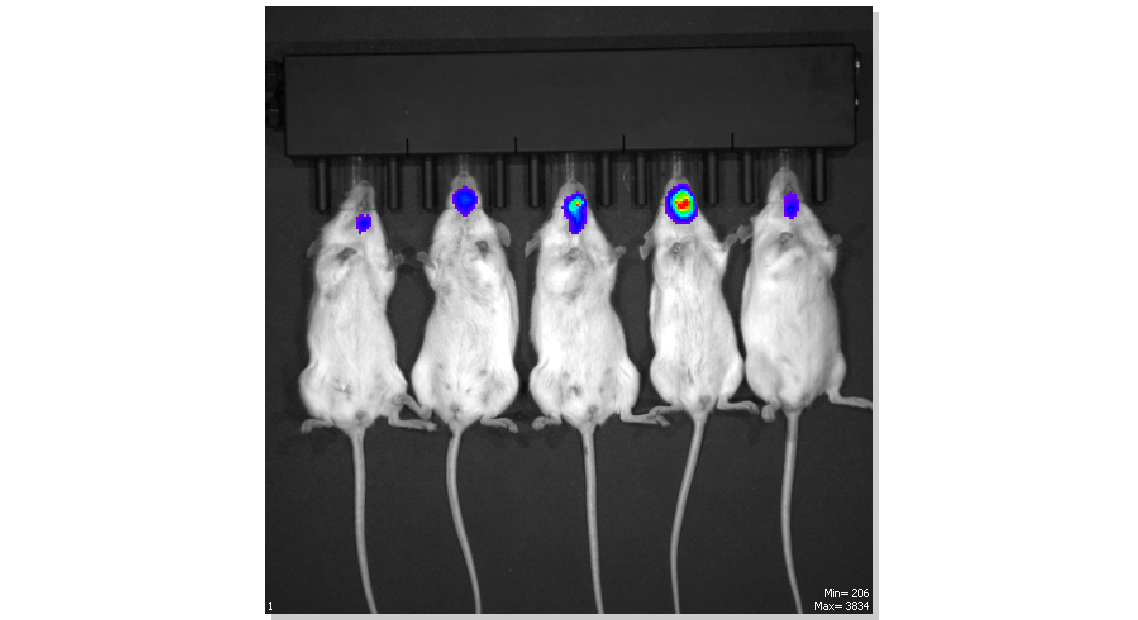

Supplement: Supplementary file 11 — Source data Fig. 7 [file 44319_2026_751_MOESM11_ESM.zip › Raw_data_Figure 7/Figure 7I/INL20231204083459_SEQ/INL20231204083459_SEQ.PNG]

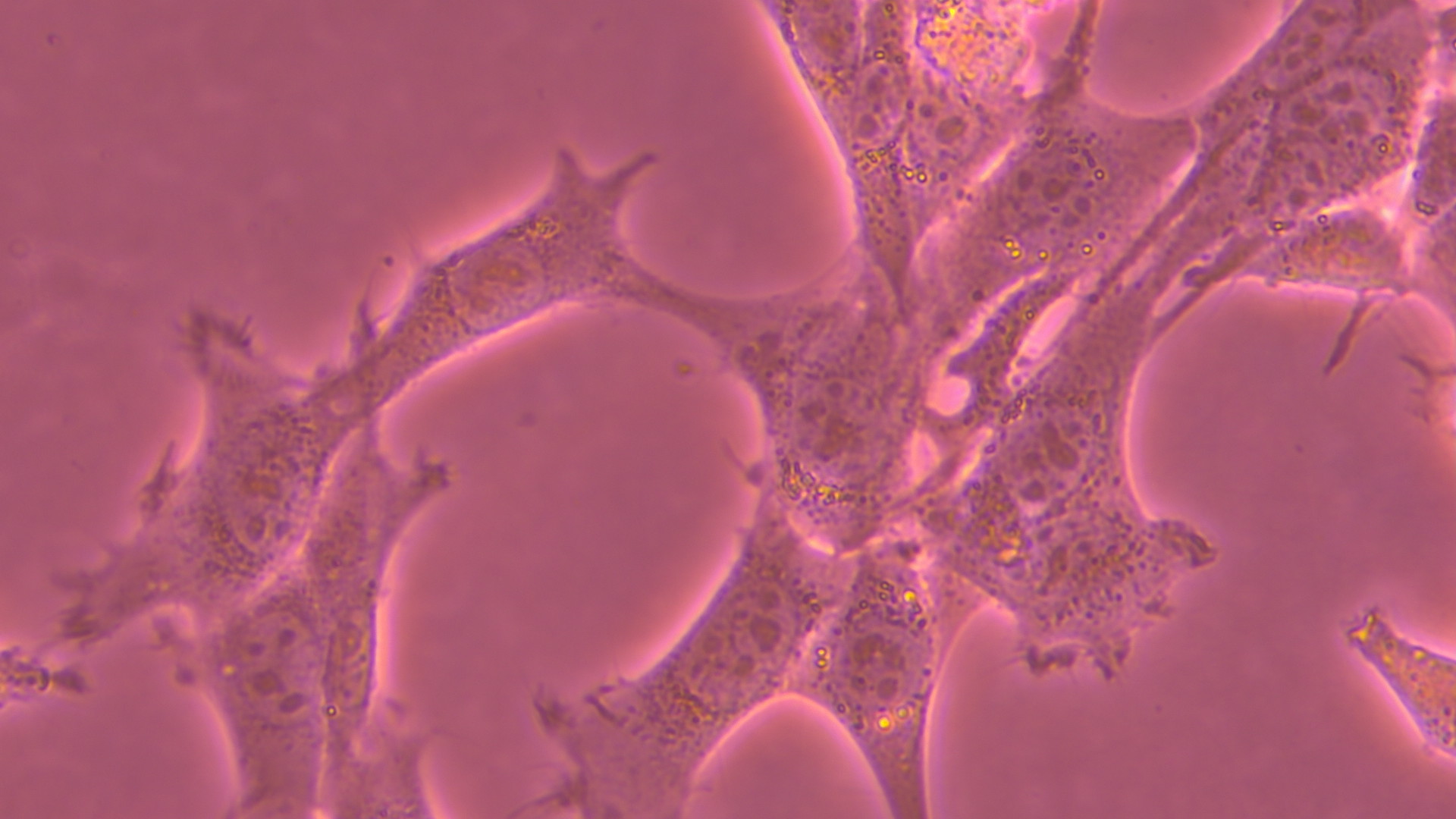

Supplement: Supplementary file 12 — Figure EV1 Source Data [file 44319_2026_751_MOESM12_ESM.zip › Raw_data_Figure EV1/Figure EV1A/ko7_1_2023230130103115o.jpg]

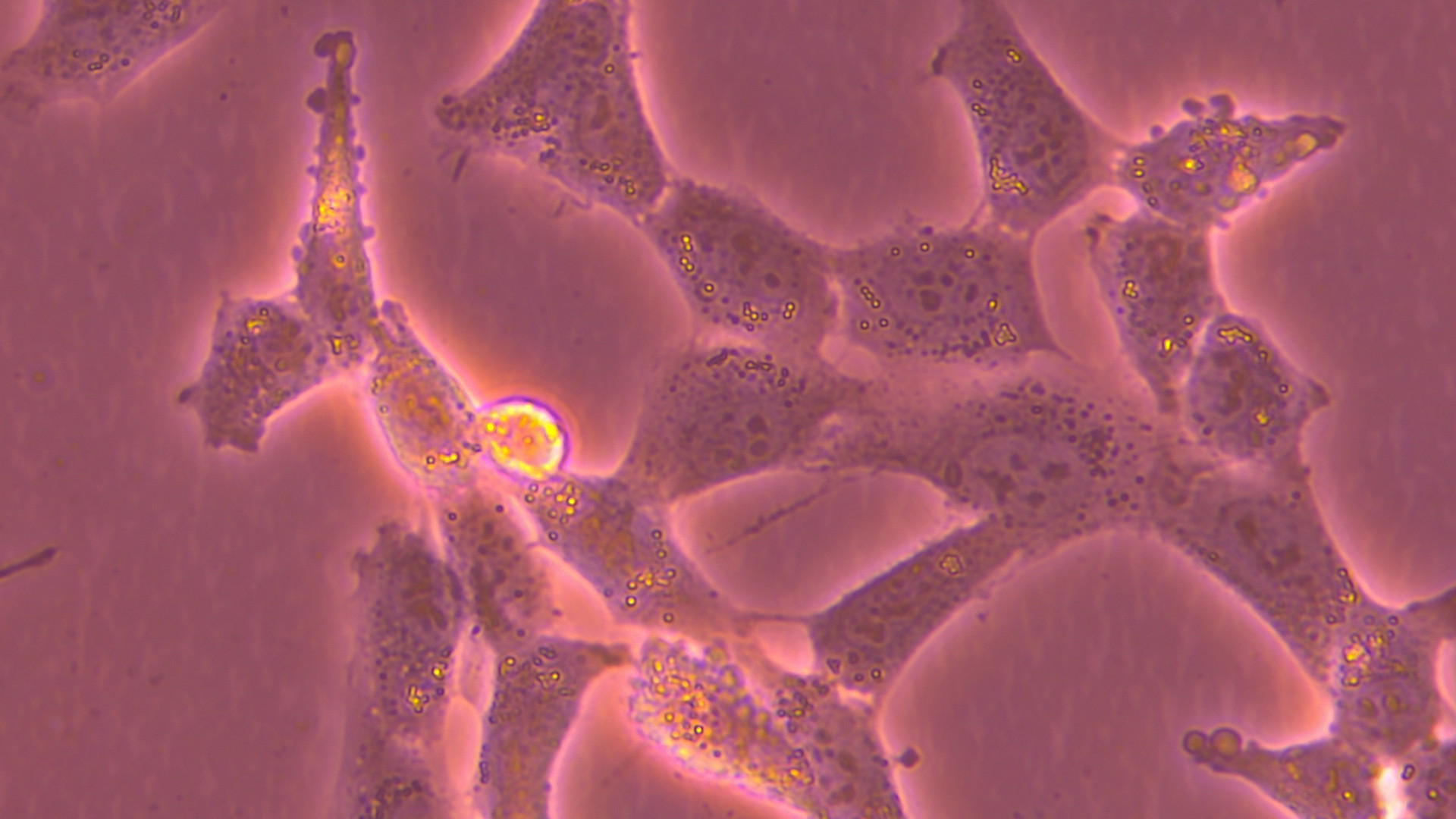

Supplement: Supplementary file 12 — Figure EV1 Source Data [file 44319_2026_751_MOESM12_ESM.zip › Raw_data_Figure EV1/Figure EV1A/wt7_1_2023230130103023o.jpg]

## Slide 1
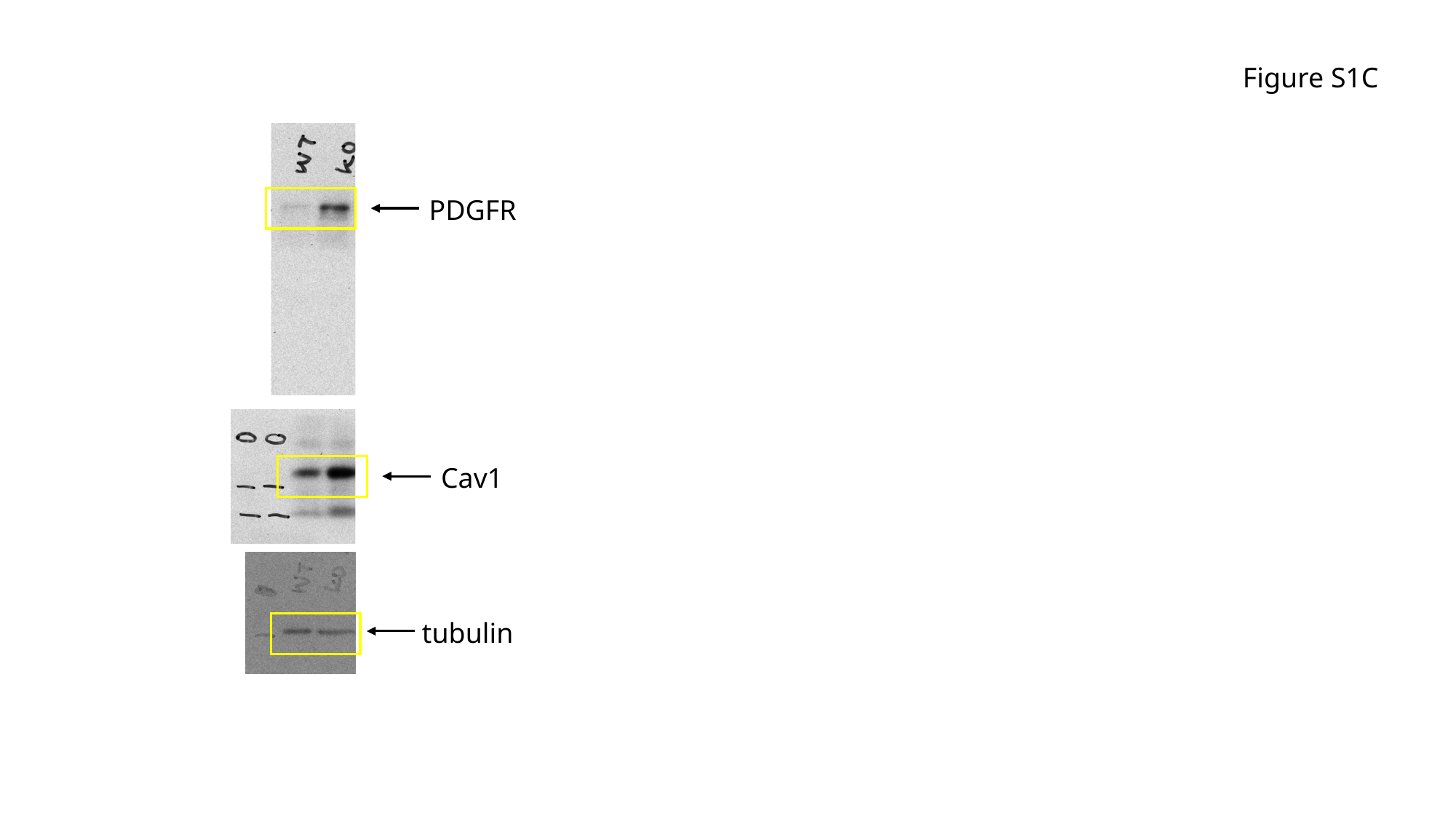

Figure S1C
PDGFR
Cav1
tubulin

Supplement: Supplementary file 12 — Figure EV1 Source Data [file 44319_2026_751_MOESM12_ESM.zip › Raw_data_Figure EV1/Figure EV1C/raw_blots_S1C.pptx]

## Slide 1
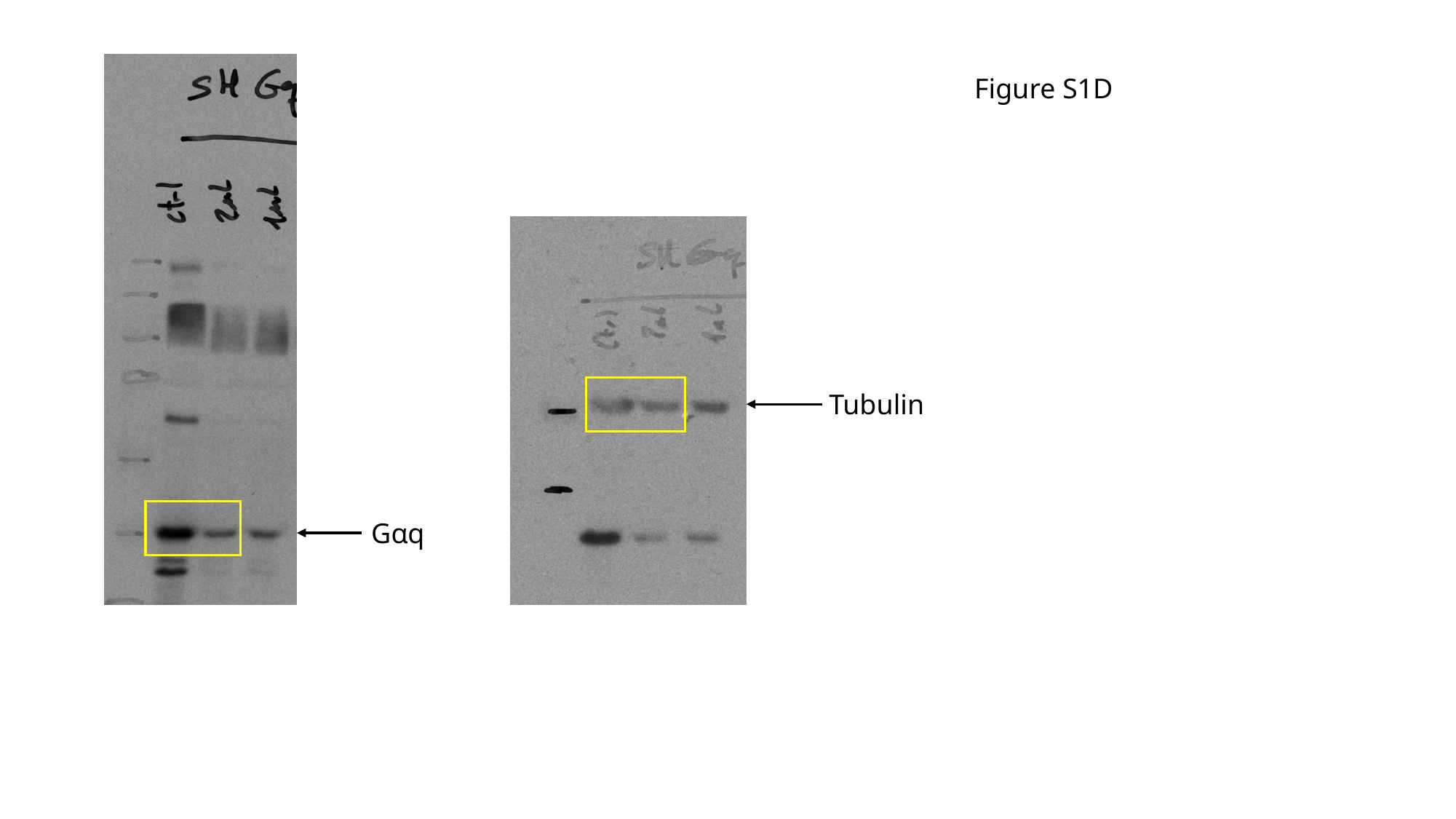

Figure S1D
Tubulin
Gαq

Supplement: Supplementary file 12 — Figure EV1 Source Data [file 44319_2026_751_MOESM12_ESM.zip › Raw_data_Figure EV1/Figure EV1D/raw_blots_EV1D.pptx]

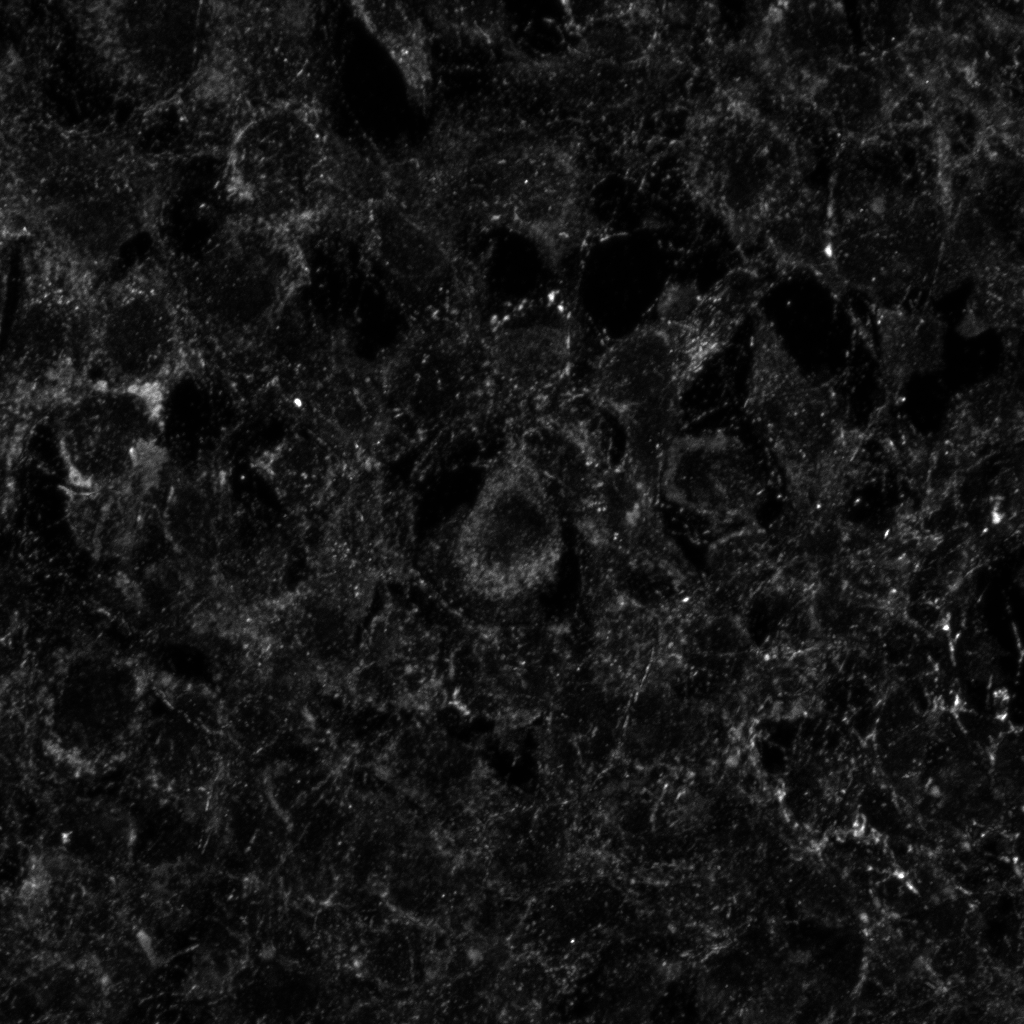

Supplement: Supplementary file 12 — Figure EV1 Source Data [file 44319_2026_751_MOESM12_ESM.zip › Raw_data_Figure EV1/Figure EV1D/WT sh control col I.tif]

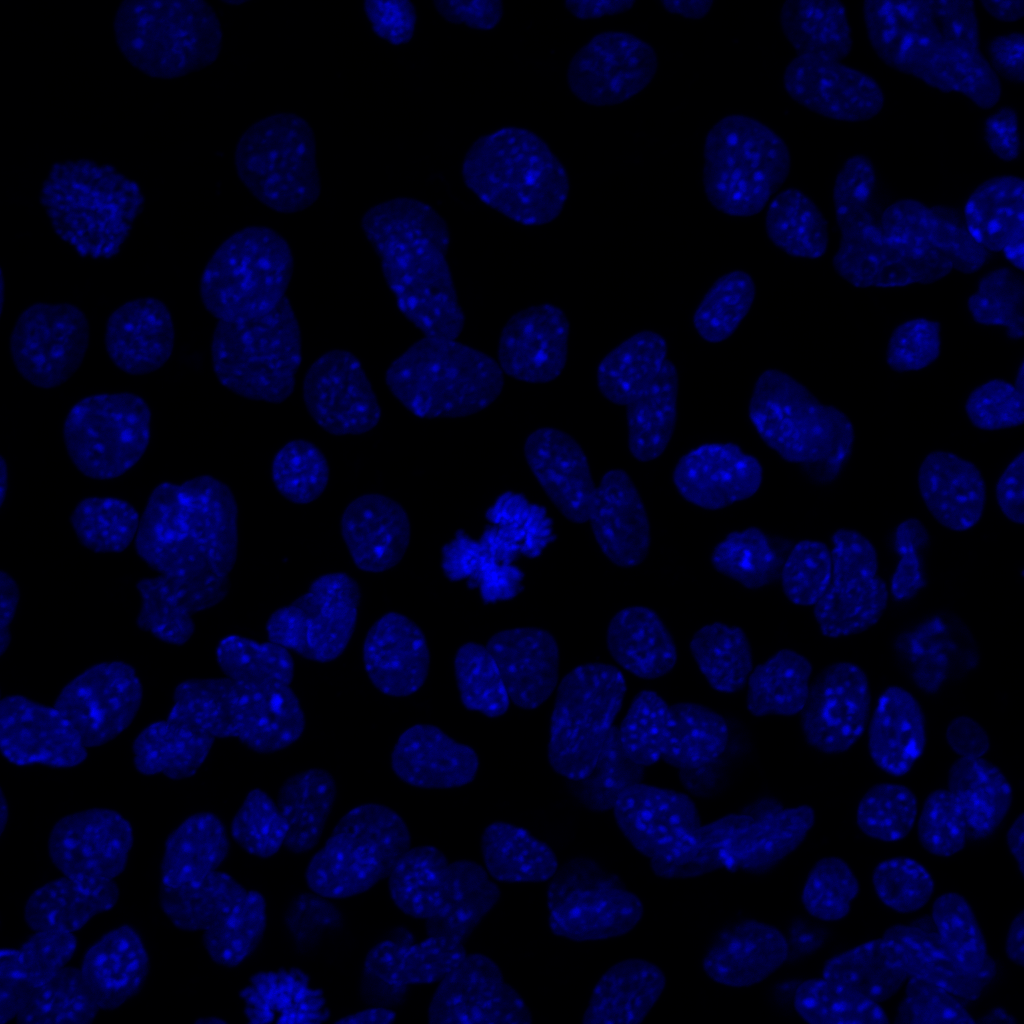

Supplement: Supplementary file 12 — Figure EV1 Source Data [file 44319_2026_751_MOESM12_ESM.zip › Raw_data_Figure EV1/Figure EV1D/WT sh control nuclei.tif]

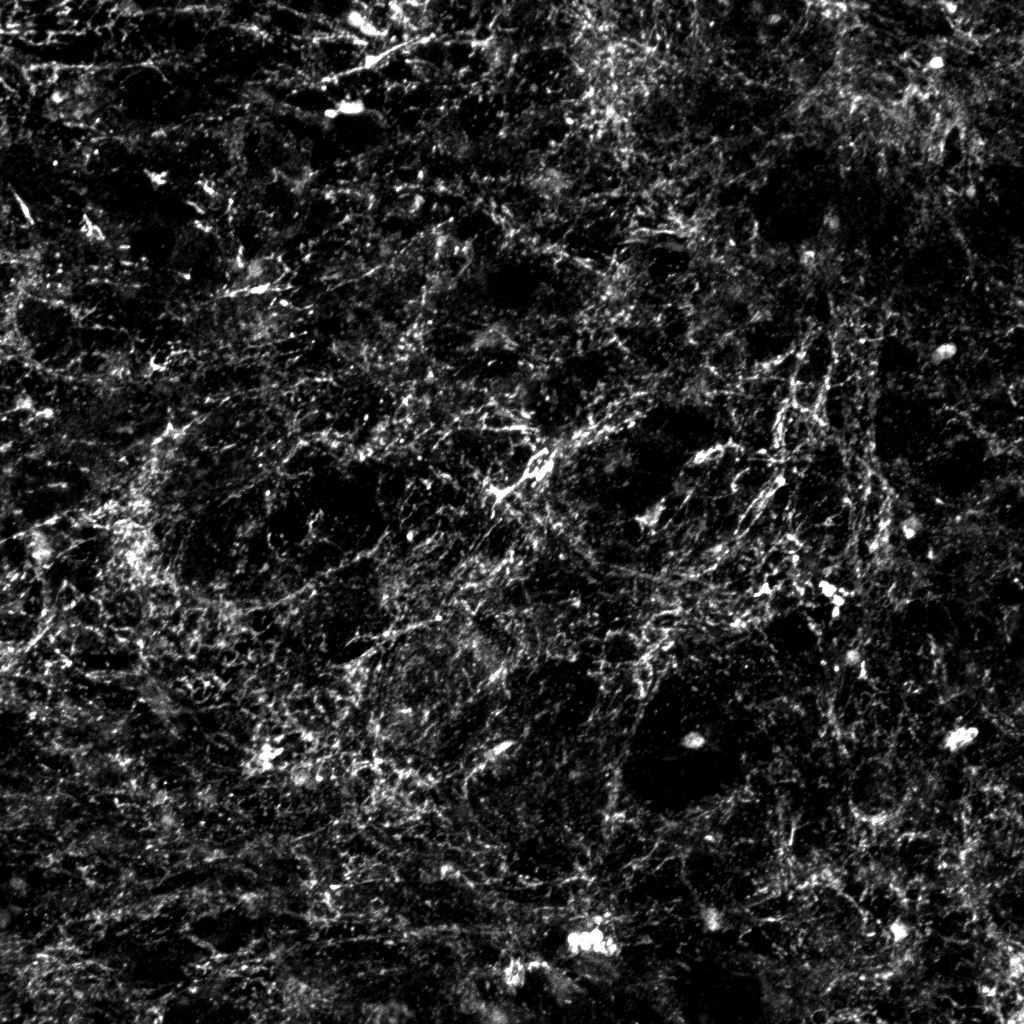

Supplement: Supplementary file 12 — Figure EV1 Source Data [file 44319_2026_751_MOESM12_ESM.zip › Raw_data_Figure EV1/Figure EV1D/WT shGq col I.tif]

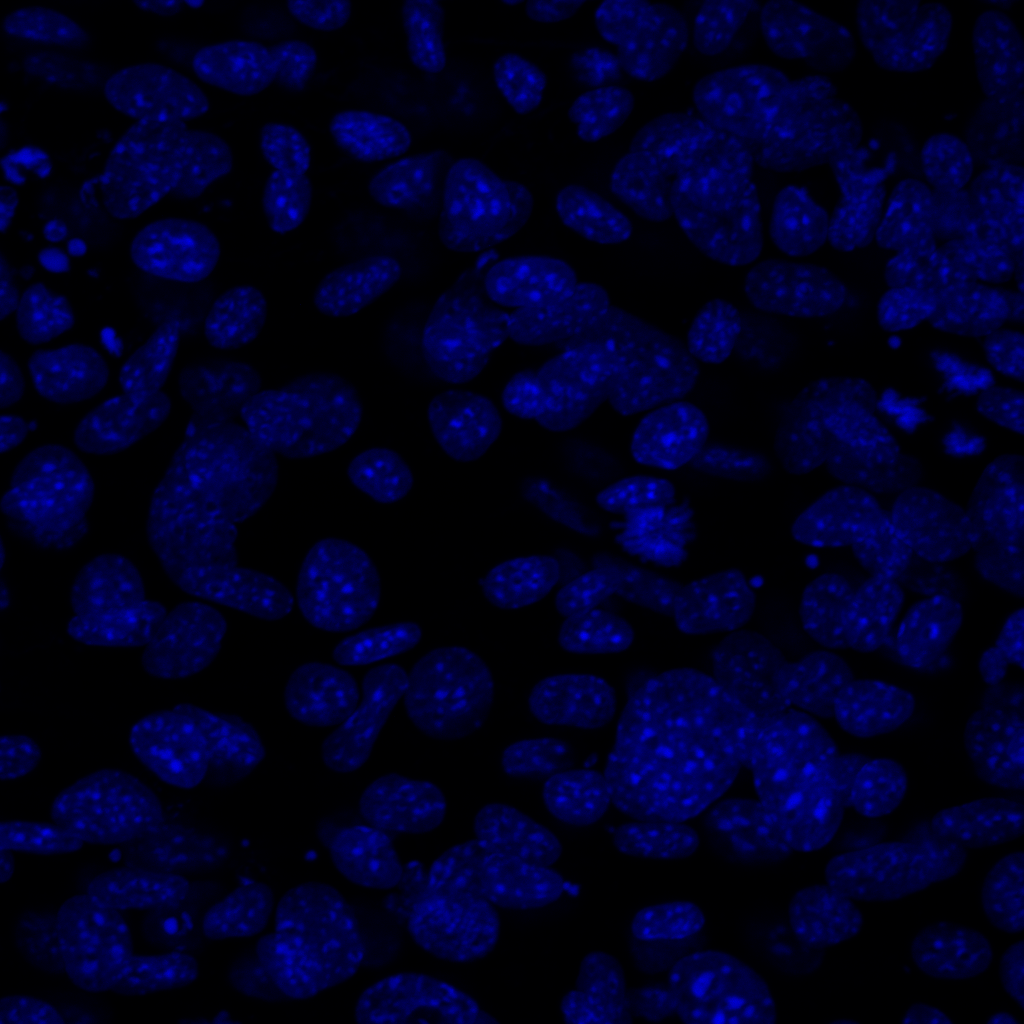

Supplement: Supplementary file 12 — Figure EV1 Source Data [file 44319_2026_751_MOESM12_ESM.zip › Raw_data_Figure EV1/Figure EV1D/WT shGq nuclei.tif]

## Slide 1
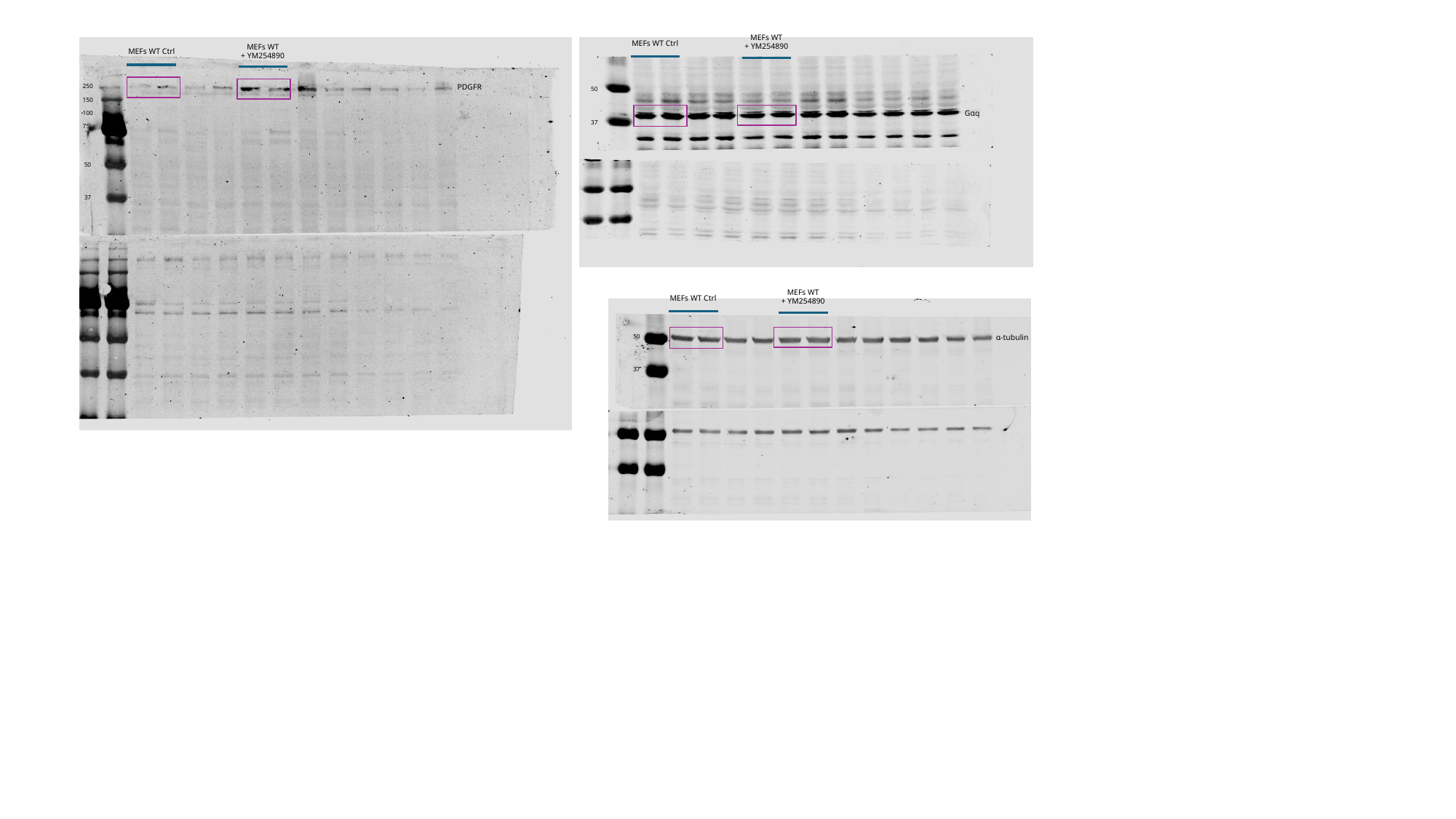

MEFs WT
+ YM254890
MEFs WT Ctrl
MEFs WT
+ YM254890
MEFs WT Ctrl
250
PDGFR
150
100
75
50
37
50
Gαq
37
MEFs WT
+ YM254890
MEFs WT Ctrl
50
α-tubulin
37

Supplement: Supplementary file 12 — Figure EV1 Source Data [file 44319_2026_751_MOESM12_ESM.zip › Raw_data_Figure EV1/Figure EV1E/raw_blots_S1E.pptx]

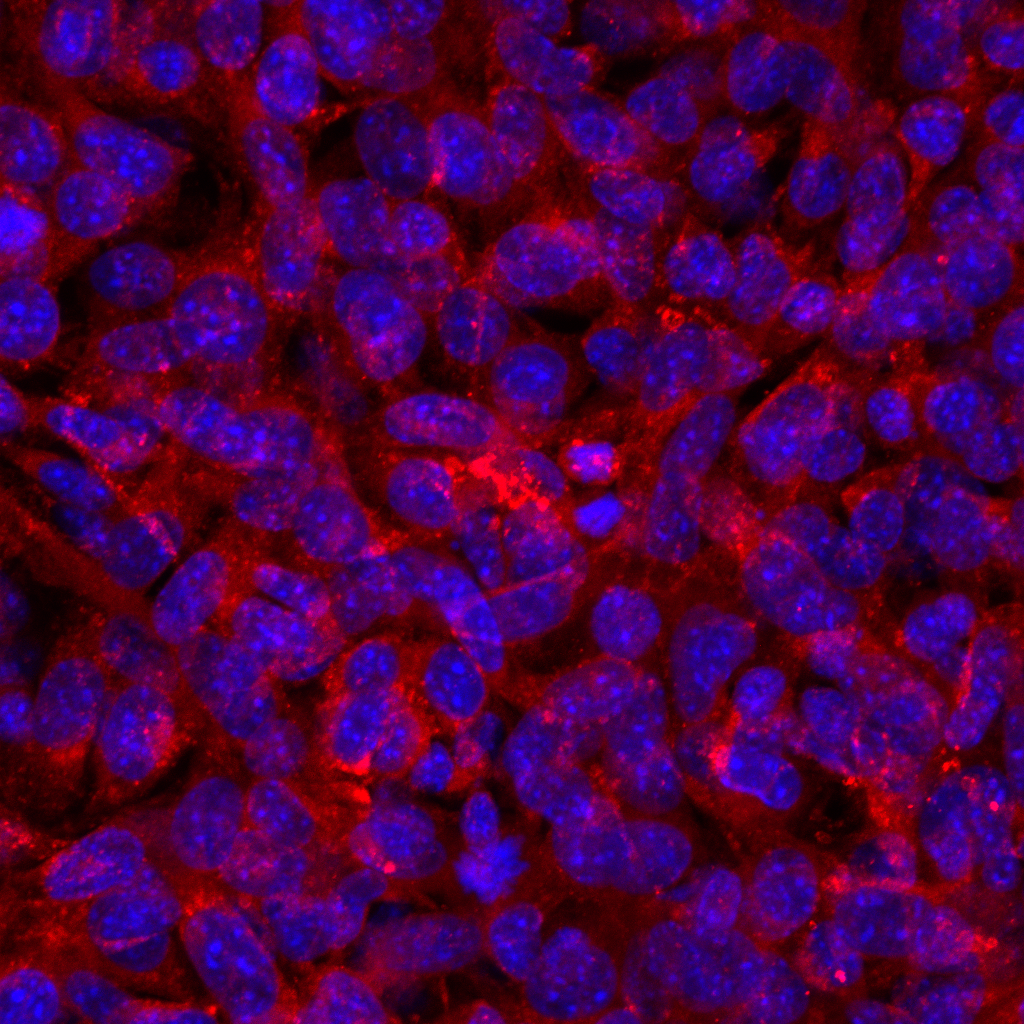

Supplement: Supplementary file 12 — Figure EV1 Source Data [file 44319_2026_751_MOESM12_ESM.zip › Raw_data_Figure EV1/Figure EV1F/C1-C3-MAX_WT MEFs FN555 LAMP1 647.tif]

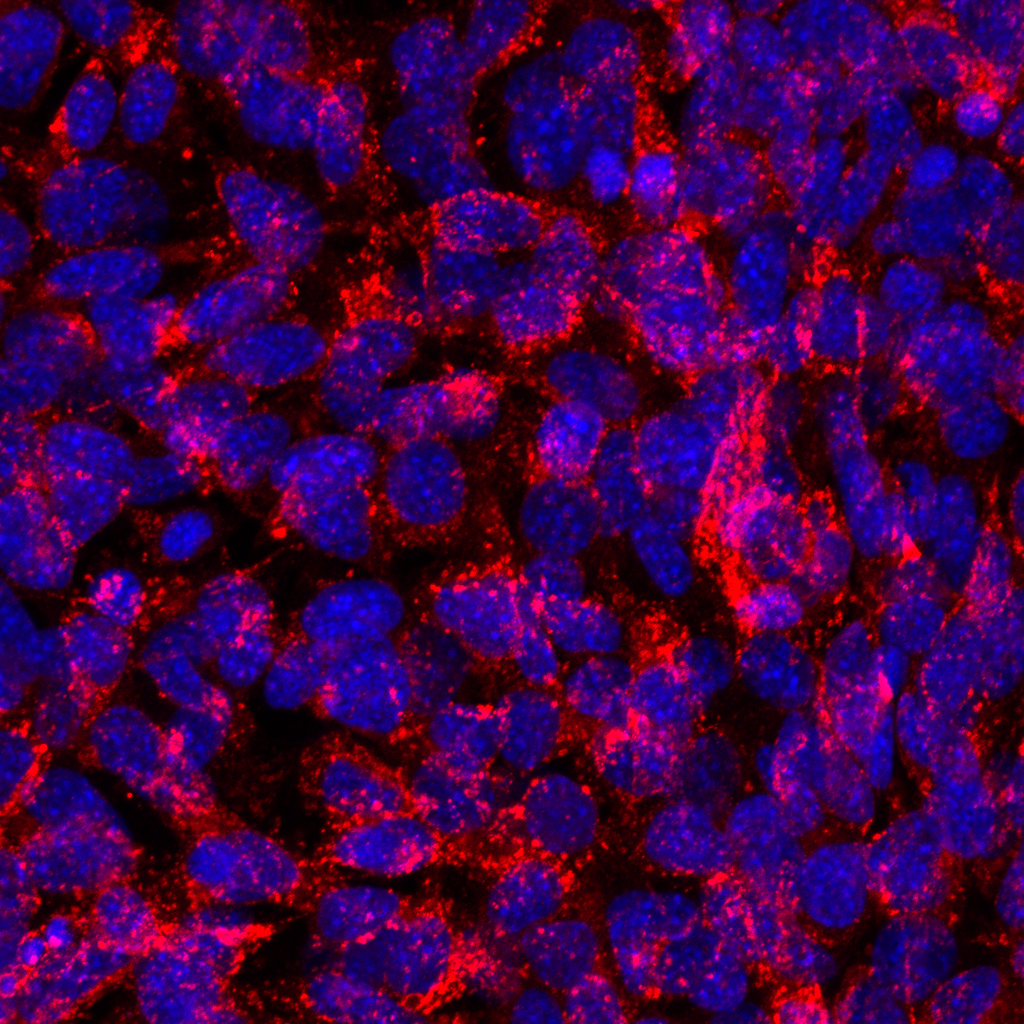

Supplement: Supplementary file 12 — Figure EV1 Source Data [file 44319_2026_751_MOESM12_ESM.zip › Raw_data_Figure EV1/Figure EV1F/C1_C3-MAX_WT MEFs + YM FN555 LAMP1 647.tif]

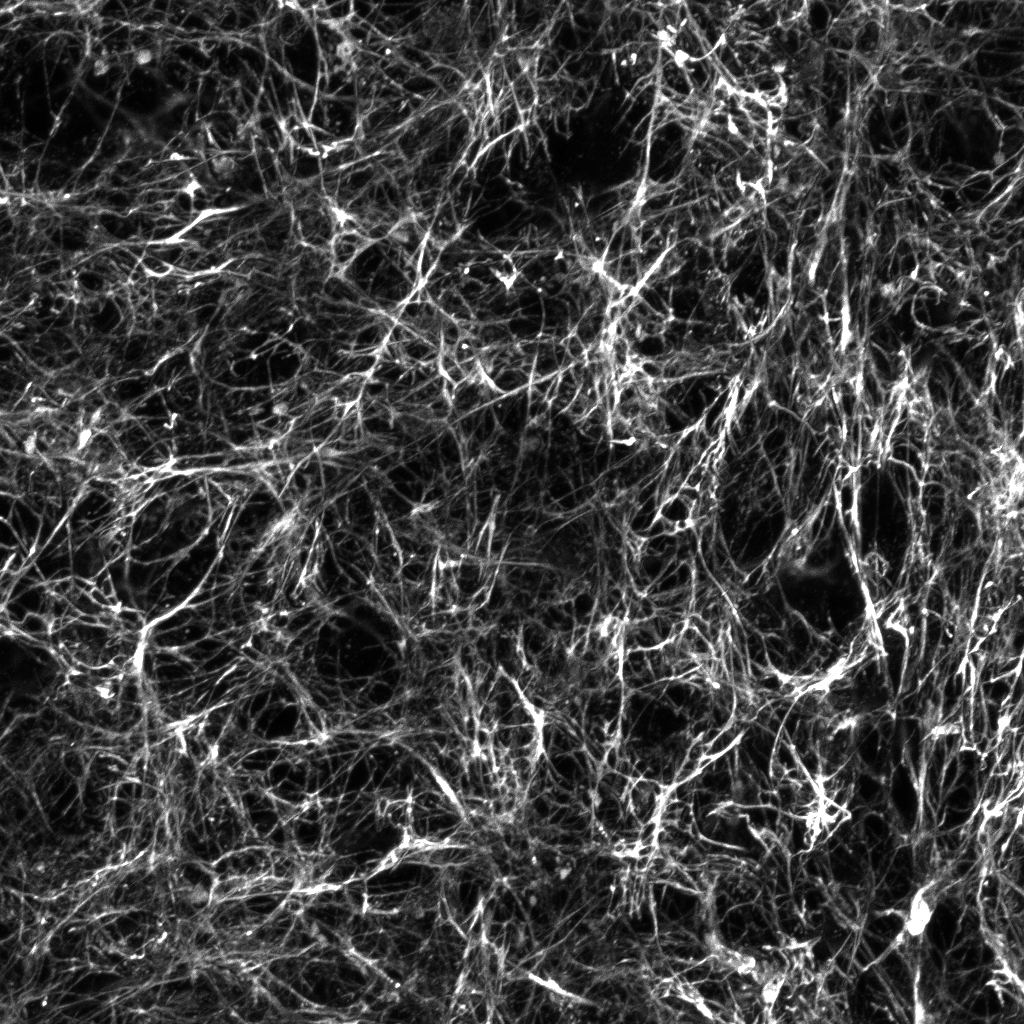

Supplement: Supplementary file 12 — Figure EV1 Source Data [file 44319_2026_751_MOESM12_ESM.zip › Raw_data_Figure EV1/Figure EV1F/C2-MAX_WT MEFs + YM FN555 LAMP1 647.tif]

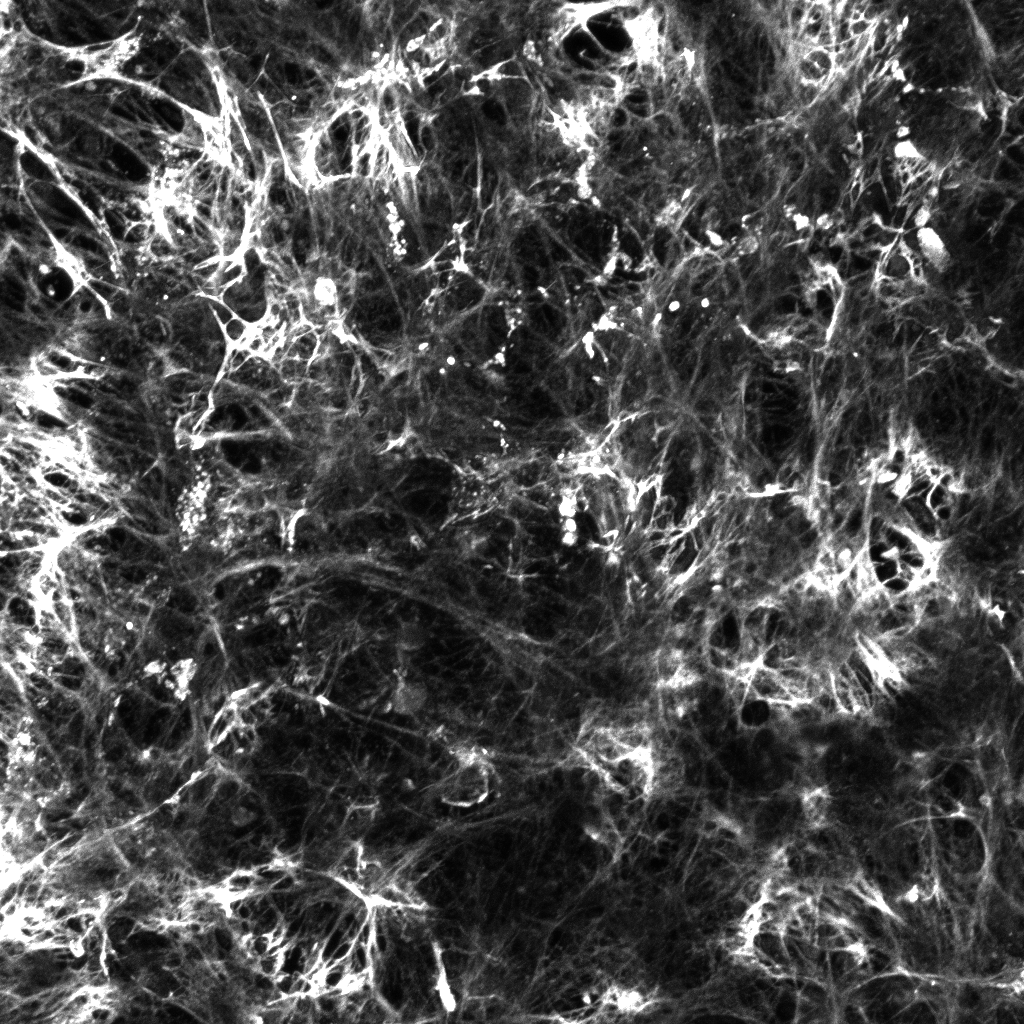

Supplement: Supplementary file 12 — Figure EV1 Source Data [file 44319_2026_751_MOESM12_ESM.zip › Raw_data_Figure EV1/Figure EV1F/C2-MAX_WT MEFs FN555 LAMP1 647.tif]

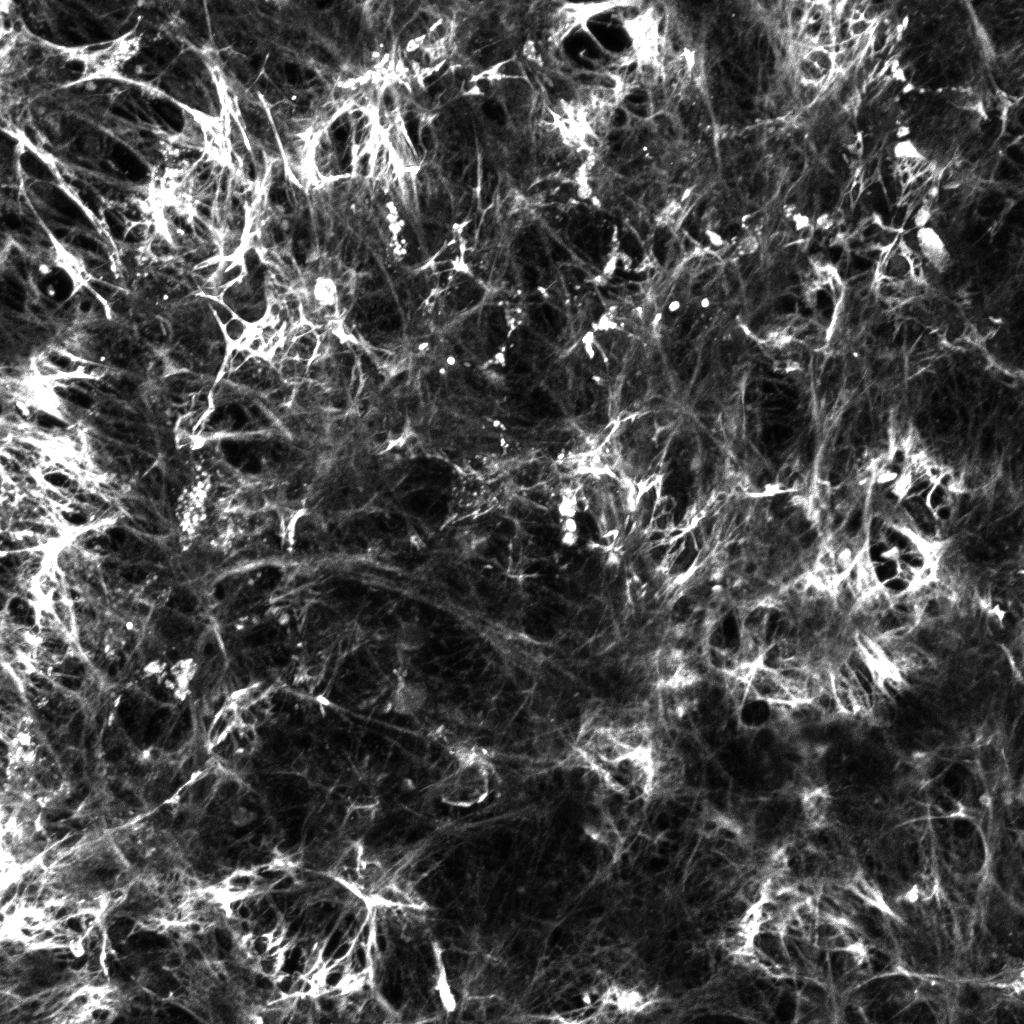

Supplement: Supplementary file 12 — Figure EV1 Source Data [file 44319_2026_751_MOESM12_ESM.zip › Raw_data_Figure EV1/Figure EV1F/WT control FN.tif]

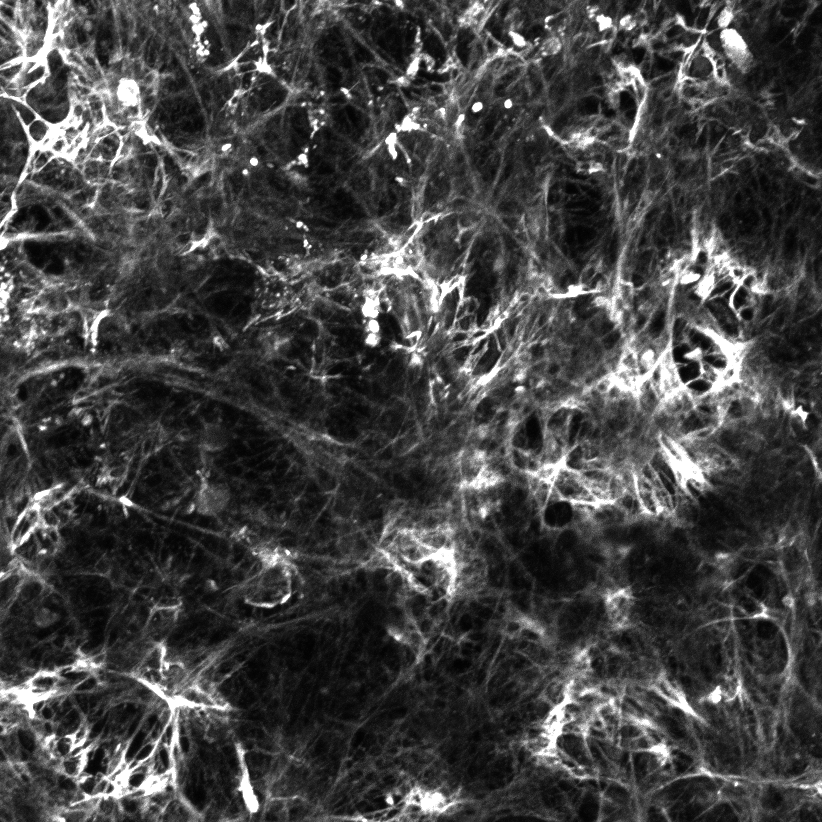

Supplement: Supplementary file 12 — Figure EV1 Source Data [file 44319_2026_751_MOESM12_ESM.zip › Raw_data_Figure EV1/Figure EV1F/WT control FNcropped.tif]

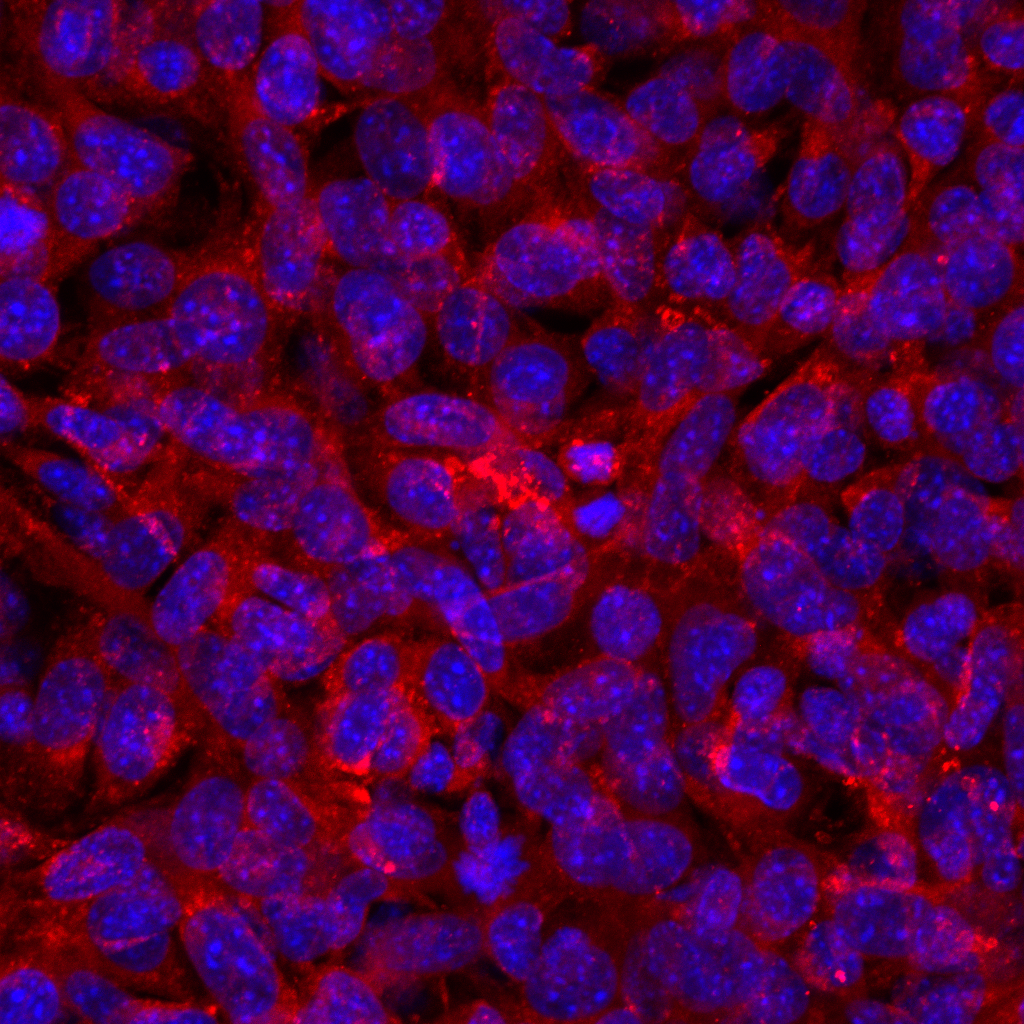

Supplement: Supplementary file 12 — Figure EV1 Source Data [file 44319_2026_751_MOESM12_ESM.zip › Raw_data_Figure EV1/Figure EV1F/WT control LAMP1 nuclei.tif]

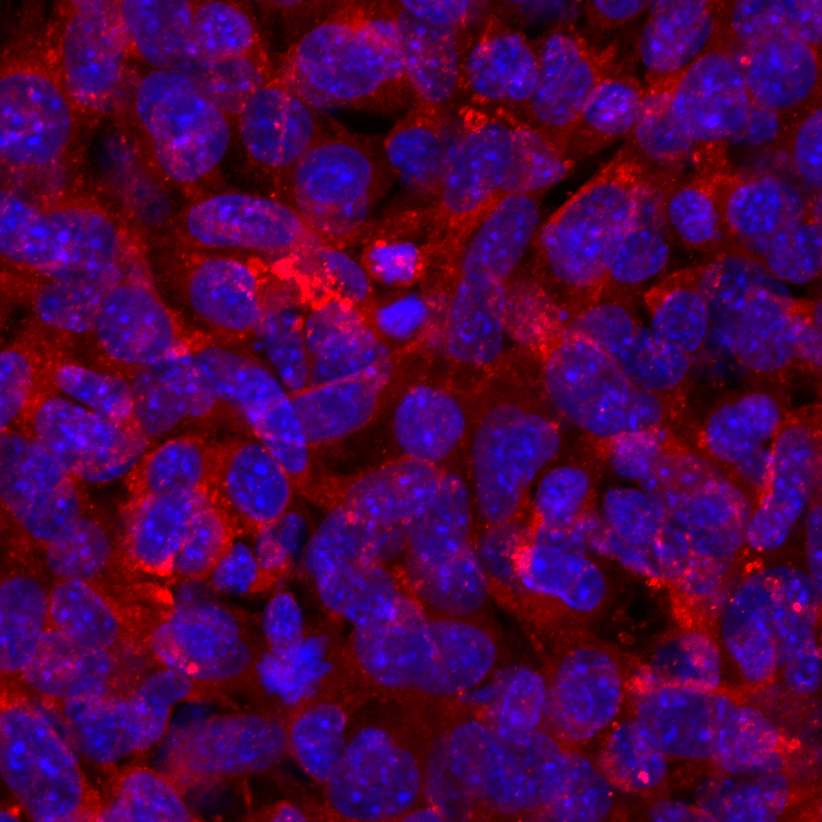

Supplement: Supplementary file 12 — Figure EV1 Source Data [file 44319_2026_751_MOESM12_ESM.zip › Raw_data_Figure EV1/Figure EV1F/WT control LAMP1 nucleicropped.tif]

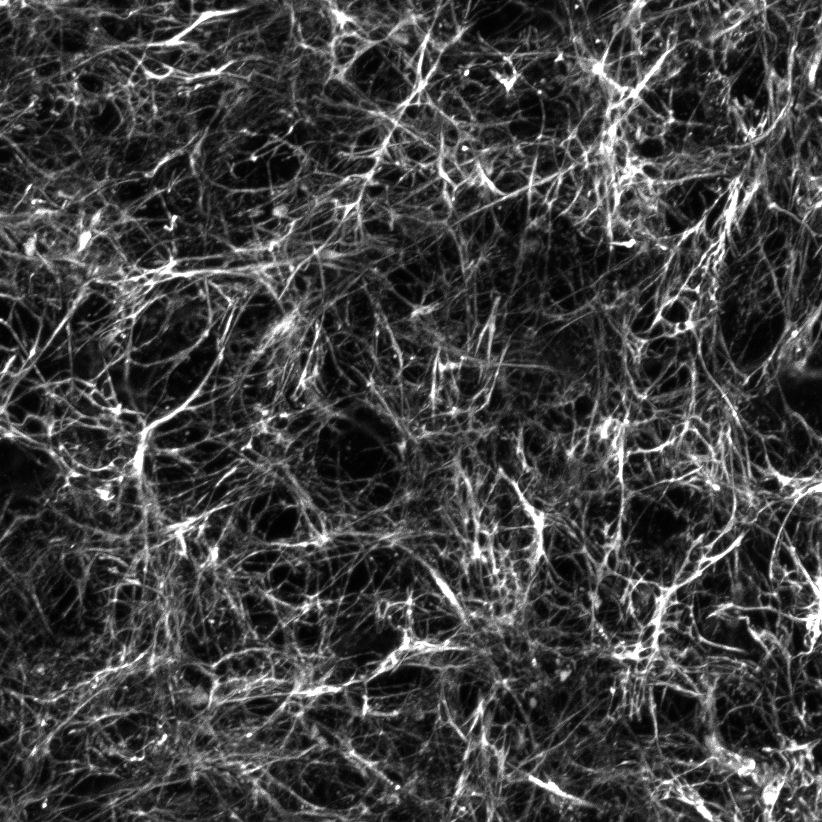

Supplement: Supplementary file 12 — Figure EV1 Source Data [file 44319_2026_751_MOESM12_ESM.zip › Raw_data_Figure EV1/Figure EV1F/WT+YM FN cropped.tif]

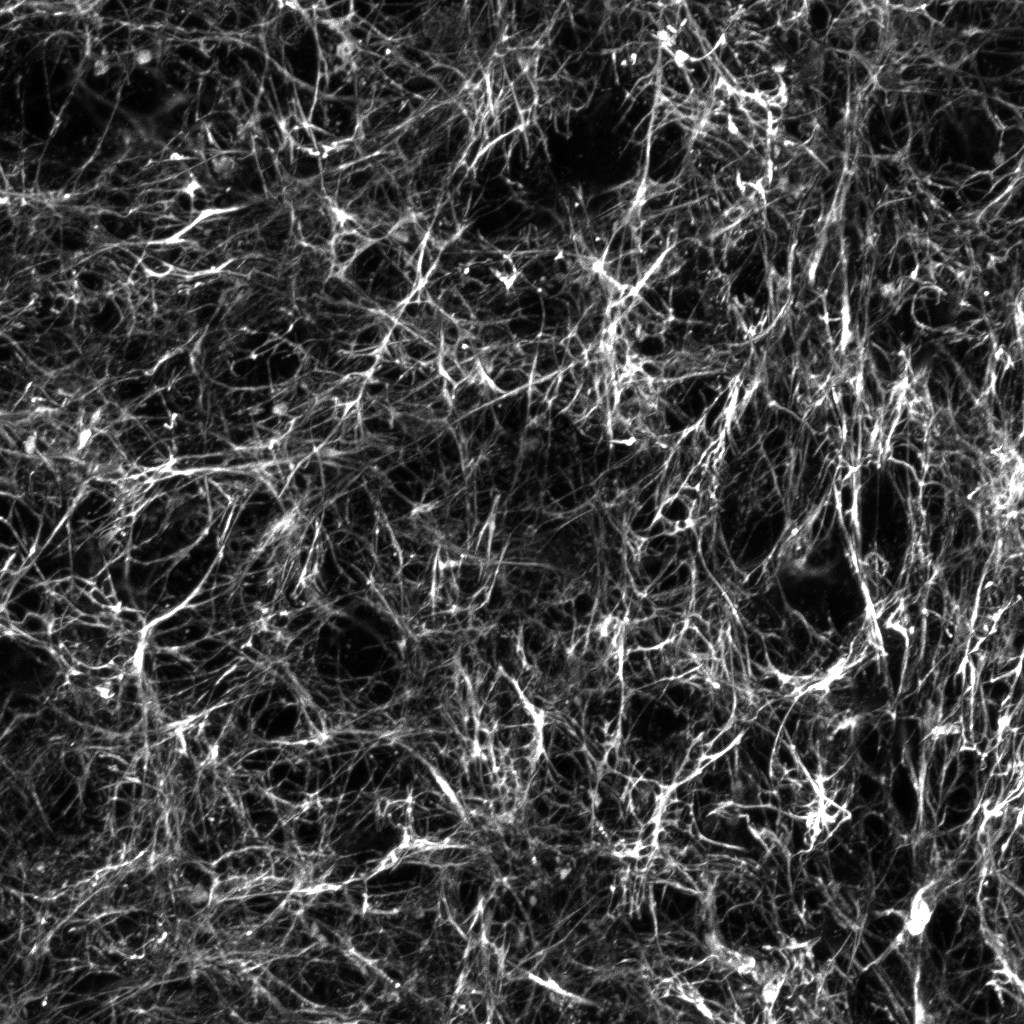

Supplement: Supplementary file 12 — Figure EV1 Source Data [file 44319_2026_751_MOESM12_ESM.zip › Raw_data_Figure EV1/Figure EV1F/WT+YM FN.tif]

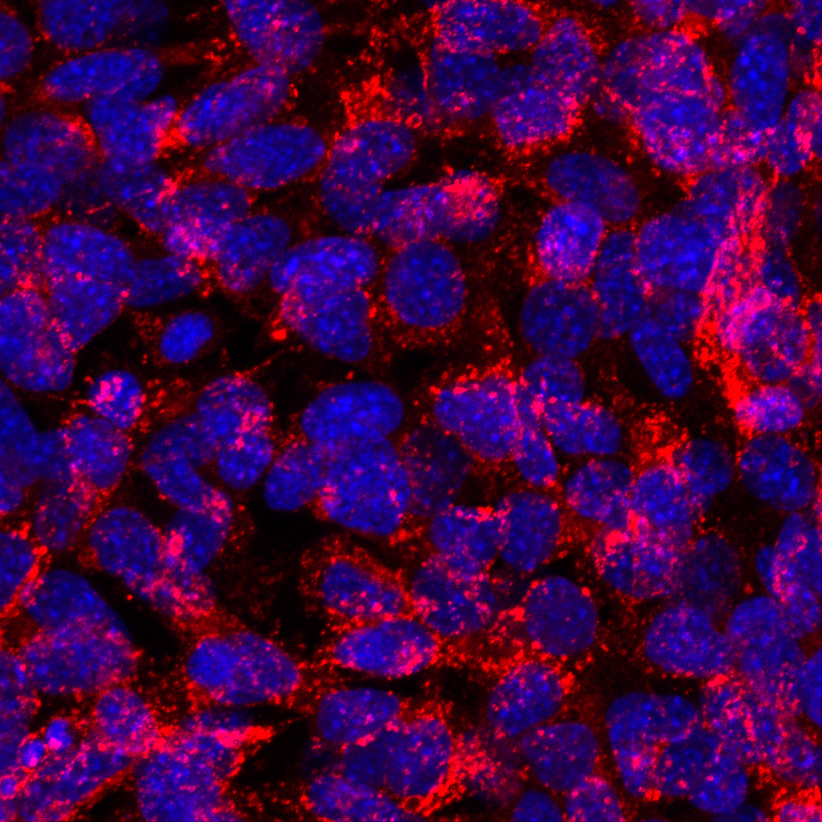

Supplement: Supplementary file 12 — Figure EV1 Source Data [file 44319_2026_751_MOESM12_ESM.zip › Raw_data_Figure EV1/Figure EV1F/WT+YM LAMP1 nuclei cropped.tif]

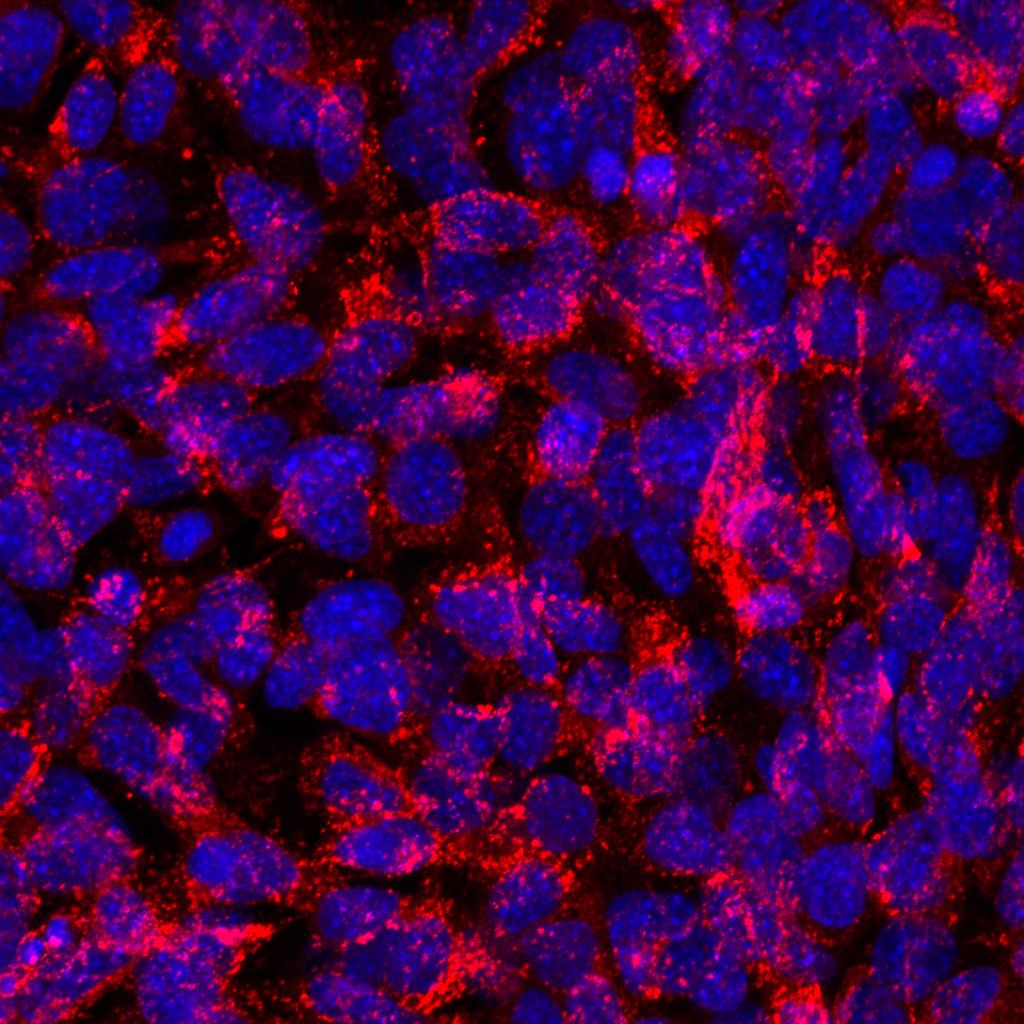

Supplement: Supplementary file 12 — Figure EV1 Source Data [file 44319_2026_751_MOESM12_ESM.zip › Raw_data_Figure EV1/Figure EV1F/WT+YM LAMP1 nuclei.tif]

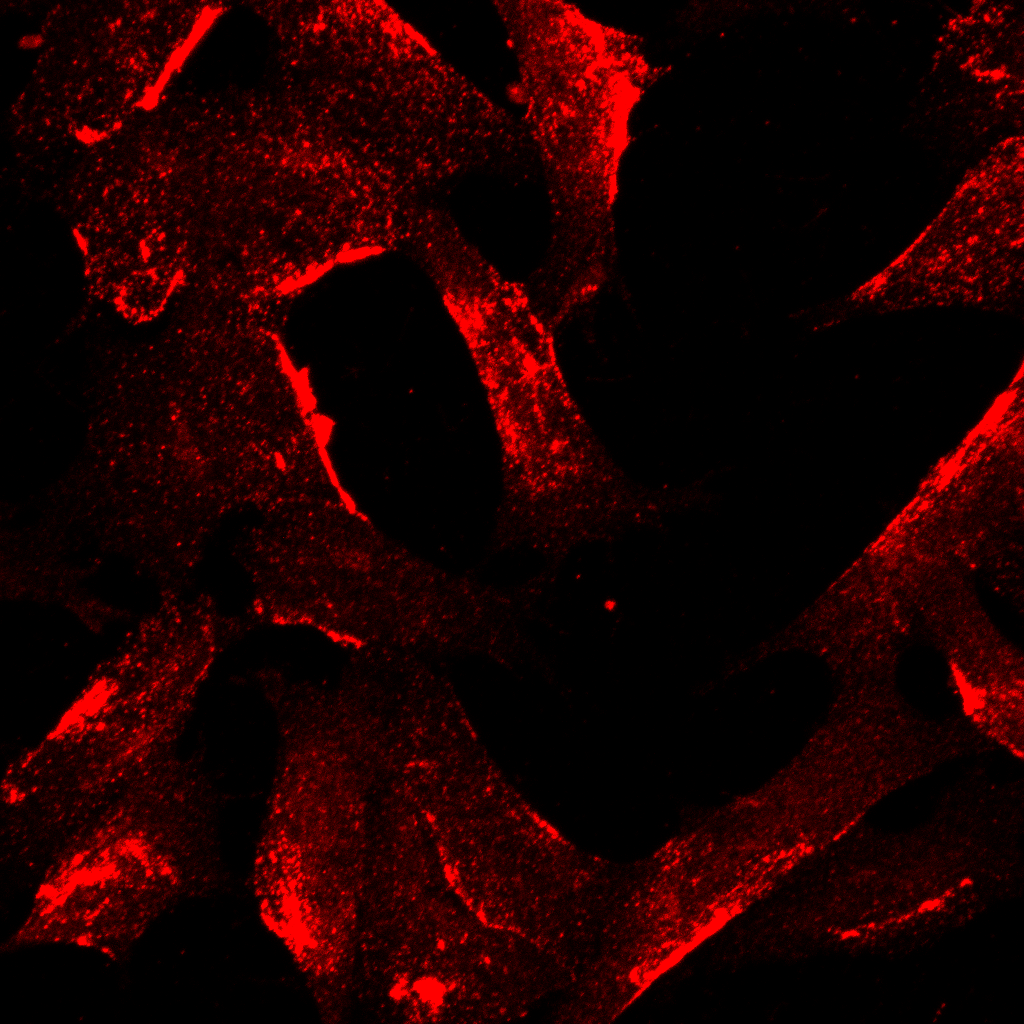

Supplement: Supplementary file 12 — Figure EV1 Source Data [file 44319_2026_751_MOESM12_ESM.zip › Raw_data_Figure EV1/Figure EV1G/C3-WT ctrl Cav1 LAMP 4_Maximum intensity projection.tif]

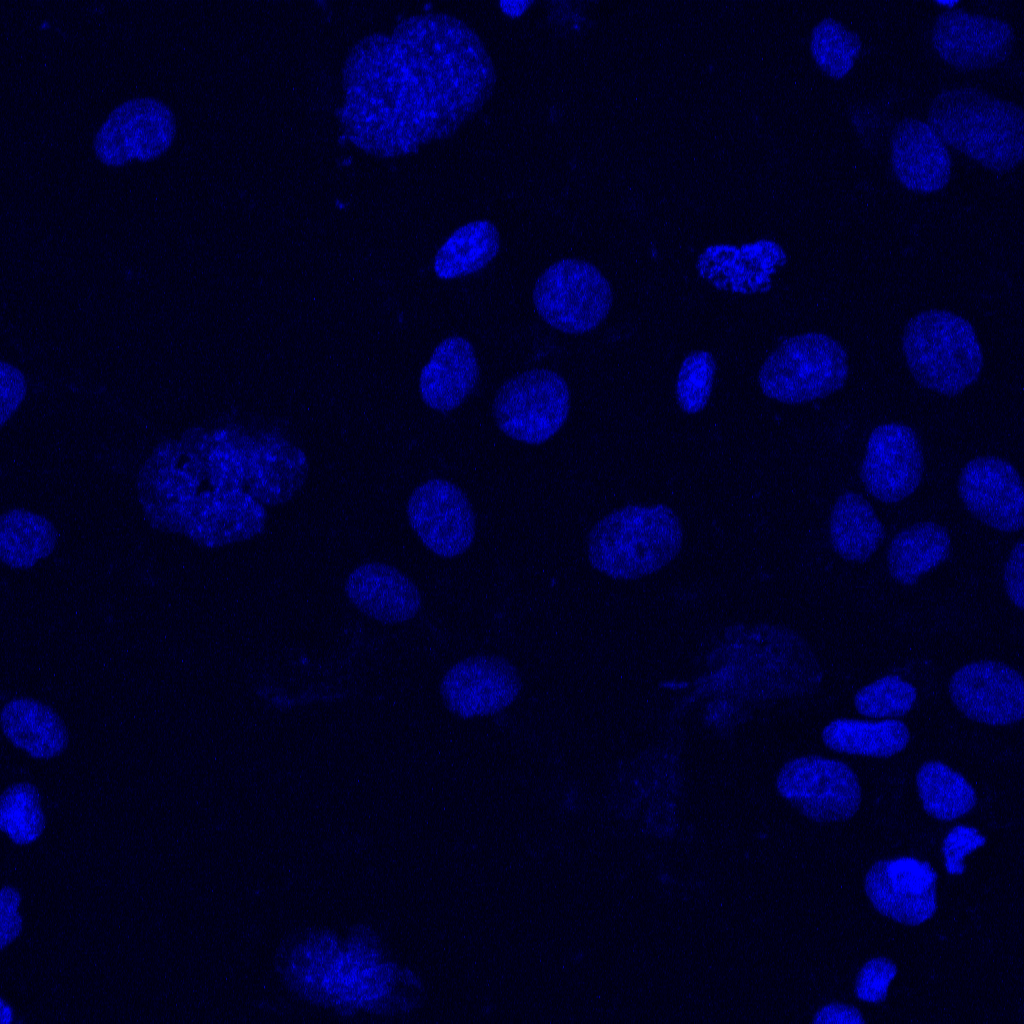

Supplement: Supplementary file 12 — Figure EV1 Source Data [file 44319_2026_751_MOESM12_ESM.zip › Raw_data_Figure EV1/Figure EV1H/C1-MAX_HN13 + fibros KO SMA Factin Ecad.tif]

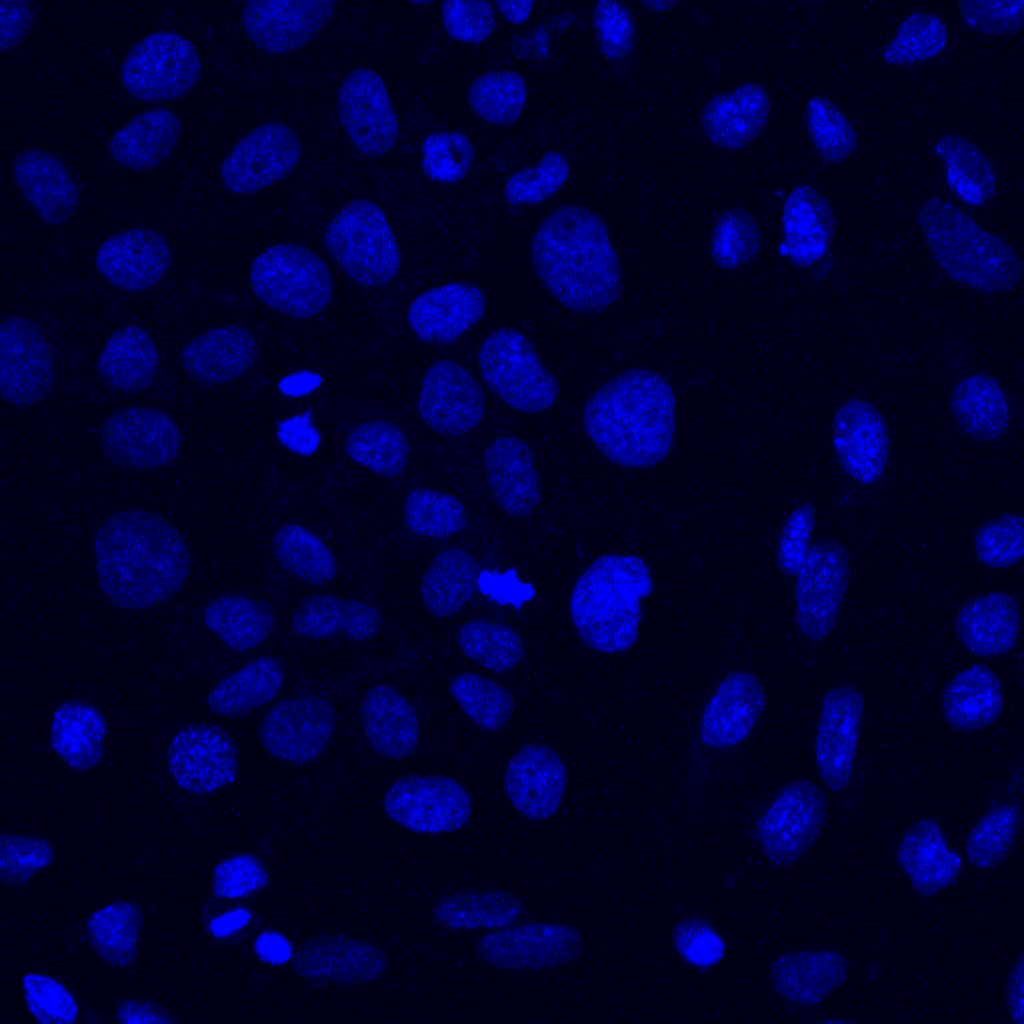

Supplement: Supplementary file 12 — Figure EV1 Source Data [file 44319_2026_751_MOESM12_ESM.zip › Raw_data_Figure EV1/Figure EV1H/C1-MAX_HN13 + fibros WT SMA Factin Ecad.tif]

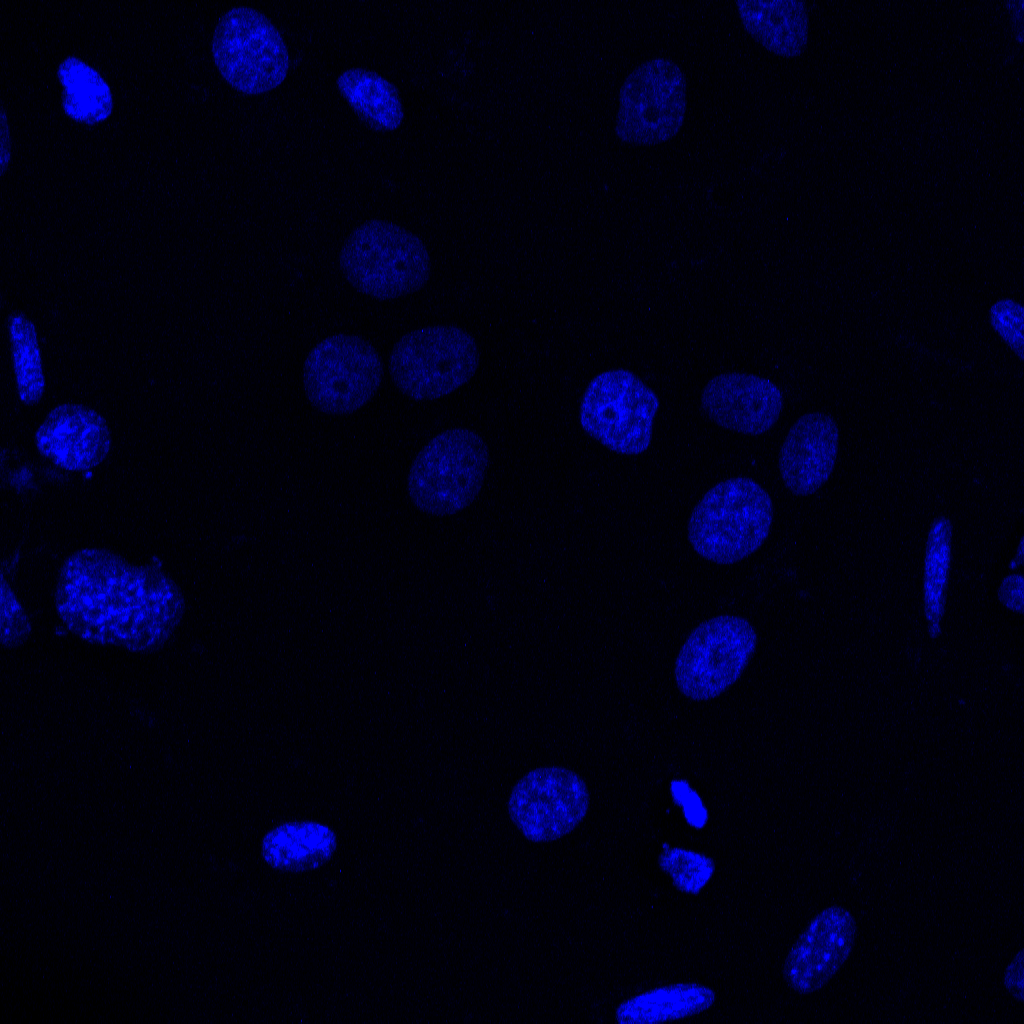

Supplement: Supplementary file 12 — Figure EV1 Source Data [file 44319_2026_751_MOESM12_ESM.zip › Raw_data_Figure EV1/Figure EV1H/C1-MAX_UMSCC KO SMA Factin Ecad.tif]

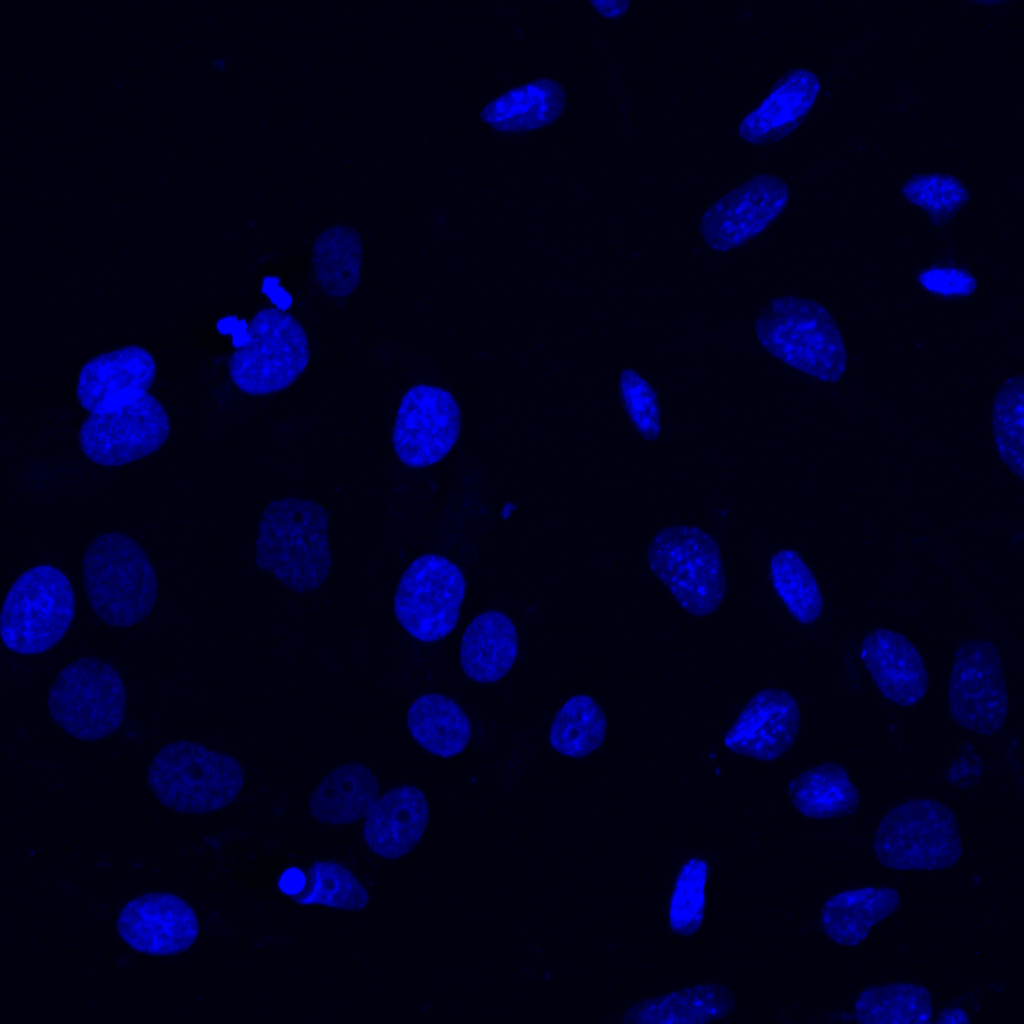

Supplement: Supplementary file 12 — Figure EV1 Source Data [file 44319_2026_751_MOESM12_ESM.zip › Raw_data_Figure EV1/Figure EV1H/C1-MAX_UMSCC WTSMA Factin Ecad.tif]

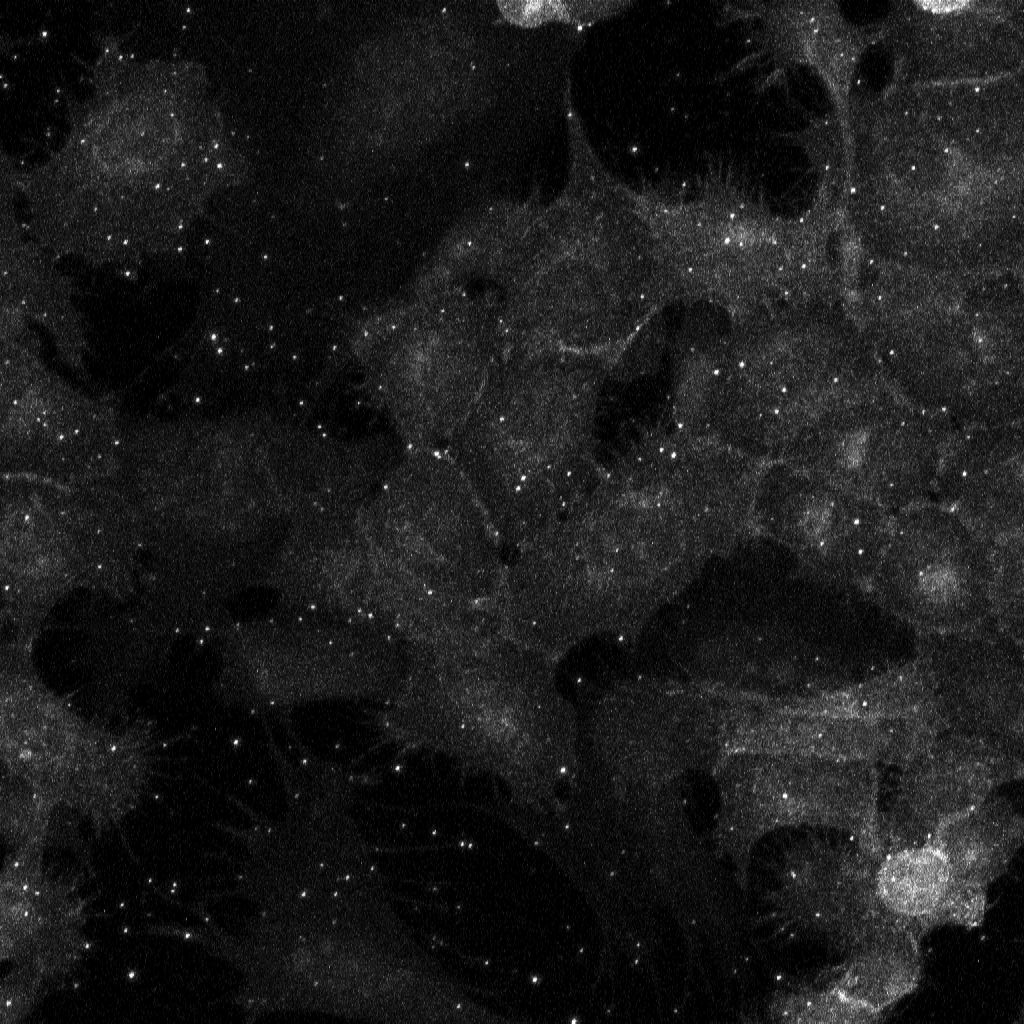

Supplement: Supplementary file 12 — Figure EV1 Source Data [file 44319_2026_751_MOESM12_ESM.zip › Raw_data_Figure EV1/Figure EV1H/C4-MAX_HN13 + fibros KO SMA Factin Ecad.tif]

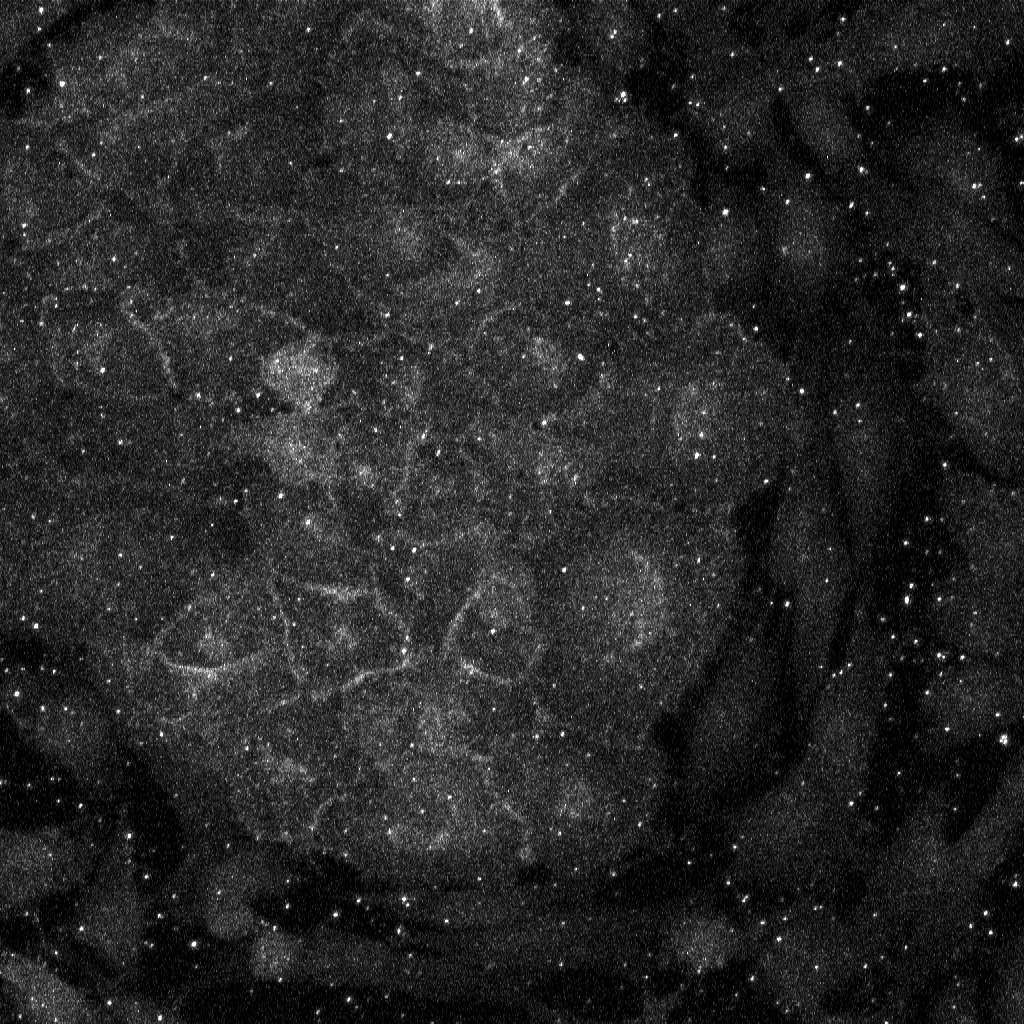

Supplement: Supplementary file 12 — Figure EV1 Source Data [file 44319_2026_751_MOESM12_ESM.zip › Raw_data_Figure EV1/Figure EV1H/C4-MAX_HN13 + fibros WT SMA Factin Ecad.tif]

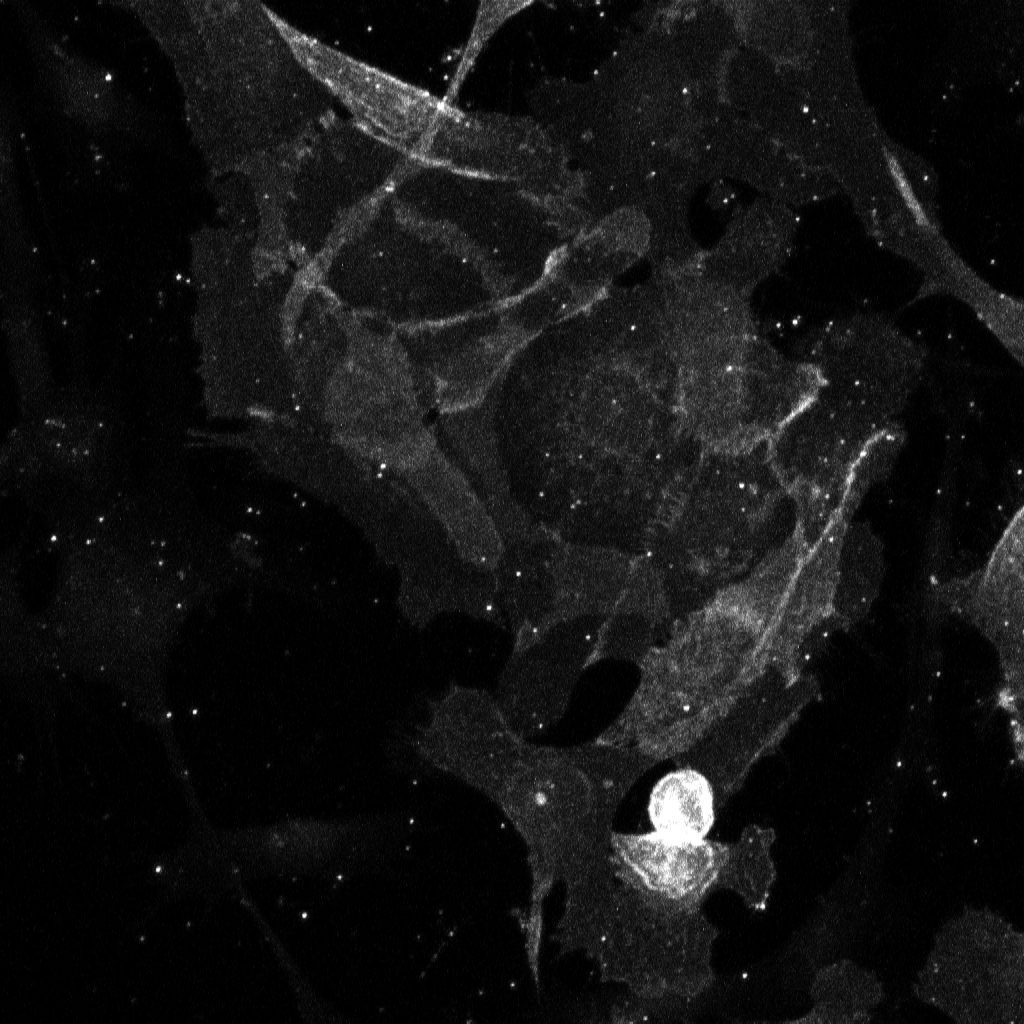

Supplement: Supplementary file 12 — Figure EV1 Source Data [file 44319_2026_751_MOESM12_ESM.zip › Raw_data_Figure EV1/Figure EV1H/C4-MAX_UMSCC KO SMA Factin Ecad.tif]

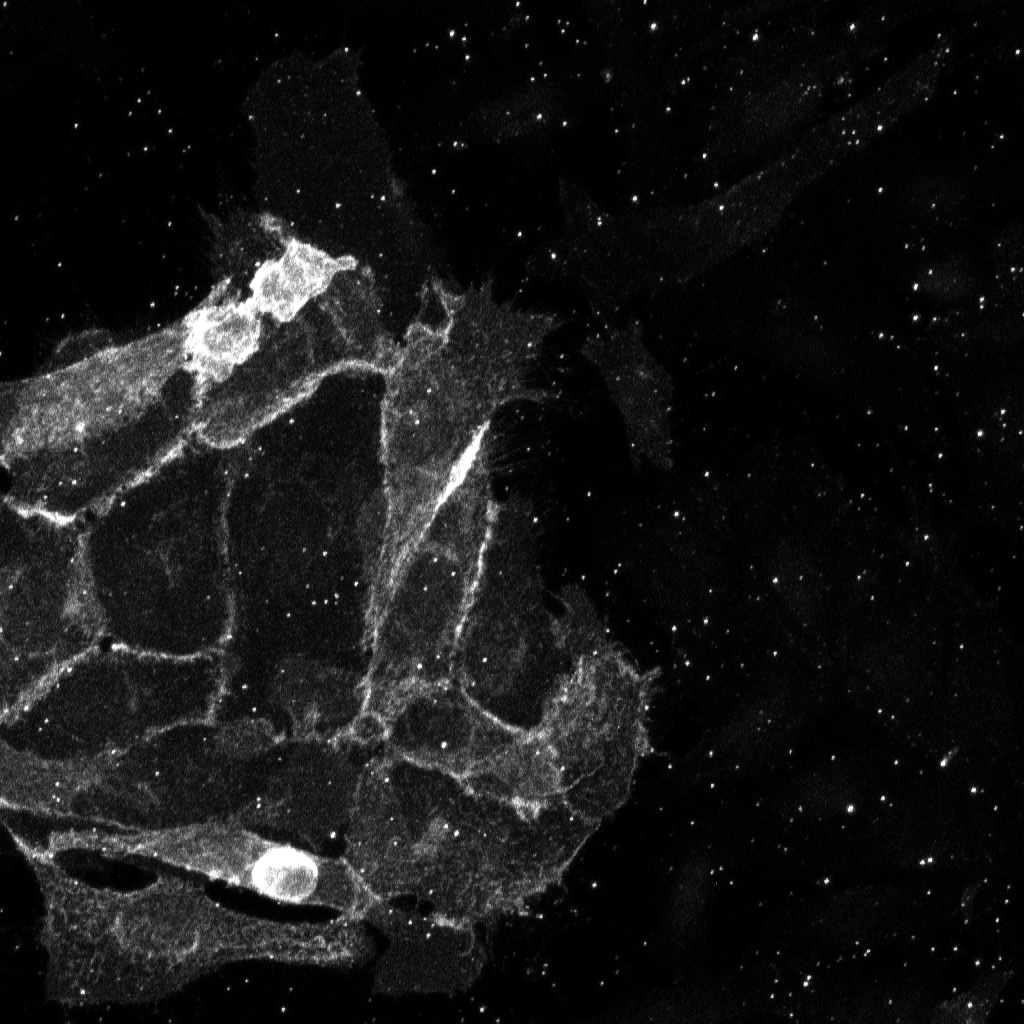

Supplement: Supplementary file 12 — Figure EV1 Source Data [file 44319_2026_751_MOESM12_ESM.zip › Raw_data_Figure EV1/Figure EV1H/C4-MAX_UMSCC WTSMA Factin Ecad.tif]

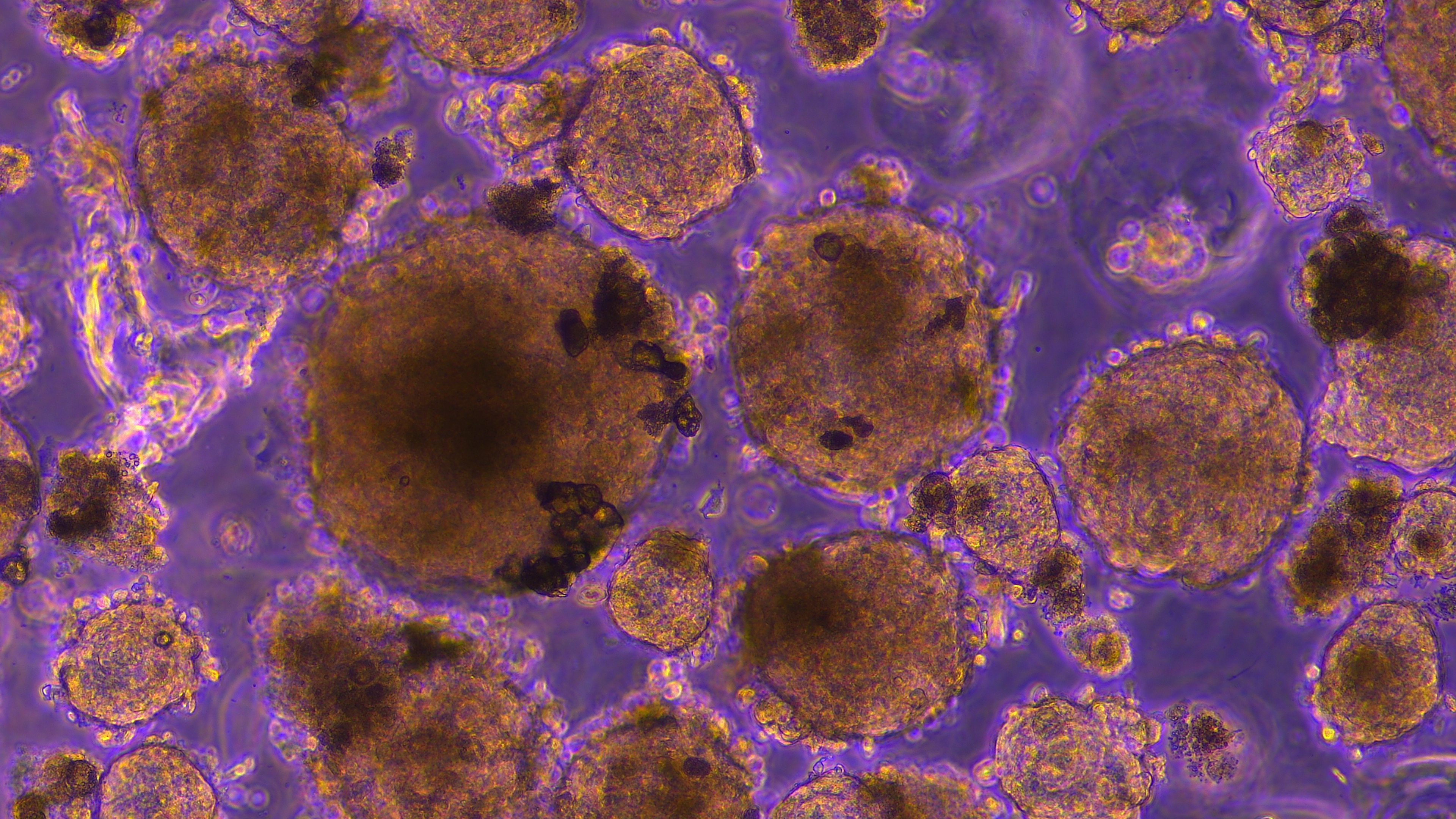

Supplement: Supplementary file 12 — Figure EV1 Source Data [file 44319_2026_751_MOESM12_ESM.zip › Raw_data_Figure EV1/Figure EV1I/gqko221115140955o.jpg]

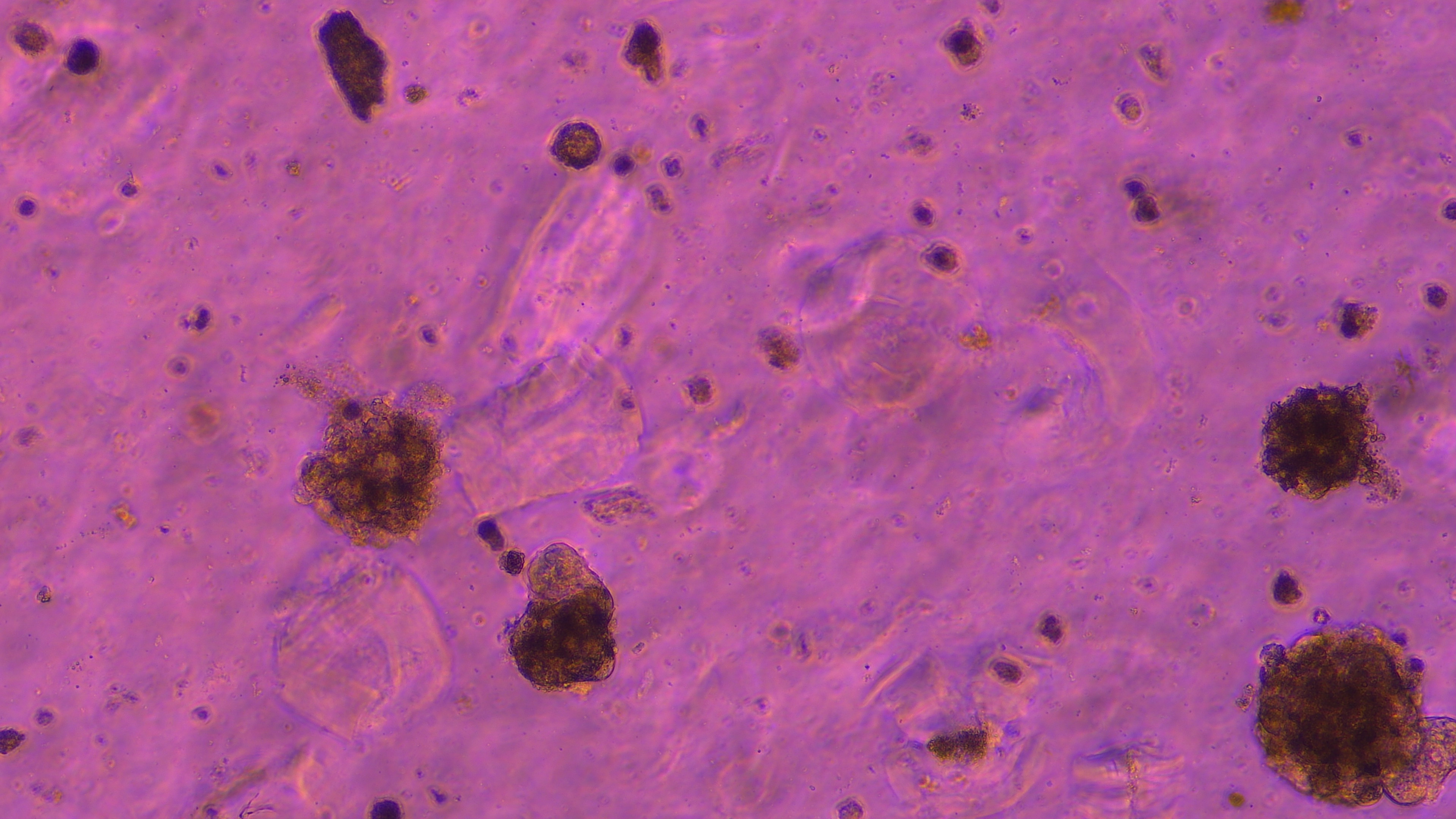

Supplement: Supplementary file 12 — Figure EV1 Source Data [file 44319_2026_751_MOESM12_ESM.zip › Raw_data_Figure EV1/Figure EV1I/wt221115140835o.jpg]

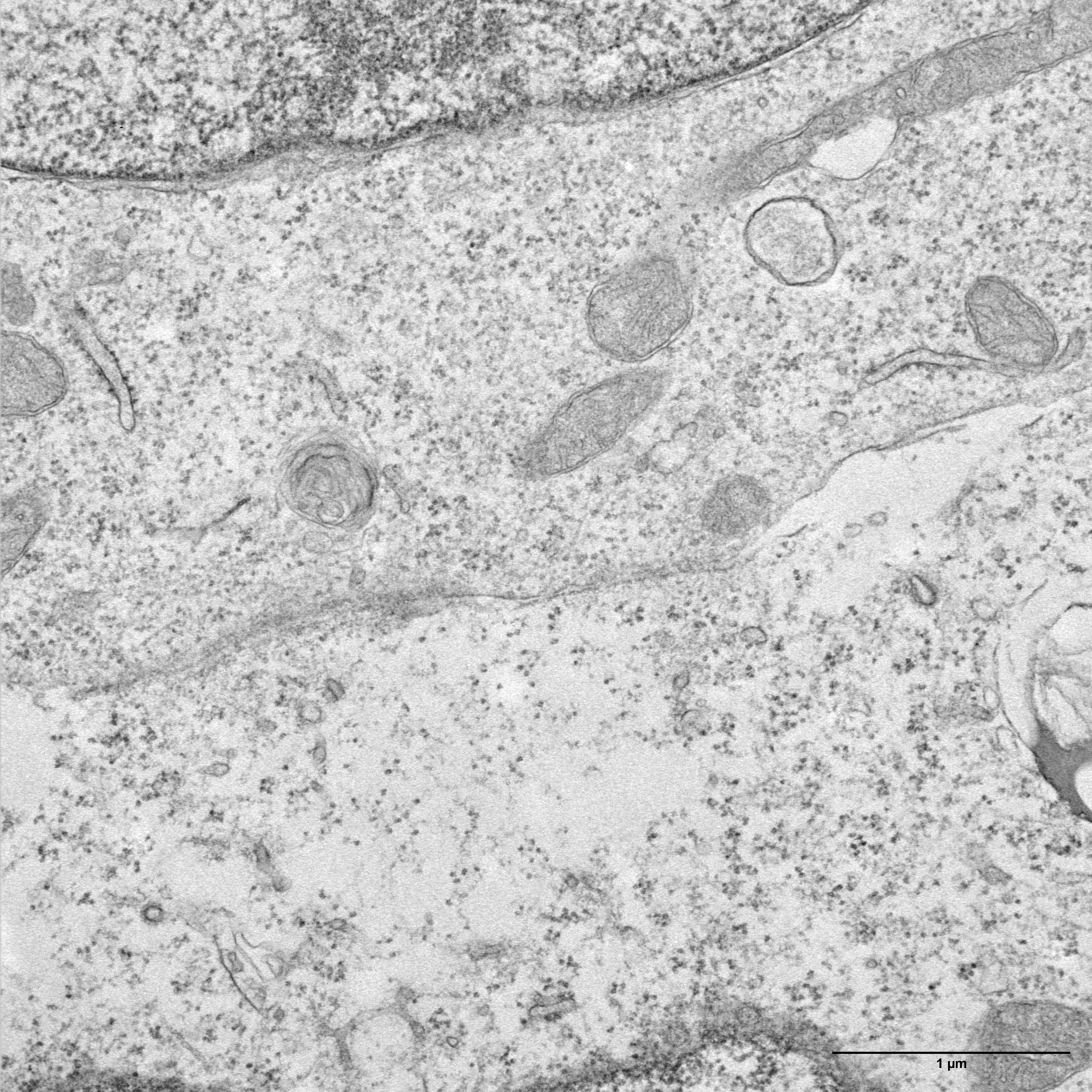

Supplement: Supplementary file 13 — Figure EV2 Source Data [file 44319_2026_751_MOESM13_ESM.zip › Raw_data_Figure EV2/Figure EV2A/10kX_0019 WT.tif]

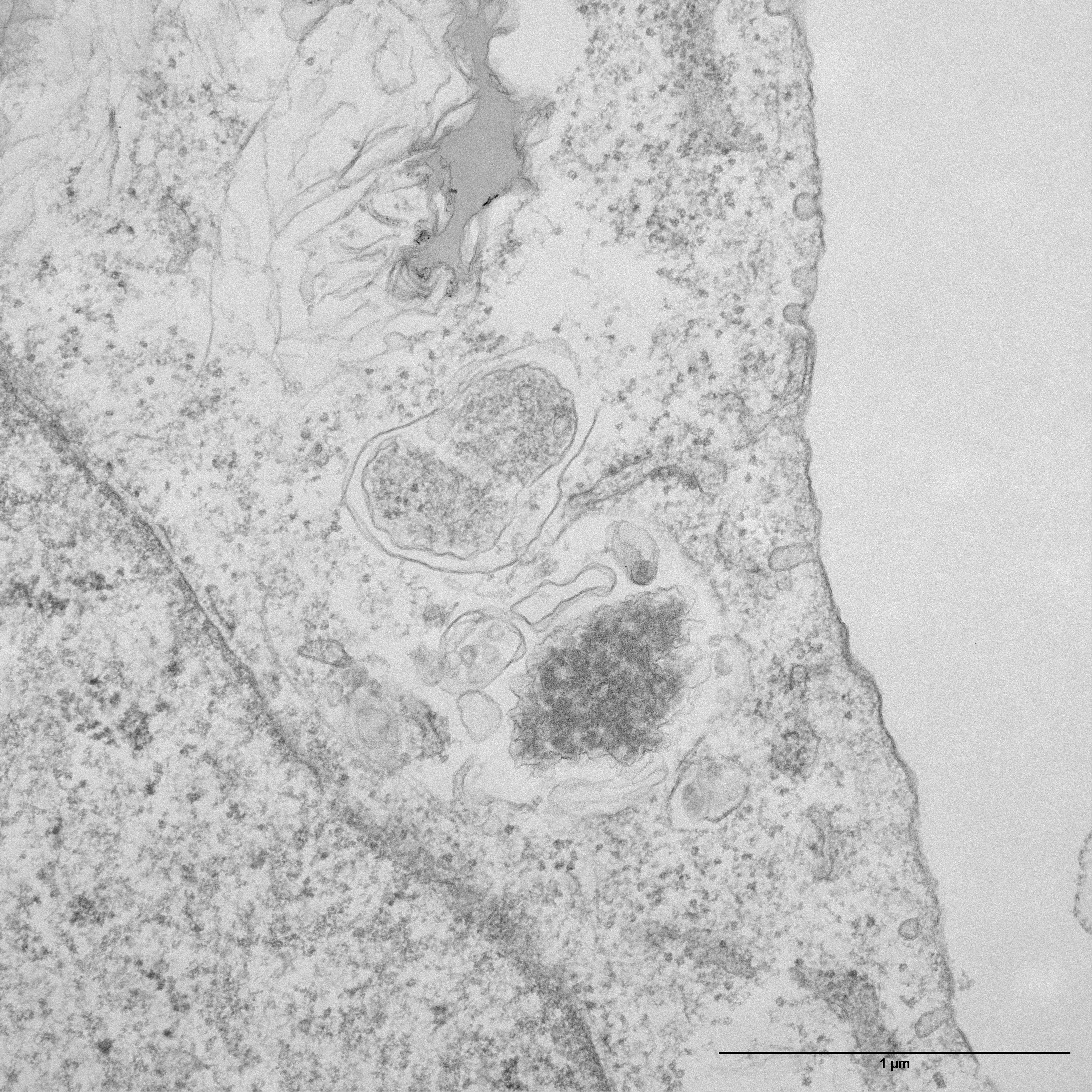

Supplement: Supplementary file 13 — Figure EV2 Source Data [file 44319_2026_751_MOESM13_ESM.zip › Raw_data_Figure EV2/Figure EV2A/15kX_0016 GqKO.tif]

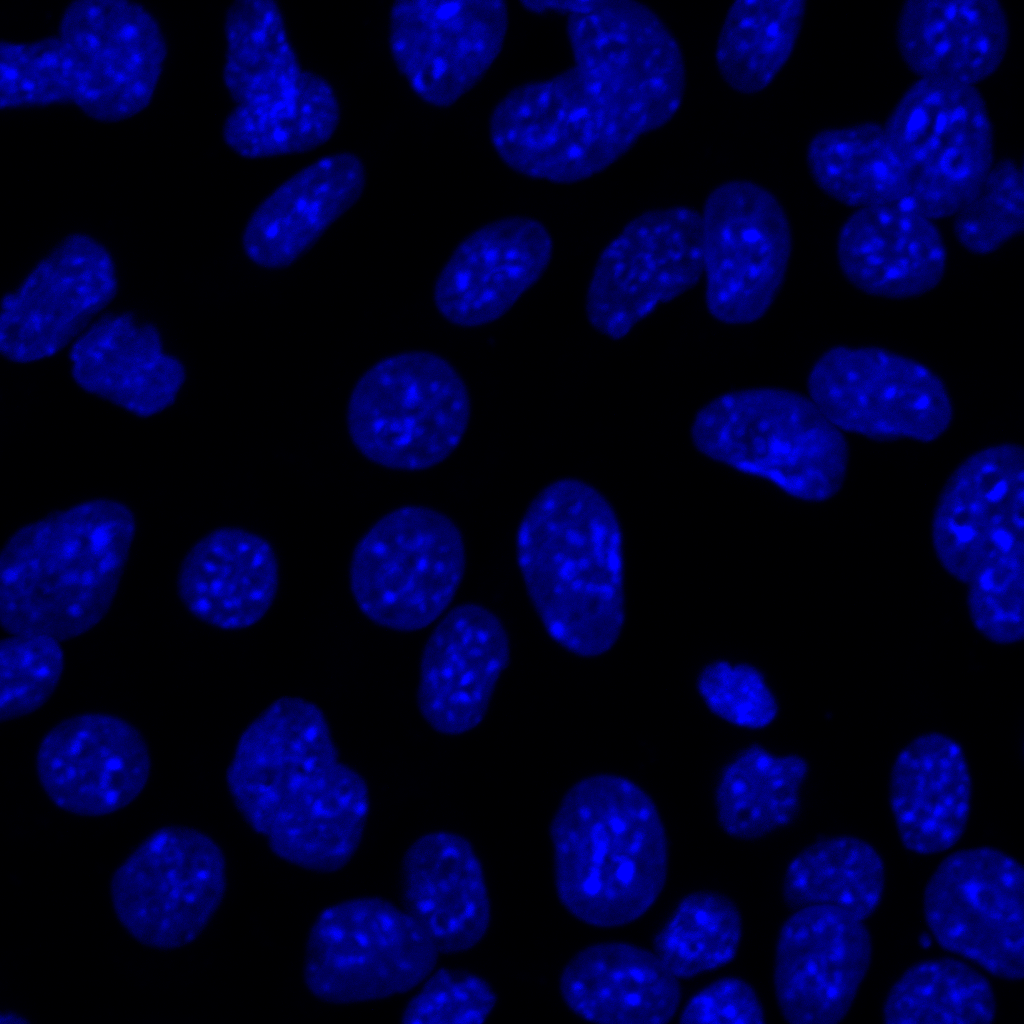

Supplement: Supplementary file 13 — Figure EV2 Source Data [file 44319_2026_751_MOESM13_ESM.zip › Raw_data_Figure EV2/Figure EV2B/WT shctrl nuclei.tif]

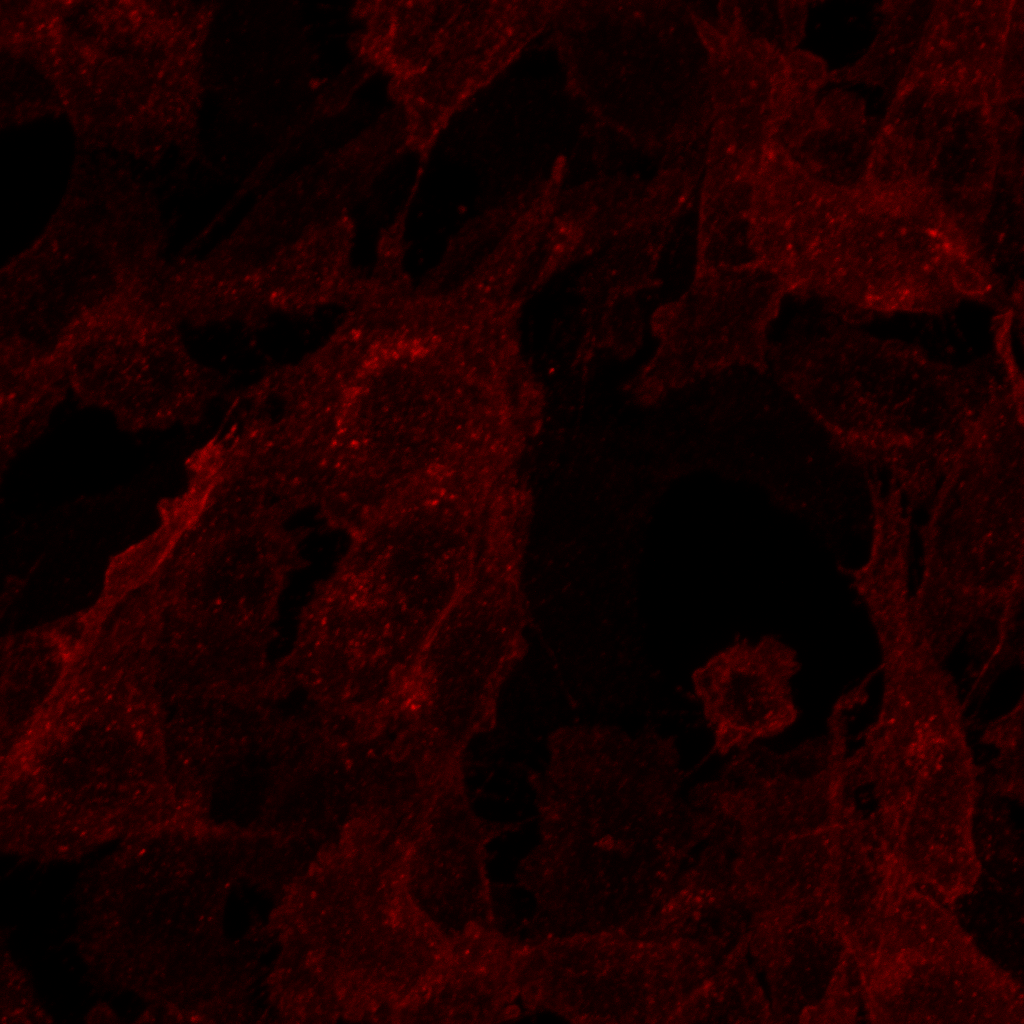

Supplement: Supplementary file 13 — Figure EV2 Source Data [file 44319_2026_751_MOESM13_ESM.zip › Raw_data_Figure EV2/Figure EV2B/WT shctrl PDGFR.tif]

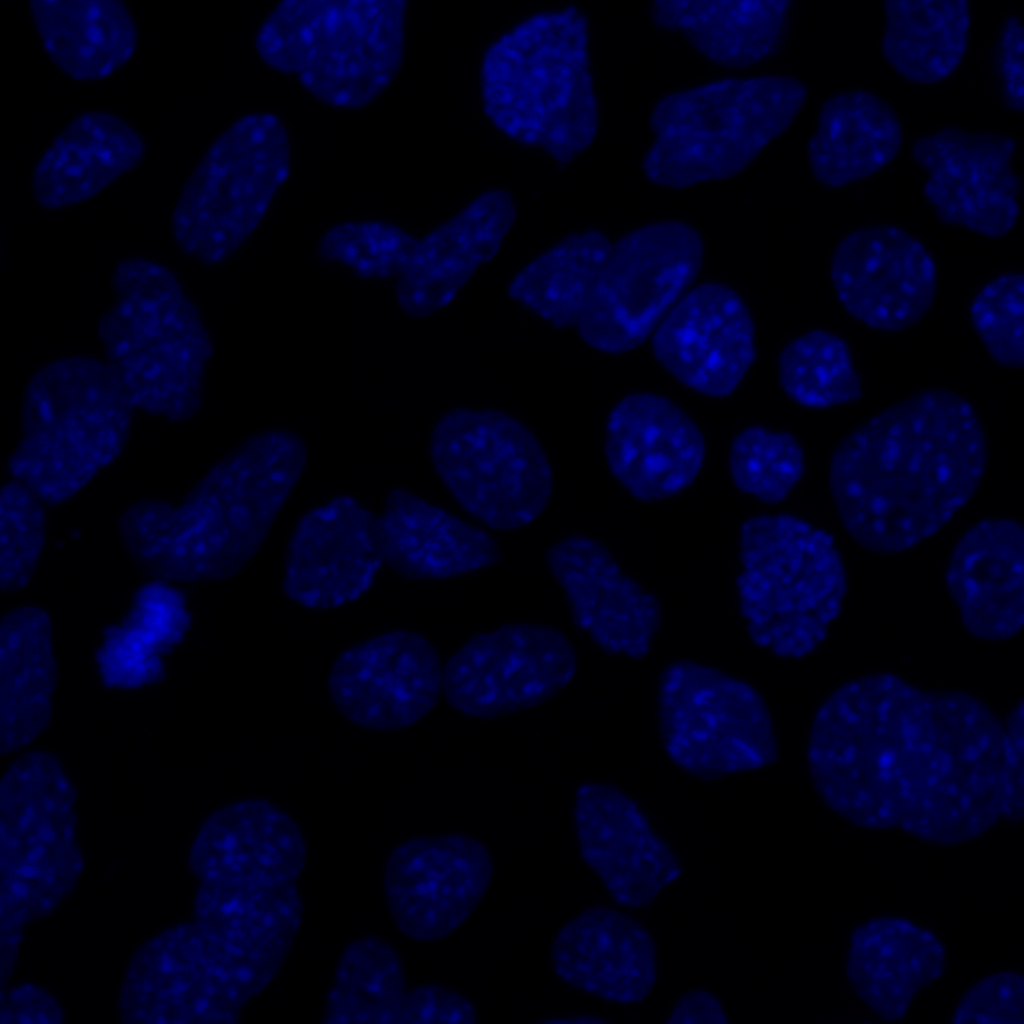

Supplement: Supplementary file 13 — Figure EV2 Source Data [file 44319_2026_751_MOESM13_ESM.zip › Raw_data_Figure EV2/Figure EV2B/WT shGq nuclei.tif]

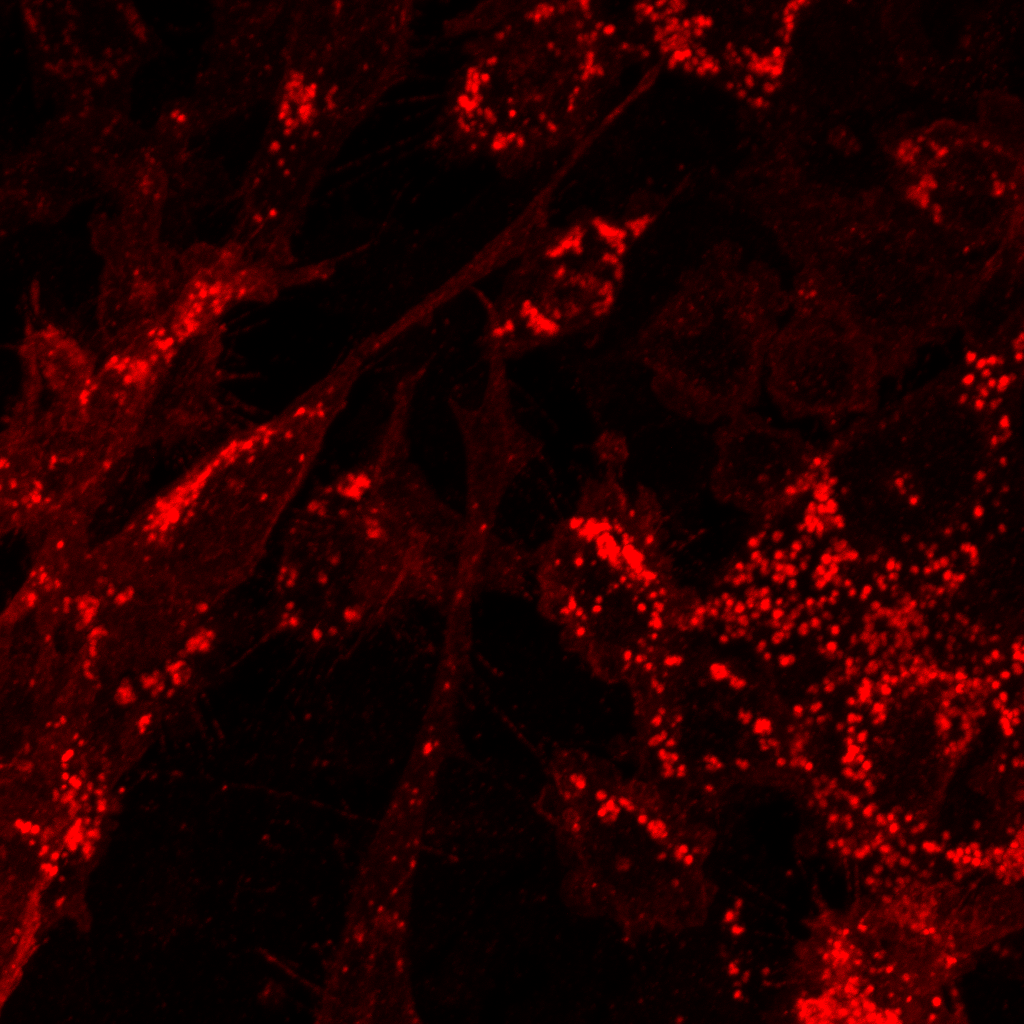

Supplement: Supplementary file 13 — Figure EV2 Source Data [file 44319_2026_751_MOESM13_ESM.zip › Raw_data_Figure EV2/Figure EV2B/WT shGq PDGFR.tif]

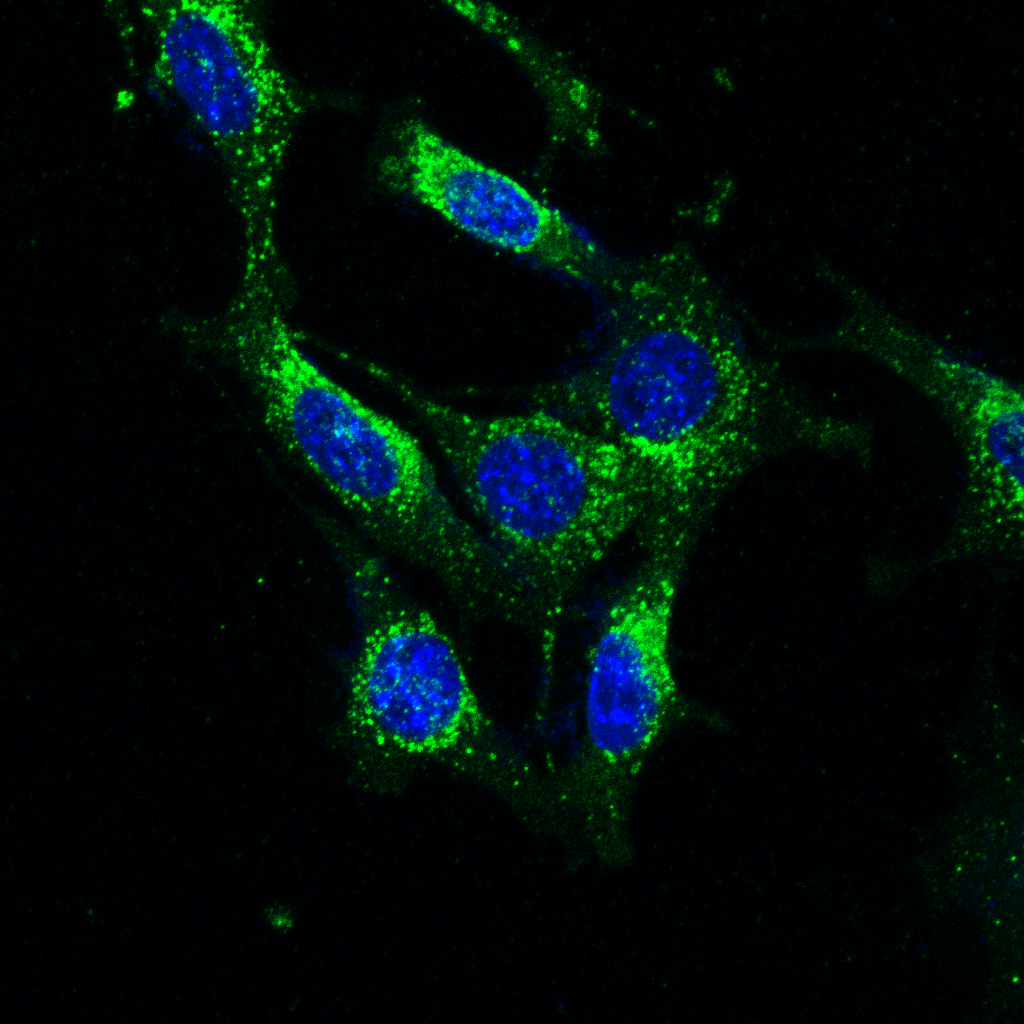

Supplement: Supplementary file 13 — Figure EV2 Source Data [file 44319_2026_751_MOESM13_ESM.zip › Raw_data_Figure EV2/Figure EV2C/KO MEFs ctrl LBPA 2.tif]

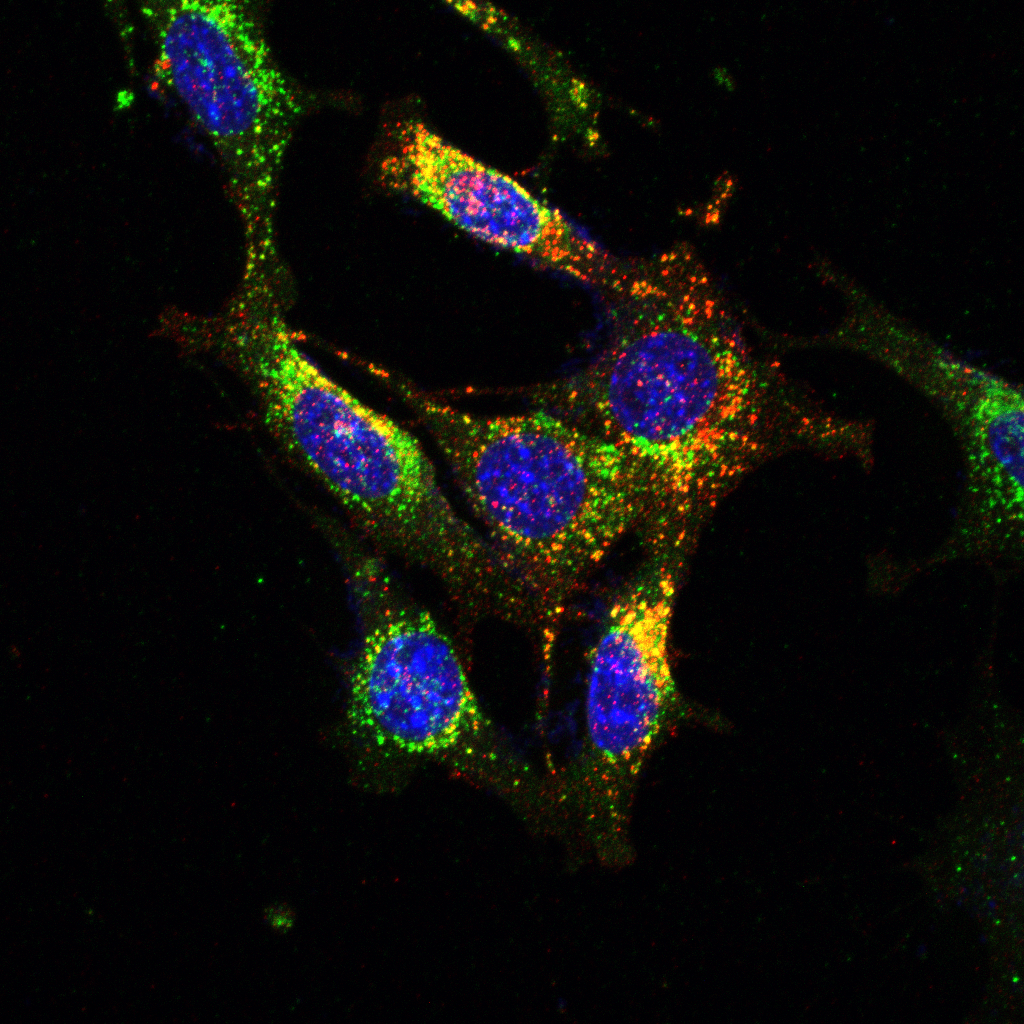

Supplement: Supplementary file 13 — Figure EV2 Source Data [file 44319_2026_751_MOESM13_ESM.zip › Raw_data_Figure EV2/Figure EV2C/KO MEFs ctrl merge 2.tif]

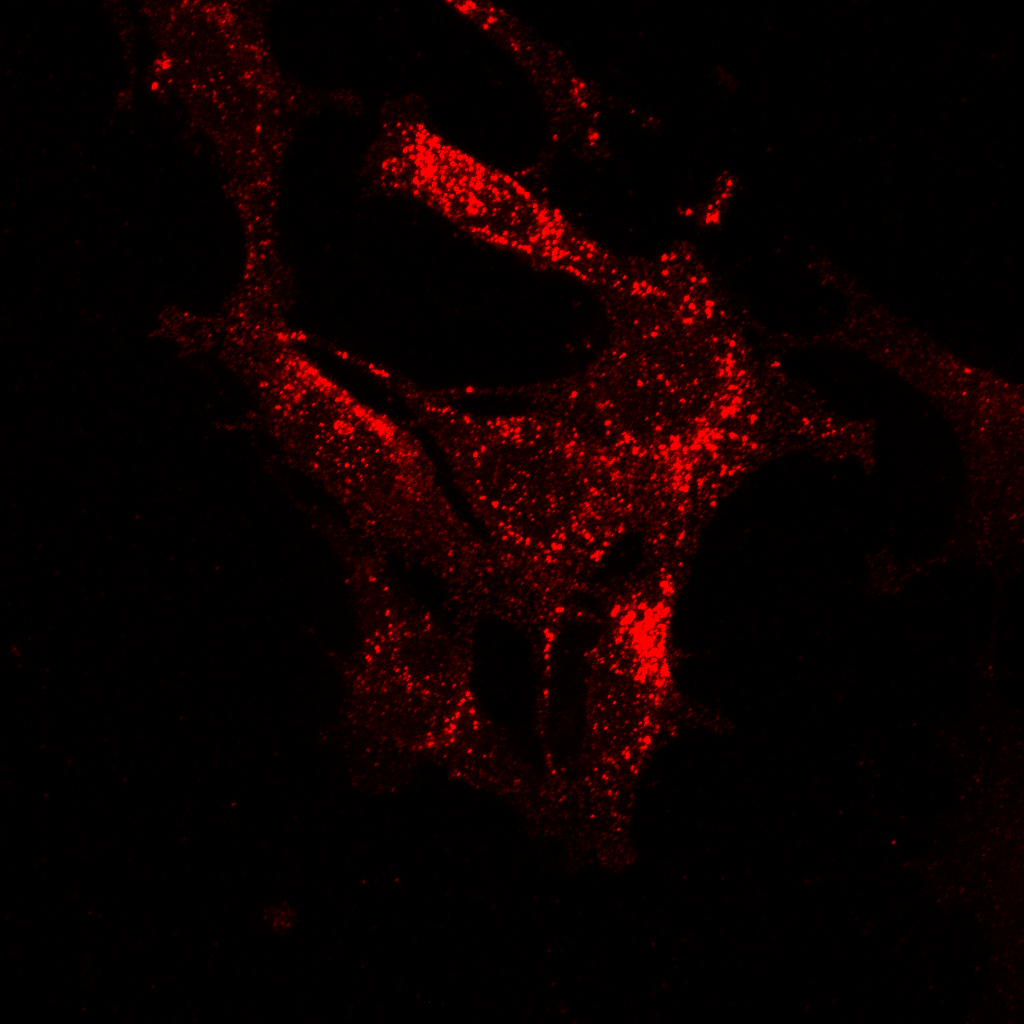

Supplement: Supplementary file 13 — Figure EV2 Source Data [file 44319_2026_751_MOESM13_ESM.zip › Raw_data_Figure EV2/Figure EV2C/KO MEFs ctrl PDGFR 2.tif]

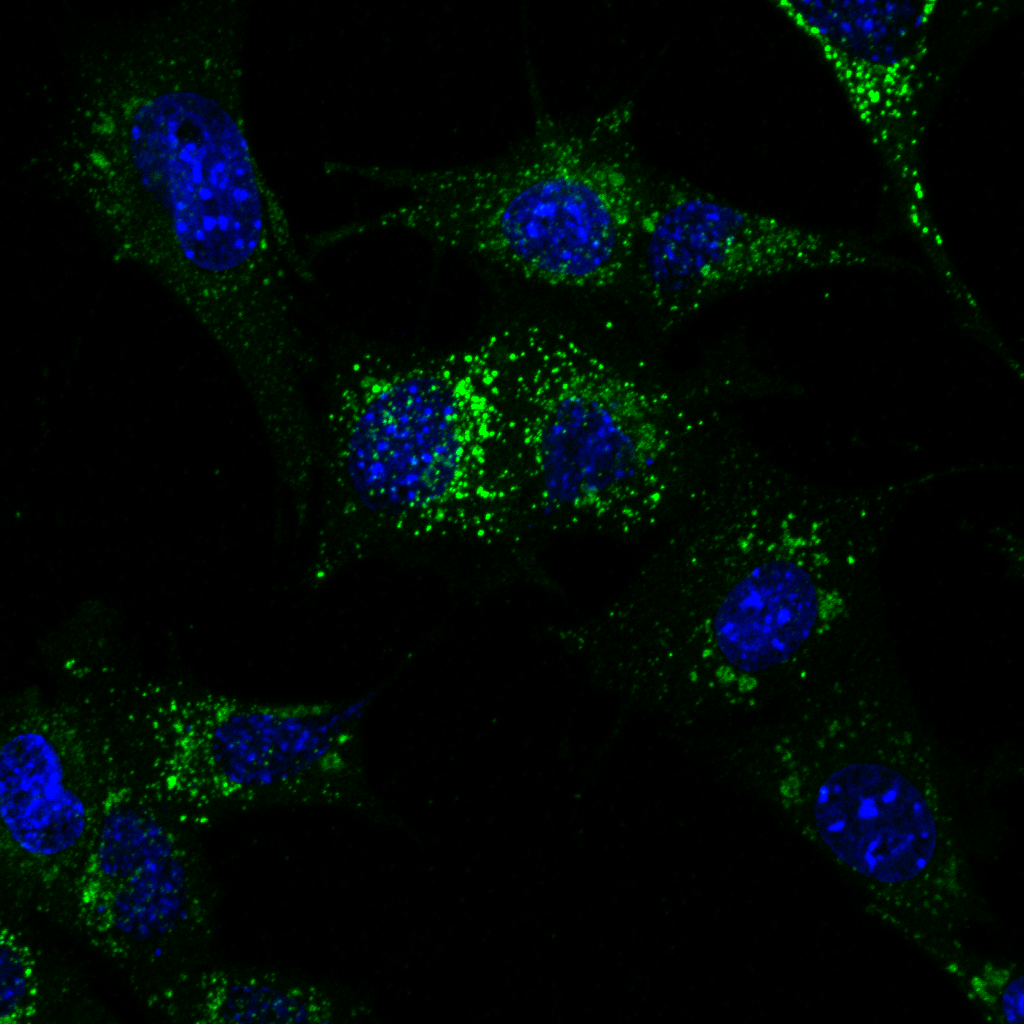

Supplement: Supplementary file 13 — Figure EV2 Source Data [file 44319_2026_751_MOESM13_ESM.zip › Raw_data_Figure EV2/Figure EV2C/WT MEFs ctrl blue and LPBA 2.tif]

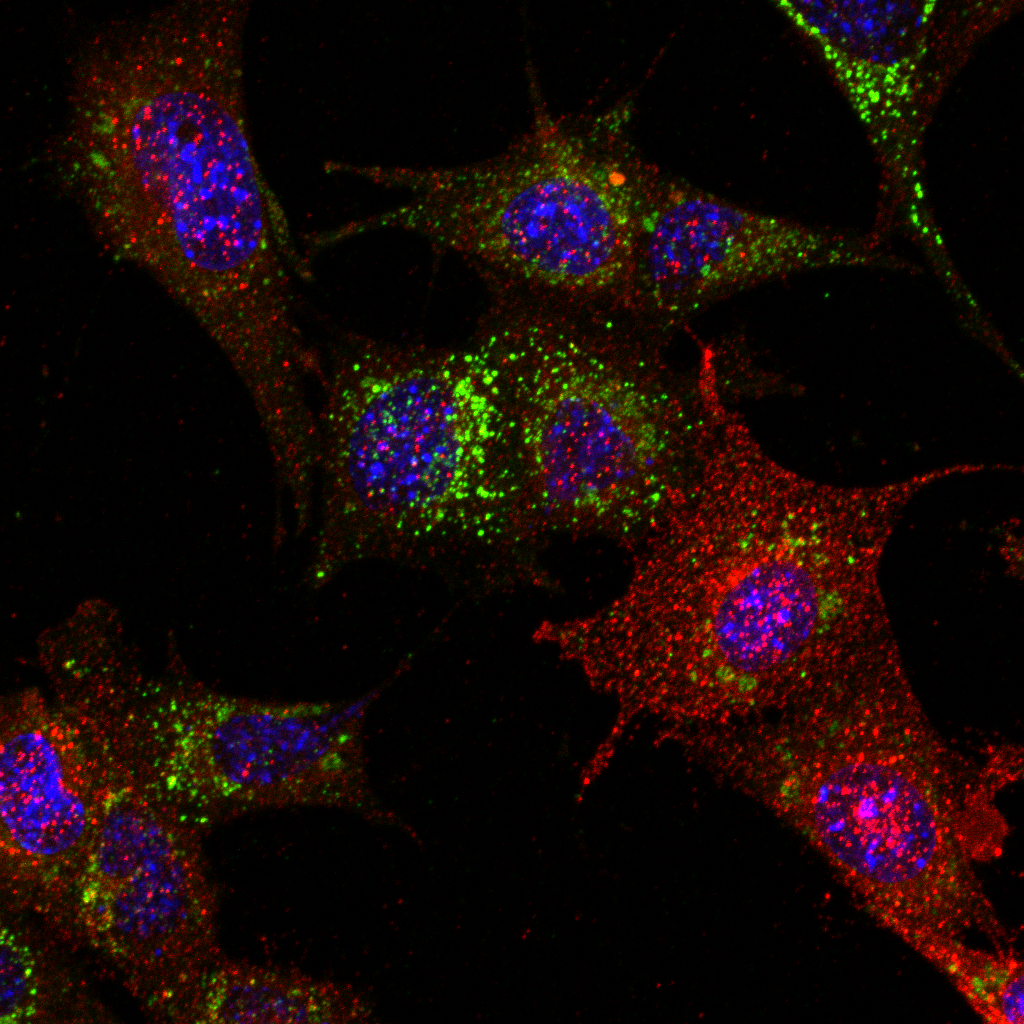

Supplement: Supplementary file 13 — Figure EV2 Source Data [file 44319_2026_751_MOESM13_ESM.zip › Raw_data_Figure EV2/Figure EV2C/WT MEFs ctrl merge 2.tif]

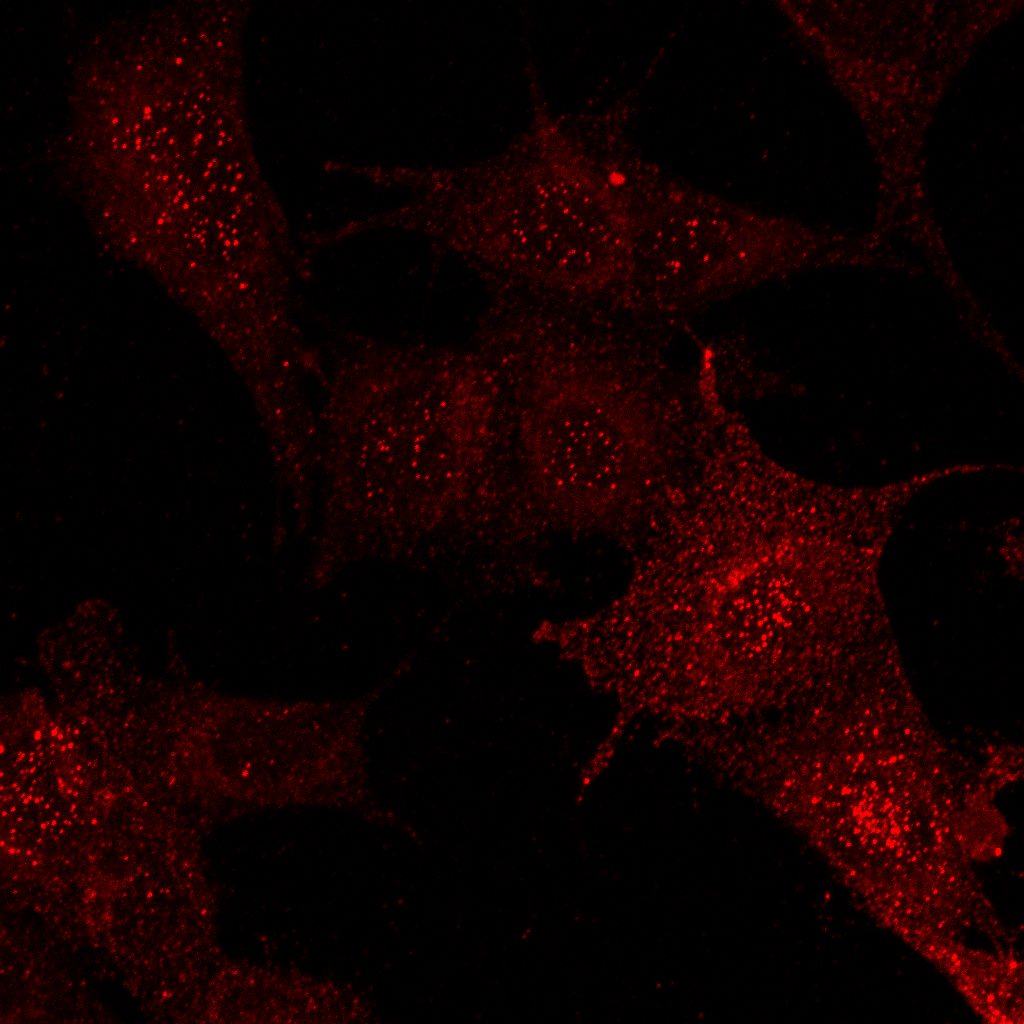

Supplement: Supplementary file 13 — Figure EV2 Source Data [file 44319_2026_751_MOESM13_ESM.zip › Raw_data_Figure EV2/Figure EV2C/WT MEFs ctrl PDGFR 2.tif]

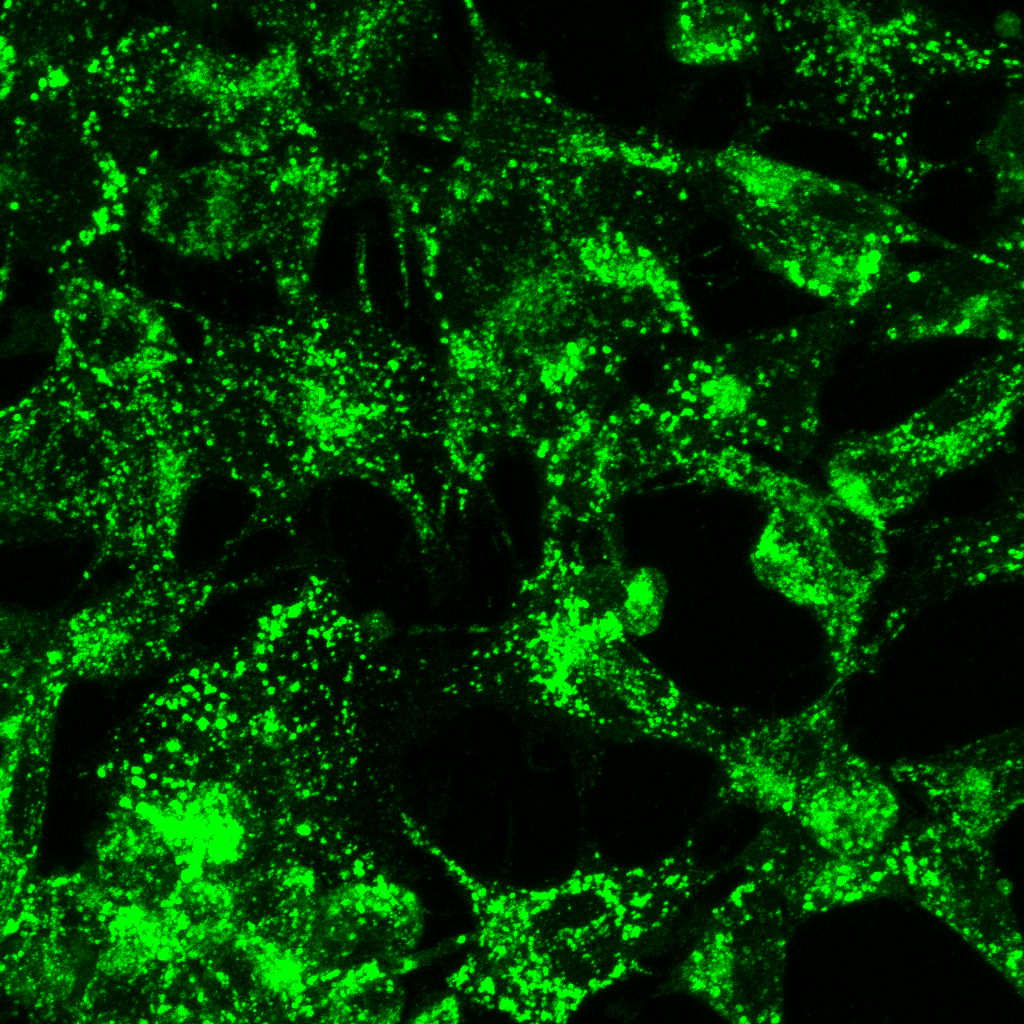

Supplement: Supplementary file 13 — Figure EV2 Source Data [file 44319_2026_751_MOESM13_ESM.zip › Raw_data_Figure EV2/Figure EV2D/GqKOLAMP1 72 h.tif]

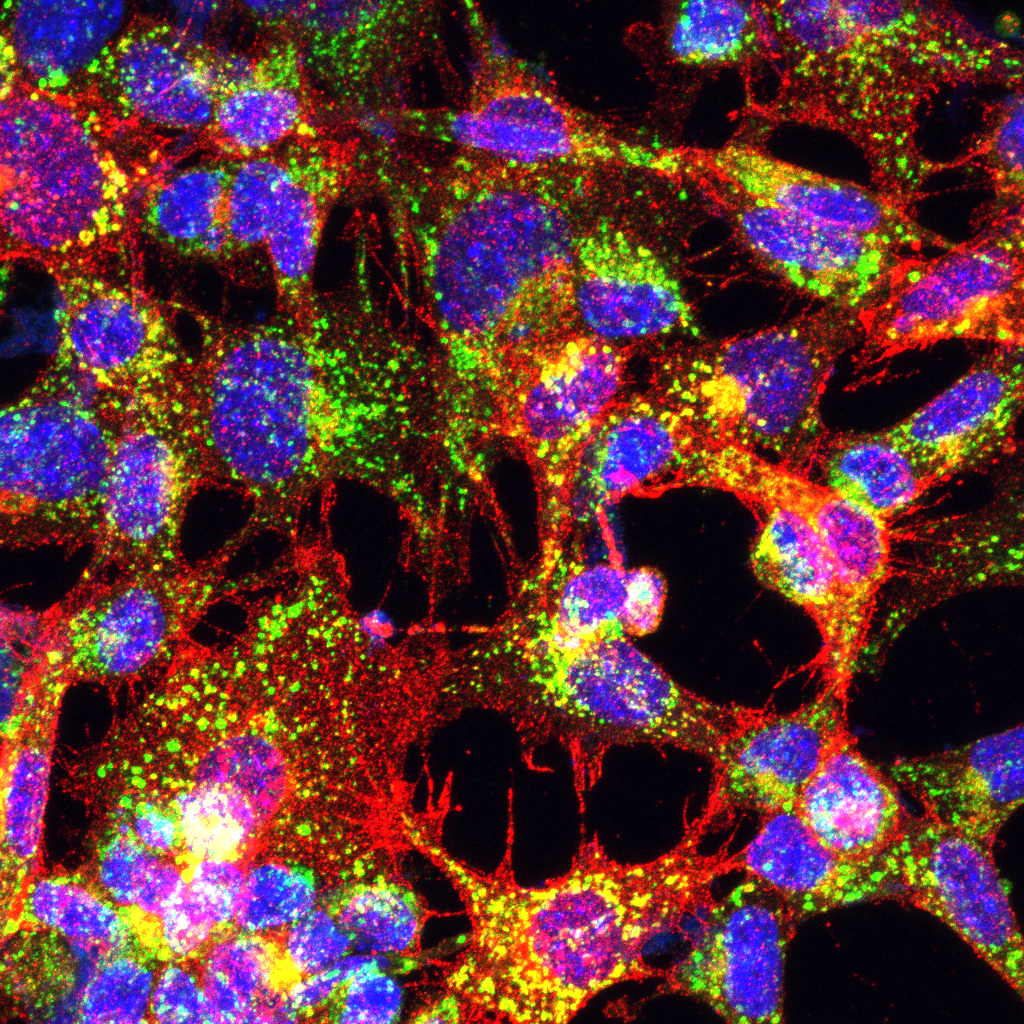

Supplement: Supplementary file 13 — Figure EV2 Source Data [file 44319_2026_751_MOESM13_ESM.zip › Raw_data_Figure EV2/Figure EV2D/GqKOLAMP1_PDGFR 72 h merge.tif]

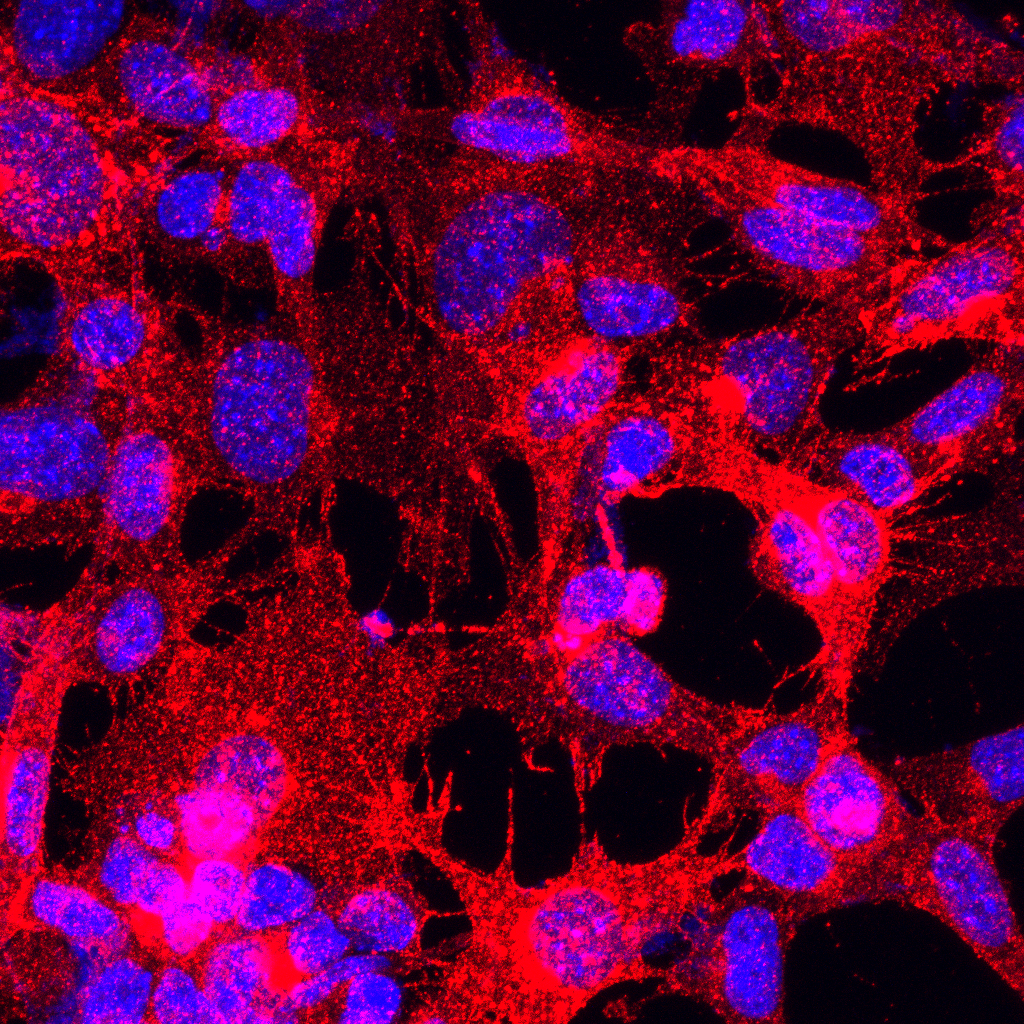

Supplement: Supplementary file 13 — Figure EV2 Source Data [file 44319_2026_751_MOESM13_ESM.zip › Raw_data_Figure EV2/Figure EV2D/GqKOPDGFR 72 h.tif]

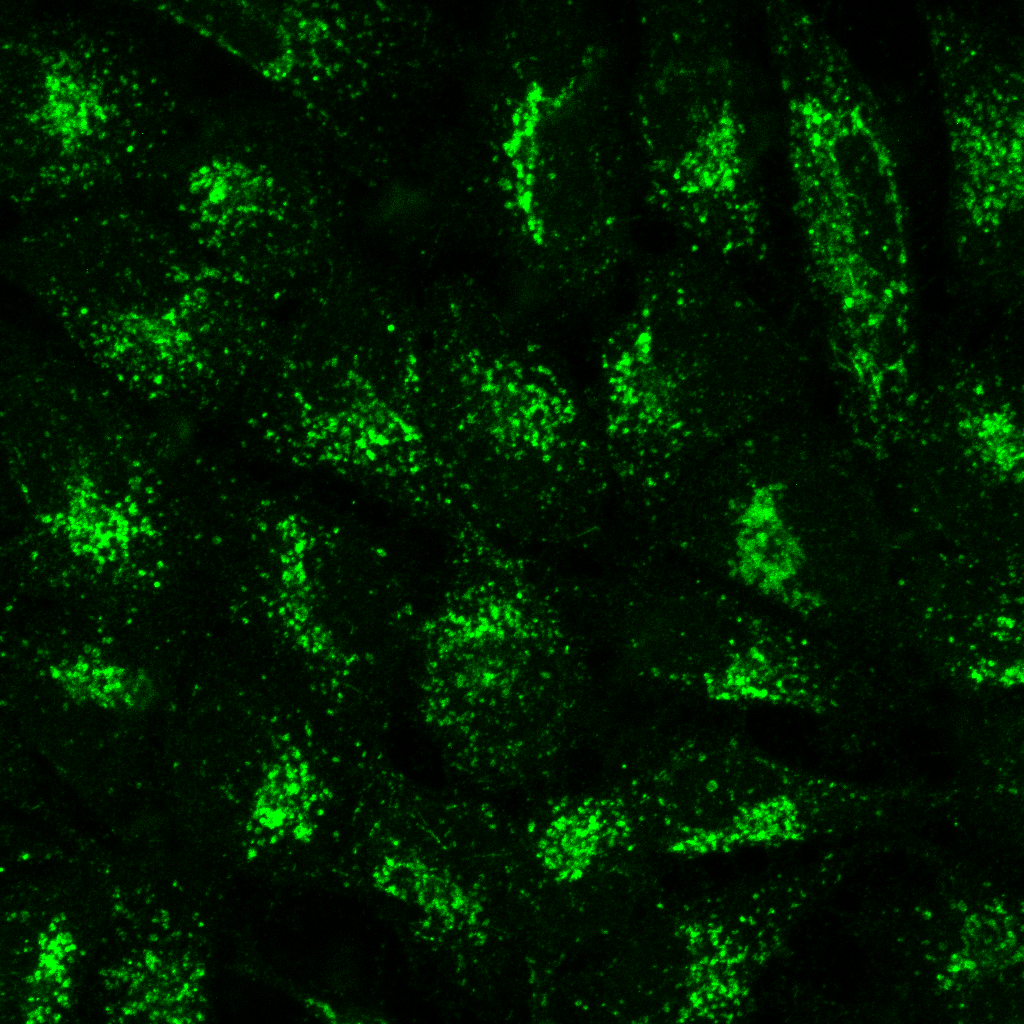

Supplement: Supplementary file 13 — Figure EV2 Source Data [file 44319_2026_751_MOESM13_ESM.zip › Raw_data_Figure EV2/Figure EV2D/WT LAMP2 72 h.tif]

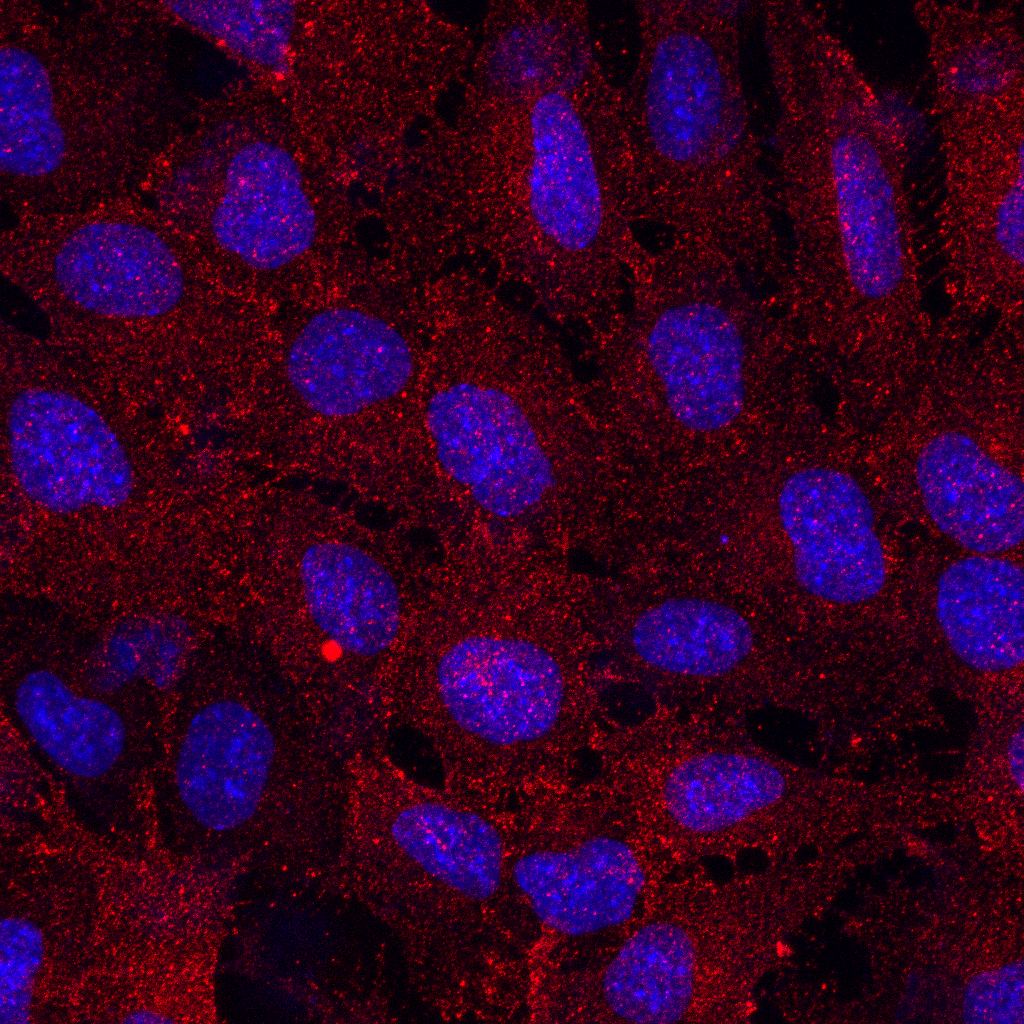

Supplement: Supplementary file 13 — Figure EV2 Source Data [file 44319_2026_751_MOESM13_ESM.zip › Raw_data_Figure EV2/Figure EV2D/WT PDGFR 72 h.tif]

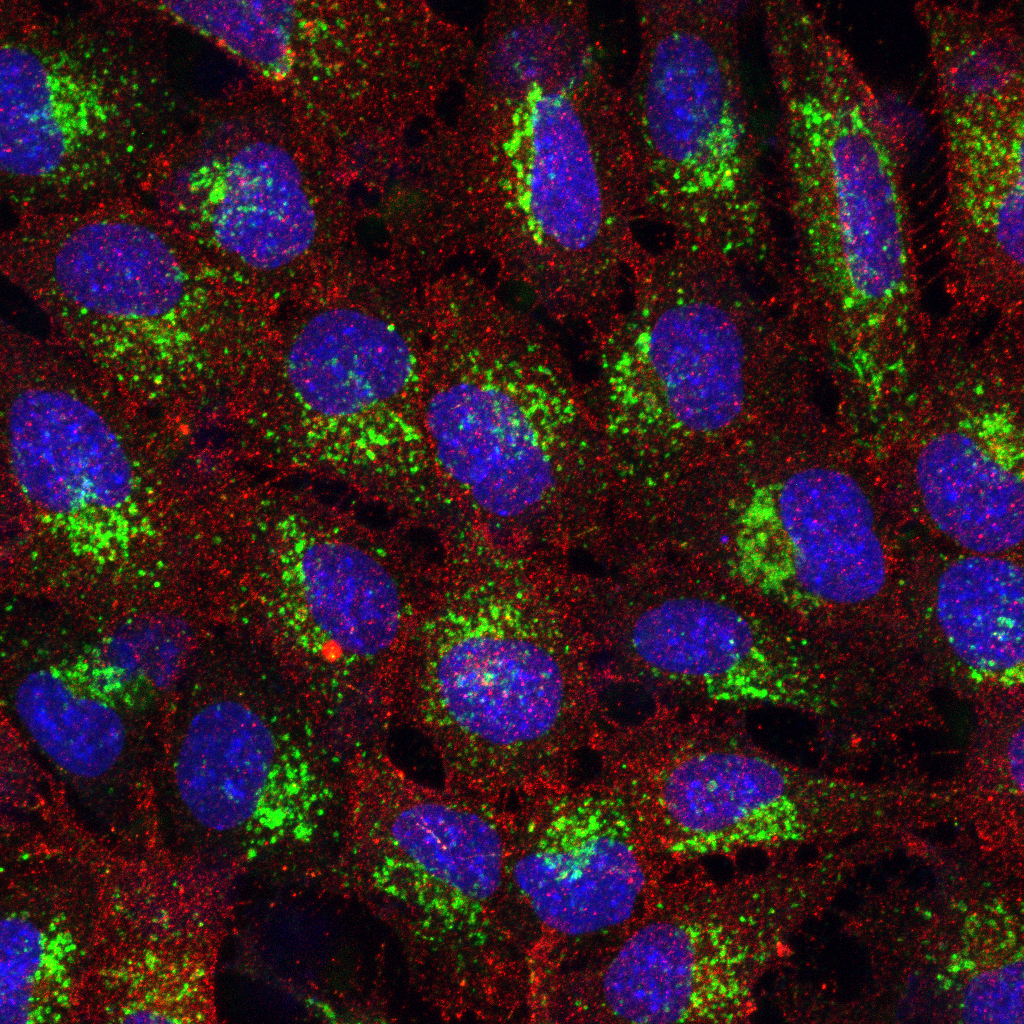

Supplement: Supplementary file 13 — Figure EV2 Source Data [file 44319_2026_751_MOESM13_ESM.zip › Raw_data_Figure EV2/Figure EV2D/WT PDGFR_LAMP 72 h merge.tif]

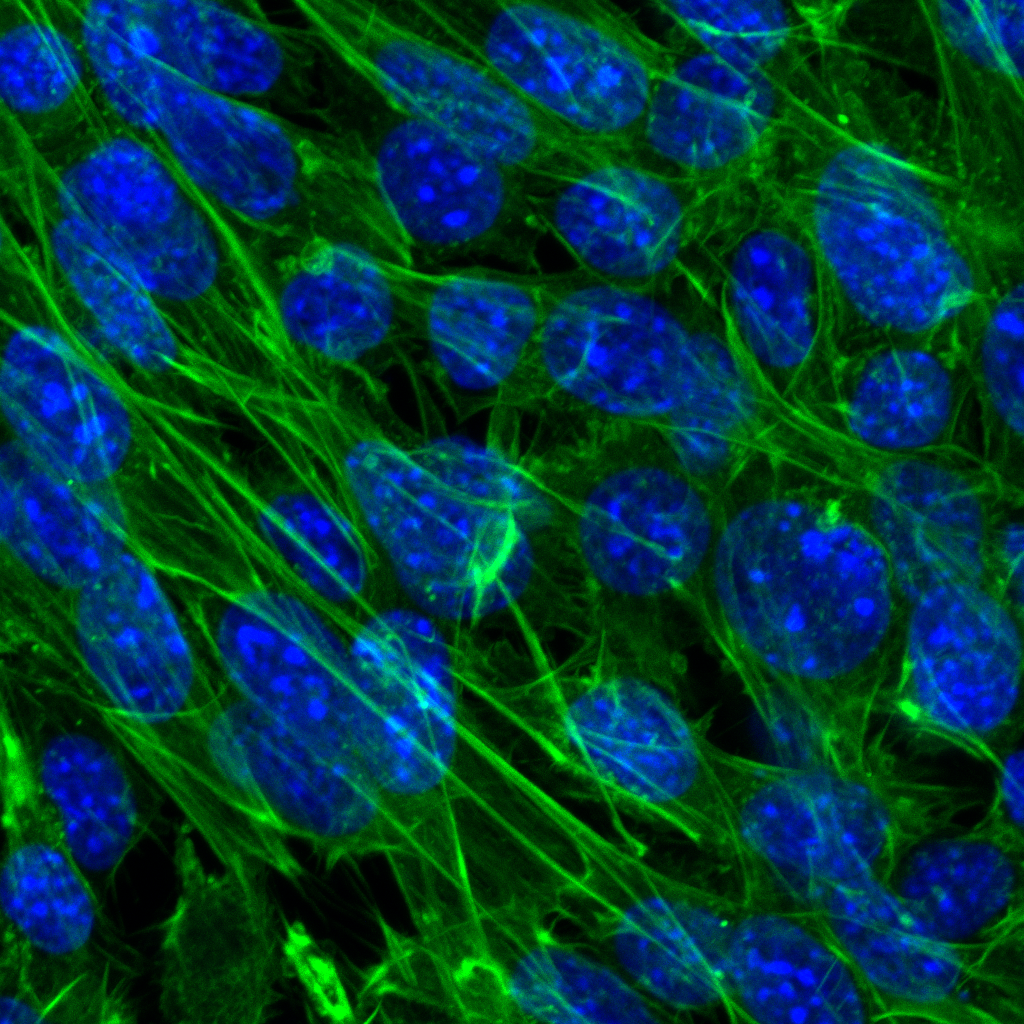

Supplement: Supplementary file 13 — Figure EV2 Source Data [file 44319_2026_751_MOESM13_ESM.zip › Raw_data_Figure EV2/Figure EV2E/WT shcontrol nuclei Factin.tif]

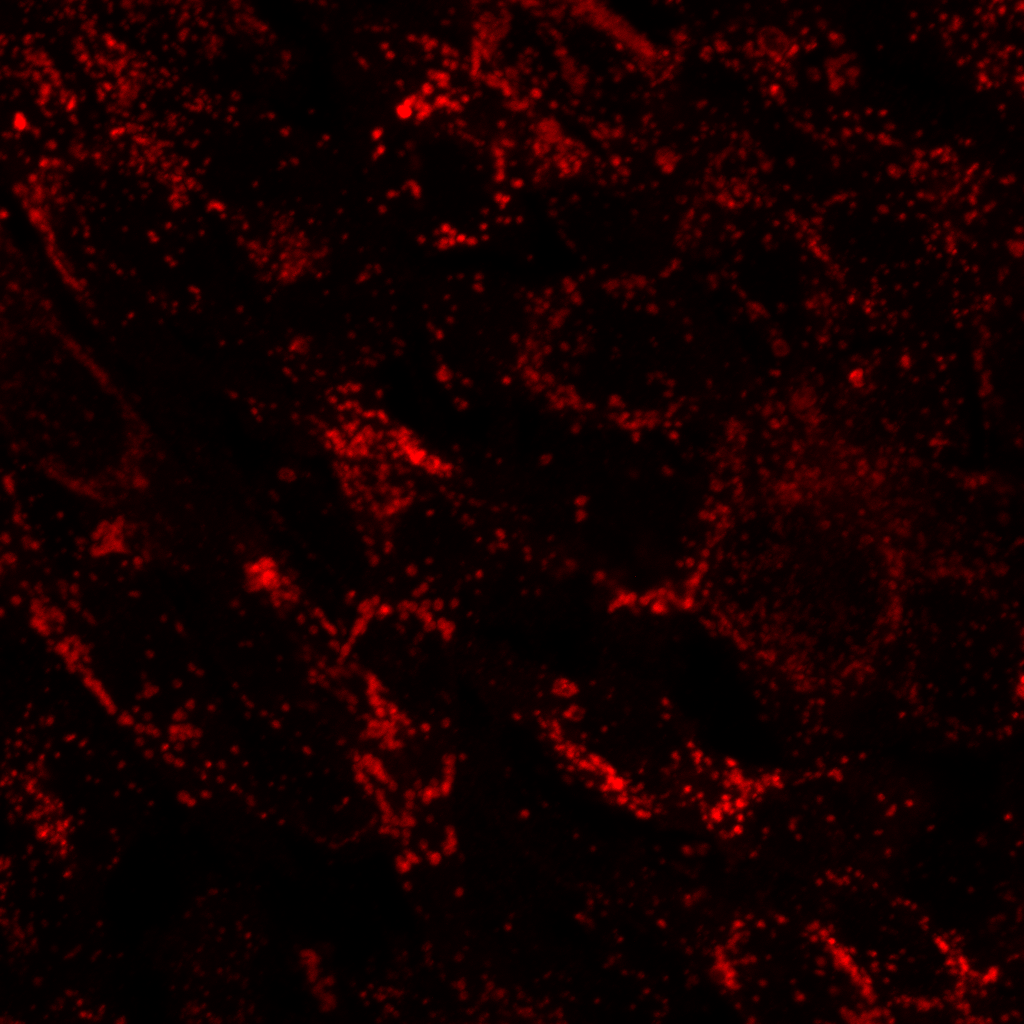

Supplement: Supplementary file 13 — Figure EV2 Source Data [file 44319_2026_751_MOESM13_ESM.zip › Raw_data_Figure EV2/Figure EV2E/WT shcontrolLAMP1.tif]

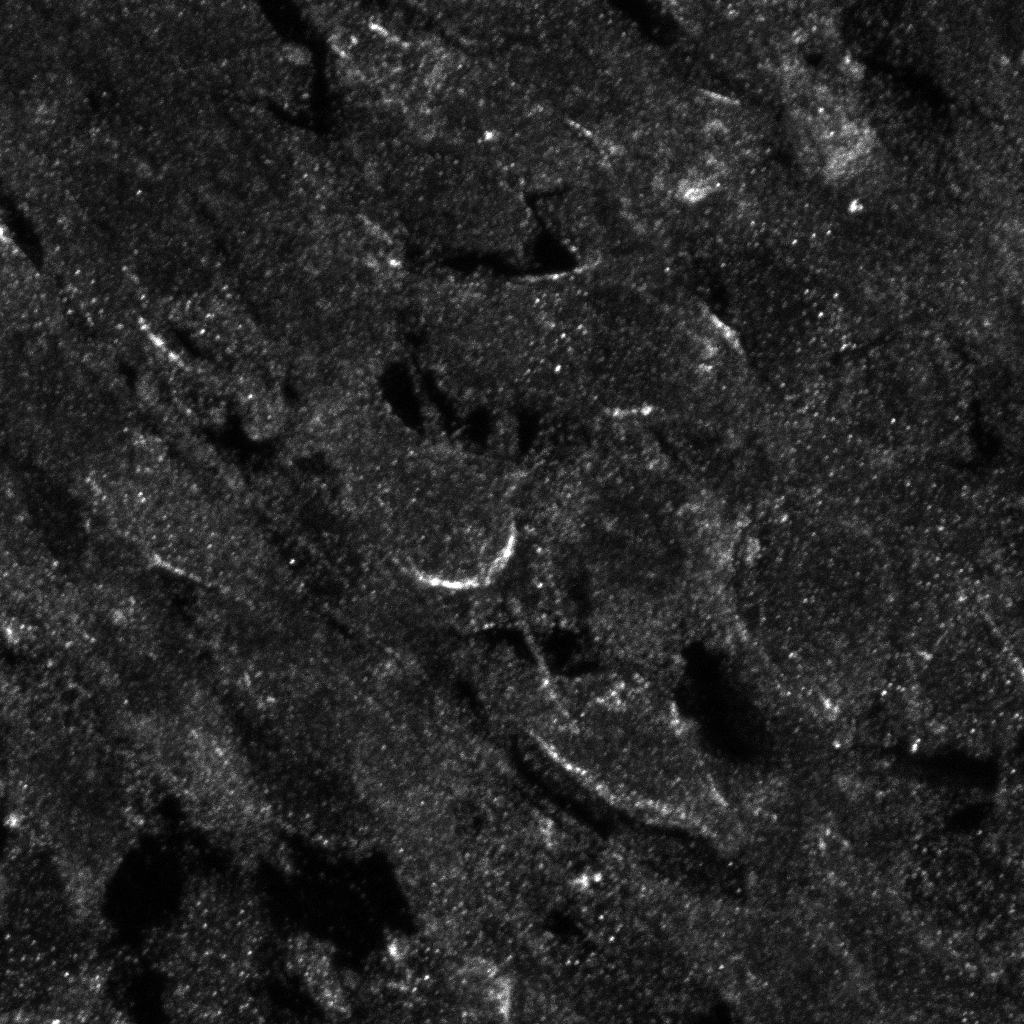

Supplement: Supplementary file 13 — Figure EV2 Source Data [file 44319_2026_751_MOESM13_ESM.zip › Raw_data_Figure EV2/Figure EV2E/WT shcontrolPTRF.tif]

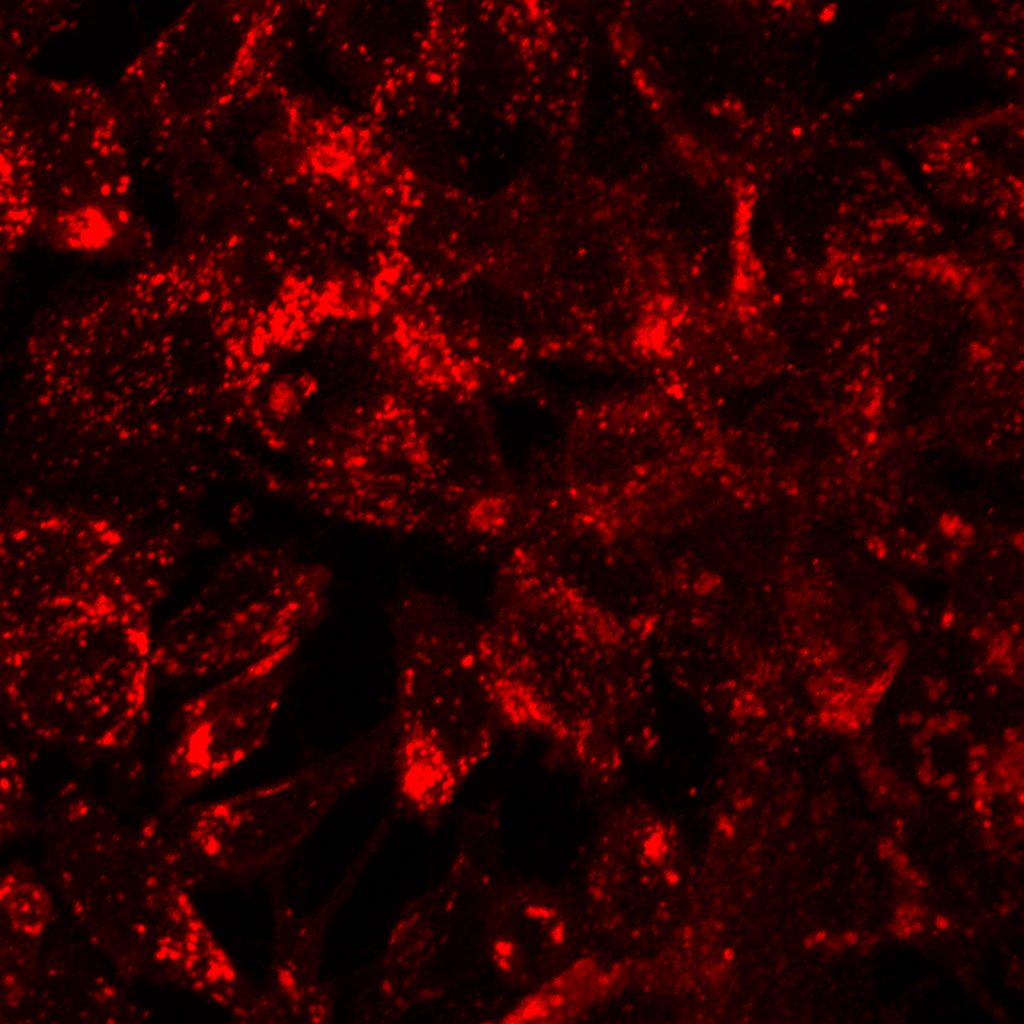

Supplement: Supplementary file 13 — Figure EV2 Source Data [file 44319_2026_751_MOESM13_ESM.zip › Raw_data_Figure EV2/Figure EV2E/WT shGq LAMP1.tif]

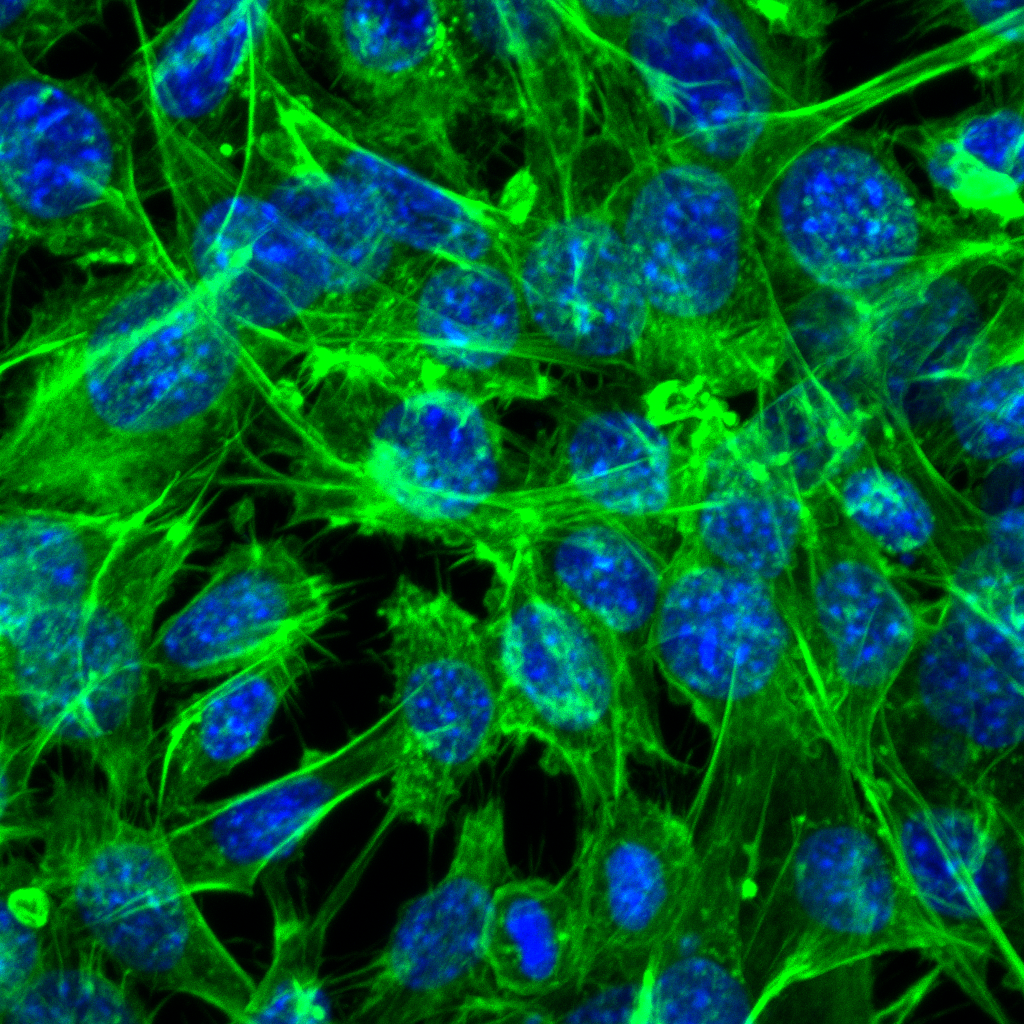

Supplement: Supplementary file 13 — Figure EV2 Source Data [file 44319_2026_751_MOESM13_ESM.zip › Raw_data_Figure EV2/Figure EV2E/WT shGq nuclei Factin.tif]

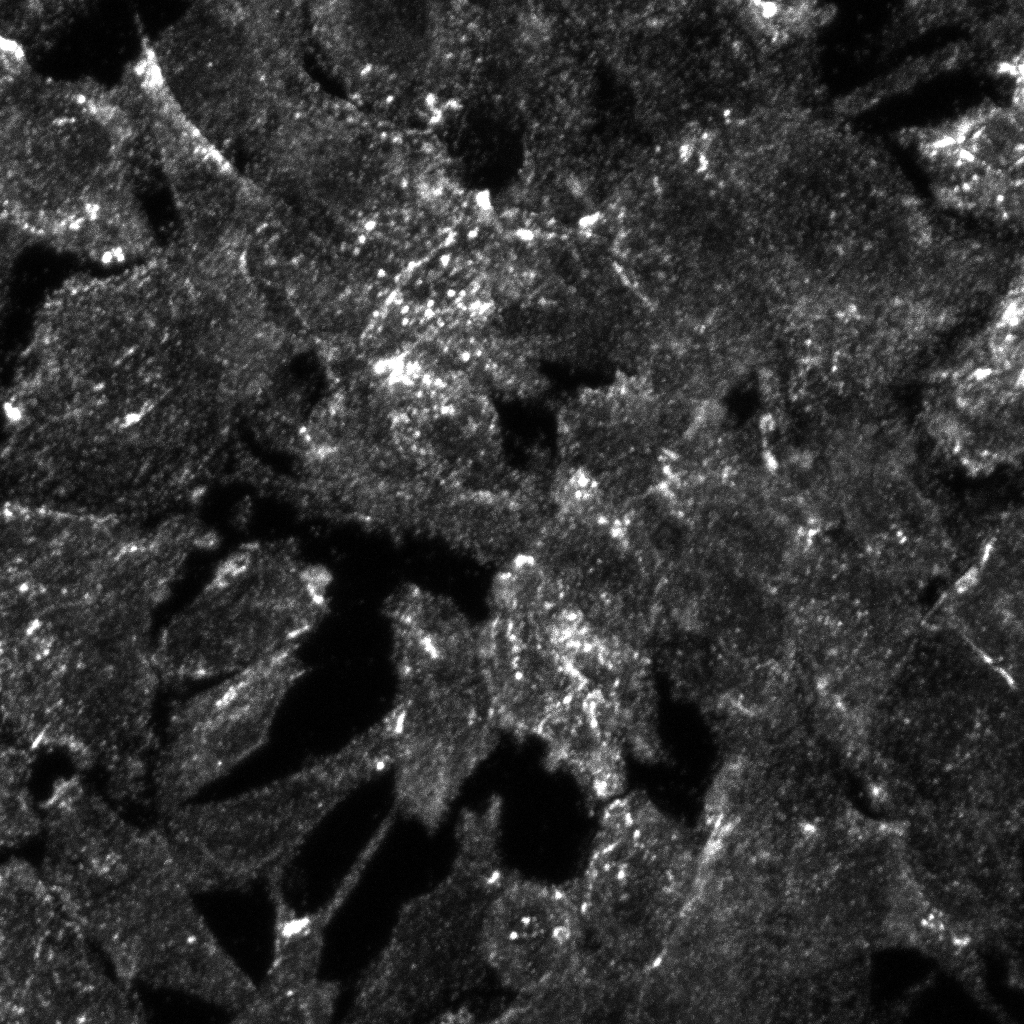

Supplement: Supplementary file 13 — Figure EV2 Source Data [file 44319_2026_751_MOESM13_ESM.zip › Raw_data_Figure EV2/Figure EV2E/WT shGq PTRF.tif]
