# Supplementary material for: Loss of Gαq reshapes fibroblast traits and drives tumor-stroma remodeling in oral cancer progression
Source: EMBO Rep. 2026 Apr 10;27(10):2639–74. doi: 10.1038/s44319-026-00751-2 (PMC13219523; doi:10.1038/s44319-026-00751-2)
Supplement: Supplementary file 11 — Source data Fig. 7 [file 44319_2026_751_MOESM11_ESM.zip › Raw_data_Figure 7/Figure 7A/raw_blots_7A.pptx]

## Slide 1
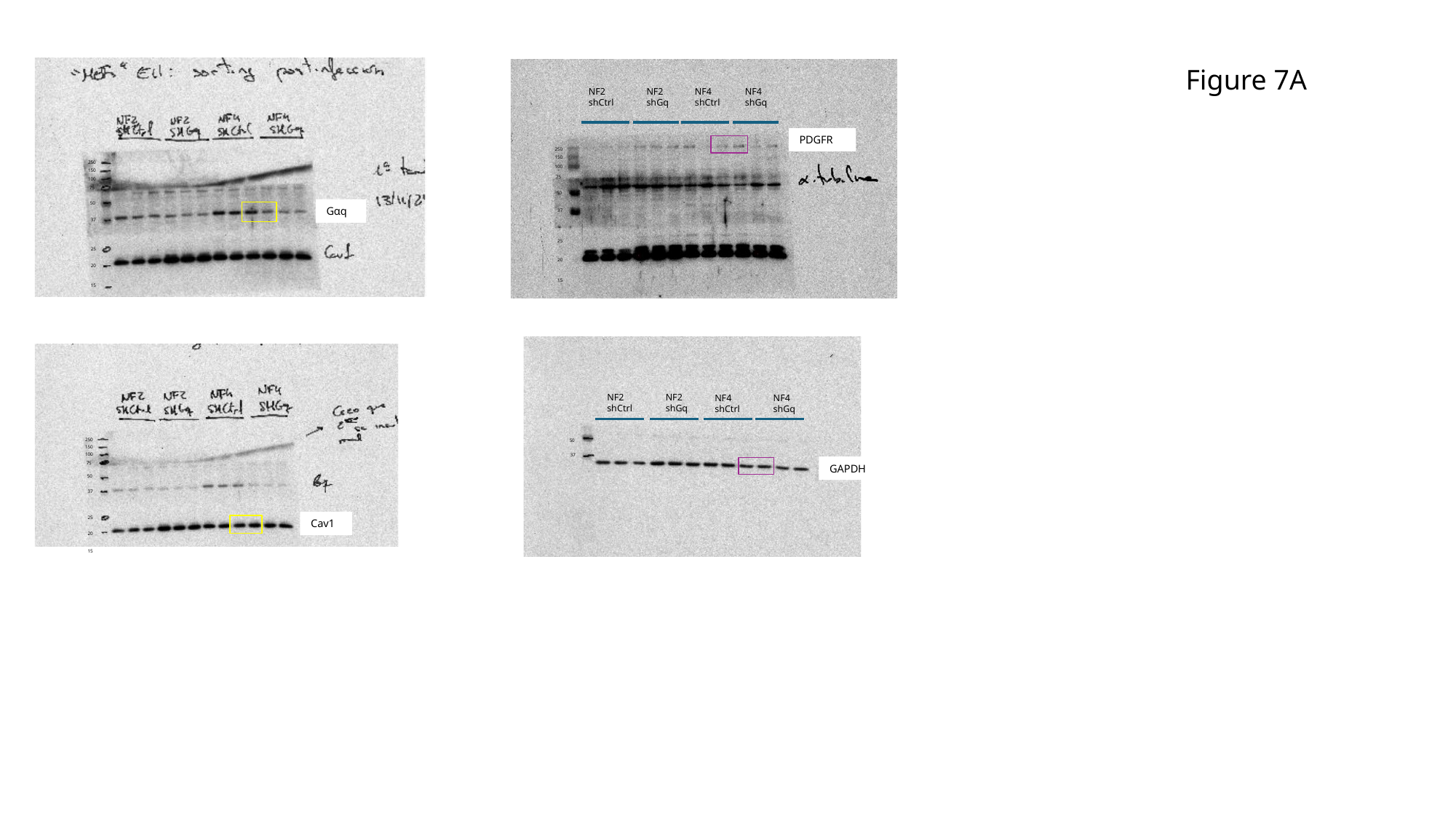

250
150
100
75
50
Gαq
37
25
20
15
Figure 7A
NF4
shGq
NF2
shCtrl
NF2
shGq
NF4
shCtrl
PDGFR
250
150
100
75
50
37
25
20
15
NF2
shCtrl
NF2
shGq
NF4
shCtrl
NF4
shGq
50
37
GAPDH
250
150
100
75
50
37
25
Cav1
20
15
