# Supplementary material for: Loss of Gαq reshapes fibroblast traits and drives tumor-stroma remodeling in oral cancer progression
Source: EMBO Rep. 2026 Apr 10;27(10):2639–74. doi: 10.1038/s44319-026-00751-2 (PMC13219523; doi:10.1038/s44319-026-00751-2)
Supplement: Supplementary file 11 — Source data Fig. 7 [file 44319_2026_751_MOESM11_ESM.zip › Raw_data_Figure 7/Figure 7E/raw_blots_7E.pptx]

## Slide 1
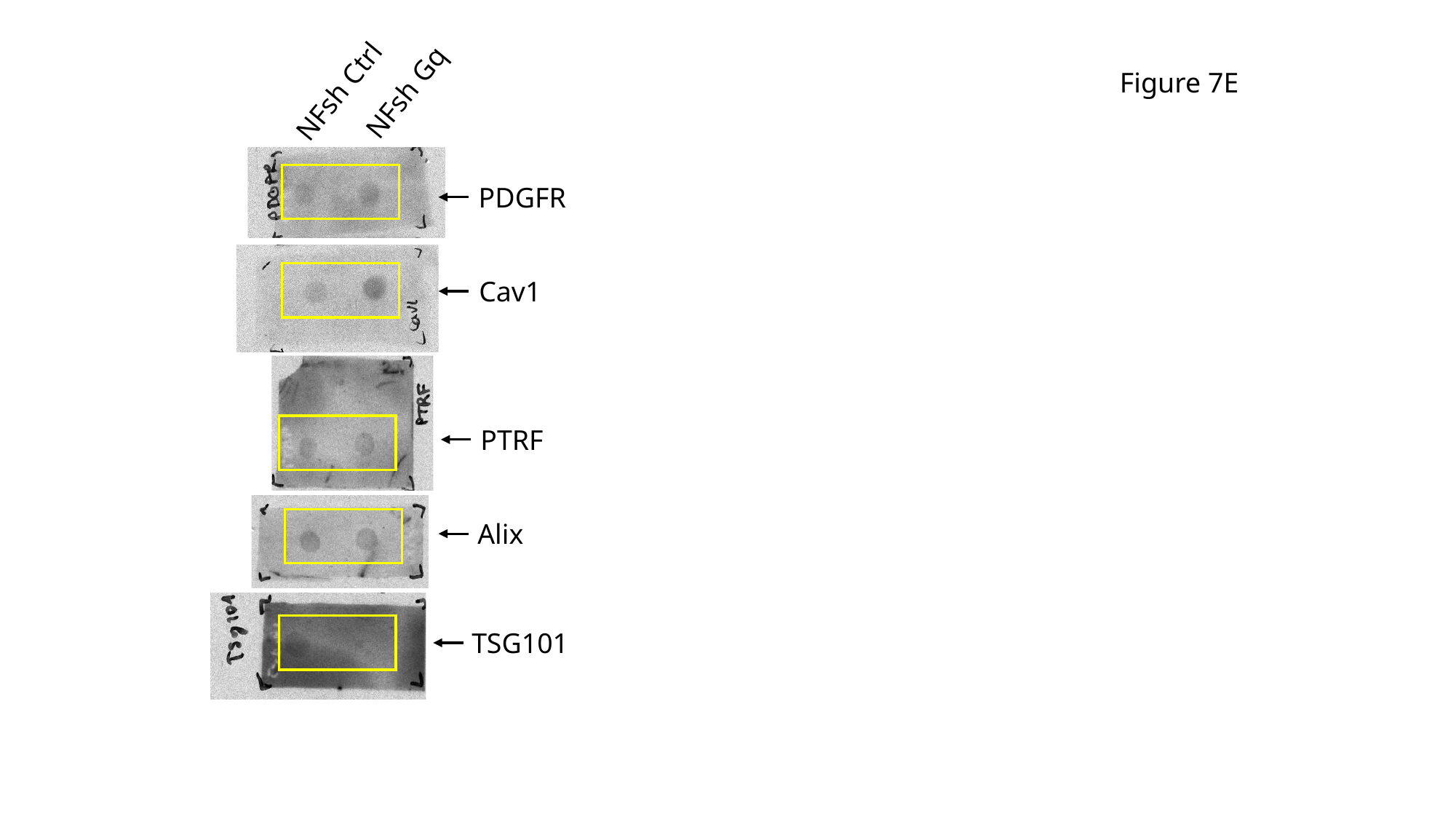

Figure 7E
NFsh Ctrl
NFsh Gq
PDGFR
Cav1
PTRF
Alix
TSG101

## Slide 2
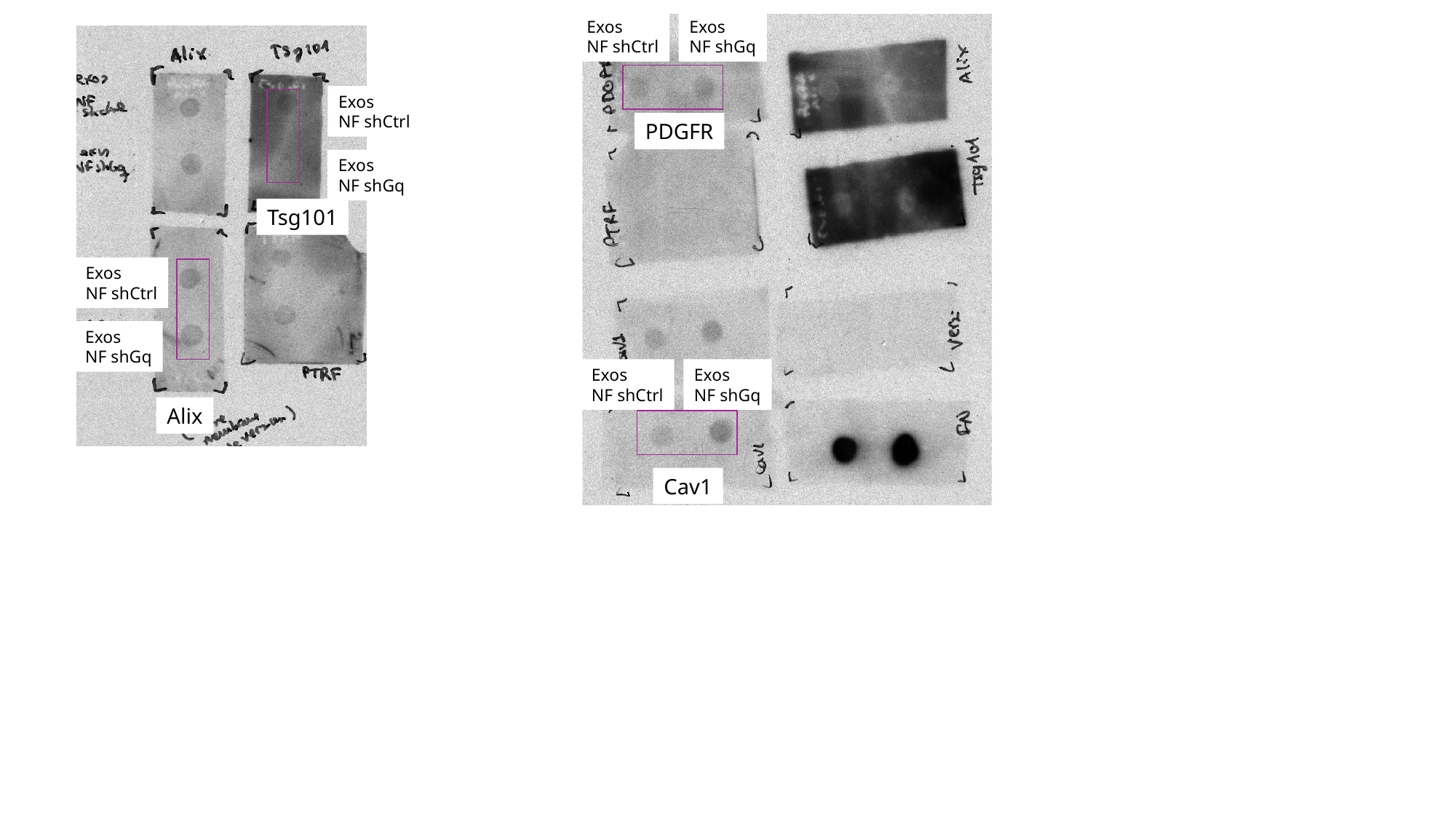

Exos
NF shCtrl
Exos
NF shGq
Exos
NF shCtrl
PDGFR
Exos
NF shGq
Tsg101
Exos
NF shCtrl
Exos
NF shGq
Exos
NF shCtrl
Exos
NF shGq
Alix
Cav1
