# Supplementary material for: Loss of Gαq reshapes fibroblast traits and drives tumor-stroma remodeling in oral cancer progression
Source: EMBO Rep. 2026 Apr 10;27(10):2639–74. doi: 10.1038/s44319-026-00751-2 (PMC13219523; doi:10.1038/s44319-026-00751-2)
Supplement: Supplementary file 13 — Figure EV2 Source Data [file 44319_2026_751_MOESM13_ESM.zip › Raw_data_Figure EV2/Figure EV2J/Raw_blots_EV2J.pptx]

## Slide 1
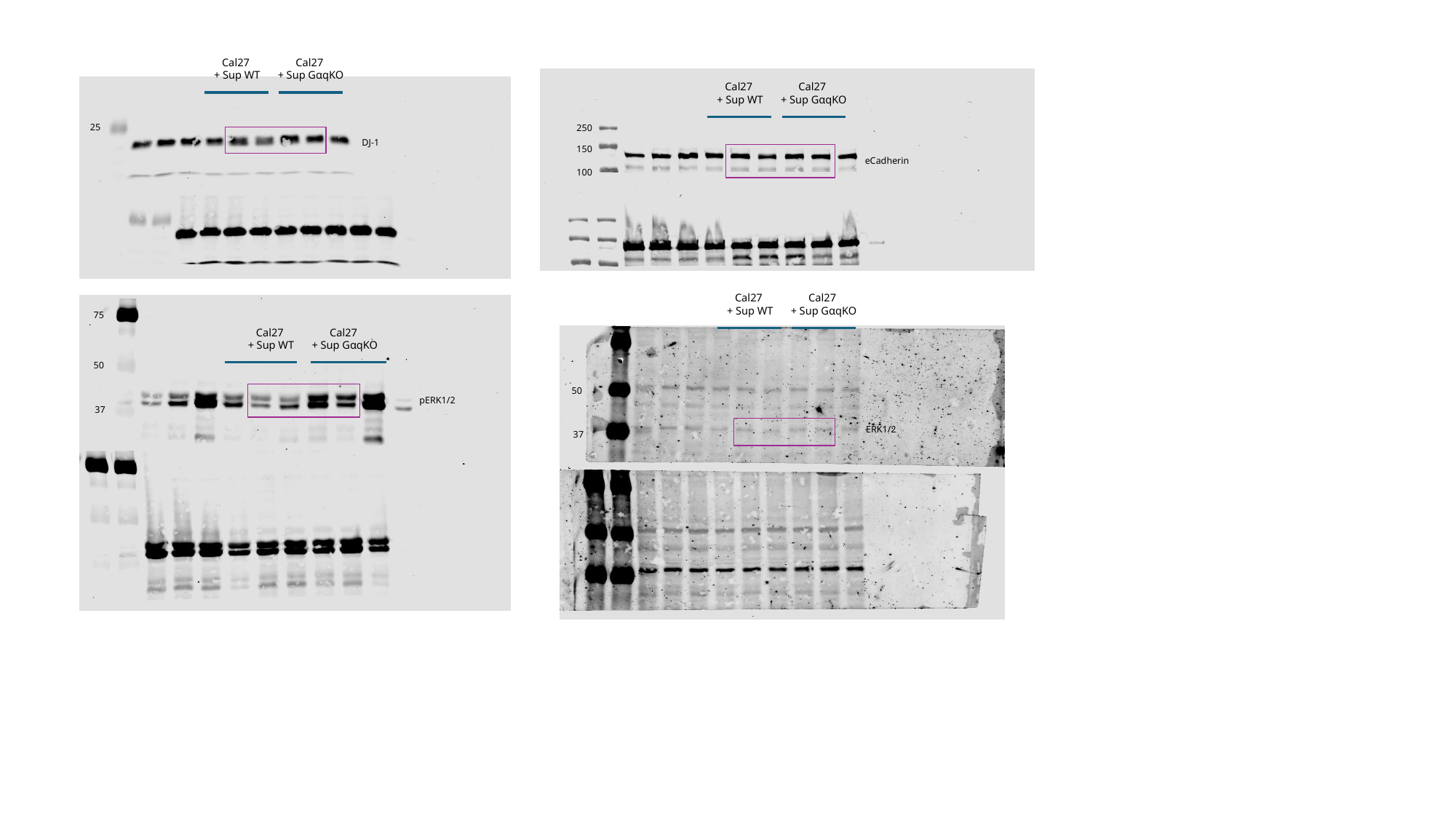

Cal27
+ Sup WT
Cal27
+ Sup GαqKO
Cal27
+ Sup WT
Cal27
+ Sup GαqKO
25
250
DJ-1
150
eCadherin
100
Cal27
+ Sup WT
Cal27
+ Sup GαqKO
75
Cal27
+ Sup WT
Cal27
+ Sup GαqKO
50
50
pERK1/2
37
ERK1/2
37
