# Supplementary figures and images for: Loss of Gαq reshapes fibroblast traits and drives tumor-stroma remodeling in oral cancer progression (part 5 of 5)
Source: EMBO Rep. 2026 Apr 10;27(10):2639–74. doi: 10.1038/s44319-026-00751-2 (PMC13219523; doi:10.1038/s44319-026-00751-2)

## Slide 1
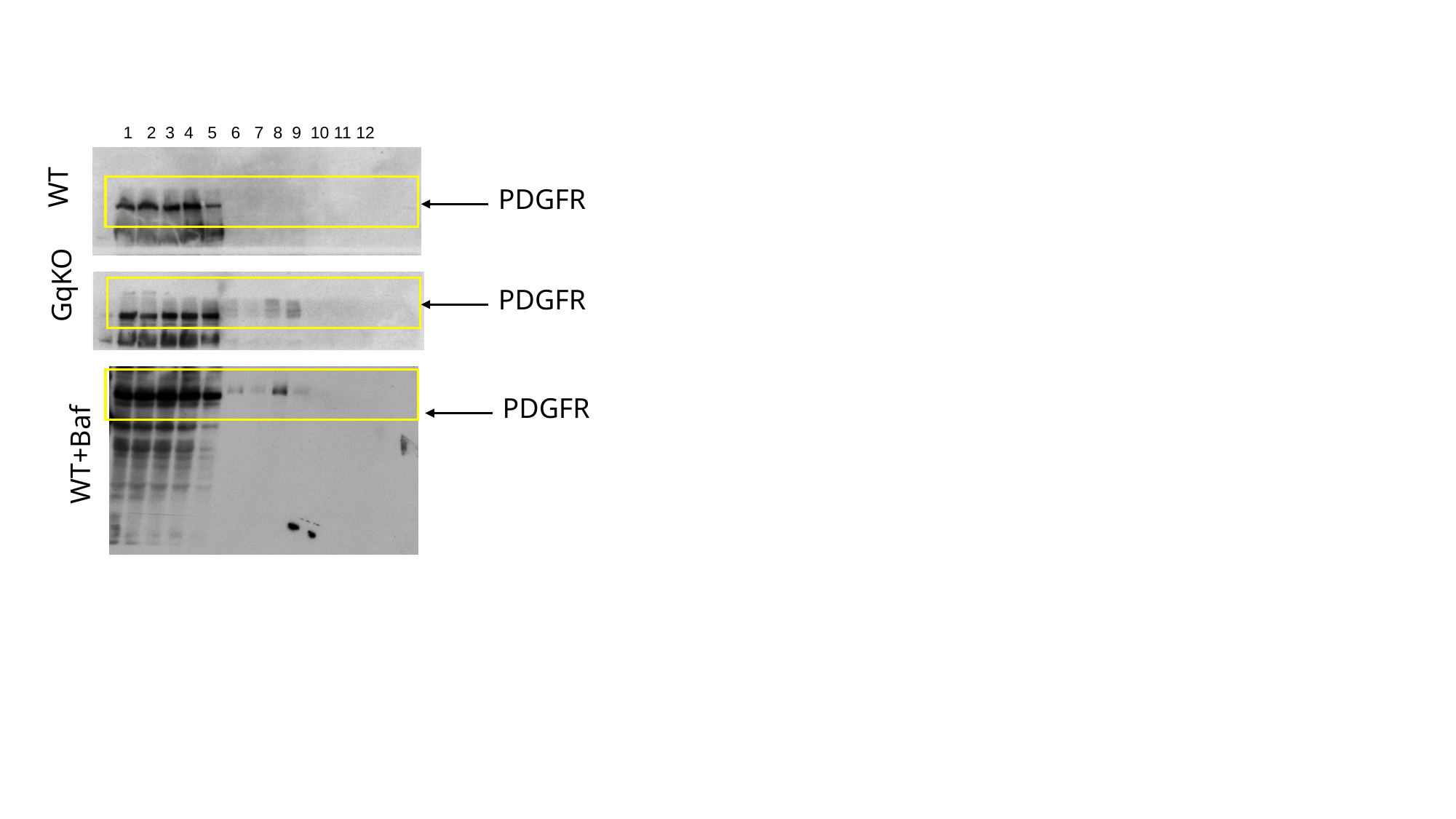

1 2 3 4 5 6 7 8 9 10 11 12
WT
PDGFR
GqKO
PDGFR
PDGFR
WT+Baf

Supplement: Supplementary file 13 — Figure EV2 Source Data [file 44319_2026_751_MOESM13_ESM.zip › Raw_data_Figure EV2/Figure EV2F/raw_blots_S3F.pptx]

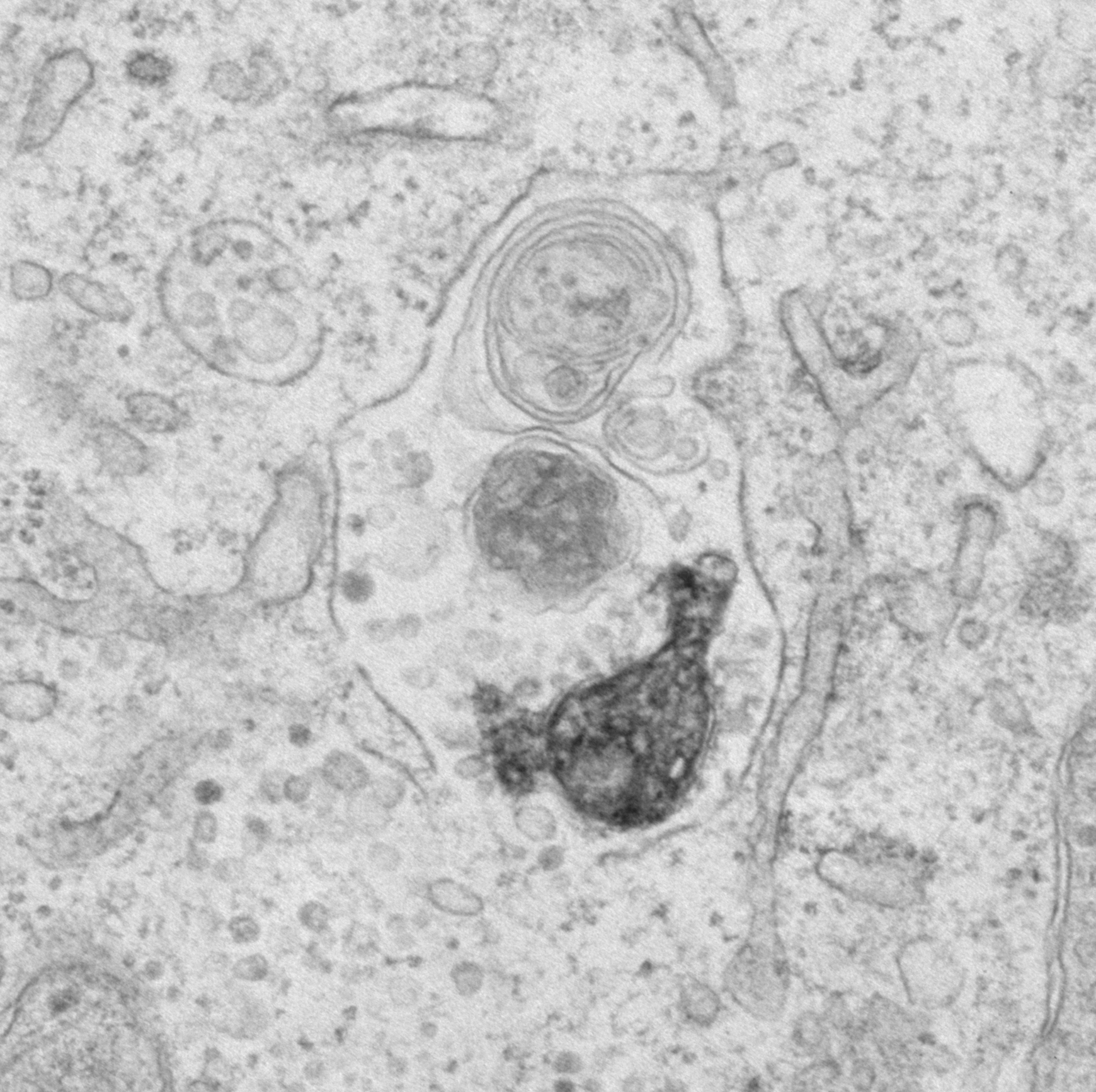

Supplement: Supplementary file 13 — Figure EV2 Source Data [file 44319_2026_751_MOESM13_ESM.zip › Raw_data_Figure EV2/Figure EV2G/10kX_0001 cropped.tif]

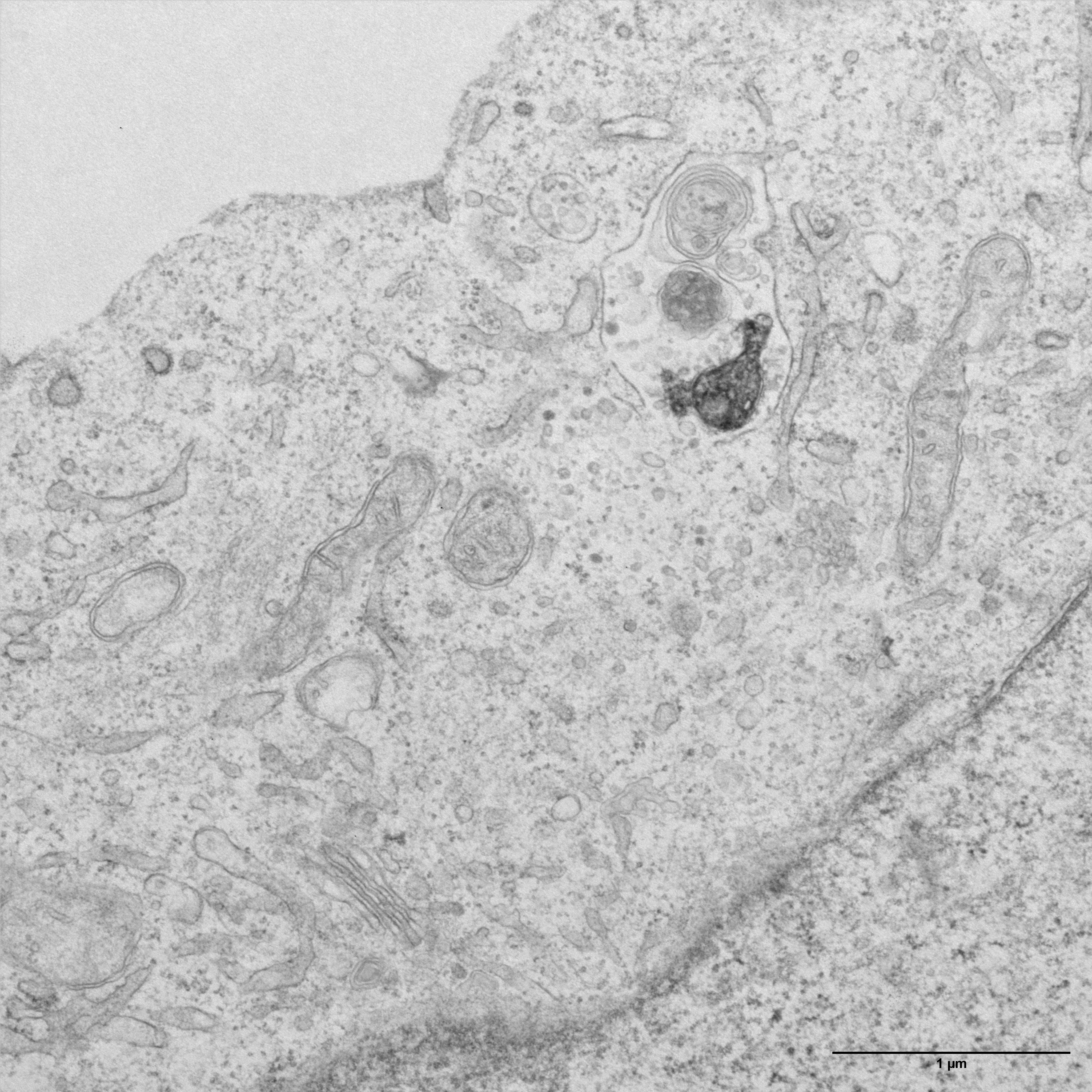

Supplement: Supplementary file 13 — Figure EV2 Source Data [file 44319_2026_751_MOESM13_ESM.zip › Raw_data_Figure EV2/Figure EV2G/10kX_0001.tif]

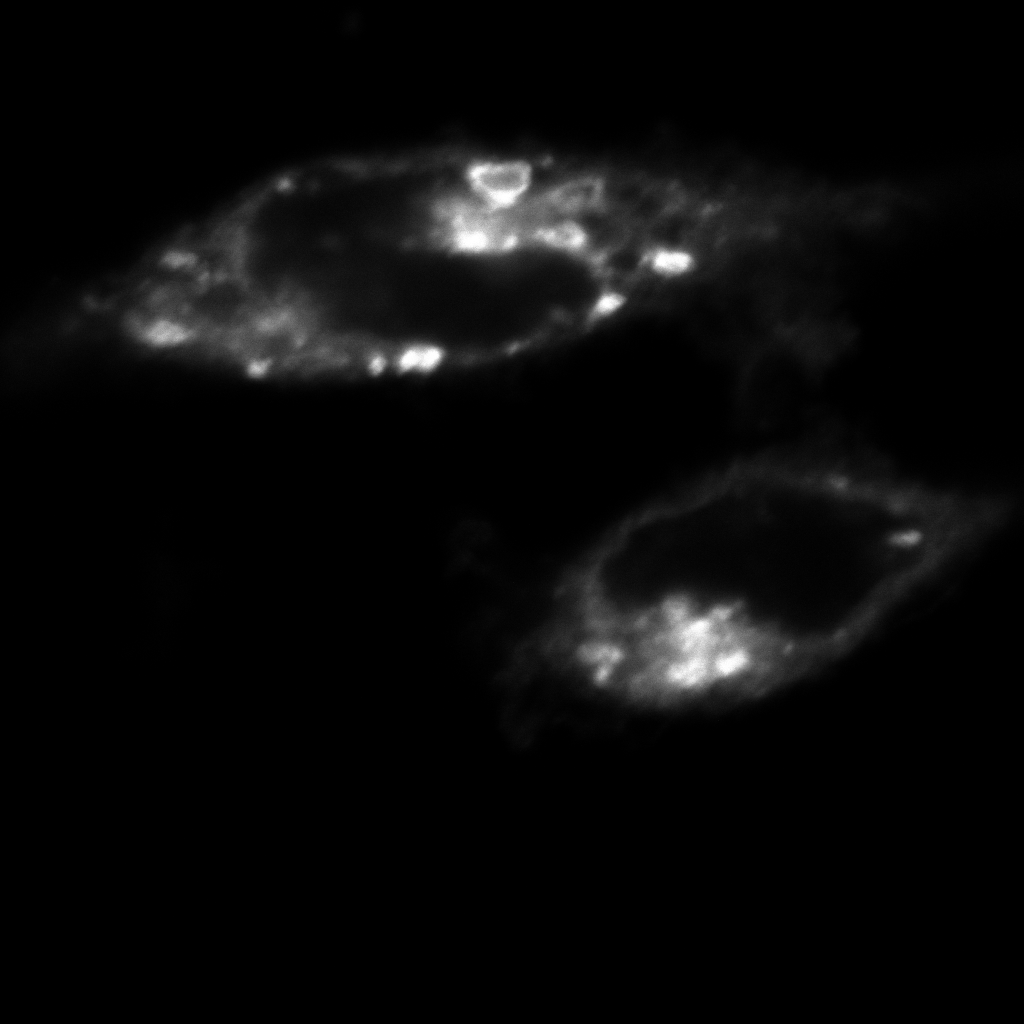

Supplement: Supplementary file 13 — Figure EV2 Source Data [file 44319_2026_751_MOESM13_ESM.zip › Raw_data_Figure EV2/Figure EV2H/C2-GqKO Q79 PDGFR 647.tif]

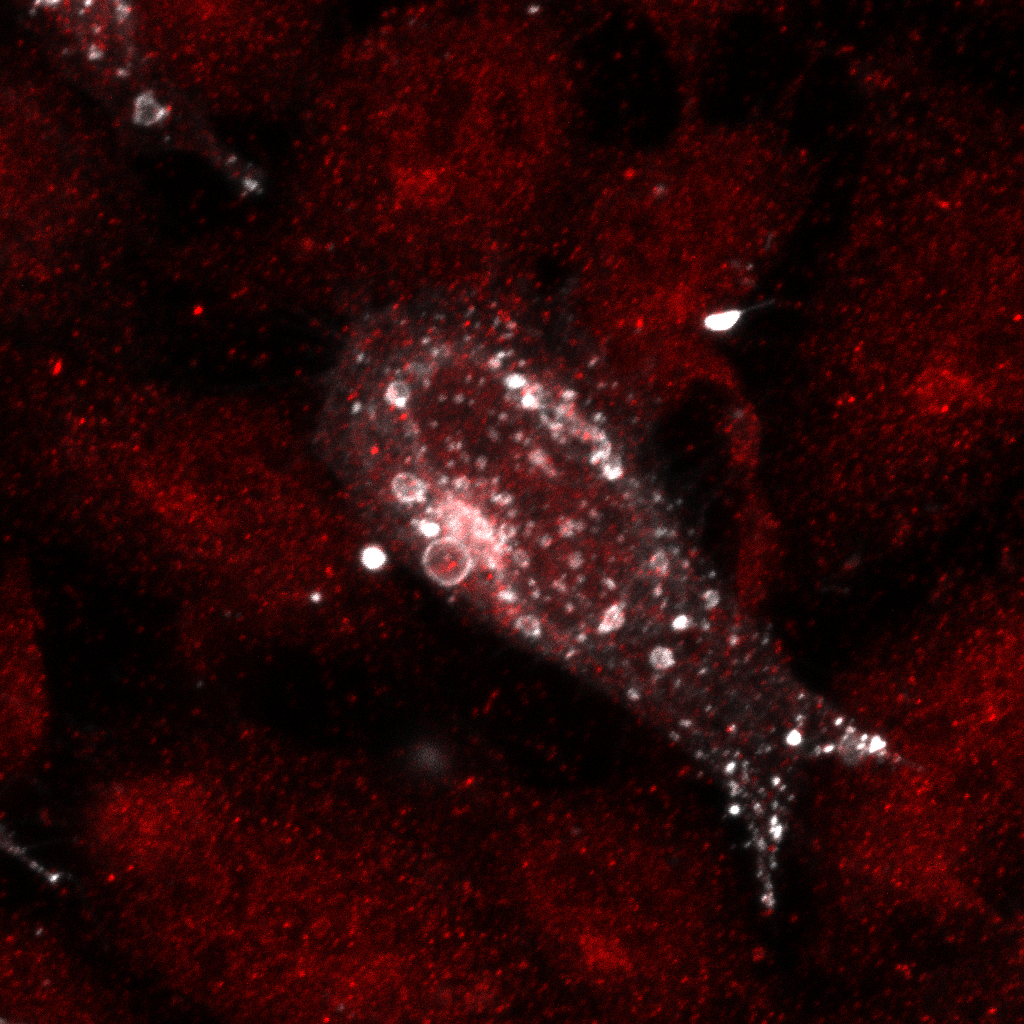

Supplement: Supplementary file 13 — Figure EV2 Source Data [file 44319_2026_751_MOESM13_ESM.zip › Raw_data_Figure EV2/Figure EV2H/C2-MAX_WT Q79 LAMP2 647 4 merge.tif]

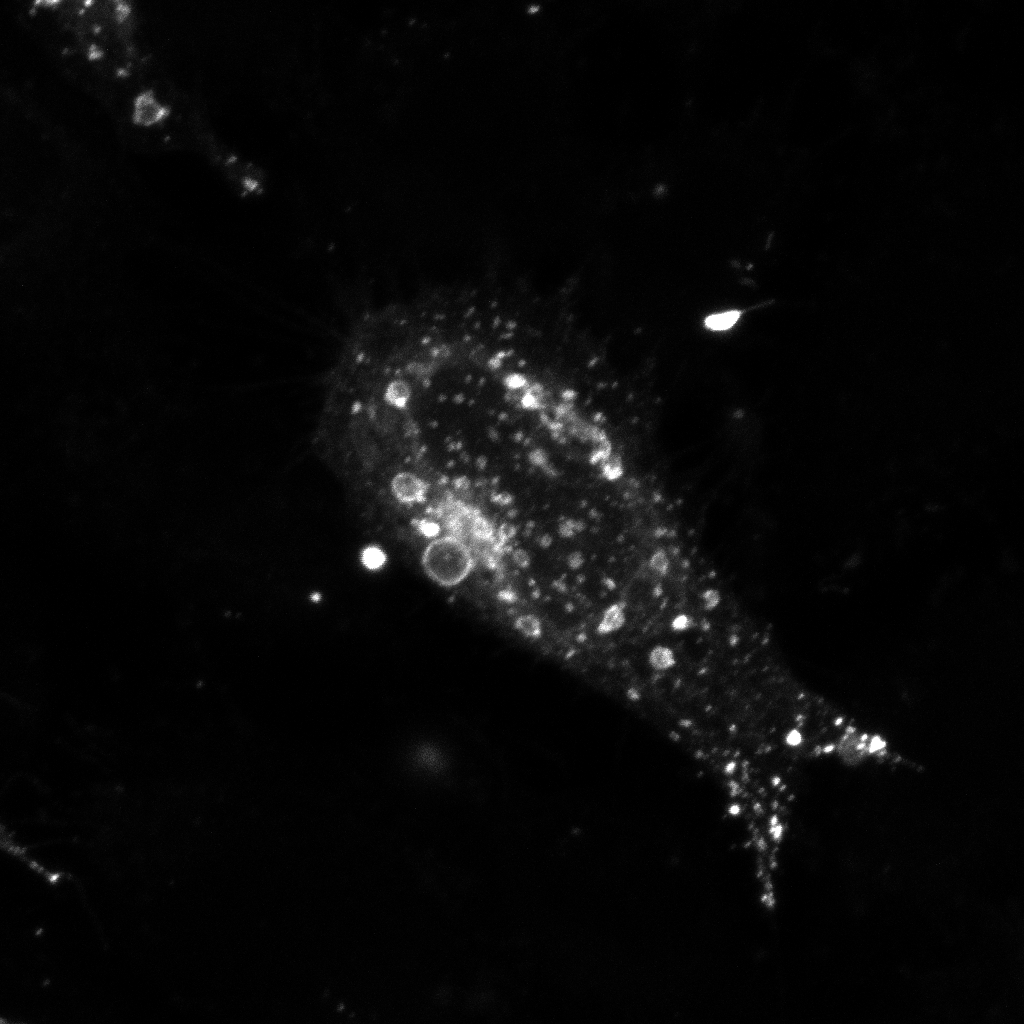

Supplement: Supplementary file 13 — Figure EV2 Source Data [file 44319_2026_751_MOESM13_ESM.zip › Raw_data_Figure EV2/Figure EV2H/C2-MAX_WT Q79 LAMP2 647 4.tif]

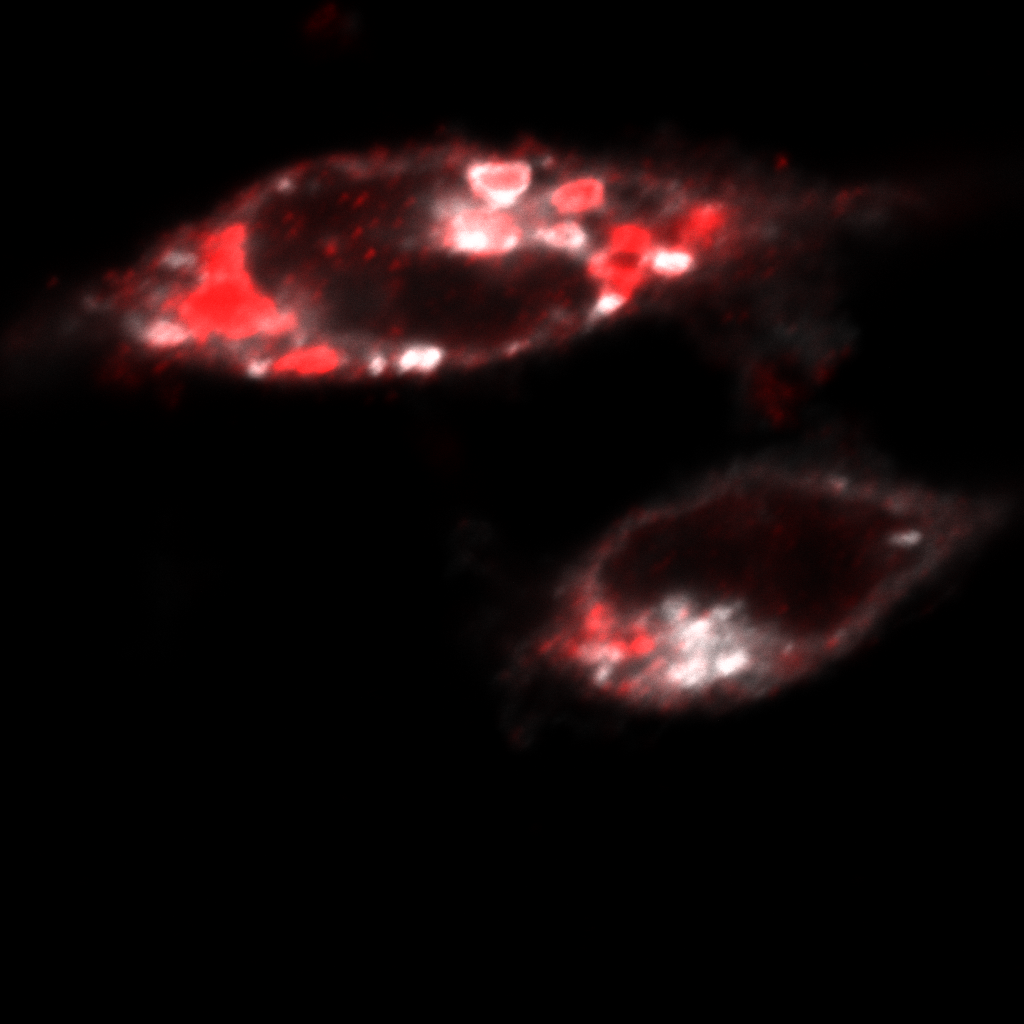

Supplement: Supplementary file 13 — Figure EV2 Source Data [file 44319_2026_751_MOESM13_ESM.zip › Raw_data_Figure EV2/Figure EV2H/C3-GqKO Q79 PDGFR 647 merge.tif]

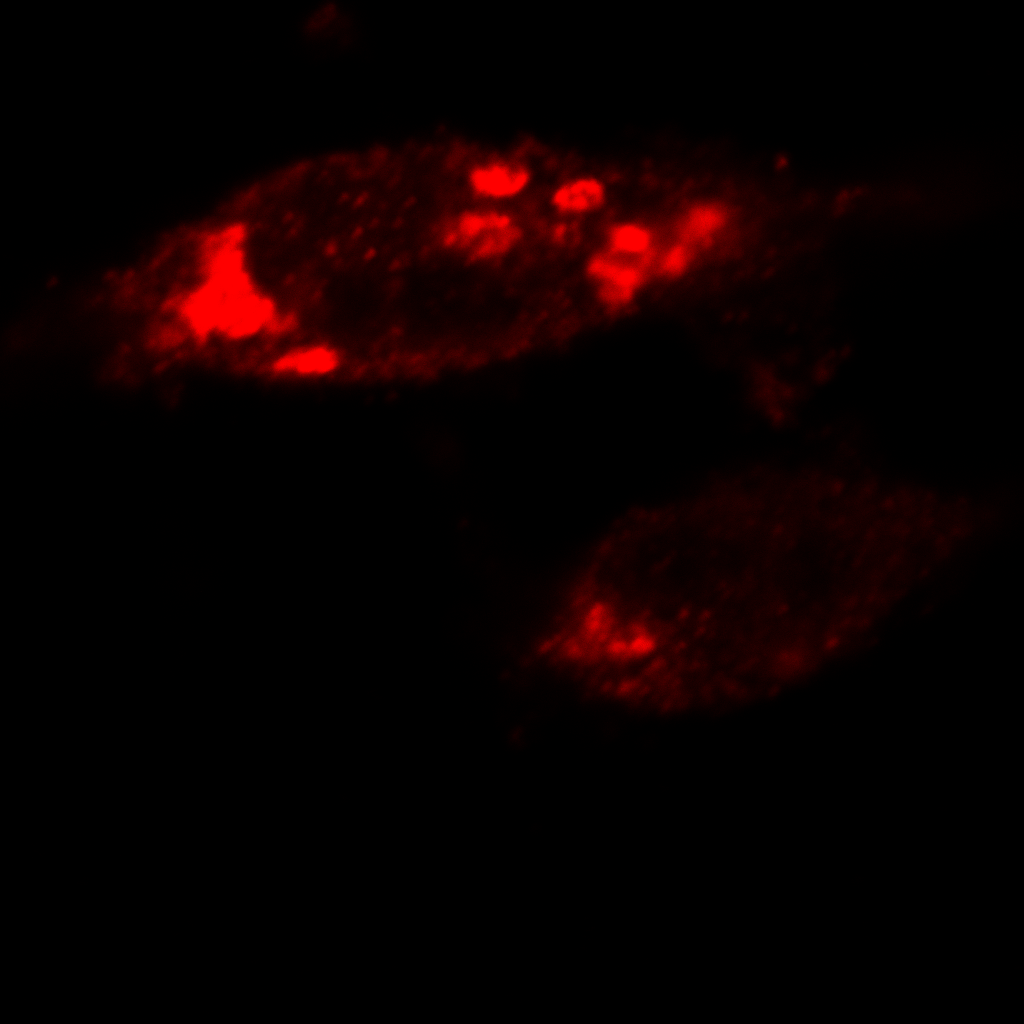

Supplement: Supplementary file 13 — Figure EV2 Source Data [file 44319_2026_751_MOESM13_ESM.zip › Raw_data_Figure EV2/Figure EV2H/C3-GqKO Q79 PDGFR 647.tif]

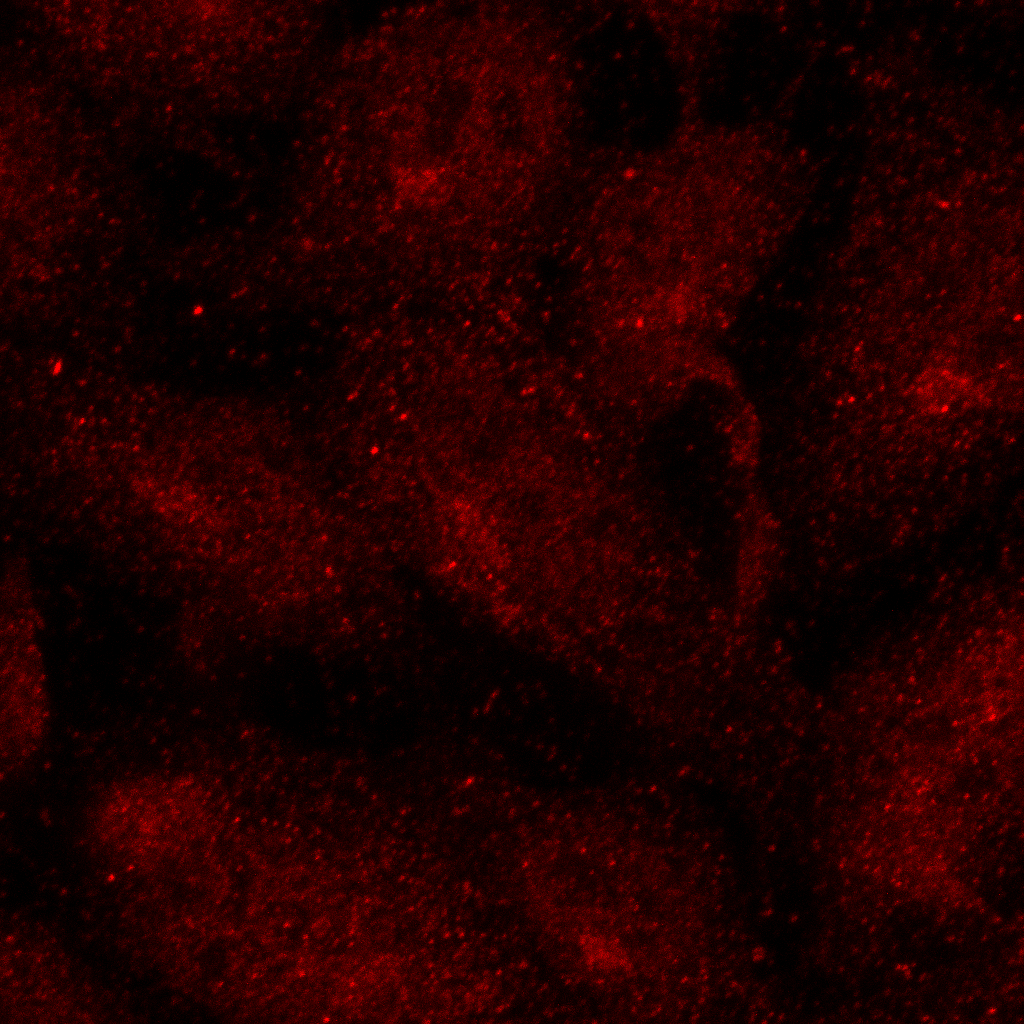

Supplement: Supplementary file 13 — Figure EV2 Source Data [file 44319_2026_751_MOESM13_ESM.zip › Raw_data_Figure EV2/Figure EV2H/C3-MAX_WT Q79 LAMP2 647 4.tif]

## Slide 1
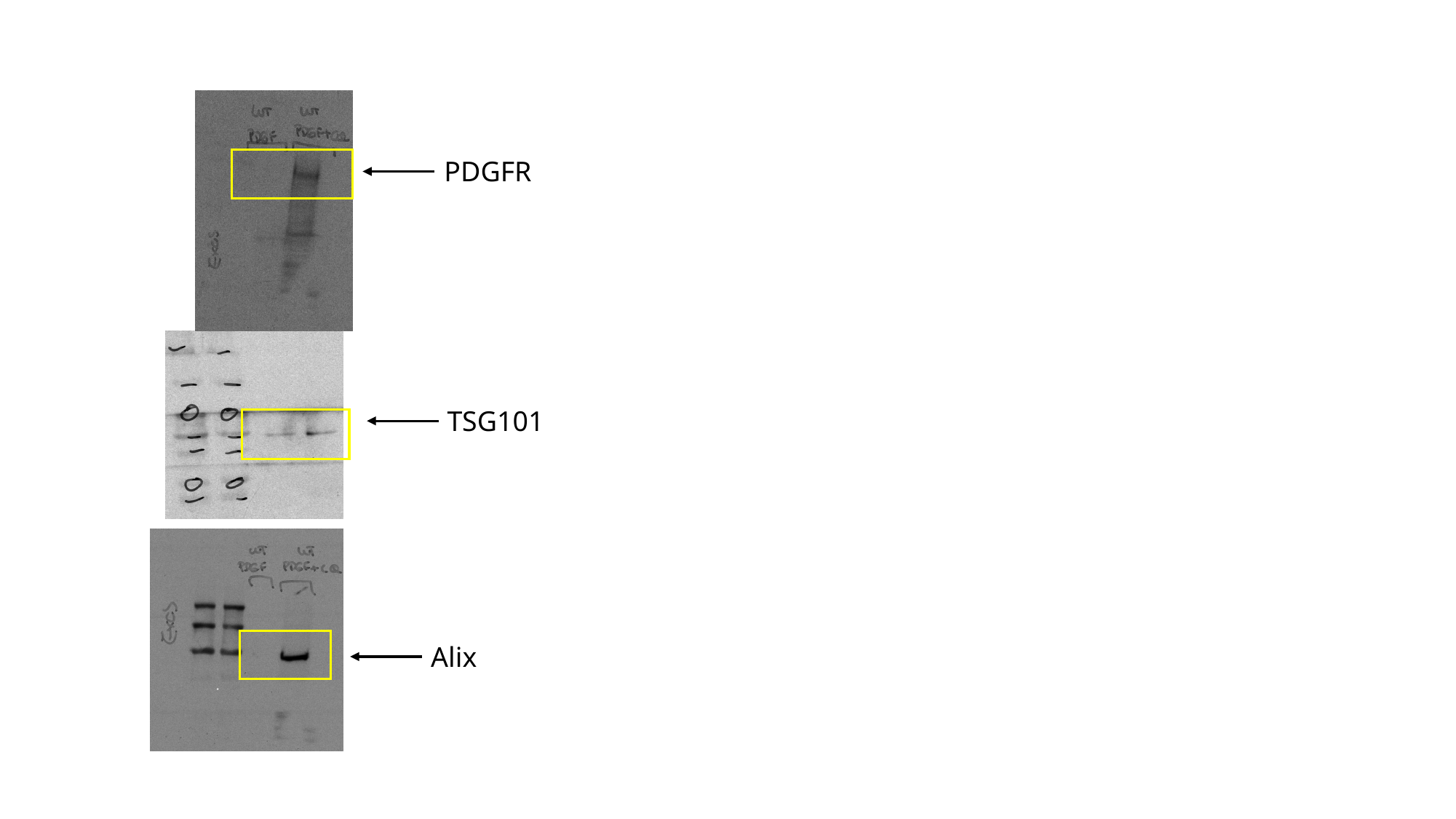

PDGFR
TSG101
Alix

Supplement: Supplementary file 13 — Figure EV2 Source Data [file 44319_2026_751_MOESM13_ESM.zip › Raw_data_Figure EV2/Figure EV2I/raw_data_EV2I.pptx]

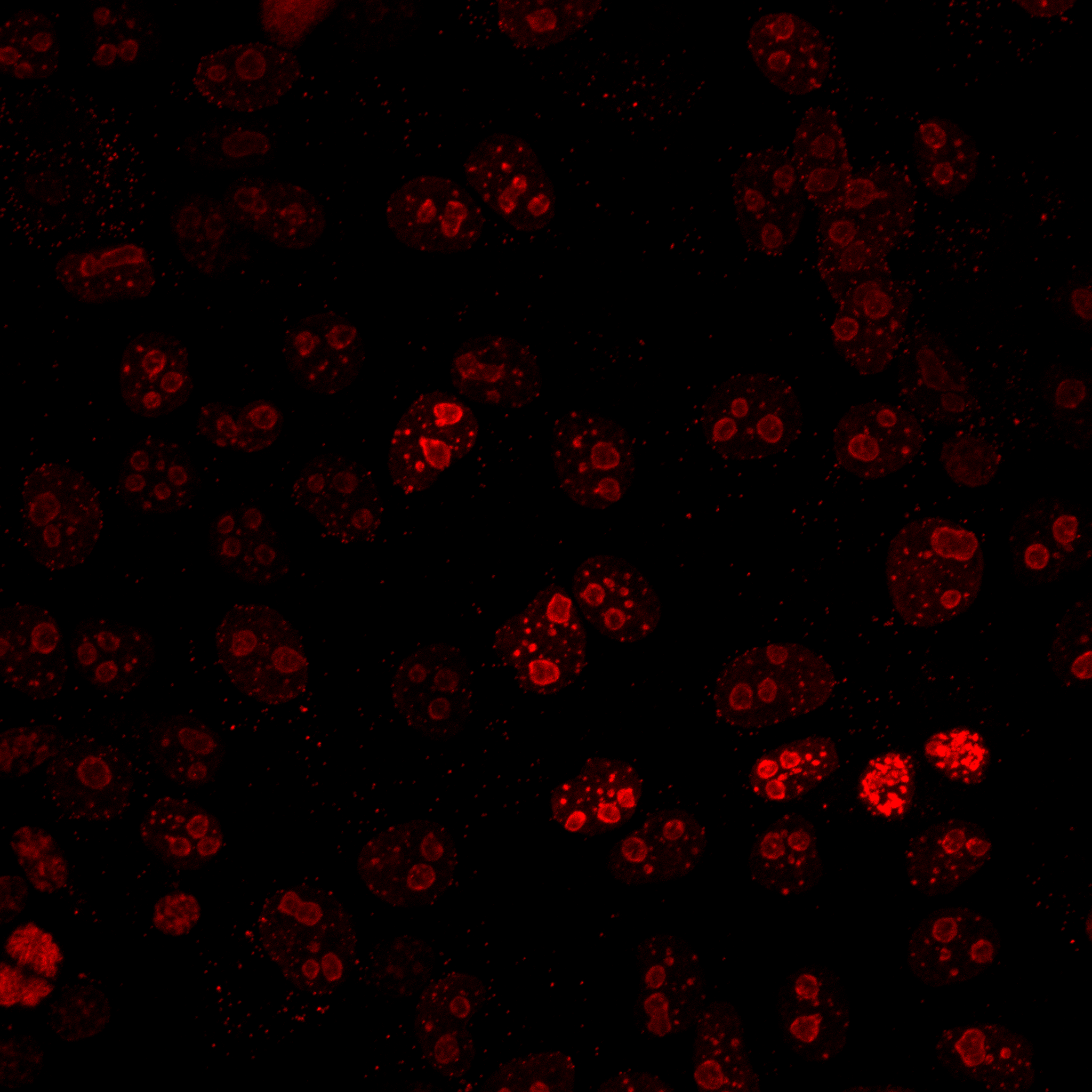

Supplement: Supplementary file 13 — Figure EV2 Source Data [file 44319_2026_751_MOESM13_ESM.zip › Raw_data_Figure EV2/Figure EV2K/C2-MAX_Cal27 + AG1295 + Exosomas MEFs Gq KO Ki67 555.tif]

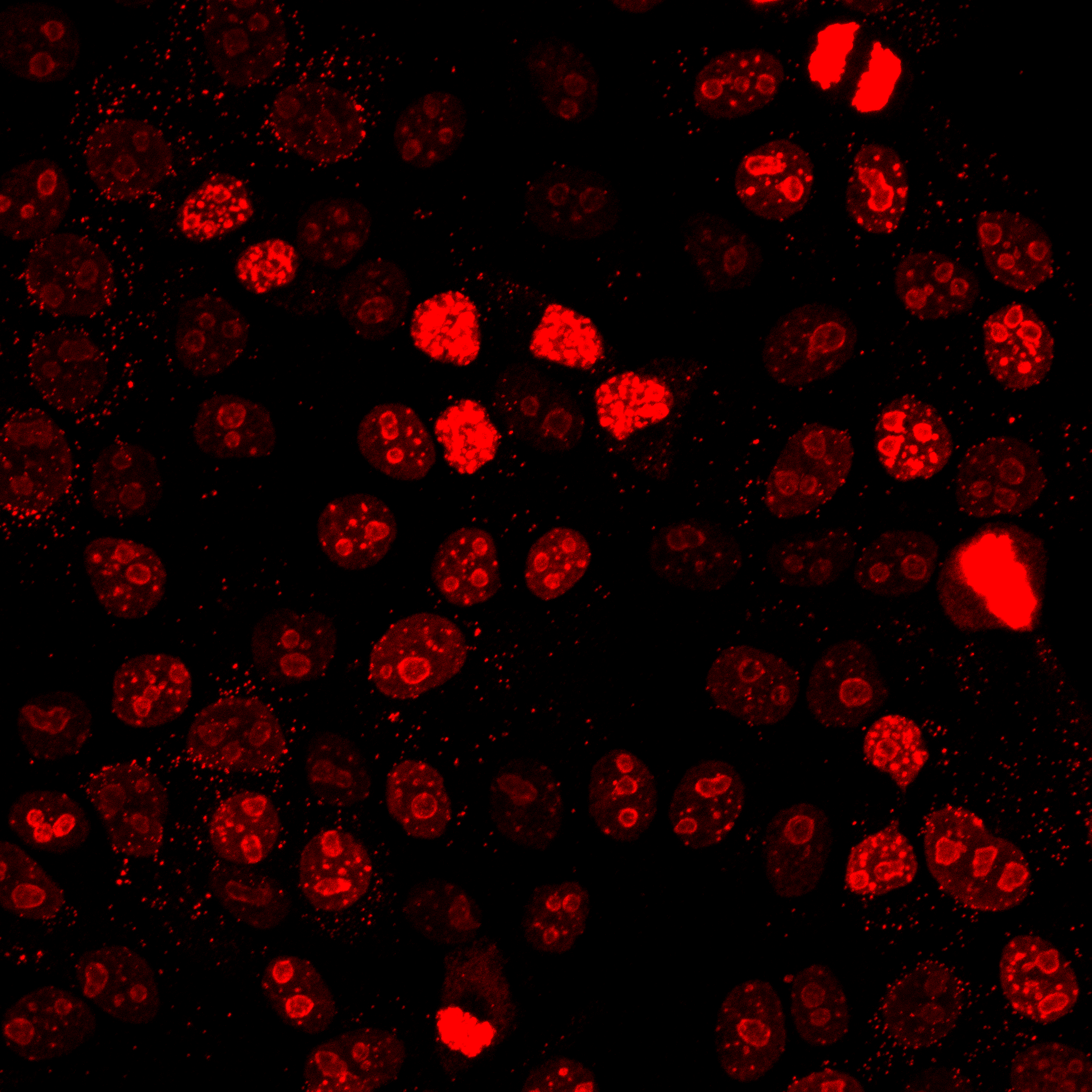

Supplement: Supplementary file 13 — Figure EV2 Source Data [file 44319_2026_751_MOESM13_ESM.zip › Raw_data_Figure EV2/Figure EV2K/C2-MAX_Cal27 + Exosomas MEFs Gq KO Ki67 555.tif]

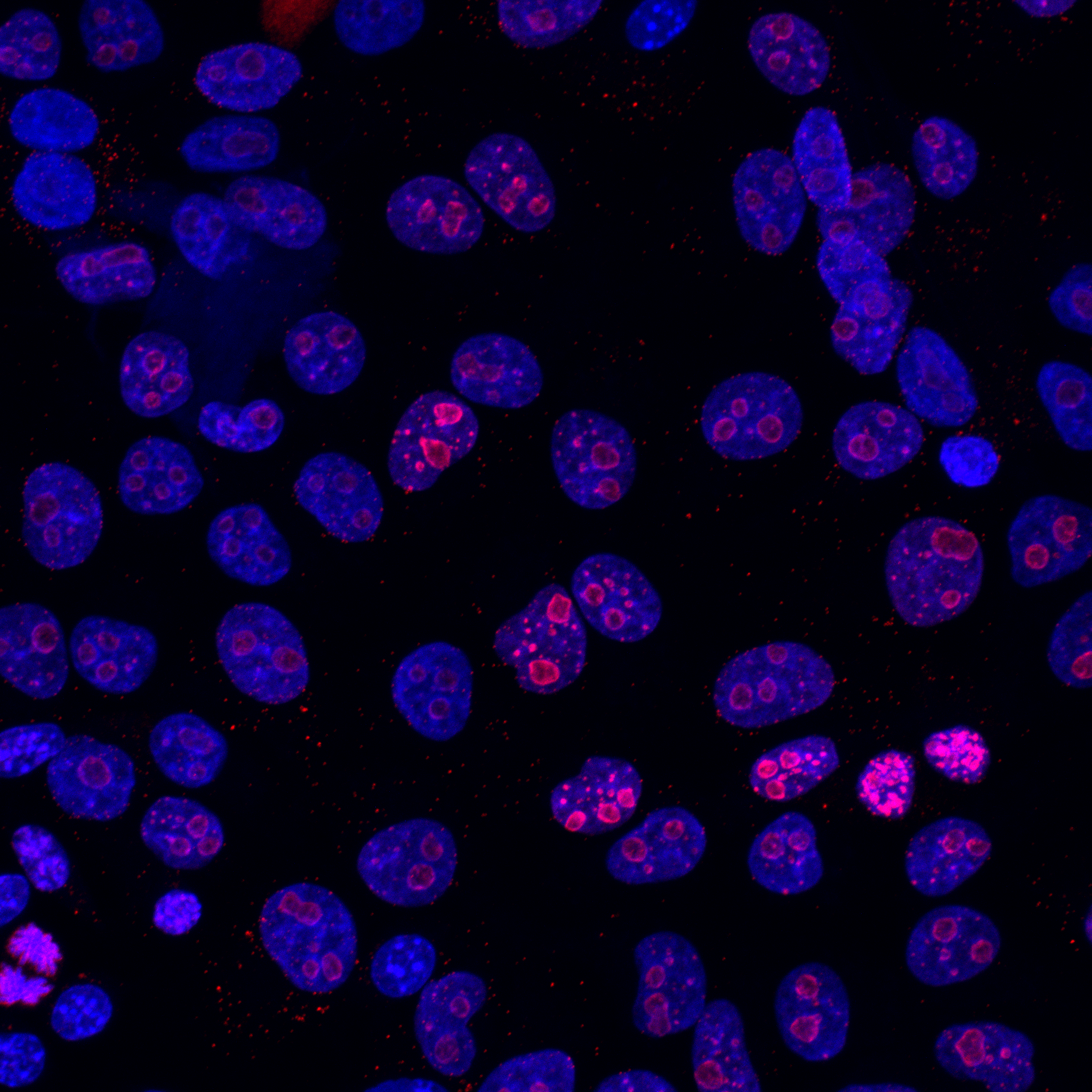

Supplement: Supplementary file 13 — Figure EV2 Source Data [file 44319_2026_751_MOESM13_ESM.zip › Raw_data_Figure EV2/Figure EV2K/Composite_MAX_Cal27 + AG1295 + Exosomas MEFs Gq KO DAPI Ki67 555.tif]

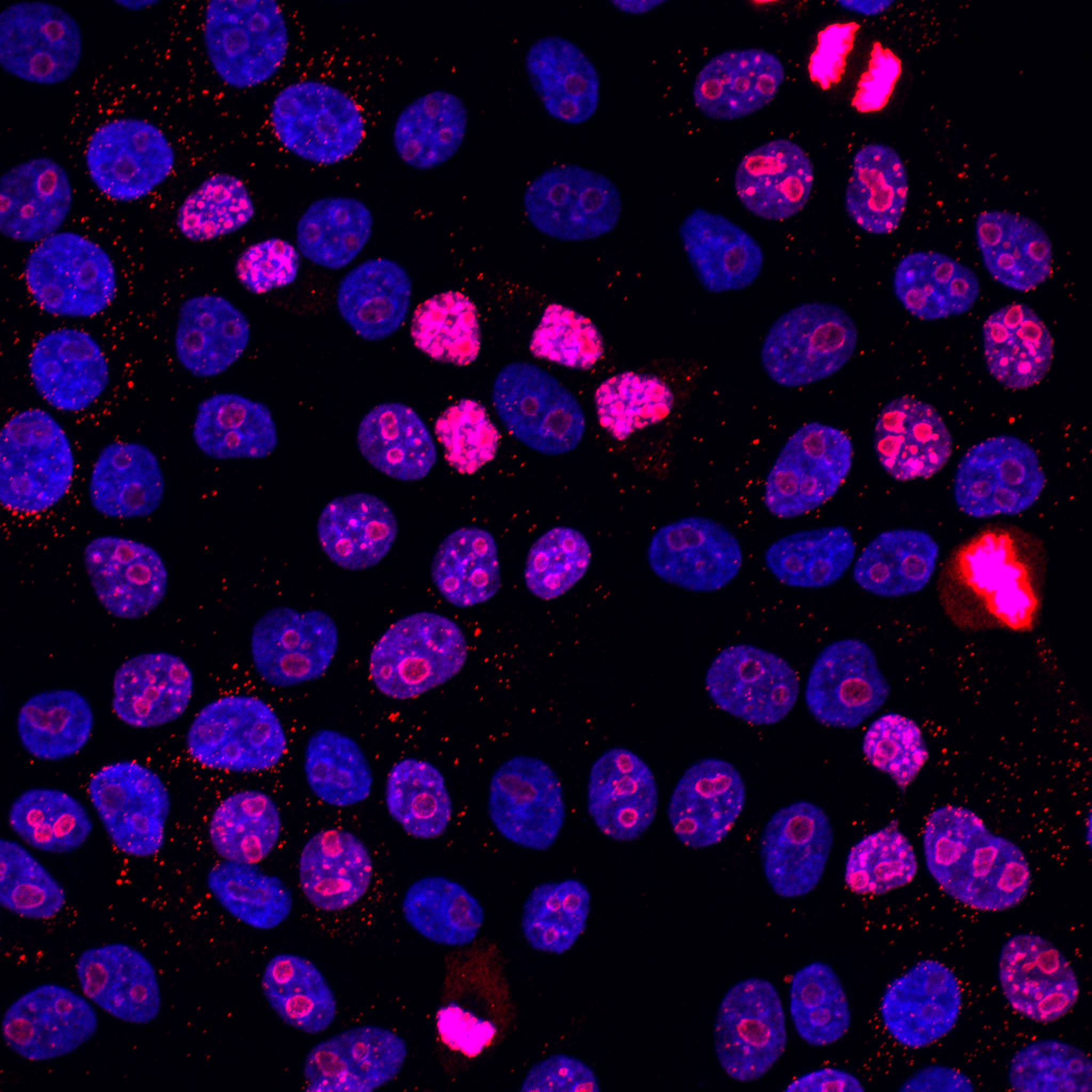

Supplement: Supplementary file 13 — Figure EV2 Source Data [file 44319_2026_751_MOESM13_ESM.zip › Raw_data_Figure EV2/Figure EV2K/Composite_MAX_Cal27 + Exosomas MEFs Gq KO DAPI Ki67 555.tif]

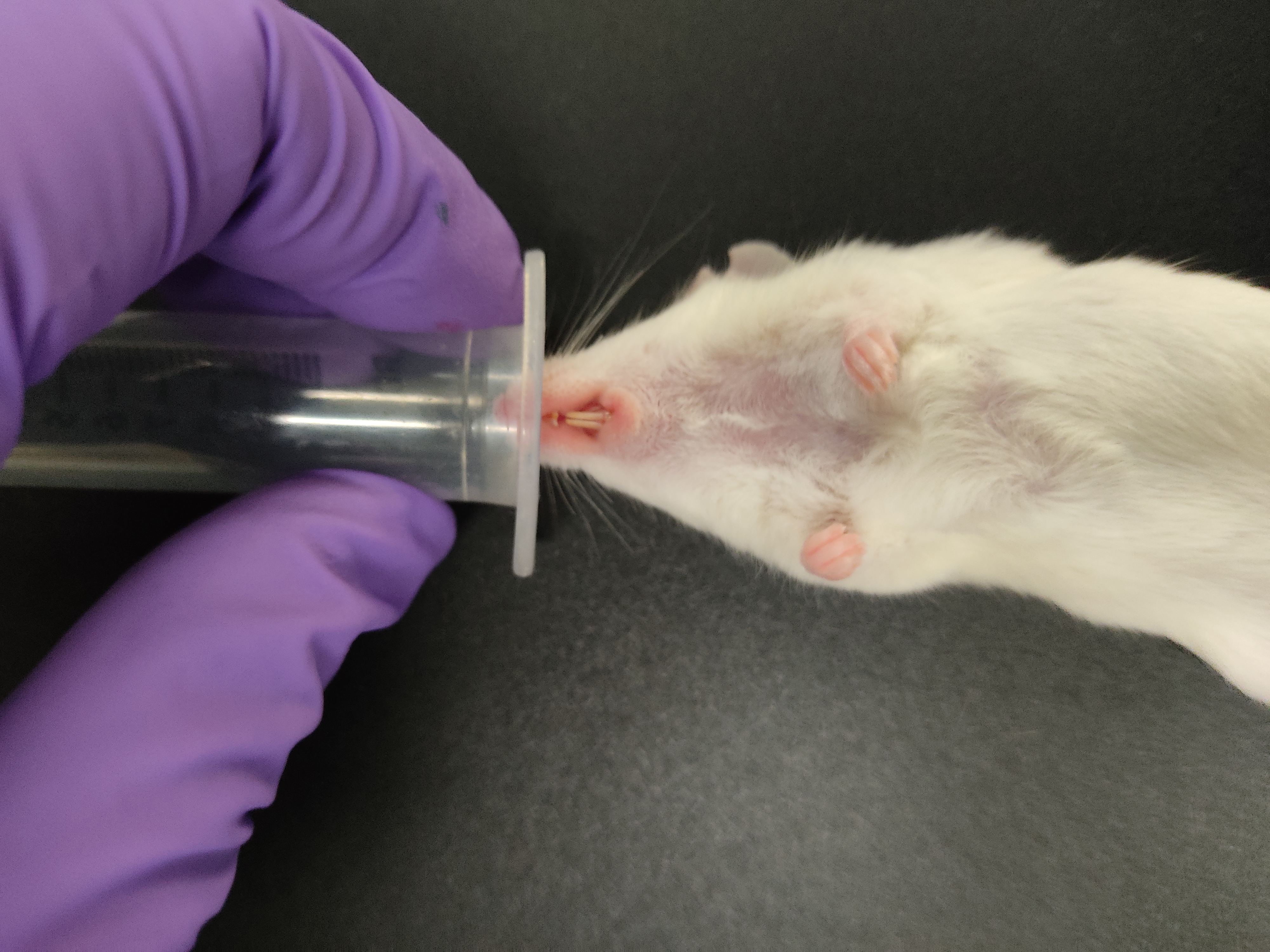

Supplement: Supplementary file 14 — Figure EV3 Source Data [file 44319_2026_751_MOESM14_ESM.zip › Raw_data_Figure EV3/Figure EV3A/IMG_20230123_102506 Cal27 alone.jpg]

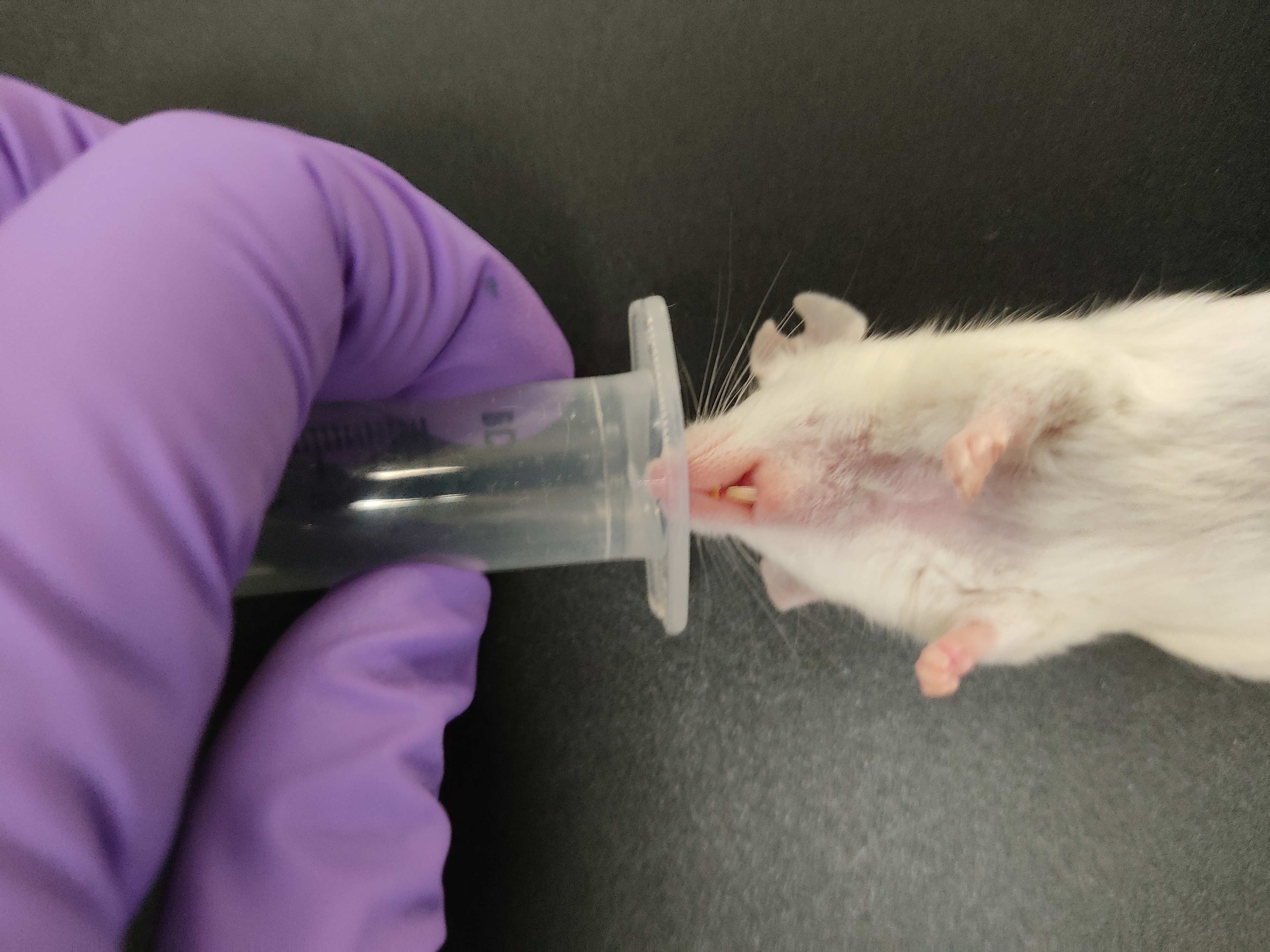

Supplement: Supplementary file 14 — Figure EV3 Source Data [file 44319_2026_751_MOESM14_ESM.zip › Raw_data_Figure EV3/Figure EV3A/IMG_20230123_102633 Cal27+WTMEFs.jpg]

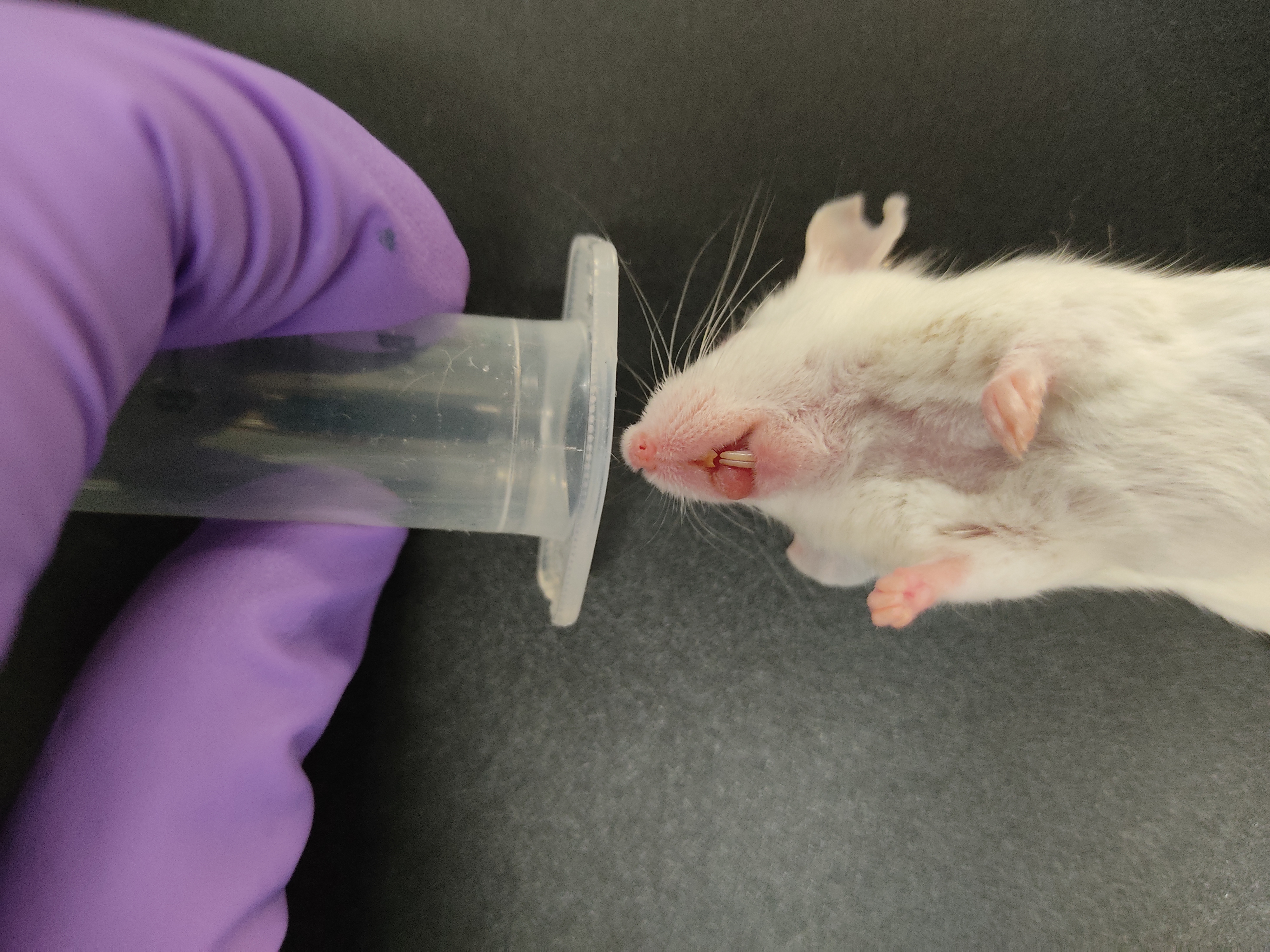

Supplement: Supplementary file 14 — Figure EV3 Source Data [file 44319_2026_751_MOESM14_ESM.zip › Raw_data_Figure EV3/Figure EV3A/IMG_20230123_102812 Cal27+GqKO.jpg]

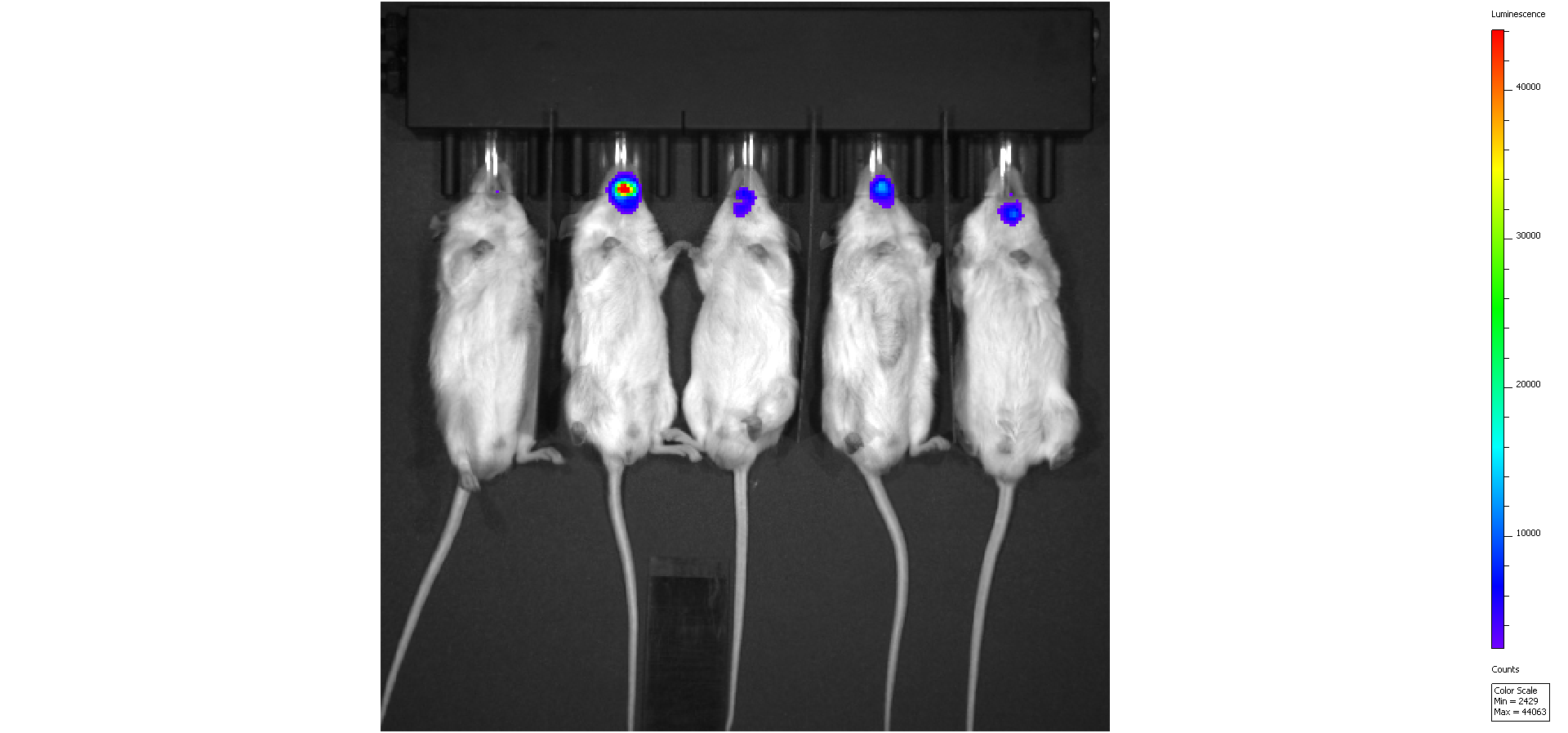

Supplement: Supplementary file 14 — Figure EV3 Source Data [file 44319_2026_751_MOESM14_ESM.zip › Raw_data_Figure EV3/Figure EV3B/INL20240306095831_SEQ/INL20240306095831_001/INL20240306095831_001.PNG]

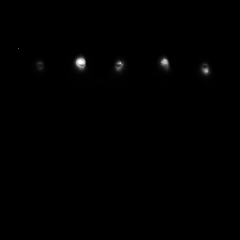

Supplement: Supplementary file 14 — Figure EV3 Source Data [file 44319_2026_751_MOESM14_ESM.zip › Raw_data_Figure EV3/Figure EV3B/INL20240306095831_SEQ/INL20240306095831_001/luminescent.TIF]

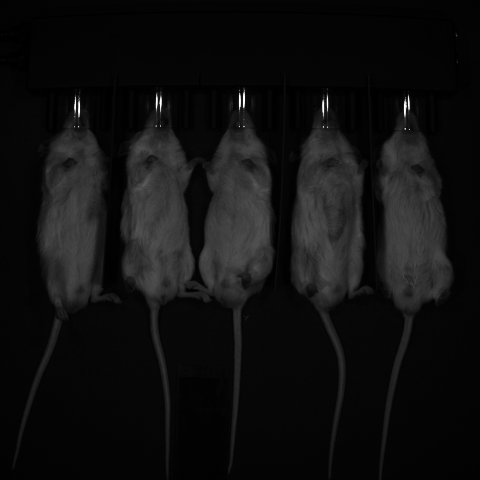

Supplement: Supplementary file 14 — Figure EV3 Source Data [file 44319_2026_751_MOESM14_ESM.zip › Raw_data_Figure EV3/Figure EV3B/INL20240306095831_SEQ/INL20240306095831_001/photograph.TIF]

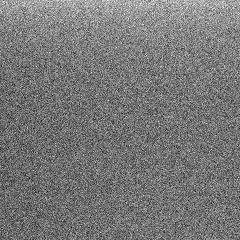

Supplement: Supplementary file 14 — Figure EV3 Source Data [file 44319_2026_751_MOESM14_ESM.zip › Raw_data_Figure EV3/Figure EV3B/INL20240306095831_SEQ/INL20240306095831_001/readbiasonly.TIF]

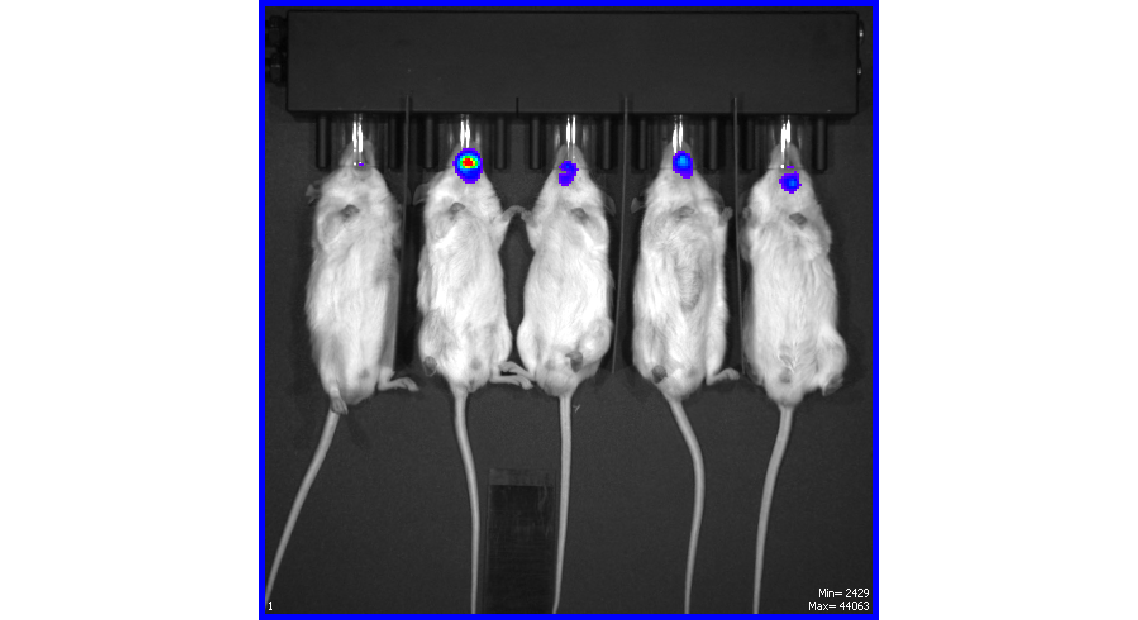

Supplement: Supplementary file 14 — Figure EV3 Source Data [file 44319_2026_751_MOESM14_ESM.zip › Raw_data_Figure EV3/Figure EV3B/INL20240306095831_SEQ/INL20240306095831_SEQ.PNG]

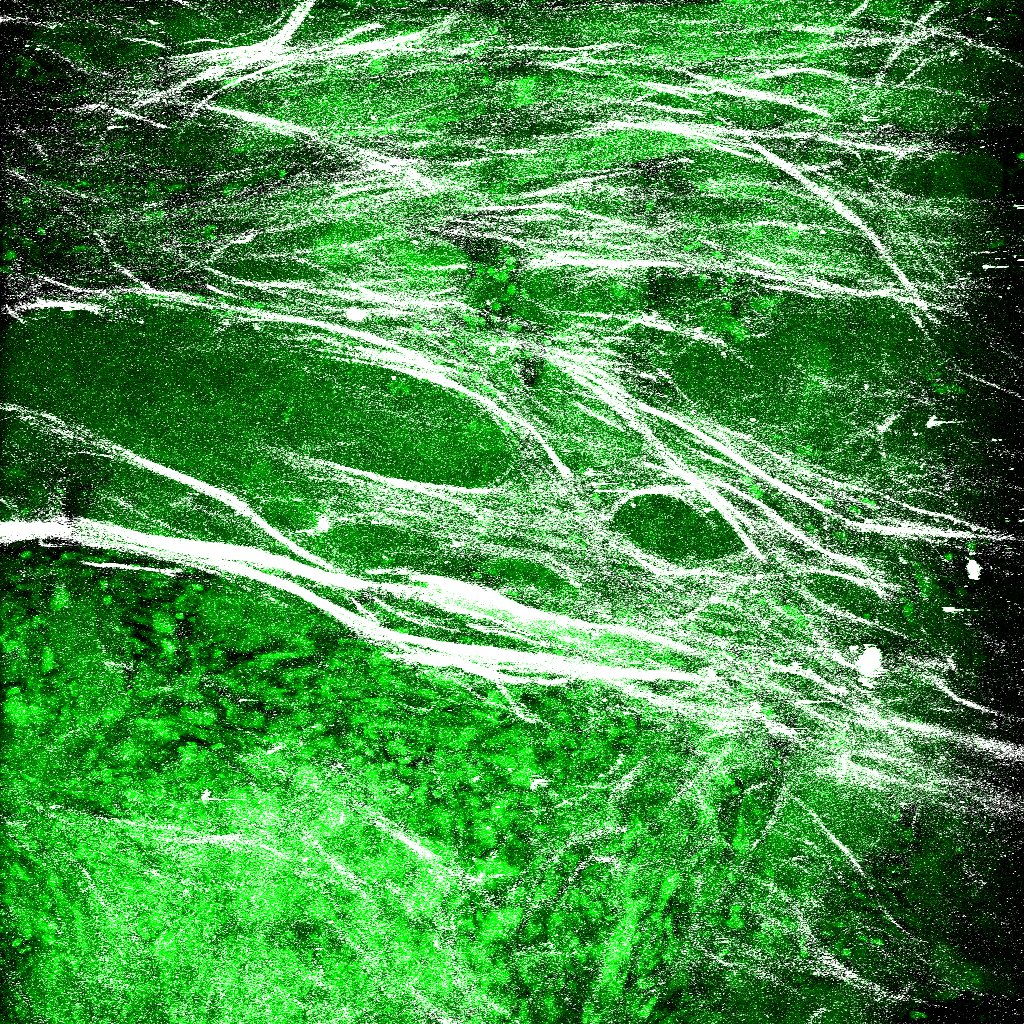

Supplement: Supplementary file 14 — Figure EV3 Source Data [file 44319_2026_751_MOESM14_ESM.zip › Raw_data_Figure EV3/Figure EV3C/Composite selected ko 2.jpg]

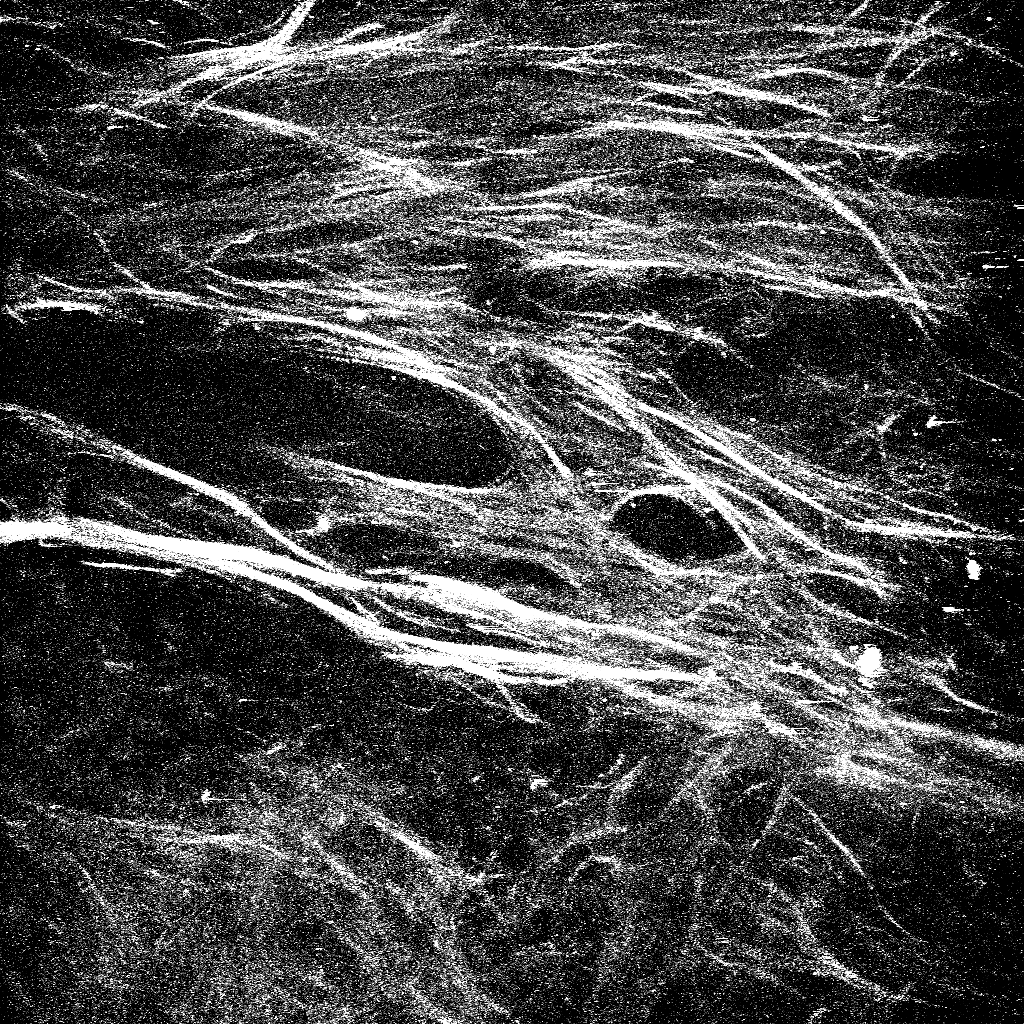

Supplement: Supplementary file 14 — Figure EV3 Source Data [file 44319_2026_751_MOESM14_ESM.zip › Raw_data_Figure EV3/Figure EV3C/shg ko selected 2.tif]

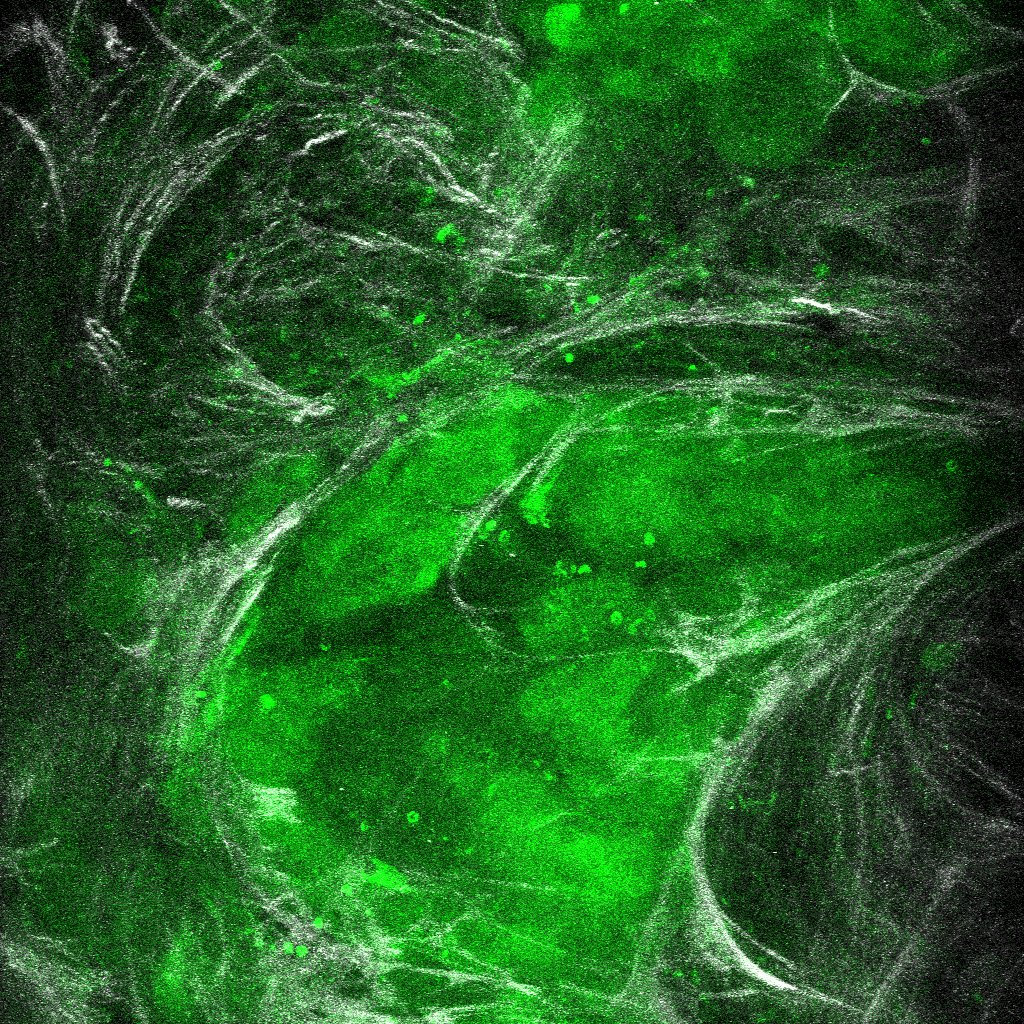

Supplement: Supplementary file 14 — Figure EV3 Source Data [file 44319_2026_751_MOESM14_ESM.zip › Raw_data_Figure EV3/Figure EV3C/WT Composite 2.jpg]

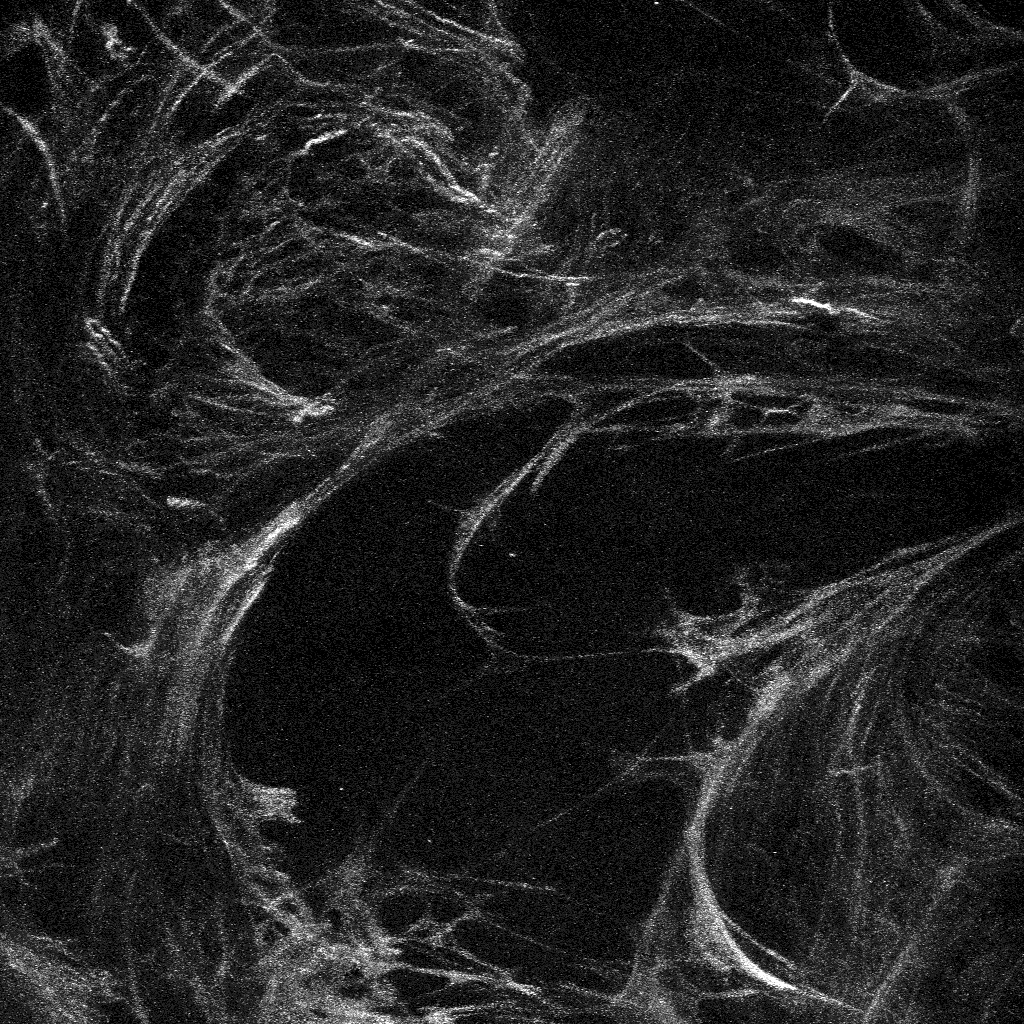

Supplement: Supplementary file 14 — Figure EV3 Source Data [file 44319_2026_751_MOESM14_ESM.zip › Raw_data_Figure EV3/Figure EV3C/WT shg2.tif]

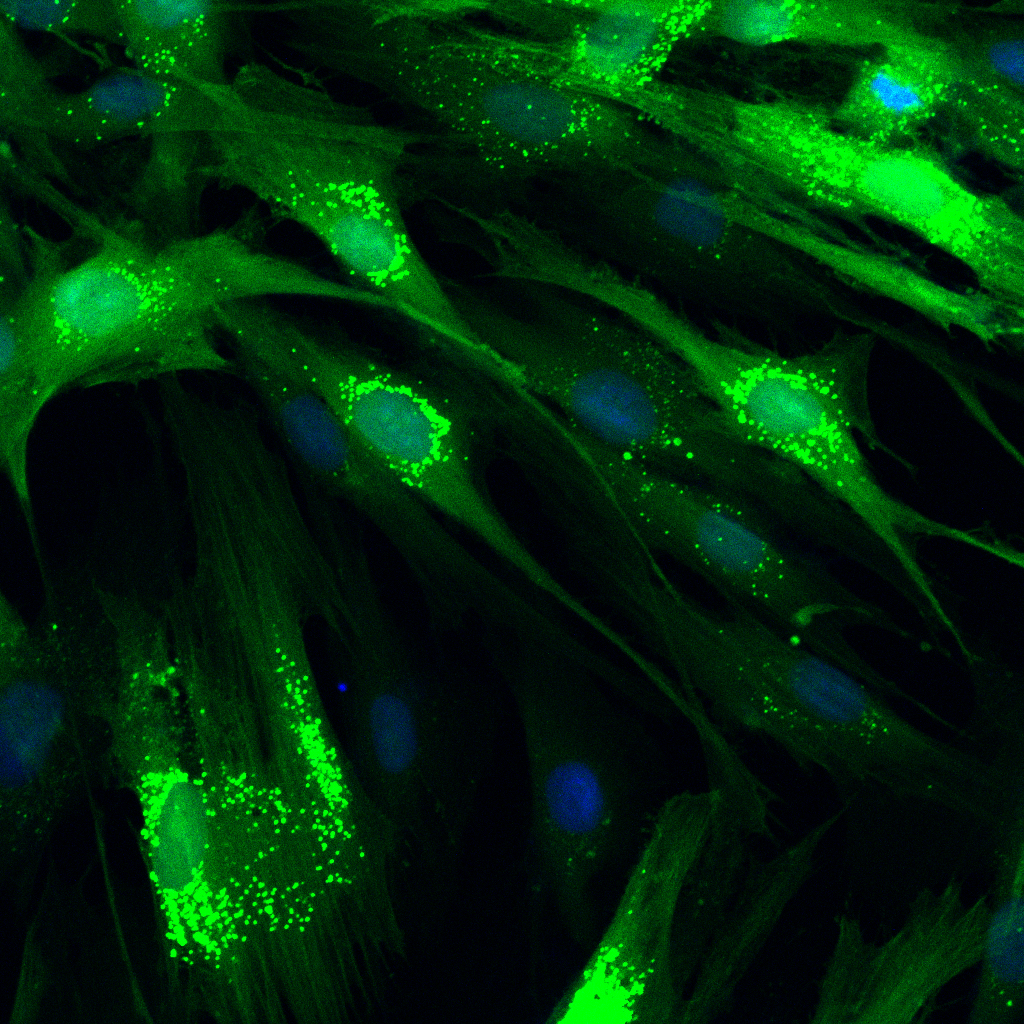

Supplement: Supplementary file 15 — Figure EV4 Source Data [file 44319_2026_751_MOESM15_ESM.zip › Raw_data_Figure EV4/Figure EV4A/NFshctrl PTRF green-nuclei.tif]

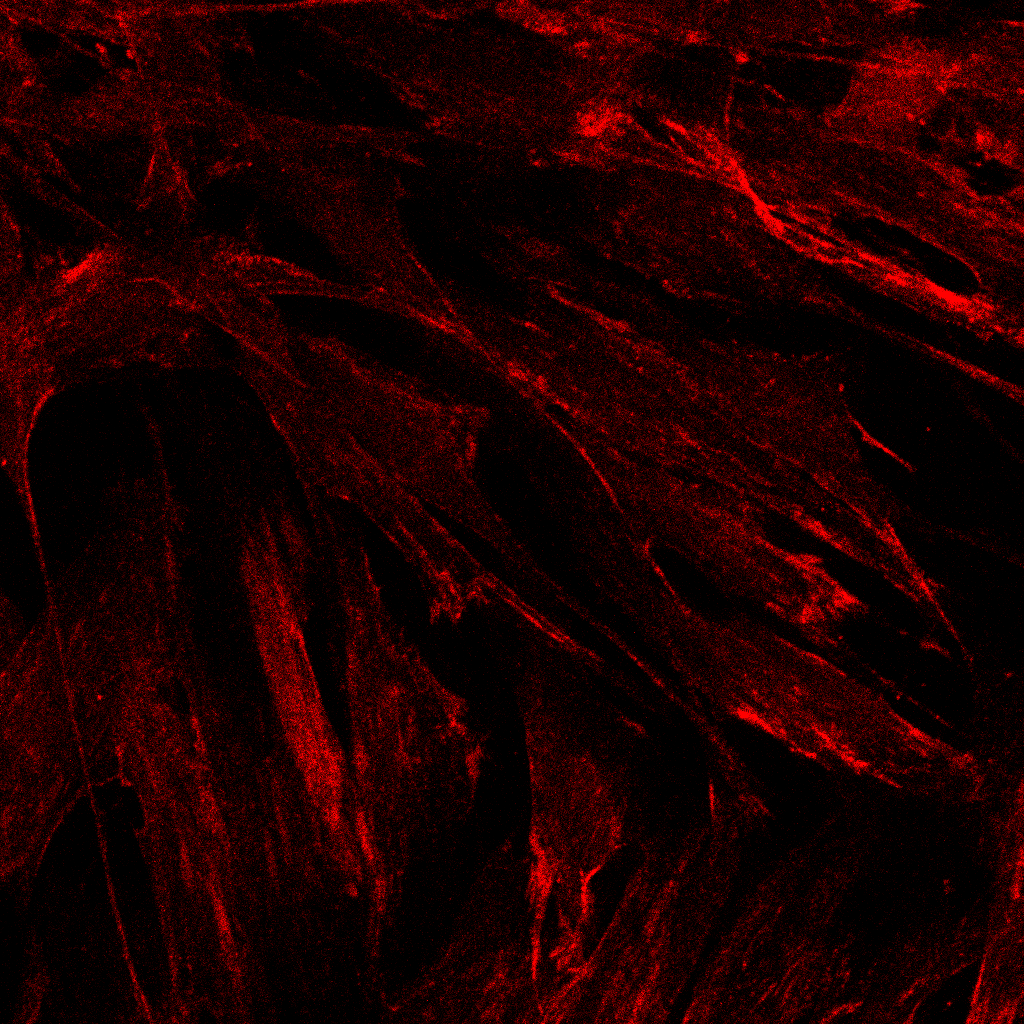

Supplement: Supplementary file 15 — Figure EV4 Source Data [file 44319_2026_751_MOESM15_ESM.zip › Raw_data_Figure EV4/Figure EV4A/NFshctrl PTRFred.tif]

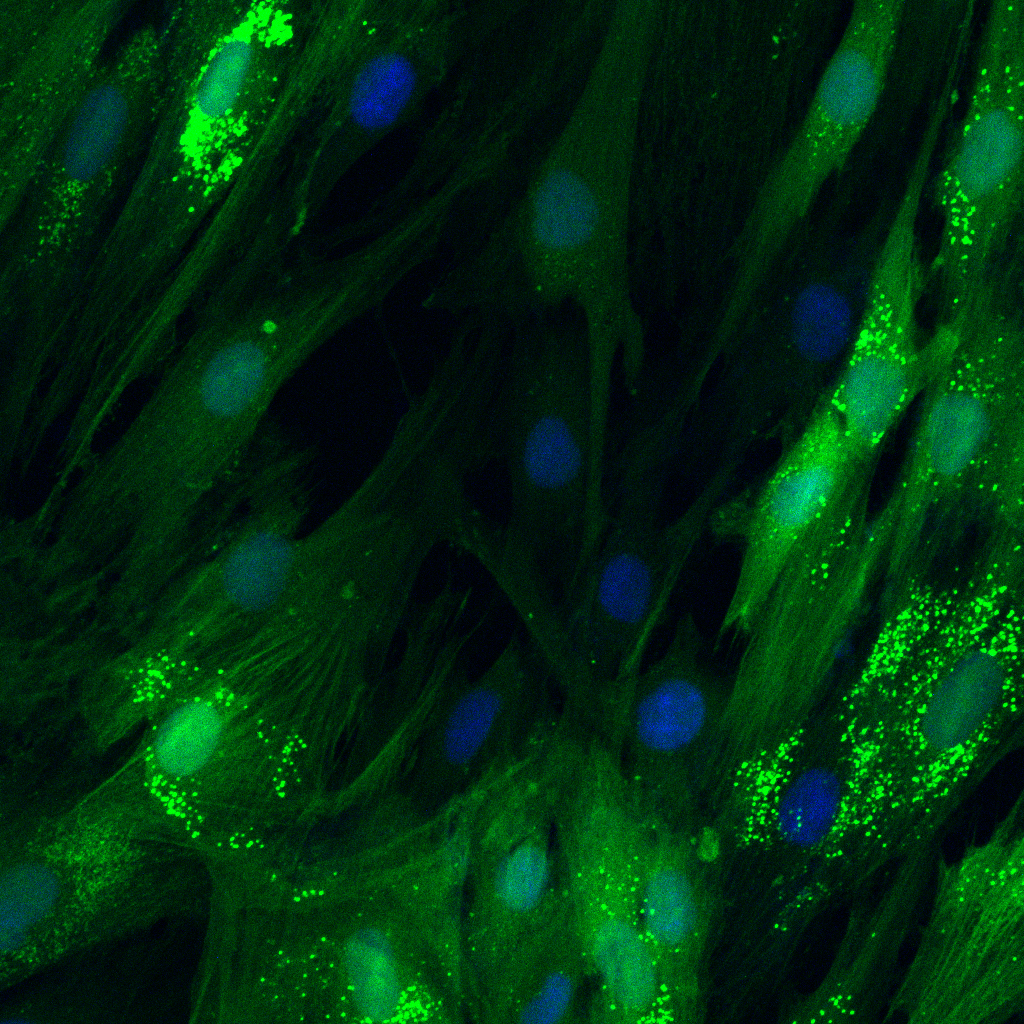

Supplement: Supplementary file 15 — Figure EV4 Source Data [file 44319_2026_751_MOESM15_ESM.zip › Raw_data_Figure EV4/Figure EV4A/NFshGq PTRF green-nuclei.tif]

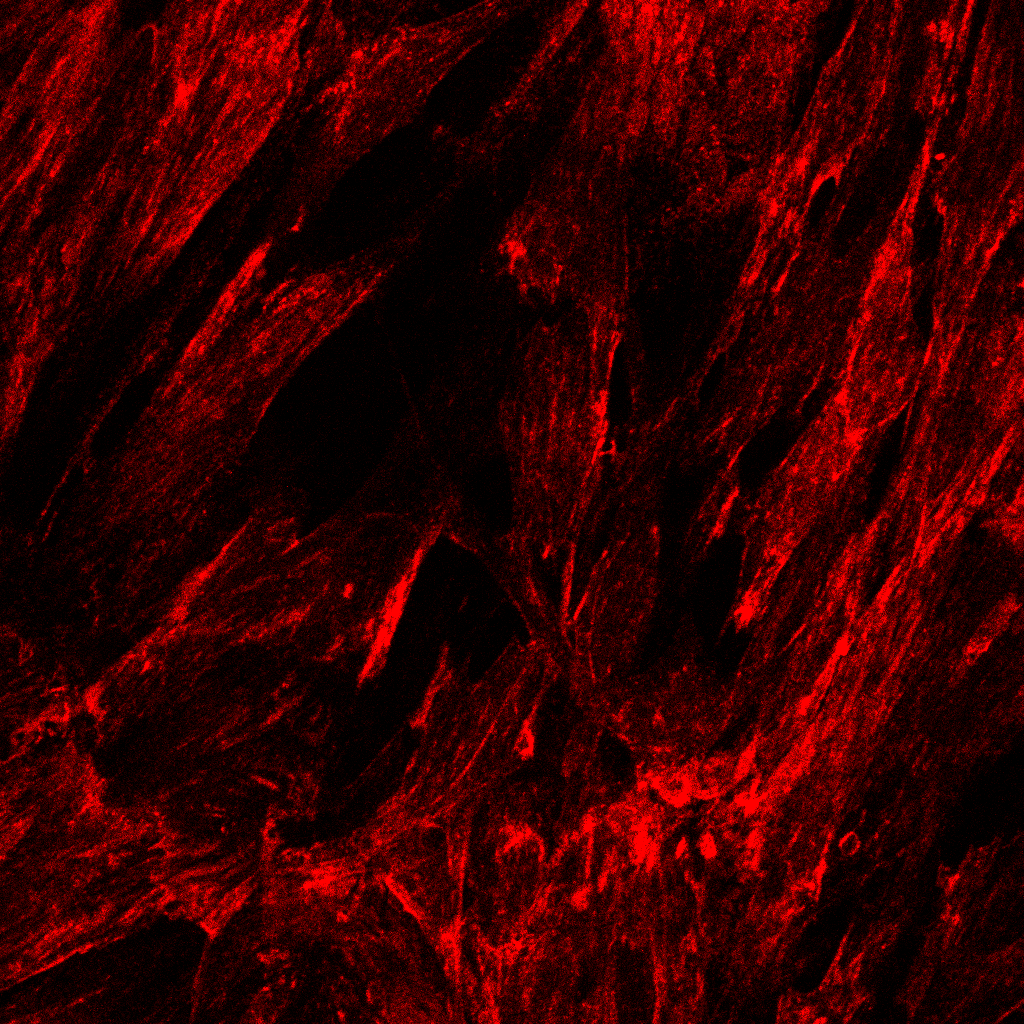

Supplement: Supplementary file 15 — Figure EV4 Source Data [file 44319_2026_751_MOESM15_ESM.zip › Raw_data_Figure EV4/Figure EV4A/NFshGq PTRFred.tif]

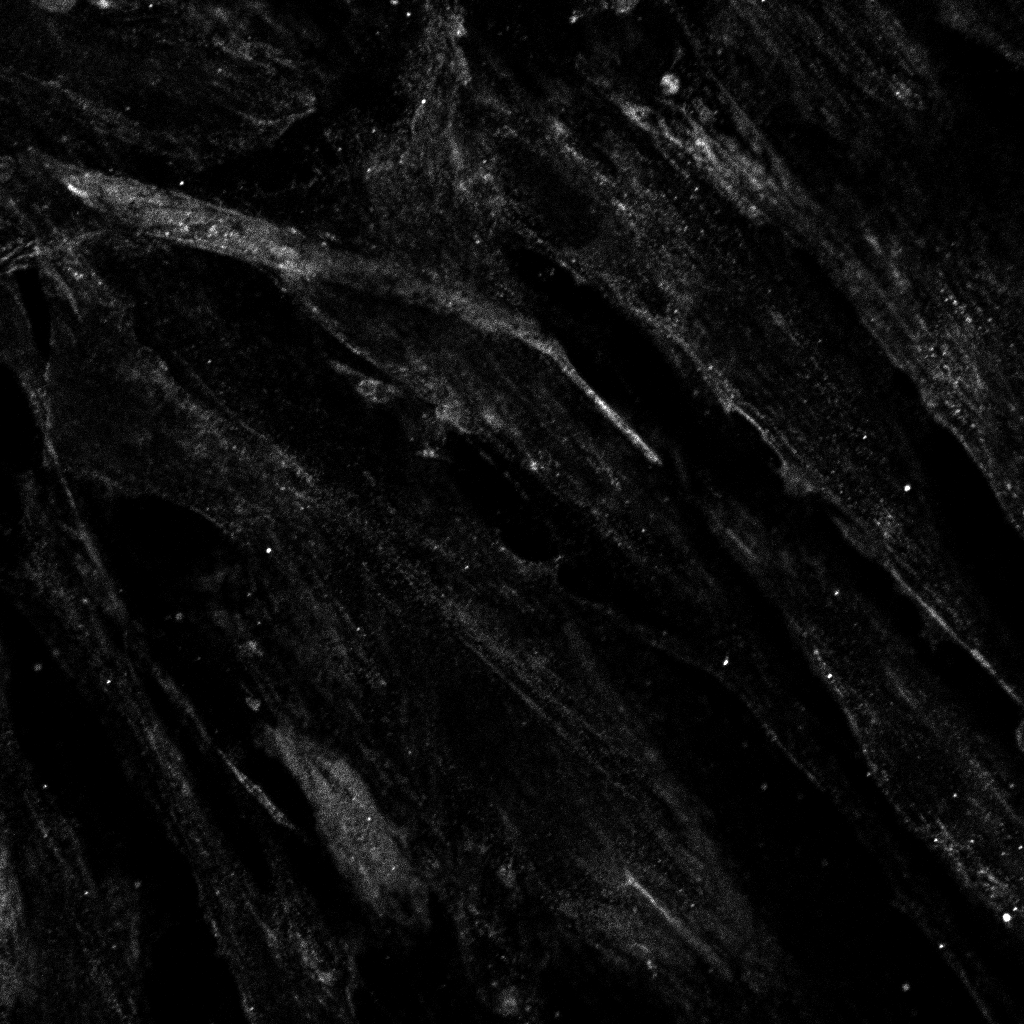

Supplement: Supplementary file 15 — Figure EV4 Source Data [file 44319_2026_751_MOESM15_ESM.zip › Raw_data_Figure EV4/Figure EV4B/NF shctrl cav1 gray.tif]

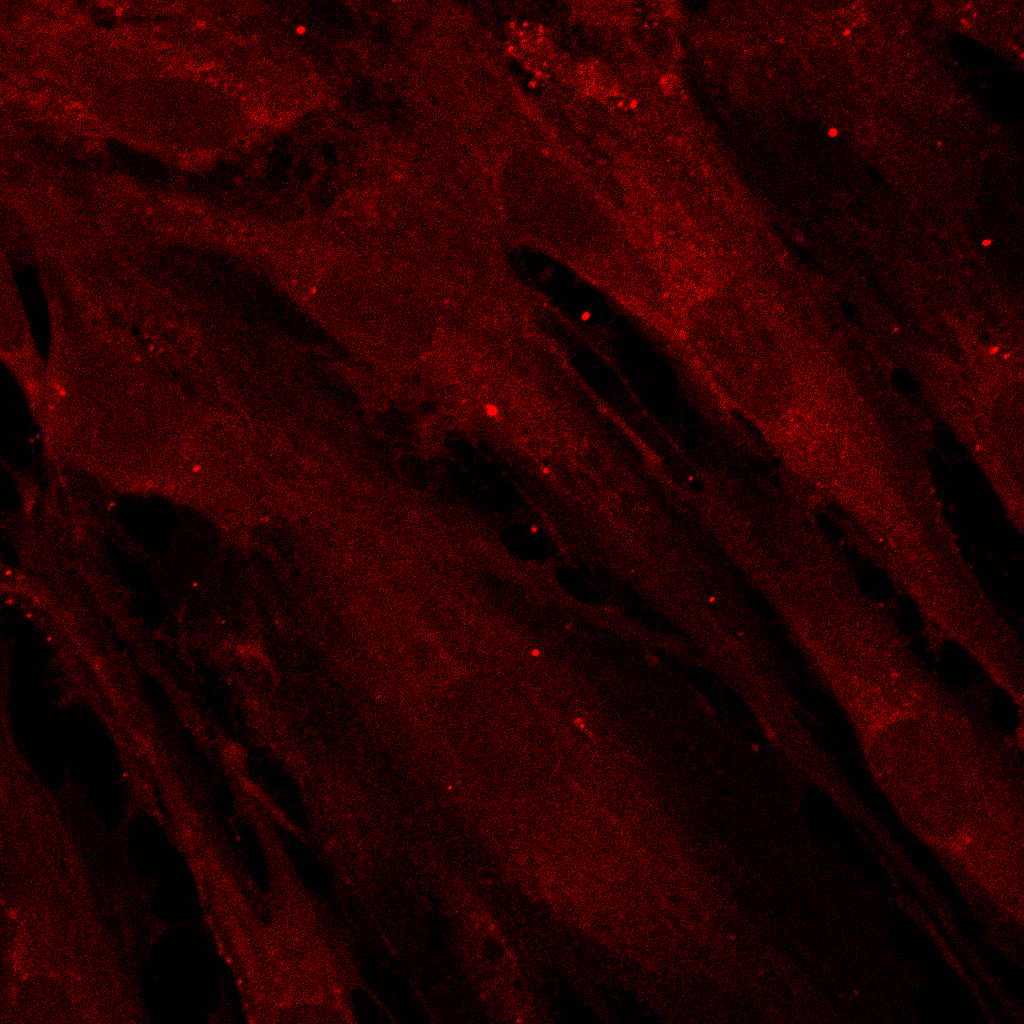

Supplement: Supplementary file 15 — Figure EV4 Source Data [file 44319_2026_751_MOESM15_ESM.zip › Raw_data_Figure EV4/Figure EV4B/NF shctrl chtx red.tif]

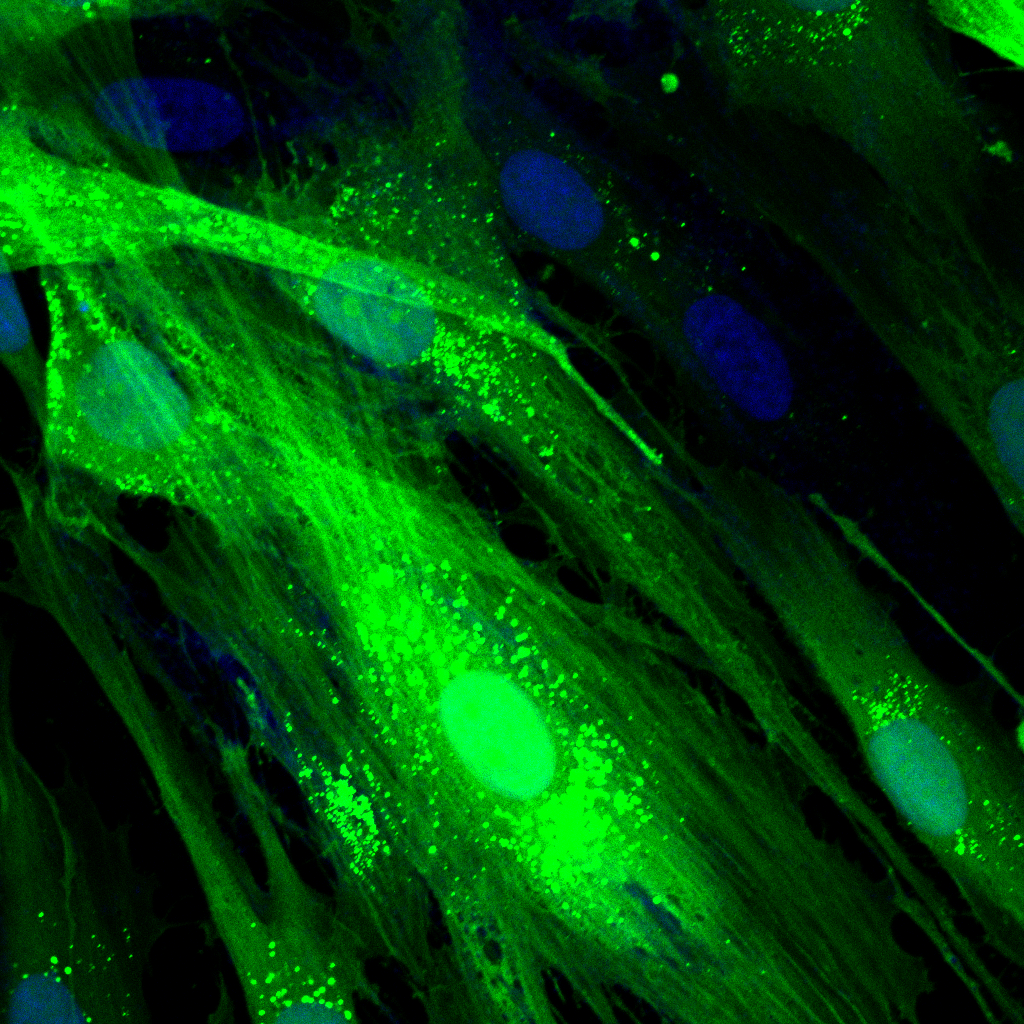

Supplement: Supplementary file 15 — Figure EV4 Source Data [file 44319_2026_751_MOESM15_ESM.zip › Raw_data_Figure EV4/Figure EV4B/NF shctrl green-blue.tif]

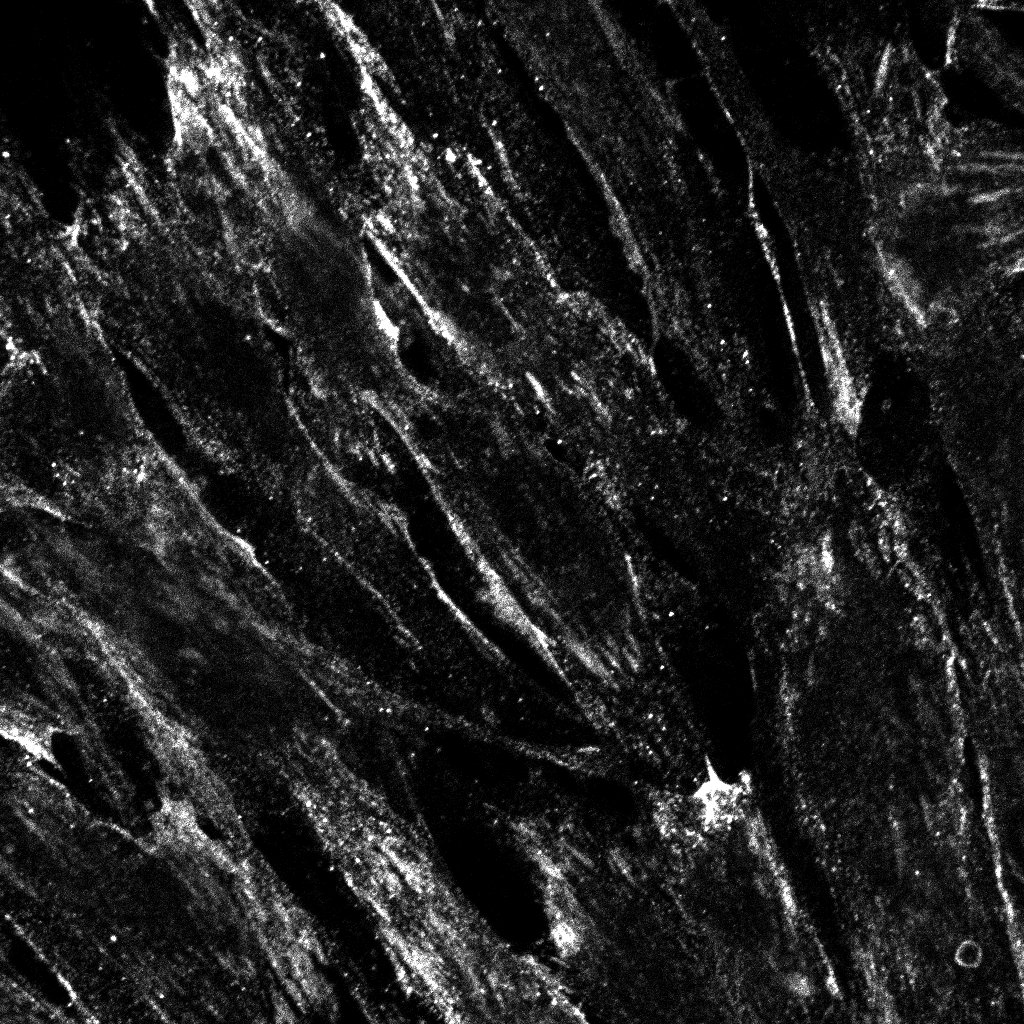

Supplement: Supplementary file 15 — Figure EV4 Source Data [file 44319_2026_751_MOESM15_ESM.zip › Raw_data_Figure EV4/Figure EV4B/NF shGq cav1 gray.jpg]

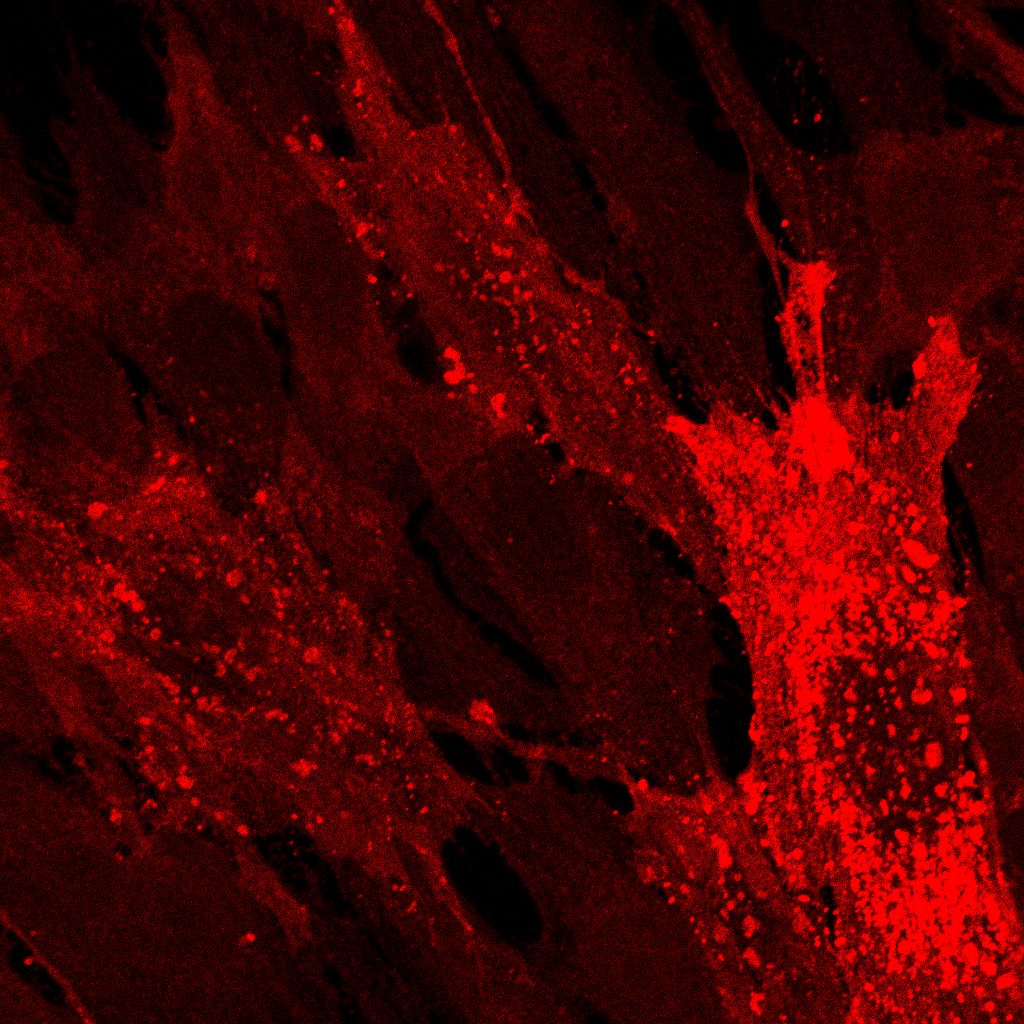

Supplement: Supplementary file 15 — Figure EV4 Source Data [file 44319_2026_751_MOESM15_ESM.zip › Raw_data_Figure EV4/Figure EV4B/NF shGq chtx red.tif]

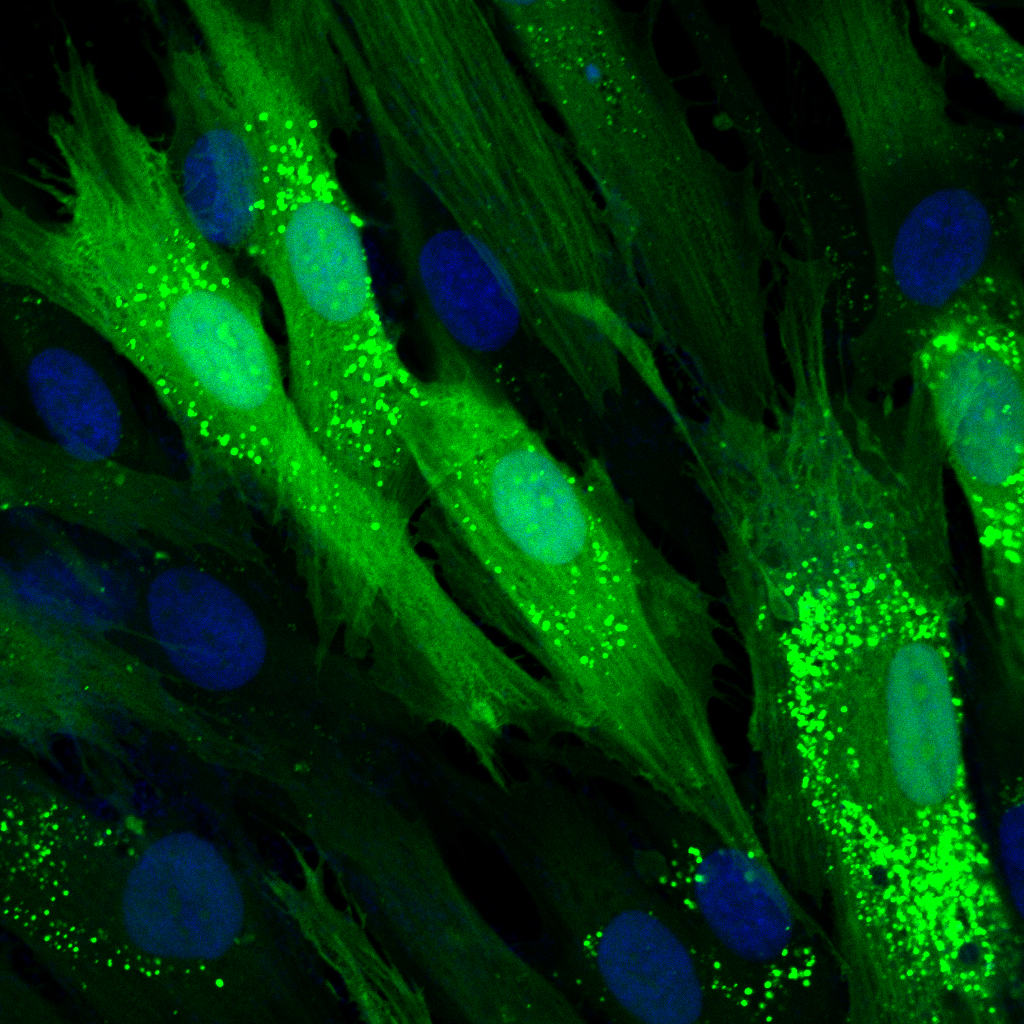

Supplement: Supplementary file 15 — Figure EV4 Source Data [file 44319_2026_751_MOESM15_ESM.zip › Raw_data_Figure EV4/Figure EV4B/NF shGq green-blue.tif]

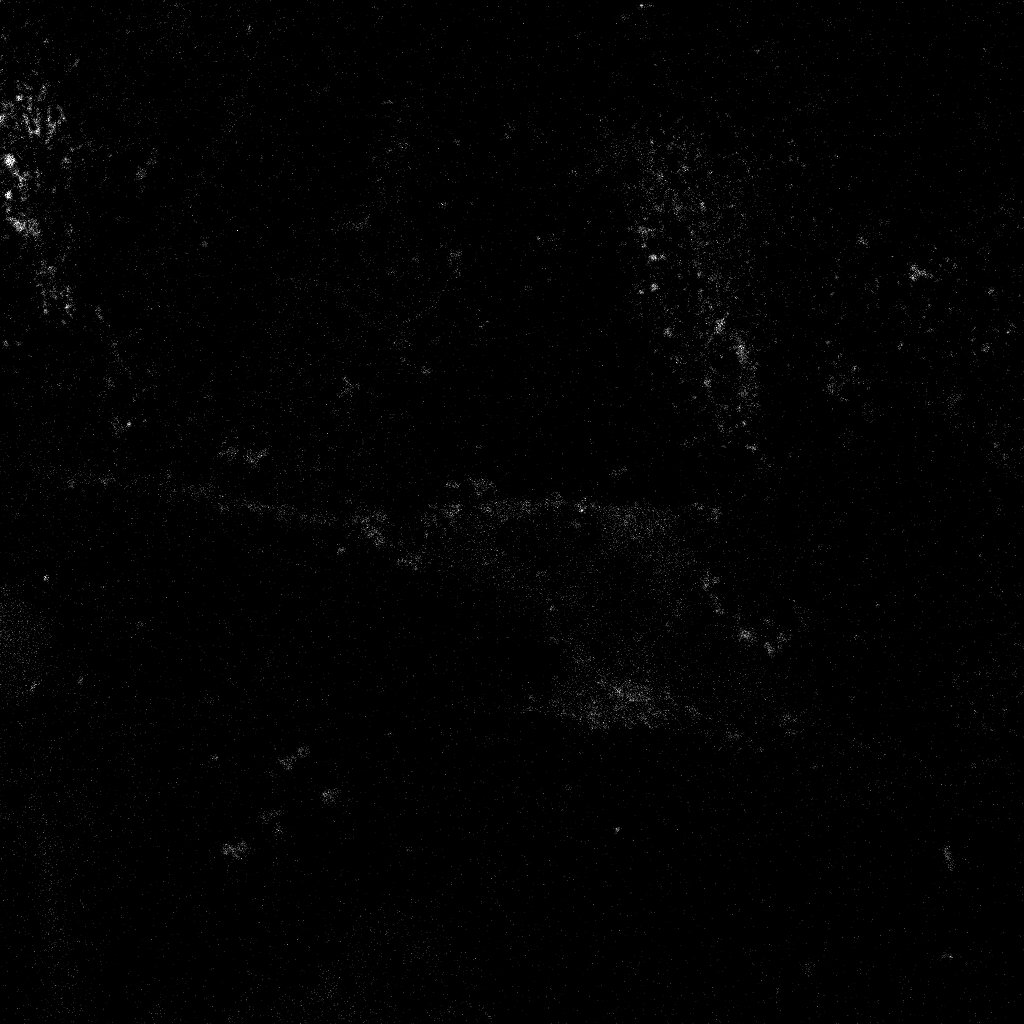

Supplement: Supplementary file 15 — Figure EV4 Source Data [file 44319_2026_751_MOESM15_ESM.zip › Raw_data_Figure EV4/Figure EV4C/NFshcontrol cer 2.jpg]

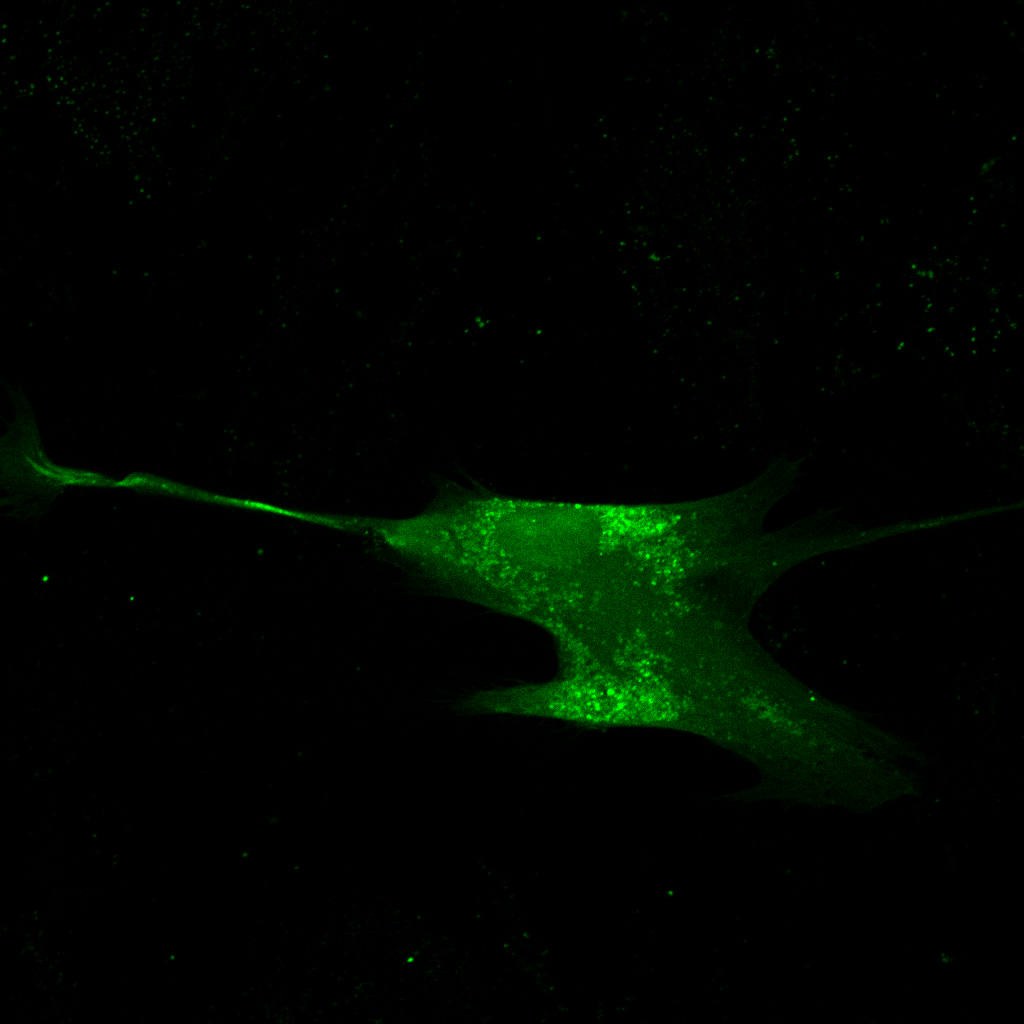

Supplement: Supplementary file 15 — Figure EV4 Source Data [file 44319_2026_751_MOESM15_ESM.zip › Raw_data_Figure EV4/Figure EV4C/NFshcontrol GFP 2.tif]

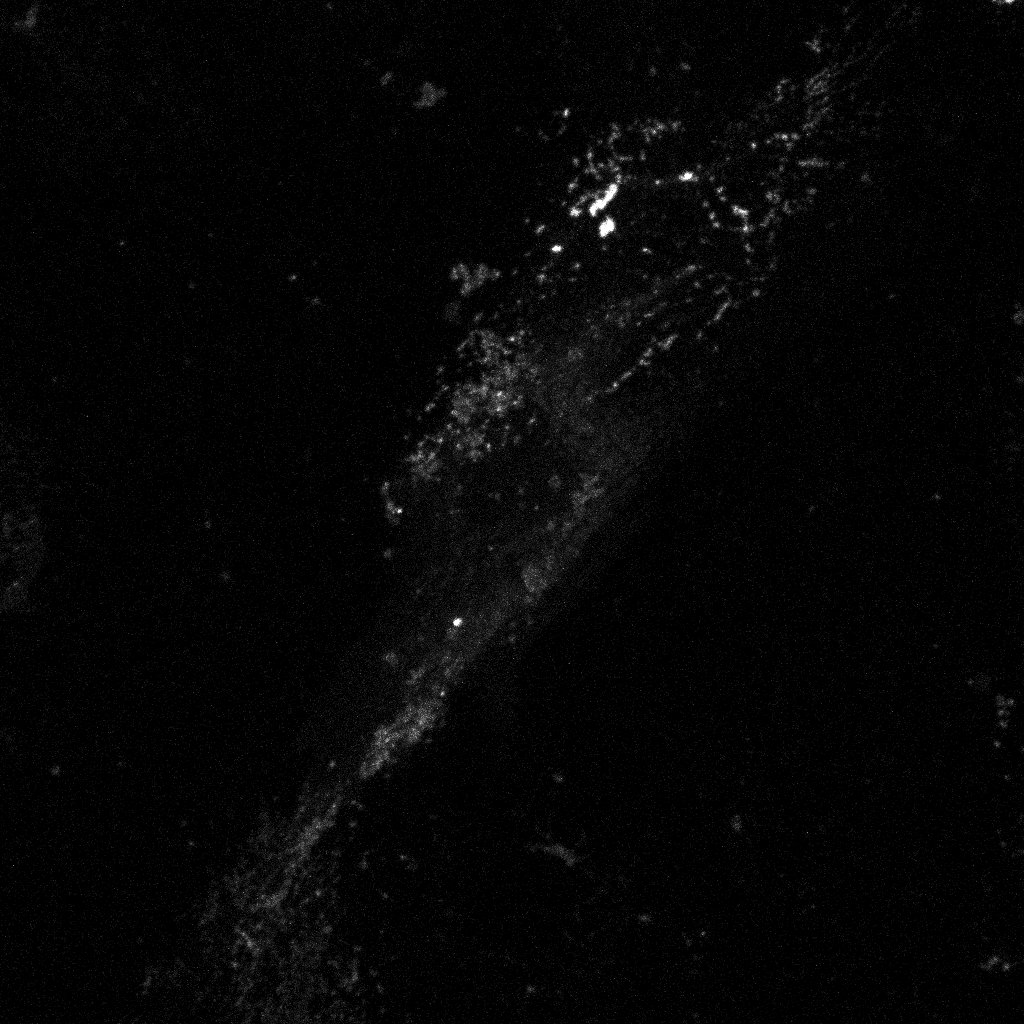

Supplement: Supplementary file 15 — Figure EV4 Source Data [file 44319_2026_751_MOESM15_ESM.zip › Raw_data_Figure EV4/Figure EV4C/NFshGq cer.jpg]

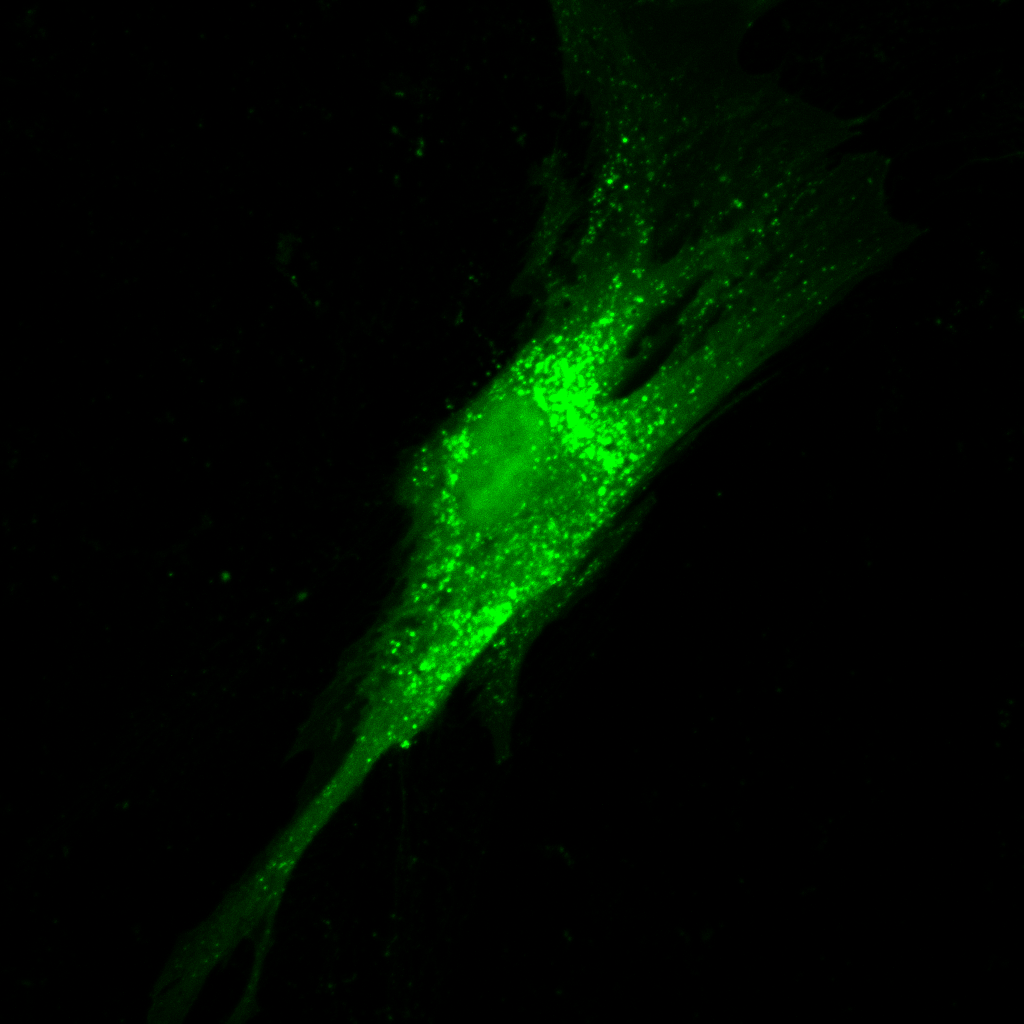

Supplement: Supplementary file 15 — Figure EV4 Source Data [file 44319_2026_751_MOESM15_ESM.zip › Raw_data_Figure EV4/Figure EV4C/NFshGq GFP.tif]

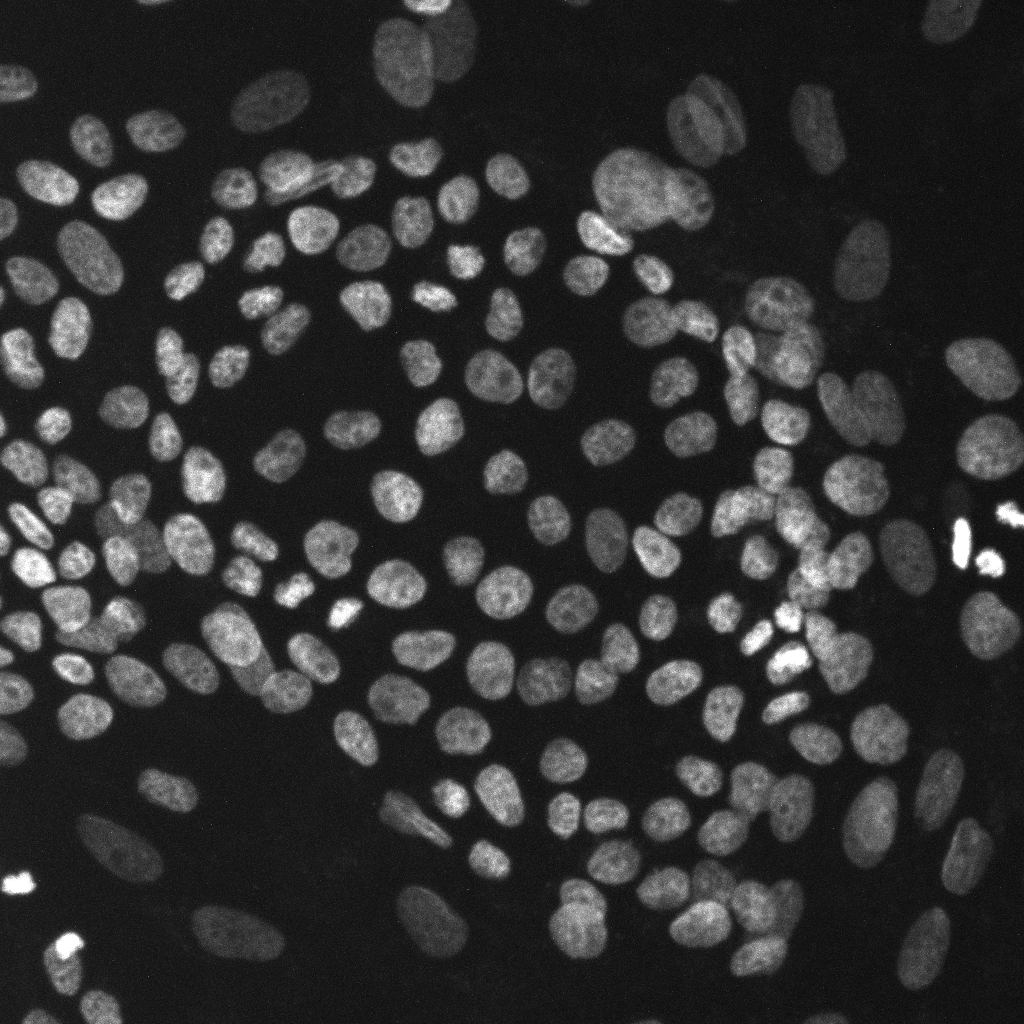

Supplement: Supplementary file 15 — Figure EV4 Source Data [file 44319_2026_751_MOESM15_ESM.zip › Raw_data_Figure EV4/Figure EV4D/MAX_Cal27 sin marcar + NFshctrl green colI 647 3.tif]

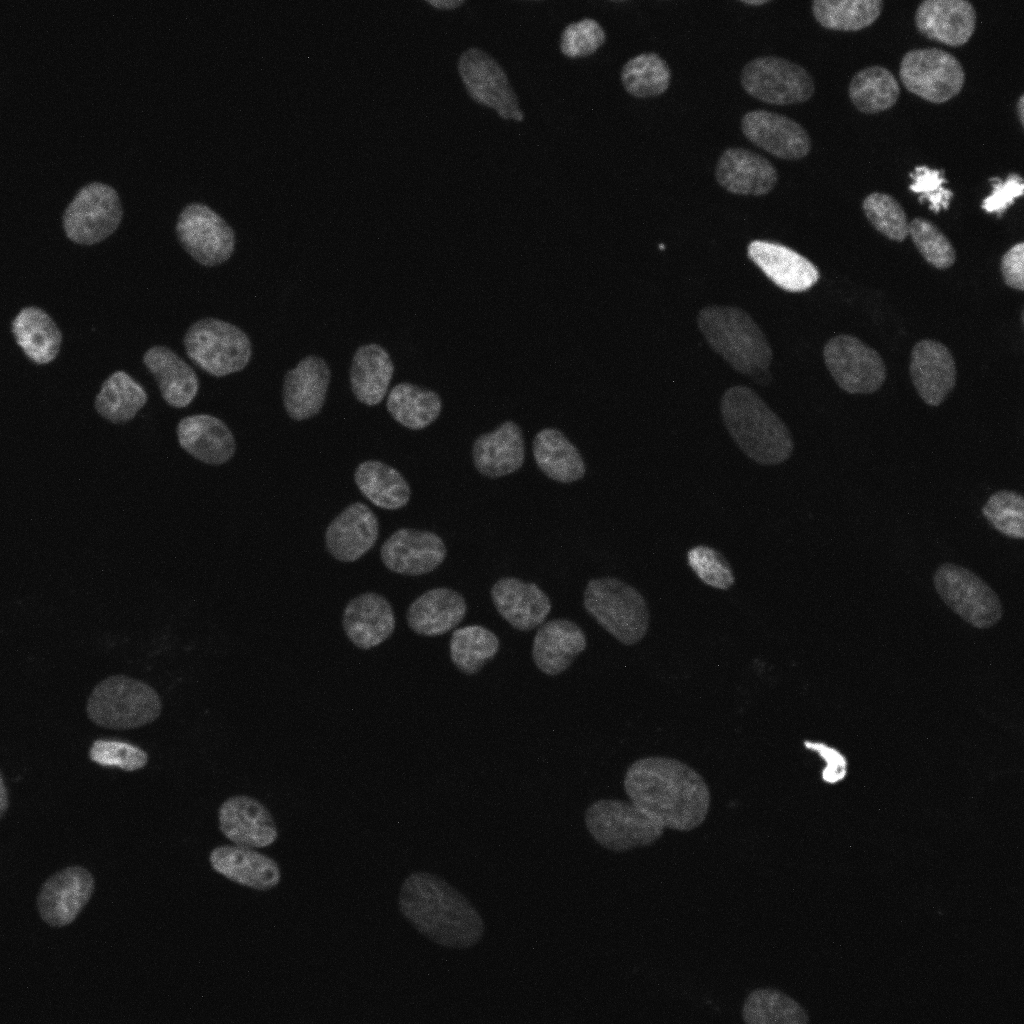

Supplement: Supplementary file 15 — Figure EV4 Source Data [file 44319_2026_751_MOESM15_ESM.zip › Raw_data_Figure EV4/Figure EV4D/MAX_Cal27 sin marcar + NFshGq green colI 647.tif]

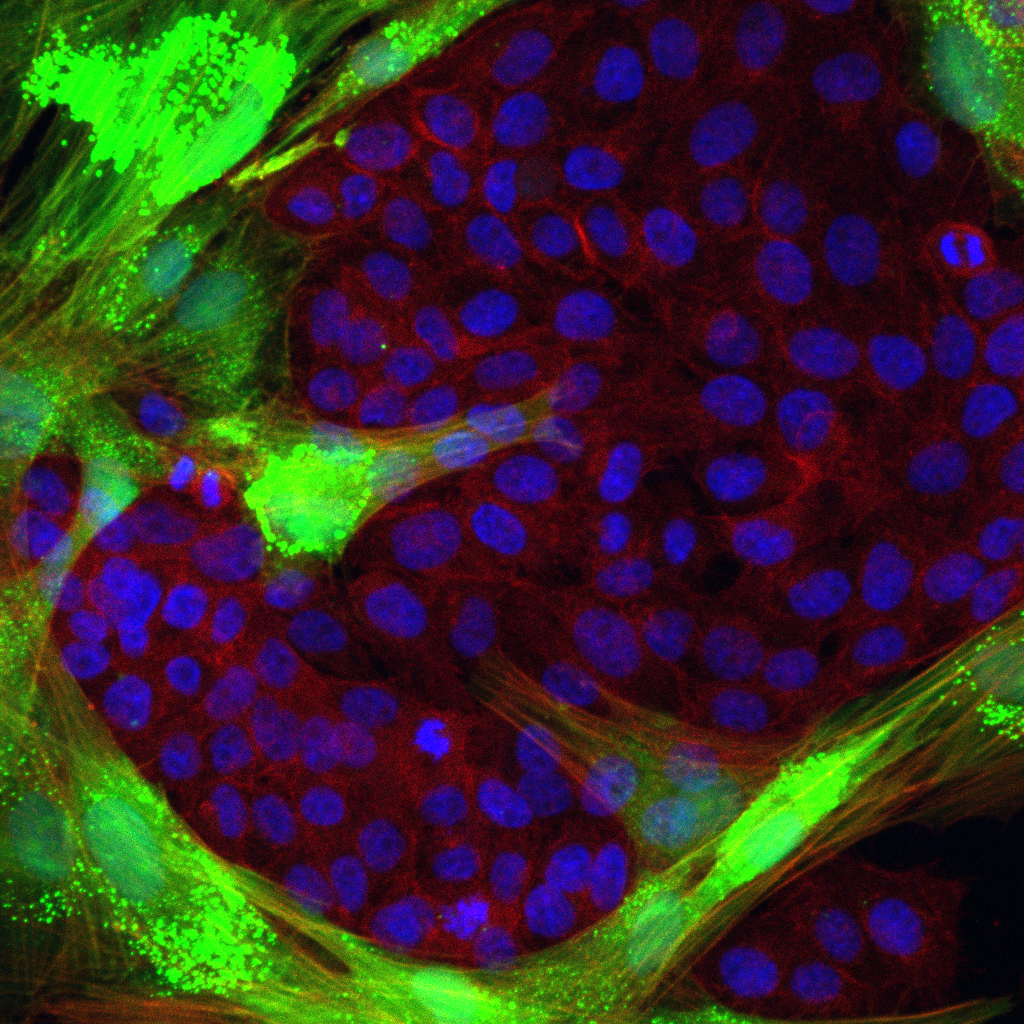

Supplement: Supplementary file 15 — Figure EV4 Source Data [file 44319_2026_751_MOESM15_ESM.zip › Raw_data_Figure EV4/Figure EV4D/NFshctrl +cal27 de FN blue-green-red.tif]

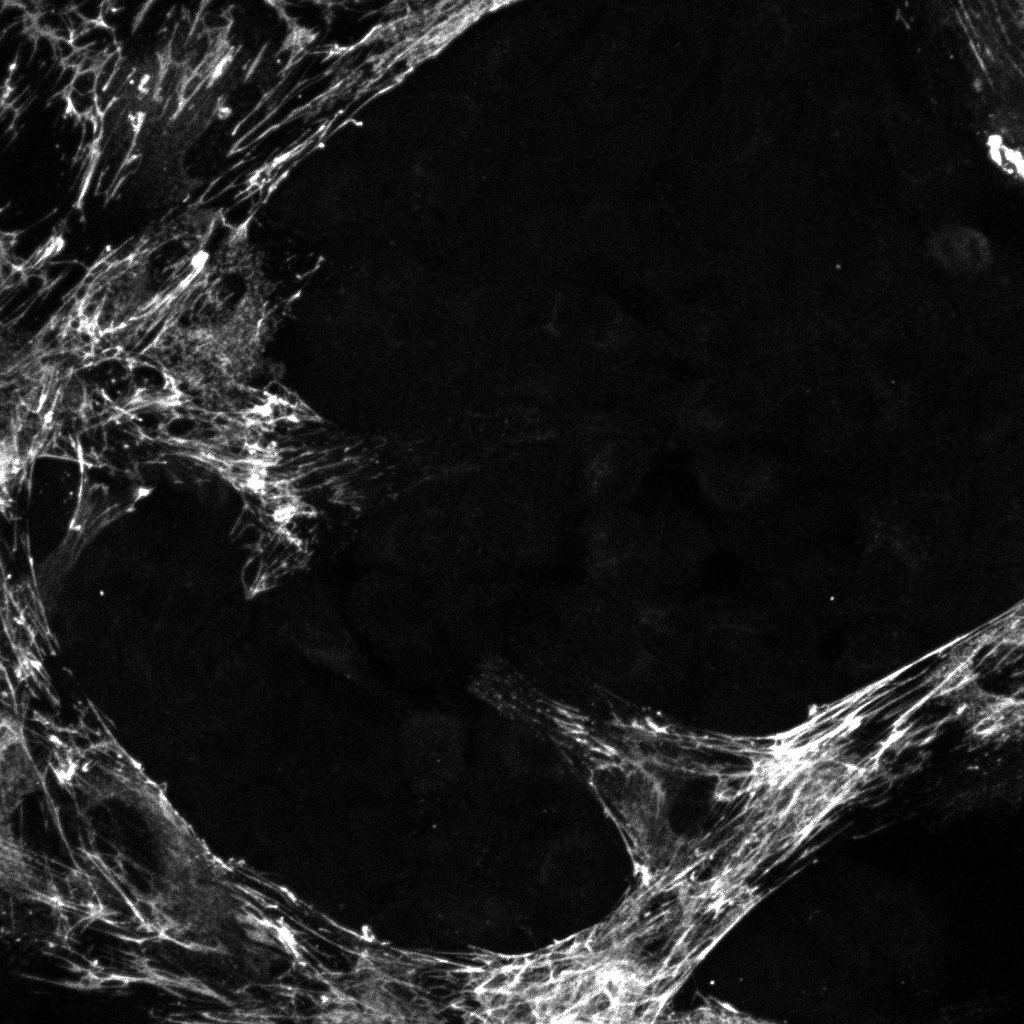

Supplement: Supplementary file 15 — Figure EV4 Source Data [file 44319_2026_751_MOESM15_ESM.zip › Raw_data_Figure EV4/Figure EV4D/NFshctrl +cal27 de FN gray.jpg]

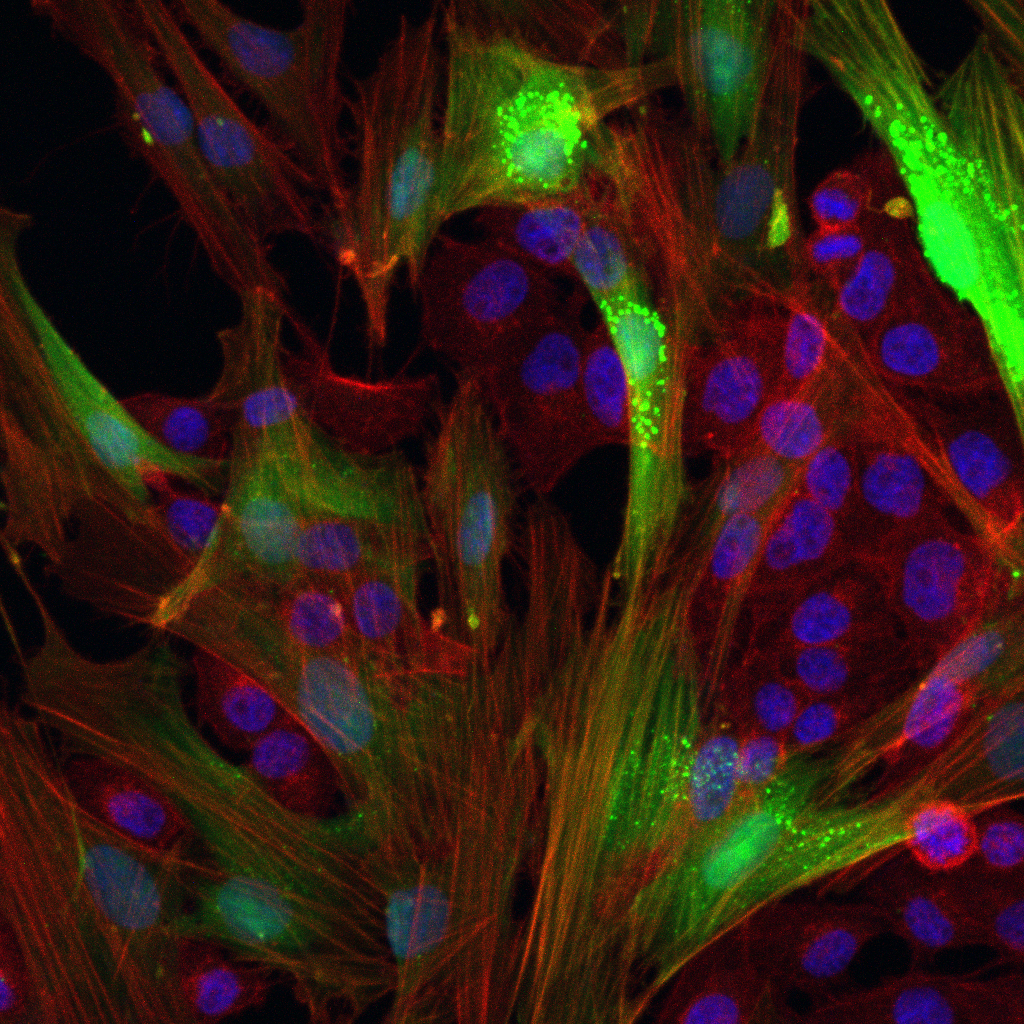

Supplement: Supplementary file 15 — Figure EV4 Source Data [file 44319_2026_751_MOESM15_ESM.zip › Raw_data_Figure EV4/Figure EV4D/NFshGq+cal27 de FN blue-green-red.tif]

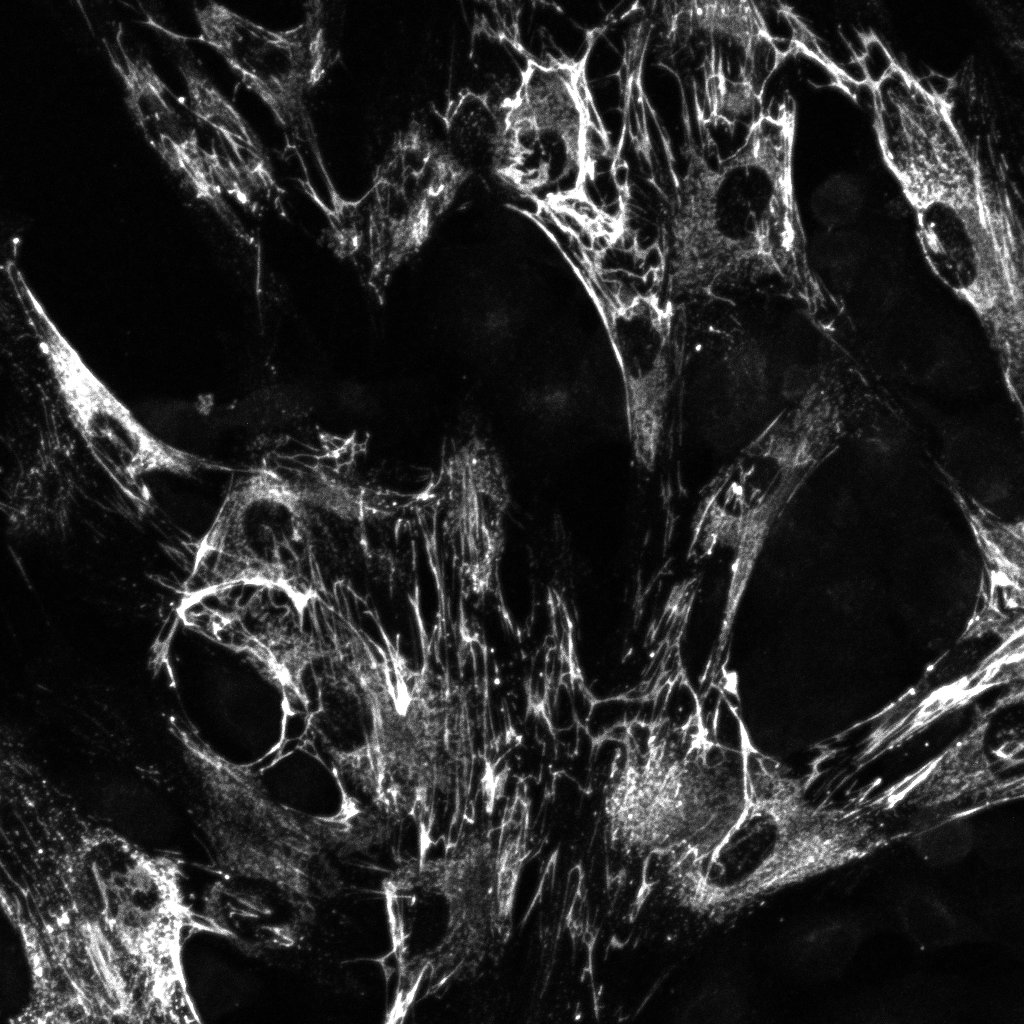

Supplement: Supplementary file 15 — Figure EV4 Source Data [file 44319_2026_751_MOESM15_ESM.zip › Raw_data_Figure EV4/Figure EV4D/NFshGq+cal27 de FN gray.jpg]

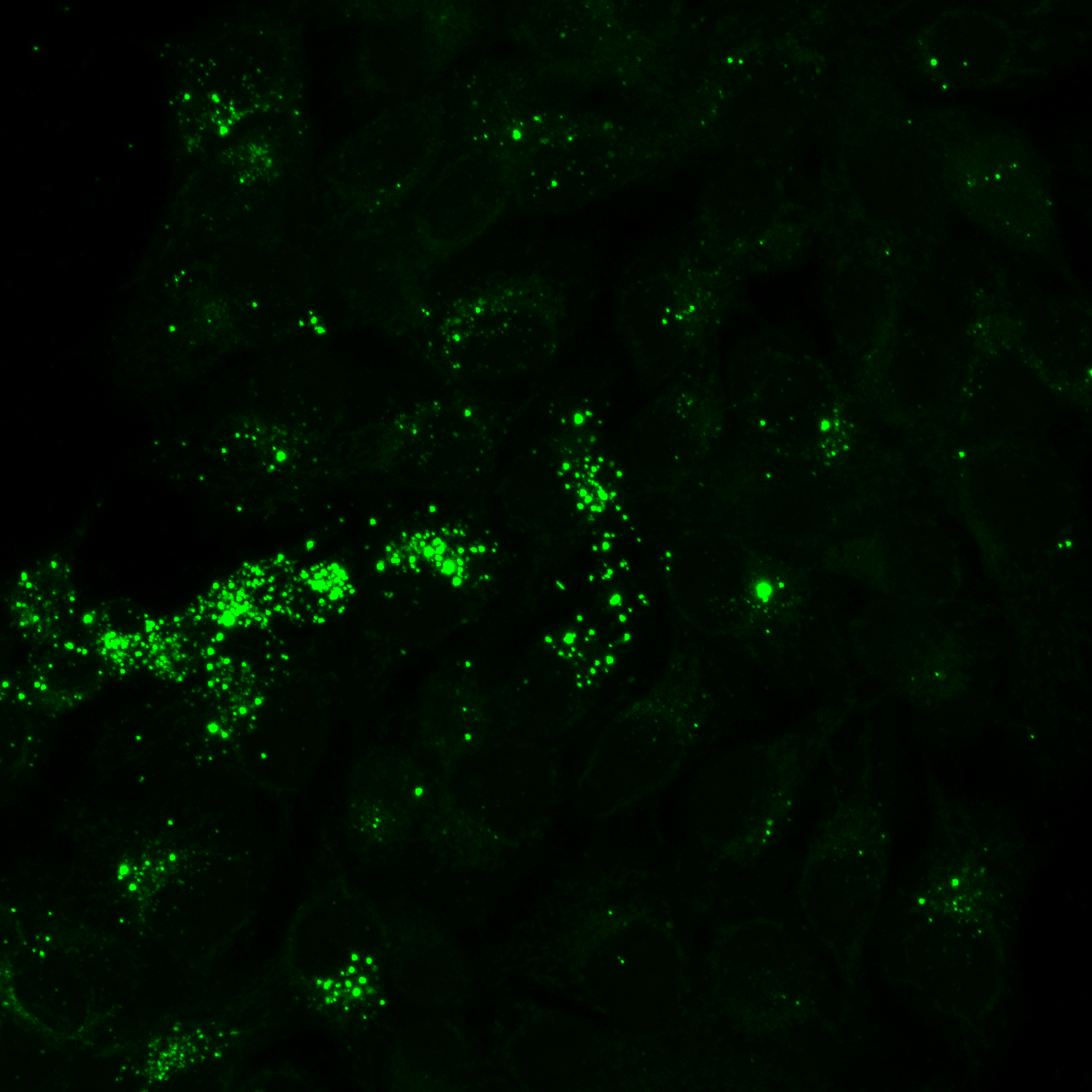

Supplement: Supplementary file 15 — Figure EV4 Source Data [file 44319_2026_751_MOESM15_ESM.zip › Raw_data_Figure EV4/Figure EV4E/C2-MAX_Cal27 + Exos NF shCtrl Exos GFP.tif]

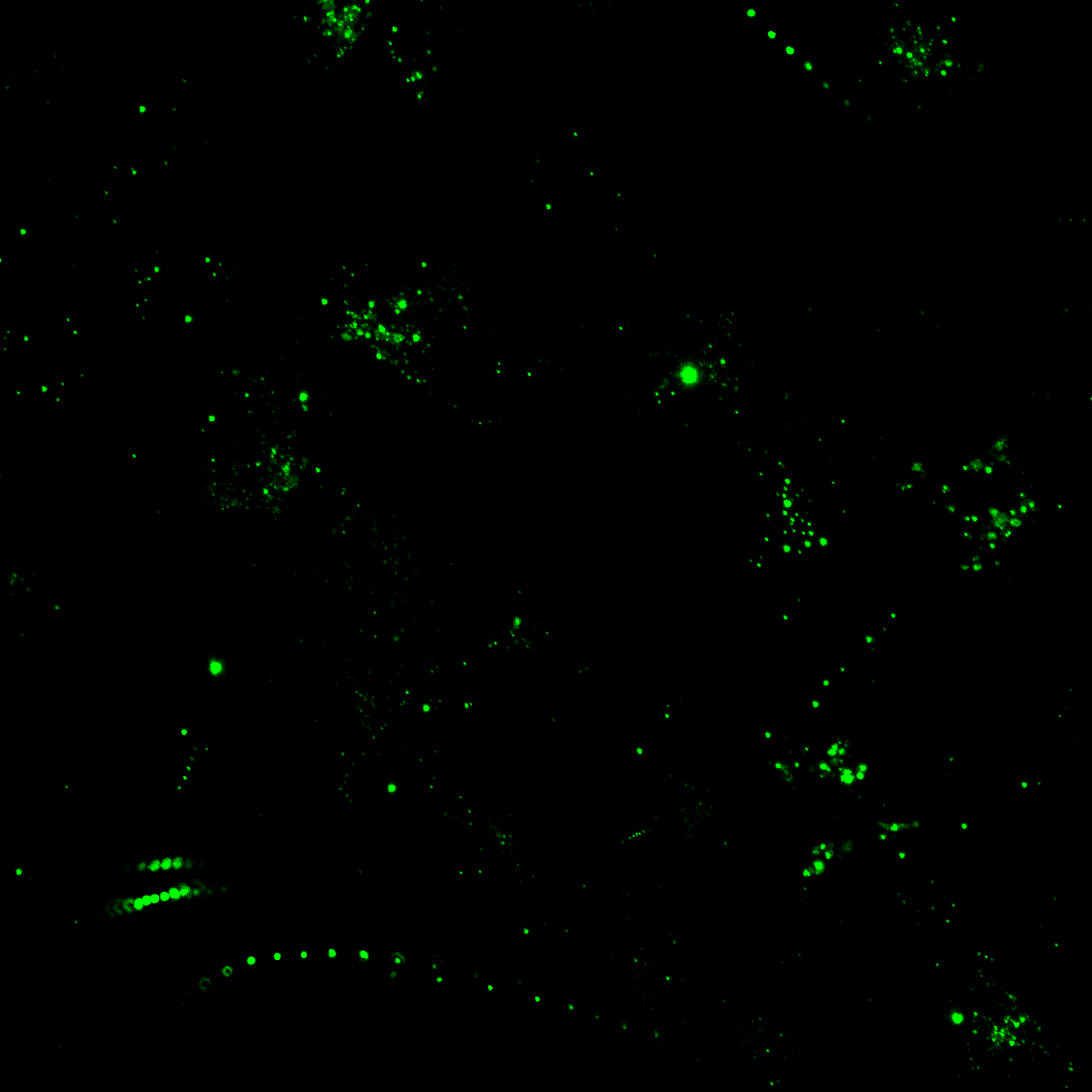

Supplement: Supplementary file 15 — Figure EV4 Source Data [file 44319_2026_751_MOESM15_ESM.zip › Raw_data_Figure EV4/Figure EV4E/C2-MAX_Cal27 + Exos NF shGq Exos GFP.tif]

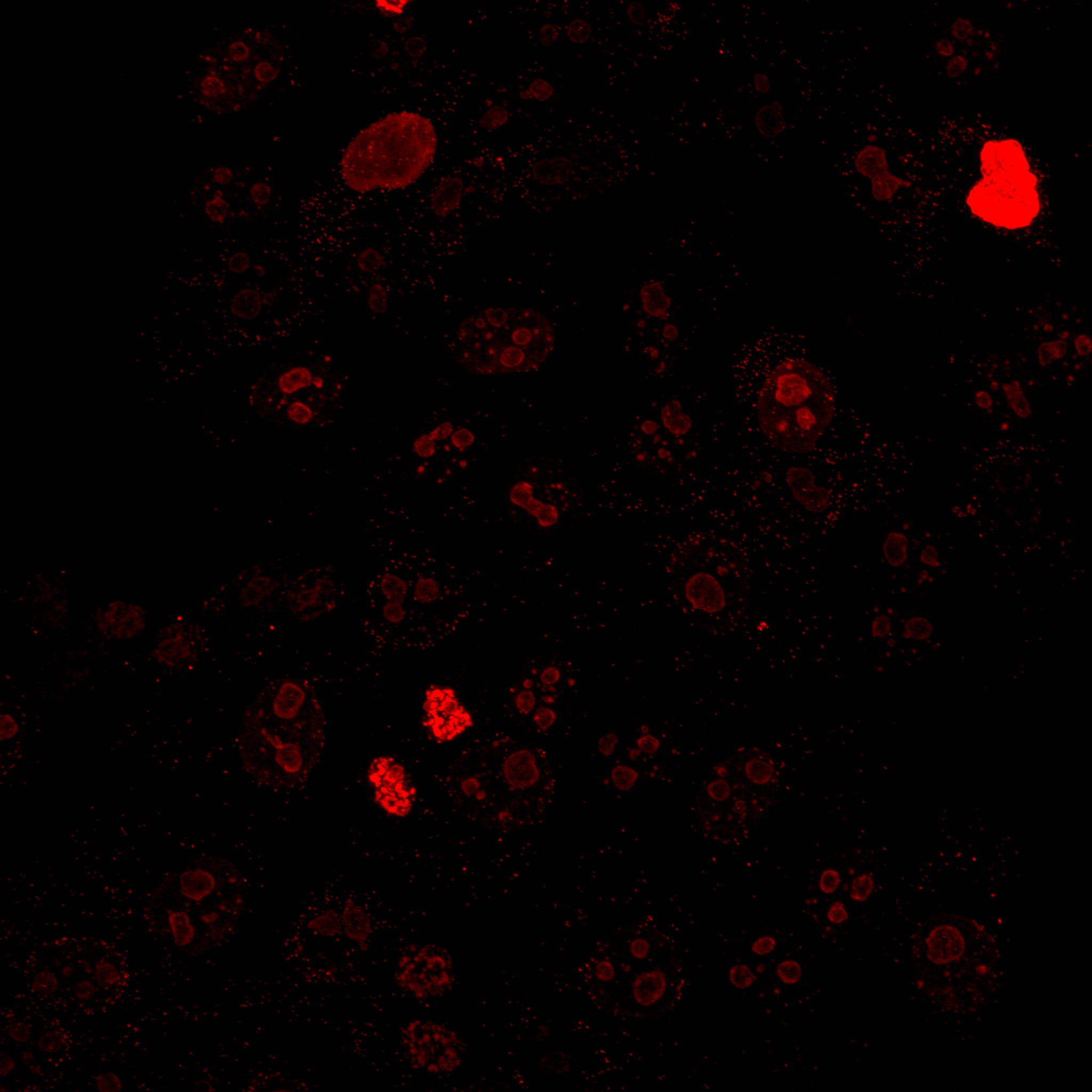

Supplement: Supplementary file 15 — Figure EV4 Source Data [file 44319_2026_751_MOESM15_ESM.zip › Raw_data_Figure EV4/Figure EV4E/C3-MAX_Cal27 + Exos NF shCtrl Ki67 555.tif]

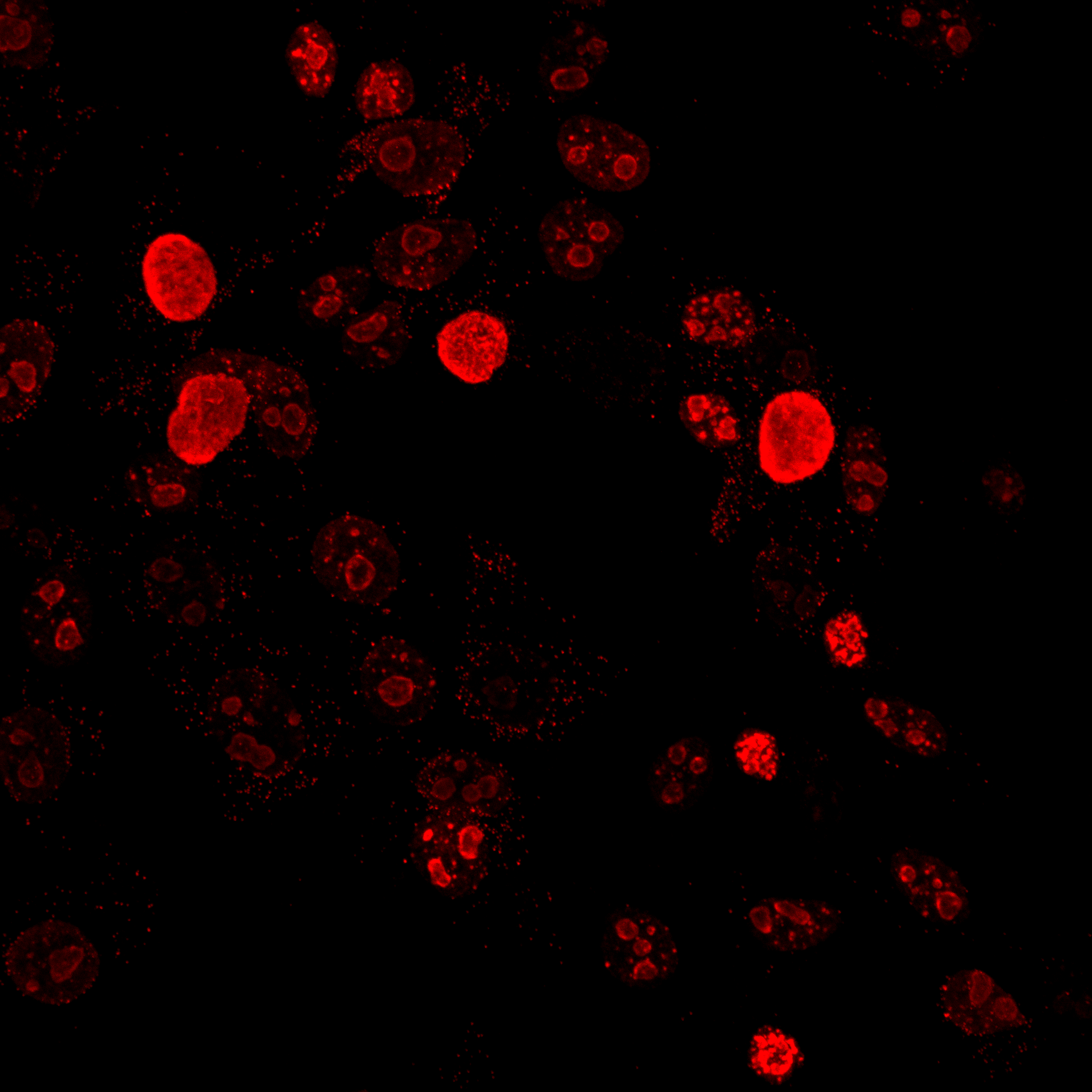

Supplement: Supplementary file 15 — Figure EV4 Source Data [file 44319_2026_751_MOESM15_ESM.zip › Raw_data_Figure EV4/Figure EV4E/C3-MAX_Cal27 + Exos NF shGq Ki67 555,.tif]

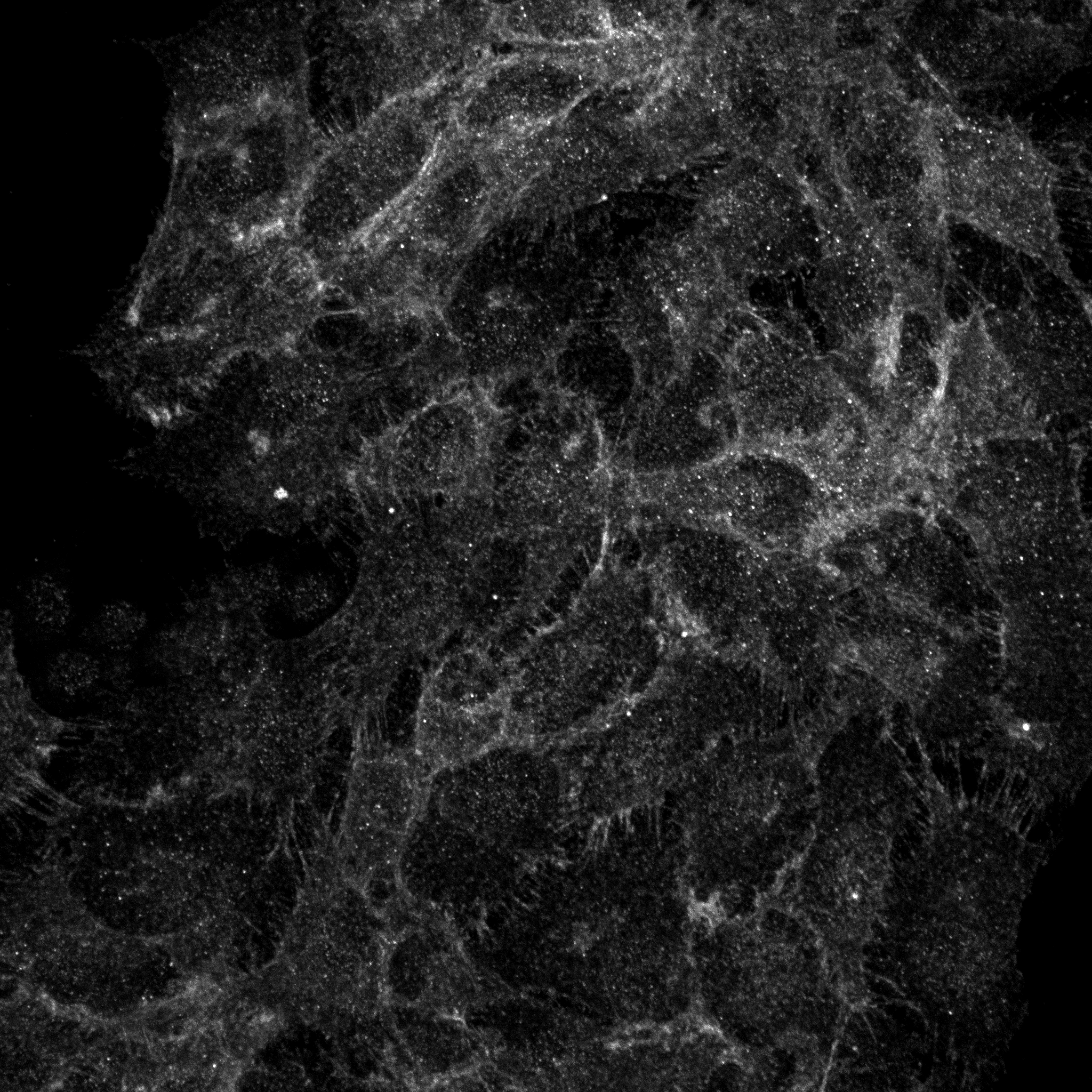

Supplement: Supplementary file 15 — Figure EV4 Source Data [file 44319_2026_751_MOESM15_ESM.zip › Raw_data_Figure EV4/Figure EV4E/C4-MAX_Cal27 + Exos NF shCtrl ECadh 647.tif]

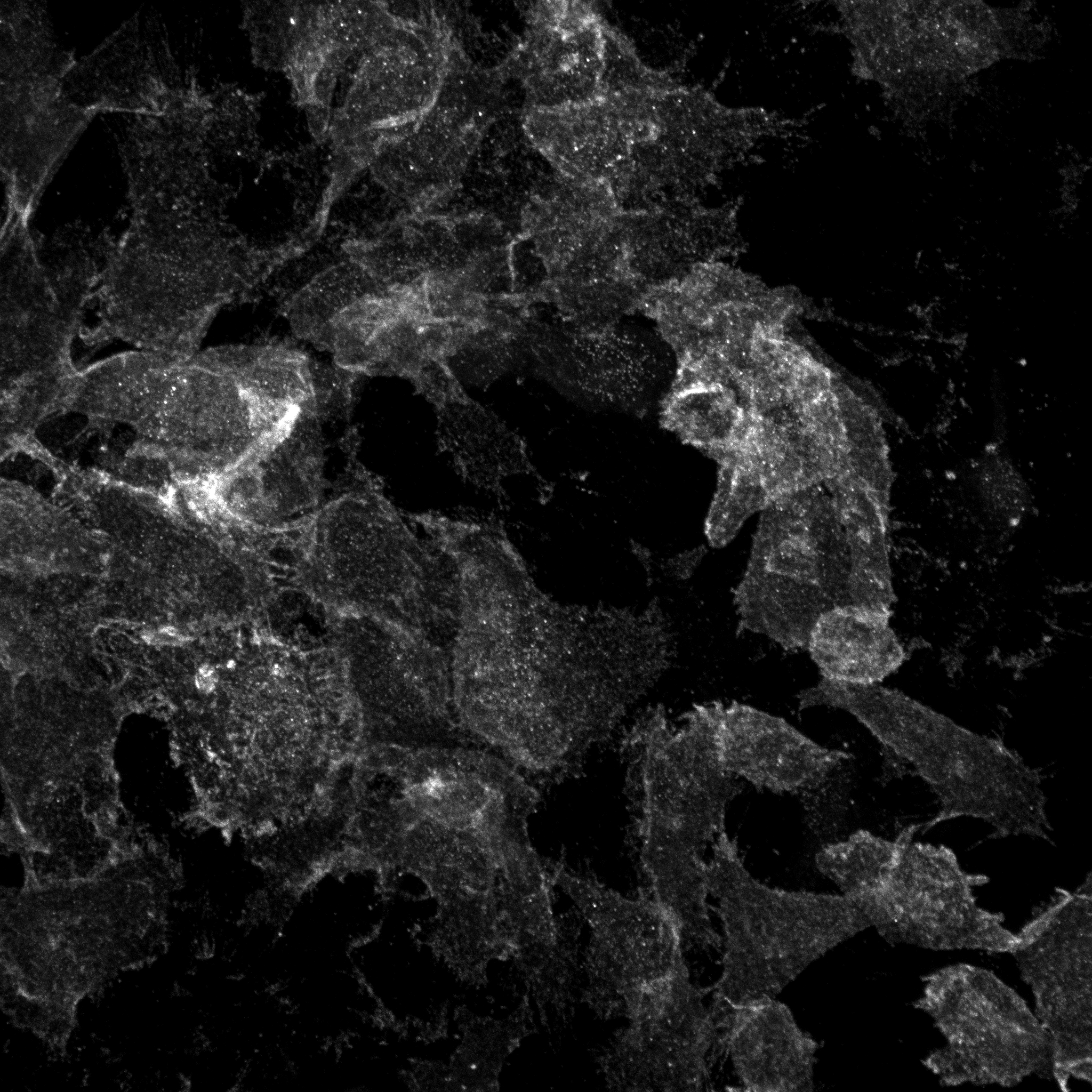

Supplement: Supplementary file 15 — Figure EV4 Source Data [file 44319_2026_751_MOESM15_ESM.zip › Raw_data_Figure EV4/Figure EV4E/C4-MAX_Cal27 + Exos NF shGq ECadh 647.tif]

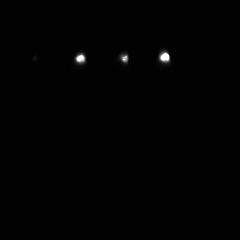

Supplement: Supplementary file 15 — Figure EV4 Source Data [file 44319_2026_751_MOESM15_ESM.zip › Raw_data_Figure EV4/Figure EV4F/INL20251001114137_SEQ/INL20251001114137_001/luminescent.TIF]

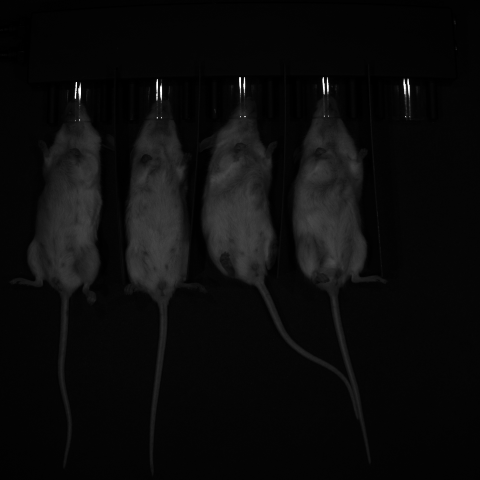

Supplement: Supplementary file 15 — Figure EV4 Source Data [file 44319_2026_751_MOESM15_ESM.zip › Raw_data_Figure EV4/Figure EV4F/INL20251001114137_SEQ/INL20251001114137_001/photograph.TIF]

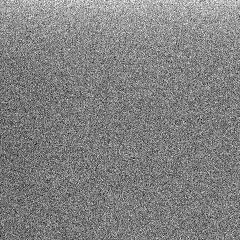

Supplement: Supplementary file 15 — Figure EV4 Source Data [file 44319_2026_751_MOESM15_ESM.zip › Raw_data_Figure EV4/Figure EV4F/INL20251001114137_SEQ/INL20251001114137_001/readbiasonly.TIF]

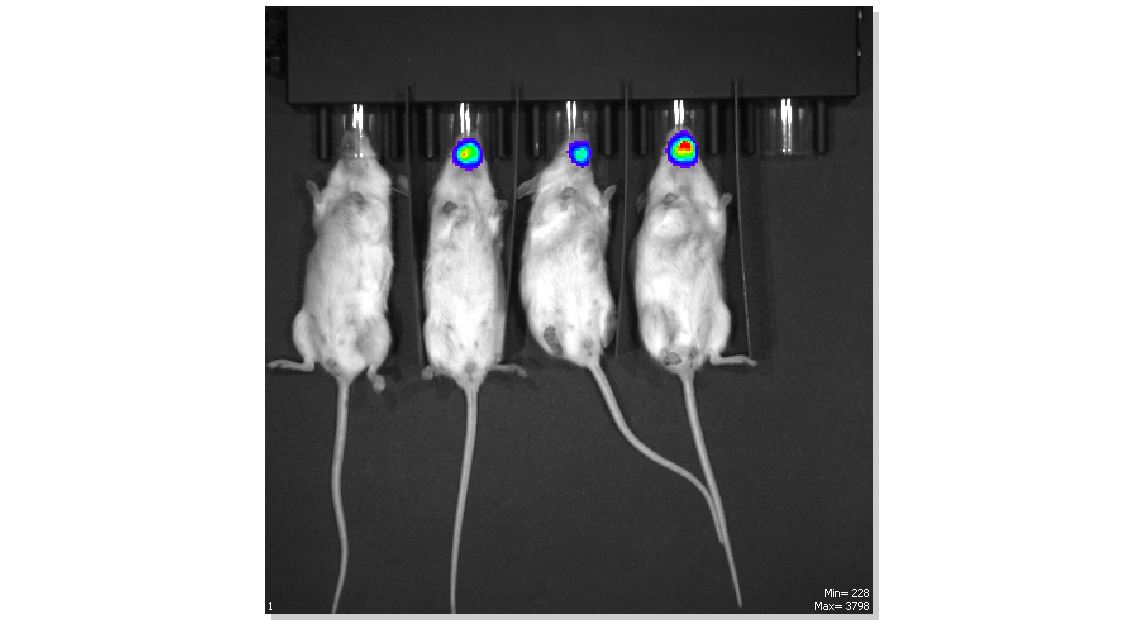

Supplement: Supplementary file 15 — Figure EV4 Source Data [file 44319_2026_751_MOESM15_ESM.zip › Raw_data_Figure EV4/Figure EV4F/INL20251001114137_SEQ/INL20251001114137_SEQ.PNG]

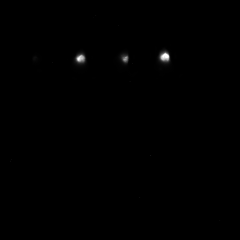

Supplement: Supplementary file 15 — Figure EV4 Source Data [file 44319_2026_751_MOESM15_ESM.zip › Raw_data_Figure EV4/Figure EV4F/INL20251001114246_SEQ/INL20251001114246_001/luminescent.TIF]

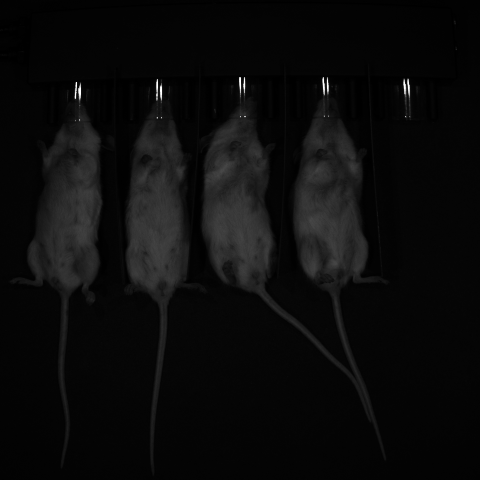

Supplement: Supplementary file 15 — Figure EV4 Source Data [file 44319_2026_751_MOESM15_ESM.zip › Raw_data_Figure EV4/Figure EV4F/INL20251001114246_SEQ/INL20251001114246_001/photograph.TIF]

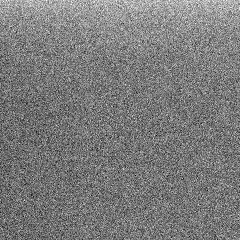

Supplement: Supplementary file 15 — Figure EV4 Source Data [file 44319_2026_751_MOESM15_ESM.zip › Raw_data_Figure EV4/Figure EV4F/INL20251001114246_SEQ/INL20251001114246_001/readbiasonly.TIF]

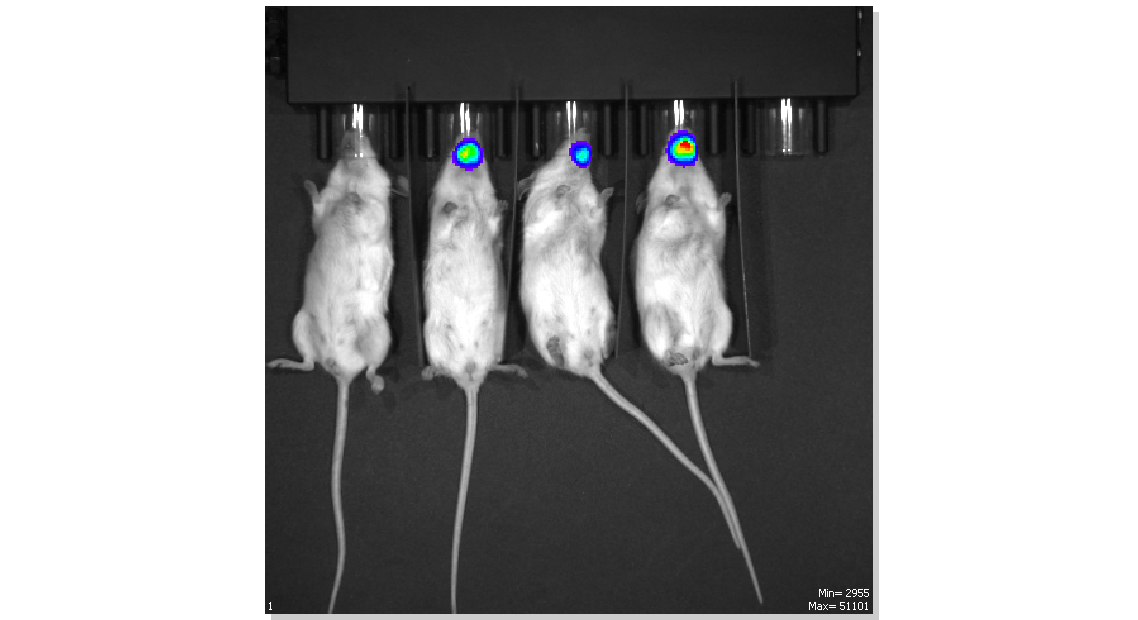

Supplement: Supplementary file 15 — Figure EV4 Source Data [file 44319_2026_751_MOESM15_ESM.zip › Raw_data_Figure EV4/Figure EV4F/INL20251001114246_SEQ/INL20251001114246_SEQ.PNG]

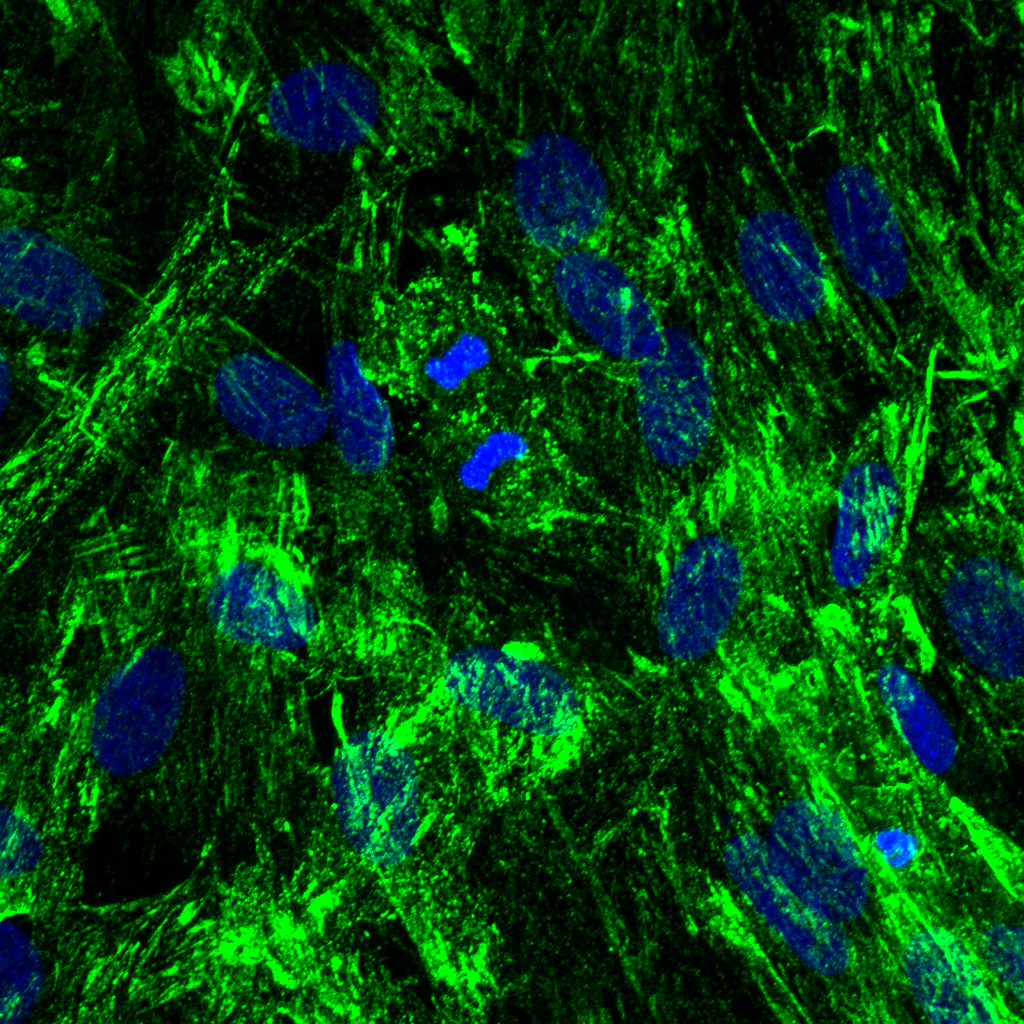

Supplement: Supplementary file 15 — Figure EV4 Source Data [file 44319_2026_751_MOESM15_ESM.zip › Raw_data_Figure EV4/Figure EV4G/CAFs nuclei Cav1.tif]

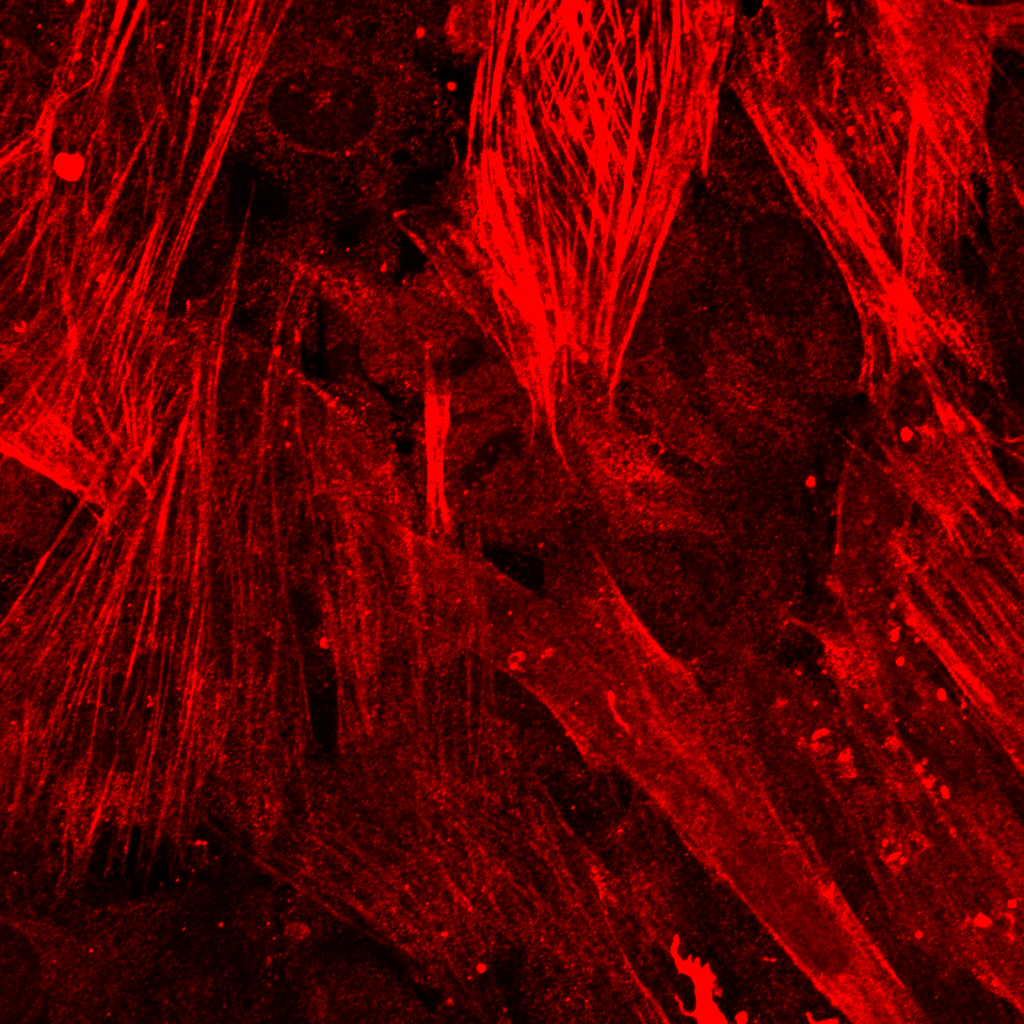

Supplement: Supplementary file 15 — Figure EV4 Source Data [file 44319_2026_751_MOESM15_ESM.zip › Raw_data_Figure EV4/Figure EV4G/CAFs SMA.tif]

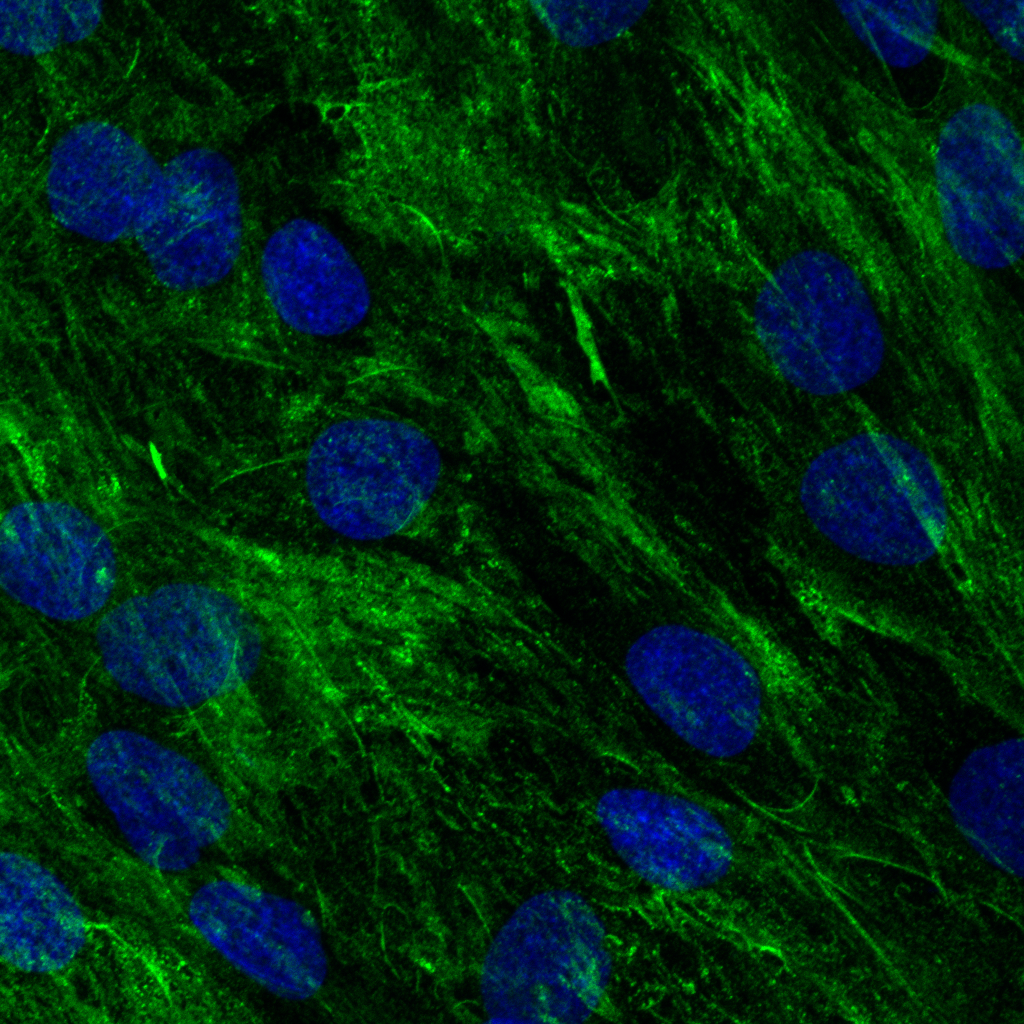

Supplement: Supplementary file 15 — Figure EV4 Source Data [file 44319_2026_751_MOESM15_ESM.zip › Raw_data_Figure EV4/Figure EV4G/NFs nuclei_cav1 2.tif]

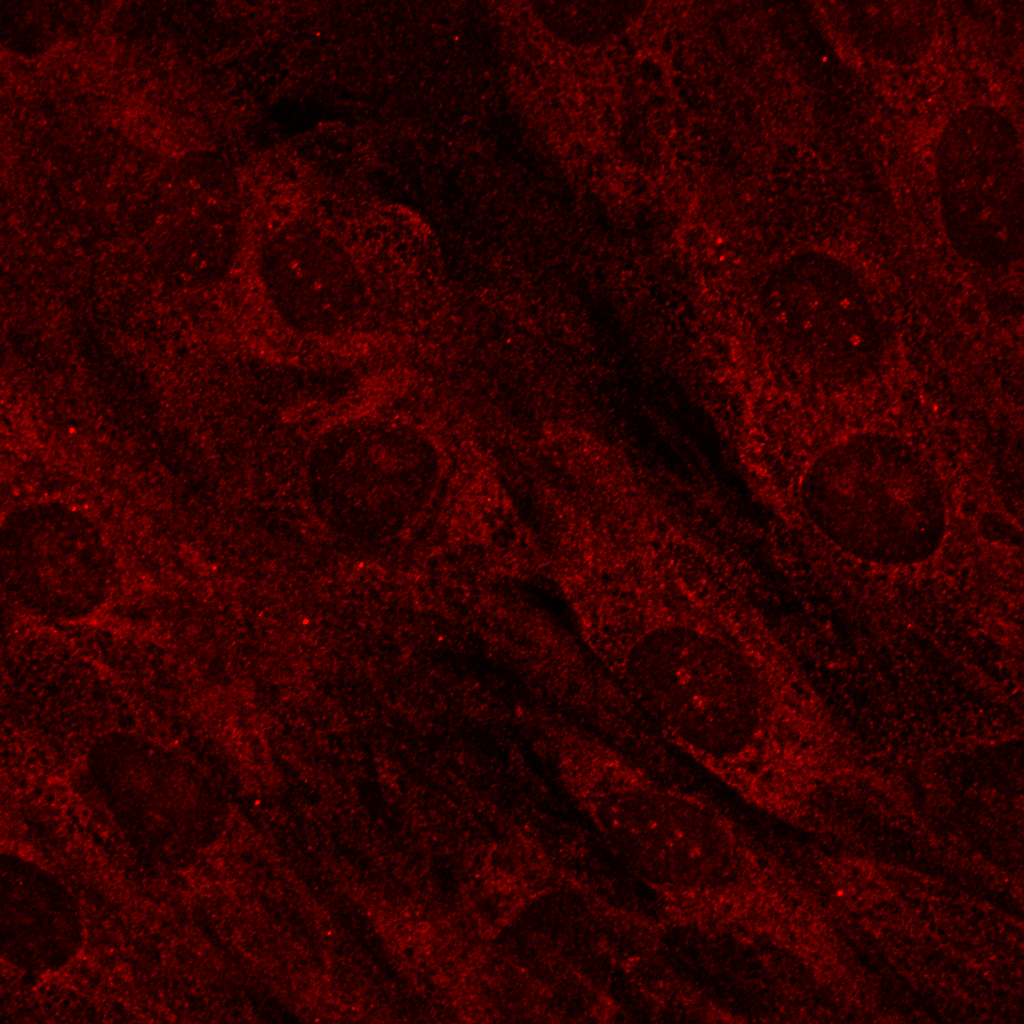

Supplement: Supplementary file 15 — Figure EV4 Source Data [file 44319_2026_751_MOESM15_ESM.zip › Raw_data_Figure EV4/Figure EV4G/NFs SMA 2.tif]

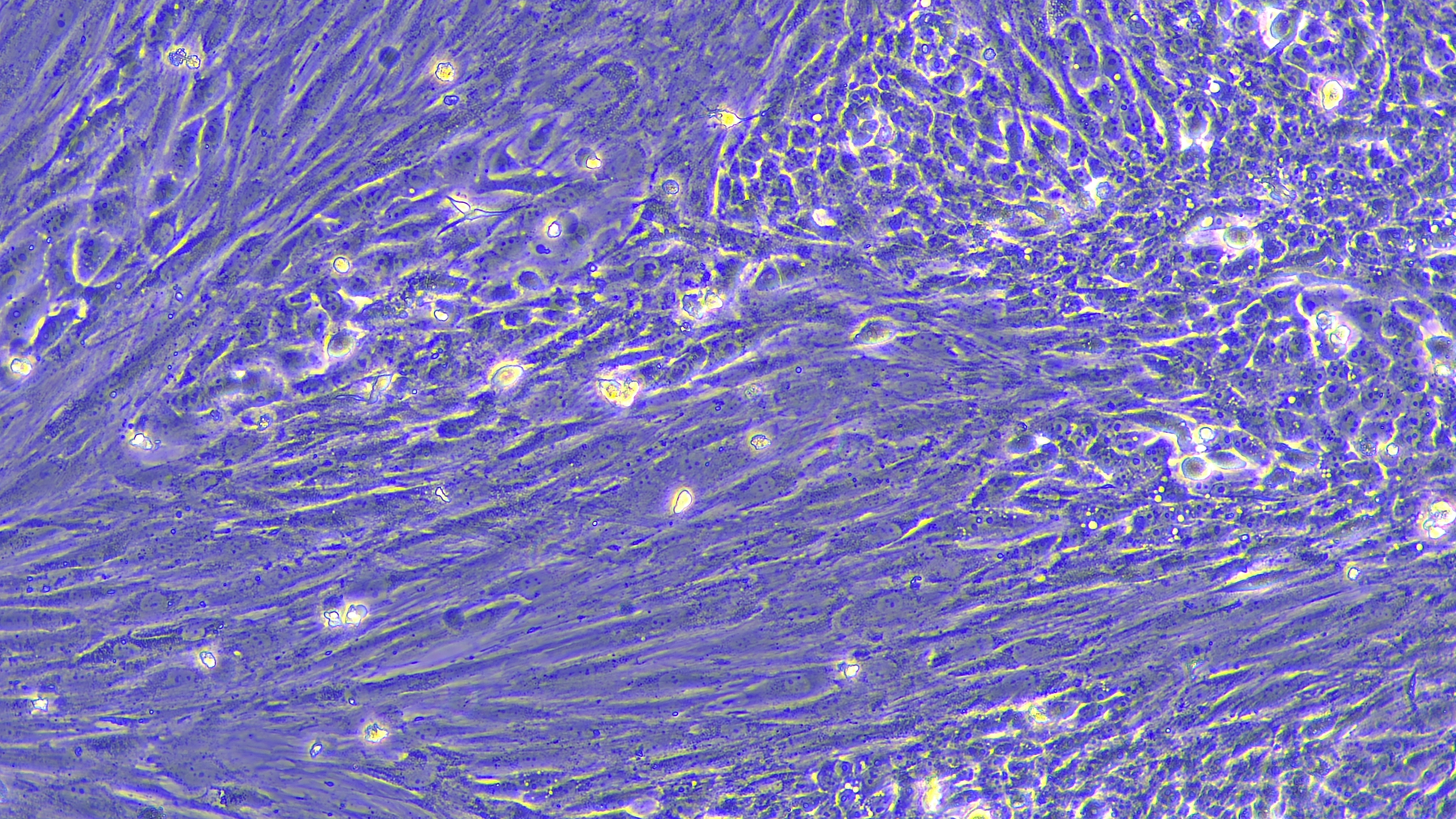

Supplement: Supplementary file 15 — Figure EV4 Source Data [file 44319_2026_751_MOESM15_ESM.zip › Raw_data_Figure EV4/Figure EV4H/caf+cal275d2311201204514in.tif]

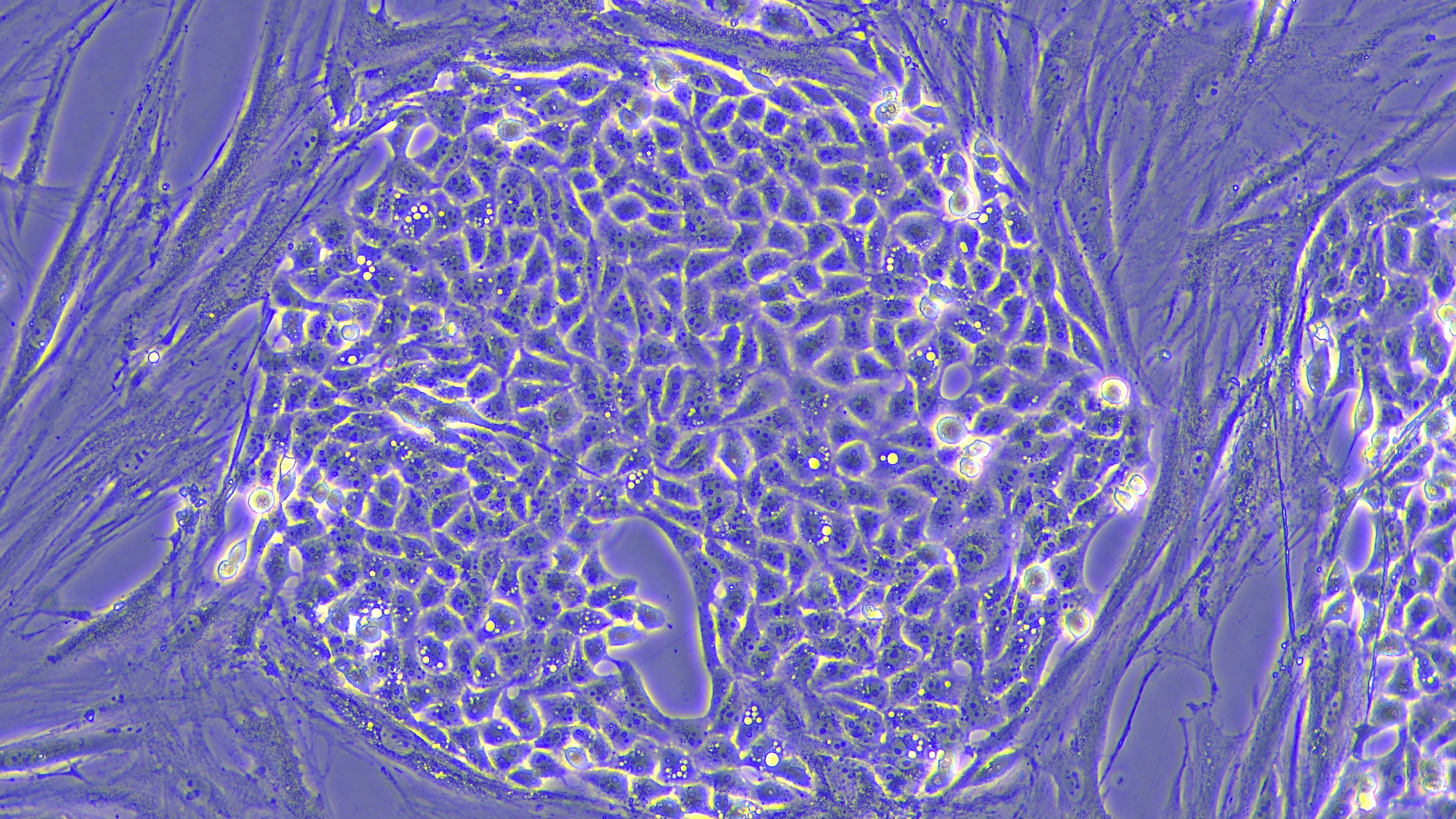

Supplement: Supplementary file 15 — Figure EV4 Source Data [file 44319_2026_751_MOESM15_ESM.zip › Raw_data_Figure EV4/Figure EV4H/nf2+cal275d2311201200350in.tif]
