# Supplementary material for: Loss of Gαq reshapes fibroblast traits and drives tumor-stroma remodeling in oral cancer progression
Source: EMBO Rep. 2026 Apr 10;27(10):2639–74. doi: 10.1038/s44319-026-00751-2 (PMC13219523; doi:10.1038/s44319-026-00751-2)
Supplement: Supplementary file 16 — Figure EV5 Source Data [file 44319_2026_751_MOESM16_ESM.zip › Raw_data_Figure EV5/EV5B/DI_MARTINO_MATRISOME_HIGHLY_PROLIFERATIVE_HNSCC.html]

Details for gene set DI\_MARTINO\_MATRISOME\_HIGHLY\_PROLIFERATIVE\_HNSCC[GSEA]

|  || Dataset | MEFs\_Gq\_norm\_counts\_Gq\_norm\_counts\_collapsed\_to\_symbols.categorical.cls #ko\_versus\_wt.categorical.cls #ko\_versus\_wt\_repos |
| Phenotype | categorical.cls#ko\_versus\_wt\_repos |
| Upregulated in class | 1 |
| GeneSet | DI\_MARTINO\_MATRISOME\_HIGHLY\_PROLIFERATIVE\_HNSCC |
| Enrichment Score (ES) | 0.5520091 |
| Normalized Enrichment Score (NES) | 1.5917275 |
| Nominal p-value | 0.021028038 |
| FDR q-value | 0.078240864 |
| FWER p-Value | 0.113 |
Table: GSEA Results Summary

  

Fig 1: Enrichment plot: DI\_MARTINO\_MATRISOME\_HIGHLY\_PROLIFERATIVE\_HNSCC      
 Profile of the Running ES Score & Positions of GeneSet Members on the Rank Ordered List

  

| SYMBOL | TITLE | RANK IN GENE LIST | RANK METRIC SCORE | RUNNING ES | CORE ENRICHMENT || 1 | FBN2 | fibrillin 2 [Source:HGNC Symbol;Acc:HGNC:3604] | 11 | 4.940 | 0.1167 | Yes |
| 2 | TGFBI | transforming growth factor beta induced [Source:HGNC Symbol;Acc:HGNC:11771] | 58 | 4.701 | 0.2255 | Yes |
| 3 | MFAP5 | microfibril associated protein 5 [Source:HGNC Symbol;Acc:HGNC:29673] | 256 | 3.695 | 0.3011 | Yes |
| 4 | EMILIN2 | elastin microfibril interfacer 2 [Source:HGNC Symbol;Acc:HGNC:19881] | 279 | 3.625 | 0.3859 | Yes |
| 5 | POSTN | periostin [Source:HGNC Symbol;Acc:HGNC:16953] | 290 | 3.569 | 0.4701 | Yes |
| 6 | IGFBP7 | insulin like growth factor binding protein 7 [Source:HGNC Symbol;Acc:HGNC:5476] | 564 | 2.448 | 0.5114 | Yes |
| 7 | COL8A1 | collagen type VIII alpha 1 chain [Source:HGNC Symbol;Acc:HGNC:2215] | 854 | 1.881 | 0.5382 | Yes |
| 8 | S100A6 | S100 calcium binding protein A6 [Source:HGNC Symbol;Acc:HGNC:10496] | 1510 | 1.266 | 0.5278 | Yes |
| 9 | LGALS1 | galectin 1 [Source:HGNC Symbol;Acc:HGNC:6561] | 1585 | 1.211 | 0.5520 | Yes |
| 10 | VTN | vitronectin [Source:HGNC Symbol;Acc:HGNC:12724] | 2378 | 0.873 | 0.5238 | No |
| 11 | EMILIN1 | elastin microfibril interfacer 1 [Source:HGNC Symbol;Acc:HGNC:19880] | 3045 | 0.715 | 0.4996 | No |
| 12 | ANXA2 | annexin A2 [Source:HGNC Symbol;Acc:HGNC:537] | 3843 | 0.532 | 0.4630 | No |
| 13 | PLG | plasminogen [Source:HGNC Symbol;Acc:HGNC:9071] | 4830 | 0.422 | 0.4120 | No |
| 14 | ECM1 | extracellular matrix protein 1 [Source:HGNC Symbol;Acc:HGNC:3153] | 6078 | 0.237 | 0.3406 | No |
| 15 | COL7A1 | collagen type VII alpha 1 chain [Source:HGNC Symbol;Acc:HGNC:2214] | 8295 | -0.011 | 0.2039 | No |
| 16 | TNC | tenascin C [Source:HGNC Symbol;Acc:HGNC:5318] | 9036 | -0.116 | 0.1609 | No |
| 17 | CBLN2 | cerebellin 2 precursor [Source:HGNC Symbol;Acc:HGNC:1544] | 11086 | -0.426 | 0.0444 | No |
| 18 | ITIH1 | inter-alpha-trypsin inhibitor heavy chain 1 [Source:HGNC Symbol;Acc:HGNC:6166] | 11337 | -0.434 | 0.0392 | No |
| 19 | TGFB1 | transforming growth factor beta 1 [Source:HGNC Symbol;Acc:HGNC:11766] | 11456 | -0.452 | 0.0427 | No |
| 20 | COL4A2 | collagen type IV alpha 2 chain [Source:HGNC Symbol;Acc:HGNC:2203] | 12022 | -0.534 | 0.0204 | No |
| 21 | COL12A1 | collagen type XII alpha 1 chain [Source:HGNC Symbol;Acc:HGNC:2188] | 12383 | -0.603 | 0.0125 | No |
| 22 | ITIH2 | inter-alpha-trypsin inhibitor heavy chain 2 [Source:HGNC Symbol;Acc:HGNC:6167] | 13353 | -0.843 | -0.0274 | No |
| 23 | FN1 | fibronectin 1 [Source:HGNC Symbol;Acc:HGNC:3778] | 14363 | -1.243 | -0.0602 | No |
| 24 | LTBP1 | latent transforming growth factor beta binding protein 1 [Source:HGNC Symbol;Acc:HGNC:6714] | 14445 | -1.302 | -0.0343 | No |
| 25 | COL16A1 | collagen type XVI alpha 1 chain [Source:HGNC Symbol;Acc:HGNC:2193] | 14531 | -1.357 | -0.0073 | No |
| 26 | COL6A2 | collagen type VI alpha 2 chain [Source:HGNC Symbol;Acc:HGNC:2212] | 14601 | -1.410 | 0.0219 | No |
| 27 | FBLN2 | fibulin 2 [Source:HGNC Symbol;Acc:HGNC:3601] | 15678 | -3.245 | 0.0325 | No |
Table: GSEA details [plain text format]

  

Fig 2: DI\_MARTINO\_MATRISOME\_HIGHLY\_PROLIFERATIVE\_HNSCC      
 Blue-Pink O' Gram in the Space of the Analyzed GeneSet

  

Fig 3: DI\_MARTINO\_MATRISOME\_HIGHLY\_PROLIFERATIVE\_HNSCC: Random ES distribution      
 Gene set null distribution of ES for **DI\_MARTINO\_MATRISOME\_HIGHLY\_PROLIFERATIVE\_HNSCC**

  
