# Supplementary material for: Loss of Gαq reshapes fibroblast traits and drives tumor-stroma remodeling in oral cancer progression
Source: EMBO Rep. 2026 Apr 10;27(10):2639–74. doi: 10.1038/s44319-026-00751-2 (PMC13219523; doi:10.1038/s44319-026-00751-2)
Supplement: Supplementary file 16 — Figure EV5 Source Data [file 44319_2026_751_MOESM16_ESM.zip › Raw_data_Figure EV5/EV5C/OBRADOVIC_2022_SUPTAB2_DIFF_1.html]

Details for gene set OBRADOVIC\_2022\_SUPTAB2\_DIFF\_1[GSEA]

|  || Dataset | MEFs\_Gq\_norm\_counts\_Gq\_norm\_counts\_collapsed\_to\_symbols.categorical.cls #ko\_versus\_wt.categorical.cls #ko\_versus\_wt\_repos |
| Phenotype | categorical.cls#ko\_versus\_wt\_repos |
| Upregulated in class | 1 |
| GeneSet | OBRADOVIC\_2022\_SUPTAB2\_DIFF\_1 |
| Enrichment Score (ES) | 0.42711988 |
| Normalized Enrichment Score (NES) | 1.5637456 |
| Nominal p-value | 0.0 |
| FDR q-value | 0.023079274 |
| FWER p-Value | 0.046 |
Table: GSEA Results Summary

  

Fig 1: Enrichment plot: OBRADOVIC\_2022\_SUPTAB2\_DIFF\_1      
 Profile of the Running ES Score & Positions of GeneSet Members on the Rank Ordered List

  

| SYMBOL | TITLE | RANK IN GENE LIST | RANK METRIC SCORE | RUNNING ES | CORE ENRICHMENT || 1 | OSR2 | odd-skipped related transciption factor 2 [Source:HGNC Symbol;Acc:HGNC:15830] | 32 | 4.855 | 0.0379 | Yes |
| 2 | FST | follistatin [Source:HGNC Symbol;Acc:HGNC:3971] | 81 | 4.603 | 0.0728 | Yes |
| 3 | C3 | complement C3 [Source:HGNC Symbol;Acc:HGNC:1318] | 135 | 4.291 | 0.1048 | Yes |
| 4 | STC1 | stanniocalcin 1 [Source:HGNC Symbol;Acc:HGNC:11373] | 136 | 4.290 | 0.1401 | Yes |
| 5 | TGFBR3 | transforming growth factor beta receptor 3 [Source:HGNC Symbol;Acc:HGNC:11774] | 164 | 4.163 | 0.1726 | Yes |
| 6 | EGR3 | early growth response 3 [Source:HGNC Symbol;Acc:HGNC:3240] | 234 | 3.803 | 0.1996 | Yes |
| 7 | PLPP3 | phospholipid phosphatase 3 [Source:HGNC Symbol;Acc:HGNC:9229] | 287 | 3.596 | 0.2260 | Yes |
| 8 | TNFAIP2 | TNF alpha induced protein 2 [Source:HGNC Symbol;Acc:HGNC:11895] | 304 | 3.456 | 0.2534 | Yes |
| 9 | GYPC | glycophorin C (Gerbich blood group) [Source:HGNC Symbol;Acc:HGNC:4704] | 309 | 3.435 | 0.2814 | Yes |
| 10 | IGFBP6 | insulin like growth factor binding protein 6 [Source:HGNC Symbol;Acc:HGNC:5475] | 320 | 3.387 | 0.3086 | Yes |
| 11 | CFH | complement factor H [Source:HGNC Symbol;Acc:HGNC:4883] | 356 | 3.209 | 0.3328 | Yes |
| 12 | RDH10 | retinol dehydrogenase 10 [Source:HGNC Symbol;Acc:HGNC:19975] | 410 | 2.970 | 0.3540 | Yes |
| 13 | C1R | complement C1r [Source:HGNC Symbol;Acc:HGNC:1246] | 429 | 2.865 | 0.3764 | Yes |
| 14 | PTGS2 | prostaglandin-endoperoxide synthase 2 [Source:HGNC Symbol;Acc:HGNC:9605] | 443 | 2.834 | 0.3989 | Yes |
| 15 | BDKRB2 | bradykinin receptor B2 [Source:HGNC Symbol;Acc:HGNC:1030] | 684 | 2.147 | 0.4017 | Yes |
| 16 | LTBP4 | latent transforming growth factor beta binding protein 4 [Source:HGNC Symbol;Acc:HGNC:6717] | 782 | 1.983 | 0.4120 | Yes |
| 17 | MGST1 | microsomal glutathione S-transferase 1 [Source:HGNC Symbol;Acc:HGNC:7061] | 798 | 1.956 | 0.4271 | Yes |
| 18 | KRT10 | keratin 10 [Source:HGNC Symbol;Acc:HGNC:6413] | 1257 | 1.429 | 0.4105 | No |
| 19 | CELF2 | CUGBP Elav-like family member 2 [Source:HGNC Symbol;Acc:HGNC:2550] | 1418 | 1.319 | 0.4114 | No |
| 20 | NFKB1 | nuclear factor kappa B subunit 1 [Source:HGNC Symbol;Acc:HGNC:7794] | 1726 | 1.131 | 0.4016 | No |
| 21 | MEDAG | mesenteric estrogen dependent adipogenesis [Source:HGNC Symbol;Acc:HGNC:25926] | 2359 | 0.876 | 0.3696 | No |
| 22 | DIO2 | iodothyronine deiodinase 2 [Source:HGNC Symbol;Acc:HGNC:2884] | 2369 | 0.875 | 0.3763 | No |
| 23 | NR4A3 | nuclear receptor subfamily 4 group A member 3 [Source:HGNC Symbol;Acc:HGNC:7982] | 2519 | 0.848 | 0.3740 | No |
| 24 | KLF2 | KLF transcription factor 2 [Source:HGNC Symbol;Acc:HGNC:6347] | 2798 | 0.780 | 0.3632 | No |
| 25 | NCOA7 | nuclear receptor coactivator 7 [Source:HGNC Symbol;Acc:HGNC:21081] | 2837 | 0.768 | 0.3671 | No |
| 26 | LMNA | lamin A/C [Source:HGNC Symbol;Acc:HGNC:6636] | 2934 | 0.743 | 0.3673 | No |
| 27 | DNAJB1 | DnaJ heat shock protein family (Hsp40) member B1 [Source:HGNC Symbol;Acc:HGNC:5270] | 3134 | 0.690 | 0.3606 | No |
| 28 | FOSB | FosB proto-oncogene, AP-1 transcription factor subunit [Source:HGNC Symbol;Acc:HGNC:3797] | 3137 | 0.689 | 0.3662 | No |
| 29 | APLP2 | amyloid beta precursor like protein 2 [Source:HGNC Symbol;Acc:HGNC:598] | 3485 | 0.602 | 0.3496 | No |
| 30 | PLAC9 | placenta associated 9 [Source:HGNC Symbol;Acc:HGNC:19255] | 3684 | 0.559 | 0.3419 | No |
| 31 | GPC3 | glypican 3 [Source:HGNC Symbol;Acc:HGNC:4451] | 3739 | 0.548 | 0.3431 | No |
| 32 | SFRP1 | secreted frizzled related protein 1 [Source:HGNC Symbol;Acc:HGNC:10776] | 3867 | 0.529 | 0.3395 | No |
| 33 | NR4A2 | nuclear receptor subfamily 4 group A member 2 [Source:HGNC Symbol;Acc:HGNC:7981] | 4374 | 0.455 | 0.3119 | No |
| 34 | A2M | alpha-2-macroglobulin [Source:HGNC Symbol;Acc:HGNC:7] | 4567 | 0.444 | 0.3036 | No |
| 35 | DPYSL2 | dihydropyrimidinase like 2 [Source:HGNC Symbol;Acc:HGNC:3014] | 5283 | 0.357 | 0.2622 | No |
| 36 | HMOX1 | heme oxygenase 1 [Source:HGNC Symbol;Acc:HGNC:5013] | 5367 | 0.341 | 0.2599 | No |
| 37 | DAB2 | DAB adaptor protein 2 [Source:HGNC Symbol;Acc:HGNC:2662] | 5435 | 0.332 | 0.2584 | No |
| 38 | WTAP | WT1 associated protein [Source:HGNC Symbol;Acc:HGNC:16846] | 5645 | 0.302 | 0.2480 | No |
| 39 | GSN | gelsolin [Source:HGNC Symbol;Acc:HGNC:4620] | 5654 | 0.301 | 0.2499 | No |
| 40 | SOD2 | superoxide dismutase 2 [Source:HGNC Symbol;Acc:HGNC:11180] | 6059 | 0.240 | 0.2268 | No |
| 41 | ECM1 | extracellular matrix protein 1 [Source:HGNC Symbol;Acc:HGNC:3153] | 6078 | 0.237 | 0.2277 | No |
| 42 | NFATC1 | nuclear factor of activated T cells 1 [Source:HGNC Symbol;Acc:HGNC:7775] | 6144 | 0.228 | 0.2255 | No |
| 43 | RAI2 | retinoic acid induced 2 [Source:HGNC Symbol;Acc:HGNC:9835] | 6212 | 0.218 | 0.2232 | No |
| 44 | EIF4E | eukaryotic translation initiation factor 4E [Source:HGNC Symbol;Acc:HGNC:3287] | 6280 | 0.208 | 0.2207 | No |
| 45 | GPRC5A | G protein-coupled receptor class C group 5 member A [Source:HGNC Symbol;Acc:HGNC:9836] | 6415 | 0.185 | 0.2139 | No |
| 46 | LEPR | leptin receptor [Source:HGNC Symbol;Acc:HGNC:6554] | 6423 | 0.183 | 0.2150 | No |
| 47 | TRIB1 | tribbles pseudokinase 1 [Source:HGNC Symbol;Acc:HGNC:16891] | 7081 | 0.089 | 0.1750 | No |
| 48 | GADD45A | growth arrest and DNA damage inducible alpha [Source:HGNC Symbol;Acc:HGNC:4095] | 7154 | 0.078 | 0.1711 | No |
| 49 | CES1 | carboxylesterase 1 [Source:HGNC Symbol;Acc:HGNC:1863] | 7217 | 0.068 | 0.1679 | No |
| 50 | NAF1 | nuclear assembly factor 1 ribonucleoprotein [Source:HGNC Symbol;Acc:HGNC:25126] | 7271 | 0.060 | 0.1651 | No |
| 51 | NR4A1 | nuclear receptor subfamily 4 group A member 1 [Source:HGNC Symbol;Acc:HGNC:7980] | 8540 | -0.043 | 0.0868 | No |
| 52 | VEGFA | vascular endothelial growth factor A [Source:HGNC Symbol;Acc:HGNC:12680] | 8557 | -0.045 | 0.0861 | No |
| 53 | FBLN5 | fibulin 5 [Source:HGNC Symbol;Acc:HGNC:3602] | 8636 | -0.058 | 0.0818 | No |
| 54 | TNFSF13B | TNF superfamily member 13b [Source:HGNC Symbol;Acc:HGNC:11929] | 8818 | -0.084 | 0.0712 | No |
| 55 | HSPB8 | heat shock protein family B (small) member 8 [Source:HGNC Symbol;Acc:HGNC:30171] | 9607 | -0.200 | 0.0240 | No |
| 56 | MAP3K8 | mitogen-activated protein kinase kinase kinase 8 [Source:HGNC Symbol;Acc:HGNC:6860] | 10153 | -0.280 | -0.0075 | No |
| 57 | MEG3 | maternally expressed 3 [Source:HGNC Symbol;Acc:HGNC:14575] | 10499 | -0.334 | -0.0261 | No |
| 58 | LAMA2 | laminin subunit alpha 2 [Source:HGNC Symbol;Acc:HGNC:6482] | 10522 | -0.338 | -0.0247 | No |
| 59 | EIF4A3 | eukaryotic translation initiation factor 4A3 [Source:HGNC Symbol;Acc:HGNC:18683] | 10553 | -0.343 | -0.0238 | No |
| 60 | DUSP1 | dual specificity phosphatase 1 [Source:HGNC Symbol;Acc:HGNC:3064] | 10852 | -0.388 | -0.0391 | No |
| 61 | KRT5 | keratin 5 [Source:HGNC Symbol;Acc:HGNC:6442] | 11091 | -0.426 | -0.0503 | No |
| 62 | SQSTM1 | sequestosome 1 [Source:HGNC Symbol;Acc:HGNC:11280] | 11352 | -0.436 | -0.0629 | No |
| 63 | NFIB | nuclear factor I B [Source:HGNC Symbol;Acc:HGNC:7785] | 11753 | -0.498 | -0.0836 | No |
| 64 | NTRK2 | neurotrophic receptor tyrosine kinase 2 [Source:HGNC Symbol;Acc:HGNC:8032] | 12129 | -0.553 | -0.1023 | No |
| 65 | CRLF1 | cytokine receptor like factor 1 [Source:HGNC Symbol;Acc:HGNC:2364] | 12278 | -0.582 | -0.1067 | No |
| 66 | TXN | thioredoxin [Source:HGNC Symbol;Acc:HGNC:12435] | 12338 | -0.595 | -0.1055 | No |
| 67 | TGIF1 | TGFB induced factor homeobox 1 [Source:HGNC Symbol;Acc:HGNC:11776] | 12420 | -0.613 | -0.1054 | No |
| 68 | GLA | galactosidase alpha [Source:HGNC Symbol;Acc:HGNC:4296] | 12864 | -0.716 | -0.1270 | No |
| 69 | KLF4 | KLF transcription factor 4 [Source:HGNC Symbol;Acc:HGNC:6348] | 12951 | -0.738 | -0.1263 | No |
| 70 | ABL2 | ABL proto-oncogene 2, non-receptor tyrosine kinase [Source:HGNC Symbol;Acc:HGNC:77] | 13084 | -0.771 | -0.1281 | No |
| 71 | CREB5 | cAMP responsive element binding protein 5 [Source:HGNC Symbol;Acc:HGNC:16844] | 13118 | -0.781 | -0.1238 | No |
| 72 | CFD | complement factor D [Source:HGNC Symbol;Acc:HGNC:2771] | 14026 | -1.076 | -0.1712 | No |
| 73 | TMEM176B | transmembrane protein 176B [Source:HGNC Symbol;Acc:HGNC:29596] | 14656 | -1.441 | -0.1984 | No |
| 74 | CCND2 | cyclin D2 [Source:HGNC Symbol;Acc:HGNC:1583] | 14748 | -1.514 | -0.1916 | No |
| 75 | KDM6B | lysine demethylase 6B [Source:HGNC Symbol;Acc:HGNC:29012] | 15249 | -2.148 | -0.2049 | No |
| 76 | PBX1 | PBX homeobox 1 [Source:HGNC Symbol;Acc:HGNC:8632] | 15371 | -2.377 | -0.1929 | No |
| 77 | FABP3 | fatty acid binding protein 3 [Source:HGNC Symbol;Acc:HGNC:3557] | 15458 | -2.585 | -0.1769 | No |
| 78 | TNFAIP3 | TNF alpha induced protein 3 [Source:HGNC Symbol;Acc:HGNC:11896] | 15653 | -3.145 | -0.1631 | No |
| 79 | JUND | JunD proto-oncogene, AP-1 transcription factor subunit [Source:HGNC Symbol;Acc:HGNC:6206] | 15731 | -3.441 | -0.1396 | No |
| 80 | BTG2 | BTG anti-proliferation factor 2 [Source:HGNC Symbol;Acc:HGNC:1131] | 15768 | -3.609 | -0.1122 | No |
| 81 | AKAP12 | A-kinase anchoring protein 12 [Source:HGNC Symbol;Acc:HGNC:370] | 15777 | -3.624 | -0.0829 | No |
| 82 | EFEMP1 | EGF containing fibulin extracellular matrix protein 1 [Source:HGNC Symbol;Acc:HGNC:3218] | 15929 | -4.224 | -0.0575 | No |
| 83 | ATF3 | activating transcription factor 3 [Source:HGNC Symbol;Acc:HGNC:785] | 15996 | -4.451 | -0.0250 | No |
| 84 | FHL1 | four and a half LIM domains 1 [Source:HGNC Symbol;Acc:HGNC:3702] | 16037 | -4.600 | 0.0104 | No |
Table: GSEA details [plain text format]

  

Fig 2: OBRADOVIC\_2022\_SUPTAB2\_DIFF\_1      
 Blue-Pink O' Gram in the Space of the Analyzed GeneSet

  

Fig 3: OBRADOVIC\_2022\_SUPTAB2\_DIFF\_1: Random ES distribution      
 Gene set null distribution of ES for **OBRADOVIC\_2022\_SUPTAB2\_DIFF\_1**

  
