# Supplementary material for: Loss of Gαq reshapes fibroblast traits and drives tumor-stroma remodeling in oral cancer progression
Source: EMBO Rep. 2026 Apr 10;27(10):2639–74. doi: 10.1038/s44319-026-00751-2 (PMC13219523; doi:10.1038/s44319-026-00751-2)
Supplement: Supplementary file 17 — Expanded View Figures [file 44319_2026_751_MOESM17_ESM.pdf]

# Expanded View Figures

**Figure EV1. Lack or inhibition of Gαq activity modifies fibroblast-specific features.**

(**A**) Phase-contrast microscope images of WT and GαqKO MEFs 24 h after seeding (Scale bar, 25 µm). (**B**) Confocal analysis of F-actin and Gαq subcellular distribution in WT and GαqKO MEFs (Scale bar 25 µm). (**C**) Western blot analysis of Cav1 and PDGFR expression in fibroblasts after 96 h in culture. (**D**) Knock-down efficiency of Gαq in WT MEFs upon lentiviral infection of short-hairpin RNA constructs was assessed by Western blot analysis and PDGFR distribution upon Gαq depletion by confocal microscopy. Z-stack projections of confocal microscopy images showing increased collagen I matrix deposition in Gαq-depleted MEFs (Scale bar, 50 µm). Zoomed images are also shown. (**E**) Western blot analysis of PDGFR expression levels in WT MEFs treated for 24 h with the Gαq inhibitor YM254890 (5 µM). (**F**, **G**) Fibronectin matrix deposition (in gray) and lysosomal distribution (in red) (Scale bar 50 µm) (**F**) or Cav1 staining (**G**) in WT MEFs treated with the Gαq inhibitor YM254890 (5 µM). A LAMP1 antibody was used as a lysosome marker (Scale bar 25 µm). (**H**) Microscopy images of HN13 and UMSCC47 cells co-cultured with WT or GαqKO fibroblasts. Cells were stained for the epithelial-mesenchymal transition marker E-cadherin (in gray), and nuclei were stained with Hoechst. Zoomed images show E-cadherin redistribution from cell-cell contact sites to intracellular compartments in the presence of GαqKO fibroblasts (arrows) (Scale bar, 25 µm). (**I**) Representative phase-contrast images (Scale bar,) of spheroids formed in Matrigel after 72 h of co-culture of Cal27 tumor cells and either WT or GαqKO MEFs (Scale bar, 150 µm). Source data are available online for this figure.

 © The Author(s)

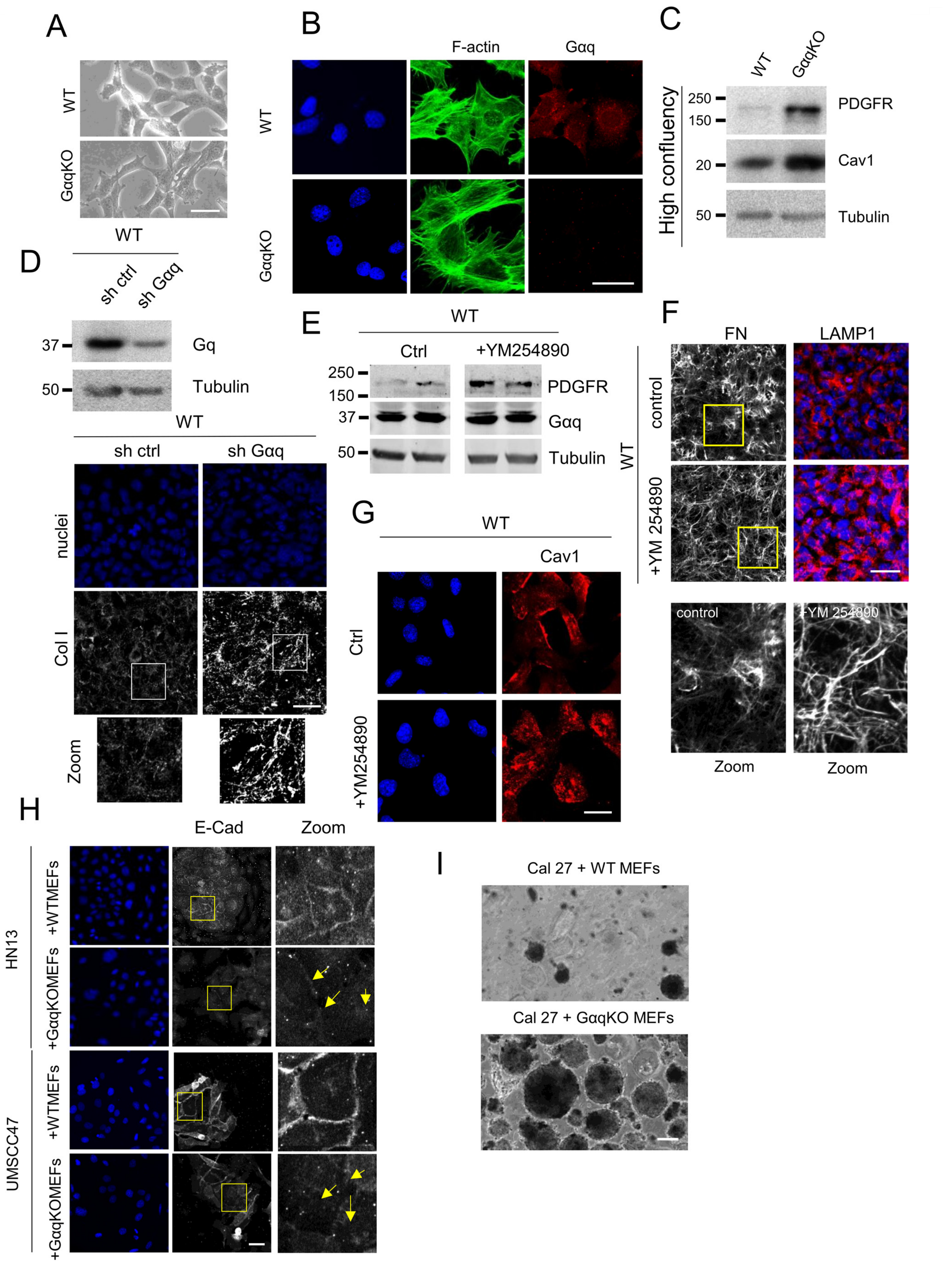
A
WT
GαqKO
B
F-actin
Gαq
WT
GαqKO
C
WT
GαqKO
High confluency
250
150
20
50
PDGFR
Cav1
Tubulin
D
WT
sh ctrl
sh Gαq
37
50
Gq
Tubulin
WT
sh ctrl
sh Gαq
nuclei
Col I
Zoom
E
WT
Ctrl
+YM254890
250
150
37
50
PDGFR
Gαq
Tubulin
F
FN
LAMP1
control
+YM 254890
WT
control
+YM 254890
Zoom
Zoom
G
WT
Cav1
Ctrl
+YM254890
H
E-Cad
Zoom
HN13
+WTMEFs
+GαqKOMEFs
UMSCC47
+WTMEFs
+GαqKOMEFs
I
Cal 27 + WT MEFs
Cal 27 + GαqKO MEFs

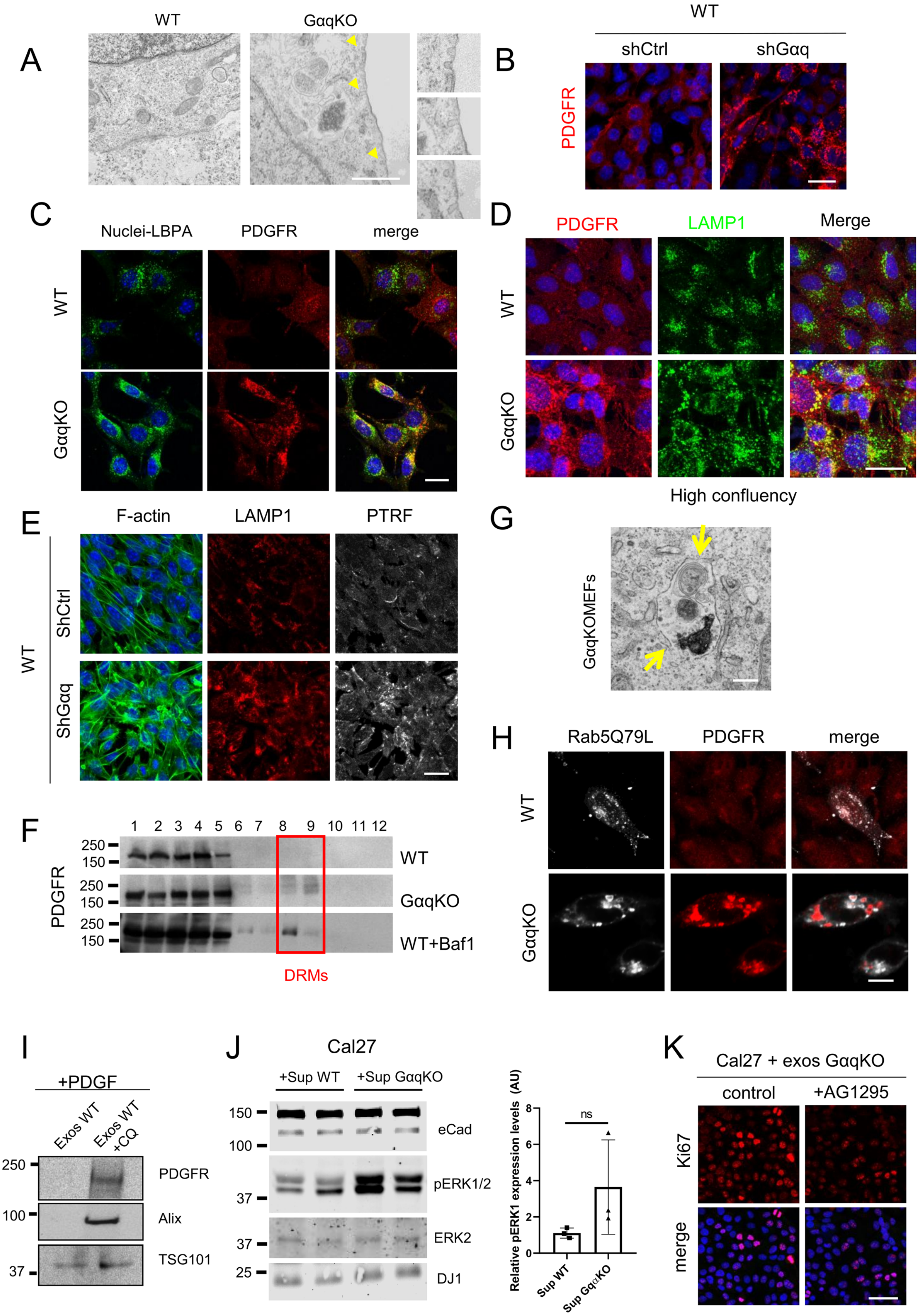
A
WT
GαqKO
B
WT
shCtrl
shGαq
PDGFR
C
Nuclei-LBPA
PDGFR
merge
WT
GαqKO
D
PDGFR
LAMP1
Merge
WT
GαqKO
High confluency
E
F-actin
LAMP1
PTRF
WT
ShCtrl
ShGαq
G
GαqKOMEFs
F
1 2 3 4 5 6 7 8 9 10 11 12
PDGFR
250
150
WT
GαqKO
WT+Baf1
DRMs
H
Rab5Q79L
PDGFR
merge
WT
GαqKO
I
+PDGF
Exos WT
Exos WT +CQ
250
100
37
PDGFR
Alix
TSG101
J
Cal27
+Sup WT
+Sup GαqKO
150
100
37
25
eCad
pERK1/2
ERK2
DJ1
Relative pERK1 expression levels (AU)
ns
Sup WT
Sup GαqKO
K
Cal27 + exos GαqKO
control
+AG1295
Ki67
merge

 © The Author(s)

**Figure EV2. Absence of Gαq alters Cav1 dynamics, lysosomal localization, and turnover.**

(**A**) WT and GαqKO MEFs were analyzed by electron microscopy. A higher number of caveolae (indicated by arrows) are present in GαqKO MEFs compared to WT (Scale bar 1 μm). (**B**) Distribution pattern of PDGFR in WT MEFs upon Gαq silencing by lentiviral infection with a short-hairpin RNA targeting Gαq (Scale bar, 25 μm). (**C**) Confocal microscopy analysis of the subcellular distribution of the MBV marker LBPA (green) and PDGFR (red) in WT and GαqKO MEFs. Colocalization is shown in yellow (Scale bar, 25 μm). (**D**) Confocal microscopy analysis of LAMP1 (green) and PDGFR (red) subcellular distribution in WT and GαqKO MEFs under confluent conditions (Scale bar, 25 μm). (**E**) Distribution pattern of F-actin, LAMP1 and PTRF upon Gαq silencing in WT MEFs by lentiviral infection with a short-hairpin RNA targeting Gαq (Scale bar, 25 μm). (**F**) PDGFR expression distribution by Western blot analysis of sucrose density gradient fractions from WT and GαqKO MEFs and WT MEFs treated with Bafilomycin 1 (1 nM). The red box denotes DRMs-enriched fractions. (**G**) Electron microscopy image showing an aberrantly loaded lysosome in GαqKO MEFs, indicated by the yellow arrows (Scale bar 200 nm). (**H**) Distribution of PDGFR (red) in MEFs expressing Rab5(Q79L) (gray). Aberrant accumulation of PDFGR in MVBs is preferentially observed in GαqKO MEFs (Scale bar, 10 μm). (**I**) Western blot analysis of the indicated proteins in exosomes derived from PDGF-treated WT cells in the absence or presence of chloroquine (CQ, 1 μM). TSG101 is used as an exosomal marker. (**J**) Western blot analysis showing ERK activation in Cal27 cells treated with supernatants from WT and GαqKO MEFs; quantification of p-ERK levels is shown. (**K**) Effect of GαqKO MEF-derived exosomes and PDGFR inhibitor AG1295 on Cal27 cell proliferation assessed by Ki67 staining (Scale bar, 50 μm). Data information: In (J) data are presented as mean ± SD, $n = 3$ biological replicates. Statistical significance in (**J**) was determined using an unpaired *t*-test with Welch's correction (ns $p = 0.2321$). Source data are available online for this figure.

© The Author(s)

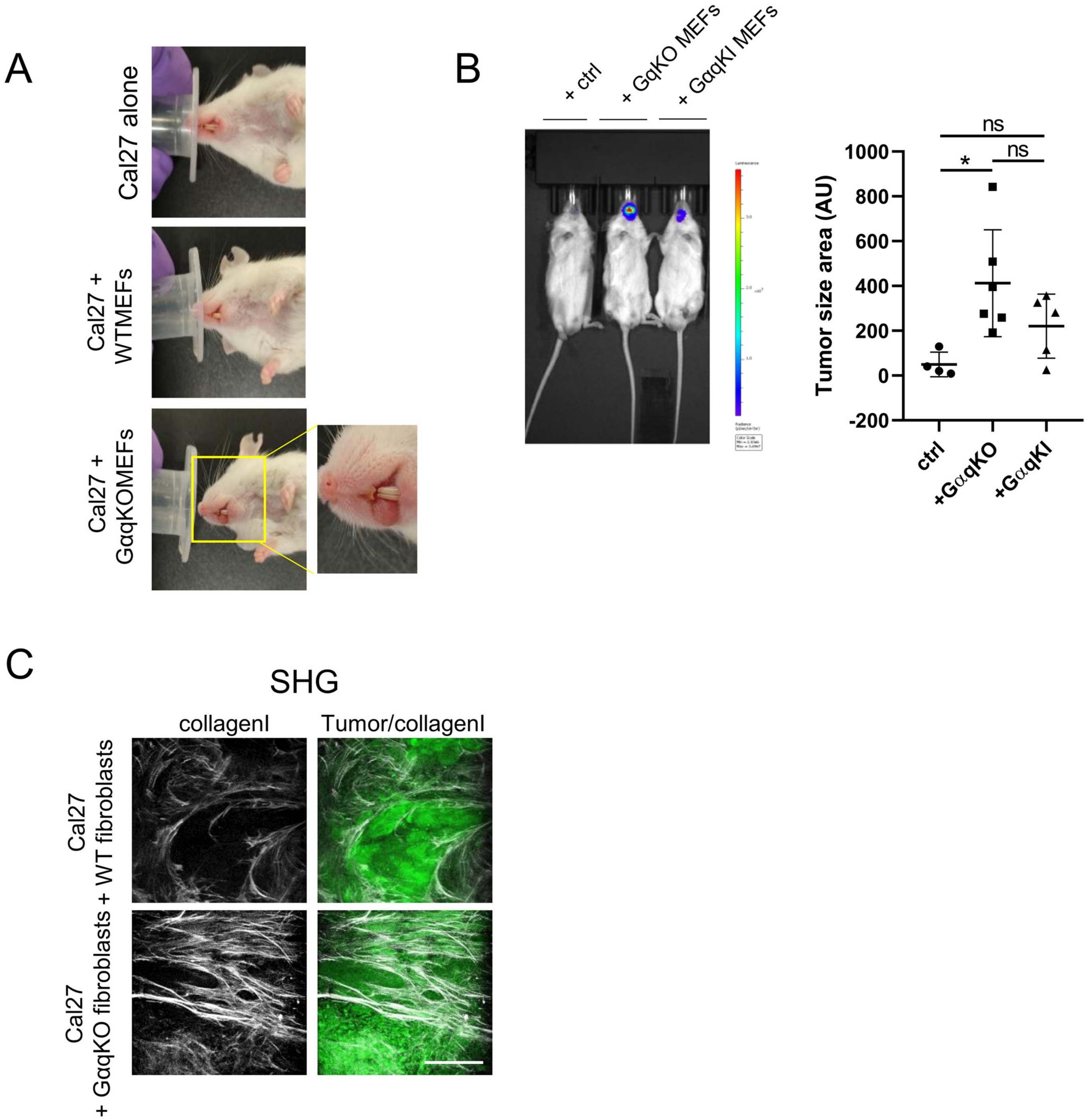


**Figure EV3. Large desmoplastic stroma-bearing tumors are induced by GαqKO MEFs in vivo.**

(**A**) Representative macroscopic images of tongue tumor generated by orthotopic injection of Cal27 oral cancer cells alone or in combination with WT or GαqKO MEFs. (**B**) Bioluminescence detection of tumor cells from orthotopic injection of Cal27 cells alone or in combination with either GαqKO MEFs or a Gαq-reconstituted version (GαqKI MEFs). Graphs depict tumor size determined by analysis of H&E-stained tissue images ($n \geq 4$ animals per condition) (**C**) Representative images of self-assembled collagen matrix organization in the indicated tumors, as measured by second harmonic generation (SHG) microscopy (Scale bar, 50 µm). Data information: In (**B**), data were presented as mean ± SD, $n \geq 4$ animals per condition. Statistical significance in (**B**) was determined using a one-way ANOVA (Ctrl vs. GαqKI, ns $p = 0.5292$; GαqKO vs. GαqKI, ns $p = 0.2977$; Ctrl vs. GαqKO, $^*p = 0.0243$). Source data are available online for this figure.

 © The Author(s)

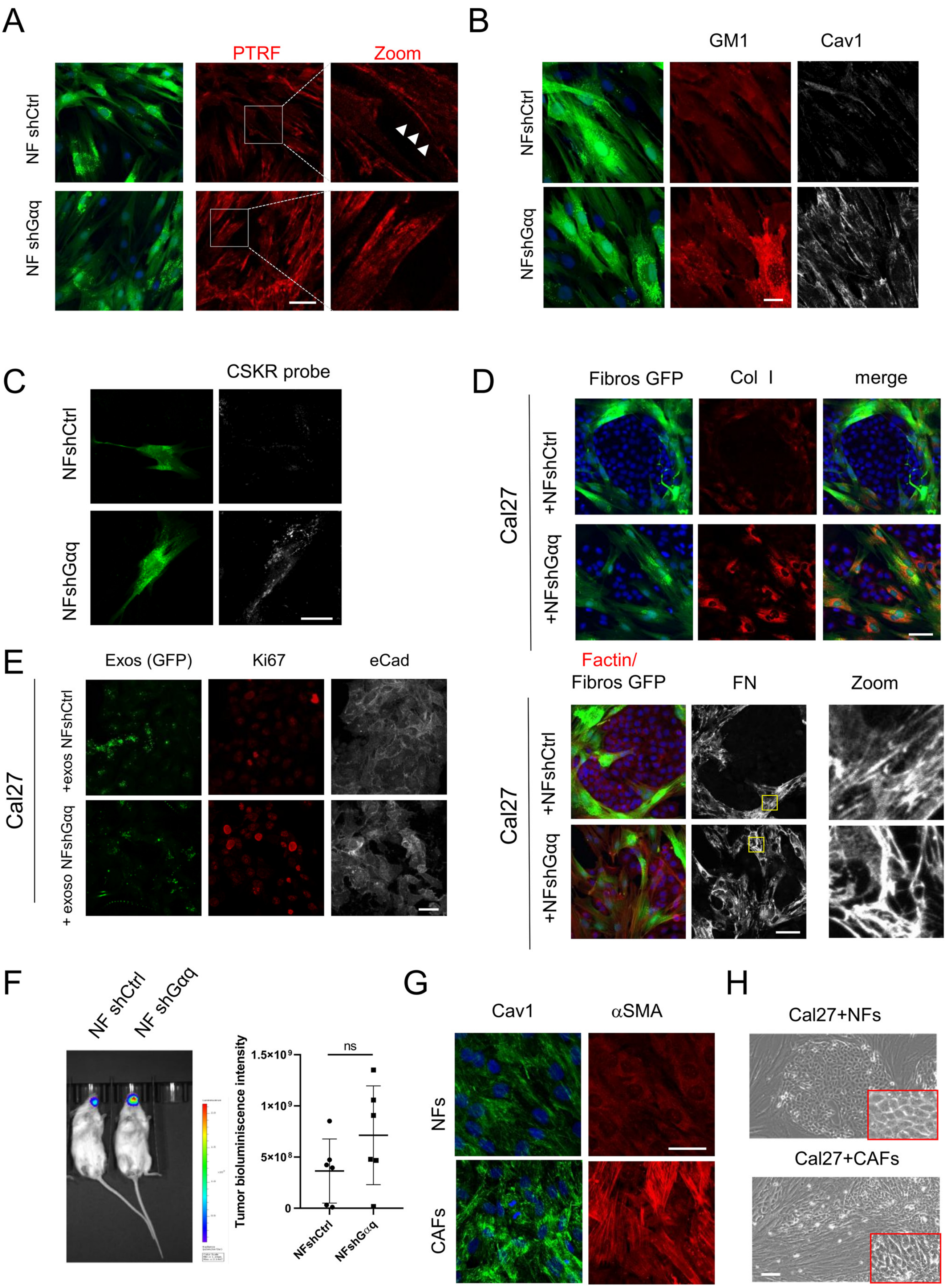

A
PTRF
Zoom
NF shCtrl
NF shGαq
B
GM1
Cav1
NFshCtrl
NFshGαq
C
CSKR probe
NFshCtrl
NFshGαq
D
Fibros GFP
Col I
merge
Cal27
+NFshCtrl
+NFshGαq
E
Exos (GFP)
Ki67
eCad
Cal27
+exos NFshCtrl
+ exoso NFshGαq
Factin/
Fibros GFP
FN
Zoom
Cal27
+NFshCtrl
+NFshGαq
F
NF shCtrl
NF shGαq
Tumor bioluminiscence intensity
1.5×10⁹
1×10⁹
5×10⁸
0
ns
NFshCtrl
NFshGαq
G
Cav1
αSMA
NFs
CAFs
H
Cal27+NFs
Cal27+CAFs

**Figure EV4. Effects of Gαq knockdown on normal human fibroblasts.**

Analysis by confocal microscopy of PTRF (**A**) or GM1 (**B**) staining in control (NF shctrl) and Gαq-silenced (NF shGαq) human fibroblasts (Scale bar, 25 µm). (**C**) Distribution of ceramide probe corresponding to eGFP-tagged KSR1 CA3 domain (KSR, aa 317–400) (ceramide-binding domain) (in gray) in NF shctrl and NF shGαq human fibroblasts (Scale bar 25 µm). (**D**) Analysis of matrix deposited in co-cultures of GFP-labeled NF shctrl or NF shGαq human fibroblasts with Cal27 cells. Fibroblasts are shown in green, FN in gray, collagen I in red, and DAPI-labeled nuclei in blue (Scale bar, 50 µm). (**E**) Staining pattern of Ki67 in Cal27 cells treated with exosomes derived from NF shctrl or NFs shGαq (Scale bar, 50 µm). (**F**) Representative bioluminescence images of orthotopic tongue tumors generated by co-injection of a luciferase-expressing tumor tongue cancer cell line (Cal27-luc) with either NF shctrl or NF shGαq human fibroblasts. (**G**) Confocal microscopy images displaying F-actin (green) and α-SMA (red) in NFs or CAFs (Scale bar, 50 µm). (**H**) Phase-contrast microscope images of one selected pair of NF-CAFs co-culture with Cal27 cells (Scale bar, 100 µm), showing differential morphological features. Data information: In (**F**) data were presented as mean ± SD, $n = 6$ animals per condition. Statistical significance in (**F**) was determined using an unpaired $t$-test (ns $p = 0.1676$). Source data are available online for this figure.


© The Author(s)

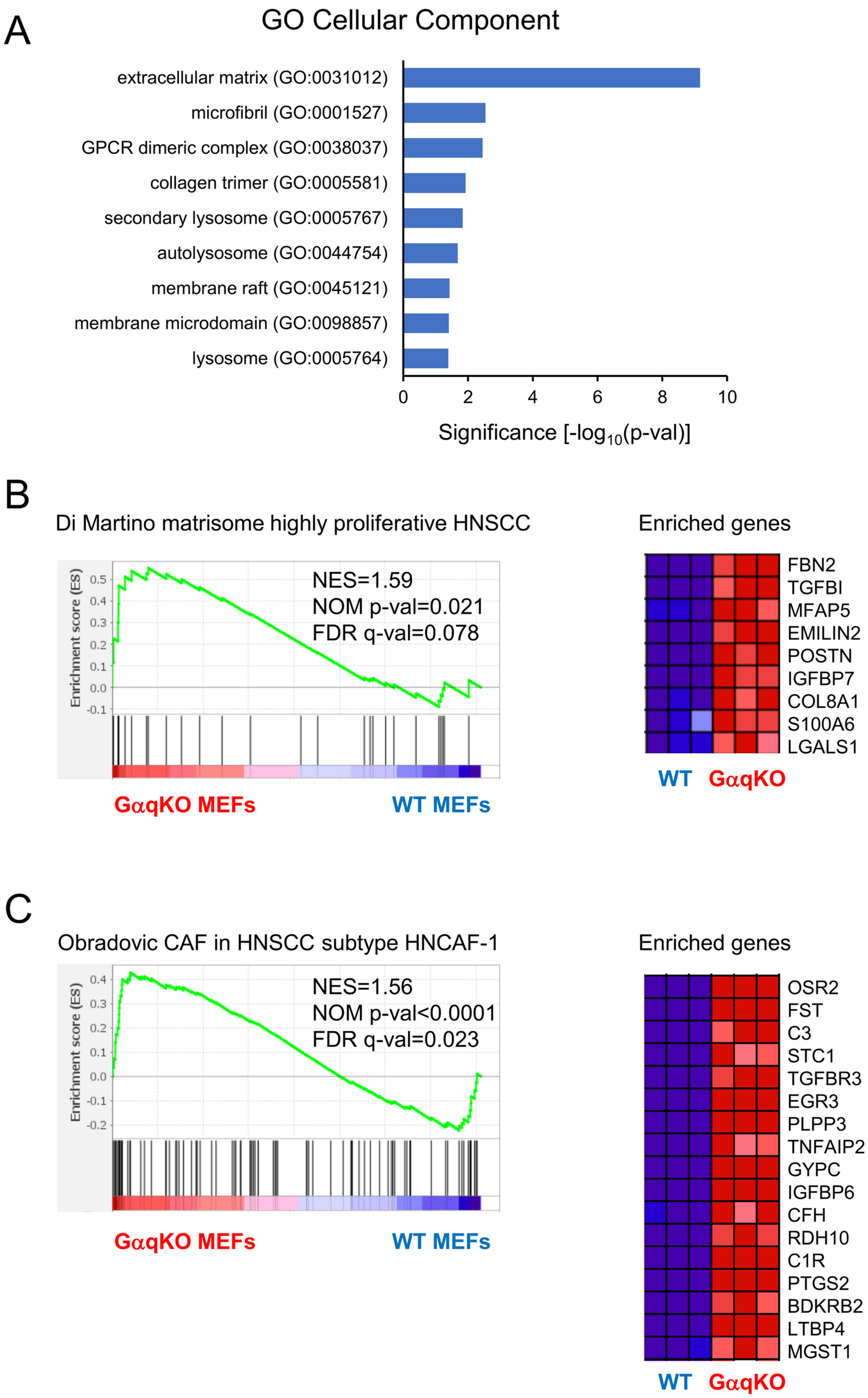
A
GO Cellular Component
extracellular matrix (GO:0031012)
microfibril (GO:0001527)
GPCR dimeric complex (GO:0038037)
collagen trimer (GO:0005581)
secondary lysosome (GO:0005767)
autolysosome (GO:0044754)
membrane raft (GO:0045121)
membrane microdomain (GO:0098857)
lysosome (GO:0005764)
0 2 4 6 8 10
Significance [-log10(p-val)]
B
Di Martino matrisome highly proliferative HNSCC
Enrichment score (ES)
NES=1.59
NOM p-val=0.021
FDR q-val=0.078
GαqKO MEFs
WT MEFs
Enriched genes
FBN2
TGFBI
MFAP5
EMILIN2
POSTN
IGFBP7
COL8A1
S100A6
LGALS1
WT GαqKO
C
Obradovic CAF in HNSCC subtype HNCAF-1
Enrichment score (ES)
NES=1.56
NOM p-val<0.0001
FDR q-val=0.023
GαqKO MEFs
WT MEFs
Enriched genes
OSR2
FST
C3
STC1
TGFBR3
EGR3
PLPP3
TNFAIP2
GYPC
IGFBP6
CFH
RDH10
C1R
PTGS2
BDKRB2
LTBP4
MGST1
WT GαqKO

© The Author(s) 

**Figure EV5. RNA-Seq profiling of WT and GαqKO MEFs.**

(**A**) Overexpressed genes in GαqKO versus WT MEFs showed overrepresentation of Gene Ontology cellular component terms related to extracellular matrix (ECM) organization and membrane microdomain remodeling, which validates our proteomic observations. Barplots represent the significance of the overrepresentation as -$\log_{10}$(*p* val) upon the hypergeometric distribution test. (**B**) GSEA analysis from GαqKO MEFs showed enrichment with a matrisome signature of highly proliferative HNSCC (Data ref: Di Martino et al, 2021a; Di Martino et al, 2021b). (**C**) GSEA analysis from GαqKO MEFs showed enrichment with a signature of the HNCAF-1 population of CAFs obtained from HNSCC, which is defined by an immunostimulatory phenotype and enriched in extracellular matrix organization and immune-related pathways (Obradovic et al, 2022). Enriched genes in (**B**, **C**) are shown on the right of each panel. See M&M sections for further details. Source data are available online for this figure.

 © The Author(s)
