# Supplementary material for: Loss of Gαq reshapes fibroblast traits and drives tumor-stroma remodeling in oral cancer progression
Source: EMBO Rep. 2026 Apr 10;27(10):2639–74. doi: 10.1038/s44319-026-00751-2 (PMC13219523; doi:10.1038/s44319-026-00751-2)
Supplement: Supplementary file 1 — Peer Review File [file 44319_2026_751_MOESM1_ESM.pdf]

EMBO reports

Loss of Gαq reshapes fibroblast traits and drives tumor-stroma remodeling in oral cancer progression

Catalina Ribas, Inmaculada Navarro-Lérida, Raquel Huertas-Lárez, Gemma Paredes-García, Dafne García-Mateos, María Jiménez-López, Llara Prieto-Fernández, Ania Pascual, Virginia Ávila-Oca, Juan Antonio López, Miguel Del Pozo, Saúl Álvarez-Teijeiro, Juana García-Pedrero, Ramón García-Escudero, and Federico Mayor Jr.

Corresponding author(s): Catalina Ribas (cribas@cbm.csic.es) , Federico Mayor Jr. (fmayor@cbm.csic.es), Inmaculada Navarro-Lérida (inmaculada.navarro@uam.es)

Review Timeline:

Submission Date:	21st May 25
Editorial Decision:	24th Jun 25
Revision Received:	19th Dec 25
Editorial Decision:	27th Jan 26
Revision Received:	18th Feb 26
Accepted:	10th Mar 26

Editor: Achim Breiling

Transaction Report:

(Note: With the exception of the correction of typographical or spelling errors that could be a source of ambiguity, letters and reports are not edited. Depending on transfer agreements, referee reports obtained elsewhere may or may not be included in this compilation. Referee reports are anonymous unless the Referee chooses to sign their reports.)

1st Editorial Decision **24th Jun 2025**

Dear Dr. Ribas,

Thank you for the transfer of your manuscript to EMBO reports. I have now received the reports from the three referees that were asked to evaluate your study, which can be found at the end of this email.

As you will see, the referees think that these findings are of interest. However, they have several comments, concerns, and suggestions, indicating that a major revision of the manuscript is necessary to allow publication of the study in EMBO reports. As the reports are below, and all the referee concerns need to be addressed, I will not detail them here. It will however be central to address the concerns regarding the biological relevance and clinical significance of the work, as e.g. detailed by referee #2.

Given the constructive referee comments, I would like to invite you to revise your manuscript with the understanding that the concerns of the referees must be addressed in the revised manuscript and/or in a detailed point-by-point response. Acceptance of your manuscript will depend on a positive outcome of a second round of review. It is EMBO reports policy to allow a single round of revision only and acceptance of the manuscript will therefore depend on the completeness of your responses included in the next, final version of the manuscript.

Revised manuscripts should be submitted within three months of a request for revision. Please contact me to discuss the revision (also by video chat) if you have questions or comments regarding the revision, or should you need additional time.

When submitting your revised manuscript, please also carefully review the instructions that follow below.

PLEASE NOTE THAT upon resubmission revised manuscripts are subjected to an initial quality control prior to exposition to re-review. Upon failure in the initial quality control, the manuscripts are sent back to the authors, which may lead to delays. Frequent reasons for such a failure are the lack of the data availability section (please see below) and the presence of statistics based on n=2 (the authors are then asked to present scatter plots or provide more data points).

When submitting your revised manuscript, we will require:

1) a .docx formatted version of the final manuscript text (including legends for main figures, EV figures and tables), but without the figures included. Figure legends should be compiled at the end of the manuscript text.

2) individual production quality figure files as .eps, .tif, .jpg (one file per figure), of main figures and EV figures. Please upload these as separate, individual files upon re-submission.

The Expanded View format, which will be displayed in the main HTML of the paper in a collapsible format, has replaced the Supplementary information. You can submit up to 5 images as Expanded View. Please follow the nomenclature Figure EV1, Figure EV2 etc. The figure legend for these should be included in the main manuscript document file in a section called Expanded View Figure Legends after the main Figure Legends section. Additional Supplementary material should be supplied as a single pdf file labeled Appendix. The Appendix should have page numbers and needs to include a table of content on the first page (with page numbers) and legends for all content. Please follow the nomenclature Appendix Figure Sx, Appendix Table Sx etc. throughout the text, and also label the figures and tables according to this nomenclature.

For more details, please refer to our guide to authors:
http://www.embopress.org/page/journal/14693178/authorguide#manuscriptpreparation

Please consult our guide for figure preparation:
http://wol-prod-cdn.literatumonline.com/pb-assets/embo-site/EMBOPress_Figure_Guidelines_061115-1561436025777.pdf

See also the guidelines for figure legend preparation:
https://www.embopress.org/page/journal/14693178/authorguide#figureformat

3) a .docx formatted letter INCLUDING the reviewers' reports and your detailed point-by-point responses to their comments. As part of the EMBO Press transparent editorial process, the point-by-point response is part of the Review Process File (RPF), which will be published alongside your paper.

4) a complete author checklist, which you can download from our author guidelines (https://www.embopress.org/page/journal/14693178/authorguide). Please insert page numbers in the checklist to indicate where the requested information can be found in the manuscript. The completed author checklist will also be part of the RPF.

Please also follow our guidelines for the use of living organisms, and the respective reporting guidelines:
http://www.embopress.org/page/journal/14693178/authorguide#livingorganisms

5) that primary datasets produced in this study (e.g. RNA-seq, ChIP-seq, structural and array data) are deposited in an appropriate public database. If no primary datasets have been deposited, please also state this in a dedicated section (e.g. 'No primary datasets have been generated and deposited'), see below.

See also: http://embor.embopress.org/authorguide#datadeposition

Please remember to provide a reviewer password if the datasets are not yet public.

The accession numbers and database should be listed in a formal "Data Availability" section that follows the model below. This is now mandatory (like the COI statement). Please note that the Data Availability Section is restricted to new primary data that are part of this study. This section is mandatory. As indicated above, if no primary datasets have been deposited, please state this in this section

Data availability

The datasets produced in this study are available in the following databases:

- RNA-Seq data: Gene Expression Omnibus GSE46843 (https://www.ncbi.nlm.nih.gov/geo/query/acc.cgi?acc=GSE46843)
- [data type]: [name of the resource] [accession number/identifier/doi] ([URL or identifiers.org/DATABASE:ACCESSION])

*** Note - All links should resolve to a page where the data can be accessed. ***

Moreover, I have these editorial requests:

6) We now request the publication of original source data with the aim of making primary data more accessible and transparent to the reader. You will receive a separate email with instructions for providing source data with your revised manuscript, including information how to upload and organize the files.

7) Our journal encourages inclusion of *data citations in the reference list* to directly cite datasets that were re-used and obtained from public databases. Data citations in the article text are distinct from normal bibliographical citations and should directly link to the database records from which the data can be accessed. In the main text, data citations are formatted as follows: "Data ref: Smith et al, 2001" or "Data ref: NCBI Sequence Read Archive PRJNA342805, 2017". In the Reference list, data citations must be labeled with "[DATASET]". A data reference must provide the database name, accession number/identifiers and a resolvable link to the landing page from which the data can be accessed at the end of the reference. Further instructions are available at: http://www.embopress.org/page/journal/14693178/authorguide#referencesformat

8) Regarding data quantification and statistics, please make sure that the number "n" for how many independent experiments were performed, their nature (biological versus technical replicates), the bars and error bars (e.g. SEM, SD) and the test used to calculate p-values is indicated in the respective figure legends (also for EV and Appendix figures). Please also check that all the p-values are explained in the legend, and that these fit to those shown in the figure. Please provide statistical testing where applicable. Please avoid the phrase 'independent experiment', but clearly state if these were biological or technical replicates. Please also indicate (e.g. with n.s.) if testing was performed, but the differences are not significant. In case n=2, please show the data as separate datapoints without error bars and statistics. See also: http://www.embopress.org/page/journal/14693178/authorguide#statisticalanalysis

If n<5, please show single datapoints for diagrams.

9) Please add scale bars of similar style and thickness to microscopic images, using clearly visible black or white bars (depending on the background). Please place these in the lower right corner of the images themselves. Please do not write on or near the bars in the image but define the size in the respective figure legend.

10) Please also note our reference format:
http://www.embopress.org/page/journal/14693178/authorguide#referencesformat

11) We updated our journal's competing interests policy in January 2022 and request authors to consider both actual and perceived competing interests. Please review the policy https://www.embopress.org/competing-interests and update your competing interests if necessary. Please name this section 'Disclosure and Competing Interests Statement' and put it after the Acknowledgements section.

12) We now use CRediT to specify the contributions of each author in the journal submission system. CRediT replaces the author contribution section. Please use the free text box to provide more detailed descriptions and do NOT provide your final manuscript text file with an author contributions section. See also our guide to authors:
https://www.embopress.org/page/journal/14693178/authorguide#authorshipguidelines

13) All Materials and Methods need to be described in the main text using our 'Structured Methods' format, which is required for all research articles. According to this format, the Methods section should include a Reagents and Tools Table (listing key reagents, experimental models, software, and relevant equipment and including their sources and relevant identifiers), uploaded as separate file, and a Methods section in which we encourage the authors to describe their methods using a step-by-step protocol format with bullet points, to facilitate the adoption of the methodologies across labs. More information on how to adhere to this format as well as downloadable templates (.doc) for the Reagents and Tools Table can be found in our author guidelines (section 'Structured Methods'):

https://www.embopress.org/page/journal/14693178/authorguide#manuscriptpreparation

14) Please order the manuscript sections like this, using these names:
Title page - Abstract - Keywords - Introduction - Results - Discussion - Methods - Data availability section - Acknowledgements - Disclosure and Competing Interests Statement - References - Figure legends - Expanded View Figure legends

15) Please make sure that all the funding information is also entered into the online submission system and that it is complete and similar to the one in the acknowledgement section of the manuscript text file.

Please note that all corresponding authors are required to supply an ORCID ID for their name upon submission of a revised manuscript. Please find instructions on how to link the ORCID ID to the account in our manuscript tracking system in our Author guidelines: http://www.embopress.org/page/journal/14693178/authorguide#authorshipguidelines

We would also welcome the submission of cover suggestions, or motifs to be used by our Graphics Illustrator in designing a cover.

As part of the EMBO publication's Transparent Editorial Process, EMBO reports publishes online a Review Process File (RPF) to accompany accepted manuscripts. This File will be published in conjunction with your paper and will include the referee reports, your point-by-point response and all pertinent correspondence relating to the manuscript.

You are able to opt out of this by letting the editorial office know (emboreports@embo.org). If you do opt out, the Review Process File link will point to the following statement: "No Review Process File is available with this article, as the authors have chosen not to make the review process public in this case."

I look forward to seeing a revised version of your manuscript when it is ready. Please let me know if you have questions or comments regarding the revision.

Yours sincerely,

Achim Breiling
Senior Editor
EMBO Reports

Referee #1:

This study provides evidence that Gαq loss in mouse fibroblasts promotes cancer-associated fibroblast-like activation, enhancing head and neck squamous cell carcinoma (HNSCC) progression through extracellular matrix remodeling and exosome-mediated growth factor receptor release.
The research employs a comprehensive experimental design combining in vitro 2D/3D co-culture systems with in vivo orthotopic allograft models, strengthening the reliability and translational potential of the findings. High-throughput proteomic analysis reveals novel insights into altered endosomal/lysosomal systems and PDGFR trafficking defects in GαqKO fibroblasts, representing a significant advancement in CAF biology understanding. A key discovery is the identification of exosome-mediated PDGFR release as a compensatory mechanism for impaired lysosomal degradation, with potential prognostic implications. A significant weakness is the use of mouse embryonic fibroblasts (MEFs) as a surrogate for tumor-associated CAFs, which brings into question the overall clinical significance of the results. This is a special concern given the very limited analysis of clinical samples. In the absence of further experimentation, this limitation should be clearly acknowledged in both abstract and discussion.

Specific points:

1) A fundamental concern lies in the use of mouse embryonic fibroblasts (MEFs) as a surrogate for tumor-associated CAFs. This model may not accurately represent the complex biology of human CAFs in the tumor microenvironment. Furthermore, the

interspecies mixing of mouse fibroblasts with human cancer cells may limit the translational validity of the stromal-tumor interactions observed.

2) The only figure using patient-derived CAFs, Figure 7, provides limited validation with only descriptive immunofluorescence for PDGFR, α-SMA, and fibronectin/collagen I (panels C-E), and no quantification across multiple strains is offered. Notably, there is no significant difference found in Gαq protein levels between normal fibroblasts (NFs) and CAFs in human samples, as shown by Western blot (panel B) and PCR (panel A, $p = 0.06$)-undermining the central hypothesis of the manuscript.

3) The bioluminescence imaging of admixed Cal27 cells with NFs/CAFs lacks a luminescence scale and quantification, rendering the data uninterpretable. Only two animals per condition are shown, with no statistical analysis provided, raising concerns about the reliability and reproducibility of these findings.

4) The rationale for serum starvation (Figure 1B) as a stimulus for CAF activation in Gαq-deficient MEFs remains unclear, particularly in the absence of growth factors and Gαq signaling. No mechanistic explanation is provided. Additionally, fluorescence images often lack scale bars and quantification, and it is unclear how serum starvation affects human dermal fibroblasts (HDFs), which are not evaluated in this context.

5) The PDGFR blot appears to lack signal in GαqKO samples, but the lanes are visibly spliced. For transparency and proper interpretation, full blots with corresponding WT controls on the same membrane should be shown.

6) Areas for further investigation include limited exploration of Gαq reconstitution effects and, more importantly, the variability in human CAF samples. The specific mechanisms linking Gαq deficiency to exosome biogenesis, including roles of Rab proteins and lipid alterations, require additional characterization for complete mechanistic understanding.

Referee #2:

Previous studies have demonstrated that Gαq plays a crucial role in controlling autophagy, and that autophagic and exosomal pathways are interconnected. Both cellular processes depend on the regulation of the endolysosomal system and rely on the convergence of degradative and secretory regulators. This manuscript presents evidence that Gαq limits exosome release through autophagy-dependent secretion mechanisms. In support of the model, Gαq KO cells exhibit accumulation of PDGFR and caveolin in intraluminal vesicles, multi-vesicular bodies, and ultimately to exosomes. Gαq KO also exhibited matrix remodeling and contractile features, resembling those seen in malignant cancer-associated fibroblasts. Injection of GαqKO MEFs to immunodeficient mice led to tumor growth, stromal remodeling, and increased tumor aggressiveness, similar to that seen in human head and neck cancers. The authors conclude that loss of Gq leads to exosome release, and this release facilitates the secretion of tumor growth factors otherwise unable to undergo proper lysosomal degradation, leading to tumor growth.

One major concern is the lack of mechanistic basis for the observed effects. Deleting a G protein has profound effects on cell morphology and protein abundance. How does this occur?

Another major concern is the lack of biological relevance. Deleting a G protein accelerates tumor progression. When does this occur?

There is no evidence that Gαq is absent in tumors. To the contrary, there is strong evidence that hyperactive (GTPase deficient) mutants are oncogenic. The difference between loss-of-function and gain-of-function mutants is not clearly acknowledged by the authors ("Within this system, mutated versions of the Gαq protein are present in different tumor types, particularly in uveal and cutaneous melanomas"). Thus, the work is quite thorough and well done, but it is not clear that have learned anything new about Gq function or how perturbations to Gq contribute to any disease. The mechanisms underlying the specific sorting of certain growth factor receptors to exosomes and the crosstalk between secretory and autophagy pathways in pathological contexts remain unclear. At best, this paper describes a tool that can be used to show that loss of autophagy contributes to the production of exosomes.

MINOR

YM254890 is a rapid and selective inhibitor of Gq/11 and as such represents an important tool to validate the findings. It is only used to validate one experiment in Fig. 1D. and should be used throughout as is done with the autophagy inhibitor chloroquine.

Many of the images lack scale bars

Fig.8 Degradative is misspelled

Referee #3:

Navarro-Lerida et al., indicated that GaqKOMEFs have an ability to promote growth and invasion of HNSCC, when cocultured on culture dishes and/or coinjected with carcinoma cells into immunodeficient mice. Gaq loss in fibroblasts induces the iCAF state. Stromal Gaq loss also induces accumulation of PDGFR presumably through impairment of PDGFR trafficking and PDGFR-rich exosome production. This study interestingly demonstrated that Gaq inhibition in fibroblasts confers iCAF state and tumor-promoting ability. PDGFR was highlighted to be upregulated in GaqKOMEFs and CAFs, however roles of PDGFR on iCAF state and tumor-promoting ability of these fibroblasts remain to be elucidated. There are several issues to be resolved prior to the publication by the following.

Major critiques,
1. How do GaqKOMEFs promote tumor growth and invasion? Molecular mechanism(s) underlying tumor-promoting ability of GaqKOMEFs remain(s) unclear. Does inhibition of PDGFR expression on these fibroblasts attenuate tumor growth and invasion? The molecular mechanism (s) should be elucidated in the paper.
2. Is Gaq staining on fibroblasts also attenuated in tumor-associated stroma in HNSCC patients?
Is the Gaq staining intensity associated with clinical outcome in the patients? The clinical correlation of Gaq attenuation in stromal fibroblasts should be provided.
3. The author indicated that GaqKOMEFs promote tumor invasion. How do carcinoma cells increase their ability to invade. Mechanistic insight including partial EMT induced in tumor cells should be provided.
4. In Figure 6D, increased invasiveness of tumor cells is shown in Cal27 tumor admixed with GaqKOMEFs. To formally conclude that GaqKOMEFs stimulate tumor invasiveness in vivo, invasiveness would be quantified, for example, by counting the number of tumor budding. Whether coinjection with GaqKOMEFs boosts metastasis of carcinoma cells into distant organs should also be examined.
5. Two different PDGFRs, such as alpha and beta forms are known. However, the authors mentioned just as PDGFR without discriminating alpha and beta forms in the text as well as figures. Which forms of PDGFRs are increased on GaqKOMEFs?
6. How does Gaq expression go down in CAFs? The mechanistic insight should be mentioned in Discussion.
7. Once PDGFR is transmitted to cancer cells from CAFs via exosomes, how does PDGFR act to activate their downstream signal? The mechanistic insight should also be mentioned in Discussion.

Minor issues,
1. The authors described "Gaq" as "Gq" in all figures but not in the text and figure legends.
2. Description as to GqKO should be stated in the text and figure legends, as appropriate.

MS# EMBOR-2025-61979-T

Manuscript Title: "Loss of Gαq reshapes fibroblast traits and drives tumor-stroma remodeling in oral cancer progression"

POINT BY POINT RESPONSES TO REVIEWERS

REVIEWER´S COMMENT

Reviewer: 1

Comments to the Author

This study provides evidence that Gαq loss in mouse fibroblasts promotes cancer-associated fibroblast-like activation, enhancing head and neck squamous cell carcinoma (HNSCC) progression through extracellular matrix remodeling and exosome-mediated growth factor receptor release.
The research employs a comprehensive experimental design combining in vitro 2D/3D co-culture systems with in vivo orthotopic allograft models, strengthening the reliability and translational potential of the findings. High-throughput proteomic analysis reveals novel insights into altered endosomal/lysosomal systems and PDGFR trafficking defects in GαqKO fibroblasts, representing a significant advancement in CAF biology understanding. A key discovery is the identification of exosome-mediated PDGFR release as a compensatory mechanism for impaired lysosomal degradation, with potential prognostic implications. A significant weakness is the use of mouse embryonic fibroblasts (MEFs) as a surrogate for tumor-associated CAFs, which brings into question the overall clinical significance of the results. This is a special concern given the very limited analysis of clinical samples. In the absence of further experimentation, this limitation should be clearly acknowledged in both abstract and discussion.

We sincerely thank Reviewer 1 for the thoughtful and positive evaluation of our work and for the insightful feedback, which has greatly contributed to strengthening the revised version of our work.

We recognize the reviewer for raising the concern regarding the use of mouse embryonic fibroblasts (MEFs) as the only surrogate for Cancer-Associated Fibroblasts (CAFs). Although MEFs do not entirely recapitulate the complexity and cellular heterogeneity of bona fide CAFs, particularly in studies with direct clinical relevance, they remain a robust, widely used and experimentally accepted model. Their homogeneity, reproducibility, and ease of genetic and experimental manipulation make them particularly suitable for mechanistic studies and -omics analyses, as it is the case of our work.

Our aim was to elucidate the role of Gαq expression in fibroblasts using MEFs, as an initial model. This strategy allowed us to characterize the influence of Gαq on fibroblast behavior and to identify phenotypic traits reminiscent of CAFs that may contribute to the progression of head and neck squamous cell carcinoma (HNSCC). Nevertheless, **in the revised manuscript we have substantially expanded our experimental evidence to address the reviewer´s concern** and strengthen the translational relevance of our findings.

First, we now include an extensive and deeper characterization of **the effect of downregulating Gαq expression levels in normal human fibroblasts** (NF) by using a short hairpin RNA lentiviral system (NFshGαq) **(see new Figure 7A-**

F panels and Figure EV4A-F in the revised version). In the original submission, only limited data on lysosomal organization and fibronectin distribution were provided comparing NFshGαq fibroblasts to their NFsh control counterparts (former Figure S7). In the revised version, we have incorporated a full set of functional *in vitro* and *in vivo* assays. We show that **human NFs with decreased Gαq levels display the same signatures previously observed in GαqKO MEFs**: increased matrix deposition, upregulation of caveolar components and lysosomal markers, increased intracellular accumulation of PDGFR, GM1 and ceramide. Gαq-deficient human fibroblasts also markedly altered the architecture, proliferation and invasion potential of co-cultured Cal27 cells, and exosomes secreted by these fibroblasts promote in these oral cancer cells effects similar to those observed with GαqKO MEFs-derived exosomes. Finally, animals co-injected with Gαq-downregulated NFs also displayed increased tumor growth *in vivo*.

Overall, these data indicate that Gαq loss in both MEFs and human fibroblasts closely resemble features of HNSCC-derived CAFs, reinforcing stromal Gαq as a key modulator of HNSCC progression.

Second, to further substantiate this point, we now provide RNA-seq analysis comparing wild-type (WT) and GαqKO MEFs. These transcriptomic data validate our proteomic observations, revealing significant enrichment of pathways related to extracellular matrix (ECM) remodelling and membrane microdomain reorganization in Gαq-deficient fibroblasts **(see new Figure EV5A)**. Moreover, extended comparative analysis demonstrate that GαqKO MEFs share a strong transcriptional overlap with CAFs isolated from HNSCC patients. We now show in new Figures **EV5B and EV5C** that GαqKO MEFs share similarity to the "matrisome" profiles of highly aggressive HNSCC tumors and that they exhibit significant enrichment for the HNCAF-1 transcriptional program described by Obradovic A, et al. *Clin Cancer Res* (2022), a CAF population associated with poor overall survival in HNSCC and characterized by pro-inflammatory and immunomodulatory functions. These observations further suggests that Gαq-deficient fibroblasts are likely involved in modulating inflammatory/immunological responses within the tumor microenvironment, one of the critical hallmarks of functionally active CAFs.

Figure for referee with unpublished data and its description has been removed upon request by the authors.

Overall, in accordance with the reviewer's recommendation, we have revised the text and discussion to incorporate the new findings and acknowledge the limitations associated with using MEFs. We are aware that our work represents an initial step toward clinical translation. Further analyses involving clinical samples will be the focus of a future manuscript.

SPECIFIC POINTS

A detailed point-by-point response to all specific points raised by the reviewer is provided below:

1) A fundamental concern lies in the use of mouse embryonic fibroblasts (MEFs) as a surrogate for tumor-associated CAFs. This model may not accurately represent the complex biology of human CAFs in the tumor microenvironment. Furthermore, the interspecies mixing of mouse fibroblasts with human cancer cells may limit the translational validity of the stromal-tumor interactions observed.

As detailed above, we have now performed all key experiments previously performed with GαqKO MEFs using human fibroblasts with targeted downregulation of Gαq (see **new Figure 7 and Figure EV4**). The fact that human Gαq-deficient fibroblasts exhibit nearly identical behaviour compared to GαqKO MEFs in both *in vitro* and *in vivo* analysis supports the occurrence of conserved mechanisms and the translational applicability and relevance of our conclusions regarding the stromal-tumor interactions, addressing the raised question regarding interspecies differences.

Also detailed above, the new comparative analysis of RNAseq data with those obtained in patient-derived CAFs further support the critical role of Gαq in modulating the stromal compartment in HNSCC.

2) The only figure using patient-derived CAFs, Figure 7, provides limited validation with only descriptive immunofluorescence for PDGFR, α-SMA, and fibronectin/collagen I (panels C-E), and no quantification across multiple strains is offered. Notably, there is no significant difference found in Gαq protein levels between normal fibroblasts (NFs) and CAFs in human samples, as shown by Western blot (panel B) and PCR (panel A, p = 0.06)-undermining the central hypothesis of the manuscript.

The role of Gαq signaling networks in stromal fibroblasts remains largely unexplored, and our study represents one of the first comprehensive efforts to characterize its molecular and functional impact in the tumor microenvironment.

As indicated by the reviewer, we have initially characterized several key features of patient-derived CAFs (Figs. 7G-I and EV4G-H in the new version). Regarding Gαq protein levels in patient-derived CAFs, our data show variability between sample pairs, and a moderate but consistent downregulation (circa 50% in average) of Gαq expression in the CAFs compared to NFs obtained simultaneously from healthy tissue of the same patients (Fig. 7G in the new version).

Notably, as detailed above, in the new set of experiments using normal human fibroblasts with reduced Gαq expression (new Figure 7A-F panels and Figures EV4 A-F), we find that a partial reduction of Gαq protein levels (~60%) in human fibroblasts is sufficient to elicit robust CAF-like properties, comparable to those resulting from complete genetic deletion in GαqKO MEFs.

This supports the idea that even modest quantitative changes in Gαq expression may have significant functional consequences, consistent with what has been described for other regulatory proteins such as PTEN, among others, where limited reductions result in major pathological effects (10.1158/0008-5472.CAN-10-2488). Although Gαq is not abundantly expressed in fibroblasts under basal conditions, it plays a central role as a key signaling hub coordinating multiple processes, including those regulating cytoskeletal dynamics, vesicular trafficking, and metabolic regulation. Due to this integrative role, it is likely that **even small decreases in Gαq expression levels** (and/or changes in stimuli modulating such Gq networks) **can disrupt critical pathways and lead to pronounced phenotypic alterations**. This might be particularly relevant in the tumor microenvironment, where such changes may

enhance fibroblast activation and promote a pro-tumoral state amplified via paracrine/secretory pathways.

Finally, we also acknowledge the heterogeneity of patient-derived CAFs, which requires a more exhaustive analysis that will be addressed in a future work. While additional quantification of the different markers across CAF strains is not included in this manuscript, these samples are currently being analyzed in more detail as part of a forthcoming more clinically oriented study.

3) The bioluminescence imaging of admixed Cal27 cells with NFs/CAFs lacks a luminescence scale and quantification, rendering the data uninterpretable. Only two animals per condition are shown, with no statistical analysis provided, raising concerns about the reliability and reproducibility of these findings.

We thank the reviewer for this helpful comment. The images originally shown in the previous version were intended as representative examples of the observed results. As requested, we have now incorporated the bioluminescence intensity scale into Figure 7I to ensure proper interpretation of the imaging data. We also clarify that study was conducted using 5 animals in the normal fibroblasts (NF) group and 7 animals in the CAF group. To strengthen the analysis, we have now included quantification of tumour size measurements **(see graph in new Fig. 7I),** consistent with enhanced tumour burden in the presence of CAFs vs NFs.

4) The rationale for serum starvation (Figure 1B) as a stimulus for CAF activation in Gαq-deficient MEFs remains unclear, particularly in the absence of growth factors and Gαq signaling. No mechanistic explanation is provided. Additionally, fluorescence images often lack scale bars and quantification, and it is unclear how serum starvation affects human dermal fibroblasts (HDFs), which are not evaluated in this context.

We thank the reviewer for this insightful observation. In our study, we implemented nutrient deprivation for up to 48 hours to more accurately replicate the nutrient-limited conditions of the tumor microenvironment and to evaluate the impact of Gαq deficiency under physiologically relevant stress. Importantly, nutrient deprivation significantly alters endocytic, degradative, and secretory pathways in fibroblasts, processes closely linked to Gαq-regulated autophagy and signaling (10.1083/jcb.201711002; 10.1016/j.devcel.2017.03.015; 10.1111/boc.201300063; 10.1016/j.celrep.2025.115896). In solid tumors, poor vascularization limits the availability of nutrients and growth factors, selecting for metabolically adaptable fibroblasts with CAF-like characteristics. In line with this, GαqKO MEFs exhibited enhanced survival compared to WT MEFs nutrient limitation, suggesting that loss of Gαq confers a metabolic adaptation that may contribute to their protumoral phenotype.

Notably, serum starvation (commonly used to induce a quiescent yet metabolically active state and to standardize collagen contraction assays) induced in WT MEFs several phenotypic changes that resemble those observed in GαqKO MEFs under basal conditions, including comparable PDGFR distribution and α-SMA expression patterns **(see figure below for your**

consideration). This suggests that the absence of Gαq partially mimics the cellular state induced by metabolic stress.

Together, these results underscore that serum starvation is both biologically relevant and mechanistically informative within our experimental design, Specifically, as demonstrated, GαqKO cells exhibit enhanced survival under such stressful conditions, highlighting that Gαq deficiency promotes metabolic reprogramming similar to the adaptation of fibroblasts in nutrient-deprived tumor microenvironments.

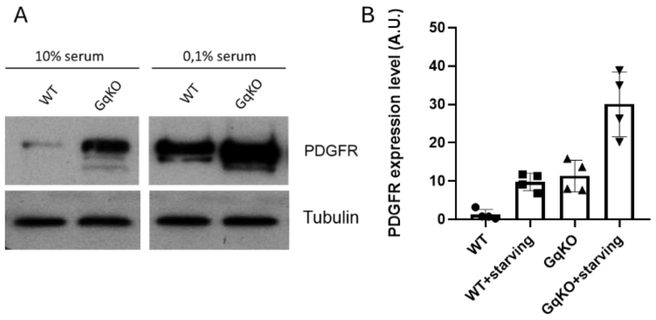

Figure for reviewer: A) PDGFR protein expression in wild-type and GαqKO MEFs under basal (10% serum) and serum-starved (0.1% serum) conditions. B) The graph depicts PDGFR levels normalized to the tubulin loading control (mean ± SD, n=4).

Regarding labelling of fluorescence images, we have now incorporated scale bars in all panels in line with the comments of the reviewer.

5) The PDGFR blot appears to lack signal in GαqKO samples, but the lanes are visibly spliced. For transparency and proper interpretation, full blots with corresponding WT controls on the same membrane should be shown.

We thank the reviewer for this careful observation. The PDGFR blot shown in Figure 4B was assembled from membranes that also contained additional experimental conditions not directly relevant to the current manuscript. For this reason, the lanes included in the original blot were not loaded consecutively, and the splice was intentionally indicated to maintain transparency regarding the composition of the panel.

Furthermore, WT and GαqKO samples were performed and run on separate gels, which explains the discontinuity in the figure layout. To fully address the reviewer's concern and ensure complete transparency, we show here **(see figure below for your consideration)** a full, unedited Western blots, in which the relevant lanes corresponding to the conditions analyzed in the manuscript are clearly indicated. Additionally, we also provide the corresponding cropped shortcuts alongside the full blots to facilitate interpretation of the specific bands discussed.

We hope this explanation resolves any concerns regarding the integrity or interpretation of the PDGFR Western blot data in Figure 4B.

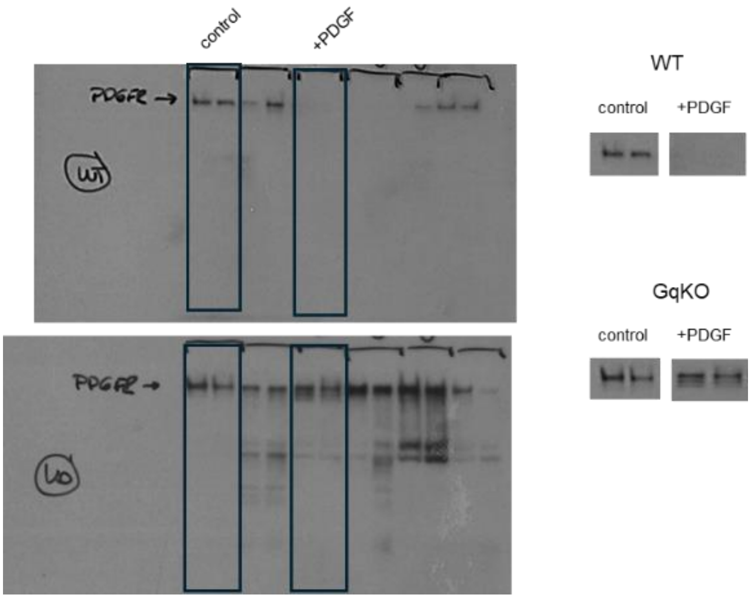

Figure for reviewer: Full scanned blots. Representative Western blots showing PDGFR expression in WT and Gαq-KO MEFs under basal conditions and following stimulation with PDGF (20 ng/mL). The complete, unedited blot image is provided for analysis.

6) Areas for further investigation include limited exploration of Gαq reconstitution effects and, more importantly, the variability in human CAF samples. The specific mechanisms linking Gαq deficiency to exosome biogenesis, including roles of Rab proteins and lipid alterations, require additional characterization for complete mechanistic understanding.

The point referring to the variability in human CAF samples has been discussed in point 2 above.

We thank the reviewer for highlighting the importance of further exploring the mechanisms underlying the altered degradative and enhanced secretory phenotypes observed in GαqKO MEFs. In the revised manuscript, we have expanded this section by integrating new data that support the potential role of lipid remodelling, specifically ceramide accumulation, as a key driver of these effects.

Loss of Gαq would alter signaling via its canonical effector PLCbeta in response to a variety of stimuli, thus likely unbalancing diacylglycerol (DAG) production. Given the functional and structural parallels between DAG and ceramide, we hypothesized that Gαq loss may trigger a compensatory increase in ceramide levels. Ceramide is a well-established regulator of the balance between degradation and secretion: it promotes exosome biogenesis through neutral sphingomyelinase (nSMase) activation, and its inhibition by GW4869 markedly reduces exosome release. Moreover, elevated ceramide can destabilize lysosomal membranes, alter intralysosomal pH, and hinder fusion with autophagosomes or multivesicular bodies, thereby favoring exosome secretion over degradation.

Using a ceramide-specific fluorescent probe we have **revealed a marked increase in ceramide levels in GαqKO MEFs** compared with WT MEFs, with prominent colocalization within LAMP1-positive lysosomal compartments **(see new Fig. 4E)**. Consistently, human fibroblasts with partial Gαq downregulation (NFs shGαq) also exhibited elevated intracellular ceramide (**new Fig. EV4C**), supporting the notion that loss of Gαq promotes ceramide accumulation and its relevance to the human stromal context.

Moreover, treatment of GαqKO MEFs with the **GW4869** nSMase1/2 inhibitor (known to prevent the conversion of sphingomyelin to ceramide and to diminish exosome release and modulate lysosomal and autophagic pathways), **markedly decreased the expression of the CAF-associated markers** Cav1 and PDGFR **(new Fig. 4F) and improved lysosomal organization and normalization of the Caveolin-1 plasma membrane pattern (new Fig. 4G).** These results indicate that reducing ceramide levels restores lysosomal integrity and partially reverts the phenotype toward that of WT cells, supporting the conclusion that ceramide accumulation is a key driver of the defects observed in Gαq-deficient fibroblasts.

In sum, our findings support a model in which ceramide accumulation in GαqKO MEFs, concurrent with changes in other key modulators of intracellular trafficking such as Caveolin1, would disrupt lysosomal homeostasis and redirects vesicular trafficking toward enhanced exosome release. The altered ECM environment fostered by Gαq-deficient fibroblasts, along with the presence in exosomes of PDGFR and other GF receptors, would then drive aberrant growth and invasion of oral cancer cells.

Although further experiments are underway to deepen our understanding of this mechanism, the new data included in the revised manuscript provide a strong mechanistic explanation for the defects associated with Gαq deficiency. Moreover, they also suggest that modulating sphingolipid metabolism may represent a potential therapeutic strategy in HNSCC.

Note for reviewer:

The data sets produced in this study are available in the following databases:

-Proteomics data: The mass-spectrometry proteomics datasets derived from lysates of WT and GαqKO MEFs, as well as their corresponding exosomes, have been deposited in the ProteomeXchange Consortium via the PRIDE partner repository (Perez-Riverol et al., 2025) under the accession number PXD061641.

Project accession: PXD061641

Project accession: PXD061641

Access token (for reviewers): oCvOMfCqXQL6

Alternatively, the dataset may be accessed by logging into the PRIDE repository using the credentials provided below.

Username: reviewer_pxd061641@ebi.ac.uk

Password: EruOFvQLSb8y

Accession of RNAseq:

-RNA-seq data: RNA-sequencing datasets generated from lysates of wild-type (WT) and GαqKO MEFs are available in the Gene Expression Omnibus (GEO) under accession GSE312614.

https://www.ncbi.nlm.nih.gov/geo/query/acc.cgi?acc=GSE312614

Access token: gxwnqsqklhejfcz

Reviewer: 2

Comments to the Author

MAJOR POINTS

1) Previous studies have demonstrated that Gαq plays a crucial role in controlling autophagy, and that autophagic and exosomal pathways are interconnected. Both cellular processes depend on the regulation of the endolysosomal system and rely on the convergence of degradative and secretory regulators. This manuscript presents evidence that Gαq limits exosome release through autophagy-dependent secretion mechanisms. In support of the model, Gαq KO cells exhibit accumulation of PDGFR and caveolin in intraluminal vesicles, multi-vesicular bodies, and ultimately to exosomes. Gαq KO also exhibited matrix remodeling and contractile features, resembling those seen in malignant cancer-associated fibroblasts. Injection of GαqKO MEFs to immunodeficient mice led to tumor growth, stromal remodeling, and increased tumor aggressiveness, similar to that seen in human head and neck cancers. The authors conclude that loss of Gαq leads to exosome release, and this release facilitates the secretion of tumor growth factors otherwise unable to undergo proper lysosomal degradation, leading to tumor growth.

We sincerely thank the reviewer for the time and effort dedicated to evaluating our manuscript. We highly appreciate the constructive comments and valuable suggestions, which allowed us to improve the clarity, quality, and overall impact of our study.

2) One major concern is the lack of mechanistic basis for the observed effects. Deleting a G protein has profound effects on cell morphology and protein abundance. How does this occur?

We thank the reviewer for this important and insightful comment. In the revised manuscript, we have expanded the mechanistic basis underlying the altered degradative and secretory phenotype observed in Gαq-deficient (GαqKO) MEFs.

As described now in detail in the revised Results section, we reasoned that loss of Gαq would alter signaling via its canonical effector PLCbeta in response to a variety of stimuli, thus likely unbalancing diacylglycerol (DAG) production. Given the functional and structural parallels between DAG and ceramide, we hypothesized that Gαq loss may trigger a compensatory increase in ceramide, a

well-established regulator of endolysosomal dynamics, exosome biogenesis, and vesicle fate decisions.

Using a ceramide-specific fluorescent probe we have **revealed a marked increase in ceramide levels in GαqKO MEFs** compared with WT MEFs, with prominent colocalization within LAMP1-positive lysosomal compartments **(see new Fig. 4E)**. Consistently, human fibroblasts with partial Gαq downregulation (NFs shGαq) also exhibited elevated intracellular ceramide (**new Fig. EV4C**), supporting the notion that loss of Gαq promotes ceramide accumulation and its relevance to the human stromal context.

Moreover, treatment of GαqKO MEFs with the **GW4869** neutral sphingomyelinase1/2 inhibitor (known to prevent the conversion of sphingomyelin to ceramide and to diminish exosome release and modulate lysosomal and autophagic pathways), **markedly decreased the expression of the CAF-associated markers** Cav1 and PDGFR **(new Fig. 4F) and improved lysosomal organization and normalization of the Caveolin-1 plasma membrane pattern (new Fig. 4G).** These results indicate that reducing ceramide levels restores lysosomal integrity and partially reverts the phenotype toward that of WT cells, supporting the conclusion that ceramide accumulation is a key driver of the defects observed in Gαq-deficient fibroblasts.

In sum, our findings support a model in which ceramide accumulation in GαqKO MEFs, concurrent with changes in other key modulators of intracellular trafficking such as Caveolin1, would disrupt lysosomal homeostasis and redirects vesicular trafficking toward enhanced exosome release. The altered ECM environment fostered by Gαq-deficient fibroblasts, along with the presence in exosomes of PDGFR and other GF receptors, would then drive aberrant growth and invasion of oral cancer cells.

3) Another major concern is the lack of biological relevance. Deleting a G protein accelerates tumor progression. When does this occur?

To date, most studies in the cancer field have focused on the role of gain-of-function mutations of GPCRs or G proteins. As for Gαq, gain of function of the GNAQ/GNA11 gene has been reported particularly in uveal melanoma, where constitutively active forms of Gαq are detected in up to 66% of cases, whereas there is currently no evidence that loss of Gαq in tumor cells promotes oncogenesis. The potential impact of Gαq depletion in tumor cells, while of great interest, lies beyond the scope of this manuscript.

The focus of our work has been the role of Gαq within the tumor stroma, and specifically in fibroblasts, which has remained largely unexplored. Our study addresses this gap by demonstrating that loss or reduction of Gαq in fibroblasts profoundly alters stromal behaviour, leading to impaired lysosomal degradation and enhanced exosome secretion, features highly relevant to cancer-associated fibroblast (CAF) function.

In the revised version of the manuscript, in response to the issue of biological relevance raised by you and by other reviewer, we have included additional data to further support the translational and clinical relevance of stromal Gαq in modulating CAF activity and, consequently, tumor progression.

First, we now include an extensive and deeper characterization of **the effect of downregulating Gαq expression levels in normal human fibroblasts** (NF) by using a short hairpin RNA lentiviral system (NFshGαq) **(see new Figure 7A-F panels and Figures EV4 A-E in the revised version).** In the original submission, only limited data on lysosomal organization and fibronectin distribution were provided comparing NFshGαq fibroblasts to their NFsh control counterparts (former Figure S7). In the revised version, we have incorporated a full set of functional in vitro and in vivo assays. We show that **human NFs with decreased Gαq levels display the same signatures previously observed in GαqKO MEFs**: increased matrix deposition, upregulation of caveolar components and lysosomal markers, increased intracellular accumulation of PDGFR, GM1 and ceramide. Gαq-deficient human fibroblasts also markedly altered the architecture, proliferation and invasion potential of co-cultured Cal27 cells, and exosomes secreted by these fibroblasts promote in these oral cancer cells effects similar to those observed with GαqKO MEFs-derived exosomes. Finally, animals co-injected with Gαq-downregulated NFs also displayed increased tumor growth in vivo.

Overall, these data indicate that Gαq loss in both MEFs and human fibroblasts closely resemble features of HNSCC-derived CAFs, reinforcing stromal Gαq as a key modulator of HNSCC progression.

Second, to further substantiate this point, we now provide RNA-seq analysis comparing wild-type (WT) and GαqKO MEFs. These transcriptomic data validate our proteomic observations, revealing significant enrichment of pathways related to extracellular matrix (ECM) remodelling and membrane microdomain reorganization in Gαq-deficient fibroblasts **(see new Figure EV5A)**. Moreover, extended comparative analysis demonstrate that GαqKO MEFs share a strong transcriptional overlap with CAFs isolated from HNSCC patients. We now show in new Figures **EV5B and EV5C** that GαqKO MEFs share similarity to the “matrisome” profiles of highly aggressive HNSCC tumors and that they exhibit significant enrichment for the HNCAF-1 transcriptional program described by Obradovic A.et al. *Clin Cancer Res* (2022), a CAF population associated with poor overall survival in HNSCC and characterized by pro-inflammatory and immunomodulatory functions. These observations further suggests that Gαq-deficient fibroblasts are likely involved in modulating inflammatory/immunological responses within the tumor microenvironment, one of the critical hallmarks of functionally active CAFs.

In sum, these data indicate that tight regulation of Gαq expression is key for maintaining stromal homeostasis and in controlling tumor microenvironment responses. Of note, we show that Gαq protein levels in patient-derived CAFs, even considering the variability between sample pairs, display a moderate but

consistent downregulation in the CAFs compared to NFs obtained simultaneously from healthy tissue of the same patients (Fig. 7G in the new version). Since we find that a partial reduction of Gαq protein levels (~60%) in human fibroblasts is sufficient to elicit robust CAF-like properties, even modest quantitative changes in Gαq expression may have significant functional consequences, consistent with its central role as a key signaling hub coordinating multiple processes, including those regulating cytoskeletal dynamics, vesicular trafficking, and metabolic regulation. Due to this integrative role, it is likely that **even small decreases in Gαq expression levels** (and/or changes in stimuli modulating such Gq networks) **can disrupt critical pathways and lead to pronounced phenotypic alterations**. This might be particularly relevant in the tumor microenvironment, where such changes may enhance fibroblast activation and promote a pro-tumoral state amplified via paracrine/secretory pathways.

MINOR POINTS

- YM254890 is a rapid and selective inhibitor of Gαq /11 and as such represents an important tool to validate the findings. It is only used to validate one experiment in Fig. 1D. and should be used throughout as is done with the autophagy inhibitor chloroquine.

We appreciate the reviewer's suggestion regarding the use of YM-254890 as a pharmacological tool to mimic Gαq loss-of-function. YM-254890 is indeed a well-characterized and selective inhibitor of Gαq/11, known to block GDP/GTP exchange, thereby preventing downstream PLCβ activation and the generation of DAG and IP3 (10.1101/2024.11.25.625299; 10.3389/fcvm.2015.00014).

In the revised manuscript, we have expanded the use of YM-254890 beyond the initial experiment in Figure 1D, where we reported an altered fibronectin deposition and lysosomal localization. Thus, as shown in the **new Figure EV1 E-G**, YM-254890 treatment in WT MEFs partially phenocopies the GαqKO phenotype, inducing CAF-like features such as increased PDGFR expression and intracellular Cav1 distribution. These results strongly suggests that inhibition of Gαq signaling contributes directly to the fibroblast alterations observed in the knockout model.

Furthermore, the partial overlap between YM-254890–treated cells and GαqKO MEFs is consistent with our hypothesis that inhibition of Gαq activity, and the consequent impairment of DAG production through this drug, may also lead to compensatory ceramide accumulation, as we have previously reported to occur in GαqKO MEFs, contributing to lysosomal dysfunction and enhanced secretory activity.

While YM-254890 inhibition provides valuable mechanistic insights into Gαq-mediated signaling, we acknowledge its inherent limitations. Gαq not only functions as a signaling molecule but also performs important scaffolding and regulatory functions interacting with multiple proteins that may not be fully

recapitulated by pharmacological inhibition. Additionally, the effects of YM-254890 are dose- and time-dependent, and prolonged treatment can lead to compensatory changes or off-target responses. These considerations underscore the need for careful interpretation of pharmacological data in comparison with our genetic Gαq-knockout model. Further experiments will be required to fully elucidate how acute versus chronic Gαq inhibition modulates CAF-like behaviour and tumor microenvironment remodelling.

- *Many of the images lack scale bars*

We thank the reviewer for noting the missing scale bars. These have now been appropriately included in all relevant figures in the revised manuscript.

- Fig.8 Degradative is misspelled

We thank the reviewer for pointing out the typographical error. The word “degradative” has now been corrected in the revised version of the manuscript.

Note for reviewer:

The data sets produced in this study are available in the following databases:

-Proteomics data: The mass-spectrometry proteomics datasets derived from lysates of WT and GαqKO MEFs, as well as their corresponding exosomes, have been deposited in the ProteomeXchange Consortium via the PRIDE partner repository (Perez-Riverol et al., 2025) under the accession number PXD061641.

Project accession: PXD061641

Project accession: PXD061641

Access token (for reviewers): oCvOMfCqXQL6

Alternatively, the dataset may be accessed by logging into the PRIDE repository using the credentials provided below.

Username: reviewer_pxd061641@ebi.ac.uk

Password: EruOFvQLSb8y

Accession of RNAseq:

-RNA-seq data: RNA-sequencing datasets generated from lysates of wild-type (WT) and GαqKO MEFs are available in the Gene Expression Omnibus (GEO) under accession GSE312614.

https://www.ncbi.nlm.nih.gov/geo/query/acc.cgi?acc=GSE312614

Access token: gxwnqsqklhejfcz

Reviewer: 3

Comments to the Author

Navarro-Lerida et al., indicated that GaqKOMEFs have an ability to promote growth and invasion of HNSCC, when cocultured on culture dishes and/or coinjected with carcinoma cells into immunodeficient mice. Gaq loss in fibroblasts induces the iCAF state. Stromal Gaq loss also induces accumulation of PDGFR presumably through impairment of PDGFR trafficking and PDGFR-rich exosome production. This study interestingly demonstrated that Gaq inhibition in fibroblasts confers iCAF state and tumor-promoting ability. PDGFR was highlighted to be upregulated in GaqKOMEFs and CAFs, however roles of PDGFR on iCAF state and tumor-promoting ability of these fibroblasts remain to be elucidated. There are several issues to be resolved prior to the publication by the following.

MAJOR POINTS

1) How do GaqKOMEFs promote tumor growth and invasion? Molecular mechanism(s) underlying tumor-promoting ability of GaqKOMEFs remain(s) unclear. Does inhibition of PDGFR expression on these fibroblasts attenuate tumor growth and invasion? The molecular mechanism (s) should be elucidated in the paper.

We sincerely thank Reviewer 3 for the overall positive evaluation and insightful comments.

In response to the first point raised, we have conducted a comprehensive series of new experiments and analyses to shed light on the molecular mechanisms underlying the tumor-promoting capacity of Gαq-deficient fibroblasts in head and neck squamous cell carcinoma (HNSCC). Our findings, supported by both new data and existing literature, reveal a multifaceted mechanism involving the enhanced metastatic potential and increased growth produced by GαqKO MEFs.

On the one hand, to strengthen the biological relevance of our findings (also addressing your point 4, see below), we performed new *in vivo* experiments in NSG mice co-injected with Cal27 tumor cells and either WT or GαqKO fibroblasts to address the lack of *in vivo* metastasis data in the original submission. We find that co-injection with GαqKO fibroblasts promotes a significantly increased Ki67 staining in primary tumors (**new Figure 6F**) and enhanced metastatic burden, particularly in the lungs, where 75% of Cal27/GαqKO mice developed metastases **(new Figure 6G)**. These results indicate that Gαq-deficient fibroblasts enhance both proliferation capacity and metastasic dissemination.

Mechanistically, our revised manuscript shows that:

a) Loss of Gαq triggers a lipid remodeling process characterized by **a marked increase in ceramide levels in GαqKO MEFs** compared with WT, with prominent colocalization within LAMP1-positive lysosomal compartments **(see new Fig. 4E)**. Ceramide, a central regulator of sphingolipid metabolism, promotes lysosomal exocytosis and exosome release, which may explain the increased secretory and altered degradative capacities of GαqKO MEFs. Moreover, ceramide can be converted into gangliosides such as GM1, which we found to be aberrantly enriched in Gαq KO cells (Figure 3D). Elevated GM1 disrupts lipid rafts and destabilizes caveolae, causing Caveolin-1 internalization and mislocalization (10.1242/jcs.01009, 10.1111/j.1600-0854.2009.01022.x) which could also partially explain the altered expression of most of caveolae components in our proteomic analysis (Figure 3C) and the increased internalization displayed by Cav1 in GαqKO fibroblasts (Figure. 3D). Consistently, treatment of GαqKO MEFs with the **GW4869** neutral sphingomyelinase1/2 inhibitor (known to prevent the conversion of sphingomyelin to ceramide and to diminish exosome release and modulate lysosomal and autophagic pathways), **markedly decreased the expression of the CAF-associated markers** Cav1 and PDGFR **(new Fig. 4F) and improved lysosomal organization and normalization of the caveolin-1 plasma membrane pattern (new Fig. 4G).** These results indicate that reducing ceramide levels restores lysosomal integrity and partially reverts the phenotype toward that of WT cells, supporting the conclusion that ceramide accumulation is a key driver of the membrane disorganization and hypersecretory fibroblast phenotype observed in Gαq-deficient fibroblasts.

It should also be noted that the revised manuscript now includes an extensive and deeper characterization of **the effect of downregulating Gαq expression levels in normal human fibroblasts** (NF) by using a short hairpin RNA lentiviral system (NFshGαq) **(see new Figure 7A-F panels and Figures EV4 A-F in the revised version).** In the original submission, only limited data on lysosomal organization and fibronectin distribution were provided comparing NFshGαq fibroblasts to their NFsh control counterparts (former Figure S7). In the revised version, we have incorporated a full set of functional *in vitro* and *in vivo* assays. We show that **human NFs with decreased Gαq levels display the same signatures previously observed in GαqKO MEFs**: increased matrix deposition, upregulation of caveolar components and lysosomal markers, increased intracellular accumulation of PDGFR, GM1 and ceramide. Gαq-deficient human fibroblasts also markedly altered the architecture, proliferation and invasion potential of co-cultured Cal27 cells, and exosomes secreted by these fibroblasts promote in these oral cancer cells effects similar to those observed with GαqKO MEFs-derived exosomes. Finally, animals co-injected with Gαq-downregulated NFs also displayed increased tumor growth *in vivo*.

In sum, our findings support a model in which ceramide accumulation upon Gαq downregulation in fibroblasts, concurrent with changes in other key modulators of intracellular trafficking such as Caveolin1, would disrupt lysosomal

homeostasis and redirects vesicular trafficking toward enhanced exosome release. The altered ECM environment fostered by Gαq-deficient fibroblasts, along with the presence in exosomes of PDGFR and other GF receptors, would then drive aberrant growth and invasion of oral cancer cells.

b) Regarding the specific role of altered PDGFR expression and trafficking in GαqKO MEFs on tumor growth and invasion, our data point that GαqKO fibroblasts redirect PDGFR trafficking from clathrin- to caveolae-mediated pathways, promoting its incorporation into exosomes, as supported by gradient fractionation and colocalization analyses showing PDGFR enrichment in caveolar fractions (Figures 3F–G).

Once released, GαqKO fibroblasts-derived exosomes efficiently transfer PDGFR to Cal27 cells, leading to a marked decrease in Cav1 expression and strong increase in Ki67 proliferation marker (**Figures 5E and 5F**). Similar effects were observed using exosomes from human Gαq-depleted fibroblasts (**new Figure 7F**), reinforcing the translational relevance of the mechanism.

Supporting a direct role for PDGFR in mediating the effects produced by exosomes derived from Gαq-deficient fibroblasts, PDGFRα or PDGFRβ overexpression in oral cancer Cal27 cells reduced Cav-1 and PTRF levels (**new Fig. 5G**). In contrast, pharmacological inhibition of PDGFR with AG1295 partially attenuated the proliferative response induced by GαqKO-derived exosomes, further validating PDGFR as a critical mediator of this signaling axis (**new Appendix Fig S2C**).

Of note, Caveolin-1 is a well-established negative regulator of ERK/MAPK signaling, and its loss has been linked to sustained ERK activation, cell proliferation and tumor growth (10.1128/MCB.00315-09, 10.1152/ajpcell.00380.2002,10.1152/ajpcell.00380.2002,0.1038/sj.onc.1205300).In addition, Cav-1 deficiency has been linked to epithelial–mesenchymal transition (EMT) and metastatic progression (10.15252/emmm.201404127, 10.1016/j.ajpath.2012.10.022, 10.1186/s12943-025-02469-6). Indeed, this mechanism appears to be operative in our model. Cal27 cells treated with GαqKO-derived exosomes (Fig. 5E-F) or their conditioned medium (Appendix Fig. S2B) or in co-culture with GαqKO fibroblasts (Figure 2C) exhibited features as persistent ERK activation, E-cadherin relocalization and increased vimentin expression, consistent with enhanced proliferative activity and the induction of an EMT-like phenotype.

In summary, our findings support a model in which Gαq loss in fibroblasts promotes tumor progression through lipid remodeling, caveolae destabilization, and exosome-mediated PDGFR transfer. This leads to Cav-1 downregulation, ERK pathway activation, and enhanced proliferation and metastatic potential on the tumoral cells resulting in an increased proliferative and invasive capacity. These results are consistent with and supported by multiple studies in the literature, and we believe they significantly strengthen the mechanistic foundation of our manuscript.

2a) Is Gaq staining on fibroblasts also attenuated in tumor-associated stroma in HNSCC patients?

We appreciate the reviewer's interest in this point. Unfortunately, reliable immunostaining of Gαq, even in wild-type (WT) fibroblasts, remains technically challenging. Although commercially available antibodies against Gαq perform reliably in Western blot assays, they do not provide specific signal for imaging-based applications (particularly in tissue), which limits their utility for spatial or diagnostic analyses. This limitation is particularly evident even when comparing WT fibroblasts to GαqKO cells, which lack Gαq expression as indicated by Western blot approaches. So, translating this into tumor-associated stroma of HNSCC is even more difficult.

Despite this limitation, our new set of experiments using normal human fibroblasts with reduced Gαq expression (**new Figure 7A-F panels and Figures EV4 A-F**, see above) provide strong evidence that **even a modest reduction in Gαq expression in human fibroblasts is sufficient to induce a phenotype that closely resembles that of GαqKO MEFs**, supporting the idea that even modest quantitative changes in Gαq expression may have significant functional consequences.

In addition, we now provide RNA-seq analysis comparing wild-type (WT) and Gαq KO MEFs. These transcriptomic data validate our proteomic observations, revealing significant enrichment of pathways related to extracellular matrix (ECM) remodelling and membrane microdomain reorganization in Gαq-deficient fibroblasts **(see new Figure EV5A)**. Moreover, extended comparative analysis demonstrate that GαqKO MEFs share a strong transcriptional overlap with CAFs isolated from HNSCC patients. We now show in **new Figures EV5B and EV5C** that GαqKO MEFs share similarity to the "matrisome" profiles of highly aggressive HNSCC tumors and that they exhibit significant enrichment for the HNCAF-1 transcriptional program described by Obradovic et al. (2022), a CAF population associated with poor overall survival in HNSCC and characterized by pro-inflammatory and immunomodulatory functions. These observations further suggests that Gαq-deficient fibroblasts are likely involved in modulating inflammatory/immunological responses within the tumor microenvironment, one of the critical hallmarks of functionally active CAFs.

Therefore, although direct Gαq immunostaining cannot be currently used as a diagnostic readout, our study identifies a relevant protein signature associated with Gαq deficiency. This signature includes upregulation of PDGFR, Cav-1, PTRF, ceramide, LAMP1 and Collagen I **(see new Figure 8C)**. Notably, this molecular profile is consistently observed in GαqKO MEFs, in Gαq-knockdown human normal fibroblasts and in patient-derived CAFs from HNSCC tumors **(see new panels in Figures 7 and EV4)**, thus reinforcing the translational relevance of our observations and putting forward this signature as a functional surrogate marker of Gαq deficiency in tumor-associated stroma.

2b) Is the Gaq staining intensity associated with clinical outcome in the patients? The clinical correlation of Gaq attenuation in stromal fibroblasts should be provided.

This point has already been addressed in our response to the previous comment.

3) The author indicated that GaqKOMEFs promote tumor invasion. How do carcinoma cells increase their ability to invade. Mechanistic insight including partial EMT induced in tumor cells should be provided.

In the initial version of our manuscript, we reported that Gαq KO fibroblasts induce a partial EMT phenotype in Cal27 cells **(now included as new Figure 2C)**. This was evidenced by altered E-cadherin distribution observed via immunofluorescence, although no significant changes were detected at the protein level by Western blot. Based on these findings, we proposed that the EMT induced by Gαq KO fibroblasts was partial in nature.

In the revised manuscript, we have further investigated the underlying mechanism and now demonstrate that this partial EMT is associated to caveolin-1 (Cav-1) downregulation, triggered by exosomes enriched in PDGFR secreted by GαqKO fibroblasts **(please see our detailed response to point 1 above)**. Although PDGFR appears to be a key contributor, it is likely that additional exosomal components also participate in reducing Cav-1 expression levels. Future studies will be necessary to determine the specific molecular mediators involved in this effect.

Caveolin-1 is a multifunctional protein whose role in cancer is highly context-dependent. Nevertheless, numerous studies have shown that Cav-1 downregulation is associated with increased proliferation, EMT induction, and enhanced tumor aggressiveness. In the specific context of HNSCC, Cav-1 expression has been reported to act either as a tumor suppressor or as a tumor promoter, depending on its interaction with other signaling pathways. For instance, Cav-1 overexpression has been associated with abnormal expression of E-cadherin/β-catenin complex components and ErbB receptors, as well as lymph node metastasis (10.1002/jcp.24034). Conversely, reduced Cav-1 expression has been shown to facilitate EMT and metastatic behavior through α5β1 integrin upregulation (10.18632/oncotarget.6099). These findings collectively support our observations and reinforce the relevance of Cav-1 downregulation in the EMT-like changes induced by exosomes from GαqKO fibroblasts.

4) In Figure 6D, increased invasiveness of tumor cells is shown in Cal27 tumor admixed with GaqKOMEFs. To formally conclude that GaqKOMEFs stimulate tumor invasiveness in vivo, invasiveness would be quantified, for example, by counting the number of tumor budding. Whether coinjection with GaqKOMEFs boosts metastasis of carcinoma cells into distant organs should also be examined.

We thank the reviewer for this valuable observation. In response, we have now incorporated representative images that clearly illustrate the marked differences

in the number of tumor buddings observed between conditions. These images illustrate that, in tumors triggered by Cal27 cells co-injected with GαqKO MEFs, the number of tumor buds (defined as small invasive clusters at the tumor edge) is drastically and markedly higher than when Cal27 tumor cells are co-injected with WT MEFs **(see figure below for your consideration)**.

Regarding metastasic dissemination, we fully agree with the reviewer that this is an important aspect of tumor invasiveness. We have performed an analysis of distant metastasis and found that co-injection with GαqKO fibroblasts enhanced metastatic burden, particularly in the lungs, where 75% of Cal27/GαqKO mice developed metastases **(new Figure 6G)**. These results indicate that Gαq-deficient fibroblasts enhance both proliferation capacity and metastasic dissemination of carcinoma cells *in vivo*.

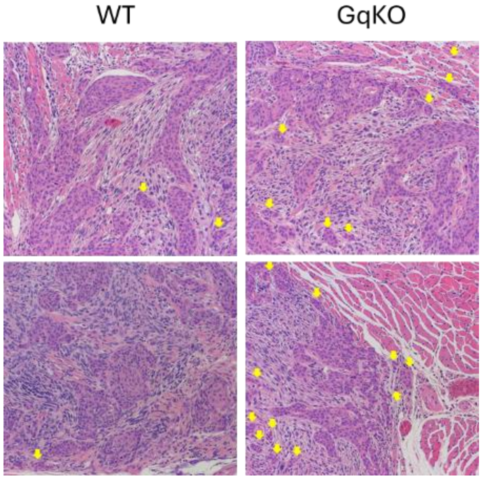

Figure for reviewer: H&E staining of tumors generated by coinjection of CAL27 cells with WT or Gαq-KO MEFs in NSG mice. Tumor budding events are indicated by yellow arrows. Notably, tumors formed in the presence of GαqKO MEFs display a markedly higher degree of tumor budding compared to those generated with WT MEFs.

5) Two different PDGFRs, such as alpha and beta forms are known. However, the authors mentioned just as PDGFR without discriminating alpha and beta forms in the text as well as figures. Which forms of PDGFRs are increased on GaqKOMEFs?

We thank the reviewer for bringing attention to the distinction between PDGFRα and PDGFRβ. Although the antibody used in our studies recognizes both isoforms, our proteomic analysis indicates that PDGFRα is the isoform most significantly dysregulated in GαqKO fibroblasts. As shown in the revised manuscript (**see new Fig. 5F)** and discussed in our response to point 1**,** overexpression of either PDGFRα or PDGFRβ in Cal27 cells induces a similar reduced Cav-1 and PTRF levels phenotype. A more detailed analysis will be required to precisely evaluate the specific contribution of each isoform**.**

6) How does Gaq expression go down in CAFs? The mechanistic insight should be mentioned in Discussion.

We thank the reviewer for highlighting this important point. The modulation of Gαq expression may occur at multiple levels, and several regulatory mechanisms could underlie the reduction of its expression in our model, which specific mechanisms would need additional investigation in the future. It is also possible that changes in the stimuli modulating Gαq signaling networks may trigger phenotypic effects similar to those triggered by decreased Gαq expression levels.

Indeed, the transcriptional regulation of GNAQ has been only partially characterized. Promoter studies have shown that GNAQ expression is controlled by specific promoter polymorphisms and by the early growth response factor EGR-1, which directly binds the GNAQ promoter and enhances its transcription in response to phorbol ester stimulation and growth factor–related signals (10.1093/eurheartj/ehm618; 10.1111/j.1538-7836.2006.02229.x; 10.1097/FPC.0b013e32833b7497).

Interestingly, in silico analyses identify multiple predicted binding sites for AP-1, ATF-2, c-Fos/c-Jun and SRF within the GNAQ promoter and nearby regulatory regions, suggesting that stress- and cytokine-responsive transcription factors could also contribute to its regulation in a cell-type-specific manner (https://www.genecards.org/cgi-bin/carddisp.pl?gene=GNAQ).

Epigenetic modifications, including DNA methylation or histone acetylation at the GNAQ promoter, have also been reported in certain cancers and may lead to transcriptional silencing (10.3390/ijms242216442)

Post-transcriptionally, microRNAs predicted to target GNAQ mRNA may also contribute to the reduction of Gαq protein levels, as suggested by recent bioinformatic analyses (10.1007/s00018-020-03612-w). In addition, several studies have shown that Gαq protein abundance is controlled also post-translationally through ubiquitination and proteasome-dependent degradation (10.1111/j.1742-4658.2005.04934.x; 10.1016/j.bbrc.2013.04.103)

We have now incorporated a summary of these potential mechanistic insights in the Discussion of the revised manuscript.

MINOR ISSUES

1. The authors described "Gaq" as "Gαq " in all figures but not in the text and figure legends.
2. Description as to Gαq KO should be stated in the text and figure legends, as appropriate.

We thank the reviewer for these helpful observations.

We acknowledge the inconsistency in the use of “Gaq” versus “Gαq” in the figures and text. This issue has been corrected throughout the revised version to ensure consistent terminology across the main text, figure legends, and all figures.

We agree that the description of “GαqKO” should be clearly stated in both the text and the figure legends. This clarification has been included in the revised manuscript to ensure that the designation “GαqKO” is consistently defined and properly referenced.

Note for reviewer:

The data sets produced in this study are available in the following databases:

-Proteomics data: The mass-spectrometry proteomics datasets derived from lysates of WT and GαqKO MEFs, as well as their corresponding exosomes, have been deposited in the ProteomeXchange Consortium via the PRIDE partner repository (Perez-Riverol et al., 2025) under the accession number PXD061641.

Dear Dr. Ribas,

Thank you for the submission of your revised manuscript to our editorial offices. I have now received the reports from the three referees that I asked to re-evaluate the study, you will find below. As you will see, the referees #1 and #2 now fully support publication of your study in EMBO reports. In contrast, referee #3 has remaining concerns and requests to improve the manuscript.

After cross-commenting with the other two referees, I have decided to proceed with the manuscript, and ask you to address the remaining concerns textually, i.e. by a revised abstract and discussion, detailing limitations of the study and tempering the conclusion that "GaqKO-MEF-derived PDGFR mediates tumor progression", in a final revised manuscript. Please also provide a final p-b-p-response regarding the remaining referee points and the editorial requests below.

Editorial requests:

- Please order the manuscript sections like this, using only these names:
Title page - Abstract - Keywords - Introduction - Results - Discussion - Methods - Data availability section - Acknowledgements - Disclosure and Competing Interests Statement - References - Figure legends - Expanded View Figure legends

- Please remove now the referee access information from the Data Availability section and make sure that the datasets are public latest upon online publication of the manuscript. Please also add direct links to each dataset.

- Please check again that the number "n" for how many independent experiments were performed, their nature (biological versus technical replicates), the bars and error bars (e.g. SEM, SD) and the test used to calculate p-values is indicated in the respective figure legends (main, EV and Appendix figures). Please also check that all the p-values are explained in the legend, and that these fit to those shown in the figure. Please provide statistical testing where applicable. Please avoid the phrase 'independent experiment' but clearly state if these were biological or technical replicates. Please also indicate (e.g. with n.s.) if testing was performed, but the differences are not significant. In case n=2, please show the data as separate datapoints without error bars and statistics. See also:

https://link.springer.com/journal/44319/submission-guidelines#cms-Figure-and-data-presentation

If n<5, please show single datapoints for diagrams. It seems presently some diagrams are missing statistics (e.g. 3G, 7I and S2B) or the n.s. (e.g. 4B). Please check. Moreover:
- Please note that the exact p values are not provided in the legends of figures 1A-D; 2E, 4D, E, F; 5A, G; 6A, B, D; 7A, D, E, I; EV5 C
-Please indicate the statistical test used for data analysis in the legends of figures 1A-D; 2E, 3A, B; 4D, E, F; 5A, B, G; 6B, D; 7A, D, E, I; EV5 A-C"
-Please note that information related to n is missing in the legend of figure EV4 F
-Please note that the error bars are not defined in the legends of figures EV3 B, EV4 F. "
-Please note that the scale bar needs to be defined for figures 3D, 4E, 5F, 6E, F; EV2 E
-Please note that scale bar and its definition are missing for figures 2A, 5C, 6B, D, G; EV1 A, EV2 C, EV4 H, S1 B
-Please note that the dotted borders are not defined in the legend of figure 6B. This needs to be rectified.
-Please note that the yellow dashed borders are not defined in the legend of figure 6B. This needs to be rectified.
-Please note that the asterisk is not defined in the legend of figure 2C. This needs to be rectified.
-Please note that the arrow heads are not defined in the legend of figure 3D, 4G, 7B, EV5 A. This needs to be rectified.
-Please note that the yellow arrow is not defined in the legend of figure EV2 G. This needs to be rectified."
- Please note that the data callouts in the text for the data citation (Di Martino JS, Nobre AR, Mondal C, Taha I, Farias EF, Fertig EJ, Naba A, Aguirre Ghiso JA, Bravo-Cordero
JJ GSEA (2021a)) does not include "Data ref:" as a prefix."

- Please make sure that all the funding information is also entered into the online submission system and that it is complete and similar to the one in the acknowledgement section of the manuscript text file. Presently, the grants Principado de Asturias BioBank (PT23/00077), financed jointly by Servicio de Salud del Principado de Asturias, Instituto de Salud Carlos III and Fundacion Bancaria Cajastur, and integrated in the Spanish National Biobanks Network.; Severo Ochoa Center of Excellence (grant CEX2020-001041-S funded by MICIU/AEI/10.13039/501100011033 are missing in the online system. Please check.

- Please add scale bars of similar style and thickness to all microscopic images, using clearly visible black or white bars (depending on the background). Please place these in the lower right corner of the images themselves. Please do not write on or near the bars in the image but define the size in the respective figure legend. Presently, most scale bars are rather thin or small and will be not well visible online. Please provide thicker and larger scale bars.

- Please indicate molecular weights for all Western blot panels shown.

- Please make sure that each final figure panel is called out separately and that the panels are called out sequentially. Presently, it seems there are no separate callouts for panel 8A-C. Please check.

- Please move the panels of the two Appendix figures to the EV figures (there is enough space). Then, we do not need an Appendix file. Then, please update all the affected callouts.

- The correct nomenclature for the datasets is 'Dataset EVx' (not Dataset Sx). Please use the correct nomenclature for their names, in their legends and for their callouts.

- Please check the magnification box in Fig. 5C (bottom panel). The box on the right seems not to match to the highlighted square on the left.

- Please provide the numerical source data as excel files.

In addition, I would need from you uploaded separately:
- a schematic summary figure as separate file that provides a sketch of the major findings (not a data image) in jpeg or tiff format (with the exact width of 550 pixels and a height of not more than 400 pixels) that can be used as a visual synopsis on our website. The image provided contains partly text that is not readable. Please provide this with bigger fonts.

I look forward to seeing the further revised version of your manuscript when it is ready. Please let me know if you have questions regarding the revision.

Best,

Achim Breiling
Senior Editor
EMBO Reports

Referee #1:

The authors have adequately addressed my concerns and should be congratulated for the excellent piece of work.

Referee #2:

The authors have done a satisfactory job of addressing my concerns about biological relevance and mechanistic basis for the Gq knockout. The additional explanatory text and supporting figures have further strengthened this manuscrip.

Referee #3:

Whether GaqKO-MEF-derived PDGFR mediates tumor progression remains unclear. To address this issue, as the reviewer mentioned previously, PDGFR expression needs to be inhibited on GaqKO-MEFs prior to employment of various in vitro and in vivo co-mixing experiments using cancer cells. Whether either PDGFR alpha or beta contributes to the GaqKO-MEF-primed tumor progression should also be elucidated, as the reviewer pointed out previously. The level of Gaq protein expression is downregulated in CAFs compared to NFs without statistical significance in Fig. 7G. There is also no quantitative datum of PDGFR expression among six different CAFs and the patient-matched NFs. Whether Gaq expression is downregulated and PDGFR expression is conversely upregulated on CAFs in HNSCC patients is not demonstrated in this revised version due to the technical difficulty of Gaq staining, nonetheless of the reviewer's suggestion.

Overall, it is not convincing that Gaq-downregulation in CAFs is biologically relevant and important for tumor progression in HNSCC patients. Whether CAF-primed PDGFR mediates tumor progression has also been not clear.

MS# EMBOR-2025-61979V2

Manuscript Title: “Loss of Gαq reshapes fibroblast traits and drives tumor-stroma remodeling in oral cancer progression”

POINT BY POINT RESPONSES TO REVIEWERS

REVIEWER´S COMMENT

Reviewer: 1

The authors have adequately addressed my concerns and should be congratulated for the excellent piece of work.

We sincerely thank the reviewer for your generous comment and positive evaluation of our work. We greatly appreciate your recognition, as well the time and care you devoted to reviewing the manuscript, which were invaluable in improving its quality.

Reviewer: 2

The authors have done a satisfactory job of addressing my concerns about biological relevance and mechanistic basis for the Gq knockout. The additional explanatory text and supporting figures have further strengthened this manuscript.

We sincerely thank the reviewer for your thoughtful and positive assessment of our manuscript. We greatly appreciate your careful and the constructive feedback provided throughout the review process, which have significantly contributed to improving the quality and clarity of the manuscript.

Reviewer: 3

Whether GaqKO-MEF-derived PDGFR mediates tumor progression remains unclear. To address this issue, as the reviewer mentioned previously, PDGFR expression needs to be inhibited on GaqKO-MEFs prior to employment of various in vitro and in vivo co-mixing experiments using cancer cells. Whether either PDGFR alpha or beta contributes to the GaqKO-MEF-primed tumor progression should also be elucidated, as the reviewer pointed out previously. The level of Gaq protein expression is downregulated in CAFs compared to NFs without statistical significance in Fig. 7G. There is also no quantitative datum of PDGFR expression among six different CAFs and the patient-matched NFs. Whether Gaq expression is downregulated and PDGFR expression is conversely upregulated on CAFs in HNSCC patients is not demonstrated in this revised version due to the technical difficulty of Gaq staining, nonetheless of the reviewer's suggestion.

Overall, it is not convincing that Gaq-downregulation in CAFs is biologically relevant and important for tumor progression in HNSCC patients. Whether CAF-primed PDGFR mediates tumor progression has also been not clear.

We sincerely thank the reviewer for their insightful and constructive feedback and for reiterating these important points.

The central conclusion of our study is that loss of Gαq reprograms fibroblast traits toward a CAF-like phenotype with a robust pro-tumorigenic effect, validated *in vivo*. Importantly, this effect is not solely dependent on PDGFR signaling but reflects broader Gαq-dependent reprogramming where this phenotype is accompanied by a coordinated remodeling of vesicular trafficking/EV outputs and ECM/secretory programs, resulting in a pro-tumorigenic exosomal secretome. Given that PDGFR has been proposed as a robust stromal activation/CAF marker in OSCC, with association to stromal remodeling and aggressive disease features, PDGFR was chosen as a representative, but not exclusive, biologically relevant marker.

We acknowledge that direct PDGFR inhibition in GαqKO-MEFs prior to co-mixing would be of interest to further clarify the role of this pathway in the observed effects. In this regard, we have provided evidence that pharmacological PDGFR blockade with AG1295 partially attenuated the proliferative response induced by GαqKO-derived exosome (Fig. EV2K) consistent with PDGFR being a functionally relevant component of this signaling axis. Nonetheless, additional cargo components, including other growth factors, DNA, RNA, and microRNAs, may contribute to the observed phenotype. In light of the reviewer's concern, we have tempered our wording throughout the manuscript (and specifically added an additional sentence in the Discussion section, page #17) to state more clearly that PDGFR alone does not fully mediates GqKO-mediated tumor progression but rather acts as a biologically grounded candidate effector/component within a broader Gαq-dependent stromal rewiring.

On PDGFRα vs PDGFRβ, we agree that isoform-specific dissection would add mechanistic precision. However, PDGFR regulation by Gαq in fibroblasts is highly complex and clarifying their relative contributions will require further dedicated isoform-resolved studies. Alternatively, we have provided functional evidence supporting the contribution of both isoforms to the GαqKO-fibroblast EV axis since enforced expression of PDGFRα or PDGFRβ in Cal27 oral cancer cells recapitulated key downstream of tumor-promoting signals (Fig 5G) Notably, apart from the potential PDGFRα versus PDGFRβ contributions, we have also observed an alternative, lower–molecular-weight PDGFR species (~60–70 kDa) in Gαq-deficient cells, potentially representing a proteolytically processed receptor selectively incorporated into exosomes. This highlights the pivotal role of altered vesicular dynamics and PDGFR processing in tumor progression. Fully deciphering the detailed molecular mechanisms underlying PDGFR trafficking and processing would require substantial effort and the development of new experimental approaches in future studies of our lab. A sentence has been added to the Discussion to stress the interest of this future area of research (page #17)

Regarding CAFs, we agree with the reviewer that the decrease in Gαq protein in CAFs vs matched NFs does not reach statistical significance in our dataset, and we have revised the text in the Results and Discussion sections to state this explicitly (page #14 and #18, respectively). Given the well-documented heterogeneity of CAF states in HNSCC/OSCC and the limited number of available matched primary cultures, lack of statistical significance does not exclude biological relevance, but it does warrant

cautious interpretation. In this regard, we have performed a set of experiments using normal human fibroblasts with reduced Gαq expression providing strong evidence that even a modest reduction in Gαq expression in human fibroblasts may have significant functional consequences. For similar reasons, PDGFR levels can vary substantially across CAF preparations; therefore, we frame our patient-derived observations as supportive but not definitive, and we emphasize that our strongest evidence for stromal relevance remains the consistent *in vivo* functional impact of Gαq loss in fibroblasts on tumor progression. A more comprehensive, patient-matched quantitative analysis is ongoing and CAF heterogeneity and tumor-stage–dependent variation further emphasizes the need for population-resolved analyses, which we plan to report separately in a future study.

For patient immunostaining, paraffin-embedded samples limit reliable Gαq detection. We are exploring OCT-fixed sections and complementary approaches, including high-sensitivity *in situ* hybridization (RNAscope) and reporter systems, to robustly assess Gαq expression and localization in future studies.

We have revised the manuscript to clarify these limitations, explicitly noting the lack of statistical significance in Gαq downregulation and the need for further quantitative PDGFR analyses. Overall, the revision tempers our conclusions and outline future experimental strategies aimed at directly addressing the reviewer´s points. It is important to notice that our findings provide a foundational framework linking Gαq signaling, stromal reprogramming, and tumor progression. Nevertheless, we anticipate that future studies will expand upon these insights to establish their biological and clinical relevance in HNSCC patients.

Dr. Catalina Ribas
Universidad Autonoma de Madrid-Centro de Biologiaa Molecular Severo Ochoa
Molecular Biology
C/Nicolas Cabrera, 1
Madrid, Madrid 28049
Spain

Dear Dr. Ribas,

Thank you for the submission of your final revised manuscript to our editorial offices. In now went through this and your final p-b-p-response and consider the remaining points of referee #3 and the editorial requests as adequately addressed.

I am thus very pleased to accept your manuscript for publication in the next available issue of EMBO reports. Thank you for your contribution to our journal.

Your manuscript will be processed for publication by EMBO Press. It will be copy edited and you will receive page proofs prior to publication. Please note that you will be contacted by Springer Nature Author Services to complete licensing and payment information.

You may qualify for financial assistance for your publication charges - either via a Springer Nature fully open access agreement or an EMBO initiative. Check your eligibility: https://link.springer.com/journal/44319/how-to-publish-with-us

Should you be planning a Press Release on your article, please get in contact with embo_production@springernature.com as early as possible in order to coordinate publication and release dates.

If you have any questions, please do not hesitate to contact the Editorial Office. Thank you for your contribution to EMBO Reports.

Yours sincerely,

Achim Breiling
Senior Editor
EMBO Reports

>>> Please note that it is EMBO Reports policy for the transcript of the editorial process (containing referee reports and your response letter) to be published as an online supplement to each paper. If you do NOT want this, you will need to inform the Editorial Office via email immediately. More information is available here: https://link.springer.com/partners/embo-press/editorial-policies#Peer%20review

EMBO Press Author Checklist

Corresponding Author Name: Inmaculada Navarro Lérida, Federico Mayor, Catalina Ribas
Journal Submitted to: EMBO Reports
Manuscript Number: EMBOR-2025-61979-T

USEFUL LINKS FOR COMPLETING THIS FORM
The EMBO Journal - Author Guidelines
EMBO Reports - Author Guidelines
Molecular Systems Biology - Author Guidelines
EMBO Molecular Medicine - Author Guidelines

Reporting Checklist for Life Science Articles (updated January
This checklist is adapted from Materials Design Analysis Reporting (MDAR) Checklist for Authors. MDAR establishes a minimum set of requirements in transparent reporting in the life sciences (see Statement of Task: 10.31222/osf.io/9sm4x). Please follow the journal's guidelines in preparing your
Please note that a copy of this checklist will be published alongside your article.

Abridged guidelines for figures
1. Data
The data shown in figures should satisfy the following conditions:
- ➞ the data were obtained and processed according to the field's best practice and are presented to reflect the results of the experiments in an accurate and unbiased manner.
- ➞ ideally, figure panels should include only measurements that are directly comparable to each other and obtained with the same assay.
- ➞ plots include clearly labeled error bars for independent experiments and sample sizes. Unless justified, error bars should not be shown for technical
- ➞ if n<5, the individual data points from each experiment should be plotted. Any statistical test employed should be justified.
- ➞ Source Data should be included to report the data underlying figures according to the guidelines set out in the authorship guidelines on Data

2. Captions
Each figure caption should contain the following information, for each panel where they are relevant:
- ➞ a specification of the experimental system investigated (eg cell line, species name).
- ➞ the assay(s) and method(s) used to carry out the reported observations and measurements.
- ➞ an explicit mention of the biological and chemical entity(ies) that are being measured.
- ➞ an explicit mention of the biological and chemical entity(ies) that are altered/varied/perturbed in a controlled manner.
- ➞ the exact sample size (n) for each experimental group/condition, given as a number, not a range;
- ➞ a description of the sample collection allowing the reader to understand whether the samples represent technical or biological replicates (including how many animals, litters, cultures, etc.).
- ➞ a statement of how many times the experiment shown was independently replicated in the laboratory.
- ➞ definitions of statistical methods and measures:
  - common tests, such as t-test (please specify whether paired vs. unpaired), simple χ2 tests, Wilcoxon and Mann-Whitney tests, can be unambiguously identified by name only, but more complex techniques should be described in the methods section;
  - are tests one-sided or two-sided?
  - are there adjustments for multiple comparisons?
  - exact statistical test results, e.g., P values = x but not P values < x;
  - definition of 'center values' as median or average;
  - definition of error bars as s.d. or s.e.m.

Please complete ALL of the questions below.
Select "Not Applicable" only when the requested information is not relevant for your study.

Materials

Newly Created Materials	Information included in the manuscript?	In which section is the information available? (Reagents and Tools Table, Materials and Methods, Figures, Data Availability Section)
New materials and reagents need to be available; do any restrictions apply?	Not Applicable	

Antibodies	Information included in the manuscript?	In which section is the information available? (Reagents and Tools Table, Materials and Methods, Figures, Data Availability Section)
For **antibodies** provide the following information:<br>- Commercial antibodies: RRID (if possible) or supplier name, catalogue number and or/clone number<br>- Non-commercial: RRID or citation	Yes	Reagents and Tools Table and Materials and Methods (Page 29)

DNA and RNA sequences	Information included in the manuscript?	In which section is the information available? (Reagents and Tools Table, Materials and Methods, Figures, Data Availability Section)
**Short novel DNA or RNA including primers, probes**: provide the sequences.	Yes	Reagents and Tools Table and Materials and Methods (Page 29)

Cell materials	Information included in the manuscript?	In which section is the information available? (Reagents and Tools Table, Materials and Methods, Figures, Data Availability Section)
**Cell lines:** Provide species information, strain. Provide accession number in repository **OR** supplier name, catalog number, clone number, and/**OR** RRID.	Yes	Reagents and Tools Table and Materials and Methods (Page 20)
**Primary cultures:** Provide species, strain, sex of origin, genetic modification status.	Yes	Materials and Methods (Page 20)
Report if the cell lines were recently **authenticated** (e.g., by STR profiling) and tested for mycoplasma contamination.	Yes	Materials and Methods (Page 20)

Experimental animals	Information included in the manuscript?	In which section is the information available? (Reagents and Tools Table, Materials and Methods, Figures, Data Availability Section)
**Laboratory animals or Model organisms:** Provide species, strain, sex, age, genetic modification status. Provide accession number in repository **OR** supplier name, catalog number, clone number, **OR** RRID.	Yes	Reagents and Tools Table and Materials and Methods (Page 20)
**Animal observed in or captured from the field:** Provide species, sex, and age where possible.	Not Applicable	
Please detail **housing and husbandry conditions**.	Yes	Materials and Methods (Page 20)

Plants and microbes	Information included in the manuscript?	In which section is the information available? (Reagents and Tools Table, Materials and Methods, Figures, Data Availability Section)
**Plants:** provide species and strain, ecotype and cultivar where relevant, unique accession number if available, and source (including location for collected wild specimens).	Not Applicable	
**Microbes:** provide species and strain, unique accession number if available, and source.	Not Applicable	

Human research participants	Information included in the manuscript?	In which section is the information available? (Reagents and Tools Table, Materials and Methods, Figures, Data Availability Section)
If collected and within the bounds of privacy constraints report on age, sex and gender or ethnicity for all study participants.	Not Applicable	

Core facilities	Information included in the manuscript?	In which section is the information available? (Reagents and Tools Table, Materials and Methods, Figures, Data Availability Section)
If your work benefited from core facilities, was their service mentioned in the acknowledgments section?	Yes	ACKNOWLEDGMENTS (Page 31)

Design

Study protocol	Information included in the manuscript?	In which section is the information available? (Reagents and Tools Table, Materials and Methods, Figures, Data Availability Section)
If study protocol has been **pre-registered, provide DOI in the manuscript**. For clinical trials, provide the trial registration number **OR** cite DOI.	Not Applicable	
Report the **clinical trial registration number** (at ClinicalTrials.gov or equivalent), where applicable.	Not Applicable	

Laboratory protocol	Information included in the manuscript?	In which section is the information available? (Reagents and Tools Table, Materials and Methods, Figures, Data Availability Section)
Provide DOI OR other citation details if **external detailed step-by-step protocols** are available.	Not Applicable	

Experimental study design and statistics	Information included in the manuscript?	In which section is the information available? (Reagents and Tools Table, Materials and Methods, Figures, Data Availability Section)
Include a statement about **sample size** estimate even if no statistical methods were used.	Yes	Materials and Methods (Page30)
Were any steps taken to minimize the effects of subjective bias when allocating animals/samples to treatment (e.g. **randomization procedure**)? If yes, have they been described?	Yes	Materials and Methods (Page 30)
Include a statement about **blinding** even if no blinding was done.	Yes	Materials and Methods (Page 30)
Describe **inclusion/exclusion criteria** if samples or animals were excluded from the analysis. Were the criteria pre-established?<br><br>If sample or data points were omitted from analysis, report if this was due to attrition or intentional exclusion and provide justification.	Not Applicable	
For every figure, are **statistical tests** justified as appropriate? Do the data meet the assumptions of the tests (e.g., normal distribution)? Describe any methods used to assess it. Is there an estimate of variation within each group of data? Is the variance similar between the groups that are being statistically compared?	Yes	Materials and Methods (Page 30)

Sample definition and in-laboratory replication	Information included in the manuscript?	In which section is the information available? (Reagents and Tools Table, Materials and Methods, Figures, Data Availability Section)
In the figure legends: state number of times the experiment was **replicated** in laboratory.	Yes	Figures
In the figure legends: define whether data describe **technical or biological replicates**.	Yes	Figures

Ethics

Ethics	Information included in the manuscript?	In which section is the information available? (Reagents and Tools Table, Materials and Methods, Figures, Data Availability Section)
Studies involving **human participants**: State details of **authority granting ethics approval** (IRB or equivalent committee(s), provide reference number for approval.	Yes	Materials and Methods (Page 21)
Studies involving **human participants**: Include a statement confirming that **informed consent** was obtained from all subjects and that the experiments conformed to the principles set out in the WMA Declaration of Helsinki and the Department of Health and Human Services Belmont Report.	Yes	Materials and Methods (Page 21)
Studies involving **human participants:** For publication of **patient photos**, include a statement confirming that consent to publish was obtained.	Not Applicable	
Studies involving experimental **animals**: State details of **authority granting ethics approval** (IRB or equivalent committee(s), provide reference number for approval. Include a statement of compliance with ethical regulations.	Yes	Materials and Methods (Page 20)
Studies involving **specimen and field samples:** State if relevant **permits** obtained, provide details of authority approving study; if none were required, explain why.	Not Applicable	

Dual Use Research of Concern (DURC)	Information included in the manuscript?	In which section is the information available? (Reagents and Tools Table, Materials and Methods, Figures, Data Availability Section)
Could your study fall under dual use research restrictions? Please check biosecurity documents and list of **select agents and toxins** (CDC): https://www.selectagents.gov/sat/list.htm	Not Applicable	
If you used a select agent, is the security level of the lab appropriate and reported in the manuscript?	Not Applicable	
If a study is subject to dual use research of concern regulations, is the name of the **authority granting approval and reference number** for the regulatory approval provided in the manuscript?	Not Applicable	

Reporting

The MDAR framework recommends adoption of discipline-specific guidelines, established and endorsed through community initiatives. Journals have their own policy about requiring specific guidelines and recommendations to complement MDAR.

Adherence to community standards	Information included in the manuscript?	In which section is the information available? (Reagents and Tools Table, Materials and Methods, Figures, Data Availability Section)
State if relevant guidelines or checklists (e.g., **ICMJE, MIBBI, ARRIVE, PRISMA**) have been followed or provided.	Not Applicable	
For **tumor marker prognostic studies,** we recommend that you follow the **REMARK** reporting guidelines (see link list at top right). See author guidelines, under 'Reporting Guidelines'. Please confirm you have followed these guidelines.	Not Applicable	
For **phase II and III randomized controlled trials**, please refer to the **CONSORT** flow diagram (see link list at top right) and submit the CONSORT checklist (see link list at top right) with your submission. See author guidelines, under 'Reporting Guidelines'. Please confirm you have submitted this list.	Not Applicable	

Data Availability

Data availability	Information included in the manuscript?	In which section is the information available? (Reagents and Tools Table, Materials and Methods, Figures, Data Availability Section)
Have **primary datasets** been deposited according to the journal's guidelines (see 'Data Deposition' section) and the respective accession numbers provided in the Data Availability Section?	Yes	Data availability (Pages 30-31)
Were **human clinical and genomic datasets** deposited in a public access-controlled repository in accordance to ethical obligations to the patients and to the applicable consent agreement?	Not Applicable	
Are **computational models** that are central and integral to a study available without restrictions in a machine-readable form? Were the relevant accession numbers or links provided?	Not Applicable	
If publicly available data were reused, provide the respective **data citations in the reference list**.	Yes	References (Page 33)
